# Supplementary material for: Matriptase activation of Gq drives epithelial disruption and inflammation via RSK and DUOX
Source: eLife. 2021 Jun 24;10:e66596. doi: 10.7554/eLife.66596 (PMC8291973; doi:10.7554/eLife.66596)
Supplement: Figure 1—source data 3. [file elife-66596-fig1-data3.pdf]

# 2011_07
# NGSE110704

## Analysis Information

| | | | |
|---|---|---|---|
| Report Type | Protein-Peptide Summary by Spot | Analysis Type | Combined (MS+MS/MS) |
| Sample Set Name | NGSE110704 | Database | NCBInr |
| Analysis Name | Nebrafish_Top50 | Creation Date | 08/26/2011 13:07:53 |
| Reported By | 08/29/2011 11:15:22 - admin | Last Modified | 08/26/2011 18:29:58 |

| | |
|---|---|
| MS Acq. : Proc. Methods | (Unspecified) : (Unspecified) |
| Interpretation Method | (Unspecified) |

| | | | | | |
|---|---|---|---|---|---|
| **Gel Idx/Pos** | 174/H1 | **Instr./Gel Origin** | BA2060/110707A | **Process Status** | Analysis Succeeded |
| **Plate [#] Name** | [1] 31496 | **Instrument Sample Name** | | **Spectra** | 11 |

| Rank | Protein Name | Accession No. | Protein MW | Protein PI | Pep. Count | Protein Score | Protein Score C. I. % | Total Ion Score | Total Ion C. I. % |
|---|---|---|---|---|---|---|---|---|---|
| 1 | pyruvate carboxylase, mitochondrial [Danio rerio] | gi\|18858695 | 129801.9 | 6.44 | 21 | 295 | 100 | 219 | 100 |

**Peptide Information**

| Calc. Mass | Obsrv. Mass | ± da | ± ppm | Start Seq. | End Seq. | Sequence | Ion Score | C. I. % | Modification | Rank | Result Type |
|---|---|---|---|---|---|---|---|---|---|---|---|
| 877.4162 | 877.423 | 0.0068 | 8 | 388 | 395 | GFQPDTGR | | | | | Mascot |
| 1064.5299 | 1064.5403 | 0.0104 | 10 | 720 | 727 | YSLDYYLK | | | | | Mascot |
| 1100.6099 | 1100.6183 | 0.0084 | 8 | 140 | 149 | FIGPSPEVVR | | | | | Mascot |
| 1111.5854 | 1111.5952 | 0.0098 | 9 | 574 | 583 | DAHQSLLATR | | | | | Mascot |
| 1151.6418 | 1151.6522 | 0.0104 | 9 | 246 | 255 | HIEVQILGDK | | | | | Mascot |
| 1164.5796 | 1164.5923 | 0.0127 | 11 | 322 | 330 | HYFIEVNSR | | | | | Mascot |
| 1165.5823 | 1165.7551 | 0.1728 | 148 | 620 | 628 | FLSECPWKR | | | | | Mascot |
| 1263.6481 | 1263.6631 | 0.015 | 12 | 256 | 265 | YGNVIHLYER | 59 | 99.97 | | | Mascot |
| 1263.6692 | 1263.6631 | -0.0061 | -5 | 80 | 90 | QKADEAYLIGR | | | | | Mascot |
| 1275.6296 | 1275.6111 | -0.0185 | -15 | 369 | 379 | IQINGCAIQCR | | | Carbamidomethyl (C)[6] | | Mascot |
| 1351.7004 | 1351.7147 | 0.0143 | 11 | 1076 | 1086 | EVFFELNGQLR | | | | | Mascot |
| 1474.6705 | 1474.6882 | 0.0177 | 12 | 845 | 857 | GLYAPFDCTATMK | | | Carbamidomethyl (C)[8] | | Mascot |
| 1502.8069 | 1502.7963 | -0.0106 | -7 | 1132 | 1145 | VEKGQPLCVLSAMK | | | | | Mascot |
| 1518.8019 | 1518.7903 | -0.0116 | -8 | 1132 | 1145 | VEKGQPLCVLSAMK | | | Oxidation (M)[13] | | Mascot |
| 1658.7665 | 1658.9414 | 0.1749 | 105 | 664 | 677 | FCEVAKENGMDIFR | | | | | Mascot |
| 1658.7665 | 1658.9414 | 0.1749 | 105 | 664 | 677 | FCEVAKENGMDIFR | 3 | 0 | | | Mascot |

| | | | | | | | | | | | |
|---|---|---|---|---|---|---|---|---|---|---|---|
| 1721.7911 | 1721.8103 | 0.0192 | 11 | 65 | 79 | TVAVYSEQDTGQMHR | | | | | Mascot |
| 1737.7861 | 1737.8036 | 0.0175 | 10 | 65 | 79 | TVAVYSEQDTGQMHR | | | Oxidation (M)[13] | | Mascot |
| 1768.8751 | 1768.8936 | 0.0185 | 10 | 303 | 318 | QVGYENAGTVEFLVDK | | | | | Mascot |
| 1800.8551 | 1800.8799 | 0.0248 | 14 | 647 | 663 | GANAVGYTNYPDNAVFK | | | | | Mascot |
| 1857.9381 | 1857.9545 | 0.0164 | 9 | 222 | 239 | AYSEALAAFGNGALFVEK | | | | | Mascot |
| 1993.9396 | 1994.0005 | 0.0609 | 31 | 65 | 81 | TVAVYSEQDTGQMHRQK | | | Oxidation (M)[13] | | Mascot |
| 2545.3257 | 2545.3594 | 0.0337 | 13 | 331 | 352 | LQVEHTVTEEITDVDLVH AQLR | 161 | 100 | | | Mascot |
| 2545.3257 | 2545.3594 | 0.0337 | 13 | 331 | 352 | LQVEHTVTEEITDVDLVH AQLR | | | | | Mascot |
| 3364.5286 | 3364.6116 | 0.083 | 25 | 858 | 888 | SGNADVYENEIPGGQYT NLHFQAHSMGLGNK | | | Oxidation (M)[26] | | Mascot |

| | | | | | | | | | | |
|---|---|---|---|---|---|---|---|---|---|---|
| 2 | Pc protein [Danio rerio] | gi\|190338031 | 129917 | 6.44 | 21 | 294 | 100 | 219 | 100 | |

**Peptide Information**

| Calc. Mass | Obsrv. Mass | ± da | ± ppm | Start Seq. | End Seq. | Sequence | Ion Score | C. I. % | Modification | Rank | Result Type |
|---|---|---|---|---|---|---|---|---|---|---|---|
| 877.4162 | 877.423 | 0.0068 | 8 | 388 | 395 | GFQPDTGR | | | | | Mascot |
| 1064.5299 | 1064.5403 | 0.0104 | 10 | 720 | 727 | YSLDYYLK | | | | | Mascot |
| 1100.6099 | 1100.6183 | 0.0084 | 8 | 140 | 149 | FIGPSPEVVR | | | | | Mascot |
| 1111.5854 | 1111.5952 | 0.0098 | 9 | 574 | 583 | DAHQSLLATR | | | | | Mascot |
| 1151.6418 | 1151.6522 | 0.0104 | 9 | 246 | 255 | HIEVQILGDK | | | | | Mascot |
| 1164.5796 | 1164.5923 | 0.0127 | 11 | 322 | 330 | HYFIEVNSR | | | | | Mascot |
| 1165.5823 | 1165.7551 | 0.1728 | 148 | 620 | 628 | FLSECPWKR | | | | | Mascot |
| 1263.6481 | 1263.6631 | 0.015 | 12 | 256 | 265 | YGNVIHLYER | 59 | 99.97 | | | Mascot |
| 1263.6692 | 1263.6631 | -0.0061 | -5 | 80 | 90 | QKADEAYLIGR | | | | | Mascot |
| 1275.6296 | 1275.6111 | -0.0185 | -15 | 369 | 379 | IQINGCAIQCR | | | Carbamidomethyl (C)[6] | | Mascot |
| 1351.7004 | 1351.7147 | 0.0143 | 11 | 1077 | 1087 | EVFFELNGQLR | | | | | Mascot |
| 1474.6705 | 1474.6882 | 0.0177 | 12 | 845 | 857 | GLYAPFDCTATMK | | | Carbamidomethyl (C)[8] | | Mascot |
| 1502.8069 | 1502.7963 | -0.0106 | -7 | 1133 | 1146 | VEKGQPLCVLSAMK | | | | | Mascot |
| 1518.8019 | 1518.7903 | -0.0116 | -8 | 1133 | 1146 | VEKGQPLCVLSAMK | | | Oxidation (M)[13] | | Mascot |
| 1658.7665 | 1658.9414 | 0.1749 | 105 | 664 | 677 | FCEVAKENGMDIFR | | | | | Mascot |
| 1658.7665 | 1658.9414 | 0.1749 | 105 | 664 | 677 | FCEVAKENGMDIFR | 3 | 0 | | | Mascot |
| 1721.7911 | 1721.8103 | 0.0192 | 11 | 65 | 79 | TVAVYSEQDTGQMHR | | | | | Mascot |
| 1737.7861 | 1737.8036 | 0.0175 | 10 | 65 | 79 | TVAVYSEQDTGQMHR | | | Oxidation (M)[13] | | Mascot |
| 1768.8751 | 1768.8936 | 0.0185 | 10 | 303 | 318 | QVGYENAGTVEFLVDK | | | | | Mascot |
| 1800.8551 | 1800.8799 | 0.0248 | 14 | 647 | 663 | GANAVGYTNYPDNAVFK | | | | | Mascot |
| 1857.9381 | 1857.9545 | 0.0164 | 9 | 222 | 239 | AYSEALAAFGNGALFVEK | | | | | Mascot |
| 1993.9396 | 1994.0005 | 0.0609 | 31 | 65 | 81 | TVAVYSEQDTGQMHRQK | | | Oxidation (M)[13] | | Mascot |

| Calc. Mass | Obsrv. Mass | ± da | ± ppm | Start Seq. | End Seq. | Sequence | Ion Score | C. I. % | Modification | Rank | Result Type |
|---|---|---|---|---|---|---|---|---|---|---|---|
| 2545.3257 | 2545.3594 | 0.0337 | 13 | 331 | 352 | LQVEHTVTEEITDVDLVH AQLR | 161 | 100 | | | Mascot |
| 2545.3257 | 2545.3594 | 0.0337 | 13 | 331 | 352 | LQVEHTVTEEITDVDLVH AQLR | | | | | Mascot |
| 3364.5286 | 3364.6116 | 0.083 | 25 | 858 | 888 | SGNADVYENEIPGGQYT NLHFQAHSMGLGNK | | | Oxidation (M)[26] | | Mascot |

| | | | | | | | | | | |
|---|---|---|---|---|---|---|---|---|---|---|
| 3 | pyruvate carboxylase [Danio rerio] | gi\|169153894 | 51981.8 | 7.64 | 12 | 272 | 100 | 219 | 100 | |

**Peptide Information**

| Calc. Mass | Obsrv. Mass | ± da | ± ppm | Start Seq. | End Seq. | Sequence | Ion Score | C. I. % | Modification | Rank | Result Type |
|---|---|---|---|---|---|---|---|---|---|---|---|
| 877.4162 | 877.423 | 0.0068 | 8 | 388 | 395 | GFQPDTGR | | | | | Mascot |
| 1100.6099 | 1100.6183 | 0.0084 | 8 | 140 | 149 | FIGPSPEVVR | | | | | Mascot |
| 1151.6418 | 1151.6522 | 0.0104 | 9 | 246 | 255 | HIEVQILGDK | | | | | Mascot |
| 1164.5796 | 1164.5923 | 0.0127 | 11 | 322 | 330 | HYFIEVNSR | | | | | Mascot |
| 1263.6481 | 1263.6631 | 0.015 | 12 | 256 | 265 | YGNVIHLYER | 59 | 99.97 | | | Mascot |
| 1263.6692 | 1263.6631 | -0.0061 | -5 | 80 | 90 | QKADEAYLIGR | | | | | Mascot |
| 1275.6296 | 1275.6111 | -0.0185 | -15 | 369 | 379 | IQINGCAIQCR | | | Carbamidomethyl (C)[6] | | Mascot |
| 1721.7911 | 1721.8103 | 0.0192 | 11 | 65 | 79 | TVAVYSEQDTGQMHR | | | | | Mascot |
| 1737.7861 | 1737.8036 | 0.0175 | 10 | 65 | 79 | TVAVYSEQDTGQMHR | | | Oxidation (M)[13] | | Mascot |
| 1768.8751 | 1768.8936 | 0.0185 | 10 | 303 | 318 | QVGYENAGTVEFLVDK | | | | | Mascot |
| 1857.9381 | 1857.9545 | 0.0164 | 9 | 222 | 239 | AYSEALAAFGNGALFVEK | | | | | Mascot |
| 1993.9396 | 1994.0005 | 0.0609 | 31 | 65 | 81 | TVAVYSEQDTGQMHRQK | | | Oxidation (M)[13] | | Mascot |
| 2545.3257 | 2545.3594 | 0.0337 | 13 | 331 | 352 | LQVEHTVTEEITDVDLVH AQLR | 161 | 100 | | | Mascot |
| 2545.3257 | 2545.3594 | 0.0337 | 13 | 331 | 352 | LQVEHTVTEEITDVDLVH AQLR | | | | | Mascot |

| | | | | | | | | | | |
|---|---|---|---|---|---|---|---|---|---|---|
| 4 | novel protein similar to vertebrate vasodilator-stimulated phosphoprotein (VASP) [Danio rerio] | gi\|169145929 | 44137.1 | 8.42 | 11 | 55 | 84.57 | | | |

**Peptide Information**

| Calc. Mass | Obsrv. Mass | ± da | ± ppm | Start Seq. | End Seq. | Sequence | Ion Score | C. I. % | Modification | Rank | Result Type |
|---|---|---|---|---|---|---|---|---|---|---|---|
| 980.4465 | 980.4255 | -0.021 | -21 | 2 | 10 | SESSICQAR | | | | | Mascot |
| 1111.4871 | 1111.5952 | 0.1081 | 97 | 1 | 10 | MSESSICQAR | | | | | Mascot |
| 1501.791 | 1501.6599 | -0.1311 | -87 | 22 | 35 | KWVPAGTGPQAFSR | | | | | Mascot |
| 1501.791 | 1501.6599 | -0.1311 | -87 | 22 | 35 | KWVPAGTGPQAFSR | | | | | Mascot |
| 1502.8213 | 1502.7963 | -0.025 | -17 | 404 | 416 | NEIIGAFIQELQK | | | | | Mascot |
| 1561.7295 | 1561.8402 | 0.1107 | 71 | 72 | 83 | YNQATPNFHQWR | | | | | Mascot |
| 1658.9224 | 1658.9414 | 0.019 | 11 | 404 | 417 | NEIIGAFIQELQKR | | | | | Mascot |
| 1658.9224 | 1658.9414 | 0.019 | 11 | 404 | 417 | NEIIGAFIQELQKR | | | | | Mascot |

| Calc. Mass | Obsrv. Mass | ± da | ± ppm | Start Seq. | End Seq. | Sequence | Ion Score | C. I. % | Modification | Rank | Result Type |
|---|---|---|---|---|---|---|---|---|---|---|---|
| 1859.9299 | 1859.9314 | 0.0015 | 1 | 69 | 83 | GLKYNQATPNFHQWR | | | | | Mascot |
| 1859.9718 | 1859.9314 | -0.0404 | -22 | 53 | 68 | KMQTDQQVVINCPIVK | | | Oxidation (M)[2] | | Mascot |
| 2176.9849 | 2176.9849 | 0 | 0 | 2 | 21 | SESSICQARATVMIYDDGSK | | | Oxidation (M)[13] | | Mascot |
| 2283.9705 | 2284.2034 | 0.2329 | 102 | 366 | 386 | MKPVSQSAEAESGEGESEMER | | | Oxidation (M)[1] | | Mascot |
| 2683.1868 | 2683.2405 | 0.0537 | 20 | 292 | 322 | NSVSMPSGGGGGGGGGGGGGLMGEMSAILAR | | | Oxidation (M)[5,22,25] | | Mascot |

| | | | | | | | | |
|---|---|---|---|---|---|---|---|---|
| 5 | Zgc:110347 [Danio rerio] | gi\|62204405 | 21385.7 | 8.64 | 7 | 44 | 0 |

**Peptide Information**

| Calc. Mass | Obsrv. Mass | ± da | ± ppm | Start Seq. | End Seq. | Sequence | Ion Score | C. I. % | Modification | Rank | Result Type |
|---|---|---|---|---|---|---|---|---|---|---|---|
| 980.4465 | 980.4255 | -0.021 | -21 | 2 | 10 | SESSICQAR | | | | | Mascot |
| 1111.4871 | 1111.5952 | 0.1081 | 97 | 1 | 10 | MSESSICQAR | | | | | Mascot |
| 1501.791 | 1501.6599 | -0.1311 | -87 | 22 | 35 | KWVPAGTGPQAFSR | | | | | Mascot |
| 1501.791 | 1501.6599 | -0.1311 | -87 | 22 | 35 | KWVPAGTGPQAFSR | | | | | Mascot |
| 1561.7295 | 1561.8402 | 0.1107 | 71 | 72 | 83 | YNQATPNFHQWR | | | | | Mascot |
| 1859.9299 | 1859.9314 | 0.0015 | 1 | 69 | 83 | GLKYNQATPNFHQWR | | | | | Mascot |
| 1859.9718 | 1859.9314 | -0.0404 | -22 | 53 | 68 | KMQTDQQVVINCPIVK | | | Oxidation (M)[2] | | Mascot |
| 2176.9849 | 2176.9849 | 0 | 0 | 2 | 21 | SESSICQARATVMIYDDGSK | | | Oxidation (M)[13] | | Mascot |

| | | | | | | | | |
|---|---|---|---|---|---|---|---|---|
| 6 | vasodilator-stimulated phosphoprotein [Danio rerio] | gi\|237681322 | 46817.5 | 8.74 | 10 | 43 | 0 |

**Peptide Information**

| Calc. Mass | Obsrv. Mass | ± da | ± ppm | Start Seq. | End Seq. | Sequence | Ion Score | C. I. % | Modification | Rank | Result Type |
|---|---|---|---|---|---|---|---|---|---|---|---|
| 980.4465 | 980.4255 | -0.021 | -21 | 2 | 10 | SESSICQAR | | | | | Mascot |
| 1111.4871 | 1111.5952 | 0.1081 | 97 | 1 | 10 | MSESSICQAR | | | | | Mascot |
| 1501.791 | 1501.6599 | -0.1311 | -87 | 22 | 35 | KWVPAGTGPQAFSR | | | | | Mascot |
| 1501.791 | 1501.6599 | -0.1311 | -87 | 22 | 35 | KWVPAGTGPQAFSR | | | | | Mascot |
| 1502.8213 | 1502.7963 | -0.025 | -17 | 429 | 441 | NEIIGAFIQELQK | | | | | Mascot |
| 1561.7295 | 1561.8402 | 0.1107 | 71 | 72 | 83 | YNQATPNFHQWR | | | | | Mascot |
| 1658.9224 | 1658.9414 | 0.019 | 11 | 429 | 442 | NEIIGAFIQELQKR | | | | | Mascot |
| 1658.9224 | 1658.9414 | 0.019 | 11 | 429 | 442 | NEIIGAFIQELQKR | | | | | Mascot |
| 1859.9299 | 1859.9314 | 0.0015 | 1 | 69 | 83 | GLKYNQATPNFHQWR | | | | | Mascot |
| 1859.9718 | 1859.9314 | -0.0404 | -22 | 53 | 68 | KMQTDQQVVINCPIVK | | | Oxidation (M)[2] | | Mascot |
| 2176.9849 | 2176.9849 | 0 | 0 | 2 | 21 | SESSICQARATVMIYDDGSK | | | Oxidation (M)[13] | | Mascot |
| 2283.9705 | 2284.2034 | 0.2329 | 102 | 391 | 411 | MKPVSQSAEAESGEGESEMER | | | Oxidation (M)[1] | | Mascot |

| | | | | | | | | |
|---|---|---|---|---|---|---|---|---|
| 7 | novel NACHT domain containing protein [Danio rerio] | gi\|157886383 | 55615.1 | 5.32 | 10 | 42 | 0 |

Peptide Information

| Calc. Mass | Obsrv. Mass | ± da | ± ppm | Start Seq. | End Seq. | Sequence | Ion Score | C. I. % | Modification | Rank | Result Type |
|---|---|---|---|---|---|---|---|---|---|---|---|
| 964.5284 | 964.4287 | -0.0997 | -103 | 410 | 417 | VAFQQLMK | | | | | Mascot |
| 980.5233 | 980.4255 | -0.0978 | -100 | 410 | 417 | VAFQQLMK | | | Oxidation (M)[7] | | Mascot |
| 1077.5497 | 1077.5269 | -0.0228 | -21 | 398 | 406 | LTDEDMILK | | | | | Mascot |
| 1139.4819 | 1139.5138 | 0.0319 | 28 | 39 | 48 | SSEPSCVSMR | | | Carbamidomethyl (C)[6] | | Mascot |
| 1280.5245 | 1280.6072 | 0.0827 | 65 | 3 | 13 | SDPEPSCVSMR | | | Carbamidomethyl (C)[7], Oxidation (M)[10] | | Mascot |
| 1322.5504 | 1322.6648 | 0.1144 | 86 | 14 | 24 | SDSMDQPMHFK | | | | | Mascot |
| 1363.6094 | 1363.6885 | 0.0791 | 58 | 2 | 13 | RSDPEPSCVSMR | | | | | Mascot |
| 1363.6094 | 1363.6885 | 0.0791 | 58 | 2 | 13 | RSDPEPSCVSMR | | | | | Mascot |
| 1379.6042 | 1379.6506 | 0.0464 | 34 | 2 | 13 | RSDPEPSCVSMR | | | Oxidation (M)[11] | | Mascot |
| 1483.725 | 1483.6519 | -0.0731 | -49 | 234 | 245 | VLFIFDGLDECR | | | Carbamidomethyl (C)[11] | | Mascot |
| 2318.0791 | 2318.2329 | 0.1538 | 66 | 429 | 449 | ECGIDVAEASVYSGLCTQ IFR | | | Carbamidomethyl (C)[2] | | Mascot |
| 2661.2388 | 2661.2351 | -0.0037 | -1 | 49 | 72 | SDDESVHQPLHVKSGDT ETDLSHK | | | | | Mascot |
| 3364.7532 | 3364.6116 | -0.1416 | -42 | 361 | 389 | MLSEAETAEIPKTLTQMY THFLILQTNIK | | | | | Mascot |

| | | | | | | | |
|---|---|---|---|---|---|---|---|
| 8 | GR [Danio rerio] | gi\|68132008 | 35860.4 | 9.13 | 11 | 42 | 0 |

Peptide Information

| Calc. Mass | Obsrv. Mass | ± da | ± ppm | Start Seq. | End Seq. | Sequence | Ion Score | C. I. % | Modification | Rank | Result Type |
|---|---|---|---|---|---|---|---|---|---|---|---|
| 877.4083 | 877.423 | 0.0147 | 17 | 16 | 22 | NDCIIDK | | | Carbamidomethyl (C)[3] | | Mascot |
| 1045.5941 | 1045.5701 | -0.024 | -23 | 135 | 143 | WAKALPGFR | | | | | Mascot |
| 1351.645 | 1351.7147 | 0.0697 | 52 | 302 | 313 | SLSVEFPEMLAE | | | | | Mascot |
| 1352.6483 | 1352.7048 | 0.0565 | 42 | 35 | 46 | KCLMAGMNLEAR | | | Oxidation (M)[4] | | Mascot |
| 1352.6483 | 1352.7048 | 0.0565 | 42 | 36 | 47 | CLMAGMNLEARK | | | Oxidation (M)[3] | | Mascot |
| 1474.6855 | 1474.6882 | 0.0027 | 2 | 3 | 15 | AVEGQHNYLCAGR | | | Carbamidomethyl (C)[10] | | Mascot |
| 1658.7223 | 1658.9414 | 0.2191 | 132 | 194 | 206 | LPYMSDQCEQMLK | | | Carbamidomethyl (C)[8], Oxidation (M)[4] | | Mascot |
| 1658.7223 | 1658.9414 | 0.2191 | 132 | 194 | 206 | LPYMSDQCEQMLK | | | Carbamidomethyl (C)[8], Oxidation (M)[4] | | Mascot |
| 1860.8363 | 1860.9238 | 0.0875 | 47 | 192 | 206 | MKLPYMSDQCEQMLK | | | Oxidation (M)[1] | | Mascot |
| 2284.0979 | 2284.2034 | 0.1055 | 46 | 96 | 116 | AIEPDTLYAGYDSTIPDTS VR | | | | | Mascot |
| 2362.1416 | 2362.1172 | -0.0244 | -10 | 207 | 225 | ISNEFVRLQVSTEEYLCM K | | | Carbamidomethyl (C)[17], Oxidation (M)[18] | | Mascot |
| 2683.1697 | 2683.2405 | 0.0708 | 26 | 170 | 191 | SYQHCNGNMLCFAPDLV INEER | | | Carbamidomethyl (C)[5,11], Oxidation (M)[9] | | Mascot |

| | | | | | | | |
|---|---|---|---|---|---|---|---|
| 9 | novel NACHT domain containing protein [Danio rerio] | gi\|157886384 | 52335.5 | 5.14 | 10 | 42 | 0 |

Peptide Information

| Calc. Mass | Obsrv. Mass | ± da | ± ppm | Start Seq. | End Seq. | Sequence | Ion Score | C. I. % | Modification | Rank | Result Type |
|---|---|---|---|---|---|---|---|---|---|---|---|
| 964.5284 | 964.4287 | -0.0997 | -103 | 412 | 419 | VAFQQLMK | | | | | Mascot |
| 980.5233 | 980.4255 | -0.0978 | -100 | 412 | 419 | VAFQQLMK | | | Oxidation (M)[7] | | Mascot |
| 1077.5497 | 1077.5269 | -0.0228 | -21 | 400 | 408 | LTDEDMILK | | | | | Mascot |
| 1151.4819 | 1151.6522 | 0.1703 | 148 | 42 | 51 | EADPSCVSMR | | | Carbamidomethyl (C)[6] | | Mascot |
| 1423.6886 | 1423.7625 | 0.0739 | 52 | 52 | 63 | SDTFMNQPINIK | | | Oxidation (M)[5] | | Mascot |
| 1483.725 | 1483.6519 | -0.0731 | -49 | 236 | 247 | VLFIFDGLDECR | | | Carbamidomethyl (C)[11] | | Mascot |
| 1545.7842 | 1545.8455 | 0.0613 | 40 | 145 | 157 | CRDIFTPLPGQNK | | | Carbamidomethyl (C)[1] | | Mascot |
| 1561.7275 | 1561.8402 | 0.1127 | 72 | 3 | 16 | SEPEPSCVSIRSDR | | | | | Mascot |
| 1658.7769 | 1658.9414 | 0.1645 | 99 | 26 | 39 | SEDTQTHLSTFHQK | | | | | Mascot |
| 1658.7769 | 1658.9414 | 0.1645 | 99 | 26 | 39 | SEDTQTHLSTFHQK | | | | | Mascot |
| 2318.0791 | 2318.2329 | 0.1538 | 66 | 431 | 451 | ECGIDVAEASVYSGLCTQ IFR | | | Carbamidomethyl (C)[2] | | Mascot |
| 3364.7532 | 3364.6116 | -0.1416 | -42 | 363 | 391 | MLSEAETAEIPKTLTQMY THFLILQTNIK | | | | | Mascot |

10 PREDICTED: copine-4-like [Danio rerio] gi|189530019 62246.7 5.53 11 39 0

Peptide Information

| Calc. Mass | Obsrv. Mass | ± da | ± ppm | Start Seq. | End Seq. | Sequence | Ion Score | C. I. % | Modification | Rank | Result Type |
|---|---|---|---|---|---|---|---|---|---|---|---|
| 996.4819 | 996.6053 | 0.1234 | 124 | 66 | 74 | SCINPTFSK | | | | | Mascot |
| 1100.5345 | 1100.6183 | 0.0838 | 76 | 354 | 363 | MFPAFGFGAR | | | | | Mascot |
| 1139.5731 | 1139.5138 | -0.0593 | -52 | 172 | 180 | SDPFLEIYR | | | | | Mascot |
| 1363.6311 | 1363.6885 | 0.0574 | 42 | 417 | 428 | VANFASEEMHTK | | | | | Mascot |
| 1363.6311 | 1363.6885 | 0.0574 | 42 | 417 | 428 | VANFASEEMHTK | | | | | Mascot |
| 1379.626 | 1379.6506 | 0.0246 | 18 | 417 | 428 | VANFASEEMHTK | | | Oxidation (M)[9] | | Mascot |
| 1483.6271 | 1483.6519 | 0.0248 | 17 | 541 | 553 | CPSEFESSRTFGH | | | | | Mascot |
| 1501.6852 | 1501.6599 | -0.0253 | -17 | 49 | 60 | MQSHGQWLEVDR | | | Oxidation (M)[1] | | Mascot |
| 1501.6852 | 1501.6599 | -0.0253 | -17 | 49 | 60 | MQSHGQWLEVDR | | | Oxidation (M)[1] | | Mascot |
| 1545.7729 | 1545.8455 | 0.0726 | 47 | 193 | 205 | TETVMNNLNPVWK | | | | | Mascot |
| 1561.7679 | 1561.8402 | 0.0723 | 46 | 193 | 205 | TETVMNNLNPVWK | | | Oxidation (M)[5] | | Mascot |
| 1593.8417 | 1593.7393 | -0.1024 | -64 | 273 | 286 | NYKNSGIVILNQCK | | | | | Mascot |
| 1593.8417 | 1593.7393 | -0.1024 | -64 | 273 | 286 | NYKNSGIVILNQCK | | | | | Mascot |
| 1721.8315 | 1721.8103 | -0.0212 | -12 | 222 | 235 | ILKCTVWDWDSNGK | | | Carbamidomethyl (C)[4] | | Mascot |
| 2225.0359 | 2225.1482 | 0.1123 | 50 | 104 | 123 | EADCLGAMECTLGQIVS QRK | | | Carbamidomethyl (C)[4], Oxidation (M)[8] | | Mascot |
| 2661.3149 | 2661.2351 | -0.0798 | -30 | 429 | 451 | EAMQYFILLILTDGVITDM ADTR | | | Oxidation (M)[3,19] | | Mascot |

| | | | | | | | | |
|---|---|---|---|---|---|---|---|---|
| 11 | PREDICTED: zinc finger protein 729-like [Danio rerio] | gi\|326665698 | 111881.8 | 9.22 | 19 | 38 | 0 | |

**Peptide Information**

| Calc. Mass | Obsrv. Mass | ± da | ± ppm | Start Seq. | End Seq. | Sequence | Ion Score | C. I. % | Modification | Rank | Result Type |
|---|---|---|---|---|---|---|---|---|---|---|---|
| 882.4791 | 882.575 | 0.0959 | 109 | 858 | 864 | NLITHER | | | | | Mascot |
| 1064.6034 | 1064.5403 | -0.0631 | -59 | 694 | 702 | HMLIHTGKK | | | | | Mascot |
| 1100.5847 | 1100.6183 | 0.0336 | 31 | 638 | 646 | IHTGERPYK | | | | | Mascot |
| 1111.5854 | 1111.5952 | 0.0098 | 9 | 715 | 724 | LSSGLNDHLR | | | | | Mascot |
| 1263.502 | 1263.6631 | 0.1611 | 128 | 365 | 373 | EYVCFECEK | | | Carbamidomethyl (C)[4,7] | | Mascot |
| 1263.502 | 1263.6631 | 0.1611 | 128 | 365 | 373 | EYVCFECEK | | | Carbamidomethyl (C)[4,7] | | Mascot |
| 1322.7063 | 1322.6648 | -0.0415 | -31 | 955 | 965 | QVRFSQSETLK | | | | | Mascot |
| 1352.5834 | 1352.7048 | 0.1214 | 90 | 703 | 714 | AHTCDQCGKSFR | | | | | Mascot |
| 1352.5834 | 1352.7048 | 0.1214 | 90 | 703 | 714 | AHTCDQCGKSFR | | | | | Mascot |
| 1379.5203 | 1379.6506 | 0.1303 | 94 | 16 | 27 | DSENMSDPEPCR | | | | | Mascot |
| 1501.787 | 1501.6599 | -0.1271 | -85 | 712 | 724 | SFRLSSGLNDHLR | | | | | Mascot |
| 1501.787 | 1501.6599 | -0.1271 | -85 | 712 | 724 | SFRLSSGLNDHLR | | | | | Mascot |
| 1502.709 | 1502.7963 | 0.0873 | 58 | 527 | 540 | VHATEKPSCSVCGK | | | Carbamidomethyl (C)[9] | | Mascot |
| 1529.8435 | 1529.8517 | 0.0082 | 5 | 374 | 386 | TFITSGELKLHQR | | | | | Mascot |
| 1545.7227 | 1545.8455 | 0.1228 | 79 | 346 | 358 | SFGRSSQHDIHMK | | | Oxidation (M)[12] | | Mascot |
| 1658.886 | 1658.9414 | 0.0554 | 33 | 2 | 15 | QEYSHQAQVKSLLK | | | | | Mascot |
| 1658.886 | 1658.9414 | 0.0554 | 33 | 2 | 15 | QEYSHQAQVKSLLK | | | | | Mascot |
| 1737.8047 | 1737.8036 | -0.0011 | -1 | 554 | 568 | IHTGVREYVCSECGK | | | Carbamidomethyl (C)[10] | | Mascot |
| 1751.8315 | 1751.8743 | 0.0428 | 24 | 315 | 330 | CGKGFSQSSSLNLHMR | | | | | Mascot |
| 1791.8629 | 1791.9619 | 0.099 | 55 | 303 | 317 | IHTGEKPFTCTRCGK | | | Carbamidomethyl (C)[10,13] | | Mascot |
| 1800.7437 | 1800.8799 | 0.1362 | 76 | 790 | 804 | CSHCDMRFSWLGCQK | | | | | Mascot |
| 1807.8279 | 1807.9532 | 0.1253 | 69 | 958 | 972 | FSQSETLKNTQECHV | | | Carbamidomethyl (C)[13] | | Mascot |
| 1857.7651 | 1857.9545 | 0.1894 | 102 | 790 | 804 | CSHCDMRFSWLGCQK | | | Carbamidomethyl (C)[1] | | Mascot |
| 2545.2727 | 2545.3594 | 0.0867 | 34 | 318 | 339 | GFSQSSSLNLHMRIHTGE KPFR | | | Oxidation (M)[12] | | Mascot |
| 2545.2727 | 2545.3594 | 0.0867 | 34 | 318 | 339 | GFSQSSSLNLHMRIHTGE KPFR | | | Oxidation (M)[12] | | Mascot |

| | | | | | | | | |
|---|---|---|---|---|---|---|---|---|
| 12 | PREDICTED: protein NLRC3 [Danio rerio] | gi\|189529139 | 76683.4 | 5.89 | 11 | 38 | 0 | |

**Peptide Information**

| Calc. Mass | Obsrv. Mass | ± da | ± ppm | Start Seq. | End Seq. | Sequence | Ion Score | C. I. % | Modification | Rank | Result Type |
|---|---|---|---|---|---|---|---|---|---|---|---|
| 964.5284 | 964.4287 | -0.0997 | -103 | 431 | 438 | VAFQQLMK | | | | | Mascot |
| 980.5233 | 980.4255 | -0.0978 | -100 | 431 | 438 | VAFQQLMK | | | Oxidation (M)[7] | | Mascot |

| Calc. Mass | Obsrv. Mass | ± da | ± ppm | Start Seq. | End Seq. | Sequence | Ion Score | C. I. % | Modification | Rank | Result Type |
|---|---|---|---|---|---|---|---|---|---|---|---|
| 1077.5497 | 1077.5269 | -0.0228 | -21 | 419 | 427 | LTDEDMILK | | | | | Mascot |
| 1139.4819 | 1139.5138 | 0.0319 | 28 | 60 | 69 | SSEPSCVSMR | | | Carbamidomethyl (C)[6] | | Mascot |
| 1280.5245 | 1280.6072 | 0.0827 | 65 | 24 | 34 | SDPEPSCVSMR | | | Carbamidomethyl (C)[7], Oxidation (M)[10] | | Mascot |
| 1322.5504 | 1322.6648 | 0.1144 | 86 | 35 | 45 | SDSMDQPMHFK | | | | | Mascot |
| 1363.6094 | 1363.6885 | 0.0791 | 58 | 23 | 34 | RSDPEPSCVSMR | | | | | Mascot |
| 1363.6094 | 1363.6885 | 0.0791 | 58 | 23 | 34 | RSDPEPSCVSMR | | | | | Mascot |
| 1379.6042 | 1379.6506 | 0.0464 | 34 | 23 | 34 | RSDPEPSCVSMR | | | Oxidation (M)[11] | | Mascot |
| 1483.725 | 1483.6519 | -0.0731 | -49 | 255 | 266 | VLFIFDGLDECR | | | Carbamidomethyl (C)[11] | | Mascot |
| 1751.8599 | 1751.8743 | 0.0144 | 8 | 507 | 520 | DEFEQINKQSLWSK | | | | | Mascot |
| 2318.0791 | 2318.2329 | 0.1538 | 66 | 450 | 470 | ECGIDVAEASVYSGLCTQ IFR | | | Carbamidomethyl (C)[2] | | Mascot |
| 2661.2388 | 2661.2351 | -0.0037 | -1 | 70 | 93 | SDDESVHQPLHVKSGDT ETDLSHK | | | | | Mascot |
| 3364.7532 | 3364.6116 | -0.1416 | -42 | 382 | 410 | MLSEAETAEIPKTLTQMY THFLILQTNIK | | | | | Mascot |

| | | | | | | | |
|---|---|---|---|---|---|---|---|
| 13 | DBF4 homolog [Danio rerio] | gi\|205830371 | 69253.7 | 9.28 | 12 | 38 | 0 |

**Peptide Information**

| Calc. Mass | Obsrv. Mass | ± da | ± ppm | Start Seq. | End Seq. | Sequence | Ion Score | C. I. % | Modification | Rank | Result Type |
|---|---|---|---|---|---|---|---|---|---|---|---|
| 1045.4731 | 1045.5701 | 0.097 | 93 | 16 | 25 | LEGHNCASSK | | | | | Mascot |
| 1165.6034 | 1165.7551 | 0.1517 | 130 | 78 | 87 | YLVSCKPEAR | | | | | Mascot |
| 1275.5166 | 1275.6111 | 0.0945 | 74 | 290 | 300 | KGGYCECCEVK | | | Carbamidomethyl (C)[5] | | Mascot |
| 1280.6304 | 1280.6072 | -0.0232 | -18 | 30 | 40 | STCDLQNKPFK | | | | | Mascot |
| 1483.8156 | 1483.6519 | -0.1637 | -110 | 583 | 595 | SEKPPASLLDLWK | | | | | Mascot |
| 1501.7897 | 1501.6599 | -0.1298 | -86 | 164 | 176 | VLHVDDVISYVDK | | | | | Mascot |
| 1501.7897 | 1501.6599 | -0.1298 | -86 | 164 | 176 | VLHVDDVISYVDK | | | | | Mascot |
| 1593.8092 | 1593.7393 | -0.0699 | -44 | 554 | 567 | QKQVSASHIESSHR | | | | | Mascot |
| 1593.8092 | 1593.7393 | -0.0699 | -44 | 554 | 567 | QKQVSASHIESSHR | | | | | Mascot |
| 1658.8245 | 1658.9414 | 0.1169 | 70 | 190 | 204 | GAASIQHTEKNSYPR | | | | | Mascot |
| 1658.8245 | 1658.9414 | 0.1169 | 70 | 190 | 204 | GAASIQHTEKNSYPR | 3 | 0 | | | Mascot |
| 1721.7332 | 1721.8103 | 0.0771 | 45 | 291 | 305 | GGYCECCEVKFENLK | | | | | Mascot |
| 1791.8979 | 1791.9619 | 0.064 | 36 | 328 | 344 | VTAGLTCDLVNISTPCK | | | Carbamidomethyl (C)[7] | | Mascot |
| 1859.012 | 1858.9478 | -0.0642 | -35 | 54 | 70 | TQLLENDIKTLGGTVEK | | | | | Mascot |
| 2224.989 | 2225.1482 | 0.1592 | 72 | 241 | 259 | SFPPCSPFLMEEQGKDD PK | | | Carbamidomethyl (C)[5], Oxidation (M)[10] | | Mascot |

| | | | | | | | |
|---|---|---|---|---|---|---|---|
| 14 | novel protein similar to vertebrate beta-1,4-N-acetyl-galactosaminyl transferase 3 (B4GALNT3) [Dani | gi\|220672916 | 50299.9 | 5.53 | 9 | 37 | 0 |

**Peptide Information**

| Calc. Mass | Obsrv. Mass | ± da | ± ppm | Start Seq. | End Seq. | Sequence | Ion Score | C. I. % | Modification | Rank | Result Type |
|---|---|---|---|---|---|---|---|---|---|---|---|
| 964.4119 | 964.4287 | 0.0168 | 17 | 186 | 193 | TDDWGSQR | | | | | Mascot |
| 1322.6368 | 1322.6648 | 0.028 | 21 | 366 | 377 | SDLDRIGGMNTK | | | Oxidation (M)[9] | | Mascot |
| 1474.8013 | 1474.6882 | -0.1131 | -77 | 146 | 157 | IVNVEKKPDYNR | | | | | Mascot |
| 1545.7809 | 1545.8455 | 0.0646 | 42 | 276 | 287 | YQYLRLSGNFER | | | | | Mascot |
| 1658.9587 | 1658.9414 | -0.0173 | -10 | 394 | 407 | ILQGGLEVERLYLR | | | | | Mascot |
| 1658.9587 | 1658.9414 | -0.0173 | -10 | 394 | 407 | ILQGGLEVERLYLR | | | | | Mascot |
| 1791.96 | 1791.9619 | 0.0019 | 1 | 158 | 173 | GSRYLLELDLLEASGR | | | | | Mascot |
| 1857.9528 | 1857.9545 | 0.0017 | 1 | 94 | 108 | LLDLHSMRSDWIDLK | | | Oxidation (M)[7] | | Mascot |
| 1859.8997 | 1859.9314 | 0.0317 | 17 | 230 | 243 | WVKQFISDMEEIYR | | | Oxidation (M)[9] | | Mascot |
| 1859.8997 | 1859.9314 | 0.0317 | 17 | 230 | 243 | WVKQFISDMEEIYR | | | Oxidation (M)[9] | | Mascot |
| 2661.2585 | 2661.2351 | -0.0234 | -9 | 27 | 49 | WGHANEDRNGVEKPIQD NAMHVK | | | Oxidation (M)[20] | | Mascot |

| | | | | | | | |
|---|---|---|---|---|---|---|---|
| 15 | novel protein similar to human and rodent member RAS oncogene family RAB7 (RAB7) [Danio rerio] | gi\|33468618 | 19809.1 | 6.58 | 6 | 37 | 0 |

Peptide Information

| Calc. Mass | Obsrv. Mass | ± da | ± ppm | Start Seq. | End Seq. | Sequence | Ion Score | C. I. % | Modification | Rank | Result Type |
|---|---|---|---|---|---|---|---|---|---|---|---|
| 1064.5986 | 1064.5403 | -0.0583 | -55 | 39 | 48 | ATIGVDFLTK | | | | | Mascot |
| 1363.7117 | 1363.6885 | -0.0232 | -17 | 165 | 176 | TFHSVARAALQY | | | | | Mascot |
| 1363.7117 | 1363.6885 | -0.0232 | -17 | 165 | 176 | TFHSVARAALQY | | | | | Mascot |
| 1859.0061 | 1858.9478 | -0.0583 | -31 | 33 | 48 | FTNLYRATIGVDFLTK | | | | | Mascot |
| 2031.948 | 2032.0435 | 0.0955 | 47 | 140 | 157 | AQKWCVDIGAEYFESSA K | | | | | Mascot |
| 2177.2078 | 2176.9849 | -0.2229 | -102 | 2 | 21 | TEWNTPHLKLILIGNSGV GK | | | | | Mascot |
| 2683.2893 | 2683.2405 | -0.0488 | -18 | 81 | 104 | GAHCCLLVFDVTSSVSFE ALDVWK | | | Carbamidomethyl (C)[4] | | Mascot |

| | | | | | | | |
|---|---|---|---|---|---|---|---|
| 16 | PREDICTED: hypothetical protein LOC100537292 [Danio rerio] | gi\|326672395 | 272747.6 | 8.49 | 22 | 37 | 0 |

Peptide Information

| Calc. Mass | Obsrv. Mass | ± da | ± ppm | Start Seq. | End Seq. | Sequence | Ion Score | C. I. % | Modification | Rank | Result Type |
|---|---|---|---|---|---|---|---|---|---|---|---|
| 1045.5889 | 1045.5701 | -0.0188 | -18 | 755 | 763 | ILIQNETSK | | | | | Mascot |
| 1064.4751 | 1064.5403 | 0.0652 | 61 | 232 | 241 | MSCPDAEALK | | | | | Mascot |
| 1100.6058 | 1100.6183 | 0.0125 | 11 | 2252 | 2260 | KVENVEQVR | | | | | Mascot |
| 1139.5803 | 1139.5138 | -0.0665 | -58 | 1247 | 1256 | HNIDADVLSR | | | | | Mascot |
| 1275.582 | 1275.6111 | 0.0291 | 23 | 477 | 488 | CGEEGHIATKCK | | | | | Mascot |

| | | | | | | | | | | | |
|---|---|---|---|---|---|---|---|---|---|---|---|
| 1352.6298 | 1352.7048 | 0.075 | 55 | 391 | 402 | SNIASSMMIENR | | | | | Mascot |
| 1352.6567 | 1352.7048 | 0.0481 | 36 | 1082 | 1092 | SFLGFCGFYRR | | | | | Mascot |
| 1474.7471 | 1474.6882 | -0.0589 | -40 | 972 | 985 | MPQGITGAPATFQR | | | | | Mascot |
| 1501.8295 | 1501.6599 | -0.1696 | -113 | 1573 | 1586 | TLLSMLGTLEPAQK | | | | | Mascot |
| 1501.8295 | 1501.6599 | -0.1696 | -113 | 2046 | 2059 | TLLSMLGTLEPAQK | | | | | Mascot |
| 1593.7577 | 1593.7393 | -0.0184 | -12 | 1277 | 1290 | AICQLSNLSWSDEK | | | | | Mascot |
| 1593.7908 | 1593.7393 | -0.0515 | -32 | 824 | 837 | NVFSTQEWDVGLAK | | | | | Mascot |
| 1658.707 | 1658.9414 | 0.2344 | 141 | 199 | 211 | LMISECECSEKEK | | | Carbamidomethyl (C)[6,8], Oxidation (M)[2] | | Mascot |
| 1658.707 | 1658.9414 | 0.2344 | 141 | 199 | 211 | LMISECECSEKEK | | | Carbamidomethyl (C)[6,8], Oxidation (M)[2] | | Mascot |
| 1716.9564 | 1717.0227 | 0.0663 | 39 | 2046 | 2061 | TLLSMLGTLEPAQKSK | | | | | Mascot |
| 1721.8857 | 1721.8103 | -0.0754 | -44 | 823 | 837 | KNVFSTQEWDVGLAK | | | | | Mascot |
| 1751.8058 | 1751.8743 | 0.0685 | 39 | 1136 | 1149 | WDNACEQAFQEIIK | | | Carbamidomethyl (C)[5] | | Mascot |
| 1808.0792 | 1807.9532 | -0.126 | -70 | 1390 | 1404 | VQLVLPEKYHLTVLR | | | | | Mascot |
| 1860.8981 | 1860.9238 | 0.0257 | 14 | 654 | 670 | MTNGEGTNITRSAHSLR | | | Oxidation (M)[1] | | Mascot |
| 2016.0198 | 2016.0427 | 0.0229 | 11 | 1992 | 2008 | FFVHYGLPSRIHSDQGR | | | | | Mascot |
| 2225.0479 | 2225.1482 | 0.1003 | 45 | 1 | 18 | MSFRSDPQLQSELLEWC R | | | | | Mascot |
| 2233.168 | 2233.1099 | -0.0581 | -26 | 304 | 322 | VEQLIRGAVESDMMLLQL R | | | Oxidation (M)[13,14] | | Mascot |
| 2283.2244 | 2283.2114 | -0.013 | -6 | 1199 | 1217 | LSASEQRYHIHQLEFLAL K | | | | | Mascot |
| 2379.1497 | 2379.3738 | 0.2241 | 94 | 913 | 934 | TTPDQYVTPRIDDALDCL AGSK | | | | | Mascot |
| 2379.1497 | 2379.3738 | 0.2241 | 94 | 913 | 934 | TTPDQYVTPRIDDALDCL AGSK | | | | | Mascot |

17 PREDICTED: protein NLRC3 [Danio rerio] gi|326674410 79200.8 6.13 11 36 0

**Peptide Information**

| Calc. Mass | Obsrv. Mass | ± da | ± ppm | Start Seq. | End Seq. | Sequence | Ion Score | C. I. % | Modification | Rank | Result Type |
|---|---|---|---|---|---|---|---|---|---|---|---|
| 964.5284 | 964.4287 | -0.0997 | -103 | 428 | 435 | VAFQQLMK | | | | | Mascot |
| 980.5233 | 980.4255 | -0.0978 | -100 | 428 | 435 | VAFQQLMK | | | Oxidation (M)[7] | | Mascot |
| 1077.5497 | 1077.5269 | -0.0228 | -21 | 416 | 424 | LTDEDMILK | | | | | Mascot |
| 1151.4819 | 1151.6522 | 0.1703 | 148 | 58 | 67 | EADPSCVSMR | | | Carbamidomethyl (C)[6] | | Mascot |
| 1423.6886 | 1423.7625 | 0.0739 | 52 | 68 | 79 | SDTFMNQPINIK | | | Oxidation (M)[5] | | Mascot |
| 1483.725 | 1483.6519 | -0.0731 | -49 | 252 | 263 | VLFIFDGLDECR | | | Carbamidomethyl (C)[11] | | Mascot |
| 1545.7842 | 1545.8455 | 0.0613 | 40 | 161 | 173 | CRDIFTPLPGQNK | | | Carbamidomethyl (C)[1] | | Mascot |
| 1561.7275 | 1561.8402 | 0.1127 | 72 | 19 | 32 | SEPEPSCVSIRSDR | | | | | Mascot |
| 1658.7769 | 1658.9414 | 0.1645 | 99 | 42 | 55 | SEDTQTHLSTFHQK | | | | | Mascot |
| 1658.7769 | 1658.9414 | 0.1645 | 99 | 42 | 55 | SEDTQTHLSTFHQK | | | | | Mascot |

| Calc. Mass | Obsrv. Mass | ± da | ± ppm | Start Seq. | End Seq. | Sequence | Ion Score | C. I. % | Modification | Rank | Result Type |
|---|---|---|---|---|---|---|---|---|---|---|---|
| 2211.0459 | 2211.1294 | 0.0835 | 38 | 11 | 29 | VDSVHQERSEPEPSCVSIR | | | Carbamidomethyl (C)[15] | | Mascot |
| 2211.0459 | 2211.1294 | 0.0835 | 38 | 11 | 29 | VDSVHQERSEPEPSCVSIR | | | Carbamidomethyl (C)[15] | | Mascot |
| 2318.0791 | 2318.2329 | 0.1538 | 66 | 447 | 467 | ECGIDVAEASVYSGLCTQIFR | | | Carbamidomethyl (C)[2] | | Mascot |
| 3364.7532 | 3364.6116 | -0.1416 | -42 | 379 | 407 | MLSEAETAEIPKTLTQMYTHFLILQTNIK | | | | | Mascot |

18 annexin A1b [Danio rerio] gi|32308159 37416.3 6.11 8 36 0

**Peptide Information**

| Calc. Mass | Obsrv. Mass | ± da | ± ppm | Start Seq. | End Seq. | Sequence | Ion Score | C. I. % | Modification | Rank | Result Type |
|---|---|---|---|---|---|---|---|---|---|---|---|
| 1045.5061 | 1045.5701 | 0.064 | 61 | 201 | 209 | ALYEAGEHR | | | | | Mascot |
| 1064.5404 | 1064.5403 | -0.0001 | 0 | 141 | 149 | TNNEIKAMK | | | Oxidation (M)[8] | | Mascot |
| 1275.5979 | 1275.6111 | 0.0132 | 10 | 1 | 10 | MAFFNSFFHK | | | | | Mascot |
| 1483.8478 | 1483.6519 | -0.1959 | -132 | 55 | 68 | GVDEAAIIEVLAKR | | | | | Mascot |
| 1751.9075 | 1751.8743 | -0.0332 | -19 | 34 | 49 | FSQAFNAQNDAAVLKK | | | | | Mascot |
| 1860.8915 | 1860.9238 | 0.0323 | 17 | 2 | 17 | AFFNSFFHKISEDGSK | | | | | Mascot |
| 2177.127 | 2176.9849 | -0.1421 | -65 | 247 | 265 | ALESELHGHLEDCLLTIVK | | | Carbamidomethyl (C)[13] | | Mascot |
| 2545.2273 | 2545.3594 | 0.1321 | 52 | 307 | 328 | IMQEYSTMQGQSLQAAIQKETK | | | Oxidation (M)[2,8] | | Mascot |
| 2545.2273 | 2545.3594 | 0.1321 | 52 | 307 | 328 | IMQEYSTMQGQSLQAAIQKETK | | | Oxidation (M)[2,8] | | Mascot |

19 glucocorticoid receptor [Danio rerio] gi|99028943 81868.7 6.07 13 36 0

**Peptide Information**

| Calc. Mass | Obsrv. Mass | ± da | ± ppm | Start Seq. | End Seq. | Sequence | Ion Score | C. I. % | Modification | Rank | Result Type |
|---|---|---|---|---|---|---|---|---|---|---|---|
| 877.4083 | 877.423 | 0.0147 | 17 | 427 | 433 | NDCIIDK | | | Carbamidomethyl (C)[3] | | Mascot |
| 980.5047 | 980.4255 | -0.0792 | -81 | 16 | 23 | LNTLDYNK | | | | | Mascot |
| 1045.5941 | 1045.5701 | -0.024 | -23 | 546 | 554 | WAKALPGFR | | | | | Mascot |
| 1064.4677 | 1064.5403 | 0.0726 | 68 | 1 | 10 | MDQGGLENGK | | | Oxidation (M)[1] | | Mascot |
| 1352.6483 | 1352.7048 | 0.0565 | 42 | 446 | 457 | KCLMAGMNLEAR | | | Oxidation (M)[4] | | Mascot |
| 1352.6483 | 1352.7048 | 0.0565 | 42 | 447 | 458 | CLMAGMNLEARK | | | Oxidation (M)[3] | | Mascot |
| 1474.6855 | 1474.6882 | 0.0027 | 2 | 414 | 426 | AVEGQHNYLCAGR | | | Carbamidomethyl (C)[10] | | Mascot |
| 1658.7223 | 1658.9414 | 0.2191 | 132 | 605 | 617 | LPYMSDQCEQMLK | | | Carbamidomethyl (C)[8], Oxidation (M)[4] | | Mascot |
| 1658.7223 | 1658.9414 | 0.2191 | 132 | 605 | 617 | LPYMSDQCEQMLK | | | Carbamidomethyl (C)[8], Oxidation (M)[4] | | Mascot |
| 1860.8363 | 1860.9238 | 0.0875 | 47 | 603 | 617 | MKLPYMSDQCEQMLK | | | Oxidation (M)[1] | | Mascot |
| 2233.0002 | 2233.1099 | 0.1097 | 49 | 359 | 380 | NFSSSPYSRPEDSTATSSAGGK | | | | | Mascot |
| 2284.0979 | 2284.2034 | 0.1055 | 46 | 507 | 527 | AIEPDTLYAGYDSTIPDTSVR | | | | | Mascot |

| Calc. Mass | Obsrv. Mass | ± da | ± ppm | Start Seq. | End Seq. | Sequence | Ion Score | C. I. % | Modification | Rank | Result Type |
|---|---|---|---|---|---|---|---|---|---|---|---|
| 2362.1416 | 2362.1172 | -0.0244 | -10 | 618 | 636 | ISNEFVRLQVSTEEYLCMK | | | Carbamidomethyl (C)[17], Oxidation (M)[18] | | Mascot |
| 2683.2014 | 2683.2405 | 0.0391 | 15 | 144 | 167 | TEDFSPMIKGDMDLDQDSFGHIGK | | | | | Mascot |

20 SHC-transforming protein 2 [Danio rerio] gi|113678838 56008 7.05 10 35 0

**Peptide Information**

| Calc. Mass | Obsrv. Mass | ± da | ± ppm | Start Seq. | End Seq. | Sequence | Ion Score | C. I. % | Modification | Rank | Result Type |
|---|---|---|---|---|---|---|---|---|---|---|---|
| 877.4811 | 877.423 | -0.0581 | -66 | 96 | 103 | TLQSIMGK | | | | | Mascot |
| 1111.5742 | 1111.5952 | 0.021 | 19 | 247 | 257 | EPPVGGVVDSR | | | | | Mascot |
| 1352.728 | 1352.7048 | -0.0232 | -17 | 391 | 402 | RAPVAPTEDQLR | | | | | Mascot |
| 1352.728 | 1352.7048 | -0.0232 | -17 | 392 | 403 | APVAPTEDQLRR | | | | | Mascot |
| 1363.7362 | 1363.6885 | -0.0477 | -35 | 96 | 107 | TLQSIMGKSNLR | | | Oxidation (M)[6] | | Mascot |
| 1363.7362 | 1363.6885 | -0.0477 | -35 | 96 | 107 | TLQSIMGKSNLR | | | Oxidation (M)[6] | | Mascot |
| 1502.7164 | 1502.7963 | 0.0799 | 53 | 48 | 59 | YMGCIEVLKSMR | | | Carbamidomethyl (C)[4], Oxidation (M)[2] | | Mascot |
| 1518.7113 | 1518.7903 | 0.079 | 52 | 48 | 59 | YMGCIEVLKSMR | | | Carbamidomethyl (C)[4], Oxidation (M)[2,11] | | Mascot |
| 1529.774 | 1529.8517 | 0.0777 | 51 | 72 | 85 | EAINRLCESVPGGK | | | Carbamidomethyl (C)[7] | | Mascot |
| 1768.8687 | 1768.8936 | 0.0249 | 14 | 357 | 370 | KDIFDMRPFEDALR | | | Oxidation (M)[6] | | Mascot |
| 2177.0808 | 2176.9849 | -0.0959 | -44 | 371 | 389 | LHEASGVCVLEDKWPSPPR | | | Carbamidomethyl (C)[8] | | Mascot |
| 2284.1501 | 2284.2034 | 0.0533 | 23 | 203 | 222 | AIPAMDSHAEYPNILSRNIR | | | Oxidation (M)[5] | | Mascot |

21 glucocorticoid receptor beta [Danio rerio] gi|151177061 80782 6.02 13 34 0

**Peptide Information**

| Calc. Mass | Obsrv. Mass | ± da | ± ppm | Start Seq. | End Seq. | Sequence | Ion Score | C. I. % | Modification | Rank | Result Type |
|---|---|---|---|---|---|---|---|---|---|---|---|
| 877.4083 | 877.423 | 0.0147 | 17 | 427 | 433 | NDCIIDK | | | Carbamidomethyl (C)[3] | | Mascot |
| 980.5047 | 980.4255 | -0.0792 | -81 | 16 | 23 | LNTLDYNK | | | | | Mascot |
| 1045.5941 | 1045.5701 | -0.024 | -23 | 546 | 554 | WAKALPGFR | | | | | Mascot |
| 1064.4677 | 1064.5403 | 0.0726 | 68 | 1 | 10 | MDQGGLENGK | | | Oxidation (M)[1] | | Mascot |
| 1352.6483 | 1352.7048 | 0.0565 | 42 | 446 | 457 | KCLMAGMNLEAR | | | Oxidation (M)[4] | | Mascot |
| 1352.6483 | 1352.7048 | 0.0565 | 42 | 447 | 458 | CLMAGMNLEARK | | | Oxidation (M)[3] | | Mascot |
| 1474.6855 | 1474.6882 | 0.0027 | 2 | 414 | 426 | AVEGQHNYLCAGR | | | Carbamidomethyl (C)[10] | | Mascot |
| 1658.7223 | 1658.9414 | 0.2191 | 132 | 605 | 617 | LPYMSDQCEQMLK | | | Carbamidomethyl (C)[8], Oxidation (M)[4] | | Mascot |
| 1658.7223 | 1658.9414 | 0.2191 | 132 | 605 | 617 | LPYMSDQCEQMLK | | | Carbamidomethyl (C)[8], Oxidation (M)[4] | | Mascot |
| 1860.8363 | 1860.9238 | 0.0875 | 47 | 603 | 617 | MKLPYMSDQCEQMLK | | | Oxidation (M)[1] | | Mascot |
| 2233.0002 | 2233.1099 | 0.1097 | 49 | 359 | 380 | NFSSSPYSRPEDSTATSSAGGK | | | | | Mascot |
| 2284.0979 | 2284.2034 | 0.1055 | 46 | 507 | 527 | AIEPDTLYAGYDSTIPDTSVR | | | | | Mascot |

| Calc. Mass | Obsrv. Mass | ± da | ± ppm | Start Seq. | End Seq. | Sequence | Ion Score | C. I. % | Modification | Rank | Result Type |
|---|---|---|---|---|---|---|---|---|---|---|---|
| 2362.1416 | 2362.1172 | -0.0244 | -10 | 618 | 636 | ISNEFVRLQVSTEEYLCM K | | | Carbamidomethyl (C)[17], Oxidation (M)[18] | | Mascot |
| 2683.2014 | 2683.2405 | 0.0391 | 15 | 144 | 167 | TEDFSPMIKGDMDLDQD SFGHIGK | | | | | Mascot |

| | | | | | | | |
|---|---|---|---|---|---|---|---|
| 22 | finTRIM family protein [Danio rerio] | gi\|208019329 | 6388.3 | 6.95 | 4 | 34 | 0 |

Peptide Information

| Calc. Mass | Obsrv. Mass | ± da | ± ppm | Start Seq. | End Seq. | Sequence | Ion Score | C. I. % | Modification | Rank | Result Type |
|---|---|---|---|---|---|---|---|---|---|---|---|
| 1502.8247 | 1502.7963 | -0.0284 | -19 | 5 | 17 | NTMLAEVVEQLKK | | | | | Mascot |
| 1518.8196 | 1518.7903 | -0.0293 | -19 | 5 | 17 | NTMLAEVVEQLKK | | | Oxidation (M)[3] | | Mascot |
| 1800.9888 | 1800.8799 | -0.1089 | -60 | 1 | 16 | ALNKNTMLAEVVEQLK | | | | | Mascot |
| 1859.012 | 1858.9478 | -0.0642 | -35 | 43 | 60 | QSPQVLSGVSELLLSKSS | | | | | Mascot |
| 2210.9441 | 2211.1294 | 0.1853 | 84 | 20 | 40 | HHTAAVPAGDGDVECDV CTEK | | | Carbamidomethyl (C)[15] | | Mascot |
| 2210.9441 | 2211.1294 | 0.1853 | 84 | 20 | 40 | HHTAAVPAGDGDVECDV CTEK | | | Carbamidomethyl (C)[15] | | Mascot |

| | | | | | | | |
|---|---|---|---|---|---|---|---|
| 23 | Nop56 protein [Danio rerio] | gi\|34784843 | 52829.9 | 9.07 | 9 | 34 | 0 |

Peptide Information

| Calc. Mass | Obsrv. Mass | ± da | ± ppm | Start Seq. | End Seq. | Sequence | Ion Score | C. I. % | Modification | Rank | Result Type |
|---|---|---|---|---|---|---|---|---|---|---|---|
| 964.5574 | 964.4287 | -0.1287 | -133 | 369 | 376 | ISRYLANK | | | | | Mascot |
| 980.5676 | 980.4255 | -0.1421 | -145 | 128 | 135 | LHFHSLVK | | | | | Mascot |
| 1045.571 | 1045.5701 | -0.0009 | -1 | 91 | 100 | KPMLGVSDAK | | | | | Mascot |
| 1275.6943 | 1275.6111 | -0.0832 | -65 | 78 | 89 | LFLETNLPSGGK | | | | | Mascot |
| 1751.9591 | 1751.8743 | -0.0848 | -48 | 349 | 364 | YGLIFHSTFIGRAAAK | | | | | Mascot |
| 1808.0177 | 1807.9532 | -0.0645 | -36 | 128 | 144 | LHFHSLVKGLTAQAASK | | | | | Mascot |
| 2016.0621 | 2016.0427 | -0.0194 | -10 | 136 | 155 | GLTAQAASKAQLGLGHS YSR | | | | | Mascot |
| 2211.2358 | 2211.1294 | -0.1064 | -48 | 1 | 20 | MVLLHVLFEHAAGYALLA VK | | | Oxidation (M)[1] | | Mascot |
| 2211.2358 | 2211.1294 | -0.1064 | -48 | 1 | 20 | MVLLHVLFEHAAGYALLA VK | | | Oxidation (M)[1] | | Mascot |
| 2362.0347 | 2362.1172 | 0.0825 | 35 | 222 | 241 | KELSEEMLEAMEEITMDS AK | | | Oxidation (M)[7,11,16] | | Mascot |

| | | | | | | | |
|---|---|---|---|---|---|---|---|
| 24 | hypothetical protein LOC494576 [Danio rerio] | gi\|61806526 | 23120.7 | 8.19 | 6 | 33 | 0 |

Peptide Information

| Calc. Mass | Obsrv. Mass | ± da | ± ppm | Start Seq. | End Seq. | Sequence | Ion Score | C. I. % | Modification | Rank | Result Type |
|---|---|---|---|---|---|---|---|---|---|---|---|
| 1064.5986 | 1064.5403 | -0.0583 | -55 | 39 | 48 | ATIGVDFLTK | | | | | Mascot |
| 1545.7656 | 1545.8455 | 0.0799 | 52 | 157 | 170 | EDIGVDKTFHSAAR | | | | | Mascot |
| 1859.0061 | 1858.9478 | -0.0583 | -31 | 33 | 48 | FTNLYRATIGVDFLTK | | | | | Mascot |

| Calc. Mass | Obsrv. Mass | ± da | ± ppm | Start Seq. | End Seq. | Sequence | Ion Score | C. I. % | Modification | Rank | Result Type |
|---|---|---|---|---|---|---|---|---|---|---|---|
| 2031.948 | 2032.0435 | 0.0955 | 47 | 139 | 156 | AQKWCVDIGAEYFESSA K | | | | | Mascot |
| 2177.2078 | 2176.9849 | -0.2229 | -102 | 2 | 21 | TEWNTPHLKLILIGNSGV GK | | | | | Mascot |
| 2683.2893 | 2683.2405 | -0.0488 | -18 | 80 | 103 | GAHCCLLVFDVTSSVSFE ALDVWK | | | Carbamidomethyl (C)[4] | | Mascot |

25 novel protein similar to BCL2-related ovarian killer (bok, zgc:64041) [Danio rerio] gi|169154887 24238.7 7.71 7 33 0

**Peptide Information**

| Calc. Mass | Obsrv. Mass | ± da | ± ppm | Start Seq. | End Seq. | Sequence | Ion Score | C. I. % | Modification | Rank | Result Type |
|---|---|---|---|---|---|---|---|---|---|---|---|
| 842.5134 | 842.5162 | 0.0028 | 3 | 164 | 170 | SLVPWLK | | | | | Mascot |
| 980.4288 | 980.4255 | -0.0033 | -3 | 181 | 188 | CVVNMDSR | | | Carbamidomethyl (C)[1] | | Mascot |
| 1139.5449 | 1139.5138 | -0.0311 | -27 | 1 | 9 | MRDMNVFAR | | | | | Mascot |
| 1275.6262 | 1275.6111 | -0.0151 | -12 | 36 | 45 | ELCRDFIHSR | | | | | Mascot |
| 1351.7039 | 1351.7147 | 0.0108 | 8 | 29 | 39 | ELVFQSKELCR | | | | | Mascot |
| 1791.8881 | 1791.9619 | 0.0738 | 41 | 173 | 188 | GGWVDILKCVVNMDSR | | | | | Mascot |
| 1800.8407 | 1800.8799 | 0.0392 | 22 | 77 | 90 | LGDELECMRPYVYR | | | Carbamidomethyl (C)[7] | | Mascot |
| 1807.8829 | 1807.9532 | 0.0703 | 39 | 173 | 188 | GGWVDILKCVVNMDSR | | | Oxidation (M)[13] | | Mascot |

26 nucleolar protein 56 [Danio rerio] gi|41054986 54124.7 9.27 9 33 0

**Peptide Information**

| Calc. Mass | Obsrv. Mass | ± da | ± ppm | Start Seq. | End Seq. | Sequence | Ion Score | C. I. % | Modification | Rank | Result Type |
|---|---|---|---|---|---|---|---|---|---|---|---|
| 964.5574 | 964.4287 | -0.1287 | -133 | 369 | 376 | ISRYLANK | | | | | Mascot |
| 980.5676 | 980.4255 | -0.1421 | -145 | 128 | 135 | LHFHSLVK | | | | | Mascot |
| 1045.571 | 1045.5701 | -0.0009 | -1 | 91 | 100 | KPMLGVSDAK | | | | | Mascot |
| 1275.6943 | 1275.6111 | -0.0832 | -65 | 78 | 89 | LFLETNLPSGGK | | | | | Mascot |
| 1751.9591 | 1751.8743 | -0.0848 | -48 | 349 | 364 | YGLIFHSTFIGRAAAK | | | | | Mascot |
| 1808.0177 | 1807.9532 | -0.0645 | -36 | 128 | 144 | LHFHSLVKGLTAQAASK | | | | | Mascot |
| 2016.0621 | 2016.0427 | -0.0194 | -10 | 136 | 155 | GLTAQAASKAQLGLGHS YSR | | | | | Mascot |
| 2211.2358 | 2211.1294 | -0.1064 | -48 | 1 | 20 | MVLLHVLFEHAAGYALLA VK | | | Oxidation (M)[1] | | Mascot |
| 2211.2358 | 2211.1294 | -0.1064 | -48 | 1 | 20 | MVLLHVLFEHAAGYALLA VK | | | Oxidation (M)[1] | | Mascot |
| 2362.0347 | 2362.1172 | 0.0825 | 35 | 222 | 241 | KELSEEMLEAMEEITMDS AK | | | Oxidation (M)[7,11,16] | | Mascot |

27 pyruvate carboxylase, like [Danio rerio] gi|83415094 24854.1 9.35 7 33 0

**Peptide Information**

| Calc. Mass | Obsrv. Mass | ± da | ± ppm | Start | End | Sequence | Ion | C. I. % | Modification | Rank | Result Type |
|---|---|---|---|---|---|---|---|---|---|---|---|

| Calc. Mass | Obsrv. Mass | ± da | ± ppm | Start Seq. | End Seq. | Sequence | Ion Score | C. I. % | Modification | Rank | Result Type |
|---|---|---|---|---|---|---|---|---|---|---|---|
| 1100.6099 | 1100.6183 | 0.0084 | 8 | 140 | 149 | FIGPTPDVVR | | | | | Mascot |
| 1322.6157 | 1322.6648 | 0.0491 | 37 | 127 | 139 | ADFAQACVDAGVR | | | | | Mascot |
| 1379.6372 | 1379.6506 | 0.0134 | 10 | 127 | 139 | ADFAQACVDAGVR | | | Carbamidomethyl (C)[7] | | Mascot |
| 1717.0046 | 1717.0227 | 0.0181 | 11 | 91 | 106 | GLPPVAAYLHIPDIIK | | | | | Mascot |
| 1721.7911 | 1721.8103 | 0.0192 | 11 | 65 | 79 | TVAVYSEQDTGQMHR | | | | | Mascot |
| 1737.7861 | 1737.8036 | 0.0175 | 10 | 65 | 79 | TVAVYSEQDTGQMHR | | | Oxidation (M)[13] | | Mascot |
| 1792.0374 | 1791.9619 | -0.0755 | -42 | 2 | 18 | LLQVGGRHSALGLLAMR | | | | | Mascot |
| 1808.0322 | 1807.9532 | -0.079 | -44 | 2 | 18 | LLQVGGRHSALGLLAMR | | | Oxidation (M)[16] | | Mascot |
| 1860.8875 | 1860.9238 | 0.0363 | 20 | 110 | 126 | ANDVDAIHPGYGFLSER | | | | | Mascot |
| 1993.9396 | 1994.0005 | 0.0609 | 31 | 65 | 81 | TVAVYSEQDTGQMHRQK | | | Oxidation (M)[13] | | Mascot |

28 keratin-like [Danio rerio] gi|41055898 44743.4 4.87 8 33 0

**Peptide Information**

| Calc. Mass | Obsrv. Mass | ± da | ± ppm | Start Seq. | End Seq. | Sequence | Ion Score | C. I. % | Modification | Rank | Result Type |
|---|---|---|---|---|---|---|---|---|---|---|---|
| 1064.5623 | 1064.5403 | -0.022 | -21 | 390 | 398 | KLLDGEDFK | | | | | Mascot |
| 1423.6482 | 1423.7625 | 0.1143 | 80 | 334 | 345 | NTEIRSNGEMEK | | | Oxidation (M)[10] | | Mascot |
| 1474.5607 | 1474.6882 | 0.1275 | 86 | 250 | 261 | GQDMSQVMEDMR | | | Oxidation (M)[4,8,11] | | Mascot |
| 1483.7322 | 1483.6519 | -0.0803 | -54 | 36 | 49 | ISSASAMSYRSAPR | | | | | Mascot |
| 1858.0181 | 1857.9545 | -0.0636 | -34 | 158 | 173 | LVLQIDNARLAVDDFR | | | | | Mascot |
| 2031.8569 | 2032.0435 | 0.1866 | 92 | 250 | 266 | GQDMSQVMEDMRANYE K | | | | | Mascot |
| 2211.0784 | 2211.1294 | 0.051 | 23 | 362 | 380 | ANITEQGQEYEALLNMK MK | | | | | Mascot |
| 2211.0784 | 2211.1294 | 0.051 | 23 | 362 | 380 | ANITEQGQEYEALLNMK MK | | | | | Mascot |
| 2545.1504 | 2545.3594 | 0.209 | 82 | 61 | 89 | VSSAGMGSGAGGSLLAS SGSSGGMLGNEK | | | Oxidation (M)[6,24] | | Mascot |
| 2545.1504 | 2545.3594 | 0.209 | 82 | 61 | 89 | VSSAGMGSGAGGSLLAS SGSSGGMLGNEK | | | Oxidation (M)[6,24] | | Mascot |

29 Zgc:109744 [Danio rerio] gi|71534073 88043.4 5.85 11 33 0

**Peptide Information**

| Calc. Mass | Obsrv. Mass | ± da | ± ppm | Start Seq. | End Seq. | Sequence | Ion Score | C. I. % | Modification | Rank | Result Type |
|---|---|---|---|---|---|---|---|---|---|---|---|
| 1045.6041 | 1045.5701 | -0.034 | -33 | 489 | 497 | IPSSLPIYR | | | | | Mascot |
| 1064.5121 | 1064.5403 | 0.0282 | 26 | 582 | 589 | YIFCLYDK | | | | | Mascot |
| 1151.5361 | 1151.6522 | 0.1161 | 101 | 590 | 599 | LLECSQDTSR | | | | | Mascot |
| 1164.5314 | 1164.5923 | 0.0609 | 52 | 707 | 717 | AVNQTEGCASK | | | Carbamidomethyl (C)[8] | | Mascot |

| | | | | | | | | | | | |
|---|---|---|---|---|---|---|---|---|---|---|---|
| 1351.6488 | 1351.7147 | 0.0659 | 49 | 629 | 640 | DNADNQYGTLLK | | | | | Mascot |
| 1363.7303 | 1363.6885 | -0.0418 | -31 | 478 | 487 | RLWLAFCLER | 1 | 0 | Carbamidomethyl (C)[7] | | Mascot |
| 1363.7303 | 1363.6885 | -0.0418 | -31 | 478 | 487 | RLWLAFCLER | | | Carbamidomethyl (C)[7] | | Mascot |
| 1751.8282 | 1751.8743 | 0.0461 | 26 | 287 | 300 | HEPMQHEPKQHEPK | | | | | Mascot |
| 1857.9891 | 1857.9545 | -0.0346 | -19 | 362 | 377 | VVSLDLLQNHFNAMLK | | | Oxidation (M)[14] | | Mascot |
| 2016.0073 | 2016.0427 | 0.0354 | 18 | 62 | 77 | EEQDVSWVDLIQWLQK | | | | | Mascot |
| 2318.0249 | 2318.2329 | 0.208 | 90 | 458 | 477 | YINMASEMLCPSDVTNP NYR | | | | | Mascot |
| 3364.6729 | 3364.6116 | -0.0613 | -18 | 330 | 361 | TDTGDNVTLLDIAVLSWQ KMAGIYGGPNEAAK | | | Oxidation (M)[20] | | Mascot |

| | | | | | | | |
|---|---|---|---|---|---|---|---|
| 30 | hypothetical protein LOC550535 [Danio rerio] | gi\|62955647 | 31091 | 4.83 | 7 | 33 | 0 |

**Peptide Information**

| Calc. Mass | Obsrv. Mass | ± da | ± ppm | Start Seq. | End Seq. | Sequence | Ion Score | C. I. % | Modification | Rank | Result Type |
|---|---|---|---|---|---|---|---|---|---|---|---|
| 1280.6229 | 1280.6072 | -0.0157 | -12 | 75 | 84 | DEVIYERNSR | | | | | Mascot |
| 1316.594 | 1316.6786 | 0.0846 | 64 | 65 | 74 | NELECAVYYR | | | Carbamidomethyl (C)[5] | | Mascot |
| 1322.6812 | 1322.6648 | -0.0164 | -12 | 82 | 93 | NSRFGEVGTLSR | | | | | Mascot |
| 1352.6627 | 1352.7048 | 0.0421 | 31 | 136 | 147 | CDFLLDASQGKR | | | | | Mascot |
| 1352.6627 | 1352.7048 | 0.0421 | 31 | 136 | 147 | CDFLLDASQGKR | | | | | Mascot |
| 1518.6616 | 1518.7903 | 0.1287 | 85 | 184 | 196 | NSECFAAWCRFGK | | | | | Mascot |
| 1737.8475 | 1737.8036 | -0.0439 | -25 | 132 | 146 | NEIKCDFLLDASQGK | | | Carbamidomethyl (C)[5] | | Mascot |
| 1859.9531 | 1859.9314 | -0.0217 | -12 | 159 | 176 | ALSSDAVVQNAMEQVGL K | | | | | Mascot |
| 1859.9531 | 1859.9314 | -0.0217 | -12 | 159 | 176 | ALSSDAVVQNAMEQVGL K | | | | | Mascot |

| | | | | | | | |
|---|---|---|---|---|---|---|---|
| 31 | PREDICTED: vacuolar protein sorting-associated protein 41 homolog [Danio rerio] | gi\|125853163 | 99028.4 | 5.84 | 14 | 33 | 0 |

**Peptide Information**

| Calc. Mass | Obsrv. Mass | ± da | ± ppm | Start Seq. | End Seq. | Sequence | Ion Score | C. I. % | Modification | Rank | Result Type |
|---|---|---|---|---|---|---|---|---|---|---|---|
| 877.5141 | 877.423 | -0.0911 | -104 | 527 | 533 | ALEIYLR | | | | | Mascot |
| 996.4744 | 996.6053 | 0.1309 | 131 | 409 | 417 | GDYDTAARK | | | | | Mascot |
| 1045.5272 | 1045.5701 | 0.0429 | 41 | 2 | 10 | AEVEEQGRK | | | | | Mascot |
| 1064.4677 | 1064.5403 | 0.0726 | 68 | 1 | 9 | MAEVEEQGR | | | Oxidation (M)[1] | | Mascot |
| 1095.4636 | 1095.5372 | 0.0736 | 67 | 326 | 333 | NFQENQCR | | | Carbamidomethyl (C)[7] | | Mascot |
| 1280.7109 | 1280.6072 | -0.1037 | -81 | 536 | 545 | HKDVYQLIHK | | | | | Mascot |
| 1316.6263 | 1316.6786 | 0.0523 | 40 | 247 | 257 | DPTEMRDLPSR | | | | | Mascot |
| 1352.713 | 1352.7048 | -0.0082 | -6 | 554 | 564 | DKIVLLMDFDK | | | Oxidation (M)[7] | | Mascot |
| 1352.713 | 1352.7048 | -0.0082 | -6 | 554 | 564 | DKIVLLMDFDK | | | Oxidation (M)[7] | | Mascot |

| | | | | | | | | | | | |
|---|---|---|---|---|---|---|---|---|---|---|---|
| 1474.8264 | 1474.6882 | -0.1382 | -94 | 67 | 79 | VFLLDIQGNVTQK | | | | | Mascot |
| 1501.7242 | 1501.6599 | -0.0643 | -43 | 375 | 387 | YEEALMAAEISFK | | | | | Mascot |
| 1501.7242 | 1501.6599 | -0.0643 | -43 | 375 | 387 | YEEALMAAEISFK | | | | | Mascot |
| 1529.655 | 1529.8517 | 0.1967 | 129 | 326 | 336 | NFQENQCRDYR | | | Carbamidomethyl (C)[7] | | Mascot |
| 1751.9949 | 1751.8743 | -0.1206 | -69 | 763 | 777 | ILVADSLSLLQRMHR | | | | | Mascot |
| 1859.0248 | 1858.9478 | -0.077 | -41 | 456 | 470 | LRPAIYEMILHDFLK | | | | | Mascot |
| 2283.1292 | 2283.2114 | 0.0822 | 36 | 334 | 352 | DYRLEHSEGESLFYIISPK | | | | | Mascot |

32 PREDICTED: paired amphipathic helix protein Sin3a [Danio rerio] gi|292616206 144029.5 7.26 16 33 0

**Peptide Information**

| Calc. Mass | Obsrv. Mass | ± da | ± ppm | Start Seq. | End Seq. | Sequence | Ion Score | C. I. % | Modification | Rank | Result Type |
|---|---|---|---|---|---|---|---|---|---|---|---|
| 877.3252 | 877.423 | 0.0978 | 111 | 1157 | 1162 | MECMFK | | | Carbamidomethyl (C)[3], Oxidation (M)[1,4] | | Mascot |
| 964.4523 | 964.4287 | -0.0236 | -24 | 1092 | 1098 | WPDYVER | | | | | Mascot |
| 996.5697 | 996.6053 | 0.0356 | 36 | 1194 | 1201 | VSTRLHQR | | | | | Mascot |
| 1095.5469 | 1095.5372 | -0.0097 | -9 | 315 | 323 | FQGQPEVYK | | | | | Mascot |
| 1151.5651 | 1151.6522 | 0.0871 | 76 | 1026 | 1037 | ATGGSLSTQSSR | | | | | Mascot |
| 1352.728 | 1352.7048 | -0.0232 | -17 | 394 | 406 | AEAVRIDHGGTVK | | | | | Mascot |
| 1352.728 | 1352.7048 | -0.0232 | -17 | 394 | 406 | AEAVRIDHGGTVK | | | | | Mascot |
| 1363.6648 | 1363.6885 | 0.0237 | 17 | 414 | 424 | QRFNQNGCQIR | | | | | Mascot |
| 1363.6648 | 1363.6885 | 0.0237 | 17 | 414 | 424 | QRFNQNGCQIR | | | | | Mascot |
| 1379.7053 | 1379.6506 | -0.0547 | -40 | 117 | 128 | VEDALSYLDQVK | | | | | Mascot |
| 1502.7056 | 1502.7963 | 0.0907 | 60 | 522 | 533 | ECAHIESFPKER | | | Carbamidomethyl (C)[2] | | Mascot |
| 1529.7794 | 1529.8517 | 0.0723 | 47 | 1198 | 1208 | LHQRFQMWLDR | | | | | Mascot |
| 1545.8384 | 1545.8455 | 0.0071 | 5 | 856 | 869 | SKLLFSHTAASQQK | | | | | Mascot |
| 1658.74 | 1658.9414 | 0.2014 | 121 | 534 | 548 | ATEGIAMEIDYASCK | | | Carbamidomethyl (C)[14] | | Mascot |
| 1658.74 | 1658.9414 | 0.2014 | 121 | 534 | 548 | ATEGIAMEIDYASCK | | | Carbamidomethyl (C)[14] | | Mascot |
| 1791.9388 | 1791.9619 | 0.0231 | 13 | 324 | 337 | SFLEILHTYQKEQR | | | | | Mascot |
| 1800.9351 | 1800.8799 | -0.0552 | -31 | 647 | 662 | FRLDNTIGGSSEVIHR | | | | | Mascot |
| 2283.104 | 2283.2114 | 0.1074 | 47 | 1092 | 1110 | WPDYVERYLNTSSASPE LR | | | | | Mascot |
| 2362.0989 | 2362.1172 | 0.0183 | 8 | 1225 | 1244 | WLMGEGQDGLLPCRTV CEPK | | | Carbamidomethyl (C)[13,17], Oxidation (M)[3] | | Mascot |

33 Wu:fa07b03 protein [Danio rerio] gi|112419196 32306.1 8.35 7 33 0

**Peptide Information**

| Calc. Mass | Obsrv. Mass | ± da | ± ppm | Start Seq. | End Seq. | Sequence | Ion Score | C. I. % | Modification | Rank | Result Type |
|---|---|---|---|---|---|---|---|---|---|---|---|

| Calc. Mass | Obsrv. Mass | ± da | ± ppm | Start Seq. | End Seq. | Sequence | Ion Score | C. I. % | Modification | Rank | Result Type |
|---|---|---|---|---|---|---|---|---|---|---|---|
| 1139.6418 | 1139.5138 | -0.128 | -112 | 108 | 118 | TGAHIELSLAK | | | | | Mascot |
| 1151.5878 | 1151.6522 | 0.0644 | 56 | 220 | 228 | AVERLCFEK | | | Carbamidomethyl (C)[6] | | Mascot |
| 1164.5929 | 1164.5923 | -0.0006 | -1 | 119 | 129 | DQGLSIMVTGK | | | Oxidation (M)[7] | | Mascot |
| 1363.6906 | 1363.6885 | -0.0021 | -2 | 229 | 240 | AFHPFIAGAYNR | | | | | Mascot |
| 1363.6906 | 1363.6885 | -0.0021 | -2 | 229 | 240 | AFHPFIAGAYNR | | | | | Mascot |
| 1501.7605 | 1501.6599 | -0.1006 | -67 | 186 | 199 | SDDPNANIRITGTK | | | | | Mascot |
| 1501.7605 | 1501.6599 | -0.1006 | -67 | 186 | 199 | SDDPNANIRITGTK | | | | | Mascot |
| 1791.8694 | 1791.9619 | 0.0925 | 52 | 1 | 16 | MSSVAVLTQESFAEHR | | | | | Mascot |
| 1807.8643 | 1807.9532 | 0.0889 | 49 | 1 | 16 | MSSVAVLTQESFAEHR | | | Oxidation (M)[1] | | Mascot |
| 2177.1018 | 2176.9849 | -0.1169 | -54 | 1 | 20 | MSSVAVLTQESFAEHRS GLK | | | | | Mascot |

34 intraflagellar transport protein 172 homolog [Danio rerio] gi|50539690 197598.9 5.6 19 33 0

Peptide Information

| Calc. Mass | Obsrv. Mass | ± da | ± ppm | Start Seq. | End Seq. | Sequence | Ion Score | C. I. % | Modification | Rank | Result Type |
|---|---|---|---|---|---|---|---|---|---|---|---|
| 877.46 | 877.423 | -0.037 | -42 | 782 | 789 | AAWLAMSK | | | | | Mascot |
| 882.4501 | 882.575 | 0.1249 | 142 | 828 | 834 | ALECYRK | | | | | Mascot |
| 1064.5483 | 1064.5403 | -0.008 | -8 | 815 | 823 | AGDLFERTR | | | | | Mascot |
| 1095.5867 | 1095.5372 | -0.0495 | -45 | 1291 | 1299 | AVDCYLKVK | | | Carbamidomethyl (C)[4] | | Mascot |
| 1139.4899 | 1139.5138 | 0.0239 | 21 | 1489 | 1497 | DRDCAEAFR | | | Carbamidomethyl (C)[4] | | Mascot |
| 1165.6034 | 1165.7551 | 0.1517 | 130 | 1480 | 1488 | MFQELVNLR | | | Oxidation (M)[1] | | Mascot |
| 1263.6588 | 1263.6631 | 0.0043 | 3 | 1310 | 1320 | CWMKAAELAIK | | | | | Mascot |
| 1263.6588 | 1263.6631 | 0.0043 | 3 | 1310 | 1320 | CWMKAAELAIK | | | | | Mascot |
| 1275.6692 | 1275.6111 | -0.0581 | -46 | 51 | 61 | DRFTTKPADPK | | | | | Mascot |
| 1351.7368 | 1351.7147 | -0.0221 | -16 | 1215 | 1225 | EFQKAEAFLLR | | | | | Mascot |
| 1352.7208 | 1352.7048 | -0.016 | -12 | 53 | 64 | FTTKPADPKYGK | | | | | Mascot |
| 1352.7208 | 1352.7048 | -0.016 | -12 | 53 | 64 | FTTKPADPKYGK | | | | | Mascot |
| 1363.6675 | 1363.6885 | 0.021 | 15 | 1643 | 1653 | EWVLTVSMDQR | | | | | Mascot |
| 1363.6675 | 1363.6885 | 0.021 | 15 | 1643 | 1653 | EWVLTVSMDQR | | | | | Mascot |
| 1379.6624 | 1379.6506 | -0.0118 | -9 | 1643 | 1653 | EWVLTVSMDQR | | | Oxidation (M)[8] | | Mascot |
| 1474.8086 | 1474.6882 | -0.1204 | -82 | 1679 | 1691 | SLPCVITGYPVLR | | | Carbamidomethyl (C)[4] | | Mascot |
| 1502.7169 | 1502.7963 | 0.0794 | 53 | 1698 | 1710 | RPGMAANKDDWNK | | | | | Mascot |
| 1518.7118 | 1518.7903 | 0.0785 | 52 | 1698 | 1710 | RPGMAANKDDWNK | | | Oxidation (M)[4] | | Mascot |
| 1529.6545 | 1529.8517 | 0.1972 | 129 | 1238 | 1250 | DAGMWTDAMRICK | | | Oxidation (M)[4,9] | | Mascot |
| 1658.9159 | 1658.9414 | 0.0255 | 15 | 1168 | 1182 | AGKPKEAVLMHVHNK | | | | | Mascot |
| 1658.9159 | 1658.9414 | 0.0255 | 15 | 1168 | 1182 | AGKPKEAVLMHVHNK | | | | | Mascot |

| Calc. Mass | Obsrv. Mass | ± da | ± ppm | Start Seq. | End Seq. | Sequence | Ion Score | C. I. % | Modification | Rank | Result Type |
|---|---|---|---|---|---|---|---|---|---|---|---|
| 1716.9465 | 1717.0227 | 0.0762 | 44 | 1679 | 1693 | SLPCVITGYPVLRNK | | | Carbamidomethyl (C)[4] | | Mascot |
| 2211.0508 | 2211.1294 | 0.0786 | 36 | 19 | 38 | VTCMAWAPNNAKFAVCT IDR | | | | | Mascot |
| 2211.0508 | 2211.1294 | 0.0786 | 36 | 19 | 38 | VTCMAWAPNNAKFAVCT IDR | | | | | Mascot |
| 2284.0671 | 2284.2034 | 0.1363 | 60 | 19 | 38 | VTCMAWAPNNAKFAVCT IDR | | | Carbamidomethyl (C)[3], Oxidation (M)[4] | | Mascot |
| 2318.0396 | 2318.2329 | 0.1933 | 83 | 297 | 318 | DGSRLSVGTLCGGVEMF DCCLR | | | | | Mascot |
| 2683.3008 | 2683.2405 | -0.0603 | -22 | 520 | 543 | STMLSFCPYVQWVPGSD VVVAQNR | | | | | Mascot |

| | | | | | | | |
|---|---|---|---|---|---|---|---|
| 35 | hypothetical protein LOC569975 [Danio rerio] | gi\|71834622 | 27594.2 | 6.28 | 8 | 32 | 0 |

**Peptide Information**

| Calc. Mass | Obsrv. Mass | ± da | ± ppm | Start Seq. | End Seq. | Sequence | Ion Score | C. I. % | Modification | Rank | Result Type |
|---|---|---|---|---|---|---|---|---|---|---|---|
| 877.3897 | 877.423 | 0.0333 | 38 | 15 | 21 | EESEEVR | | | | | Mascot |
| 1322.5656 | 1322.6648 | 0.0992 | 75 | 84 | 93 | GFFFSCLECR | | | Carbamidomethyl (C)[6,9] | | Mascot |
| 1474.7914 | 1474.6882 | -0.1032 | -70 | 138 | 148 | EHLLEHYRIHK | | | | | Mascot |
| 1483.6052 | 1483.6519 | 0.0467 | 31 | 211 | 221 | CDQCERSYTHK | | | Carbamidomethyl (C)[1,4] | | Mascot |
| 1800.8771 | 1800.8799 | 0.0028 | 2 | 146 | 160 | IHKVEKPFPCEECGK | | | Carbamidomethyl (C)[10] | | Mascot |
| 1807.8254 | 1807.9532 | 0.1278 | 71 | 94 | 107 | MSFESKEHLMEHFR | | | | | Mascot |
| 1857.8986 | 1857.9545 | 0.0559 | 30 | 146 | 160 | IHKVEKPFPCEECGK | | | Carbamidomethyl (C)[10,13] | | Mascot |
| 1860.8732 | 1860.9238 | 0.0506 | 27 | 202 | 216 | IHELDKPFKCDQCER | | | | | Mascot |
| 2031.8761 | 2032.0435 | 0.1674 | 82 | 84 | 99 | GFFFSCLECRMSFESK | | | Carbamidomethyl (C)[6,9] | | Mascot |

| | | | | | | | |
|---|---|---|---|---|---|---|---|
| 36 | PREDICTED: e3 ubiquitin-protein ligase BRE1A [Danio rerio] | gi\|189527718 | 115551 | 5.66 | 16 | 32 | 0 |

**Peptide Information**

| Calc. Mass | Obsrv. Mass | ± da | ± ppm | Start Seq. | End Seq. | Sequence | Ion Score | C. I. % | Modification | Rank | Result Type |
|---|---|---|---|---|---|---|---|---|---|---|---|
| 842.5206 | 842.5162 | -0.0044 | -5 | 61 | 67 | ALQIKNR | | | | | Mascot |
| 980.4795 | 980.4255 | -0.054 | -55 | 910 | 917 | EKESFNAR | | | | | Mascot |
| 1045.5823 | 1045.5701 | -0.0122 | -12 | 786 | 793 | LMQQLREK | | | | | Mascot |
| 1151.5474 | 1151.6522 | 0.1048 | 91 | 189 | 198 | VESSCKQASR | | | Carbamidomethyl (C)[5] | | Mascot |
| 1280.5908 | 1280.6072 | 0.0164 | 13 | 958 | 968 | LTCPCCNSRVK | | | Carbamidomethyl (C)[3] | | Mascot |
| 1322.7137 | 1322.6648 | -0.0489 | -37 | 682 | 692 | LLLDMYRSAPK | | | Oxidation (M)[5] | | Mascot |
| 1379.5909 | 1379.6506 | 0.0597 | 43 | 998 | 1009 | CNAAFGANDFHR | | | Carbamidomethyl (C)[1] | | Mascot |
| 1502.7996 | 1502.7963 | -0.0033 | -2 | 859 | 871 | ELGLRTQALEMNK | | | | | Mascot |
| 1518.7944 | 1518.7903 | -0.0041 | -3 | 859 | 871 | ELGLRTQALEMNK | | | Oxidation (M)[11] | | Mascot |
| 1638.7826 | 1638.8779 | 0.0953 | 58 | 317 | 332 | VCGEASSVCGTITINK | | | Carbamidomethyl (C)[2] | | Mascot |

| | | | | | | | | | | | |
|---|---|---|---|---|---|---|---|---|---|---|---|
| 1658.8846 | 1658.9414 | 0.0568 | 34 | 816 | 829 | EEKEELADQLLTLK | | | | | Mascot |
| 1658.8846 | 1658.9414 | 0.0568 | 34 | 816 | 829 | EEKEELADQLLTLK | | | | | Mascot |
| 1716.8551 | 1717.0227 | 0.1676 | 98 | 216 | 231 | DLESGKDGGLWDVAVR | | | | | Mascot |
| 1721.9293 | 1721.8103 | -0.119 | -69 | 296 | 309 | ENRLNTHLVEVLER | | | | | Mascot |
| 1737.8622 | 1737.8036 | -0.0586 | -34 | 317 | 333 | VCGEASSVCGTITINKR | | | | | Mascot |
| 1768.9188 | 1768.8936 | -0.0252 | -14 | 6 | 23 | RPSDAGSSATLGAPPEKK | | | | | Mascot |
| 1857.876 | 1857.9545 | 0.0785 | 42 | 429 | 443 | QLDHMENDEVSLQRK | | | Oxidation (M)[5] | | Mascot |
| 1858.7871 | 1858.9478 | 0.1607 | 86 | 122 | 138 | CDQSGSEPVESQPPEGR | | | Carbamidomethyl (C)[1] | | Mascot |

37 phosphatidylinositol 4-kinase alpha [Danio rerio] gi|77993310 232707.4 6.2 20 32 0 5 0

**Peptide Information**

| Calc. Mass | Obsrv. Mass | ± da | ± ppm | Start Seq. | End Seq. | Sequence | Ion Score | C. I. % | Modification | Rank | Result Type |
|---|---|---|---|---|---|---|---|---|---|---|---|
| 877.4373 | 877.423 | -0.0143 | -16 | 689 | 696 | ASERASEK | | | | | Mascot |
| 1077.5356 | 1077.5269 | -0.0087 | -8 | 1768 | 1776 | RCGVSELEK | | | Carbamidomethyl (C)[2] | | Mascot |
| 1095.5979 | 1095.5372 | -0.0607 | -55 | 841 | 848 | LEYMRVLR | | | Oxidation (M)[4] | | Mascot |
| 1164.5895 | 1164.5923 | 0.0028 | 2 | 983 | 992 | ITVPDTYEAR | | | | | Mascot |
| 1263.6401 | 1263.6631 | 0.023 | 18 | 1009 | 1019 | EAMKWAPSVTK | | | Oxidation (M)[3] | | Mascot |
| 1263.6401 | 1263.6631 | 0.023 | 18 | 1009 | 1019 | EAMKWAPSVTK | | | Oxidation (M)[3] | | Mascot |
| 1280.6368 | 1280.6072 | -0.0296 | -23 | 139 | 149 | YSNTEEPLLSK | | | | | Mascot |
| 1322.7103 | 1322.6648 | -0.0455 | -34 | 1489 | 1498 | YINLTEKQWK | | | | | Mascot |
| 1351.6384 | 1351.7147 | 0.0763 | 56 | 324 | 333 | MLRDSLYYMK | | | Oxidation (M)[1,9] | | Mascot |
| 1379.6511 | 1379.6506 | -0.0005 | 0 | 629 | 640 | ASSMVYSTKDYK | | | | | Mascot |
| 1529.7166 | 1529.8517 | 0.1351 | 88 | 849 | 860 | SQESDRFQVMFR | | | | | Mascot |
| 1545.7114 | 1545.8455 | 0.1341 | 87 | 849 | 860 | SQESDRFQVMFR | | | Oxidation (M)[10] | | Mascot |
| 1593.8231 | 1593.7393 | -0.0838 | -53 | 154 | 169 | IVPQPPGTGDGAEVTR | 5 | 0 | | | Mascot |
| 1593.8231 | 1593.7393 | -0.0838 | -53 | 154 | 169 | IVPQPPGTGDGAEVTR | | | | | Mascot |
| 1638.7648 | 1638.8779 | 0.1131 | 69 | 870 | 884 | DKSGMMQCVIAVGDK | | | Carbamidomethyl (C)[8] | | Mascot |
| 1658.828 | 1658.9414 | 0.1134 | 68 | 885 | 898 | VFDVFLQMMADKAK | | | Oxidation (M)[8] | | Mascot |
| 1658.828 | 1658.9414 | 0.1134 | 68 | 885 | 898 | VFDVFLQMMADKAK | | | Oxidation (M)[8] | | Mascot |
| 1717.0298 | 1717.0227 | -0.0071 | -4 | 453 | 467 | DFLVVPSPVLVKLYK | | | | | Mascot |
| 1800.9717 | 1800.8799 | -0.0918 | -51 | 312 | 326 | TFSDPLYVAIFKMLR | | | | | Mascot |
| 1858.9333 | 1858.9478 | 0.0145 | 8 | 465 | 480 | LYKYHSQYTTGAGEIK | | | | | Mascot |
| 1859.8666 | 1859.9314 | 0.0648 | 35 | 2018 | 2033 | FNPNMSEKEAAAFMIK | | | Oxidation (M)[5,14] | | Mascot |
| 1859.8666 | 1859.9314 | 0.0648 | 35 | 2018 | 2033 | FNPNMSEKEAAAFMIK | | | Oxidation (M)[5,14] | | Mascot |
| 2176.9998 | 2176.9849 | -0.0149 | -7 | 1953 | 1971 | LTDEMVMIMGGKMEATPFK | | | Oxidation (M)[5,7,9] | | Mascot |
| 2225.2078 | 2225.1482 | -0.0596 | -27 | 1499 | 1518 | DNVNLAWSIAPYLALQLP | | | | | Mascot |

| | | | | | | | | | | | |
|---|---|---|---|---|---|---|---|---|---|---|---|
| 2283.0857 | 2283.2114 | 0.1257 | 55 | 500 | 518 | AR SQPSMYEQLRDISINNIC R | | | Oxidation (M)[5] | | Mascot |

38 novel protein similar to vertebrate (PIWIL2) [Danio rerio] gi|123232882 22624.7 6.37 6 32 0

Peptide Information

| Calc. Mass | Obsrv. Mass | ± da | ± ppm | Start Seq. | End Seq. | Sequence | Ion Score | C. I. % | Modification | Rank | Result Type |
|---|---|---|---|---|---|---|---|---|---|---|---|
| 1379.7893 | 1379.6506 | -0.1387 | -101 | 115 | 128 | GAAIGPVPTLDIQK | | | | | Mascot |
| 1423.7614 | 1423.7625 | 0.0011 | 1 | 40 | 52 | GLIMPIDEPLPGR | | | Oxidation (M)[4] | | Mascot |
| 1483.7825 | 1483.6519 | -0.1306 | -88 | 135 | 148 | MPELQAEVAPTVAK | | | | | Mascot |
| 1518.7184 | 1518.7903 | 0.0719 | 47 | 186 | 198 | DLRDQADDQPFAK | | | | | Mascot |
| 1800.8334 | 1800.8799 | 0.0465 | 26 | 199 | 214 | DGVQQAIWGQICSQNP | | | Carbamidomethyl (C)[12] | | Mascot |
| 2233.1646 | 2233.1099 | -0.0547 | -24 | 149 | 171 | VGSPGTGSSLVSMFRGL GIEPGK | | | | | Mascot |

39 PREDICTED: ephrin type-B receptor 1 [Danio rerio] gi|326665193 107747.2 6.03 12 32 0

Peptide Information

| Calc. Mass | Obsrv. Mass | ± da | ± ppm | Start Seq. | End Seq. | Sequence | Ion Score | C. I. % | Modification | Rank | Result Type |
|---|---|---|---|---|---|---|---|---|---|---|---|
| 877.4236 | 877.423 | -0.0006 | -1 | 247 | 254 | ACPPGTFK | | | Carbamidomethyl (C)[2] | | Mascot |
| 996.5359 | 996.6053 | 0.0694 | 70 | 630 | 638 | TLKAGYSEK | | | | | Mascot |
| 1151.6895 | 1151.6522 | -0.0373 | -32 | 932 | 942 | IGVTLAGHQKK | | | | | Mascot |
| 1165.61 | 1165.7551 | 0.1451 | 124 | 849 | 858 | FTDIVNTLDK | | | | | Mascot |
| 1280.6416 | 1280.6072 | -0.0344 | -27 | 69 | 79 | FTVRDCSSIPR | | | | | Mascot |
| 1518.7482 | 1518.7903 | 0.0421 | 28 | 557 | 570 | LQHYSTGRGSPGMK | | | | | Mascot |
| 1658.9012 | 1658.9414 | 0.0402 | 24 | 764 | 777 | IPVRWTSPEAIAYR | | | | | Mascot |
| 1658.9012 | 1658.9414 | 0.0402 | 24 | 764 | 777 | IPVRWTSPEAIAYR | 3 | 0 | | | Mascot |
| 1721.8826 | 1721.8103 | -0.0723 | -42 | 471 | 485 | VDGLRPGTMYVMQVR | | | | | Mascot |
| 1737.8774 | 1737.8036 | -0.0738 | -42 | 471 | 485 | VDGLRPGTMYVMQVR | | | Oxidation (M)[9] | | Mascot |
| 1859.7898 | 1859.9314 | 0.1416 | 76 | 288 | 305 | ADGEQPDMPCTSVPSGP R | | | Oxidation (M)[8] | | Mascot |
| 1859.7898 | 1859.9314 | 0.1416 | 76 | 288 | 305 | ADGEQPDMPCTSVPSGP R | | | Oxidation (M)[8] | | Mascot |
| 2232.918 | 2233.1099 | 0.1919 | 86 | 216 | 234 | LYCNGDGDWMVPIGSCT CK | | | Carbamidomethyl (C)[3,16,18] | | Mascot |
| 2318.3079 | 2318.2329 | -0.075 | -32 | 862 | 883 | NPTSLKAVASIPTIPSQPL LDR | | | | | Mascot |
| 2362.1609 | 2362.1172 | -0.0437 | -19 | 641 | 660 | RDFLSEASIMGQFDHPNII R | | | Oxidation (M)[10] | | Mascot |

40 RAB39B, member RAS oncogene family [Danio rerio] gi|148922827 24462.5 6.54 6 32 0

Peptide Information

| Calc. Mass | Obsrv. Mass | ± da | ± ppm | Start Seq. | End Seq. | Sequence | Ion Score | C. I. % | Modification | Rank | Result Type |
|---|---|---|---|---|---|---|---|---|---|---|---|
| 1316.6593 | 1316.6786 | 0.0193 | 15 | 60 | 70 | LQIWDTAGQER | | | | | Mascot |
| 1518.8274 | 1518.7903 | -0.0371 | -24 | 81 | 94 | NSVGGLLLFDITNR | | | | | Mascot |
| 1658.8319 | 1658.9414 | 0.1095 | 66 | 141 | 155 | LAAAYGMRYVETSAR | | | | | Mascot |
| 1658.8319 | 1658.9414 | 0.1095 | 66 | 141 | 155 | LAAAYGMRYVETSAR | | | | | Mascot |
| 1800.8776 | 1800.8799 | 0.0023 | 1 | 95 | 108 | RSFQNVHEWLEEAR | | | | | Mascot |
| 2362.1462 | 2362.1172 | -0.029 | -12 | 28 | 48 | FTEGRFAQVSDPTVGVD FFSR | | | | | Mascot |
| 2379.1899 | 2379.3738 | 0.1839 | 77 | 170 | 190 | DIFELVKCGDITIQEGWE GVK | | | | | Mascot |
| 2379.1899 | 2379.3738 | 0.1839 | 77 | 170 | 190 | DIFELVKCGDITIQEGWE GVK | | | | | Mascot |

41 RecName: Full=Intraflagellar transport protein 172 homolog gi|82232659 197655 5.61 19 32 0

**Peptide Information**

| Calc. Mass | Obsrv. Mass | ± da | ± ppm | Start Seq. | End Seq. | Sequence | Ion Score | C. I. % | Modification | Rank | Result Type |
|---|---|---|---|---|---|---|---|---|---|---|---|
| 877.46 | 877.423 | -0.037 | -42 | 782 | 789 | AAWLAMSK | | | | | Mascot |
| 882.4501 | 882.575 | 0.1249 | 142 | 828 | 834 | ALECYRK | | | | | Mascot |
| 1064.5483 | 1064.5403 | -0.008 | -8 | 815 | 823 | AGDLFERTR | | | | | Mascot |
| 1095.5867 | 1095.5372 | -0.0495 | -45 | 1291 | 1299 | AVDCYLKVK | | | Carbamidomethyl (C)[4] | | Mascot |
| 1139.4899 | 1139.5138 | 0.0239 | 21 | 1489 | 1497 | DRDCAEAFR | | | Carbamidomethyl (C)[4] | | Mascot |
| 1165.6034 | 1165.7551 | 0.1517 | 130 | 1480 | 1488 | MFQELVNLR | | | Oxidation (M)[1] | | Mascot |
| 1263.6588 | 1263.6631 | 0.0043 | 3 | 1310 | 1320 | CWMKAAELAIK | | | | | Mascot |
| 1263.6588 | 1263.6631 | 0.0043 | 3 | 1310 | 1320 | CWMKAAELAIK | | | | | Mascot |
| 1275.6692 | 1275.6111 | -0.0581 | -46 | 51 | 61 | DRFTTKPADPK | | | | | Mascot |
| 1351.7368 | 1351.7147 | -0.0221 | -16 | 1215 | 1225 | EFQKAEAFLLR | | | | | Mascot |
| 1352.7208 | 1352.7048 | -0.016 | -12 | 53 | 64 | FTTKPADPKYGK | | | | | Mascot |
| 1352.7208 | 1352.7048 | -0.016 | -12 | 53 | 64 | FTTKPADPKYGK | | | | | Mascot |
| 1363.6675 | 1363.6885 | 0.021 | 15 | 1643 | 1653 | EWVLTVSMDQR | | | | | Mascot |
| 1363.6675 | 1363.6885 | 0.021 | 15 | 1643 | 1653 | EWVLTVSMDQR | | | | | Mascot |
| 1379.6624 | 1379.6506 | -0.0118 | -9 | 1643 | 1653 | EWVLTVSMDQR | | | Oxidation (M)[8] | | Mascot |
| 1474.8086 | 1474.6882 | -0.1204 | -82 | 1679 | 1691 | SLPCVITGYPVLR | | | Carbamidomethyl (C)[4] | | Mascot |
| 1502.7169 | 1502.7963 | 0.0794 | 53 | 1698 | 1710 | RPGMAANKDDWNK | | | | | Mascot |
| 1518.7118 | 1518.7903 | 0.0785 | 52 | 1698 | 1710 | RPGMAANKDDWNK | | | Oxidation (M)[4] | | Mascot |
| 1529.6545 | 1529.8517 | 0.1972 | 129 | 1238 | 1250 | DAGMWTDAMRICK | | | Oxidation (M)[4,9] | | Mascot |
| 1658.9159 | 1658.9414 | 0.0255 | 15 | 1168 | 1182 | AGKPKEAVLMHVHNK | | | | | Mascot |

| | | | | | | | | | | | |
|---|---|---|---|---|---|---|---|---|---|---|---|
| 1658.9159 | 1658.9414 | 0.0255 | 15 | 1168 | 1182 | AGKPKEAVLMHVHNK | | | | | Mascot |
| 1716.9465 | 1717.0227 | 0.0762 | 44 | 1679 | 1693 | SLPCVITGYPVLRNK | | | Carbamidomethyl (C)[4] | | Mascot |
| 2211.0508 | 2211.1294 | 0.0786 | 36 | 19 | 38 | VTCMAWAPNNAKFAVCTIDR | | | | | Mascot |
| 2211.0508 | 2211.1294 | 0.0786 | 36 | 19 | 38 | VTCMAWAPNNAKFAVCTIDR | | | | | Mascot |
| 2284.0671 | 2284.2034 | 0.1363 | 60 | 19 | 38 | VTCMAWAPNNAKFAVCTIDR | | | Carbamidomethyl (C)[3], Oxidation (M)[4] | | Mascot |
| 2318.0396 | 2318.2329 | 0.1933 | 83 | 297 | 318 | DGSRLSVGTLCGGVEMFDCCLR | | | | | Mascot |
| 2683.3008 | 2683.2405 | -0.0603 | -22 | 520 | 543 | STMLSFCPYVQWVPGSDVVVAQNR | | | | | Mascot |

42 PREDICTED: dynein heavy chain 7, axonemal [Danio rerio] gi|189523512 455205.4 5.74 31 31 0

Peptide Information

| Calc. Mass | Obsrv. Mass | ± da | ± ppm | Start Seq. | End Seq. | Sequence | Ion Score | C. I. % | Modification | Rank | Result Type |
|---|---|---|---|---|---|---|---|---|---|---|---|
| 877.4698 | 877.423 | -0.0468 | -53 | 695 | 701 | LELVMEK | | | Oxidation (M)[5] | | Mascot |
| 964.492 | 964.4287 | -0.0633 | -66 | 2966 | 2972 | WLKNMEK | | | Oxidation (M)[5] | | Mascot |
| 980.5159 | 980.4255 | -0.0904 | -92 | 52 | 59 | KQEYSGIR | | | | | Mascot |
| 1064.5048 | 1064.5403 | 0.0355 | 33 | 2698 | 2706 | VEDYWGPAK | | | | | Mascot |
| 1100.5444 | 1100.6183 | 0.0739 | 67 | 3937 | 3945 | QCYISPVYK | | | | | Mascot |
| 1164.5929 | 1164.5923 | -0.0006 | -1 | 986 | 995 | DTMKQVSLDK | | | | | Mascot |
| 1275.6725 | 1275.6111 | -0.0614 | -48 | 3767 | 3777 | DSCVNIQKAIK | | | Carbamidomethyl (C)[3] | | Mascot |
| 1316.6879 | 1316.6786 | -0.0093 | -7 | 1588 | 1599 | VLAAALNDICEK | | | Carbamidomethyl (C)[10] | | Mascot |
| 1322.6627 | 1322.6648 | 0.0021 | 2 | 2738 | 2748 | YITNPDFVPEK | | | | | Mascot |
| 1352.7572 | 1352.7048 | -0.0524 | -39 | 1949 | 1959 | SVYILDFLLNR | | | | | Mascot |
| 1352.7572 | 1352.7048 | -0.0524 | -39 | 1949 | 1959 | SVYILDFLLNR | | | | | Mascot |
| 1379.626 | 1379.6506 | 0.0246 | 18 | 3726 | 3737 | LPQDFDTDAAMR | | | | | Mascot |
| 1423.725 | 1423.7625 | 0.0375 | 26 | 2724 | 2735 | DNIPVSYMSIIR | | | Oxidation (M)[8] | | Mascot |
| 1483.7063 | 1483.6519 | -0.0544 | -37 | 3032 | 3044 | LGDSTIEYAPDFR | | | | | Mascot |
| 1561.8431 | 1561.8402 | -0.0029 | -2 | 1767 | 1781 | GTKELSPTSNTSLVK | | | | | Mascot |
| 1593.79 | 1593.7393 | -0.0507 | -32 | 1842 | 1855 | EMLVGGLSEETRTR | | | Oxidation (M)[2] | | Mascot |
| 1593.79 | 1593.7393 | -0.0507 | -32 | 1842 | 1855 | EMLVGGLSEETRTR | | | Oxidation (M)[2] | | Mascot |
| 1638.8156 | 1638.8779 | 0.0623 | 38 | 552 | 565 | LCEEYDTIANKALR | | | | | Mascot |
| 1721.7529 | 1721.8103 | 0.0574 | 33 | 2031 | 2042 | QWLDHWNWYDMK | | | | | Mascot |
| 1737.7479 | 1737.8036 | 0.0557 | 32 | 2031 | 2042 | QWLDHWNWYDMK | | | Oxidation (M)[11] | | Mascot |
| 1800.9602 | 1800.8799 | -0.0803 | -45 | 2973 | 2988 | SNSLHVIKLSDADFVR | | | | | Mascot |
| 1858.9004 | 1858.9478 | 0.0474 | 25 | 2415 | 2430 | QTDGSTLALFNMFLER | | | Oxidation (M)[12] | | Mascot |
| 1859.9233 | 1859.9314 | 0.0081 | 4 | 108 | 126 | SVTEISTPSTSGPPTAAEK | | | | | Mascot |

| | | | | | | | | | | | |
|---|---|---|---|---|---|---|---|---|---|---|---|
| 1859.9233 | 1859.9314 | 0.0081 | 4 | 108 | 126 | SVTEISTPSTSGPPTAAEK | | | | | Mascot |
| 1861.0767 | 1860.9238 | -0.1529 | -82 | 1198 | 1214 | QNRVTLGALVVLDVHAR | | | | | Mascot |
| 1994.1433 | 1994.0005 | -0.1428 | -72 | 1930 | 1948 | LLITHQKPTVFVGPTGTGK | | | | | Mascot |
| 2016.067 | 2016.0427 | -0.0243 | -12 | 2275 | 2291 | APMNLVLFRFAIEHVCR | | | | | Mascot |
| 2032.0619 | 2032.0435 | -0.0184 | -9 | 2275 | 2291 | APMNLVLFRFAIEHVCR | | | Oxidation (M)[3] | | Mascot |
| 2210.8865 | 2211.1294 | 0.2429 | 110 | 3601 | 3618 | YMTGECNYGGRVTDDWDR | | | Carbamidomethyl (C)[6], Oxidation (M)[2] | | Mascot |
| 2210.8865 | 2211.1294 | 0.2429 | 110 | 3601 | 3618 | YMTGECNYGGRVTDDWDR | | | Carbamidomethyl (C)[6], Oxidation (M)[2] | | Mascot |
| 2225.0154 | 2225.1482 | 0.1328 | 60 | 3226 | 3243 | DHFTYSLYCNVCRSLFEK | | | | | Mascot |
| 2283.2383 | 2283.2114 | -0.0269 | -12 | 3806 | 3825 | SYPSLKPLGGYISDFLERLK | | | | | Mascot |
| 2284.1025 | 2284.2034 | 0.1009 | 44 | 3024 | 3044 | QGGSVCIRLGDSTIEYAPDFR | | | | | Mascot |
| 2318.2095 | 2318.2329 | 0.0234 | 10 | 3424 | 3446 | LTSLSLGQGQGPIAMSMIETGVK | | | | | Mascot |
| 2362.1167 | 2362.1172 | 0.0005 | 0 | 3738 | 3757 | KYPTSYNQSMNTVLVQEMGR | | | Oxidation (M)[10] | | Mascot |
| 2661.3489 | 2661.2351 | -0.1138 | -43 | 1417 | 1440 | TVAMMVPDYALIAEIVLYSCGFVR | | | | | Mascot |
| 2683.3547 | 2683.2405 | -0.1142 | -43 | 2714 | 2735 | FLQSLHEYNKDNIPVSYMSIIR | | | Oxidation (M)[18] | | Mascot |

| | | | | | | | | |
|---|---|---|---|---|---|---|---|---|
| 43 | PREDICTED: hypothetical protein LOC497432 [Danio rerio] | gi\|326676501 | 147234.8 | 5.75 | 19 | 31 | 0 | |

**Peptide Information**

| Calc. Mass | Obsrv. Mass | ± da | ± ppm | Start Seq. | End Seq. | Sequence | Ion Score | C. I. % | Modification | Rank | Result Type |
|---|---|---|---|---|---|---|---|---|---|---|---|
| 877.4413 | 877.423 | -0.0183 | -21 | 594 | 601 | FAPQTGEK | | | | | Mascot |
| 996.4996 | 996.6053 | 0.1057 | 106 | 1020 | 1027 | NDYKESLK | | | | | Mascot |
| 1095.5503 | 1095.5372 | -0.0131 | -12 | 87 | 96 | GNCGEYVLLK | | | | | Mascot |
| 1111.4871 | 1111.5952 | 0.1081 | 97 | 218 | 227 | VGSCEIMNSR | | | Oxidation (M)[7] | | Mascot |
| 1139.5739 | 1139.5138 | -0.0601 | -53 | 908 | 917 | SSKTACIHHR | | | | | Mascot |
| 1151.5249 | 1151.6522 | 0.1273 | 111 | 1004 | 1013 | LNTTDEEMAK | | | | | Mascot |
| 1263.6943 | 1263.6631 | -0.0312 | -25 | 729 | 740 | IIAVYGADADKK | | | | | Mascot |
| 1263.6943 | 1263.6631 | -0.0312 | -25 | 729 | 740 | IIAVYGADADKK | | | | | Mascot |
| 1322.6949 | 1322.6648 | -0.0301 | -23 | 649 | 661 | ETIGVFGKTGEGK | | | | | Mascot |
| 1351.6648 | 1351.7147 | 0.0499 | 37 | 911 | 922 | TACIHHRNNSAK | | | | | Mascot |
| 1474.6995 | 1474.6882 | -0.0113 | -8 | 582 | 593 | SSEKFMEAVQYR | | | | | Mascot |
| 1501.6517 | 1501.6599 | 0.0082 | 5 | 392 | 403 | TVCTEAMLCCLK | | | Carbamidomethyl (C)[3,9,10], Oxidation (M)[7] | | Mascot |
| 1501.6517 | 1501.6599 | 0.0082 | 5 | 392 | 403 | TVCTEAMLCCLK | | | Carbamidomethyl (C)[3,9,10], Oxidation (M)[7] | | Mascot |
| 1561.7423 | 1561.8402 | 0.0979 | 63 | 1240 | 1252 | SLMCYICESLKSK | | | Carbamidomethyl (C)[4] | | Mascot |
| 1658.8359 | 1658.9414 | 0.1055 | 64 | 186 | 198 | MNPVKQDFTFYIR | | | | | Mascot |

| | | | | | | | | | | | | |
|---|---|---|---|---|---|---|---|---|---|---|---|---|
| 1658.8359 | 1658.9414 | 0.1055 | 64 | 186 | 198 | MNPVKQDFTFYIR | | | | | Mascot |
| 1751.8641 | 1751.8743 | 0.0102 | 6 | 162 | 175 | MHNKLLLCVENFMK | | | Oxidation (M)[1,13] | | Mascot |
| 1769.0176 | 1768.8936 | -0.124 | -70 | 432 | 447 | CQGCPLKPVLTALKLK | | | Carbamidomethyl (C)[1] | | Mascot |
| 1791.7869 | 1791.9619 | 0.175 | 98 | 205 | 217 | LDYFFIPDECMWR | | | Carbamidomethyl (C)[10] | | Mascot |
| 1807.7819 | 1807.9532 | 0.1713 | 95 | 205 | 217 | LDYFFIPDECMWR | | | Carbamidomethyl (C)[10], Oxidation (M)[11] | | Mascot |
| 1993.9034 | 1994.0005 | 0.0971 | 49 | 1224 | 1239 | DSMFEEAKMQFLNEFK | | | | | Mascot |
| 2284.1277 | 2284.2034 | 0.0757 | 33 | 807 | 826 | QELLEHIVLFDVPGTGDC NK | | | Carbamidomethyl (C)[18] | | Mascot |
| 2362.1074 | 2362.1172 | 0.0098 | 4 | 779 | 797 | CHIQQSEYNPGGWYWPL VK | | | Carbamidomethyl (C)[1] | | Mascot |

44 PREDICTED: NACHT, LRR and PYD domains-containing protein 1 [Danio rerio] gi|326674408 105937.2 5.98 12 31 0

**Peptide Information**

| Calc. Mass | Obsrv. Mass | ± da | ± ppm | Start Seq. | End Seq. | Sequence | Ion Score | C. I. % | Modification | Rank | Result Type |
|---|---|---|---|---|---|---|---|---|---|---|---|
| 964.5284 | 964.4287 | -0.0997 | -103 | 433 | 440 | VAFQQLMK | | | | | Mascot |
| 980.5233 | 980.4255 | -0.0978 | -100 | 433 | 440 | VAFQQLMK | | | Oxidation (M)[7] | | Mascot |
| 1077.5497 | 1077.5269 | -0.0228 | -21 | 421 | 429 | LTDEDMILK | | | | | Mascot |
| 1280.5245 | 1280.6072 | 0.0827 | 65 | 24 | 34 | SDPEPSCVSMR | | | Carbamidomethyl (C)[7], Oxidation (M)[10] | | Mascot |
| 1363.6094 | 1363.6885 | 0.0791 | 58 | 23 | 34 | RSDPEPSCVSMR | | | | | Mascot |
| 1363.6094 | 1363.6885 | 0.0791 | 58 | 23 | 34 | RSDPEPSCVSMR | | | | | Mascot |
| 1379.6914 | 1379.6506 | -0.0408 | -30 | 326 | 337 | VTEVRGFNDSQK | | | | | Mascot |
| 1423.6774 | 1423.7625 | 0.0851 | 60 | 908 | 919 | LFSDLMDDPLNK | | | Oxidation (M)[6] | | Mascot |
| 1483.725 | 1483.6519 | -0.0731 | -49 | 257 | 268 | VLFIFDGLDECR | | | Carbamidomethyl (C)[11] | | Mascot |
| 1737.9065 | 1737.8036 | -0.1029 | -59 | 876 | 892 | GCAALASALRSNPEHLK | | | | | Mascot |
| 1768.9229 | 1768.8936 | -0.0293 | -17 | 893 | 907 | HLDLSDNLQIISSWK | | | | | Mascot |
| 2225.1052 | 2225.1482 | 0.043 | 19 | 751 | 771 | LGLYDCGLTDKGCAALA SALR | | | Carbamidomethyl (C)[6,13] | | Mascot |
| 2318.0791 | 2318.2329 | 0.1538 | 66 | 452 | 472 | ECGIDVAEASVYSGLCTQ IFR | | | Carbamidomethyl (C)[2] | | Mascot |
| 3364.7532 | 3364.6116 | -0.1416 | -42 | 384 | 412 | MLSEAETAEIPKTLTQMY THFLILQTNIK | | | | | Mascot |

45 RAB39B, member RAS oncogene family [Danio rerio] gi|121582364 24474.5 6.54 6 31 0

**Peptide Information**

| Calc. Mass | Obsrv. Mass | ± da | ± ppm | Start Seq. | End Seq. | Sequence | Ion Score | C. I. % | Modification | Rank | Result Type |
|---|---|---|---|---|---|---|---|---|---|---|---|
| 1316.6593 | 1316.6786 | 0.0193 | 15 | 60 | 70 | LQIWDTAGQER | | | | | Mascot |
| 1518.8274 | 1518.7903 | -0.0371 | -24 | 81 | 94 | NSVGGLLLFDITNR | | | | | Mascot |
| 1545.7543 | 1545.8455 | 0.0912 | 59 | 177 | 190 | NGEISIQEGWEGVK | | | | | Mascot |

| | | | | | | | | | | | | |
|---|---|---|---|---|---|---|---|---|---|---|---|---|
| 1658.8319 | 1658.9414 | 0.1095 | 66 | 141 | 155 | LAAAYGMRYVETSAR | | | | | Mascot |
| 1658.8319 | 1658.9414 | 0.1095 | 66 | 141 | 155 | LAAAYGMRYVETSAR | | | | | Mascot |
| 1800.8776 | 1800.8799 | 0.0023 | 1 | 95 | 108 | RSFQNVHEWLEEAR | | | | | Mascot |
| 2362.1462 | 2362.1172 | -0.029 | -12 | 28 | 48 | FTEGRFAQVSDPTVGVD FFSR | | | | | Mascot |

| | | | | | | | | |
|---|---|---|---|---|---|---|---|---|
| 46 | brain-derived neurotrophic factor [Danio rerio] | gi\|169145642 | 25880.8 | 5.62 | 6 | 31 | 0 | |

Peptide Information

| Calc. Mass | Obsrv. Mass | ± da | ± ppm | Start Seq. | End Seq. | Sequence | Ion Score | C. I. % | Modification | Rank | Result Type |
|---|---|---|---|---|---|---|---|---|---|---|---|
| 877.4196 | 877.423 | 0.0034 | 4 | 225 | 232 | EGCRGIDK | | | | | Mascot |
| 964.4053 | 964.4287 | 0.0234 | 24 | 233 | 239 | RHYNSQC | | | Carbamidomethyl (C)[7] | | Mascot |
| 980.5094 | 980.4255 | -0.0839 | -86 | 1 | 7 | MFQQVRR | | | Oxidation (M)[1] | | Mascot |
| 1993.9535 | 1994.0005 | 0.047 | 24 | 167 | 184 | GELSVCDSISQWVTAVD K | | | Carbamidomethyl (C)[6] | | Mascot |
| 2545.3542 | 2545.3594 | 0.0052 | 2 | 186 | 209 | TAIDMSGQTVTVLEKVPV TNGQLK | | | Oxidation (M)[5] | | Mascot |
| 2545.3542 | 2545.3594 | 0.0052 | 2 | 186 | 209 | TAIDMSGQTVTVLEKVPV TNGQLK | | | Oxidation (M)[5] | | Mascot |
| 2683.3301 | 2683.2405 | -0.0896 | -33 | 8 | 30 | VMTILFVTMVISYFSCMR AAPMR | | | Oxidation (M)[2] | | Mascot |

| | | | | | | | | |
|---|---|---|---|---|---|---|---|---|
| 47 | novel protein similar to H.sapiens TTN, titin (TTN) [Danio rerio] | gi\|169154823 | 842970 | 5.81 | 39 | 31 | 0 | |

Peptide Information

| Calc. Mass | Obsrv. Mass | ± da | ± ppm | Start Seq. | End Seq. | Sequence | Ion Score | C. I. % | Modification | Rank | Result Type |
|---|---|---|---|---|---|---|---|---|---|---|---|
| 842.444 | 842.5162 | 0.0722 | 86 | 3102 | 3108 | SYKVSMK | | | | | Mascot |
| 877.4447 | 877.423 | -0.0217 | -25 | 6550 | 6557 | NLSVEMGK | | | | | Mascot |
| 882.4679 | 882.575 | 0.1071 | 121 | 2483 | 2489 | YKVSTER | | | | | Mascot |
| 964.4227 | 964.4287 | 0.006 | 6 | 4320 | 4327 | LHTTMDCK | | | Oxidation (M)[5] | | Mascot |
| 980.4366 | 980.4255 | -0.0111 | -11 | 1600 | 1606 | YRMHSDR | | | Oxidation (M)[3] | | Mascot |
| 996.536 | 996.6053 | 0.0693 | 70 | 2586 | 2593 | LTITETYR | | | | | Mascot |
| 1045.5347 | 1045.5701 | 0.0354 | 34 | 6656 | 6665 | CQIGGTPEIK | | | | | Mascot |
| 1064.5657 | 1064.5403 | -0.0254 | -24 | 7229 | 7237 | KETTVEMVK | | | | | Mascot |
| 1077.5323 | 1077.5269 | -0.0054 | -5 | 1326 | 1336 | APEAPGPEPGR | | | | | Mascot |
| 1095.5867 | 1095.5372 | -0.0495 | -45 | 4924 | 4933 | VSCTAALFVK | | | Carbamidomethyl (C)[3] | | Mascot |
| 1100.5582 | 1100.6183 | 0.0601 | 55 | 5147 | 5156 | EPANITEQAK | | | | | Mascot |
| 1111.5928 | 1111.5952 | 0.0024 | 2 | 7404 | 7414 | IVCGPHISSAK | | | | | Mascot |
| 1151.5249 | 1151.6522 | 0.1273 | 111 | 2297 | 2306 | NGVEIEMSEK | | | Oxidation (M)[7] | | Mascot |
| 1263.6368 | 1263.6631 | 0.0263 | 21 | 5550 | 5560 | GTAPFEVTWQK | | | | | Mascot |

| | | | | | | | | |
|---|---|---|---|---|---|---|---|---|
| 1263.6368 | 1263.6631 | 0.0263 | 21 | 5550 | 5560 | GTAPFEVTWQK | | Mascot |
| 1280.6337 | 1280.6072 | -0.0265 | -21 | 5020 | 5032 | CSCLVTVKGDAGK | | Mascot |
| 1316.5684 | 1316.6786 | 0.1102 | 84 | 5081 | 5091 | GSMPMTISWMK | Oxidation (M)[3,5,10] | Mascot |
| 1352.6844 | 1352.7048 | 0.0204 | 15 | 6293 | 6304 | WFKDGVELSSGK | | Mascot |
| 1352.6844 | 1352.7048 | 0.0204 | 15 | 6293 | 6304 | WFKDGVELSSGK | | Mascot |
| 1379.6835 | 1379.6506 | -0.0329 | -24 | 5376 | 5388 | KEVSSGIDCSVQK | | Mascot |
| 1502.7308 | 1502.7963 | 0.0655 | 44 | 4983 | 4995 | YQMAFTDSVATLR | | Mascot |
| 1518.7257 | 1518.7903 | 0.0646 | 43 | 4983 | 4995 | YQMAFTDSVATLR | Oxidation (M)[3] | Mascot |
| 1529.6359 | 1529.8517 | 0.2158 | 141 | 5689 | 5701 | YTCQAMNEAGQEK | Carbamidomethyl (C)[3] | Mascot |
| 1545.6307 | 1545.8455 | 0.2148 | 139 | 5689 | 5701 | YTCQAMNEAGQEK | Carbamidomethyl (C)[3], Oxidation (M)[6] | Mascot |
| 1561.7605 | 1561.8402 | 0.0797 | 51 | 3296 | 3308 | EHSEVIPHTQQEK | | Mascot |
| 1593.7908 | 1593.7393 | -0.0515 | -32 | 5550 | 5563 | GTAPFEVTWQKDSK | | Mascot |
| 1593.7908 | 1593.7393 | -0.0515 | -32 | 5550 | 5563 | GTAPFEVTWQKDSK | | Mascot |
| 1638.9061 | 1638.8779 | -0.0282 | -17 | 7253 | 7266 | DIDIVELLRNVDPK | | Mascot |
| 1716.9166 | 1717.0227 | 0.1061 | 62 | 2970 | 2984 | VELSQLSAAEKWVEK | | Mascot |
| 1721.9506 | 1721.8103 | -0.1403 | -81 | 5625 | 5639 | IENVVTVLGKMVEFK | Oxidation (M)[11] | Mascot |
| 1751.7177 | 1751.8743 | 0.1566 | 89 | 4062 | 4077 | DHEGEYTCEAVGEAGK | Carbamidomethyl (C)[8] | Mascot |
| 1807.9041 | 1807.9532 | 0.0491 | 27 | 1897 | 1912 | MAVGRVETTCNLTVEK | Carbamidomethyl (C)[10] | Mascot |
| 1859.928 | 1859.9314 | 0.0034 | 2 | 2211 | 2226 | NQELKPSANVQIMSER | Oxidation (M)[13] | Mascot |
| 1859.928 | 1859.9314 | 0.0034 | 2 | 2211 | 2226 | NQELKPSANVQIMSER | Oxidation (M)[13] | Mascot |
| 1993.9358 | 1994.0005 | 0.0647 | 32 | 4299 | 4315 | LECQVDEDTGVSIIWMR | | Mascot |
| 2016.0253 | 2016.0427 | 0.0174 | 9 | 5993 | 6010 | MGEPLQLECKVTGTAPLR | Carbamidomethyl (C)[9], Oxidation (M)[1] | Mascot |
| 2032.0167 | 2032.0435 | 0.0268 | 13 | 6754 | 6772 | VAGSSPMEVSWLKDGEALR | | Mascot |
| 2177.1304 | 2176.9849 | -0.1455 | -67 | 5062 | 5080 | MDNTNVLLAKDVSLQCVLK | Carbamidomethyl (C)[16], Oxidation (M)[1] | Mascot |
| 2211.1438 | 2211.1294 | -0.0144 | -7 | 6494 | 6513 | SCEIALRGDTAILELHNIDK | | Mascot |
| 2211.1438 | 2211.1294 | -0.0144 | -7 | 6494 | 6513 | SCEIALRGDTAILELHNIDK | | Mascot |
| 2232.9575 | 2233.1099 | 0.1524 | 68 | 1132 | 1149 | MINEFGYCSLDYEVAYSR | Carbamidomethyl (C)[8], Oxidation (M)[1] | Mascot |
| 2283.1899 | 2283.2114 | 0.0215 | 9 | 3240 | 3260 | EHPSQVMSALTESVTPLTIVK | Oxidation (M)[7] | Mascot |
| 2318.1003 | 2318.2329 | 0.1326 | 57 | 5854 | 5872 | HKILQDENSMSLDIECLEK | Carbamidomethyl (C)[16], Oxidation (M)[10] | Mascot |
| 2379.092 | 2379.3738 | 0.2818 | 118 | 4144 | 4165 | HHTSSLEIFSPAVADSGEYTCK | | Mascot |
| 2379.1709 | 2379.3738 | 0.2029 | 85 | 7325 | 7345 | MEQTEPVTVARDITDQTTTVK | Oxidation (M)[1] | Mascot |
| 2545.1196 | 2545.3594 | 0.2398 | 94 | 4481 | 4502 | GCTAHLECEMTGTAPFEITWFK | Carbamidomethyl (C)[2], Oxidation (M)[10] | Mascot |
| 2545.1196 | 2545.3594 | 0.2398 | 94 | 4481 | 4502 | GCTAHLECEMTGTAPFEITWFK | Carbamidomethyl (C)[2], Oxidation (M)[10] | Mascot |

48 cartilage acidic protein 1a [Danio rerio] gi|122114565 58974.9 4.83 9 31 0

Peptide Information

| Calc. Mass | Obsrv. Mass | ± da | ± ppm | Start Seq. | End Seq. | Sequence | Ion Score | C. I. % | Modification | Rank | Result Type |
|---|---|---|---|---|---|---|---|---|---|---|---|
| 877.4559 | 877.423 | -0.0329 | -37 | 167 | 174 | SVACVDRK | | | | | Mascot |
| 980.5597 | 980.4255 | -0.1342 | -137 | 445 | 453 | GAKVVMFTK | | | | | Mascot |
| 996.5546 | 996.6053 | 0.0507 | 51 | 445 | 453 | GAKVVMFTK | | | Oxidation (M)[6] | | Mascot |
| 1151.5691 | 1151.6522 | 0.0831 | 72 | 307 | 315 | LYLQDSQER | | | | | Mascot |
| 1502.7533 | 1502.7963 | 0.043 | 29 | 448 | 460 | VVMFTKHSGSHTR | | | Oxidation (M)[3] | | Mascot |
| 1658.7849 | 1658.9414 | 0.1565 | 94 | 158 | 173 | GVANHMAGRSVACVDR | | | Oxidation (M)[6] | | Mascot |
| 1658.7849 | 1658.9414 | 0.1565 | 94 | 158 | 173 | GVANHMAGRSVACVDR | | | Oxidation (M)[6] | | Mascot |
| 1716.9391 | 1717.0227 | 0.0836 | 49 | 225 | 241 | YTGGRGVVVGPILSDAR | | | | | Mascot |
| 1859.8187 | 1859.9314 | 0.1127 | 61 | 97 | 115 | DPAGNAIGVTACDVDGD GR | | | Carbamidomethyl (C)[12] | | Mascot |
| 1859.8268 | 1859.9314 | 0.1046 | 56 | 242 | 257 | SDIFCDNENGPNFLFK | | | | | Mascot |
| 2661.2805 | 2661.2351 | -0.0454 | -17 | 294 | 315 | TDIVYGNWNGPHRLYLQ DSQER | | | | | Mascot |

| | | | | | | | |
|---|---|---|---|---|---|---|---|
| 49 | PREDICTED: DENN domain-containing protein 2A [Danio rerio] | gi\|326679977 | 79687.7 | 8.23 | 10 | 30 | 0 |

Peptide Information

| Calc. Mass | Obsrv. Mass | ± da | ± ppm | Start Seq. | End Seq. | Sequence | Ion Score | C. I. % | Modification | Rank | Result Type |
|---|---|---|---|---|---|---|---|---|---|---|---|
| 964.4516 | 964.4287 | -0.0229 | -24 | 2 | 11 | PSASTMTGGR | | | | | Mascot |
| 980.4465 | 980.4255 | -0.021 | -21 | 2 | 11 | PSASTMTGGR | | | Oxidation (M)[6] | | Mascot |
| 1095.4922 | 1095.5372 | 0.045 | 41 | 1 | 11 | MPSASTMTGGR | | | | | Mascot |
| 1111.4871 | 1111.5952 | 0.1081 | 97 | 1 | 11 | MPSASTMTGGR | | | Oxidation (M)[1] | | Mascot |
| 1280.6189 | 1280.6072 | -0.0117 | -9 | 193 | 204 | RVSQSTESSSGR | | | | | Mascot |
| 1501.8308 | 1501.6599 | -0.1709 | -114 | 379 | 391 | RAISPALVQPFMR | | | Oxidation (M)[12] | | Mascot |
| 1501.8308 | 1501.6599 | -0.1709 | -114 | 379 | 391 | RAISPALVQPFMR | | | Oxidation (M)[12] | | Mascot |
| 1518.7581 | 1518.7903 | 0.0322 | 21 | 242 | 253 | TLERDLMDLQER | | | | | Mascot |
| 1800.9677 | 1800.8799 | -0.0878 | -49 | 392 | 408 | GIMEAPFPAPGRTISIK | | | Oxidation (M)[3] | | Mascot |
| 2016.1165 | 2016.0427 | -0.0738 | -37 | 267 | 284 | TKAGVPYLPEVTQQFPLK | | | | | Mascot |
| 2284.0662 | 2284.2034 | 0.1372 | 60 | 668 | 687 | GLFEMRAQDYLDSLPGG ENR | | | Oxidation (M)[5] | | Mascot |
| 2379.1624 | 2379.3738 | 0.2114 | 89 | 641 | 659 | RFLEVFMETQMFAGFLQ ER | | | | | Mascot |
| 2379.1624 | 2379.3738 | 0.2114 | 89 | 641 | 659 | RFLEVFMETQMFAGFLQ ER | | | | | Mascot |
| 2545.1624 | 2545.3594 | 0.197 | 77 | 144 | 167 | VSPPSTPSSPDDTPCLS GDPYNRR | | | | | Mascot |
| 2545.1624 | 2545.3594 | 0.197 | 77 | 144 | 167 | VSPPSTPSSPDDTPCLS GDPYNRR | | | | | Mascot |

| 50 | NOP56 [Danio rerio] | gi\|49619095 | 60635 | 8.72 | 9 | 30 | 0 |
|---|---|---|---|---|---|---|---|

Peptide Information

| Calc. Mass | Obsrv. Mass | ± da | ± ppm | Start Seq. | End Seq. | Sequence | Ion Score | C. I. % | Modification | Rank | Result Type |
|---|---|---|---|---|---|---|---|---|---|---|---|
| 964.5574 | 964.4287 | -0.1287 | -133 | 369 | 376 | ISRYLANK | | | | | Mascot |
| 980.5676 | 980.4255 | -0.1421 | -145 | 128 | 135 | LHFHSLVK | | | | | Mascot |
| 1045.571 | 1045.5701 | -0.0009 | -1 | 91 | 100 | KPMLGVSDAK | | | | | Mascot |
| 1275.6943 | 1275.6111 | -0.0832 | -65 | 78 | 89 | LFLETNLPSGGK | | | | | Mascot |
| 1751.9591 | 1751.8743 | -0.0848 | -48 | 349 | 364 | YGLIFHSTFIGRAAAK | | | | | Mascot |
| 1808.0177 | 1807.9532 | -0.0645 | -36 | 128 | 144 | LHFHSLVKGLTAQAASK | | | | | Mascot |
| 2016.0621 | 2016.0427 | -0.0194 | -10 | 136 | 155 | GLTAQAASKAQLGLGHS YSR | | | | | Mascot |
| 2211.2358 | 2211.1294 | -0.1064 | -48 | 1 | 20 | MVLLHVLFEHAAGYALLA VK | | | Oxidation (M)[1] | | Mascot |
| 2211.2358 | 2211.1294 | -0.1064 | -48 | 1 | 20 | MVLLHVLFEHAAGYALLA VK | | | Oxidation (M)[1] | | Mascot |
| 2362.0347 | 2362.1172 | 0.0825 | 35 | 222 | 241 | KELSEEMLEAMEEITMDS AK | | | Oxidation (M)[7,11,16] | | Mascot |

| Gel Idx/Pos | 175/H2 | Instr./Gel Origin | BA2060/110707A | Process Status | Analysis Succeeded |
|---|---|---|---|---|---|
| Plate [#] Name | [1] 31496 | Instrument Sample Name | | Spectra | 11 |

| Rank | Protein Name | Accession No. | Protein MW | Protein PI | Pep. Count | Protein Score | Protein Score C. I. % | Total Ion Score | Total Ion C. I. % |
|---|---|---|---|---|---|---|---|---|---|
| 1 | novel protein similar to H.sapiens NCL, nucleolin (NCL, zgc:152810) [Danio rerio] | gi\|220672795 | 74887.4 | 4.62 | 19 | 557 | 100 | 459 | 100 |

Peptide Information

| Calc. Mass | Obsrv. Mass | ± da | ± ppm | Start Seq. | End Seq. | Sequence | Ion Score | C. I. % | Modification | Rank | Result Type |
|---|---|---|---|---|---|---|---|---|---|---|---|
| 882.577 | 882.5717 | -0.0053 | -6 | 349 | 356 | KLLGQPVK | | | | | Mascot |
| 940.5137 | 940.5209 | 0.0072 | 8 | 414 | 421 | GIAYIEFK | | | | | Mascot |
| 1022.4901 | 1022.5009 | 0.0108 | 11 | 571 | 580 | DSFDGAIAAR | | | | | Mascot |
| 1024.4694 | 1024.5231 | 0.0537 | 52 | 532 | 539 | LEYSQNDR | | | | | Mascot |
| 1058.5476 | 1058.5569 | 0.0093 | 9 | 313 | 321 | EGLEIQDVR | | | | | Mascot |
| 1146.6014 | 1146.6381 | 0.0367 | 32 | 689 | 699 | GGFGDRPQGKK | | | | | Mascot |
| 1205.6161 | 1205.6255 | 0.0094 | 8 | 394 | 403 | EIFDQAVDIR | 86 | 100 | | | Mascot |
| 1205.6161 | 1205.6255 | 0.0094 | 8 | 394 | 403 | EIFDQAVDIR | | | | | Mascot |
| 1252.642 | 1252.7349 | 0.0929 | 74 | 298 | 308 | DFDELKSAISK | | | | | Mascot |
| 1309.6132 | 1309.6219 | 0.0087 | 7 | 532 | 541 | LEYSQNDRER | | | | | Mascot |
| 1359.6499 | 1359.6603 | 0.0104 | 8 | 429 | 441 | ALEEAQGSDVQGR | | | | | Mascot |

| Calc. Mass | Obsrv. Mass | ± da | ± ppm | Start Seq. | End Seq. | Sequence | Ion Score | C. I. % | Modification | Rank | Result Type |
|---|---|---|---|---|---|---|---|---|---|---|---|
| 1380.6866 | 1380.6865 | -0.0001 | 0 | 529 | 539 | SIRLEYSQNDR | | | | | Mascot |
| 1434.7224 | 1434.7333 | 0.0109 | 8 | 382 | 393 | NLPYSITQDDLR | | | | | Mascot |
| 1434.7224 | 1434.7333 | 0.0109 | 8 | 382 | 393 | NLPYSITQDDLR | 97 | 100 | | | Mascot |
| 1563.6956 | 1563.7495 | 0.0539 | 34 | 606 | 620 | AAKEAMDDGEIDGNK | | | | | Mascot |
| 1563.6956 | 1563.7495 | 0.0539 | 34 | 606 | 620 | AAKEAMDDGEIDGNK | | | | | Mascot |
| 1661.7693 | 1661.7815 | 0.0122 | 7 | 328 | 341 | FGYVDFASEEELQK | | | | | Mascot |
| 1677.6849 | 1677.6991 | 0.0142 | 8 | 592 | 605 | GFGFVDFDNEQDCK | 74 | 99.999 | Carbamidomethyl (C)[13] | | Mascot |
| 1677.6849 | 1677.6991 | 0.0142 | 8 | 592 | 605 | GFGFVDFDNEQDCK | | | Carbamidomethyl (C)[13] | | Mascot |
| 2297.0891 | 2297.1531 | 0.064 | 28 | 559 | 580 | GLSDDTTDQTLKDSFDG AIAAR | | | | | Mascot |
| 2396.2344 | 2396.2654 | 0.031 | 13 | 465 | 486 | VLVVNNLAFSASEDSLQS VFEK | | | | | Mascot |
| 2587.231 | 2587.2673 | 0.0363 | 14 | 281 | 303 | TDGEGFSLFLGNLNNNK DFDELK | | | | | Mascot |
| 3249.4275 | 3249.4709 | 0.0434 | 13 | 501 | 528 | GYAFVEFENVEDSKEALE NCNNTDIEGR | | | Carbamidomethyl (C)[20] | | Mascot |
| 3249.4275 | 3249.4709 | 0.0434 | 13 | 501 | 528 | GYAFVEFENVEDSKEALE NCNNTDIEGR | 202 | 100 | Carbamidomethyl (C)[20] | | Mascot |

2 nucleolin [Danio rerio] gi|49618989 70862.8 4.74 17 543 100 459 100

**Peptide Information**

| Calc. Mass | Obsrv. Mass | ± da | ± ppm | Start Seq. | End Seq. | Sequence | Ion Score | C. I. % | Modification | Rank | Result Type |
|---|---|---|---|---|---|---|---|---|---|---|---|
| 882.577 | 882.5717 | -0.0053 | -6 | 312 | 319 | KLLGQPVK | | | | | Mascot |
| 1022.4901 | 1022.5009 | 0.0108 | 11 | 534 | 543 | DSFDGAIAAR | | | | | Mascot |
| 1024.4694 | 1024.5231 | 0.0537 | 52 | 495 | 502 | LEYSQNDR | | | | | Mascot |
| 1058.5476 | 1058.5569 | 0.0093 | 9 | 276 | 284 | EGLEIQDVR | | | | | Mascot |
| 1205.6161 | 1205.6255 | 0.0094 | 8 | 357 | 366 | EIFDQAVDIR | 86 | 100 | | | Mascot |
| 1205.6161 | 1205.6255 | 0.0094 | 8 | 357 | 366 | EIFDQAVDIR | | | | | Mascot |
| 1252.642 | 1252.7349 | 0.0929 | 74 | 261 | 271 | DFDELKSAISK | | | | | Mascot |
| 1309.6132 | 1309.6219 | 0.0087 | 7 | 495 | 504 | LEYSQNDRER | | | | | Mascot |
| 1359.6499 | 1359.6603 | 0.0104 | 8 | 392 | 404 | ALEEAQGSDVQGR | | | | | Mascot |
| 1380.6866 | 1380.6865 | -0.0001 | 0 | 492 | 502 | SIRLEYSQNDR | | | | | Mascot |
| 1434.7224 | 1434.7333 | 0.0109 | 8 | 345 | 356 | NLPYSITQDDLR | | | | | Mascot |
| 1434.7224 | 1434.7333 | 0.0109 | 8 | 345 | 356 | NLPYSITQDDLR | 97 | 100 | | | Mascot |
| 1563.6956 | 1563.7495 | 0.0539 | 34 | 569 | 583 | AAKEAMDDGEIDGNK | | | | | Mascot |
| 1563.6956 | 1563.7495 | 0.0539 | 34 | 569 | 583 | AAKEAMDDGEIDGNK | | | | | Mascot |
| 1661.7693 | 1661.7815 | 0.0122 | 7 | 291 | 304 | FGYVDFASEEELQK | | | | | Mascot |
| 1677.6849 | 1677.6991 | 0.0142 | 8 | 555 | 568 | GFGFVDFDNEQDCK | 74 | 99.999 | Carbamidomethyl (C)[13] | | Mascot |
| 1677.6849 | 1677.6991 | 0.0142 | 8 | 555 | 568 | GFGFVDFDNEQDCK | | | Carbamidomethyl (C)[13] | | Mascot |

| | | | | | | | | | | | |
|---|---|---|---|---|---|---|---|---|---|---|---|
| 2297.0891 | 2297.1531 | 0.064 | 28 | 522 | 543 | GLSDDTTDQTLKDSFDG AIAAR | | | | | Mascot |
| 2396.2344 | 2396.2654 | 0.031 | 13 | 428 | 449 | VLVVNNLAFSASEDSLQS VFEK | | | | | Mascot |
| 2587.231 | 2587.2673 | 0.0363 | 14 | 244 | 266 | TDGEGFSLFLGNLNNNK DFDELK | | | | | Mascot |
| 3249.4275 | 3249.4709 | 0.0434 | 13 | 464 | 491 | GYAFVEFENVEDSKEALE NCNNTDIEGR | | | Carbamidomethyl (C)[20] | | Mascot |
| 3249.4275 | 3249.4709 | 0.0434 | 13 | 464 | 491 | GYAFVEFENVEDSKEALE NCNNTDIEGR | 202 | 100 | Carbamidomethyl (C)[20] | | Mascot |

| | | | | | | | | | | |
|---|---|---|---|---|---|---|---|---|---|---|
| 3 | PREDICTED: neutral alpha-glucosidase AB-like [Danio rerio] | gi\|292621001 | 106709.9 | 5.34 | 25 | 389 | 100 | 256 | 100 | |

**Peptide Information**

| Calc. Mass | Obsrv. Mass | ± da | ± ppm | Start Seq. | End Seq. | Sequence | Ion Score | C. I. % | Modification | Rank | Result Type |
|---|---|---|---|---|---|---|---|---|---|---|---|
| 883.442 | 883.4474 | 0.0054 | 6 | 595 | 602 | AFFAGSQR | | | | | Mascot |
| 973.5061 | 973.5176 | 0.0115 | 12 | 574 | 582 | ATAEGQIQR | | | | | Mascot |
| 1041.584 | 1041.5912 | 0.0072 | 7 | 168 | 176 | GLLGFEHLR | | | | | Mascot |
| 1058.6469 | 1058.5569 | -0.09 | -85 | 143 | 151 | LIVSARPFR | | | | | Mascot |
| 1155.6885 | 1155.6981 | 0.0096 | 8 | 79 | 87 | VHLLLELYR | | | | | Mascot |
| 1231.5194 | 1231.6849 | 0.1655 | 134 | 34 | 43 | TCDQSSFCKR | | | Carbamidomethyl (C)[2] | | Mascot |
| 1252.6024 | 1252.7349 | 0.1325 | 106 | 444 | 453 | EMLQGLMDKR | | | Oxidation (M)[2,7] | | Mascot |
| 1317.7273 | 1317.7367 | 0.0094 | 7 | 583 | 594 | SGGVERPFVLTR | | | | | Mascot |
| 1317.7273 | 1317.7367 | 0.0094 | 7 | 583 | 594 | SGGVERPFVLTR | 8 | 0 | | | Mascot |
| 1332.7006 | 1332.71 | 0.0094 | 7 | 56 | 67 | ALLDTLELSDSR | | | | | Mascot |
| 1434.9154 | 1434.7333 | -0.1821 | -127 | 95 | 106 | VKINELKPLKPR | | | | | Mascot |
| 1434.9154 | 1434.7333 | -0.1821 | -127 | 95 | 106 | VKINELKPLKPR | | | | | Mascot |
| 1448.7532 | 1448.757 | 0.0038 | 3 | 562 | 573 | DVHNIYGLYVQK | | | | | Mascot |
| 1451.7489 | 1451.7589 | 0.01 | 7 | 743 | 755 | DLLVHPVTEEGSR | | | | | Mascot |
| 1563.7379 | 1563.7495 | 0.0116 | 7 | 655 | 666 | WYQTGAYQPFFR | | | | | Mascot |
| 1563.7379 | 1563.7495 | 0.0116 | 7 | 655 | 666 | WYQTGAYQPFFR | 91 | 100 | | | Mascot |
| 1642.8588 | 1642.8677 | 0.0089 | 5 | 677 | 690 | EPWLFGPENTALIR | | | | | Mascot |
| 1691.8217 | 1691.8334 | 0.0117 | 7 | 431 | 443 | YFTWDPHKFPEPK | | | | | Mascot |
| 1727.8486 | 1727.8621 | 0.0135 | 8 | 728 | 742 | ETATFTIDDEFLIGR | | | | | Mascot |
| 1727.8486 | 1727.8621 | 0.0135 | 8 | 728 | 742 | ETATFTIDDEFLIGR | 115 | 100 | | | Mascot |
| 1785.9415 | 1785.9579 | 0.0164 | 9 | 152 | 167 | LDIMEGPEVLLSLNSR | | | | | Mascot |
| 1798.9598 | 1798.9722 | 0.0124 | 7 | 676 | 690 | REPWLFGPENTALIR | | | | | Mascot |
| 1798.9598 | 1798.9722 | 0.0124 | 7 | 676 | 690 | REPWLFGPENTALIR | 43 | 98.55 | | | Mascot |
| 1860.8663 | 1860.8818 | 0.0155 | 8 | 603 | 619 | YGAVWTGDNAADWGHL K | | | | | Mascot |
| 1909.896 | 1909.9108 | 0.0148 | 8 | 331 | 347 | MLDFVQGSSETPQTDVR | | | | | Mascot |

| | | | | | | | | | | | |
|---|---|---|---|---|---|---|---|---|---|---|---|
| 1925.891 | 1925.9102 | 0.0192 | 10 | 331 | 347 | MLDFVQGSSETPQTDVR | | | Oxidation (M)[1] | | Mascot |
| 2028.0695 | 2028.0717 | 0.0022 | 1 | 921 | 938 | KPGINAGMDWTILLQSQR | | | | | Mascot |
| 2044.0645 | 2044.0875 | 0.023 | 11 | 921 | 938 | KPGINAGMDWTILLQSQR | | | Oxidation (M)[8] | | Mascot |
| 2071.0051 | 2071.0205 | 0.0154 | 7 | 903 | 920 | ETPVEFEFDASMSVLTLR | | | | | Mascot |
| 2087.0002 | 2087.0176 | 0.0174 | 8 | 903 | 920 | ETPVEFEFDASMSVLTLR | | | Oxidation (M)[12] | | Mascot |
| 2233.0515 | 2233.1084 | 0.0569 | 25 | 811 | 830 | SSACMENDPYTLYVALSPKK | | | Oxidation (M)[5] | | Mascot |
| 2354.2615 | 2354.2368 | -0.0247 | -10 | 777 | 797 | HNGAQNLYIPVTLSSIPVFQR | | | | | Mascot |
| 2677.0872 | 2677.1145 | 0.0273 | 10 | 490 | 511 | NYEGWCWPGNSGYPDFTNPEMR | | | Carbamidomethyl (C)[6] | | Mascot |

| | | | | | | | | | | |
|---|---|---|---|---|---|---|---|---|---|---|
| 4 | PREDICTED: neutral alpha-glucosidase AB-like [Danio rerio] | gi\|326673856 | 84660.6 | 5.01 | 19 | 272 | 100 | 178 | 100 | |

**Peptide Information**

| Calc. Mass | Obsrv. Mass | ± da | ± ppm | Start Seq. | End Seq. | Sequence | Ion Score | C. I. % | Modification | Rank | Result Type |
|---|---|---|---|---|---|---|---|---|---|---|---|
| 883.442 | 883.4474 | 0.0054 | 6 | 595 | 602 | AFFAGSQR | | | | | Mascot |
| 973.5061 | 973.5176 | 0.0115 | 12 | 574 | 582 | ATAEGQIQR | | | | | Mascot |
| 1055.5996 | 1055.606 | 0.0064 | 6 | 168 | 176 | GLLAFEHLR | 37 | 94.65 | | | Mascot |
| 1055.5996 | 1055.606 | 0.0064 | 6 | 168 | 176 | GLLAFEHLR | | | | | Mascot |
| 1058.6469 | 1058.5569 | -0.09 | -85 | 143 | 151 | LIVSARPFR | | | | | Mascot |
| 1146.6266 | 1146.6381 | 0.0115 | 10 | 46 | 55 | TLKPGQSPYR | | | | | Mascot |
| 1155.6885 | 1155.6981 | 0.0096 | 8 | 79 | 87 | VHLLLELYR | | | | | Mascot |
| 1231.5194 | 1231.6849 | 0.1655 | 134 | 34 | 43 | TCDQSSFCKR | | | Carbamidomethyl (C)[2] | | Mascot |
| 1317.7273 | 1317.7367 | 0.0094 | 7 | 583 | 594 | SGGVERPFVLTR | | | | | Mascot |
| 1317.7273 | 1317.7367 | 0.0094 | 7 | 583 | 594 | SGGVERPFVLTR | 8 | 0 | | | Mascot |
| 1332.7006 | 1332.71 | 0.0094 | 7 | 56 | 67 | ALLDTLELSDSR | | | | | Mascot |
| 1434.9154 | 1434.7333 | -0.1821 | -127 | 95 | 106 | VKINELKPLKPR | | | | | Mascot |
| 1434.9154 | 1434.7333 | -0.1821 | -127 | 95 | 106 | VKINELKPLKPR | | | | | Mascot |
| 1448.7532 | 1448.757 | 0.0038 | 3 | 562 | 573 | DVHNIYGLYVQK | | | | | Mascot |
| 1563.7379 | 1563.7495 | 0.0116 | 7 | 655 | 666 | WYQTGAYQPFFR | | | | | Mascot |
| 1563.7379 | 1563.7495 | 0.0116 | 7 | 655 | 666 | WYQTGAYQPFFR | 91 | 100 | | | Mascot |
| 1642.8588 | 1642.8677 | 0.0089 | 5 | 677 | 690 | EPWLFGPENTALIR | | | | | Mascot |
| 1691.8217 | 1691.8334 | 0.0117 | 7 | 431 | 443 | YFTWDPHKFPEPK | | | | | Mascot |
| 1785.9415 | 1785.9579 | 0.0164 | 9 | 152 | 167 | LDIMEGPEVLLSLNSR | | | | | Mascot |
| 1798.9598 | 1798.9722 | 0.0124 | 7 | 676 | 690 | REPWLFGPENTALIR | | | | | Mascot |
| 1798.9598 | 1798.9722 | 0.0124 | 7 | 676 | 690 | REPWLFGPENTALIR | 43 | 98.55 | | | Mascot |
| 1860.8663 | 1860.8818 | 0.0155 | 8 | 603 | 619 | YGAVWTGDNAADWGHLK | | | | | Mascot |
| 1909.896 | 1909.9108 | 0.0148 | 8 | 331 | 347 | MLDFVQGSSETPQTDVR | | | | | Mascot |

| Calc. Mass | Obsrv. Mass | ± da | ± ppm | Start Seq. | End Seq. | Sequence | Ion Score | C. I. % | Modification | Rank | Result Type |
|---|---|---|---|---|---|---|---|---|---|---|---|
| 1925.891 | 1925.9102 | 0.0192 | 10 | 331 | 347 | MLDFVQGSSETPQTDVR | | | Oxidation (M)[1] | | Mascot |
| 2677.0872 | 2677.1145 | 0.0273 | 10 | 490 | 511 | NYEGWCWPGNSGYPDF TNPEMR | | | Carbamidomethyl (C)[6] | | Mascot |

| | | | | | | | | | | |
|---|---|---|---|---|---|---|---|---|---|---|
| 5 | Zgc:171967 protein [Danio rerio] | gi\|190339135 | 104064.6 | 5.22 | 12 | 120 | 100 | 88 | 100 | |

**Peptide Information**

| Calc. Mass | Obsrv. Mass | ± da | ± ppm | Start Seq. | End Seq. | Sequence | Ion Score | C. I. % | Modification | Rank | Result Type |
|---|---|---|---|---|---|---|---|---|---|---|---|
| 883.442 | 883.4474 | 0.0054 | 6 | 576 | 583 | AFFAGSQR | | | | | Mascot |
| 996.5585 | 996.6018 | 0.0433 | 43 | 453 | 460 | IHNEIRSK | | | | | Mascot |
| 1055.5996 | 1055.606 | 0.0064 | 6 | 163 | 171 | GLLAFEHLR | 37 | 94.65 | | | Mascot |
| 1055.5996 | 1055.606 | 0.0064 | 6 | 163 | 171 | GLLAFEHLR | | | | | Mascot |
| 1205.5811 | 1205.6255 | 0.0444 | 37 | 533 | 542 | DAVHGVWEHR | | | | | Mascot |
| 1205.5811 | 1205.6255 | 0.0444 | 37 | 533 | 542 | DAVHGVWEHR | | | | | Mascot |
| 1231.5194 | 1231.6849 | 0.1655 | 134 | 29 | 38 | TCDQSSFCKR | | | Carbamidomethyl (C)[2] | | Mascot |
| 1309.6383 | 1309.6219 | -0.0164 | -13 | 253 | 263 | LKSTENSDPYR | | | | | Mascot |
| 1317.7273 | 1317.7367 | 0.0094 | 7 | 564 | 575 | SGGVERPFVLTR | | | | | Mascot |
| 1317.7273 | 1317.7367 | 0.0094 | 7 | 564 | 575 | SGGVERPFVLTR | 8 | 0 | | | Mascot |
| 1332.7006 | 1332.71 | 0.0094 | 7 | 51 | 62 | ALLDTLELSDSR | | | | | Mascot |
| 1434.9154 | 1434.7333 | -0.1821 | -127 | 90 | 101 | VKINELKPIKPR | | | | | Mascot |
| 1434.9154 | 1434.7333 | -0.1821 | -127 | 90 | 101 | VKINELKPIKPR | | | | | Mascot |
| 1448.7532 | 1448.757 | 0.0038 | 3 | 543 | 554 | DVHNLYGLYVQK | | | | | Mascot |
| 1642.8588 | 1642.8677 | 0.0089 | 5 | 658 | 671 | EPWLFGPENTALIR | | | | | Mascot |
| 1798.9598 | 1798.9722 | 0.0124 | 7 | 657 | 671 | REPWLFGPENTALIR | | | | | Mascot |
| 1798.9598 | 1798.9722 | 0.0124 | 7 | 657 | 671 | REPWLFGPENTALIR | 43 | 98.55 | | | Mascot |

| | | | | | | | | | | |
|---|---|---|---|---|---|---|---|---|---|---|
| 6 | hypothetical protein LOC100126019 [Danio rerio] | gi\|157787145 | 49134.9 | 8.2 | 6 | 69 | 99.477 | 51 | 99.767 | |

**Peptide Information**

| Calc. Mass | Obsrv. Mass | ± da | ± ppm | Start Seq. | End Seq. | Sequence | Ion Score | C. I. % | Modification | Rank | Result Type |
|---|---|---|---|---|---|---|---|---|---|---|---|
| 883.442 | 883.4474 | 0.0054 | 6 | 47 | 54 | AFFAGSQR | | | | | Mascot |
| 1205.5811 | 1205.6255 | 0.0444 | 37 | 4 | 13 | DAVHGVWEHR | | | | | Mascot |
| 1205.5811 | 1205.6255 | 0.0444 | 37 | 4 | 13 | DAVHGVWEHR | | | | | Mascot |
| 1317.7273 | 1317.7367 | 0.0094 | 7 | 35 | 46 | SGGVERPFVLTR | | | | | Mascot |
| 1317.7273 | 1317.7367 | 0.0094 | 7 | 35 | 46 | SGGVERPFVLTR | 8 | 0 | | | Mascot |
| 1448.7532 | 1448.757 | 0.0038 | 3 | 14 | 25 | DVHNLYGLYVQK | | | | | Mascot |
| 1642.8588 | 1642.8677 | 0.0089 | 5 | 129 | 142 | EPWLFGPENTALIR | | | | | Mascot |
| 1798.9598 | 1798.9722 | 0.0124 | 7 | 128 | 142 | REPWLFGPENTALIR | | | | | Mascot |

| | | | | | | | | | | | |
|---|---|---|---|---|---|---|---|---|---|---|---|
| 1798.9598 | 1798.9722 | 0.0124 | 7 | 128 | 142 | REPWLFGPENTALIR | 43 | 98.55 | | | Mascot |

| 7 | calcium-binding protein 39-like [Danio rerio] | gi\|55925566 | 38782.2 | 6.81 | 10 | 51 | 62.986 |
|---|---|---|---|---|---|---|---|

Peptide Information

| Calc. Mass | Obsrv. Mass | ± da | ± ppm | Start Seq. | End Seq. | Sequence | Ion Score | C. I. % | Modification | Rank | Result Type |
|---|---|---|---|---|---|---|---|---|---|---|---|
| 882.5155 | 882.5717 | 0.0562 | 64 | 186 | 192 | DLLTRHK | | | | | Mascot |
| 1041.6302 | 1041.5912 | -0.039 | -37 | 228 | 236 | LLGELLLDR | | | | | Mascot |
| 1115.667 | 1115.5195 | -0.1475 | -132 | 10 | 19 | SPTEIVKTLK | | | | | Mascot |
| 1165.6068 | 1165.7517 | 0.1449 | 124 | 254 | 262 | LMMNLLRDK | | | Oxidation (M)[2,3] | | Mascot |
| 1252.6466 | 1252.7349 | 0.0883 | 70 | 145 | 154 | ECIRHEPLAK | | | Carbamidomethyl (C)[2] | | Mascot |
| 1359.7379 | 1359.6603 | -0.0776 | -57 | 213 | 223 | LLHSENYVTKR | | | | | Mascot |
| 1451.7233 | 1451.7589 | 0.0356 | 25 | 35 | 47 | ASEEVSKCLVAMK | | | Carbamidomethyl (C)[8] | | Mascot |
| 1563.7954 | 1563.7495 | -0.0459 | -29 | 263 | 275 | SPNIQFEAFHVFK | | | | | Mascot |
| 1563.7954 | 1563.7495 | -0.0459 | -29 | 263 | 275 | SPNIQFEAFHVFK | | | | | Mascot |
| 2028.1058 | 2028.0717 | -0.0341 | -17 | 228 | 244 | LLGELLLDRHNFTVMTR | | | | | Mascot |
| 2044.1008 | 2044.0875 | -0.0133 | -7 | 228 | 244 | LLGELLLDRHNFTVMTR | | | Oxidation (M)[15] | | Mascot |
| 2396.1519 | 2396.2654 | 0.1135 | 47 | 129 | 148 | GYETPQVALNCGIMLRECIR | | | Carbamidomethyl (C)[11,18], Oxidation (M)[14] | | Mascot |

| 8 | Synbl protein [Danio rerio] | gi\|141796272 | 31226.2 | 5.37 | 9 | 44 | 0 |
|---|---|---|---|---|---|---|---|

Peptide Information

| Calc. Mass | Obsrv. Mass | ± da | ± ppm | Start Seq. | End Seq. | Sequence | Ion Score | C. I. % | Modification | Rank | Result Type |
|---|---|---|---|---|---|---|---|---|---|---|---|
| 842.5709 | 842.5159 | -0.055 | -65 | 18 | 24 | VEKLLLK | | | | | Mascot |
| 883.3904 | 883.4474 | 0.057 | 65 | 25 | 31 | YNTENSR | | | | | Mascot |
| 1045.6769 | 1045.5726 | -0.1043 | -100 | 171 | 179 | LLFLLSALR | | | | | Mascot |
| 1058.6503 | 1058.5569 | -0.0934 | -88 | 256 | 264 | LRLIAGIMR | | | Oxidation (M)[8] | | Mascot |
| 1115.6605 | 1115.5195 | -0.141 | -126 | 46 | 55 | LCQGLLTVLR | | | | | Mascot |
| 1563.7384 | 1563.7495 | 0.0111 | 7 | 2 | 16 | SLSDILSEIDGGDDK | | | | | Mascot |
| 1563.7384 | 1563.7495 | 0.0111 | 7 | 2 | 16 | SLSDILSEIDGGDDK | | | | | Mascot |
| 1625.8169 | 1625.8036 | -0.0133 | -8 | 32 | 45 | TFAFDLEEEGARIK | | | | | Mascot |
| 1691.8334 | 1691.8334 | 0 | 0 | 2 | 17 | SLSDILSEIDGGDDKK | | | | | Mascot |
| 2283.0193 | 2283.2014 | 0.1821 | 80 | 98 | 118 | ICVAGHEGEPLEEAQSAEEER | | | | | Mascot |

| 9 | novel protein similar to human and mouse GRIP and coiled-coil domain containing 1 (GCC1) [Danio rer | gi\|56207500 | 91060.6 | 5.23 | 14 | 42 | 0 |
|---|---|---|---|---|---|---|---|

Peptide Information

| Calc. Mass | Obsrv. Mass | ± da | ± ppm | Start Seq. | End Seq. | Sequence | Ion Score | C. I. % | Modification | Rank | Result Type |
|---|---|---|---|---|---|---|---|---|---|---|---|
| 842.4518 | 842.5159 | 0.0641 | 76 | 31 | 36 | YQTRFK | | | | | Mascot |
| 902.524 | 902.5206 | -0.0034 | -4 | 518 | 525 | MRAQGVLK | | | | | Mascot |
| 1022.5152 | 1022.5009 | -0.0143 | -14 | 744 | 752 | DGANLEYLK | | | | | Mascot |
| 1058.5112 | 1058.5569 | 0.0457 | 43 | 83 | 92 | ALPSDLGDDR | | | | | Mascot |
| 1115.5691 | 1115.5195 | -0.0496 | -44 | 215 | 223 | QELEELQAR | | | | | Mascot |
| 1309.6747 | 1309.6219 | -0.0528 | -40 | 705 | 715 | LEEDLHQLQGK | | | | | Mascot |
| 1359.6499 | 1359.6603 | 0.0104 | 8 | 531 | 542 | DGNQAKELEEAR | | | | | Mascot |
| 1434.7145 | 1434.7333 | 0.0188 | 13 | 390 | 402 | VSELSELLGACEK | | | Carbamidomethyl (C)[11] | | Mascot |
| 1434.7145 | 1434.7333 | 0.0188 | 13 | 390 | 402 | VSELSELLGACEK | 4 | 0 | Carbamidomethyl (C)[11] | | Mascot |
| 1477.7314 | 1477.7069 | -0.0245 | -17 | 378 | 389 | MEEIRVASLEER | | | Oxidation (M)[1] | | Mascot |
| 1661.8527 | 1661.7815 | -0.0712 | -43 | 390 | 404 | VSELSELLGACEKAR | | | Carbamidomethyl (C)[11] | | Mascot |
| 1677.8806 | 1677.6991 | -0.1815 | -108 | 552 | 565 | YINLRIQSDEAEVK | | | | | Mascot |
| 1677.8806 | 1677.6991 | -0.1815 | -108 | 552 | 565 | YINLRIQSDEAEVK | | | | | Mascot |
| 1785.8501 | 1785.9579 | 0.1078 | 60 | 481 | 496 | LLEGGEKDHEVSESEK | | | | | Mascot |
| 2027.978 | 2028.0717 | 0.0937 | 46 | 230 | 246 | QHQAELQNLQEQFAESK | | | | | Mascot |
| 2297.1077 | 2297.1531 | 0.0454 | 20 | 293 | 313 | ATLAEAMQAADRGLDYE SQLK | | | Oxidation (M)[7] | | Mascot |

10 novel protein [Danio rerio] gi|157888764 66709.9 9.51 10 41 0 7 0

**Peptide Information**

| Calc. Mass | Obsrv. Mass | ± da | ± ppm | Start Seq. | End Seq. | Sequence | Ion Score | C. I. % | Modification | Rank | Result Type |
|---|---|---|---|---|---|---|---|---|---|---|---|
| 883.3679 | 883.4474 | 0.0795 | 90 | 121 | 127 | FEDESEK | | | | | Mascot |
| 1055.632 | 1055.606 | -0.026 | -25 | 533 | 541 | EGRPKLSLR | 3 | 0 | | | Mascot |
| 1055.632 | 1055.606 | -0.026 | -25 | 533 | 541 | EGRPKLSLR | | | | | Mascot |
| 1146.5974 | 1146.6381 | 0.0407 | 35 | 371 | 381 | SGSKSRPSGQR | | | | | Mascot |
| 1317.6481 | 1317.7367 | 0.0886 | 67 | 237 | 246 | WVTPNQTCRR | | | Carbamidomethyl (C)[8] | | Mascot |
| 1317.6481 | 1317.7367 | 0.0886 | 67 | 237 | 246 | WVTPNQTCRR | | | Carbamidomethyl (C)[8] | | Mascot |
| 1448.6257 | 1448.757 | 0.1313 | 91 | 561 | 573 | SNSMEHLCSPTAR | | | Oxidation (M)[4] | | Mascot |
| 1477.6926 | 1477.7069 | 0.0143 | 10 | 542 | 554 | RFFSAMGLNSMGK | | | Oxidation (M)[6,11] | | Mascot |
| 1625.8242 | 1625.8036 | -0.0206 | -13 | 222 | 236 | STHGITDSQEKAVPR | | | | | Mascot |
| 1661.8389 | 1661.7815 | -0.0574 | -35 | 543 | 557 | FFSAMGLNSMGKLVK | | | Oxidation (M)[5,10] | | Mascot |
| 1798.9293 | 1798.9722 | 0.0429 | 24 | 477 | 493 | DSKSTVPVSSDSHLALR | | | | | Mascot |
| 1798.9293 | 1798.9722 | 0.0429 | 24 | 477 | 493 | DSKSTVPVSSDSHLALR | 7 | 0 | | | Mascot |
| 2225.0359 | 2225.1377 | 0.1018 | 46 | 197 | 216 | DEMLRTCEACGSILAAAQ TK | | | Carbamidomethyl (C)[7,10] | | Mascot |

| | | | | | | | |
|---|---|---|---|---|---|---|---|
| 11 | PREDICTED: zgc:171573 [Danio rerio] | gi\|68441609 | 43885.4 | 8.27 | 10 | 40 | 0 |

Peptide Information

| Calc. Mass | Obsrv. Mass | ± da | ± ppm | Start Seq. | End Seq. | Sequence | Ion Score | C. I. % | Modification | Rank | Result Type |
|---|---|---|---|---|---|---|---|---|---|---|---|
| 902.4689 | 902.5206 | 0.0517 | 57 | 87 | 93 | DERQLNK | | | | | Mascot |
| 1022.5265 | 1022.5009 | -0.0256 | -25 | 27 | 34 | QERIFSDK | | | | | Mascot |
| 1252.5593 | 1252.7349 | 0.1756 | 140 | 358 | 367 | YYSQFNTSSR | | | | | Mascot |
| 1317.7307 | 1317.7367 | 0.006 | 5 | 304 | 315 | MSSARAALLLER | | | | | Mascot |
| 1317.7307 | 1317.7367 | 0.006 | 5 | 304 | 315 | MSSARAALLLER | | | | | Mascot |
| 1332.6682 | 1332.71 | 0.0418 | 31 | 110 | 120 | EFDLNDPELLK | | | | | Mascot |
| 1727.8711 | 1727.8621 | -0.009 | -5 | 344 | 357 | KHLQDVYTNIPDER | | | | | Mascot |
| 1727.8711 | 1727.8621 | -0.009 | -5 | 344 | 357 | KHLQDVYTNIPDER | | | | | Mascot |
| 2027.9087 | 2028.0717 | 0.163 | 80 | 169 | 184 | QEENQYDQIRMSLDSR | | | Oxidation (M)[11] | | Mascot |
| 2211.042 | 2211.1277 | 0.0857 | 39 | 65 | 83 | VYADDLLCSDGIACILEQ R | | | Carbamidomethyl (C)[8,14] | | Mascot |
| 2211.042 | 2211.1277 | 0.0857 | 39 | 65 | 83 | VYADDLLCSDGIACILEQ R | | | Carbamidomethyl (C)[8,14] | | Mascot |
| 2225.2288 | 2225.1377 | -0.0911 | -41 | 198 | 217 | DLLIATKDFNLALAAEITH R | | | | | Mascot |
| 2396.0645 | 2396.2654 | 0.2009 | 84 | 261 | 279 | DCYKGMSPQELQTYTQY QR | | | Carbamidomethyl (C)[2] | | Mascot |

| | | | | | | | |
|---|---|---|---|---|---|---|---|
| 12 | Ift81 protein [Danio rerio] | gi\|145337899 | 29908.3 | 5.76 | 9 | 40 | 0 |

Peptide Information

| Calc. Mass | Obsrv. Mass | ± da | ± ppm | Start Seq. | End Seq. | Sequence | Ion Score | C. I. % | Modification | Rank | Result Type |
|---|---|---|---|---|---|---|---|---|---|---|---|
| 883.4164 | 883.4474 | 0.031 | 35 | 218 | 223 | QMKMWK | | | Oxidation (M)[2,4] | | Mascot |
| 1045.5636 | 1045.5726 | 0.009 | 9 | 14 | 21 | TEEILRER | | | | | Mascot |
| 1115.6055 | 1115.5195 | -0.086 | -77 | 155 | 163 | EIIESQIQR | | | | | Mascot |
| 1448.6976 | 1448.757 | 0.0594 | 41 | 135 | 146 | TLREETAQEESR | | | | | Mascot |
| 1581.7651 | 1581.8031 | 0.038 | 24 | 221 | 232 | MWKDLETLLECK | | | Carbamidomethyl (C)[11], Oxidation (M)[1] | | Mascot |
| 1727.8018 | 1727.8621 | 0.0603 | 35 | 113 | 128 | AQYETCAAGLESNRSK | | | | | Mascot |
| 1727.8018 | 1727.8621 | 0.0603 | 35 | 113 | 128 | AQYETCAAGLESNRSK | | | | | Mascot |
| 1912.9399 | 1912.9994 | 0.0595 | 31 | 96 | 111 | ALRQEHAELAPEYEEK | | | | | Mascot |
| 2225.0879 | 2225.1377 | 0.0498 | 22 | 147 | 163 | YHHINCEREIIESQIQR | | | Carbamidomethyl (C)[6] | | Mascot |
| 2283.1914 | 2283.2014 | 0.01 | 4 | 234 | 254 | QCYLKAQSQAPIGHVIQD VGK | | | | | Mascot |

| | | | | | | | |
|---|---|---|---|---|---|---|---|
| 13 | hypothetical protein LOC492329 [Danio rerio] | gi\|55925514 | 119622.9 | 8.99 | 17 | 39 | 0 |

Peptide Information

| Calc. Mass | Obsrv. Mass | ± da | ± ppm | Start | End | Sequence | Ion | C. I. % | Modification | Rank | Result Type |
|---|---|---|---|---|---|---|---|---|---|---|---|

| | | | | Seq. | Seq. | | Score | | | | |
|---|---|---|---|---|---|---|---|---|---|---|---|
| 842.4366 | 842.5159 | 0.0793 | 94 | 90 | 96 | NTTLEHK | | | | | Mascot |
| 1058.4645 | 1058.5569 | 0.0924 | 87 | 694 | 701 | KEACEMFK | | | Carbamidomethyl (C)[4], Oxidation (M)[6] | | Mascot |
| 1231.7191 | 1231.6849 | -0.0342 | -28 | 179 | 190 | AAMLVKVSSIGR | | | | | Mascot |
| 1434.7006 | 1434.7333 | 0.0327 | 23 | 231 | 244 | CTTTPGGKIAADSR | | | Carbamidomethyl (C)[1] | | Mascot |
| 1434.7522 | 1434.7333 | -0.0189 | -13 | 666 | 677 | KVSIANMLSWNR | | | Oxidation (M)[7] | | Mascot |
| 1451.6445 | 1451.7589 | 0.1144 | 79 | 774 | 784 | FCCYLEGYIQR | | | Carbamidomethyl (C)[2] | | Mascot |
| 1477.7614 | 1477.7069 | -0.0545 | -37 | 682 | 693 | KPMLMTNDRTVR | | | Oxidation (M)[3] | | Mascot |
| 1563.8376 | 1563.7495 | -0.0881 | -56 | 794 | 806 | ITQFILDQQDSKK | | | | | Mascot |
| 1563.8376 | 1563.7495 | -0.0881 | -56 | 793 | 805 | KITQFILDQQDSK | | | | | Mascot |
| 1625.8131 | 1625.8036 | -0.0095 | -6 | 956 | 968 | ELEEPLIPMTFYK | | | Oxidation (M)[9] | | Mascot |
| 1691.8898 | 1691.8334 | -0.0564 | -33 | 667 | 681 | VSIANMLSWNRGSIK | | | Oxidation (M)[6] | | Mascot |
| 1730.9119 | 1730.9097 | -0.0022 | -1 | 327 | 341 | MKLLQHPAQPHTSSR | | | | | Mascot |
| 1860.8909 | 1860.8818 | -0.0091 | -5 | 542 | 558 | TNNNPSMAFTPLDIPGR | | | Oxidation (M)[7] | | Mascot |
| 1913.0248 | 1912.9994 | -0.0254 | -13 | 718 | 734 | HAALLIVTKCWGMQGLR | | | Oxidation (M)[13] | | Mascot |
| 1925.9347 | 1925.9102 | -0.0245 | -13 | 118 | 135 | TPLPPVEPCTKEPECASK | | | | | Mascot |
| 2086.9612 | 2087.0176 | 0.0564 | 27 | 18 | 35 | EYMYVNLTTGICGWDPPK | | | | | Mascot |
| 2225.1133 | 2225.1377 | 0.0244 | 11 | 753 | 773 | SLAAGWELMAVCLAFFAPSPK | | | Oxidation (M)[9] | | Mascot |

14 hypothetical protein LOC100126120 [Danio rerio] gi|157954484 52911.3 7.59 12 39 0

**Peptide Information**

| Calc. Mass | Obsrv. Mass | ± da | ± ppm | Start Seq. | End Seq. | Sequence | Ion Score | C. I. % | Modification | Rank | Result Type |
|---|---|---|---|---|---|---|---|---|---|---|---|
| 1022.5126 | 1022.5009 | -0.0117 | -11 | 407 | 414 | NHERTHTK | | | | | Mascot |
| 1045.6516 | 1045.5726 | -0.079 | -76 | 399 | 406 | FRQLQILK | | | | | Mascot |
| 1058.4281 | 1058.5569 | 0.1288 | 122 | 244 | 252 | EFVCSECGK | | | Carbamidomethyl (C)[4] | | Mascot |
| 1115.4496 | 1115.5195 | 0.0699 | 63 | 244 | 252 | EFVCSECGK | | | Carbamidomethyl (C)[4,7] | | Mascot |
| 1252.7412 | 1252.7349 | -0.0063 | -5 | 141 | 150 | TFIKPSYLKR | | | | | Mascot |
| 1332.653 | 1332.71 | 0.057 | 43 | 11 | 21 | QEETEELIDVK | | | | | Mascot |
| 1451.6021 | 1451.7589 | 0.1568 | 108 | 22 | 33 | QESEELSEDEEK | | | | | Mascot |
| 1625.8217 | 1625.8036 | -0.0181 | -11 | 318 | 330 | LHQMIHTGERPYK | | | Oxidation (M)[4] | | Mascot |
| 1677.736 | 1677.6991 | -0.0369 | -22 | 244 | 258 | EFVCSECGKSFNTAR | | | | | Mascot |
| 1677.736 | 1677.6991 | -0.0369 | -22 | 244 | 258 | EFVCSECGKSFNTAR | | | | | Mascot |
| 1798.8615 | 1798.9722 | 0.1107 | 62 | 275 | 289 | CSHCDKSFIYLQNLK | | | | | Mascot |
| 1798.8615 | 1798.9722 | 0.1107 | 62 | 275 | 289 | CSHCDKSFIYLQNLK | | | | | Mascot |
| 1912.9044 | 1912.9994 | 0.095 | 50 | 275 | 289 | CSHCDKSFIYLQNLK | | | Carbamidomethyl (C)[1,4] | | Mascot |

| Calc. Mass | Obsrv. Mass | ± da | ± ppm | Start Seq. | End Seq. | Sequence | Ion Score | C. I. % | Modification | Rank | Result Type |
|---|---|---|---|---|---|---|---|---|---|---|---|
| 2086.9895 | 2087.0176 | 0.0281 | 13 | 122 | 140 | VYQSVQAEVKSNSCSLCGK | | | Carbamidomethyl (C)[14] | | Mascot |
| 2210.978 | 2211.1277 | 0.1497 | 68 | 435 | 452 | SLHLQWHMMIHAGEETQM | | | Oxidation (M)[8,9] | | Mascot |
| 2210.978 | 2211.1277 | 0.1497 | 68 | 435 | 452 | SLHLQWHMMIHAGEETQM | | | Oxidation (M)[8,9] | | Mascot |
| 2283.0579 | 2283.2014 | 0.1435 | 63 | 318 | 336 | LHQMIHTGERPYKCSHCDK | | | | | Mascot |

15 tektin 2 (testicular) [Danio rerio] gi|94733681 48602.7 5.71 9 38 0

**Peptide Information**

| Calc. Mass | Obsrv. Mass | ± da | ± ppm | Start Seq. | End Seq. | Sequence | Ion Score | C. I. % | Modification | Rank | Result Type |
|---|---|---|---|---|---|---|---|---|---|---|---|
| 1045.5789 | 1045.5726 | -0.0063 | -6 | 68 | 75 | INDITRWK | | | | | Mascot |
| 1115.5691 | 1115.5195 | -0.0496 | -44 | 140 | 149 | EVEVIDGAQR | | | | | Mascot |
| 1798.9657 | 1798.9722 | 0.0065 | 4 | 165 | 179 | LQEARQQLTIDLQDK | | | | | Mascot |
| 1798.9657 | 1798.9722 | 0.0065 | 4 | 165 | 179 | LQEARQQLTIDLQDK | | | | | Mascot |
| 1909.8518 | 1909.9108 | 0.059 | 31 | 80 | 96 | ACAQEVDEEMDALTLSK | | | Carbamidomethyl (C)[2] | | Mascot |
| 1925.8467 | 1925.9102 | 0.0635 | 33 | 80 | 96 | ACAQEVDEEMDALTLSK | | | Carbamidomethyl (C)[2], Oxidation (M)[10] | | Mascot |
| 2070.9199 | 2071.0205 | 0.1006 | 49 | 269 | 284 | THHEEQAHHDMQWQLK | | | Oxidation (M)[11] | | Mascot |
| 2211.0261 | 2211.1277 | 0.1016 | 46 | 268 | 284 | RTHHEEQAHHDMQWQLK | | | | | Mascot |
| 2211.0261 | 2211.1277 | 0.1016 | 46 | 268 | 284 | RTHHEEQAHHDMQWQLK | | | | | Mascot |
| 2283.1365 | 2283.2014 | 0.0649 | 28 | 2 | 22 | ATLSSKPASRYSVSDWATNNK | | | | | Mascot |
| 2337.0947 | 2337.2498 | 0.1551 | 66 | 76 | 96 | ENLKACAQEVDEEMDALTLSK | | | | | Mascot |
| 2396.2966 | 2396.2654 | -0.0312 | -13 | 102 | 123 | ALAATVLPLEVTAECLNLREGR | | | Carbamidomethyl (C)[15] | | Mascot |

16 ATR-interacting protein [Danio rerio] gi|113678194 81922.4 5.71 13 38 0

**Peptide Information**

| Calc. Mass | Obsrv. Mass | ± da | ± ppm | Start Seq. | End Seq. | Sequence | Ion Score | C. I. % | Modification | Rank | Result Type |
|---|---|---|---|---|---|---|---|---|---|---|---|
| 973.5424 | 973.5176 | -0.0248 | -25 | 206 | 214 | LQSVERGGK | | | | | Mascot |
| 1024.4945 | 1024.5231 | 0.0286 | 28 | 236 | 244 | ENFSAEISK | | | | | Mascot |
| 1309.6356 | 1309.6219 | -0.0137 | -10 | 151 | 160 | QAHQEREQQR | | | | | Mascot |
| 1448.7065 | 1448.757 | 0.0505 | 35 | 563 | 574 | LCLHDYPCPFLK | | | | | Mascot |
| 1477.9001 | 1477.7069 | -0.1932 | -131 | 631 | 642 | TVVVVLHRQWLK | | | | | Mascot |
| 1642.8403 | 1642.8677 | 0.0274 | 17 | 465 | 478 | NMLGEIQNQMPVLR | | | | | Mascot |
| 1665.79 | 1665.9108 | 0.1208 | 73 | 54 | 68 | RSFDAVLACADEPSK | | | Carbamidomethyl (C)[9] | | Mascot |
| 1798.9414 | 1798.9722 | 0.0308 | 17 | 465 | 479 | NMLGEIQNQMPVLRR | | | | | Mascot |
| 1799.0021 | 1798.9722 | -0.0299 | -17 | 505 | 520 | ILSVLAERAEDNLISR | | | | | Mascot |

| | | | | | | | | | | | |
|---|---|---|---|---|---|---|---|---|---|---|---|
| 1860.9813 | 1860.8818 | -0.0995 | -53 | 586 | 601 | SSTEDAWSRLELVVIR | | | | | Mascot |
| 2087.0955 | 2087.0176 | -0.0779 | -37 | 442 | 459 | ALQHIVCYSKEALEDVIR | | | | | Mascot |
| 2297.0107 | 2297.1531 | 0.1424 | 62 | 611 | 630 | AGTWAALCESSCQCVNE VVR | | | Carbamidomethyl (C)[8,12,14] | | Mascot |
| 3249.7043 | 3249.4709 | -0.2334 | -72 | 521 | 548 | LQVVMSSPVLSKCLTLET TYSIVHLCVR | | | Carbamidomethyl (C)[13,26], Oxidation (M)[5] | | Mascot |
| 3249.7043 | 3249.4709 | -0.2334 | -72 | 521 | 548 | LQVVMSSPVLSKCLTLET TYSIVHLCVR | | | Carbamidomethyl (C)[13,26], Oxidation (M)[5] | | Mascot |

17 PREDICTED: coronin-2B [Danio rerio] gi|292627941 53560.2 7.91 10 37 0

Peptide Information

| Calc. Mass | Obsrv. Mass | ± da | ± ppm | Start Seq. | End Seq. | Sequence | Ion Score | C. I. % | Modification | Rank | Result Type |
|---|---|---|---|---|---|---|---|---|---|---|---|
| 882.5519 | 882.5717 | 0.0198 | 22 | 199 | 205 | LRIIDPR | | | | | Mascot |
| 883.442 | 883.4474 | 0.0054 | 6 | 71 | 77 | VDPHYPR | | | | | Mascot |
| 1022.5741 | 1022.5009 | -0.0732 | -72 | 231 | 239 | RVFTTGVSR | | | | | Mascot |
| 1252.7233 | 1252.7349 | 0.0116 | 9 | 398 | 408 | RPNPVSLRASR | | | | | Mascot |
| 1317.7776 | 1317.7367 | -0.0409 | -31 | 386 | 397 | GPVLISLKEAYK | | | | | Mascot |
| 1317.7776 | 1317.7367 | -0.0409 | -31 | 386 | 397 | GPVLISLKEAYK | | | | | Mascot |
| 1359.7123 | 1359.6603 | -0.052 | -38 | 310 | 322 | SAAPQKSLCVMPK | | | | | Mascot |
| 1691.8097 | 1691.8334 | 0.0237 | 14 | 324 | 337 | GVDVSECEIFRFYK | | | | | Mascot |
| 1913.1041 | 1912.9994 | -0.1047 | -55 | 338 | 354 | LVTFRGLVEPVSMIVPR | | | | | Mascot |
| 2211.0435 | 2211.1277 | 0.0842 | 38 | 71 | 89 | VDPHYPRVCGHQGSVM DIK | | | Carbamidomethyl (C)[9], Oxidation (M)[16] | | Mascot |
| 2211.0435 | 2211.1277 | 0.0842 | 38 | 71 | 89 | VDPHYPRVCGHQGSVM DIK | | | Carbamidomethyl (C)[9], Oxidation (M)[16] | | Mascot |
| 2396.1414 | 2396.2654 | 0.124 | 52 | 135 | 156 | VGLIEWHPTCSGILFSAG YDCK | | | | | Mascot |

18 hypothetical protein [Danio rerio] gi|295821973 4314 4.67 4 37 0

Peptide Information

| Calc. Mass | Obsrv. Mass | ± da | ± ppm | Start Seq. | End Seq. | Sequence | Ion Score | C. I. % | Modification | Rank | Result Type |
|---|---|---|---|---|---|---|---|---|---|---|---|
| 973.4149 | 973.5176 | 0.1027 | 106 | 32 | 39 | EGFVEYTE | | | | | Mascot |
| 1317.6 | 1317.7367 | 0.1367 | 104 | 17 | 28 | ATACQMLVCYAK | | | Oxidation (M)[6] | | Mascot |
| 1317.6 | 1317.7367 | 0.1367 | 104 | 17 | 28 | ATACQMLVCYAK | | | Oxidation (M)[6] | | Mascot |
| 1785.8696 | 1785.9579 | 0.0883 | 49 | 17 | 31 | ATACQMLVCYAKELK | | | Carbamidomethyl (C)[4,9] | | Mascot |
| 2086.9971 | 2087.0176 | 0.0205 | 10 | 10 | 28 | TAGLEEKATACQMLVCY AK | | | Carbamidomethyl (C)[11] | | Mascot |

19 PREDICTED: MAM domain-containing glycosylphosphatidylinositol anchor protein 1-like, partial [Danio gi|326673130 86011.6 8.77 12 36 0

Peptide Information

| Calc. Mass | Obsrv. Mass | ± da | ± ppm | Start Seq. | End Seq. | Sequence | Ion Score | C. I. % | Modification | Rank | Result Type |
|---|---|---|---|---|---|---|---|---|---|---|---|
| 973.4777 | 973.5176 | 0.0399 | 41 | 347 | 353 | VNYTWYK | | | | | Mascot |
| 996.4567 | 996.6018 | 0.1451 | 146 | 731 | 738 | ICGFTQDR | | | Carbamidomethyl (C)[2] | | Mascot |
| 1022.4571 | 1022.5009 | 0.0438 | 43 | 323 | 331 | DSESIQMGR | | | | | Mascot |
| 1058.5048 | 1058.5569 | 0.0521 | 49 | 137 | 146 | CTVNSNPPAR | | | | | Mascot |
| 1115.5262 | 1115.5195 | -0.0067 | -6 | 137 | 146 | CTVNSNPPAR | | | Carbamidomethyl (C)[1] | | Mascot |
| 1317.7083 | 1317.7367 | 0.0284 | 22 | 32 | 43 | EGDTLVLQCLVK | | | | | Mascot |
| 1317.7083 | 1317.7367 | 0.0284 | 22 | 32 | 43 | EGDTLVLQCLVK | | | | | Mascot |
| 1451.6947 | 1451.7589 | 0.0642 | 44 | 200 | 212 | NVCGIPDSSVTFR | | | Carbamidomethyl (C)[3] | | Mascot |
| 1477.7579 | 1477.7069 | -0.051 | -35 | 493 | 505 | CQTAPYNGLNIKR | | | | | Mascot |
| 1625.8182 | 1625.8036 | -0.0146 | -9 | 544 | 556 | SNPNRIVSAYWYR | | | | | Mascot |
| 1642.8257 | 1642.8677 | 0.042 | 26 | 86 | 100 | YYCKAENGVGVAAIK | | | Carbamidomethyl (C)[3] | | Mascot |
| 1661.7588 | 1661.7815 | 0.0227 | 14 | 576 | 589 | FKLDTTNNGTYECR | | | | | Mascot |
| 1665.7247 | 1665.9108 | 0.1861 | 112 | 461 | 474 | DLPMPSGDWMVETR | | | Oxidation (M)[4,10] | | Mascot |
| 1730.8972 | 1730.9097 | 0.0125 | 7 | 147 | 160 | FIWQRANNPIEQSK | | | | | Mascot |

| | | | | | | | | | | |
|---|---|---|---|---|---|---|---|---|---|---|
| 20 | PREDICTED: hypothetical protein LOC100149379 [Danio rerio] | gi\|326679369 | 173931.4 | 5.87 | 16 | 34 | 0 | 17 | 0 |

Peptide Information

| Calc. Mass | Obsrv. Mass | ± da | ± ppm | Start Seq. | End Seq. | Sequence | Ion Score | C. I. % | Modification | Rank | Result Type |
|---|---|---|---|---|---|---|---|---|---|---|---|
| 902.4941 | 902.5206 | 0.0265 | 29 | 678 | 685 | EKADLQAK | | | | | Mascot |
| 1045.5347 | 1045.5726 | 0.0379 | 36 | 1206 | 1214 | ETILPCQGK | | | Carbamidomethyl (C)[6] | | Mascot |
| 1058.6569 | 1058.5569 | -0.1 | -94 | 627 | 635 | TQVTKLLQK | | | | | Mascot |
| 1205.5327 | 1205.6255 | 0.0928 | 77 | 1141 | 1150 | NQCDASNQIR | 17 | 0 | Carbamidomethyl (C)[3] | | Mascot |
| 1205.5327 | 1205.6255 | 0.0928 | 77 | 1141 | 1150 | NQCDASNQIR | | | Carbamidomethyl (C)[3] | | Mascot |
| 1252.6685 | 1252.7349 | 0.0664 | 53 | 1359 | 1368 | IKEGLQFDFR | | | | | Mascot |
| 1262.5681 | 1262.684 | 0.1159 | 92 | 407 | 416 | LEQMVEENDR | | | | | Mascot |
| 1317.6719 | 1317.7367 | 0.0648 | 49 | 1315 | 1325 | MPDKLLQSEEK | | | | | Mascot |
| 1317.6719 | 1317.7367 | 0.0648 | 49 | 1315 | 1325 | MPDKLLQSEEK | | | | | Mascot |
| 1332.7378 | 1332.71 | -0.0278 | -21 | 632 | 642 | LLQKINDMVMK | | | | | Mascot |
| 1434.6642 | 1434.7333 | 0.0691 | 48 | 407 | 417 | LEQMVEENDRR | | | Oxidation (M)[4] | | Mascot |
| 1434.6642 | 1434.7333 | 0.0691 | 48 | 407 | 417 | LEQMVEENDRR | | | Oxidation (M)[4] | | Mascot |
| 1451.7238 | 1451.7589 | 0.0351 | 24 | 830 | 842 | HETGQNPKLEGNK | | | | | Mascot |
| 1563.6454 | 1563.7495 | 0.1041 | 67 | 1346 | 1358 | ECGQIYESWSSMK | | | Oxidation (M)[12] | | Mascot |

| | | | | | | | | | | | |
|---|---|---|---|---|---|---|---|---|---|---|---|
| 1563.6454 | 1563.7495 | 0.1041 | 67 | 1346 | 1358 | ECGQIYESWSSMK | | | Oxidation (M)[12] | | Mascot |
| 1661.7186 | 1661.7815 | 0.0629 | 38 | 1282 | 1294 | WIMNMLDDFTSEK | | | Oxidation (M)[3,5] | | Mascot |
| 1912.995 | 1912.9994 | 0.0044 | 2 | 391 | 406 | HHYLELNTQVSMLVTK | | | | | Mascot |
| 2028.072 | 2028.0717 | -0.0003 | 0 | 1319 | 1335 | LLQSEEKLNNISEQINR | | | | | Mascot |
| 2071.0164 | 2071.0205 | 0.0041 | 2 | 1464 | 1481 | AQMNSIDYFLVVDTEGLR | | | | | Mascot |
| 2087.0112 | 2087.0176 | 0.0064 | 3 | 1464 | 1481 | AQMNSIDYFLVVDTEGLR | | | Oxidation (M)[3] | | Mascot |
| 2297.3149 | 2297.1531 | -0.1618 | -70 | 294 | 313 | LITLVELPALTLLSQKEEMR | | | | | Mascot |

| | | | | | | | | |
|---|---|---|---|---|---|---|---|---|
| 21 | novel protein similar to vertebrate protein kinase C, theta (PRKCQ) [Danio rerio] | gi\|55962227 | 81064.5 | 7.59 | 14 | 34 | 0 | |

**Peptide Information**

| Calc. Mass | Obsrv. Mass | ± da | ± ppm | Start Seq. | End Seq. | Sequence | Ion Score | C. I. % | Modification | Rank | Result Type |
|---|---|---|---|---|---|---|---|---|---|---|---|
| 850.4781 | 850.546 | 0.0679 | 80 | 31 | 37 | GQVYKQK | | | | | Mascot |
| 940.492 | 940.5209 | 0.0289 | 31 | 386 | 394 | MLGKGSFGK | | | Oxidation (M)[1] | | Mascot |
| 1024.6084 | 1024.5231 | -0.0853 | -83 | 56 | 64 | GRVMHVVVK | | | | | Mascot |
| 1045.5789 | 1045.5726 | -0.0063 | -6 | 614 | 621 | LFVREPER | | | | | Mascot |
| 1252.7048 | 1252.7349 | 0.0301 | 24 | 402 | 413 | GSGQFFAVKALK | | | | | Mascot |
| 1359.5238 | 1359.6603 | 0.1365 | 100 | 257 | 267 | CEECGMNVHHK | | | Carbamidomethyl (C)[1], Oxidation (M)[6] | | Mascot |
| 1380.7004 | 1380.6865 | -0.0139 | -10 | 24 | 35 | EAVETEKGQVYK | | | | | Mascot |
| 1661.6652 | 1661.7815 | 0.1163 | 70 | 257 | 270 | CEECGMNVHHKCQK | | | Oxidation (M)[6] | | Mascot |
| 1665.9032 | 1665.9108 | 0.0076 | 5 | 376 | 389 | FTIDSFILHKMLGK | | | Oxidation (M)[11] | | Mascot |
| 1727.8322 | 1727.8621 | 0.0299 | 17 | 173 | 186 | EFVWGLNKQGYQCR | | | | | Mascot |
| 1727.8322 | 1727.8621 | 0.0299 | 17 | 173 | 186 | EFVWGLNKQGYQCR | | | | | Mascot |
| 1785.7828 | 1785.9579 | 0.1751 | 98 | 253 | 267 | QGLKCEECGMNVHHK | | | Carbamidomethyl (C)[5], Oxidation (M)[10] | | Mascot |
| 1925.829 | 1925.9102 | 0.0812 | 42 | 415 | 430 | DVVLMDDDVECTMVER | | | Carbamidomethyl (C)[11] | | Mascot |
| 2028.0259 | 2028.0717 | 0.0458 | 23 | 482 | 499 | STFYAAEIICGLQFLHSK | | | | | Mascot |
| 2284.0989 | 2284.197 | 0.0981 | 43 | 537 | 556 | TCTFCGTPDYIAPEILLGQK | | | Carbamidomethyl (C)[2,5] | | Mascot |

| | | | | | | | | |
|---|---|---|---|---|---|---|---|---|
| 22 | PREDICTED: catenin alpha-1-like [Danio rerio] | gi\|326679802 | 78904.3 | 5.52 | 10 | 34 | 0 | |

**Peptide Information**

| Calc. Mass | Obsrv. Mass | ± da | ± ppm | Start Seq. | End Seq. | Sequence | Ion Score | C. I. % | Modification | Rank | Result Type |
|---|---|---|---|---|---|---|---|---|---|---|---|
| 1205.6637 | 1205.6255 | -0.0382 | -32 | 617 | 626 | LVYDGVRDIR | 12 | 0 | | | Mascot |
| 1205.6637 | 1205.6255 | -0.0382 | -32 | 617 | 626 | LVYDGVRDIR | | | | | Mascot |
| 1231.6165 | 1231.6849 | 0.0684 | 56 | 88 | 98 | EELTAAVEDVR | | | | | Mascot |
| 1332.6754 | 1332.71 | 0.0346 | 26 | 359 | 370 | EKSDALNTAIDR | | | | | Mascot |

| Calc. Mass | Obsrv. Mass | ± da | ± ppm | Start Seq. | End Seq. | Sequence | Ion Score | C. I. % | Modification | Rank | Result Type |
|---|---|---|---|---|---|---|---|---|---|---|---|
| 1359.7114 | 1359.6603 | -0.0511 | -38 | 88 | 99 | EELTAAVEDVRK | | | | | Mascot |
| 1451.7158 | 1451.7589 | 0.0431 | 30 | 361 | 373 | SDALNTAIDRMTK | | | Oxidation (M)[11] | | Mascot |
| 1691.8711 | 1691.8334 | -0.0377 | -22 | 164 | 178 | VRNAGTEQDLGIQYK | | | | | Mascot |
| 1730.8741 | 1730.9097 | 0.0356 | 21 | 526 | 540 | CVIALQEKDVDGLDR | | | Carbamidomethyl (C)[1] | | Mascot |
| 1909.9436 | 1909.9108 | -0.0328 | -17 | 342 | 358 | QALQDLLSEYMGNAGRK | | | Oxidation (M)[11] | | Mascot |
| 2071.0818 | 2071.0205 | -0.0613 | -30 | 57 | 75 | KAHVLAASVDEATQNFLEK | | | | | Mascot |
| 2211.0381 | 2211.1277 | 0.0896 | 41 | 309 | 329 | LESIISGAALMADSSCTRDDR | | | | | Mascot |
| 2211.0381 | 2211.1277 | 0.0896 | 41 | 309 | 329 | LESIISGAALMADSSCTRDDR | | | | | Mascot |
| 2284.0544 | 2284.197 | 0.1426 | 62 | 309 | 329 | LESIISGAALMADSSCTRDDR | | | Carbamidomethyl (C)[16], Oxidation (M)[11] | | Mascot |

23 lactosylceramide alpha-2,3-sialyltransferase [Danio rerio] gi|117606180 41829.7 9.18 10 33 0

**Peptide Information**

| Calc. Mass | Obsrv. Mass | ± da | ± ppm | Start Seq. | End Seq. | Sequence | Ion Score | C. I. % | Modification | Rank | Result Type |
|---|---|---|---|---|---|---|---|---|---|---|---|
| 973.5101 | 973.5176 | 0.0075 | 8 | 226 | 233 | TPVSLWDR | | | | | Mascot |
| 1022.4836 | 1022.5009 | 0.0173 | 17 | 66 | 73 | ECRPSFAR | | | Carbamidomethyl (C)[2] | | Mascot |
| 1041.438 | 1041.5912 | 0.1532 | 147 | 187 | 195 | MSYPESCPK | | | | | Mascot |
| 1058.6027 | 1058.5569 | -0.0458 | -43 | 140 | 150 | CVVVGNGGILK | | | | | Mascot |
| 1115.624 | 1115.5195 | -0.1045 | -94 | 140 | 150 | CVVVGNGGILK | | | Carbamidomethyl (C)[1] | | Mascot |
| 1332.7383 | 1332.71 | -0.0283 | -21 | 55 | 65 | VHSHVREILDK | | | | | Mascot |
| 1380.6287 | 1380.6865 | 0.0578 | 42 | 4 | 14 | VMKQSSCYFSK | | | Carbamidomethyl (C)[7], Oxidation (M)[2] | | Mascot |
| 1563.7948 | 1563.7495 | -0.0453 | -29 | 61 | 73 | EILDKECRPSFAR | | | | | Mascot |
| 1563.7948 | 1563.7495 | -0.0453 | -29 | 61 | 73 | EILDKECRPSFAR | | | | | Mascot |
| 1691.8978 | 1691.8334 | -0.0644 | -38 | 234 | 247 | LFFWQNVPMSVPVK | | | | | Mascot |
| 1910.1105 | 1909.9108 | -0.1997 | -105 | 16 | 32 | TMILLLSLALMSLAFLK | | | Oxidation (M)[2,11] | | Mascot |
| 2297.01 | 2297.1531 | 0.1431 | 62 | 187 | 205 | MSYPESCPKVWEDTDPDLK | | | Carbamidomethyl (C)[7] | | Mascot |

24 PREDICTED: ephrin type-A receptor 7-like [Danio rerio] gi|292629941 40322.6 8.74 8 33 0

**Peptide Information**

| Calc. Mass | Obsrv. Mass | ± da | ± ppm | Start Seq. | End Seq. | Sequence | Ion Score | C. I. % | Modification | Rank | Result Type |
|---|---|---|---|---|---|---|---|---|---|---|---|
| 902.5128 | 902.5206 | 0.0078 | 9 | 13 | 20 | GCLKLPSK | | | Carbamidomethyl (C)[2] | | Mascot |
| 1055.5415 | 1055.606 | 0.0645 | 61 | 350 | 358 | AQVIQMHGR | | | Oxidation (M)[6] | | Mascot |
| 1055.5415 | 1055.606 | 0.0645 | 61 | 350 | 358 | AQVIQMHGR | | | Oxidation (M)[6] | | Mascot |
| 1359.6975 | 1359.6603 | -0.0372 | -27 | 258 | 270 | SPDNIGSSTLGRR | | | | | Mascot |

| | | | | | | | | | | | |
|---|---|---|---|---|---|---|---|---|---|---|---|
| 1581.9144 | 1581.8031 | -0.1113 | -70 | 122 | 136 | LAAHKVLVNSSLVCK | | | | | Mascot |
| 2070.959 | 2071.0205 | 0.0615 | 30 | 304 | 321 | YKDNFTAAGYCYLESVA R | | | | | Mascot |
| 2283.2012 | 2283.2014 | 0.0002 | 0 | 89 | 110 | HEGQLTVLQLADLLSGVA SGMK | | | Oxidation (M)[21] | | Mascot |
| 2284.1133 | 2284.197 | 0.0837 | 37 | 67 | 87 | GNTMMIVVESMVNGALD SFLR | | | | | Mascot |
| 3249.6006 | 3249.4709 | -0.1297 | -40 | 210 | 237 | AIEDGFRLPAPLNCPPSL HQLMLDCWQK | | | Carbamidomethyl (C)[14] | | Mascot |
| 3249.6006 | 3249.4709 | -0.1297 | -40 | 210 | 237 | AIEDGFRLPAPLNCPPSL HQLMLDCWQK | | | Carbamidomethyl (C)[14] | | Mascot |

| | | | | | | | |
|---|---|---|---|---|---|---|---|
| 25 | PREDICTED: zinc finger protein 484-like [Danio rerio] | gi\|326680627 | 46462.1 | 9.69 | 11 | 33 | 0 |

**Peptide Information**

| Calc. Mass | Obsrv. Mass | ± da | ± ppm | Start Seq. | End Seq. | Sequence | Ion Score | C. I. % | Modification | Rank | Result Type |
|---|---|---|---|---|---|---|---|---|---|---|---|
| 1022.4472 | 1022.5009 | 0.0537 | 53 | 209 | 216 | STFNNHMR | | | Oxidation (M)[7] | | Mascot |
| 1058.4142 | 1058.5569 | 0.1427 | 135 | 2 | 10 | CNFSSNQCR | | | | | Mascot |
| 1115.4972 | 1115.5195 | 0.0223 | 20 | 298 | 306 | HMMIHTGEK | | | Oxidation (M)[2,3] | | Mascot |
| 1155.5792 | 1155.6981 | 0.1189 | 103 | 316 | 324 | SFERNFDLK | | | | | Mascot |
| 1205.4496 | 1205.6255 | 0.1759 | 146 | 1 | 10 | MCNFSSNQCR | | | Oxidation (M)[1] | | Mascot |
| 1205.4496 | 1205.6255 | 0.1759 | 146 | 1 | 10 | MCNFSSNQCR | | | Oxidation (M)[1] | | Mascot |
| 1317.5497 | 1317.7367 | 0.187 | 142 | 1 | 11 | MCNFSSNQCRK | | | | | Mascot |
| 1317.5497 | 1317.7367 | 0.187 | 142 | 1 | 11 | MCNFSSNQCRK | | | | | Mascot |
| 1661.8138 | 1661.7815 | -0.0323 | -19 | 273 | 287 | IHTGEKPFICTQCGK | | | | | Mascot |
| 1691.7628 | 1691.8334 | 0.0706 | 42 | 217 | 231 | THTGEKPFACAQCGK | | | Carbamidomethyl (C)[10,13] | | Mascot |
| 2070.9331 | 2071.0205 | 0.0874 | 42 | 375 | 393 | SSHLNEHLQCGMSSSVP NK | | | Oxidation (M)[12] | | Mascot |
| 2210.9814 | 2211.1277 | 0.1463 | 66 | 298 | 315 | HMMIHTGEKQFTCTLCG K | | | Carbamidomethyl (C)[13,16], Oxidation (M)[2,3] | | Mascot |
| 2210.9814 | 2211.1277 | 0.1463 | 66 | 298 | 315 | HMMIHTGEKQFTCTLCG K | | | Carbamidomethyl (C)[13,16], Oxidation (M)[2,3] | | Mascot |
| 2283.0063 | 2283.2014 | 0.1951 | 85 | 58 | 76 | CQQCGKSFTQNSNLEVH MR | | | Carbamidomethyl (C)[1], Oxidation (M)[18] | | Mascot |

| | | | | | | | |
|---|---|---|---|---|---|---|---|
| 26 | novel protein similar to bloodthirsty (bty) [Danio rerio] | gi\|148725963 | 63618.3 | 5.75 | 11 | 33 | 0 |

**Peptide Information**

| Calc. Mass | Obsrv. Mass | ± da | ± ppm | Start Seq. | End Seq. | Sequence | Ion Score | C. I. % | Modification | Rank | Result Type |
|---|---|---|---|---|---|---|---|---|---|---|---|
| 1146.6517 | 1146.6381 | -0.0136 | -12 | 362 | 370 | ITQTELKWK | | | | | Mascot |
| 1661.6604 | 1661.7815 | 0.1211 | 73 | 179 | 191 | DDQTCVCSFCIEK | | | Carbamidomethyl (C)[5,7,10] | | Mascot |
| 1677.7617 | 1677.6991 | -0.0626 | -37 | 522 | 535 | LYPYFSPCPNYEGK | | | | | Mascot |
| 1677.8782 | 1677.6991 | -0.1791 | -107 | 457 | 471 | ITLTPGNGFCTVWLR | | | | | Mascot |
| 1909.9147 | 1909.9108 | -0.0039 | -2 | 154 | 168 | LMDPVRNLEDYICQK | | | Carbamidomethyl (C)[13], Oxidation (M)[2] | | Mascot |

| | | | | | | | | | | | |
|---|---|---|---|---|---|---|---|---|---|---|---|
| 2027.8732 | 2028.0717 | 0.1985 | 98 | 127 | 142 | SCLVCQSSYCQTHLER | | | Carbamidomethyl (C)[2,5,10] | | Mascot |
| 2071.0818 | 2071.0205 | -0.0613 | -30 | 332 | 348 | NWPEISLKTQENLETLR | | | | | Mascot |
| 2225.0247 | 2225.1377 | 0.113 | 51 | 261 | 278 | AIERCQTELLEMMEEQQK | | | Oxidation (M)[12] | | Mascot |
| 2283.0679 | 2283.2014 | 0.1335 | 58 | 124 | 142 | ALKSCLVCQSSYCQTHLER | | | Carbamidomethyl (C)[5,8] | | Mascot |
| 2283.9038 | 2284.197 | 0.2932 | 128 | 66 | 83 | CWNNSQTCSCPYCKETFR | | | Carbamidomethyl (C)[1,8] | | Mascot |
| 3249.377 | 3249.4709 | 0.0939 | 29 | 179 | 206 | DDQTCVCSFCIEKDHENHNTVPLEEESK | | | | | Mascot |
| 3249.377 | 3249.4709 | 0.0939 | 29 | 179 | 206 | DDQTCVCSFCIEKDHENHNTVPLEEESK | | | | | Mascot |

| | | | | | | | | |
|---|---|---|---|---|---|---|---|---|
| 27 | PREDICTED: hypothetical protein LOC100148510 [Danio rerio] | gi\|326667766 | 309668.8 | 6.59 | 20 | 33 | 0 | |

**Peptide Information**

| Calc. Mass | Obsrv. Mass | ± da | ± ppm | Start Seq. | End Seq. | Sequence | Ion Score | C. I. % | Modification | Rank | Result Type |
|---|---|---|---|---|---|---|---|---|---|---|---|
| 842.5093 | 842.5159 | 0.0066 | 8 | 135 | 142 | VVIGIADR | | | | | Mascot |
| 850.4417 | 850.546 | 0.1043 | 123 | 338 | 344 | FLANTER | | | | | Mascot |
| 883.4784 | 883.4474 | -0.031 | -35 | 1527 | 1533 | LHSHYVK | | | | | Mascot |
| 902.4941 | 902.5206 | 0.0265 | 29 | 427 | 433 | VEKELER | | | | | Mascot |
| 996.6564 | 996.6018 | -0.0546 | -55 | 1597 | 1605 | LGSLLPKIR | | | | | Mascot |
| 1115.6167 | 1115.5195 | -0.0972 | -87 | 321 | 331 | TSINVRSPGGK | | | | | Mascot |
| 1146.5823 | 1146.6381 | 0.0558 | 49 | 967 | 977 | IAADLCELNGK | | | | | Mascot |
| 1155.6554 | 1155.6981 | 0.0427 | 37 | 549 | 558 | EMSKLLIGHK | | | | | Mascot |
| 1165.6285 | 1165.7517 | 0.1232 | 106 | 2054 | 2062 | QLEMFKELK | | | | | Mascot |
| 1205.6484 | 1205.6255 | -0.0229 | -19 | 2671 | 2681 | TVSDSSKIVNR | 14 | 0 | | | Mascot |
| 1205.6484 | 1205.6255 | -0.0229 | -19 | 2671 | 2681 | TVSDSSKIVNR | | | | | Mascot |
| 1252.566 | 1252.7349 | 0.1689 | 135 | 368 | 378 | STACEMDLVQR | | | | | Mascot |
| 1309.5875 | 1309.6219 | 0.0344 | 26 | 368 | 378 | STACEMDLVQR | | | Carbamidomethyl (C)[4] | | Mascot |
| 1332.6715 | 1332.71 | 0.0385 | 29 | 148 | 158 | LQMEPELTLDK | | | Oxidation (M)[3] | | Mascot |
| 1359.6573 | 1359.6603 | 0.003 | 2 | 1716 | 1727 | SGFTSDIKTTMR | | | Oxidation (M)[11] | | Mascot |
| 1434.7443 | 1434.7333 | -0.011 | -8 | 1735 | 1746 | NLMESKICKPAK | | | Carbamidomethyl (C)[8], Oxidation (M)[3] | | Mascot |
| 1434.7443 | 1434.7333 | -0.011 | -8 | 1735 | 1746 | NLMESKICKPAK | | | Carbamidomethyl (C)[8], Oxidation (M)[3] | | Mascot |
| 1448.6863 | 1448.757 | 0.0707 | 49 | 1431 | 1443 | EASLVEDQSTVDR | | | | | Mascot |
| 1581.7504 | 1581.8031 | 0.0527 | 33 | 573 | 586 | DQNEHDENLGAVLK | | | | | Mascot |
| 1677.7941 | 1677.6991 | -0.095 | -57 | 2392 | 2406 | CSYGSHSSTFLVVYK | | | | | Mascot |
| 1677.7941 | 1677.6991 | -0.095 | -57 | 2392 | 2406 | CSYGSHSSTFLVVYK | | | | | Mascot |
| 1785.9528 | 1785.9579 | 0.0051 | 3 | 1083 | 1100 | ATPVVATGLSPAQLMTGR | | | Oxidation (M)[15] | | Mascot |
| 2027.9919 | 2028.0717 | 0.0798 | 39 | 2481 | 2498 | TSGDEVFLTGLRPDTEYK | | | | | Mascot |

| | | | | | | | | | | | | |
|---|---|---|---|---|---|---|---|---|---|---|---|---|
| 2284.2659 | 2284.197 | -0.0689 | -30 | 989 | 1008 | DIEIAHLQTLSSFNVISRLK | | | | | Mascot |

| 28 | PREDICTED: tetratricopeptide repeat protein 17 [Danio rerio] | gi\|326669568 | 133164.1 | 5.96 | 14 | 32 | 0 |
|---|---|---|---|---|---|---|---|

Peptide Information

| Calc. Mass | Obsrv. Mass | ± da | ± ppm | Start Seq. | End Seq. | Sequence | Ion Score | C. I. % | Modification | Rank | Result Type |
|---|---|---|---|---|---|---|---|---|---|---|---|
| 1279.6865 | 1279.7827 | 0.0962 | 75 | 348 | 357 | LEQRLEAQHR | | | | | Mascot |
| 1309.647 | 1309.6219 | -0.0251 | -19 | 1083 | 1093 | QALNYAPHHMK | | | | | Mascot |
| 1317.6545 | 1317.7367 | 0.0822 | 62 | 1013 | 1024 | GGIHYTGESQLR | | | | | Mascot |
| 1317.6545 | 1317.7367 | 0.0822 | 62 | 1013 | 1024 | GGIHYTGESQLR | | | | | Mascot |
| 1434.5768 | 1434.7333 | 0.1565 | 109 | 816 | 827 | AEGGEEEEQDWR | | | | | Mascot |
| 1434.5768 | 1434.7333 | 0.1565 | 109 | 816 | 827 | AEGGEEEEQDWR | | | | | Mascot |
| 1448.6984 | 1448.757 | 0.0586 | 40 | 553 | 565 | MPDCSLKNVVGNR | | | Oxidation (M)[1] | | Mascot |
| 1451.7787 | 1451.7589 | -0.0198 | -14 | 265 | 277 | DVALVNMANVLHR | | | | | Mascot |
| 1625.7917 | 1625.8036 | 0.0119 | 7 | 1033 | 1046 | DKFPSQSFEQVGTR | | | | | Mascot |
| 1661.9109 | 1661.7815 | -0.1294 | -78 | 954 | 969 | VDLTTVASTWLAVSAK | | | | | Mascot |
| 1860.9861 | 1860.8818 | -0.1043 | -56 | 262 | 277 | QHKDVALVNMANVLHR | | | Oxidation (M)[10] | | Mascot |
| 1912.9375 | 1912.9994 | 0.0619 | 32 | 1054 | 1069 | NQTSWVLSSMAALYWR | | | | | Mascot |
| 2233.1111 | 2233.1084 | -0.0027 | -1 | 1147 | 1165 | AMHWYESTLKLQPEFGPAK | | | | | Mascot |
| 2284.1472 | 2284.197 | 0.0498 | 22 | 711 | 730 | QALSLSVHCGQCRASLPLMR | | | Carbamidomethyl (C)[9,12] | | Mascot |
| 2354.2258 | 2354.2368 | 0.011 | 5 | 630 | 651 | GNGTQALVCLRQALHSAPPQHR | | | | | Mascot |
| 3249.6111 | 3249.4709 | -0.1402 | -43 | 1119 | 1146 | MAVEIAPHFVVNHFTLANVYIAMEEFEK | | | | | Mascot |
| 3249.6111 | 3249.4709 | -0.1402 | -43 | 1119 | 1146 | MAVEIAPHFVVNHFTLANVYIAMEEFEK | | | | | Mascot |

| 29 | PREDICTED: LAG1 longevity assurance homolog 2-like [Danio rerio] | gi\|68440265 | 45162.1 | 8.57 | 8 | 32 | 0 |
|---|---|---|---|---|---|---|---|

Peptide Information

| Calc. Mass | Obsrv. Mass | ± da | ± ppm | Start Seq. | End Seq. | Sequence | Ion Score | C. I. % | Modification | Rank | Result Type |
|---|---|---|---|---|---|---|---|---|---|---|---|
| 1115.5455 | 1115.5195 | -0.026 | -23 | 251 | 258 | MFNYARWK | | | | | Mascot |
| 1205.6273 | 1205.6255 | -0.0018 | -1 | 103 | 112 | LSKQTGWTER | | | | | Mascot |
| 1205.6273 | 1205.6255 | -0.0018 | -1 | 103 | 112 | LSKQTGWTER | | | | | Mascot |
| 1317.7008 | 1317.7367 | 0.0359 | 27 | 94 | 105 | NPTQSSVEKLSK | | | | | Mascot |
| 1317.7008 | 1317.7367 | 0.0359 | 27 | 94 | 105 | NPTQSSVEKLSK | | | | | Mascot |
| 1448.7532 | 1448.757 | 0.0038 | 3 | 2 | 13 | LAGLSELFWQER | | | | | Mascot |
| 1581.6287 | 1581.8031 | 0.1744 | 110 | 345 | 357 | DETDESEEEEELK | | | | | Mascot |

| Calc. Mass | Obsrv. Mass | ± da | ± ppm | Start Seq. | End Seq. | Sequence | Ion Score | C. I. % | Modification | Rank | Result Type |
|---|---|---|---|---|---|---|---|---|---|---|---|
| 1727.7831 | 1727.8621 | 0.079 | 46 | 330 | 344 | FLSSNSSVEDERSDR | | | | | Mascot |
| 1727.7831 | 1727.8621 | 0.079 | 46 | 330 | 344 | FLSSNSSVEDERSDR | | | | | Mascot |
| 1925.8961 | 1925.9102 | 0.0141 | 7 | 364 | 381 | KNGAAQNGHATYNNNHSK | | | | | Mascot |
| 2027.8914 | 2028.0717 | 0.1803 | 89 | 365 | 383 | NGAAQNGHATYNNNHSKTE | | | | | Mascot |

30 Zgc:173726 protein [Danio rerio] gi|160774356 52871.3 8.07 11 32 0

**Peptide Information**

| Calc. Mass | Obsrv. Mass | ± da | ± ppm | Start Seq. | End Seq. | Sequence | Ion Score | C. I. % | Modification | Rank | Result Type |
|---|---|---|---|---|---|---|---|---|---|---|---|
| 1022.5126 | 1022.5009 | -0.0117 | -11 | 407 | 414 | NHERTHTK | | | | | Mascot |
| 1045.6516 | 1045.5726 | -0.079 | -76 | 399 | 406 | FRQLQILK | | | | | Mascot |
| 1058.4281 | 1058.5569 | 0.1288 | 122 | 244 | 252 | EFVCSECGK | | | Carbamidomethyl (C)[4] | | Mascot |
| 1115.4496 | 1115.5195 | 0.0699 | 63 | 244 | 252 | EFVCSECGK | | | Carbamidomethyl (C)[4,7] | | Mascot |
| 1252.7412 | 1252.7349 | -0.0063 | -5 | 141 | 150 | TFIKPSYLKR | | | | | Mascot |
| 1332.653 | 1332.71 | 0.057 | 43 | 11 | 21 | QEETEELIDVK | | | | | Mascot |
| 1451.6021 | 1451.7589 | 0.1568 | 108 | 22 | 33 | QESEELSEDEEK | | | | | Mascot |
| 1625.8217 | 1625.8036 | -0.0181 | -11 | 318 | 330 | LHQMIHTGERPYK | | | Oxidation (M)[4] | | Mascot |
| 1677.736 | 1677.6991 | -0.0369 | -22 | 244 | 258 | EFVCSECGKSFNTAR | | | | | Mascot |
| 1677.736 | 1677.6991 | -0.0369 | -22 | 244 | 258 | EFVCSECGKSFNTAR | | | | | Mascot |
| 1798.8615 | 1798.9722 | 0.1107 | 62 | 275 | 289 | CSHCDKSFIYLQNLK | | | | | Mascot |
| 1798.8615 | 1798.9722 | 0.1107 | 62 | 275 | 289 | CSHCDKSFIYLQNLK | | | | | Mascot |
| 1912.9044 | 1912.9994 | 0.095 | 50 | 275 | 289 | CSHCDKSFIYLQNLK | | | Carbamidomethyl (C)[1,4] | | Mascot |
| 2086.9895 | 2087.0176 | 0.0281 | 13 | 122 | 140 | VYQSVQAEVKSNSCSLCGK | | | Carbamidomethyl (C)[14] | | Mascot |
| 2283.0579 | 2283.2014 | 0.1435 | 63 | 318 | 336 | LHQMIHTGERPYKCSHCDK | | | | | Mascot |

31 hypothetical protein LOC798684 [Danio rerio] gi|192455690 14330.4 9.37 5 32 0

**Peptide Information**

| Calc. Mass | Obsrv. Mass | ± da | ± ppm | Start Seq. | End Seq. | Sequence | Ion Score | C. I. % | Modification | Rank | Result Type |
|---|---|---|---|---|---|---|---|---|---|---|---|
| 1581.7512 | 1581.8031 | 0.0519 | 33 | 2 | 16 | ATMVYQVQAPACGAR | | | Oxidation (M)[3] | | Mascot |
| 1785.808 | 1785.9579 | 0.1499 | 84 | 1 | 16 | MATMVYQVQAPACGAR | | | Carbamidomethyl (C)[13], Oxidation (M)[1,4] | | Mascot |
| 1925.9677 | 1925.9102 | -0.0575 | -30 | 111 | 127 | LCDLQVEPGATFITYQK | | | | | Mascot |
| 2233.0786 | 2233.1084 | 0.0298 | 13 | 1 | 20 | MATMVYQVQAPACGARQHVR | | | Oxidation (M)[1] | | Mascot |
| 2337.1001 | 2337.2498 | 0.1497 | 64 | 111 | 131 | LCDLQVEPGATFITYQKCHGG | | | Carbamidomethyl (C)[2] | | Mascot |

32 protein kinase C, theta [Danio rerio] gi|269315875 81814.9 8.06 14 32 0

Peptide Information

| Calc. Mass | Obsrv. Mass | ± da | ± ppm | Start Seq. | End Seq. | Sequence | Ion Score | C. I. % | Modification | Rank | Result Type |
|---|---|---|---|---|---|---|---|---|---|---|---|
| 850.4781 | 850.546 | 0.0679 | 80 | 42 | 48 | GQVYKQK | | | | | Mascot |
| 940.492 | 940.5209 | 0.0289 | 31 | 391 | 399 | MLGKGSFGK | | | Oxidation (M)[1] | | Mascot |
| 1024.6084 | 1024.5231 | -0.0853 | -83 | 67 | 75 | GRVMHVVVK | | | | | Mascot |
| 1045.5789 | 1045.5726 | -0.0063 | -6 | 619 | 626 | LFVREPER | | | | | Mascot |
| 1252.7048 | 1252.7349 | 0.0301 | 24 | 407 | 418 | GSGQFFAVKALK | | | | | Mascot |
| 1359.5238 | 1359.6603 | 0.1365 | 100 | 262 | 272 | CEECGMNVHHK | | | Carbamidomethyl (C)[1], Oxidation (M)[6] | | Mascot |
| 1380.7004 | 1380.6865 | -0.0139 | -10 | 35 | 46 | EAVETEKGQVYK | | | | | Mascot |
| 1661.6652 | 1661.7815 | 0.1163 | 70 | 262 | 275 | CEECGMNVHHKCQK | | | Oxidation (M)[6] | | Mascot |
| 1665.9032 | 1665.9108 | 0.0076 | 5 | 381 | 394 | FTIDSFILHKMLGK | | | Oxidation (M)[11] | | Mascot |
| 1727.8322 | 1727.8621 | 0.0299 | 17 | 178 | 191 | EFVWGLNKQGYQCR | | | | | Mascot |
| 1727.8322 | 1727.8621 | 0.0299 | 17 | 178 | 191 | EFVWGLNKQGYQCR | | | | | Mascot |
| 1785.7828 | 1785.9579 | 0.1751 | 98 | 258 | 272 | QGLKCEECGMNVHHK | | | Carbamidomethyl (C)[5], Oxidation (M)[10] | | Mascot |
| 1925.829 | 1925.9102 | 0.0812 | 42 | 420 | 435 | DVVLMDDDVECTMVER | | | Carbamidomethyl (C)[11] | | Mascot |
| 2028.0259 | 2028.0717 | 0.0458 | 23 | 487 | 504 | STFYAAEIICGLQFLHSK | | | | | Mascot |
| 2284.0989 | 2284.197 | 0.0981 | 43 | 542 | 561 | TCTFCGTPDYIAPEILLGQK | | | Carbamidomethyl (C)[2,5] | | Mascot |

| | | | | | | | | |
|---|---|---|---|---|---|---|---|---|
| 33 | Wu:fo94f09 protein [Danio rerio] | gi\|92096553 | 74291.7 | 8.89 | 11 | 32 | 0 | |

Peptide Information

| Calc. Mass | Obsrv. Mass | ± da | ± ppm | Start Seq. | End Seq. | Sequence | Ion Score | C. I. % | Modification | Rank | Result Type |
|---|---|---|---|---|---|---|---|---|---|---|---|
| 973.4117 | 973.5176 | 0.1059 | 109 | 347 | 355 | CDLCSFASK | | | | | Mascot |
| 1146.6742 | 1146.6381 | -0.0361 | -31 | 273 | 282 | ANLKSHVHLK | | | | | Mascot |
| 1205.5004 | 1205.6255 | 0.1251 | 104 | 283 | 293 | HSASDSFHCSK | | | | | Mascot |
| 1205.5004 | 1205.6255 | 0.1251 | 104 | 283 | 293 | HSASDSFHCSK | | | | | Mascot |
| 1262.5219 | 1262.684 | 0.1621 | 128 | 283 | 293 | HSASDSFHCSK | | | Carbamidomethyl (C)[9] | | Mascot |
| 1434.5632 | 1434.7333 | 0.1701 | 119 | 262 | 272 | CDLCDYRCAMK | | | Carbamidomethyl (C)[1,4] | | Mascot |
| 1434.5632 | 1434.7333 | 0.1701 | 119 | 262 | 272 | CDLCDYRCAMK | | | Carbamidomethyl (C)[1,4] | | Mascot |
| 1451.6794 | 1451.7589 | 0.0795 | 55 | 79 | 92 | NAGASESLQTCVSK | | | Carbamidomethyl (C)[11] | | Mascot |
| 1625.7072 | 1625.8036 | 0.0964 | 59 | 595 | 609 | ESLNIDGSSSQDMSR | | | | | Mascot |
| 1642.7788 | 1642.8677 | 0.0889 | 54 | 65 | 78 | QNCCQIPSTHISVR | | | Carbamidomethyl (C)[3] | | Mascot |
| 1912.868 | 1912.9994 | 0.1314 | 69 | 253 | 268 | VHSGEKPYKCDLCDYR | | | | | Mascot |
| 2210.9263 | 2211.1277 | 0.2014 | 91 | 310 | 329 | QHQPAQPLQCSECSYSCSSK | | | | | Mascot |
| 2210.9263 | 2211.1277 | 0.2014 | 91 | 310 | 329 | QHQPAQPLQCSECSYSCSSK | | | | | Mascot |

| Calc. Mass | Obsrv. Mass | ± da | ± ppm | Start Seq. | End Seq. | Sequence | Ion Score | C. I. % | Modification | Rank | Result Type |
|---|---|---|---|---|---|---|---|---|---|---|---|
| 2233.0964 | 2233.1084 | 0.012 | 5 | 206 | 224 | CQICPYASRNSSQLTVHLR | | | Carbamidomethyl (C)[1] | | Mascot |
| 2283.0798 | 2283.2014 | 0.1216 | 53 | 197 | 214 | IHYDERPFKCQICPYASR | | | Carbamidomethyl (C)[10] | | Mascot |

| | | | | | | | |
|---|---|---|---|---|---|---|---|
| 34 | junctophilin 1b [Danio rerio] | gi\|113195560 | 73661.8 | 9.34 | 9 | 32 | 0 |

**Peptide Information**

| Calc. Mass | Obsrv. Mass | ± da | ± ppm | Start Seq. | End Seq. | Sequence | Ion Score | C. I. % | Modification | Rank | Result Type |
|---|---|---|---|---|---|---|---|---|---|---|---|
| 850.4682 | 850.546 | 0.0778 | 91 | 78 | 83 | GRWLYR | | | | | Mascot |
| 1055.5918 | 1055.606 | 0.0142 | 13 | 600 | 609 | APTPKPVMAK | | | Oxidation (M)[8] | | Mascot |
| 1055.6095 | 1055.606 | -0.0035 | -3 | 610 | 618 | ESKPEPKLK | | | | | Mascot |
| 1115.603 | 1115.5195 | -0.0835 | -75 | 591 | 599 | MPPPVKQYR | | | | | Mascot |
| 1205.7001 | 1205.6255 | -0.0746 | -62 | 213 | 223 | GSLFGSLKQLR | 7 | 0 | | | Mascot |
| 1205.7001 | 1205.6255 | -0.0746 | -62 | 213 | 223 | GSLFGSLKQLR | | | | | Mascot |
| 1262.6277 | 1262.684 | 0.0563 | 45 | 447 | 457 | SPSGSPHFYRK | | | | | Mascot |
| 1642.7101 | 1642.8677 | 0.1576 | 96 | 314 | 327 | RHGYGCTMFPDGTK | | | Carbamidomethyl (C)[6], Oxidation (M)[8] | | Mascot |
| 1727.8387 | 1727.8621 | 0.0234 | 14 | 412 | 426 | DLSPNFHQPGPDYIK | | | | | Mascot |
| 1727.8387 | 1727.8621 | 0.0234 | 14 | 412 | 426 | DLSPNFHQPGPDYIK | | | | | Mascot |
| 1909.7333 | 1909.9108 | 0.1775 | 93 | 6 | 22 | FDFDDGGTFCGGWEDGK | | | Carbamidomethyl (C)[10] | | Mascot |

| | | | | | | | |
|---|---|---|---|---|---|---|---|
| 35 | tektin 2 [Danio rerio] | gi\|37682089 | 48740.9 | 5.63 | 8 | 31 | 0 |

**Peptide Information**

| Calc. Mass | Obsrv. Mass | ± da | ± ppm | Start Seq. | End Seq. | Sequence | Ion Score | C. I. % | Modification | Rank | Result Type |
|---|---|---|---|---|---|---|---|---|---|---|---|
| 1045.5789 | 1045.5726 | -0.0063 | -6 | 68 | 75 | INDITRWK | | | | | Mascot |
| 1115.5691 | 1115.5195 | -0.0496 | -44 | 140 | 149 | EVEVIDGAQR | | | | | Mascot |
| 1798.9657 | 1798.9722 | 0.0065 | 4 | 168 | 182 | LQEARQQLTIDLQDK | | | | | Mascot |
| 1798.9657 | 1798.9722 | 0.0065 | 4 | 168 | 182 | LQEARQQLTIDLQDK | | | | | Mascot |
| 1909.8518 | 1909.9108 | 0.059 | 31 | 80 | 96 | ACAQEVDEEMDALTLSK | | | Carbamidomethyl (C)[2] | | Mascot |
| 1925.8467 | 1925.9102 | 0.0635 | 33 | 80 | 96 | ACAQEVDEEMDALTLSK | | | Carbamidomethyl (C)[2], Oxidation (M)[10] | | Mascot |
| 2070.9199 | 2071.0205 | 0.1006 | 49 | 272 | 287 | THHEEQAHHDMQWQLK | | | Oxidation (M)[11] | | Mascot |
| 2211.0261 | 2211.1277 | 0.1016 | 46 | 271 | 287 | RTHHEEQAHHDMQWQLK | | | | | Mascot |
| 2211.0261 | 2211.1277 | 0.1016 | 46 | 271 | 287 | RTHHEEQAHHDMQWQLK | | | | | Mascot |
| 2283.1365 | 2283.2014 | 0.0649 | 28 | 2 | 22 | ATLSSKPASRYSVSDWATNNK | | | | | Mascot |
| 2396.2966 | 2396.2654 | -0.0312 | -13 | 102 | 123 | ALAATVLPLEVTAECLNLREGR | | | Carbamidomethyl (C)[15] | | Mascot |

| | | | | | | | |
|---|---|---|---|---|---|---|---|
| 36 | Ect2 protein [Danio rerio] | gi\|66911847 | 109863.5 | 8.59 | 14 | 31 | 0 |

Peptide Information

| Calc. Mass | Obsrv. Mass | ± da | ± ppm | Start Seq. | End Seq. | Sequence | Ion Score | C. I. % | Modification | Rank | Result Type |
|---|---|---|---|---|---|---|---|---|---|---|---|
| 940.404 | 940.5209 | 0.1169 | 124 | 816 | 823 | DMDSTLSR | | | Oxidation (M)[2] | | Mascot |
| 973.4995 | 973.5176 | 0.0181 | 19 | 562 | 568 | CERQKPR | | | Carbamidomethyl (C)[1] | | Mascot |
| 1231.5624 | 1231.6849 | 0.1225 | 99 | 631 | 640 | EVMTHINEDK | | | Oxidation (M)[3] | | Mascot |
| 1317.6831 | 1317.7367 | 0.0536 | 41 | 1 | 13 | MADSSILTLGPGR | | | | | Mascot |
| 1317.6831 | 1317.7367 | 0.0536 | 41 | 1 | 13 | MADSSILTLGPGR | | | | | Mascot |
| 1380.7052 | 1380.6865 | -0.0187 | -14 | 953 | 964 | CRDLLAPPEPGR | | | Carbamidomethyl (C)[1] | | Mascot |
| 1434.7303 | 1434.7333 | 0.003 | 2 | 784 | 795 | TLCRQVANTICR | | | Carbamidomethyl (C)[3] | | Mascot |
| 1434.7303 | 1434.7333 | 0.003 | 2 | 784 | 795 | TLCRQVANTICR | | | Carbamidomethyl (C)[3] | | Mascot |
| 1448.7969 | 1448.757 | -0.0399 | -28 | 217 | 229 | VTHLIARSTHGEK | | | | | Mascot |
| 1563.6924 | 1563.7495 | 0.0571 | 37 | 871 | 884 | MSRMSSSSTLAMSR | | | Oxidation (M)[1,4] | | Mascot |
| 1563.6924 | 1563.7495 | 0.0571 | 37 | 871 | 884 | MSRMSSSSTLAMSR | | | Oxidation (M)[1,4] | | Mascot |
| 1625.7588 | 1625.8036 | 0.0448 | 28 | 920 | 934 | TPCSPERADEPAAPK | | | Carbamidomethyl (C)[3] | | Mascot |
| 1691.8706 | 1691.8334 | -0.0372 | -22 | 54 | 68 | VVLVGEACKMAELEK | | | Carbamidomethyl (C)[8], Oxidation (M)[10] | | Mascot |
| 1785.9391 | 1785.9579 | 0.0188 | 11 | 232 | 247 | LAVCMGTPILTPDWIR | | | | | Mascot |
| 2337.1426 | 2337.2498 | 0.1072 | 46 | 874 | 896 | MSSSSTLAMSRSASTFSL TGSIK | | | | | Mascot |
| 2677.4929 | 2677.1145 | -0.3784 | -141 | 575 | 597 | INQSKPECGRQTLVELLI RPVQR | | | | | Mascot |
| 3249.6257 | 3249.4709 | -0.1548 | -48 | 163 | 190 | GEPLPFSSRPLYSMTML NLSLCFTGFRK | | | Carbamidomethyl (C)[22] | | Mascot |
| 3249.6257 | 3249.4709 | -0.1548 | -48 | 163 | 190 | GEPLPFSSRPLYSMTML NLSLCFTGFRK | | | Carbamidomethyl (C)[22] | | Mascot |

| | | | | | | | | |
|---|---|---|---|---|---|---|---|---|
| 37 | novel protein similar to TNF receptor-associated factor 5 (TRAF5) [Danio rerio] | gi\|94732880 | 29458.8 | 6.45 | 8 | 31 | 0 |

Peptide Information

| Calc. Mass | Obsrv. Mass | ± da | ± ppm | Start Seq. | End Seq. | Sequence | Ion Score | C. I. % | Modification | Rank | Result Type |
|---|---|---|---|---|---|---|---|---|---|---|---|
| 1024.5105 | 1024.5231 | 0.0126 | 12 | 127 | 134 | ACPHERVR | | | Carbamidomethyl (C)[2] | | Mascot |
| 1055.4939 | 1055.606 | 0.1121 | 106 | 222 | 230 | NYGCTVRDK | | | | | Mascot |
| 1055.4939 | 1055.606 | 0.1121 | 106 | 222 | 230 | NYGCTVRDK | | | | | Mascot |
| 1317.662 | 1317.7367 | 0.0747 | 57 | 116 | 126 | FSLCNLQDHLK | | | | | Mascot |
| 1317.662 | 1317.7367 | 0.0747 | 57 | 116 | 126 | FSLCNLQDHLK | | | | | Mascot |
| 1380.606 | 1380.6865 | 0.0805 | 58 | 1 | 13 | MAAEENESSGLSR | | | | | Mascot |
| 1448.6256 | 1448.757 | 0.1314 | 91 | 135 | 146 | CSHSDCSDIVLR | | | Carbamidomethyl (C)[1,6] | | Mascot |
| 1563.7042 | 1563.7495 | 0.0453 | 29 | 169 | 181 | QNFPVSQMMGHQK | | | Oxidation (M)[8,9] | | Mascot |
| 1563.7042 | 1563.7495 | 0.0453 | 29 | 169 | 181 | QNFPVSQMMGHQK | | | Oxidation (M)[8,9] | | Mascot |

| | | | | | | | | | | | |
|---|---|---|---|---|---|---|---|---|---|---|---|
| 2070.8599 | 2071.0205 | 0.1606 | 78 | 182 | 200 | SSCPDVEVPCSNNCTQMIK | | | Oxidation (M)[17] | | Mascot |
| 2210.9661 | 2211.1277 | 0.1616 | 73 | 182 | 201 | SSCPDVEVPCSNNCTQMIKR | | | | | Mascot |
| 2210.9661 | 2211.1277 | 0.1616 | 73 | 182 | 201 | SSCPDVEVPCSNNCTQMIKR | | | | | Mascot |
| 2283.9824 | 2284.197 | 0.2146 | 94 | 182 | 201 | SSCPDVEVPCSNNCTQMIKR | | | Carbamidomethyl (C)[3], Oxidation (M)[17] | | Mascot |

38 Si:ch211-154o6.7 protein [Danio rerio] gi|92097789 59687.2 8.6 10 31 0

**Peptide Information**

| Calc. Mass | Obsrv. Mass | ± da | ± ppm | Start Seq. | End Seq. | Sequence | Ion Score | C. I. % | Modification | Rank | Result Type |
|---|---|---|---|---|---|---|---|---|---|---|---|
| 902.5054 | 902.5206 | 0.0152 | 17 | 18 | 25 | AQNTVLTR | | | | | Mascot |
| 1055.4648 | 1055.606 | 0.1412 | 134 | 294 | 302 | GFPMTNNMK | | | Oxidation (M)[4] | | Mascot |
| 1055.4648 | 1055.606 | 0.1412 | 134 | 294 | 302 | GFPMTNNMK | | | Oxidation (M)[4] | | Mascot |
| 1058.6066 | 1058.5569 | -0.0497 | -47 | 18 | 26 | AQNTVLTRR | | | | | Mascot |
| 1661.897 | 1661.7815 | -0.1155 | -69 | 12 | 25 | ENIQFKAQNTVLTR | | | | | Mascot |
| 1665.874 | 1665.9108 | 0.0368 | 22 | 305 | 319 | AGVKPSNTQYMSRVK | | | | | Mascot |
| 1677.8339 | 1677.6991 | -0.1348 | -80 | 491 | 506 | QPCCVSLVPGVYLDGK | | | | | Mascot |
| 1677.8339 | 1677.6991 | -0.1348 | -80 | 491 | 506 | QPCCVSLVPGVYLDGK | | | | | Mascot |
| 1691.8785 | 1691.8334 | -0.0451 | -27 | 455 | 468 | EKPQKIGLYLDCER | | | | | Mascot |
| 1909.8163 | 1909.9108 | 0.0945 | 49 | 125 | 140 | LADMEKMLMCSEHDEK | | | | | Mascot |
| 1925.8112 | 1925.9102 | 0.099 | 51 | 125 | 140 | LADMEKMLMCSEHDEK | | | Oxidation (M)[4] | | Mascot |
| 2677.115 | 2677.1145 | -0.0005 | 0 | 40 | 61 | CSVCLSDFTDPVTLSCEHSFCR | | | Carbamidomethyl (C)[1,4,16,21] | | Mascot |
| 3249.4795 | 3249.4709 | -0.0086 | -3 | 62 | 90 | QCITGHMQASLGPSACPECQKPYNEKDLK | | | Carbamidomethyl (C)[2], Oxidation (M)[7] | | Mascot |
| 3249.4795 | 3249.4709 | -0.0086 | -3 | 62 | 90 | QCITGHMQASLGPSACPECQKPYNEKDLK | | | Carbamidomethyl (C)[2], Oxidation (M)[7] | | Mascot |

39 novel protein [Danio rerio] gi|56208048 32038.8 9.45 7 31 0

**Peptide Information**

| Calc. Mass | Obsrv. Mass | ± da | ± ppm | Start Seq. | End Seq. | Sequence | Ion Score | C. I. % | Modification | Rank | Result Type |
|---|---|---|---|---|---|---|---|---|---|---|---|
| 1155.5793 | 1155.6981 | 0.1188 | 103 | 126 | 135 | DKFTFPTGSR | | | | | Mascot |
| 1252.6644 | 1252.7349 | 0.0705 | 56 | 91 | 101 | IPVRDPDSPTR | | | | | Mascot |
| 1309.6429 | 1309.6219 | -0.021 | -16 | 275 | 286 | VNQGSGFCSRVR | | | | | Mascot |
| 1380.6471 | 1380.6865 | 0.0394 | 29 | 232 | 242 | CQRTTACSLQR | | | Carbamidomethyl (C)[1,7] | | Mascot |
| 1448.705 | 1448.757 | 0.052 | 36 | 31 | 43 | SSNKPDENMISVK | | | | | Mascot |
| 1677.7351 | 1677.6991 | -0.036 | -21 | 2 | 17 | TGDDFQPNNPSGTAEK | | | | | Mascot |
| 1677.7351 | 1677.6991 | -0.036 | -21 | 2 | 17 | TGDDFQPNNPSGTAEK | | | | | Mascot |
| 1909.9324 | 1909.9108 | -0.0216 | -11 | 31 | 47 | SSNKPDENMISVKAFDK | | | | | Mascot |

| | | | | | | | | | | | |
|---|---|---|---|---|---|---|---|---|---|---|---|
| 1925.9272 | 1925.9102 | -0.017 | -9 | 31 | 47 | SSNKPDENMISVKAFDK | | | Oxidation (M)[9] | | Mascot |

40 piwi-like protein 2 [Danio rerio] gi|124286793 116155.3 8.99 13 31 0

**Peptide Information**

| Calc. Mass | Obsrv. Mass | ± da | ± ppm | Start Seq. | End Seq. | Sequence | Ion Score | C. I. % | Modification | Rank | Result Type |
|---|---|---|---|---|---|---|---|---|---|---|---|
| 902.4689 | 902.5206 | 0.0517 | 57 | 715 | 721 | VELRDDR | | | | | Mascot |
| 1205.6346 | 1205.6255 | -0.0091 | -8 | 55 | 65 | AFSVLGEMPVR | | | | | Mascot |
| 1205.6346 | 1205.6255 | -0.0091 | -8 | 55 | 65 | AFSVLGEMPVR | | | | | Mascot |
| 1317.6686 | 1317.7367 | 0.0681 | 52 | 502 | 512 | IDDIEWSKSPK | | | | | Mascot |
| 1317.6686 | 1317.7367 | 0.0681 | 52 | 502 | 512 | IDDIEWSKSPK | | | | | Mascot |
| 1332.7126 | 1332.71 | -0.0026 | -2 | 739 | 750 | VQLVVCIMTGNR | | | | | Mascot |
| 1434.7522 | 1434.7333 | -0.0189 | -13 | 53 | 65 | GRAFSVLGEMPVR | | | Oxidation (M)[10] | | Mascot |
| 1434.7522 | 1434.7333 | -0.0189 | -13 | 53 | 65 | GRAFSVLGEMPVR | | | Oxidation (M)[10] | | Mascot |
| 1581.8206 | 1581.8031 | -0.0175 | -11 | 55 | 68 | AFSVLGEMPVRFGR | | | Oxidation (M)[8] | | Mascot |
| 1661.839 | 1661.7815 | -0.0575 | -35 | 294 | 309 | GSPIPIGSNYIPICCK | | | | | Mascot |
| 1727.8931 | 1727.8621 | -0.031 | -18 | 760 | 775 | LCCIQSPVPSQAINVR | | | | | Mascot |
| 1727.8931 | 1727.8621 | -0.031 | -18 | 760 | 775 | LCCIQSPVPSQAINVR | | | | | Mascot |
| 1913.0095 | 1912.9994 | -0.0101 | -5 | 759 | 775 | KLCCIQSPVPSQAINVR | | | Carbamidomethyl (C)[3] | | Mascot |
| 1926.059 | 1925.9102 | -0.1488 | -77 | 69 | 87 | GITQSIAAEPLVGMARGV R | | | | | Mascot |
| 2070.9656 | 2071.0205 | 0.0549 | 27 | 513 | 530 | DTFTLADGSVTTFVDYYR | | | | | Mascot |
| 2233.1646 | 2233.1084 | -0.0562 | -25 | 149 | 171 | VGSPGTGSSLVSMFRGL GIEPGK | | | | | Mascot |
| 2587.4238 | 2587.2673 | -0.1565 | -60 | 559 | 581 | VITGEILLLPELSFMTGIPE KMR | | | | | Mascot |

41 PREDICTED: oocyte zinc finger protein XlCOF6-like [Danio rerio] gi|326680772 41201.6 9 9 31 0

**Peptide Information**

| Calc. Mass | Obsrv. Mass | ± da | ± ppm | Start Seq. | End Seq. | Sequence | Ion Score | C. I. % | Modification | Rank | Result Type |
|---|---|---|---|---|---|---|---|---|---|---|---|
| 1563.7333 | 1563.7495 | 0.0162 | 10 | 102 | 114 | SFNQSSNINLHMR | | | Oxidation (M)[12] | | Mascot |
| 1563.7333 | 1563.7495 | 0.0162 | 10 | 102 | 114 | SFNQSSNINLHMR | | | Oxidation (M)[12] | | Mascot |
| 1625.6948 | 1625.8036 | 0.1088 | 67 | 298 | 310 | SFYCSSHLNQHMR | | | Oxidation (M)[12] | | Mascot |
| 1661.8138 | 1661.7815 | -0.0323 | -19 | 227 | 241 | IHTGEKPFICTQCGK | | | | | Mascot |
| 1665.7723 | 1665.9108 | 0.1385 | 83 | 311 | 325 | IHTGEKPYTCTQCGK | | | | | Mascot |
| 1677.7836 | 1677.6991 | -0.0845 | -50 | 115 | 128 | IHTGEKPFTCTQCR | | | Carbamidomethyl (C)[10] | | Mascot |
| 1677.7836 | 1677.6991 | -0.0845 | -50 | 115 | 128 | IHTGEKPFTCTQCR | | | Carbamidomethyl (C)[10] | | Mascot |

| | | | | | | | | | | | | |
|---|---|---|---|---|---|---|---|---|---|---|---|---|
| 2233.0237 | 2233.1084 | 0.0847 | 38 | 270 | 288 | SFNCSSQLNQHMRIHTG EK | | | Oxidation (M)[12] | | Mascot |
| 2337.1873 | 2337.2498 | 0.0625 | 27 | 339 | 359 | IHTGEKPAASVWEEFQQ LIGP | | | | | Mascot |
| 3249.4333 | 3249.4709 | 0.0376 | 12 | 186 | 213 | SFSLSSSFNQHMRMHTG EKPFTCTQCGK | | | Carbamidomethyl (C)[23], Oxidation (M)[12] | | Mascot |
| 3249.4775 | 3249.4709 | -0.0066 | -2 | 242 | 269 | SFSYSSHLNHHMRIHTGE KPFTCTQCGK | | | Oxidation (M)[12] | | Mascot |

42 PREDICTED: protein G7c-like [Danio rerio] gi|326665656 34821.8 8.47 7 31 0

Peptide Information

| Calc. Mass | Obsrv. Mass | ± da | ± ppm | Start Seq. | End Seq. | Sequence | Ion Score | C. I. % | Modification | Rank | Result Type |
|---|---|---|---|---|---|---|---|---|---|---|---|
| 1045.6074 | 1045.5726 | -0.0348 | -33 | 111 | 119 | NLIIKGMEK | | | | | Mascot |
| 1058.5629 | 1058.5569 | -0.006 | -6 | 294 | 302 | FSIFSSKSR | | | | | Mascot |
| 1115.5844 | 1115.5195 | -0.0649 | -58 | 291 | 300 | GSRFSIFSSK | | | | | Mascot |
| 1317.7018 | 1317.7367 | 0.0349 | 26 | 56 | 67 | QLNPKTLAMACK | | | | | Mascot |
| 1317.7018 | 1317.7367 | 0.0349 | 26 | 56 | 67 | QLNPKTLAMACK | | | | | Mascot |
| 1380.7594 | 1380.6865 | -0.0729 | -53 | 27 | 38 | NHQDITSIAILR | | | | | Mascot |
| 1434.8175 | 1434.7333 | -0.0842 | -59 | 281 | 293 | LVGLHQDKPKGSR | | | | | Mascot |
| 1434.8175 | 1434.7333 | -0.0842 | -59 | 281 | 293 | LVGLHQDKPKGSR | | | | | Mascot |
| 1642.8547 | 1642.8677 | 0.013 | 8 | 46 | 60 | NADGNFVTPKQLNPK | | | | | Mascot |

43 PREDICTED: zinc finger protein 64 homolog, isoforms 1 and 2 [Danio rerio] gi|292627186 71408.1 8.74 11 31 0

Peptide Information

| Calc. Mass | Obsrv. Mass | ± da | ± ppm | Start Seq. | End Seq. | Sequence | Ion Score | C. I. % | Modification | Rank | Result Type |
|---|---|---|---|---|---|---|---|---|---|---|---|
| 973.4117 | 973.5176 | 0.1059 | 109 | 323 | 331 | CDLCSFASK | | | | | Mascot |
| 1146.6742 | 1146.6381 | -0.0361 | -31 | 249 | 258 | ANLKSHVHLK | | | | | Mascot |
| 1205.5004 | 1205.6255 | 0.1251 | 104 | 259 | 269 | HSASDSFHCSK | | | | | Mascot |
| 1205.5004 | 1205.6255 | 0.1251 | 104 | 259 | 269 | HSASDSFHCSK | | | | | Mascot |
| 1262.5219 | 1262.684 | 0.1621 | 128 | 259 | 269 | HSASDSFHCSK | | | Carbamidomethyl (C)[9] | | Mascot |
| 1434.5632 | 1434.7333 | 0.1701 | 119 | 238 | 248 | CDLCDYRCAMK | | | Carbamidomethyl (C)[1,4] | | Mascot |
| 1434.5632 | 1434.7333 | 0.1701 | 119 | 238 | 248 | CDLCDYRCAMK | | | Carbamidomethyl (C)[1,4] | | Mascot |
| 1451.6794 | 1451.7589 | 0.0795 | 55 | 55 | 68 | NAGASESLQTCVSK | | | Carbamidomethyl (C)[11] | | Mascot |
| 1625.7072 | 1625.8036 | 0.0964 | 59 | 571 | 585 | ESLNIDGSSSQDMSR | | | | | Mascot |
| 1642.7788 | 1642.8677 | 0.0889 | 54 | 41 | 54 | QNCCQIPSTHISVR | | | Carbamidomethyl (C)[3] | | Mascot |
| 1912.868 | 1912.9994 | 0.1314 | 69 | 229 | 244 | VHSGEKPYKCDLCDYR | | | | | Mascot |
| 2210.9263 | 2211.1277 | 0.2014 | 91 | 286 | 305 | QHQPAQPLQCSECSYSC SSK | | | | | Mascot |

| Calc. Mass | Obsrv. Mass | ± da | ± ppm | Start Seq. | End Seq. | Sequence | Ion Score | C. I. % | Modification | Rank | Result Type |
|---|---|---|---|---|---|---|---|---|---|---|---|
| 2210.9263 | 2211.1277 | 0.2014 | 91 | 286 | 305 | QHQPAQPLQCSECSYSC SSK | | | | | Mascot |
| 2233.0964 | 2233.1084 | 0.012 | 5 | 182 | 200 | CQICPYASRNSSQLTVHL R | | | Carbamidomethyl (C)[1] | | Mascot |
| 2283.0798 | 2283.2014 | 0.1216 | 53 | 173 | 190 | IHYDERPFKCQICPYASR | | | Carbamidomethyl (C)[10] | | Mascot |

| | | | | | | | | | |
|---|---|---|---|---|---|---|---|---|---|
| 44 | PREDICTED: insulin-like [Danio rerio] | gi\|326666044 | 7864.3 | 10.45 | 4 | 30 | 0 | | |

Peptide Information

| Calc. Mass | Obsrv. Mass | ± da | ± ppm | Start Seq. | End Seq. | Sequence | Ion Score | C. I. % | Modification | Rank | Result Type |
|---|---|---|---|---|---|---|---|---|---|---|---|
| 1309.6932 | 1309.6219 | -0.0713 | -54 | 62 | 72 | IMIRNSATFLQ | | | Oxidation (M)[2] | | Mascot |
| 2071.1401 | 2071.0205 | -0.1196 | -58 | 27 | 44 | RLCGIQLVEALLIVCGEK | | | Carbamidomethyl (C)[3,15] | | Mascot |
| 2297.228 | 2297.1531 | -0.0749 | -33 | 2 | 25 | QGSGLLLLSLCAGSLLTH SGAGGK | | | Carbamidomethyl (C)[11] | | Mascot |
| 2396.3079 | 2396.2654 | -0.0425 | -18 | 2 | 26 | QGSGLLLLSLCAGSLLTH SGAGGKR | | | | | Mascot |

| | | | | | | | | | |
|---|---|---|---|---|---|---|---|---|---|
| 45 | PREDICTED: hypothetical protein LOC565437 [Danio rerio] | gi\|326673124 | 224597.9 | 4.83 | 1 | | | 21 | 0 |

Peptide Information

| Calc. Mass | Obsrv. Mass | ± da | ± ppm | Start Seq. | End Seq. | Sequence | Ion Score | C. I. % | Modification | Rank | Result Type |
|---|---|---|---|---|---|---|---|---|---|---|---|
| 1205.5466 | 1205.6255 | 0.0789 | 65 | 894 | 903 | DPKCEESEIR | 21 | 0 | | 2 | Mascot |

| | | | | | | | | | |
|---|---|---|---|---|---|---|---|---|---|
| 46 | novel collagen protein [Danio rerio] | gi\|169158763 | 111361.2 | 9.91 | 1 | | | 20 | 0 |

Peptide Information

| Calc. Mass | Obsrv. Mass | ± da | ± ppm | Start Seq. | End Seq. | Sequence | Ion Score | C. I. % | Modification | Rank | Result Type |
|---|---|---|---|---|---|---|---|---|---|---|---|
| 1205.7001 | 1205.6255 | -0.0746 | -62 | 939 | 948 | ELSLIQPHLR | 20 | 0 | | 3 | Mascot |

| | | | | | | | | | |
|---|---|---|---|---|---|---|---|---|---|
| 47 | PREDICTED: hypothetical protein LOC560266 [Danio rerio] | gi\|326676456 | 255155.6 | 8.69 | 1 | | | 20 | 0 |

Peptide Information

| Calc. Mass | Obsrv. Mass | ± da | ± ppm | Start Seq. | End Seq. | Sequence | Ion Score | C. I. % | Modification | Rank | Result Type |
|---|---|---|---|---|---|---|---|---|---|---|---|
| 1205.5393 | 1205.6255 | 0.0862 | 72 | 414 | 423 | EIRSEEDDGR | 20 | 0 | | 4 | Mascot |

| | | | | | | | | | |
|---|---|---|---|---|---|---|---|---|---|
| 48 | Brd8 protein [Danio rerio] | gi\|28277586 | 93582.2 | 4.71 | 1 | | | 19 | 0 |

Peptide Information

| Calc. Mass | Obsrv. Mass | ± da | ± ppm | Start Seq. | End Seq. | Sequence | Ion Score | C. I. % | Modification | Rank | Result Type |
|---|---|---|---|---|---|---|---|---|---|---|---|
| 1205.5757 | 1205.6255 | 0.0498 | 41 | 839 | 849 | QDTVEKDGGTR | 19 | 0 | | 5 | Mascot |

| | | | | | | | | | |
|---|---|---|---|---|---|---|---|---|---|
| 49 | inositol hexaphosphate kinase 2 [Danio rerio] | gi\|77404257 | 48702.1 | 6.01 | 1 | | 18 | 0 | |

Peptide Information

| Calc. Mass | Obsrv. Mass | ± da | ± ppm | Start Seq. | End Seq. | Sequence | Ion Score | C. I. % | Modification | Rank | Result Type |
|---|---|---|---|---|---|---|---|---|---|---|---|
| 1205.671 | 1205.6255 | -0.0455 | -38 | 208 | 217 | YRVPCVLDLK | 18 | 0 | | 6 | Mascot |

| | | | | | | | | | |
|---|---|---|---|---|---|---|---|---|---|
| 50 | PREDICTED: hypothetical protein LOC100320750 [Danio rerio] | gi\|292623710 | 15386.5 | 4.36 | 1 | | 18 | 0 | |

Peptide Information

| Calc. Mass | Obsrv. Mass | ± da | ± ppm | Start Seq. | End Seq. | Sequence | Ion Score | C. I. % | Modification | Rank | Result Type |
|---|---|---|---|---|---|---|---|---|---|---|---|
| 1205.5831 | 1205.6255 | 0.0424 | 35 | 1 | 10 | MDQSLDIDIR | 18 | 0 | | 7 | Mascot |

| | | | | | |
|---|---|---|---|---|---|
| **Gel Idx/Pos** | 176/H3 | **Instr./Gel Origin** | BA2060/110707A | **Process Status** | Analysis Succeeded |
| **Plate [#] Name** | [1] 31496 | **Instrument Sample Name** | | **Spectra** | 11 |

| Rank | Protein Name | Accession No. | Protein MW | Protein PI | Pep. Count | Protein Score | Protein Score C. I. % | Total Ion Score | Total Ion C. I. % |
|---|---|---|---|---|---|---|---|---|---|
| 1 | liver glycogen phosphorylase [Danio rerio] | gi\|56606106 | 111467.3 | 5.5 | 33 | 1,050 | 100 | 796 | 100 |

Peptide Information

| Calc. Mass | Obsrv. Mass | ± da | ± ppm | Start Seq. | End Seq. | Sequence | Ion Score | C. I. % | Modification | Rank | Result Type |
|---|---|---|---|---|---|---|---|---|---|---|---|
| 842.5206 | 842.5123 | -0.0083 | -10 | 853 | 859 | RAINQIK | | | | | Mascot |
| 862.4781 | 862.4818 | 0.0037 | 4 | 947 | 954 | IPPPGEPR | | | | | Mascot |
| 893.4151 | 893.416 | 0.0009 | 1 | 500 | 506 | YGNPWEK | | | | | Mascot |
| 914.5458 | 914.5472 | 0.0014 | 2 | 713 | 719 | HLQIIYK | | | | | Mascot |
| 916.4846 | 916.4868 | 0.0022 | 2 | 586 | 593 | NLAENISR | | | | | Mascot |
| 946.4741 | 946.4759 | 0.0018 | 2 | 564 | 571 | APNDFNLR | | | | | Mascot |
| 946.4741 | 946.4759 | 0.0018 | 2 | 250 | 257 | APNDFNLR | 55 | 99.905 | | | Mascot |
| 994.5832 | 994.5829 | -0.0003 | 0 | 35 | 42 | HLHFTLVK | | | | | Mascot |
| 1053.5728 | 1053.576 | 0.0032 | 3 | 755 | 762 | VIFLENYR | | | | | Mascot |
| 1053.5728 | 1053.576 | 0.0032 | 3 | 755 | 762 | VIFLENYR | 47 | 99.413 | | | Mascot |
| 1161.5575 | 1161.5631 | 0.0056 | 5 | 476 | 484 | YEYGIFNQK | | | | | Mascot |
| 1176.5677 | 1176.6029 | 0.0352 | 30 | 428 | 438 | MSPIEEGGGKR | | | Oxidation (M)[1] | | Mascot |
| 1220.5834 | 1220.5864 | 0.003 | 2 | 886 | 895 | VFADYESYVK | | | | | Mascot |
| 1355.7892 | 1355.6809 | -0.1083 | -80 | 18 | 30 | GIVGVENVAELKK | | | | | Mascot |

| | | | | | | | | | | |
|---|---|---|---|---|---|---|---|---|---|---|
| 1355.7892 | 1355.7896 | 0.0004 | 0 | 18 | 30 | GIVGVENVAELKK | | | | Mascot |
| 1363.653 | 1363.6586 | 0.0056 | 4 | 673 | 683 | LDWDTAWDITK | | | | Mascot |
| 1371.6692 | 1371.6747 | 0.0055 | 4 | 51 | 61 | DYYFALSHTVR | | | | Mascot |
| 1371.6692 | 1371.6747 | 0.0055 | 4 | 51 | 61 | DYYFALSHTVR | 91 | 100 | | Mascot |
| 1386.5995 | 1386.6051 | 0.0056 | 4 | 71 | 81 | TQQFCYEADPK | | | Carbamidomethyl (C)[5] | Mascot |
| 1442.6951 | 1442.6982 | 0.0031 | 2 | 594 | 605 | VLYPNDNFFEGK | | | | Mascot |
| 1511.7013 | 1511.7045 | 0.0032 | 2 | 633 | 646 | NTSGPLSFDSFPDK | | | | Mascot |
| 1519.754 | 1519.7588 | 0.0048 | 3 | 673 | 684 | LDWDTAWDITKR | | | | Mascot |
| 1542.7006 | 1542.7043 | 0.0037 | 2 | 71 | 82 | TQQFCYEADPKR | | | Carbamidomethyl (C)[5] | Mascot |
| 1556.7454 | 1556.7462 | 0.0008 | 1 | 83 | 94 | VYYLSLEFYMGR | | | Oxidation (M)[10] | Mascot |
| 1618.7133 | 1618.717 | 0.0037 | 2 | 487 | 499 | DGWQVEEADDWLR | | | | Mascot |
| 1624.7965 | 1624.8014 | 0.0049 | 3 | 258 | 271 | DFNVGDYIQAVLDR | 118 | 100 | | Mascot |
| 1624.7965 | 1624.8014 | 0.0049 | 3 | 572 | 585 | DFNVGDYIQAVLDR | | | | Mascot |
| 1719.8788 | 1719.884 | 0.0052 | 3 | 507 | 520 | ARPEFMLPVHFYGR | | | | Mascot |
| 1735.8737 | 1735.8815 | 0.0078 | 4 | 507 | 520 | ARPEFMLPVHFYGR | | | Oxidation (M)[6] | Mascot |
| 1735.8737 | 1735.8815 | 0.0078 | 4 | 193 | 206 | ARPEFMLPVHFYGR | 13 | 0 | Oxidation (M)[6] | Mascot |
| 1751.9326 | 1751.9026 | -0.03 | -17 | 611 | 625 | QEYFVVAATLQDVIR | | | | Mascot |
| 1781.9756 | 1781.9781 | 0.0025 | 1 | 735 | 751 | LITSVADVVNNDPVIGR | 136 | 100 | | Mascot |
| 1781.9756 | 1781.9781 | 0.0025 | 1 | 735 | 751 | LITSVADVVNNDPVIGR | | | | Mascot |
| 1859.8922 | 1859.8964 | 0.0042 | 2 | 485 | 499 | IKDGWQVEEADDWLR | 81 | 100 | | Mascot |
| 1859.8922 | 1859.8964 | 0.0042 | 2 | 485 | 499 | IKDGWQVEEADDWLR | | | | Mascot |
| 1861.9443 | 1861.932 | -0.0123 | -7 | 685 | 700 | TFAYTNHTVLPEALER | | | | Mascot |
| 1970.9969 | 1971.0005 | 0.0036 | 2 | 838 | 853 | GYNALQYYENLPELKR | 114 | 100 | | Mascot |
| 1970.9969 | 1971.0005 | 0.0036 | 2 | 838 | 853 | GYNALQYYENLPELKR | | | | Mascot |
| 1993.1117 | 1993.1134 | 0.0017 | 1 | 609 | 625 | LKQEYFVVAATLQDVIR | | | | Mascot |
| 1993.1117 | 1993.1134 | 0.0017 | 1 | 295 | 311 | LKQEYFVVAATLQDVIR | 150 | 100 | | Mascot |
| 2066.0327 | 2066.0371 | 0.0044 | 2 | 929 | 946 | TITEYATEIWGVEPTDLK | | | | Mascot |
| 2120.0991 | 2120.1094 | 0.0103 | 5 | 647 | 665 | VAIQLNDTHPAMAIPELMR | | | | Mascot |
| 2136.094 | 2136.0969 | 0.0029 | 1 | 647 | 665 | VAIQLNDTHPAMAIPELMR | | | Oxidation (M)[12] | Mascot |
| 2152.0889 | 2152.093 | 0.0041 | 2 | 647 | 665 | VAIQLNDTHPAMAIPELMR | | | Oxidation (M)[12,18] | Mascot |
| 2248.1543 | 2248.1423 | -0.012 | -5 | 406 | 424 | INLIHLNHIASLFPEDMDR | | | | Mascot |
| 2264.1492 | 2264.1484 | -0.0008 | 0 | 406 | 424 | INLIHLNHIASLFPEDMDR | | | Oxidation (M)[17] | Mascot |
| 2283.2739 | 2283.1802 | -0.0937 | -41 | 731 | 751 | MIIKLITSVADVVNNDPVIGR | | | Oxidation (M)[1] | Mascot |

2    glycogen phosphorylase, muscle form [Danio rerio]    gi|66472494    96870.5    6.67    18    105    100    47    99.413

Peptide Information

| Calc. Mass | Obsrv. Mass | ± da | ± ppm | Start Seq. | End Seq. | Sequence | Ion Score | C. I. % | Modification | Rank | Result Type |
|---|---|---|---|---|---|---|---|---|---|---|---|
| 842.4617 | 842.5123 | 0.0506 | 60 | 835 | 842 | LAAPDDIK | | | | | Mascot |
| 845.4628 | 845.4667 | 0.0039 | 5 | 571 | 576 | IHEYKR | | | | | Mascot |
| 862.424 | 862.4818 | 0.0578 | 67 | 365 | 371 | AWDICVR | | | | | Mascot |
| 893.4151 | 893.416 | 0.0009 | 1 | 186 | 192 | YGNPWEK | | | | | Mascot |
| 914.5166 | 914.5472 | 0.0306 | 33 | 484 | 491 | TNGITPRR | | | | | Mascot |
| 916.4846 | 916.4868 | 0.0022 | 2 | 271 | 278 | NLAENISR | | | | | Mascot |
| 994.5832 | 994.5829 | -0.0003 | 0 | 35 | 42 | HLHFTLVK | | | | | Mascot |
| 1053.5728 | 1053.576 | 0.0032 | 3 | 643 | 650 | VIFLENYR | | | | | Mascot |
| 1053.5728 | 1053.576 | 0.0032 | 3 | 643 | 650 | VIFLENYR | 47 | 99.413 | | | Mascot |
| 1197.4882 | 1197.5883 | 0.1001 | 84 | 471 | 479 | DFYEMDPHK | | | Oxidation (M)[5] | | Mascot |
| 1355.6742 | 1355.6809 | 0.0067 | 5 | 51 | 61 | DYYFALAHTVR | | | | | Mascot |
| 1355.6742 | 1355.7896 | 0.1154 | 85 | 51 | 61 | DYYFALAHTVR | | | | | Mascot |
| 1371.7994 | 1371.6747 | -0.1247 | -91 | 459 | 470 | IHSDILKATVFK | | | | | Mascot |
| 1371.7994 | 1371.6747 | -0.1247 | -91 | 459 | 470 | IHSDILKATVFK | | | | | Mascot |
| 1386.6536 | 1386.6051 | -0.0485 | -35 | 71 | 81 | TQQSYYEKDPK | | | | | Mascot |
| 1442.6951 | 1442.6982 | 0.0031 | 2 | 279 | 290 | VLYPNDNFFEGK | | | | | Mascot |
| 1511.7754 | 1511.7045 | -0.0709 | -47 | 50 | 61 | RDYYFALAHTVR | | | | | Mascot |
| 1556.7454 | 1556.7462 | 0.0008 | 1 | 83 | 94 | VYYISLEFYMGR | | | Oxidation (M)[10] | | Mascot |
| 1961.0538 | 1960.9794 | -0.0744 | -38 | 576 | 590 | RQLLNCLHIITFYNR | | | Carbamidomethyl (C)[6] | | Mascot |
| 2007.1273 | 2007.0828 | -0.0445 | -22 | 294 | 310 | LKQEYFVVAATLQDIIR | | | | | Mascot |
| 2120.1023 | 2120.1094 | 0.0071 | 3 | 817 | 834 | TISQYAREIWGVEPTLEK | | | | | Mascot |

3 brain glycogen phosphorylase Pygb [Danio rerio] gi|46403219 97393.7 6.11 14 103 100 60 99.969

Peptide Information

| Calc. Mass | Obsrv. Mass | ± da | ± ppm | Start Seq. | End Seq. | Sequence | Ion Score | C. I. % | Modification | Rank | Result Type |
|---|---|---|---|---|---|---|---|---|---|---|---|
| 845.4628 | 845.4667 | 0.0039 | 5 | 571 | 576 | IHEYKR | | | | | Mascot |
| 893.4151 | 893.416 | 0.0009 | 1 | 186 | 192 | YGNPWEK | | | | | Mascot |
| 914.5166 | 914.5472 | 0.0306 | 33 | 484 | 491 | TNGITPRR | | | | | Mascot |
| 916.4846 | 916.4868 | 0.0022 | 2 | 271 | 278 | NLAENISR | | | | | Mascot |
| 994.5832 | 994.5829 | -0.0003 | 0 | 35 | 42 | HLHFTLVK | | | | | Mascot |
| 1053.5728 | 1053.576 | 0.0032 | 3 | 643 | 650 | VIFLENYR | | | | | Mascot |
| 1053.5728 | 1053.576 | 0.0032 | 3 | 643 | 650 | VIFLENYR | 47 | 99.413 | | | Mascot |
| 1220.682 | 1220.5864 | -0.0956 | -78 | 599 | 609 | FVPRTVMIGGK | | | Oxidation (M)[7] | | Mascot |
| 1355.6742 | 1355.6809 | 0.0067 | 5 | 51 | 61 | DYYFALAHTVR | | | | | Mascot |

| | | | | | | | | | | | |
|---|---|---|---|---|---|---|---|---|---|---|---|
| 1355.6742 | 1355.7896 | 0.1154 | 85 | 51 | 61 | DYYFALAHTVR | | | | | Mascot |
| 1442.6951 | 1442.6982 | 0.0031 | 2 | 279 | 290 | VLYPNDNFFEGK | | | | | Mascot |
| 1735.8737 | 1735.8815 | 0.0078 | 4 | 193 | 206 | ARPEYMLPVHFYGR | | | | | Mascot |
| 1735.8737 | 1735.8815 | 0.0078 | 4 | 193 | 206 | ARPEYMLPVHFYGR | 13 | 0 | | | Mascot |
| 1751.8685 | 1751.9026 | 0.0341 | 19 | 193 | 206 | ARPEYMLPVHFYGR | | | Oxidation (M)[6] | | Mascot |
| 1838.8821 | 1838.8909 | 0.0088 | 5 | 171 | 185 | IAHGWQIEEADDWLR | | | | | Mascot |
| 1970.8953 | 1971.0005 | 0.1052 | 53 | 772 | 787 | FKVFADYESYISCQDR | | | | | Mascot |
| 1970.8953 | 1971.0005 | 0.1052 | 53 | 772 | 787 | FKVFADYESYISCQDR | | | | | Mascot |
| 2007.1273 | 2007.0828 | -0.0445 | -22 | 294 | 310 | LKQEYFVVAATLQDIIR | | | | | Mascot |
| 2120.0964 | 2120.1094 | 0.013 | 6 | 439 | 458 | RINMAHLCVVGSHAVNG VAR | | | Oxidation (M)[4] | | Mascot |

4 glycogen phosphorylase, brain form [Danio rerio] gi|136256027 97365.7 6.11 13 97 100 60 99.969

**Peptide Information**

| Calc. Mass | Obsrv. Mass | ± da | ± ppm | Start Seq. | End Seq. | Sequence | Ion Score | C. I. % | Modification | Rank | Result Type |
|---|---|---|---|---|---|---|---|---|---|---|---|
| 845.4628 | 845.4667 | 0.0039 | 5 | 571 | 576 | IHEYKR | | | | | Mascot |
| 893.4151 | 893.416 | 0.0009 | 1 | 186 | 192 | YGNPWEK | | | | | Mascot |
| 914.5166 | 914.5472 | 0.0306 | 33 | 484 | 491 | TNGITPRR | | | | | Mascot |
| 916.4846 | 916.4868 | 0.0022 | 2 | 271 | 278 | NLAENISR | | | | | Mascot |
| 994.5832 | 994.5829 | -0.0003 | 0 | 35 | 42 | HLHFTLVK | | | | | Mascot |
| 1053.5728 | 1053.576 | 0.0032 | 3 | 643 | 650 | VIFLENYR | | | | | Mascot |
| 1053.5728 | 1053.576 | 0.0032 | 3 | 643 | 650 | VIFLENYR | 47 | 99.413 | | | Mascot |
| 1220.682 | 1220.5864 | -0.0956 | -78 | 599 | 609 | FVPRTVMIGGK | | | Oxidation (M)[7] | | Mascot |
| 1355.6742 | 1355.6809 | 0.0067 | 5 | 51 | 61 | DYYFALAHTVR | | | | | Mascot |
| 1355.6742 | 1355.7896 | 0.1154 | 85 | 51 | 61 | DYYFALAHTVR | | | | | Mascot |
| 1442.6951 | 1442.6982 | 0.0031 | 2 | 279 | 290 | VLYPNDNFFEGK | | | | | Mascot |
| 1735.8737 | 1735.8815 | 0.0078 | 4 | 193 | 206 | ARPEYMLPVHFYGR | | | | | Mascot |
| 1735.8737 | 1735.8815 | 0.0078 | 4 | 193 | 206 | ARPEYMLPVHFYGR | 13 | 0 | | | Mascot |
| 1751.8685 | 1751.9026 | 0.0341 | 19 | 193 | 206 | ARPEYMLPVHFYGR | | | Oxidation (M)[6] | | Mascot |
| 1838.8821 | 1838.8909 | 0.0088 | 5 | 171 | 185 | IAHGWQIEEADDWLR | | | | | Mascot |
| 2007.1273 | 2007.0828 | -0.0445 | -22 | 294 | 310 | LKQEYFVVAATLQDIIR | | | | | Mascot |
| 2120.0964 | 2120.1094 | 0.013 | 6 | 439 | 458 | RINMAHLCVVGSHAVNG VAR | | | Oxidation (M)[4] | | Mascot |

5 novel protein similar to vertebrate NifU-like N-terminal domain containing (NIFUN) [Danio rerio] gi|37606130 17748.2 8.87 7 52 73.186 14 0

**Peptide Information**

| Calc. Mass | Obsrv. Mass | ± da | ± ppm | Start | End | Sequence | Ion | C. I. % | Modification | Rank | Result Type |
|---|---|---|---|---|---|---|---|---|---|---|---|

| Calc. Mass | Obsrv. Mass | ± da | ± ppm | Start Seq. | End Seq. | Sequence | Ion Score | C. I. % | Modification | Rank | Result Type |
|---|---|---|---|---|---|---|---|---|---|---|---|
| 862.5145 | 862.4818 | -0.0327 | -38 | 81 | 87 | IIDARFK | | | | | Mascot |
| 916.5461 | 916.4868 | -0.0593 | -65 | 116 | 123 | IKNTEIAK | | | | | Mascot |
| 946.525 | 946.4759 | -0.0491 | -52 | 1 | 9 | MALAAAARR | | | Oxidation (M)[1] | | Mascot |
| 946.525 | 946.4759 | -0.0491 | -52 | 1 | 9 | MALAAAARR | 14 | 0 | Oxidation (M)[1] | | Mascot |
| 1045.5636 | 1045.5667 | 0.0031 | 3 | 44 | 53 | NVGSLDKNAK | | | | | Mascot |
| 1161.5746 | 1161.5631 | -0.0115 | -10 | 155 | 164 | DEKETLAEAR | | | | | Mascot |
| 2100.043 | 2100.0022 | -0.0408 | -19 | 88 | 108 | TFGCGSAIASSSLATEWVKGK | | | | | Mascot |
| 2283.1545 | 2283.1802 | 0.0257 | 11 | 124 | 143 | ELCLPPVKLHCSMLAEDAIK | | | Carbamidomethyl (C)[3], Oxidation (M)[13] | | Mascot |

6 calcium/calmodulin-dependent protein kinase II inhibitor 2 [Danio rerio] gi|223029393 8468.1 5.24 6 50 57.502

**Peptide Information**

| Calc. Mass | Obsrv. Mass | ± da | ± ppm | Start Seq. | End Seq. | Sequence | Ion Score | C. I. % | Modification | Rank | Result Type |
|---|---|---|---|---|---|---|---|---|---|---|---|
| 842.5206 | 842.5123 | -0.0083 | -10 | 48 | 55 | LGQIGRAK | | | | | Mascot |
| 845.4363 | 845.4667 | 0.0304 | 36 | 57 | 63 | VVIEDDR | | | | | Mascot |
| 994.4509 | 994.5829 | 0.132 | 133 | 70 | 79 | GMTDKSSPGV | | | Oxidation (M)[2] | | Mascot |
| 1001.5374 | 1001.566 | 0.0286 | 29 | 56 | 63 | RVVIEDDR | | | | | Mascot |
| 1542.8373 | 1542.7043 | -0.133 | -86 | 57 | 69 | VVIEDDRIDEVLK | | | | | Mascot |
| 3337.4082 | 3337.7339 | 0.3257 | 98 | 12 | 43 | MSGYGADTDVSQMSFSCGLQDTNSFFGASQAK | | | | | Mascot |

7 MCM4 minichromosome maintenance deficient 4, mitotin (S. cerevisiae) [Danio rerio] gi|33989734 83986.4 5.88 12 50 52.316 10 0

**Peptide Information**

| Calc. Mass | Obsrv. Mass | ± da | ± ppm | Start Seq. | End Seq. | Sequence | Ion Score | C. I. % | Modification | Rank | Result Type |
|---|---|---|---|---|---|---|---|---|---|---|---|
| 845.4839 | 845.4667 | -0.0172 | -20 | 98 | 105 | ADLGSVRK | | | | | Mascot |
| 855.4359 | 855.4388 | 0.0029 | 3 | 671 | 677 | DYIAFAR | | | | | Mascot |
| 914.5166 | 914.5472 | 0.0306 | 33 | 87 | 95 | SGIRGTPAR | | | | | Mascot |
| 994.5792 | 994.5829 | 0.0037 | 4 | 722 | 730 | LAEAHAKVR | | | | | Mascot |
| 1001.5486 | 1001.566 | 0.0174 | 17 | 96 | 104 | QRADLGSVR | | | | | Mascot |
| 1109.5851 | 1109.5858 | 0.0007 | 1 | 396 | 404 | THIDAIHFR | | | | | Mascot |
| 1587.8601 | 1587.7875 | -0.0726 | -46 | 364 | 377 | VQPGDRVNITGIYR | | | | | Mascot |
| 1719.921 | 1719.884 | -0.037 | -22 | 707 | 721 | GMVSAYPRQLESLIR | | | | | Mascot |
| 1735.9159 | 1735.8815 | -0.0344 | -20 | 707 | 721 | GMVSAYPRQLESLIR | | | Oxidation (M)[2] | | Mascot |
| 1735.9701 | 1735.8815 | -0.0886 | -51 | 611 | 625 | TTIENIQLPHTLLSR | 4 | 0 | | | Mascot |

| Calc. Mass | Obsrv. Mass | ± da | ± ppm | Start Seq. | End Seq. | Sequence | Ion Score | C. I. % | Modification | Rank | Result Type |
|---|---|---|---|---|---|---|---|---|---|---|---|
| 1790.8556 | 1790.9106 | 0.055 | 31 | 12 | 28 | DPPTPASEDPLSPPSQR | | | | | Mascot |
| 1992.9219 | 1993.1134 | 0.1915 | 96 | 162 | 178 | EEENAGLDLNEPLYMQK | | | | | Mascot |
| 1992.9219 | 1993.1134 | 0.1915 | 96 | 162 | 178 | EEENAGLDLNEPLYMQK | 10 | 0 | | | Mascot |
| 2225.0754 | 2225.1245 | 0.0491 | 22 | 60 | 80 | SVLQSEIDASSPLMYGTP SSR | | | | | Mascot |

8 DNA replication licensing factor MCM4 [Danio rerio] gi|321267495 94464.9 5.99 13 47 4.859 10 0

**Peptide Information**

| Calc. Mass | Obsrv. Mass | ± da | ± ppm | Start Seq. | End Seq. | Sequence | Ion Score | C. I. % | Modification | Rank | Result Type |
|---|---|---|---|---|---|---|---|---|---|---|---|
| 845.4839 | 845.4667 | -0.0172 | -20 | 98 | 105 | ADLGSVRK | | | | | Mascot |
| 855.4359 | 855.4388 | 0.0029 | 3 | 671 | 677 | DYIAFAR | | | | | Mascot |
| 914.5166 | 914.5472 | 0.0306 | 33 | 87 | 95 | SGIRGTPAR | | | | | Mascot |
| 994.5792 | 994.5829 | 0.0037 | 4 | 722 | 730 | LAEAHAKVR | | | | | Mascot |
| 1001.5486 | 1001.566 | 0.0174 | 17 | 96 | 104 | QRADLGSVR | | | | | Mascot |
| 1109.5851 | 1109.5858 | 0.0007 | 1 | 396 | 404 | THIDAIHFR | | | | | Mascot |
| 1197.5898 | 1197.5883 | -0.0015 | -1 | 802 | 810 | YQQLFDDLR | | | | | Mascot |
| 1587.8601 | 1587.7875 | -0.0726 | -46 | 364 | 377 | VQPGDRVNITGIYR | | | | | Mascot |
| 1719.921 | 1719.884 | -0.037 | -22 | 707 | 721 | GMVSAYPRQLESLIR | | | | | Mascot |
| 1735.9159 | 1735.8815 | -0.0344 | -20 | 707 | 721 | GMVSAYPRQLESLIR | | | Oxidation (M)[2] | | Mascot |
| 1735.9701 | 1735.8815 | -0.0886 | -51 | 611 | 625 | TTIENIQLPHTLLSR | 4 | 0 | | | Mascot |
| 1790.8556 | 1790.9106 | 0.055 | 31 | 12 | 28 | DPPTPASEDPLSPPSQR | | | | | Mascot |
| 1992.9219 | 1993.1134 | 0.1915 | 96 | 162 | 178 | EEENAGLDLNEPLYMQK | | | | | Mascot |
| 1992.9219 | 1993.1134 | 0.1915 | 96 | 162 | 178 | EEENAGLDLNEPLYMQK | 10 | 0 | | | Mascot |
| 2225.0754 | 2225.1245 | 0.0491 | 22 | 60 | 80 | SVLQSEIDASSPLMYGTP SSR | | | | | Mascot |

9 Si:dkeyp-11g8.5 protein [Danio rerio] gi|133778765 35914.5 9.16 9 42 0

**Peptide Information**

| Calc. Mass | Obsrv. Mass | ± da | ± ppm | Start Seq. | End Seq. | Sequence | Ion Score | C. I. % | Modification | Rank | Result Type |
|---|---|---|---|---|---|---|---|---|---|---|---|
| 862.4814 | 862.4818 | 0.0004 | 0 | 43 | 50 | MVLASVAR | | | Oxidation (M)[1] | | Mascot |
| 994.5203 | 994.5829 | 0.0626 | 63 | 288 | 296 | TDATLAQFK | | | | | Mascot |
| 1371.7128 | 1371.6747 | -0.0381 | -28 | 276 | 287 | VSRTHFDPTGVR | | | | | Mascot |
| 1371.7227 | 1371.6747 | -0.048 | -35 | 77 | 89 | VLRGPTQADESAK | | | | | Mascot |
| 1386.7886 | 1386.6051 | -0.1835 | -132 | 38 | 50 | ELAARMVLASVAR | | | | | Mascot |
| 1442.6338 | 1442.6982 | 0.0644 | 45 | 119 | 131 | QLPCQCHGSMPGK | | | Carbamidomethyl (C)[4] | | Mascot |
| 1735.8345 | 1735.8815 | 0.047 | 27 | 204 | 219 | TLQKVEEADTSDQSGK | | | | | Mascot |

| | | | | | | | | | | | |
|---|---|---|---|---|---|---|---|---|---|---|---|
| 1735.8345 | 1735.8815 | 0.047 | 27 | 204 | 219 | TLQKVEEADTSDQSGK | | | | | Mascot |
| 1781.9254 | 1781.9781 | 0.0527 | 30 | 104 | 118 | VFLKPGSMVEENLYR | | | | | Mascot |
| 1781.9254 | 1781.9781 | 0.0527 | 30 | 104 | 118 | VFLKPGSMVEENLYR | | | | | Mascot |
| 3337.5913 | 3337.7339 | 0.1426 | 43 | 93 | 118 | QLLHCQWCEERVFLKPG SMVEENLYR | | | Carbamidomethyl (C)[5,8], Oxidation (M)[19] | | Mascot |

| | | | | | | | |
|---|---|---|---|---|---|---|---|
| 10 | novel protein similar to vertebrate polymerase (DNA directed) kappa (POLK) [Danio rerio] | gi\|169153899 | 97852.6 | 6.97 | 16 | 41 | 0 |

Peptide Information

| Calc. Mass | Obsrv. Mass | ± da | ± ppm | Start Seq. | End Seq. | Sequence | Ion Score | C. I. % | Modification | Rank | Result Type |
|---|---|---|---|---|---|---|---|---|---|---|---|
| 845.4727 | 845.4667 | -0.006 | -7 | 89 | 95 | LTVDLER | | | | | Mascot |
| 864.3879 | 864.4891 | 0.1012 | 117 | 26 | 33 | AGMEGLDR | | | Oxidation (M)[3] | | Mascot |
| 893.476 | 893.416 | -0.06 | -67 | 74 | 80 | ITKEQMK | | | Oxidation (M)[6] | | Mascot |
| 916.4482 | 916.4868 | 0.0386 | 42 | 81 | 88 | QAQAEVDR | | | | | Mascot |
| 1371.6433 | 1371.6747 | 0.0314 | 23 | 804 | 814 | NMRENQSVEHK | | | | | Mascot |
| 1371.6433 | 1371.6747 | 0.0314 | 23 | 804 | 814 | NMRENQSVEHK | | | | | Mascot |
| 1519.7122 | 1519.7588 | 0.0466 | 31 | 656 | 669 | QEELSGEPSSLESK | | | | | Mascot |
| 1571.9268 | 1571.7905 | -0.1363 | -87 | 830 | 842 | LILLQIHTDHIQK | | | | | Mascot |
| 1781.8486 | 1781.9781 | 0.1295 | 73 | 234 | 249 | AEVFGTSAEEAVREMR | | | | | Mascot |
| 1781.8486 | 1781.9781 | 0.1295 | 73 | 234 | 249 | AEVFGTSAEEAVREMR | | | | | Mascot |
| 1790.761 | 1790.9106 | 0.1496 | 84 | 639 | 655 | GSAMDSNEHTDLSAAER | | | | | Mascot |
| 1838.7571 | 1838.8909 | 0.1338 | 73 | 372 | 387 | TFGEMSDAGEQYSLCK | | | Carbamidomethyl (C)[15], Oxidation (M)[5] | | Mascot |
| 1859.8956 | 1859.8964 | 0.0008 | 0 | 623 | 638 | DVNLTAFNQHIDECLK | | | | | Mascot |
| 1859.8956 | 1859.8964 | 0.0008 | 0 | 623 | 638 | DVNLTAFNQHIDECLK | | | | | Mascot |
| 1960.7712 | 1960.9794 | 0.2082 | 106 | 682 | 700 | ASTSTSEPMADGDDGND FK | | | Oxidation (M)[9] | | Mascot |
| 2211.064 | 2211.1106 | 0.0466 | 21 | 604 | 622 | ISTQVLQCLTCPVCNKEM K | | | Carbamidomethyl (C)[8], Oxidation (M)[18] | | Mascot |
| 2225.0974 | 2225.1245 | 0.0271 | 12 | 256 | 277 | TSLTASAGIAPNMMLAKV CSDK | | | Oxidation (M)[13] | | Mascot |
| 2248.0737 | 2248.1423 | 0.0686 | 31 | 620 | 638 | EMKDVNLTAFNQHIDECL K | | | | | Mascot |
| 2264.0686 | 2264.1484 | 0.0798 | 35 | 620 | 638 | EMKDVNLTAFNQHIDECL K | | | Oxidation (M)[2] | | Mascot |
| 2283.0347 | 2283.1802 | 0.1455 | 64 | 847 | 867 | DMIIHEDFAGFTSGNNVN ASK | | | Oxidation (M)[2] | | Mascot |

| | | | | | | | |
|---|---|---|---|---|---|---|---|
| 11 | PREDICTED: FCH and double SH3 domains protein 2-like, partial [Danio rerio] | gi\|326675898 | 21477.2 | 9.13 | 7 | 40 | 0 |

Peptide Information

| Calc. Mass | Obsrv. Mass | ± da | ± ppm | Start Seq. | End Seq. | Sequence | Ion Score | C. I. % | Modification | Rank | Result Type |
|---|---|---|---|---|---|---|---|---|---|---|---|

| Calc. Mass | Obsrv. Mass | ± da | ± ppm | Start Seq. | End Seq. | Sequence | Ion Score | C. I. % | Modification | Rank | Result Type |
|---|---|---|---|---|---|---|---|---|---|---|---|
| 893.4628 | 893.416 | -0.0468 | -52 | 93 | 99 | NVYGVWR | | | | | Mascot |
| 946.5138 | 946.4759 | -0.0379 | -40 | 140 | 147 | KCIEQLGR | | | | | Mascot |
| 946.5138 | 946.4759 | -0.0379 | -40 | 140 | 147 | KCIEQLGR | | | | | Mascot |
| 1053.5436 | 1053.576 | 0.0324 | 31 | 26 | 34 | NTHVEQLGR | | | | | Mascot |
| 1053.5436 | 1053.576 | 0.0324 | 31 | 26 | 34 | NTHVEQLGR | 7 | 0 | | | Mascot |
| 1386.7595 | 1386.6051 | -0.1544 | -111 | 1 | 13 | MASLCHLKIVAGK | | | Oxidation (M)[1] | | Mascot |
| 1587.8588 | 1587.7875 | -0.0713 | -45 | 148 | 161 | IQTELQDSVKDLAK | | | | | Mascot |
| 1751.9398 | 1751.9026 | -0.0372 | -21 | 20 | 34 | VTQELKNTHVEQLGR | | | | | Mascot |
| 1861.8828 | 1861.932 | 0.0492 | 26 | 78 | 92 | DWPGIKPDDQRTDYR | | | | | Mascot |

| | | | | | | | | |
|---|---|---|---|---|---|---|---|---|
| 12 | PREDICTED: supervillin [Danio rerio] | gi\|326672283 | 147558.1 | 5.63 | 18 | 40 | 0 | |

**Peptide Information**

| Calc. Mass | Obsrv. Mass | ± da | ± ppm | Start Seq. | End Seq. | Sequence | Ion Score | C. I. % | Modification | Rank | Result Type |
|---|---|---|---|---|---|---|---|---|---|---|---|
| 842.5457 | 842.5123 | -0.0334 | -40 | 717 | 724 | KVAVQLGK | | | | | Mascot |
| 946.4992 | 946.4759 | -0.0233 | -25 | 297 | 303 | EEQWKVK | | | | | Mascot |
| 946.4992 | 946.4759 | -0.0233 | -25 | 297 | 303 | EEQWKVK | | | | | Mascot |
| 981.5952 | 981.5854 | -0.0098 | -10 | 463 | 471 | NPLRALAAR | | | | | Mascot |
| 1109.5771 | 1109.5858 | 0.0087 | 8 | 989 | 997 | LFCVRGETK | | | Carbamidomethyl (C)[3] | | Mascot |
| 1179.5562 | 1179.666 | 0.1098 | 93 | 385 | 394 | NSTIEVMEEK | | | | | Mascot |
| 1179.5674 | 1179.666 | 0.0986 | 84 | 1058 | 1068 | EMGLNSNSSLK | | | | | Mascot |
| 1220.5688 | 1220.5864 | 0.0176 | 14 | 73 | 83 | DVGNARDQMAK | | | Oxidation (M)[9] | | Mascot |
| 1386.7528 | 1386.6051 | -0.1477 | -107 | 779 | 789 | FLDWAERKPPK | | | | | Mascot |
| 1442.629 | 1442.6982 | 0.0692 | 48 | 1091 | 1102 | KAYDCMLEDPGK | | | Carbamidomethyl (C)[5], Oxidation (M)[6] | | Mascot |
| 1519.7421 | 1519.7588 | 0.0167 | 11 | 276 | 289 | SSADVEPKMSLAER | | | | | Mascot |
| 1571.7853 | 1571.7905 | 0.0052 | 3 | 632 | 644 | EFWSLLGGKTDYR | | | | | Mascot |
| 1582.6663 | 1582.7301 | 0.0638 | 40 | 975 | 988 | GCREGSSTNTGDWR | | | Carbamidomethyl (C)[2] | | Mascot |
| 1832.8007 | 1832.9417 | 0.141 | 77 | 395 | 409 | EKEVMTFDDEETFGR | | | | | Mascot |
| 1859.965 | 1859.8964 | -0.0686 | -37 | 1296 | 1310 | DEFNTLPNWKQLNLK | | | | | Mascot |
| 1859.965 | 1859.8964 | -0.0686 | -37 | 1296 | 1310 | DEFNTLPNWKQLNLK | | | | | Mascot |
| 1861.8578 | 1861.932 | 0.0742 | 40 | 579 | 593 | QCFFWSGEFANVIEK | | | Carbamidomethyl (C)[2] | | Mascot |
| 2006.9698 | 2007.0828 | 0.113 | 56 | 139 | 156 | STSLDLEPGEQERTTICK | | | | | Mascot |
| 2248.1528 | 2248.1423 | -0.0105 | -5 | 558 | 578 | LVEPTANSLNSGDCYLLI TPK | | | | | Mascot |
| 2284.2092 | 2284.1797 | -0.0295 | -13 | 958 | 977 | EPPCFLQLFLGGLVIHKG CR | | | Carbamidomethyl (C)[4] | | Mascot |

| | | | | | | | | |
|---|---|---|---|---|---|---|---|---|
| 13 | RecName: Full=Acyl-CoA synthetase family member 2, mitochondrial; Flags: Precursor | gi\|123914455 | 67470.3 | 8.42 | 12 | 40 | 0 | |

Peptide Information

| Calc. Mass | Obsrv. Mass | ± da | ± ppm | Start Seq. | End Seq. | Sequence | Ion Score | C. I. % | Modification | Rank | Result Type |
|---|---|---|---|---|---|---|---|---|---|---|---|
| 842.5458 | 842.5123 | -0.0335 | -40 | 5 | 11 | ILLTNLR | | | | | Mascot |
| 864.4243 | 864.4891 | 0.0648 | 75 | 472 | 478 | ECITKDR | | | | | Mascot |
| 870.5771 | 870.542 | -0.0351 | -40 | 500 | 506 | IKDLIIR | | | | | Mascot |
| 907.4665 | 907.4691 | 0.0026 | 3 | 470 | 476 | TRECITK | | | Carbamidomethyl (C)[4] | | Mascot |
| 914.5417 | 914.5472 | 0.0055 | 6 | 331 | 338 | ANLRAIEK | | | | | Mascot |
| 1161.5754 | 1161.5631 | -0.0123 | -11 | 164 | 174 | VQCNAVVCPTK | | | | | Mascot |
| 1197.5027 | 1197.5883 | 0.0856 | 71 | 177 | 185 | SQHYCDMLK | | | Carbamidomethyl (C)[5], Oxidation (M)[7] | | Mascot |
| 1511.7886 | 1511.7045 | -0.0841 | -56 | 421 | 434 | IVTVGCISPHTEAK | | | Carbamidomethyl (C)[6] | | Mascot |
| 1582.8621 | 1582.7301 | -0.132 | -83 | 420 | 434 | KIVTVGCISPHTEAK | | | | | Mascot |
| 1624.6876 | 1624.8014 | 0.1138 | 70 | 536 | 548 | DERMGEEVCACIR | 1 | 0 | Carbamidomethyl (C)[9,11] | | Mascot |
| 1624.6876 | 1624.8014 | 0.1138 | 70 | 536 | 548 | DERMGEEVCACIR | | | Carbamidomethyl (C)[9,11] | | Mascot |
| 2065.9688 | 2066.0371 | 0.0683 | 33 | 479 | 495 | WYKTGDIASLDQFAYCK | | | Carbamidomethyl (C)[16] | | Mascot |
| 2211.1689 | 2211.1106 | -0.0583 | -26 | 435 | 455 | VVDPTTGEIVPLGAQGEL MIR | | | Oxidation (M)[19] | | Mascot |

| | | | | | | | | | | |
|---|---|---|---|---|---|---|---|---|---|---|
| 14 | PREDICTED: titin [Danio rerio] | gi\|189523699 | 3645668 | 6.16 | 72 | 39 | 0 | 9 | 0 |

Peptide Information

| Calc. Mass | Obsrv. Mass | ± da | ± ppm | Start Seq. | End Seq. | Sequence | Ion Score | C. I. % | Modification | Rank | Result Type |
|---|---|---|---|---|---|---|---|---|---|---|---|
| 842.5094 | 842.5123 | 0.0029 | 3 | 15232 | 15239 | LSIPGVTR | | | | | Mascot |
| 845.4587 | 845.4667 | 0.008 | 9 | 29341 | 29347 | IRAENSR | | | | | Mascot |
| 855.4206 | 855.4388 | 0.0182 | 21 | 8657 | 8663 | EEAIEHK | | | | | Mascot |
| 862.4814 | 862.4818 | 0.0004 | 0 | 27341 | 27348 | IVCSSVVR | | | | | Mascot |
| 864.4937 | 864.4891 | -0.0046 | -5 | 20241 | 20247 | GYIVERK | | | | | Mascot |
| 870.4203 | 870.542 | 0.1217 | 140 | 6908 | 6915 | LSDSGTYK | | | | | Mascot |
| 882.4679 | 882.5694 | 0.1015 | 115 | 2876 | 2882 | YKVSTER | | | | | Mascot |
| 893.4145 | 893.416 | 0.0015 | 2 | 23146 | 23153 | TSEGCIAR | | | Carbamidomethyl (C)[5] | | Mascot |
| 907.4705 | 907.4691 | -0.0014 | -2 | 27434 | 27440 | NSLLWMK | | | Oxidation (M)[6] | | Mascot |
| 914.5669 | 914.5472 | -0.0197 | -22 | 9214 | 9221 | DKIGLQLK | | | | | Mascot |
| 916.4985 | 916.4868 | -0.0117 | -13 | 9747 | 9754 | GDEILLEK | | | | | Mascot |
| 946.5203 | 946.4759 | -0.0444 | -47 | 12485 | 12493 | AGQEITLSK | | | | | Mascot |
| 946.5832 | 946.4759 | -0.1073 | -113 | 2700 | 2707 | FRIVVQGK | | | | | Mascot |
| 981.5112 | 981.5854 | 0.0742 | 76 | 3392 | 3399 | RFSSSLER | | | | | Mascot |
| 994.5203 | 994.5829 | 0.0626 | 63 | 9416 | 9423 | EKEVFSQK | | | | | Mascot |
| 1001.6141 | 1001.566 | -0.0481 | -48 | 11629 | 11636 | LEFVRPIK | | | | | Mascot |

| | | | | | | | | | | |
|---|---|---|---|---|---|---|---|---|---|---|
| 1045.5347 | 1045.5667 | 0.032 | 31 | 7144 | 7153 | CQIGGTPEIK | | | | Mascot |
| 1053.5364 | 1053.576 | 0.0396 | 38 | 9270 | 9277 | RDIYFPDK | 5 | 0 | | Mascot |
| 1053.584 | 1053.576 | -0.008 | -8 | 24308 | 24315 | YEFRVIAR | | | | Mascot |
| 1109.6255 | 1109.5858 | -0.0397 | -36 | 17783 | 17790 | NFVFLKWR | | | | Mascot |
| 1161.703 | 1161.5631 | -0.1399 | -120 | 9689 | 9698 | VKAVPYPFIK | | | | Mascot |
| 1176.5677 | 1176.6029 | 0.0352 | 30 | 17549 | 17559 | AVNMIGEGETR | | | | Mascot |
| 1179.6732 | 1179.666 | -0.0072 | -6 | 28209 | 28218 | ITGYIVETRK | | | | Mascot |
| 1179.7096 | 1179.666 | -0.0436 | -37 | 10001 | 10010 | KEITPTHILK | | | | Mascot |
| 1197.6011 | 1197.5883 | -0.0128 | -11 | 15414 | 15423 | GTSYWSRVNK | | | | Mascot |
| 1220.5865 | 1220.5864 | -0.0001 | 0 | 18604 | 18614 | RGSDDIVTSDR | | | | Mascot |
| 1355.714 | 1355.6809 | -0.0331 | -24 | 13271 | 13281 | EYLFRVIACNK | | | | Mascot |
| 1355.7794 | 1355.7896 | 0.0102 | 8 | 18483 | 18494 | NSATLGWLKPLR | | | | Mascot |
| 1363.7004 | 1363.6586 | -0.0418 | -31 | 29924 | 29934 | GLKENVEYHFK | | | | Mascot |
| 1371.6387 | 1371.6747 | 0.036 | 26 | 26182 | 26194 | YDITASNSNGTTK | | | | Mascot |
| 1371.7478 | 1371.6747 | -0.0731 | -53 | 3410 | 3422 | LTPAEVTIGESVR | 11 | 0 | | Mascot |
| 1386.6648 | 1386.6051 | -0.0597 | -43 | 17536 | 17546 | GLTEDTEYRFR | | | | Mascot |
| 1419.7227 | 1419.7164 | -0.0063 | -4 | 4116 | 4127 | ESPFVSDVKQQR | | | | Mascot |
| 1442.6945 | 1442.6982 | 0.0037 | 3 | 6367 | 6379 | ADTGTYTCKLANK | | | Carbamidomethyl (C)[8] | Mascot |
| 1511.7375 | 1511.7045 | -0.033 | -22 | 22038 | 22050 | IGGLEEGIEYEFR | | | | Mascot |
| 1519.7274 | 1519.7588 | 0.0314 | 21 | 11644 | 11656 | ETAEFSIELSHEK | | | | Mascot |
| 1542.7369 | 1542.7043 | -0.0326 | -21 | 19518 | 19530 | ITNLEAGQAYCFR | | | Carbamidomethyl (C)[11] | Mascot |
| 1556.7526 | 1556.7462 | -0.0064 | -4 | 31126 | 31138 | MTASEALEHPWLR | | | Oxidation (M)[1] | Mascot |
| 1571.7853 | 1571.7905 | 0.0052 | 3 | 21648 | 21660 | IGDLQEGNLYFFR | | | | Mascot |
| 1582.7716 | 1582.7301 | -0.0415 | -26 | 17623 | 17635 | TMLTNNFPVVQMR | | | Oxidation (M)[2,12] | Mascot |
| 1587.7649 | 1587.7875 | 0.0226 | 14 | 6149 | 6161 | DESWILEDPSIQR | | | | Mascot |
| 1618.7959 | 1618.717 | -0.0789 | -49 | 30832 | 30847 | FGVSDPSAPTDPVTTK | | | | Mascot |
| 1624.8074 | 1624.8014 | -0.006 | -4 | 15583 | 15596 | IFMDISADELLCVR | | | | Mascot |
| 1624.8727 | 1624.8014 | -0.0713 | -44 | 7363 | 7376 | ISCVENTPHLTILK | | | Carbamidomethyl (C)[3] | Mascot |
| 1719.8734 | 1719.884 | 0.0106 | 6 | 23154 | 23169 | DPCDPPGTPVPVIVTR | | | Carbamidomethyl (C)[3] | Mascot |
| 1735.8496 | 1735.8815 | 0.0319 | 18 | 25594 | 25610 | GVNKYGTGEALESDPAK | | | | Mascot |
| 1735.9589 | 1735.8815 | -0.0774 | -45 | 26957 | 26971 | NTEYSTTLIIPKVTR | 2 | 0 | | Mascot |
| 1751.8997 | 1751.9026 | 0.0029 | 2 | 9851 | 9865 | EVHLPVIQDDSMLQK | | | | Mascot |
| 1781.8956 | 1781.9781 | 0.0825 | 46 | 8632 | 8646 | DVYFSAKQDEEIIPK | | | | Mascot |
| 1781.9644 | 1781.9781 | 0.0137 | 8 | 19357 | 19372 | DILLAPEAELDADLRK | | | | Mascot |
| 1790.9183 | 1790.9106 | -0.0077 | -4 | 7435 | 7450 | VAGSQPLTVNWYKDGR | | | | Mascot |
| 1832.9574 | 1832.9417 | -0.0157 | -9 | 18442 | 18458 | VMAVNKYGIGLPQDSPK | | | Oxidation (M)[2] | Mascot |

| | | | | | | | | | | | |
|---|---|---|---|---|---|---|---|---|---|---|---|
| 1838.9 | 1838.8909 | -0.0091 | -5 | 25318 | 25333 | DPCAPPSQPRVMNITR | | | Carbamidomethyl (C)[3] | | Mascot |
| 1859.9021 | 1859.8964 | -0.0057 | -3 | 28719 | 28734 | ELDPGEKYSLTYTSTR | | | | | Mascot |
| 1859.928 | 1859.8964 | -0.0316 | -17 | 2604 | 2619 | NQELKPSANVQIMSER | | | Oxidation (M)[13] | | Mascot |
| 1861.9337 | 1861.932 | -0.0017 | -1 | 12684 | 12698 | LQVCEIRPRDQGEYR | | | | | Mascot |
| 1960.9498 | 1960.9794 | 0.0296 | 15 | 1778 | 1794 | TEEGYYEAISSVELKSR | | | | | Mascot |
| 1971.0375 | 1971.0005 | -0.037 | -19 | 13319 | 13335 | IFLSWEPPKWDGGTPIK | | | | | Mascot |
| 1971.0375 | 1971.0005 | -0.037 | -19 | 13319 | 13335 | IFLSWEPPKWDGGTPIK | | | | | Mascot |
| 1992.98 | 1993.1134 | 0.1334 | 67 | 2757 | 2773 | DTITFEVTVNYEGITYK | | | | | Mascot |
| 1993.027 | 1993.1134 | 0.0864 | 43 | 32557 | 32575 | AVQESVSSKIQGGSEMVI K | | | Oxidation (M)[16] | | Mascot |
| 2007.0182 | 2007.0828 | 0.0646 | 32 | 19472 | 19490 | DHVSITWDAPLIDGGSPV K | | | | | Mascot |
| 2066.0474 | 2066.0371 | -0.0103 | -5 | 976 | 993 | KQDVTYVTEVTAMVQEP K | | | | | Mascot |
| 2120.1089 | 2120.1094 | 0.0005 | 0 | 5556 | 5574 | MDNTNVLLAKDVSLQCV LK | | | Oxidation (M)[1] | | Mascot |
| 2136.0906 | 2136.0969 | 0.0063 | 3 | 22659 | 22677 | SAFISVRVLDTPDMPANF R | | | | | Mascot |
| 2152.0854 | 2152.093 | 0.0076 | 4 | 22659 | 22677 | SAFISVRVLDTPDMPANF R | | | Oxidation (M)[14] | | Mascot |
| 2211.1438 | 2211.1106 | -0.0332 | -15 | 6982 | 7001 | SCEIALRGDTAILELHNID K | | | | | Mascot |
| 2225.1382 | 2225.1245 | -0.0137 | -6 | 29539 | 29559 | VAATNRFGIGGFLQSEVV ECK | | | | | Mascot |
| 2232.9575 | 2233.0947 | 0.1372 | 61 | 1525 | 1542 | MINEFGYCSLDYEVAYSR | | | Carbamidomethyl (C)[8], Oxidation (M)[1] | | Mascot |
| 2248.0107 | 2248.1423 | 0.1316 | 59 | 7076 | 7094 | YNITTTESSCILECLNSDK | | | Carbamidomethyl (C)[10,14] | | Mascot |
| 2264.2537 | 2264.1484 | -0.1053 | -47 | 25712 | 25732 | AEDPLFLPSPPAKPKIIDS TK | | | | | Mascot |
| 2283.1899 | 2283.1802 | -0.0097 | -4 | 3763 | 3783 | EHPSQVMSALTESVTPLT IVK | | | Oxidation (M)[7] | | Mascot |
| 2284.1819 | 2284.1797 | -0.0022 | -1 | 21598 | 21618 | EITKDSVSLIWDPPVIDGG SR | | | | | Mascot |
| 3337.4834 | 3337.7339 | 0.2505 | 75 | 4869 | 4900 | DTGTYTCTAANEAGSSS CSSSVTVQEPPVFVK | | | Carbamidomethyl (C)[7,18] | | Mascot |

| | | | | | | | | |
|---|---|---|---|---|---|---|---|---|
| 15 | Si:dkey-63j12.1 [Danio rerio] | gi\|63100606 | 31611.3 | 10.27 | 10 | 39 | 0 | |

**Peptide Information**

| Calc. Mass | Obsrv. Mass | ± da | ± ppm | Start Seq. | End Seq. | Sequence | Ion Score | C. I. % | Modification | Rank | Result Type |
|---|---|---|---|---|---|---|---|---|---|---|---|
| 893.5025 | 893.416 | -0.0865 | -97 | 189 | 195 | CKIFLNR | | | | | Mascot |
| 916.5098 | 916.4868 | -0.023 | -25 | 150 | 158 | TPGAVESKK | | | | | Mascot |
| 1045.5748 | 1045.5667 | -0.0081 | -8 | 237 | 245 | RASQVLSER | | | | | Mascot |
| 1179.5536 | 1179.666 | 0.1124 | 95 | 198 | 207 | TSMTANQDRR | | | | | Mascot |
| 1179.5536 | 1179.666 | 0.1124 | 95 | 198 | 207 | TSMTANQDRR | | | | | Mascot |
| 1571.7379 | 1571.7905 | 0.0526 | 33 | 175 | 188 | LMGDACSVNMVIYR | | | | | Mascot |
| 1587.7328 | 1587.7875 | 0.0547 | 34 | 175 | 188 | LMGDACSVNMVIYR | | | Oxidation (M)[2] | | Mascot |

| Calc. Mass | Obsrv. Mass | ± da | ± ppm | Start Seq. | End Seq. | Sequence | Ion Score | C. I. % | Modification | Rank | Result Type |
|---|---|---|---|---|---|---|---|---|---|---|---|
| 1790.849 | 1790.9106 | 0.0616 | 34 | 159 | 174 | SPAATNQTNFINCQPK | | | Carbamidomethyl (C)[13] | | Mascot |
| 1859.8634 | 1859.8964 | 0.033 | 18 | 175 | 190 | LMGDACSVNMVIYRCK | | | Carbamidomethyl (C)[6] | | Mascot |
| 1859.8634 | 1859.8964 | 0.033 | 18 | 175 | 190 | LMGDACSVNMVIYRCK | | | Carbamidomethyl (C)[6] | | Mascot |
| 1861.9225 | 1861.932 | 0.0095 | 5 | 158 | 174 | KSPAATNQTNFINCQPK | | | | | Mascot |
| 1960.8865 | 1960.9794 | 0.0929 | 47 | 256 | 272 | TSHMMHGARQFAEAAQR | | | Oxidation (M)[4,5] | | Mascot |
| 2233.0027 | 2233.0947 | 0.092 | 41 | 119 | 136 | CMFVQVLYHACQNYWEAK | | | | | Mascot |

| | | | | | | | | | |
|---|---|---|---|---|---|---|---|---|---|
| 16 | cullin 1-like [Danio rerio] | gi\|47087291 | 89546.8 | 8.18 | 14 | 38 | 0 | | |

**Peptide Information**

| Calc. Mass | Obsrv. Mass | ± da | ± ppm | Start Seq. | End Seq. | Sequence | Ion Score | C. I. % | Modification | Rank | Result Type |
|---|---|---|---|---|---|---|---|---|---|---|---|
| 864.4825 | 864.4891 | 0.0066 | 8 | 220 | 227 | GPTLSVYK | | | | | Mascot |
| 882.4501 | 882.5694 | 0.1193 | 135 | 320 | 326 | MYNLVSR | | | | | Mascot |
| 893.4363 | 893.416 | -0.0203 | -23 | 514 | 520 | DLNEQFK | | | | | Mascot |
| 914.5128 | 914.5472 | 0.0344 | 38 | 680 | 687 | VNINVPMK | | | | | Mascot |
| 946.5277 | 946.4759 | -0.0518 | -55 | 750 | 757 | CIDILIEK | | | | | Mascot |
| 946.5277 | 946.4759 | -0.0518 | -55 | 750 | 757 | CIDILIEK | | | | | Mascot |
| 1176.6194 | 1176.6029 | -0.0165 | -14 | 167 | 175 | ECLFRPLNK | | | Carbamidomethyl (C)[2] | | Mascot |
| 1582.7642 | 1582.7301 | -0.0341 | -22 | 314 | 326 | NEDLGRMYNLVSR | | | Oxidation (M)[7] | | Mascot |
| 1587.7584 | 1587.7875 | 0.0291 | 18 | 492 | 504 | QACGFEYTSKLQR | | | Carbamidomethyl (C)[3] | | Mascot |
| 1618.7384 | 1618.717 | -0.0214 | -13 | 228 | 240 | EYFEAQFLADTER | | | | | Mascot |
| 1624.8224 | 1624.8014 | -0.021 | -13 | 26 | 39 | SGIQQVYTRQSMAR | | | | | Mascot |
| 1624.8224 | 1624.8014 | -0.021 | -13 | 26 | 39 | SGIQQVYTRQSMAR | | | | | Mascot |
| 1735.9449 | 1735.8815 | -0.0634 | -37 | 725 | 739 | HQQLLAEVLNQLSSR | | | | | Mascot |
| 1735.9449 | 1735.8815 | -0.0634 | -37 | 725 | 739 | HQQLLAEVLNQLSSR | | | | | Mascot |
| 2152.126 | 2152.093 | -0.033 | -15 | 577 | 594 | LTWLYHLSKGELVTNCFK | | | | | Mascot |
| 2264.2622 | 2264.1484 | -0.1138 | -50 | 725 | 743 | HQQLLAEVLNQLSSRFKPR | | | | | Mascot |
| 2283.1423 | 2283.1802 | 0.0379 | 17 | 96 | 115 | SYLTNLLKDGEDLMDESVLK | | | | | Mascot |

| | | | | | | | | | |
|---|---|---|---|---|---|---|---|---|---|
| 17 | titin a [Danio rerio] | gi\|110005909 | 3646149.2 | 6.18 | 72 | 38 | 0 | 9 | 0 |

**Peptide Information**

| Calc. Mass | Obsrv. Mass | ± da | ± ppm | Start Seq. | End Seq. | Sequence | Ion Score | C. I. % | Modification | Rank | Result Type |
|---|---|---|---|---|---|---|---|---|---|---|---|
| 842.5094 | 842.5123 | 0.0029 | 3 | 15232 | 15239 | LSIPGVTR | | | | | Mascot |
| 845.4587 | 845.4667 | 0.008 | 9 | 29341 | 29347 | IRAENSR | | | | | Mascot |
| 855.4206 | 855.4388 | 0.0182 | 21 | 8657 | 8663 | EEAIEHK | | | | | Mascot |

| | | | | | | | | | | |
|---|---|---|---|---|---|---|---|---|---|---|
| 862.4814 | 862.4818 | 0.0004 | 0 | 27341 | 27348 | IVCSSVVR | | | | Mascot |
| 864.4937 | 864.4891 | -0.0046 | -5 | 20241 | 20247 | GYIVERK | | | | Mascot |
| 870.493 | 870.542 | 0.049 | 56 | 8546 | 8553 | TDIPLPSK | | | | Mascot |
| 882.4679 | 882.5694 | 0.1015 | 115 | 2876 | 2882 | YKVSTER | | | | Mascot |
| 893.4145 | 893.416 | 0.0015 | 2 | 23146 | 23153 | TSEGCIAR | | | Carbamidomethyl (C)[5] | Mascot |
| 907.4705 | 907.4691 | -0.0014 | -2 | 27434 | 27440 | NSLLWMK | | | Oxidation (M)[6] | Mascot |
| 914.5669 | 914.5472 | -0.0197 | -22 | 9214 | 9221 | DKIGLQLK | | | | Mascot |
| 916.4985 | 916.4868 | -0.0117 | -13 | 9747 | 9754 | GDEILLEK | | | | Mascot |
| 946.5203 | 946.4759 | -0.0444 | -47 | 12485 | 12493 | AGQEITLSK | | | | Mascot |
| 946.5832 | 946.4759 | -0.1073 | -113 | 2700 | 2707 | FRIVVQGK | | | | Mascot |
| 981.5112 | 981.5854 | 0.0742 | 76 | 3392 | 3399 | RFSSSLER | | | | Mascot |
| 994.5203 | 994.5829 | 0.0626 | 63 | 9416 | 9423 | EKEVFSQK | | | | Mascot |
| 1001.5625 | 1001.566 | 0.0035 | 3 | 8691 | 8698 | EEIINQKK | | | | Mascot |
| 1045.5347 | 1045.5667 | 0.032 | 31 | 7144 | 7153 | CQIGGTPEIK | | | | Mascot |
| 1053.5364 | 1053.576 | 0.0396 | 38 | 9270 | 9277 | RDIYFPDK | 5 | 0 | | Mascot |
| 1053.584 | 1053.576 | -0.008 | -8 | 24308 | 24315 | YEFRVIAR | | | | Mascot |
| 1109.6255 | 1109.5858 | -0.0397 | -36 | 17783 | 17790 | NFVFLKWR | | | | Mascot |
| 1161.703 | 1161.5631 | -0.1399 | -120 | 9689 | 9698 | VKAVPYPFIK | | | | Mascot |
| 1176.5677 | 1176.6029 | 0.0352 | 30 | 17549 | 17559 | AVNMIGEGETR | | | | Mascot |
| 1179.6732 | 1179.666 | -0.0072 | -6 | 28209 | 28218 | ITGYIVETRK | | | | Mascot |
| 1179.7096 | 1179.666 | -0.0436 | -37 | 10001 | 10010 | KEITPTHILK | | | | Mascot |
| 1197.6011 | 1197.5883 | -0.0128 | -11 | 15414 | 15423 | GTSYWSRVNK | | | | Mascot |
| 1220.5865 | 1220.5864 | -0.0001 | 0 | 18604 | 18614 | RGSDDIVTSDR | | | | Mascot |
| 1355.714 | 1355.6809 | -0.0331 | -24 | 13271 | 13281 | EYLFRVIACNK | | | | Mascot |
| 1355.7794 | 1355.7896 | 0.0102 | 8 | 18483 | 18494 | NSATLGWLKPLR | | | | Mascot |
| 1363.7004 | 1363.6586 | -0.0418 | -31 | 29924 | 29934 | GLKENVEYHFK | | | | Mascot |
| 1371.6387 | 1371.6747 | 0.036 | 26 | 26182 | 26194 | YDITASNSNGTTK | | | | Mascot |
| 1371.7478 | 1371.6747 | -0.0731 | -53 | 3410 | 3422 | LTPAEVTIGESVR | 11 | 0 | | Mascot |
| 1386.6648 | 1386.6051 | -0.0597 | -43 | 17536 | 17546 | GLTEDTEYRFR | | | | Mascot |
| 1419.7227 | 1419.7164 | -0.0063 | -4 | 4116 | 4127 | ESPFVSDVKQQR | | | | Mascot |
| 1442.6945 | 1442.6982 | 0.0037 | 3 | 6367 | 6379 | ADTGTYTCKLANK | | | Carbamidomethyl (C)[8] | Mascot |
| 1511.7375 | 1511.7045 | -0.033 | -22 | 22038 | 22050 | IGGLEEGIEYEFR | | | | Mascot |
| 1519.7274 | 1519.7588 | 0.0314 | 21 | 11644 | 11656 | ETAEFSIELSHEK | | | | Mascot |
| 1542.7369 | 1542.7043 | -0.0326 | -21 | 19518 | 19530 | ITNLEAGQAYCFR | | | Carbamidomethyl (C)[11] | Mascot |
| 1556.7526 | 1556.7462 | -0.0064 | -4 | 31126 | 31138 | MTASEALEHPWLR | | | Oxidation (M)[1] | Mascot |
| 1571.7853 | 1571.7905 | 0.0052 | 3 | 21648 | 21660 | IGDLQEGNLYFFR | | | | Mascot |
| 1582.7716 | 1582.7301 | -0.0415 | -26 | 17623 | 17635 | TMLTNNFPVVQMR | | | Oxidation (M)[2,12] | Mascot |

| | | | | | | | | | | |
|---|---|---|---|---|---|---|---|---|---|---|
| 1587.7649 | 1587.7875 | 0.0226 | 14 | 6149 | 6161 | DESWILEDPSIQR | | | | Mascot |
| 1618.7959 | 1618.717 | -0.0789 | -49 | 30832 | 30847 | FGVSDPSAPTDPVTTK | | | | Mascot |
| 1624.8074 | 1624.8014 | -0.006 | -4 | 15583 | 15596 | IFMDISADELLCVR | | | | Mascot |
| 1624.8727 | 1624.8014 | -0.0713 | -44 | 7363 | 7376 | ISCVENTPHLTILK | | | Carbamidomethyl (C)[3] | Mascot |
| 1719.8734 | 1719.884 | 0.0106 | 6 | 23154 | 23169 | DPCDPPGTPVPVIVTR | | | Carbamidomethyl (C)[3] | Mascot |
| 1735.8496 | 1735.8815 | 0.0319 | 18 | 25594 | 25610 | GVNKYGTGEALESDPAK | | | | Mascot |
| 1735.9589 | 1735.8815 | -0.0774 | -45 | 26957 | 26971 | NTEYSTTLIIPKVTR | 2 | 0 | | Mascot |
| 1751.7177 | 1751.9026 | 0.1849 | 106 | 4585 | 4600 | DHEGEYTCEAVGEAGK | | | Carbamidomethyl (C)[8] | Mascot |
| 1781.8956 | 1781.9781 | 0.0825 | 46 | 8632 | 8646 | DVYFSAKQDEEIIPK | | | | Mascot |
| 1781.9644 | 1781.9781 | 0.0137 | 8 | 19357 | 19372 | DILLAPEAELDADLRK | | | | Mascot |
| 1790.9183 | 1790.9106 | -0.0077 | -4 | 7435 | 7450 | VAGSQPLTVNWYKDGR | | | | Mascot |
| 1832.9562 | 1832.9417 | -0.0145 | -8 | 522 | 537 | ELLLIPPEMTTTTTEK | | | Oxidation (M)[9] | Mascot |
| 1838.9 | 1838.8909 | -0.0091 | -5 | 25318 | 25333 | DPCAPPSQPRVMNITR | | | Carbamidomethyl (C)[3] | Mascot |
| 1859.9021 | 1859.8964 | -0.0057 | -3 | 28719 | 28734 | ELDPGEKYSLTYTSTR | | | | Mascot |
| 1859.928 | 1859.8964 | -0.0316 | -17 | 2604 | 2619 | NQELKPSANVQIMSER | | | Oxidation (M)[13] | Mascot |
| 1861.9337 | 1861.932 | -0.0017 | -1 | 12684 | 12698 | LQVCEIRPRDQGEYR | | | | Mascot |
| 1960.9498 | 1960.9794 | 0.0296 | 15 | 1778 | 1794 | TEEGYYEAISSVELKSR | | | | Mascot |
| 1971.0375 | 1971.0005 | -0.037 | -19 | 13319 | 13335 | IFLSWEPPKWDGGTPIK | | | | Mascot |
| 1971.0375 | 1971.0005 | -0.037 | -19 | 13319 | 13335 | IFLSWEPPKWDGGTPIK | | | | Mascot |
| 1992.98 | 1993.1134 | 0.1334 | 67 | 2757 | 2773 | DTITFEVTVNYEGITYK | | | | Mascot |
| 1993.027 | 1993.1134 | 0.0864 | 43 | 32557 | 32575 | AVQESVSSKIQGGSEMVIK | | | Oxidation (M)[16] | Mascot |
| 2007.0182 | 2007.0828 | 0.0646 | 32 | 19472 | 19490 | DHVSITWDAPLIDGGSPVK | | | | Mascot |
| 2066.0474 | 2066.0371 | -0.0103 | -5 | 976 | 993 | KQDVTYVTEVTAMVQEPK | | | | Mascot |
| 2120.1089 | 2120.1094 | 0.0005 | 0 | 5556 | 5574 | MDNTNVLLAKDVSLQCVLK | | | Oxidation (M)[1] | Mascot |
| 2136.0906 | 2136.0969 | 0.0063 | 3 | 22659 | 22677 | SAFISVRVLDTPDMPANFR | | | | Mascot |
| 2152.0854 | 2152.093 | 0.0076 | 4 | 22659 | 22677 | SAFISVRVLDTPDMPANFR | | | Oxidation (M)[14] | Mascot |
| 2211.1438 | 2211.1106 | -0.0332 | -15 | 6982 | 7001 | SCEIALRGDTAILELHNIDK | | | | Mascot |
| 2225.1382 | 2225.1245 | -0.0137 | -6 | 29539 | 29559 | VAATNRFGIGGFLQSEVVECK | | | | Mascot |
| 2232.9575 | 2233.0947 | 0.1372 | 61 | 1525 | 1542 | MINEFGYCSLDYEVAYSR | | | Carbamidomethyl (C)[8], Oxidation (M)[1] | Mascot |
| 2248.0107 | 2248.1423 | 0.1316 | 59 | 7076 | 7094 | YNITTTESSCILECLNSDK | | | Carbamidomethyl (C)[10,14] | Mascot |
| 2264.2537 | 2264.1484 | -0.1053 | -47 | 25712 | 25732 | AEDPLFLPSPPAKPKIIDSTK | | | | Mascot |
| 2283.1899 | 2283.1802 | -0.0097 | -4 | 3763 | 3783 | EHPSQVMSALTESVTPLTIVK | | | Oxidation (M)[7] | Mascot |
| 2284.1819 | 2284.1797 | -0.0022 | -1 | 21598 | 21618 | EITKDSVSLIWDPPVIDGGSR | | | | Mascot |
| 3337.4834 | 3337.7339 | 0.2505 | 75 | 4869 | 4900 | DTGTYTCTAANEAGSSS | | | Carbamidomethyl (C)[7,18] | Mascot |

CSSSVTVQEPPVFVK

| | | | | | | |
|---|---|---|---|---|---|---|
| 18 | RecName: Full=Methionine aminopeptidase 1; Short=MAP 1; Short=MetAP 1; AltName: Full=Peptidase M 1 | gi\|172046804 | 43538.2 | 6.23 | 9 | 38 | 0 |

**Peptide Information**

| Calc. Mass | Obsrv. Mass | ± da | ± ppm | Start Seq. | End Seq. | Sequence | Ion Score | C. I. % | Modification | Rank | Result Type |
|---|---|---|---|---|---|---|---|---|---|---|---|
| 845.5025 | 845.4667 | -0.0358 | -42 | 140 | 146 | VVCKLAR | | | Carbamidomethyl (C)[3] | | Mascot |
| 914.5305 | 914.5472 | 0.0167 | 18 | 270 | 277 | ELGNIIQK | | | | | Mascot |
| 1001.4621 | 1001.566 | 0.1039 | 104 | 289 | 297 | SYCGHGIHK | | | | | Mascot |
| 1719.8767 | 1719.884 | 0.0073 | 4 | 129 | 143 | ILNAEEIEGMRVVCK | | | Oxidation (M)[10] | | Mascot |
| 1832.8088 | 1832.9417 | 0.1329 | 73 | 191 | 207 | SCCTSVNEVICHGIPDR | | | | | Mascot |
| 2006.8542 | 2007.0828 | 0.2286 | 114 | 2 | 19 | AAVETRECETEGCHSEAK | | | Carbamidomethyl (C)[8] | | Mascot |
| 2210.9111 | 2211.1106 | 0.1995 | 90 | 1 | 19 | MAAVETRECETEGCHSEAK | | | Carbamidomethyl (C)[9,14], Oxidation (M)[1] | | Mascot |
| 2225.0781 | 2225.1245 | 0.0464 | 21 | 278 | 297 | HAQANGFSVVRSYCGHGIHK | | | Carbamidomethyl (C)[14] | | Mascot |
| 2248.1277 | 2248.1423 | 0.0146 | 6 | 353 | 372 | SAQFEHTLLVTETGCEILTR | | | | | Mascot |

| | | | | | | | |
|---|---|---|---|---|---|---|---|
| 19 | E74-like factor 2a (ets domain transcription factor) [Danio rerio] | gi\|51870940 | 55871.4 | 4.95 | 10 | 38 | 0 |

**Peptide Information**

| Calc. Mass | Obsrv. Mass | ± da | ± ppm | Start Seq. | End Seq. | Sequence | Ion Score | C. I. % | Modification | Rank | Result Type |
|---|---|---|---|---|---|---|---|---|---|---|---|
| 845.4111 | 845.4667 | 0.0556 | 66 | 296 | 303 | ETPAASNR | | | | | Mascot |
| 916.5363 | 916.4868 | -0.0495 | -54 | 227 | 234 | AVSKLWGR | | | | | Mascot |
| 946.5567 | 946.4759 | -0.0808 | -85 | 222 | 230 | LVDSKAVSK | | | | | Mascot |
| 946.5567 | 946.4759 | -0.0808 | -85 | 222 | 230 | LVDSKAVSK | | | | | Mascot |
| 981.5073 | 981.5854 | 0.0781 | 80 | 204 | 211 | ETCPKYIK | | | | | Mascot |
| 1001.5122 | 1001.566 | 0.0538 | 54 | 295 | 303 | RETPAASNR | | | | | Mascot |
| 1355.6688 | 1355.6809 | 0.0121 | 9 | 495 | 506 | VEPPEVSVDQEK | | | | | Mascot |
| 1355.6688 | 1355.7896 | 0.1208 | 89 | 495 | 506 | VEPPEVSVDQEK | | | | | Mascot |
| 1386.6028 | 1386.6051 | 0.0023 | 2 | 168 | 179 | NCCDGSLDLVYK | | | Carbamidomethyl (C)[2] | | Mascot |
| 1542.7039 | 1542.7043 | 0.0004 | 0 | 168 | 180 | NCCDGSLDLVYKR | | | Carbamidomethyl (C)[2] | | Mascot |
| 1618.817 | 1618.717 | -0.1 | -62 | 120 | 133 | STEELLSEVEVEVR | | | | | Mascot |
| 2225.0867 | 2225.1245 | 0.0378 | 17 | 99 | 119 | TIEAAEALLHMDSPASLQGDR | | | | | Mascot |

| | | | | | | | |
|---|---|---|---|---|---|---|---|
| 20 | Heterogeneous nuclear ribonucleoprotein K [Danio rerio] | gi\|32451938 | 46356.1 | 8.16 | 10 | 37 | 0 |

Peptide Information

| Calc. Mass | Obsrv. Mass | ± da | ± ppm | Start Seq. | End Seq. | Sequence | Ion Score | C. I. % | Modification | Rank | Result Type |
|---|---|---|---|---|---|---|---|---|---|---|---|
| 893.4575 | 893.416 | -0.0415 | -46 | 148 | 155 | DSTQTTIK | | | | | Mascot |
| 916.4053 | 916.4868 | 0.0815 | 89 | 305 | 311 | GHMDERR | | | Oxidation (M)[3] | | Mascot |
| 994.4886 | 994.5829 | 0.0943 | 95 | 225 | 233 | RPMGGFSSR | | | | | Mascot |
| 1045.5823 | 1045.5667 | -0.0156 | -15 | 175 | 183 | AERVVQCIK | | | | | Mascot |
| 1053.4669 | 1053.576 | 0.1091 | 104 | 116 | 124 | GVDFDCELR | | | | | Mascot |
| 1053.4669 | 1053.576 | 0.1091 | 104 | 116 | 124 | GVDFDCELR | | | | | Mascot |
| 1618.6663 | 1618.717 | 0.0507 | 31 | 319 | 333 | NDRYGGSMNDGYGGR | | | | | Mascot |
| 1782.0623 | 1781.9781 | -0.0842 | -47 | 87 | 103 | ILSVSADIPTVAEILLK | | | | | Mascot |
| 1782.0623 | 1781.9781 | -0.0842 | -47 | 87 | 103 | ILSVSADIPTVAEILLK | | | | | Mascot |
| 1790.9316 | 1790.9106 | -0.021 | -12 | 38 | 52 | NSDEMVELRILLQSK | | | Oxidation (M)[5] | | Mascot |
| 2100.1807 | 2100.0022 | -0.1785 | -85 | 178 | 195 | VVQCIKTMLELIVEAPIK | | | Carbamidomethyl (C)[4], Oxidation (M)[8] | | Mascot |
| 2136.0576 | 2136.0969 | 0.0393 | 18 | 156 | 174 | LFQECCPQSTDRVVLVG GK | | | Carbamidomethyl (C)[5] | | Mascot |

21 PREDICTED: zinc finger protein 420-like [Danio rerio] gi|326675809 60606.6 8.42 13 37 0

Peptide Information

| Calc. Mass | Obsrv. Mass | ± da | ± ppm | Start Seq. | End Seq. | Sequence | Ion Score | C. I. % | Modification | Rank | Result Type |
|---|---|---|---|---|---|---|---|---|---|---|---|
| 855.4682 | 855.4388 | -0.0294 | -34 | 202 | 208 | VHKNETK | | | | | Mascot |
| 981.5251 | 981.5854 | 0.0603 | 61 | 386 | 394 | TYITAGDLK | | | | | Mascot |
| 1001.4972 | 1001.566 | 0.0688 | 69 | 2 | 10 | SDPEPCLIK | | | | | Mascot |
| 1109.6201 | 1109.5858 | -0.0343 | -31 | 386 | 395 | TYITAGDLKK | | | | | Mascot |
| 1386.6028 | 1386.6051 | 0.0023 | 2 | 347 | 357 | EKPYSCSVCEK | | | Carbamidomethyl (C)[6,9] | | Mascot |
| 1790.8677 | 1790.9106 | 0.0429 | 24 | 324 | 338 | CDHCGKTFLRPSELK | | | Carbamidomethyl (C)[1] | | Mascot |
| 1859.8868 | 1859.8964 | 0.0096 | 5 | 47 | 61 | QEETEEQIDLIVEER | | | | | Mascot |
| 1859.8868 | 1859.8964 | 0.0096 | 5 | 47 | 61 | QEETEEQIDLIVEER | | | | | Mascot |
| 1861.8142 | 1861.932 | 0.1178 | 63 | 259 | 273 | IHTGEKPYMCSHCNK | | | Carbamidomethyl (C)[10,13] | | Mascot |
| 1960.8939 | 1960.9794 | 0.0855 | 44 | 259 | 274 | IHTGEKPYMCSHCNKR | | | Carbamidomethyl (C)[10] | | Mascot |
| 2099.897 | 2100.0022 | 0.1052 | 50 | 237 | 254 | EYMCSECEKTFITSGDLK | | | Oxidation (M)[3] | | Mascot |
| 2248.1292 | 2248.1423 | 0.0131 | 6 | 161 | 179 | SFVQISHLNQHMIIHNGE K | | | Oxidation (M)[12] | | Mascot |
| 2264.0989 | 2264.1484 | 0.0495 | 22 | 302 | 320 | SFRQSAHLNEHMLIHNG EK | | | Oxidation (M)[12] | | Mascot |
| 2284.1187 | 2284.1797 | 0.061 | 27 | 445 | 463 | RPSNLNEHMMIHTGERP HK | | | | | Mascot |

22 PREDICTED: hypothetical protein LOC100538018 [Danio rerio] gi|326665968 5761.7 7.85 5 36 0

Peptide Information

| Calc. Mass | Obsrv. Mass | ± da | ± ppm | Start Seq. | End Seq. | Sequence | Ion Score | C. I. % | Modification | Rank | Result Type |
|---|---|---|---|---|---|---|---|---|---|---|---|
| 862.3901 | 862.4818 | 0.0917 | 106 | 9 | 15 | TELDNDR | | | | | Mascot |
| 914.4261 | 914.5472 | 0.1211 | 132 | 2 | 8 | MTHQNQR | | | | | Mascot |
| 1045.4667 | 1045.5667 | 0.1 | 96 | 1 | 8 | MMTHQNQR | | | | | Mascot |
| 1511.6254 | 1511.7045 | 0.0791 | 52 | 17 | 28 | ACEYTETNCHVK | | | Carbamidomethyl (C)[2,9] | | Mascot |
| 1582.6989 | 1582.7301 | 0.0312 | 20 | 16 | 28 | KACEYTETNCHVK | | | Carbamidomethyl (C)[3] | | Mascot |

| | | | | | | | |
|---|---|---|---|---|---|---|---|
| 23 | PREDICTED: hypothetical protein LOC100536324 [Danio rerio] | gi\|326675438 | 274951.1 | 9.13 | 23 | 36 | 0 |

Peptide Information

| Calc. Mass | Obsrv. Mass | ± da | ± ppm | Start Seq. | End Seq. | Sequence | Ion Score | C. I. % | Modification | Rank | Result Type |
|---|---|---|---|---|---|---|---|---|---|---|---|
| 845.4475 | 845.4667 | 0.0192 | 23 | 1116 | 1123 | EALAQASR | | | | | Mascot |
| 855.4094 | 855.4388 | 0.0294 | 34 | 103 | 109 | YVTEESK | | | | | Mascot |
| 870.5406 | 870.542 | 0.0014 | 2 | 1174 | 1181 | VLSPVTVR | | | | | Mascot |
| 994.5363 | 994.5829 | 0.0466 | 47 | 870 | 878 | VACHPGVRR | | | | | Mascot |
| 1001.5486 | 1001.566 | 0.0174 | 17 | 1116 | 1124 | EALAQASRR | | | | | Mascot |
| 1053.5146 | 1053.576 | 0.0614 | 58 | 397 | 404 | EAMDRYIR | | | | | Mascot |
| 1053.5146 | 1053.576 | 0.0614 | 58 | 397 | 404 | EAMDRYIR | | | | | Mascot |
| 1355.7794 | 1355.6809 | -0.0985 | -73 | 1145 | 1155 | VWLSTRDLPLR | | | | | Mascot |
| 1355.7834 | 1355.7896 | 0.0062 | 5 | 957 | 968 | FSKAAHFIPLPK | | | | | Mascot |
| 1363.6522 | 1363.6586 | 0.0064 | 5 | 1836 | 1847 | ELLDDGMANNKK | | | Oxidation (M)[7] | | Mascot |
| 1371.7015 | 1371.6747 | -0.0268 | -20 | 974 | 985 | ETAQAVVDHVFR | | | | | Mascot |
| 1371.7015 | 1371.6747 | -0.0268 | -20 | 974 | 985 | ETAQAVVDHVFR | | | | | Mascot |
| 1386.7587 | 1386.6051 | -0.1536 | -111 | 2252 | 2263 | EIVSRQELDAVK | | | | | Mascot |
| 1419.72 | 1419.7164 | -0.0036 | -3 | 2336 | 2346 | HTHDTQIRQQR | | | | | Mascot |
| 1571.7959 | 1571.7905 | -0.0054 | -3 | 2146 | 2158 | MDHDVRQLISSVR | | | Oxidation (M)[1] | | Mascot |
| 1582.7489 | 1582.7301 | -0.0188 | -12 | 1662 | 1676 | LGNSDSLCSRSSTQK | | | | | Mascot |
| 1618.8112 | 1618.717 | -0.0942 | -58 | 1357 | 1371 | GSPSEYGLPPNYIPK | | | | | Mascot |
| 1624.8363 | 1624.8014 | -0.0349 | -21 | 257 | 271 | TPLPTTSPEPMQLGR | | | | | Mascot |
| 1624.8363 | 1624.8014 | -0.0349 | -21 | 257 | 271 | TPLPTTSPEPMQLGR | | | | | Mascot |
| 1719.7391 | 1719.884 | 0.1449 | 84 | 241 | 256 | VSGASWMNEGPSNDPR | | | Oxidation (M)[7] | | Mascot |
| 1781.9432 | 1781.9781 | 0.0349 | 20 | 802 | 818 | LFDVPGGEATPEAILPR | | | | | Mascot |
| 1781.9432 | 1781.9781 | 0.0349 | 20 | 802 | 818 | LFDVPGGEATPEAILPR | | | | | Mascot |
| 1832.8848 | 1832.9417 | 0.0569 | 31 | 2264 | 2278 | EALLQCQTEYEHIQK | | | | | Mascot |

| | | | | | | | | | | | |
|---|---|---|---|---|---|---|---|---|---|---|---|
| 1859.8453 | 1859.8964 | 0.0511 | 27 | 240 | 256 | RVSGASWMNEGPSNDPR | | | | | Mascot |
| 1859.8453 | 1859.8964 | 0.0511 | 27 | 240 | 256 | RVSGASWMNEGPSNDPR | | | | | Mascot |
| 1971.1241 | 1971.0005 | -0.1236 | -63 | 1423 | 1440 | VVLMCQVVQRNALIGALK | | | Oxidation (M)[4] | | Mascot |
| 1971.1241 | 1971.0005 | -0.1236 | -63 | 1423 | 1440 | VVLMCQVVQRNALIGALK | | | Oxidation (M)[4] | | Mascot |
| 2211.0825 | 2211.1106 | 0.0281 | 13 | 1907 | 1925 | VEIFFPSMQALAQQMDVEK | | | | | Mascot |
| 2248.1311 | 2248.1423 | 0.0112 | 5 | 1795 | 1816 | MEANGAILGVSLIMDDVLSSGR | | | | | Mascot |
| 2264.126 | 2264.1484 | 0.0224 | 10 | 1795 | 1816 | MEANGAILGVSLIMDDVLSSGR | | | Oxidation (M)[1] | | Mascot |

| | | | | | | | | | |
|---|---|---|---|---|---|---|---|---|---|
| 24 | PREDICTED: oocyte zinc finger protein XlCOF6-like [Danio rerio] | gi\|326667152 | 41141.8 | 8.96 | 8 | 36 | 0 | 12 | 0 |

**Peptide Information**

| Calc. Mass | Obsrv. Mass | ± da | ± ppm | Start Seq. | End Seq. | Sequence | Ion Score | C. I. % | Modification | Rank | Result Type |
|---|---|---|---|---|---|---|---|---|---|---|---|
| 893.4474 | 893.416 | -0.0314 | -35 | 74 | 82 | SFGGNGNLK | | | | | Mascot |
| 907.4268 | 907.4691 | 0.0423 | 47 | 39 | 45 | EEEHHVK | | | | | Mascot |
| 1001.4178 | 1001.566 | 0.1482 | 148 | 66 | 73 | FTCTQCGK | | | Carbamidomethyl (C)[3,6] | | Mascot |
| 1109.5269 | 1109.5858 | 0.0589 | 53 | 302 | 310 | SSHLNQHMR | | | | | Mascot |
| 1556.7499 | 1556.7462 | -0.0037 | -2 | 298 | 310 | SFGRSSHLNQHMR | | | | | Mascot |
| 1582.7543 | 1582.7301 | -0.0242 | -15 | 242 | 254 | SFGRSSYLNQHMR | | | | | Mascot |
| 1624.8013 | 1624.8014 | 0.0001 | 0 | 270 | 282 | IFGRSSYLNQHMR | 12 | 0 | Oxidation (M)[12] | | Mascot |
| 1624.8013 | 1624.8014 | 0.0001 | 0 | 270 | 282 | IFGRSSYLNQHMR | | | Oxidation (M)[12] | | Mascot |
| 2211.0432 | 2211.1106 | 0.0674 | 30 | 227 | 245 | IHTGEKPFTCTQCGKSFGR | | | Carbamidomethyl (C)[10,13] | | Mascot |

| | | | | | | | | | |
|---|---|---|---|---|---|---|---|---|---|
| 25 | LOC794225 protein [Danio rerio] | gi\|111306309 | 57174.6 | 7.72 | 12 | 36 | 0 | | |

**Peptide Information**

| Calc. Mass | Obsrv. Mass | ± da | ± ppm | Start Seq. | End Seq. | Sequence | Ion Score | C. I. % | Modification | Rank | Result Type |
|---|---|---|---|---|---|---|---|---|---|---|---|
| 893.4219 | 893.416 | -0.0059 | -7 | 243 | 249 | MPEMTLR | | | Oxidation (M)[1] | | Mascot |
| 916.4846 | 916.4868 | 0.0022 | 2 | 151 | 157 | RELQENK | | | | | Mascot |
| 994.4509 | 994.5829 | 0.132 | 133 | 319 | 326 | QMQSEVEK | | | Oxidation (M)[2] | | Mascot |
| 1053.5398 | 1053.576 | 0.0362 | 34 | 21 | 29 | VHESLMPPK | | | Oxidation (M)[6] | | Mascot |
| 1053.5398 | 1053.576 | 0.0362 | 34 | 21 | 29 | VHESLMPPK | | | Oxidation (M)[6] | | Mascot |
| 1419.7009 | 1419.7164 | 0.0155 | 11 | 474 | 486 | THSTSTKMGNLSR | | | | | Mascot |
| 1511.7925 | 1511.7045 | -0.088 | -58 | 66 | 77 | AQIRDLEERPER | | | | | Mascot |
| 1556.9006 | 1556.7462 | -0.1544 | -99 | 303 | 316 | SVANQKVVLQLTEK | | | | | Mascot |
| 1571.7847 | 1571.7905 | 0.0058 | 4 | 481 | 494 | MGNLSRSAYTSLQK | | | Oxidation (M)[1] | | Mascot |

| | | | | | | | | | | | |
|---|---|---|---|---|---|---|---|---|---|---|---|
| 1587.7948 | 1587.7875 | -0.0073 | -5 | 223 | 236 | EMLQHVAAVAAEFR | | | Oxidation (M)[2] | | Mascot |
| 1624.7887 | 1624.8014 | 0.0127 | 8 | 78 | 90 | YQLKCDELEVQEK | | | | | Mascot |
| 1624.7887 | 1624.8014 | 0.0127 | 8 | 78 | 90 | YQLKCDELEVQEK | | | | | Mascot |
| 1961.0558 | 1960.9794 | -0.0764 | -39 | 419 | 437 | SQMMQKLLAVLDGAAALGK | | | Oxidation (M)[3] | | Mascot |
| 2282.9929 | 2283.1802 | 0.1873 | 82 | 42 | 61 | TGNMEETEQKNAEELTESDK | | | | | Mascot |

| | | | | | | | | |
|---|---|---|---|---|---|---|---|---|
| 26 | PREDICTED: uncharacterized protein C3orf63 [Danio rerio] | gi\|326679102 | 191042.7 | 6.38 | 18 | 35 | 0 | |

Peptide Information

| Calc. Mass | Obsrv. Mass | ± da | ± ppm | Start Seq. | End Seq. | Sequence | Ion Score | C. I. % | Modification | Rank | Result Type |
|---|---|---|---|---|---|---|---|---|---|---|---|
| 907.5836 | 907.4691 | -0.1145 | -126 | 581 | 587 | LIQLHKR | | | | | Mascot |
| 1053.6666 | 1053.576 | -0.0906 | -86 | 154 | 162 | VKIIENIPK | | | | | Mascot |
| 1053.6666 | 1053.576 | -0.0906 | -86 | 154 | 162 | VKIIENIPK | | | | | Mascot |
| 1220.5946 | 1220.5864 | -0.0082 | -7 | 1129 | 1138 | FLEEQGSPWK | | | | | Mascot |
| 1363.762 | 1363.6586 | -0.1034 | -76 | 843 | 854 | YVTPLPVQGYVK | | | | | Mascot |
| 1386.7263 | 1386.6051 | -0.1212 | -87 | 35 | 46 | LLSTFYLDATSR | | | | | Mascot |
| 1442.6177 | 1442.6982 | 0.0805 | 56 | 1616 | 1630 | DSALASSSSCSQGSR | | | | | Mascot |
| 1556.7745 | 1556.7462 | -0.0283 | -18 | 1656 | 1670 | GLLPLPGAPMGAMCR | | | Carbamidomethyl (C)[14], Oxidation (M)[10] | | Mascot |
| 1571.6473 | 1571.7905 | 0.1432 | 91 | 319 | 331 | CGMHCSLYEVMGK | | | Carbamidomethyl (C)[1,5] | | Mascot |
| 1587.6422 | 1587.7875 | 0.1453 | 92 | 319 | 331 | CGMHCSLYEVMGK | | | Carbamidomethyl (C)[1,5], Oxidation (M)[3] | | Mascot |
| 1624.8329 | 1624.8014 | -0.0315 | -19 | 950 | 963 | EVHLFQPEKSADPK | | | | | Mascot |
| 1624.9056 | 1624.8014 | -0.1042 | -64 | 945 | 958 | LGTVKEVHLFQPEK | | | | | Mascot |
| 1735.8683 | 1735.8815 | 0.0132 | 8 | 1032 | 1047 | SLGNIACNPLNYLESK | | | | | Mascot |
| 1735.953 | 1735.8815 | -0.0715 | -41 | 212 | 226 | HVLPYAVVSFQYKGK | | | | | Mascot |
| 2007.0481 | 2007.0828 | 0.0347 | 17 | 808 | 824 | LMEAQHIIYNSQPPLPR | | | | | Mascot |
| 2119.8855 | 2120.1094 | 0.2239 | 106 | 1671 | 1689 | GGHNNSMYNPRGGPPDMMR | | | Oxidation (M)[7,17] | | Mascot |
| 2135.8804 | 2136.0969 | 0.2165 | 101 | 1671 | 1689 | GGHNNSMYNPRGGPPDMMR | | | Oxidation (M)[7,17,18] | | Mascot |
| 2233.1387 | 2233.0947 | -0.044 | -20 | 28 | 46 | EFEDVVKLLSTFYLDATSR | | | | | Mascot |
| 2264.134 | 2264.1484 | 0.0144 | 6 | 1032 | 1051 | SLGNIACNPLNYLESKSQQK | | | Carbamidomethyl (C)[7] | | Mascot |
| 2284.0034 | 2284.1797 | 0.1763 | 77 | 714 | 732 | VDEEQVTQDFLEEQTACSR | | | Carbamidomethyl (C)[17] | | Mascot |
| 3337.4944 | 3337.7339 | 0.2395 | 72 | 1616 | 1649 | DSALASSSSCSQGSRTPVNSSSESQGGSQAPPAR | | | Carbamidomethyl (C)[10] | | Mascot |

| | | | | | | | | |
|---|---|---|---|---|---|---|---|---|
| 27 | plexin B2b long isoform [Danio rerio] | gi\|222354810 | 207361.6 | 6.44 | 19 | 35 | 0 | |

Peptide Information

| Calc. Mass | Obsrv. Mass | ± da | ± ppm | Start | End | Sequence | Ion | C. I. % | Modification | Rank | Result Type |
|---|---|---|---|---|---|---|---|---|---|---|---|

| | | | | Seq. | Seq. | | Score | | | | |
|---|---|---|---|---|---|---|---|---|---|---|---|
| 870.4791 | 870.542 | 0.0629 | 72 | 1735 | 1741 | TEHKLSR | | | | | Mascot |
| 907.4995 | 907.4691 | -0.0304 | -33 | 372 | 378 | DIVNRYK | | | | | Mascot |
| 914.473 | 914.5472 | 0.0742 | 81 | 908 | 914 | TVQFTYR | | | | | Mascot |
| 994.5502 | 994.5829 | 0.0327 | 33 | 479 | 487 | VPVQACHLK | | | | | Mascot |
| 1109.4714 | 1109.5858 | 0.1144 | 103 | 488 | 497 | QDCQSCVAQK | | | | | Mascot |
| 1442.7849 | 1442.6982 | -0.0867 | -60 | 1175 | 1187 | RDTGSDPLDLLIK | | | | | Mascot |
| 1511.6406 | 1511.7045 | 0.0639 | 42 | 498 | 509 | DPYCGWCVLEGR | | | Carbamidomethyl (C)[4,7] | | Mascot |
| 1519.8148 | 1519.7588 | -0.056 | -37 | 1114 | 1127 | SVITENSMIIVTGR | | | | | Mascot |
| 1542.8196 | 1542.7043 | -0.1153 | -75 | 1621 | 1633 | SMTKAITEIYLTR | | | Oxidation (M)[2] | | Mascot |
| 1582.7583 | 1582.7301 | -0.0282 | -18 | 523 | 535 | HGWLWSPNQQCVK | | | | | Mascot |
| 1587.8417 | 1587.7875 | -0.0542 | -34 | 685 | 698 | FENPDPVLIPVGYK | | | | | Mascot |
| 1618.851 | 1618.717 | -0.134 | -83 | 1381 | 1394 | TLLLGLMEDYVHSK | | | | | Mascot |
| 1624.8037 | 1624.8014 | -0.0023 | -1 | 1246 | 1258 | HQLENLEESVRDR | | | | | Mascot |
| 1624.8152 | 1624.8014 | -0.0138 | -8 | 377 | 391 | YKCGADFLPSPLASR | | | | | Mascot |
| 1719.8041 | 1719.884 | 0.0799 | 46 | 72 | 87 | DAKGCTPPVSTCQDLK | | | Carbamidomethyl (C)[5] | | Mascot |
| 1781.9102 | 1781.9781 | 0.0679 | 38 | 872 | 888 | YSVSTSVICEIGPASLR | | | | | Mascot |
| 1781.9102 | 1781.9781 | 0.0679 | 38 | 872 | 888 | YSVSTSVICEIGPASLR | | | | | Mascot |
| 1838.9316 | 1838.8909 | -0.0407 | -22 | 872 | 888 | YSVSTSVICEIGPASLR | | | Carbamidomethyl (C)[9] | | Mascot |
| 2066.0779 | 2066.0371 | -0.0408 | -20 | 1789 | 1805 | EHTDKLNTQVALHQLYR | | | | | Mascot |
| 2136.1118 | 2136.0969 | -0.0149 | -7 | 329 | 347 | NINQKLLAIVTACYTDNG K | | | Carbamidomethyl (C)[13] | | Mascot |
| 3337.5974 | 3337.7339 | 0.1365 | 41 | 1086 | 1113 | TFVQLDNLQMELTPFAYY PNPTFDELSK | | | Oxidation (M)[10] | | Mascot |

28 PREDICTED: protein-glutamine gamma-glutamyltransferase 2-like [Danio rerio] gi|326668941 74951.8 5.69 11 35 0

**Peptide Information**

| Calc. Mass | Obsrv. Mass | ± da | ± ppm | Start Seq. | End Seq. | Sequence | Ion Score | C. I. % | Modification | Rank | Result Type |
|---|---|---|---|---|---|---|---|---|---|---|---|
| 862.4338 | 862.4818 | 0.048 | 56 | 16 | 22 | VDLECVK | | | Carbamidomethyl (C)[5] | | Mascot |
| 994.4774 | 994.5829 | 0.1055 | 106 | 196 | 203 | CNPVYVSR | | | Carbamidomethyl (C)[1] | | Mascot |
| 1045.5425 | 1045.5667 | 0.0242 | 23 | 437 | 445 | AVYTEAVHR | | | | | Mascot |
| 1571.6425 | 1571.7905 | 0.148 | 94 | 1 | 15 | MCSSSSSNTPDQGVR | | | Oxidation (M)[1] | | Mascot |
| 1582.8013 | 1582.7301 | -0.0712 | -45 | 524 | 537 | ALSFTLPYSAYGHR | | | | | Mascot |
| 1587.7723 | 1587.7875 | 0.0152 | 10 | 138 | 150 | QEYIMSEQGVLYK | | | | | Mascot |
| 1618.8403 | 1618.717 | -0.1233 | -76 | 538 | 551 | MLENNCIKVSAIAR | | | Carbamidomethyl (C)[6] | | Mascot |
| 1624.8541 | 1624.8014 | -0.0527 | -32 | 413 | 427 | AVGSSSRLDITDLYK | | | | | Mascot |

| | | | | | | | | | | | |
|---|---|---|---|---|---|---|---|---|---|---|---|
| 1624.8541 | 1624.8014 | -0.0527 | -32 | 413 | 427 | AVGSSSRLDITDLYK | | | | | Mascot |
| 1960.923 | 1960.9794 | 0.0564 | 29 | 255 | 270 | YGQCWVFAAVMCTVLR | | | Carbamidomethyl (C)[4,12] | | Mascot |
| 2211.0017 | 2211.1106 | 0.1089 | 49 | 2 | 22 | CSSSSSNTPDQGVRVDL ECVK | | | | | Mascot |
| 2248.0598 | 2248.1423 | 0.0825 | 37 | 271 | 290 | CLGIPCRVVTNYQSAHDT DR | | | | | Mascot |

29 novel protein similar to vertebrate kinesin family member 5A (KIF5A) [Danio rerio] gi|169158591 96952.9 5.41 13 35 0 5 0

Peptide Information

| Calc. Mass | Obsrv. Mass | ± da | ± ppm | Start Seq. | End Seq. | Sequence | Ion Score | C. I. % | Modification | Rank | Result Type |
|---|---|---|---|---|---|---|---|---|---|---|---|
| 882.5117 | 882.5694 | 0.0577 | 65 | 596 | 602 | LYISKMK | | | | | Mascot |
| 907.4917 | 907.4691 | -0.0226 | -25 | 603 | 610 | SEVKSMVK | | | | | Mascot |
| 1001.4832 | 1001.566 | 0.0828 | 83 | 547 | 554 | EMANHQKK | | | Oxidation (M)[2] | | Mascot |
| 1053.6051 | 1053.576 | -0.0291 | -28 | 640 | 648 | ISQHEAKIK | | | | | Mascot |
| 1053.6051 | 1053.576 | -0.0291 | -28 | 640 | 648 | ISQHEAKIK | | | | | Mascot |
| 1386.7046 | 1386.6051 | -0.0995 | -72 | 678 | 689 | ISAQEKVHAMEK | | | Oxidation (M)[10] | | Mascot |
| 1571.8275 | 1571.7905 | -0.037 | -24 | 566 | 580 | DLTEIGIAVGNNDIK | | | | | Mascot |
| 1624.7314 | 1624.8014 | 0.07 | 43 | 1 | 14 | MADPAECTIKVMCR | 7 | 0 | Carbamidomethyl (C)[13] | | Mascot |
| 1624.7314 | 1624.8014 | 0.07 | 43 | 1 | 14 | MADPAECTIKVMCR | | | Carbamidomethyl (C)[7] | | Mascot |
| 1790.8741 | 1790.9106 | 0.0365 | 20 | 327 | 341 | NTVCVNVELTAEQWK | | | Carbamidomethyl (C)[4] | | Mascot |
| 1838.8674 | 1838.8909 | 0.0235 | 13 | 188 | 203 | SNRHVAVTNMNEHSSR | | | | | Mascot |
| 1861.9476 | 1861.932 | -0.0156 | -8 | 327 | 342 | NTVCVNVELTAEQWKK | | | | | Mascot |
| 1970.9198 | 1971.0005 | 0.0807 | 41 | 296 | 313 | TTIVICCSPSAFNEAETK | | | Carbamidomethyl (C)[6] | | Mascot |
| 1970.9198 | 1971.0005 | 0.0807 | 41 | 296 | 313 | TTIVICCSPSAFNEAETK | | | Carbamidomethyl (C)[6] | | Mascot |
| 2264.1558 | 2264.1484 | -0.0074 | -3 | 581 | 600 | HEGSGLIDEEFTVARLYIS K | | | | | Mascot |
| 2284.1819 | 2284.1797 | -0.0022 | -1 | 257 | 278 | SLSSLGNVISALAEGSTY VPYR | | | | | Mascot |

30 novel protein [Danio rerio] gi|148726576 48128.2 9.12 9 35 0

Peptide Information

| Calc. Mass | Obsrv. Mass | ± da | ± ppm | Start Seq. | End Seq. | Sequence | Ion Score | C. I. % | Modification | Rank | Result Type |
|---|---|---|---|---|---|---|---|---|---|---|---|
| 893.509 | 893.416 | -0.093 | -104 | 154 | 161 | KLGSDVFK | | | | | Mascot |
| 914.5781 | 914.5472 | -0.0309 | -34 | 13 | 20 | ALVKSLQR | | | | | Mascot |
| 946.5064 | 946.4759 | -0.0305 | -32 | 5 | 12 | VSVDNRTR | | | | | Mascot |
| 946.5064 | 946.4759 | -0.0305 | -32 | 5 | 12 | VSVDNRTR | | | | | Mascot |
| 1045.6 | 1045.5667 | -0.0333 | -32 | 17 | 25 | SLQRVTDVK | | | | | Mascot |
| 1220.6382 | 1220.5864 | -0.0518 | -42 | 374 | 383 | LDYLQAEGRR | | | | | Mascot |

| | | | | | | | | | | | |
|---|---|---|---|---|---|---|---|---|---|---|---|
| 1511.8944 | 1511.7045 | -0.1899 | -126 | 404 | 416 | GIVQKLAEWVQLK | | | | | Mascot |
| 1751.8698 | 1751.9026 | 0.0328 | 19 | 309 | 324 | YETATKTSASTYTSLK | | | | | Mascot |
| 1960.9269 | 1960.9794 | 0.0525 | 27 | 237 | 252 | SHYLGSCQHKMWQAIR | | | Oxidation (M)[11] | | Mascot |
| 2152.0896 | 2152.093 | 0.0034 | 2 | 409 | 426 | LAEWVQLKADMYDGLPFR | | | | | Mascot |

| | | | | | | | | | |
|---|---|---|---|---|---|---|---|---|---|
| 31 | PREDICTED: zinc finger protein 180-like [Danio rerio] | gi\|292613218 | 41233.8 | 9.06 | 8 | 35 | 0 | 12 | 0 |

Peptide Information

| Calc. Mass | Obsrv. Mass | ± da | ± ppm | Start Seq. | End Seq. | Sequence | Ion Score | C. I. % | Modification | Rank | Result Type |
|---|---|---|---|---|---|---|---|---|---|---|---|
| 893.4474 | 893.416 | -0.0314 | -35 | 74 | 82 | SFGGNGNLK | | | | | Mascot |
| 907.4268 | 907.4691 | 0.0423 | 47 | 39 | 45 | EEEHHVK | | | | | Mascot |
| 1001.4178 | 1001.566 | 0.1482 | 148 | 66 | 73 | FTCTQCGK | | | Carbamidomethyl (C)[3,6] | | Mascot |
| 1109.5269 | 1109.5858 | 0.0589 | 53 | 302 | 310 | SSHLNQHMR | | | | | Mascot |
| 1556.7499 | 1556.7462 | -0.0037 | -2 | 298 | 310 | SFGRSSHLNQHMR | | | | | Mascot |
| 1582.7543 | 1582.7301 | -0.0242 | -15 | 242 | 254 | SFGRSSYLNQHMR | | | | | Mascot |
| 1624.8013 | 1624.8014 | 0.0001 | 0 | 270 | 282 | IFGRSSYLNQHMR | 12 | 0 | Oxidation (M)[12] | | Mascot |
| 1624.8013 | 1624.8014 | 0.0001 | 0 | 270 | 282 | IFGRSSYLNQHMR | | | Oxidation (M)[12] | | Mascot |
| 2211.0432 | 2211.1106 | 0.0674 | 30 | 227 | 245 | IHTGEKPFTCTQCGKSFGR | | | Carbamidomethyl (C)[10,13] | | Mascot |

| | | | | | | | |
|---|---|---|---|---|---|---|---|
| 32 | RecName: Full=Tudor domain-containing protein 7B | gi\|334351210 | 121757 | 8.62 | 14 | 35 | 0 |

Peptide Information

| Calc. Mass | Obsrv. Mass | ± da | ± ppm | Start Seq. | End Seq. | Sequence | Ion Score | C. I. % | Modification | Rank | Result Type |
|---|---|---|---|---|---|---|---|---|---|---|---|
| 864.4495 | 864.4891 | 0.0396 | 46 | 939 | 945 | MEEKALK | | | Oxidation (M)[1] | | Mascot |
| 870.4679 | 870.542 | 0.0741 | 85 | 984 | 991 | HELVSGTK | | | | | Mascot |
| 914.5305 | 914.5472 | 0.0167 | 18 | 867 | 875 | KTSPPLGSK | | | | | Mascot |
| 916.441 | 916.4868 | 0.0458 | 50 | 465 | 472 | YIGEGYSK | | | | | Mascot |
| 946.5567 | 946.4759 | -0.0808 | -85 | 578 | 586 | KLESIATGK | | | | | Mascot |
| 946.5567 | 946.4759 | -0.0808 | -85 | 578 | 586 | KLESIATGK | | | | | Mascot |
| 1109.5811 | 1109.5858 | 0.0047 | 4 | 122 | 130 | TSLRQPDHR | | | | | Mascot |
| 1161.5609 | 1161.5631 | 0.0022 | 2 | 367 | 375 | LYQDTYKCK | | | | | Mascot |
| 1419.7743 | 1419.7164 | -0.0579 | -41 | 956 | 966 | VENNWYRVLVK | | | | | Mascot |
| 1519.8114 | 1519.7588 | -0.0526 | -35 | 404 | 416 | AILYAKVVEDENR | | | | | Mascot |
| 1582.7782 | 1582.7301 | -0.0481 | -30 | 1054 | 1066 | VMIYLVDTSQEER | | | | | Mascot |
| 1751.8281 | 1751.9026 | 0.0745 | 43 | 949 | 962 | NQVCAAKVENNWYR | | | Carbamidomethyl (C)[4] | | Mascot |
| 2152.1497 | 2152.093 | -0.0567 | -26 | 13 | 32 | AVLQSSKSGLSLSDLQVEYK | | | | | Mascot |

| | | | | | | | | | | | |
|---|---|---|---|---|---|---|---|---|---|---|---|
| 2225.1013 | 2225.1245 | 0.0232 | 10 | 925 | 942 | LVVLMGEMILHYNKMEEK | | | Oxidation (M)[5,8,15] | | Mascot |
| 2283.0308 | 2283.1802 | 0.1494 | 65 | 1067 | 1085 | DIWLHDIMAEFTDEMTKT A | | | Oxidation (M)[8] | | Mascot |

| | | | | | | | | |
|---|---|---|---|---|---|---|---|---|
| 33 | PREDICTED: neuroblast differentiation-associated protein AHNAK [Danio rerio] | gi\|292618776 | 360101 | 9.01 | 26 | 34 | 0 | |

Peptide Information

| Calc. Mass | Obsrv. Mass | ± da | ± ppm | Start Seq. | End Seq. | Sequence | Ion Score | C. I. % | Modification | Rank | Result Type |
|---|---|---|---|---|---|---|---|---|---|---|---|
| 845.4727 | 845.4667 | -0.006 | -7 | 584 | 591 | SPDASKLK | | | | | Mascot |
| 864.4681 | 864.4891 | 0.021 | 24 | 3301 | 3307 | MNVLKLM | | | Oxidation (M)[1] | | Mascot |
| 914.5822 | 914.5472 | -0.035 | -38 | 2414 | 2421 | LPKFGKPK | | | | | Mascot |
| 946.572 | 946.4759 | -0.0961 | -102 | 2520 | 2528 | FGLSGPKLK | | | | | Mascot |
| 946.572 | 946.4759 | -0.0961 | -102 | 1135 | 1143 | FGLSGPKLK | | | | | Mascot |
| 1109.533 | 1109.5858 | 0.0528 | 48 | 2227 | 2236 | MPSLNMSGLK | | | Oxidation (M)[1,6] | | Mascot |
| 1176.6107 | 1176.6029 | -0.0078 | -7 | 472 | 482 | TGLDSTDINIK | | | | | Mascot |
| 1363.6926 | 1363.6586 | -0.034 | -25 | 2319 | 2330 | TPEMDLSAPKFK | | | | | Mascot |
| 1419.6937 | 1419.7164 | 0.0227 | 16 | 2084 | 2095 | TPDMDLSPPRFK | | | Oxidation (M)[4] | | Mascot |
| 1542.8599 | 1542.7043 | -0.1556 | -101 | 132 | 145 | SKVNIATLLQNADR | | | | | Mascot |
| 1556.6608 | 1556.7462 | 0.0854 | 55 | 146 | 158 | MCLAPSETEFDEK | | | Carbamidomethyl (C)[2] | | Mascot |
| 1582.827 | 1582.7301 | -0.0969 | -61 | 3073 | 3086 | MHANNFAIPRGNIK | | | | | Mascot |
| 1735.7744 | 1735.8815 | 0.1071 | 62 | 3246 | 3258 | RHYTTDDICYTYK | | | Carbamidomethyl (C)[9] | | Mascot |
| 1735.7744 | 1735.8815 | 0.1071 | 62 | 3246 | 3258 | RHYTTDDICYTYK | | | Carbamidomethyl (C)[9] | | Mascot |
| 1781.8119 | 1781.9781 | 0.1662 | 93 | 1581 | 1596 | TDLTVPDMDMPSGKMK | | | Oxidation (M)[8] | | Mascot |
| 1781.8119 | 1781.9781 | 0.1662 | 93 | 2279 | 2294 | TDLTVPDMDMPSGKMK | | | Oxidation (M)[8] | | Mascot |
| 1790.8524 | 1790.9106 | 0.0582 | 32 | 3209 | 3224 | TENLPVRNSNMGMSPK | | | Oxidation (M)[11] | | Mascot |
| 1832.9324 | 1832.9417 | 0.0093 | 5 | 2794 | 2810 | GHKPDITAPDMNVNVPK | | | | | Mascot |
| 1859.8844 | 1859.8964 | 0.012 | 6 | 2393 | 2409 | GNKPDITGPDMDFDIPK | | | | | Mascot |
| 1859.9904 | 1859.8964 | -0.094 | -51 | 1287 | 1303 | LNMAKPKMPSLNMSGLK | | | | | Mascot |
| 1960.9321 | 1960.9794 | 0.0473 | 24 | 2077 | 2093 | TPDYDLKTPDMDLSPPR | | | | | Mascot |
| 1970.9232 | 1971.0005 | 0.0773 | 39 | 333 | 351 | AALSGPKMMTSEMDVST SK | | | | | Mascot |
| 1970.9232 | 1971.0005 | 0.0773 | 39 | 333 | 351 | AALSGPKMMTSEMDVST SK | | | | | Mascot |
| 1992.9695 | 1993.1134 | 0.1439 | 72 | 1948 | 1965 | GGIDTPDMDLTLRQADFK | | | | | Mascot |
| 1992.9695 | 1993.1134 | 0.1439 | 72 | 1948 | 1965 | GGIDTPDMDLTLRQADFK | | | | | Mascot |
| 2065.9602 | 2066.0371 | 0.0769 | 37 | 2493 | 2511 | GMTTDLTPPDINMPSGK MK | | | Oxidation (M)[2,13] | | Mascot |
| 2120.0803 | 2120.1094 | 0.0291 | 14 | 2258 | 2278 | LNAGMSSPDIDIHGPGLK GPK | | | Oxidation (M)[5] | | Mascot |
| 2135.9663 | 2136.0969 | 0.1306 | 61 | 2689 | 2708 | GPHMDADIDHPDLNLSA SSK | | | Oxidation (M)[4] | | Mascot |

| | | | | | | | | | | | | |
|---|---|---|---|---|---|---|---|---|---|---|---|---|
| 2225.0464 | 2225.1245 | 0.0781 | 35 | 1248 | 1267 | IKGGIDTPDMDMTLPQADFK | | | Oxidation (M)[10,12] | | Mascot |
| 2283.1648 | 2283.1802 | 0.0154 | 7 | 2583 | 2603 | KPNIDMNAPDLDINSPSGKLK | | | Oxidation (M)[6] | | Mascot |
| 2284.1853 | 2284.1797 | -0.0056 | -2 | 2630 | 2650 | TPQIKGGIDTPDMDLNLLQAK | | | Oxidation (M)[13] | | Mascot |

34 PREDICTED: zinc finger protein 502-like [Danio rerio] gi|326667257 34114.4 9.49 11 34 0

**Peptide Information**

| Calc. Mass | Obsrv. Mass | ± da | ± ppm | Start Seq. | End Seq. | Sequence | Ion Score | C. I. % | Modification | Rank | Result Type |
|---|---|---|---|---|---|---|---|---|---|---|---|
| 842.4553 | 842.5123 | 0.057 | 68 | 285 | 291 | SLINHMK | | | | | Mascot |
| 862.4702 | 862.4818 | 0.0116 | 13 | 2 | 8 | ENLMVLK | | | Oxidation (M)[4] | | Mascot |
| 916.5071 | 916.4868 | -0.0203 | -22 | 36 | 43 | TSSRGRPR | | | | | Mascot |
| 994.5237 | 994.5829 | 0.0592 | 60 | 252 | 260 | SLTCKDSIK | | | | | Mascot |
| 1001.5275 | 1001.566 | 0.0385 | 38 | 265 | 272 | IHSGERFR | | | | | Mascot |
| 1197.5793 | 1197.5883 | 0.009 | 8 | 146 | 155 | QNGNLEVHMR | | | | | Mascot |
| 1735.8254 | 1735.8815 | 0.0561 | 32 | 72 | 86 | VHTGEKPYTCKQCGK | | | Carbamidomethyl (C)[10] | | Mascot |
| 1735.8254 | 1735.8815 | 0.0561 | 32 | 72 | 86 | VHTGEKPYTCKQCGK | | | Carbamidomethyl (C)[10] | | Mascot |
| 1781.8058 | 1781.9781 | 0.1723 | 97 | 156 | 170 | THTGERSFICTQCGK | | | Carbamidomethyl (C)[10,13] | | Mascot |
| 1781.8058 | 1781.9781 | 0.1723 | 97 | 156 | 170 | THTGERSFICTQCGK | | | Carbamidomethyl (C)[10,13] | | Mascot |
| 1832.8748 | 1832.9417 | 0.0669 | 37 | 112 | 127 | YGKSFYTTGNLAAHMR | | | Oxidation (M)[15] | | Mascot |
| 1970.9575 | 1971.0005 | 0.043 | 22 | 83 | 99 | QCGKSFYTIGNLTVHMR | | | Oxidation (M)[16] | | Mascot |
| 1970.9575 | 1971.0005 | 0.043 | 22 | 83 | 99 | QCGKSFYTIGNLTVHMR | | | Oxidation (M)[16] | | Mascot |
| 2099.9524 | 2100.0022 | 0.0498 | 24 | 128 | 145 | IHTGEEPYSCLQCGKSYK | | | Carbamidomethyl (C)[10] | | Mascot |

35 PREDICTED: e3 ubiquitin-protein ligase HERC2, partial [Danio rerio] gi|326668760 399265.3 6.21 26 34 0

**Peptide Information**

| Calc. Mass | Obsrv. Mass | ± da | ± ppm | Start Seq. | End Seq. | Sequence | Ion Score | C. I. % | Modification | Rank | Result Type |
|---|---|---|---|---|---|---|---|---|---|---|---|
| 842.4267 | 842.5123 | 0.0856 | 102 | 1467 | 1473 | WGSVTHR | | | | | Mascot |
| 855.4067 | 855.4388 | 0.0321 | 38 | 301 | 307 | THQEQGR | | | | | Mascot |
| 862.4927 | 862.4818 | -0.0109 | -13 | 676 | 682 | ISTRVMR | | | | | Mascot |
| 907.4605 | 907.4691 | 0.0086 | 9 | 1578 | 1584 | RHHSSQR | | | | | Mascot |
| 994.4622 | 994.5829 | 0.1207 | 121 | 895 | 902 | TERSQDMK | | | | | Mascot |
| 1045.5524 | 1045.5667 | 0.0143 | 14 | 806 | 815 | ILVESGANDK | | | | | Mascot |
| 1053.5648 | 1053.576 | 0.0112 | 11 | 533 | 541 | MLLEVTFGK | | | Oxidation (M)[1] | | Mascot |
| 1053.5648 | 1053.576 | 0.0112 | 11 | 533 | 541 | MLLEVTFGK | 4 | 0 | Oxidation (M)[1] | | Mascot |
| 1176.5864 | 1176.6029 | 0.0165 | 14 | 1378 | 1387 | ENIQVGMMVR | | | | | Mascot |

| | | | | | | | | | | | |
|---|---|---|---|---|---|---|---|---|---|---|---|
| 1179.6343 | 1179.666 | 0.0317 | 27 | 3592 | 3600 | LKYAIHFCK | | | Carbamidomethyl (C)[8] | | Mascot |
| 1179.6343 | 1179.666 | 0.0317 | 27 | 3592 | 3600 | LKYAIHFCK | | | Carbamidomethyl (C)[8] | | Mascot |
| 1197.5316 | 1197.5883 | 0.0567 | 47 | 1822 | 1832 | VYACGEATNGR | | | Carbamidomethyl (C)[4] | | Mascot |
| 1355.6525 | 1355.6809 | 0.0284 | 21 | 2269 | 2279 | VSPHPWMDSKR | | | Oxidation (M)[7] | | Mascot |
| 1355.6525 | 1355.7896 | 0.1371 | 101 | 2269 | 2279 | VSPHPWMDSKR | | | Oxidation (M)[7] | | Mascot |
| 1419.7451 | 1419.7164 | -0.0287 | -20 | 3222 | 3233 | DRQHGPVVELNR | | | | | Mascot |
| 1511.7383 | 1511.7045 | -0.0338 | -22 | 2462 | 2474 | RHDPLTIMDGANR | | | Oxidation (M)[8] | | Mascot |
| 1542.641 | 1542.7043 | 0.0633 | 41 | 370 | 382 | SESADLEECKMER | | | Oxidation (M)[11] | | Mascot |
| 1556.908 | 1556.7462 | -0.1618 | -104 | 2998 | 3012 | VPMKIDSLTGLGVVK | | | | | Mascot |
| 1582.9026 | 1582.7301 | -0.1725 | -109 | 1631 | 1643 | LELFPDMLVHKLK | | | | | Mascot |
| 1587.8345 | 1587.7875 | -0.047 | -30 | 1894 | 1906 | MNCDKPRLIEALK | | | Carbamidomethyl (C)[3] | | Mascot |
| 1624.917 | 1624.8014 | -0.1156 | -71 | 2701 | 2714 | RSPLPRPFLDEVAK | | | | | Mascot |
| 1624.917 | 1624.8014 | -0.1156 | -71 | 2701 | 2714 | RSPLPRPFLDEVAK | | | | | Mascot |
| 1838.8715 | 1838.8909 | 0.0194 | 11 | 1579 | 1593 | HHSSQRGMLIDEWSR | | | | | Mascot |
| 1859.7034 | 1859.8964 | 0.193 | 104 | 1534 | 1548 | SCDDFDFCESCFKTR | | | Carbamidomethyl (C)[2] | | Mascot |
| 1859.7034 | 1859.8964 | 0.193 | 104 | 1534 | 1548 | SCDDFDFCESCFKTR | | | Carbamidomethyl (C)[2] | | Mascot |
| 1861.6648 | 1861.932 | 0.2672 | 144 | 1532 | 1546 | CRSCDDFDFCESCFK | | | Carbamidomethyl (C)[1] | | Mascot |
| 1961.0273 | 1960.9794 | -0.0479 | -24 | 2203 | 2219 | QQALSHVLSSLQIMYAR | | | Oxidation (M)[14] | | Mascot |
| 2211.1665 | 2211.1106 | -0.0559 | -25 | 1261 | 1280 | TSPVPPAPIVLQLMEMGFQR | | | | | Mascot |
| 2233.1255 | 2233.0947 | -0.0308 | -14 | 3327 | 3345 | DCFLLNPAAKSPLHISMFR | | | Carbamidomethyl (C)[2], Oxidation (M)[17] | | Mascot |
| 2264.0864 | 2264.1484 | 0.062 | 27 | 380 | 400 | MEREGTNEEPPAPLSPAPVDK | | | | | Mascot |
| 2284.2158 | 2284.1797 | -0.0361 | -16 | 533 | 551 | MLLEVTFGKLYAWSIQNVR | | | Oxidation (M)[1] | | Mascot |

36 PREDICTED: inactive ubiquitin carboxyl-terminal hydrolase 53-like [Danio rerio] gi|326663976 107397.4 8.94 14 34 0

**Peptide Information**

| Calc. Mass | Obsrv. Mass | ± da | ± ppm | Start Seq. | End Seq. | Sequence | Ion Score | C. I. % | Modification | Rank | Result Type |
|---|---|---|---|---|---|---|---|---|---|---|---|
| 870.4944 | 870.542 | 0.0476 | 55 | 816 | 822 | TPPFRPR | | | | | Mascot |
| 893.3781 | 893.416 | 0.0379 | 42 | 947 | 953 | MENQSER | | | | | Mascot |
| 916.4886 | 916.4868 | -0.0018 | -2 | 67 | 74 | HALAETFK | | | | | Mascot |
| 994.4622 | 994.5829 | 0.1207 | 121 | 377 | 385 | TPMQSSTSR | | | | | Mascot |
| 1053.5323 | 1053.576 | 0.0437 | 41 | 435 | 444 | LSSPPENGPR | | | | | Mascot |
| 1053.5323 | 1053.576 | 0.0437 | 41 | 435 | 444 | LSSPPENGPR | | | | | Mascot |
| 1109.5004 | 1109.5858 | 0.0854 | 77 | 477 | 487 | SNQGMASGVSR | | | Oxidation (M)[5] | | Mascot |
| 1220.6211 | 1220.5864 | -0.0347 | -28 | 268 | 277 | HYLAFAFHSK | | | | | Mascot |

| Calc. Mass | Obsrv. Mass | ± da | ± ppm | Start Seq. | End Seq. | Sequence | Ion Score | C. I. % | Modification | Rank | Result Type |
|---|---|---|---|---|---|---|---|---|---|---|---|
| 1355.6887 | 1355.6809 | -0.0078 | -6 | 531 | 540 | RQQQQQQQQR | | | | | Mascot |
| 1355.6887 | 1355.7896 | 0.1009 | 74 | 531 | 540 | RQQQQQQQQR | | | | | Mascot |
| 1419.6831 | 1419.7164 | 0.0333 | 23 | 190 | 202 | NCPSNCGQSIKIR | | | | | Mascot |
| 1511.7775 | 1511.7045 | -0.073 | -48 | 15 | 28 | SYQPGSMISLALTK | | | Oxidation (M)[7] | | Mascot |
| 1571.7369 | 1571.7905 | 0.0536 | 34 | 560 | 572 | QKGLMTIYEDDSR | | | Oxidation (M)[5] | | Mascot |
| 1832.8998 | 1832.9417 | 0.0419 | 23 | 461 | 476 | TEHSSVHPRVSPQESR | | | | | Mascot |
| 1859.9697 | 1859.8964 | -0.0733 | -39 | 918 | 932 | YHHQRPLVLSMPEPR | | | | | Mascot |
| 1859.9697 | 1859.8964 | -0.0733 | -39 | 918 | 932 | YHHQRPLVLSMPEPR | | | | | Mascot |
| 2248.0518 | 2248.1423 | 0.0905 | 40 | 786 | 806 | SSYPWEESAIQSGPSIAS HSK | | | | | Mascot |

| | | | | | | | |
|---|---|---|---|---|---|---|---|
| 37 | truncated RAF1 [Danio rerio] | gi\|41224402 | 32290.4 | 9.5 | 8 | 33 | 0 |

**Peptide Information**

| Calc. Mass | Obsrv. Mass | ± da | ± ppm | Start Seq. | End Seq. | Sequence | Ion Score | C. I. % | Modification | Rank | Result Type |
|---|---|---|---|---|---|---|---|---|---|---|---|
| 845.3934 | 845.4667 | 0.0733 | 87 | 33 | 40 | SPAPCQSR | | | | | Mascot |
| 882.4791 | 882.5694 | 0.0903 | 102 | 215 | 222 | IPSSSVHR | | | | | Mascot |
| 946.5567 | 946.4759 | -0.0808 | -85 | 10 | 18 | TVTNGLLTK | | | | | Mascot |
| 946.5567 | 946.4759 | -0.0808 | -85 | 10 | 18 | TVTNGLLTK | | | | | Mascot |
| 1001.5526 | 1001.566 | 0.0134 | 13 | 53 | 60 | VFLPNQQR | | | | | Mascot |
| 1045.452 | 1045.5667 | 0.1147 | 110 | 165 | 172 | FHEHCSTK | | | Carbamidomethyl (C)[5] | | Mascot |
| 1371.7855 | 1371.6747 | -0.1108 | -81 | 50 | 60 | TIRVFLPNQQR | | | | | Mascot |
| 1371.7855 | 1371.6747 | -0.1108 | -81 | 50 | 60 | TIRVFLPNQQR | | | | | Mascot |
| 1556.7843 | 1556.7462 | -0.0381 | -24 | 19 | 32 | DSVFESSYVSPVIK | | | | | Mascot |
| 2211.0908 | 2211.1106 | 0.0198 | 9 | 81 | 99 | VRGLQPECCAVFSLHPG QR | | | Carbamidomethyl (C)[8,9] | | Mascot |

| | | | | | | | |
|---|---|---|---|---|---|---|---|
| 38 | PREDICTED: GDH/6PGL endoplasmic bifunctional protein-like [Danio rerio] | gi\|326668807 | 89431.1 | 6.59 | 11 | 33 | 0 |

**Peptide Information**

| Calc. Mass | Obsrv. Mass | ± da | ± ppm | Start Seq. | End Seq. | Sequence | Ion Score | C. I. % | Modification | Rank | Result Type |
|---|---|---|---|---|---|---|---|---|---|---|---|
| 845.4515 | 845.4667 | 0.0152 | 18 | 210 | 216 | IDHYLGK | | | | | Mascot |
| 855.373 | 855.4388 | 0.0658 | 77 | 121 | 127 | TAEDYEK | | | | | Mascot |
| 1179.6117 | 1179.666 | 0.0543 | 46 | 369 | 378 | ALDERVSYAR | | | | | Mascot |
| 1179.6117 | 1179.666 | 0.0543 | 46 | 369 | 378 | ALDERVSYAR | | | | | Mascot |
| 1371.8093 | 1371.6747 | -0.1346 | -98 | 245 | 256 | IEIVLKETLDAK | | | | | Mascot |
| 1371.8093 | 1371.6747 | -0.1346 | -98 | 245 | 256 | IEIVLKETLDAK | | | | | Mascot |
| 1386.7739 | 1386.6051 | -0.1688 | -122 | 210 | 221 | IDHYLGKQVVSK | | | | | Mascot |

| | | | | | | | | | | | |
|---|---|---|---|---|---|---|---|---|---|---|---|
| 1618.9275 | 1618.717 | -0.2105 | -130 | 716 | 729 | LVTLTESPIKPHQR | | | | | Mascot |
| 1624.8118 | 1624.8014 | -0.0104 | -6 | 461 | 474 | EAYGELISHVFFGR | | | | | Mascot |
| 1624.8118 | 1624.8014 | -0.0104 | -6 | 461 | 474 | EAYGELISHVFFGR | | | | | Mascot |
| 1735.8319 | 1735.8815 | 0.0496 | 29 | 553 | 567 | SSEMISAWAQELVER | | | | | Mascot |
| 1735.8319 | 1735.8815 | 0.0496 | 29 | 553 | 567 | SSEMISAWAQELVER | | | | | Mascot |
| 1751.8269 | 1751.9026 | 0.0757 | 43 | 553 | 567 | SSEMISAWAQELVER | | | Oxidation (M)[4] | | Mascot |
| 1861.9918 | 1861.932 | -0.0598 | -32 | 505 | 521 | IYPGGVSNGNLLNFQLR | | | | | Mascot |
| 1993.1188 | 1993.1134 | -0.0054 | -3 | 29 | 48 | QSLGHVTVVVVGGTGDL ARK | | | | | Mascot |
| 1993.1188 | 1993.1134 | -0.0054 | -3 | 29 | 48 | QSLGHVTVVVVGGTGDL ARK | | | | | Mascot |
| 2248.1287 | 2248.1423 | 0.0136 | 6 | 1 | 19 | MENTGIWELLLMAIICGQ R | | | Carbamidomethyl (C)[16] | | Mascot |
| 2264.1235 | 2264.1484 | 0.0249 | 11 | 1 | 19 | MENTGIWELLLMAIICGQ R | | | Carbamidomethyl (C)[16], Oxidation (M)[1] | | Mascot |

39 novel lipin protein [Danio rerio] gi|220673084 33713 5.92 7 33 0

**Peptide Information**

| Calc. Mass | Obsrv. Mass | ± da | ± ppm | Start Seq. | End Seq. | Sequence | Ion Score | C. I. % | Modification | Rank | Result Type |
|---|---|---|---|---|---|---|---|---|---|---|---|
| 916.4345 | 916.4868 | 0.0523 | 57 | 129 | 135 | FLYCSAR | | | Carbamidomethyl (C)[4] | | Mascot |
| 981.4492 | 981.5854 | 0.1362 | 139 | 136 | 144 | AIGMADMTR | | | Oxidation (M)[4] | | Mascot |
| 1511.6708 | 1511.7045 | 0.0337 | 22 | 2 | 15 | KEESSSSDEDLSAK | | | | | Mascot |
| 1838.9282 | 1838.8909 | -0.0373 | -20 | 212 | 227 | DTDVFSYKEVGVPLNR | | | | | Mascot |
| 1970.9758 | 1971.0005 | 0.0247 | 13 | 195 | 211 | NLFLPNTEPFYAAFGNR | | | | | Mascot |
| 1970.9758 | 1971.0005 | 0.0247 | 13 | 195 | 211 | NLFLPNTEPFYAAFGNR | | | | | Mascot |
| 2120.0164 | 2120.1094 | 0.093 | 44 | 136 | 153 | AIGMADMTRGYLHWVNE R | | | | | Mascot |
| 2136.0112 | 2136.0969 | 0.0857 | 40 | 136 | 153 | AIGMADMTRGYLHWVNE R | | | Oxidation (M)[4] | | Mascot |
| 2152.0061 | 2152.093 | 0.0869 | 40 | 136 | 153 | AIGMADMTRGYLHWVNE R | | | Oxidation (M)[4,7] | | Mascot |
| 2247.9287 | 2248.1423 | 0.2136 | 95 | 266 | 284 | SSSSDFPCSDTFSQFTY WR | | | | | Mascot |

40 Wingless-type MMTV integration site family, member 16 [Danio rerio] gi|42542493 26754.7 9.08 9 33 0

**Peptide Information**

| Calc. Mass | Obsrv. Mass | ± da | ± ppm | Start Seq. | End Seq. | Sequence | Ion Score | C. I. % | Modification | Rank | Result Type |
|---|---|---|---|---|---|---|---|---|---|---|---|
| 855.3699 | 855.4388 | 0.0689 | 81 | 90 | 96 | TMLTDCR | | | Oxidation (M)[2] | | Mascot |
| 907.4995 | 907.4691 | -0.0304 | -33 | 55 | 62 | RFIDSAAK | | | | | Mascot |
| 916.5322 | 916.4868 | -0.0454 | -50 | 174 | 182 | LGVTGTRGR | | | | | Mascot |
| 994.5792 | 994.5829 | 0.0037 | 4 | 2 | 11 | AAGLVHAVTR | | | | | Mascot |

| Calc. Mass | Obsrv. Mass | ± da | ± ppm | Start Seq. | End Seq. | Sequence | Ion Score | C. I. % | Modification | Rank | Result Type |
|---|---|---|---|---|---|---|---|---|---|---|---|
| 1161.5139 | 1161.5631 | 0.0492 | 42 | 97 | 107 | CHGVSGSCAVK | | | Carbamidomethyl (C)[1,8] | | Mascot |
| 1371.6296 | 1371.6747 | 0.0451 | 33 | 108 | 118 | TCWRTMAAFER | | | | | Mascot |
| 1371.6296 | 1371.6747 | 0.0451 | 33 | 108 | 118 | TCWRTMAAFER | | | | | Mascot |
| 1419.7227 | 1419.7164 | -0.0063 | -4 | 127 | 138 | YETSVHVVDRSK | | | | | Mascot |
| 1442.5167 | 1442.6982 | 0.1815 | 126 | 229 | 239 | CETMNDMHTCK | | | Carbamidomethyl (C)[1,10], Oxidation (M)[4] | | Mascot |
| 1582.6229 | 1582.7301 | 0.1072 | 68 | 228 | 239 | RCETMNDMHTCK | | | Carbamidomethyl (C)[2,11] | | Mascot |

41 PREDICTED: rho GTPase-activating protein 21-like [Danio rerio] gi|326679518 211650.9 8.29 19 33 0

**Peptide Information**

| Calc. Mass | Obsrv. Mass | ± da | ± ppm | Start Seq. | End Seq. | Sequence | Ion Score | C. I. % | Modification | Rank | Result Type |
|---|---|---|---|---|---|---|---|---|---|---|---|
| 862.5145 | 862.4818 | -0.0327 | -38 | 934 | 940 | QIYAVLR | | | | | Mascot |
| 893.4474 | 893.416 | -0.0314 | -35 | 1377 | 1385 | SKGSWGSGK | | | | | Mascot |
| 916.5098 | 916.4868 | -0.023 | -25 | 84 | 92 | VNGESIIGK | | | | | Mascot |
| 1045.5248 | 1045.5667 | 0.0419 | 40 | 372 | 379 | RLYMYGSR | | | | | Mascot |
| 1053.4377 | 1053.576 | 0.1383 | 131 | 888 | 896 | CSEDSGSRR | | | Carbamidomethyl (C)[1] | | Mascot |
| 1053.5186 | 1053.576 | 0.0574 | 54 | 941 | 948 | GHCLYLYK | | | Carbamidomethyl (C)[3] | | Mascot |
| 1161.5609 | 1161.5631 | 0.0022 | 2 | 1 | 10 | MSFLFSSQAK | | | Oxidation (M)[1] | | Mascot |
| 1176.5239 | 1176.6029 | 0.079 | 67 | 71 | 80 | DEDNISRGDR | | | | | Mascot |
| 1197.6123 | 1197.5883 | -0.024 | -20 | 1829 | 1837 | DWIVSEHRR | | | | | Mascot |
| 1220.5464 | 1220.5864 | 0.04 | 33 | 1186 | 1196 | GMADIDIQDDK | | | | | Mascot |
| 1386.6694 | 1386.6051 | -0.0643 | -46 | 447 | 458 | LGGAECARNWPR | | | Carbamidomethyl (C)[6] | | Mascot |
| 1571.7952 | 1571.7905 | -0.0047 | -3 | 126 | 139 | DITALAYSQDAYLK | | | | | Mascot |
| 1790.8627 | 1790.9106 | 0.0479 | 27 | 1838 | 1855 | RGSQGSQASTELEGQAGK | | | | | Mascot |
| 1861.955 | 1861.932 | -0.023 | -12 | 1143 | 1158 | FVPLIVEVCCNLVEER | | | | | Mascot |
| 1992.9583 | 1993.1134 | 0.1551 | 78 | 1039 | 1056 | EYNTLMSPPSSKTEPSPK | | | | | Mascot |
| 1992.9583 | 1993.1134 | 0.1551 | 78 | 1039 | 1056 | EYNTLMSPPSSKTEPSPK | | | | | Mascot |
| 2066.1077 | 2066.0371 | -0.0706 | -34 | 623 | 640 | TPLIANHMPHNAKQLQPR | | | | | Mascot |
| 2151.9829 | 2152.093 | 0.1101 | 51 | 897 | 915 | DSNSDVFTDATKEGSLYFR | | | | | Mascot |
| 2248.0549 | 2248.1423 | 0.0874 | 39 | 477 | 496 | AYSCDYLGRNSDIATSIVDK | | | Carbamidomethyl (C)[4] | | Mascot |
| 2283.0723 | 2283.1802 | 0.1079 | 47 | 1781 | 1799 | HTMGGQRDFAELDVIHDWR | | | | | Mascot |

42 v-raf-1 murine leukemia viral oncogene homolog 1a [Danio rerio] gi|289449304 70289 9.46 11 32 0

**Peptide Information**

| Calc. Mass | Obsrv. Mass | ± da | ± ppm | Start | End | Sequence | Ion | C. I. % | Modification | Rank | Result Type |
|---|---|---|---|---|---|---|---|---|---|---|---|

| | | | | Seq. | Seq. | | Score | | | | |
|---|---|---|---|---|---|---|---|---|---|---|---|
| 845.3934 | 845.4667 | 0.0733 | 87 | 33 | 40 | SPAPCQSR | | | | | Mascot |
| 882.4791 | 882.5694 | 0.0903 | 102 | 215 | 222 | IPSSSVHR | | | | | Mascot |
| 946.5389 | 946.4759 | -0.063 | -67 | 563 | 570 | LVADCIKK | | | Carbamidomethyl (C)[5] | | Mascot |
| 946.5567 | 946.4759 | -0.0808 | -85 | 10 | 18 | TVTNGLLTK | | | | | Mascot |
| 1001.5526 | 1001.566 | 0.0134 | 13 | 53 | 60 | VFLPNQQR | | | | | Mascot |
| 1045.452 | 1045.5667 | 0.1147 | 110 | 165 | 172 | FHEHCSTK | | | Carbamidomethyl (C)[5] | | Mascot |
| 1371.7855 | 1371.6747 | -0.1108 | -81 | 50 | 60 | TIRVFLPNQQR | | | | | Mascot |
| 1371.7855 | 1371.6747 | -0.1108 | -81 | 50 | 60 | TIRVFLPNQQR | | | | | Mascot |
| 1556.7843 | 1556.7462 | -0.0381 | -24 | 19 | 32 | DSVFESSYVSPVIK | | | | | Mascot |
| 1582.812 | 1582.7301 | -0.0819 | -52 | 381 | 393 | HVNILLFMGYMTK | | | Oxidation (M)[8] | | Mascot |
| 1861.9476 | 1861.932 | -0.0156 | -8 | 447 | 462 | DMKSNNIFLHEGLTVK | | | Oxidation (M)[2] | | Mascot |
| 2211.0908 | 2211.1106 | 0.0198 | 9 | 81 | 99 | VRGLQPECCAVFSLHPGQR | | | Carbamidomethyl (C)[8,9] | | Mascot |

| | | | | | | | |
|---|---|---|---|---|---|---|---|
| 43 | CXC chemokine 64 [Danio rerio] | gi\|187608560 | 11150.2 | 10.14 | 6 | 32 | 0 |

Peptide Information

| Calc. Mass | Obsrv. Mass | ± da | ± ppm | Start Seq. | End Seq. | Sequence | Ion Score | C. I. % | Modification | Rank | Result Type |
|---|---|---|---|---|---|---|---|---|---|---|---|
| 855.5046 | 855.4388 | -0.0658 | -77 | 22 | 28 | IPDLKNR | | | | | Mascot |
| 1045.6326 | 1045.5667 | -0.0659 | -63 | 59 | 67 | LEIVVTLMK | | | | | Mascot |
| 1519.7355 | 1519.7588 | 0.0233 | 15 | 29 | 42 | CLCADKGANNVNLK | | | Carbamidomethyl (C)[1] | | Mascot |
| 1542.8308 | 1542.7043 | -0.1265 | -82 | 73 | 86 | CLNPESNLGKNILK | | | | | Mascot |
| 1971.0441 | 1971.0005 | -0.0436 | -22 | 2 | 19 | KTAAAFVALGCFLMVEVK | | | Carbamidomethyl (C)[11], Oxidation (M)[14] | | Mascot |
| 1971.0441 | 1971.0005 | -0.0436 | -22 | 3 | 21 | TAAAFVALGCFLMVEVKGK | | | Oxidation (M)[13] | | Mascot |

| | | | | | | | |
|---|---|---|---|---|---|---|---|
| 44 | plexin b2b [Danio rerio] | gi\|309243104 | 203041.6 | 6.62 | 18 | 32 | 0 |

Peptide Information

| Calc. Mass | Obsrv. Mass | ± da | ± ppm | Start Seq. | End Seq. | Sequence | Ion Score | C. I. % | Modification | Rank | Result Type |
|---|---|---|---|---|---|---|---|---|---|---|---|
| 870.4791 | 870.542 | 0.0629 | 72 | 1699 | 1705 | TEHKLSR | | | | | Mascot |
| 907.4995 | 907.4691 | -0.0304 | -33 | 372 | 378 | DIVNRYK | | | | | Mascot |
| 914.473 | 914.5472 | 0.0742 | 81 | 908 | 914 | TVQFTYR | | | | | Mascot |
| 994.5502 | 994.5829 | 0.0327 | 33 | 479 | 487 | VPVQACHLK | | | | | Mascot |
| 1109.4714 | 1109.5858 | 0.1144 | 103 | 488 | 497 | QDCQSCVAQK | | | | | Mascot |
| 1442.7849 | 1442.6982 | -0.0867 | -60 | 1175 | 1187 | RDTGSDPLDLLIK | | | | | Mascot |
| 1511.6406 | 1511.7045 | 0.0639 | 42 | 498 | 509 | DPYCGWCVLEGR | | | Carbamidomethyl (C)[4,7] | | Mascot |

| Calc. Mass | Obsrv. Mass | ± da | ± ppm | Start Seq. | End Seq. | Sequence | Ion Score | C. I. % | Modification | Rank | Result Type |
|---|---|---|---|---|---|---|---|---|---|---|---|
| 1519.8148 | 1519.7588 | -0.056 | -37 | 1114 | 1127 | SVITENSMIIVTGR | | | | | Mascot |
| 1542.8196 | 1542.7043 | -0.1153 | -75 | 1585 | 1597 | SMTKAITEIYLTR | | | Oxidation (M)[2] | | Mascot |
| 1587.8417 | 1587.7875 | -0.0542 | -34 | 685 | 698 | FENPDPVLIPVGYK | | | | | Mascot |
| 1618.851 | 1618.717 | -0.134 | -83 | 1345 | 1358 | TLLLGLMEDYVHSK | | | | | Mascot |
| 1624.8037 | 1624.8014 | -0.0023 | -1 | 1246 | 1258 | HQLENLEESVRDR | | | | | Mascot |
| 1624.8152 | 1624.8014 | -0.0138 | -8 | 377 | 391 | YKCGADFLPSPLASR | | | | | Mascot |
| 1719.8041 | 1719.884 | 0.0799 | 46 | 72 | 87 | DAKGCTPPVSTCQDLK | | | Carbamidomethyl (C)[5] | | Mascot |
| 1781.9102 | 1781.9781 | 0.0679 | 38 | 872 | 888 | YSVSTSVICEIGPASLR | | | | | Mascot |
| 1781.9102 | 1781.9781 | 0.0679 | 38 | 872 | 888 | YSVSTSVICEIGPASLR | | | | | Mascot |
| 1838.9316 | 1838.8909 | -0.0407 | -22 | 872 | 888 | YSVSTSVICEIGPASLR | | | Carbamidomethyl (C)[9] | | Mascot |
| 2066.0779 | 2066.0371 | -0.0408 | -20 | 1753 | 1769 | EHTDKLNTQVALHQLYR | | | | | Mascot |
| 2136.1118 | 2136.0969 | -0.0149 | -7 | 329 | 347 | NINQKLLAIVTACYTDNG K | | | Carbamidomethyl (C)[13] | | Mascot |
| 3337.5974 | 3337.7339 | 0.1365 | 41 | 1086 | 1113 | TFVQLDNLQMELTPFAYY PNPTFDELSK | | | Oxidation (M)[10] | | Mascot |

45 phosphorylase, glycogen (muscle) b [Danio rerio] gi|41054784 36631.2 6.27 8 32 0

**Peptide Information**

| Calc. Mass | Obsrv. Mass | ± da | ± ppm | Start Seq. | End Seq. | Sequence | Ion Score | C. I. % | Modification | Rank | Result Type |
|---|---|---|---|---|---|---|---|---|---|---|---|
| 893.4151 | 893.416 | 0.0009 | 1 | 186 | 192 | YGNPWEK | | | | | Mascot |
| 994.5832 | 994.5829 | -0.0003 | 0 | 35 | 42 | HLHFTLVK | | | | | Mascot |
| 1045.5525 | 1045.5667 | 0.0142 | 14 | 2 | 10 | SKPLTDQEK | | | | | Mascot |
| 1176.5929 | 1176.6029 | 0.01 | 8 | 1 | 10 | MSKPLTDQEK | | | | | Mascot |
| 1355.6742 | 1355.6809 | 0.0067 | 5 | 51 | 61 | DYYFALAHTVR | | | | | Mascot |
| 1355.6742 | 1355.7896 | 0.1154 | 85 | 51 | 61 | DYYFALAHTVR | | | | | Mascot |
| 1442.678 | 1442.6982 | 0.0202 | 14 | 236 | 246 | NNMLMHHDRFK | | | | | Mascot |
| 1511.7754 | 1511.7045 | -0.0709 | -47 | 50 | 61 | RDYYFALAHTVR | | | | | Mascot |
| 1556.7454 | 1556.7462 | 0.0008 | 1 | 83 | 94 | VYYLSLEFYMGR | | | Oxidation (M)[10] | | Mascot |

46 novel protein similar to vertebrate syntaxin binding protein 6 (amisyn) (STXBP6) [Danio rerio] gi|94734057 31605.2 10.27 9 32 0

**Peptide Information**

| Calc. Mass | Obsrv. Mass | ± da | ± ppm | Start Seq. | End Seq. | Sequence | Ion Score | C. I. % | Modification | Rank | Result Type |
|---|---|---|---|---|---|---|---|---|---|---|---|
| 893.5025 | 893.416 | -0.0865 | -97 | 189 | 195 | CKIFLNR | | | | | Mascot |
| 916.5098 | 916.4868 | -0.023 | -25 | 150 | 158 | TPGAVESKK | | | | | Mascot |
| 1045.5748 | 1045.5667 | -0.0081 | -8 | 237 | 245 | RASQVLSER | | | | | Mascot |
| 1179.5536 | 1179.666 | 0.1124 | 95 | 198 | 207 | TSMTANQDRR | | | | | Mascot |

| | | | | | | | | | | | |
|---|---|---|---|---|---|---|---|---|---|---|---|
| 1179.5536 | 1179.666 | 0.1124 | 95 | 198 | 207 | TSMTANQDRR | | | | | Mascot |
| 1571.7379 | 1571.7905 | 0.0526 | 33 | 175 | 188 | LMGDACSVNMVIYR | | | | | Mascot |
| 1587.7328 | 1587.7875 | 0.0547 | 34 | 175 | 188 | LMGDACSVNMVIYR | | | Oxidation (M)[2] | | Mascot |
| 1790.849 | 1790.9106 | 0.0616 | 34 | 159 | 174 | SPAATNQTNFINCQPK | | | Carbamidomethyl (C)[13] | | Mascot |
| 1859.8634 | 1859.8964 | 0.033 | 18 | 175 | 190 | LMGDACSVNMVIYRCK | | | Carbamidomethyl (C)[6] | | Mascot |
| 1859.8634 | 1859.8964 | 0.033 | 18 | 175 | 190 | LMGDACSVNMVIYRCK | | | Carbamidomethyl (C)[6] | | Mascot |
| 1861.9225 | 1861.932 | 0.0095 | 5 | 158 | 174 | KSPAATNQTNFINCQPK | | | | | Mascot |
| 2233.0027 | 2233.0947 | 0.092 | 41 | 119 | 136 | CMFVQVLYHACQNFWEAK | | | Oxidation (M)[2] | | Mascot |

47 methionyl aminopeptidase 1 [Danio rerio] gi|220679064 45363 6.14 8 32 0

**Peptide Information**

| Calc. Mass | Obsrv. Mass | ± da | ± ppm | Start Seq. | End Seq. | Sequence | Ion Score | C. I. % | Modification | Rank | Result Type |
|---|---|---|---|---|---|---|---|---|---|---|---|
| 845.5025 | 845.4667 | -0.0358 | -42 | 159 | 165 | VVCKLAR | | | Carbamidomethyl (C)[3] | | Mascot |
| 864.4243 | 864.4891 | 0.0648 | 75 | 19 | 26 | SMAAVETR | | | | | Mascot |
| 914.5305 | 914.5472 | 0.0167 | 18 | 289 | 296 | ELGNIIQK | | | | | Mascot |
| 1001.4621 | 1001.566 | 0.1039 | 104 | 308 | 316 | SYCGHGIHK | | | | | Mascot |
| 1719.8767 | 1719.884 | 0.0073 | 4 | 148 | 162 | ILNAEEIEGMRVVCK | | | Oxidation (M)[10] | | Mascot |
| 1832.8088 | 1832.9417 | 0.1329 | 73 | 210 | 226 | SCCTSVNEVICHGIPDR | | | | | Mascot |
| 2225.0781 | 2225.1245 | 0.0464 | 21 | 297 | 316 | HAQANGFSVVRSYCGHGIHK | | | Carbamidomethyl (C)[14] | | Mascot |
| 2248.1277 | 2248.1423 | 0.0146 | 6 | 372 | 391 | SAQFEHTLLVTETGCEILTR | | | | | Mascot |

48 V-type proton ATPase subunit C 1-A [Danio rerio] gi|41151984 44286.9 6.93 8 32 0

**Peptide Information**

| Calc. Mass | Obsrv. Mass | ± da | ± ppm | Start Seq. | End Seq. | Sequence | Ion Score | C. I. % | Modification | Rank | Result Type |
|---|---|---|---|---|---|---|---|---|---|---|---|
| 864.4573 | 864.4891 | 0.0318 | 37 | 232 | 238 | KAIDDFR | | | | | Mascot |
| 893.4839 | 893.416 | -0.0679 | -76 | 243 | 249 | ENKFTVR | | | | | Mascot |
| 1001.5162 | 1001.566 | 0.0498 | 50 | 233 | 240 | AIDDFRHK | | | | | Mascot |
| 1386.7335 | 1386.6051 | -0.1284 | -93 | 128 | 139 | QVSQIDNDLKAR | | | | | Mascot |
| 1442.7137 | 1442.6982 | -0.0155 | -11 | 105 | 115 | FQWDMAKYPIK | | | Oxidation (M)[5] | | Mascot |
| 1624.7999 | 1624.8014 | 0.0015 | 1 | 71 | 84 | KVAQYMADVLEDSR | 4 | 0 | | | Mascot |
| 1624.7999 | 1624.8014 | 0.0015 | 1 | 71 | 84 | KVAQYMADVLEDSR | | | | | Mascot |
| 1832.9501 | 1832.9417 | -0.0084 | -5 | 29 | 44 | TNNLSTNNKFNIPDLK | | | | | Mascot |
| 1961.0677 | 1960.9794 | -0.0883 | -45 | 310 | 326 | YGLPVNFQAMLLQPNKK | | | | | Mascot |

49 novel protein [Danio rerio] gi|169146110 25865.2 5.07 7 32 0

Peptide Information

| Calc. Mass | Obsrv. Mass | ± da | ± ppm | Start Seq. | End Seq. | Sequence | Ion Score | C. I. % | Modification | Rank | Result Type |
|---|---|---|---|---|---|---|---|---|---|---|---|
| 893.4475 | 893.416 | -0.0315 | -35 | 26 | 32 | NTFVQER | | | | | Mascot |
| 914.5166 | 914.5472 | 0.0306 | 33 | 88 | 95 | QLAAAERR | | | | | Mascot |
| 1045.5411 | 1045.5667 | 0.0256 | 24 | 99 | 107 | VIADLEEEK | | | | | Mascot |
| 1179.5319 | 1179.666 | 0.1341 | 114 | 73 | 82 | LVMAHCTNMK | | | Oxidation (M)[3,9] | | Mascot |
| 1179.5319 | 1179.666 | 0.1341 | 114 | 73 | 82 | LVMAHCTNMK | | | Oxidation (M)[3,9] | | Mascot |
| 1220.5585 | 1220.5864 | 0.0279 | 23 | 73 | 82 | LVMAHCTNMK | | | Carbamidomethyl (C)[6], Oxidation (M)[3] | | Mascot |
| 1386.7335 | 1386.6051 | -0.1284 | -93 | 129 | 139 | DRLLQQLESER | | | | | Mascot |
| 1992.8927 | 1993.1134 | 0.2207 | 111 | 111 | 128 | AQDAAAGDDITDMLERE R | | | Oxidation (M)[13] | | Mascot |
| 1992.8927 | 1993.1134 | 0.2207 | 111 | 111 | 128 | AQDAAAGDDITDMLERE R | | | Oxidation (M)[13] | | Mascot |
| 2248.2546 | 2248.1423 | -0.1123 | -50 | 2 | 21 | LLSILEGELEAQDVVIHTL R | | | | | Mascot |

| | | | | | | | | | |
|---|---|---|---|---|---|---|---|---|---|
| 50 | cytochrome P450, family 2, subfamily AD, polypeptide 3 [Danio rerio] | gi\|70887751 | 56608.1 | 6.25 | 1 | | | 20 | 0 |

Peptide Information

| Calc. Mass | Obsrv. Mass | ± da | ± ppm | Start Seq. | End Seq. | Sequence | Ion Score | C. I. % | Modification | Rank | Result Type |
|---|---|---|---|---|---|---|---|---|---|---|---|
| 946.4774 | 946.4759 | -0.0015 | -2 | 439 | 447 | ACLGENLAR | 20 | 0 | | 2 | Mascot |

| Gel Idx/Pos | 177/H4 | Instr./Gel Origin | BA2060/110707A | Process Status | Analysis Succeeded |
|---|---|---|---|---|---|
| Plate [#] Name | [1] 31496 | Instrument Sample Name | | Spectra | 11 |

| Rank | Protein Name | Accession No. | Protein MW | Protein PI | Pep. Count | Protein Score | Protein Score C. I. % | Total Ion Score | Total Ion C. I. % |
|---|---|---|---|---|---|---|---|---|---|
| 1 | Methylenetetrahydrofolate dehydrogenase (NADP+ dependent) [Danio rerio] | gi\|49902652 | 100427.7 | 7.28 | 13 | 403 | 100 | 364 | 100 |

Peptide Information

| Calc. Mass | Obsrv. Mass | ± da | ± ppm | Start Seq. | End Seq. | Sequence | Ion Score | C. I. % | Modification | Rank | Result Type |
|---|---|---|---|---|---|---|---|---|---|---|---|
| 950.4875 | 950.4852 | -0.0023 | -2 | 402 | 409 | LNAFACVR | | | Carbamidomethyl (C)[6] | | Mascot |
| 982.5679 | 982.567 | -0.0009 | -1 | 478 | 486 | LVPLAEGQR | | | | | Mascot |
| 1088.6211 | 1088.615 | -0.0061 | -6 | 487 | 495 | KFSPVQINR | | | | | Mascot |
| 1155.6885 | 1155.6885 | 0 | 0 | 878 | 888 | GVPTGFVLPIR | | | | | Mascot |
| 1155.6885 | 1155.6885 | 0 | 0 | 878 | 888 | GVPTGFVLPIR | 37 | 95.174 | | | Mascot |
| 1458.7224 | 1458.7205 | -0.0019 | -1 | 520 | 531 | LDIDPESVTWQR | | | | | Mascot |

| | | | | | | | | | | | |
|---|---|---|---|---|---|---|---|---|---|---|---|
| 1458.7224 | 1458.7205 | -0.0019 | -1 | 520 | 531 | LDIDPESVTWQR | 89 | 100 | | | Mascot |
| 1592.8165 | 1592.814 | -0.0025 | -2 | 336 | 349 | EIGLLSDEVELYGR | 136 | 100 | | | Mascot |
| 1592.8165 | 1592.814 | -0.0025 | -2 | 336 | 349 | EIGLLSDEVELYGR | | | | | Mascot |
| 2125.9893 | 2125.9878 | -0.0015 | -1 | 721 | 738 | EYIEENLTLLEAGCANMR | | | Carbamidomethyl (C)[14] | | Mascot |
| 2141.9841 | 2141.9885 | 0.0044 | 2 | 721 | 738 | EYIEENLTLLEAGCANMR | | | Carbamidomethyl (C)[14], Oxidation (M)[17] | | Mascot |
| 2204.105 | 2204.1025 | -0.0025 | -1 | 595 | 615 | SGQPVTTEDLGVCGALTVLMR | | | Carbamidomethyl (C)[13] | | Mascot |
| 2218.1133 | 2218.1294 | 0.0161 | 7 | 228 | 249 | TGAVVIDCGINTIADSSRPSGK | | | Carbamidomethyl (C)[8] | | Mascot |
| 2220.0999 | 2220.1204 | 0.0205 | 9 | 595 | 615 | SGQPVTTEDLGVCGALTVLMR | | | Carbamidomethyl (C)[13], Oxidation (M)[20] | | Mascot |
| 2238.1111 | 2238.1086 | -0.0025 | -1 | 657 | 678 | LVGPQGFVVTEAGFGADIGMEK | | | Oxidation (M)[20] | | Mascot |
| 2269.2664 | 2269.2627 | -0.0037 | -2 | 93 | 112 | IHGLIVQLPLDSIHTINTER | | | | | Mascot |
| 2269.2664 | 2269.2627 | -0.0037 | -2 | 93 | 112 | IHGLIVQLPLDSIHTINTER | 101 | 100 | | | Mascot |
| 2297.0068 | 2297.1938 | 0.187 | 81 | 137 | 156 | GDLGDCFIPCTPNGCMELIK | | | Carbamidomethyl (C)[6,10,15] | | Mascot |
| 2500.1309 | 2500.1299 | -0.001 | 0 | 775 | 799 | EAGAFDAVPCSHWADGGAGAVDLAR | | | Carbamidomethyl (C)[10] | | Mascot |

| | | | | | | | | | | |
|---|---|---|---|---|---|---|---|---|---|---|
| 2 | C-1-tetrahydrofolate synthase, cytoplasmic [Danio rerio] | gi\|41054762 | 100397.5 | 7.06 | 11 | 302 | 100 | 275 | 100 | |

**Peptide Information**

| Calc. Mass | Obsrv. Mass | ± da | ± ppm | Start Seq. | End Seq. | Sequence | Ion Score | C. I. % | Modification | Rank | Result Type |
|---|---|---|---|---|---|---|---|---|---|---|---|
| 950.4875 | 950.4852 | -0.0023 | -2 | 402 | 409 | LNAFACVR | | | Carbamidomethyl (C)[6] | | Mascot |
| 982.5679 | 982.567 | -0.0009 | -1 | 478 | 486 | LVPLAEGQR | | | | | Mascot |
| 1088.6211 | 1088.615 | -0.0061 | -6 | 487 | 495 | KFSPVQINR | | | | | Mascot |
| 1155.6885 | 1155.6885 | 0 | 0 | 878 | 888 | GVPTGFVLPIR | | | | | Mascot |
| 1155.6885 | 1155.6885 | 0 | 0 | 878 | 888 | GVPTGFVLPIR | 37 | 95.174 | | | Mascot |
| 1592.8165 | 1592.814 | -0.0025 | -2 | 336 | 349 | EIGLLSDEVELYGR | 136 | 100 | | | Mascot |
| 1592.8165 | 1592.814 | -0.0025 | -2 | 336 | 349 | EIGLLSDEVELYGR | | | | | Mascot |
| 2125.9893 | 2125.9878 | -0.0015 | -1 | 721 | 738 | EYIEENLTLLEAGCANMR | | | Carbamidomethyl (C)[14] | | Mascot |
| 2141.9841 | 2141.9885 | 0.0044 | 2 | 721 | 738 | EYIEENLTLLEAGCANMR | | | Carbamidomethyl (C)[14], Oxidation (M)[17] | | Mascot |
| 2204.105 | 2204.1025 | -0.0025 | -1 | 595 | 615 | SGQPVTTEDLGVCGALTVLMR | | | Carbamidomethyl (C)[13] | | Mascot |
| 2220.0999 | 2220.1204 | 0.0205 | 9 | 595 | 615 | SGQPVTTEDLGVCGALTVLMR | | | Carbamidomethyl (C)[13], Oxidation (M)[20] | | Mascot |
| 2238.1111 | 2238.1086 | -0.0025 | -1 | 657 | 678 | LVGPQGFVVTEAGFGADIGMEK | | | Oxidation (M)[20] | | Mascot |
| 2269.2664 | 2269.2627 | -0.0037 | -2 | 93 | 112 | IHGLIVQLPLDSIHTINTER | | | | | Mascot |
| 2269.2664 | 2269.2627 | -0.0037 | -2 | 93 | 112 | IHGLIVQLPLDSIHTINTER | 101 | 100 | | | Mascot |
| 2297.0068 | 2297.1938 | 0.187 | 81 | 137 | 156 | GDLGDCFIPCTPNGCMELIK | | | Carbamidomethyl (C)[6,10,15] | | Mascot |
| 2500.1309 | 2500.1299 | -0.001 | 0 | 775 | 799 | EAGAFDAVPCSHWADGGAGAVDLAR | | | Carbamidomethyl (C)[10] | | Mascot |

| 3 | vitellogenin 1 [Danio rerio] | gi\|94733730 | 149140.4 | 8.74 | 18 | 300 | 100 | 252 | 100 |
|---|---|---|---|---|---|---|---|---|---|

Peptide Information

| Calc. Mass | Obsrv. Mass | ± da | ± ppm | Start Seq. | End Seq. | Sequence | Ion Score | C. I. % | Modification | Rank | Result Type |
|---|---|---|---|---|---|---|---|---|---|---|---|
| 888.5301 | 888.5256 | -0.0045 | -5 | 425 | 432 | FATIPALR | | | | | Mascot |
| 1023.5105 | 1023.5066 | -0.0039 | -4 | 195 | 203 | DVGLAYTER | | | | | Mascot |
| 1088.5405 | 1088.615 | 0.0745 | 68 | 415 | 424 | MTASLATHEK | | | | | Mascot |
| 1097.6677 | 1097.6646 | -0.0031 | -3 | 514 | 523 | VQVDAILALR | | | | | Mascot |
| 1115.6935 | 1115.6925 | -0.001 | -1 | 333 | 341 | FVQLVQLLR | | | | | Mascot |
| 1115.6935 | 1115.6925 | -0.001 | -1 | 333 | 341 | FVQLVQLLR | 52 | 99.845 | | | Mascot |
| 1121.599 | 1121.5931 | -0.0059 | -5 | 348 | 356 | IEAIWSQFK | | | | | Mascot |
| 1382.7889 | 1382.6827 | -0.1062 | -77 | 303 | 315 | ISDAPAQIVEVLK | | | | | Mascot |
| 1432.6671 | 1432.6503 | -0.0168 | -12 | 184 | 194 | DLSHCQERIMK | | | Carbamidomethyl (C)[5], Oxidation (M)[10] | | Mascot |
| 1463.8944 | 1463.8929 | -0.0015 | -1 | 532 | 544 | LVQPVALQLVLDR | | | | | Mascot |
| 1515.7478 | 1515.7451 | -0.0027 | -2 | 733 | 745 | VFGQEVAYVNFDK | | | | | Mascot |
| 1622.8536 | 1622.8505 | -0.0031 | -2 | 668 | 683 | AYLAGAAADVIEFGVR | | | | | Mascot |
| 1622.8536 | 1622.8505 | -0.0031 | -2 | 668 | 683 | AYLAGAAADVIEFGVR | 121 | 100 | | | Mascot |
| 1666.8767 | 1666.8771 | 0.0004 | 0 | 483 | 498 | VMGNAGHPSSLKPIMK | | | | | Mascot |
| 1695.8999 | 1695.8931 | -0.0068 | -4 | 119 | 134 | VFAPAGVSPTVMNLHR | | | | | Mascot |
| 1699.9377 | 1699.9332 | -0.0045 | -3 | 34 | 49 | YEALLLGGLPQEGLAR | | | | | Mascot |
| 1699.9377 | 1699.9332 | -0.0045 | -3 | 34 | 49 | YEALLLGGLPQEGLAR | 80 | 100 | | | Mascot |
| 1711.8948 | 1711.8909 | -0.0039 | -2 | 119 | 134 | VFAPAGVSPTVMNLHR | | | Oxidation (M)[12] | | Mascot |
| 1954.8745 | 1954.8737 | -0.0008 | 0 | 147 | 163 | TQNIYEMQEAGAQGVCR | | | Carbamidomethyl (C)[16] | | Mascot |
| 2269.1975 | 2269.2627 | 0.0652 | 29 | 1179 | 1198 | ADQKLLGYQLAAYFDKPT AR | | | | | Mascot |
| 2269.1975 | 2269.2627 | 0.0652 | 29 | 1179 | 1198 | ADQKLLGYQLAAYFDKPT AR | | | | | Mascot |
| 2299.2292 | 2299.1831 | -0.0461 | -20 | 1039 | 1058 | QINIIDDDTPEGQAFLLKL R | | | | | Mascot |
| 2627.3716 | 2627.3674 | -0.0042 | -2 | 897 | 920 | VEALPVELPEHIASASFET YAVVR | | | | | Mascot |

| 4 | novel protein similar to vitellogenin 1 (vg1) [Danio rerio] | gi\|94733731 | 149169.2 | 8.84 | 13 | 275 | 100 | 252 | 100 |
|---|---|---|---|---|---|---|---|---|---|

Peptide Information

| Calc. Mass | Obsrv. Mass | ± da | ± ppm | Start Seq. | End Seq. | Sequence | Ion Score | C. I. % | Modification | Rank | Result Type |
|---|---|---|---|---|---|---|---|---|---|---|---|
| 1023.5105 | 1023.5066 | -0.0039 | -4 | 199 | 207 | DVGLAYTER | | | | | Mascot |
| 1097.6677 | 1097.6646 | -0.0031 | -3 | 518 | 527 | VQVDAILALR | | | | | Mascot |
| 1115.6935 | 1115.6925 | -0.001 | -1 | 337 | 345 | FVQLVQLLR | | | | | Mascot |

| | | | | | | | | | | | |
|---|---|---|---|---|---|---|---|---|---|---|---|
| 1115.6935 | 1115.6925 | -0.001 | -1 | 337 | 345 | FVQLVQLLR | 52 | 99.845 | | | Mascot |
| 1121.599 | 1121.5931 | -0.0059 | -5 | 352 | 360 | IEAIWSQFK | | | | | Mascot |
| 1432.6671 | 1432.6503 | -0.0168 | -12 | 188 | 198 | DLSHCQERIMK | | | Carbamidomethyl (C)[5], Oxidation (M)[10] | | Mascot |
| 1463.8944 | 1463.8929 | -0.0015 | -1 | 536 | 548 | LVQPVALQLVLDR | | | | | Mascot |
| 1515.7478 | 1515.7451 | -0.0027 | -2 | 737 | 749 | VFGQEVAYVNFDK | | | | | Mascot |
| 1622.8536 | 1622.8505 | -0.0031 | -2 | 672 | 687 | AYLAGAAADVIEFGVR | | | | | Mascot |
| 1622.8536 | 1622.8505 | -0.0031 | -2 | 672 | 687 | AYLAGAAADVIEFGVR | 121 | 100 | | | Mascot |
| 1666.8767 | 1666.8771 | 0.0004 | 0 | 487 | 502 | VMGNAGHPSSLKPIMK | | | | | Mascot |
| 1699.9377 | 1699.9332 | -0.0045 | -3 | 38 | 53 | YEALLLGGLPQEGLAR | | | | | Mascot |
| 1699.9377 | 1699.9332 | -0.0045 | -3 | 38 | 53 | YEALLLGGLPQEGLAR | 80 | 100 | | | Mascot |
| 1954.8745 | 1954.8737 | -0.0008 | 0 | 151 | 167 | TQNIYEMQEAGAQGVCR | | | Carbamidomethyl (C)[16] | | Mascot |
| 2269.1975 | 2269.2627 | 0.0652 | 29 | 1181 | 1200 | ADQKLLGYQLAAYFDKPTAR | | | | | Mascot |
| 2269.1975 | 2269.2627 | 0.0652 | 29 | 1181 | 1200 | ADQKLLGYQLAAYFDKPTAR | | | | | Mascot |
| 2299.2292 | 2299.1831 | -0.0461 | -20 | 1043 | 1062 | QINIIDDDTPEGQAFLLKLR | | | | | Mascot |

| | | | | | | | | | | |
|---|---|---|---|---|---|---|---|---|---|---|
| 5 | Vtg1 protein [Danio rerio] | gi\|145337918 | 116565.5 | 9.07 | 14 | 211 | 100 | 174 | 100 | |

**Peptide Information**

| Calc. Mass | Obsrv. Mass | ± da | ± ppm | Start Seq. | End Seq. | Sequence | Ion Score | C. I. % | Modification | Rank | Result Type |
|---|---|---|---|---|---|---|---|---|---|---|---|
| 888.5301 | 888.5256 | -0.0045 | -5 | 131 | 138 | FATIPALR | | | | | Mascot |
| 1088.5405 | 1088.615 | 0.0745 | 68 | 121 | 130 | MTASLATHEK | | | | | Mascot |
| 1097.6677 | 1097.6646 | -0.0031 | -3 | 220 | 229 | VQVDAILALR | | | | | Mascot |
| 1115.6935 | 1115.6925 | -0.001 | -1 | 39 | 47 | FVQLVQLLR | | | | | Mascot |
| 1115.6935 | 1115.6925 | -0.001 | -1 | 39 | 47 | FVQLVQLLR | 52 | 99.845 | | | Mascot |
| 1121.599 | 1121.5931 | -0.0059 | -5 | 54 | 62 | IEAIWSQFK | | | | | Mascot |
| 1382.7889 | 1382.6827 | -0.1062 | -77 | 9 | 21 | ISDAPAQIVEVLK | | | | | Mascot |
| 1463.8944 | 1463.8929 | -0.0015 | -1 | 238 | 250 | LVQPVALQLVLDR | | | | | Mascot |
| 1515.7478 | 1515.7451 | -0.0027 | -2 | 439 | 451 | VFGQEVAYVNFDK | | | | | Mascot |
| 1622.8536 | 1622.8505 | -0.0031 | -2 | 374 | 389 | AYLAGAAADVIEFGVR | | | | | Mascot |
| 1622.8536 | 1622.8505 | -0.0031 | -2 | 374 | 389 | AYLAGAAADVIEFGVR | 121 | 100 | | | Mascot |
| 1666.8767 | 1666.8771 | 0.0004 | 0 | 189 | 204 | VMGNAGHPSSLKPIMK | | | | | Mascot |
| 1699.9047 | 1699.9332 | 0.0285 | 17 | 303 | 320 | ITAPDMASVAGAANVAIK | | | | | Mascot |
| 1699.9047 | 1699.9332 | 0.0285 | 17 | 303 | 320 | ITAPDMASVAGAANVAIK | | | | | Mascot |
| 2269.1975 | 2269.2627 | 0.0652 | 29 | 885 | 904 | ADQKLLGYQLAAYFDKPTAR | | | | | Mascot |
| 2269.1975 | 2269.2627 | 0.0652 | 29 | 885 | 904 | ADQKLLGYQLAAYFDKPTAR | | | | | Mascot |
| 2299.2292 | 2299.1831 | -0.0461 | -20 | 745 | 764 | QINIIDDDTPEGQAFLLKL | | | | | Mascot |

| | | | | | | | | | | | |
|---|---|---|---|---|---|---|---|---|---|---|---|
| 2627.3716 | 2627.3674 | -0.0042 | -2 | 603 | 626 | R VEALPVELPEHIASASFET YAVVR | | | | | Mascot |

| | | | | | | | | | | |
|---|---|---|---|---|---|---|---|---|---|---|
| 6 | vitellogenin 1 [Danio rerio] | gi\|21391472 | 127954.2 | 8.69 | 14 | 207 | 100 | 174 | 100 | |

**Peptide Information**

| Calc. Mass | Obsrv. Mass | ± da | ± ppm | Start Seq. | End Seq. | Sequence | Ion Score | C. I. % | Modification | Rank | Result Type |
|---|---|---|---|---|---|---|---|---|---|---|---|
| 888.5301 | 888.5256 | -0.0045 | -5 | 234 | 241 | FATIPALR | | | | | Mascot |
| 1023.5105 | 1023.5066 | -0.0039 | -4 | 4 | 12 | DVGLAYTER | | | | | Mascot |
| 1088.5405 | 1088.615 | 0.0745 | 68 | 224 | 233 | MTASLATHEK | | | | | Mascot |
| 1097.6677 | 1097.6646 | -0.0031 | -3 | 323 | 332 | VQVDAILALR | | | | | Mascot |
| 1115.6935 | 1115.6925 | -0.001 | -1 | 142 | 150 | FVQLVQLLR | | | | | Mascot |
| 1115.6935 | 1115.6925 | -0.001 | -1 | 142 | 150 | FVQLVQLLR | 52 | 99.845 | | | Mascot |
| 1121.599 | 1121.5931 | -0.0059 | -5 | 157 | 165 | IEAIWSQFK | | | | | Mascot |
| 1382.7889 | 1382.6827 | -0.1062 | -77 | 112 | 124 | ISDAPAQIVEVLK | | | | | Mascot |
| 1463.8944 | 1463.8929 | -0.0015 | -1 | 341 | 353 | LVQPVALQLVLDR | | | | | Mascot |
| 1515.7478 | 1515.7451 | -0.0027 | -2 | 542 | 554 | VFGQEVAYVNFDK | | | | | Mascot |
| 1622.8536 | 1622.8505 | -0.0031 | -2 | 477 | 492 | AYLAGAAADVIEFGVR | | | | | Mascot |
| 1622.8536 | 1622.8505 | -0.0031 | -2 | 477 | 492 | AYLAGAAADVIEFGVR | 121 | 100 | | | Mascot |
| 1666.8767 | 1666.8771 | 0.0004 | 0 | 292 | 307 | VMGNAGHPSSLKPIMK | | | | | Mascot |
| 1699.9047 | 1699.9332 | 0.0285 | 17 | 406 | 423 | ITAPDMASVAGAANVAIK | | | | | Mascot |
| 1699.9047 | 1699.9332 | 0.0285 | 17 | 406 | 423 | ITAPDMASVAGAANVAIK | | | | | Mascot |
| 2269.1975 | 2269.2627 | 0.0652 | 29 | 986 | 1005 | ADQKLLGYQLAAYFDKPT AR | | | | | Mascot |
| 2269.1975 | 2269.2627 | 0.0652 | 29 | 986 | 1005 | ADQKLLGYQLAAYFDKPT AR | | | | | Mascot |
| 2299.2292 | 2299.1831 | -0.0461 | -20 | 848 | 867 | QINIIDDDTPEGQAFLLKL R | | | | | Mascot |

| | | | | | | | | | | |
|---|---|---|---|---|---|---|---|---|---|---|
| 7 | hypothetical protein LOC678536 [Danio rerio] | gi\|160420306 | 148750.2 | 8.89 | 19 | 184 | 100 | 130 | 100 | |

**Peptide Information**

| Calc. Mass | Obsrv. Mass | ± da | ± ppm | Start Seq. | End Seq. | Sequence | Ion Score | C. I. % | Modification | Rank | Result Type |
|---|---|---|---|---|---|---|---|---|---|---|---|
| 864.4461 | 864.5085 | 0.0624 | 72 | 87 | 94 | DSFVPATK | | | | | Mascot |
| 1023.5105 | 1023.5066 | -0.0039 | -4 | 195 | 203 | DVGLAYTER | | | | | Mascot |
| 1045.5465 | 1045.5631 | 0.0166 | 16 | 1346 | 1353 | DFLSWIHK | | | | | Mascot |
| 1097.6677 | 1097.6646 | -0.0031 | -3 | 514 | 523 | VQVDAILALR | | | | | Mascot |
| 1115.6935 | 1115.6925 | -0.001 | -1 | 333 | 341 | FVQLVQLLR | | | | | Mascot |
| 1115.6935 | 1115.6925 | -0.001 | -1 | 333 | 341 | FVQLVQLLR | 52 | 99.845 | | | Mascot |
| 1121.599 | 1121.5931 | -0.0059 | -5 | 348 | 356 | IEAIWSQFK | | | | | Mascot |

| | | | | | | | | | | | |
|---|---|---|---|---|---|---|---|---|---|---|---|
| 1382.7889 | 1382.6827 | -0.1062 | -77 | 303 | 315 | ISDAPAQIVEVLK | | | | | Mascot |
| 1432.6671 | 1432.6503 | -0.0168 | -12 | 184 | 194 | DLSHCQERIMK | | | Carbamidomethyl (C)[5], Oxidation (M)[10] | | Mascot |
| 1477.91 | 1477.9099 | -0.0001 | 0 | 532 | 544 | LVQPIALQLVLDR | | | | | Mascot |
| 1515.7478 | 1515.7451 | -0.0027 | -2 | 733 | 745 | VFGQEVAYVNFDK | | | | | Mascot |
| 1608.838 | 1608.8344 | -0.0036 | -2 | 668 | 683 | AYLAGAAADVVEFGVR | | | | | Mascot |
| 1666.8767 | 1666.8771 | 0.0004 | 0 | 483 | 498 | VMGNAGHPSSLKPIMK | | | | | Mascot |
| 1695.8999 | 1695.8931 | -0.0068 | -4 | 119 | 134 | VFAPAGVSPTVMNLHR | | | | | Mascot |
| 1699.9377 | 1699.9332 | -0.0045 | -3 | 34 | 49 | YEALLLGGLPQEGLAR | | | | | Mascot |
| 1699.9377 | 1699.9332 | -0.0045 | -3 | 34 | 49 | YEALLLGGLPQEGLAR | 80 | 100 | | | Mascot |
| 1711.8948 | 1711.8909 | -0.0039 | -2 | 119 | 134 | VFAPAGVSPTVMNLHR | | | Oxidation (M)[12] | | Mascot |
| 1936.9181 | 1936.916 | -0.0021 | -1 | 147 | 163 | TQNIYELQEAGAQGVCR | | | Carbamidomethyl (C)[16] | | Mascot |
| 2218.1641 | 2218.1294 | -0.0347 | -16 | 1326 | 1345 | DIYLPVTVPINPDGTFTID K | | | | | Mascot |
| 2233.2009 | 2233.082 | -0.1189 | -53 | 593 | 614 | SLTRITAPDMAYVAGAAN VAIK | | | | | Mascot |
| 2269.1975 | 2269.2627 | 0.0652 | 29 | 1177 | 1196 | ADQKLLGYQLAAYFDKPT AR | | | | | Mascot |
| 2269.1975 | 2269.2627 | 0.0652 | 29 | 1177 | 1196 | ADQKLLGYQLAAYFDKPT AR | | | | | Mascot |
| 2299.2292 | 2299.1831 | -0.0461 | -20 | 1039 | 1058 | QINIIDDDTPEGQAFLLKL R | | | | | Mascot |

8 novel protein similar to vitellogenin 1 (vg1) [Danio rerio] gi|94733733 149143.4 8.92 19 183 100 130 100

**Peptide Information**

| Calc. Mass | Obsrv. Mass | ± da | ± ppm | Start Seq. | End Seq. | Sequence | Ion Score | C. I. % | Modification | Rank | Result Type |
|---|---|---|---|---|---|---|---|---|---|---|---|
| 864.4461 | 864.5085 | 0.0624 | 72 | 91 | 98 | DSFVPATK | | | | | Mascot |
| 1023.5105 | 1023.5066 | -0.0039 | -4 | 199 | 207 | DVGLAYTER | | | | | Mascot |
| 1045.5465 | 1045.5631 | 0.0166 | 16 | 1350 | 1357 | DFLSWIHK | | | | | Mascot |
| 1097.6677 | 1097.6646 | -0.0031 | -3 | 518 | 527 | VQVDAILALR | | | | | Mascot |
| 1115.6935 | 1115.6925 | -0.001 | -1 | 337 | 345 | FVQLVQLLR | | | | | Mascot |
| 1115.6935 | 1115.6925 | -0.001 | -1 | 337 | 345 | FVQLVQLLR | 52 | 99.845 | | | Mascot |
| 1121.599 | 1121.5931 | -0.0059 | -5 | 352 | 360 | IEAIWSQFK | | | | | Mascot |
| 1382.7889 | 1382.6827 | -0.1062 | -77 | 307 | 319 | ISDAPAQIVEVLK | | | | | Mascot |
| 1432.6671 | 1432.6503 | -0.0168 | -12 | 188 | 198 | DLSHCQERIMK | | | Carbamidomethyl (C)[5], Oxidation (M)[10] | | Mascot |
| 1477.91 | 1477.9099 | -0.0001 | 0 | 536 | 548 | LVQPIALQLVLDR | | | | | Mascot |
| 1515.7478 | 1515.7451 | -0.0027 | -2 | 737 | 749 | VFGQEVAYVNFDK | | | | | Mascot |
| 1608.838 | 1608.8344 | -0.0036 | -2 | 672 | 687 | AYLAGAAADVVEFGVR | | | | | Mascot |
| 1666.8767 | 1666.8771 | 0.0004 | 0 | 487 | 502 | VMGNAGHPSSLKPIMK | | | | | Mascot |
| 1695.8999 | 1695.8931 | -0.0068 | -4 | 123 | 138 | VFAPAGVSPTVMNLHR | | | | | Mascot |

| | | | | | | | | | | | |
|---|---|---|---|---|---|---|---|---|---|---|---|
| 1699.9377 | 1699.9332 | -0.0045 | -3 | 38 | 53 | YEALLLGGLPQEGLAR | | | | | Mascot |
| 1699.9377 | 1699.9332 | -0.0045 | -3 | 38 | 53 | YEALLLGGLPQEGLAR | 80 | 100 | | | Mascot |
| 1711.8948 | 1711.8909 | -0.0039 | -2 | 123 | 138 | VFAPAGVSPTVMNLHR | | | Oxidation (M)[12] | | Mascot |
| 1936.9181 | 1936.916 | -0.0021 | -1 | 151 | 167 | TQNIYELQEAGAQGVCR | | | Carbamidomethyl (C)[16] | | Mascot |
| 2218.1641 | 2218.1294 | -0.0347 | -16 | 1330 | 1349 | DIYLPVTVPINPDGTFTIDK | | | | | Mascot |
| 2233.2009 | 2233.082 | -0.1189 | -53 | 597 | 618 | SLTRITAPDMAYVAGAANVAIK | | | | | Mascot |
| 2269.1975 | 2269.2627 | 0.0652 | 29 | 1181 | 1200 | ADQKLLGYQLAAYFDKPTAR | | | | | Mascot |
| 2269.1975 | 2269.2627 | 0.0652 | 29 | 1181 | 1200 | ADQKLLGYQLAAYFDKPTAR | | | | | Mascot |
| 2299.2292 | 2299.1831 | -0.0461 | -20 | 1043 | 1062 | QINIIDDDTPEGQAFLLKLR | | | | | Mascot |

9 vitellogenin 1 [Danio rerio] gi|166795887 149452.5 8.68 17 174 100 132 100

**Peptide Information**

| Calc. Mass | Obsrv. Mass | ± da | ± ppm | Start Seq. | End Seq. | Sequence | Ion Score | C. I. % | Modification | Rank | Result Type |
|---|---|---|---|---|---|---|---|---|---|---|---|
| 888.5301 | 888.5256 | -0.0045 | -5 | 425 | 432 | FATIPALR | | | | | Mascot |
| 1088.5405 | 1088.615 | 0.0745 | 68 | 415 | 424 | MTASLATHEK | | | | | Mascot |
| 1097.6677 | 1097.6646 | -0.0031 | -3 | 514 | 523 | VQVDAILALR | | | | | Mascot |
| 1115.6935 | 1115.6925 | -0.001 | -1 | 333 | 341 | FVQLVQLLR | | | | | Mascot |
| 1115.6935 | 1115.6925 | -0.001 | -1 | 333 | 341 | FVQLVQLLR | 52 | 99.845 | | | Mascot |
| 1121.599 | 1121.5931 | -0.0059 | -5 | 348 | 356 | IEAIWSQFK | | | | | Mascot |
| 1382.7889 | 1382.6827 | -0.1062 | -77 | 303 | 315 | ISDAPAQIVEVLK | | | | | Mascot |
| 1432.6671 | 1432.6503 | -0.0168 | -12 | 184 | 194 | DLSHCQERIMK | | | Carbamidomethyl (C)[5], Oxidation (M)[10] | | Mascot |
| 1463.8944 | 1463.8929 | -0.0015 | -1 | 532 | 544 | LVQPVALQLVLDR | | | | | Mascot |
| 1515.7478 | 1515.7451 | -0.0027 | -2 | 733 | 745 | VFGQEVAYVNFDK | | | | | Mascot |
| 1666.8767 | 1666.8771 | 0.0004 | 0 | 483 | 498 | VMGNAGHPSSLKPIMK | | | | | Mascot |
| 1695.8999 | 1695.8931 | -0.0068 | -4 | 119 | 134 | VFAPAGVSPTVMNLHR | | | | | Mascot |
| 1699.9377 | 1699.9332 | -0.0045 | -3 | 34 | 49 | YEALLLGGLPQEGLAR | | | | | Mascot |
| 1699.9377 | 1699.9332 | -0.0045 | -3 | 34 | 49 | YEALLLGGLPQEGLAR | 80 | 100 | | | Mascot |
| 1711.8948 | 1711.8909 | -0.0039 | -2 | 119 | 134 | VFAPAGVSPTVMNLHR | | | Oxidation (M)[12] | | Mascot |
| 1954.8745 | 1954.8737 | -0.0008 | 0 | 147 | 163 | TQNIYEMQEAGAQGVCR | | | Carbamidomethyl (C)[16] | | Mascot |
| 2233.2009 | 2233.082 | -0.1189 | -53 | 593 | 614 | SLTRITAPDMAYVAGAANVAIK | | | | | Mascot |
| 2269.1975 | 2269.2627 | 0.0652 | 29 | 1179 | 1198 | ADQKLLGYQLAAYFDKPTAR | | | | | Mascot |
| 2269.1975 | 2269.2627 | 0.0652 | 29 | 1179 | 1198 | ADQKLLGYQLAAYFDKPTAR | | | | | Mascot |
| 2299.2292 | 2299.1831 | -0.0461 | -20 | 1039 | 1058 | QINIIDDDTPEGQAFLLKLR | | | | | Mascot |
| 2627.3716 | 2627.3674 | -0.0042 | -2 | 897 | 920 | VEALPVELPEHIASASFET | | | | | Mascot |

YAVVR

| | | | | | | | | | | |
|---|---|---|---|---|---|---|---|---|---|---|
| 10 | novel protein similar to vitellogenin 1 (vg1) [Danio rerio] | gi\|94733734 | 149808.5 | 8.83 | 16 | 169 | 100 | 132 | 100 |

Peptide Information

| Calc. Mass | Obsrv. Mass | ± da | ± ppm | Start Seq. | End Seq. | Sequence | Ion Score | C. I. % | Modification | Rank | Result Type |
|---|---|---|---|---|---|---|---|---|---|---|---|
| 864.4461 | 864.5085 | 0.0624 | 72 | 91 | 98 | DSFVPATK | | | | | Mascot |
| 1023.5105 | 1023.5066 | -0.0039 | -4 | 199 | 207 | DVGLAYTER | | | | | Mascot |
| 1097.6677 | 1097.6646 | -0.0031 | -3 | 518 | 527 | VQVDAILALR | | | | | Mascot |
| 1115.6935 | 1115.6925 | -0.001 | -1 | 337 | 345 | FVQLVQLLR | | | | | Mascot |
| 1115.6935 | 1115.6925 | -0.001 | -1 | 337 | 345 | FVQLVQLLR | 52 | 99.845 | | | Mascot |
| 1121.599 | 1121.5931 | -0.0059 | -5 | 352 | 360 | IEAIWSQFK | | | | | Mascot |
| 1432.6671 | 1432.6503 | -0.0168 | -12 | 188 | 198 | DLSHCQERIMK | | | Carbamidomethyl (C)[5], Oxidation (M)[10] | | Mascot |
| 1463.8944 | 1463.8929 | -0.0015 | -1 | 536 | 548 | LVQPVALQLVLDR | | | | | Mascot |
| 1515.7478 | 1515.7451 | -0.0027 | -2 | 737 | 749 | VFGQEVAYVNFDK | | | | | Mascot |
| 1666.8767 | 1666.8771 | 0.0004 | 0 | 487 | 502 | VMGNAGHPSSLKPIMK | | | | | Mascot |
| 1695.8999 | 1695.8931 | -0.0068 | -4 | 123 | 138 | VFAPAGVSPTVMNLHR | | | | | Mascot |
| 1699.9377 | 1699.9332 | -0.0045 | -3 | 38 | 53 | YEALLLGGLPQEGLAR | | | | | Mascot |
| 1699.9377 | 1699.9332 | -0.0045 | -3 | 38 | 53 | YEALLLGGLPQEGLAR | 80 | 100 | | | Mascot |
| 1711.8948 | 1711.8909 | -0.0039 | -2 | 123 | 138 | VFAPAGVSPTVMNLHR | | | Oxidation (M)[12] | | Mascot |
| 1936.9181 | 1936.916 | -0.0021 | -1 | 151 | 167 | TQNIYELQEAGAQGVCR | | | Carbamidomethyl (C)[16] | | Mascot |
| 2194.0469 | 2194.0884 | 0.0415 | 19 | 1063 | 1083 | EILDTEAKNAPVSSESSS SSR | | | | | Mascot |
| 2233.2009 | 2233.082 | -0.1189 | -53 | 597 | 618 | SLTRITAPDMAYVAGAAN VAIK | | | | | Mascot |
| 2269.1975 | 2269.2627 | 0.0652 | 29 | 1186 | 1205 | ADQKLLGYQLAAYFDKPT AR | | | | | Mascot |
| 2269.1975 | 2269.2627 | 0.0652 | 29 | 1186 | 1205 | ADQKLLGYQLAAYFDKPT AR | | | | | Mascot |
| 2299.2292 | 2299.1831 | -0.0461 | -20 | 1043 | 1062 | QINIIDDDTPEGQAFLLKL R | | | | | Mascot |

| | | | | | | | | | | |
|---|---|---|---|---|---|---|---|---|---|---|
| 11 | vitellogenin 6 [Danio rerio] | gi\|303227889 | 150936.1 | 8.84 | 16 | 168 | 100 | 132 | 100 |

Peptide Information

| Calc. Mass | Obsrv. Mass | ± da | ± ppm | Start Seq. | End Seq. | Sequence | Ion Score | C. I. % | Modification | Rank | Result Type |
|---|---|---|---|---|---|---|---|---|---|---|---|
| 864.4461 | 864.5085 | 0.0624 | 72 | 98 | 105 | DSFVPATK | | | | | Mascot |
| 1023.5105 | 1023.5066 | -0.0039 | -4 | 206 | 214 | DVGLAYTER | | | | | Mascot |
| 1097.6677 | 1097.6646 | -0.0031 | -3 | 525 | 534 | VQVDAILALR | | | | | Mascot |
| 1115.6935 | 1115.6925 | -0.001 | -1 | 344 | 352 | FVQLVQLLR | | | | | Mascot |
| 1115.6935 | 1115.6925 | -0.001 | -1 | 344 | 352 | FVQLVQLLR | 52 | 99.845 | | | Mascot |

| | | | | | | | | | | | |
|---|---|---|---|---|---|---|---|---|---|---|---|
| 1121.599 | 1121.5931 | -0.0059 | -5 | 359 | 367 | IEAIWSQFK | | | | | Mascot |
| 1432.6671 | 1432.6503 | -0.0168 | -12 | 195 | 205 | DLSHCQERIMK | | | Carbamidomethyl (C)[5], Oxidation (M)[10] | | Mascot |
| 1463.8944 | 1463.8929 | -0.0015 | -1 | 543 | 555 | LVQPVALQLVLDR | | | | | Mascot |
| 1515.7478 | 1515.7451 | -0.0027 | -2 | 744 | 756 | VFGQEVAYVNFDK | | | | | Mascot |
| 1666.8767 | 1666.8771 | 0.0004 | 0 | 494 | 509 | VMGNAGHPSSLKPIMK | | | | | Mascot |
| 1695.8999 | 1695.8931 | -0.0068 | -4 | 130 | 145 | VFAPAGVSPTVMNLHR | | | | | Mascot |
| 1699.9377 | 1699.9332 | -0.0045 | -3 | 45 | 60 | YEALLLGGLPQEGLAR | | | | | Mascot |
| 1699.9377 | 1699.9332 | -0.0045 | -3 | 45 | 60 | YEALLLGGLPQEGLAR | 80 | 100 | | | Mascot |
| 1711.8948 | 1711.8909 | -0.0039 | -2 | 130 | 145 | VFAPAGVSPTVMNLHR | | | Oxidation (M)[12] | | Mascot |
| 1936.9181 | 1936.916 | -0.0021 | -1 | 158 | 174 | TQNIYELQEAGAQGVCR | | | Carbamidomethyl (C)[16] | | Mascot |
| 2194.0469 | 2194.0884 | 0.0415 | 19 | 1070 | 1090 | EILDTEAKNAPVSSESSSSR | | | | | Mascot |
| 2233.2009 | 2233.082 | -0.1189 | -53 | 604 | 625 | SLTRITAPDMAYVAGAANVAIK | | | | | Mascot |
| 2269.1975 | 2269.2627 | 0.0652 | 29 | 1197 | 1216 | ADQKLLGYQLAAYFDKPTAR | | | | | Mascot |
| 2269.1975 | 2269.2627 | 0.0652 | 29 | 1197 | 1216 | ADQKLLGYQLAAYFDKPTAR | | | | | Mascot |
| 2299.2292 | 2299.1831 | -0.0461 | -20 | 1050 | 1069 | QINIIDDDTPEGQAFLLKLR | | | | | Mascot |

| | | | | | | | | | | |
|---|---|---|---|---|---|---|---|---|---|---|
| 12 | novel protein similar to vitellogenin 1 (vg1) [Danio rerio] | gi\|94732723 | 147084.2 | 8.73 | 12 | 150 | 100 | 130 | 100 | |

**Peptide Information**

| Calc. Mass | Obsrv. Mass | ± da | ± ppm | Start Seq. | End Seq. | Sequence | Ion Score | C. I. % | Modification | Rank | Result Type |
|---|---|---|---|---|---|---|---|---|---|---|---|
| 1097.6677 | 1097.6646 | -0.0031 | -3 | 514 | 523 | VQVDAILALR | | | | | Mascot |
| 1115.6935 | 1115.6925 | -0.001 | -1 | 333 | 341 | FVQLVQLLR | | | | | Mascot |
| 1115.6935 | 1115.6925 | -0.001 | -1 | 333 | 341 | FVQLVQLLR | 52 | 99.845 | | | Mascot |
| 1432.6671 | 1432.6503 | -0.0168 | -12 | 184 | 194 | DLSHCQERIMK | | | Carbamidomethyl (C)[5], Oxidation (M)[10] | | Mascot |
| 1477.91 | 1477.9099 | -0.0001 | 0 | 532 | 544 | LVQPIALQLVLDR | | | | | Mascot |
| 1515.7478 | 1515.7451 | -0.0027 | -2 | 733 | 745 | VFGQEVAYVNFDK | | | | | Mascot |
| 1622.7743 | 1622.8505 | 0.0762 | 47 | 981 | 994 | GCVEVHSYNAAFIR | | | Carbamidomethyl (C)[2] | | Mascot |
| 1622.7743 | 1622.8505 | 0.0762 | 47 | 981 | 994 | GCVEVHSYNAAFIR | | | Carbamidomethyl (C)[2] | | Mascot |
| 1625.6742 | 1625.8387 | 0.1645 | 101 | 1098 | 1113 | SSSSSSSSMSSSRMSK | | | Oxidation (M)[9] | | Mascot |
| 1666.8767 | 1666.8771 | 0.0004 | 0 | 483 | 498 | VMGNAGHPSSLKPIMK | | | | | Mascot |
| 1699.9377 | 1699.9332 | -0.0045 | -3 | 34 | 49 | YEALLLGGLPQEGLAR | | | | | Mascot |
| 1699.9377 | 1699.9332 | -0.0045 | -3 | 34 | 49 | YEALLLGGLPQEGLAR | 80 | 100 | | | Mascot |
| 1936.9181 | 1936.916 | -0.0021 | -1 | 147 | 163 | TQNIYELQEAGAQGVCR | | | Carbamidomethyl (C)[16] | | Mascot |
| 2217.9824 | 2218.1294 | 0.147 | 66 | 192 | 210 | IMKDVGLAYTESCPECTER | | | Carbamidomethyl (C)[13], Oxidation (M)[2] | | Mascot |
| 3337.7539 | 3337.7656 | 0.0117 | 4 | 1164 | 1193 | LLGYQLAAYFDKPTARVQLIASSIAENDNR | | | | | Mascot |

| 13 | vitellogenin 7 [Danio rerio] | gi\|156713467 | 148739.2 | 8.76 | 9 | 142 | 100 | 130 | 100 |
|---|---|---|---|---|---|---|---|---|---|

Peptide Information

| Calc. Mass | Obsrv. Mass | ± da | ± ppm | Start Seq. | End Seq. | Sequence | Ion Score | C. I. % | Modification | Rank | Result Type |
|---|---|---|---|---|---|---|---|---|---|---|---|
| 1097.6677 | 1097.6646 | -0.0031 | -3 | 514 | 523 | VQVDAILALR | | | | | Mascot |
| 1115.6935 | 1115.6925 | -0.001 | -1 | 333 | 341 | FVQLVQLLR | | | | | Mascot |
| 1115.6935 | 1115.6925 | -0.001 | -1 | 333 | 341 | FVQLVQLLR | 52 | 99.845 | | | Mascot |
| 1432.6671 | 1432.6503 | -0.0168 | -12 | 184 | 194 | DLSHCQERIMK | | | Carbamidomethyl (C)[5], Oxidation (M)[10] | | Mascot |
| 1477.91 | 1477.9099 | -0.0001 | 0 | 532 | 544 | LVQPIALQLVLDR | | | | | Mascot |
| 1515.7478 | 1515.7451 | -0.0027 | -2 | 733 | 745 | VFGQEVAYVNFDK | | | | | Mascot |
| 1666.8767 | 1666.8771 | 0.0004 | 0 | 483 | 498 | VMGNAGHPSSLKPIMK | | | | | Mascot |
| 1699.9377 | 1699.9332 | -0.0045 | -3 | 34 | 49 | YEALLLGGLPQEGLAR | | | | | Mascot |
| 1699.9377 | 1699.9332 | -0.0045 | -3 | 34 | 49 | YEALLLGGLPQEGLAR | 80 | 100 | | | Mascot |
| 2217.9824 | 2218.1294 | 0.147 | 66 | 192 | 210 | IMKDVGLAYTESCPECTE R | | | Carbamidomethyl (C)[13], Oxidation (M)[2] | | Mascot |
| 3337.7539 | 3337.7656 | 0.0117 | 4 | 1179 | 1208 | LLGYQLAAYFDKPTARVQ LIASSIAENDNR | | | | | Mascot |

| 14 | vitellogenin 1 [Danio rerio] | gi\|21391474 | 21118.2 | 9 | 4 | 99 | 100 | 80 | 100 |
|---|---|---|---|---|---|---|---|---|---|

Peptide Information

| Calc. Mass | Obsrv. Mass | ± da | ± ppm | Start Seq. | End Seq. | Sequence | Ion Score | C. I. % | Modification | Rank | Result Type |
|---|---|---|---|---|---|---|---|---|---|---|---|
| 864.4461 | 864.5085 | 0.0624 | 72 | 87 | 94 | DSFVPATK | | | | | Mascot |
| 1695.8999 | 1695.8931 | -0.0068 | -4 | 119 | 134 | VFAPAGVSPTVMNLHR | | | | | Mascot |
| 1699.9377 | 1699.9332 | -0.0045 | -3 | 34 | 49 | YEALLLGGLPQEGLAR | | | | | Mascot |
| 1699.9377 | 1699.9332 | -0.0045 | -3 | 34 | 49 | YEALLLGGLPQEGLAR | 80 | 100 | | | Mascot |
| 1711.8948 | 1711.8909 | -0.0039 | -2 | 119 | 134 | VFAPAGVSPTVMNLHR | | | Oxidation (M)[12] | | Mascot |
| 1936.9181 | 1936.916 | -0.0021 | -1 | 147 | 163 | TQNIYELQEAGAQGVCR | | | Carbamidomethyl (C)[16] | | Mascot |

| 15 | Zgc:136383 protein [Danio rerio] | gi\|159155252 | 123957.1 | 8.78 | 14 | 86 | 99.988 | 52 | 99.845 |
|---|---|---|---|---|---|---|---|---|---|

Peptide Information

| Calc. Mass | Obsrv. Mass | ± da | ± ppm | Start Seq. | End Seq. | Sequence | Ion Score | C. I. % | Modification | Rank | Result Type |
|---|---|---|---|---|---|---|---|---|---|---|---|
| 1045.5465 | 1045.5631 | 0.0166 | 16 | 1123 | 1130 | DFLSWIHK | | | | | Mascot |
| 1097.6677 | 1097.6646 | -0.0031 | -3 | 291 | 300 | VQVDAILALR | | | | | Mascot |
| 1115.6935 | 1115.6925 | -0.001 | -1 | 110 | 118 | FVQLVQLLR | | | | | Mascot |
| 1115.6935 | 1115.6925 | -0.001 | -1 | 110 | 118 | FVQLVQLLR | 52 | 99.845 | | | Mascot |
| 1121.599 | 1121.5931 | -0.0059 | -5 | 125 | 133 | IEAIWSQFK | | | | | Mascot |

| Calc. Mass | Obsrv. Mass | ± da | ± ppm | Start Seq. | End Seq. | Sequence | Ion Score | C. I. % | Modification | Rank | Result Type |
|---|---|---|---|---|---|---|---|---|---|---|---|
| 1382.7889 | 1382.6827 | -0.1062 | -77 | 80 | 92 | ISDAPAQIVEVLK | | | | | Mascot |
| 1477.91 | 1477.9099 | -0.0001 | 0 | 309 | 321 | LVQPIALQLVLDR | | | | | Mascot |
| 1515.7478 | 1515.7451 | -0.0027 | -2 | 510 | 522 | VFGQEVAYVNFDK | | | | | Mascot |
| 1608.838 | 1608.8344 | -0.0036 | -2 | 445 | 460 | AYLAGAAADVVEFGVR | | | | | Mascot |
| 1666.8767 | 1666.8771 | 0.0004 | 0 | 260 | 275 | VMGNAGHPSSLKPIMK | | | | | Mascot |
| 1699.9524 | 1699.9332 | -0.0192 | -11 | 1087 | 1101 | SLNVIARIPEITMSR | | | | | Mascot |
| 1699.9524 | 1699.9332 | -0.0192 | -11 | 1087 | 1101 | SLNVIARIPEITMSR | | | | | Mascot |
| 2218.1641 | 2218.1294 | -0.0347 | -16 | 1103 | 1122 | DIYLPVTVPINPDGTFTIDK | | | | | Mascot |
| 2233.2009 | 2233.082 | -0.1189 | -53 | 370 | 391 | SLTRITAPDMAYVAGAANVAIK | | | | | Mascot |
| 2269.1975 | 2269.2627 | 0.0652 | 29 | 954 | 973 | ADQKLLGYQLAAYFDKPTAR | | | | | Mascot |
| 2269.1975 | 2269.2627 | 0.0652 | 29 | 954 | 973 | ADQKLLGYQLAAYFDKPTAR | | | | | Mascot |
| 2299.2292 | 2299.1831 | -0.0461 | -20 | 816 | 835 | QINIIDDDTPEGQAFLLKLR | | | | | Mascot |

16 Unknown (protein for MGC:174137) [Danio rerio] gi|152012733 17051.5 7.71 1 84 99.984 80 100

**Peptide Information**

| Calc. Mass | Obsrv. Mass | ± da | ± ppm | Start Seq. | End Seq. | Sequence | Ion Score | C. I. % | Modification | Rank | Result Type |
|---|---|---|---|---|---|---|---|---|---|---|---|
| 1699.9377 | 1699.9332 | -0.0045 | -3 | 34 | 49 | YEALLLGGLPQEGLAR | | | | | Mascot |
| 1699.9377 | 1699.9332 | -0.0045 | -3 | 34 | 49 | YEALLLGGLPQEGLAR | 80 | 100 | | | Mascot |

17 novel protein similar to vitellogenin 1 (vg1) [Danio rerio] gi|94732724 24001.7 5.6 1 52 72.561 52 99.845

**Peptide Information**

| Calc. Mass | Obsrv. Mass | ± da | ± ppm | Start Seq. | End Seq. | Sequence | Ion Score | C. I. % | Modification | Rank | Result Type |
|---|---|---|---|---|---|---|---|---|---|---|---|
| 1115.6935 | 1115.6925 | -0.001 | -1 | 128 | 136 | FVQLVQLLR | | | | | Mascot |
| 1115.6935 | 1115.6925 | -0.001 | -1 | 128 | 136 | FVQLVQLLR | 52 | 99.845 | | | Mascot |

18 Synembryn-like [Danio rerio] gi|94574123 59482.8 5.37 10 48 29.471 17 0

**Peptide Information**

| Calc. Mass | Obsrv. Mass | ± da | ± ppm | Start Seq. | End Seq. | Sequence | Ion Score | C. I. % | Modification | Rank | Result Type |
|---|---|---|---|---|---|---|---|---|---|---|---|
| 842.5709 | 842.5099 | -0.061 | -72 | 18 | 24 | VEKLLLK | | | | | Mascot |
| 868.4999 | 868.538 | 0.0381 | 44 | 251 | 257 | DSLHKLR | | | | | Mascot |
| 1045.6769 | 1045.5631 | -0.1138 | -109 | 171 | 179 | LLFLLSALR | | | | | Mascot |
| 1115.6605 | 1115.6925 | 0.032 | 29 | 46 | 55 | LCQGLLTVLR | | | | | Mascot |
| 1115.6605 | 1115.6925 | 0.032 | 29 | 46 | 55 | LCQGLLTVLR | 17 | 0 | | | Mascot |

| Calc. Mass | Obsrv. Mass | ± da | ± ppm | Start Seq. | End Seq. | Sequence | Ion Score | C. I. % | Modification | Rank | Result Type |
|---|---|---|---|---|---|---|---|---|---|---|---|
| 1164.6736 | 1164.5659 | -0.1077 | -92 | 76 | 86 | DKHVLGPVATK | | | | | Mascot |
| 1382.7136 | 1382.6827 | -0.0309 | -22 | 399 | 410 | QGAAEFLFVLCK | | | Carbamidomethyl (C)[11] | | Mascot |
| 1625.8169 | 1625.8387 | 0.0218 | 13 | 32 | 45 | TFAFDLEEEGARIK | | | | | Mascot |
| 1695.8809 | 1695.8931 | 0.0122 | 7 | 315 | 328 | NMDTVQILLNFMIK | | | Oxidation (M)[2] | | Mascot |
| 1711.8757 | 1711.8909 | 0.0152 | 9 | 315 | 328 | NMDTVQILLNFMIK | | | Oxidation (M)[2,12] | | Mascot |
| 1954.9539 | 1954.8737 | -0.0802 | -41 | 486 | 501 | EYEAQRLVSMIDELSR | | | Oxidation (M)[10] | | Mascot |
| 2283.0193 | 2283.176 | 0.1567 | 69 | 98 | 118 | ICVAGHEGEPLEEAQSAEEER | | | | | Mascot |
| 2283.0193 | 2283.176 | 0.1567 | 69 | 98 | 118 | ICVAGHEGEPLEEAQSAEEER | | | | | Mascot |

19 Synbl protein [Danio rerio] gi|141796272 31226.2 5.37 7 47 11.209 17 0

**Peptide Information**

| Calc. Mass | Obsrv. Mass | ± da | ± ppm | Start Seq. | End Seq. | Sequence | Ion Score | C. I. % | Modification | Rank | Result Type |
|---|---|---|---|---|---|---|---|---|---|---|---|
| 842.5709 | 842.5099 | -0.061 | -72 | 18 | 24 | VEKLLLK | | | | | Mascot |
| 868.4999 | 868.538 | 0.0381 | 44 | 251 | 257 | DSLHKLR | | | | | Mascot |
| 1045.6769 | 1045.5631 | -0.1138 | -109 | 171 | 179 | LLFLLSALR | | | | | Mascot |
| 1115.6605 | 1115.6925 | 0.032 | 29 | 46 | 55 | LCQGLLTVLR | | | | | Mascot |
| 1115.6605 | 1115.6925 | 0.032 | 29 | 46 | 55 | LCQGLLTVLR | 17 | 0 | | | Mascot |
| 1164.6736 | 1164.5659 | -0.1077 | -92 | 76 | 86 | DKHVLGPVATK | | | | | Mascot |
| 1625.8169 | 1625.8387 | 0.0218 | 13 | 32 | 45 | TFAFDLEEEGARIK | | | | | Mascot |
| 2283.0193 | 2283.176 | 0.1567 | 69 | 98 | 118 | ICVAGHEGEPLEEAQSAEEER | | | | | Mascot |
| 2283.0193 | 2283.176 | 0.1567 | 69 | 98 | 118 | ICVAGHEGEPLEEAQSAEEER | | | | | Mascot |

20 Synbl protein [Danio rerio] gi|63101126 33515.4 5.82 7 47 7.024 17 0

**Peptide Information**

| Calc. Mass | Obsrv. Mass | ± da | ± ppm | Start Seq. | End Seq. | Sequence | Ion Score | C. I. % | Modification | Rank | Result Type |
|---|---|---|---|---|---|---|---|---|---|---|---|
| 842.5709 | 842.5099 | -0.061 | -72 | 41 | 47 | VEKLLLK | | | | | Mascot |
| 868.4999 | 868.538 | 0.0381 | 44 | 274 | 280 | DSLHKLR | | | | | Mascot |
| 1045.6769 | 1045.5631 | -0.1138 | -109 | 194 | 202 | LLFLLSALR | | | | | Mascot |
| 1115.6605 | 1115.6925 | 0.032 | 29 | 69 | 78 | LCQGLLTVLR | | | | | Mascot |
| 1115.6605 | 1115.6925 | 0.032 | 29 | 69 | 78 | LCQGLLTVLR | 17 | 0 | | | Mascot |
| 1164.6736 | 1164.5659 | -0.1077 | -92 | 99 | 109 | DKHVLGPVATK | | | | | Mascot |
| 1625.8169 | 1625.8387 | 0.0218 | 13 | 55 | 68 | TFAFDLEEEGARIK | | | | | Mascot |
| 2283.0193 | 2283.176 | 0.1567 | 69 | 121 | 141 | ICVAGHEGEPLEEAQSAEEER | | | | | Mascot |
| 2283.0193 | 2283.176 | 0.1567 | 69 | 121 | 141 | ICVAGHEGEPLEEAQSAEEER | | | | | Mascot |

| | | | | | | | | | |
|---|---|---|---|---|---|---|---|---|---|
| 21 | novel protein [Danio rerio] | gi\|94733078 | 59497.8 | 5.33 | 9 | 43 | 0 | 17 | 0 |

Peptide Information

| Calc. Mass | Obsrv. Mass | ± da | ± ppm | Start Seq. | End Seq. | Sequence | Ion Score | C. I. % | Modification | Rank | Result Type |
|---|---|---|---|---|---|---|---|---|---|---|---|
| 842.5709 | 842.5099 | -0.061 | -72 | 18 | 24 | VEKLLLK | | | | | Mascot |
| 868.4999 | 868.538 | 0.0381 | 44 | 251 | 257 | DSLHKLR | | | | | Mascot |
| 1045.6769 | 1045.5631 | -0.1138 | -109 | 171 | 179 | LLFLLSALR | | | | | Mascot |
| 1115.6605 | 1115.6925 | 0.032 | 29 | 46 | 55 | LCQGLLTVLR | | | | | Mascot |
| 1115.6605 | 1115.6925 | 0.032 | 29 | 46 | 55 | LCQGLLTVLR | 17 | 0 | | | Mascot |
| 1164.6736 | 1164.5659 | -0.1077 | -92 | 76 | 86 | DKHVLGPVATK | | | | | Mascot |
| 1382.7136 | 1382.6827 | -0.0309 | -22 | 399 | 410 | QGAAEFLFVLCK | | | Carbamidomethyl (C)[11] | | Mascot |
| 1625.8169 | 1625.8387 | 0.0218 | 13 | 32 | 45 | TFAFDLEEEGARIK | | | | | Mascot |
| 1954.9539 | 1954.8737 | -0.0802 | -41 | 486 | 501 | EYEAQRLVSMIDELSR | | | Oxidation (M)[10] | | Mascot |
| 2283.0193 | 2283.176 | 0.1567 | 69 | 98 | 118 | ICVAGHEGEPLEEAQSAE EER | | | | | Mascot |
| 2283.0193 | 2283.176 | 0.1567 | 69 | 98 | 118 | ICVAGHEGEPLEEAQSAE EER | | | | | Mascot |

| | | | | | | | |
|---|---|---|---|---|---|---|---|
| 22 | BarH-like 1.2 [Danio rerio] | gi\|62955401 | 22067.8 | 6.9 | 7 | 43 | 0 |

Peptide Information

| Calc. Mass | Obsrv. Mass | ± da | ± ppm | Start Seq. | End Seq. | Sequence | Ion Score | C. I. % | Modification | Rank | Result Type |
|---|---|---|---|---|---|---|---|---|---|---|---|
| 1023.5833 | 1023.5066 | -0.0767 | -75 | 81 | 89 | TVTSSFLIR | | | | | Mascot |
| 1155.5865 | 1155.6885 | 0.102 | 88 | 54 | 63 | SSVEDAVHRR | | | | | Mascot |
| 1155.5865 | 1155.6885 | 0.102 | 88 | 53 | 62 | RSSVEDAVHR | | | | | Mascot |
| 1608.7612 | 1608.8344 | 0.0732 | 46 | 121 | 134 | LHSNSSSDSEYRVK | | | | | Mascot |
| 1622.886 | 1622.8505 | -0.0355 | -22 | 66 | 80 | ALGLDSPLQISQQPR | | | | | Mascot |
| 1622.886 | 1622.8505 | -0.0355 | -22 | 66 | 80 | ALGLDSPLQISQQPR | | | | | Mascot |
| 2299.062 | 2299.1831 | 0.1211 | 53 | 31 | 52 | SPAELSPRSDLDSGCSS PPSPR | | | Carbamidomethyl (C)[15] | | Mascot |
| 2627.4514 | 2627.3674 | -0.084 | -32 | 66 | 89 | ALGLDSPLQISQQPRTVT SSFLIR | | | | | Mascot |

| | | | | | | | |
|---|---|---|---|---|---|---|---|
| 23 | novel protein similar to vertebrate complement component 3 (C3) [Danio rerio] | gi\|169153736 | 187400.5 | 8.04 | 19 | 41 | 0 |

Peptide Information

| Calc. Mass | Obsrv. Mass | ± da | ± ppm | Start Seq. | End Seq. | Sequence | Ion Score | C. I. % | Modification | Rank | Result Type |
|---|---|---|---|---|---|---|---|---|---|---|---|
| 842.5206 | 842.5099 | -0.0107 | -13 | 516 | 523 | GKIIQAGR | | | | | Mascot |
| 982.5931 | 982.567 | -0.0261 | -27 | 971 | 979 | LQLDAVVPK | | | | | Mascot |

| | | | | | | | | | | | |
|---|---|---|---|---|---|---|---|---|---|---|---|
| 1088.5591 | 1088.615 | 0.0559 | 51 | 709 | 717 | ARMIEPMPK | | | Oxidation (M)[3] | | Mascot |
| 1097.679 | 1097.6646 | -0.0144 | -13 | 518 | 527 | IIQAGRVNVK | | | | | Mascot |
| 1121.6201 | 1121.5931 | -0.027 | -24 | 1059 | 1067 | EEAIKYIQK | | | | | Mascot |
| 1155.5641 | 1155.6885 | 0.1244 | 108 | 1388 | 1397 | TSSDVEKSFR | | | | | Mascot |
| 1155.5641 | 1155.6885 | 0.1244 | 108 | 1388 | 1397 | TSSDVEKSFR | | | | | Mascot |
| 1179.619 | 1179.5988 | -0.0202 | -17 | 137 | 146 | AEKVMPVSFR | | | Oxidation (M)[5] | | Mascot |
| 1475.7965 | 1475.7527 | -0.0438 | -30 | 1467 | 1478 | EAEIISFRLNQR | | | | | Mascot |
| 1515.8451 | 1515.7451 | -0.1 | -66 | 862 | 875 | STPSVNVVMVILEK | | | | | Mascot |
| 1592.8014 | 1592.814 | 0.0126 | 8 | 343 | 358 | ASVLTSSGSDLVEAEK | | | | | Mascot |
| 1592.8014 | 1592.814 | 0.0126 | 8 | 343 | 358 | ASVLTSSGSDLVEAEK | | | | | Mascot |
| 1608.8632 | 1608.8344 | -0.0288 | -18 | 285 | 299 | GVAYVLFGIEINGEK | | | | | Mascot |
| 1622.7996 | 1622.8505 | 0.0509 | 31 | 1101 | 1114 | VFSMAHSFINVNEK | | | | | Mascot |
| 1622.8033 | 1622.8505 | 0.0472 | 29 | 1186 | 1199 | SRQAAGYLTEHFSR | | | | | Mascot |
| 2142.1965 | 2141.9885 | -0.208 | -97 | 411 | 429 | RPISVHSGTINIHINMPKK | | | | | Mascot |
| 2204.1228 | 2204.1025 | -0.0203 | -9 | 307 | 326 | QIDDVLNGGSVSLTMEEIKR | | | | | Mascot |
| 2217.2349 | 2217.189 | -0.0459 | -21 | 182 | 202 | TISLEIQNPDGIAVHGIGRVK | | | | | Mascot |
| 2218.1797 | 2218.1294 | -0.0503 | -23 | 1 | 19 | MLLQLLLWGTLLCQICDGK | | | Carbamidomethyl (C)[13] | | Mascot |
| 2220.1177 | 2220.1204 | 0.0027 | 1 | 307 | 326 | QIDDVLNGGSVSLTMEEIKR | | | Oxidation (M)[15] | | Mascot |
| 2233.1533 | 2233.082 | -0.0713 | -32 | 911 | 932 | TGELPLQVTAVAASFMGQDAVK | | | | | Mascot |
| 2238.2129 | 2238.1086 | -0.1043 | -47 | 73 | 92 | VVKVTVVVYDYPNSQTALSR | | | | | Mascot |

| | | | | | | | | |
|---|---|---|---|---|---|---|---|---|
| 24 | Zgc:171573 protein [Danio rerio] | gi\|166796220 | 28101.6 | 9.65 | 8 | 41 | 0 | |

**Peptide Information**

| Calc. Mass | Obsrv. Mass | ± da | ± ppm | Start Seq. | End Seq. | Sequence | Ion Score | C. I. % | Modification | Rank | Result Type |
|---|---|---|---|---|---|---|---|---|---|---|---|
| 864.4937 | 864.5085 | 0.0148 | 17 | 30 | 36 | IFSDKVR | | | | | Mascot |
| 1097.5334 | 1097.6646 | 0.1312 | 120 | 2 | 10 | NQVEHLSDR | | | | | Mascot |
| 1121.5255 | 1121.5931 | 0.0676 | 60 | 168 | 176 | KQEENNMTK | | | | | Mascot |
| 1608.9067 | 1608.8344 | -0.0723 | -45 | 127 | 141 | ILPGLVGEDLGQKNR | | | | | Mascot |
| 1625.7046 | 1625.8387 | 0.1341 | 82 | 177 | 189 | SECLWTAELSNCR | | | Carbamidomethyl (C)[3,12] | | Mascot |
| 1711.9701 | 1711.8909 | -0.0792 | -46 | 35 | 50 | VRTIGVDIDALGQQVK | | | | | Mascot |
| 1937.045 | 1936.916 | -0.129 | -67 | 122 | 139 | QEGQRILPGLVGEDLGQK | | | | | Mascot |
| 2211.042 | 2211.1021 | 0.0601 | 27 | 65 | 83 | VYADDLLCSDGIACILEQR | | | Carbamidomethyl (C)[8,14] | | Mascot |

| | | | | | | | | |
|---|---|---|---|---|---|---|---|---|
| 25 | PREDICTED: l-amino-acid oxidase [Danio rerio] | gi\|326674145 | 56155.4 | 6.03 | 10 | 40 | 0 | |

Peptide Information

| Calc. Mass | Obsrv. Mass | ± da | ± ppm | Start Seq. | End Seq. | Sequence | Ion Score | C. I. % | Modification | Rank | Result Type |
|---|---|---|---|---|---|---|---|---|---|---|---|
| 868.4523 | 868.538 | 0.0857 | 99 | 181 | 187 | LQDDLHK | | | | | Mascot |
| 888.4785 | 888.5256 | 0.0471 | 53 | 366 | 373 | SITDLPSR | | | | | Mascot |
| 1088.5582 | 1088.615 | 0.0568 | 52 | 495 | 504 | AADKINDDVK | | | | | Mascot |
| 1121.5739 | 1121.5931 | 0.0192 | 17 | 354 | 362 | FWKNNDGIK | | | | | Mascot |
| 1432.6559 | 1432.6503 | -0.0056 | -4 | 181 | 192 | LQDDLHKMGCEK | | | Oxidation (M)[8] | | Mascot |
| 1448.8583 | 1448.9192 | 0.0609 | 42 | 52 | 67 | HVLIVGAGAAGLTAAK | | | | | Mascot |
| 1475.6722 | 1475.7527 | 0.0805 | 55 | 27 | 38 | CLQDSDYDYLLK | | | | | Mascot |
| 1592.9635 | 1592.814 | -0.1495 | -94 | 108 | 121 | IPSFHRILLAVAQK | | | | | Mascot |
| 1592.9635 | 1592.814 | -0.1495 | -94 | 108 | 121 | IPSFHRILLAVAQK | | | | | Mascot |
| 1625.7489 | 1625.8387 | 0.0898 | 55 | 94 | 107 | NGREGWYAELGAMR | | | Oxidation (M)[13] | | Mascot |
| 2283.1477 | 2283.176 | 0.0283 | 12 | 317 | 336 | ATLFFDFNPPLSADKMEA LR | | | | | Mascot |
| 2283.1477 | 2283.176 | 0.0283 | 12 | 317 | 336 | ATLFFDFNPPLSADKMEA LR | | | | | Mascot |
| 2299.1428 | 2299.1831 | 0.0403 | 18 | 317 | 336 | ATLFFDFNPPLSADKMEA LR | | | Oxidation (M)[16] | | Mascot |

26 PREDICTED: caspase-8-like [Danio rerio] gi|125845888 39333.1 7.51 10 39 0

Peptide Information

| Calc. Mass | Obsrv. Mass | ± da | ± ppm | Start Seq. | End Seq. | Sequence | Ion Score | C. I. % | Modification | Rank | Result Type |
|---|---|---|---|---|---|---|---|---|---|---|---|
| 996.5043 | 996.5963 | 0.092 | 92 | 296 | 303 | NLEKHCPR | | | | | Mascot |
| 1121.5334 | 1121.5931 | 0.0597 | 53 | 272 | 281 | STTNGHVSYR | | | | | Mascot |
| 1164.5565 | 1164.5659 | 0.0094 | 8 | 5 | 14 | ESVDKICENK | | | | | Mascot |
| 1179.5826 | 1179.5988 | 0.0162 | 14 | 207 | 216 | DPFTGVKCQK | | | Carbamidomethyl (C)[8] | | Mascot |
| 1382.6521 | 1382.6827 | 0.0306 | 22 | 285 | 295 | EGSWFIQSLCR | | | Carbamidomethyl (C)[10] | | Mascot |
| 1448.793 | 1448.9192 | 0.1262 | 87 | 327 | 338 | QMPIYEVALTRK | | | | | Mascot |
| 1493.7126 | 1493.7314 | 0.0188 | 13 | 80 | 91 | QDCVLENFPMLK | | | Carbamidomethyl (C)[3] | | Mascot |
| 1954.9989 | 1954.8737 | -0.1252 | -64 | 223 | 240 | LFFIQGCRGANSLQACVK | | | | | Mascot |
| 2204.1082 | 2204.1025 | -0.0057 | -3 | 252 | 271 | VEGDNLDITIPSDTDFLIA R | | | | | Mascot |
| 2627.3608 | 2627.3674 | 0.0066 | 3 | 300 | 322 | HCPRGADILTILLYVNNEV SSQK | | | Carbamidomethyl (C)[2] | | Mascot |

27 protein aurora borealis [Danio rerio] gi|55925389 60297.1 5.05 8 39 0 16 0

Peptide Information

| Calc. Mass | Obsrv. Mass | ± da | ± ppm | Start Seq. | End Seq. | Sequence | Ion Score | C. I. % | Modification | Rank | Result Type |
|---|---|---|---|---|---|---|---|---|---|---|---|

| Calc. Mass | Obsrv. Mass | ± da | ± ppm | Start Seq. | End Seq. | Sequence | Ion Score | C. I. % | Modification | Rank | Result Type |
|---|---|---|---|---|---|---|---|---|---|---|---|
| 882.5407 | 882.5657 | 0.025 | 28 | 278 | 285 | SPILSPIR | | | | | Mascot |
| 1023.5469 | 1023.5066 | -0.0403 | -39 | 48 | 57 | SSSATPAKFK | | | | | Mascot |
| 1121.5885 | 1121.5931 | 0.0046 | 4 | 121 | 129 | QSMKLPHHK | | | Oxidation (M)[3] | | Mascot |
| 1164.5579 | 1164.5659 | 0.008 | 7 | 334 | 343 | SCSPFQSRPR | | | | | Mascot |
| 1434.7019 | 1434.7253 | 0.0234 | 16 | 334 | 345 | SCSPFQSRPRNR | | | | | Mascot |
| 1458.7546 | 1458.7205 | -0.0341 | -23 | 286 | 298 | LQQSVTPESGERK | | | | | Mascot |
| 1458.7546 | 1458.7205 | -0.0341 | -23 | 286 | 298 | LQQSVTPESGERK | 16 | 0 | | | Mascot |
| 1954.9504 | 1954.8737 | -0.0767 | -39 | 79 | 95 | QAVFFSQARADSEIEEK | | | | | Mascot |
| 2194.1826 | 2194.0884 | -0.0942 | -43 | 278 | 297 | SPILSPIRLQQSVTPESGE R | | | | | Mascot |

| | | | | | | | |
|---|---|---|---|---|---|---|---|
| 28 | gamma-soluble NSF attachment protein [Danio rerio] | gi\|61806530 | 33925.8 | 5.45 | 9 | 39 | 0 |

**Peptide Information**

| Calc. Mass | Obsrv. Mass | ± da | ± ppm | Start Seq. | End Seq. | Sequence | Ion Score | C. I. % | Modification | Rank | Result Type |
|---|---|---|---|---|---|---|---|---|---|---|---|
| 1045.5598 | 1045.5631 | 0.0033 | 3 | 123 | 131 | LMEPLDLSK | | | | | Mascot |
| 1279.7303 | 1279.7744 | 0.0441 | 34 | 196 | 206 | CIAQVLVQLHR | | | | | Mascot |
| 1463.7489 | 1463.8929 | 0.144 | 98 | 166 | 178 | QQKFDEAAASLQK | | | | | Mascot |
| 1592.8213 | 1592.814 | -0.0073 | -5 | 1 | 14 | MAQKISEAHEHIAK | | | | | Mascot |
| 1592.8213 | 1592.814 | -0.0073 | -5 | 1 | 14 | MAQKISEAHEHIAK | | | | | Mascot |
| 1608.8163 | 1608.8344 | 0.0181 | 11 | 1 | 14 | MAQKISEAHEHIAK | | | Oxidation (M)[1] | | Mascot |
| 2126.1538 | 2125.9878 | -0.166 | -78 | 196 | 214 | CIAQVLVQLHRADYVAAQ K | | | | | Mascot |
| 2142.115 | 2141.9885 | -0.1265 | -59 | 255 | 273 | SPLVTYMDNDYAKLAISL K | | | | | Mascot |
| 2218.0815 | 2218.1294 | 0.0479 | 22 | 26 | 45 | WKPDYDSAASEYAKAAV AFK | | | | | Mascot |
| 2225.022 | 2225.1177 | 0.0957 | 43 | 21 | 39 | TSFMKWKPDYDSAASEY AK | | | | | Mascot |
| 2225.022 | 2225.1177 | 0.0957 | 43 | 21 | 39 | TSFMKWKPDYDSAASEY AK | | | | | Mascot |
| 2299.1062 | 2299.1831 | 0.0769 | 33 | 49 | 68 | QLEQAKDAYLQEAEAHA NNR | | | | | Mascot |

| | | | | | | | |
|---|---|---|---|---|---|---|---|
| 29 | PREDICTED: cyclin-dependent kinase 2-associated protein 1-like [Danio rerio] | gi\|326671486 | 9491 | 9.3 | 5 | 39 | 0 |

**Peptide Information**

| Calc. Mass | Obsrv. Mass | ± da | ± ppm | Start Seq. | End Seq. | Sequence | Ion Score | C. I. % | Modification | Rank | Result Type |
|---|---|---|---|---|---|---|---|---|---|---|---|
| 850.4451 | 850.5413 | 0.0962 | 113 | 58 | 64 | SAMERLK | | | Oxidation (M)[3] | | Mascot |
| 950.4247 | 950.4852 | 0.0605 | 64 | 76 | 83 | ECLAETER | | | | | Mascot |
| 1121.595 | 1121.5931 | -0.0019 | -2 | 48 | 57 | EIRPTYAGSK | | | | | Mascot |
| 1432.7212 | 1432.6503 | -0.0709 | -49 | 72 | 83 | GLVRECLAETER | | | Carbamidomethyl (C)[6] | | Mascot |

| | | | | | | | | | | | |
|---|---|---|---|---|---|---|---|---|---|---|---|
| 1695.8483 | 1695.8931 | 0.0448 | 26 | 48 | 62 | EIRPTYAGSKSAMER | | | | | Mascot |
| 1711.8431 | 1711.8909 | 0.0478 | 28 | 48 | 62 | EIRPTYAGSKSAMER | | | Oxidation (M)[13] | | Mascot |

| | | | | | | | |
|---|---|---|---|---|---|---|---|
| 30 | ADP/ATP translocase 1 [Danio rerio] | gi\|47550717 | 32667.2 | 9.74 | 8 | 37 | 0 |

Peptide Information

| Calc. Mass | Obsrv. Mass | ± da | ± ppm | Start Seq. | End Seq. | Sequence | Ion Score | C. I. % | Modification | Rank | Result Type |
|---|---|---|---|---|---|---|---|---|---|---|---|
| 950.4764 | 950.4852 | 0.0088 | 9 | 253 | 260 | GTIDCWKK | | | | | Mascot |
| 996.4536 | 996.5963 | 0.1427 | 143 | 237 | 244 | RMMMQSGR | | | | | Mascot |
| 1082.5551 | 1082.5773 | 0.0222 | 21 | 1 | 10 | MSDAAISFLK | | | | | Mascot |
| 1097.5297 | 1097.6646 | 0.1349 | 123 | 44 | 52 | QITADMQYK | | | | | Mascot |
| 1622.7954 | 1622.8505 | 0.0551 | 34 | 148 | 163 | GAAEREFTGLGNCVAK | | | | | Mascot |
| 1622.7954 | 1622.8505 | 0.0551 | 34 | 148 | 163 | GAAEREFTGLGNCVAK | | | | | Mascot |
| 1689.8635 | 1689.9281 | 0.0646 | 38 | 81 | 94 | YFPTQALNFAFKDK | | | | | Mascot |
| 2283.2053 | 2283.176 | -0.0293 | -13 | 1 | 23 | MSDAAISFLKDFLAGGVAAAISK | | | | | Mascot |
| 2283.2053 | 2283.176 | -0.0293 | -13 | 1 | 23 | MSDAAISFLKDFLAGGVAAAISK | | | | | Mascot |
| 2284.2236 | 2284.1724 | -0.0512 | -22 | 73 | 92 | GNLANVIRYFPTQALNFAFK | | | | | Mascot |
| 2299.2002 | 2299.1831 | -0.0171 | -7 | 1 | 23 | MSDAAISFLKDFLAGGVAAAISK | | | Oxidation (M)[1] | | Mascot |

| | | | | | | | |
|---|---|---|---|---|---|---|---|
| 31 | novel protein similar to H.sapiens TTN, titin (TTN) [Danio rerio] | gi\|169154823 | 842970 | 5.81 | 42 | 36 | 0 |

Peptide Information

| Calc. Mass | Obsrv. Mass | ± da | ± ppm | Start Seq. | End Seq. | Sequence | Ion Score | C. I. % | Modification | Rank | Result Type |
|---|---|---|---|---|---|---|---|---|---|---|---|
| 842.444 | 842.5099 | 0.0659 | 78 | 3102 | 3108 | SYKVSMK | | | | | Mascot |
| 850.4814 | 850.5413 | 0.0599 | 70 | 1088 | 1094 | LTSVRMK | | | Oxidation (M)[6] | | Mascot |
| 870.4203 | 870.5364 | 0.1161 | 133 | 6420 | 6427 | LSDSGTYK | | | | | Mascot |
| 882.4679 | 882.5657 | 0.0978 | 111 | 2483 | 2489 | YKVSTER | | | | | Mascot |
| 996.536 | 996.5963 | 0.0603 | 61 | 2586 | 2593 | LTITETYR | | | | | Mascot |
| 1023.5217 | 1023.5066 | -0.0151 | -15 | 6866 | 6874 | DIRTGGNYK | | | | | Mascot |
| 1045.5347 | 1045.5631 | 0.0284 | 27 | 6656 | 6665 | CQIGGTPEIK | | | | | Mascot |
| 1051.5571 | 1051.7139 | 0.1568 | 149 | 6666 | 6674 | ISWFKADGK | | | | | Mascot |
| 1088.631 | 1088.615 | -0.016 | -15 | 6362 | 6371 | LKDTQSIVGK | | | | | Mascot |
| 1115.5367 | 1115.6925 | 0.1558 | 140 | 1711 | 1719 | EDQGKYTFK | | | | | Mascot |
| 1115.6418 | 1115.6925 | 0.0507 | 45 | 2536 | 2544 | EITVLEKQR | | | | | Mascot |
| 1121.5771 | 1121.5931 | 0.016 | 14 | 1241 | 1250 | CRVTGYPTPK | | | | | Mascot |

| | | | | | | | | |
|---|---|---|---|---|---|---|---|---|
| 1155.6521 | 1155.6885 | 0.0364 | 31 | 980 | 989 | YPHIKIEGAK | | Mascot |
| 1155.6521 | 1155.6885 | 0.0364 | 31 | 980 | 989 | YPHIKIEGAK | | Mascot |
| 1279.6376 | 1279.7744 | 0.1368 | 107 | 3711 | 3722 | SETTTAVVSEQK | | Mascot |
| 1432.7213 | 1432.6503 | -0.071 | -50 | 7324 | 7335 | RMEQTEPVTVAR | Oxidation (M)[2] | Mascot |
| 1458.7838 | 1458.7205 | -0.0633 | -43 | 5831 | 5843 | ISGSLPITVEWEK | | Mascot |
| 1458.8049 | 1458.7205 | -0.0844 | -58 | 3261 | 3273 | AEPLSTLETEKIK | | Mascot |
| 1463.8944 | 1463.8929 | -0.0015 | -1 | 123 | 136 | LDVRVTGIPAPVVK | | Mascot |
| 1475.804 | 1475.7527 | -0.0513 | -35 | 1964 | 1976 | GKHYSLTIINAMK | | Mascot |
| 1477.7645 | 1477.9099 | 0.1454 | 98 | 4285 | 4298 | SFESTTVAVGNPLR | | Mascot |
| 1493.6438 | 1493.7314 | 0.0876 | 59 | 5126 | 5138 | YTCHAQNEAGSQK | Carbamidomethyl (C)[3] | Mascot |
| 1555.7209 | 1555.749 | 0.0281 | 18 | 1587 | 1599 | WMKNNAEIFSGDK | Oxidation (M)[2] | Mascot |
| 1592.7697 | 1592.814 | 0.0443 | 28 | 5321 | 5335 | NQAGSDQCSGTLKVK | Carbamidomethyl (C)[8] | Mascot |
| 1592.7697 | 1592.814 | 0.0443 | 28 | 5321 | 5335 | NQAGSDQCSGTLKVK | Carbamidomethyl (C)[8] | Mascot |
| 1622.6752 | 1622.8505 | 0.1753 | 108 | 2945 | 2958 | KDYEGEYSCTASNR | | Mascot |
| 1622.7876 | 1622.8505 | 0.0629 | 39 | 4732 | 4746 | VGKEQCSCSINVLDK | | Mascot |
| 1625.7258 | 1625.8387 | 0.1129 | 69 | 5508 | 5522 | NEAGSESCSMDLRVK | | Mascot |
| 1666.7238 | 1666.8771 | 0.1533 | 92 | 6892 | 6907 | ADSGRYSCHASNDVGK | | Mascot |
| 1689.9242 | 1689.9281 | 0.0039 | 2 | 207 | 221 | TIISASQISQTRQTR | | Mascot |
| 1695.958 | 1695.8931 | -0.0649 | -38 | 833 | 846 | LYKPVFVQKPSSFR | | Mascot |
| 1699.8538 | 1699.9332 | 0.0794 | 47 | 1552 | 1566 | QVVSFTQTLEDAYAK | | Mascot |
| 1699.8538 | 1699.9332 | 0.0794 | 47 | 1552 | 1566 | QVVSFTQTLEDAYAK | | Mascot |
| 2125.9932 | 2125.9878 | -0.0054 | -3 | 2455 | 2472 | AVFECELSEPNVQVTWM K | Oxidation (M)[17] | Mascot |
| 2194.1101 | 2194.0884 | -0.0217 | -10 | 742 | 761 | LPVVLPEDEGIYTAFASN MK | | Mascot |
| 2203.9885 | 2204.1025 | 0.114 | 52 | 1567 | 1584 | EKDTMVTFECETNEPFVK | Carbamidomethyl (C)[10] | Mascot |
| 2211.1438 | 2211.1021 | -0.0417 | -19 | 6494 | 6513 | SCEIALRGDTAILELHNID K | | Mascot |
| 2216.9626 | 2217.189 | 0.2264 | 102 | 1132 | 1149 | MINEFGYCSLDYEVAYSR | Carbamidomethyl (C)[8] | Mascot |
| 2218.0696 | 2218.1294 | 0.0598 | 27 | 5489 | 5507 | LEIISSDIKDSGVYYCEAR | Carbamidomethyl (C)[16] | Mascot |
| 2219.9834 | 2220.1204 | 0.137 | 62 | 1567 | 1584 | EKDTMVTFECETNEPFVK | Carbamidomethyl (C)[10], Oxidation (M)[5] | Mascot |
| 2232.9575 | 2233.082 | 0.1245 | 56 | 1132 | 1149 | MINEFGYCSLDYEVAYSR | Carbamidomethyl (C)[8], Oxidation (M)[1] | Mascot |
| 2238.0933 | 2238.1086 | 0.0153 | 7 | 2454 | 2472 | KAVFECELSEPNVQVTW MK | | Mascot |
| 2269.1724 | 2269.2627 | 0.0903 | 40 | 2563 | 2581 | NGVEIQFSVDQRFNYAIIR | | Mascot |
| 2269.1724 | 2269.2627 | 0.0903 | 40 | 2563 | 2581 | NGVEIQFSVDQRFNYAIIR | | Mascot |
| 2283.104 | 2283.176 | 0.072 | 32 | 2409 | 2430 | NVHFGDVGDYKFVAGSA ETAAK | | Mascot |
| 2283.1899 | 2283.176 | -0.0139 | -6 | 3240 | 3260 | EHPSQVMSALTESVTPLT IVK | Oxidation (M)[7] | Mascot |
| 2297.1516 | 2297.1938 | 0.0422 | 18 | 6550 | 6570 | NLSVEMGKSLILECTYAG SPK | Carbamidomethyl (C)[14] | Mascot |

| Calc. Mass | Obsrv. Mass | ± da | ± ppm | Start Seq. | End Seq. | Sequence | Ion Score | C. I. % | Modification | Rank | Result Type |
|---|---|---|---|---|---|---|---|---|---|---|---|
| 3337.4834 | 3337.7656 | 0.2822 | 85 | 4346 | 4377 | DTGTYTCTAANEAGSSSCSSSVTVQEPPVFVK | | | Carbamidomethyl (C)[7,18] | | Mascot |

| | | | | | | | | | |
|---|---|---|---|---|---|---|---|---|---|
| 32 | butyrophilin-like [Danio rerio] | gi\|168229192 | 30370 | 5.21 | 7 | 34 | 0 | | |

Peptide Information

| Calc. Mass | Obsrv. Mass | ± da | ± ppm | Start Seq. | End Seq. | Sequence | Ion Score | C. I. % | Modification | Rank | Result Type |
|---|---|---|---|---|---|---|---|---|---|---|---|
| 888.4672 | 888.5256 | 0.0584 | 66 | 83 | 90 | ESLSPDLK | | | | | Mascot |
| 1023.4537 | 1023.5066 | 0.0529 | 52 | 152 | 160 | HGNHCGKDR | | | | | Mascot |
| 1043.5779 | 1043.4567 | -0.1212 | -116 | 220 | 227 | HLLTMRTR | | | Oxidation (M)[5] | | Mascot |
| 1082.559 | 1082.5773 | 0.0183 | 17 | 74 | 82 | HAEQITIDR | | | | | Mascot |
| 1689.7496 | 1689.9281 | 0.1785 | 106 | 47 | 61 | QAGTDCENGPVEERK | | | Carbamidomethyl (C)[6] | | Mascot |
| 2218.3647 | 2218.1294 | -0.2353 | -106 | 17 | 37 | ALFITVIICALLGLAAVILYK | | | | | Mascot |
| 2233.0217 | 2233.082 | 0.0603 | 27 | 248 | 267 | IVDCPVEEDSPAEDTEPFLK | | | | | Mascot |

| | | | | | | | | | |
|---|---|---|---|---|---|---|---|---|---|
| 33 | PREDICTED: zinc finger protein 845-like isoform 1 [Danio rerio] | gi\|326664549 | 110747.2 | 8.71 | 12 | 34 | 0 | 16 | 0 |

Peptide Information

| Calc. Mass | Obsrv. Mass | ± da | ± ppm | Start Seq. | End Seq. | Sequence | Ion Score | C. I. % | Modification | Rank | Result Type |
|---|---|---|---|---|---|---|---|---|---|---|---|
| 996.5546 | 996.5963 | 0.0417 | 42 | 692 | 700 | CFGTKISLK | | | | | Mascot |
| 1043.5844 | 1043.4567 | -0.1277 | -122 | 220 | 229 | SSSGKPQKPK | | | | | Mascot |
| 1088.5483 | 1088.615 | 0.0667 | 61 | 890 | 898 | THTGERPYK | | | | | Mascot |
| 1115.6418 | 1115.6925 | 0.0507 | 45 | 39 | 48 | EVASLEAKLR | | | | | Mascot |
| 1115.6418 | 1115.6925 | 0.0507 | 45 | 39 | 48 | EVASLEAKLR | 16 | 0 | | | Mascot |
| 1121.552 | 1121.5931 | 0.0411 | 37 | 905 | 913 | CFSRPEAVR | | | Carbamidomethyl (C)[1] | | Mascot |
| 1711.8328 | 1711.8909 | 0.0581 | 34 | 773 | 787 | QALQCSYCPKLLCNK | | | | | Mascot |
| 2216.9924 | 2217.189 | 0.1966 | 89 | 590 | 607 | HERTHTGEKPYQCSQCQK | | | Carbamidomethyl (C)[13] | | Mascot |
| 2220.0283 | 2220.1204 | 0.0921 | 41 | 220 | 238 | SSSGKPQKPKNYHCEQCGK | | | Carbamidomethyl (C)[14,17] | | Mascot |
| 2225.0227 | 2225.1177 | 0.095 | 43 | 673 | 691 | THEIIHTGEKPYQCSHCNK | | | | | Mascot |
| 2225.0227 | 2225.1177 | 0.095 | 43 | 729 | 747 | THEIIHTGEKPYQCSHCNK | | | | | Mascot |
| 2269.0059 | 2269.2627 | 0.2568 | 113 | 313 | 332 | THTGARPYQCSHCQKSFCSK | | | | | Mascot |
| 2269.0059 | 2269.2627 | 0.2568 | 113 | 313 | 332 | THTGARPYQCSHCQKSFCSK | | | | | Mascot |
| 2284.0386 | 2284.1724 | 0.1338 | 59 | 919 | 937 | HTGEKPYHCSLCSERFSFR | | | | | Mascot |
| 2627.2388 | 2627.3674 | 0.1286 | 49 | 246 | 266 | LMIHIRAHTGERPFQCNQCDK | | | Carbamidomethyl (C)[16,19], Oxidation (M)[2] | | Mascot |

| | | | | | | | |
|---|---|---|---|---|---|---|---|
| 34 | probable E3 ubiquitin-protein ligase MYCBP2 [Danio rerio] | gi\|302393529 | 501527.2 | 6.49 | 27 | 33 | 0 |

## Peptide Information

| Calc. Mass | Obsrv. Mass | ± da | ± ppm | Start Seq. | End Seq. | Sequence | Ion Score | C. I. % | Modification | Rank | Result Type |
|---|---|---|---|---|---|---|---|---|---|---|---|
| 850.4702 | 850.5413 | 0.0711 | 84 | 3680 | 3687 | SITISCVK | | | | | Mascot |
| 870.4103 | 870.5364 | 0.1261 | 145 | 4446 | 4453 | AYFGGEAR | | | | | Mascot |
| 950.4173 | 950.4852 | 0.0679 | 71 | 898 | 906 | DGSSERGDK | | | | | Mascot |
| 982.4952 | 982.567 | 0.0718 | 73 | 378 | 387 | VGSGYSGTVR | | | | | Mascot |
| 1043.5745 | 1043.4567 | -0.1178 | -113 | 4193 | 4200 | THQRQVFK | | | | | Mascot |
| 1082.639 | 1082.5773 | -0.0617 | -57 | 2786 | 2794 | KVPLMEPLR | | | | | Mascot |
| 1115.599 | 1115.6925 | 0.0935 | 84 | 2379 | 2387 | RPSENMLIR | | | | | Mascot |
| 1115.599 | 1115.6925 | 0.0935 | 84 | 2379 | 2387 | RPSENMLIR | 13 | 0 | | | Mascot |
| 1121.5619 | 1121.5931 | 0.0312 | 28 | 2304 | 2312 | SIQQENMKK | | | Oxidation (M)[7] | | Mascot |
| 1155.5901 | 1155.6885 | 0.0984 | 85 | 3708 | 3717 | VTSMIFLCGK | | | Carbamidomethyl (C)[8] | | Mascot |
| 1155.5901 | 1155.6885 | 0.0984 | 85 | 3708 | 3717 | VTSMIFLCGK | | | Carbamidomethyl (C)[8] | | Mascot |
| 1179.7096 | 1179.5988 | -0.1108 | -94 | 2797 | 2807 | STSPKPKLPPK | | | | | Mascot |
| 1279.7004 | 1279.7744 | 0.074 | 58 | 1383 | 1393 | QQTSEPVHILK | | | | | Mascot |
| 1382.6434 | 1382.6827 | 0.0393 | 28 | 2772 | 2783 | SDSYTLDPDTLR | | | | | Mascot |
| 1432.6274 | 1432.6503 | 0.0229 | 16 | 4077 | 4089 | DMAAGHLSDAWSR | | | Oxidation (M)[2] | | Mascot |
| 1448.7347 | 1448.9192 | 0.1845 | 127 | 1087 | 1098 | CLLINKLDGSCR | | | Carbamidomethyl (C)[1,11] | | Mascot |
| 1458.6174 | 1458.7205 | 0.1031 | 71 | 1131 | 1143 | EMCCYNAVIADAR | | | | | Mascot |
| 1458.7223 | 1458.7205 | -0.0018 | -1 | 1210 | 1221 | VYSKEDYSVVNR | | | | | Mascot |
| 1475.7675 | 1475.7527 | -0.0148 | -10 | 4401 | 4412 | ALMRLEYEGLHK | | | Oxidation (M)[3] | | Mascot |
| 1515.6389 | 1515.7451 | 0.1062 | 70 | 1131 | 1143 | EMCCYNAVIADAR | | | Carbamidomethyl (C)[3] | | Mascot |
| 1555.7858 | 1555.749 | -0.0368 | -24 | 2147 | 2161 | ELAYLGSVCAAALMK | | | Oxidation (M)[14] | | Mascot |
| 1592.6688 | 1592.814 | 0.1452 | 91 | 739 | 751 | EEKSMMCQPGMHK | | | Carbamidomethyl (C)[7] | | Mascot |
| 1592.6688 | 1592.814 | 0.1452 | 91 | 739 | 751 | EEKSMMCQPGMHK | | | Carbamidomethyl (C)[7] | | Mascot |
| 1608.6637 | 1608.8344 | 0.1707 | 106 | 739 | 751 | EEKSMMCQPGMHK | | | Carbamidomethyl (C)[7], Oxidation (M)[5] | | Mascot |
| 1625.839 | 1625.8387 | -0.0003 | 0 | 3703 | 3717 | DIGNKVTSMIFLCGK | | | | | Mascot |
| 1699.7592 | 1699.9332 | 0.174 | 102 | 2969 | 2983 | NSLSCQQGFDEKESK | | | | | Mascot |
| 1699.7592 | 1699.9332 | 0.174 | 102 | 2969 | 2983 | NSLSCQQGFDEKESK | | | | | Mascot |
| 2204.2544 | 2204.1025 | -0.1519 | -69 | 3999 | 4018 | LGILDMFLGCIAKALTVQL K | | | Carbamidomethyl (C)[10] | | Mascot |
| 2210.9666 | 2211.1021 | 0.1355 | 61 | 4239 | 4258 | EHTGKPASSSSDACRFC GTR | | | Carbamidomethyl (C)[14,17] | | Mascot |
| 2217.155 | 2217.189 | 0.034 | 15 | 2244 | 2262 | WLQPDSYADPQKTSLILN K | | | | | Mascot |
| 2220.2493 | 2220.1204 | -0.1289 | -58 | 3999 | 4018 | LGILDMFLGCIAKALTVQL K | | | Carbamidomethyl (C)[10], Oxidation (M)[6] | | Mascot |
| 2233.0837 | 2233.082 | -0.0017 | -1 | 626 | 646 | LETKMAVYTACNNGSSSI | | | Oxidation (M)[5] | | Mascot |

| Calc. Mass | Obsrv. Mass | ± da | ± ppm | Start Seq. | End Seq. | Sequence | Ion Score | C. I. % | Modification | Rank | Result Type |
|---|---|---|---|---|---|---|---|---|---|---|---|
| | | | | | | VTK | | | | | |
| 2269.2273 | 2269.2627 | 0.0354 | 16 | 863 | 882 | GPLVFPGPLFMNQREQV LAR | | | | | Mascot |
| 2269.2273 | 2269.2627 | 0.0354 | 16 | 863 | 882 | GPLVFPGPLFMNQREQV LAR | | | | | Mascot |
| 3337.3352 | 3337.7656 | 0.4304 | 129 | 4495 | 4522 | CRYCCSVAVFFCFGTTH FCNACHDDFQR | | | Carbamidomethyl (C)[1] | | Mascot |

| | | | | | | | | |
|---|---|---|---|---|---|---|---|---|
| 35 | protein tyrosine kinase [Danio rerio] | gi\|3093296 | 81950.7 | 6.66 | 12 | 33 | 0 | |

Peptide Information

| Calc. Mass | Obsrv. Mass | ± da | ± ppm | Start Seq. | End Seq. | Sequence | Ion Score | C. I. % | Modification | Rank | Result Type |
|---|---|---|---|---|---|---|---|---|---|---|---|
| 850.4668 | 850.5413 | 0.0745 | 88 | 574 | 581 | IGDFGLTK | | | | | Mascot |
| 1023.475 | 1023.5066 | 0.0316 | 31 | 267 | 275 | MVHGYVCSK | | | | | Mascot |
| 1088.4895 | 1088.615 | 0.1255 | 115 | 585 | 593 | DNEGYYTVK | | | | | Mascot |
| 1155.623 | 1155.6885 | 0.0655 | 57 | 313 | 322 | IPWIAPECVK | | | | | Mascot |
| 1155.623 | 1155.6885 | 0.0655 | 57 | 313 | 322 | IPWIAPECVK | | | | | Mascot |
| 1164.7211 | 1164.5659 | -0.1552 | -133 | 176 | 186 | VVLKVLGSGHR | | | | | Mascot |
| 1448.822 | 1448.9192 | 0.0972 | 67 | 75 | 86 | ELLEHLQGQLLR | | | | | Mascot |
| 1689.8927 | 1689.9281 | 0.0354 | 21 | 267 | 281 | MVHGYVCSKNILVAR | | | | | Mascot |
| 1936.9988 | 1936.916 | -0.0828 | -43 | 471 | 485 | EEQSNNLWREIHILR | | | | | Mascot |
| 2218.063 | 2218.1294 | 0.0664 | 30 | 534 | 552 | TLLNYSVQICQGMDYLGS R | | | Carbamidomethyl (C)[10] | | Mascot |
| 2284.1528 | 2284.1724 | 0.0196 | 9 | 408 | 427 | QNPSIQPVPMLEVDPTVF EK | | | Oxidation (M)[10] | | Mascot |
| 2500.2217 | 2500.1299 | -0.0918 | -37 | 334 | 354 | WSFGTTLWEICYNGEIPL KDK | | | | | Mascot |
| 2627.2268 | 2627.3674 | 0.1406 | 54 | 217 | 237 | HQENIMVEEFVQYGPLDL FMR | | | Oxidation (M)[6,20] | | Mascot |

| | | | | | | | | |
|---|---|---|---|---|---|---|---|---|
| 36 | Esrom [Danio rerio] | gi\|56961715 | 501488.2 | 6.51 | 27 | 33 | 0 | |

Peptide Information

| Calc. Mass | Obsrv. Mass | ± da | ± ppm | Start Seq. | End Seq. | Sequence | Ion Score | C. I. % | Modification | Rank | Result Type |
|---|---|---|---|---|---|---|---|---|---|---|---|
| 850.4702 | 850.5413 | 0.0711 | 84 | 3680 | 3687 | SITISCVK | | | | | Mascot |
| 870.4103 | 870.5364 | 0.1261 | 145 | 4446 | 4453 | AYFGGEAR | | | | | Mascot |
| 950.4173 | 950.4852 | 0.0679 | 71 | 898 | 906 | DGSSERGDK | | | | | Mascot |
| 982.4952 | 982.567 | 0.0718 | 73 | 378 | 387 | VGSGYSGTVR | | | | | Mascot |
| 1043.5745 | 1043.4567 | -0.1178 | -113 | 4193 | 4200 | THQRQVFK | | | | | Mascot |
| 1082.639 | 1082.5773 | -0.0617 | -57 | 2786 | 2794 | KVPLMEPLR | | | | | Mascot |
| 1115.599 | 1115.6925 | 0.0935 | 84 | 2379 | 2387 | RPSENMLIR | | | | | Mascot |
| 1115.599 | 1115.6925 | 0.0935 | 84 | 2379 | 2387 | RPSENMLIR | 13 | 0 | | | Mascot |
| 1121.5619 | 1121.5931 | 0.0312 | 28 | 2304 | 2312 | SIQQENMKK | | | Oxidation (M)[7] | | Mascot |

| | | | | | | | | | | | |
|---|---|---|---|---|---|---|---|---|---|---|---|
| 1155.5901 | 1155.6885 | 0.0984 | 85 | 3708 | 3717 | VTSMIFLCGK | | | Carbamidomethyl (C)[8] | | Mascot |
| 1155.5901 | 1155.6885 | 0.0984 | 85 | 3708 | 3717 | VTSMIFLCGK | | | Carbamidomethyl (C)[8] | | Mascot |
| 1179.7096 | 1179.5988 | -0.1108 | -94 | 2797 | 2807 | STSPKPKLPPK | | | | | Mascot |
| 1279.7004 | 1279.7744 | 0.074 | 58 | 1383 | 1393 | QQTSEPVHILK | | | | | Mascot |
| 1382.6434 | 1382.6827 | 0.0393 | 28 | 2772 | 2783 | SDSYTLDPDTLR | | | | | Mascot |
| 1432.6274 | 1432.6503 | 0.0229 | 16 | 4077 | 4089 | DMAAGHLSDAWSR | | | Oxidation (M)[2] | | Mascot |
| 1448.7347 | 1448.9192 | 0.1845 | 127 | 1087 | 1098 | CLLINKLDGSCR | | | Carbamidomethyl (C)[1,11] | | Mascot |
| 1458.6174 | 1458.7205 | 0.1031 | 71 | 1131 | 1143 | EMCCYNAVIADAR | | | | | Mascot |
| 1458.7223 | 1458.7205 | -0.0018 | -1 | 1210 | 1221 | VYSKEDYSVVNR | | | | | Mascot |
| 1475.7675 | 1475.7527 | -0.0148 | -10 | 4401 | 4412 | ALMRLEYEGLHK | | | Oxidation (M)[3] | | Mascot |
| 1515.6389 | 1515.7451 | 0.1062 | 70 | 1131 | 1143 | EMCCYNAVIADAR | | | Carbamidomethyl (C)[3] | | Mascot |
| 1555.7858 | 1555.749 | -0.0368 | -24 | 2147 | 2161 | ELAYLGSVCAAALMK | | | Oxidation (M)[14] | | Mascot |
| 1592.6688 | 1592.814 | 0.1452 | 91 | 739 | 751 | EEKSMMCQPGMHK | | | Carbamidomethyl (C)[7] | | Mascot |
| 1592.6688 | 1592.814 | 0.1452 | 91 | 739 | 751 | EEKSMMCQPGMHK | | | Carbamidomethyl (C)[7] | | Mascot |
| 1608.6637 | 1608.8344 | 0.1707 | 106 | 739 | 751 | EEKSMMCQPGMHK | | | Carbamidomethyl (C)[7], Oxidation (M)[5] | | Mascot |
| 1625.839 | 1625.8387 | -0.0003 | 0 | 3703 | 3717 | DIGNKVTSMIFLCGK | | | | | Mascot |
| 1699.7592 | 1699.9332 | 0.174 | 102 | 2969 | 2983 | NSLSCQQGFDEKESK | | | | | Mascot |
| 1699.7592 | 1699.9332 | 0.174 | 102 | 2969 | 2983 | NSLSCQQGFDEKESK | | | | | Mascot |
| 2204.2544 | 2204.1025 | -0.1519 | -69 | 3999 | 4018 | LGILDMFLGCIAKALTVQLK | | | Carbamidomethyl (C)[10] | | Mascot |
| 2210.9666 | 2211.1021 | 0.1355 | 61 | 4239 | 4258 | EHTGKPASSSSDACRFCGTR | | | Carbamidomethyl (C)[14,17] | | Mascot |
| 2217.155 | 2217.189 | 0.034 | 15 | 2244 | 2262 | WLQPDSYADPQKTSLILNK | | | | | Mascot |
| 2220.2493 | 2220.1204 | -0.1289 | -58 | 3999 | 4018 | LGILDMFLGCIAKALTVQLK | | | Carbamidomethyl (C)[10], Oxidation (M)[6] | | Mascot |
| 2233.0837 | 2233.082 | -0.0017 | -1 | 626 | 646 | LETKMAVYTACNNGSSSIVTK | | | Oxidation (M)[5] | | Mascot |
| 2269.2273 | 2269.2627 | 0.0354 | 16 | 863 | 882 | GPLVFPGPLFMNQREQVLAR | | | | | Mascot |
| 2269.2273 | 2269.2627 | 0.0354 | 16 | 863 | 882 | GPLVFPGPLFMNQREQVLAR | | | | | Mascot |
| 3337.3352 | 3337.7656 | 0.4304 | 129 | 4495 | 4522 | CRYCCSVAVFFCFGTTHFCNACHDDFQR | | | Carbamidomethyl (C)[1] | | Mascot |

| | | | | | | | |
|---|---|---|---|---|---|---|---|
| 37 | novel protein similar to a human protein associated with Myc [Danio rerio] | gi\|26788088 | 335701.2 | 6.91 | 21 | 33 | 0 |

Peptide Information

| Calc. Mass | Obsrv. Mass | ± da | ± ppm | Start Seq. | End Seq. | Sequence | Ion Score | C. I. % | Modification | Rank | Result Type |
|---|---|---|---|---|---|---|---|---|---|---|---|
| 950.4173 | 950.4852 | 0.0679 | 71 | 446 | 454 | DGSSERGDK | | | | | Mascot |
| 1082.639 | 1082.5773 | -0.0617 | -57 | 2452 | 2460 | KVPLMEPLR | | | | | Mascot |
| 1115.599 | 1115.6925 | 0.0935 | 84 | 1878 | 1886 | RPSENMLIR | | | | | Mascot |
| 1115.599 | 1115.6925 | 0.0935 | 84 | 1878 | 1886 | RPSENMLIR | 13 | 0 | | | Mascot |

| Calc. Mass | Obsrv. Mass | ± da | ± ppm | Start Seq. | End Seq. | Sequence | Ion Score | C. I. % | Modification | Rank | Result Type |
|---|---|---|---|---|---|---|---|---|---|---|---|
| 1121.5619 | 1121.5931 | 0.0312 | 28 | 1803 | 1811 | SIQQENMKK | | | Oxidation (M)[7] | | Mascot |
| 1179.7096 | 1179.5988 | -0.1108 | -94 | 2463 | 2473 | STSPKPKLPPK | | | | | Mascot |
| 1279.7004 | 1279.7744 | 0.074 | 58 | 931 | 941 | QQTSEPVHILK | | | | | Mascot |
| 1382.6434 | 1382.6827 | 0.0393 | 28 | 2438 | 2449 | SDSYTLDPDTLR | | | | | Mascot |
| 1448.7347 | 1448.9192 | 0.1845 | 127 | 635 | 646 | CLLINKLDGSCR | | | Carbamidomethyl (C)[1,11] | | Mascot |
| 1458.6174 | 1458.7205 | 0.1031 | 71 | 679 | 691 | EMCCYNAVIADAR | | | | | Mascot |
| 1458.7223 | 1458.7205 | -0.0018 | -1 | 758 | 769 | VYSKEDYSVVNR | | | | | Mascot |
| 1475.7853 | 1475.7527 | -0.0326 | -22 | 455 | 468 | DASKITTYPPGAVR | | | | | Mascot |
| 1515.6389 | 1515.7451 | 0.1062 | 70 | 679 | 691 | EMCCYNAVIADAR | | | Carbamidomethyl (C)[3] | | Mascot |
| 1555.7858 | 1555.749 | -0.0368 | -24 | 1685 | 1699 | ELAYLGSVCAAALMK | | | Oxidation (M)[14] | | Mascot |
| 1592.6688 | 1592.814 | 0.1452 | 91 | 271 | 283 | EEKSMMCQPGMHK | | | Carbamidomethyl (C)[7] | | Mascot |
| 1592.6688 | 1592.814 | 0.1452 | 91 | 271 | 283 | EEKSMMCQPGMHK | | | Carbamidomethyl (C)[7] | | Mascot |
| 1608.8024 | 1608.8344 | 0.032 | 20 | 2077 | 2091 | TGPSGHNIRSCPNLR | | | | | Mascot |
| 1699.7592 | 1699.9332 | 0.174 | 102 | 2635 | 2649 | NSLSCQQGFDEKESK | | | | | Mascot |
| 1699.7592 | 1699.9332 | 0.174 | 102 | 2635 | 2649 | NSLSCQQGFDEKESK | | | | | Mascot |
| 1955.0564 | 1954.8737 | -0.1827 | -93 | 2086 | 2103 | SCPNLRGIPIGMLVLGNK | | | Carbamidomethyl (C)[2], Oxidation (M)[12] | | Mascot |
| 2204.1995 | 2204.1025 | -0.097 | -44 | 350 | 369 | GIFDAVKEMIPLDLLLGTSR | | | Oxidation (M)[9] | | Mascot |
| 2217.155 | 2217.189 | 0.034 | 15 | 1743 | 1761 | WLQPDSYADPQKTSLILNK | | | | | Mascot |
| 2219.9001 | 2220.1204 | 0.2203 | 99 | 2698 | 2716 | CEDGKESVSYEMTSHSSMR | | | Carbamidomethyl (C)[1] | | Mascot |
| 2233.0837 | 2233.082 | -0.0017 | -1 | 158 | 178 | LETKMAVYTACNNGSSSIVTK | | | Oxidation (M)[5] | | Mascot |
| 2269.2273 | 2269.2627 | 0.0354 | 16 | 411 | 430 | GPLVFPGPLFMNQREQVLAR | | | | | Mascot |
| 2269.2273 | 2269.2627 | 0.0354 | 16 | 411 | 430 | GPLVFPGPLFMNQREQVLAR | | | | | Mascot |

| | | | | | | | | |
|---|---|---|---|---|---|---|---|---|
| 38 | PREDICTED: partner and localizer of BRCA2 [Danio rerio] | gi\|292609460 | 35211.4 | 5.68 | 7 | 33 | 0 | |

Peptide Information

| Calc. Mass | Obsrv. Mass | ± da | ± ppm | Start Seq. | End Seq. | Sequence | Ion Score | C. I. % | Modification | Rank | Result Type |
|---|---|---|---|---|---|---|---|---|---|---|---|
| 1432.7577 | 1432.6503 | -0.1074 | -75 | 289 | 301 | ATVVLDESCRLAR | | | | | Mascot |
| 1434.7223 | 1434.7253 | 0.003 | 2 | 151 | 163 | QNIQTFAAVEGEK | | | | | Mascot |
| 1477.7931 | 1477.9099 | 0.1168 | 79 | 129 | 142 | VSMLTLSQEGSVVK | | | | | Mascot |
| 1493.788 | 1493.7314 | -0.0566 | -38 | 129 | 142 | VSMLTLSQEGSVVK | | | Oxidation (M)[3] | | Mascot |
| 1592.7948 | 1592.814 | 0.0192 | 12 | 284 | 298 | DSVAKATVVLDESCR | 4 | 0 | | | Mascot |
| 1592.7948 | 1592.814 | 0.0192 | 12 | 284 | 298 | DSVAKATVVLDESCR | | | | | Mascot |
| 2220.0974 | 2220.1204 | 0.023 | 10 | 1 | 19 | MHTFKALEDGCVLDLCLVR | | | Carbamidomethyl (C)[11] | | Mascot |

| | | | | | | | | | | | | |
|---|---|---|---|---|---|---|---|---|---|---|---|---|
| 2233.2446 | 2233.082 | -0.1626 | -73 | 174 | 193 | TLLIWSMKSGQLLQTICLGK | | | | | | Mascot |
| 2284.1531 | 2284.1724 | 0.0193 | 8 | 182 | 202 | SGQLLQTICLGKTLTMTNCMK | | | | | | Mascot |

39 Barhl1.2 protein [Danio rerio] gi|161611956 32929.5 9.15 7 33 0

Peptide Information

| Calc. Mass | Obsrv. Mass | ± da | ± ppm | Start Seq. | End Seq. | Sequence | Ion Score | C. I. % | Modification | Rank | Result Type |
|---|---|---|---|---|---|---|---|---|---|---|---|
| 1023.5118 | 1023.5066 | -0.0052 | -5 | 201 | 207 | TWYQNRR | | | | | Mascot |
| 1155.5865 | 1155.6885 | 0.102 | 88 | 54 | 63 | SSVEDAVHRR | | | | | Mascot |
| 1155.5865 | 1155.6885 | 0.102 | 88 | 53 | 62 | RSSVEDAVHR | | | | | Mascot |
| 1608.7612 | 1608.8344 | 0.0732 | 46 | 121 | 134 | LHSNSSSDSEYRVK | | | | | Mascot |
| 1622.886 | 1622.8505 | -0.0355 | -22 | 66 | 80 | ALGLDSPLQISQQPR | | | | | Mascot |
| 1622.886 | 1622.8505 | -0.0355 | -22 | 66 | 80 | ALGLDSPLQISQQPR | | | | | Mascot |
| 2299.062 | 2299.1831 | 0.1211 | 53 | 31 | 52 | SPAELSPRSDLDSGCSSPPSPR | | | Carbamidomethyl (C)[15] | | Mascot |
| 2627.4514 | 2627.3674 | -0.084 | -32 | 66 | 89 | ALGLDSPLQISQQPRTVTSSFLIR | | | | | Mascot |

40 Zgc:171915 protein [Danio rerio] gi|156230187 32406 8.38 7 33 0

Peptide Information

| Calc. Mass | Obsrv. Mass | ± da | ± ppm | Start Seq. | End Seq. | Sequence | Ion Score | C. I. % | Modification | Rank | Result Type |
|---|---|---|---|---|---|---|---|---|---|---|---|
| 842.5246 | 842.5099 | -0.0147 | -17 | 263 | 269 | GKIHIFK | | | | | Mascot |
| 1382.8187 | 1382.6827 | -0.136 | -98 | 149 | 160 | LPLKVLIACNNK | | | Carbamidomethyl (C)[9] | | Mascot |
| 1477.7943 | 1477.9099 | 0.1156 | 78 | 229 | 240 | VTSIPVCYRNLR | | | Carbamidomethyl (C)[7] | | Mascot |
| 1515.7761 | 1515.7451 | -0.031 | -20 | 51 | 66 | ALDEAAATGGLNLSGR | | | | | Mascot |
| 1625.8179 | 1625.8387 | 0.0208 | 13 | 265 | 277 | IHIFKYLNMEACK | | | Oxidation (M)[9] | | Mascot |
| 1666.8444 | 1666.8771 | 0.0327 | 20 | 265 | 277 | IHIFKYLNMEACK | | | Carbamidomethyl (C)[12] | | Mascot |
| 2211.1841 | 2211.1021 | -0.082 | -37 | 210 | 228 | LPPELAELPLVRLDFSCNK | | | Carbamidomethyl (C)[17] | | Mascot |
| 2627.4338 | 2627.3674 | -0.0664 | -25 | 241 | 264 | HLQSIILDNNPLQSPPAQICIKGK | | | | | Mascot |

41 LOC100149217 protein [Danio rerio] gi|158253697 48680.3 9.76 11 32 0

Peptide Information

| Calc. Mass | Obsrv. Mass | ± da | ± ppm | Start Seq. | End Seq. | Sequence | Ion Score | C. I. % | Modification | Rank | Result Type |
|---|---|---|---|---|---|---|---|---|---|---|---|
| 1023.572 | 1023.5066 | -0.0654 | -64 | 75 | 83 | TFLISSDLK | | | | | Mascot |
| 1043.4285 | 1043.4567 | 0.0282 | 27 | 22 | 30 | GFACTQCEK | | | Carbamidomethyl (C)[4] | | Mascot |
| 1155.5865 | 1155.6885 | 0.102 | 88 | 190 | 199 | QSSSLNNHIR | | | | | Mascot |

| | | | | | | | | | | | |
|---|---|---|---|---|---|---|---|---|---|---|---|
| 1155.5865 | 1155.6885 | 0.102 | 88 | 190 | 199 | QSSSLNNHIR | | | | | Mascot |
| 1493.7166 | 1493.7314 | 0.0148 | 10 | 327 | 339 | SFTQSSSLVNHMR | | | | | Mascot |
| 1625.8217 | 1625.8387 | 0.017 | 10 | 140 | 152 | LHQMIHTGERPYK | | | Oxidation (M)[4] | | Mascot |
| 1689.7948 | 1689.9281 | 0.1333 | 79 | 284 | 298 | IHTGEKPHVCSHCNK | | | | | Mascot |
| 1695.7828 | 1695.8931 | 0.1103 | 65 | 228 | 242 | LHTNEKLYSCAECGK | | | | | Mascot |
| 1711.7778 | 1711.8909 | 0.1131 | 66 | 377 | 391 | CDQCGETFLRPSDLK | | | | | Mascot |
| 2193.9475 | 2194.0884 | 0.1409 | 64 | 168 | 186 | THEMIHTGEKPYTCSQC GR | | | Oxidation (M)[4] | | Mascot |
| 2218.0127 | 2218.1294 | 0.1167 | 53 | 308 | 326 | VHERSHTGEKPYTCTQC GK | | | Carbamidomethyl (C)[14] | | Mascot |
| 2283.0579 | 2283.176 | 0.1181 | 52 | 140 | 158 | LHQMIHTGERPYKCSHC DK | | | | | Mascot |
| 2283.0579 | 2283.176 | 0.1181 | 52 | 140 | 158 | LHQMIHTGERPYKCSHC DK | | | | | Mascot |
| 2299.053 | 2299.1831 | 0.1301 | 57 | 140 | 158 | LHQMIHTGERPYKCSHC DK | | | Oxidation (M)[4] | | Mascot |

| | | | | | | | |
|---|---|---|---|---|---|---|---|
| 42 | small GTPase RhoA [Danio rerio] | gi\|56118288 | 21849 | 5.83 | 7 | 32 | 0 |

**Peptide Information**

| Calc. Mass | Obsrv. Mass | ± da | ± ppm | Start Seq. | End Seq. | Sequence | Ion Score | C. I. % | Modification | Rank | Result Type |
|---|---|---|---|---|---|---|---|---|---|---|---|
| 982.4662 | 982.567 | 0.1008 | 103 | 169 | 176 | EVFEMATR | | | | | Mascot |
| 1023.5906 | 1023.5066 | -0.084 | -82 | 19 | 27 | TCLLIVFSK | | | | | Mascot |
| 1088.5768 | 1088.615 | 0.0382 | 35 | 8 | 18 | LVIVGDGACGK | | | Carbamidomethyl (C)[9] | | Mascot |
| 1155.5502 | 1155.6885 | 0.1383 | 120 | 120 | 128 | DLRNDEHTR | | | | | Mascot |
| 1155.5502 | 1155.6885 | 0.1383 | 120 | 120 | 128 | DLRNDEHTR | | | | | Mascot |
| 1164.5062 | 1164.5659 | 0.0597 | 51 | 141 | 150 | AEEGRDMANR | | | Oxidation (M)[7] | | Mascot |
| 1592.8213 | 1592.814 | -0.0073 | -5 | 169 | 182 | EVFEMATRAALQAR | | | | | Mascot |
| 1592.8213 | 1592.814 | -0.0073 | -5 | 169 | 182 | EVFEMATRAALQAR | | | | | Mascot |
| 1608.8163 | 1608.8344 | 0.0181 | 11 | 169 | 182 | EVFEMATRAALQAR | | | Oxidation (M)[5] | | Mascot |
| 1936.835 | 1936.916 | 0.081 | 42 | 146 | 162 | DMANRIGAFGYMECSAK | | | Carbamidomethyl (C)[14], Oxidation (M)[2] | | Mascot |

| | | | | | | | |
|---|---|---|---|---|---|---|---|
| 43 | complement component 3-like isoform 1 [Danio rerio] | gi\|153792045 | 188748 | 7.66 | 17 | 32 | 0 |

**Peptide Information**

| Calc. Mass | Obsrv. Mass | ± da | ± ppm | Start Seq. | End Seq. | Sequence | Ion Score | C. I. % | Modification | Rank | Result Type |
|---|---|---|---|---|---|---|---|---|---|---|---|
| 842.5206 | 842.5099 | -0.0107 | -13 | 516 | 523 | GKIIQAGR | | | | | Mascot |
| 982.5931 | 982.567 | -0.0261 | -27 | 971 | 979 | LQLDAVVPK | | | | | Mascot |
| 1088.5591 | 1088.615 | 0.0559 | 51 | 709 | 717 | ARMIEPMPK | | | Oxidation (M)[3] | | Mascot |
| 1097.679 | 1097.6646 | -0.0144 | -13 | 518 | 527 | IIQAGRVNVK | | | | | Mascot |
| 1121.6201 | 1121.5931 | -0.027 | -24 | 1049 | 1057 | EEAIKYIQK | | | | | Mascot |

| | | | | | | | | | | | |
|---|---|---|---|---|---|---|---|---|---|---|---|
| 1155.5641 | 1155.6885 | 0.1244 | 108 | 1378 | 1387 | TSSDVEKSFR | | | | | Mascot |
| 1155.5641 | 1155.6885 | 0.1244 | 108 | 1378 | 1387 | TSSDVEKSFR | | | | | Mascot |
| 1475.7965 | 1475.7527 | -0.0438 | -30 | 1457 | 1468 | EAEIISFRLNQR | | | | | Mascot |
| 1515.8451 | 1515.7451 | -0.1 | -66 | 862 | 875 | STPSVNVVMVILEK | | | | | Mascot |
| 1592.8014 | 1592.814 | 0.0126 | 8 | 343 | 358 | ASVLTSSGSDLVEAEK | | | | | Mascot |
| 1592.8014 | 1592.814 | 0.0126 | 8 | 343 | 358 | ASVLTSSGSDLVEAEK | | | | | Mascot |
| 1608.8632 | 1608.8344 | -0.0288 | -18 | 285 | 299 | GVAYVLFGIEINGEK | | | | | Mascot |
| 1622.8033 | 1622.8505 | 0.0472 | 29 | 1176 | 1189 | SRQAAGYLTEHFSR | | | | | Mascot |
| 1622.8033 | 1622.8505 | 0.0472 | 29 | 1176 | 1189 | SRQAAGYLTEHFSR | | | | | Mascot |
| 1625.7992 | 1625.8387 | 0.0395 | 24 | 1091 | 1104 | VFSMAHSFITVNEK | | | Oxidation (M)[4] | | Mascot |
| 2142.1965 | 2141.9885 | -0.208 | -97 | 411 | 429 | RPISVHSGTINIHINMPKK | | | | | Mascot |
| 2204.1228 | 2204.1025 | -0.0203 | -9 | 307 | 326 | QIDDVLNGGSVSLTMEEI KR | | | | | Mascot |
| 2217.2349 | 2217.189 | -0.0459 | -21 | 182 | 202 | TISLEIQNPDGIAVHGIGR VK | | | | | Mascot |
| 2220.1177 | 2220.1204 | 0.0027 | 1 | 307 | 326 | QIDDVLNGGSVSLTMEEI KR | | | Oxidation (M)[15] | | Mascot |
| 2233.1533 | 2233.082 | -0.0713 | -32 | 911 | 932 | TGELPLQVTAVAASFMG QDAVK | | | | | Mascot |
| 2238.2129 | 2238.1086 | -0.1043 | -47 | 73 | 92 | VVKVTVVVYDYPNSQTAL SR | | | | | Mascot |

| | | | | | | | | | | |
|---|---|---|---|---|---|---|---|---|---|---|
| 44 | hypothetical protein LOC100158483 [Xenopus (Silurana) tropicalis] | gi\|189230142 | 60297.2 | 5.13 | 7 | 32 | 0 | 16 | 0 | |

**Peptide Information**

| Calc. Mass | Obsrv. Mass | ± da | ± ppm | Start Seq. | End Seq. | Sequence | Ion Score | C. I. % | Modification | Rank | Result Type |
|---|---|---|---|---|---|---|---|---|---|---|---|
| 882.5407 | 882.5657 | 0.025 | 28 | 278 | 285 | SPILSPIR | | | | | Mascot |
| 1023.5469 | 1023.5066 | -0.0403 | -39 | 48 | 57 | SSSATPAKFK | | | | | Mascot |
| 1121.5885 | 1121.5931 | 0.0046 | 4 | 121 | 129 | QSMKLPHHK | | | Oxidation (M)[3] | | Mascot |
| 1164.5942 | 1164.5659 | -0.0283 | -24 | 334 | 343 | SCSPFKSRPR | | | | | Mascot |
| 1458.7546 | 1458.7205 | -0.0341 | -23 | 286 | 298 | LQQSVTPESGERK | | | | | Mascot |
| 1458.7546 | 1458.7205 | -0.0341 | -23 | 286 | 298 | LQQSVTPESGERK | 16 | 0 | | | Mascot |
| 1954.9504 | 1954.8737 | -0.0767 | -39 | 79 | 95 | QAVFFSQARADSEIEEK | | | | | Mascot |
| 2194.0266 | 2194.0884 | 0.0618 | 28 | 320 | 339 | SGESPLVEGCSPIRSCSP FK | | | Carbamidomethyl (C)[10,16] | | Mascot |

| | | | | | | | | | | |
|---|---|---|---|---|---|---|---|---|---|---|
| 45 | novel protein similar to vertebrate transmembrane protein 16H (TMEM16H) [Danio rerio] | gi\|169158193 | 112029.6 | 5.99 | 9 | 32 | 0 | 21 | 0 | |

**Peptide Information**

| Calc. Mass | Obsrv. Mass | ± da | ± ppm | Start Seq. | End Seq. | Sequence | Ion Score | C. I. % | Modification | Rank | Result Type |
|---|---|---|---|---|---|---|---|---|---|---|---|
| 864.4686 | 864.5085 | 0.0399 | 46 | 789 | 794 | LEYQRR | | | | | Mascot |

| Calc. Mass | Obsrv. Mass | ± da | ± ppm | Start Seq. | End Seq. | Sequence | Ion Score | C. I. % | Modification | Rank | Result Type |
|---|---|---|---|---|---|---|---|---|---|---|---|
| 982.5203 | 982.567 | 0.0467 | 48 | 856 | 864 | FSSTGDKLK | | | | | Mascot |
| 1115.6782 | 1115.6925 | 0.0143 | 13 | 2 | 11 | SSAVLEKLLR | | | | | Mascot |
| 1115.6782 | 1115.6925 | 0.0143 | 13 | 2 | 11 | SSAVLEKLLR | 21 | 0 | | | Mascot |
| 1121.4891 | 1121.5931 | 0.104 | 93 | 614 | 624 | AAGGENNTMEK | | | | | Mascot |
| 1463.8654 | 1463.8929 | 0.0275 | 19 | 437 | 448 | LKEMLATLLIFR | | | Oxidation (M)[4] | | Mascot |
| 1515.7537 | 1515.7451 | -0.0086 | -6 | 527 | 540 | VPKTEEVDDDAGLK | | | | | Mascot |
| 1555.7097 | 1555.749 | 0.0393 | 25 | 203 | 215 | QPLDDVCDYFGVK | | | Carbamidomethyl (C)[7] | | Mascot |
| 1666.921 | 1666.8771 | -0.0439 | -26 | 174 | 186 | RVILQMFPLHEQR | | | | | Mascot |
| 2283.1477 | 2283.176 | 0.0283 | 12 | 769 | 788 | YVIHVAIPDVPNWVADEMAK | | | Oxidation (M)[18] | | Mascot |
| 2283.1477 | 2283.176 | 0.0283 | 12 | 769 | 788 | YVIHVAIPDVPNWVADEMAK | | | Oxidation (M)[18] | | Mascot |

| | | | | | | | |
|---|---|---|---|---|---|---|---|
| 46 | PREDICTED: hypothetical protein LOC562947 [Danio rerio] | gi\|292610701 | 116105.3 | 5.91 | 1 | 21 | 0 |

**Peptide Information**

| Calc. Mass | Obsrv. Mass | ± da | ± ppm | Start Seq. | End Seq. | Sequence | Ion Score | C. I. % | Modification | Rank | Result Type |
|---|---|---|---|---|---|---|---|---|---|---|---|
| 1115.6782 | 1115.6925 | 0.0143 | 13 | 2 | 11 | SSAVLEKLLR | 21 | 0 | | 2 | Mascot |

| | | | | | | | |
|---|---|---|---|---|---|---|---|
| 47 | PREDICTED: wu:fb77e12 [Danio rerio] | gi\|326679169 | 160931.2 | 6.5 | 1 | 19 | 0 |

**Peptide Information**

| Calc. Mass | Obsrv. Mass | ± da | ± ppm | Start Seq. | End Seq. | Sequence | Ion Score | C. I. % | Modification | Rank | Result Type |
|---|---|---|---|---|---|---|---|---|---|---|---|
| 1115.5328 | 1115.6925 | 0.1597 | 143 | 480 | 489 | DTPSDPQSIR | 19 | 0 | | 3 | Mascot |

| | | | | | | | |
|---|---|---|---|---|---|---|---|
| 48 | PREDICTED: poly [ADP-ribose] polymerase 14-like [Danio rerio] | gi\|326671190 | 198536.4 | 6.57 | 1 | 18 | 0 |

**Peptide Information**

| Calc. Mass | Obsrv. Mass | ± da | ± ppm | Start Seq. | End Seq. | Sequence | Ion Score | C. I. % | Modification | Rank | Result Type |
|---|---|---|---|---|---|---|---|---|---|---|---|
| 1115.6241 | 1115.6925 | 0.0684 | 61 | 1417 | 1425 | EMINNLIIR | 18 | 0 | | 4 | Mascot |

| | | | | | | | |
|---|---|---|---|---|---|---|---|
| 49 | PREDICTED: low-density lipoprotein receptor-related protein 1B, partial [Danio rerio] | gi\|326678476 | 326092.8 | 5.15 | 1 | 17 | 0 |

**Peptide Information**

| Calc. Mass | Obsrv. Mass | ± da | ± ppm | Start Seq. | End Seq. | Sequence | Ion Score | C. I. % | Modification | Rank | Result Type |
|---|---|---|---|---|---|---|---|---|---|---|---|
| 1115.6418 | 1115.6925 | 0.0507 | 45 | 2089 | 2098 | KDAVDVVTIR | 17 | 0 | | 5 | Mascot |

| | | | | | | | |
|---|---|---|---|---|---|---|---|
| 50 | PREDICTED: hypothetical protein LOC100536674 | gi\|326678668 | 141952.3 | 9.22 | 1 | 16 | 0 |

[Danio rerio]

Peptide Information

| Calc. Mass | Obsrv. Mass | ± da | ± ppm | Start Seq. | End Seq. | Sequence | Ion Score | C. I. % | Modification | Rank | Result Type |
|---|---|---|---|---|---|---|---|---|---|---|---|
| 1115.6354 | 1115.6925 | 0.0571 | 51 | 263 | 271 | VMKPEARIR | 16 | 0 | Oxidation (M)[2] | 7 | Mascot |

| Gel Idx/Pos | 178/H5 | Instr./Gel Origin | BA2060/110707A | Process Status | Analysis Succeeded |
|---|---|---|---|---|---|
| Plate [#] Name | [1] 31496 | Instrument Sample Name | | Spectra | 11 |

| Rank | Protein Name | Accession No. | Protein MW | Protein PI | Pep. Count | Protein Score | Protein Score C. I. % | Total Ion Score | Total Ion C. I. % |
|---|---|---|---|---|---|---|---|---|---|
| 1 | transitional endoplasmic reticulum ATPase [Danio rerio] | gi\|41393119 | 89367.7 | 5.14 | 34 | 1,310 | 100 | 1047 | 100 |

Peptide Information

| Calc. Mass | Obsrv. Mass | ± da | ± ppm | Start Seq. | End Seq. | Sequence | Ion Score | C. I. % | Modification | Rank | Result Type |
|---|---|---|---|---|---|---|---|---|---|---|---|
| 819.4722 | 819.4641 | -0.0081 | -10 | 149 | 155 | GDIFLVR | | | | | Mascot |
| 837.5305 | 837.522 | -0.0085 | -10 | 232 | 239 | AIGVKPPR | | | | | Mascot |
| 947.5672 | 947.5545 | -0.0127 | -13 | 148 | 155 | KGDIFLVR | | | | | Mascot |
| 986.5339 | 986.519 | -0.0149 | -15 | 218 | 225 | EMVELPLR | | | | | Mascot |
| 989.4897 | 989.4801 | -0.0096 | -10 | 701 | 708 | ESIENEIR | | | | | Mascot |
| 1051.5242 | 1051.5104 | -0.0138 | -13 | 46 | 53 | MDELQLFR | | | | | Mascot |
| 1067.519 | 1067.5068 | -0.0122 | -11 | 46 | 53 | MDELQLFR | | | Oxidation (M)[1] | | Mascot |
| 1075.5378 | 1075.5247 | -0.0131 | -12 | 278 | 287 | LAGESESNLR | | | | | Mascot |
| 1145.5909 | 1145.5791 | -0.0118 | -10 | 701 | 709 | ESIENEIRR | | | | | Mascot |
| 1162.531 | 1162.5184 | -0.0126 | -11 | 733 | 741 | KDHFEEAMR | | | | | Mascot |
| 1172.6674 | 1172.6554 | -0.012 | -10 | 240 | 251 | GILLYGPPGTGK | | | | | Mascot |
| 1242.6324 | 1242.6195 | -0.0129 | -10 | 366 | 377 | EVDIGIPDATGR | | | | | Mascot |
| 1251.619 | 1251.6042 | -0.0148 | -12 | 513 | 524 | GVLFYGPPGCGK | | | Carbamidomethyl (C)[10] | | Mascot |
| 1329.691 | 1329.6761 | -0.0149 | -11 | 454 | 465 | WALSQSNPSALR | 96 | 100 | | | Mascot |
| 1329.691 | 1329.6761 | -0.0149 | -11 | 454 | 465 | WALSQSNPSALR | | | | | Mascot |
| 1540.804 | 1540.7844 | -0.0196 | -13 | 96 | 109 | LGDVISIQPCPDVK | | | Carbamidomethyl (C)[10] | | Mascot |
| 1556.8571 | 1556.8376 | -0.0195 | -13 | 639 | 651 | LDQLIYIPLPDEK | | | | | Mascot |
| 1578.7944 | 1578.7739 | -0.0205 | -13 | 530 | 543 | AIANECQANFISIK | | | Carbamidomethyl (C)[6] | | Mascot |
| 1578.7944 | 1578.7739 | -0.0205 | -13 | 530 | 543 | AIANECQANFISIK | 67 | 99.996 | Carbamidomethyl (C)[6] | | Mascot |
| 1629.8054 | 1629.7854 | -0.02 | -12 | 754 | 766 | KYEMFAQTLQQSR | 88 | 100 | | | Mascot |
| 1629.8054 | 1629.7854 | -0.02 | -12 | 754 | 766 | KYEMFAQTLQQSR | | | | | Mascot |

| | | | | | | | | | | | |
|---|---|---|---|---|---|---|---|---|---|---|---|
| 1645.8003 | 1645.7821 | -0.0182 | -11 | 754 | 766 | KYEMFAQTLQQSR | | | Oxidation (M)[4] | | Mascot |
| 1660.8289 | 1660.7837 | -0.0452 | -27 | 363 | 377 | FDREVDIGIPDATGR | | | | | Mascot |
| 1799.8051 | 1799.783 | -0.0221 | -12 | 678 | 693 | MTNGFSGADLTEICQR | | | Carbamidomethyl (C)[14] | | Mascot |
| 1810.9949 | 1810.9752 | -0.0197 | -11 | 296 | 312 | NAPAIIFIDELDAIAPK | | | | | Mascot |
| 1815.8 | 1815.782 | -0.018 | -10 | 678 | 693 | MTNGFSGADLTEICQR | | | Carbamidomethyl (C)[14], Oxidation (M)[1] | | Mascot |
| 1823.9174 | 1823.894 | -0.0234 | -13 | 488 | 502 | ELQELVQYPVEHPDK | | | | | Mascot |
| 1823.9174 | 1823.894 | -0.0234 | -13 | 488 | 502 | ELQELVQYPVEHPDK | 62 | 99.986 | | | Mascot |
| 1923.952 | 1923.9279 | -0.0241 | -13 | 568 | 584 | QAAPCVLFFDELDSIAK | | | Carbamidomethyl (C)[5] | | Mascot |
| 1951.9218 | 1951.9008 | -0.021 | -11 | 544 | 560 | GPELLTMWFGESEANVR | 102 | 100 | Oxidation (M)[7] | | Mascot |
| 1951.9218 | 1951.9008 | -0.021 | -11 | 544 | 560 | GPELLTMWFGESEANVR | | | Oxidation (M)[7] | | Mascot |
| 2124.9204 | 2124.8955 | -0.0249 | -12 | 192 | 210 | EDEEESLNEVGYDDIGGVR | | | | | Mascot |
| 2124.9204 | 2124.8955 | -0.0249 | -12 | 192 | 210 | EDEEESLNEVGYDDIGGVR | 142 | 100 | | | Mascot |
| 2146.155 | 2146.1343 | -0.0207 | -10 | 339 | 358 | AHVIVMAATNRPNSIDPALR | | | | | Mascot |
| 2155.9812 | 2155.9529 | -0.0283 | -13 | 714 | 732 | QTNPSAMEVEEDDPVPEIR | | | | | Mascot |
| 2160.103 | 2160.0588 | -0.0442 | -20 | 26 | 45 | LIVDESINEDNSVVSLSQAK | | | | | Mascot |
| 2162.1499 | 2162.1187 | -0.0312 | -14 | 339 | 358 | AHVIVMAATNRPNSIDPALR | | | Oxidation (M)[6] | | Mascot |
| 2171.9761 | 2171.9614 | -0.0147 | -7 | 714 | 732 | QTNPSAMEVEEDDPVPEIR | | | Oxidation (M)[7] | | Mascot |
| 2256.137 | 2256.0806 | -0.0564 | -25 | 257 | 277 | AVANETGAFFFLINGPEIMSK | | | | | Mascot |
| 2284.0762 | 2284.1499 | 0.0737 | 32 | 714 | 733 | QTNPSAMEVEEDDPVPEIRK | | | | | Mascot |
| 2457.1199 | 2457.344 | 0.2241 | 91 | 712 | 732 | ERQTNPSAMEVEEDDPVPEIR | | | Oxidation (M)[9] | | Mascot |
| 2484.2617 | 2484.2402 | -0.0215 | -9 | 466 | 487 | ETVVEVPNITWEDIGGLDDVKR | | | | | Mascot |
| 2484.2617 | 2484.2402 | -0.0215 | -9 | 466 | 487 | ETVVEVPNITWEDIGGLDDVKR | 217 | 100 | | | Mascot |
| 2518.4253 | 2518.4089 | -0.0164 | -7 | 616 | 638 | NVFIIGATNRPDIIDPAILRPGR | 22 | 0 | | | Mascot |
| 2518.4253 | 2518.4089 | -0.0164 | -7 | 616 | 638 | NVFIIGATNRPDIIDPAILRPGR | | | | | Mascot |
| 3081.502 | 3081.4858 | -0.0162 | -5 | 165 | 191 | VVETDPSPYCIVAPDTVIHCEGEPIKR | | | Carbamidomethyl (C)[10,20] | | Mascot |
| 3672.7922 | 3672.7708 | -0.0214 | -6 | 390 | 424 | LADDVDLEQVANETHGHVGADLAALCSEAALQAIR | 252 | 100 | Carbamidomethyl (C)[26] | | Mascot |
| 3672.7922 | 3672.7708 | -0.0214 | -6 | 390 | 424 | LADDVDLEQVANETHGHVGADLAALCSEAALQAIR | | | Carbamidomethyl (C)[26] | | Mascot |

| | | | | | | | | | | |
|---|---|---|---|---|---|---|---|---|---|---|
| 2 | CDC48 [Danio rerio] | gi\|34740143 | 89351.7 | 5.13 | 32 | 1,280 | 100 | 1047 | 100 | |

**Peptide Information**

| Calc. Mass | Obsrv. Mass | ± da | ± ppm | Start Seq. | End Seq. | Sequence | Ion Score | C. I. % | Modification | Rank | Result Type |
|---|---|---|---|---|---|---|---|---|---|---|---|
| 819.4722 | 819.4641 | -0.0081 | -10 | 149 | 155 | GDIFLVR | | | | | Mascot |

| | | | | | | | | | | |
|---|---|---|---|---|---|---|---|---|---|---|
| 837.5305 | 837.522 | -0.0085 | -10 | 232 | 239 | AIGVKPPR | | | | Mascot |
| 947.5672 | 947.5545 | -0.0127 | -13 | 148 | 155 | KGDIFLVR | | | | Mascot |
| 986.5339 | 986.519 | -0.0149 | -15 | 218 | 225 | EMVELPLR | | | | Mascot |
| 989.4897 | 989.4801 | -0.0096 | -10 | 701 | 708 | ESIENEIR | | | | Mascot |
| 1051.5242 | 1051.5104 | -0.0138 | -13 | 46 | 53 | MDELQLFR | | | | Mascot |
| 1067.519 | 1067.5068 | -0.0122 | -11 | 46 | 53 | MDELQLFR | | | Oxidation (M)[1] | Mascot |
| 1075.5378 | 1075.5247 | -0.0131 | -12 | 278 | 287 | LAGESESNLR | | | | Mascot |
| 1162.531 | 1162.5184 | -0.0126 | -11 | 733 | 741 | KDHFEEAMR | | | | Mascot |
| 1172.6674 | 1172.6554 | -0.012 | -10 | 240 | 251 | GILLYGPPGTGK | | | | Mascot |
| 1242.6324 | 1242.6195 | -0.0129 | -10 | 366 | 377 | EVDIGIPDATGR | | | | Mascot |
| 1251.619 | 1251.6042 | -0.0148 | -12 | 513 | 524 | GVLFYGPPGCGK | | | Carbamidomethyl (C)[10] | Mascot |
| 1329.691 | 1329.6761 | -0.0149 | -11 | 454 | 465 | WALSQSNPSALR | 96 | 100 | | Mascot |
| 1329.691 | 1329.6761 | -0.0149 | -11 | 454 | 465 | WALSQSNPSALR | | | | Mascot |
| 1540.804 | 1540.7844 | -0.0196 | -13 | 96 | 109 | LGDVISIQPCPDVK | | | Carbamidomethyl (C)[10] | Mascot |
| 1578.7944 | 1578.7739 | -0.0205 | -13 | 530 | 543 | AIANECQANFISIK | | | Carbamidomethyl (C)[6] | Mascot |
| 1578.7944 | 1578.7739 | -0.0205 | -13 | 530 | 543 | AIANECQANFISIK | 67 | 99.996 | Carbamidomethyl (C)[6] | Mascot |
| 1629.8054 | 1629.7854 | -0.02 | -12 | 754 | 766 | KYEMFAQTLQQSR | 88 | 100 | | Mascot |
| 1629.8054 | 1629.7854 | -0.02 | -12 | 754 | 766 | KYEMFAQTLQQSR | | | | Mascot |
| 1645.8003 | 1645.7821 | -0.0182 | -11 | 754 | 766 | KYEMFAQTLQQSR | | | Oxidation (M)[4] | Mascot |
| 1660.8289 | 1660.7837 | -0.0452 | -27 | 363 | 377 | FDREVDIGIPDATGR | | | | Mascot |
| 1799.8051 | 1799.783 | -0.0221 | -12 | 678 | 693 | MTNGFSGADLTEICQR | | | Carbamidomethyl (C)[14] | Mascot |
| 1810.9949 | 1810.9752 | -0.0197 | -11 | 296 | 312 | NAPAIIFIDELDAIAPK | | | | Mascot |
| 1815.8 | 1815.782 | -0.018 | -10 | 678 | 693 | MTNGFSGADLTEICQR | | | Carbamidomethyl (C)[14], Oxidation (M)[1] | Mascot |
| 1823.9174 | 1823.894 | -0.0234 | -13 | 488 | 502 | ELQELVQYPVEHPDK | | | | Mascot |
| 1823.9174 | 1823.894 | -0.0234 | -13 | 488 | 502 | ELQELVQYPVEHPDK | 62 | 99.986 | | Mascot |
| 1923.952 | 1923.9279 | -0.0241 | -13 | 568 | 584 | QAAPCVLFFDELDSIAK | | | Carbamidomethyl (C)[5] | Mascot |
| 1951.9218 | 1951.9008 | -0.021 | -11 | 544 | 560 | GPELLTMWFGESEANVR | 102 | 100 | Oxidation (M)[7] | Mascot |
| 1951.9218 | 1951.9008 | -0.021 | -11 | 544 | 560 | GPELLTMWFGESEANVR | | | Oxidation (M)[7] | Mascot |
| 2124.9204 | 2124.8955 | -0.0249 | -12 | 192 | 210 | EDEEESLNEVGYDDIGGVR | | | | Mascot |
| 2124.9204 | 2124.8955 | -0.0249 | -12 | 192 | 210 | EDEEESLNEVGYDDIGGVR | 142 | 100 | | Mascot |
| 2146.155 | 2146.1343 | -0.0207 | -10 | 339 | 358 | AHVIVMAATNRPNSIDPALR | | | | Mascot |
| 2155.9812 | 2155.9529 | -0.0283 | -13 | 714 | 732 | QTNPSAMEVEEDDPVPEIR | | | | Mascot |
| 2160.103 | 2160.0588 | -0.0442 | -20 | 26 | 45 | LIVDESINEDNSVVSLSQAK | | | | Mascot |
| 2162.1499 | 2162.1187 | -0.0312 | -14 | 339 | 358 | AHVIVMAATNRPNSIDPALR | | | Oxidation (M)[6] | Mascot |
| 2171.9761 | 2171.9614 | -0.0147 | -7 | 714 | 732 | QTNPSAMEVEEDDPVPEIR | | | Oxidation (M)[7] | Mascot |

| Calc. Mass | Obsrv. Mass | ± da | ± ppm | Start Seq. | End Seq. | Sequence | Ion Score | C. I. % | Modification | Rank | Result Type |
|---|---|---|---|---|---|---|---|---|---|---|---|
| 2256.137 | 2256.0806 | -0.0564 | -25 | 257 | 277 | AVANETGAFFFLINGPEIMSK | | | | | Mascot |
| 2284.0762 | 2284.1499 | 0.0737 | 32 | 714 | 733 | QTNPSAMEVEEDDPVPEIRK | | | | | Mascot |
| 2457.1199 | 2457.344 | 0.2241 | 91 | 712 | 732 | ERQTNPSAMEVEEDDPVPEIR | | | Oxidation (M)[9] | | Mascot |
| 2484.2617 | 2484.2402 | -0.0215 | -9 | 466 | 487 | ETVVEVPNITWEDIGGLDDVKR | | | | | Mascot |
| 2484.2617 | 2484.2402 | -0.0215 | -9 | 466 | 487 | ETVVEVPNITWEDIGGLDDVKR | 217 | 100 | | | Mascot |
| 2518.4253 | 2518.4089 | -0.0164 | -7 | 616 | 638 | NVFIIGATNRPDIIDPAILRPGR | 22 | 0 | | | Mascot |
| 2518.4253 | 2518.4089 | -0.0164 | -7 | 616 | 638 | NVFIIGATNRPDIIDPAILRPGR | | | | | Mascot |
| 3081.502 | 3081.4858 | -0.0162 | -5 | 165 | 191 | VVETDPSPYCIVAPDTVIHCEGEPIKR | | | Carbamidomethyl (C)[10,20] | | Mascot |
| 3672.7922 | 3672.7708 | -0.0214 | -6 | 390 | 424 | LADDVDLEQVANETHGHVGADLAALCSEAALQAIR | 252 | 100 | Carbamidomethyl (C)[26] | | Mascot |
| 3672.7922 | 3672.7708 | -0.0214 | -6 | 390 | 424 | LADDVDLEQVANETHGHVGADLAALCSEAALQAIR | | | Carbamidomethyl (C)[26] | | Mascot |

| | | | | | | | | | | |
|---|---|---|---|---|---|---|---|---|---|---|
| 3 | transitional endoplasmic reticulum ATPase-like [Danio rerio] | gi\|90093334 | 89437.9 | 5.44 | 15 | 363 | 100 | 308 | 100 | |

**Peptide Information**

| Calc. Mass | Obsrv. Mass | ± da | ± ppm | Start Seq. | End Seq. | Sequence | Ion Score | C. I. % | Modification | Rank | Result Type |
|---|---|---|---|---|---|---|---|---|---|---|---|
| 819.4722 | 819.4641 | -0.0081 | -10 | 151 | 157 | GDIFLVR | | | | | Mascot |
| 837.5305 | 837.522 | -0.0085 | -10 | 234 | 241 | AIGVKPPR | | | | | Mascot |
| 986.5339 | 986.519 | -0.0149 | -15 | 220 | 227 | EMVELPLR | | | | | Mascot |
| 1075.5378 | 1075.5247 | -0.0131 | -12 | 280 | 289 | LAGESESNLR | | | | | Mascot |
| 1162.531 | 1162.5184 | -0.0126 | -11 | 735 | 743 | KDHFEEAMR | | | | | Mascot |
| 1172.6674 | 1172.6554 | -0.012 | -10 | 242 | 253 | GILLYGPPGTGK | | | | | Mascot |
| 1251.619 | 1251.6042 | -0.0148 | -12 | 515 | 526 | GVLFYGPPGCGK | | | Carbamidomethyl (C)[10] | | Mascot |
| 1329.691 | 1329.6761 | -0.0149 | -11 | 456 | 467 | WALSQSNPSALR | 96 | 100 | | | Mascot |
| 1329.691 | 1329.6761 | -0.0149 | -11 | 456 | 467 | WALSQSNPSALR | | | | | Mascot |
| 1629.8054 | 1629.7854 | -0.02 | -12 | 756 | 768 | KYEMFAQTLQQSR | 88 | 100 | | | Mascot |
| 1629.8054 | 1629.7854 | -0.02 | -12 | 756 | 768 | KYEMFAQTLQQSR | | | | | Mascot |
| 1645.8003 | 1645.7821 | -0.0182 | -11 | 756 | 768 | KYEMFAQTLQQSR | | | Oxidation (M)[4] | | Mascot |
| 1810.9949 | 1810.9752 | -0.0197 | -11 | 298 | 314 | NAPAIIFIDELDAIAPK | | | | | Mascot |
| 1823.8262 | 1823.894 | 0.0678 | 37 | 68 | 83 | QTVCIVLTDDTCGNER | | | Carbamidomethyl (C)[4] | | Mascot |
| 1823.8262 | 1823.894 | 0.0678 | 37 | 68 | 83 | QTVCIVLTDDTCGNER | | | Carbamidomethyl (C)[4] | | Mascot |
| 1951.9218 | 1951.9008 | -0.021 | -11 | 546 | 562 | GPELLTMWFGESEANVR | 102 | 100 | Oxidation (M)[7] | | Mascot |
| 1951.9218 | 1951.9008 | -0.021 | -11 | 546 | 562 | GPELLTMWFGESEANVR | | | Oxidation (M)[7] | | Mascot |
| 2156.011 | 2155.9529 | -0.0581 | -27 | 680 | 698 | ITEGFSGADLTEICQRACK | | | Carbamidomethyl (C)[14,18] | | Mascot |
| 2256.137 | 2256.0806 | -0.0564 | -25 | 259 | 279 | AVANETGAFFFLINGPEI | | | | | Mascot |

| Calc. Mass | Obsrv. Mass | ± da | ± ppm | Start Seq. | End Seq. | Sequence | Ion Score | C. I. % | Modification | Rank | Result Type |
|---|---|---|---|---|---|---|---|---|---|---|---|
| 2518.4253 | 2518.4089 | -0.0164 | -7 | 618 | 640 | MSK NVFIIGATNRPDIIDPAILR PGR | 22 | 0 | | | Mascot |
| 2518.4253 | 2518.4089 | -0.0164 | -7 | 618 | 640 | NVFIIGATNRPDIIDPAILR PGR | | | | | Mascot |

4 Zgc:162848 protein [Danio rerio] gi|141795670 51350.1 5.88 9 52 71.922 17 0

Peptide Information

| Calc. Mass | Obsrv. Mass | ± da | ± ppm | Start Seq. | End Seq. | Sequence | Ion Score | C. I. % | Modification | Rank | Result Type |
|---|---|---|---|---|---|---|---|---|---|---|---|
| 947.4792 | 947.5545 | 0.0753 | 79 | 90 | 98 | AETDAATLR | | | | | Mascot |
| 1045.5411 | 1045.55 | 0.0089 | 9 | 174 | 182 | DAVEEELLK | | | | | Mascot |
| 1126.6327 | 1126.5721 | -0.0606 | -54 | 351 | 360 | EAPLRVAQSR | | | | | Mascot |
| 1329.7485 | 1329.6761 | -0.0724 | -54 | 196 | 206 | KTLDQTINQIR | 17 | 0 | | | Mascot |
| 1329.7485 | 1329.6761 | -0.0724 | -54 | 196 | 206 | KTLDQTINQIR | | | | | Mascot |
| 1473.7656 | 1473.7635 | -0.0021 | -1 | 271 | 283 | LAEREETASQALR | | | | | Mascot |
| 1816.9058 | 1816.7664 | -0.1394 | -77 | 361 | 374 | LHYRTLRPNMEQCR | | | | | Mascot |
| 1967.8777 | 1967.8976 | 0.0199 | 10 | 223 | 238 | QQAYTLDDHCGRFSNR | | | Carbamidomethyl (C)[10] | | Mascot |
| 1967.8777 | 1967.8976 | 0.0199 | 10 | 223 | 238 | QQAYTLDDHCGRFSNR | 3 | 0 | Carbamidomethyl (C)[10] | | Mascot |
| 2154.9873 | 2154.936 | -0.0513 | -24 | 245 | 262 | HPSSAVLQDQVCDEEVW R | | | Carbamidomethyl (C)[12] | | Mascot |
| 2283.0823 | 2283.1531 | 0.0708 | 31 | 245 | 263 | HPSSAVLQDQVCDEEVW RK | | | Carbamidomethyl (C)[12] | | Mascot |

5 PREDICTED: myosin-X [Danio rerio] gi|326665155 237030.3 6.18 21 46 0

Peptide Information

| Calc. Mass | Obsrv. Mass | ± da | ± ppm | Start Seq. | End Seq. | Sequence | Ion Score | C. I. % | Modification | Rank | Result Type |
|---|---|---|---|---|---|---|---|---|---|---|---|
| 1045.6 | 1045.55 | -0.05 | -48 | 1416 | 1425 | GSSQANLKLK | | | | | Mascot |
| 1145.468 | 1145.5791 | 0.1111 | 97 | 2 | 10 | DNFFTEGCR | | | Carbamidomethyl (C)[8] | | Mascot |
| 1165.6323 | 1165.7307 | 0.0984 | 84 | 1893 | 1902 | SSFLEGTLRR | | | | | Mascot |
| 1172.6633 | 1172.6554 | -0.0079 | -7 | 693 | 703 | ALALSEDLKGR | | | | | Mascot |
| 1329.7122 | 1329.6761 | -0.0361 | -27 | 571 | 581 | DDVLNLLRESR | | | | | Mascot |
| 1329.7122 | 1329.6761 | -0.0361 | -27 | 571 | 581 | DDVLNLLRESR | | | | | Mascot |
| 1438.6665 | 1438.7064 | 0.0399 | 28 | 1942 | 1953 | LQGMEQDQAMKK | | | Oxidation (M)[4,10] | | Mascot |
| 1520.7744 | 1520.8969 | 0.1225 | 81 | 466 | 477 | HIFSLEQLEYNK | | | | | Mascot |
| 1533.7921 | 1533.7505 | -0.0416 | -27 | 1679 | 1691 | YAAFSQDALRHVR | | | | | Mascot |
| 1540.6486 | 1540.7844 | 0.1358 | 88 | 2056 | 2069 | FSTCQSISSHGSNW | | | | | Mascot |
| 1718.9111 | 1718.8932 | -0.0179 | -10 | 1830 | 1844 | YPAPEETLQFLAALR | | | | | Mascot |
| 1799.8938 | 1799.783 | -0.1108 | -62 | 1207 | 1219 | WCVLKDETFLWFR | | | Carbamidomethyl (C)[2] | | Mascot |
| 1800.9564 | 1800.7736 | -0.1828 | -102 | 687 | 701 | YYVLMRALALSEDLK | | | Oxidation (M)[5] | | Mascot |

| Calc. Mass | Obsrv. Mass | ± da | ± ppm | Start Seq. | End Seq. | Sequence | Ion Score | C. I. % | Modification | Rank | Result Type |
|---|---|---|---|---|---|---|---|---|---|---|---|
| 1823.8956 | 1823.894 | -0.0016 | -1 | 1525 | 1539 | ENCLNSEVVEQIYKR | | | | | Mascot |
| 1823.8956 | 1823.894 | -0.0016 | -1 | 1525 | 1539 | ENCLNSEVVEQIYKR | | | | | Mascot |
| 1952.0594 | 1951.9008 | -0.1586 | -81 | 2029 | 2045 | ELLFNTSMVVDIAKLMK | | | | | Mascot |
| 1952.0594 | 1951.9008 | -0.1586 | -81 | 2029 | 2045 | ELLFNTSMVVDIAKLMK | | | | | Mascot |
| 1968.0544 | 1967.8976 | -0.1568 | -80 | 2029 | 2045 | ELLFNTSMVVDIAKLMK | | | Oxidation (M)[8] | | Mascot |
| 1968.0544 | 1967.8976 | -0.1568 | -80 | 2029 | 2045 | ELLFNTSMVVDIAKLMK | | | Oxidation (M)[8] | | Mascot |
| 2124.9551 | 2124.8955 | -0.0596 | -28 | 400 | 416 | DSMAMALYSQCFTWIIR | | | Carbamidomethyl (C)[11], Oxidation (M)[3,5] | | Mascot |
| 2124.9551 | 2124.8955 | -0.0596 | -28 | 400 | 416 | DSMAMALYSQCFTWIIR | | | Carbamidomethyl (C)[11], Oxidation (M)[3,5] | | Mascot |
| 2233.0972 | 2233.0588 | -0.0384 | -17 | 124 | 142 | HHLGEIPPHIYAIANECYR | | | | | Mascot |
| 2256.0601 | 2256.0806 | 0.0205 | 9 | 968 | 986 | AAQQFLTSLNFDEIDECVR | | | Carbamidomethyl (C)[17] | | Mascot |
| 2283.1501 | 2283.1531 | 0.003 | 1 | 1913 | 1932 | QEEEPAAIEVWLQDEKAGLK | | | | | Mascot |
| 2484.3279 | 2484.2402 | -0.0877 | -35 | 330 | 353 | LLSGILHVGNIEFLTAGGAQVCSK | | | Carbamidomethyl (C)[22] | | Mascot |
| 2484.3279 | 2484.2402 | -0.0877 | -35 | 330 | 353 | LLSGILHVGNIEFLTAGGAQVCSK | | | Carbamidomethyl (C)[22] | | Mascot |
| 2518.2129 | 2518.4089 | 0.196 | 78 | 189 | 212 | TASSHVEEAILESSPIMEAFGNAK | | | | | Mascot |
| 2518.2129 | 2518.4089 | 0.196 | 78 | 189 | 212 | TASSHVEEAILESSPIMEAFGNAK | | | | | Mascot |
| 3672.7249 | 3672.7708 | 0.0459 | 12 | 1798 | 1829 | LFCFSEPENVPADSVEFAFMFEQAHEAVIRGR | | | | | Mascot |
| 3672.7249 | 3672.7708 | 0.0459 | 12 | 1798 | 1829 | LFCFSEPENVPADSVEFAFMFEQAHEAVIRGR | | | | | Mascot |

6 PREDICTED: nephronectin-like [Danio rerio] gi|326664063 25190.6 6.49 10 46 0

**Peptide Information**

| Calc. Mass | Obsrv. Mass | ± da | ± ppm | Start Seq. | End Seq. | Sequence | Ion Score | C. I. % | Modification | Rank | Result Type |
|---|---|---|---|---|---|---|---|---|---|---|---|
| 819.4393 | 819.4641 | 0.0248 | 30 | 141 | 147 | LDCISLR | | | | | Mascot |
| 882.5083 | 882.5549 | 0.0466 | 53 | 2 | 8 | PFKSYIK | | | | | Mascot |
| 947.5342 | 947.5545 | 0.0203 | 21 | 140 | 147 | KLDCISLR | | | | | Mascot |
| 1126.4768 | 1126.5721 | 0.0953 | 85 | 148 | 156 | CQFGCQIDR | | | Carbamidomethyl (C)[1] | | Mascot |
| 1162.469 | 1162.5184 | 0.0494 | 42 | 84 | 93 | CSCMEGYKGK | | | Carbamidomethyl (C)[1] | | Mascot |
| 1520.6732 | 1520.8969 | 0.2237 | 147 | 71 | 83 | RPCENGFCIGPDR | | | Carbamidomethyl (C)[3] | | Mascot |
| 2000.8485 | 2001.062 | 0.2135 | 107 | 42 | 59 | APGVCALEPSMTCCYGWR | | | Carbamidomethyl (C)[5] | | Mascot |
| 2040.9412 | 2041.073 | 0.1318 | 65 | 141 | 156 | LDCISLRCQFGCQIDR | | | Carbamidomethyl (C)[3,8,12] | | Mascot |
| 2159.9849 | 2160.0588 | 0.0739 | 34 | 157 | 175 | NDEVGCVCPPGLQLAPDYR | | | Carbamidomethyl (C)[6,8] | | Mascot |
| 2161.822 | 2162.1187 | 0.2967 | 137 | 179 | 197 | DINECDDPSHTCTEHQSCK | | | | | Mascot |

7 26S proteasome non-ATPase regulatory subunit 2 [Danio rerio] gi|41054527 99415.5 5.24 14 43 0

Peptide Information

| Calc. Mass | Obsrv. Mass | ± da | ± ppm | Start Seq. | End Seq. | Sequence | Ion Score | C. I. % | Modification | Rank | Result Type |
|---|---|---|---|---|---|---|---|---|---|---|---|
| 1165.6112 | 1165.7307 | 0.1195 | 103 | 249 | 257 | FNRYPEALR | | | | | Mascot |
| 1473.7842 | 1473.7635 | -0.0207 | -14 | 432 | 446 | SGALLACGIVNSGVR | | | Carbamidomethyl (C)[7] | | Mascot |
| 1520.9159 | 1520.8969 | -0.019 | -12 | 678 | 692 | AVPLALALISVSNPR | | | | | Mascot |
| 1629.8893 | 1629.7854 | -0.1039 | -64 | 730 | 743 | LAAMLRQLAQYHAK | | | Oxidation (M)[4] | | Mascot |
| 1629.8893 | 1629.7854 | -0.1039 | -64 | 730 | 743 | LAAMLRQLAQYHAK | | | Oxidation (M)[4] | | Mascot |
| 1718.9047 | 1718.8932 | -0.0115 | -7 | 803 | 817 | SHYILYGLVAAMQPR | | | | | Mascot |
| 1810.8905 | 1810.9752 | 0.0847 | 47 | 599 | 613 | VQQLLHICSEHFDNK | | | | | Mascot |
| 1973.9716 | 1973.9464 | -0.0252 | -13 | 134 | 150 | LLGSQEELASWGHEYVR | | | | | Mascot |
| 2001.0837 | 2001.062 | -0.0217 | -11 | 818 | 834 | MLVTFDEELRPLPVSVR | | | | | Mascot |
| 2041.0964 | 2041.073 | -0.0234 | -11 | 560 | 578 | GEAIETTLAALQVVPEPF R | | | | | Mascot |
| 2049.0876 | 2049.0649 | -0.0227 | -11 | 850 | 868 | AITGFQTHTTPVLLAHGE R | | | | | Mascot |
| 2172.0818 | 2171.9614 | -0.1204 | -55 | 159 | 176 | EWQEIEEGDKAQQEVLL K | | | | | Mascot |
| 2225.1191 | 2225.0969 | -0.0222 | -10 | 258 | 276 | LALMLNDVELVENIFTSC K | | | Carbamidomethyl (C)[18], Oxidation (M)[4] | | Mascot |
| 2457.3638 | 2457.344 | -0.0198 | -8 | 869 | 890 | AELATEEFIPVTPILEGFVI LR | | | | | Mascot |
| 2518.4966 | 2518.4089 | -0.0877 | -35 | 678 | 701 | AVPLALALISVSNPRLNIL DTLSK | | | | | Mascot |
| 2518.4966 | 2518.4089 | -0.0877 | -35 | 678 | 701 | AVPLALALISVSNPRLNIL DTLSK | | | | | Mascot |

| | | | | | | | | | | |
|---|---|---|---|---|---|---|---|---|---|---|
| 8 | LOC559407 protein [Danio rerio] | gi\|108742068 | 52727.9 | 5.76 | 8 | 42 | 0 | 17 | 0 |

Peptide Information

| Calc. Mass | Obsrv. Mass | ± da | ± ppm | Start Seq. | End Seq. | Sequence | Ion Score | C. I. % | Modification | Rank | Result Type |
|---|---|---|---|---|---|---|---|---|---|---|---|
| 947.4792 | 947.5545 | 0.0753 | 79 | 102 | 110 | AETDAATLR | | | | | Mascot |
| 1045.5411 | 1045.55 | 0.0089 | 9 | 186 | 194 | DAVEEELLK | | | | | Mascot |
| 1126.6327 | 1126.5721 | -0.0606 | -54 | 363 | 372 | EAPLRVAQSR | | | | | Mascot |
| 1329.7485 | 1329.6761 | -0.0724 | -54 | 208 | 218 | KTLDQTINQIR | 17 | 0 | | | Mascot |
| 1329.7485 | 1329.6761 | -0.0724 | -54 | 208 | 218 | KTLDQTINQIR | | | | | Mascot |
| 1473.7656 | 1473.7635 | -0.0021 | -1 | 283 | 295 | LAEREETASQALR | | | | | Mascot |
| 1816.9058 | 1816.7664 | -0.1394 | -77 | 373 | 386 | LHYRTLRPNMEQCR | | | | | Mascot |
| 2154.9873 | 2154.936 | -0.0513 | -24 | 257 | 274 | HPSSAVLQDQVCDEEVW R | | | Carbamidomethyl (C)[12] | | Mascot |
| 2283.0823 | 2283.1531 | 0.0708 | 31 | 257 | 275 | HPSSAVLQDQVCDEEVW RK | | | Carbamidomethyl (C)[12] | | Mascot |

| | | | | | | | | | | |
|---|---|---|---|---|---|---|---|---|---|---|
| 9 | tektin-4 [Danio rerio] | gi\|224589086 | 51528.2 | 5.75 | 8 | 41 | 0 | 17 | 0 |

**Peptide Information**

| Calc. Mass | Obsrv. Mass | ± da | ± ppm | Start Seq. | End Seq. | Sequence | Ion Score | C. I. % | Modification | Rank | Result Type |
|---|---|---|---|---|---|---|---|---|---|---|---|
| 947.4792 | 947.5545 | 0.0753 | 79 | 91 | 99 | AETDAATLR | | | | | Mascot |
| 1045.5411 | 1045.55 | 0.0089 | 9 | 175 | 183 | DAVEEELLK | | | | | Mascot |
| 1126.6327 | 1126.5721 | -0.0606 | -54 | 352 | 361 | EAPLRVAQSR | | | | | Mascot |
| 1329.7485 | 1329.6761 | -0.0724 | -54 | 197 | 207 | KTLDQTINQIR | 17 | 0 | | | Mascot |
| 1329.7485 | 1329.6761 | -0.0724 | -54 | 197 | 207 | KTLDQTINQIR | | | | | Mascot |
| 1473.7656 | 1473.7635 | -0.0021 | -1 | 272 | 284 | LAEREETASQALR | | | | | Mascot |
| 1816.9058 | 1816.7664 | -0.1394 | -77 | 362 | 375 | LHYRTLRPNMEQCR | | | | | Mascot |
| 2154.9873 | 2154.936 | -0.0513 | -24 | 246 | 263 | HPSSAVLQDQVCDEEVW R | | | Carbamidomethyl (C)[12] | | Mascot |
| 2283.0823 | 2283.1531 | 0.0708 | 31 | 246 | 264 | HPSSAVLQDQVCDEEVW RK | | | Carbamidomethyl (C)[12] | | Mascot |

| | | | | | | | | |
|---|---|---|---|---|---|---|---|---|
| 10 | PREDICTED: hypothetical protein LOC100537298 [Danio rerio] | gi\|326667816 | 6120 | 8.38 | 6 | 41 | 0 | |

**Peptide Information**

| Calc. Mass | Obsrv. Mass | ± da | ± ppm | Start Seq. | End Seq. | Sequence | Ion Score | C. I. % | Modification | Rank | Result Type |
|---|---|---|---|---|---|---|---|---|---|---|---|
| 1165.5895 | 1165.7307 | 0.1412 | 121 | 10 | 18 | CLRVNYGQR | | | Carbamidomethyl (C)[1] | | Mascot |
| 1282.6572 | 1282.6714 | 0.0142 | 11 | 2 | 12 | AESSIFTRCLR | | | | | Mascot |
| 1578.7511 | 1578.7739 | 0.0228 | 14 | 38 | 51 | VLSCMHLPTCAIMK | | | Oxidation (M)[5,13] | | Mascot |
| 1578.7511 | 1578.7739 | 0.0228 | 14 | 38 | 51 | VLSCMHLPTCAIMK | | | Oxidation (M)[5,13] | | Mascot |
| 1660.8042 | 1660.7837 | -0.0205 | -12 | 38 | 51 | VLSCMHLPTCAIMK | | | Carbamidomethyl (C)[4,10] | | Mascot |
| 1718.8573 | 1718.8932 | 0.0359 | 21 | 37 | 51 | RVLSCMHLPTCAIMK | | | Oxidation (M)[6] | | Mascot |
| 1816.9053 | 1816.7664 | -0.1389 | -76 | 37 | 51 | RVLSCMHLPTCAIMK | | | Carbamidomethyl (C)[5,11] | | Mascot |
| 1974.0043 | 1973.9464 | -0.0579 | -29 | 38 | 54 | VLSCMHLPTCAIMKVTL | | | Carbamidomethyl (C)[4,10] | | Mascot |
| 2225.0073 | 2225.0969 | 0.0896 | 40 | 19 | 37 | CANDCPDVLYNTAEQSL RR | | | Carbamidomethyl (C)[1] | | Mascot |

| | | | | | | | | |
|---|---|---|---|---|---|---|---|---|
| 11 | PREDICTED: TBC1 domain family member 9 isoform 2 [Danio rerio] | gi\|125804583 | 142731.7 | 5.41 | 16 | 40 | 0 | |

**Peptide Information**

| Calc. Mass | Obsrv. Mass | ± da | ± ppm | Start Seq. | End Seq. | Sequence | Ion Score | C. I. % | Modification | Rank | Result Type |
|---|---|---|---|---|---|---|---|---|---|---|---|
| 842.5457 | 842.5001 | -0.0456 | -54 | 193 | 199 | EVKLVVR | | | | | Mascot |
| 947.4468 | 947.5545 | 0.1077 | 114 | 469 | 476 | SPEEFNPK | | | | | Mascot |
| 1172.5405 | 1172.6554 | 0.1149 | 98 | 628 | 636 | MLPDYYNTR | | | | | Mascot |
| 1556.8795 | 1556.8376 | -0.0419 | -27 | 196 | 208 | LVVRWADVTQLEK | | | | | Mascot |

| | | | | | | | | | | | |
|---|---|---|---|---|---|---|---|---|---|---|---|
| 1629.8629 | 1629.7854 | -0.0775 | -48 | 506 | 519 | TKDLVLQGIPENMR | | | Oxidation (M)[13] | | Mascot |
| 1629.8629 | 1629.7854 | -0.0775 | -48 | 506 | 519 | TKDLVLQGIPENMR | | | Oxidation (M)[13] | | Mascot |
| 1645.5845 | 1645.7821 | 0.1976 | 120 | 1073 | 1085 | HNNEDDDEEEDER | | | | | Mascot |
| 1660.821 | 1660.7837 | -0.0373 | -22 | 1157 | 1170 | LQCEDIADDTVLIR | | | Carbamidomethyl (C)[3] | | Mascot |
| 1973.9385 | 1973.9464 | 0.0079 | 4 | 1231 | 1248 | KVGHQHMSVSDYEISLS G | | | | | Mascot |
| 2001.062 | 2001.062 | 0 | 0 | 241 | 257 | LMEQLANIAMRQLLDNK | | | | | Mascot |
| 2048.8953 | 2049.0649 | 0.1696 | 83 | 489 | 505 | NHFTEYGQGVCMYRTDK | | | | | Mascot |
| 2146.1213 | 2146.1343 | 0.013 | 6 | 908 | 926 | EFISGLSVLCHGDLTEKL K | | | Carbamidomethyl (C)[10] | | Mascot |
| 2160.0793 | 2160.0588 | -0.0205 | -9 | 2 | 19 | WVNPEEVLLASALWMTE R | | | Oxidation (M)[15] | | Mascot |
| 2225.137 | 2225.0969 | -0.0401 | -18 | 1100 | 1119 | KPCDITPPLDEIKLEDSSP K | | | | | Mascot |
| 2457.2231 | 2457.344 | 0.1209 | 49 | 220 | 240 | VSTRCSEHVFSVFLNINE TFK | | | | | Mascot |
| 2484.2122 | 2484.2402 | 0.028 | 11 | 446 | 466 | QFNLNDNRVPTATQALM TMYR | | | | | Mascot |
| 2484.2122 | 2484.2402 | 0.028 | 11 | 446 | 466 | QFNLNDNRVPTATQALM TMYR | | | | | Mascot |
| 2518.262 | 2518.4089 | 0.1469 | 58 | 561 | 582 | DLHRSLPEHPAFQNEMGI AALR | | | Oxidation (M)[16] | | Mascot |
| 2518.262 | 2518.4089 | 0.1469 | 58 | 561 | 582 | DLHRSLPEHPAFQNEMGI AALR | | | Oxidation (M)[16] | | Mascot |

| | | | | | | | |
|---|---|---|---|---|---|---|---|
| 12 | Prmt1 protein [Danio rerio] | gi\|27882547 | 43538.7 | 5.73 | 10 | 39 | 0 |

**Peptide Information**

| Calc. Mass | Obsrv. Mass | ± da | ± ppm | Start Seq. | End Seq. | Sequence | Ion Score | C. I. % | Modification | Rank | Result Type |
|---|---|---|---|---|---|---|---|---|---|---|---|
| 1051.651 | 1051.5104 | -0.1406 | -134 | 142 | 150 | LDHIVTIIK | | | | | Mascot |
| 1051.651 | 1051.7001 | 0.0491 | 47 | 142 | 150 | LDHIVTIIK | | | | | Mascot |
| 1075.618 | 1075.5247 | -0.0933 | -87 | 251 | 260 | QLVSTACLIK | | | | | Mascot |
| 1162.5166 | 1162.5184 | 0.0018 | 2 | 14 | 23 | CVLHELCCSR | | | | | Mascot |
| 1251.658 | 1251.6042 | -0.0538 | -43 | 203 | 213 | ATLYVTAIEDR | | | | | Mascot |
| 1556.7373 | 1556.8376 | 0.1003 | 64 | 364 | 376 | GQLCEVSKTSEYR | | | Carbamidomethyl (C)[4] | | Mascot |
| 1718.7771 | 1718.8932 | 0.1161 | 68 | 10 | 23 | EQNKCVLHELCCSR | | | Carbamidomethyl (C)[5] | | Mascot |
| 1924.0459 | 1923.9279 | -0.118 | -61 | 121 | 138 | VIGIECSSISDYAVKIVK | | | | | Mascot |
| 2172.053 | 2171.9614 | -0.0916 | -42 | 353 | 371 | DLDFTVDIDFKGQLCEVS K | | | | | Mascot |
| 2457.1365 | 2457.344 | 0.2075 | 84 | 217 | 235 | DYKIHWWENVYGLDMSC IK | | | Carbamidomethyl (C)[17] | | Mascot |
| 2484.2129 | 2484.2402 | 0.0273 | 11 | 285 | 304 | NDYIHALVTYFNIEFTRCH K | | | | | Mascot |
| 2484.2129 | 2484.2402 | 0.0273 | 11 | 285 | 304 | NDYIHALVTYFNIEFTRCH K | | | | | Mascot |

| | | | | | | | |
|---|---|---|---|---|---|---|---|
| 13 | Metap2 protein [Danio rerio] | gi\|46249906 | 52802.2 | 5.82 | 10 | 39 | 0 |

Peptide Information

| Calc. Mass | Obsrv. Mass | ± da | ± ppm | Start Seq. | End Seq. | Sequence | Ion Score | C. I. % | Modification | Rank | Result Type |
|---|---|---|---|---|---|---|---|---|---|---|---|
| 819.4207 | 819.4641 | 0.0434 | 53 | 277 | 284 | DATNTGIK | | | | | Mascot |
| 1165.6184 | 1165.7307 | 0.1123 | 96 | 166 | 175 | QAAEAHRQVR | | | | | Mascot |
| 1282.7075 | 1282.6714 | -0.0361 | -28 | 418 | 428 | LGETKYLMALK | | | Oxidation (M)[8] | | Mascot |
| 1533.7689 | 1533.7505 | -0.0184 | -12 | 277 | 291 | DATNTGIKCAGIDVR | | | | | Mascot |
| 1800.7646 | 1800.7736 | 0.009 | 5 | 152 | 165 | FLDQANEDMWNDFR | | | | | Mascot |
| 1816.7595 | 1816.7664 | 0.0069 | 4 | 152 | 165 | FLDQANEDMWNDFR | | | Oxidation (M)[9] | | Mascot |
| 2040.9832 | 2041.073 | 0.0898 | 44 | 46 | 65 | AAAVENHDGEGDTDIAK VTK | | | | | Mascot |
| 2146.1226 | 2146.1343 | 0.0117 | 5 | 394 | 412 | AKHLLNVVNENFGTLAFC R | | | | | Mascot |
| 2156.0588 | 2155.9529 | -0.1059 | -49 | 177 | 194 | YVQSWIKPGMTMIEICEK | | | | | Mascot |
| 2160.113 | 2160.0588 | -0.0542 | -25 | 396 | 413 | HLLNVVNENFGTLAFCRR | | | Carbamidomethyl (C)[16] | | Mascot |
| 2172.0537 | 2171.9614 | -0.0923 | -42 | 177 | 194 | YVQSWIKPGMTMIEICEK | | | Oxidation (M)[10] | | Mascot |
| 2284.1538 | 2284.1499 | -0.0039 | -2 | 176 | 194 | KYVQSWIKPGMTMIEICE K | | | | | Mascot |

| | | | | | | | | | |
|---|---|---|---|---|---|---|---|---|---|
| 14 | Echinoderm microtubule associated protein like 1 [Danio rerio] | gi\|115313535 | 30885.7 | 9.96 | 8 | 39 | 0 | | |

Peptide Information

| Calc. Mass | Obsrv. Mass | ± da | ± ppm | Start Seq. | End Seq. | Sequence | Ion Score | C. I. % | Modification | Rank | Result Type |
|---|---|---|---|---|---|---|---|---|---|---|---|
| 986.5741 | 986.519 | -0.0551 | -56 | 57 | 65 | SALADVVRR | | | | | Mascot |
| 1165.5782 | 1165.7307 | 0.1525 | 131 | 176 | 185 | EPVFSAGMRR | | | Oxidation (M)[8] | | Mascot |
| 1473.7617 | 1473.7635 | 0.0018 | 1 | 45 | 56 | VQMQEDEIQLLK | | | | | Mascot |
| 1533.7577 | 1533.7505 | -0.0072 | -5 | 66 | 78 | LNISEEQQAMLNK | | | Oxidation (M)[10] | | Mascot |
| 1645.8578 | 1645.7821 | -0.0757 | -46 | 66 | 79 | LNISEEQQAMLNKK | | | | | Mascot |
| 1718.8191 | 1718.8932 | 0.0741 | 43 | 212 | 228 | SSSAVPSQSTNNPSEVK | | | | | Mascot |
| 1973.9572 | 1973.9464 | -0.0108 | -5 | 251 | 267 | KCTSQPVHSPLEMPTYR | | | | | Mascot |
| 2457.4485 | 2457.344 | -0.1045 | -43 | 84 | 106 | ARPMIAALPLRPTVNNGT VLPKK | | | | | Mascot |

| | | | | | | | | | |
|---|---|---|---|---|---|---|---|---|---|
| 15 | PREDICTED: atrial natriuretic peptide receptor 1 [Danio rerio] | gi\|292609933 | 123931.2 | 7.58 | 11 | 38 | 0 | 15 | 0 |

Peptide Information

| Calc. Mass | Obsrv. Mass | ± da | ± ppm | Start Seq. | End Seq. | Sequence | Ion Score | C. I. % | Modification | Rank | Result Type |
|---|---|---|---|---|---|---|---|---|---|---|---|
| 842.5246 | 842.5001 | -0.0245 | -29 | 634 | 640 | KAVHFIK | | | | | Mascot |
| 947.468 | 947.5545 | 0.0865 | 91 | 881 | 888 | TAELQEEK | | | | | Mascot |

| Calc. Mass | Obsrv. Mass | ± da | ± ppm | Start Seq. | End Seq. | Sequence | Ion Score | C. I. % | Modification | Rank | Result Type |
|---|---|---|---|---|---|---|---|---|---|---|---|
| 1075.563 | 1075.5247 | -0.0383 | -36 | 881 | 889 | TAELQEEKK | | | | | Mascot |
| 1126.5964 | 1126.5721 | -0.0243 | -22 | 135 | 144 | LGAFFVEICK | | | | | Mascot |
| 1329.7849 | 1329.6761 | -0.1088 | -82 | 902 | 914 | SVASQLIAGKTVR | 15 | 0 | | | Mascot |
| 1329.7849 | 1329.6761 | -0.1088 | -82 | 902 | 914 | SVASQLIAGKTVR | | | | | Mascot |
| 1556.894 | 1556.8376 | -0.0564 | -36 | 990 | 1003 | EIARMSLAIVQGLR | | | | | Mascot |
| 1816.9565 | 1816.7664 | -0.1901 | -105 | 1004 | 1018 | SFHSPHVPEQQLRVR | | | | | Mascot |
| 1923.8688 | 1923.9279 | 0.0591 | 31 | 415 | 431 | DSPDCGFRGELCINLER | | | | | Mascot |
| 1968.0259 | 1967.8976 | -0.1283 | -65 | 12 | 29 | FTLLISMPNTSIPFSSGR | | | | | Mascot |
| 1968.0259 | 1967.8976 | -0.1283 | -65 | 12 | 29 | FTLLISMPNTSIPFSSGR | 4 | 0 | | | Mascot |
| 2049.1636 | 2049.0649 | -0.0987 | -48 | 200 | 218 | VLNEMAQVARIIVISAHGK | | | | | Mascot |
| 2484.2339 | 2484.2402 | 0.0063 | 3 | 400 | 422 | GVQILWPGGLTSPPKDS PDCGFR | | | Carbamidomethyl (C)[20] | | Mascot |
| 2484.2339 | 2484.2402 | 0.0063 | 3 | 400 | 422 | GVQILWPGGLTSPPKDS PDCGFR | | | Carbamidomethyl (C)[20] | | Mascot |

16 Wars protein [Danio rerio] gi|182890772 52294.4 6.24 8 38 0 8 0

Peptide Information

| Calc. Mass | Obsrv. Mass | ± da | ± ppm | Start Seq. | End Seq. | Sequence | Ion Score | C. I. % | Modification | Rank | Result Type |
|---|---|---|---|---|---|---|---|---|---|---|---|
| 1251.6038 | 1251.6042 | 0.0004 | 0 | 214 | 224 | DIIACGFDVNK | | | Carbamidomethyl (C)[5] | | Mascot |
| 1329.7122 | 1329.6761 | -0.0361 | -27 | 105 | 115 | VDQELVDRISR | | | | | Mascot |
| 1329.7122 | 1329.6761 | -0.0361 | -27 | 105 | 115 | VDQELVDRISR | | | | | Mascot |
| 1951.9807 | 1951.9008 | -0.0799 | -41 | 442 | 457 | HKHVTDDIVQQFMTPR | | | | | Mascot |
| 1951.9807 | 1951.9008 | -0.0799 | -41 | 442 | 457 | HKHVTDDIVQQFMTPR | | | | | Mascot |
| 1967.9379 | 1967.8976 | -0.0403 | -20 | 2 | 19 | SSPLDLYECVTAQGETV R | | | | | Mascot |
| 1967.9379 | 1967.8976 | -0.0403 | -20 | 2 | 19 | SSPLDLYECVTAQGETV R | 10 | 0 | | | Mascot |
| 2049.1279 | 2049.0649 | -0.063 | -31 | 156 | 174 | GPSSQAIHVGHLIPFIFTK | | | | | Mascot |
| 2155.9998 | 2155.9529 | -0.0469 | -22 | 1 | 19 | MSSPLDLYECVTAQGET VR | | | Carbamidomethyl (C)[10] | | Mascot |
| 2171.9946 | 2171.9614 | -0.0332 | -15 | 1 | 19 | MSSPLDLYECVTAQGET VR | | | Carbamidomethyl (C)[10], Oxidation (M)[1] | | Mascot |
| 2457.136 | 2457.344 | 0.208 | 85 | 295 | 314 | DVQCLIPCAIDQDPYFRM TR | | | Carbamidomethyl (C)[4], Oxidation (M)[18] | | Mascot |
| 2484.2109 | 2484.2402 | 0.0293 | 12 | 1 | 22 | MSSPLDLYECVTAQGET VRSIK | | | Carbamidomethyl (C)[10] | | Mascot |
| 2484.2109 | 2484.2402 | 0.0293 | 12 | 1 | 22 | MSSPLDLYECVTAQGET VRSIK | | | Carbamidomethyl (C)[10] | | Mascot |

17 PREDICTED: DNA replication licensing factor MCM8-like [Danio rerio] gi|326675408 93684.5 7.98 13 38 0

Peptide Information

| Calc. Mass | Obsrv. Mass | ± da | ± ppm | Start Seq. | End Seq. | Sequence | Ion Score | C. I. % | Modification | Rank | Result Type |
|---|---|---|---|---|---|---|---|---|---|---|---|

| Calc. Mass | Obsrv. Mass | ± da | ± ppm | Start Seq. | End Seq. | Sequence | Ion Score | C. I. % | Modification | Rank | Result Type |
|---|---|---|---|---|---|---|---|---|---|---|---|
| 947.4904 | 947.5545 | 0.0641 | 68 | 629 | 639 | SGAVSGAAATR | | | | | Mascot |
| 989.4972 | 989.4801 | -0.0171 | -17 | 156 | 163 | DLKEMPEK | | | | | Mascot |
| 1051.4772 | 1051.5104 | 0.0332 | 32 | 276 | 284 | CLQSECRGR | | | | | Mascot |
| 1145.7153 | 1145.5791 | -0.1362 | -119 | 230 | 239 | FVVIRGTVVR | | | | | Mascot |
| 1162.5521 | 1162.5184 | -0.0337 | -29 | 305 | 314 | VQELMSGDQR | | | | | Mascot |
| 1251.611 | 1251.6042 | -0.0068 | -5 | 776 | 787 | SQLGSGMSNRSK | | | | | Mascot |
| 1473.7301 | 1473.7635 | 0.0334 | 23 | 473 | 485 | SQMLQAVCNVAPR | | | Carbamidomethyl (C)[8] | | Mascot |
| 1578.8308 | 1578.7739 | -0.0569 | -36 | 805 | 817 | SMFELQDLRQLAK | | | | | Mascot |
| 1578.8599 | 1578.7739 | -0.086 | -54 | 145 | 158 | QVSHSLPDLARDLK | | | | | Mascot |
| 1660.9237 | 1660.7837 | -0.14 | -84 | 235 | 249 | GTVVRVSNIKPLCMK | | | Oxidation (M)[14] | | Mascot |
| 1923.9698 | 1923.9279 | -0.0419 | -22 | 401 | 416 | DLYAIQEIQAQEDLFK | | | | | Mascot |
| 2001.095 | 2001.062 | -0.033 | -16 | 454 | 472 | IPIRGDPHMLIVGDPGLG K | | | Oxidation (M)[9] | | Mascot |
| 2518.2717 | 2518.4089 | 0.1372 | 54 | 66 | 88 | GNAPQVSQARVTQATLDI TCPYK | | | Carbamidomethyl (C)[20] | | Mascot |
| 2518.2717 | 2518.4089 | 0.1372 | 54 | 66 | 88 | GNAPQVSQARVTQATLDI TCPYK | | | Carbamidomethyl (C)[20] | | Mascot |

| | | | | | | | |
|---|---|---|---|---|---|---|---|
| 18 | methionyl aminopeptidase 2 [Danio rerio] | gi\|41054491 | 53147.4 | 5.74 | 10 | 37 | 0 |

Peptide Information

| Calc. Mass | Obsrv. Mass | ± da | ± ppm | Start Seq. | End Seq. | Sequence | Ion Score | C. I. % | Modification | Rank | Result Type |
|---|---|---|---|---|---|---|---|---|---|---|---|
| 819.4207 | 819.4641 | 0.0434 | 53 | 280 | 287 | DATNTGIK | | | | | Mascot |
| 1165.6184 | 1165.7307 | 0.1123 | 96 | 169 | 178 | QAAEAHRQVR | | | | | Mascot |
| 1282.7075 | 1282.6714 | -0.0361 | -28 | 421 | 431 | LGETKYLMALK | | | Oxidation (M)[8] | | Mascot |
| 1533.7689 | 1533.7505 | -0.0184 | -12 | 280 | 294 | DATNTGIKCAGIDVR | | | | | Mascot |
| 1800.7646 | 1800.7736 | 0.009 | 5 | 155 | 168 | FLDQANEDMWNDFR | | | | | Mascot |
| 1816.7595 | 1816.7664 | 0.0069 | 4 | 155 | 168 | FLDQANEDMWNDFR | | | Oxidation (M)[9] | | Mascot |
| 2040.9832 | 2041.073 | 0.0898 | 44 | 49 | 68 | AAAVENHDGEGDTDIAK VTK | | | | | Mascot |
| 2146.1226 | 2146.1343 | 0.0117 | 5 | 397 | 415 | AKHLLNVVNENFGTLAFC R | | | | | Mascot |
| 2156.0588 | 2155.9529 | -0.1059 | -49 | 180 | 197 | YVQSWIKPGMTMIEICEK | | | | | Mascot |
| 2160.113 | 2160.0588 | -0.0542 | -25 | 399 | 416 | HLLNVVNENFGTLAFCRR | | | Carbamidomethyl (C)[16] | | Mascot |
| 2172.0537 | 2171.9614 | -0.0923 | -42 | 180 | 197 | YVQSWIKPGMTMIEICEK | | | Oxidation (M)[10] | | Mascot |
| 2284.1538 | 2284.1499 | -0.0039 | -2 | 179 | 197 | KYVQSWIKPGMTMIEICE K | | | | | Mascot |

| | | | | | | | |
|---|---|---|---|---|---|---|---|
| 19 | hypothetical protein LOC406277 [Danio rerio] | gi\|47086959 | 38211.2 | 9.31 | 8 | 36 | 0 |

Peptide Information

| Calc. Mass | Obsrv. Mass | ± da | ± ppm | Start | End | Sequence | Ion | C. I. % | Modification | Rank | Result Type |
|---|---|---|---|---|---|---|---|---|---|---|---|

| | | | | Seq. | Seq. | | Score | | | | |
|---|---|---|---|---|---|---|---|---|---|---|---|
| 1251.5575 | 1251.6042 | 0.0467 | 37 | 36 | 45 | NFAFVHMDDR | | | | | Mascot |
| 1282.6174 | 1282.6714 | 0.054 | 42 | 226 | 236 | ARPYGVAYDDR | | | | | Mascot |
| 1799.8883 | 1799.783 | -0.1053 | -59 | 95 | 110 | ALFEDYGTVAECAIIK | | | Carbamidomethyl (C)[12] | | Mascot |
| 1816.9149 | 1816.7664 | -0.1485 | -82 | 20 | 35 | SLFSQYGTVTECAIIK | | | Carbamidomethyl (C)[12] | | Mascot |
| 1823.9949 | 1823.894 | -0.1009 | -55 | 237 | 254 | RPGGIPPPPPPPSSAIMR | | | | | Mascot |
| 1823.9949 | 1823.894 | -0.1009 | -55 | 237 | 254 | RPGGIPPPPPPPSSAIMR | | | | | Mascot |
| 2041.1328 | 2041.073 | -0.0598 | -29 | 2 | 19 | VKIFIGNLPQQAEVDELK | | | | | Mascot |
| 2172.1733 | 2171.9614 | -0.2119 | -98 | 1 | 19 | MVKIFIGNLPQQAEVDEL K | | | | | Mascot |
| 2457.0967 | 2457.344 | 0.2473 | 101 | 147 | 167 | SRPRGEEDDYGHPDAGY WPPR | | | | | Mascot |

20 lefty1 [Danio rerio] gi|18858969 41014.5 8.65 10 36 0

**Peptide Information**

| Calc. Mass | Obsrv. Mass | ± da | ± ppm | Start Seq. | End Seq. | Sequence | Ion Score | C. I. % | Modification | Rank | Result Type |
|---|---|---|---|---|---|---|---|---|---|---|---|
| 986.4689 | 986.519 | 0.0501 | 51 | 305 | 312 | NYGYGERK | | | | | Mascot |
| 996.5546 | 996.5847 | 0.0301 | 30 | 58 | 65 | NKYISMLK | | | | | Mascot |
| 1520.7413 | 1520.8969 | 0.1556 | 102 | 20 | 32 | GFTHEDMKDALLK | | | Oxidation (M)[7] | | Mascot |
| 1533.8748 | 1533.7505 | -0.1243 | -81 | 45 | 57 | RDLENLVIPTHVK | | | | | Mascot |
| 1823.9104 | 1823.894 | -0.0164 | -9 | 313 | 328 | CAVVESAPLPMMYLVK | | | Carbamidomethyl (C)[1], Oxidation (M)[11] | | Mascot |
| 1823.9104 | 1823.894 | -0.0164 | -9 | 313 | 328 | CAVVESAPLPMMYLVK | | | Carbamidomethyl (C)[1], Oxidation (M)[11] | | Mascot |
| 1952.0054 | 1951.9008 | -0.1046 | -54 | 313 | 329 | CAVVESAPLPMMYLVKK | | | Carbamidomethyl (C)[1], Oxidation (M)[11] | | Mascot |
| 1952.0599 | 1951.9008 | -0.1591 | -82 | 165 | 181 | TSLVDSRLIPIHETGWK | | | | | Mascot |
| 1968.0002 | 1967.8976 | -0.1026 | -52 | 313 | 329 | CAVVESAPLPMMYLVKK | | | Carbamidomethyl (C)[1], Oxidation (M)[11,12] | | Mascot |
| 1968.0002 | 1967.8976 | -0.1026 | -52 | 312 | 328 | KCAVVESAPLPMMYLVK | | | Carbamidomethyl (C)[2], Oxidation (M)[12,13] | | Mascot |
| 2283.0999 | 2283.1531 | 0.0532 | 23 | 84 | 104 | GIPGNADISGEFVYSDTT RQR | | | | | Mascot |
| 2484.1655 | 2484.2402 | 0.0747 | 30 | 6 | 27 | AACLLCAALFAMARGFTH EDMK | | | Carbamidomethyl (C)[3,6] | | Mascot |
| 2484.1655 | 2484.2402 | 0.0747 | 30 | 6 | 27 | AACLLCAALFAMARGFTH EDMK | | | Carbamidomethyl (C)[3,6] | | Mascot |

21 novel protein similar to TBC1 domain family, member 9 (TBC1D9) [Danio rerio] gi|122890626 138295.5 5.38 15 35 0

**Peptide Information**

| Calc. Mass | Obsrv. Mass | ± da | ± ppm | Start Seq. | End Seq. | Sequence | Ion Score | C. I. % | Modification | Rank | Result Type |
|---|---|---|---|---|---|---|---|---|---|---|---|
| 842.5457 | 842.5001 | -0.0456 | -54 | 152 | 158 | EVKLVVR | | | | | Mascot |
| 947.4468 | 947.5545 | 0.1077 | 114 | 428 | 435 | SPEEFNPK | | | | | Mascot |

| | | | | | | | | | | | |
|---|---|---|---|---|---|---|---|---|---|---|---|
| 1172.5405 | 1172.6554 | 0.1149 | 98 | 587 | 595 | MLPDYYNTR | | | | | Mascot |
| 1556.8795 | 1556.8376 | -0.0419 | -27 | 155 | 167 | LVVRWADVTQLEK | | | | | Mascot |
| 1629.8629 | 1629.7854 | -0.0775 | -48 | 465 | 478 | TKDLVLQGIPENMR | | | Oxidation (M)[13] | | Mascot |
| 1629.8629 | 1629.7854 | -0.0775 | -48 | 465 | 478 | TKDLVLQGIPENMR | | | Oxidation (M)[13] | | Mascot |
| 1645.5845 | 1645.7821 | 0.1976 | 120 | 1032 | 1044 | HNNEDDDEEEDER | | | | | Mascot |
| 1660.821 | 1660.7837 | -0.0373 | -22 | 1116 | 1129 | LQCEDIADDTVLIR | | | Carbamidomethyl (C)[3] | | Mascot |
| 1973.9385 | 1973.9464 | 0.0079 | 4 | 1190 | 1207 | KVGHQHMSVSDYEISLS G | | | | | Mascot |
| 2001.062 | 2001.062 | 0 | 0 | 200 | 216 | LMEQLANIAMRQLLDNK | | | | | Mascot |
| 2048.8953 | 2049.0649 | 0.1696 | 83 | 448 | 464 | NHFTEYGQGVCMYRTDK | | | | | Mascot |
| 2146.1213 | 2146.1343 | 0.013 | 6 | 867 | 885 | EFISGLSVLCHGDLTEKL K | | | Carbamidomethyl (C)[10] | | Mascot |
| 2225.137 | 2225.0969 | -0.0401 | -18 | 1059 | 1078 | KPCDITPPLDEIKLEDSSP K | | | | | Mascot |
| 2457.2231 | 2457.344 | 0.1209 | 49 | 179 | 199 | VSTRCSEHVFSVFLNINE TFK | | | | | Mascot |
| 2484.2122 | 2484.2402 | 0.028 | 11 | 405 | 425 | QFNLNDNRVPTATQALM TMYR | | | | | Mascot |
| 2484.2122 | 2484.2402 | 0.028 | 11 | 405 | 425 | QFNLNDNRVPTATQALM TMYR | | | | | Mascot |
| 2518.262 | 2518.4089 | 0.1469 | 58 | 520 | 541 | DLHRSLPEHPAFQNEMGI AALR | | | Oxidation (M)[16] | | Mascot |
| 2518.262 | 2518.4089 | 0.1469 | 58 | 520 | 541 | DLHRSLPEHPAFQNEMGI AALR | | | Oxidation (M)[16] | | Mascot |

| | | | | | | | |
|---|---|---|---|---|---|---|---|
| 22 | novel protein similar to vertebrate anillin, actin binding protein (scraps homolog, Drosophila) (AN | gi\|94733767 | 120112.9 | 9.01 | 15 | 35 | 0 |

**Peptide Information**

| Calc. Mass | Obsrv. Mass | ± da | ± ppm | Start Seq. | End Seq. | Sequence | Ion Score | C. I. % | Modification | Rank | Result Type |
|---|---|---|---|---|---|---|---|---|---|---|---|
| 819.4182 | 819.4641 | 0.0459 | 56 | 1064 | 1069 | NLWMQK | | | | | Mascot |
| 986.5629 | 986.519 | -0.0439 | -44 | 749 | 757 | AALKAELDR | | | | | Mascot |
| 1067.5223 | 1067.5068 | -0.0155 | -15 | 1044 | 1052 | CKDTLCVTK | | | Carbamidomethyl (C)[1] | | Mascot |
| 1075.5015 | 1075.5247 | 0.0232 | 22 | 730 | 739 | GSQVEAEAER | | | | | Mascot |
| 1126.6102 | 1126.5721 | -0.0381 | -34 | 299 | 309 | STSVVPPSPQK | | | | | Mascot |
| 1172.5802 | 1172.6554 | 0.0752 | 64 | 131 | 141 | MVVCPPQSPAK | | | Oxidation (M)[1] | | Mascot |
| 1251.6943 | 1251.6042 | -0.0901 | -72 | 331 | 342 | IPPSTQPSSPLK | | | | | Mascot |
| 1438.8992 | 1438.7064 | -0.1928 | -134 | 784 | 796 | GSISLLELRLPLK | | | | | Mascot |
| 1520.8141 | 1520.8969 | 0.0828 | 54 | 597 | 610 | KPMTSTPANSFLVK | | | | | Mascot |
| 1556.6533 | 1556.8376 | 0.1843 | 118 | 70 | 83 | CSDENTFLAGEENK | | | | | Mascot |
| 1924.0068 | 1923.9279 | -0.0789 | -41 | 885 | 903 | KSNLQTPVMASPGGPNA VR | | | | | Mascot |
| 2125.042 | 2124.8955 | -0.1465 | -69 | 204 | 223 | SHNLDHPPATPTNSEVP VGR | | | | | Mascot |
| 2125.042 | 2124.8955 | -0.1465 | -69 | 204 | 223 | SHNLDHPPATPTNSEVP | | | | | Mascot |

| Calc. Mass | Obsrv. Mass | ± da | ± ppm | Start Seq. | End Seq. | Sequence | Ion Score | C. I. % | Modification | Rank | Result Type |
|---|---|---|---|---|---|---|---|---|---|---|---|
| | | | | | | VGR | | | | | |
| 2161.9966 | 2162.1187 | 0.1221 | 56 | 677 | 695 | EDVSQRMEMNGSSSHINIK | | | | | Mascot |
| 2484.0999 | 2484.2402 | 0.1403 | 56 | 974 | 993 | WCVLSGYCISYWTYPDDEKR | | | | | Mascot |
| 2484.0999 | 2484.2402 | 0.1403 | 56 | 973 | 992 | RWCVLSGYCISYWTYPDDEK | | | | | Mascot |

| | | | | | | | |
|---|---|---|---|---|---|---|---|
| 23 | leucine-rich repeat LGI family, member 2b [Danio rerio] | gi\|88900439 | 62395.5 | 5.89 | 10 | 35 | 0 |

**Peptide Information**

| Calc. Mass | Obsrv. Mass | ± da | ± ppm | Start Seq. | End Seq. | Sequence | Ion Score | C. I. % | Modification | Rank | Result Type |
|---|---|---|---|---|---|---|---|---|---|---|---|
| 882.4944 | 882.5549 | 0.0605 | 69 | 127 | 133 | YAFRGLR | | | | | Mascot |
| 1242.6113 | 1242.6195 | 0.0082 | 7 | 305 | 314 | YDQAQNKFTK | | | | | Mascot |
| 1282.679 | 1282.6714 | -0.0076 | -6 | 121 | 130 | IEEINKYAFR | | | | | Mascot |
| 1438.7649 | 1438.7064 | -0.0585 | -41 | 134 | 146 | DVTHLSLANNNLK | | | | | Mascot |
| 1578.8527 | 1578.7739 | -0.0788 | -50 | 533 | 545 | TKVFEHILVDYSK | | | | | Mascot |
| 1578.8527 | 1578.7739 | -0.0788 | -50 | 533 | 545 | TKVFEHILVDYSK | | | | | Mascot |
| 1973.9055 | 1973.9464 | 0.0409 | 21 | 184 | 201 | RSNATISDVYCASPSAMK | | | Carbamidomethyl (C)[11], Oxidation (M)[17] | | Mascot |
| 2041.1337 | 2041.073 | -0.0607 | -30 | 13 | 29 | KCLLLTLLFVSSFQLCR | | | Carbamidomethyl (C)[2] | | Mascot |
| 2233.0923 | 2233.0588 | -0.0335 | -15 | 474 | 491 | EQHYLILSSDYSFSQIFR | | | | | Mascot |
| 2284.0439 | 2284.1499 | 0.106 | 46 | 36 | 57 | CPSGCTCTTDSIICIGSSLLPR | | | Carbamidomethyl (C)[1] | | Mascot |
| 2457.0793 | 2457.344 | 0.2647 | 108 | 166 | 184 | GNMFHCDCESMWLMLWLKR | | | Carbamidomethyl (C)[6] | | Mascot |

| | | | | | | | |
|---|---|---|---|---|---|---|---|
| 24 | LOC100148802 protein [Danio rerio] | gi\|120537559 | 28840.7 | 9.21 | 7 | 35 | 0 |

**Peptide Information**

| Calc. Mass | Obsrv. Mass | ± da | ± ppm | Start Seq. | End Seq. | Sequence | Ion Score | C. I. % | Modification | Rank | Result Type |
|---|---|---|---|---|---|---|---|---|---|---|---|
| 986.5628 | 986.519 | -0.0438 | -44 | 36 | 45 | EAKGGLNLGK | | | | | Mascot |
| 1162.6466 | 1162.5184 | -0.1282 | -110 | 153 | 162 | EKFNSTVIPK | | | | | Mascot |
| 1251.6262 | 1251.6042 | -0.022 | -18 | 65 | 74 | GSIMFQERQR | | | | | Mascot |
| 1578.7251 | 1578.7739 | 0.0488 | 31 | 52 | 64 | DVMMEELNLNTNR | | | | | Mascot |
| 1578.7251 | 1578.7739 | 0.0488 | 31 | 52 | 64 | DVMMEELNLNTNR | | | | | Mascot |
| 1718.8132 | 1718.8932 | 0.08 | 47 | 248 | 263 | APRGWGIGYSPESNDL | | | | | Mascot |
| 1810.9309 | 1810.9752 | 0.0443 | 24 | 155 | 169 | FNSTVIPKLYCSPWR | | | | | Mascot |
| 1952.0348 | 1951.9008 | -0.134 | -69 | 134 | 152 | GSPNALAPGYSGPLREIPR | | | | | Mascot |
| 1952.0348 | 1951.9008 | -0.134 | -69 | 134 | 152 | GSPNALAPGYSGPLREIPR | | | | | Mascot |

| | | | | | | | |
|---|---|---|---|---|---|---|---|
| 25 | PREDICTED: putative uncharacterized zinc finger protein 814-like [Danio rerio] | gi\|292610426 | 61962.5 | 9.03 | 14 | 35 | 0 |

Peptide Information

| Calc. Mass | Obsrv. Mass | ± da | ± ppm | Start Seq. | End Seq. | Sequence | Ion Score | C. I. % | Modification | Rank | Result Type |
|---|---|---|---|---|---|---|---|---|---|---|---|
| 996.5472 | 996.5847 | 0.0375 | 38 | 350 | 358 | IHTGEKPSK | | | | | Mascot |
| 1045.5789 | 1045.55 | -0.0289 | -28 | 365 | 373 | SFTHKGQLK | | | | | Mascot |
| 1520.7638 | 1520.8969 | 0.1331 | 88 | 490 | 502 | LHTDERPHVCSVK | | | | | Mascot |
| 1660.7571 | 1660.7837 | 0.0266 | 16 | 210 | 224 | IHTGEKPYTCSHCGK | | | | | Mascot |
| 1718.7625 | 1718.8932 | 0.1307 | 76 | 154 | 168 | LHTEDGPFICGQCGK | | | Carbamidomethyl (C)[10,13] | | Mascot |
| 1799.8414 | 1799.783 | -0.0584 | -32 | 350 | 364 | IHTGEKPSKCPECEK | | | Carbamidomethyl (C)[10,13] | | Mascot |
| 1810.7986 | 1810.9752 | 0.1766 | 98 | 528 | 543 | EQTEELLGQCPFLCGS | | | Carbamidomethyl (C)[10] | | Mascot |
| 1923.9731 | 1923.9279 | -0.0452 | -23 | 2 | 17 | EFIKMEIVDSIDAELR | | | Oxidation (M)[5] | | Mascot |
| 1952.0197 | 1951.9008 | -0.1189 | -61 | 512 | 527 | EVIEEFLLFPETCRVK | | | | | Mascot |
| 1952.0197 | 1951.9008 | -0.1189 | -61 | 512 | 527 | EVIEEFLLFPETCRVK | | | | | Mascot |
| 2146.0281 | 2146.1343 | 0.1062 | 49 | 402 | 420 | THSRTHSGVKPYSCLQCGK | | | Carbamidomethyl (C)[14] | | Mascot |
| 2156.0198 | 2155.9529 | -0.0669 | -31 | 374 | 392 | DHMRIHSGEKPYACPLCAK | | | | | Mascot |
| 2172.0146 | 2171.9614 | -0.0532 | -24 | 374 | 392 | DHMRIHSGEKPYACPLCAK | | | Oxidation (M)[3] | | Mascot |
| 2225.1106 | 2225.0969 | -0.0137 | -6 | 178 | 196 | HHVKIHYAGKPFTCELCGK | | | Carbamidomethyl (C)[14] | | Mascot |
| 2256.022 | 2256.0806 | 0.0586 | 26 | 262 | 280 | SHMRIHTGEKPFSCHQCGK | | | Carbamidomethyl (C)[14], Oxidation (M)[3] | | Mascot |
| 2484.1746 | 2484.2402 | 0.0656 | 26 | 62 | 84 | LDSEDPTIIHTSEKPNTCPVCGK | | | | | Mascot |
| 2484.1746 | 2484.2402 | 0.0656 | 26 | 62 | 84 | LDSEDPTIIHTSEKPNTCPVCGK | | | | | Mascot |

26 protein arginine N-methyltransferase 1 [Danio rerio] gi|41055247 39343.7 5.47 8 35 0

Peptide Information

| Calc. Mass | Obsrv. Mass | ± da | ± ppm | Start Seq. | End Seq. | Sequence | Ion Score | C. I. % | Modification | Rank | Result Type |
|---|---|---|---|---|---|---|---|---|---|---|---|
| 1051.651 | 1051.5104 | -0.1406 | -134 | 105 | 113 | LDHIVTIIK | | | | | Mascot |
| 1051.651 | 1051.7001 | 0.0491 | 47 | 105 | 113 | LDHIVTIIK | | | | | Mascot |
| 1075.618 | 1075.5247 | -0.0933 | -87 | 214 | 223 | QLVSTACLIK | | | | | Mascot |
| 1251.658 | 1251.6042 | -0.0538 | -43 | 166 | 176 | ATLYVTAIEDR | | | | | Mascot |
| 1556.7373 | 1556.8376 | 0.1003 | 64 | 327 | 339 | GQLCEVSKTSEYR | | | Carbamidomethyl (C)[4] | | Mascot |
| 1924.0459 | 1923.9279 | -0.118 | -61 | 84 | 101 | VIGIECSSISDYAVKIVK | | | | | Mascot |
| 1951.8914 | 1951.9008 | 0.0094 | 5 | 2 | 20 | EVSQGESSAKPAAEDMTSK | | | | | Mascot |
| 1951.8914 | 1951.9008 | 0.0094 | 5 | 2 | 20 | EVSQGESSAKPAAEDMTSK | | | | | Mascot |
| 1967.8862 | 1967.8976 | 0.0114 | 6 | 2 | 20 | EVSQGESSAKPAAEDMTSK | | | Oxidation (M)[16] | | Mascot |

| Calc. Mass | Obsrv. Mass | ± da | ± ppm | Start Seq. | End Seq. | Sequence | Ion Score | C. I. % | Modification | Rank | Result Type |
|---|---|---|---|---|---|---|---|---|---|---|---|
| 1967.8862 | 1967.8976 | 0.0114 | 6 | 2 | 20 | EVSQGESSAKPAAEDMTSK | | | Oxidation (M)[16] | | Mascot |
| 2172.053 | 2171.9614 | -0.0916 | -42 | 316 | 334 | DLDFTVDIDFKGQLCEVSK | | | | | Mascot |
| 2484.2129 | 2484.2402 | 0.0273 | 11 | 248 | 267 | NDYIHALVTYFNIEFTRCHK | | | | | Mascot |
| 2484.2129 | 2484.2402 | 0.0273 | 11 | 248 | 267 | NDYIHALVTYFNIEFTRCHK | | | | | Mascot |

| | | | | | | | | | | |
|---|---|---|---|---|---|---|---|---|---|---|
| 27 | pyruvate dehydrogenase kinase, isozyme 3b [Danio rerio] | gi\|123702577 | 21003.7 | 8.49 | 7 | 34 | 0 | | | |

**Peptide Information**

| Calc. Mass | Obsrv. Mass | ± da | ± ppm | Start Seq. | End Seq. | Sequence | Ion Score | C. I. % | Modification | Rank | Result Type |
|---|---|---|---|---|---|---|---|---|---|---|---|
| 882.4468 | 882.5549 | 0.1081 | 123 | 30 | 36 | QFLDFGR | | | | | Mascot |
| 1045.5499 | 1045.55 | 0.0001 | 0 | 43 | 50 | TSYMFLRK | | | | | Mascot |
| 1165.6836 | 1165.7307 | 0.0471 | 40 | 1 | 9 | MKLFICLLK | | | Carbamidomethyl (C)[6] | | Mascot |
| 1556.7162 | 1556.8376 | 0.1214 | 78 | 30 | 42 | QFLDFGRENACEK | | | | | Mascot |
| 1800.8949 | 1800.7736 | -0.1213 | -67 | 118 | 133 | HNDVVPTMAQGVIEYK | | | | | Mascot |
| 1816.8898 | 1816.7664 | -0.1234 | -68 | 118 | 133 | HNDVVPTMAQGVIEYK | | | Oxidation (M)[8] | | Mascot |
| 2155.0852 | 2154.936 | -0.1492 | -69 | 96 | 113 | RSPEDPHTLNDFLEMLIK | | | | | Mascot |
| 2284.1641 | 2284.1499 | -0.0142 | -6 | 97 | 115 | SPEDPHTLNDFLEMLIKIR | | | Oxidation (M)[14] | | Mascot |

| | | | | | | | | | | |
|---|---|---|---|---|---|---|---|---|---|---|
| 28 | tryptophanyl-tRNA synthetase, cytoplasmic [Danio rerio] | gi\|41152126 | 52339.4 | 6.2 | 7 | 34 | 0 | 10 | 0 | |

**Peptide Information**

| Calc. Mass | Obsrv. Mass | ± da | ± ppm | Start Seq. | End Seq. | Sequence | Ion Score | C. I. % | Modification | Rank | Result Type |
|---|---|---|---|---|---|---|---|---|---|---|---|
| 1251.6038 | 1251.6042 | 0.0004 | 0 | 214 | 224 | DIIACGFDVNK | | | Carbamidomethyl (C)[5] | | Mascot |
| 1329.7122 | 1329.6761 | -0.0361 | -27 | 105 | 115 | VDQELVDRISR | | | | | Mascot |
| 1329.7122 | 1329.6761 | -0.0361 | -27 | 105 | 115 | VDQELVDRISR | | | | | Mascot |
| 1967.9379 | 1967.8976 | -0.0403 | -20 | 2 | 19 | SSPLDLYECVTAQGETVR | | | | | Mascot |
| 1967.9379 | 1967.8976 | -0.0403 | -20 | 2 | 19 | SSPLDLYECVTAQGETVR | 10 | 0 | | | Mascot |
| 2049.1279 | 2049.0649 | -0.063 | -31 | 156 | 174 | GPSSQAIHVGHLIPFIFTK | | | | | Mascot |
| 2155.9998 | 2155.9529 | -0.0469 | -22 | 1 | 19 | MSSPLDLYECVTAQGETVR | | | Carbamidomethyl (C)[10] | | Mascot |
| 2171.9946 | 2171.9614 | -0.0332 | -15 | 1 | 19 | MSSPLDLYECVTAQGETVR | | | Carbamidomethyl (C)[10], Oxidation (M)[1] | | Mascot |
| 2457.136 | 2457.344 | 0.208 | 85 | 295 | 314 | DVQCLIPCAIDQDPYFRMTR | | | Carbamidomethyl (C)[4], Oxidation (M)[18] | | Mascot |
| 2484.2109 | 2484.2402 | 0.0293 | 12 | 1 | 22 | MSSPLDLYECVTAQGETVRSIK | | | Carbamidomethyl (C)[10] | | Mascot |
| 2484.2109 | 2484.2402 | 0.0293 | 12 | 1 | 22 | MSSPLDLYECVTAQGETVRSIK | | | Carbamidomethyl (C)[10] | | Mascot |

| | | | | | | | | | | |
|---|---|---|---|---|---|---|---|---|---|---|
| 29 | Tryptophanyl-tRNA synthetase [Danio rerio] | gi\|55777372 | 52313.4 | 6.33 | 7 | 34 | 0 | 10 | 0 | |

**Peptide Information**

| Calc. Mass | Obsrv. Mass | ± da | ± ppm | Start Seq. | End Seq. | Sequence | Ion Score | C. I. % | Modification | Rank | Result Type |
|---|---|---|---|---|---|---|---|---|---|---|---|
| 1251.6038 | 1251.6042 | 0.0004 | 0 | 214 | 224 | DIIACGFDVNK | | | Carbamidomethyl (C)[5] | | Mascot |
| 1329.7122 | 1329.6761 | -0.0361 | -27 | 105 | 115 | VDQELVDRISR | | | | | Mascot |
| 1329.7122 | 1329.6761 | -0.0361 | -27 | 105 | 115 | VDQELVDRISR | | | | | Mascot |
| 1967.9379 | 1967.8976 | -0.0403 | -20 | 2 | 19 | SSPLDLYECVTAQGETVR | | | | | Mascot |
| 1967.9379 | 1967.8976 | -0.0403 | -20 | 2 | 19 | SSPLDLYECVTAQGETVR | 10 | 0 | | | Mascot |
| 2049.1279 | 2049.0649 | -0.063 | -31 | 156 | 174 | GPSSQAIHVGHLIPFIFTK | | | | | Mascot |
| 2155.9998 | 2155.9529 | -0.0469 | -22 | 1 | 19 | MSSPLDLYECVTAQGETVR | | | Carbamidomethyl (C)[10] | | Mascot |
| 2171.9946 | 2171.9614 | -0.0332 | -15 | 1 | 19 | MSSPLDLYECVTAQGETVR | | | Carbamidomethyl (C)[10], Oxidation (M)[1] | | Mascot |
| 2457.136 | 2457.344 | 0.208 | 85 | 295 | 314 | DVQCLIPCAIDQDPYFRMTR | | | Carbamidomethyl (C)[4], Oxidation (M)[18] | | Mascot |
| 2484.2109 | 2484.2402 | 0.0293 | 12 | 1 | 22 | MSSPLDLYECVTAQGETVRSIK | | | Carbamidomethyl (C)[10] | | Mascot |
| 2484.2109 | 2484.2402 | 0.0293 | 12 | 1 | 22 | MSSPLDLYECVTAQGETVRSIK | | | Carbamidomethyl (C)[10] | | Mascot |

30 coiled-coil domain-containing protein 80 precursor [Danio rerio] gi|55742450 99857.2 9.69 10 34 0 21 0

**Peptide Information**

| Calc. Mass | Obsrv. Mass | ± da | ± ppm | Start Seq. | End Seq. | Sequence | Ion Score | C. I. % | Modification | Rank | Result Type |
|---|---|---|---|---|---|---|---|---|---|---|---|
| 842.459 | 842.5001 | 0.0411 | 49 | 90 | 97 | RPAQGGTR | | | | | Mascot |
| 1045.5312 | 1045.55 | 0.0188 | 18 | 599 | 606 | IDHYQLEK | | | | | Mascot |
| 1329.7485 | 1329.6761 | -0.0724 | -54 | 771 | 782 | EGISATLVRDIR | 21 | 0 | | | Mascot |
| 1329.7485 | 1329.6761 | -0.0724 | -54 | 771 | 782 | EGISATLVRDIR | | | | | Mascot |
| 1438.6379 | 1438.7064 | 0.0685 | 48 | 66 | 77 | QQMQGDEGVQFR | | | Oxidation (M)[3] | | Mascot |
| 1578.7441 | 1578.7739 | 0.0298 | 19 | 66 | 78 | QQMQGDEGVQFRR | | | | | Mascot |
| 1578.7441 | 1578.7739 | 0.0298 | 19 | 65 | 77 | RQQMQGDEGVQFR | | | | | Mascot |
| 1660.8363 | 1660.7837 | -0.0526 | -32 | 599 | 611 | IDHYQLEKDKPMK | | | Oxidation (M)[12] | | Mascot |
| 2041.0166 | 2041.073 | 0.0564 | 28 | 150 | 166 | LMMSLLKPEVYCELAER | | | Oxidation (M)[2] | | Mascot |
| 2233.1155 | 2233.0588 | -0.0567 | -25 | 336 | 356 | STTTTTTTTTTTTRPTTTTTR | | | | | Mascot |
| 2457.1941 | 2457.344 | 0.1499 | 61 | 232 | 251 | YPYPVRLEAMYEVIDQSPMR | | | | | Mascot |

31 PREDICTED: myozenin-2 [Danio rerio] gi|292620473 31899.1 9.54 7 33 0

**Peptide Information**

| Calc. Mass | Obsrv. Mass | ± da | ± ppm | Start Seq. | End Seq. | Sequence | Ion Score | C. I. % | Modification | Rank | Result Type |
|---|---|---|---|---|---|---|---|---|---|---|---|

| | | | | | | | | | | | | |
|---|---|---|---|---|---|---|---|---|---|---|---|---|
| 986.5628 | 986.519 | -0.0438 | -44 | 60 | 69 | EAKGGLNLGK | | | | | Mascot |
| 1162.6466 | 1162.5184 | -0.1282 | -110 | 177 | 186 | EKFNSTVIPK | | | | | Mascot |
| 1251.6262 | 1251.6042 | -0.022 | -18 | 89 | 98 | GSIMFQERQR | | | | | Mascot |
| 1578.7251 | 1578.7739 | 0.0488 | 31 | 76 | 88 | DVMMEELNLNTNR | | | | | Mascot |
| 1578.7251 | 1578.7739 | 0.0488 | 31 | 76 | 88 | DVMMEELNLNTNR | | | | | Mascot |
| 1718.8132 | 1718.8932 | 0.08 | 47 | 272 | 287 | APRGWGIGYSPESNDL | | | | | Mascot |
| 1810.9309 | 1810.9752 | 0.0443 | 24 | 179 | 193 | FNSTVIPKLYCSPWR | | | | | Mascot |
| 1952.0348 | 1951.9008 | -0.134 | -69 | 158 | 176 | GSPNALAPGYSGPLREIP R | | | | | Mascot |
| 1952.0348 | 1951.9008 | -0.134 | -69 | 158 | 176 | GSPNALAPGYSGPLREIP R | | | | | Mascot |

| | | | | | | | |
|---|---|---|---|---|---|---|---|
| 32 | leucine-rich repeat LGI family, member 2b [Danio rerio] | gi\|220679474 | 63071.9 | 6.09 | 10 | 33 | 0 |

**Peptide Information**

| Calc. Mass | Obsrv. Mass | ± da | ± ppm | Start Seq. | End Seq. | Sequence | Ion Score | C. I. % | Modification | Rank | Result Type |
|---|---|---|---|---|---|---|---|---|---|---|---|
| 882.4944 | 882.5549 | 0.0605 | 69 | 133 | 139 | YAFRGLR | | | | | Mascot |
| 1242.6113 | 1242.6195 | 0.0082 | 7 | 311 | 320 | YDQAQNKFTK | | | | | Mascot |
| 1282.679 | 1282.6714 | -0.0076 | -6 | 127 | 136 | IEEINKYAFR | | | | | Mascot |
| 1438.7649 | 1438.7064 | -0.0585 | -41 | 140 | 152 | DVTHLSLANNNLK | | | | | Mascot |
| 1578.8527 | 1578.7739 | -0.0788 | -50 | 539 | 551 | TKVFEHILVDYSK | | | | | Mascot |
| 1578.8527 | 1578.7739 | -0.0788 | -50 | 539 | 551 | TKVFEHILVDYSK | | | | | Mascot |
| 1973.9055 | 1973.9464 | 0.0409 | 21 | 190 | 207 | RSNATISDVYCASPSAMK | | | Carbamidomethyl (C)[11], Oxidation (M)[17] | | Mascot |
| 2139.1816 | 2138.9331 | -0.2485 | -116 | 20 | 37 | CLLLTLLFVGSFQLCRAK | | | Carbamidomethyl (C)[1,15] | | Mascot |
| 2233.0923 | 2233.0588 | -0.0335 | -15 | 480 | 497 | EQHYLILSSDYSFSQIFR | | | | | Mascot |
| 2284.0439 | 2284.1499 | 0.106 | 46 | 42 | 63 | CPSGCTCTTDSIICIGSSL LPR | | | Carbamidomethyl (C)[1] | | Mascot |
| 2457.0793 | 2457.344 | 0.2647 | 108 | 172 | 190 | GNMFHCDCESMWLMLW LKR | | | Carbamidomethyl (C)[6] | | Mascot |

| | | | | | | | |
|---|---|---|---|---|---|---|---|
| 33 | Zgc:152774 protein [Danio rerio] | gi\|62202673 | 48140.9 | 9.53 | 8 | 33 | 0 |

**Peptide Information**

| Calc. Mass | Obsrv. Mass | ± da | ± ppm | Start Seq. | End Seq. | Sequence | Ion Score | C. I. % | Modification | Rank | Result Type |
|---|---|---|---|---|---|---|---|---|---|---|---|
| 1075.5128 | 1075.5247 | 0.0119 | 11 | 80 | 88 | MLSFGFTEK | | | Oxidation (M)[1] | | Mascot |
| 1162.5997 | 1162.5184 | -0.0813 | -70 | 336 | 346 | VGCQIKSSGQR | | | | | Mascot |
| 1578.7303 | 1578.7739 | 0.0436 | 28 | 317 | 328 | EHFGIMMYHKNR | | | Oxidation (M)[6] | | Mascot |
| 1578.7303 | 1578.7739 | 0.0436 | 28 | 317 | 328 | EHFGIMMYHKNR | | | Oxidation (M)[6] | | Mascot |
| 1967.9677 | 1967.8976 | -0.0701 | -36 | 122 | 140 | NGGCQSVGMLSQSFLQA IK | | | | | Mascot |

| | | | | | | | | | | | | |
|---|---|---|---|---|---|---|---|---|---|---|---|---|
| | 1967.9677 | 1967.8976 | -0.0701 | -36 | 122 | 140 | NGGCQSVGMLSQSFLQAIK | | | | | Mascot |
| | 1973.8579 | 1973.9464 | 0.0885 | 45 | 59 | 76 | DQLCLSFTDNGSGMTPSK | | | Carbamidomethyl (C)[4], Oxidation (M)[14] | | Mascot |
| | 2040.9841 | 2041.073 | 0.0889 | 44 | 122 | 140 | NGGCQSVGMLSQSFLQAIK | | | Carbamidomethyl (C)[4], Oxidation (M)[9] | | Mascot |
| | 2139.1379 | 2138.9331 | -0.2048 | -96 | 347 | 366 | SGVGVIGVIECNFLKPAHNK | | | Carbamidomethyl (C)[11] | | Mascot |
| | 2457.2144 | 2457.344 | 0.1296 | 53 | 174 | 194 | YSLFQSESELQEQLDSIQGKK | | | | | Mascot |
| | 3081.6213 | 3081.4858 | -0.1355 | -44 | 141 | 169 | AQAVIVPIAPFNQQTNALVVTEDSEASLR | | | | | Mascot |

| | | | | | | | | |
|---|---|---|---|---|---|---|---|---|
| 34 | PREDICTED: echinoderm microtubule associated protein like 6 [Danio rerio] | gi\|326672044 | 191942.5 | 7.32 | 18 | 33 | 0 | |

**Peptide Information**

| Calc. Mass | Obsrv. Mass | ± da | ± ppm | Start Seq. | End Seq. | Sequence | Ion Score | C. I. % | Modification | Rank | Result Type |
|---|---|---|---|---|---|---|---|---|---|---|---|
| 842.4617 | 842.5001 | 0.0384 | 46 | 433 | 439 | EDLPQLK | | | | | Mascot |
| 996.5546 | 996.5847 | 0.0301 | 30 | 714 | 721 | CLKTYAIK | | | Carbamidomethyl (C)[1] | | Mascot |
| 1051.5167 | 1051.5104 | -0.0063 | -6 | 519 | 528 | DYVASGQVGR | | | | | Mascot |
| 1051.651 | 1051.7001 | 0.0491 | 47 | 1117 | 1126 | AAPLPDKLVK | | | | | Mascot |
| 1145.5143 | 1145.5791 | 0.0648 | 57 | 1077 | 1085 | EKNMDYTTK | | | Oxidation (M)[4] | | Mascot |
| 1172.6382 | 1172.6554 | 0.0172 | 15 | 1544 | 1553 | DRSVAIQDIR | | | | | Mascot |
| 1282.6677 | 1282.6714 | 0.0037 | 3 | 983 | 994 | LLATGDDFGFVK | | | | | Mascot |
| 1533.7994 | 1533.7505 | -0.0489 | -32 | 176 | 188 | FWALCGNALTPKR | | | Carbamidomethyl (C)[5] | | Mascot |
| 1578.8123 | 1578.7739 | -0.0384 | -24 | 862 | 875 | EIISDIRFSQDAGK | | | | | Mascot |
| 1578.8123 | 1578.7739 | -0.0384 | -24 | 862 | 875 | EIISDIRFSQDAGK | | | | | Mascot |
| 1800.8737 | 1800.7736 | -0.1001 | -56 | 1717 | 1732 | YVISVGGNDCSVFVWR | | | | | Mascot |
| 1816.8759 | 1816.7664 | -0.1095 | -60 | 548 | 564 | GQHQRGVCALEFTADGK | | | | | Mascot |
| 1823.9208 | 1823.894 | -0.0268 | -15 | 296 | 311 | ILAGTQDSEIFEVMVR | | | Oxidation (M)[14] | | Mascot |
| 1823.9208 | 1823.894 | -0.0268 | -15 | 296 | 311 | ILAGTQDSEIFEVMVR | | | Oxidation (M)[14] | | Mascot |
| 1968.076 | 1967.8976 | -0.1784 | -91 | 723 | 741 | AALSPSSKGLLLEDNPSIR | | | | | Mascot |
| 1968.076 | 1967.8976 | -0.1784 | -91 | 723 | 741 | AALSPSSKGLLLEDNPSIR | | | | | Mascot |
| 2139.0862 | 2138.9331 | -0.1531 | -72 | 1616 | 1634 | QIHEVPSGKLVSDQALMDR | | | Oxidation (M)[17] | | Mascot |
| 2160.0325 | 2160.0588 | 0.0263 | 12 | 897 | 915 | VGICKGASSCITHVDWDVR | | | Carbamidomethyl (C)[4,10] | | Mascot |
| 2283.2019 | 2283.1531 | -0.0488 | -21 | 1285 | 1304 | IFVVEFRPDSDTQFVSVGIK | | | | | Mascot |
| 3081.3723 | 3081.4858 | 0.1135 | 37 | 231 | 260 | TIQAAHGAGIFSMHSCEEGFATGGRDGCVR | | | Oxidation (M)[13] | | Mascot |
| 3672.8223 | 3672.7708 | -0.0515 | -14 | 765 | 799 | SGPMTLLVQGHMEGEVWGLAAHPLLPICATVSDDK | | | | | Mascot |
| 3672.8223 | 3672.7708 | -0.0515 | -14 | 765 | 799 | SGPMTLLVQGHMEGEV | | | | | Mascot |

| | | | | | | | |
|---|---|---|---|---|---|---|---|
| | | | | WGLAAHPLLPICATVSDD K | | | |
| 35 | novel protein [Danio rerio] | gi\|220679558 | 45035 | 4.99 | 8 | 32 | 0 |

Peptide Information

| Calc. Mass | Obsrv. Mass | ± da | ± ppm | Start Seq. | End Seq. | Sequence | Ion Score | C. I. % | Modification | Rank | Result Type |
|---|---|---|---|---|---|---|---|---|---|---|---|
| 819.4094 | 819.4641 | 0.0547 | 67 | 375 | 381 | LDETDVK | | | | | Mascot |
| 842.473 | 842.5001 | 0.0271 | 32 | 382 | 388 | DPKDVLR | | | | | Mascot |
| 882.4791 | 882.5549 | 0.0758 | 86 | 1 | 7 | VSTYTRR | | | | | Mascot |
| 989.5197 | 989.4801 | -0.0396 | -40 | 236 | 243 | LRAEMQNK | | | | | Mascot |
| 1045.5889 | 1045.55 | -0.0389 | -37 | 54 | 62 | VIDTLESLR | | | | | Mascot |
| 1242.5541 | 1242.6195 | 0.0654 | 53 | 7 | 16 | RCPGCQMIYR | | | Oxidation (M)[7] | | Mascot |
| 1718.8629 | 1718.8932 | 0.0303 | 18 | 202 | 215 | LVEELMKQEVDDVR | | | Oxidation (M)[6] | | Mascot |
| 2256.0278 | 2256.0806 | 0.0528 | 23 | 253 | 274 | ICGSSGGCSVVMCPHGV VYSLK | | | Carbamidomethyl (C)[2], Oxidation (M)[12] | | Mascot |

| | | | | | | | |
|---|---|---|---|---|---|---|---|
| 36 | TUWD12 [Danio rerio] | gi\|37682095 | 54354 | 6.31 | 9 | 32 | 0 |

Peptide Information

| Calc. Mass | Obsrv. Mass | ± da | ± ppm | Start Seq. | End Seq. | Sequence | Ion Score | C. I. % | Modification | Rank | Result Type |
|---|---|---|---|---|---|---|---|---|---|---|---|
| 819.4458 | 819.4641 | 0.0183 | 22 | 454 | 460 | LTLTEDK | | | | | Mascot |
| 1045.5789 | 1045.55 | -0.0289 | -28 | 212 | 219 | IWDIRTNK | | | | | Mascot |
| 1162.6313 | 1162.5184 | -0.1129 | -97 | 117 | 127 | LVTASDDKSVK | | | | | Mascot |
| 1165.5708 | 1165.7307 | 0.1599 | 137 | 107 | 116 | SVNFSRDGQR | | | | | Mascot |
| 1172.6497 | 1172.6554 | 0.0057 | 5 | 41 | 50 | SLMIWNLAPK | | | | | Mascot |
| 1815.8442 | 1815.782 | -0.0622 | -34 | 16 | 31 | GHKDVISCADFNPNNK | | | Carbamidomethyl (C)[8] | | Mascot |
| 2000.854 | 2001.062 | 0.208 | 104 | 395 | 410 | THTQAREEEDENQEER | | | | | Mascot |
| 2139.2073 | 2138.9331 | -0.2742 | -128 | 262 | 280 | LIYTLHGHKGPVLTVTFS R | | | | | Mascot |
| 2160.0537 | 2160.0588 | 0.0051 | 2 | 463 | 480 | TCLDNQVLLMQQNQQLD R | | | | | Mascot |
| 2233.0701 | 2233.0588 | -0.0113 | -5 | 463 | 480 | TCLDNQVLLMQQNQQLD R | | | Carbamidomethyl (C)[2], Oxidation (M)[10] | | Mascot |

| | | | | | | | | | |
|---|---|---|---|---|---|---|---|---|---|
| 37 | novel protein similar to H.sapiens natriuretic peptide receptor [Danio rerio] | gi\|220679014 | 36034.1 | 6.04 | 5 | 32 | 0 | 15 | 0 |

Peptide Information

| Calc. Mass | Obsrv. Mass | ± da | ± ppm | Start Seq. | End Seq. | Sequence | Ion Score | C. I. % | Modification | Rank | Result Type |
|---|---|---|---|---|---|---|---|---|---|---|---|
| 947.468 | 947.5545 | 0.0865 | 91 | 100 | 107 | TAELQEEK | | | | | Mascot |
| 1075.563 | 1075.5247 | -0.0383 | -36 | 100 | 108 | TAELQEEKK | | | | | Mascot |

| | | | | | | | | | | | |
|---|---|---|---|---|---|---|---|---|---|---|---|
| 1329.7849 | 1329.6761 | -0.1088 | -82 | 121 | 133 | SVASQLIAGKTVR | 15 | 0 | | | Mascot |
| 1329.7849 | 1329.6761 | -0.1088 | -82 | 121 | 133 | SVASQLIAGKTVR | | | | | Mascot |
| 1556.894 | 1556.8376 | -0.0564 | -36 | 209 | 222 | EIARMSLAIVQGLR | | | | | Mascot |
| 1816.9565 | 1816.7664 | -0.1901 | -105 | 223 | 237 | SFHSPHVPEQQLRVR | | | | | Mascot |

| | | | | | | | |
|---|---|---|---|---|---|---|---|
| 38 | LOC100148802 protein [Danio rerio] | gi\|148745689 | 29506 | 9.62 | 7 | 32 | 0 |

**Peptide Information**

| Calc. Mass | Obsrv. Mass | ± da | ± ppm | Start Seq. | End Seq. | Sequence | Ion Score | C. I. % | Modification | Rank | Result Type |
|---|---|---|---|---|---|---|---|---|---|---|---|
| 986.5628 | 986.519 | -0.0438 | -44 | 37 | 46 | EAKGGLNLGK | | | | | Mascot |
| 1162.6466 | 1162.5184 | -0.1282 | -110 | 154 | 163 | EKFNSTVIPK | | | | | Mascot |
| 1251.6262 | 1251.6042 | -0.022 | -18 | 66 | 75 | GSIMFQERQR | | | | | Mascot |
| 1578.7251 | 1578.7739 | 0.0488 | 31 | 53 | 65 | DVMMEELNLNTNR | | | | | Mascot |
| 1578.7251 | 1578.7739 | 0.0488 | 31 | 53 | 65 | DVMMEELNLNTNR | | | | | Mascot |
| 1718.8132 | 1718.8932 | 0.08 | 47 | 249 | 264 | APRGWGIGYSPESNDL | | | | | Mascot |
| 1810.9309 | 1810.9752 | 0.0443 | 24 | 156 | 170 | FNSTVIPKLYCSPWR | | | | | Mascot |
| 1952.0348 | 1951.9008 | -0.134 | -69 | 135 | 153 | GSPNALAPGYSGPLREIP R | | | | | Mascot |
| 1952.0348 | 1951.9008 | -0.134 | -69 | 135 | 153 | GSPNALAPGYSGPLREIP R | | | | | Mascot |

| | | | | | | | |
|---|---|---|---|---|---|---|---|
| 39 | novel protein similar to vertebrate leucine rich repeat (in FLII) interacting protein family [Danio | gi\|169154866 | 56467.8 | 6.27 | 11 | 32 | 0 |

**Peptide Information**

| Calc. Mass | Obsrv. Mass | ± da | ± ppm | Start Seq. | End Seq. | Sequence | Ion Score | C. I. % | Modification | Rank | Result Type |
|---|---|---|---|---|---|---|---|---|---|---|---|
| 819.4219 | 819.4641 | 0.0422 | 51 | 81 | 86 | YSRHTR | | | | | Mascot |
| 842.5458 | 842.5001 | -0.0457 | -54 | 291 | 297 | KILALER | | | | | Mascot |
| 947.4727 | 947.5545 | 0.0818 | 86 | 314 | 320 | DEVCRLR | | | Carbamidomethyl (C)[4] | | Mascot |
| 1145.6022 | 1145.5791 | -0.0231 | -20 | 125 | 136 | ASSARASPVGSR | | | | | Mascot |
| 1520.7737 | 1520.8969 | 0.1232 | 81 | 325 | 339 | KNGIVMGSEVTANGK | | | Oxidation (M)[6] | | Mascot |
| 1629.8442 | 1629.7854 | -0.0588 | -36 | 411 | 424 | SEQEVTALEQNIIR | | | | | Mascot |
| 1629.8442 | 1629.7854 | -0.0588 | -36 | 411 | 424 | SEQEVTALEQNIIR | | | | | Mascot |
| 1645.869 | 1645.7821 | -0.0869 | -53 | 326 | 341 | NGIVMGSEVTANGKIR | | | | | Mascot |
| 1718.7228 | 1718.8932 | 0.1704 | 99 | 68 | 80 | WGHIEQWMEDSER | | | Oxidation (M)[8] | | Mascot |
| 1799.8671 | 1799.783 | -0.0841 | -47 | 104 | 120 | GSLRPSDYNGFLGSSSR | | | | | Mascot |
| 2000.8899 | 2001.062 | 0.1721 | 86 | 157 | 175 | GSCDTSISADTEASIREM K | | | | | Mascot |
| 2124.9192 | 2124.8955 | -0.0237 | -11 | 68 | 83 | WGHIEQWMEDSERYSR | | | Oxidation (M)[8] | | Mascot |

| | | | | | | | | | | | | |
|---|---|---|---|---|---|---|---|---|---|---|---|---|
| 2124.9192 | 2124.8955 | -0.0237 | -11 | 68 | 83 | WGHIEQWMEDSERYSR | | | Oxidation (M)[8] | | Mascot |

40 Zgc:158627 [Danio rerio] gi|120537829 45729.4 9.41 8 32 0

Peptide Information

| Calc. Mass | Obsrv. Mass | ± da | ± ppm | Start Seq. | End Seq. | Sequence | Ion Score | C. I. % | Modification | Rank | Result Type |
|---|---|---|---|---|---|---|---|---|---|---|---|
| 947.454 | 947.5545 | 0.1005 | 106 | 362 | 371 | AAAGESQTGR | | | | | Mascot |
| 1473.7292 | 1473.7635 | 0.0343 | 23 | 375 | 388 | SKPDANISSQQNGK | | | | | Mascot |
| 1556.7373 | 1556.8376 | 0.1003 | 64 | 346 | 361 | SGAETASGMAAFSLTR | | | | | Mascot |
| 1578.7832 | 1578.7739 | -0.0093 | -6 | 288 | 301 | EVSPCYKSSPPLSK | | | Carbamidomethyl (C)[5] | | Mascot |
| 1578.7832 | 1578.7739 | -0.0093 | -6 | 288 | 301 | EVSPCYKSSPPLSK | | | Carbamidomethyl (C)[5] | | Mascot |
| 1629.8303 | 1629.7854 | -0.0449 | -28 | 374 | 388 | RSKPDANISSQQNGK | | | | | Mascot |
| 1629.8303 | 1629.7854 | -0.0449 | -28 | 374 | 388 | RSKPDANISSQQNGK | | | | | Mascot |
| 1816.9122 | 1816.7664 | -0.1458 | -80 | 179 | 194 | IPEFIRSGHSGTLSCR | | | Carbamidomethyl (C)[15] | | Mascot |
| 2001.0247 | 2001.062 | 0.0373 | 19 | 42 | 62 | EEDRLALAGTAGLAGDA AATK | | | | | Mascot |
| 2146.1138 | 2146.1343 | 0.0205 | 10 | 389 | 408 | LRQYSESPGETGGIVSQ KPL | | | | | Mascot |

41 beta-sarcoglycan [Danio rerio] gi|77993312 34432.7 8.71 8 32 0

Peptide Information

| Calc. Mass | Obsrv. Mass | ± da | ± ppm | Start Seq. | End Seq. | Sequence | Ion Score | C. I. % | Modification | Rank | Result Type |
|---|---|---|---|---|---|---|---|---|---|---|---|
| 819.4094 | 819.4641 | 0.0547 | 67 | 147 | 153 | LSVEEDK | | | | | Mascot |
| 1067.519 | 1067.5068 | -0.0122 | -11 | 222 | 231 | GNEGVYIMGK | | | | | Mascot |
| 1162.5739 | 1162.5184 | -0.0555 | -48 | 36 | 45 | AGYVPIEEER | | | | | Mascot |
| 1473.7994 | 1473.7635 | -0.0359 | -24 | 106 | 118 | QKADMGIVHPLHK | | | | | Mascot |
| 1520.8253 | 1520.8969 | 0.0716 | 47 | 218 | 231 | AIIRGNEGVYIMGK | | | | | Mascot |
| 1540.8118 | 1540.7844 | -0.0274 | -18 | 36 | 48 | AGYVPIEEERLHK | | | | | Mascot |
| 1800.9238 | 1800.7736 | -0.1502 | -83 | 126 | 141 | DQDLVITGNNNPVVFR | | | | | Mascot |
| 2282.9587 | 2283.1531 | 0.1944 | 85 | 293 | 313 | VQVKYSNMGCQTSDNP CGAAH | | | Carbamidomethyl (C)[10], Oxidation (M)[8] | | Mascot |

42 rho-kinase 2 [Danio rerio] gi|291163435 162039.2 5.75 17 31 0

Peptide Information

| Calc. Mass | Obsrv. Mass | ± da | ± ppm | Start Seq. | End Seq. | Sequence | Ion Score | C. I. % | Modification | Rank | Result Type |
|---|---|---|---|---|---|---|---|---|---|---|---|
| 819.4207 | 819.4641 | 0.0434 | 53 | 810 | 816 | SSEQQLK | | | | | Mascot |
| 989.5699 | 989.4801 | -0.0898 | -91 | 932 | 939 | VMKELEIK | | | | | Mascot |
| 996.522 | 996.5847 | 0.0627 | 63 | 945 | 952 | HRQDLAEK | | | | | Mascot |

| | | | | | | | | | | | |
|---|---|---|---|---|---|---|---|---|---|---|---|
| 1051.5782 | 1051.5104 | -0.0678 | -64 | 61 | 69 | YEKAVSNLK | | | | | Mascot |
| 1051.5782 | 1051.7001 | 0.1219 | 116 | 61 | 69 | YEKAVSNLK | | | | | Mascot |
| 1126.5851 | 1126.5721 | -0.013 | -12 | 536 | 545 | NQISHLSAEK | | | | | Mascot |
| 1242.5306 | 1242.6195 | 0.0889 | 72 | 748 | 757 | ENSVLDCDYK | | | Carbamidomethyl (C)[7] | | Mascot |
| 1251.6804 | 1251.6042 | -0.0762 | -61 | 758 | 767 | QAKHELQELR | | | | | Mascot |
| 1282.7378 | 1282.6714 | -0.0664 | -52 | 87 | 98 | GAFGAVQLVRHK | | | | | Mascot |
| 1329.6719 | 1329.6761 | 0.0042 | 3 | 1000 | 1010 | QMKSLTVSYEK | | | Oxidation (M)[2] | | Mascot |
| 1329.6831 | 1329.6761 | -0.007 | -5 | 200 | 210 | DIKPDNMLLDR | | | | | Mascot |
| 1438.7358 | 1438.7064 | -0.0294 | -20 | 310 | 322 | DAKDLICAFLSSR | | | | | Mascot |
| 1473.7908 | 1473.7635 | -0.0273 | -19 | 659 | 670 | RQLQEDLTTLEK | | | | | Mascot |
| 1520.7566 | 1520.8969 | 0.1403 | 92 | 329 | 341 | TGVDEIKCHPFFK | | | | | Mascot |
| 1629.7827 | 1629.7854 | 0.0027 | 2 | 884 | 896 | DAQQRIEDLQEER | | | | | Mascot |
| 1629.7827 | 1629.7854 | 0.0027 | 2 | 884 | 896 | DAQQRIEDLQEER | | | | | Mascot |
| 1810.8528 | 1810.9752 | 0.1224 | 68 | 297 | 312 | NSLTFPDDIEMSKDAK | | | | | Mascot |
| 1951.838 | 1951.9008 | 0.0628 | 32 | 216 | 232 | LADFGTCMKMDSTGMVR | 2 | 0 | Carbamidomethyl (C)[7], Oxidation (M)[10,15] | | Mascot |
| 1951.838 | 1951.9008 | 0.0628 | 32 | 216 | 232 | LADFGTCMKMDSTGMVR | | | Carbamidomethyl (C)[7], Oxidation (M)[8,10] | | Mascot |
| 1967.833 | 1967.8976 | 0.0646 | 33 | 216 | 232 | LADFGTCMKMDSTGMVR | | | Carbamidomethyl (C)[7], Oxidation (M)[8,10,15] | | Mascot |
| 1967.833 | 1967.8976 | 0.0646 | 33 | 216 | 232 | LADFGTCMKMDSTGMVR | | | Carbamidomethyl (C)[7], Oxidation (M)[8,10,15] | | Mascot |
| 2000.9303 | 2001.062 | 0.1317 | 66 | 742 | 757 | LMQLEKENSVLDCDYK | | | Carbamidomethyl (C)[13], Oxidation (M)[2] | | Mascot |

43 Transferrin receptor 1a [Danio rerio] gi|190337022 84943 6.38 11 31 0

**Peptide Information**

| Calc. Mass | Obsrv. Mass | ± da | ± ppm | Start Seq. | End Seq. | Sequence | Ion Score | C. I. % | Modification | Rank | Result Type |
|---|---|---|---|---|---|---|---|---|---|---|---|
| 837.4386 | 837.522 | 0.0834 | 100 | 431 | 437 | TITDMIK | | | Oxidation (M)[5] | | Mascot |
| 842.4842 | 842.5001 | 0.0159 | 19 | 66 | 72 | LNQRSPK | | | | | Mascot |
| 947.5672 | 947.5545 | -0.0127 | -13 | 401 | 408 | YLVIGVQR | | | | | Mascot |
| 1045.5935 | 1045.55 | -0.0435 | -42 | 683 | 690 | IINNRIMR | | | Oxidation (M)[7] | | Mascot |
| 1067.5844 | 1067.5068 | -0.0776 | -73 | 625 | 633 | SYVSQIRSK | | | | | Mascot |
| 1799.9174 | 1799.783 | -0.1344 | -75 | 203 | 219 | NNVVGTTEGYLAYSAIK | | | | | Mascot |
| 1816.9705 | 1816.7664 | -0.2041 | -112 | 385 | 400 | IHNVFGVIKGYLDADR | | | | | Mascot |
| 1951.8596 | 1951.9008 | 0.0412 | 21 | 672 | 687 | NSDLDDMEQCRIINNR | | | Oxidation (M)[7] | | Mascot |
| 1951.8596 | 1951.9008 | 0.0412 | 21 | 672 | 687 | NSDLDDMEQCRIINNR | | | Oxidation (M)[7] | | Mascot |
| 2040.947 | 2041.073 | 0.126 | 62 | 477 | 494 | MFSYISLDDLISGSDSFK | | | Oxidation (M)[1] | | Mascot |
| 2049.1128 | 2049.0649 | -0.0479 | -23 | 691 | 708 | VEGSLLSPYVSPRQTLFR | | | | | Mascot |
| 2283.0339 | 2283.1531 | 0.1192 | 52 | 662 | 682 | AASALTSAIRNSDLDDMEQCR | | | Oxidation (M)[17] | | Mascot |

| 44 | finTRIM family protein [Danio rerio] | gi\|208019415 | 24902.8 | 9.08 | 8 | 31 | 0 |
|---|---|---|---|---|---|---|---|

Peptide Information

| Calc. Mass | Obsrv. Mass | ± da | ± ppm | Start Seq. | End Seq. | Sequence | Ion Score | C. I. % | Modification | Rank | Result Type |
|---|---|---|---|---|---|---|---|---|---|---|---|
| 996.522 | 996.5847 | 0.0627 | 63 | 72 | 80 | HNLIDATGR | | | | | Mascot |
| 1045.5636 | 1045.55 | -0.0136 | -13 | 195 | 203 | DQEKITVGR | | | | | Mascot |
| 1075.6007 | 1075.5247 | -0.076 | -71 | 139 | 146 | FQQQIQKR | | | | | Mascot |
| 1251.6328 | 1251.6042 | -0.0286 | -23 | 163 | 173 | FAQTAVKDSER | | | | | Mascot |
| 1578.8597 | 1578.7739 | -0.0858 | -54 | 150 | 162 | LQELNEAVKIHER | | | | | Mascot |
| 1578.8597 | 1578.7739 | -0.0858 | -54 | 150 | 162 | LQELNEAVKIHER | | | | | Mascot |
| 1800.9888 | 1800.7736 | -0.2152 | -119 | 1 | 16 | ALNKNTMLAEVVEQLK | | | | | Mascot |
| 1816.9836 | 1816.7664 | -0.2172 | -120 | 1 | 16 | ALNKNTMLAEVVEQLK | | | Oxidation (M)[7] | | Mascot |
| 2154.8928 | 2154.936 | 0.0432 | 20 | 20 | 40 | HHTAAVPAGDGDVECDS CTGR | | | Carbamidomethyl (C)[15] | | Mascot |
| 2282.9878 | 2283.1531 | 0.1653 | 72 | 20 | 41 | HHTAAVPAGDGDVECDS CTGRK | | | Carbamidomethyl (C)[15] | | Mascot |

| 45 | novel protein [Danio rerio] | gi\|126632583 | 6089.9 | 8.37 | 5 | 31 | 0 |
|---|---|---|---|---|---|---|---|

Peptide Information

| Calc. Mass | Obsrv. Mass | ± da | ± ppm | Start Seq. | End Seq. | Sequence | Ion Score | C. I. % | Modification | Rank | Result Type |
|---|---|---|---|---|---|---|---|---|---|---|---|
| 1165.5895 | 1165.7307 | 0.1412 | 121 | 10 | 18 | CLRVNYGQR | | | Carbamidomethyl (C)[1] | | Mascot |
| 1578.7035 | 1578.7739 | 0.0704 | 45 | 38 | 51 | VSSCMYLPTCAIMK | | | Oxidation (M)[5,13] | | Mascot |
| 1578.7035 | 1578.7739 | 0.0704 | 45 | 38 | 51 | VSSCMYLPTCAIMK | | | Oxidation (M)[5,13] | | Mascot |
| 1660.7566 | 1660.7837 | 0.0271 | 16 | 38 | 51 | VSSCMYLPTCAIMK | | | Carbamidomethyl (C)[4,10] | | Mascot |
| 1718.8097 | 1718.8932 | 0.0835 | 49 | 37 | 51 | RVSSCMYLPTCAIMK | | | Oxidation (M)[6] | | Mascot |
| 1816.8577 | 1816.7664 | -0.0913 | -50 | 37 | 51 | RVSSCMYLPTCAIMK | | | Carbamidomethyl (C)[5,11] | | Mascot |
| 1973.9567 | 1973.9464 | -0.0103 | -5 | 38 | 54 | VSSCMYLPTCAIMKVTL | | | Carbamidomethyl (C)[4,10] | | Mascot |
| 2225.0073 | 2225.0969 | 0.0896 | 40 | 19 | 37 | CANDCPDVLYNTAEQSL RR | | | Carbamidomethyl (C)[1] | | Mascot |

| 46 | tetratricopeptide repeat protein 38 [Danio rerio] | gi\|170763463 | 52758.4 | 5.84 | 7 | 31 | 0 | 12 | 0 |
|---|---|---|---|---|---|---|---|---|---|

Peptide Information

| Calc. Mass | Obsrv. Mass | ± da | ± ppm | Start Seq. | End Seq. | Sequence | Ion Score | C. I. % | Modification | Rank | Result Type |
|---|---|---|---|---|---|---|---|---|---|---|---|
| 996.5038 | 996.5847 | 0.0809 | 81 | 1 | 8 | MICVVIMR | | | Oxidation (M)[1,7] | | Mascot |
| 1145.6538 | 1145.5791 | -0.0747 | -65 | 457 | 466 | LIQRAHSLHA | | | | | Mascot |
| 1660.7716 | 1660.7837 | 0.0121 | 7 | 432 | 444 | EHQQLARCMLMER | | | Oxidation (M)[9] | | Mascot |
| 1718.892 | 1718.8932 | 0.0012 | 1 | 95 | 109 | TVELADTQQLTSREK | | | | | Mascot |

| Calc. Mass | Obsrv. Mass | ± da | ± ppm | Start Seq. | End Seq. | Sequence | Ion Score | C. I. % | Modification | Rank | Result Type |
|---|---|---|---|---|---|---|---|---|---|---|---|
| 1799.8666 | 1799.783 | -0.0836 | -46 | 285 | 300 | SGAMLDIVDSCSLLYR | | | Carbamidomethyl (C)[11] | | Mascot |
| 1815.8616 | 1815.782 | -0.0796 | -44 | 285 | 300 | SGAMLDIVDSCSLLYR | | | Carbamidomethyl (C)[11], Oxidation (M)[4] | | Mascot |
| 1967.9821 | 1967.8976 | -0.0845 | -43 | 265 | 281 | GNYEAALKIFDEQVSQR | | | | | Mascot |
| 1967.9821 | 1967.8976 | -0.0845 | -43 | 265 | 281 | GNYEAALKIFDEQVSQR | 12 | 0 | | | Mascot |
| 2146.0342 | 2146.1343 | 0.1001 | 47 | 282 | 300 | CVKSGAMLDIVDSCSLLYR | | | Carbamidomethyl (C)[1], Oxidation (M)[7] | | Mascot |

| | | | | | | | | | |
|---|---|---|---|---|---|---|---|---|---|
| 47 | hypothetical protein LOC796228 [Danio rerio] | gi\|160333607 | 34755.4 | 7.57 | 6 | 31 | 0 | 7 | 0 |

**Peptide Information**

| Calc. Mass | Obsrv. Mass | ± da | ± ppm | Start Seq. | End Seq. | Sequence | Ion Score | C. I. % | Modification | Rank | Result Type |
|---|---|---|---|---|---|---|---|---|---|---|---|
| 819.428 | 819.4641 | 0.0361 | 44 | 165 | 171 | VAMEEIK | | | | | Mascot |
| 1145.6525 | 1145.5791 | -0.0734 | -64 | 144 | 154 | AVQSSVLTNVK | | | | | Mascot |
| 1660.7773 | 1660.7837 | 0.0064 | 4 | 177 | 191 | NETSATQHQTIESSK | | | | | Mascot |
| 1800.92 | 1800.7736 | -0.1464 | -81 | 270 | 285 | ASFDLPYTGTVKITCK | | | Carbamidomethyl (C)[15] | | Mascot |
| 1967.9684 | 1967.8976 | -0.0708 | -36 | 95 | 111 | TTKGVEFFAHMTDWGLK | | | | | Mascot |
| 1967.9684 | 1967.8976 | -0.0708 | -36 | 95 | 111 | TTKGVEFFAHMTDWGLK | 7 | 0 | | | Mascot |
| 2284.0854 | 2284.1499 | 0.0645 | 28 | 98 | 116 | GVEFFAHMTDWGLKTEYPR | | | | | Mascot |

| | | | | | | | | | |
|---|---|---|---|---|---|---|---|---|---|
| 48 | signaling molecule lefty1 [Danio rerio] | gi\|4959540 | 40991.5 | 8.65 | 9 | 31 | 0 | | |

**Peptide Information**

| Calc. Mass | Obsrv. Mass | ± da | ± ppm | Start Seq. | End Seq. | Sequence | Ion Score | C. I. % | Modification | Rank | Result Type |
|---|---|---|---|---|---|---|---|---|---|---|---|
| 986.4689 | 986.519 | 0.0501 | 51 | 305 | 312 | NYGYGERK | | | | | Mascot |
| 996.5546 | 996.5847 | 0.0301 | 30 | 58 | 65 | NKYISMLK | | | | | Mascot |
| 1520.7413 | 1520.8969 | 0.1556 | 102 | 20 | 32 | GFTHEDMKDALLK | | | Oxidation (M)[7] | | Mascot |
| 1823.9104 | 1823.894 | -0.0164 | -9 | 313 | 328 | CAVVESAPLPMMYLVK | | | Carbamidomethyl (C)[1], Oxidation (M)[11] | | Mascot |
| 1823.9104 | 1823.894 | -0.0164 | -9 | 313 | 328 | CAVVESAPLPMMYLVK | | | Carbamidomethyl (C)[1], Oxidation (M)[11] | | Mascot |
| 1952.0054 | 1951.9008 | -0.1046 | -54 | 313 | 329 | CAVVESAPLPMMYLVKK | | | Carbamidomethyl (C)[1], Oxidation (M)[11] | | Mascot |
| 1952.0599 | 1951.9008 | -0.1591 | -82 | 165 | 181 | TSLVDSRLIPIHETGWK | | | | | Mascot |
| 1968.0002 | 1967.8976 | -0.1026 | -52 | 313 | 329 | CAVVESAPLPMMYLVKK | | | Carbamidomethyl (C)[1], Oxidation (M)[11,12] | | Mascot |
| 1968.0002 | 1967.8976 | -0.1026 | -52 | 312 | 328 | KCAVVESAPLPMMYLVK | | | Carbamidomethyl (C)[2], Oxidation (M)[12,13] | | Mascot |
| 2283.0999 | 2283.1531 | 0.0532 | 23 | 84 | 104 | GIPGNADISGEFVYSDTTRQR | | | | | Mascot |
| 2484.1655 | 2484.2402 | 0.0747 | 30 | 6 | 27 | AACLLCAALFAMARGFTHEDMK | | | Carbamidomethyl (C)[3,6] | | Mascot |
| 2484.1655 | 2484.2402 | 0.0747 | 30 | 6 | 27 | AACLLCAALFAMARGFTHEDMK | | | Carbamidomethyl (C)[3,6] | | Mascot |

| | | | | | | | | | |
|---|---|---|---|---|---|---|---|---|---|
| 49 | bone morphogenetic protein 3 [Danio rerio] | gi\|118150546 | 51942.4 | 9.4 | 1 | | | 23 | 0 |

Peptide Information

| Calc. Mass | Obsrv. Mass | ± da | ± ppm | Start Seq. | End Seq. | Sequence | Ion Score | C. I. % | Modification | Rank | Result Type |
|---|---|---|---|---|---|---|---|---|---|---|---|
| 1629.882 | 1629.7854 | -0.0966 | -59 | 195 | 208 | HHDQLLIGININSR | 23 | 0 | | 2 | Mascot |

| | | | | | | | | | |
|---|---|---|---|---|---|---|---|---|---|
| 50 | Ccdc80 protein [Danio rerio] | gi\|59862002 | 24401.9 | 5.97 | 1 | | | 21 | 0 |

Peptide Information

| Calc. Mass | Obsrv. Mass | ± da | ± ppm | Start Seq. | End Seq. | Sequence | Ion Score | C. I. % | Modification | Rank | Result Type |
|---|---|---|---|---|---|---|---|---|---|---|---|
| 1329.7485 | 1329.6761 | -0.0724 | -54 | 113 | 124 | EGISATLVRDIR | 21 | 0 | | 2 | Mascot |

| | | | | | |
|---|---|---|---|---|---|
| **Gel Idx/Pos** | 179/H6 | **Instr./Gel Origin** | BA2060/110707A | **Process Status** | Analysis Succeeded |
| **Plate [#] Name** | [1] 31496 | **Instrument Sample Name** | | **Spectra** | 11 |

| Rank | Protein Name | Accession No. | Protein MW | Protein PI | Pep. Count | Protein Score | Protein Score C. I. % | Total Ion Score | Total Ion C. I. % |
|---|---|---|---|---|---|---|---|---|---|
| 1 | gelsolin b [Danio rerio] | gi\|148230943 | 80133.8 | 5.62 | 12 | 257 | 100 | 217 | 100 |

Peptide Information

| Calc. Mass | Obsrv. Mass | ± da | ± ppm | Start Seq. | End Seq. | Sequence | Ion Score | C. I. % | Modification | Rank | Result Type |
|---|---|---|---|---|---|---|---|---|---|---|---|
| 853.3621 | 853.3546 | -0.0075 | -9 | 179 | 184 | CNQFER | | | Carbamidomethyl (C)[1] | | Mascot |
| 875.4661 | 875.4556 | -0.0105 | -12 | 104 | 110 | TFLGYFK | | | | | Mascot |
| 987.537 | 987.524 | -0.013 | -13 | 13 | 20 | QTGLQVWR | 51 | 99.81 | | | Mascot |
| 987.537 | 987.524 | -0.013 | -13 | 13 | 20 | QTGLQVWR | | | | | Mascot |
| 1007.4792 | 1007.4681 | -0.0111 | -11 | 676 | 684 | YIESDPAGR | | | | | Mascot |
| 1022.5053 | 1022.495 | -0.0103 | -10 | 2 | 9 | VHHPEFEK | | | | | Mascot |
| 1100.5735 | 1100.5676 | -0.0059 | -5 | 367 | 376 | VPFDASSLHK | | | | | Mascot |
| 1294.5262 | 1294.5137 | -0.0125 | -10 | 193 | 202 | DIRDNECCGR | | | Carbamidomethyl (C)[7,8] | | Mascot |
| 1456.6777 | 1456.6569 | -0.0208 | -14 | 308 | 320 | AAEDFISQMGYPK | | | | | Mascot |
| 1472.6726 | 1472.6549 | -0.0177 | -12 | 308 | 320 | AAEDFISQMGYPK | | | Oxidation (M)[9] | | Mascot |
| 1625.7476 | 1625.7463 | -0.0013 | -1 | 203 | 216 | AKLFVCEEGSENDK | | | Carbamidomethyl (C)[6] | | Mascot |
| 1952.0123 | 1951.9854 | -0.0269 | -14 | 321 | 337 | HTEVQIIPENGETPLFK | | | | | Mascot |
| 2097.1299 | 2097.1064 | -0.0235 | -11 | 451 | 472 | GASAILAAQLDAELGGSA VQVR | | | | | Mascot |
| 2097.1299 | 2097.1064 | -0.0235 | -11 | 451 | 472 | GASAILAAQLDAELGGSA VQVR | 165 | 100 | | | Mascot |
| 2152.0959 | 2152.0684 | -0.0275 | -13 | 32 | 50 | NLYGGFYTGDTYLVLNTI K | | | | | Mascot |

| | | | | | | | | | |
|---|---|---|---|---|---|---|---|---|---|
| 2 | acylamino-acid-releasing enzyme [Danio rerio] | gi\|38564417 | 81411.5 | 5.72 | 9 | 125 | 100 | 105 | 100 |

**Peptide Information**

| Calc. Mass | Obsrv. Mass | ± da | ± ppm | Start Seq. | End Seq. | Sequence | Ion Score | C. I. % | Modification | Rank | Result Type |
|---|---|---|---|---|---|---|---|---|---|---|---|
| 1100.5887 | 1100.5676 | -0.0211 | -19 | 116 | 123 | QFLEIWHK | | | | | Mascot |
| 1205.6525 | 1205.6239 | -0.0286 | -24 | 433 | 444 | VGFLPGKGSESK | | | | | Mascot |
| 1272.6868 | 1272.7003 | 0.0135 | 11 | 663 | 674 | TPVLLMLGEGDK | | | | | Mascot |
| 1587.8853 | 1587.8649 | -0.0204 | -13 | 5 | 17 | VLRDPVEISQLYR | | | | | Mascot |
| 1587.8853 | 1587.8649 | -0.0204 | -13 | 5 | 17 | VLRDPVEISQLYR | 43 | 98.845 | | | Mascot |
| 1625.7588 | 1625.7463 | -0.0125 | -8 | 74 | 88 | SVVYSGPSGNCTEIR | | | Carbamidomethyl (C)[11] | | Mascot |
| 1815.8911 | 1815.87 | -0.0211 | -12 | 349 | 365 | AGDDGFTGIYSFLLSPR | | | | | Mascot |
| 1865.8453 | 1865.8218 | -0.0235 | -13 | 38 | 52 | YGGQYSNIYTEWTQR | 61 | 99.981 | | | Mascot |
| 1865.8453 | 1865.8218 | -0.0235 | -13 | 38 | 52 | YGGQYSNIYTEWTQR | | | | | Mascot |
| 1961.9901 | 1961.97 | -0.0201 | -10 | 312 | 328 | IVYLESGVFGPHLQCSR | | | Carbamidomethyl (C)[15] | | Mascot |
| 2472.1936 | 2472.1626 | -0.031 | -13 | 395 | 415 | EVTCLTSQSQEGSWNLLNIHR | | | Carbamidomethyl (C)[4] | | Mascot |

3 Acylpeptide hydrolase [Danio rerio] gi|55777111 81351.5 5.66 8 121 100 105 100

**Peptide Information**

| Calc. Mass | Obsrv. Mass | ± da | ± ppm | Start Seq. | End Seq. | Sequence | Ion Score | C. I. % | Modification | Rank | Result Type |
|---|---|---|---|---|---|---|---|---|---|---|---|
| 1100.5887 | 1100.5676 | -0.0211 | -19 | 116 | 123 | QFLEIWHK | | | | | Mascot |
| 1205.6525 | 1205.6239 | -0.0286 | -24 | 433 | 444 | VGFLPGKGSESK | | | | | Mascot |
| 1272.6868 | 1272.7003 | 0.0135 | 11 | 663 | 674 | TPVLLMLGEGDK | | | | | Mascot |
| 1587.8853 | 1587.8649 | -0.0204 | -13 | 5 | 17 | VLRDPVEISQLYR | | | | | Mascot |
| 1587.8853 | 1587.8649 | -0.0204 | -13 | 5 | 17 | VLRDPVEISQLYR | 43 | 98.845 | | | Mascot |
| 1625.7588 | 1625.7463 | -0.0125 | -8 | 74 | 88 | SVVYSGPSGNCTEIR | | | Carbamidomethyl (C)[11] | | Mascot |
| 1865.8453 | 1865.8218 | -0.0235 | -13 | 38 | 52 | YGGQYSNIYTEWTQR | 61 | 99.981 | | | Mascot |
| 1865.8453 | 1865.8218 | -0.0235 | -13 | 38 | 52 | YGGQYSNIYTEWTQR | | | | | Mascot |
| 1961.9901 | 1961.97 | -0.0201 | -10 | 312 | 328 | IVYLESGVFGPHLQCSR | | | Carbamidomethyl (C)[15] | | Mascot |
| 2472.1936 | 2472.1626 | -0.031 | -13 | 395 | 415 | EVTCLTSQSQEGSWNLLNIHR | | | Carbamidomethyl (C)[4] | | Mascot |

4 coiled-coil-helix-coiled-coil-helix domain-containing protein 6 [Danio rerio] gi|53933232 27523 9.24 10 54 83.843

**Peptide Information**

| Calc. Mass | Obsrv. Mass | ± da | ± ppm | Start Seq. | End Seq. | Sequence | Ion Score | C. I. % | Modification | Rank | Result Type |
|---|---|---|---|---|---|---|---|---|---|---|---|
| 850.4165 | 850.5328 | 0.1163 | 137 | 164 | 169 | NEERFR | | | | | Mascot |
| 850.4165 | 850.5328 | 0.1163 | 137 | 164 | 169 | NEERFR | | | | | Mascot |

| | | | | | | | | | | | |
|---|---|---|---|---|---|---|---|---|---|---|---|
| 1045.5385 | 1045.5532 | 0.0147 | 14 | 108 | 116 | EAARQDISR | | | | | Mascot |
| 1235.5936 | 1235.6188 | 0.0252 | 20 | 211 | 221 | EQTLQCSDLAK | | | | | Mascot |
| 1294.6526 | 1294.5137 | -0.1389 | -107 | 75 | 85 | TTFPDSKEELK | | | | | Mascot |
| 1371.6395 | 1371.6206 | -0.0189 | -14 | 222 | 232 | EYMQCINAAKK | | | Carbamidomethyl (C)[5], Oxidation (M)[3] | | Mascot |
| 1638.7805 | 1638.8395 | 0.059 | 36 | 168 | 181 | FRMSAEQFHAAATR | | | Oxidation (M)[3] | | Mascot |
| 1874.9719 | 1874.9208 | -0.0511 | -27 | 87 | 101 | RYEQQQAIIQEELAR | | | | | Mascot |
| 1961.9498 | 1961.97 | 0.0202 | 10 | 170 | 187 | MSAEQFHAAATRSEANIK | | | | | Mascot |
| 2317.1904 | 2317.1436 | -0.0468 | -20 | 188 | 207 | ARNVEPVCLNLQAQILNCYR | | | | | Mascot |
| 2501.147 | 2501.2251 | 0.0781 | 31 | 211 | 231 | EQTLQCSDLAKEYMQCINAAK | | | Carbamidomethyl (C)[6,16] | | Mascot |

5 hypothetical protein LOC553448 [Danio rerio] gi|224496090 145429.2 8.16 17 47 7.024

**Peptide Information**

| Calc. Mass | Obsrv. Mass | ± da | ± ppm | Start Seq. | End Seq. | Sequence | Ion Score | C. I. % | Modification | Rank | Result Type |
|---|---|---|---|---|---|---|---|---|---|---|---|
| 850.4781 | 850.5328 | 0.0547 | 64 | 161 | 169 | AAPPAPAQK | | | | | Mascot |
| 850.4781 | 850.5328 | 0.0547 | 64 | 369 | 377 | AAPPAPAQK | | | | | Mascot |
| 856.4523 | 856.5167 | 0.0644 | 75 | 170 | 178 | SAPPASAQK | | | | | Mascot |
| 1007.5077 | 1007.4681 | -0.0396 | -39 | 781 | 789 | VASVEVEMK | | | Oxidation (M)[8] | | Mascot |
| 1100.6058 | 1100.5676 | -0.0382 | -35 | 657 | 665 | LQQENSILR | | | | | Mascot |
| 1156.6321 | 1156.597 | -0.0351 | -30 | 611 | 620 | EQDIIALQAR | | | | | Mascot |
| 1163.6089 | 1163.6011 | -0.0078 | -7 | 1285 | 1295 | MTKDLGQAATK | | | | | Mascot |
| 1179.6038 | 1179.5908 | -0.013 | -11 | 1285 | 1295 | MTKDLGQAATK | | | Oxidation (M)[1] | | Mascot |
| 1199.5902 | 1199.5568 | -0.0334 | -28 | 473 | 483 | AEESKQDAPPK | | | | | Mascot |
| 1235.6782 | 1235.6188 | -0.0594 | -48 | 1014 | 1023 | TKWLQDFAVK | | | | | Mascot |
| 1272.6179 | 1272.7003 | 0.0824 | 65 | 701 | 711 | NDALQADEQLR | | | | | Mascot |
| 1312.7332 | 1312.6016 | -0.1316 | -100 | 610 | 620 | REQDIIALQAR | | | | | Mascot |
| 1472.7703 | 1472.6549 | -0.1154 | -78 | 1110 | 1121 | VQELEETAERLR | | | | | Mascot |
| 1587.849 | 1587.8649 | 0.0159 | 10 | 537 | 550 | AGIIQDTWQKATQK | | | | | Mascot |
| 1587.849 | 1587.8649 | 0.0159 | 10 | 537 | 550 | AGIIQDTWQKATQK | | | | | Mascot |
| 1625.8606 | 1625.7463 | -0.1143 | -70 | 790 | 803 | ERTGHLESLQSQLK | | | | | Mascot |
| 2194.0366 | 2194.0774 | 0.0408 | 19 | 1262 | 1281 | SAEDTAAMGDVVQLKECLEK | | | Carbamidomethyl (C)[17] | | Mascot |
| 2225.1521 | 2225.0945 | -0.0576 | -26 | 758 | 778 | NNLQAQLEQAQKDAAALAEAK | | | | | Mascot |
| 2225.1521 | 2225.0945 | -0.0576 | -26 | 758 | 778 | NNLQAQLEQAQKDAAALAEAK | | | | | Mascot |
| 2233.1743 | 2233.063 | -0.1113 | -50 | 954 | 972 | ISEKESLMTTLQQEVQQLK | | | | | Mascot |
| 2317.1379 | 2317.1436 | 0.0057 | 2 | 1164 | 1184 | QALADTESQLNSAQKEAQTQR | | | | | Mascot |

| | | | | | | | |
|---|---|---|---|---|---|---|---|
| 6 | PREDICTED: pericentrin [Danio rerio] | gi\|326668478 | 491858 | 4.98 | 35 | 46 | 0 |

**Peptide Information**

| Calc. Mass | Obsrv. Mass | ± da | ± ppm | Start Seq. | End Seq. | Sequence | Ion Score | C. I. % | Modification | Rank | Result Type |
|---|---|---|---|---|---|---|---|---|---|---|---|
| 836.3818 | 836.3273 | -0.0545 | -65 | 3557 | 3563 | CSLEAEK | | | Carbamidomethyl (C)[1] | | Mascot |
| 842.4995 | 842.5021 | 0.0026 | 3 | 15 | 21 | AKLAHFR | | | | | Mascot |
| 850.4087 | 850.5328 | 0.1241 | 146 | 2129 | 2136 | TPSSMTAR | | | | | Mascot |
| 850.4087 | 850.5328 | 0.1241 | 146 | 2129 | 2136 | TPSSMTAR | | | | | Mascot |
| 853.3897 | 853.3546 | -0.0351 | -41 | 27 | 34 | SESSSTQK | | | | | Mascot |
| 864.3581 | 864.4857 | 0.1276 | 148 | 1982 | 1988 | EESDQEK | | | | | Mascot |
| 875.458 | 875.4556 | -0.0024 | -3 | 2850 | 2857 | SLGEERGK | | | | | Mascot |
| 882.5155 | 882.5558 | 0.0403 | 46 | 3606 | 3612 | SEIHKLR | | | | | Mascot |
| 962.4424 | 962.5256 | 0.0832 | 86 | 1946 | 1953 | ELEEEASR | | | | | Mascot |
| 987.5105 | 987.524 | 0.0135 | 14 | 2715 | 2722 | QAEIEELR | 7 | 0 | | | Mascot |
| 987.5105 | 987.524 | 0.0135 | 14 | 2715 | 2722 | QAEIEELR | | | | | Mascot |
| 1007.4904 | 1007.4681 | -0.0223 | -22 | 427 | 434 | HLQEEHSK | | | | | Mascot |
| 1022.4935 | 1022.495 | 0.0015 | 1 | 3557 | 3565 | CSLEAEKSR | | | | | Mascot |
| 1045.4908 | 1045.5532 | 0.0624 | 60 | 1472 | 1480 | QAEAEVNER | | | | | Mascot |
| 1100.5695 | 1100.5676 | -0.0019 | -2 | 1216 | 1224 | VALQEQQER | | | | | Mascot |
| 1156.5454 | 1156.597 | 0.0516 | 45 | 3752 | 3761 | RQESASAHDR | | | | | Mascot |
| 1163.566 | 1163.6011 | 0.0351 | 30 | 3404 | 3412 | ELLCRMADR | | | Carbamidomethyl (C)[4] | | Mascot |
| 1179.5674 | 1179.5908 | 0.0234 | 20 | 1115 | 1124 | TSVEQLQSCK | | | Carbamidomethyl (C)[9] | | Mascot |
| 1199.6993 | 1199.5568 | -0.1425 | -119 | 2856 | 2866 | GKVAVLEQEVK | | | | | Mascot |
| 1235.7358 | 1235.6188 | -0.117 | -95 | 3888 | 3897 | LLELELLHQK | | | | | Mascot |
| 1272.7046 | 1272.7003 | -0.0043 | -3 | 1342 | 1353 | LVEGALEDSVLK | | | | | Mascot |
| 1294.646 | 1294.5137 | -0.1323 | -102 | 4205 | 4216 | MYTSPVLGAAER | | | | | Mascot |
| 1371.7842 | 1371.6206 | -0.1636 | -119 | 1989 | 2000 | AVALEELEKTLR | | | | | Mascot |
| 1456.6294 | 1456.6569 | 0.0275 | 19 | 3995 | 4007 | SSAVTEECMVSEK | | | Carbamidomethyl (C)[8] | | Mascot |
| 1472.6243 | 1472.6549 | 0.0306 | 21 | 3995 | 4007 | SSAVTEECMVSEK | | | Carbamidomethyl (C)[8], Oxidation (M)[9] | | Mascot |
| 1475.7271 | 1475.7354 | 0.0083 | 6 | 2723 | 2734 | SLVERLQCDQER | | | | | Mascot |
| 1587.7649 | 1587.8649 | 0.1 | 63 | 390 | 402 | TKYEADISNYDLR | | | | | Mascot |
| 1587.8047 | 1587.8649 | 0.0602 | 38 | 1603 | 1616 | SLECQESPQVVALK | | | Carbamidomethyl (C)[4] | | Mascot |
| 1625.901 | 1625.7463 | -0.1547 | -95 | 1954 | 1966 | QRLHFLEEAELLK | | | | | Mascot |
| 1638.881 | 1638.8395 | -0.0415 | -25 | 2528 | 2541 | TVTQLEQELQKGHK | | | | | Mascot |
| 1815.9633 | 1815.87 | -0.0933 | -51 | 358 | 372 | VELNQEMVRLDNLVK | | | Oxidation (M)[7] | | Mascot |
| 1993.9937 | 1993.9501 | -0.0436 | -22 | 3014 | 3031 | ELHEQQEAAVAKQGEAQ | | | | | Mascot |

| Calc. Mass | Obsrv. Mass | ± da | ± ppm | Start Seq. | End Seq. | Sequence | Ion Score | C. I. % | Modification | Rank | Result Type |
|---|---|---|---|---|---|---|---|---|---|---|---|
| | | | | | | K | | | | | |
| 2082.9834 | 2082.9888 | 0.0054 | 3 | 3995 | 4012 | SSAVTEECMVSEKLAWQK | | | Carbamidomethyl (C)[8] | | Mascot |
| 2247.1404 | 2247.0789 | -0.0615 | -27 | 3304 | 3321 | LELENTERLDAHFVEYLR | | | | | Mascot |
| 2284.1013 | 2284.1504 | 0.0491 | 21 | 3955 | 3974 | ENSEFSECLAALSEEKISLK | | | Carbamidomethyl (C)[8] | | Mascot |
| 2300.0935 | 2300.1565 | 0.063 | 27 | 3545 | 3563 | LQQEQTQSHDLRCSLEAEK | | | Carbamidomethyl (C)[13] | | Mascot |
| 2317.1956 | 2317.1436 | -0.052 | -22 | 849 | 868 | LQALEDQVKSMENVLETELK | | | | | Mascot |
| 2501.2075 | 2501.2251 | 0.0176 | 7 | 2956 | 2977 | LEELEEHVQADMVNLSALETSK | | | Oxidation (M)[12] | | Mascot |

| | | | | | | | | |
|---|---|---|---|---|---|---|---|---|
| 7 | transmembrane protein 200A [Danio rerio] | gi\|113679869 | 54934.7 | 9.28 | 11 | 46 | 0 | |

**Peptide Information**

| Calc. Mass | Obsrv. Mass | ± da | ± ppm | Start Seq. | End Seq. | Sequence | Ion Score | C. I. % | Modification | Rank | Result Type |
|---|---|---|---|---|---|---|---|---|---|---|---|
| 970.5316 | 970.4955 | -0.0361 | -37 | 413 | 420 | LTVQPEQR | | | | | Mascot |
| 1163.6068 | 1163.6011 | -0.0057 | -5 | 422 | 430 | HPSWPRLDR | | | | | Mascot |
| 1371.5983 | 1371.6206 | 0.0223 | 16 | 353 | 366 | SNSATESASSTSSR | | | | | Mascot |
| 1456.7253 | 1456.6569 | -0.0684 | -47 | 245 | 257 | GGFCLLSPTYKDR | | | | | Mascot |
| 1587.925 | 1587.8649 | -0.0601 | -38 | 1 | 16 | MIATGGVITGLAALKR | | | Oxidation (M)[1] | | Mascot |
| 1587.925 | 1587.8649 | -0.0601 | -38 | 1 | 16 | MIATGGVITGLAALKR | | | Oxidation (M)[1] | | Mascot |
| 1874.7466 | 1874.9208 | 0.1742 | 93 | 181 | 197 | CTNGACMGPYGGDTEIR | | | Carbamidomethyl (C)[1,6], Oxidation (M)[7] | | Mascot |
| 2096.8938 | 2097.1064 | 0.2126 | 101 | 226 | 244 | FSHRTSSADDDDGLMSEAR | | | | | Mascot |
| 2096.8938 | 2097.1064 | 0.2126 | 101 | 226 | 244 | FSHRTSSADDDDGLMSEAR | | | | | Mascot |
| 2194.1899 | 2194.0774 | -0.1125 | -51 | 280 | 299 | CQTRSIVSSSISAFTLPVIK | | | Carbamidomethyl (C)[1] | | Mascot |
| 2259.9426 | 2260.127 | 0.1844 | 82 | 178 | 197 | EQKCTNGACMGPYGGDTEIR | | | Carbamidomethyl (C)[4,9], Oxidation (M)[10] | | Mascot |
| 2284.0952 | 2284.1504 | 0.0552 | 24 | 17 | 37 | QDSTRSQYHLSAQSPGPAPEK | | | | | Mascot |
| 3337.8242 | 3337.7273 | -0.0969 | -29 | 57 | 87 | LYSASGFFLVLGVLILMAGIAMAVLGYWPHK | | | | | Mascot |

| | | | | | | | | |
|---|---|---|---|---|---|---|---|---|
| 8 | PREDICTED: hypothetical protein LOC100537182 [Danio rerio] | gi\|326677217 | 4808.4 | 4.86 | 5 | 44 | 0 | |

**Peptide Information**

| Calc. Mass | Obsrv. Mass | ± da | ± ppm | Start Seq. | End Seq. | Sequence | Ion Score | C. I. % | Modification | Rank | Result Type |
|---|---|---|---|---|---|---|---|---|---|---|---|
| 853.3906 | 853.3546 | -0.036 | -42 | 11 | 17 | VCKNMDK | | | Oxidation (M)[5] | | Mascot |
| 856.5614 | 856.5167 | -0.0447 | -52 | 2 | 9 | AAVLITLR | | | | | Mascot |
| 987.6019 | 987.524 | -0.0779 | -79 | 1 | 9 | MAAVLITLR | | | | | Mascot |
| 987.6019 | 987.524 | -0.0779 | -79 | 1 | 9 | MAAVLITLR | | | | | Mascot |
| 1100.4677 | 1100.5676 | 0.0999 | 91 | 14 | 21 | NMDKYEER | | | Oxidation (M)[2] | | Mascot |

| | | | | | | | | | | | |
|---|---|---|---|---|---|---|---|---|---|---|---|
| 2317.0942 | 2317.1436 | 0.0494 | 21 | 22 | 43 | IWEELTGQAGDNELEGK VQGSG | | | | | Mascot |

| | | | | | | | |
|---|---|---|---|---|---|---|---|
| 9 | novel protein similar to H.sapiens TTN, titin (TTN) [Danio rerio] | gi\|169154823 | 842970 | 5.81 | 44 | 42 | 0 |

**Peptide Information**

| Calc. Mass | Obsrv. Mass | ± da | ± ppm | Start Seq. | End Seq. | Sequence | Ion Score | C. I. % | Modification | Rank | Result Type |
|---|---|---|---|---|---|---|---|---|---|---|---|
| 842.444 | 842.5021 | 0.0581 | 69 | 3102 | 3108 | SYKVSMK | | | | | Mascot |
| 850.4814 | 850.5328 | 0.0514 | 60 | 1088 | 1094 | LTSVRMK | | | Oxidation (M)[6] | | Mascot |
| 850.4814 | 850.5328 | 0.0514 | 60 | 1088 | 1094 | LTSVRMK | | | Oxidation (M)[6] | | Mascot |
| 853.3839 | 853.3546 | -0.0293 | -34 | 1858 | 1863 | WYQNDK | | | | | Mascot |
| 856.4886 | 856.5167 | 0.0281 | 33 | 6818 | 6824 | LTLQEPR | | | | | Mascot |
| 870.4203 | 870.532 | 0.1117 | 128 | 6420 | 6427 | LSDSGTYK | | | | | Mascot |
| 882.4679 | 882.5558 | 0.0879 | 100 | 2483 | 2489 | YKVSTER | | | | | Mascot |
| 970.5792 | 970.4955 | -0.0837 | -86 | 3602 | 3609 | QQRALVQK | | | | | Mascot |
| 987.5581 | 987.524 | -0.0341 | -35 | 4726 | 4734 | VEVSNRVGK | | | | | Mascot |
| 987.5581 | 987.524 | -0.0341 | -35 | 4726 | 4734 | VEVSNRVGK | | | | | Mascot |
| 996.536 | 996.584 | 0.048 | 48 | 2586 | 2593 | LTITETYR | | | | | Mascot |
| 996.536 | 996.584 | 0.048 | 48 | 2586 | 2593 | LTITETYR | | | | | Mascot |
| 1007.5043 | 1007.4681 | -0.0362 | -36 | 3593 | 3601 | ESPFVSDVK | | | | | Mascot |
| 1045.5347 | 1045.5532 | 0.0185 | 18 | 6656 | 6665 | CQIGGTPEIK | | | | | Mascot |
| 1051.5571 | 1051.7001 | 0.143 | 136 | 6666 | 6674 | ISWFKADGK | | | | | Mascot |
| 1051.5571 | 1051.7001 | 0.143 | 136 | 6666 | 6674 | ISWFKADGK | | | | | Mascot |
| 1100.5582 | 1100.5676 | 0.0094 | 9 | 5147 | 5156 | EPANITEQAK | | | | | Mascot |
| 1156.4939 | 1156.597 | 0.1031 | 89 | 7094 | 7104 | ATNEAGSEFCK | | | | | Mascot |
| 1163.5435 | 1163.6011 | 0.0576 | 50 | 5993 | 6002 | MGEPLQLECK | | | Oxidation (M)[1] | | Mascot |
| 1199.5613 | 1199.5568 | -0.0045 | -4 | 1404 | 1413 | DESYEGMLKK | | | | | Mascot |
| 1205.646 | 1205.6239 | -0.0221 | -18 | 4383 | 4392 | ILWKQGMSSR | | | | | Mascot |
| 1235.6161 | 1235.6188 | 0.0027 | 2 | 790 | 800 | RVQSTSPMSAR | | | Oxidation (M)[8] | | Mascot |
| 1272.5962 | 1272.7003 | 0.1041 | 82 | 4882 | 4892 | EMLTGGTCFIK | | | Carbamidomethyl (C)[8], Oxidation (M)[2] | | Mascot |
| 1279.6376 | 1279.7589 | 0.1213 | 95 | 3711 | 3722 | SETTTAVVSEQK | | | | | Mascot |
| 1312.6348 | 1312.6016 | -0.0332 | -25 | 4387 | 4398 | QGMSSRLSCTVK | | | Oxidation (M)[3] | | Mascot |
| 1371.6208 | 1371.6206 | -0.0002 | 0 | 7094 | 7106 | ATNEAGSEFCKSK | | | | | Mascot |
| 1412.7605 | 1412.6305 | -0.13 | -92 | 273 | 285 | VSTARQQSPSPVR | | | | | Mascot |
| 1456.7617 | 1456.6569 | -0.1048 | -72 | 7158 | 7170 | VGGDPIPNVKWMK | | | Oxidation (M)[12] | | Mascot |
| 1472.6145 | 1472.6549 | 0.0404 | 27 | 5689 | 5701 | YTCQAMNEAGQEK | | | | | Mascot |
| 1475.804 | 1475.7354 | -0.0686 | -46 | 1964 | 1976 | GKHYSLTIINAMK | | | | | Mascot |

| Calc. Mass | Obsrv. Mass | ± da | ± ppm | Start Seq. | End Seq. | Sequence | Ion Score | C. I. % | Modification | Rank | Result Type |
|---|---|---|---|---|---|---|---|---|---|---|---|
| 1587.7393 | 1587.8649 | 0.1256 | 79 | 4879 | 4892 | EDKEMLTGGTCFIK | | | Oxidation (M)[5] | | Mascot |
| 1587.7649 | 1587.8649 | 0.1 | 63 | 5655 | 5667 | DESWILEDPSIQR | | | | | Mascot |
| 1625.7258 | 1625.7463 | 0.0205 | 13 | 5508 | 5522 | NEAGSESCSMDLRVK | | | | | Mascot |
| 1638.9061 | 1638.8395 | -0.0666 | -41 | 7253 | 7266 | DIDIVELLRNVDPK | | | | | Mascot |
| 1815.8191 | 1815.87 | 0.0509 | 28 | 5122 | 5138 | HGGKYTCHAQNEAGSQ K | | | | | Mascot |
| 1993.9358 | 1993.9501 | 0.0143 | 7 | 4299 | 4315 | LECQVDEDTGVSIIWMR | | | | | Mascot |
| 2097.0547 | 2097.1064 | 0.0517 | 25 | 6191 | 6208 | GSQPMTVSWVQDKDHIL R | | | | | Mascot |
| 2097.0547 | 2097.1064 | 0.0517 | 25 | 6191 | 6208 | GSQPMTVSWVQDKDHIL R | | | | | Mascot |
| 2194.1101 | 2194.0774 | -0.0327 | -15 | 742 | 761 | LPVVLPEDEGIYTAFASN MK | | | | | Mascot |
| 2211.1438 | 2211.0801 | -0.0637 | -29 | 6494 | 6513 | SCEIALRGDTAILELHNID K | | | | | Mascot |
| 2232.9575 | 2233.063 | 0.1055 | 47 | 1132 | 1149 | MINEFGYCSLDYEVAYSR | | | Carbamidomethyl (C)[8], Oxidation (M)[1] | | Mascot |
| 2260.1277 | 2260.127 | -0.0007 | 0 | 5668 | 5688 | TFENNVATLTIPVCESIHS GK | | | | | Mascot |
| 2283.104 | 2283.1536 | 0.0496 | 22 | 2409 | 2430 | NVHFGDVGDYKFVAGSA ETAAK | | | | | Mascot |
| 2283.1899 | 2283.1536 | -0.0363 | -16 | 3240 | 3260 | EHPSQVMSALTESVTPLT IVK | | | Oxidation (M)[7] | | Mascot |
| 2297.1516 | 2297.1614 | 0.0098 | 4 | 6550 | 6570 | NLSVEMGKSLILECTYAG SPK | | | Carbamidomethyl (C)[14] | | Mascot |
| 2317.1492 | 2317.1436 | -0.0056 | -2 | 5668 | 5688 | TFENNVATLTIPVCESIHS GK | | | Carbamidomethyl (C)[14] | | Mascot |
| 2472.1033 | 2472.1626 | 0.0593 | 24 | 4481 | 4502 | GCTAHLECEMTGTAPFEI TWFK | | | | | Mascot |
| 2486.2078 | 2486.1956 | -0.0122 | -5 | 2501 | 2525 | MSDAGEYSVVAGSSVSK ANLTVEGK | | | | | Mascot |
| 2501.1753 | 2501.2251 | 0.0498 | 20 | 463 | 486 | DETMSEISSVSVDTGAPA APFFVK | | | Oxidation (M)[4] | | Mascot |
| 3337.4834 | 3337.7273 | 0.2439 | 73 | 4346 | 4377 | DTGTYTCTAANEAGSSS CSSSVTVQEPPVFVK | | | Carbamidomethyl (C)[7,18] | | Mascot |

| | | | | | | | |
|---|---|---|---|---|---|---|---|
| 10 | PREDICTED: transposon TX1 uncharacterized 149 kDa protein-like [Danio rerio] | gi\|326678664 | 127486.3 | 9.2 | 18 | 41 | 0 |

**Peptide Information**

| Calc. Mass | Obsrv. Mass | ± da | ± ppm | Start Seq. | End Seq. | Sequence | Ion Score | C. I. % | Modification | Rank | Result Type |
|---|---|---|---|---|---|---|---|---|---|---|---|
| 850.5145 | 850.5328 | 0.0183 | 22 | 117 | 123 | GRILYTK | | | | | Mascot |
| 850.5145 | 850.5328 | 0.0183 | 22 | 117 | 123 | GRILYTK | | | | | Mascot |
| 853.46 | 853.3546 | -0.1054 | -123 | 388 | 394 | AKFCSIK | | | Carbamidomethyl (C)[4] | | Mascot |
| 875.4668 | 875.4556 | -0.0112 | -13 | 1 | 6 | MYRLHR | | | | | Mascot |
| 996.6564 | 996.584 | -0.0724 | -73 | 535 | 543 | RAVLSLLPK | | | | | Mascot |
| 996.6564 | 996.584 | -0.0724 | -73 | 535 | 543 | RAVLSLLPK | | | | | Mascot |
| 1022.5629 | 1022.495 | -0.0679 | -66 | 1050 | 1058 | KANIFQSSK | | | | | Mascot |
| 1045.5538 | 1045.5532 | -0.0006 | -1 | 940 | 947 | RTADLQWR | | | | | Mascot |

| Calc. Mass | Obsrv. Mass | ± da | ± ppm | Start Seq. | End Seq. | Sequence | Ion Score | C. I. % | Modification | Rank | Result Type |
|---|---|---|---|---|---|---|---|---|---|---|---|
| 1100.6211 | 1100.5676 | -0.0535 | -49 | 200 | 208 | KNGLVDIWR | | | | | Mascot |
| 1235.6453 | 1235.6188 | -0.0265 | -21 | 283 | 292 | LLQDVLFCER | | | | | Mascot |
| 1312.6896 | 1312.6016 | -0.088 | -67 | 370 | 380 | KAELGSFLYER | | | | | Mascot |
| 1638.889 | 1638.8395 | -0.0495 | -30 | 914 | 926 | ELLWTPTTYFLVR | | | | | Mascot |
| 1815.6643 | 1815.87 | 0.2057 | 113 | 1062 | 1078 | TEVDDGCSDDGMESGN K | | | Carbamidomethyl (C)[7] | | Mascot |
| 1912.9507 | 1912.9598 | 0.0091 | 5 | 847 | 862 | CIVLDPPEDIICEIQR | | | Carbamidomethyl (C)[1] | | Mascot |
| 2097.0281 | 2097.1064 | 0.0783 | 37 | 476 | 493 | DITFKELTEAMQQLSNGR | | | Oxidation (M)[11] | | Mascot |
| 2097.0281 | 2097.1064 | 0.0783 | 37 | 476 | 493 | DITFKELTEAMQQLSNGR | | | Oxidation (M)[11] | | Mascot |
| 2260.208 | 2260.127 | -0.081 | -36 | 681 | 700 | QGCPLSGQLYSLMIEPLL LK | | | Carbamidomethyl (C)[3] | | Mascot |
| 2283.1914 | 2283.1536 | -0.0378 | -17 | 319 | 337 | AQIRVFCQNYTLNSSLQI K | | | Carbamidomethyl (C)[7] | | Mascot |
| 2283.1914 | 2283.1536 | -0.0378 | -17 | 319 | 337 | AQIRVFCQNYTLNSSLQI K | | | Carbamidomethyl (C)[7] | | Mascot |
| 2299.041 | 2299.1592 | 0.1182 | 51 | 390 | 408 | FCSIKDMDAPSSYFFNLE K | | | Carbamidomethyl (C)[2] | | Mascot |
| 2472.3716 | 2472.1626 | -0.209 | -85 | 681 | 702 | QGCPLSGQLYSLMIEPLL LKLR | | | | | Mascot |
| 2486.2349 | 2486.1956 | -0.0393 | -16 | 124 | 145 | IEIEGFLFGFINVYAPNNG SER | | | | | Mascot |

11 homeobox protein Hox-C1a [Danio rerio] gi|18858843 34195.5 8.59 8 41 0

Peptide Information

| Calc. Mass | Obsrv. Mass | ± da | ± ppm | Start Seq. | End Seq. | Sequence | Ion Score | C. I. % | Modification | Rank | Result Type |
|---|---|---|---|---|---|---|---|---|---|---|---|
| 850.4087 | 850.5328 | 0.1241 | 146 | 196 | 202 | CSDRELK | | | | | Mascot |
| 850.4087 | 850.5328 | 0.1241 | 146 | 196 | 202 | CSDRELK | | | | | Mascot |
| 1007.4727 | 1007.4681 | -0.0046 | -5 | 17 | 25 | GYHATGVMR | | | Oxidation (M)[8] | | Mascot |
| 1100.5007 | 1100.5676 | 0.0669 | 61 | 113 | 122 | GFSPSSDTFR | | | | | Mascot |
| 1865.8619 | 1865.8218 | -0.0401 | -21 | 276 | 293 | EGLAQGLMLISGCDEDS K | | | | | Mascot |
| 1865.8619 | 1865.8218 | -0.0401 | -21 | 276 | 293 | EGLAQGLMLISGCDEDS K | | | | | Mascot |
| 1912.9585 | 1912.9598 | 0.0013 | 1 | 9 | 25 | DTTSLFIKGYHATGVMR | | | Oxidation (M)[16] | | Mascot |
| 1993.9569 | 1993.9501 | -0.0068 | -3 | 276 | 294 | EGLAQGLMLISGCDEDS KK | | | | | Mascot |
| 2247.0459 | 2247.0789 | 0.033 | 15 | 123 | 142 | TFSSAHCELGPVNNTQT EVR | | | Carbamidomethyl (C)[7] | | Mascot |
| 2298.0774 | 2298.1658 | 0.0884 | 38 | 273 | 293 | MLREGLAQGLMLISGCD EDSK | | | Oxidation (M)[1,11] | | Mascot |

12 novel protein similar to vertebrate NADH dehydrogenase (ubiquinone) 1 alpha subcomplex, assembly fa gi|220678361 11073.7 9.86 6 40 0

Peptide Information

| Calc. Mass | Obsrv. Mass | ± da | ± ppm | Start Seq. | End Seq. | Sequence | Ion Score | C. I. % | Modification | Rank | Result Type |
|---|---|---|---|---|---|---|---|---|---|---|---|

| Calc. Mass | Obsrv. Mass | ± da | ± ppm | Start Seq. | End Seq. | Sequence | Ion Score | C. I. % | Modification | Rank | Result Type |
|---|---|---|---|---|---|---|---|---|---|---|---|
| 842.5246 | 842.5021 | -0.0225 | -27 | 74 | 80 | KHFGLLK | | | | | Mascot |
| 875.5308 | 875.4556 | -0.0752 | -86 | 2 | 9 | AVSRTTLK | | | | | Mascot |
| 996.5512 | 996.584 | 0.0328 | 33 | 87 | 95 | WIGPEGKPI | | | | | Mascot |
| 996.5512 | 996.584 | 0.0328 | 33 | 87 | 95 | WIGPEGKPI | | | | | Mascot |
| 1022.5662 | 1022.495 | -0.0712 | -70 | 1 | 9 | MAVSRTTLK | | | Oxidation (M)[1] | | Mascot |
| 1163.6201 | 1163.6011 | -0.019 | -16 | 31 | 40 | CASIQITRSK | | | Carbamidomethyl (C)[1] | | Mascot |
| 1272.6405 | 1272.7003 | 0.0598 | 47 | 10 | 20 | SLYGVCGVVYR | | | Carbamidomethyl (C)[6] | | Mascot |

13 si:ch1073-126c3.2 [Danio rerio] gi|221307488 24754.2 6.97 7 40 0

Peptide Information

| Calc. Mass | Obsrv. Mass | ± da | ± ppm | Start Seq. | End Seq. | Sequence | Ion Score | C. I. % | Modification | Rank | Result Type |
|---|---|---|---|---|---|---|---|---|---|---|---|
| 864.4243 | 864.4857 | 0.0614 | 71 | 1 | 8 | MESATIGR | | | | | Mascot |
| 1371.6572 | 1371.6206 | -0.0366 | -27 | 132 | 143 | SRLFEECSTATK | | | | | Mascot |
| 1638.8069 | 1638.8395 | 0.0326 | 20 | 199 | 213 | SELAKVETTESESTK | | | | | Mascot |
| 1951.9277 | 1951.9854 | 0.0577 | 30 | 204 | 221 | VETTESESTKLLCGNINN | | | | | Mascot |
| 2211.0295 | 2211.0801 | 0.0506 | 23 | 114 | 131 | IYCKPMCNAGYDFNFLRR | | | | | Mascot |
| 2284.0459 | 2284.1504 | 0.1045 | 46 | 114 | 131 | IYCKPMCNAGYDFNFLRR | | | Carbamidomethyl (C)[3], Oxidation (M)[6] | | Mascot |
| 2298.0503 | 2298.1658 | 0.1155 | 50 | 112 | 130 | DKIYCKPMCNAGYDFNFLR | | | | | Mascot |
| 2472.1611 | 2472.1626 | 0.0015 | 1 | 162 | 184 | SHIAVSGAPSAYFPQGEDCHITK | | | Carbamidomethyl (C)[19] | | Mascot |

14 ribosome production factor 2 homolog [Danio rerio] gi|47550781 35438.5 10 9 40 0

Peptide Information

| Calc. Mass | Obsrv. Mass | ± da | ± ppm | Start Seq. | End Seq. | Sequence | Ion Score | C. I. % | Modification | Rank | Result Type |
|---|---|---|---|---|---|---|---|---|---|---|---|
| 850.5396 | 850.5328 | -0.0068 | -8 | 197 | 203 | SYKVLLK | | | | | Mascot |
| 850.5396 | 850.5328 | -0.0068 | -8 | 197 | 203 | SYKVLLK | | | | | Mascot |
| 856.4523 | 856.5167 | 0.0644 | 75 | 2 | 9 | SHTDGIVK | | | | | Mascot |
| 987.4927 | 987.524 | 0.0313 | 32 | 1 | 9 | MSHTDGIVK | | | | | Mascot |
| 987.4927 | 987.524 | 0.0313 | 32 | 1 | 9 | MSHTDGIVK | | | | | Mascot |
| 1163.5327 | 1163.6011 | 0.0684 | 59 | 228 | 237 | NHTASDDLYK | | | | | Mascot |
| 1952.0117 | 1951.9854 | -0.0263 | -13 | 32 | 50 | NSMIIKGGNTSSTVSQALK | | | Oxidation (M)[3] | | Mascot |
| 2247.2607 | 2247.0789 | -0.1818 | -81 | 172 | 192 | VSAVRLAGLEHVLHFTALEGK | | | | | Mascot |
| 2260.0566 | 2260.127 | 0.0704 | 31 | 108 | 125 | MFDFHVLDMFELGIENFR | | | | | Mascot |
| 2283.0784 | 2283.1536 | 0.0752 | 33 | 135 | 154 | CPEGTKPMLVFAGDLFETDR | | | Carbamidomethyl (C)[1] | | Mascot |

| | | | | | | | | | | | |
|---|---|---|---|---|---|---|---|---|---|---|---|
| 2283.0784 | 2283.1536 | 0.0752 | 33 | 135 | 154 | CPEGTKPMLVFAGDLFE TDR | | | Carbamidomethyl (C)[1] | | Mascot |
| 2298.2427 | 2298.1658 | -0.0769 | -33 | 177 | 196 | LAGLEHVLHFTALEGKIY MR | | | | | Mascot |
| 2299.0732 | 2299.1592 | 0.086 | 37 | 135 | 154 | CPEGTKPMLVFAGDLFE TDR | | | Carbamidomethyl (C)[1], Oxidation (M)[8] | | Mascot |

15 PREDICTED: b-cell receptor CD22-like [Danio rerio] gi|326664439 59170.3 6.94 10 40 0

Peptide Information

| Calc. Mass | Obsrv. Mass | ± da | ± ppm | Start Seq. | End Seq. | Sequence | Ion Score | C. I. % | Modification | Rank | Result Type |
|---|---|---|---|---|---|---|---|---|---|---|---|
| 836.4512 | 836.3273 | -0.1239 | -148 | 86 | 92 | LQYLGDK | | | | | Mascot |
| 875.458 | 875.4556 | -0.0024 | -3 | 342 | 350 | GETLVGSGR | | | | | Mascot |
| 882.454 | 882.5558 | 0.1018 | 115 | 283 | 290 | ARNAHGEK | | | | | Mascot |
| 987.5945 | 987.524 | -0.0705 | -71 | 262 | 270 | SGRILNISK | 2 | 0 | | | Mascot |
| 987.5945 | 987.524 | -0.0705 | -71 | 262 | 270 | SGRILNISK | | | | | Mascot |
| 1022.5265 | 1022.495 | -0.0315 | -31 | 115 | 123 | FTTNVANQK | | | | | Mascot |
| 1100.4742 | 1100.5676 | 0.0934 | 85 | 271 | 280 | ISSDDSGEYK | | | | | Mascot |
| 1205.5586 | 1205.6239 | 0.0653 | 54 | 106 | 114 | KDEGQYYFR | | | | | Mascot |
| 1412.7209 | 1412.6305 | -0.0904 | -64 | 161 | 171 | LTDTPTFIWYR | | | | | Mascot |
| 1874.9105 | 1874.9208 | 0.0103 | 5 | 157 | 171 | SSCKLTDTPTFIWYR | | | Carbamidomethyl (C)[3] | | Mascot |
| 2472.2051 | 2472.1626 | -0.0425 | -17 | 198 | 219 | CAVHGHTLTSPEVYLNV MYPPK | | | Oxidation (M)[18] | | Mascot |

16 PREDICTED: interleukin-1 receptor-like 2-like [Danio rerio] gi|326670867 62378.6 7.52 6 39 0 24 7.384

Peptide Information

| Calc. Mass | Obsrv. Mass | ± da | ± ppm | Start Seq. | End Seq. | Sequence | Ion Score | C. I. % | Modification | Rank | Result Type |
|---|---|---|---|---|---|---|---|---|---|---|---|
| 850.4338 | 850.5328 | 0.099 | 116 | 540 | 547 | KGTDSMVL | | | | | Mascot |
| 850.4338 | 850.5328 | 0.099 | 116 | 540 | 547 | KGTDSMVL | | | | | Mascot |
| 864.4308 | 864.4857 | 0.0549 | 64 | 128 | 135 | DELTSTAK | | | | | Mascot |
| 987.537 | 987.524 | -0.013 | -13 | 507 | 514 | TQGILNWR | 24 | 7.384 | | | Mascot |
| 987.537 | 987.524 | -0.013 | -13 | 507 | 514 | TQGILNWR | | | | | Mascot |
| 1235.5508 | 1235.6188 | 0.068 | 55 | 157 | 167 | NCQPLNMSNSK | | | | | Mascot |
| 1412.6256 | 1412.6305 | 0.0049 | 3 | 352 | 364 | SVCSRSVACSDGK | | | Carbamidomethyl (C)[3,9] | | Mascot |
| 1874.9218 | 1874.9208 | -0.001 | -1 | 39 | 54 | TYTFVAGLAFNLQCNR | | | Carbamidomethyl (C)[14] | | Mascot |

17 polymerase I and transcript release factor isoform 2 [Danio rerio] gi|167621556 51742.2 5.07 4 38 0 29 69.543

Peptide Information

| Calc. Mass | Obsrv. Mass | ± da | ± ppm | Start | End | Sequence | Ion | C. I. % | Modification | Rank | Result Type |
|---|---|---|---|---|---|---|---|---|---|---|---|

| | | | | Seq. | Seq. | | Score | | | | |
|---|---|---|---|---|---|---|---|---|---|---|---|
| 987.5945 | 987.524 | -0.0705 | -71 | 128 | 136 | VSVNVKSVR | 29 | 69.543 | | | Mascot |
| 987.5945 | 987.524 | -0.0705 | -71 | 128 | 136 | VSVNVKSVR | | | | | Mascot |
| 1156.5667 | 1156.597 | 0.0303 | 26 | 249 | 258 | TVFSKGNMEK | | | Oxidation (M)[8] | | Mascot |
| 1163.6782 | 1163.6011 | -0.0771 | -66 | 244 | 253 | VQNIKTVFSK | | | | | Mascot |
| 1199.6935 | 1199.5568 | -0.1367 | -114 | 367 | 376 | VPPFTFHVKK | | | | | Mascot |

| | | | | | | | |
|---|---|---|---|---|---|---|---|
| 18 | PREDICTED: 1-phosphatidylinositol-4,5-bisphosphate phosphodiesterase beta-4-like [Danio rerio] | gi\|326675406 | 130576.6 | 6.89 | 16 | 38 | 0 |

Peptide Information

| Calc. Mass | Obsrv. Mass | ± da | ± ppm | Start Seq. | End Seq. | Sequence | Ion Score | C. I. % | Modification | Rank | Result Type |
|---|---|---|---|---|---|---|---|---|---|---|---|
| 842.4981 | 842.5021 | 0.004 | 5 | 442 | 448 | LKPEVEK | | | | | Mascot |
| 853.4413 | 853.3546 | -0.0867 | -102 | 318 | 324 | SSVEIYR | | | | | Mascot |
| 970.5931 | 970.4955 | -0.0976 | -101 | 442 | 449 | LKPEVEKK | | | | | Mascot |
| 1022.5451 | 1022.495 | -0.0501 | -49 | 595 | 602 | QMSRIYPK | | | | | Mascot |
| 1100.5115 | 1100.5676 | 0.0561 | 51 | 355 | 363 | AMCTDVLFK | | | Carbamidomethyl (C)[3], Oxidation (M)[2] | | Mascot |
| 1294.5701 | 1294.5137 | -0.0564 | -44 | 115 | 125 | ANNVCPMTCLR | | | Carbamidomethyl (C)[5], Oxidation (M)[7] | | Mascot |
| 1312.6136 | 1312.6016 | -0.012 | -9 | 261 | 271 | KGLLSCDGFCR | | | Carbamidomethyl (C)[6,10] | | Mascot |
| 1412.6331 | 1412.6305 | -0.0026 | -2 | 855 | 866 | TNQAGMMMKPQK | | | Oxidation (M)[6,7,8] | | Mascot |
| 1865.8085 | 1865.8218 | 0.0133 | 7 | 2 | 17 | DSAMDPLVFESNCFFK | | | Oxidation (M)[4] | | Mascot |
| 1865.8085 | 1865.8218 | 0.0133 | 7 | 2 | 17 | DSAMDPLVFESNCFFK | | | Oxidation (M)[4] | | Mascot |
| 1912.8562 | 1912.9598 | 0.1036 | 54 | 987 | 1003 | VKDMASAQASECSEMLR | | | Carbamidomethyl (C)[12] | | Mascot |
| 1993.9316 | 1993.9501 | 0.0185 | 9 | 56 | 73 | VLCALEASGKCESNLED R | | | Carbamidomethyl (C)[3] | | Mascot |
| 2096.9568 | 2097.1064 | 0.1496 | 71 | 642 | 658 | FEYNGSCGFLLKPDFMR | | | Carbamidomethyl (C)[7], Oxidation (M)[16] | | Mascot |
| 2096.9568 | 2097.1064 | 0.1496 | 71 | 642 | 658 | FEYNGSCGFLLKPDFMR | | | Carbamidomethyl (C)[7], Oxidation (M)[16] | | Mascot |
| 2151.9983 | 2152.0684 | 0.0701 | 33 | 325 | 342 | QVLLTGCRCVELDCWDG K | | | Carbamidomethyl (C)[7,9] | | Mascot |
| 2247.2205 | 2247.0789 | -0.1416 | -63 | 780 | 798 | NEYNKPLPLATVFCNIILK | | | Carbamidomethyl (C)[14] | | Mascot |
| 2472.1169 | 2472.1626 | 0.0457 | 18 | 333 | 354 | CVELDCWDGKGEDQEPII THGK | | | | | Mascot |
| 2486.1907 | 2486.1956 | 0.0049 | 2 | 371 | 391 | ETAFLTSEYPVILSFENHC TK | | | Carbamidomethyl (C)[19] | | Mascot |

| | | | | | | | |
|---|---|---|---|---|---|---|---|
| 19 | PREDICTED: zinc finger protein 420 isoform 2 [Danio rerio] | gi\|326678086 | 70371 | 9.08 | 13 | 36 | 0 |

Peptide Information

| Calc. Mass | Obsrv. Mass | ± da | ± ppm | Start Seq. | End Seq. | Sequence | Ion Score | C. I. % | Modification | Rank | Result Type |
|---|---|---|---|---|---|---|---|---|---|---|---|

| | | | | | | | | | | | | |
|---|---|---|---|---|---|---|---|---|---|---|---|---|
| 842.5206 | 842.5021 | -0.0185 | -22 | 25 | 31 | QRQGLLK | | | | | Mascot |
| 864.4798 | 864.4857 | 0.0059 | 7 | 591 | 597 | HRSVHTK | | | | | Mascot |
| 987.5621 | 987.524 | -0.0381 | -39 | 497 | 505 | TFITAGHLK | | | | | Mascot |
| 987.5621 | 987.524 | -0.0381 | -39 | 497 | 505 | TFITAGHLK | | | | | Mascot |
| 1007.392 | 1007.4681 | 0.0761 | 76 | 36 | 43 | MSDPEPCR | | | Carbamidomethyl (C)[7], Oxidation (M)[1] | | Mascot |
| 1045.536 | 1045.5532 | 0.0172 | 16 | 134 | 141 | FHCARNLK | | | Carbamidomethyl (C)[3] | | Mascot |
| 1100.5881 | 1100.5676 | -0.0205 | -19 | 165 | 173 | ATDLKMHLR | | | Oxidation (M)[6] | | Mascot |
| 1156.5238 | 1156.597 | 0.0732 | 63 | 575 | 584 | CDQCGKSFLR | | | | | Mascot |
| 1625.8428 | 1625.7463 | -0.0965 | -59 | 593 | 606 | SVHTKEKPPSCSLR | | | Carbamidomethyl (C)[11] | | Mascot |
| 1961.8956 | 1961.97 | 0.0744 | 38 | 454 | 469 | VHTNEKPYSCPECGKR | | | Carbamidomethyl (C)[10,13] | | Mascot |
| 2102.0085 | 2101.9805 | -0.028 | -13 | 488 | 505 | EFVCFECEKTFITAGHLK | | | | | Mascot |
| 2211.0798 | 2211.0801 | 0.0003 | 0 | 450 | 468 | IHLRVHTNEKPYSCPECG K | | | | | Mascot |
| 2283.0579 | 2283.1536 | 0.0957 | 42 | 227 | 245 | HQMIHTGEKPYKCSHCD LR | | | | | Mascot |
| 2283.0579 | 2283.1536 | 0.0957 | 42 | 227 | 245 | HQMIHTGEKPYKCSHCD LR | | | | | Mascot |
| 2298.0906 | 2298.1658 | 0.0752 | 33 | 478 | 496 | LHQHVHTGVREFVCFEC EK | | | | | Mascot |
| 2299.053 | 2299.1592 | 0.1062 | 46 | 227 | 245 | HQMIHTGEKPYKCSHCD LR | | | Oxidation (M)[3] | | Mascot |

| | | | | | | | | |
|---|---|---|---|---|---|---|---|---|
| 20 | PREDICTED: WD repeat- and FYVE domain-containing protein 4 [Danio rerio] | gi\|326673033 | 526148.3 | 6.32 | 34 | 36 | 0 |

**Peptide Information**

| Calc. Mass | Obsrv. Mass | ± da | ± ppm | Start Seq. | End Seq. | Sequence | Ion Score | C. I. % | Modification | Rank | Result Type |
|---|---|---|---|---|---|---|---|---|---|---|---|
| 836.3567 | 836.3273 | -0.0294 | -35 | 4178 | 4184 | DNMSDVR | | | | | Mascot |
| 850.5145 | 850.5328 | 0.0183 | 22 | 2616 | 2622 | RSFSLLK | | | | | Mascot |
| 850.5145 | 850.5328 | 0.0183 | 22 | 2616 | 2622 | RSFSLLK | | | | | Mascot |
| 962.4434 | 962.5256 | 0.0822 | 85 | 1341 | 1348 | MADLPMER | | | | | Mascot |
| 987.4642 | 987.524 | 0.0598 | 61 | 2447 | 2454 | DEAFNHVR | | | | | Mascot |
| 987.4642 | 987.524 | 0.0598 | 61 | 2447 | 2454 | DEAFNHVR | | | | | Mascot |
| 1007.4826 | 1007.4681 | -0.0145 | -14 | 2558 | 2566 | LASQSMDQK | | | | | Mascot |
| 1022.5009 | 1022.495 | -0.0059 | -6 | 4283 | 4291 | ASMKDPVMK | | | Oxidation (M)[3] | | Mascot |
| 1051.5492 | 1051.7001 | 0.1509 | 144 | 2205 | 2213 | DLGMIPYVK | | | Oxidation (M)[4] | | Mascot |
| 1051.5492 | 1051.7001 | 0.1509 | 144 | 2205 | 2213 | DLGMIPYVK | | | Oxidation (M)[4] | | Mascot |
| 1100.4355 | 1100.5676 | 0.1321 | 120 | 1322 | 1330 | IMSDCCLCR | | | Carbamidomethyl (C)[5] | | Mascot |
| 1156.5891 | 1156.597 | 0.0079 | 7 | 1173 | 1181 | MLQEQSHKR | | | | | Mascot |
| 1179.6844 | 1179.5908 | -0.0936 | -79 | 1279 | 1288 | DQHIATLLLR | | | | | Mascot |
| 1199.5153 | 1199.5568 | 0.0415 | 35 | 1322 | 1331 | IMSDCCLCRR | | | | | Mascot |

| | | | | | | | | | | | |
|---|---|---|---|---|---|---|---|---|---|---|---|
| 1205.667 | 1205.6239 | -0.0431 | -36 | 3995 | 4005 | LCSVVSSLKGR | | | Carbamidomethyl (C)[2] | | Mascot |
| 1272.6848 | 1272.7003 | 0.0155 | 12 | 1150 | 1159 | LIAHFSDWKR | | | | | Mascot |
| 1412.7393 | 1412.6305 | -0.1088 | -77 | 1087 | 1098 | GLRVGDFIEHNR | | | | | Mascot |
| 1456.8444 | 1456.6569 | -0.1875 | -129 | 3362 | 3374 | ILLMDSLINIPAK | | | Oxidation (M)[4] | | Mascot |
| 1587.9117 | 1587.8649 | -0.0468 | -29 | 1051 | 1063 | FHTFLANRVTIIR | | | | | Mascot |
| 1587.9117 | 1587.8649 | -0.0468 | -29 | 1051 | 1063 | FHTFLANRVTIIR | | | | | Mascot |
| 1625.7518 | 1625.7463 | -0.0055 | -3 | 1336 | 1348 | MMLQKMADLPMER | | | Oxidation (M)[1,2] | | Mascot |
| 1638.6848 | 1638.8395 | 0.1547 | 94 | 35 | 47 | SCLYMMPELGYDK | | | Carbamidomethyl (C)[2], Oxidation (M)[5,6] | | Mascot |
| 1815.9785 | 1815.87 | -0.1085 | -60 | 3735 | 3748 | MAIQWLRIEEELLR | | | Oxidation (M)[1] | | Mascot |
| 1866.0154 | 1865.8218 | -0.1936 | -104 | 1591 | 1607 | NLKPGDIVIIMDPTAPR | | | Oxidation (M)[11] | | Mascot |
| 1866.0154 | 1865.8218 | -0.1936 | -104 | 1591 | 1607 | NLKPGDIVIIMDPTAPR | | | Oxidation (M)[11] | | Mascot |
| 1952.0885 | 1951.9854 | -0.1031 | -53 | 2812 | 2830 | SCLVTVYLNATAIGTGKIK | | | | | Mascot |
| 1961.8796 | 1961.97 | 0.0904 | 46 | 777 | 792 | ENFYVDDCLKSVESEK | | | Carbamidomethyl (C)[8] | | Mascot |
| 1993.9907 | 1993.9501 | -0.0406 | -20 | 2521 | 2537 | QIMCEFGLLSTLLTHCK | | | Carbamidomethyl (C)[4] | | Mascot |
| 2097.0076 | 2097.1064 | 0.0988 | 47 | 721 | 737 | FLWWPEGDLTKDASEFR | | | | | Mascot |
| 2097.1526 | 2097.1064 | -0.0462 | -22 | 2617 | 2635 | SFSLLKTPLDSGMVVPLHR | | | | | Mascot |
| 2102.04 | 2101.9805 | -0.0595 | -28 | 758 | 776 | KTADDNQAEFPAEIPQTVK | | | | | Mascot |
| 2224.9355 | 2225.0945 | 0.159 | 71 | 257 | 274 | HLCFGCLSTSHMSRDCEK | | | Carbamidomethyl (C)[3,6,16] | | Mascot |
| 2224.9355 | 2225.0945 | 0.159 | 71 | 257 | 274 | HLCFGCLSTSHMSRDCEK | | | Carbamidomethyl (C)[3,6,16] | | Mascot |
| 2233.0127 | 2233.063 | 0.0503 | 23 | 3183 | 3201 | SWSFECLSCICPGFEVLQK | | | Carbamidomethyl (C)[6] | | Mascot |
| 2283.1948 | 2283.1536 | -0.0412 | -18 | 2024 | 2043 | IIQSSFMRSSNTVICSQILR | | | | | Mascot |
| 2283.1948 | 2283.1536 | -0.0412 | -18 | 2024 | 2043 | IIQSSFMRSSNTVICSQILR | | | | | Mascot |
| 2284.1389 | 2284.1504 | 0.0115 | 5 | 2349 | 2369 | LGEALLHLGCFQKGDPVESDR | | | | | Mascot |
| 2299.1772 | 2299.1592 | -0.018 | -8 | 2789 | 2807 | CAKHLIPGQWHHLCLVMAK | | | Carbamidomethyl (C)[1,14] | | Mascot |
| 2300.1206 | 2300.1565 | 0.0359 | 16 | 910 | 927 | NYGWDETVPQELLHRWTR | | | | | Mascot |
| 2486.2603 | 2486.1956 | -0.0647 | -26 | 1878 | 1901 | VLRSVSAAQAHNTVSALQASNCMK | | | | | Mascot |
| 3337.6045 | 3337.7273 | 0.1228 | 37 | 3638 | 3666 | EGQVSMADVSPLWEEQATKSWQLFIDSQK | | | | | Mascot |

21 PREDICTED: RNA-directed DNA polymerase from mobile element jockey-like [Danio rerio] gi|326672568 119050.6 9.55 16 36 0

**Peptide Information**

| Calc. Mass | Obsrv. Mass | ± da | ± ppm | Start Seq. | End Seq. | Sequence | Ion Score | C. I. % | Modification | Rank | Result Type |
|---|---|---|---|---|---|---|---|---|---|---|---|
| 864.3628 | 864.4857 | 0.1229 | 142 | 168 | 174 | MNENGQR | | | Oxidation (M)[1] | | Mascot |
| 870.4402 | 870.532 | 0.0918 | 105 | 916 | 922 | WLGHVCR | | | | | Mascot |

| Calc. Mass | Obsrv. Mass | ± da | ± ppm | Start Seq. | End Seq. | Sequence | Ion Score | C. I. % | Modification | Rank | Result Type |
|---|---|---|---|---|---|---|---|---|---|---|---|
| 987.5945 | 987.524 | -0.0705 | -71 | 817 | 826 | IGKAASTLAR | 6 | 0 | | | Mascot |
| 987.5945 | 987.524 | -0.0705 | -71 | 817 | 826 | IGKAASTLAR | | | | | Mascot |
| 996.4819 | 996.584 | 0.1021 | 102 | 1041 | 1048 | QSVMPQYK | | | Oxidation (M)[4] | | Mascot |
| 996.4819 | 996.584 | 0.1021 | 102 | 1041 | 1048 | QSVMPQYK | | | Oxidation (M)[4] | | Mascot |
| 1100.6534 | 1100.5676 | -0.0858 | -78 | 355 | 363 | NLQILRSTR | | | | | Mascot |
| 1163.58 | 1163.6011 | 0.0211 | 18 | 330 | 340 | ASTMIPCIEAK | | | | | Mascot |
| 1179.5748 | 1179.5908 | 0.016 | 14 | 330 | 340 | ASTMIPCIEAK | | | Oxidation (M)[4] | | Mascot |
| 1235.548 | 1235.6188 | 0.0708 | 57 | 50 | 58 | DYTFYWQGR | | | | | Mascot |
| 1279.6892 | 1279.7589 | 0.0697 | 54 | 485 | 497 | APGSDGIPPDLLK | | | | | Mascot |
| 1475.7787 | 1475.7354 | -0.0433 | -29 | 498 | 509 | QCKDSLLHPLHK | | | Carbamidomethyl (C)[2] | | Mascot |
| 1638.8519 | 1638.8395 | -0.0124 | -8 | 485 | 500 | APGSDGIPPDLLKQCK | | | | | Mascot |
| 1912.9069 | 1912.9598 | 0.0529 | 28 | 417 | 432 | SSTGEIIFDKQQQMER | | | Oxidation (M)[14] | | Mascot |
| 1961.8513 | 1961.97 | 0.1187 | 61 | 1007 | 1024 | VQSVSQGATYQCDCCGKK | | | Carbamidomethyl (C)[12] | | Mascot |
| 2101.9858 | 2101.9805 | -0.0053 | -3 | 653 | 672 | GTIQFNGSCSEPFSISSGVK | | | Carbamidomethyl (C)[9] | | Mascot |
| 2151.9863 | 2152.0684 | 0.0821 | 38 | 286 | 304 | ELDTAQTGDSALEMWESLR | | | | | Mascot |
| 2194.0308 | 2194.0774 | 0.0466 | 21 | 322 | 340 | THDWFEAKASTMIPCIEAK | | | Oxidation (M)[12] | | Mascot |
| 2233.0364 | 2233.063 | 0.0266 | 12 | 457 | 475 | CLPTMEELDEEPTAEELRK | | | | | Mascot |

22 hypothetical protein LOC795694 [Danio rerio] gi|157841207 31024 4.24 8 36 0

**Peptide Information**

| Calc. Mass | Obsrv. Mass | ± da | ± ppm | Start Seq. | End Seq. | Sequence | Ion Score | C. I. % | Modification | Rank | Result Type |
|---|---|---|---|---|---|---|---|---|---|---|---|
| 970.6043 | 970.4955 | -0.1088 | -112 | 166 | 173 | QLKEILAR | | | | | Mascot |
| 1022.4216 | 1022.495 | 0.0734 | 72 | 254 | 261 | MNECPICR | | | Carbamidomethyl (C)[4] | | Mascot |
| 1235.5442 | 1235.6188 | 0.0746 | 60 | 253 | 261 | RMNECPICR | | | Carbamidomethyl (C)[5,8] | | Mascot |
| 1638.8698 | 1638.8395 | -0.0303 | -18 | 44 | 57 | EELVELVLDQQAPR | | | | | Mascot |
| 1815.8265 | 1815.87 | 0.0435 | 24 | 28 | 41 | DYLHLHQTPTQMCR | | | Carbamidomethyl (C)[13], Oxidation (M)[12] | | Mascot |
| 1961.9537 | 1961.97 | 0.0163 | 8 | 185 | 199 | WELMEKVTHLFNDQR | | | Oxidation (M)[4] | | Mascot |
| 2193.9402 | 2194.0774 | 0.1372 | 63 | 174 | 190 | NFVNYQGCCEKWELMEK | | | Carbamidomethyl (C)[8], Oxidation (M)[15] | | Mascot |
| 2486.0562 | 2486.1956 | 0.1394 | 56 | 228 | 249 | ICMDSPIDCVLLECGHMVTCSK | | | Carbamidomethyl (C)[2], Oxidation (M)[3,17] | | Mascot |

23 novel protein [Danio rerio] gi|94733366 36191.6 9.32 10 36 0

**Peptide Information**

| Calc. Mass | Obsrv. Mass | ± da | ± ppm | Start Seq. | End Seq. | Sequence | Ion Score | C. I. % | Modification | Rank | Result Type |
|---|---|---|---|---|---|---|---|---|---|---|---|

| Calc. Mass | Obsrv. Mass | ± da | ± ppm | Start Seq. | End Seq. | Sequence | Ion Score | C. I. % | Modification | Rank | Result Type |
|---|---|---|---|---|---|---|---|---|---|---|---|
| 864.4798 | 864.4857 | 0.0059 | 7 | 292 | 298 | HRSVHTK | | | | | Mascot |
| 962.3931 | 962.5256 | 0.1325 | 138 | 108 | 114 | CSHCDKR | | | Carbamidomethyl (C)[1,4] | | Mascot |
| 987.5621 | 987.524 | -0.0381 | -39 | 198 | 206 | TFITAGHLK | | | | | Mascot |
| 987.5621 | 987.524 | -0.0381 | -39 | 198 | 206 | TFITAGHLK | | | | | Mascot |
| 1007.392 | 1007.4681 | 0.0761 | 76 | 19 | 26 | MSDPEPCR | | | Carbamidomethyl (C)[7], Oxidation (M)[1] | | Mascot |
| 1156.5238 | 1156.597 | 0.0732 | 63 | 276 | 285 | CDQCGKSFLR | | | | | Mascot |
| 1625.8428 | 1625.7463 | -0.0965 | -59 | 294 | 307 | SVHTKEKPPSCSLR | | | Carbamidomethyl (C)[11] | | Mascot |
| 1961.8956 | 1961.97 | 0.0744 | 38 | 155 | 170 | VHTNEKPYSCPECGKR | | | Carbamidomethyl (C)[10,13] | | Mascot |
| 2102.0085 | 2101.9805 | -0.028 | -13 | 189 | 206 | EFVCFECEKTFITAGHLK | | | | | Mascot |
| 2211.0798 | 2211.0801 | 0.0003 | 0 | 151 | 169 | IHLRVHTNEKPYSCPECGK | | | | | Mascot |
| 2298.0906 | 2298.1658 | 0.0752 | 33 | 179 | 197 | LHQHVHTGVREFVCFECEK | | | | | Mascot |

24 integrin beta 3a [Danio rerio] gi|82524316 85636.2 5.46 14 36 0

**Peptide Information**

| Calc. Mass | Obsrv. Mass | ± da | ± ppm | Start Seq. | End Seq. | Sequence | Ion Score | C. I. % | Modification | Rank | Result Type |
|---|---|---|---|---|---|---|---|---|---|---|---|
| 987.4603 | 987.524 | 0.0637 | 65 | 174 | 182 | MGFGAFVDK | | | Oxidation (M)[1] | | Mascot |
| 987.4603 | 987.524 | 0.0637 | 65 | 174 | 182 | MGFGAFVDK | | | Oxidation (M)[1] | | Mascot |
| 1156.552 | 1156.597 | 0.045 | 39 | 749 | 757 | EFAKFEEEK | | | | | Mascot |
| 1163.5837 | 1163.6011 | 0.0174 | 15 | 54 | 64 | GGSALSRCDLK | | | Carbamidomethyl (C)[8] | | Mascot |
| 1205.595 | 1205.6239 | 0.0289 | 24 | 762 | 771 | WEAANNPLYK | | | | | Mascot |
| 1279.5844 | 1279.7589 | 0.1745 | 136 | 624 | 635 | CPTCPDACTIKK | | | | | Mascot |
| 1312.6453 | 1312.6016 | -0.0437 | -33 | 414 | 425 | IGDMVSFSIEAK | | | Oxidation (M)[4] | | Mascot |
| 1412.662 | 1412.6305 | -0.0315 | -22 | 21 | 34 | GSNICTSRGVSTCK | | | | | Mascot |
| 1472.7128 | 1472.6549 | -0.0579 | -39 | 772 | 785 | GATTTFQNVAYRGS | | | | | Mascot |
| 1475.5746 | 1475.7354 | 0.1608 | 109 | 587 | 601 | TDTCMGSGGLMCSGR | | | | | Mascot |
| 1874.7295 | 1874.9208 | 0.1913 | 102 | 539 | 553 | VWGPFCECDDFSCPR | | | Carbamidomethyl (C)[6,8] | | Mascot |
| 2082.98 | 2082.9888 | 0.0088 | 4 | 240 | 258 | DSPEGGFDAIFQAVVCKDK | | | Carbamidomethyl (C)[16] | | Mascot |
| 2259.8708 | 2260.127 | 0.2562 | 113 | 602 | 623 | GHCVCGVCNCTQPGAYGSTCDK | | | Carbamidomethyl (C)[3] | | Mascot |
| 2284.3535 | 2284.1504 | -0.2031 | -89 | 1 | 20 | MGLNLIKVWMPVLLLISIAK | | | Oxidation (M)[1,10] | | Mascot |
| 2317.2771 | 2317.1436 | -0.1335 | -58 | 8 | 28 | VWMPVLLLISIAKGSNICTSR | | | Oxidation (M)[3] | | Mascot |

25 hypothetical protein LOC564090 [Danio rerio] gi|192453584 12976.7 10.42 5 36 0

**Peptide Information**

| Calc. Mass | Obsrv. Mass | ± da | ± ppm | Start Seq. | End Seq. | Sequence | Ion Score | C. I. % | Modification | Rank | Result Type |
|---|---|---|---|---|---|---|---|---|---|---|---|

| Calc. Mass | Obsrv. Mass | ± da | ± ppm | Start Seq. | End Seq. | Sequence | Ion Score | C. I. % | Modification | Rank | Result Type |
|---|---|---|---|---|---|---|---|---|---|---|---|
| 1100.5219 | 1100.5676 | 0.0457 | 42 | 100 | 108 | NDQQEEPIK | | | | | Mascot |
| 1625.8621 | 1625.7463 | -0.1158 | -71 | 1 | 14 | MIFGFPRPTAGYIR | | | | | Mascot |
| 2211.0889 | 2211.0801 | -0.0088 | -4 | 100 | 118 | NDQQEEPIKNISDTQQPVK | | | | | Mascot |
| 2225.123 | 2225.0945 | -0.0285 | -13 | 88 | 108 | LAPANAGMASIKNDQQEEPIK | | | | | Mascot |
| 2225.123 | 2225.0945 | -0.0285 | -13 | 88 | 108 | LAPANAGMASIKNDQQEEPIK | | | | | Mascot |
| 2260.085 | 2260.127 | 0.042 | 19 | 65 | 85 | ILQWMSRPAMNADSPGSPSAK | | | Oxidation (M)[5] | | Mascot |

| | | | | | | | | |
|---|---|---|---|---|---|---|---|---|
| 26 | truncated SH3 binding domain protein 4 [Danio rerio] | gi\|33438222 | 84564.4 | 8.75 | 12 | 35 | 0 | |

**Peptide Information**

| Calc. Mass | Obsrv. Mass | ± da | ± ppm | Start Seq. | End Seq. | Sequence | Ion Score | C. I. % | Modification | Rank | Result Type |
|---|---|---|---|---|---|---|---|---|---|---|---|
| 842.5822 | 842.5021 | -0.0801 | -95 | 634 | 640 | LIKTVIR | | | | | Mascot |
| 850.4338 | 850.5328 | 0.099 | 116 | 358 | 364 | LSNMEIK | | | Oxidation (M)[4] | | Mascot |
| 850.4338 | 850.5328 | 0.099 | 116 | 358 | 364 | LSNMEIK | | | Oxidation (M)[4] | | Mascot |
| 870.4726 | 870.532 | 0.0594 | 68 | 1 | 7 | MAAHRIR | | | Oxidation (M)[1] | | Mascot |
| 1163.6432 | 1163.6011 | -0.0421 | -36 | 742 | 750 | HPYPRQLPR | | | | | Mascot |
| 1272.7522 | 1272.7003 | -0.0519 | -41 | 476 | 487 | TLLVSEVGKQAK | | | | | Mascot |
| 1294.6637 | 1294.5137 | -0.15 | -116 | 553 | 563 | KADEISDFTLR | | | | | Mascot |
| 1371.7161 | 1371.6206 | -0.0955 | -70 | 8 | 19 | VTNNNNVLPRCK | | | | | Mascot |
| 1815.8959 | 1815.87 | -0.0259 | -14 | 432 | 446 | HVSFSSNVWNHMVKK | | | Oxidation (M)[12] | | Mascot |
| 2097.0137 | 2097.1064 | 0.0927 | 44 | 373 | 390 | VSVMTRTESPMAEIVCVR | | | Carbamidomethyl (C)[16], Oxidation (M)[4,11] | | Mascot |
| 2097.0137 | 2097.1064 | 0.0927 | 44 | 373 | 390 | VSVMTRTESPMAEIVCVR | | | Carbamidomethyl (C)[16], Oxidation (M)[4,11] | | Mascot |
| 2284.2368 | 2284.1504 | -0.0864 | -38 | 463 | 484 | TVVAIFGHDCAPKTLLVSEVGK | | | | | Mascot |
| 2300.0686 | 2300.1565 | 0.0879 | 38 | 568 | 587 | DDQDCILAQFCVQTPQPPPK | | | Carbamidomethyl (C)[5] | | Mascot |
| 3337.6052 | 3337.7273 | 0.1221 | 37 | 269 | 298 | LARSCHDLESLGQNPGWGQTQPVETNIVCK | | | Carbamidomethyl (C)[5] | | Mascot |

| | | | | | | | | |
|---|---|---|---|---|---|---|---|---|
| 27 | PREDICTED: zinc finger protein 420 isoform 1 [Danio rerio] | gi\|326678084 | 37946.4 | 9.32 | 10 | 35 | 0 | |

**Peptide Information**

| Calc. Mass | Obsrv. Mass | ± da | ± ppm | Start Seq. | End Seq. | Sequence | Ion Score | C. I. % | Modification | Rank | Result Type |
|---|---|---|---|---|---|---|---|---|---|---|---|
| 864.4798 | 864.4857 | 0.0059 | 7 | 309 | 315 | HRSVHTK | | | | | Mascot |
| 962.3931 | 962.5256 | 0.1325 | 138 | 125 | 131 | CSHCDKR | | | Carbamidomethyl (C)[1,4] | | Mascot |
| 987.5621 | 987.524 | -0.0381 | -39 | 215 | 223 | TFITAGHLK | | | | | Mascot |
| 987.5621 | 987.524 | -0.0381 | -39 | 215 | 223 | TFITAGHLK | | | | | Mascot |
| 1007.392 | 1007.4681 | 0.0761 | 76 | 36 | 43 | MSDPEPCR | | | Carbamidomethyl (C)[7], Oxidation (M)[1] | | Mascot |

| | | | | | | | | | | | |
|---|---|---|---|---|---|---|---|---|---|---|---|
| 1156.5238 | 1156.597 | 0.0732 | 63 | 293 | 302 | CDQCGKSFLR | | | | | Mascot |
| 1625.8428 | 1625.7463 | -0.0965 | -59 | 311 | 324 | SVHTKEKPPSCSLR | | | Carbamidomethyl (C)[11] | | Mascot |
| 1961.8956 | 1961.97 | 0.0744 | 38 | 172 | 187 | VHTNEKPYSCPECGKR | | | Carbamidomethyl (C)[10,13] | | Mascot |
| 2102.0085 | 2101.9805 | -0.028 | -13 | 206 | 223 | EFVCFECEKTFITAGHLK | | | | | Mascot |
| 2211.0798 | 2211.0801 | 0.0003 | 0 | 168 | 186 | IHLRVHTNEKPYSCPECG K | | | | | Mascot |
| 2298.0906 | 2298.1658 | 0.0752 | 33 | 196 | 214 | LHQHVHTGVREFVCFEC EK | | | | | Mascot |

28 PREDICTED: hypothetical protein LOC100537186 [Danio rerio] gi|326680843 136727.7 9.59 17 35 0

**Peptide Information**

| Calc. Mass | Obsrv. Mass | ± da | ± ppm | Start Seq. | End Seq. | Sequence | Ion Score | C. I. % | Modification | Rank | Result Type |
|---|---|---|---|---|---|---|---|---|---|---|---|
| 864.3628 | 864.4857 | 0.1229 | 142 | 319 | 325 | MNENGQR | | | Oxidation (M)[1] | | Mascot |
| 870.4402 | 870.532 | 0.0918 | 105 | 1069 | 1075 | WLGHVCR | | | | | Mascot |
| 987.5945 | 987.524 | -0.0705 | -71 | 970 | 979 | IGKAASTLAR | 6 | 0 | | | Mascot |
| 987.5945 | 987.524 | -0.0705 | -71 | 970 | 979 | IGKAASTLAR | | | | | Mascot |
| 996.4819 | 996.584 | 0.1021 | 102 | 1194 | 1201 | QSVMPQYK | | | Oxidation (M)[4] | | Mascot |
| 996.4819 | 996.584 | 0.1021 | 102 | 1194 | 1201 | QSVMPQYK | | | Oxidation (M)[4] | | Mascot |
| 1100.6534 | 1100.5676 | -0.0858 | -78 | 506 | 514 | NLQILRSTR | | | | | Mascot |
| 1163.58 | 1163.6011 | 0.0211 | 18 | 481 | 491 | ASTMIPCIEAK | | | | | Mascot |
| 1179.5748 | 1179.5908 | 0.016 | 14 | 481 | 491 | ASTMIPCIEAK | | | Oxidation (M)[4] | | Mascot |
| 1235.548 | 1235.6188 | 0.0708 | 57 | 50 | 58 | DYTFYWQGR | | | | | Mascot |
| 1279.6892 | 1279.7589 | 0.0697 | 54 | 636 | 648 | APGSDGIPPDLLK | | | | | Mascot |
| 1475.7787 | 1475.7354 | -0.0433 | -29 | 649 | 660 | QCKDSLLHPLHK | | | Carbamidomethyl (C)[2] | | Mascot |
| 1638.8519 | 1638.8395 | -0.0124 | -8 | 636 | 651 | APGSDGIPPDLLKQCK | | | | | Mascot |
| 1912.9069 | 1912.9598 | 0.0529 | 28 | 568 | 583 | SSTGEIIFDKQQQMER | | | Oxidation (M)[14] | | Mascot |
| 1961.8513 | 1961.97 | 0.1187 | 61 | 1160 | 1177 | VQSVSQGATYQCDCCG KK | | | Carbamidomethyl (C)[12] | | Mascot |
| 2083.0417 | 2082.9888 | -0.0529 | -25 | 754 | 770 | EQEQQMPLYISFIDLTK | | | | | Mascot |
| 2101.9858 | 2101.9805 | -0.0053 | -3 | 806 | 825 | GTIQFNGSCSEPFSISSG VK | | | Carbamidomethyl (C)[9] | | Mascot |
| 2151.9863 | 2152.0684 | 0.0821 | 38 | 437 | 455 | ELDTAQTGDSALEMWES LR | | | | | Mascot |
| 2194.0308 | 2194.0774 | 0.0466 | 21 | 473 | 491 | THDWFEAKASTMIPCIEA K | | | Oxidation (M)[12] | | Mascot |
| 2233.0364 | 2233.063 | 0.0266 | 12 | 608 | 626 | CLPTMEELDEEPTAEELR K | | | | | Mascot |

29 novel protein similar to vertebrate copine IV (CPNE4) [Danio rerio] gi|94732437 25404.6 5.47 7 35 0

**Peptide Information**

| Calc. Mass | Obsrv. Mass | ± da | ± ppm | Start Seq. | End Seq. | Sequence | Ion Score | C. I. % | Modification | Rank | Result Type |
|---|---|---|---|---|---|---|---|---|---|---|---|
| 996.4819 | 996.584 | 0.1021 | 102 | 66 | 74 | SCINPTFSK | | | | | Mascot |
| 996.4819 | 996.584 | 0.1021 | 102 | 66 | 74 | SCINPTFSK | | | | | Mascot |
| 1475.6543 | 1475.7354 | 0.0811 | 55 | 209 | 221 | TSLNSLCSGDHER | | | Carbamidomethyl (C)[7] | | Mascot |
| 1913.016 | 1912.9598 | -0.0562 | -29 | 31 | 48 | GISDRDALSKPDPCVVIK | | | | | Mascot |
| 1962.012 | 1961.97 | -0.042 | -21 | 75 | 89 | VFTLDFYFEEVQRLR | | | | | Mascot |
| 2096.9409 | 2097.1064 | 0.1655 | 79 | 104 | 122 | EADCLGAMECTLGQIVS QR | | | Carbamidomethyl (C)[4], Oxidation (M)[8] | | Mascot |
| 2096.9409 | 2097.1064 | 0.1655 | 79 | 104 | 122 | EADCLGAMECTLGQIVS QR | | | Carbamidomethyl (C)[4], Oxidation (M)[8] | | Mascot |
| 2152.0195 | 2152.0684 | 0.0489 | 23 | 104 | 123 | EADCLGAMECTLGQIVS QRK | | | | | Mascot |
| 2225.0359 | 2225.0945 | 0.0586 | 26 | 104 | 123 | EADCLGAMECTLGQIVS QRK | | | Carbamidomethyl (C)[4], Oxidation (M)[8] | | Mascot |
| 2225.0359 | 2225.0945 | 0.0586 | 26 | 104 | 123 | EADCLGAMECTLGQIVS QRK | | | Carbamidomethyl (C)[4], Oxidation (M)[8] | | Mascot |
| 2260.0801 | 2260.127 | 0.0469 | 21 | 2 | 22 | SDIYESAANSLGLFSSPC LTK | | | Carbamidomethyl (C)[18] | | Mascot |

30 Integrin beta 3a [Danio rerio] gi|190337291 85666.2 5.46 14 35 0

Peptide Information

| Calc. Mass | Obsrv. Mass | ± da | ± ppm | Start Seq. | End Seq. | Sequence | Ion Score | C. I. % | Modification | Rank | Result Type |
|---|---|---|---|---|---|---|---|---|---|---|---|
| 987.4603 | 987.524 | 0.0637 | 65 | 174 | 182 | MGFGAFVDK | | | Oxidation (M)[1] | | Mascot |
| 987.4603 | 987.524 | 0.0637 | 65 | 174 | 182 | MGFGAFVDK | | | Oxidation (M)[1] | | Mascot |
| 1156.552 | 1156.597 | 0.045 | 39 | 749 | 757 | EFAKFEEEK | | | | | Mascot |
| 1163.5837 | 1163.6011 | 0.0174 | 15 | 54 | 64 | GGSALSRCDLK | | | Carbamidomethyl (C)[8] | | Mascot |
| 1205.595 | 1205.6239 | 0.0289 | 24 | 762 | 771 | WEAANNPLYK | | | | | Mascot |
| 1279.5844 | 1279.7589 | 0.1745 | 136 | 624 | 635 | CPTCPDACTIKK | | | | | Mascot |
| 1312.6453 | 1312.6016 | -0.0437 | -33 | 414 | 425 | IGDMVSFSIEAK | | | Oxidation (M)[4] | | Mascot |
| 1412.662 | 1412.6305 | -0.0315 | -22 | 21 | 34 | GSNICTSRGVSTCK | | | | | Mascot |
| 1472.7128 | 1472.6549 | -0.0579 | -39 | 772 | 785 | GATTTFQNVAYRGS | | | | | Mascot |
| 1475.5746 | 1475.7354 | 0.1608 | 109 | 587 | 601 | TDTCMGSGGLMCSGR | | | | | Mascot |
| 1874.7295 | 1874.9208 | 0.1913 | 102 | 539 | 553 | VWGPFCECDDFSCPR | | | Carbamidomethyl (C)[6,8] | | Mascot |
| 2082.98 | 2082.9888 | 0.0088 | 4 | 240 | 258 | DSPEGGFDAIFQAVVCK DK | | | Carbamidomethyl (C)[16] | | Mascot |
| 2259.8708 | 2260.127 | 0.2562 | 113 | 602 | 623 | GHCVCGVCNCTQPGAY GSTCDK | | | Carbamidomethyl (C)[3] | | Mascot |
| 2284.3535 | 2284.1504 | -0.2031 | -89 | 1 | 20 | MGLNLIKVWMPVLLLISIA K | | | Oxidation (M)[1,10] | | Mascot |
| 2317.2771 | 2317.1436 | -0.1335 | -58 | 8 | 28 | VWMPVLLLISIAKGSNICT SR | | | Oxidation (M)[3] | | Mascot |

31 PREDICTED: hypothetical protein LOC100535533 [Danio rerio] gi|326671744 3229.6 9.19 4 35 0

**Peptide Information**

| Calc. Mass | Obsrv. Mass | ± da | ± ppm | Start Seq. | End Seq. | Sequence | Ion Score | C. I. % | Modification | Rank | Result Type |
|---|---|---|---|---|---|---|---|---|---|---|---|
| 996.4641 | 996.584 | 0.1199 | 120 | 4 | 12 | AGPVFSCMK | | | Carbamidomethyl (C)[7] | | Mascot |
| 996.4641 | 996.584 | 0.1199 | 120 | 4 | 12 | AGPVFSCMK | | | Carbamidomethyl (C)[7] | | Mascot |
| 1312.621 | 1312.6016 | -0.0194 | -15 | 1 | 12 | MGKAGPVFSCMK | | | Carbamidomethyl (C)[10] | | Mascot |
| 1993.9912 | 1993.9501 | -0.0411 | -21 | 13 | 30 | HNGHAISMEIQKTGLWTA | | | | | Mascot |
| 2317.0886 | 2317.1436 | 0.055 | 24 | 4 | 24 | AGPVFSCMKHNGHAISM EIQK | | | Oxidation (M)[8,17] | | Mascot |

| | | | | | | | | |
|---|---|---|---|---|---|---|---|---|
| 32 | PREDICTED: phenylserine dehydratase-like [Danio rerio] | gi\|326673212 | 49208.8 | 7.51 | 10 | 34 | 0 |

**Peptide Information**

| Calc. Mass | Obsrv. Mass | ± da | ± ppm | Start Seq. | End Seq. | Sequence | Ion Score | C. I. % | Modification | Rank | Result Type |
|---|---|---|---|---|---|---|---|---|---|---|---|
| 1272.6656 | 1272.7003 | 0.0347 | 27 | 163 | 172 | QYGMEIYLKK | | | | | Mascot |
| 1312.7583 | 1312.6016 | -0.1567 | -119 | 432 | 442 | LLDLLAREDVR | | | | | Mascot |
| 1456.6591 | 1456.6569 | -0.0022 | -2 | 81 | 93 | DFGAEEYLNGDVK | | | | | Mascot |
| 1472.7817 | 1472.6549 | -0.1268 | -86 | 160 | 171 | LSKQYGMEIYLK | | | | | Mascot |
| 1993.9623 | 1993.9501 | -0.0122 | -6 | 415 | 431 | VCRFTVQLADWPGDMAK | | | Carbamidomethyl (C)[2] | | Mascot |
| 2193.8818 | 2194.0774 | 0.1956 | 89 | 22 | 39 | GFSDCEEYDPFWQSAEQ R | | | | | Mascot |
| 2284.0774 | 2284.1504 | 0.073 | 32 | 243 | 263 | DYGAMVISYGSTARDSIN HAR | | | | | Mascot |
| 2298.1626 | 2298.1658 | 0.0032 | 1 | 124 | 144 | SSTPPAPEHLRFQDISAA AFR | | | | | Mascot |
| 2300.0723 | 2300.1565 | 0.0842 | 37 | 243 | 263 | DYGAMVISYGSTARDSIN HAR | | | Oxidation (M)[5] | | Mascot |
| 2317.1777 | 2317.1436 | -0.0341 | -15 | 382 | 404 | CTVDTEGAMGLAAILAGQ LPELK | | | Oxidation (M)[9] | | Mascot |
| 2486.2993 | 2486.1956 | -0.1037 | -42 | 382 | 406 | CTVDTEGAMGLAAILAGQ LPELKGK | | | | | Mascot |

| | | | | | | | | |
|---|---|---|---|---|---|---|---|---|
| 33 | Si:ch211-218c6.6 protein [Danio rerio] | gi\|61402728 | 45287.5 | 5.86 | 8 | 34 | 0 |

**Peptide Information**

| Calc. Mass | Obsrv. Mass | ± da | ± ppm | Start Seq. | End Seq. | Sequence | Ion Score | C. I. % | Modification | Rank | Result Type |
|---|---|---|---|---|---|---|---|---|---|---|---|
| 875.4329 | 875.4556 | 0.0227 | 26 | 112 | 118 | DVNRTDR | | | | | Mascot |
| 987.4927 | 987.524 | 0.0313 | 32 | 288 | 295 | HINELSMK | | | Oxidation (M)[7] | | Mascot |
| 987.4927 | 987.524 | 0.0313 | 32 | 288 | 295 | HINELSMK | | | Oxidation (M)[7] | | Mascot |
| 1156.611 | 1156.597 | -0.014 | -12 | 237 | 245 | ELHFQDVLR | | | | | Mascot |
| 1312.712 | 1312.6016 | -0.1104 | -84 | 236 | 245 | RELHFQDVLR | | | | | Mascot |

| | | | | | | | | | | | |
|---|---|---|---|---|---|---|---|---|---|---|---|
| 1951.9946 | 1951.9854 | -0.0092 | -5 | 280 | 295 | YGFNEILKHINELSMK | | | Oxidation (M)[15] | | Mascot |
| 2152.0876 | 2152.0684 | -0.0192 | -9 | 296 | 313 | LDIEEILSKSESICMQIK | | | Carbamidomethyl (C)[14], Oxidation (M)[15] | | Mascot |
| 2260.0923 | 2260.127 | 0.0347 | 15 | 254 | 272 | LPCQNFHLLVCCAILDSEK | | | Carbamidomethyl (C)[3,11] | | Mascot |
| 2317.1138 | 2317.1436 | 0.0298 | 13 | 254 | 272 | LPCQNFHLLVCCAILDSEK | | | Carbamidomethyl (C)[3,11,12] | | Mascot |
| 2501.1912 | 2501.2251 | 0.0339 | 14 | 1 | 22 | GDLGPRPEMQRTGPVTMEEWAK | | | Oxidation (M)[9] | | Mascot |

| | | | | | | | |
|---|---|---|---|---|---|---|---|
| 34 | PREDICTED: si:dkeyp-11g8.5 [Danio rerio] | gi\|326677187 | 79872.5 | 8.33 | 12 | 34 | 0 |

**Peptide Information**

| Calc. Mass | Obsrv. Mass | ± da | ± ppm | Start Seq. | End Seq. | Sequence | Ion Score | C. I. % | Modification | Rank | Result Type |
|---|---|---|---|---|---|---|---|---|---|---|---|
| 1100.5881 | 1100.5676 | -0.0205 | -19 | 167 | 175 | TDMISHLKR | | | | | Mascot |
| 1205.5046 | 1205.6239 | 0.1193 | 99 | 127 | 135 | MCICHLPCR | | | Carbamidomethyl (C)[2,4], Oxidation (M)[1] | | Mascot |
| 1294.5869 | 1294.5137 | -0.0732 | -57 | 55 | 66 | NENTTSSAVSER | | | | | Mascot |
| 1371.7128 | 1371.6206 | -0.0922 | -67 | 666 | 677 | VSRTHFDPTGVR | | | | | Mascot |
| 1815.9646 | 1815.87 | -0.0946 | -52 | 374 | 388 | SLNVTLRHYGCQIVR | | | Carbamidomethyl (C)[11] | | Mascot |
| 1993.8695 | 1993.9501 | 0.0806 | 40 | 180 | 197 | GETEASYSGNLDAYEIMK | | | Oxidation (M)[17] | | Mascot |
| 2211.1616 | 2211.0801 | -0.0815 | -37 | 353 | 373 | NVRNLGIVSVTSTDTGSLYSK | | | | | Mascot |
| 2225.1409 | 2225.0945 | -0.0464 | -21 | 299 | 320 | TGRQLDGAGDPESPIASVEVVK | | | | | Mascot |
| 2225.1409 | 2225.0945 | -0.0464 | -21 | 299 | 320 | TGRQLDGAGDPESPIASVEVVK | | | | | Mascot |
| 2297.0447 | 2297.1614 | 0.1167 | 51 | 118 | 135 | VYVEFEGQRMCICHLPCR | | | Carbamidomethyl (C)[11,13] | | Mascot |
| 2486.1504 | 2486.1956 | 0.0452 | 18 | 176 | 197 | HINKGETEASYSGNLDAYEIMK | | | Oxidation (M)[21] | | Mascot |
| 2501.293 | 2501.2251 | -0.0679 | -27 | 589 | 610 | LNASNIINESLNMLNHLYSGKR | | | | | Mascot |
| 3337.5913 | 3337.7273 | 0.136 | 41 | 449 | 474 | QLLHCQWCEERVFLKPGSMVEENLYR | | | Carbamidomethyl (C)[5,8], Oxidation (M)[19] | | Mascot |

| | | | | | | | |
|---|---|---|---|---|---|---|---|
| 35 | interleukin 4/13B [Danio rerio] | gi\|193788073 | 13301.7 | 8.6 | 6 | 34 | 0 |

**Peptide Information**

| Calc. Mass | Obsrv. Mass | ± da | ± ppm | Start Seq. | End Seq. | Sequence | Ion Score | C. I. % | Modification | Rank | Result Type |
|---|---|---|---|---|---|---|---|---|---|---|---|
| 1163.6276 | 1163.6011 | -0.0265 | -23 | 54 | 64 | KTLCQAGMALK | | | | | Mascot |
| 1179.6224 | 1179.5908 | -0.0316 | -27 | 54 | 64 | KTLCQAGMALK | | | Oxidation (M)[8] | | Mascot |
| 1371.5933 | 1371.6206 | 0.0273 | 20 | 108 | 118 | ACCHYLYSAQR | | | Carbamidomethyl (C)[2] | | Mascot |
| 1472.641 | 1472.6549 | 0.0139 | 9 | 108 | 119 | ACCHYLYSAQRT | | | Carbamidomethyl (C)[2] | | Mascot |
| 2299.269 | 2299.1592 | -0.1098 | -48 | 13 | 32 | TDEILLMEIIQSVNRILNGK | | | | | Mascot |
| 2300.0903 | 2300.1565 | 0.0662 | 29 | 36 | 54 | MDLDQFIPDIYETGHYSKK | | | | | Mascot |
| 2486.1543 | 2486.1956 | 0.0413 | 17 | 33 | 53 | GEKMDLDQFIPDIYETGH | | | | | Mascot |

YSK

| | | | | | | | | | |
|---|---|---|---|---|---|---|---|---|---|
| 36 | Zgc:171573 protein [Danio rerio] | gi\|166796220 | 28101.6 | 9.65 | 7 | 33 | 0 | | |

Peptide Information

| Calc. Mass | Obsrv. Mass | ± da | ± ppm | Start Seq. | End Seq. | Sequence | Ion Score | C. I. % | Modification | Rank | Result Type |
|---|---|---|---|---|---|---|---|---|---|---|---|
| 864.4937 | 864.4857 | -0.008 | -9 | 30 | 36 | IFSDKVR | | | | | Mascot |
| 1022.5265 | 1022.495 | -0.0315 | -31 | 27 | 34 | QERIFSDK | | | | | Mascot |
| 1235.6239 | 1235.6188 | -0.0051 | -4 | 100 | 109 | QQFQQASSRR | | | | | Mascot |
| 1456.8005 | 1456.6569 | -0.1436 | -99 | 37 | 50 | TIGVDIDALGQQVK | | | | | Mascot |
| 1625.7046 | 1625.7463 | 0.0417 | 26 | 177 | 189 | SECLWTAELSNCR | | | Carbamidomethyl (C)[3,12] | | Mascot |
| 2096.999 | 2097.1064 | 0.1074 | 51 | 65 | 83 | VYADDLLCSDGIACILEQ R | | | | | Mascot |
| 2096.999 | 2097.1064 | 0.1074 | 51 | 65 | 83 | VYADDLLCSDGIACILEQ R | | | | | Mascot |
| 2211.042 | 2211.0801 | 0.0381 | 17 | 65 | 83 | VYADDLLCSDGIACILEQ R | | | Carbamidomethyl (C)[8,14] | | Mascot |
| 2486.0745 | 2486.1956 | 0.1211 | 49 | 169 | 189 | QEENNMTKSECLWTAEL SNCR | | | | | Mascot |

| | | | | | | | | | |
|---|---|---|---|---|---|---|---|---|---|
| 37 | PREDICTED: hypothetical protein LOC567610 [Danio rerio] | gi\|189529058 | 18686.6 | 9.77 | 6 | 33 | 0 | | |

Peptide Information

| Calc. Mass | Obsrv. Mass | ± da | ± ppm | Start Seq. | End Seq. | Sequence | Ion Score | C. I. % | Modification | Rank | Result Type |
|---|---|---|---|---|---|---|---|---|---|---|---|
| 875.5131 | 875.4556 | -0.0575 | -66 | 2 | 8 | NTKIMLR | | | | | Mascot |
| 970.4476 | 970.4955 | 0.0479 | 49 | 158 | 165 | ESSYEVTR | | | | | Mascot |
| 1022.5485 | 1022.495 | -0.0535 | -52 | 1 | 8 | MNTKIMLR | | | Oxidation (M)[1] | | Mascot |
| 1312.6704 | 1312.6016 | -0.0688 | -52 | 93 | 104 | TSTTQRTTTTSK | | | | | Mascot |
| 2102.2478 | 2101.9805 | -0.2673 | -127 | 126 | 146 | VGLVIAAILFVMGILVLGC GK | | | Oxidation (M)[12] | | Mascot |
| 2299.3755 | 2299.1592 | -0.2163 | -94 | 125 | 146 | RVGLVIAAILFVMGILVLG CGK | | | Carbamidomethyl (C)[20] | | Mascot |

| | | | | | | | | | |
|---|---|---|---|---|---|---|---|---|---|
| 38 | polymerase I and transcript release factor isoform 1 [Danio rerio] | gi\|147898795 | 19874.3 | 5.41 | 1 | 33 | 0 | 29 | 69.543 |

Peptide Information

| Calc. Mass | Obsrv. Mass | ± da | ± ppm | Start Seq. | End Seq. | Sequence | Ion Score | C. I. % | Modification | Rank | Result Type |
|---|---|---|---|---|---|---|---|---|---|---|---|
| 987.5945 | 987.524 | -0.0705 | -71 | 128 | 136 | VSVNVKSVR | 29 | 69.543 | | | Mascot |
| 987.5945 | 987.524 | -0.0705 | -71 | 128 | 136 | VSVNVKSVR | | | | | Mascot |

| | | | | | | | | | |
|---|---|---|---|---|---|---|---|---|---|
| 39 | hypothetical protein LOC553738 [Danio rerio] | gi\|66472320 | 27546.3 | 9.57 | 9 | 33 | 0 | | |

Peptide Information

| Calc. Mass | Obsrv. Mass | ± da | ± ppm | Start Seq. | End Seq. | Sequence | Ion Score | C. I. % | Modification | Rank | Result Type |
|---|---|---|---|---|---|---|---|---|---|---|---|
| 853.4413 | 853.3546 | -0.0867 | -102 | 223 | 230 | FSQSGSLK | | | | | Mascot |
| 1165.6212 | 1165.7299 | 0.1087 | 93 | 194 | 203 | TFVTADDLKR | | | | | Mascot |
| 1165.6212 | 1165.7299 | 0.1087 | 93 | 194 | 203 | TFVTADDLKR | | | | | Mascot |
| 1179.615 | 1179.5908 | -0.0242 | -21 | 166 | 175 | SCTRQSSLIK | | | Carbamidomethyl (C)[2] | | Mascot |
| 1865.9031 | 1865.8218 | -0.0813 | -44 | 10 | 25 | MVRTGEKPITCTQCGK | | | Carbamidomethyl (C)[11,14] | | Mascot |
| 1865.9031 | 1865.8218 | -0.0813 | -44 | 10 | 25 | MVRTGEKPITCTQCGK | | | Carbamidomethyl (C)[11,14] | | Mascot |
| 1961.8779 | 1961.97 | 0.0921 | 47 | 207 | 222 | IHTGEKPYMCSHCDKR | | | Carbamidomethyl (C)[10] | | Mascot |
| 2233.0752 | 2233.063 | -0.0122 | -5 | 147 | 165 | FHLRVHTNERPYSCSVCGK | | | | | Mascot |
| 2284.0168 | 2284.1504 | 0.1336 | 58 | 204 | 221 | HQRIHTGEKPYMCSHCDK | | | Carbamidomethyl (C)[13,16] | | Mascot |
| 2298.0171 | 2298.1658 | 0.1487 | 65 | 151 | 169 | VHTNERPYSCSVCGKSCTR | | | Carbamidomethyl (C)[10,13,17] | | Mascot |
| 2300.0117 | 2300.1565 | 0.1448 | 63 | 204 | 221 | HQRIHTGEKPYMCSHCDK | | | Carbamidomethyl (C)[13,16], Oxidation (M)[12] | | Mascot |
| 2486.1763 | 2486.1956 | 0.0193 | 8 | 13 | 35 | TGEKPITCTQCGKSFSQSSNLNR | | | | | Mascot |

40 extended synaptotagmin-3 [Danio rerio] gi|181339841 98050.5 7.91 13 33 0

**Peptide Information**

| Calc. Mass | Obsrv. Mass | ± da | ± ppm | Start Seq. | End Seq. | Sequence | Ion Score | C. I. % | Modification | Rank | Result Type |
|---|---|---|---|---|---|---|---|---|---|---|---|
| 856.4159 | 856.5167 | 0.1008 | 118 | 460 | 466 | EVTHNEK | | | | | Mascot |
| 1045.4077 | 1045.5532 | 0.1455 | 139 | 389 | 397 | FSMDCGDVR | | | Oxidation (M)[3] | | Mascot |
| 1272.6881 | 1272.7003 | 0.0122 | 10 | 292 | 301 | VEQMRFPLPR | | | | | Mascot |
| 1294.65 | 1294.5137 | -0.1363 | -105 | 766 | 775 | MYLLPDQTWK | | | | | Mascot |
| 1412.8406 | 1412.6305 | -0.2101 | -149 | 206 | 218 | VGIKGLQLQGMLR | | | | | Mascot |
| 1587.8101 | 1587.8649 | 0.0548 | 35 | 766 | 777 | MYLLPDQTWKHR | | | | | Mascot |
| 1587.8101 | 1587.8649 | 0.0548 | 35 | 766 | 777 | MYLLPDQTWKHR | | | | | Mascot |
| 1865.9069 | 1865.8218 | -0.0851 | -46 | 405 | 419 | WYTLEDIESGQIHFK | | | | | Mascot |
| 1865.9069 | 1865.8218 | -0.0851 | -46 | 405 | 419 | WYTLEDIESGQIHFK | | | | | Mascot |
| 2194.1211 | 2194.0774 | -0.0437 | -20 | 589 | 608 | VVTSAPQDKNTSTPNRPEPR | | | | | Mascot |
| 2233.2009 | 2233.063 | -0.1379 | -62 | 541 | 559 | FSLPLVRLLNVSDMTLDQR | | | Oxidation (M)[14] | | Mascot |
| 2299.1201 | 2299.1592 | 0.0391 | 17 | 480 | 499 | TNNPNSYVEFSIDLQSQKSK | | | | | Mascot |
| 2300.1082 | 2300.1565 | 0.0483 | 21 | 786 | 805 | TVNPVFDETFEFAVSLEEAR | | | | | Mascot |
| 2472.1455 | 2472.1626 | 0.0171 | 7 | 434 | 456 | ETSDGLACAMLALYLDCASNLPK | | | Carbamidomethyl (C)[8], Oxidation (M)[10] | | Mascot |
| 2486.1995 | 2486.1956 | -0.0039 | -2 | 98 | 118 | AMDMPPWIHFADVEKAAWINK | | | Oxidation (M)[2] | | Mascot |

| | | | | | | | |
|---|---|---|---|---|---|---|---|
| 41 | PREDICTED: NACHT, LRR and PYD domains-containing protein 3-like [Danio rerio] | gi\|292610040 | 106687 | 6.59 | 11 | 32 | 0 |

Peptide Information

| Calc. Mass | Obsrv. Mass | ± da | ± ppm | Start Seq. | End Seq. | Sequence | Ion Score | C. I. % | Modification | Rank | Result Type |
|---|---|---|---|---|---|---|---|---|---|---|---|
| 970.4741 | 970.4955 | 0.0214 | 22 | 374 | 380 | HEKHYEK | | | | | Mascot |
| 987.5581 | 987.524 | -0.0341 | -35 | 496 | 504 | AVDKALQSR | 10 | 0 | | | Mascot |
| 987.5581 | 987.524 | -0.0341 | -35 | 496 | 504 | AVDKALQSR | | | | | Mascot |
| 1022.5815 | 1022.495 | -0.0865 | -85 | 394 | 401 | LAFRQLMK | | | Oxidation (M)[7] | | Mascot |
| 1179.6732 | 1179.5908 | -0.0824 | -70 | 311 | 320 | LADEIIQHLK | | | | | Mascot |
| 1475.7351 | 1475.7354 | 0.0003 | 0 | 939 | 950 | LETLHISWGFIC | | | Carbamidomethyl (C)[12] | | Mascot |
| 1625.7332 | 1625.7463 | 0.0131 | 8 | 715 | 731 | CCGVSDEGCAALASVLK | | | | | Mascot |
| 1913.014 | 1912.9598 | -0.0542 | -28 | 46 | 62 | GGIDPGFSHRKPLQTFR | | | | | Mascot |
| 1951.9397 | 1951.9854 | 0.0457 | 23 | 713 | 731 | LRCCGVSDEGCAALASV LK | | | Carbamidomethyl (C)[3] | | Mascot |
| 2083.0894 | 2082.9888 | -0.1006 | -48 | 357 | 373 | TLTEMYTHFLIIQTSIR | | | Oxidation (M)[5] | | Mascot |
| 2102.1282 | 2101.9805 | -0.1477 | -70 | 190 | 207 | DRTLSLADLLHVFFPETK | | | | | Mascot |
| 2211.0513 | 2211.0801 | 0.0288 | 13 | 478 | 495 | STRPHEFTCHTVFDLHQ R | | | | | Mascot |

| | | | | | | | |
|---|---|---|---|---|---|---|---|
| 42 | novel protein similar to Janus kinase family (a protein tyrosine kinase, leukocyte) [Danio rerio] | gi\|169145296 | 125997.7 | 6.83 | 15 | 32 | 0 |

Peptide Information

| Calc. Mass | Obsrv. Mass | ± da | ± ppm | Start Seq. | End Seq. | Sequence | Ion Score | C. I. % | Modification | Rank | Result Type |
|---|---|---|---|---|---|---|---|---|---|---|---|
| 850.4603 | 850.5328 | 0.0725 | 85 | 399 | 405 | NGMFLLR | | | | | Mascot |
| 850.4603 | 850.5328 | 0.0725 | 85 | 399 | 405 | NGMFLLR | | | | | Mascot |
| 856.3981 | 856.5167 | 0.1186 | 138 | 882 | 889 | GICYSTGR | | | | | Mascot |
| 864.4825 | 864.4857 | 0.0032 | 4 | 959 | 966 | IADFGLTK | | | | | Mascot |
| 987.4894 | 987.524 | 0.0346 | 35 | 406 | 413 | HSPKEFDK | | | | | Mascot |
| 987.4894 | 987.524 | 0.0346 | 35 | 406 | 413 | HSPKEFDK | | | | | Mascot |
| 1007.4938 | 1007.4681 | -0.0257 | -26 | 228 | 236 | CSAGERSLK | | | Carbamidomethyl (C)[1] | | Mascot |
| 1100.5959 | 1100.5676 | -0.0283 | -26 | 939 | 947 | YVHRDLAAR | | | | | Mascot |
| 1163.6354 | 1163.6011 | -0.0343 | -29 | 395 | 405 | AGGKNGMFLLR | | | | | Mascot |
| 1179.6302 | 1179.5908 | -0.0394 | -33 | 395 | 405 | AGGKNGMFLLR | | | Oxidation (M)[7] | | Mascot |
| 1475.7094 | 1475.7354 | 0.026 | 18 | 308 | 319 | RLCQEQMPLEGR | | | Oxidation (M)[7] | | Mascot |
| 1625.8025 | 1625.7463 | -0.0562 | -35 | 616 | 629 | SMCVSVSWKLDVAK | | | Carbamidomethyl (C)[3], Oxidation (M)[2] | | Mascot |
| 1962.0364 | 1961.97 | -0.0664 | -34 | 625 | 641 | LDVAKQLACALNFLEEK | | | Carbamidomethyl (C)[9] | | Mascot |
| 2097.0684 | 2097.1064 | 0.038 | 18 | 865 | 881 | EIQTISSLHCDYIVKYK | | | Carbamidomethyl (C)[10] | | Mascot |

| | | | | | | | | | | | |
|---|---|---|---|---|---|---|---|---|---|---|---|
| 2097.0684 | 2097.1064 | 0.038 | 18 | 865 | 881 | EIQTISSLHCDYIVKYK | | | Carbamidomethyl (C)[10] | | Mascot |
| 2211.0791 | 2211.0801 | 0.001 | 0 | 290 | 307 | QEWQTFCDFPQIIDISIK | | | | | Mascot |
| 2225.0115 | 2225.0945 | 0.083 | 37 | 564 | 582 | NCWESLFEAASLMSQIS HR | | | Oxidation (M)[13] | | Mascot |
| 2225.0115 | 2225.0945 | 0.083 | 37 | 564 | 582 | NCWESLFEAASLMSQIS HR | | | Oxidation (M)[13] | | Mascot |
| 2247.1333 | 2247.0789 | -0.0544 | -24 | 920 | 938 | LLLFASQICKGMEYLQSM R | | | Oxidation (M)[12] | | Mascot |
| 2299.1614 | 2299.1592 | -0.0022 | -1 | 894 | 912 | LVMEYLPYGSLIGYMEKH R | | | | | Mascot |

43 SH3 domain-binding protein 4-A [Danio rerio] gi|45387913 104947.5 7.73 13 32 0

Peptide Information

| Calc. Mass | Obsrv. Mass | ± da | ± ppm | Start Seq. | End Seq. | Sequence | Ion Score | C. I. % | Modification | Rank | Result Type |
|---|---|---|---|---|---|---|---|---|---|---|---|
| 842.5822 | 842.5021 | -0.0801 | -95 | 634 | 640 | LIKTVIR | | | | | Mascot |
| 850.4338 | 850.5328 | 0.099 | 116 | 358 | 364 | LSNMEIK | | | Oxidation (M)[4] | | Mascot |
| 850.4338 | 850.5328 | 0.099 | 116 | 358 | 364 | LSNMEIK | | | Oxidation (M)[4] | | Mascot |
| 870.4726 | 870.532 | 0.0594 | 68 | 1 | 7 | MAAHRIR | | | Oxidation (M)[1] | | Mascot |
| 1045.4796 | 1045.5532 | 0.0736 | 70 | 762 | 770 | AELDSEPER | | | | | Mascot |
| 1272.7522 | 1272.7003 | -0.0519 | -41 | 476 | 487 | TLLVSEVGKQAK | | | | | Mascot |
| 1294.6637 | 1294.5137 | -0.15 | -116 | 553 | 563 | KADEISDFTLR | | | | | Mascot |
| 1371.7161 | 1371.6206 | -0.0955 | -70 | 8 | 19 | VTNNNNVLPRCK | | | | | Mascot |
| 1412.6031 | 1412.6305 | 0.0274 | 19 | 780 | 790 | EDCLNMETKEK | | | Carbamidomethyl (C)[3], Oxidation (M)[6] | | Mascot |
| 1815.8959 | 1815.87 | -0.0259 | -14 | 432 | 446 | HVSFSSNVWNHMVKK | | | Oxidation (M)[12] | | Mascot |
| 2097.0137 | 2097.1064 | 0.0927 | 44 | 373 | 390 | VSVMTRTESPMAEIVCVR | | | Carbamidomethyl (C)[16], Oxidation (M)[4,11] | | Mascot |
| 2097.0137 | 2097.1064 | 0.0927 | 44 | 373 | 390 | VSVMTRTESPMAEIVCVR | | | Carbamidomethyl (C)[16], Oxidation (M)[4,11] | | Mascot |
| 2284.2368 | 2284.1504 | -0.0864 | -38 | 463 | 484 | TVVAIFGHDCAPKTLLVS EVGK | | | | | Mascot |
| 2300.0686 | 2300.1565 | 0.0879 | 38 | 568 | 587 | DDQDCILAQFCVQTPQP PPK | | | Carbamidomethyl (C)[5] | | Mascot |
| 3337.6052 | 3337.7273 | 0.1221 | 37 | 269 | 298 | LARSCHDLESLGQNPGW GQTQPVETNIVCK | | | Carbamidomethyl (C)[5] | | Mascot |

44 PREDICTED: RNA-directed DNA polymerase from mobile element jockey-like [Danio rerio] gi|326667425 118942.6 9.57 15 32 0

Peptide Information

| Calc. Mass | Obsrv. Mass | ± da | ± ppm | Start Seq. | End Seq. | Sequence | Ion Score | C. I. % | Modification | Rank | Result Type |
|---|---|---|---|---|---|---|---|---|---|---|---|
| 864.3628 | 864.4857 | 0.1229 | 142 | 168 | 174 | MNENGQR | | | Oxidation (M)[1] | | Mascot |
| 870.4402 | 870.532 | 0.0918 | 105 | 916 | 922 | WLGHVCR | | | | | Mascot |
| 987.5945 | 987.524 | -0.0705 | -71 | 817 | 826 | IGKAASTLAR | 6 | 0 | | | Mascot |
| 987.5945 | 987.524 | -0.0705 | -71 | 817 | 826 | IGKAASTLAR | | | | | Mascot |

| | | | | | | | | | | | |
|---|---|---|---|---|---|---|---|---|---|---|---|
| 996.4819 | 996.584 | 0.1021 | 102 | 1041 | 1048 | QSVMPQYK | | | Oxidation (M)[4] | | Mascot |
| 996.4819 | 996.584 | 0.1021 | 102 | 1041 | 1048 | QSVMPQYK | | | Oxidation (M)[4] | | Mascot |
| 1100.6534 | 1100.5676 | -0.0858 | -78 | 355 | 363 | NLQILRSTR | | | | | Mascot |
| 1163.58 | 1163.6011 | 0.0211 | 18 | 330 | 340 | ASTMIPCIEAK | | | | | Mascot |
| 1179.5748 | 1179.5908 | 0.016 | 14 | 330 | 340 | ASTMIPCIEAK | | | Oxidation (M)[4] | | Mascot |
| 1235.548 | 1235.6188 | 0.0708 | 57 | 50 | 58 | DYTFYWQGR | | | | | Mascot |
| 1279.6892 | 1279.7589 | 0.0697 | 54 | 485 | 497 | APGSDGIPPDLLK | | | | | Mascot |
| 1475.7787 | 1475.7354 | -0.0433 | -29 | 498 | 509 | QCKDSLLHPLHK | | | Carbamidomethyl (C)[2] | | Mascot |
| 1638.8519 | 1638.8395 | -0.0124 | -8 | 485 | 500 | APGSDGIPPDLLKQCK | | | | | Mascot |
| 1961.8513 | 1961.97 | 0.1187 | 61 | 1007 | 1024 | VQSVSQGATYQCDCCG KK | | | Carbamidomethyl (C)[12] | | Mascot |
| 2101.9858 | 2101.9805 | -0.0053 | -3 | 653 | 672 | GTIQFNGSCSEPFSISSG VK | | | Carbamidomethyl (C)[9] | | Mascot |
| 2151.9863 | 2152.0684 | 0.0821 | 38 | 286 | 304 | ELDTAQTGDSALEMWES LR | | | | | Mascot |
| 2194.0308 | 2194.0774 | 0.0466 | 21 | 322 | 340 | THDWFEAKASTMIPCIEA K | | | Oxidation (M)[12] | | Mascot |
| 2233.0364 | 2233.063 | 0.0266 | 12 | 457 | 475 | CLPTMEELDEEPTAEELR K | | | | | Mascot |

45 60S ribosomal protein L28 [Danio rerio] gi|41055030 15629.8 12.11 6 32 0

**Peptide Information**

| Calc. Mass | Obsrv. Mass | ± da | ± ppm | Start Seq. | End Seq. | Sequence | Ion Score | C. I. % | Modification | Rank | Result Type |
|---|---|---|---|---|---|---|---|---|---|---|---|
| 856.4787 | 856.5167 | 0.038 | 44 | 41 | 47 | FNGLIHR | | | | | Mascot |
| 1051.5895 | 1051.7001 | 0.1106 | 105 | 13 | 21 | NSSSFLIKR | | | | | Mascot |
| 1051.5895 | 1051.7001 | 0.1106 | 105 | 13 | 21 | NSSSFLIKR | | | | | Mascot |
| 1199.663 | 1199.5568 | -0.1062 | -89 | 48 | 59 | KTIGIEPAADGK | | | | | Mascot |
| 1279.6753 | 1279.7589 | 0.0836 | 65 | 69 | 80 | SGQRKPATSYGK | | | | | Mascot |
| 1294.6273 | 1294.5137 | -0.1136 | -88 | 24 | 34 | QTYSTEPNNLK | | | | | Mascot |
| 1639.0013 | 1638.8395 | -0.1618 | -99 | 114 | 128 | RVSAILSSQKPVVVR | | | | | Mascot |

46 PREDICTED: fibroblast growth factor receptor substrate 2-like [Danio rerio] gi|326668781 70183.2 5.85 10 32 0

**Peptide Information**

| Calc. Mass | Obsrv. Mass | ± da | ± ppm | Start Seq. | End Seq. | Sequence | Ion Score | C. I. % | Modification | Rank | Result Type |
|---|---|---|---|---|---|---|---|---|---|---|---|
| 970.4999 | 970.4955 | -0.0044 | -5 | 394 | 402 | GMVAAGHRR | | | Oxidation (M)[2] | | Mascot |
| 1007.5131 | 1007.4681 | -0.045 | -45 | 57 | 63 | WPYLCLR | | | Carbamidomethyl (C)[5] | | Mascot |
| 1100.5636 | 1100.5676 | 0.004 | 4 | 509 | 516 | VFNFDFRR | | | | | Mascot |
| 1163.6143 | 1163.6011 | -0.0132 | -11 | 57 | 64 | WPYLCLRR | | | Carbamidomethyl (C)[5] | | Mascot |

| Calc. Mass | Obsrv. Mass | ± da | ± ppm | Start Seq. | End Seq. | Sequence | Ion Score | C. I. % | Modification | Rank | Result Type |
|---|---|---|---|---|---|---|---|---|---|---|---|
| 1199.5878 | 1199.5568 | -0.031 | -26 | 80 | 90 | CQTGQGIFAFK | | | | | Mascot |
| 1205.49 | 1205.6239 | 0.1339 | 111 | 1 | 10 | MGSCWSCLYR | | | | | Mascot |
| 1412.7103 | 1412.6305 | -0.0798 | -56 | 79 | 90 | RCQTGQGIFAFK | | | Carbamidomethyl (C)[2] | | Mascot |
| 1815.8993 | 1815.87 | -0.0293 | -16 | 256 | 270 | FMLGPTPAQRHLMER | | | Oxidation (M)[2,13] | | Mascot |
| 2225.1672 | 2225.0945 | -0.0727 | -33 | 475 | 493 | DALQNYVNTEQLPPPTRLR | | | | | Mascot |
| 2225.1672 | 2225.0945 | -0.0727 | -33 | 475 | 493 | DALQNYVNTEQLPPPTRLR | | | | | Mascot |
| 2472.1282 | 2472.1626 | 0.0344 | 14 | 315 | 335 | HPSMEPCPESQLTYENINGLR | | | Carbamidomethyl (C)[7] | | Mascot |

| | | | | | | | |
|---|---|---|---|---|---|---|---|
| 47 | isocitrate dehydrogenase [NADP], mitochondrial [Danio rerio] | gi\|41054651 | 50364.5 | 8.35 | 9 | 32 | 0 |

**Peptide Information**

| Calc. Mass | Obsrv. Mass | ± da | ± ppm | Start Seq. | End Seq. | Sequence | Ion Score | C. I. % | Modification | Rank | Result Type |
|---|---|---|---|---|---|---|---|---|---|---|---|
| 856.4311 | 856.5167 | 0.0856 | 100 | 255 | 261 | AYDGRFK | | | | | Mascot |
| 1205.6096 | 1205.6239 | 0.0143 | 12 | 129 | 138 | MWKSPNGTIR | | | Oxidation (M)[1] | | Mascot |
| 1294.646 | 1294.5137 | -0.1323 | -102 | 192 | 203 | MVFTPADGSKNK | | | | | Mascot |
| 1371.6508 | 1371.6206 | -0.0302 | -22 | 412 | 424 | DLAGCIHGLANCK | | | Carbamidomethyl (C)[5] | | Mascot |
| 1625.8428 | 1625.7463 | -0.0965 | -59 | 21 | 35 | SPAVLAPACQSLQQR | | | Carbamidomethyl (C)[9] | | Mascot |
| 1874.9872 | 1874.9208 | -0.0664 | -35 | 359 | 375 | GRPTSTNPIASIFAWTR | | | | | Mascot |
| 2284.2078 | 2284.1504 | -0.0574 | -25 | 14 | 35 | SAATLSKSPAVLAPACQSLQQR | | | Carbamidomethyl (C)[16] | | Mascot |
| 2297.1667 | 2297.1614 | -0.0053 | -2 | 287 | 307 | LIDDMVAQVLKSSGAFVWACK | | | Oxidation (M)[5] | | Mascot |
| 2300.126 | 2300.1565 | 0.0305 | 13 | 392 | 411 | FSQTLERVCVETVESGVMTK | | | Carbamidomethyl (C)[9] | | Mascot |

| | | | | | | | |
|---|---|---|---|---|---|---|---|
| 48 | Tlk1a protein [Danio rerio] | gi\|156230424 | 38213.5 | 9.84 | 8 | 32 | 0 |

**Peptide Information**

| Calc. Mass | Obsrv. Mass | ± da | ± ppm | Start Seq. | End Seq. | Sequence | Ion Score | C. I. % | Modification | Rank | Result Type |
|---|---|---|---|---|---|---|---|---|---|---|---|
| 875.4832 | 875.4556 | -0.0276 | -32 | 184 | 191 | LATIESNK | | | | | Mascot |
| 1199.7007 | 1199.5568 | -0.1439 | -120 | 261 | 270 | LRLGHFTTVR | | | | | Mascot |
| 1371.7478 | 1371.6206 | -0.1272 | -93 | 215 | 225 | QIDEQQKLLEK | | | | | Mascot |
| 1587.8588 | 1587.8649 | 0.0061 | 4 | 184 | 197 | LATIESNKNLDLEK | | | | | Mascot |
| 1587.8588 | 1587.8649 | 0.0061 | 4 | 184 | 197 | LATIESNKNLDLEK | | | | | Mascot |
| 1815.8719 | 1815.87 | -0.0019 | -1 | 313 | 330 | KPPSASSSQSPSTNSEPK | | | | | Mascot |
| 1994.0474 | 1993.9501 | -0.0973 | -49 | 174 | 191 | SVQTDLTVMKLATIESNK | | | Oxidation (M)[9] | | Mascot |
| 2299.0779 | 2299.1592 | 0.0813 | 35 | 271 | 290 | HGASFTEQWTDGYAFQNLVK | | | | | Mascot |
| 2317.0081 | 2317.1436 | 0.1355 | 58 | 2 | 22 | MAGSPMEQMGAMEELH | | | | | Mascot |

SLDPR

| 49 | PREDICTED: hypothetical protein LOC100538026 [Danio rerio] | gi\|326673594 | 14275.3 | 8.81 | 6 | 32 | 0 |
|---|---|---|---|---|---|---|---|

Peptide Information

| Calc. Mass | Obsrv. Mass | ± da | ± ppm | Start Seq. | End Seq. | Sequence | Ion Score | C. I. % | Modification | Rank | Result Type |
|---|---|---|---|---|---|---|---|---|---|---|---|
| 1163.6276 | 1163.6011 | -0.0265 | -23 | 62 | 72 | KTLCQAGMALK | | | | | Mascot |
| 1179.6224 | 1179.5908 | -0.0316 | -27 | 62 | 72 | KTLCQAGMALK | | | Oxidation (M)[8] | | Mascot |
| 1371.5933 | 1371.6206 | 0.0273 | 20 | 116 | 126 | ACCHYLYSAQR | | | Carbamidomethyl (C)[2] | | Mascot |
| 1472.641 | 1472.6549 | 0.0139 | 9 | 116 | 127 | ACCHYLYSAQRT | | | Carbamidomethyl (C)[2] | | Mascot |
| 2299.269 | 2299.1592 | -0.1098 | -48 | 21 | 40 | TDEILLMEIIQSVNRILNGK | | | | | Mascot |
| 2300.0903 | 2300.1565 | 0.0662 | 29 | 44 | 62 | MDLDQFIPDIYETGHYSK K | | | | | Mascot |
| 2486.1543 | 2486.1956 | 0.0413 | 17 | 41 | 61 | GEKMDLDQFIPDIYETGH YSK | | | | | Mascot |

| 50 | PREDICTED: interferon-induced very large GTPase 1-like [Danio rerio] | gi\|326676497 | 194760.8 | 7 | 20 | 31 | 0 |
|---|---|---|---|---|---|---|---|

Peptide Information

| Calc. Mass | Obsrv. Mass | ± da | ± ppm | Start Seq. | End Seq. | Sequence | Ion Score | C. I. % | Modification | Rank | Result Type |
|---|---|---|---|---|---|---|---|---|---|---|---|
| 864.4178 | 864.4857 | 0.0679 | 79 | 551 | 556 | EMCRLR | | | Carbamidomethyl (C)[3] | | Mascot |
| 962.5153 | 962.5256 | 0.0103 | 11 | 77 | 85 | NEATSVTLK | | | | | Mascot |
| 987.4781 | 987.524 | 0.0459 | 46 | 1420 | 1427 | NYESFVTK | | | | | Mascot |
| 987.4781 | 987.524 | 0.0459 | 46 | 1420 | 1427 | NYESFVTK | | | | | Mascot |
| 1100.5769 | 1100.5676 | -0.0093 | -8 | 1185 | 1193 | LLHSNLEMK | | | Oxidation (M)[8] | | Mascot |
| 1163.5361 | 1163.6011 | 0.065 | 56 | 1049 | 1058 | CEADNELGKK | | | Carbamidomethyl (C)[1] | | Mascot |
| 1205.6447 | 1205.6239 | -0.0208 | -17 | 473 | 482 | LQMTVDEILK | | | Oxidation (M)[3] | | Mascot |
| 1235.6276 | 1235.6188 | -0.0088 | -7 | 274 | 283 | ECTLMLWALR | | | | | Mascot |
| 1279.6827 | 1279.7589 | 0.0762 | 60 | 1478 | 1488 | FVTNVCKNLNK | | | | | Mascot |
| 1371.7777 | 1371.6206 | -0.1571 | -115 | 1132 | 1142 | EKVLNLIQVCR | | | Carbamidomethyl (C)[10] | | Mascot |
| 1412.6362 | 1412.6305 | -0.0057 | -4 | 461 | 472 | DVKMNDDDFVAK | | | Oxidation (M)[4] | | Mascot |
| 1456.6486 | 1456.6569 | 0.0083 | 6 | 187 | 198 | RMSDENQAQSFK | | | Oxidation (M)[2] | | Mascot |
| 1475.7522 | 1475.7354 | -0.0168 | -11 | 428 | 440 | LVLVCNTNDLESR | | | | | Mascot |
| 1815.8768 | 1815.87 | -0.0068 | -4 | 881 | 896 | MSFFQQLDVMTQAAAK | | | | | Mascot |
| 1912.9507 | 1912.9598 | 0.0091 | 5 | 1601 | 1617 | LIADICSSLVASEMTFR | | | Carbamidomethyl (C)[6] | | Mascot |
| 2097.0657 | 2097.1064 | 0.0407 | 19 | 646 | 663 | KLENHIANSSLGVQHFMR | | | Oxidation (M)[17] | | Mascot |
| 2097.0657 | 2097.1064 | 0.0407 | 19 | 646 | 663 | KLENHIANSSLGVQHFMR | | | Oxidation (M)[17] | | Mascot |
| 2225.0828 | 2225.0945 | 0.0117 | 5 | 464 | 482 | MNDDDFVAKLQMTVDEIL | | | | | Mascot |

| | | | | | | | | | | | |
|---|---|---|---|---|---|---|---|---|---|---|---|
| | | | | | | K | | | | | |
| 2225.0828 | 2225.0945 | 0.0117 | 5 | 464 | 482 | MNDDDFVAKLQMTVDEIL K | | | | | Mascot |
| 2284.0913 | 2284.1504 | 0.0591 | 26 | 297 | 316 | DCSGENSIVQTDFPLISF VR | | | Carbamidomethyl (C)[2] | | Mascot |
| 2297.0974 | 2297.1614 | 0.064 | 28 | 156 | 174 | TNINMEYMLMELGLQSHL K | | | Oxidation (M)[5,8] | | Mascot |
| 2300.0759 | 2300.1565 | 0.0806 | 35 | 356 | 375 | ICNGLVEMTWYLPCGKE TDK | | | | | Mascot |
| 2486.1755 | 2486.1956 | 0.0201 | 8 | 1262 | 1282 | TEPKSDYNSADIQELLCFI DK | | | Carbamidomethyl (C)[17] | | Mascot |

| Gel Idx/Pos | 180/H7 | Instr./Gel Origin | BA2060/110707A | Process Status | Analysis Succeeded |
|---|---|---|---|---|---|
| Plate [#] Name | [1] 31496 | Instrument Sample Name | | Spectra | 11 |

| Rank | Protein Name | Accession No. | Protein MW | Protein PI | Pep. Count | Protein Score | Protein Score C. I. % | Total Ion Score | Total Ion C. I. % |
|---|---|---|---|---|---|---|---|---|---|
| 1 | gelsolin b [Danio rerio] | gi\|148230943 | 80133.8 | 5.62 | 16 | 469 | 100 | 398 | 100 |

Peptide Information

| Calc. Mass | Obsrv. Mass | ± da | ± ppm | Start Seq. | End Seq. | Sequence | Ion Score | C. I. % | Modification | Rank | Result Type |
|---|---|---|---|---|---|---|---|---|---|---|---|
| 823.4169 | 823.399 | -0.0179 | -22 | 517 | 524 | ANPAGHTR | | | | | Mascot |
| 853.3621 | 853.3448 | -0.0173 | -20 | 179 | 184 | CNQFER | | | Carbamidomethyl (C)[1] | | Mascot |
| 875.4661 | 875.4468 | -0.0193 | -22 | 104 | 110 | TFLGYFK | | | | | Mascot |
| 987.537 | 987.5167 | -0.0203 | -21 | 13 | 20 | QTGLQVWR | 52 | 99.834 | | | Mascot |
| 987.537 | 987.5167 | -0.0203 | -21 | 13 | 20 | QTGLQVWR | | | | | Mascot |
| 1007.4792 | 1007.4591 | -0.0201 | -20 | 676 | 684 | YIESDPAGR | | | | | Mascot |
| 1022.5053 | 1022.4899 | -0.0154 | -15 | 2 | 9 | VHHPEFEK | | | | | Mascot |
| 1294.5262 | 1294.5033 | -0.0229 | -18 | 193 | 202 | DIRDNECCGR | | | Carbamidomethyl (C)[7,8] | | Mascot |
| 1456.6777 | 1456.6481 | -0.0296 | -20 | 308 | 320 | AAEDFISQMGYPK | | | | | Mascot |
| 1472.6726 | 1472.6449 | -0.0277 | -19 | 308 | 320 | AAEDFISQMGYPK | 44 | 98.921 | Oxidation (M)[9] | | Mascot |
| 1472.6726 | 1472.6449 | -0.0277 | -19 | 308 | 320 | AAEDFISQMGYPK | | | Oxidation (M)[9] | | Mascot |
| 1578.6522 | 1578.7957 | 0.1435 | 91 | 377 | 391 | SEAMAAQHGMVDDGK | | | Oxidation (M)[4,10] | | Mascot |
| 1625.7476 | 1625.7368 | -0.0108 | -7 | 203 | 216 | AKLFVCEEGSENDK | | | Carbamidomethyl (C)[6] | | Mascot |
| 1952.0123 | 1951.9774 | -0.0349 | -18 | 321 | 337 | HTEVQIIPENGETPLFK | | | | | Mascot |
| 2097.1299 | 2097.0923 | -0.0376 | -18 | 451 | 472 | GASAILAAQLDAELGGSA VQVR | 210 | 100 | | | Mascot |
| 2097.1299 | 2097.0923 | -0.0376 | -18 | 451 | 472 | GASAILAAQLDAELGGSA VQVR | | | | | Mascot |
| 2121.1438 | 2121.1003 | -0.0435 | -21 | 217 | 236 | ILAILGPKPDLPDAQSEDT K | | | | | Mascot |
| 2152.0959 | 2152.0601 | -0.0358 | -17 | 32 | 50 | NLYGGFYTGDTYLVLNTI K | | | | | Mascot |
| 2152.0959 | 2152.0601 | -0.0358 | -17 | 32 | 50 | NLYGGFYTGDTYLVLNTI K | 92 | 100 | | | Mascot |

| | | | | | | | | | | | |
|---|---|---|---|---|---|---|---|---|---|---|---|
| 2155.96 | 2155.9233 | -0.0367 | -17 | 433 | 450 | QGQIIYMWQGEESSQDE K | | | | | Mascot |
| 3387.667 | 3387.6663 | -0.0007 | 0 | 399 | 428 | IEGSDKVPVDPSIYGQFY GGDSYIILYTYK | | | | | Mascot |

2 acylamino-acid-releasing enzyme [Danio rerio] gi|38564417 81411.5 5.72 8 89 99.994 78 100

**Peptide Information**

| Calc. Mass | Obsrv. Mass | ± da | ± ppm | Start Seq. | End Seq. | Sequence | Ion Score | C. I. % | Modification | Rank | Result Type |
|---|---|---|---|---|---|---|---|---|---|---|---|
| 1019.5554 | 1019.5233 | -0.0321 | -31 | 129 | 137 | CLNLTTLNK | | | | | Mascot |
| 1047.5316 | 1047.5 | -0.0316 | -30 | 107 | 115 | ENTIKGEEK | | | | | Mascot |
| 1587.8853 | 1587.8539 | -0.0314 | -20 | 5 | 17 | VLRDPVEISQLYR | | | | | Mascot |
| 1625.7588 | 1625.7368 | -0.022 | -14 | 74 | 88 | SVVYSGPSGNCTEIR | | | Carbamidomethyl (C)[11] | | Mascot |
| 1815.8911 | 1815.8549 | -0.0362 | -20 | 349 | 365 | AGDDGFTGIYSFLLSPR | | | | | Mascot |
| 1865.8453 | 1865.8141 | -0.0312 | -17 | 38 | 52 | YGGQYSNIYTEWTQR | | | | | Mascot |
| 1865.8453 | 1865.8141 | -0.0312 | -17 | 38 | 52 | YGGQYSNIYTEWTQR | 78 | 100 | | | Mascot |
| 1961.9901 | 1961.9539 | -0.0362 | -18 | 312 | 328 | IVYLESGVFGPHLQCSR | | | Carbamidomethyl (C)[15] | | Mascot |
| 2306.0581 | 2306.1123 | 0.0542 | 24 | 710 | 730 | ADAESDGFMNGALWMIQ HLSL | | | | | Mascot |

3 Acylpeptide hydrolase [Danio rerio] gi|55777111 81351.5 5.66 6 82 99.975 78 100

**Peptide Information**

| Calc. Mass | Obsrv. Mass | ± da | ± ppm | Start Seq. | End Seq. | Sequence | Ion Score | C. I. % | Modification | Rank | Result Type |
|---|---|---|---|---|---|---|---|---|---|---|---|
| 1019.5554 | 1019.5233 | -0.0321 | -31 | 129 | 137 | CLNLTTLNK | | | | | Mascot |
| 1047.5316 | 1047.5 | -0.0316 | -30 | 107 | 115 | ENTIKGEEK | | | | | Mascot |
| 1587.8853 | 1587.8539 | -0.0314 | -20 | 5 | 17 | VLRDPVEISQLYR | | | | | Mascot |
| 1625.7588 | 1625.7368 | -0.022 | -14 | 74 | 88 | SVVYSGPSGNCTEIR | | | Carbamidomethyl (C)[11] | | Mascot |
| 1865.8453 | 1865.8141 | -0.0312 | -17 | 38 | 52 | YGGQYSNIYTEWTQR | | | | | Mascot |
| 1865.8453 | 1865.8141 | -0.0312 | -17 | 38 | 52 | YGGQYSNIYTEWTQR | 78 | 100 | | | Mascot |
| 1961.9901 | 1961.9539 | -0.0362 | -18 | 312 | 328 | IVYLESGVFGPHLQCSR | | | Carbamidomethyl (C)[15] | | Mascot |

4 PREDICTED: serine/arginine repetitive matrix protein 4 [Danio rerio] gi|189518857 64253.1 11.33 13 53 76.646

**Peptide Information**

| Calc. Mass | Obsrv. Mass | ± da | ± ppm | Start Seq. | End Seq. | Sequence | Ion Score | C. I. % | Modification | Rank | Result Type |
|---|---|---|---|---|---|---|---|---|---|---|---|
| 875.458 | 875.4468 | -0.0112 | -13 | 336 | 343 | LKGEESGR | | | | | Mascot |
| 970.3716 | 970.491 | 0.1194 | 123 | 177 | 184 | SSSCMENR | | | Carbamidomethyl (C)[4] | | Mascot |
| 996.5836 | 996.5763 | -0.0073 | -7 | 59 | 67 | RLETPVGPK | | | | | Mascot |

| | | | | | | | | | | | |
|---|---|---|---|---|---|---|---|---|---|---|---|
| 996.5836 | 996.5763 | -0.0073 | -7 | 59 | 67 | RLETPVGPK | | | | | Mascot |
| 1007.5228 | 1007.4591 | -0.0637 | -63 | 377 | 385 | SRSISSQSR | | | | | Mascot |
| 1047.4346 | 1047.5 | 0.0654 | 62 | 443 | 450 | YCSSCKSR | | | Carbamidomethyl (C)[2,5] | | Mascot |
| 1279.8572 | 1279.7505 | -0.1067 | -83 | 8 | 19 | LLAGILARLLAR | | | | | Mascot |
| 1456.7166 | 1456.6481 | -0.0685 | -47 | 60 | 72 | LETPVGPKDDEEK | | | | | Mascot |
| 1467.6724 | 1467.726 | 0.0536 | 37 | 524 | 535 | SRSHSHTYSSYR | | | | | Mascot |
| 1662.9326 | 1662.7861 | -0.1465 | -88 | 498 | 511 | SSSILSWRLFPLTR | | | | | Mascot |
| 2071.8994 | 2072.0278 | 0.1284 | 62 | 218 | 235 | ISSKCCCHFSESTVSPSR | | | Carbamidomethyl (C)[5,6] | | Mascot |
| 2119.0876 | 2119.0757 | -0.0119 | -6 | 236 | 256 | SGVEGLSKVVIVQNSESS DGK | | | | | Mascot |
| 2155.9739 | 2155.9233 | -0.0506 | -23 | 259 | 279 | ADYDSGNDTSSPPSSKT GITR | | | | | Mascot |
| 2300.0105 | 2300.1404 | 0.1299 | 56 | 222 | 243 | CCCHFSESTVSPSRSGV EGLSK | | | | | Mascot |

5 PREDICTED: zinc finger protein 420 isoform 2 [Danio rerio] gi|326678086 70371 9.08 16 50 50.069

Peptide Information

| Calc. Mass | Obsrv. Mass | ± da | ± ppm | Start Seq. | End Seq. | Sequence | Ion Score | C. I. % | Modification | Rank | Result Type |
|---|---|---|---|---|---|---|---|---|---|---|---|
| 842.5206 | 842.4943 | -0.0263 | -31 | 25 | 31 | QRQGLLK | | | | | Mascot |
| 864.4798 | 864.4702 | -0.0096 | -11 | 591 | 597 | HRSVHTK | | | | | Mascot |
| 987.5621 | 987.5167 | -0.0454 | -46 | 497 | 505 | TFITAGHLK | | | | | Mascot |
| 987.5621 | 987.5167 | -0.0454 | -46 | 497 | 505 | TFITAGHLK | | | | | Mascot |
| 1007.392 | 1007.4591 | 0.0671 | 67 | 36 | 43 | MSDPEPCR | | | Carbamidomethyl (C)[7], Oxidation (M)[1] | | Mascot |
| 1019.4397 | 1019.5233 | 0.0836 | 82 | 572 | 580 | THKCDQCGK | | | | | Mascot |
| 1045.536 | 1045.5457 | 0.0097 | 9 | 134 | 141 | FHCARNLK | | | Carbamidomethyl (C)[3] | | Mascot |
| 1062.5149 | 1062.674 | 0.1591 | 150 | 110 | 117 | YNLNVHMR | | | Oxidation (M)[7] | | Mascot |
| 1578.7567 | 1578.7957 | 0.039 | 25 | 46 | 58 | QEETEELIDVMVK | | | Oxidation (M)[11] | | Mascot |
| 1625.8428 | 1625.7368 | -0.106 | -65 | 593 | 606 | SVHTKEKPPSCSLR | | | Carbamidomethyl (C)[11] | | Mascot |
| 1662.7363 | 1662.7861 | 0.0498 | 30 | 258 | 272 | THTGEKPYTCTHCGK | | | | | Mascot |
| 1961.8956 | 1961.9539 | 0.0583 | 30 | 454 | 469 | VHTNEKPYSCPECGKR | | | Carbamidomethyl (C)[10,13] | | Mascot |
| 2118.9958 | 2119.0757 | 0.0799 | 38 | 538 | 555 | VHTGEKPFACTHCGKSF R | | | Carbamidomethyl (C)[10,13] | | Mascot |
| 2211.0798 | 2211.0659 | -0.0139 | -6 | 450 | 468 | IHLRVHTNEKPYSCPECG K | | | | | Mascot |
| 2230.0605 | 2230.1414 | 0.0809 | 36 | 276 | 294 | CSSHLNEHMIIHTGEKTH R | | | | | Mascot |
| 2283.0579 | 2283.1443 | 0.0864 | 38 | 227 | 245 | HQMIHTGEKPYKCSHCD LR | | | | | Mascot |
| 2283.0579 | 2283.1443 | 0.0864 | 38 | 227 | 245 | HQMIHTGEKPYKCSHCD LR | | | | | Mascot |
| 2298.0906 | 2298.1453 | 0.0547 | 24 | 478 | 496 | LHQHVHTGVREFVCFEC EK | | | | | Mascot |

| 6 | mitochondrial import inner membrane translocase subunit TIM14 [Danio rerio] | gi\|41152151 | 12425.5 | 10.16 | 6 | 43 | 0 |
|---|---|---|---|---|---|---|---|

**Peptide Information**

| Calc. Mass | Obsrv. Mass | ± da | ± ppm | Start Seq. | End Seq. | Sequence | Ion Score | C. I. % | Modification | Rank | Result Type |
|---|---|---|---|---|---|---|---|---|---|---|---|
| 987.5469 | 987.5167 | -0.0302 | -31 | 107 | 115 | DLLDGQAKK | | | | | Mascot |
| 987.5469 | 987.5167 | -0.0302 | -31 | 107 | 115 | DLLDGQAKK | | | | | Mascot |
| 1007.458 | 1007.4591 | 0.0011 | 1 | 42 | 50 | SAFGSGYYR | | | | | Mascot |
| 1399.7791 | 1399.6824 | -0.0967 | -69 | 61 | 74 | EASLILGVSPTANK | | | | | Mascot |
| 1924.9983 | 1924.9486 | -0.0497 | -26 | 1 | 20 | MASTMVAVGLTLAAAGF TGR | | | | | Mascot |
| 1953.0375 | 1952.9709 | -0.0666 | -34 | 84 | 101 | LMILNHPDRGGSPYLAAK | | | | | Mascot |
| 2283.2278 | 2283.1443 | -0.0835 | -37 | 2 | 24 | ASTMVAVGLTLAAAGFT GRYAVR | | | | | Mascot |
| 2283.2278 | 2283.1443 | -0.0835 | -37 | 2 | 24 | ASTMVAVGLTLAAAGFT GRYAVR | | | | | Mascot |

| 7 | novel protein [Danio rerio] | gi\|94733366 | 36191.6 | 9.32 | 11 | 42 | 0 |
|---|---|---|---|---|---|---|---|

**Peptide Information**

| Calc. Mass | Obsrv. Mass | ± da | ± ppm | Start Seq. | End Seq. | Sequence | Ion Score | C. I. % | Modification | Rank | Result Type |
|---|---|---|---|---|---|---|---|---|---|---|---|
| 864.4798 | 864.4702 | -0.0096 | -11 | 292 | 298 | HRSVHTK | | | | | Mascot |
| 987.5621 | 987.5167 | -0.0454 | -46 | 198 | 206 | TFITAGHLK | | | | | Mascot |
| 987.5621 | 987.5167 | -0.0454 | -46 | 198 | 206 | TFITAGHLK | | | | | Mascot |
| 1007.392 | 1007.4591 | 0.0671 | 67 | 19 | 26 | MSDPEPCR | | | Carbamidomethyl (C)[7], Oxidation (M)[1] | | Mascot |
| 1019.4397 | 1019.5233 | 0.0836 | 82 | 273 | 281 | THKCDQCGK | | | | | Mascot |
| 1047.5681 | 1047.5 | -0.0681 | -65 | 10 | 18 | QSLLKDSEK | | | | | Mascot |
| 1625.8428 | 1625.7368 | -0.106 | -65 | 294 | 307 | SVHTKEKPPSCSLR | | | Carbamidomethyl (C)[11] | | Mascot |
| 1961.8956 | 1961.9539 | 0.0583 | 30 | 155 | 170 | VHTNEKPYSCPECGKR | | | Carbamidomethyl (C)[10,13] | | Mascot |
| 2071.9551 | 2072.0278 | 0.0727 | 35 | 77 | 94 | CFTCTLCGKGFSYNRPF K | | | | | Mascot |
| 2118.9958 | 2119.0757 | 0.0799 | 38 | 239 | 256 | VHTGEKPFACTHCGKSF R | | | Carbamidomethyl (C)[10,13] | | Mascot |
| 2211.0798 | 2211.0659 | -0.0139 | -6 | 151 | 169 | IHLRVHTNEKPYSCPECG K | | | | | Mascot |
| 2298.0906 | 2298.1453 | 0.0547 | 24 | 179 | 197 | LHQHVHTGVREFVCFEC EK | | | | | Mascot |

| 8 | RecName: Full=Serine/arginine repetitive matrix protein 4; AltName: Full=Neural-specific serine/arg | gi\|281312156 | 65482.9 | 11.48 | 11 | 41 | 0 |
|---|---|---|---|---|---|---|---|

**Peptide Information**

| Calc. Mass | Obsrv. Mass | ± da | ± ppm | Start Seq. | End Seq. | Sequence | Ion Score | C. I. % | Modification | Rank | Result Type |
|---|---|---|---|---|---|---|---|---|---|---|---|

| Calc. Mass | Obsrv. Mass | ± da | ± ppm | Start Seq. | End Seq. | Sequence | Ion Score | C. I. % | Modification | Rank | Result Type |
|---|---|---|---|---|---|---|---|---|---|---|---|
| 875.458 | 875.4468 | -0.0112 | -13 | 347 | 354 | LKGEESGR | | | | | Mascot |
| 970.3716 | 970.491 | 0.1194 | 123 | 188 | 195 | SSSCMENR | | | Carbamidomethyl (C)[4] | | Mascot |
| 1007.5228 | 1007.4591 | -0.0637 | -63 | 388 | 396 | SRSISSQSR | | | | | Mascot |
| 1047.4346 | 1047.5 | 0.0654 | 62 | 454 | 461 | YCSSCKSR | | | Carbamidomethyl (C)[2,5] | | Mascot |
| 1456.7166 | 1456.6481 | -0.0685 | -47 | 45 | 57 | LETPVGPKDDEEK | | | | | Mascot |
| 1467.6724 | 1467.726 | 0.0536 | 37 | 535 | 546 | SRSHSHTYSSYR | | | | | Mascot |
| 1662.9326 | 1662.7861 | -0.1465 | -88 | 509 | 522 | SSSILSWRLFPLTR | | | | | Mascot |
| 2071.8994 | 2072.0278 | 0.1284 | 62 | 229 | 246 | ISSKCCCHFSESTVSPSR | | | Carbamidomethyl (C)[5,6] | | Mascot |
| 2119.0876 | 2119.0757 | -0.0119 | -6 | 247 | 267 | SGVEGLSKVVIVQNSESS DGK | | | | | Mascot |
| 2155.9739 | 2155.9233 | -0.0506 | -23 | 270 | 290 | ADYDSGNDTSSPPSSKT GITR | | | | | Mascot |
| 2300.0105 | 2300.1404 | 0.1299 | 56 | 233 | 254 | CCCHFSESTVSPSRSGV EGLSK | | | | | Mascot |

| | | | | | | | |
|---|---|---|---|---|---|---|---|
| 9 | PREDICTED: clathrin light chain B-like isoform 2 [Danio rerio] | gi\|326673837 | 23720.3 | 4.55 | 7 | 40 | 0 |

**Peptide Information**

| Calc. Mass | Obsrv. Mass | ± da | ± ppm | Start Seq. | End Seq. | Sequence | Ion Score | C. I. % | Modification | Rank | Result Type |
|---|---|---|---|---|---|---|---|---|---|---|---|
| 836.397 | 836.3209 | -0.0761 | -91 | 182 | 188 | LCDFNPK | | | | | Mascot |
| 875.437 | 875.4468 | 0.0098 | 11 | 108 | 113 | WREEQK | | | | | Mascot |
| 1062.634 | 1062.674 | 0.04 | 38 | 199 | 207 | MRSVLISLK | | | Oxidation (M)[1] | | Mascot |
| 1467.9258 | 1467.726 | -0.1998 | -136 | 201 | 213 | SVLISLKQTPLLR | | | | | Mascot |
| 1952.8807 | 1952.9709 | 0.0902 | 46 | 137 | 151 | ELEDWHIHQTEQMEK | | | | | Mascot |
| 2096.9707 | 2097.0923 | 0.1216 | 58 | 136 | 151 | KELEDWHIHQTEQMEK | | | Oxidation (M)[14] | | Mascot |
| 2096.9707 | 2097.0923 | 0.1216 | 58 | 136 | 151 | KELEDWHIHQTEQMEK | | | Oxidation (M)[14] | | Mascot |
| 2211.0134 | 2211.0659 | 0.0525 | 24 | 137 | 153 | ELEDWHIHQTEQMEKNK | | | Oxidation (M)[13] | | Mascot |

| | | | | | | | |
|---|---|---|---|---|---|---|---|
| 10 | PREDICTED: phenylserine dehydratase-like [Danio rerio] | gi\|326673212 | 49208.8 | 7.51 | 11 | 40 | 0 |

**Peptide Information**

| Calc. Mass | Obsrv. Mass | ± da | ± ppm | Start Seq. | End Seq. | Sequence | Ion Score | C. I. % | Modification | Rank | Result Type |
|---|---|---|---|---|---|---|---|---|---|---|---|
| 1092.6663 | 1092.5464 | -0.1199 | -110 | 185 | 194 | GVLYLLSSLK | | | | | Mascot |
| 1456.6591 | 1456.6481 | -0.011 | -8 | 81 | 93 | DFGAEEYLNGDVK | | | | | Mascot |
| 1472.7817 | 1472.6449 | -0.1368 | -93 | 160 | 171 | LSKQYGMEIYLK | | | | | Mascot |
| 1472.7817 | 1472.6449 | -0.1368 | -93 | 160 | 171 | LSKQYGMEIYLK | | | | | Mascot |
| 1578.7621 | 1578.7957 | 0.0336 | 21 | 418 | 431 | FTVQLADWPGDMAK | | | | | Mascot |
| 1796.896 | 1796.6711 | -0.2249 | -125 | 145 | 159 | LQQSSLQKTPCTYSR | | | Carbamidomethyl (C)[11] | | Mascot |

| Calc. Mass | Obsrv. Mass | ± da | ± ppm | Start Seq. | End Seq. | Sequence | Ion Score | C. I. % | Modification | Rank | Result Type |
|---|---|---|---|---|---|---|---|---|---|---|---|
| 1952.9357 | 1952.9709 | 0.0352 | 18 | 415 | 431 | VCRFTVQLADWPGDMAK | | | Oxidation (M)[15] | | Mascot |
| 2104.1582 | 2103.949 | -0.2092 | -99 | 300 | 322 | LDAVVVPAGGHCGLLAG TAAAIK | | | | | Mascot |
| 2193.8818 | 2194.0471 | 0.1653 | 75 | 22 | 39 | GFSDCEEYDPFWQSAEQ R | | | | | Mascot |
| 2230.0332 | 2230.1414 | 0.1082 | 49 | 268 | 287 | ENGYLCLEEDDSAVYLA GLR | | | | | Mascot |
| 2284.0774 | 2284.1365 | 0.0591 | 26 | 243 | 263 | DYGAMVISYGSTARDSIN HAR | | | | | Mascot |
| 2298.1626 | 2298.1453 | -0.0173 | -8 | 124 | 144 | SSTPPAPEHLRFQDISAA AFR | | | | | Mascot |
| 2300.0723 | 2300.1404 | 0.0681 | 30 | 243 | 263 | DYGAMVISYGSTARDSIN HAR | | | Oxidation (M)[5] | | Mascot |

| | | | | | | | |
|---|---|---|---|---|---|---|---|
| 11 | PREDICTED: mitochondrial ribonuclease P protein 3-like [Danio rerio] | gi\|292623047 | 66491.7 | 8.72 | 12 | 40 | 0 |

**Peptide Information**

| Calc. Mass | Obsrv. Mass | ± da | ± ppm | Start Seq. | End Seq. | Sequence | Ion Score | C. I. % | Modification | Rank | Result Type |
|---|---|---|---|---|---|---|---|---|---|---|---|
| 842.477 | 842.4943 | 0.0173 | 21 | 389 | 395 | SFKSFVK | | | | | Mascot |
| 848.4446 | 848.5095 | 0.0649 | 76 | 572 | 577 | WLCLTR | | | Carbamidomethyl (C)[3] | | Mascot |
| 870.4211 | 870.5259 | 0.1048 | 120 | 1 | 8 | MGFVAMSK | | | | | Mascot |
| 970.4601 | 970.491 | 0.0309 | 32 | 447 | 453 | HSRNWDR | | | | | Mascot |
| 1007.5012 | 1007.4591 | -0.0421 | -42 | 142 | 149 | VMMENLVR | | | Oxidation (M)[2] | | Mascot |
| 1165.5592 | 1165.7224 | 0.1632 | 140 | 226 | 235 | EAISMLQDMK | | | | | Mascot |
| 1165.5592 | 1165.7224 | 0.1632 | 140 | 226 | 235 | EAISMLQDMK | | | | | Mascot |
| 1683.7933 | 1683.7981 | 0.0048 | 3 | 93 | 107 | HKSGLDTENDDTPVR | | | | | Mascot |
| 1815.9058 | 1815.8549 | -0.0509 | -28 | 366 | 381 | HNVMEKVIEGGDVFNK | | | | | Mascot |
| 2119.009 | 2119.0757 | 0.0667 | 31 | 244 | 263 | NYGDVIAGAVLHGDSETS WK | | | | | Mascot |
| 2230.1628 | 2230.1414 | -0.0214 | -10 | 65 | 85 | GFTPRTRPAFPHSVFSA GAAR | | | | | Mascot |
| 2297.2751 | 2297.1523 | -0.1228 | -53 | 159 | 178 | SLLSYVVQDKGTLSYEILL R | | | | | Mascot |
| 3387.8667 | 3387.6663 | -0.2004 | -59 | 412 | 442 | MLPHAAQSETLLAVVSEL EQQSLNILVLGRK | | | | | Mascot |

| | | | | | | | |
|---|---|---|---|---|---|---|---|
| 12 | PREDICTED: zinc finger protein 420 isoform 1 [Danio rerio] | gi\|326678084 | 37946.4 | 9.32 | 11 | 40 | 0 |

**Peptide Information**

| Calc. Mass | Obsrv. Mass | ± da | ± ppm | Start Seq. | End Seq. | Sequence | Ion Score | C. I. % | Modification | Rank | Result Type |
|---|---|---|---|---|---|---|---|---|---|---|---|
| 864.4798 | 864.4702 | -0.0096 | -11 | 309 | 315 | HRSVHTK | | | | | Mascot |
| 987.5621 | 987.5167 | -0.0454 | -46 | 215 | 223 | TFITAGHLK | | | | | Mascot |
| 987.5621 | 987.5167 | -0.0454 | -46 | 215 | 223 | TFITAGHLK | | | | | Mascot |

| | | | | | | | | | | | |
|---|---|---|---|---|---|---|---|---|---|---|---|
| 1007.392 | 1007.4591 | 0.0671 | 67 | 36 | 43 | MSDPEPCR | | | Carbamidomethyl (C)[7], Oxidation (M)[1] | | Mascot |
| 1019.4397 | 1019.5233 | 0.0836 | 82 | 290 | 298 | THKCDQCGK | | | | | Mascot |
| 1047.5681 | 1047.5 | -0.0681 | -65 | 27 | 35 | QSLLKDSEK | | | | | Mascot |
| 1625.8428 | 1625.7368 | -0.106 | -65 | 311 | 324 | SVHTKEKPPSCSLR | | | Carbamidomethyl (C)[11] | | Mascot |
| 1961.8956 | 1961.9539 | 0.0583 | 30 | 172 | 187 | VHTNEKPYSCPECGKR | | | Carbamidomethyl (C)[10,13] | | Mascot |
| 2071.9551 | 2072.0278 | 0.0727 | 35 | 94 | 111 | CFTCTLCGKGFSYNRPFK | | | | | Mascot |
| 2118.9958 | 2119.0757 | 0.0799 | 38 | 256 | 273 | VHTGEKPFACTHCGKSFR | | | Carbamidomethyl (C)[10,13] | | Mascot |
| 2211.0798 | 2211.0659 | -0.0139 | -6 | 168 | 186 | IHLRVHTNEKPYSCPECGK | | | | | Mascot |
| 2298.0906 | 2298.1453 | 0.0547 | 24 | 196 | 214 | LHQHVHTGVREFVCFECEK | | | | | Mascot |

13 PREDICTED: proto-oncogene DBL-like [Danio rerio] gi|326671273 124312.6 6.3 15 39 0

**Peptide Information**

| Calc. Mass | Obsrv. Mass | ± da | ± ppm | Start Seq. | End Seq. | Sequence | Ion Score | C. I. % | Modification | Rank | Result Type |
|---|---|---|---|---|---|---|---|---|---|---|---|
| 823.4494 | 823.399 | -0.0504 | -61 | 485 | 491 | MVSIAFR | | | | | Mascot |
| 850.4199 | 850.5258 | 0.1059 | 125 | 151 | 157 | SVMREGR | | | Oxidation (M)[3] | | Mascot |
| 850.4199 | 850.5258 | 0.1059 | 125 | 151 | 157 | SVMREGR | | | Oxidation (M)[3] | | Mascot |
| 1007.4938 | 1007.4591 | -0.0347 | -34 | 147 | 154 | DDIRSVMR | | | Oxidation (M)[7] | | Mascot |
| 1047.5503 | 1047.5 | -0.0503 | -48 | 1071 | 1079 | CGKDDVIIK | | | Carbamidomethyl (C)[1] | | Mascot |
| 1165.6462 | 1165.7224 | 0.0762 | 65 | 96 | 106 | TAIESFAVTVK | | | | | Mascot |
| 1165.6462 | 1165.7224 | 0.0762 | 65 | 96 | 106 | TAIESFAVTVK | | | | | Mascot |
| 1279.6398 | 1279.7505 | 0.1107 | 87 | 865 | 874 | DMARFKPMQR | | | | | Mascot |
| 1472.6144 | 1472.6449 | 0.0305 | 21 | 754 | 765 | QCSDSAFIQECK | | | Carbamidomethyl (C)[2,11] | | Mascot |
| 1472.6144 | 1472.6449 | 0.0305 | 21 | 754 | 765 | QCSDSAFIQECK | | | Carbamidomethyl (C)[2,11] | | Mascot |
| 1578.7217 | 1578.7957 | 0.074 | 47 | 618 | 629 | KIEVMHDYEENR | | | Oxidation (M)[5] | | Mascot |
| 1683.9152 | 1683.7981 | -0.1171 | -70 | 875 | 887 | HLFLHERALLFCK | | | Carbamidomethyl (C)[12] | | Mascot |
| 1866.0192 | 1865.8141 | -0.2051 | -110 | 462 | 479 | RVTAANVLTGQLGSPEPR | | | | | Mascot |
| 1866.0192 | 1865.8141 | -0.2051 | -110 | 462 | 479 | RVTAANVLTGQLGSPEPR | | | | | Mascot |
| 2072.0618 | 2072.0278 | -0.034 | -16 | 158 | 177 | QLLSNLEASKAVEGAAAEDR | | | | | Mascot |
| 2121.0137 | 2121.1003 | 0.0866 | 41 | 906 | 924 | HCLMMSAVGITENVKGDVK | | | Carbamidomethyl (C)[2], Oxidation (M)[4,5] | | Mascot |
| 2233.1282 | 2233.0454 | -0.0828 | -37 | 709 | 727 | IFLQDLESCLETPERVGAR | | | Carbamidomethyl (C)[9] | | Mascot |
| 2306.3079 | 2306.1123 | -0.1956 | -85 | 463 | 484 | VTAANVLTGQLGSPEPRLLIEK | | | | | Mascot |
| 3387.6091 | 3387.6663 | 0.0572 | 17 | 376 | 406 | SNLDILNLEFQAVLTPQLQGMGMGCGYSPMK | | | Oxidation (M)[21,23] | | Mascot |

14 PREDICTED: clathrin light chain B-like isoform 1 [Danio rerio] gi|326673839 25713.3 4.57 7 38 0

Peptide Information

| Calc. Mass | Obsrv. Mass | ± da | ± ppm | Start Seq. | End Seq. | Sequence | Ion Score | C. I. % | Modification | Rank | Result Type |
|---|---|---|---|---|---|---|---|---|---|---|---|
| 836.397 | 836.3209 | -0.0761 | -91 | 200 | 206 | LCDFNPK | | | | | Mascot |
| 875.437 | 875.4468 | 0.0098 | 11 | 108 | 113 | WREEQK | | | | | Mascot |
| 1062.634 | 1062.674 | 0.04 | 38 | 217 | 225 | MRSVLISLK | | | Oxidation (M)[1] | | Mascot |
| 1467.9258 | 1467.726 | -0.1998 | -136 | 219 | 231 | SVLISLKQTPLLR | | | | | Mascot |
| 1952.8807 | 1952.9709 | 0.0902 | 46 | 137 | 151 | ELEDWHIHQTEQMEK | | | | | Mascot |
| 2096.9707 | 2097.0923 | 0.1216 | 58 | 136 | 151 | KELEDWHIHQTEQMEK | | | Oxidation (M)[14] | | Mascot |
| 2096.9707 | 2097.0923 | 0.1216 | 58 | 136 | 151 | KELEDWHIHQTEQMEK | | | Oxidation (M)[14] | | Mascot |
| 2211.0134 | 2211.0659 | 0.0525 | 24 | 137 | 153 | ELEDWHIHQTEQMEKNK | | | Oxidation (M)[13] | | Mascot |

15 PREDICTED: pericentrin [Danio rerio] gi|326668478 491858 4.98 30 38 0

Peptide Information

| Calc. Mass | Obsrv. Mass | ± da | ± ppm | Start Seq. | End Seq. | Sequence | Ion Score | C. I. % | Modification | Rank | Result Type |
|---|---|---|---|---|---|---|---|---|---|---|---|
| 836.3818 | 836.3209 | -0.0609 | -73 | 3557 | 3563 | CSLEAEK | | | Carbamidomethyl (C)[1] | | Mascot |
| 842.4995 | 842.4943 | -0.0052 | -6 | 15 | 21 | AKLAHFR | | | | | Mascot |
| 850.4087 | 850.5258 | 0.1171 | 138 | 2129 | 2136 | TPSSMTAR | | | | | Mascot |
| 850.4087 | 850.5258 | 0.1171 | 138 | 2129 | 2136 | TPSSMTAR | | | | | Mascot |
| 853.3897 | 853.3448 | -0.0449 | -53 | 27 | 34 | SESSSTQK | | | | | Mascot |
| 864.3581 | 864.4702 | 0.1121 | 130 | 1982 | 1988 | EESDQEK | | | | | Mascot |
| 875.458 | 875.4468 | -0.0112 | -13 | 2850 | 2857 | SLGEERGK | | | | | Mascot |
| 882.5155 | 882.5498 | 0.0343 | 39 | 3606 | 3612 | SEIHKLR | | | | | Mascot |
| 987.5105 | 987.5167 | 0.0062 | 6 | 2715 | 2722 | QAEIEELR | 10 | 0 | | | Mascot |
| 987.5105 | 987.5167 | 0.0062 | 6 | 2715 | 2722 | QAEIEELR | | | | | Mascot |
| 1007.4904 | 1007.4591 | -0.0313 | -31 | 427 | 434 | HLQEEHSK | | | | | Mascot |
| 1019.5189 | 1019.5233 | 0.0044 | 4 | 3623 | 3631 | QTCLQAVEK | | | | | Mascot |
| 1022.4935 | 1022.4899 | -0.0036 | -4 | 3557 | 3565 | CSLEAEKSR | | | | | Mascot |
| 1045.4908 | 1045.5457 | 0.0549 | 53 | 1472 | 1480 | QAEAEVNER | | | | | Mascot |
| 1294.646 | 1294.5033 | -0.1427 | -110 | 4205 | 4216 | MYTSPVLGAAER | | | | | Mascot |
| 1371.7842 | 1371.6129 | -0.1713 | -125 | 1989 | 2000 | AVALEELEKTLR | | | | | Mascot |
| 1399.6079 | 1399.6824 | 0.0745 | 53 | 3995 | 4007 | SSAVTEECMVSEK | | | | | Mascot |
| 1456.6294 | 1456.6481 | 0.0187 | 13 | 3995 | 4007 | SSAVTEECMVSEK | | | Carbamidomethyl (C)[8] | | Mascot |
| 1472.6243 | 1472.6449 | 0.0206 | 14 | 3995 | 4007 | SSAVTEECMVSEK | | | Carbamidomethyl (C)[8], Oxidation (M)[9] | | Mascot |
| 1472.6243 | 1472.6449 | 0.0206 | 14 | 3995 | 4007 | SSAVTEECMVSEK | | | Carbamidomethyl (C)[8], Oxidation (M)[9] | | Mascot |

| Calc. Mass | Obsrv. Mass | ± da | ± ppm | Start Seq. | End Seq. | Sequence | Ion Score | C. I. % | Modification | Rank | Result Type |
|---|---|---|---|---|---|---|---|---|---|---|---|
| 1475.7271 | 1475.6984 | -0.0287 | -19 | 2723 | 2734 | SLVERLQCDQER | | | | | Mascot |
| 1556.7485 | 1556.7091 | -0.0394 | -25 | 1515 | 1526 | EQLRQMEETHQK | | | | | Mascot |
| 1578.7845 | 1578.7957 | 0.0112 | 7 | 3312 | 3323 | LDAHFVEYLRCR | | | Carbamidomethyl (C)[11] | | Mascot |
| 1587.8047 | 1587.8539 | 0.0492 | 31 | 1603 | 1616 | SLECQESPQVVALK | | | Carbamidomethyl (C)[4] | | Mascot |
| 1625.901 | 1625.7368 | -0.1642 | -101 | 1954 | 1966 | QRLHFLEEAELLK | | | | | Mascot |
| 1662.8195 | 1662.7861 | -0.0334 | -20 | 310 | 323 | NSQRAQEQVEAFQK | | | | | Mascot |
| 1683.8007 | 1683.7981 | -0.0026 | -2 | 1617 | 1631 | QKLQAQYDGEMSAAK | | | Oxidation (M)[11] | | Mascot |
| 1815.9633 | 1815.8549 | -0.1084 | -60 | 358 | 372 | VELNQEMVRLDNLVK | | | Oxidation (M)[7] | | Mascot |
| 1923.0294 | 1922.9604 | -0.069 | -36 | 1326 | 1341 | VTEESHREEIALLQLR | | | | | Mascot |
| 2071.9573 | 2072.0278 | 0.0705 | 34 | 2788 | 2804 | QQRASEESLCQELNSHR | | | Carbamidomethyl (C)[10] | | Mascot |
| 2103.9243 | 2103.949 | 0.0247 | 12 | 3990 | 4007 | MEQVKSSAVTEECMVSE K | | | Carbamidomethyl (C)[13], Oxidation (M)[1,14] | | Mascot |
| 2156.0002 | 2155.9233 | -0.0769 | -36 | 441 | 459 | ELLSQAQSQYSDSPHNH SK | | | | | Mascot |
| 2247.1404 | 2247.0593 | -0.0811 | -36 | 3304 | 3321 | LELENTERLDAHFVEYLR | | | | | Mascot |
| 2284.1013 | 2284.1365 | 0.0352 | 15 | 3955 | 3974 | ENSEFSECLAALSEEKISL K | | | Carbamidomethyl (C)[8] | | Mascot |
| 2300.0935 | 2300.1404 | 0.0469 | 20 | 3545 | 3563 | LQQEQTQSHDLRCSLEA EK | | | Carbamidomethyl (C)[13] | | Mascot |

| | | | | | | | | | |
|---|---|---|---|---|---|---|---|---|---|
| 16 | novel protein similar to H.sapiens DOK7, docking protein 7 (DOK7) [Danio rerio] | gi\|220676956 | 42560.3 | 7.12 | 3 | 37 | 0 | 30 | 71.498 |

Peptide Information

| Calc. Mass | Obsrv. Mass | ± da | ± ppm | Start Seq. | End Seq. | Sequence | Ion Score | C. I. % | Modification | Rank | Result Type |
|---|---|---|---|---|---|---|---|---|---|---|---|
| 987.533 | 987.5167 | -0.0163 | -17 | 53 | 60 | ERNQATLR | 30 | 71.498 | | | Mascot |
| 987.533 | 987.5167 | -0.0163 | -17 | 53 | 60 | ERNQATLR | | | | | Mascot |
| 1815.8113 | 1815.8549 | 0.0436 | 24 | 241 | 257 | LSMLSICSHHSSAGDDR | | | | | Mascot |
| 2300.3198 | 2300.1404 | -0.1794 | -78 | 23 | 42 | WVILRKPSPVADCLSLLV YK | | | | | Mascot |

| | | | | | | | | | |
|---|---|---|---|---|---|---|---|---|---|
| 17 | PREDICTED: protein Dok-7 [Danio rerio] | gi\|326664133 | 71162.3 | 6.4 | 4 | 37 | 0 | 30 | 71.498 |

Peptide Information

| Calc. Mass | Obsrv. Mass | ± da | ± ppm | Start Seq. | End Seq. | Sequence | Ion Score | C. I. % | Modification | Rank | Result Type |
|---|---|---|---|---|---|---|---|---|---|---|---|
| 987.533 | 987.5167 | -0.0163 | -17 | 53 | 60 | ERNQATLR | 30 | 71.498 | | | Mascot |
| 987.533 | 987.5167 | -0.0163 | -17 | 53 | 60 | ERNQATLR | | | | | Mascot |
| 1399.7185 | 1399.6824 | -0.0361 | -26 | 505 | 517 | LQPIICPDCGGRK | | | | | Mascot |
| 1456.7399 | 1456.6481 | -0.0918 | -63 | 505 | 517 | LQPIICPDCGGRK | | | Carbamidomethyl (C)[6] | | Mascot |
| 1815.8113 | 1815.8549 | 0.0436 | 24 | 241 | 257 | LSMLSICSHHSSAGDDR | | | | | Mascot |
| 2300.3198 | 2300.1404 | -0.1794 | -78 | 23 | 42 | WVILRKPSPVADCLSLLV YK | | | | | Mascot |

| 18 | cullin 3b [Danio rerio] | gi|299523039 | 88726.4 | 8.68 | 15 | 36 | 0 |
|---|---|---|---|---|---|---|---|

Peptide Information

| Calc. Mass | Obsrv. Mass | ± da | ± ppm | Start Seq. | End Seq. | Sequence | Ion Score | C. I. % | Modification | Rank | Result Type |
|---|---|---|---|---|---|---|---|---|---|---|---|
| 875.4217 | 875.4468 | 0.0251 | 29 | 691 | 697 | QKVDDDR | | | | | Mascot |
| 1022.5298 | 1022.4899 | -0.0399 | -39 | 1 | 10 | MSNLSKGGTK | | | | | Mascot |
| 1045.4908 | 1045.5457 | 0.0549 | 53 | 679 | 687 | QGESDPERK | | | | | Mascot |
| 1092.5507 | 1092.5464 | -0.0043 | -4 | 59 | 67 | NAYTMVLHK | | | Oxidation (M)[5] | | Mascot |
| 1165.6688 | 1165.7224 | 0.0536 | 46 | 698 | 707 | KHEIEAAIVR | | | | | Mascot |
| 1165.6688 | 1165.7224 | 0.0536 | 46 | 698 | 707 | KHEIEAAIVR | | | | | Mascot |
| 1399.7225 | 1399.6824 | -0.0401 | -29 | 414 | 424 | AMVLFRFMQEK | | | | | Mascot |
| 1556.7703 | 1556.7091 | -0.0612 | -39 | 46 | 58 | KNNSGLSFEELYR | | | | | Mascot |
| 1578.6411 | 1578.7957 | 0.1546 | 98 | 475 | 487 | DMSISNTTMDEFR | | | Oxidation (M)[2,9] | | Mascot |
| 1796.7715 | 1796.6711 | -0.1004 | -56 | 569 | 586 | EDGSDMVGGAQVTGSN TR | | | Oxidation (M)[6] | | Mascot |
| 1924.8665 | 1924.9486 | 0.0821 | 43 | 569 | 587 | EDGSDMVGGAQVTGSN TRK | | | Oxidation (M)[6] | | Mascot |
| 1962.0082 | 1961.9539 | -0.0543 | -28 | 180 | 197 | GAIRNACQMLMILGLEGR | | | Oxidation (M)[9] | | Mascot |
| 2097.0654 | 2097.0923 | 0.0269 | 13 | 271 | 288 | HMKTIVEMENSGLVHML K | | | | | Mascot |
| 2097.0654 | 2097.0923 | 0.0269 | 13 | 271 | 288 | HMKTIVEMENSGLVHML K | | | | | Mascot |
| 2194.1423 | 2194.0471 | -0.0952 | -43 | 401 | 419 | GLTEQEVESILDKAMVLF R | | | Oxidation (M)[15] | | Mascot |
| 2230.1978 | 2230.1414 | -0.0564 | -25 | 28 | 45 | YVNNIWDLLKNAIQEIQR | | | | | Mascot |
| 2306.2148 | 2306.1123 | -0.1025 | -44 | 588 | 606 | HILQVSTFQMTILMLFNN R | | | | | Mascot |

| 19 | mitogen-activated protein kinase 1 [Danio rerio] | gi|40254655 | 42048.5 | 6.32 | 8 | 36 | 0 |
|---|---|---|---|---|---|---|---|

Peptide Information

| Calc. Mass | Obsrv. Mass | ± da | ± ppm | Start Seq. | End Seq. | Sequence | Ion Score | C. I. % | Modification | Rank | Result Type |
|---|---|---|---|---|---|---|---|---|---|---|---|
| 864.4362 | 864.4702 | 0.034 | 39 | 363 | 369 | FQPGYRP | | | | | Mascot |
| 987.508 | 987.5167 | 0.0087 | 9 | 302 | 309 | MLTFNPHK | | | | | Mascot |
| 987.508 | 987.5167 | 0.0087 | 9 | 302 | 309 | MLTFNPHK | | | | | Mascot |
| 1467.7512 | 1467.726 | -0.0252 | -17 | 204 | 216 | APEIMLNSKGYTK | | | Oxidation (M)[5] | | Mascot |
| 1578.8373 | 1578.7957 | -0.0416 | -26 | 350 | 362 | ETLKELIFEETAR | | | | | Mascot |
| 1952.9865 | 1952.9709 | -0.0156 | -8 | 354 | 369 | ELIFEETARFQPGYRP | | | | | Mascot |
| 2156.0222 | 2155.9233 | -0.0989 | -46 | 1 | 24 | MATAAVSAPAGGGPNPG SGAEMVR | | | | | Mascot |
| 2246.9844 | 2247.0593 | 0.0749 | 33 | 34 | 54 | YSNLSYIGGGAYGMVCS AYDR | | | | | Mascot |
| 3387.7842 | 3387.6663 | -0.1179 | -35 | 241 | 270 | HYLDQLNHILGILGSPSQ | | | | | Mascot |

EDLNCIINIKAR

| 20 | vasa-like protein [Danio rerio] | gi\|19482136 | 76747.5 | 5.53 | 12 | 36 | 0 |
|---|---|---|---|---|---|---|---|

Peptide Information

| Calc. Mass | Obsrv. Mass | ± da | ± ppm | Start Seq. | End Seq. | Sequence | Ion Score | C. I. % | Modification | Rank | Result Type |
|---|---|---|---|---|---|---|---|---|---|---|---|
| 823.4168 | 823.399 | -0.0178 | -22 | 106 | 113 | GGFRSGSR | | | | | Mascot |
| 853.4426 | 853.3448 | -0.0978 | -115 | 197 | 204 | GFGRGGFR | | | | | Mascot |
| 882.4315 | 882.5498 | 0.1183 | 134 | 678 | 685 | VFASTDSR | | | | | Mascot |
| 970.4913 | 970.491 | -0.0003 | 0 | 531 | 538 | TMVFVETK | | | Oxidation (M)[2] | | Mascot |
| 1371.5742 | 1371.6129 | 0.0387 | 28 | 439 | 449 | MLDMGFEPEMR | | | Oxidation (M)[1] | | Mascot |
| 1467.7325 | 1467.726 | -0.0065 | -4 | 207 | 219 | NEEVFSKVTTADK | | | | | Mascot |
| 1472.709 | 1472.6449 | -0.0641 | -44 | 540 | 552 | SADFIATFLCQEK | | | | | Mascot |
| 1472.709 | 1472.6449 | -0.0641 | -44 | 540 | 552 | SADFIATFLCQEK | | | | | Mascot |
| 1815.8582 | 1815.8549 | -0.0033 | -2 | 464 | 478 | QTLMFSATYPEDIQR | | | Oxidation (M)[4] | | Mascot |
| 1865.7532 | 1865.8141 | 0.0609 | 33 | 80 | 97 | SEIDENGSDGGWNGGES R | | | | | Mascot |
| 1865.7532 | 1865.8141 | 0.0609 | 33 | 80 | 97 | SEIDENGSDGGWNGGES R | | | | | Mascot |
| 1951.9952 | 1951.9774 | -0.0178 | -9 | 403 | 420 | GCNVLCATPGRLHDLIGR | | | Carbamidomethyl (C)[2] | | Mascot |
| 2230.0444 | 2230.1414 | 0.097 | 43 | 461 | 478 | EERQTLMFSATYPEDIQR | | | Oxidation (M)[7] | | Mascot |
| 3387.5969 | 3387.6663 | 0.0694 | 20 | 259 | 289 | YDDILVDVSGSNPPKAIM TFEEAGLCDSLSK | | | Carbamidomethyl (C)[26], Oxidation (M)[18] | | Mascot |

| 21 | PREDICTED: neuronal acetylcholine receptor subunit beta-2-like [Danio rerio] | gi\|292620953 | 51265.9 | 5.66 | 9 | 36 | 0 |
|---|---|---|---|---|---|---|---|

Peptide Information

| Calc. Mass | Obsrv. Mass | ± da | ± ppm | Start Seq. | End Seq. | Sequence | Ion Score | C. I. % | Modification | Rank | Result Type |
|---|---|---|---|---|---|---|---|---|---|---|---|
| 987.4564 | 987.5167 | 0.0603 | 61 | 315 | 323 | SPSTHSMPK | | | Oxidation (M)[7] | | Mascot |
| 987.4564 | 987.5167 | 0.0603 | 61 | 315 | 323 | SPSTHSMPK | | | Oxidation (M)[7] | | Mascot |
| 1062.5652 | 1062.674 | 0.1088 | 102 | 2 | 9 | EMLYLHLK | | | Oxidation (M)[2] | | Mascot |
| 1467.6566 | 1467.726 | 0.0694 | 47 | 407 | 419 | CSADMEEAVKGVR | | | Carbamidomethyl (C)[1], Oxidation (M)[5] | | Mascot |
| 1587.6454 | 1587.8539 | 0.2085 | 131 | 82 | 93 | LMWDPEEYEGMR | | | Oxidation (M)[2,11] | | Mascot |
| 1683.7505 | 1683.7981 | 0.0476 | 28 | 82 | 94 | LMWDPEEYEGMRK | | | | | Mascot |
| 1815.8483 | 1815.8549 | 0.0066 | 4 | 151 | 164 | DFPFDQQNCTLKFR | | | Carbamidomethyl (C)[9] | | Mascot |
| 1952.9535 | 1952.9709 | 0.0174 | 9 | 147 | 162 | IEVRDFPFDQQNCTLK | | | | | Mascot |
| 2230.3857 | 2230.1414 | -0.2443 | -110 | 256 | 275 | ITLCISVLLALTVFLLLISK | | | Carbamidomethyl (C)[4] | | Mascot |
| 2297.2822 | 2297.1523 | -0.1299 | -57 | 43 | 63 | SQQVTIAIQVSLAQLISVN ER | | | | | Mascot |

| 22 | Lad1 protein [Danio rerio] | gi\|115528644 | 50936.1 | 6 | 9 | 36 | 0 |
|---|---|---|---|---|---|---|---|

Peptide Information

| Calc. Mass | Obsrv. Mass | ± da | ± ppm | Start Seq. | End Seq. | Sequence | Ion Score | C. I. % | Modification | Rank | Result Type |
|---|---|---|---|---|---|---|---|---|---|---|---|
| 882.5155 | 882.5498 | 0.0343 | 39 | 109 | 115 | HVETLRK | | | | | Mascot |
| 987.4602 | 987.5167 | 0.0565 | 57 | 188 | 196 | SQTHSNASR | | | | | Mascot |
| 987.4602 | 987.5167 | 0.0565 | 57 | 188 | 196 | SQTHSNASR | | | | | Mascot |
| 1047.5793 | 1047.5 | -0.0793 | -76 | 339 | 347 | LSSRSIQEK | | | | | Mascot |
| 1092.5354 | 1092.5464 | 0.011 | 10 | 369 | 378 | TVCLADEVSR | | | | | Mascot |
| 1796.9324 | 1796.6711 | -0.2613 | -145 | 363 | 378 | SPVVHKTVCLADEVSR | | | Carbamidomethyl (C)[9] | | Mascot |
| 1815.8242 | 1815.8549 | 0.0307 | 17 | 118 | 133 | EEGEEGEKDILDAEPR | | | | | Mascot |
| 2071.9568 | 2072.0278 | 0.071 | 34 | 155 | 173 | NIFSPEPPTNSTDTETHGK | | | | | Mascot |
| 2104.0669 | 2103.949 | -0.1179 | -56 | 420 | 438 | TFNISSSPTDLRHVDISSK | | | | | Mascot |
| 2230.1497 | 2230.1414 | -0.0083 | -4 | 1 | 20 | HTSLQPSSTLENTIMSISRK | | | | | Mascot |

23 cullin-1 [Danio rerio] gi|45433528 89971.2 8.19 13 36 0

Peptide Information

| Calc. Mass | Obsrv. Mass | ± da | ± ppm | Start Seq. | End Seq. | Sequence | Ion Score | C. I. % | Modification | Rank | Result Type |
|---|---|---|---|---|---|---|---|---|---|---|---|
| 823.409 | 823.399 | -0.01 | -12 | 35 | 41 | QSMAKSR | | | Oxidation (M)[3] | | Mascot |
| 836.3566 | 836.3209 | -0.0357 | -43 | 149 | 155 | ECDEGRK | | | | | Mascot |
| 864.4825 | 864.4702 | -0.0123 | -14 | 223 | 230 | GPTLSVYK | | | | | Mascot |
| 882.4501 | 882.5498 | 0.0997 | 113 | 323 | 329 | MYNLVSR | | | | | Mascot |
| 987.5331 | 987.5167 | -0.0164 | -17 | 466 | 473 | FYAKMLAK | | | Oxidation (M)[5] | | Mascot |
| 987.5331 | 987.5167 | -0.0164 | -17 | 466 | 473 | FYAKMLAK | | | Oxidation (M)[5] | | Mascot |
| 996.6564 | 996.5763 | -0.0801 | -80 | 710 | 718 | LLIQAAIVR | | | | | Mascot |
| 996.6564 | 996.5763 | -0.0801 | -80 | 710 | 718 | LLIQAAIVR | | | | | Mascot |
| 1399.6865 | 1399.6824 | -0.0041 | -3 | 567 | 578 | FTAFYASRHSGR | | | | | Mascot |
| 1456.7325 | 1456.6481 | -0.0844 | -58 | 1 | 13 | MSSNRTQTPHGLK | | | | | Mascot |
| 1472.7274 | 1472.6449 | -0.0825 | -56 | 1 | 13 | MSSNRTQTPHGLK | | | Oxidation (M)[1] | | Mascot |
| 1472.7274 | 1472.6449 | -0.0825 | -56 | 1 | 13 | MSSNRTQTPHGLK | | | Oxidation (M)[1] | | Mascot |
| 1587.7584 | 1587.8539 | 0.0955 | 60 | 495 | 507 | QACGFEYTSKLQR | | | Carbamidomethyl (C)[3] | | Mascot |
| 1683.9104 | 1683.7981 | -0.1123 | -67 | 156 | 169 | GIYEIYSLALVTWR | | | | | Mascot |
| 2072.0005 | 2072.0278 | 0.0273 | 13 | 248 | 264 | ESTEFLQQNPVTEYMKK | | | | | Mascot |
| 2152.126 | 2152.0601 | -0.0659 | -31 | 580 | 597 | LTWLYHLSKGELVTNCFK | | | | | Mascot |
| 2152.126 | 2152.0601 | -0.0659 | -31 | 580 | 597 | LTWLYHLSKGELVTNCFK | | | | | Mascot |
| 2247.075 | 2247.0593 | -0.0157 | -7 | 378 | 398 | YNALVMSAFNNDAGFVA | | | Oxidation (M)[6] | | Mascot |

ALDK

| | | | | | | | |
|---|---|---|---|---|---|---|---|
| 24 | probable ATP-dependent RNA helicase DDX4 [Danio rerio] | gi\|18859541 | 76956.6 | 5.64 | 12 | 36 | 0 |

Peptide Information

| Calc. Mass | Obsrv. Mass | ± da | ± ppm | Start Seq. | End Seq. | Sequence | Ion Score | C. I. % | Modification | Rank | Result Type |
|---|---|---|---|---|---|---|---|---|---|---|---|
| 823.4168 | 823.399 | -0.0178 | -22 | 106 | 113 | GGFRSGSR | | | | | Mascot |
| 853.4426 | 853.3448 | -0.0978 | -115 | 198 | 205 | GFGRGGFR | | | | | Mascot |
| 882.4315 | 882.5498 | 0.1183 | 134 | 679 | 686 | VFASTDSR | | | | | Mascot |
| 970.4913 | 970.491 | -0.0003 | 0 | 532 | 539 | TMVFVETK | | | Oxidation (M)[2] | | Mascot |
| 1371.5742 | 1371.6129 | 0.0387 | 28 | 440 | 450 | MLDMGFEPEMR | | | Oxidation (M)[1] | | Mascot |
| 1467.7325 | 1467.726 | -0.0065 | -4 | 208 | 220 | NEEVFSKVTTADK | | | | | Mascot |
| 1472.709 | 1472.6449 | -0.0641 | -44 | 541 | 553 | SADFIATFLCQEK | | | | | Mascot |
| 1472.709 | 1472.6449 | -0.0641 | -44 | 541 | 553 | SADFIATFLCQEK | | | | | Mascot |
| 1815.8582 | 1815.8549 | -0.0033 | -2 | 465 | 479 | QTLMFSATYPEDIQR | | | Oxidation (M)[4] | | Mascot |
| 1865.7532 | 1865.8141 | 0.0609 | 33 | 80 | 97 | SEIDENGSDGGWNGGES R | | | | | Mascot |
| 1865.7532 | 1865.8141 | 0.0609 | 33 | 80 | 97 | SEIDENGSDGGWNGGES R | | | | | Mascot |
| 1951.9952 | 1951.9774 | -0.0178 | -9 | 404 | 421 | GCNVLCATPGRLHDLIGR | | | Carbamidomethyl (C)[2] | | Mascot |
| 2230.0444 | 2230.1414 | 0.097 | 43 | 462 | 479 | EERQTLMFSATYPEDIQR | | | Oxidation (M)[7] | | Mascot |
| 3387.5969 | 3387.6663 | 0.0694 | 20 | 260 | 290 | YDDILVDVSGSNPPKAIM TFEEAGLCDSLSK | | | Carbamidomethyl (C)[26], Oxidation (M)[18] | | Mascot |

| | | | | | | | |
|---|---|---|---|---|---|---|---|
| 25 | truncated RAF1 [Danio rerio] | gi\|41224402 | 32290.4 | 9.5 | 7 | 35 | 0 |

Peptide Information

| Calc. Mass | Obsrv. Mass | ± da | ± ppm | Start Seq. | End Seq. | Sequence | Ion Score | C. I. % | Modification | Rank | Result Type |
|---|---|---|---|---|---|---|---|---|---|---|---|
| 882.4791 | 882.5498 | 0.0707 | 80 | 215 | 222 | IPSSSVHR | | | | | Mascot |
| 987.5006 | 987.5167 | 0.0161 | 16 | 2 | 9 | EQLQGAWR | 8 | 0 | | | Mascot |
| 987.5006 | 987.5167 | 0.0161 | 16 | 2 | 9 | EQLQGAWR | | | | | Mascot |
| 1045.452 | 1045.5457 | 0.0937 | 90 | 165 | 172 | FHEHCSTK | | | Carbamidomethyl (C)[5] | | Mascot |
| 1371.7855 | 1371.6129 | -0.1726 | -126 | 50 | 60 | TIRVFLPNQQR | | | | | Mascot |
| 1556.7843 | 1556.7091 | -0.0752 | -48 | 19 | 32 | DSVFESSYVSPVIK | | | | | Mascot |
| 1925.0459 | 1924.9486 | -0.0973 | -51 | 61 | 77 | TVVNVRPGMTLHSCLIK | | | Carbamidomethyl (C)[14] | | Mascot |
| 2097.0481 | 2097.0923 | 0.0442 | 21 | 81 | 99 | VRGLQPECCAVFSLHPG QR | | | | | Mascot |
| 2097.0481 | 2097.0923 | 0.0442 | 21 | 81 | 99 | VRGLQPECCAVFSLHPG QR | | | | | Mascot |
| 2211.0908 | 2211.0659 | -0.0249 | -11 | 81 | 99 | VRGLQPECCAVFSLHPG QR | | | Carbamidomethyl (C)[8,9] | | Mascot |

| | | | | | | | |
|---|---|---|---|---|---|---|---|
| 26 | PREDICTED: protein tyrosine phosphatase type IVA 3 [Danio rerio] | gi\|125840045 | 19894.2 | 8.99 | 6 | 35 | 0 |

**Peptide Information**

| Calc. Mass | Obsrv. Mass | ± da | ± ppm | Start Seq. | End Seq. | Sequence | Ion Score | C. I. % | Modification | Rank | Result Type |
|---|---|---|---|---|---|---|---|---|---|---|---|
| 1399.6886 | 1399.6824 | -0.0062 | -4 | 44 | 55 | TVVRVCESTYDK | | | | | Mascot |
| 1456.7101 | 1456.6481 | -0.062 | -43 | 44 | 55 | TVVRVCESTYDK | | | Carbamidomethyl (C)[6] | | Mascot |
| 1467.8604 | 1467.726 | -0.1344 | -92 | 111 | 125 | APVLVAVALIEGGMK | | | | | Mascot |
| 2071.9729 | 2072.0278 | 0.0549 | 26 | 2 | 18 | AWLNGLYPVEVCYNSMR | | | Carbamidomethyl (C)[12] | | Mascot |
| 2096.9746 | 2097.0923 | 0.1177 | 56 | 61 | 79 | HGITVMDWPFDDGAPPPTK | | | Oxidation (M)[6] | | Mascot |
| 2096.9746 | 2097.0923 | 0.1177 | 56 | 61 | 79 | HGITVMDWPFDDGAPPPTK | | | Oxidation (M)[6] | | Mascot |
| 2120.9309 | 2121.1003 | 0.1694 | 80 | 91 | 110 | SFSEDPGCCVAVHCVAGLGR | | | Carbamidomethyl (C)[8,9] | | Mascot |
| 2306.0474 | 2306.1123 | 0.0649 | 28 | 90 | 110 | KSFSEDPGCCVAVHCVAGLGR | | | Carbamidomethyl (C)[9,10,15] | | Mascot |

| | | | | | | | |
|---|---|---|---|---|---|---|---|
| 27 | vasa-like protein [Danio rerio] | gi\|19309904 | 76832.5 | 5.57 | 12 | 35 | 0 |

**Peptide Information**

| Calc. Mass | Obsrv. Mass | ± da | ± ppm | Start Seq. | End Seq. | Sequence | Ion Score | C. I. % | Modification | Rank | Result Type |
|---|---|---|---|---|---|---|---|---|---|---|---|
| 823.4168 | 823.399 | -0.0178 | -22 | 105 | 112 | GGFRSGSR | | | | | Mascot |
| 853.4426 | 853.3448 | -0.0978 | -115 | 197 | 204 | GFGRGGFR | | | | | Mascot |
| 882.4315 | 882.5498 | 0.1183 | 134 | 678 | 685 | VFASTDSR | | | | | Mascot |
| 970.4913 | 970.491 | -0.0003 | 0 | 531 | 538 | TMVFVETK | | | Oxidation (M)[2] | | Mascot |
| 1371.5742 | 1371.6129 | 0.0387 | 28 | 439 | 449 | MLDMGFEPEMR | | | Oxidation (M)[1] | | Mascot |
| 1467.7325 | 1467.726 | -0.0065 | -4 | 207 | 219 | NEEVFSKVTTADK | | | | | Mascot |
| 1472.709 | 1472.6449 | -0.0641 | -44 | 540 | 552 | SADFIATFLCQEK | | | | | Mascot |
| 1472.709 | 1472.6449 | -0.0641 | -44 | 540 | 552 | SADFIATFLCQEK | | | | | Mascot |
| 1815.8582 | 1815.8549 | -0.0033 | -2 | 464 | 478 | QTLMFSATYPEDIQR | | | Oxidation (M)[4] | | Mascot |
| 1865.7532 | 1865.8141 | 0.0609 | 33 | 79 | 96 | SEIDENGSDGGWNGGESR | | | | | Mascot |
| 1865.7532 | 1865.8141 | 0.0609 | 33 | 79 | 96 | SEIDENGSDGGWNGGESR | | | | | Mascot |
| 1951.9952 | 1951.9774 | -0.0178 | -9 | 403 | 420 | GCNVLCATPGRLHDLIGR | | | Carbamidomethyl (C)[2] | | Mascot |
| 2230.0444 | 2230.1414 | 0.097 | 43 | 461 | 478 | EERQTLMFSATYPEDIQR | | | Oxidation (M)[7] | | Mascot |
| 3387.5969 | 3387.6663 | 0.0694 | 20 | 259 | 289 | YDDILVDVSGSNPPKAIMTFEEAGLCDSLSK | | | Carbamidomethyl (C)[26], Oxidation (M)[18] | | Mascot |

| | | | | | | | | | | |
|---|---|---|---|---|---|---|---|---|---|---|
| 28 | PREDICTED: interleukin-1 receptor-like 2-like [Danio rerio] | gi\|326670867 | 62378.6 | 7.52 | 5 | 35 | 0 | 25 | 16.656 |

**Peptide Information**

| Calc. Mass | Obsrv. Mass | ± da | ± ppm | Start Seq. | End Seq. | Sequence | Ion Score | C. I. % | Modification | Rank | Result Type |
|---|---|---|---|---|---|---|---|---|---|---|---|
| 823.3614 | 823.399 | 0.0376 | 46 | 357 | 364 | SVACSDGK | | | Carbamidomethyl (C)[4] | | Mascot |
| 850.4338 | 850.5258 | 0.092 | 108 | 540 | 547 | KGTDSMVL | | | | | Mascot |
| 850.4338 | 850.5258 | 0.092 | 108 | 540 | 547 | KGTDSMVL | | | | | Mascot |
| 864.4308 | 864.4702 | 0.0394 | 46 | 128 | 135 | DELTSTAK | | | | | Mascot |
| 987.537 | 987.5167 | -0.0203 | -21 | 507 | 514 | TQGILNWR | 25 | 16.656 | | | Mascot |
| 987.537 | 987.5167 | -0.0203 | -21 | 507 | 514 | TQGILNWR | | | | | Mascot |
| 2156.1379 | 2155.9233 | -0.2146 | -100 | 430 | 449 | LIIILTSQSCSSPQKHASDK | | | | | Mascot |

29 neural orphan nuclear receptor Nur77 [Danio rerio] gi|47060283 63776.2 5.79 10 35 0

Peptide Information

| Calc. Mass | Obsrv. Mass | ± da | ± ppm | Start Seq. | End Seq. | Sequence | Ion Score | C. I. % | Modification | Rank | Result Type |
|---|---|---|---|---|---|---|---|---|---|---|---|
| 836.4368 | 836.3209 | -0.1159 | -139 | 299 | 306 | CLAVGMVK | | | Oxidation (M)[6] | | Mascot |
| 882.5519 | 882.5498 | -0.0021 | -2 | 319 | 326 | GRLPSKPK | | | | | Mascot |
| 1019.5302 | 1019.5233 | -0.0069 | -7 | 546 | 554 | TLCTQGLQR | | | | | Mascot |
| 1062.5248 | 1062.674 | 0.1492 | 140 | 31 | 39 | LAMDVTEQR | | | | | Mascot |
| 1467.697 | 1467.726 | 0.029 | 20 | 273 | 285 | YVCLANKDCPVDK | | | | | Mascot |
| 1578.7145 | 1578.7957 | 0.0812 | 51 | 431 | 443 | FIYAFMSNPETDK | | | Oxidation (M)[6] | | Mascot |
| 1683.925 | 1683.7981 | -0.1269 | -75 | 546 | 559 | TLCTQGLQRIFYLK | | | | | Mascot |
| 1796.7261 | 1796.6711 | -0.055 | -31 | 239 | 254 | CAVCGDNASCQHYGVR | | | Carbamidomethyl (C)[1,4] | | Mascot |
| 1924.9835 | 1924.9486 | -0.0349 | -18 | 519 | 536 | DHVSTSALEVGRPNSLSR | | | | | Mascot |
| 3337.5066 | 3337.7131 | 0.2065 | 62 | 2 | 30 | TCVQSQHGVQSYESSLFSSEFLNPDFTSR | | | Carbamidomethyl (C)[2] | | Mascot |

30 PREDICTED: interferon-induced very large GTPase 1-like [Danio rerio] gi|326665761 191677.2 6.42 21 34 0

Peptide Information

| Calc. Mass | Obsrv. Mass | ± da | ± ppm | Start Seq. | End Seq. | Sequence | Ion Score | C. I. % | Modification | Rank | Result Type |
|---|---|---|---|---|---|---|---|---|---|---|---|
| 842.473 | 842.4943 | 0.0213 | 25 | 920 | 927 | SIISHASK | | | | | Mascot |
| 848.4413 | 848.5095 | 0.0682 | 80 | 975 | 980 | WSWTLR | | | | | Mascot |
| 864.4573 | 864.4702 | 0.0129 | 15 | 163 | 169 | DAFKQQK | | | | | Mascot |
| 875.4217 | 875.4468 | 0.0251 | 29 | 1199 | 1205 | EDQSQIR | | | | | Mascot |
| 970.5679 | 970.491 | -0.0769 | -79 | 919 | 927 | KSIISHASK | | | | | Mascot |
| 1019.5554 | 1019.5233 | -0.0321 | -31 | 1128 | 1135 | IKDIDIMR | | | Oxidation (M)[7] | | Mascot |
| 1022.5121 | 1022.4899 | -0.0222 | -22 | 529 | 536 | TEMMNIRK | | | | | Mascot |

| Calc. Mass | Obsrv. Mass | ± da | ± ppm | Start Seq. | End Seq. | Sequence | Ion Score | C. I. % | Modification | Rank | Result Type |
|---|---|---|---|---|---|---|---|---|---|---|---|
| 1045.5887 | 1045.5457 | -0.043 | -41 | 613 | 621 | QAELEKISK | | | | | Mascot |
| 1051.5419 | 1051.6937 | 0.1518 | 144 | 1623 | 1630 | NLEQKYEK | | | | | Mascot |
| 1092.5571 | 1092.5464 | -0.0107 | -10 | 1328 | 1336 | EPIEQSVYK | | | | | Mascot |
| 1456.739 | 1456.6481 | -0.0909 | -62 | 599 | 610 | EQKDNNNKPELK | | | | | Mascot |
| 1472.6937 | 1472.6449 | -0.0488 | -33 | 636 | 648 | EISQIYESCSSVK | | | | | Mascot |
| 1472.6937 | 1472.6449 | -0.0488 | -33 | 636 | 648 | EISQIYESCSSVK | | | | | Mascot |
| 1587.7948 | 1587.8539 | 0.0591 | 37 | 622 | 635 | DLQAAAFGLEHIMR | | | Oxidation (M)[13] | | Mascot |
| 1625.7476 | 1625.7368 | -0.0108 | -7 | 870 | 883 | DEVCDAERFSDVIK | | | | | Mascot |
| 1662.8195 | 1662.7861 | -0.0334 | -20 | 509 | 522 | NKELHRPQGDEPDK | | | | | Mascot |
| 1865.8956 | 1865.8141 | -0.0815 | -44 | 1578 | 1592 | QFLESDVSFPYKDYK | | | | | Mascot |
| 1865.8956 | 1865.8141 | -0.0815 | -44 | 1578 | 1592 | QFLESDVSFPYKDYK | | | | | Mascot |
| 2152.0544 | 2152.0601 | 0.0057 | 3 | 492 | 508 | ESFLPHQGKLWHQWCQK | | | | | Mascot |
| 2152.0544 | 2152.0601 | 0.0057 | 3 | 492 | 508 | ESFLPHQGKLWHQWCQK | | | | | Mascot |
| 2194.0129 | 2194.0471 | 0.0342 | 16 | 198 | 216 | IDPMDVQMAVFHCADGFLK | | | Carbamidomethyl (C)[13] | | Mascot |
| 2225.1033 | 2225.0793 | -0.024 | -11 | 293 | 310 | SHLMNSLINEKHNTFFHR | | | | | Mascot |
| 2225.1033 | 2225.0793 | -0.024 | -11 | 293 | 310 | SHLMNSLINEKHNTFFHR | | | | | Mascot |
| 2230.0444 | 2230.1414 | 0.097 | 43 | 170 | 190 | TDTGSTSHGGDIFKAMSLYNK | | | | | Mascot |
| 3337.7024 | 3337.7131 | 0.0107 | 3 | 387 | 414 | IIQELFTNPKPLICLFTEDDSNIFELER | | | | | Mascot |

| | | | | | | | |
|---|---|---|---|---|---|---|---|
| 31 | sarcosine dehydrogenase, mitochondrial [Danio rerio] | gi\|41055040 | 74649.1 | 6.04 | 11 | 34 | 0 |

**Peptide Information**

| Calc. Mass | Obsrv. Mass | ± da | ± ppm | Start Seq. | End Seq. | Sequence | Ion Score | C. I. % | Modification | Rank | Result Type |
|---|---|---|---|---|---|---|---|---|---|---|---|
| 823.4308 | 823.399 | -0.0318 | -39 | 159 | 164 | LDEYKR | | | | | Mascot |
| 970.5138 | 970.491 | -0.0228 | -23 | 16 | 23 | QPLSPLCR | | | Carbamidomethyl (C)[7] | | Mascot |
| 1092.5758 | 1092.5464 | -0.0294 | -27 | 186 | 194 | DLYPLMNVK | | | | | Mascot |
| 1371.5846 | 1371.6129 | 0.0283 | 21 | 28 | 39 | CYSSQSTHTTEK | | | | | Mascot |
| 1399.7474 | 1399.6824 | -0.065 | -46 | 16 | 27 | QPLSPLCRATTR | | | Carbamidomethyl (C)[7] | | Mascot |
| 1475.7853 | 1475.6984 | -0.0869 | -59 | 49 | 62 | HEDGLSGPTKPLPK | | | | | Mascot |
| 1556.7196 | 1556.7091 | -0.0105 | -7 | 511 | 524 | APGSTVYTCMLNQR | | | Oxidation (M)[10] | | Mascot |
| 1587.7916 | 1587.8539 | 0.0623 | 39 | 11 | 23 | AMVCRQPLSPLCR | | | Carbamidomethyl (C)[4,12] | | Mascot |
| 1625.8038 | 1625.7368 | -0.067 | -41 | 2 | 15 | AFLGTAAWRAMVCR | | | Carbamidomethyl (C)[13], Oxidation (M)[11] | | Mascot |
| 1683.828 | 1683.7981 | -0.0299 | -18 | 1 | 15 | MAFLGTAAWRAMVCR | | | | | Mascot |
| 2120.9661 | 2121.1003 | 0.1342 | 63 | 581 | 599 | CTLIDHTEDMGMISIQGPK | | | Oxidation (M)[10,12] | | Mascot |

| | | | | | | | |
|---|---|---|---|---|---|---|---|
| 32 | synaptosome-associated protein 25a [Danio rerio] | gi\|55251216 | 22862.9 | 4.65 | 8 | 34 | 0 |

Peptide Information

| Calc. Mass | Obsrv. Mass | ± da | ± ppm | Start Seq. | End Seq. | Sequence | Ion Score | C. I. % | Modification | Rank | Result Type |
|---|---|---|---|---|---|---|---|---|---|---|---|
| 823.325 | 823.399 | 0.074 | 90 | 2 | 8 | AEDSDMR | | | | | Mascot |
| 970.3604 | 970.491 | 0.1306 | 135 | 1 | 8 | MAEDSDMR | | | Oxidation (M)[1] | | Mascot |
| 1092.5605 | 1092.5464 | -0.0141 | -13 | 32 | 40 | MLQLVEESK | | | Oxidation (M)[1] | | Mascot |
| 1371.5676 | 1371.6129 | 0.0453 | 33 | 84 | 94 | CCGLFICPCNK | | | Carbamidomethyl (C)[1,2,7] | | Mascot |
| 1475.6335 | 1475.6984 | 0.0649 | 44 | 84 | 96 | CCGLFICPCNKMK | | | Oxidation (M)[12] | | Mascot |
| 1662.8003 | 1662.7861 | -0.0142 | -9 | 46 | 59 | TLVMLDEQGEQLDR | | | Oxidation (M)[4] | | Mascot |
| 1924.8124 | 1924.9486 | 0.1362 | 71 | 2 | 17 | AEDSDMRNELADMQQR | | | Oxidation (M)[6] | | Mascot |
| 2071.8477 | 2072.0278 | 0.1801 | 87 | 1 | 17 | MAEDSDMRNELADMQQ R | | | Oxidation (M)[1,7] | | Mascot |

| | | | | | | | |
|---|---|---|---|---|---|---|---|
| 33 | hypothetical protein LOC553738 [Danio rerio] | gi\|66472320 | 27546.3 | 9.57 | 9 | 33 | 0 |

Peptide Information

| Calc. Mass | Obsrv. Mass | ± da | ± ppm | Start Seq. | End Seq. | Sequence | Ion Score | C. I. % | Modification | Rank | Result Type |
|---|---|---|---|---|---|---|---|---|---|---|---|
| 853.4413 | 853.3448 | -0.0965 | -113 | 223 | 230 | FSQSGSLK | | | | | Mascot |
| 1047.6097 | 1047.5 | -0.1097 | -105 | 143 | 150 | SFLKFHLR | | | | | Mascot |
| 1165.6212 | 1165.7224 | 0.1012 | 87 | 194 | 203 | TFVTADDLKR | | | | | Mascot |
| 1165.6212 | 1165.7224 | 0.1012 | 87 | 194 | 203 | TFVTADDLKR | | | | | Mascot |
| 1865.9031 | 1865.8141 | -0.089 | -48 | 10 | 25 | MVRTGEKPITCTQCGK | | | Carbamidomethyl (C)[11,14] | | Mascot |
| 1865.9031 | 1865.8141 | -0.089 | -48 | 10 | 25 | MVRTGEKPITCTQCGK | | | Carbamidomethyl (C)[11,14] | | Mascot |
| 1961.8779 | 1961.9539 | 0.076 | 39 | 207 | 222 | IHTGEKPYMCSHCDKR | | | Carbamidomethyl (C)[10] | | Mascot |
| 2120.9497 | 2121.1003 | 0.1506 | 71 | 64 | 81 | HMMIHTGERPFICTQCGK | | | Oxidation (M)[2,3] | | Mascot |
| 2233.0752 | 2233.0454 | -0.0298 | -13 | 147 | 165 | FHLRVHTNERPYSCSVC GK | | | | | Mascot |
| 2284.0168 | 2284.1365 | 0.1197 | 52 | 204 | 221 | HQRIHTGEKPYMCSHCD K | | | Carbamidomethyl (C)[13,16] | | Mascot |
| 2298.0171 | 2298.1453 | 0.1282 | 56 | 151 | 169 | VHTNERPYSCSVCGKSC TR | | | Carbamidomethyl (C)[10,13,17] | | Mascot |
| 2300.0117 | 2300.1404 | 0.1287 | 56 | 204 | 221 | HQRIHTGEKPYMCSHCD K | | | Carbamidomethyl (C)[13,16], Oxidation (M)[12] | | Mascot |

| | | | | | | | |
|---|---|---|---|---|---|---|---|
| 34 | immunoglobulin heavy chain variable segment 9-2 [Danio rerio] | gi\|56207380 | 12773.2 | 7.57 | 5 | 33 | 0 |

Peptide Information

| Calc. Mass | Obsrv. Mass | ± da | ± ppm | Start Seq. | End Seq. | Sequence | Ion Score | C. I. % | Modification | Rank | Result Type |
|---|---|---|---|---|---|---|---|---|---|---|---|
| 1467.755 | 1467.726 | -0.029 | -20 | 77 | 90 | STAQSVQGRFTASK | | | | | Mascot |
| 1578.7581 | 1578.7957 | 0.0376 | 24 | 91 | 103 | DSSNLYLHMNQLK | | | Oxidation (M)[9] | | Mascot |

| Calc. Mass | Obsrv. Mass | ± da | ± ppm | Start Seq. | End Seq. | Sequence | Ion Score | C. I. % | Modification | Rank | Result Type |
|---|---|---|---|---|---|---|---|---|---|---|---|
| 2097.0435 | 2097.0923 | 0.0488 | 23 | 86 | 103 | FTASKDSSNLYLHMNQLK | | | | | Mascot |
| 2097.0435 | 2097.0923 | 0.0488 | 23 | 86 | 103 | FTASKDSSNLYLHMNQLK | | | | | Mascot |
| 2225.03 | 2225.0793 | 0.0493 | 22 | 39 | 57 | LSCACSGFTLRSTNMYWIR | | | Oxidation (M)[15] | | Mascot |
| 2225.03 | 2225.0793 | 0.0493 | 22 | 39 | 57 | LSCACSGFTLRSTNMYWIR | | | Oxidation (M)[15] | | Mascot |
| 2233.0811 | 2233.0454 | -0.0357 | -16 | 58 | 76 | QAPGKGLEWIIYYYSDSDK | | | | | Mascot |

35 contactin-1a precursor [Danio rerio] gi|136256388 114246.1 6.19 13 32 0

**Peptide Information**

| Calc. Mass | Obsrv. Mass | ± da | ± ppm | Start Seq. | End Seq. | Sequence | Ion Score | C. I. % | Modification | Rank | Result Type |
|---|---|---|---|---|---|---|---|---|---|---|---|
| 875.437 | 875.4468 | 0.0098 | 11 | 74 | 81 | ANPPASYR | | | | | Mascot |
| 987.5118 | 987.5167 | 0.0049 | 5 | 383 | 390 | GHEVRFSR | | | | | Mascot |
| 987.5118 | 987.5167 | 0.0049 | 5 | 383 | 390 | GHEVRFSR | | | | | Mascot |
| 1045.5173 | 1045.5457 | 0.0284 | 27 | 562 | 569 | DAQHYQRK | | | | | Mascot |
| 1062.5513 | 1062.674 | 0.1227 | 115 | 442 | 449 | VVFECRPR | | | Carbamidomethyl (C)[5] | | Mascot |
| 1578.8082 | 1578.7957 | -0.0125 | -8 | 501 | 516 | GRANSTGSLSITDATK | | | | | Mascot |
| 1796.9249 | 1796.6711 | -0.2538 | -141 | 717 | 735 | EARPIVAPSDIGGGGGTSR | | | | | Mascot |
| 1815.9058 | 1815.8549 | -0.0509 | -28 | 883 | 897 | LDNMKPDSHYLVEVR | | | | | Mascot |
| 2071.925 | 2072.0278 | 0.1028 | 50 | 116 | 134 | HAGNYTCVASNQYGSVTSR | | | Carbamidomethyl (C)[7] | | Mascot |
| 2224.9749 | 2225.0793 | 0.1044 | 47 | 388 | 406 | FSRLGFEDSGMYQCVAENR | | | Oxidation (M)[11] | | Mascot |
| 2225.1772 | 2225.0793 | -0.0979 | -44 | 695 | 716 | VIATNTLGTGPPSEPSPKTTTR | | | | | Mascot |
| 2230.0305 | 2230.1414 | 0.1109 | 50 | 114 | 134 | SKHAGNYTCVASNQYGSVTSR | | | | | Mascot |
| 2283.092 | 2283.1443 | 0.0523 | 23 | 594 | 614 | YSCTAQTPVDNTTASAELVVR | | | Carbamidomethyl (C)[3] | | Mascot |
| 2283.092 | 2283.1443 | 0.0523 | 23 | 594 | 614 | YSCTAQTPVDNTTASAELVVR | | | Carbamidomethyl (C)[3] | | Mascot |
| 2284.2131 | 2284.1365 | -0.0766 | -34 | 442 | 460 | VVFECRPRAAPRPNITWSK | | | Carbamidomethyl (C)[5] | | Mascot |

36 WD repeat domain 8 [Danio rerio] gi|159155126 50930.5 6.22 9 32 0

**Peptide Information**

| Calc. Mass | Obsrv. Mass | ± da | ± ppm | Start Seq. | End Seq. | Sequence | Ion Score | C. I. % | Modification | Rank | Result Type |
|---|---|---|---|---|---|---|---|---|---|---|---|
| 853.4236 | 853.3448 | -0.0788 | -92 | 147 | 153 | YMALAER | | | | | Mascot |
| 870.4355 | 870.5259 | 0.0904 | 104 | 2 | 8 | NFSEVFK | | | | | Mascot |
| 1279.7633 | 1279.7505 | -0.0128 | -10 | 247 | 256 | VRILNHITWK | | | | | Mascot |
| 1399.6093 | 1399.6824 | 0.0731 | 52 | 134 | 146 | ACQKGMDFTADGR | | | | | Mascot |

| | | | | | | | | | | | |
|---|---|---|---|---|---|---|---|---|---|---|---|
| 1456.6307 | 1456.6481 | 0.0174 | 12 | 134 | 146 | ACQKGMDFTADGR | | | Carbamidomethyl (C)[2] | | Mascot |
| 1472.6256 | 1472.6449 | 0.0193 | 13 | 134 | 146 | ACQKGMDFTADGR | | | Carbamidomethyl (C)[2], Oxidation (M)[6] | | Mascot |
| 1472.6256 | 1472.6449 | 0.0193 | 13 | 134 | 146 | ACQKGMDFTADGR | | | Carbamidomethyl (C)[2], Oxidation (M)[6] | | Mascot |
| 1587.8013 | 1587.8539 | 0.0526 | 33 | 258 | 271 | ITEFEHPATITNSK | | | | | Mascot |
| 1865.8309 | 1865.8141 | -0.0168 | -9 | 347 | 361 | NGNMPQSLWVWDMQK | | | Oxidation (M)[4,13] | | Mascot |
| 1865.8309 | 1865.8141 | -0.0168 | -9 | 347 | 361 | NGNMPQSLWVWDMQK | | | Oxidation (M)[4,13] | | Mascot |
| 1924.9374 | 1924.9486 | 0.0112 | 6 | 71 | 86 | GLVQVWSLEQPDWHCK | | | | | Mascot |
| 2071.9971 | 2072.0278 | 0.0307 | 15 | 228 | 246 | SVAWSPSSQFLAIGSYDEK | | | | | Mascot |
| 2284.0525 | 2284.1365 | 0.084 | 37 | 155 | 172 | DCKDYISVFVCDDWHLLR | | | Carbamidomethyl (C)[2] | | Mascot |

37 PREDICTED: hypothetical protein LOC569436 [Danio rerio] gi|326678363 352771.2 7.92 26 32 0

**Peptide Information**

| Calc. Mass | Obsrv. Mass | ± da | ± ppm | Start Seq. | End Seq. | Sequence | Ion Score | C. I. % | Modification | Rank | Result Type |
|---|---|---|---|---|---|---|---|---|---|---|---|
| 823.3549 | 823.399 | 0.0441 | 54 | 401 | 407 | CEQCGKR | | | | | Mascot |
| 836.3818 | 836.3209 | -0.0609 | -73 | 2995 | 3002 | LGDSGMEK | | | | | Mascot |
| 842.4743 | 842.4943 | 0.02 | 24 | 2664 | 2669 | HFRINR | | | | | Mascot |
| 864.4131 | 864.4702 | 0.0571 | 66 | 603 | 610 | LGAEMSEK | | | | | Mascot |
| 875.4655 | 875.4468 | -0.0187 | -21 | 2205 | 2211 | NIVCELK | | | Carbamidomethyl (C)[4] | | Mascot |
| 987.4677 | 987.5167 | 0.049 | 50 | 94 | 101 | QTLSDHMR | 7 | 0 | | | Mascot |
| 987.4677 | 987.5167 | 0.049 | 50 | 94 | 101 | QTLSDHMR | | | | | Mascot |
| 1047.6058 | 1047.5 | -0.1058 | -101 | 1369 | 1376 | FQILQSRR | | | | | Mascot |
| 1062.55 | 1062.674 | 0.124 | 117 | 1488 | 1496 | NESMIDLLK | | | | | Mascot |
| 1092.6122 | 1092.5464 | -0.0658 | -60 | 2545 | 2554 | LFVGDMGILK | | | | | Mascot |
| 1165.5483 | 1165.7224 | 0.1741 | 149 | 922 | 932 | SKGDGPNFSEK | | | | | Mascot |
| 1165.5483 | 1165.7224 | 0.1741 | 149 | 922 | 932 | SKGDGPNFSEK | | | | | Mascot |
| 1456.6962 | 1456.6481 | -0.0481 | -33 | 94 | 105 | QTLSDHMRTHSK | | | Oxidation (M)[7] | | Mascot |
| 1467.8318 | 1467.726 | -0.1058 | -72 | 491 | 503 | TFAGPENLKIHLK | | | | | Mascot |
| 1475.647 | 1475.6984 | 0.0514 | 35 | 1333 | 1345 | RNNENNQADDASK | | | | | Mascot |
| 1578.703 | 1578.7957 | 0.0927 | 59 | 854 | 868 | GDGPNFSEQQPSSTK | | | | | Mascot |
| 1625.7523 | 1625.7368 | -0.0155 | -10 | 1 | 13 | MHSAHQEEQKCIK | | | Carbamidomethyl (C)[11] | | Mascot |
| 1683.7512 | 1683.7981 | 0.0469 | 28 | 130 | 144 | KHCGNAQHICDLCGK | | | Carbamidomethyl (C)[3] | | Mascot |
| 1815.8629 | 1815.8549 | -0.008 | -4 | 527 | 541 | ANYRFVNIQDVCAMR | | | Oxidation (M)[14] | | Mascot |
| 1865.9174 | 1865.8141 | -0.1033 | -55 | 53 | 68 | HHVKPEEISLSCSQSK | | | Carbamidomethyl (C)[12] | | Mascot |
| 1865.9174 | 1865.8141 | -0.1033 | -55 | 53 | 68 | HHVKPEEISLSCSQSK | | | Carbamidomethyl (C)[12] | | Mascot |
| 2120.877 | 2121.1003 | 0.2233 | 105 | 238 | 255 | HMCIHSGEKPYACDQCG | | | Carbamidomethyl (C)[3,13] | | Mascot |

| Calc. Mass | Obsrv. Mass | ± da | ± ppm | Start Seq. | End Seq. | Sequence | Ion Score | C. I. % | Modification | Rank | Result Type |
|---|---|---|---|---|---|---|---|---|---|---|---|
| | | | | | | K | | | | | |
| 2151.9521 | 2152.0601 | 0.108 | 50 | 131 | 149 | HCGNAQHICDLCGKSFSFK | | | Carbamidomethyl (C)[2] | | Mascot |
| 2151.9521 | 2152.0601 | 0.108 | 50 | 131 | 149 | HCGNAQHICDLCGKSFSFK | | | Carbamidomethyl (C)[2] | | Mascot |
| 2193.8933 | 2194.0471 | 0.1538 | 70 | 238 | 255 | HMCIHSGEKPYACDQCGK | | | Carbamidomethyl (C)[3,13,16], Oxidation (M)[2] | | Mascot |
| 2224.9797 | 2225.0793 | 0.0996 | 45 | 321 | 339 | HHMSLHNEEKPYVCGQCGR | | | | | Mascot |
| 2224.9797 | 2225.0793 | 0.0996 | 45 | 321 | 339 | HHMSLHNEEKPYVCGQCGR | | | | | Mascot |
| 2232.8818 | 2233.0454 | 0.1636 | 73 | 181 | 199 | YHMKMHSDETAFSCEECGK | | | | | Mascot |
| 2283.2068 | 2283.1443 | -0.0625 | -27 | 1358 | 1375 | TLTQMYTHFLRFQILQSR | | | | | Mascot |
| 2283.2068 | 2283.1443 | -0.0625 | -27 | 1358 | 1375 | TLTQMYTHFLRFQILQSR | | | | | Mascot |
| 2297.144 | 2297.1523 | 0.0083 | 4 | 2728 | 2748 | LNLSDCSISEEGYKALASALR | | | Carbamidomethyl (C)[6] | | Mascot |
| 2297.9961 | 2298.1453 | 0.1492 | 65 | 321 | 339 | HHMSLHNEEKPYVCGQCGR | | | Carbamidomethyl (C)[14], Oxidation (M)[3] | | Mascot |
| 2306.1003 | 2306.1123 | 0.012 | 5 | 1875 | 1895 | ITEDDCCVLISALNSNPSNLK | | | Carbamidomethyl (C)[6] | | Mascot |
| 3387.5833 | 3387.6663 | 0.083 | 25 | 1409 | 1438 | LLFYEKDLEDCGIDVSEASVFSGMCTQIFK | | | | | Mascot |

38 PREDICTED: hypothetical protein LOC558327 [Danio rerio] gi|326678977 53299.3 6.37 9 32 0

**Peptide Information**

| Calc. Mass | Obsrv. Mass | ± da | ± ppm | Start Seq. | End Seq. | Sequence | Ion Score | C. I. % | Modification | Rank | Result Type |
|---|---|---|---|---|---|---|---|---|---|---|---|
| 848.422 | 848.5095 | 0.0875 | 103 | 10 | 17 | GGTQTQTR | | | | | Mascot |
| 882.5155 | 882.5498 | 0.0343 | 39 | 132 | 138 | HVETLRK | | | | | Mascot |
| 987.4602 | 987.5167 | 0.0565 | 57 | 211 | 219 | SQTHSNASR | | | | | Mascot |
| 987.4602 | 987.5167 | 0.0565 | 57 | 211 | 219 | SQTHSNASR | | | | | Mascot |
| 1047.5793 | 1047.5 | -0.0793 | -76 | 362 | 370 | LSSRSIQEK | | | | | Mascot |
| 1092.5354 | 1092.5464 | 0.011 | 10 | 392 | 401 | TVCLADEVSR | | | | | Mascot |
| 1796.9324 | 1796.6711 | -0.2613 | -145 | 386 | 401 | SPVVHKTVCLADEVSR | | | Carbamidomethyl (C)[9] | | Mascot |
| 1815.8242 | 1815.8549 | 0.0307 | 17 | 141 | 156 | EEGEEGEKDILDAEPR | | | | | Mascot |
| 2071.9568 | 2072.0278 | 0.071 | 34 | 178 | 196 | NIFSPEPPTNSTDTETHGK | | | | | Mascot |
| 2104.0669 | 2103.949 | -0.1179 | -56 | 443 | 461 | TFNISSSPTDLRHVDISSK | | | | | Mascot |

39 PREDICTED: UPF0639 protein-like [Danio rerio] gi|326675781 53022.8 5.16 9 32 0

**Peptide Information**

| Calc. Mass | Obsrv. Mass | ± da | ± ppm | Start Seq. | End Seq. | Sequence | Ion Score | C. I. % | Modification | Rank | Result Type |
|---|---|---|---|---|---|---|---|---|---|---|---|
| 823.5188 | 823.399 | -0.1198 | -145 | 51 | 56 | RFFIIK | | | | | Mascot |
| 853.4413 | 853.3448 | -0.0965 | -113 | 69 | 75 | KSFETNK | | | | | Mascot |

| Calc. Mass | Obsrv. Mass | ± da | ± ppm | Start Seq. | End Seq. | Sequence | Ion Score | C. I. % | Modification | Rank | Result Type |
|---|---|---|---|---|---|---|---|---|---|---|---|
| 875.4468 | 875.4468 | 0 | 0 | 211 | 217 | TEQEQIK | | | | | Mascot |
| 882.4866 | 882.5498 | 0.0632 | 72 | 443 | 449 | AYKIMTR | | | | | Mascot |
| 1062.5314 | 1062.674 | 0.1426 | 134 | 247 | 255 | SLEELSQEK | | | | | Mascot |
| 1371.7478 | 1371.6129 | -0.1349 | -98 | 191 | 201 | LNQLLEEEKQK | | | | | Mascot |
| 1951.9099 | 1951.9774 | 0.0675 | 35 | 171 | 185 | LMEETEELCLQREQK | | | Carbamidomethyl (C)[9], Oxidation (M)[2] | | Mascot |
| 2230.1384 | 2230.1414 | 0.003 | 1 | 142 | 162 | NAQLGEAMIESLEAQGL QLAK | | | Oxidation (M)[8] | | Mascot |
| 2283.219 | 2283.1443 | -0.0747 | -33 | 370 | 389 | LKLAEEALQDLEQGLNTI ER | | | | | Mascot |
| 2283.219 | 2283.1443 | -0.0747 | -33 | 370 | 389 | LKLAEEALQDLEQGLNTI ER | | | | | Mascot |

| | | | | | | | |
|---|---|---|---|---|---|---|---|
| 40 | immunoglobulin mu heavy chain [Danio rerio] | gi\|51559357 | 23906.6 | 8.05 | 6 | 32 | 0 |

**Peptide Information**

| Calc. Mass | Obsrv. Mass | ± da | ± ppm | Start Seq. | End Seq. | Sequence | Ion Score | C. I. % | Modification | Rank | Result Type |
|---|---|---|---|---|---|---|---|---|---|---|---|
| 864.4097 | 864.4702 | 0.0605 | 70 | 209 | 215 | SDWIDTK | | | | | Mascot |
| 1467.755 | 1467.726 | -0.029 | -20 | 77 | 90 | STAQSVQGRFTASK | | | | | Mascot |
| 1578.7581 | 1578.7957 | 0.0376 | 24 | 91 | 103 | DSSNLYLHMNQLK | | | Oxidation (M)[9] | | Mascot |
| 2097.0435 | 2097.0923 | 0.0488 | 23 | 86 | 103 | FTASKDSSNLYLHMNQL K | | | | | Mascot |
| 2097.0435 | 2097.0923 | 0.0488 | 23 | 86 | 103 | FTASKDSSNLYLHMNQL K | | | | | Mascot |
| 2225.03 | 2225.0793 | 0.0493 | 22 | 39 | 57 | LSCACSGFTLRSTNMYWI R | | | Oxidation (M)[15] | | Mascot |
| 2225.03 | 2225.0793 | 0.0493 | 22 | 39 | 57 | LSCACSGFTLRSTNMYWI R | | | Oxidation (M)[15] | | Mascot |
| 2233.0811 | 2233.0454 | -0.0357 | -16 | 58 | 76 | QAPGKGLEWIIYYYSDSD K | | | | | Mascot |

| | | | | | | | |
|---|---|---|---|---|---|---|---|
| 41 | fascin 2A [Danio rerio] | gi\|184186129 | 54837.1 | 5.91 | 9 | 32 | 0 |

**Peptide Information**

| Calc. Mass | Obsrv. Mass | ± da | ± ppm | Start Seq. | End Seq. | Sequence | Ion Score | C. I. % | Modification | Rank | Result Type |
|---|---|---|---|---|---|---|---|---|---|---|---|
| 996.493 | 996.5763 | 0.0833 | 84 | 186 | 193 | TCDNRFLK | | | | | Mascot |
| 996.493 | 996.5763 | 0.0833 | 84 | 186 | 193 | TCDNRFLK | | | | | Mascot |
| 1092.5718 | 1092.5464 | -0.0254 | -23 | 1 | 11 | MSTNGISAALK | | | | | Mascot |
| 1165.6034 | 1165.7224 | 0.119 | 102 | 88 | 97 | FLIVMQSDGR | | | | | Mascot |
| 1165.6034 | 1165.7224 | 0.119 | 102 | 88 | 97 | FLIVMQSDGR | | | | | Mascot |
| 1294.5038 | 1294.5033 | -0.0005 | 0 | 77 | 87 | CEEEVPNTDCR | | | | | Mascot |
| 1815.9309 | 1815.8549 | -0.076 | -42 | 198 | 213 | LVMEHGPNTSFTLELK | | | | | Mascot |
| 2104.0742 | 2103.949 | -0.1252 | -60 | 198 | 216 | LVMEHGPNTSFTLELKSG K | | | Oxidation (M)[3] | | Mascot |
| 2230.1172 | 2230.1414 | 0.0242 | 11 | 194 | 213 | NDGKLVMEHGPNTSFTL | | | | | Mascot |

| Calc. Mass | Obsrv. Mass | ± da | ± ppm | Start Seq. | End Seq. | Sequence | Ion Score | C. I. % | Modification | Rank | Result Type |
|---|---|---|---|---|---|---|---|---|---|---|---|
| | | | | | | ELK | | | | | |
| 2246.9983 | 2247.0593 | 0.061 | 27 | 467 | 488 | GDQGGTLMGDGLSVNASSLWEH | | | Oxidation (M)[8] | | Mascot |
| 2283.2205 | 2283.1443 | -0.0762 | -33 | 2 | 22 | STNGISAALKLQFGLINHENR | | | | | Mascot |
| 2283.2205 | 2283.1443 | -0.0762 | -33 | 2 | 22 | STNGISAALKLQFGLINHENR | | | | | Mascot |

| | | | | | | | | |
|---|---|---|---|---|---|---|---|---|
| 42 | SH3 domain-binding protein 4-A [Danio rerio] | gi\|45387913 | 104947.5 | 7.73 | 13 | 32 | 0 | |

**Peptide Information**

| Calc. Mass | Obsrv. Mass | ± da | ± ppm | Start Seq. | End Seq. | Sequence | Ion Score | C. I. % | Modification | Rank | Result Type |
|---|---|---|---|---|---|---|---|---|---|---|---|
| 842.5822 | 842.4943 | -0.0879 | -104 | 634 | 640 | LIKTVIR | | | | | Mascot |
| 850.4338 | 850.5258 | 0.092 | 108 | 358 | 364 | LSNMEIK | | | Oxidation (M)[4] | | Mascot |
| 850.4338 | 850.5258 | 0.092 | 108 | 358 | 364 | LSNMEIK | | | Oxidation (M)[4] | | Mascot |
| 870.4726 | 870.5259 | 0.0533 | 61 | 1 | 7 | MAAHRIR | | | Oxidation (M)[1] | | Mascot |
| 1045.4796 | 1045.5457 | 0.0661 | 63 | 762 | 770 | AELDSEPER | | | | | Mascot |
| 1294.6637 | 1294.5033 | -0.1604 | -124 | 553 | 563 | KADEISDFTLR | | | | | Mascot |
| 1371.7161 | 1371.6129 | -0.1032 | -75 | 8 | 19 | VTNNNNVLPRCK | | | | | Mascot |
| 1815.8959 | 1815.8549 | -0.041 | -23 | 432 | 446 | HVSFSSNVWNHMVKK | | | Oxidation (M)[12] | | Mascot |
| 2097.0137 | 2097.0923 | 0.0786 | 37 | 373 | 390 | VSVMTRTESPMAEIVCVR | | | Carbamidomethyl (C)[16], Oxidation (M)[4,11] | | Mascot |
| 2097.0137 | 2097.0923 | 0.0786 | 37 | 373 | 390 | VSVMTRTESPMAEIVCVR | | | Carbamidomethyl (C)[16], Oxidation (M)[4,11] | | Mascot |
| 2121.0474 | 2121.1003 | 0.0529 | 25 | 744 | 761 | AFADALSYVNLPLTYFCR | | | Carbamidomethyl (C)[17] | | Mascot |
| 2230.2417 | 2230.1414 | -0.1003 | -45 | 813 | 831 | LIMDFVLLTTAVEVAPRWR | | | | | Mascot |
| 2284.2368 | 2284.1365 | -0.1003 | -44 | 463 | 484 | TVVAIFGHDCAPKTLLVSEVGK | | | | | Mascot |
| 2300.0686 | 2300.1404 | 0.0718 | 31 | 568 | 587 | DDQDCILAQFCVQTPQPPPK | | | Carbamidomethyl (C)[5] | | Mascot |
| 3337.6052 | 3337.7131 | 0.1079 | 32 | 269 | 298 | LARSCHDLESLGQNPGWGQTQPVETNIVCK | | | Carbamidomethyl (C)[5] | | Mascot |

| | | | | | | | | | | |
|---|---|---|---|---|---|---|---|---|---|---|
| 43 | polymerase I and transcript release factor isoform 2 [Danio rerio] | gi\|167621556 | 51742.2 | 5.07 | 3 | 32 | 0 | 24 | 6.271 | |

**Peptide Information**

| Calc. Mass | Obsrv. Mass | ± da | ± ppm | Start Seq. | End Seq. | Sequence | Ion Score | C. I. % | Modification | Rank | Result Type |
|---|---|---|---|---|---|---|---|---|---|---|---|
| 823.3978 | 823.399 | 0.0012 | 1 | 254 | 260 | GNMEKTK | | | Oxidation (M)[3] | | Mascot |
| 848.393 | 848.5095 | 0.1165 | 137 | 329 | 335 | MSVNPER | | | Oxidation (M)[1] | | Mascot |
| 987.5945 | 987.5167 | -0.0778 | -79 | 128 | 136 | VSVNVKSVR | 24 | 6.271 | | | Mascot |
| 987.5945 | 987.5167 | -0.0778 | -79 | 128 | 136 | VSVNVKSVR | | | | | Mascot |

| | | | | | | | | |
|---|---|---|---|---|---|---|---|---|
| 44 | odorant receptor, family F, subfamily 115, member 12 [Danio rerio] | gi\|82658220 | 35276.8 | 9.3 | 7 | 32 | 0 | |

**Peptide Information**

| Calc. Mass | Obsrv. Mass | ± da | ± ppm | Start Seq. | End Seq. | Sequence | Ion Score | C. I. % | Modification | Rank | Result Type |
|---|---|---|---|---|---|---|---|---|---|---|---|
| 850.4525 | 850.5258 | 0.0733 | 86 | 221 | 227 | IAMVCIK | | | Carbamidomethyl (C)[5], Oxidation (M)[3] | | Mascot |
| 850.4888 | 850.5258 | 0.037 | 44 | 301 | 307 | MLKLCVK | | | Oxidation (M)[1] | | Mascot |
| 996.4819 | 996.5763 | 0.0944 | 95 | 133 | 140 | YPTIMNNK | | | Oxidation (M)[5] | | Mascot |
| 996.4819 | 996.5763 | 0.0944 | 95 | 133 | 140 | YPTIMNNK | | | Oxidation (M)[5] | | Mascot |
| 1045.516 | 1045.5457 | 0.0297 | 28 | 83 | 91 | DLLTDPSER | | | | | Mascot |
| 1399.7792 | 1399.6824 | -0.0968 | -69 | 80 | 91 | LLKDLLTDPSER | | | | | Mascot |
| 1467.7698 | 1467.726 | -0.0438 | -30 | 133 | 144 | YPTIMNNKMIVK | | | Oxidation (M)[5] | | Mascot |
| 2298.1118 | 2298.1453 | 0.0335 | 15 | 167 | 186 | LSHCRSVIENHVCDNPSLFK | | | | | Mascot |

| | | | | | | | |
|---|---|---|---|---|---|---|---|
| 45 | Vasa protein [Danio rerio] | gi\|120537661 | 73965.2 | 5.4 | 11 | 32 | 0 |

**Peptide Information**

| Calc. Mass | Obsrv. Mass | ± da | ± ppm | Start Seq. | End Seq. | Sequence | Ion Score | C. I. % | Modification | Rank | Result Type |
|---|---|---|---|---|---|---|---|---|---|---|---|
| 823.4168 | 823.399 | -0.0178 | -22 | 90 | 97 | GGFRSGSR | | | | | Mascot |
| 853.4426 | 853.3448 | -0.0978 | -115 | 164 | 171 | GGFRGGFR | | | | | Mascot |
| 882.4315 | 882.5498 | 0.1183 | 134 | 651 | 658 | VFASTDSR | | | | | Mascot |
| 970.4913 | 970.491 | -0.0003 | 0 | 504 | 511 | TMVFVETK | | | Oxidation (M)[2] | | Mascot |
| 1371.5742 | 1371.6129 | 0.0387 | 28 | 412 | 422 | MLDMGFEPEMR | | | Oxidation (M)[1] | | Mascot |
| 1472.709 | 1472.6449 | -0.0641 | -44 | 513 | 525 | SADFIATFLCQEK | | | | | Mascot |
| 1472.709 | 1472.6449 | -0.0641 | -44 | 513 | 525 | SADFIATFLCQEK | | | | | Mascot |
| 1815.8582 | 1815.8549 | -0.0033 | -2 | 437 | 451 | QTLMFSATYPEDIQR | | | Oxidation (M)[4] | | Mascot |
| 1865.7532 | 1865.8141 | 0.0609 | 33 | 64 | 81 | SEIDENGSDGGWNGGESR | | | | | Mascot |
| 1865.7532 | 1865.8141 | 0.0609 | 33 | 64 | 81 | SEIDENGSDGGWNGGESR | | | | | Mascot |
| 1951.9952 | 1951.9774 | -0.0178 | -9 | 376 | 393 | GCNVLCATPGRLHDLIGR | | | Carbamidomethyl (C)[2] | | Mascot |
| 2230.0444 | 2230.1414 | 0.097 | 43 | 434 | 451 | EERQTLMFSATYPEDIQR | | | Oxidation (M)[7] | | Mascot |
| 3387.5969 | 3387.6663 | 0.0694 | 20 | 232 | 262 | YDDILVDVSGSNPPKAIMTFEEAGLCDSLSK | | | Carbamidomethyl (C)[26], Oxidation (M)[18] | | Mascot |

| | | | | | | | |
|---|---|---|---|---|---|---|---|
| 46 | novel protein similar to vertebrate spectrin, beta, non-erythrocytic 1 (SPTBN1) [Danio rerio] | gi\|220673237 | 79469.5 | 5.08 | 10 | 31 | 0 |

**Peptide Information**

| Calc. Mass | Obsrv. Mass | ± da | ± ppm | Start Seq. | End Seq. | Sequence | Ion Score | C. I. % | Modification | Rank | Result Type |
|---|---|---|---|---|---|---|---|---|---|---|---|
| 987.5217 | 987.5167 | -0.005 | -5 | 216 | 223 | ELDEVRAR | | | | | Mascot |
| 987.5217 | 987.5167 | -0.005 | -5 | 216 | 223 | ELDEVRAR | | | | | Mascot |

| Calc. Mass | Obsrv. Mass | ± da | ± ppm | Start Seq. | End Seq. | Sequence | Ion Score | C. I. % | Modification | Rank | Result Type |
|---|---|---|---|---|---|---|---|---|---|---|---|
| 1007.5996 | 1007.4591 | -0.1405 | -139 | 500 | 508 | LINQHAALK | | | | | Mascot |
| 1371.7566 | 1371.6129 | -0.1437 | -105 | 205 | 215 | KHMVALPLQYR | | | Oxidation (M)[3] | | Mascot |
| 1472.8108 | 1472.6449 | -0.1659 | -113 | 337 | 348 | WNSIVELVEQKK | | | | | Mascot |
| 1472.8108 | 1472.6449 | -0.1659 | -113 | 337 | 348 | WNSIVELVEQKK | | | | | Mascot |
| 1587.6921 | 1587.8539 | 0.1618 | 102 | 509 | 520 | EEIGRYEEDYER | | | | | Mascot |
| 1662.8228 | 1662.7861 | -0.0367 | -22 | 449 | 463 | RTLQGCEDSLTVAGR | | | Carbamidomethyl (C)[6] | | Mascot |
| 1924.983 | 1924.9486 | -0.0344 | -18 | 58 | 75 | MSILATQSCGKDLSSVLR | | | Oxidation (M)[1] | | Mascot |
| 2233.1934 | 2233.0454 | -0.148 | -66 | 377 | 397 | AIDATQYVGSDLGSVLAL QRR | | | | | Mascot |
| 2283.114 | 2283.1443 | 0.0303 | 13 | 166 | 185 | LVSSEDFGHDEYSTQSLL KK | | | | | Mascot |
| 2283.114 | 2283.1443 | 0.0303 | 13 | 166 | 185 | LVSSEDFGHDEYSTQSLL KK | | | | | Mascot |
| 3337.7273 | 3337.7131 | -0.0142 | -4 | 580 | 610 | QAEAFLNNQESALAHVEL PSTVETVEAAIKK | | | | | Mascot |

47 ADP/ATP translocase 1 [Danio rerio] gi|47550717 32667.2 9.74 7 31 0

**Peptide Information**

| Calc. Mass | Obsrv. Mass | ± da | ± ppm | Start Seq. | End Seq. | Sequence | Ion Score | C. I. % | Modification | Rank | Result Type |
|---|---|---|---|---|---|---|---|---|---|---|---|
| 848.4875 | 848.5095 | 0.022 | 26 | 98 | 105 | IFLGGVDK | | | | | Mascot |
| 996.4536 | 996.5763 | 0.1227 | 123 | 237 | 244 | RMMMQSGR | | | | | Mascot |
| 996.4536 | 996.5763 | 0.1227 | 123 | 237 | 244 | RMMMQSGR | | | | | Mascot |
| 1007.4978 | 1007.4591 | -0.0387 | -38 | 253 | 260 | GTIDCWKK | | | Carbamidomethyl (C)[5] | | Mascot |
| 1952.9933 | 1952.9709 | -0.0224 | -11 | 44 | 60 | QITADMQYKGIIDCVVR | | | | | Mascot |
| 2152.1648 | 2152.0601 | -0.1047 | -49 | 2 | 23 | SDAAISFLKDFLAGGVAA AISK | | | | | Mascot |
| 2152.1648 | 2152.0601 | -0.1047 | -49 | 2 | 23 | SDAAISFLKDFLAGGVAA AISK | | | | | Mascot |
| 2283.2053 | 2283.1443 | -0.061 | -27 | 1 | 23 | MSDAAISFLKDFLAGGVA AAISK | | | | | Mascot |
| 2283.2053 | 2283.1443 | -0.061 | -27 | 1 | 23 | MSDAAISFLKDFLAGGVA AAISK | | | | | Mascot |
| 2284.2236 | 2284.1365 | -0.0871 | -38 | 73 | 92 | GNLANVIRYFPTQALNFA FK | | | | | Mascot |

48 Ddx52 protein [Danio rerio] gi|81294184 66316.2 9.69 10 31 0

**Peptide Information**

| Calc. Mass | Obsrv. Mass | ± da | ± ppm | Start Seq. | End Seq. | Sequence | Ion Score | C. I. % | Modification | Rank | Result Type |
|---|---|---|---|---|---|---|---|---|---|---|---|
| 823.4645 | 823.399 | -0.0655 | -80 | 145 | 150 | IRHQNR | | | | | Mascot |
| 836.426 | 836.3209 | -0.1051 | -126 | 21 | 27 | FAKDAER | | | | | Mascot |
| 864.4573 | 864.4702 | 0.0129 | 15 | 259 | 266 | LSEGVGFR | | | | | Mascot |
| 870.5407 | 870.5259 | -0.0148 | -17 | 239 | 246 | ALIISPTR | | | | | Mascot |

| | | | | | | | | | | | |
|---|---|---|---|---|---|---|---|---|---|---|---|
| 1371.8181 | 1371.6129 | -0.2052 | -150 | 465 | 476 | IWVLICTALLAR | | | | | Mascot |
| 1625.8646 | 1625.7368 | -0.1278 | -79 | 171 | 183 | EYDLDPRIIHNIK | | | | | Mascot |
| 1961.9199 | 1961.9539 | 0.034 | 17 | 33 | 50 | SQSKDSSDQLSGISYFG R | | | | | Mascot |
| 2121.0459 | 2121.1003 | 0.0544 | 26 | 382 | 401 | NSAAETVEQQLLFVGSE NGK | | | | | Mascot |
| 2283.3223 | 2283.1443 | -0.178 | -78 | 407 | 426 | NLIKQGFLPPVLVFVQSID R | | | | | Mascot |
| 2283.3223 | 2283.1443 | -0.178 | -78 | 407 | 426 | NLIKQGFLPPVLVFVQSID R | | | | | Mascot |
| 2297.1604 | 2297.1523 | -0.0081 | -4 | 184 | 204 | AAGFQTPTPIQMQAVPL MMHK | | | | | Mascot |

| | | | | | | | | | |
|---|---|---|---|---|---|---|---|---|---|
| 49 | transitional endoplasmic reticulum ATPase-like [Danio rerio] | gi\|90093334 | 89437.9 | 5.44 | 11 | 31 | 0 | | |

**Peptide Information**

| Calc. Mass | Obsrv. Mass | ± da | ± ppm | Start Seq. | End Seq. | Sequence | Ion Score | C. I. % | Modification | Rank | Result Type |
|---|---|---|---|---|---|---|---|---|---|---|---|
| 823.3831 | 823.399 | 0.0159 | 19 | 291 | 297 | AFEEAEK | | | | | Mascot |
| 850.4417 | 850.5258 | 0.0841 | 99 | 563 | 569 | DVFDKAR | | | | | Mascot |
| 850.4417 | 850.5258 | 0.0841 | 99 | 563 | 569 | DVFDKAR | | | | | Mascot |
| 1467.8352 | 1467.726 | -0.1092 | -74 | 380 | 391 | LEILQIHTKNMK | | | | | Mascot |
| 1683.9462 | 1683.7981 | -0.1481 | -88 | 214 | 227 | QLAQIKEMVELPLR | | | Oxidation (M)[8] | | Mascot |
| 1796.8483 | 1796.6711 | -0.1772 | -99 | 680 | 695 | ITEGFSGADLTEICQR | | | Carbamidomethyl (C)[14] | | Mascot |
| 1922.9059 | 1922.9604 | 0.0545 | 28 | 67 | 83 | RQTVCIVLTDDTCGNER | | | | | Mascot |
| 1951.9218 | 1951.9774 | 0.0556 | 28 | 546 | 562 | GPELLTMWFGESEANVR | | | Oxidation (M)[7] | | Mascot |
| 2103.9954 | 2103.949 | -0.0464 | -22 | 775 | 796 | FPTAPKSGGGQGSSQGS GGHFR | | | | | Mascot |
| 2156.011 | 2155.9233 | -0.0877 | -41 | 680 | 698 | ITEGFSGADLTEICQRAC K | | | Carbamidomethyl (C)[14,18] | | Mascot |
| 2247.1172 | 2247.0593 | -0.0579 | -26 | 28 | 47 | LIVDEATNEDNSIVCLSQV K | | | Carbamidomethyl (C)[15] | | Mascot |
| 3387.6963 | 3387.6663 | -0.03 | -9 | 162 | 192 | AVEFKVVETDPTPHCIVA PDTIIHCEGEPIK | | | | | Mascot |

| | | | | | | | | | |
|---|---|---|---|---|---|---|---|---|---|
| 50 | polymerase I and transcript release factor isoform 1 [Danio rerio] | gi\|147898795 | 19874.3 | 5.41 | 1 | | | 24 | 6.271 |

**Peptide Information**

| Calc. Mass | Obsrv. Mass | ± da | ± ppm | Start Seq. | End Seq. | Sequence | Ion Score | C. I. % | Modification | Rank | Result Type |
|---|---|---|---|---|---|---|---|---|---|---|---|
| 987.5945 | 987.5167 | -0.0778 | -79 | 128 | 136 | VSVNVKSVR | 24 | 6.271 | | 4 | Mascot |

| Gel Idx/Pos | 181/H8 | Instr./Gel Origin | BA2060/110707A | Process Status | Analysis Succeeded |
|---|---|---|---|---|---|
| Plate [#] Name | [1] 31496 | Instrument Sample Name | | Spectra | 11 |

| Rank | Protein Name | Accession No. | Protein MW | Protein PI | Pep. Count | Protein Score | Protein Score C. I. % | Total Ion Score | Total Ion C. I. % |
|---|---|---|---|---|---|---|---|---|---|
| 1 | villin 2 [Danio rerio] | gi\|71892458 | 68683.2 | 5.7 | 12 | 196 | 100 | 164 | 100 |

Peptide Information

| Calc. Mass | Obsrv. Mass | ± da | ± ppm | Start Seq. | End Seq. | Sequence | Ion Score | C. I. % | Modification | Rank | Result Type |
|---|---|---|---|---|---|---|---|---|---|---|---|
| 1104.5836 | 1104.5546 | -0.029 | -26 | 238 | 246 | IGFPWSEIR | | | | | Mascot |
| 1104.5836 | 1104.5546 | -0.029 | -26 | 238 | 246 | IGFPWSEIR | 57 | 99.938 | | | Mascot |
| 1120.5634 | 1120.5374 | -0.026 | -23 | 575 | 583 | QRIDEFEAL | | | | | Mascot |
| 1182.5942 | 1182.5671 | -0.0271 | -23 | 264 | 273 | APDFVFYAPR | 66 | 99.993 | | | Mascot |
| 1182.5942 | 1182.5671 | -0.0271 | -23 | 264 | 273 | APDFVFYAPR | | | | | Mascot |
| 1233.6123 | 1233.5854 | -0.0269 | -22 | 172 | 180 | IQVWHEEHR | | | | | Mascot |
| 1233.6123 | 1233.5854 | -0.0269 | -22 | 172 | 180 | IQVWHEEHR | 42 | 98.074 | | | Mascot |
| 1310.6892 | 1310.6559 | -0.0333 | -25 | 263 | 273 | KAPDFVFYAPR | | | | | Mascot |
| 1391.6583 | 1391.7091 | 0.0508 | 37 | 317 | 327 | QMERAQLENEK | | | Oxidation (M)[2] | | Mascot |
| 1413.806 | 1413.7682 | -0.0378 | -27 | 527 | 539 | QLLALTSELAQAR | | | | | Mascot |
| 1457.7495 | 1457.7173 | -0.0322 | -22 | 140 | 151 | ELHRPGYLTSER | | | | | Mascot |
| 1531.7533 | 1531.7145 | -0.0388 | -25 | 360 | 371 | KVENELQEQMQR | | | | | Mascot |
| 1616.7448 | 1616.7039 | -0.0409 | -25 | 346 | 357 | ELMMQLYQFEER | | | | | Mascot |
| 1632.7396 | 1632.6975 | -0.0421 | -26 | 346 | 357 | ELMMQLYQFEER | | | Oxidation (M)[3] | | Mascot |
| 1648.7345 | 1648.6963 | -0.0382 | -23 | 346 | 357 | ELMMQLYQFEER | | | Oxidation (M)[3,4] | | Mascot |
| 1651.8174 | 1651.7683 | -0.0491 | -30 | 413 | 427 | SQEQLAAELAEYTAK | | | | | Mascot |
| 2207.123 | 2207.0408 | -0.0822 | -37 | 82 | 100 | AKFFPEDVADELIQDITQK | | | | | Mascot |

| Rank | Protein Name | Accession No. | Protein MW | Protein PI | Pep. Count | Protein Score | Protein Score C. I. % | Total Ion Score | Total Ion C. I. % |
|---|---|---|---|---|---|---|---|---|---|
| 2 | Ezr protein [Danio rerio] | gi\|50418501 | 40608.3 | 9.33 | 7 | 182 | 100 | 164 | 100 |

Peptide Information

| Calc. Mass | Obsrv. Mass | ± da | ± ppm | Start Seq. | End Seq. | Sequence | Ion Score | C. I. % | Modification | Rank | Result Type |
|---|---|---|---|---|---|---|---|---|---|---|---|
| 1104.5836 | 1104.5546 | -0.029 | -26 | 238 | 246 | IGFPWSEIR | | | | | Mascot |
| 1104.5836 | 1104.5546 | -0.029 | -26 | 238 | 246 | IGFPWSEIR | 57 | 99.938 | | | Mascot |
| 1182.5942 | 1182.5671 | -0.0271 | -23 | 264 | 273 | APDFVFYAPR | 66 | 99.993 | | | Mascot |
| 1182.5942 | 1182.5671 | -0.0271 | -23 | 264 | 273 | APDFVFYAPR | | | | | Mascot |
| 1233.6123 | 1233.5854 | -0.0269 | -22 | 172 | 180 | IQVWHEEHR | | | | | Mascot |

| Calc. Mass | Obsrv. Mass | ± da | ± ppm | Start Seq. | End Seq. | Sequence | Ion Score | C. I. % | Modification | Rank | Result Type |
|---|---|---|---|---|---|---|---|---|---|---|---|
| 1233.6123 | 1233.5854 | -0.0269 | -22 | 172 | 180 | IQVWHEEHR | 42 | 98.074 | | | Mascot |
| 1310.6892 | 1310.6559 | -0.0333 | -25 | 263 | 273 | KAPDFVFYAPR | | | | | Mascot |
| 1391.6583 | 1391.7091 | 0.0508 | 37 | 317 | 327 | QMERAQLENEK | | | Oxidation (M)[2] | | Mascot |
| 1457.7495 | 1457.7173 | -0.0322 | -22 | 140 | 151 | ELHRPGYLTSER | | | | | Mascot |
| 2207.123 | 2207.0408 | -0.0822 | -37 | 82 | 100 | AKFFPEDVADELIQDITQK | | | | | Mascot |

| | | | | | | | | | |
|---|---|---|---|---|---|---|---|---|---|
| 3 | Si:dkey-276i5.1 protein [Danio rerio] | gi\|66910298 | 86251.9 | 6.8 | 14 | 102 | 100 | 55 | 99.916 |

**Peptide Information**

| Calc. Mass | Obsrv. Mass | ± da | ± ppm | Start Seq. | End Seq. | Sequence | Ion Score | C. I. % | Modification | Rank | Result Type |
|---|---|---|---|---|---|---|---|---|---|---|---|
| 1042.5792 | 1042.557 | -0.0222 | -21 | 184 | 191 | NNILQVWR | | | | | Mascot |
| 1104.616 | 1104.5546 | -0.0614 | -56 | 335 | 343 | RLLEFNQGK | | | | | Mascot |
| 1104.616 | 1104.5546 | -0.0614 | -56 | 335 | 343 | RLLEFNQGK | | | | | Mascot |
| 1277.6267 | 1277.6538 | 0.0271 | 21 | 405 | 416 | AQTSGQSAQIMR | | | | | Mascot |
| 1391.743 | 1391.7091 | -0.0339 | -24 | 344 | 356 | LPFAAAQIGNSFR | | | | | Mascot |
| 1443.709 | 1443.6772 | -0.0318 | -22 | 609 | 619 | IMYSIFEHTFR | | | | | Mascot |
| 1626.7581 | 1626.7155 | -0.0426 | -26 | 476 | 489 | TSYGWIEIVGCADR | | | Carbamidomethyl (C)[11] | | Mascot |
| 1638.8553 | 1638.8115 | -0.0438 | -27 | 15 | 29 | SMLSLCSATSALLLR | | | Carbamidomethyl (C)[6], Oxidation (M)[2] | | Mascot |
| 1638.8553 | 1638.8115 | -0.0438 | -27 | 15 | 29 | SMLSLCSATSALLLR | | | Carbamidomethyl (C)[6], Oxidation (M)[2] | | Mascot |
| 1718.8306 | 1718.8324 | 0.0018 | 1 | 390 | 404 | FSNVADLDIMLYSSK | | | Oxidation (M)[10] | | Mascot |
| 1755.9122 | 1755.9348 | 0.0226 | 13 | 108 | 124 | AENAPDVDVSKAVAELK | | | | | Mascot |
| 2192.1709 | 2192.1182 | -0.0527 | -24 | 589 | 608 | TLHVEEVVPNVIEPSFGIG R | 55 | 99.916 | | | Mascot |
| 2192.1709 | 2192.1182 | -0.0527 | -24 | 589 | 608 | TLHVEEVVPNVIEPSFGIG R | | | | | Mascot |
| 2207.0437 | 2207.0408 | -0.0029 | -1 | 368 | 385 | VREFTMAEIEHFVDPNEK | | | Oxidation (M)[6] | | Mascot |
| 2221.1611 | 2221.103 | -0.0581 | -26 | 417 | 437 | LGDAVEQGVINNSVLGYF IGR | | | | | Mascot |
| 2230.1213 | 2230.1079 | -0.0134 | -6 | 386 | 404 | VHFKFSNVADLDIMLYSS K | | | Oxidation (M)[14] | | Mascot |
| 2298.9968 | 2299.1296 | 0.1328 | 58 | 539 | 557 | FAMEYLAICDECYITEQE K | | | | | Mascot |

| | | | | | | | | | |
|---|---|---|---|---|---|---|---|---|---|
| 4 | PREDICTED: glycyl-tRNA synthetase [Danio rerio] | gi\|189536638 | 86255.8 | 6.76 | 13 | 96 | 99.999 | 55 | 99.916 |

**Peptide Information**

| Calc. Mass | Obsrv. Mass | ± da | ± ppm | Start Seq. | End Seq. | Sequence | Ion Score | C. I. % | Modification | Rank | Result Type |
|---|---|---|---|---|---|---|---|---|---|---|---|
| 1042.5792 | 1042.557 | -0.0222 | -21 | 184 | 191 | NNILQVWR | | | | | Mascot |
| 1104.616 | 1104.5546 | -0.0614 | -56 | 335 | 343 | RLLEFNQGK | | | | | Mascot |
| 1104.616 | 1104.5546 | -0.0614 | -56 | 335 | 343 | RLLEFNQGK | | | | | Mascot |
| 1277.6267 | 1277.6538 | 0.0271 | 21 | 405 | 416 | AQTSGQSAQIMR | | | | | Mascot |

| | | | | | | | | | | | |
|---|---|---|---|---|---|---|---|---|---|---|---|
| 1391.743 | 1391.7091 | -0.0339 | -24 | 344 | 356 | LPFAAAQIGNSFR | | | | | Mascot |
| 1443.709 | 1443.6772 | -0.0318 | -22 | 609 | 619 | IMYSIFEHTFR | | | | | Mascot |
| 1626.7581 | 1626.7155 | -0.0426 | -26 | 476 | 489 | TSYGWIEIVGCADR | | | Carbamidomethyl (C)[11] | | Mascot |
| 1718.8306 | 1718.8324 | 0.0018 | 1 | 390 | 404 | FSNVADLDIMLYSSK | | | Oxidation (M)[10] | | Mascot |
| 1755.9122 | 1755.9348 | 0.0226 | 13 | 108 | 124 | AENAPDVDVSKAVAELK | | | | | Mascot |
| 2192.1709 | 2192.1182 | -0.0527 | -24 | 589 | 608 | TLHVEEVVPNVIEPSFGIGR | 55 | 99.916 | | | Mascot |
| 2192.1709 | 2192.1182 | -0.0527 | -24 | 589 | 608 | TLHVEEVVPNVIEPSFGIGR | | | | | Mascot |
| 2207.0437 | 2207.0408 | -0.0029 | -1 | 368 | 385 | VREFTMAEIEHFVDPNEK | | | Oxidation (M)[6] | | Mascot |
| 2221.1611 | 2221.103 | -0.0581 | -26 | 417 | 437 | LGDAVEQGVINNSVLGYFIGR | | | | | Mascot |
| 2230.1213 | 2230.1079 | -0.0134 | -6 | 386 | 404 | VHFKFSNVADLDIMLYSSK | | | Oxidation (M)[14] | | Mascot |
| 2298.9968 | 2299.1296 | 0.1328 | 58 | 539 | 557 | FAMEYLAICDECYITEQEK | | | | | Mascot |

5 ezrin like [Danio rerio] gi|66472810 70053.6 5.42 7 68 99.357 57 99.938

Peptide Information

| Calc. Mass | Obsrv. Mass | ± da | ± ppm | Start Seq. | End Seq. | Sequence | Ion Score | C. I. % | Modification | Rank | Result Type |
|---|---|---|---|---|---|---|---|---|---|---|---|
| 850.4199 | 850.5237 | 0.1038 | 122 | 582 | 588 | MGNTKQR | | | Oxidation (M)[1] | | Mascot |
| 850.4199 | 850.5237 | 0.1038 | 122 | 582 | 588 | MGNTKQR | | | Oxidation (M)[1] | | Mascot |
| 864.4977 | 864.4717 | -0.026 | -30 | 54 | 60 | GFLTWLK | | | | | Mascot |
| 1104.5836 | 1104.5546 | -0.029 | -26 | 238 | 246 | IGFPWSEIR | | | | | Mascot |
| 1104.5836 | 1104.5546 | -0.029 | -26 | 238 | 246 | IGFPWSEIR | 57 | 99.938 | | | Mascot |
| 1120.5634 | 1120.5374 | -0.026 | -23 | 587 | 595 | QRIDEFEAL | | | | | Mascot |
| 1391.6583 | 1391.7091 | 0.0508 | 37 | 317 | 327 | QMERAQLENEK | | | Oxidation (M)[2] | | Mascot |
| 1463.6794 | 1463.7169 | 0.0375 | 26 | 401 | 412 | EELARQAEDQMK | | | Oxidation (M)[11] | | Mascot |
| 1651.8174 | 1651.7683 | -0.0491 | -30 | 413 | 427 | TQEQLAADLAEYTAK | | | | | Mascot |

6 Ezrl protein [Danio rerio] gi|157422742 52248.2 7.62 6 66 98.908 57 99.938

Peptide Information

| Calc. Mass | Obsrv. Mass | ± da | ± ppm | Start Seq. | End Seq. | Sequence | Ion Score | C. I. % | Modification | Rank | Result Type |
|---|---|---|---|---|---|---|---|---|---|---|---|
| 864.4977 | 864.4717 | -0.026 | -30 | 54 | 60 | GFLTWLK | | | | | Mascot |
| 1042.6255 | 1042.557 | -0.0685 | -66 | 428 | 436 | LALLEEARK | | | | | Mascot |
| 1104.5836 | 1104.5546 | -0.029 | -26 | 238 | 246 | IGFPWSEIR | | | | | Mascot |
| 1104.5836 | 1104.5546 | -0.029 | -26 | 238 | 246 | IGFPWSEIR | 57 | 99.938 | | | Mascot |
| 1391.6583 | 1391.7091 | 0.0508 | 37 | 317 | 327 | QMERAQLENEK | | | Oxidation (M)[2] | | Mascot |
| 1463.6794 | 1463.7169 | 0.0375 | 26 | 401 | 412 | EELARQAEDQMK | | | Oxidation (M)[11] | | Mascot |

| Calc. Mass | Obsrv. Mass | ± da | ± ppm | Start Seq. | End Seq. | Sequence | Ion Score | C. I. % | Modification | Rank | Result Type |
|---|---|---|---|---|---|---|---|---|---|---|---|
| 1651.8174 | 1651.7683 | -0.0491 | -30 | 413 | 427 | TQEQLAADLAEYTAK | | | | | Mascot |

7 moesin [Danio rerio] gi|51972166 67990.8 5.84 3 63 97.665 57 99.938

Peptide Information

| Calc. Mass | Obsrv. Mass | ± da | ± ppm | Start Seq. | End Seq. | Sequence | Ion Score | C. I. % | Modification | Rank | Result Type |
|---|---|---|---|---|---|---|---|---|---|---|---|
| 1104.5836 | 1104.5546 | -0.029 | -26 | 238 | 246 | IGFPWSEIR | | | | | Mascot |
| 1104.5836 | 1104.5546 | -0.029 | -26 | 238 | 246 | IGFPWSEIR | 57 | 99.938 | | | Mascot |
| 1179.5562 | 1179.5767 | 0.0205 | 17 | 449 | 458 | ATMVQEDLEK | | | Oxidation (M)[3] | | Mascot |
| 1277.6154 | 1277.6538 | 0.0384 | 30 | 318 | 327 | MERALLEDER | | | Oxidation (M)[1] | | Mascot |

8 PREDICTED: moesin [Danio rerio] gi|326673460 67949.1 6.64 3 63 97.61 57 99.938

Peptide Information

| Calc. Mass | Obsrv. Mass | ± da | ± ppm | Start Seq. | End Seq. | Sequence | Ion Score | C. I. % | Modification | Rank | Result Type |
|---|---|---|---|---|---|---|---|---|---|---|---|
| 1104.5836 | 1104.5546 | -0.029 | -26 | 236 | 244 | IGFPWSEIR | | | | | Mascot |
| 1104.5836 | 1104.5546 | -0.029 | -26 | 236 | 244 | IGFPWSEIR | 57 | 99.938 | | | Mascot |
| 1391.745 | 1391.7091 | -0.0359 | -26 | 447 | 458 | AIMVEVDLEKTK | | | Oxidation (M)[3] | | Mascot |
| 1718.8556 | 1718.8324 | -0.0232 | -13 | 522 | 537 | LQALSSELADARDDSK | | | | | Mascot |

9 Ezrl protein [Danio rerio] gi|32450442 39229.6 9.18 2 62 97.439 57 99.938

Peptide Information

| Calc. Mass | Obsrv. Mass | ± da | ± ppm | Start Seq. | End Seq. | Sequence | Ion Score | C. I. % | Modification | Rank | Result Type |
|---|---|---|---|---|---|---|---|---|---|---|---|
| 864.4977 | 864.4717 | -0.026 | -30 | 54 | 60 | GFLTWLK | | | | | Mascot |
| 1104.5836 | 1104.5546 | -0.029 | -26 | 238 | 246 | IGFPWSEIR | | | | | Mascot |
| 1104.5836 | 1104.5546 | -0.029 | -26 | 238 | 246 | IGFPWSEIR | 57 | 99.938 | | | Mascot |

10 Rdx protein [Danio rerio] gi|29436484 52007.1 8.41 2 56 90.037 57 99.938

Peptide Information

| Calc. Mass | Obsrv. Mass | ± da | ± ppm | Start Seq. | End Seq. | Sequence | Ion Score | C. I. % | Modification | Rank | Result Type |
|---|---|---|---|---|---|---|---|---|---|---|---|
| 1104.5836 | 1104.5546 | -0.029 | -26 | 238 | 246 | IGFPWSEIR | | | | | Mascot |
| 1104.5836 | 1104.5546 | -0.029 | -26 | 238 | 246 | IGFPWSEIR | 57 | 99.938 | | | Mascot |
| 1277.6154 | 1277.6538 | 0.0384 | 30 | 318 | 327 | MERALLEDER | | | Oxidation (M)[1] | | Mascot |

11 LOC553434 protein [Danio rerio] gi|94574164 37971.8 9.24 1 56 90.037 57 99.938

Peptide Information

| Calc. Mass | Obsrv. Mass | ± da | ± ppm | Start Seq. | End Seq. | Sequence | Ion Score | C. I. % | Modification | Rank | Result Type |
|---|---|---|---|---|---|---|---|---|---|---|---|
| 1104.5836 | 1104.5546 | -0.029 | -26 | 227 | 235 | IGFPWSEIR | | | | | Mascot |
| 1104.5836 | 1104.5546 | -0.029 | -26 | 227 | 235 | IGFPWSEIR | 57 | 99.938 | | | Mascot |

| | | | | | | | |
|---|---|---|---|---|---|---|---|
| 12 | PREDICTED: serine/threonine-protein phosphatase 6 regulatory ankyrin repeat subunit C-like [Danio r | gi\|326671533 | 11978.2 | 6.02 | 6 | 48 | 20.865 |

Peptide Information

| Calc. Mass | Obsrv. Mass | ± da | ± ppm | Start Seq. | End Seq. | Sequence | Ion Score | C. I. % | Modification | Rank | Result Type |
|---|---|---|---|---|---|---|---|---|---|---|---|
| 1190.7004 | 1190.6184 | -0.082 | -69 | 22 | 32 | ALQTPLHIAAR | | | | | Mascot |
| 1493.7594 | 1493.7106 | -0.0488 | -33 | 101 | 114 | NGVSLHLLTEEPQG | | | | | Mascot |
| 2207.0762 | 2207.0408 | -0.0354 | -16 | 46 | 67 | GATVLAVDEDGHTPALA CAPNK | | | Carbamidomethyl (C)[18] | | Mascot |
| 2211.0632 | 2211.0518 | -0.0114 | -5 | 2 | 21 | CALLMLAETDDTSLISST NR | | | Carbamidomethyl (C)[1] | | Mascot |
| 2306.3049 | 2306.1018 | -0.2031 | -88 | 68 | 88 | EVAKCLALIISTMKPFIGL MK | | | | | Mascot |
| 2584.5298 | 2584.1545 | -0.3753 | -145 | 22 | 45 | ALQTPLHIAARNGLVTVV EVLLTR | | | | | Mascot |

| | | | | | | | |
|---|---|---|---|---|---|---|---|
| 13 | PREDICTED: slit and trk like 4 protein [Danio rerio] | gi\|125834768 | 92595.8 | 7.75 | 14 | 46 | 0 |

Peptide Information

| Calc. Mass | Obsrv. Mass | ± da | ± ppm | Start Seq. | End Seq. | Sequence | Ion Score | C. I. % | Modification | Rank | Result Type |
|---|---|---|---|---|---|---|---|---|---|---|---|
| 864.4434 | 864.4717 | 0.0283 | 33 | 667 | 673 | HNHKSNK | | | | | Mascot |
| 1045.52 | 1045.5411 | 0.0211 | 20 | 802 | 809 | KSDYYELK | | | | | Mascot |
| 1104.616 | 1104.5546 | -0.0614 | -56 | 415 | 424 | GVFANLTNLR | | | | | Mascot |
| 1104.616 | 1104.5546 | -0.0614 | -56 | 415 | 424 | GVFANLTNLR | | | | | Mascot |
| 1165.5959 | 1165.7177 | 0.1218 | 104 | 323 | 332 | NGQIVSYQTR | | | | | Mascot |
| 1165.5959 | 1165.7177 | 0.1218 | 104 | 323 | 332 | NGQIVSYQTR | | | | | Mascot |
| 1277.6228 | 1277.6538 | 0.031 | 24 | 569 | 578 | ELKNEIMCPK | | | Carbamidomethyl (C)[8], Oxidation (M)[7] | | Mascot |
| 1413.7008 | 1413.7682 | 0.0674 | 48 | 762 | 774 | DFSSIGVSGFEIR | | | | | Mascot |
| 1443.6793 | 1443.6772 | -0.0021 | -1 | 333 | 345 | IPPLSCPQPCTCK | | | Carbamidomethyl (C)[6] | | Mascot |
| 1463.7311 | 1463.7169 | -0.0142 | -10 | 556 | 568 | CASPVQFSNIELR | | | | | Mascot |
| 1531.6555 | 1531.7145 | 0.059 | 39 | 749 | 760 | DTMFLHNFMESK | | | Oxidation (M)[3,9] | | Mascot |
| 1616.9006 | 1616.7039 | -0.1967 | -122 | 95 | 110 | LQNIEGGAFVGLSALK | | | | | Mascot |
| 1632.7434 | 1632.6975 | -0.0459 | -28 | 346 | 360 | AHPSDFGISVSCQER | | | | | Mascot |
| 1670.7148 | 1670.817 | 0.1022 | 61 | 259 | 273 | QELCPMGTGSDFDVR | | | Oxidation (M)[6] | | Mascot |
| 1718.98 | 1718.8324 | -0.1476 | -86 | 361 | 376 | NIQSLADLIPKPPNAK | | | | | Mascot |

| Calc. Mass | Obsrv. Mass | ± da | ± ppm | Start Seq. | End Seq. | Sequence | Ion Score | C. I. % | Modification | Rank | Result Type |
|---|---|---|---|---|---|---|---|---|---|---|---|
| 2584.0491 | 2584.1545 | 0.1054 | 41 | 24 | 45 | AETCSACSCMSIENVLYVNCEK | | | Carbamidomethyl (C)[4,7,9], Oxidation (M)[10] | | Mascot |

14 PREDICTED: beta-1,3-galactosyl-O-glycosyl-glycoprotein beta-1,6-N-acetylglucosaminyltransferase-lik gi|292628442 49526.7 7.14 11 44 0

Peptide Information

| Calc. Mass | Obsrv. Mass | ± da | ± ppm | Start Seq. | End Seq. | Sequence | Ion Score | C. I. % | Modification | Rank | Result Type |
|---|---|---|---|---|---|---|---|---|---|---|---|
| 1118.6165 | 1118.604 | -0.0125 | -11 | 221 | 230 | TNAETVRSLK | | | | | Mascot |
| 1190.631 | 1190.6184 | -0.0126 | -11 | 1 | 10 | MGINNQRTLK | | | Oxidation (M)[1] | | Mascot |
| 1233.5754 | 1233.5854 | 0.01 | 8 | 407 | 415 | CMEAFLRYK | | | Carbamidomethyl (C)[1], Oxidation (M)[2] | | Mascot |
| 1233.5754 | 1233.5854 | 0.01 | 8 | 407 | 415 | CMEAFLRYK | | | Carbamidomethyl (C)[1], Oxidation (M)[2] | | Mascot |
| 1310.674 | 1310.6559 | -0.0181 | -14 | 178 | 188 | LESVIYASWSR | | | | | Mascot |
| 1328.6263 | 1328.5773 | -0.049 | -37 | 323 | 335 | MPSVPGSNSPNNK | | | | | Mascot |
| 1335.6249 | 1335.6929 | 0.068 | 51 | 298 | 307 | EIQNLMEWEK | | | Oxidation (M)[6] | | Mascot |
| 1637.8315 | 1637.7745 | -0.057 | -35 | 336 | 349 | YEQSDMNAIARLVK | | | | | Mascot |
| 2230.1108 | 2230.1079 | -0.0029 | -1 | 189 | 207 | VQADVNCMQDLLKSPVQWR | | | | | Mascot |
| 2233.1621 | 2233.03 | -0.1321 | -59 | 135 | 153 | LLRAIYAPQNVYCVHVDMK | | | | | Mascot |
| 2299.1538 | 2299.1296 | -0.0242 | -11 | 267 | 287 | KSPPPINTSMFSGNAYFVVSR | | | | | Mascot |
| 2306.1785 | 2306.1018 | -0.0767 | -33 | 135 | 153 | LLRAIYAPQNVYCVHVDMK | | | Carbamidomethyl (C)[13], Oxidation (M)[18] | | Mascot |
| 2872.3293 | 2872.3237 | -0.0056 | -2 | 347 | 371 | LVKWSYHEGDLNSGAPYPPCTGMHR | | | Carbamidomethyl (C)[20] | | Mascot |

15 PREDICTED: transformation/transcription domain-associated protein [Danio rerio] gi|326672350 433471.2 8.52 32 43 0

Peptide Information

| Calc. Mass | Obsrv. Mass | ± da | ± ppm | Start Seq. | End Seq. | Sequence | Ion Score | C. I. % | Modification | Rank | Result Type |
|---|---|---|---|---|---|---|---|---|---|---|---|
| 882.5155 | 882.5462 | 0.0307 | 35 | 2182 | 2189 | AVHSLLSR | | | | | Mascot |
| 1045.5095 | 1045.5411 | 0.0316 | 30 | 3604 | 3612 | EVQGNMVPR | | | Oxidation (M)[6] | | Mascot |
| 1165.5817 | 1165.7177 | 0.136 | 117 | 606 | 615 | VANCQTVRMK | | | Oxidation (M)[9] | | Mascot |
| 1165.5817 | 1165.7177 | 0.136 | 117 | 606 | 615 | VANCQTVRMK | | | Oxidation (M)[9] | | Mascot |
| 1182.601 | 1182.5671 | -0.0339 | -29 | 3043 | 3053 | CYLQLAGVMGK | | | | | Mascot |
| 1182.601 | 1182.5671 | -0.0339 | -29 | 3043 | 3053 | CYLQLAGVMGK | | | | | Mascot |
| 1190.5721 | 1190.6184 | 0.0463 | 39 | 1206 | 1215 | CATPLKDEEK | | | Carbamidomethyl (C)[1] | | Mascot |
| 1328.6732 | 1328.5773 | -0.0959 | -72 | 2220 | 2230 | VIYEGLTNYEK | | | | | Mascot |
| 1391.7933 | 1391.7091 | -0.0842 | -60 | 3418 | 3428 | QLPKFFLIEEK | | | | | Mascot |
| 1413.647 | 1413.7682 | 0.1212 | 86 | 2304 | 2314 | MRLSVMNMEMR | | | Oxidation (M)[1] | | Mascot |

| | | | | | | | | | | | |
|---|---|---|---|---|---|---|---|---|---|---|---|
| 1443.8352 | 1443.6772 | -0.158 | -109 | 422 | 433 | LLLNLVDCIRSK | | | Carbamidomethyl (C)[8] | | Mascot |
| 1475.8217 | 1475.7218 | -0.0999 | -68 | 1963 | 1975 | LGFTPSVTIEQRK | | | | | Mascot |
| 1493.7126 | 1493.7106 | -0.002 | -1 | 1311 | 1322 | LFTMDLNVMEHK | | | Oxidation (M)[4] | | Mascot |
| 1531.7937 | 1531.7145 | -0.0792 | -52 | 3099 | 3112 | AFSAAVQMHDVLVK | | | Oxidation (M)[8] | | Mascot |
| 1579.8301 | 1579.7042 | -0.1259 | -80 | 3722 | 3735 | CFAQPNFKVDGILK | | | | | Mascot |
| 1626.8632 | 1626.7155 | -0.1477 | -91 | 1248 | 1262 | QAMHSLQVLAQVTGK | | | Oxidation (M)[3] | | Mascot |
| 1632.8752 | 1632.6975 | -0.1777 | -109 | 1831 | 1843 | RLMTFAWPCLLPK | | | Carbamidomethyl (C)[9] | | Mascot |
| 1637.8896 | 1637.7745 | -0.1151 | -70 | 1678 | 1690 | NYSEIELLFQLLR | | | | | Mascot |
| 1638.8958 | 1638.8115 | -0.0843 | -51 | 443 | 455 | DILMRMLEVFVLK | | | Oxidation (M)[4,6] | | Mascot |
| 1638.8958 | 1638.8115 | -0.0843 | -51 | 443 | 455 | DILMRMLEVFVLK | | | Oxidation (M)[4,6] | | Mascot |
| 1648.8337 | 1648.6963 | -0.1374 | -83 | 1691 | 1704 | AFTGRFLCNMTFLK | | | | | Mascot |
| 1651.8076 | 1651.7683 | -0.0393 | -24 | 2167 | 2181 | GIAACMTCGNTKVLR | | | Carbamidomethyl (C)[5,8] | | Mascot |
| 1718.8054 | 1718.8324 | 0.027 | 16 | 3311 | 3326 | CYSVAFEKSGAVSDAK | | | Carbamidomethyl (C)[1] | | Mascot |
| 1797.9282 | 1797.9182 | -0.01 | -6 | 971 | 985 | HSLYQLLAHPNFTEK | | | | | Mascot |
| 1853.8922 | 1853.8535 | -0.0387 | -21 | 2348 | 2364 | NSGNPMATNQVPNPREK | | | | | Mascot |
| 2192.2107 | 2192.1182 | -0.0925 | -42 | 2579 | 2599 | TGALLNALVQLCHISTPLA EK | | | | | Mascot |
| 2192.2107 | 2192.1182 | -0.0925 | -42 | 2579 | 2599 | TGALLNALVQLCHISTPLA EK | | | | | Mascot |
| 2207.145 | 2207.0408 | -0.1042 | -47 | 1 | 20 | MSFVPTPSPTVVDQTTL MKK | | | | | Mascot |
| 2211.0745 | 2211.0518 | -0.0227 | -10 | 1075 | 1094 | GMDPLVLIDAIAICMAYEE K | | | Oxidation (M)[2] | | Mascot |
| 2221.103 | 2221.103 | 0 | 0 | 3074 | 3092 | EMTAEFYALKGMFLAQIN K | | | Oxidation (M)[2] | | Mascot |
| 2230.0769 | 2230.1079 | 0.031 | 14 | 1323 | 1341 | VFYTELLNLCEAEDAALM K | | | Carbamidomethyl (C)[10] | | Mascot |
| 2283.0981 | 2283.1248 | 0.0267 | 12 | 3617 | 3634 | EWALHTFPNATDYWTFR K | | | | | Mascot |
| 2283.0981 | 2283.1248 | 0.0267 | 12 | 3617 | 3634 | EWALHTFPNATDYWTFR K | | | | | Mascot |
| 2284.1384 | 2284.1189 | -0.0195 | -9 | 1175 | 1196 | ALLFVMMDLTGEVSNGA VAMAK | | | Oxidation (M)[6] | | Mascot |
| 2297.0537 | 2297.0979 | 0.0442 | 19 | 1696 | 1713 | FLCNMTFLKEYMEEEIPK | | | Oxidation (M)[5,12] | | Mascot |
| 2584.2395 | 2584.1545 | -0.085 | -33 | 1288 | 1310 | HQPANAQIGLMEGNTFC TTLQPR | | | Carbamidomethyl (C)[17] | | Mascot |
| 2872.5024 | 2872.3237 | -0.1787 | -62 | 581 | 605 | LVKYAMQALDIYQVQIAG NGQTYIR | | | Oxidation (M)[6] | | Mascot |

16 PREDICTED: hypothetical protein LOC100000098 [Danio rerio] gi|125854762 35807.9 9.77 8 40 0

**Peptide Information**

| Calc. Mass | Obsrv. Mass | ± da | ± ppm | Start Seq. | End Seq. | Sequence | Ion Score | C. I. % | Modification | Rank | Result Type |
|---|---|---|---|---|---|---|---|---|---|---|---|
| 1120.5132 | 1120.5374 | 0.0242 | 22 | 278 | 285 | LNECFYFK | | | Carbamidomethyl (C)[4] | | Mascot |
| 1233.6547 | 1233.5854 | -0.0693 | -56 | 239 | 249 | QTTINSANRTK | | | | | Mascot |

| | | | | | | | | | | | |
|---|---|---|---|---|---|---|---|---|---|---|---|
| 1233.6547 | 1233.5854 | -0.0693 | -56 | 239 | 249 | QTTINSANRTK | | | | | Mascot |
| 1457.8362 | 1457.7173 | -0.1189 | -82 | 6 | 18 | VIIPNIENATVFK | | | | | Mascot |
| 1670.8167 | 1670.817 | 0.0003 | 0 | 222 | 235 | CEEEPIDLSLPNRR | | | | | Mascot |
| 2225.0244 | 2225.0647 | 0.0403 | 18 | 114 | 132 | VIFNLPPEQDQTSDDEYSK | | | | | Mascot |
| 2225.0244 | 2225.0647 | 0.0403 | 18 | 114 | 132 | VIFNLPPEQDQTSDDEYSK | | | | | Mascot |
| 2230.1094 | 2230.1079 | -0.0015 | -1 | 288 | 307 | GEDSPKPLCPLMKLTESAEK | | | Carbamidomethyl (C)[9] | | Mascot |
| 2501.2605 | 2501.1995 | -0.061 | -24 | 39 | 59 | HVANFLTHYSLHEMLSTQTKK | | | Oxidation (M)[14] | | Mascot |
| 2872.4324 | 2872.3237 | -0.1087 | -38 | 160 | 186 | SFLDPPVEISSSSSGNEAISTRPPEIR | | | | | Mascot |

| 17 | Zgc:73317 protein [Danio rerio] | gi\|60649764 | 50586.6 | 6.03 | 10 | 38 | 0 |
|---|---|---|---|---|---|---|---|

**Peptide Information**

| Calc. Mass | Obsrv. Mass | ± da | ± ppm | Start Seq. | End Seq. | Sequence | Ion Score | C. I. % | Modification | Rank | Result Type |
|---|---|---|---|---|---|---|---|---|---|---|---|
| 842.5206 | 842.4905 | -0.0301 | -36 | 8 | 14 | LERAVVR | | | | | Mascot |
| 850.4166 | 850.5237 | 0.1071 | 126 | 291 | 298 | THHGSPSK | | | | | Mascot |
| 850.4166 | 850.5237 | 0.1071 | 126 | 291 | 298 | THHGSPSK | | | | | Mascot |
| 1042.6003 | 1042.557 | -0.0433 | -42 | 2 | 10 | EALVGRLER | | | | | Mascot |
| 1104.5718 | 1104.5546 | -0.0172 | -16 | 360 | 369 | VNSIIVDNCK | | | | | Mascot |
| 1104.5718 | 1104.5546 | -0.0172 | -16 | 360 | 369 | VNSIIVDNCK | | | | | Mascot |
| 1118.6204 | 1118.604 | -0.0164 | -15 | 311 | 320 | HPPVLELEGK | | | | | Mascot |
| 1120.5674 | 1120.5374 | -0.03 | -27 | 443 | 451 | EFPVPEQFK | | | | | Mascot |
| 1179.5562 | 1179.5767 | 0.0205 | 17 | 416 | 426 | DSLDCEIVSAK | | | | | Mascot |
| 1310.631 | 1310.6559 | 0.0249 | 19 | 201 | 211 | EHHCTGLIWSK | | | | | Mascot |
| 1853.8221 | 1853.8535 | 0.0314 | 17 | 427 | 442 | SSEMNILVPEGEDDYR | | | | | Mascot |
| 2297.0789 | 2297.0979 | 0.019 | 8 | 406 | 426 | TEGCHVYLSKDSLDCEIVSAK | | | | | Mascot |

| 18 | titin a [Danio rerio] | gi\|110005909 | 3646149.2 | 6.18 | 70 | 38 | 0 |
|---|---|---|---|---|---|---|---|

**Peptide Information**

| Calc. Mass | Obsrv. Mass | ± da | ± ppm | Start Seq. | End Seq. | Sequence | Ion Score | C. I. % | Modification | Rank | Result Type |
|---|---|---|---|---|---|---|---|---|---|---|---|
| 842.4981 | 842.4905 | -0.0076 | -9 | 8812 | 8818 | EDILPKK | | | | | Mascot |
| 850.4814 | 850.5237 | 0.0423 | 50 | 1481 | 1487 | LTSVRMK | | | Oxidation (M)[6] | | Mascot |
| 850.5396 | 850.5237 | -0.0159 | -19 | 17926 | 17932 | FLLTKTK | | | | | Mascot |
| 864.4686 | 864.4717 | 0.0031 | 4 | 10287 | 10293 | VYAEARR | | | | | Mascot |
| 870.493 | 870.5187 | 0.0257 | 30 | 8546 | 8553 | TDIPLPSK | | | | | Mascot |
| 882.4679 | 882.5462 | 0.0783 | 89 | 2876 | 2882 | YKVSTER | | | | | Mascot |

| | | | | | | | | |
|---|---|---|---|---|---|---|---|---|
| 996.536 | 996.572 | 0.036 | 36 | 2979 | 2986 | LTITETYR | | Mascot |
| 996.591 | 996.572 | -0.019 | -19 | 9064 | 9072 | MKAVPLPPK | Oxidation (M)[1] | Mascot |
| 1042.5204 | 1042.557 | 0.0366 | 35 | 27162 | 27169 | ELSEVYFR | | Mascot |
| 1045.5347 | 1045.5411 | 0.0064 | 6 | 7144 | 7153 | CQIGGTPEIK | | Mascot |
| 1051.6259 | 1051.6859 | 0.06 | 57 | 32375 | 32384 | SPIASPPRVK | | Mascot |
| 1062.6306 | 1062.6647 | 0.0341 | 32 | 28602 | 28610 | IVGYVVERK | | Mascot |
| 1104.5419 | 1104.5546 | 0.0127 | 11 | 10607 | 10615 | EAEEKEEIK | | Mascot |
| 1104.5419 | 1104.5546 | 0.0127 | 11 | 10607 | 10615 | EAEEKEEIK | | Mascot |
| 1118.6205 | 1118.604 | -0.0165 | -15 | 24180 | 24188 | EKNSILWTK | | Mascot |
| 1120.5481 | 1120.5374 | -0.0107 | -10 | 11575 | 11584 | GSQEIKTDDK | | Mascot |
| 1165.6211 | 1165.7177 | 0.0966 | 83 | 28447 | 28457 | VSAENKFGVSK | | Mascot |
| 1165.6211 | 1165.7177 | 0.0966 | 83 | 28447 | 28457 | VSAENKFGVSK | | Mascot |
| 1179.6078 | 1179.5767 | -0.0311 | -26 | 28311 | 28319 | VTWKLEEMK | Oxidation (M)[8] | Mascot |
| 1182.6187 | 1182.5671 | -0.0516 | -44 | 28547 | 28557 | VYAMNIAGLSK | Oxidation (M)[4] | Mascot |
| 1182.6729 | 1182.5671 | -0.1058 | -89 | 725 | 736 | VPETAAIPSAVK | | Mascot |
| 1190.6263 | 1190.6184 | -0.0079 | -7 | 29946 | 29956 | SLKSDESVTPK | | Mascot |
| 1221.5304 | 1221.5538 | 0.0234 | 19 | 10260 | 10269 | DKDEDPEMVK | Oxidation (M)[8] | Mascot |
| 1233.662 | 1233.5854 | -0.0766 | -62 | 32566 | 32576 | IQGGSEMVIKR | Oxidation (M)[7] | Mascot |
| 1233.6626 | 1233.5854 | -0.0772 | -63 | 5108 | 5118 | GSAPITVQWFK | | Mascot |
| 1277.7688 | 1277.6538 | -0.115 | -90 | 16200 | 16211 | EKIVVHAGGVIR | | Mascot |
| 1279.7256 | 1279.7423 | 0.0167 | 13 | 26573 | 26583 | LNTGTYELKIK | | Mascot |
| 1310.6587 | 1310.6559 | -0.0028 | -2 | 17534 | 17544 | VKGLTEDTEYR | | Mascot |
| 1328.6627 | 1328.5773 | -0.0854 | -64 | 8734 | 8744 | QDISQTKPHMK | Oxidation (M)[10] | Mascot |
| 1335.6208 | 1335.6929 | 0.0721 | 54 | 18202 | 18212 | DKVEMEGEQVR | Oxidation (M)[5] | Mascot |
| 1391.6914 | 1391.7091 | 0.0177 | 13 | 2956 | 2967 | NGVEIQFSVDQR | | Mascot |
| 1413.7737 | 1413.7682 | -0.0055 | -4 | 31447 | 31459 | VSGTPAPTLKWEK | | Mascot |
| 1443.7446 | 1443.6772 | -0.0674 | -47 | 16899 | 16912 | AMAGTHINIMAGIK | Oxidation (M)[2] | Mascot |
| 1457.7594 | 1457.7173 | -0.0421 | -29 | 1571 | 1583 | SLVEDSQLPEGRK | | Mascot |
| 1463.7264 | 1463.7169 | -0.0095 | -6 | 27984 | 27996 | LTSPEIDIDADFK | | Mascot |
| 1469.6866 | 1469.7172 | 0.0306 | 21 | 5242 | 5254 | GDSSEYKVEVSNR | | Mascot |
| 1475.7601 | 1475.7218 | -0.0383 | -26 | 30138 | 30150 | VFAENETGLSRPR | | Mascot |
| 1485.7155 | 1485.712 | -0.0035 | -2 | 19518 | 19530 | ITNLEAGQAYCFR | | Mascot |
| 1493.7166 | 1493.7106 | -0.006 | -4 | 28125 | 28136 | LSWQEVSERCTR | | Mascot |
| 1531.8115 | 1531.7145 | -0.097 | -63 | 24478 | 24490 | ETSRLSWTVVEPK | | Mascot |
| 1579.7897 | 1579.7042 | -0.0855 | -54 | 11668 | 11681 | LHPSKVVHMSEDGK | Oxidation (M)[9] | Mascot |
| 1616.806 | 1616.7039 | -0.1021 | -63 | 5165 | 5178 | QKCEATVTVQEPAR | Carbamidomethyl (C)[3] | Mascot |

| | | | | | | | | | |
|---|---|---|---|---|---|---|---|---|---|
| 1626.7209 | 1626.7155 | -0.0054 | -3 | 4784 | 4798 | ASNSVGTATCCTELR | | Carbamidomethyl (C)[10,11] | Mascot |
| 1632.7687 | 1632.6975 | -0.0712 | -44 | 17019 | 17033 | DAASPQWVPICSSSK | | Carbamidomethyl (C)[11] | Mascot |
| 1637.7628 | 1637.7745 | 0.0117 | 7 | 28440 | 28452 | YMEYTFRVSAENK | | | Mascot |
| 1638.9061 | 1638.8115 | -0.0946 | -58 | 7730 | 7743 | DIDIVELLRNVDPK | | | Mascot |
| 1638.9061 | 1638.8115 | -0.0946 | -58 | 7730 | 7743 | DIDIVELLRNVDPK | | | Mascot |
| 1648.6432 | 1648.6963 | 0.0531 | 32 | 12151 | 12164 | SVFDDEAEYECDAR | | | Mascot |
| 1651.8174 | 1651.7683 | -0.0491 | -30 | 25798 | 25813 | IGPSEPSPETDPQVAK | | | Mascot |
| 1670.7955 | 1670.817 | 0.0215 | 13 | 19173 | 19187 | DHMTVTWKAPANDGK | | | Mascot |
| 1682.7472 | 1682.7767 | 0.0295 | 18 | 6002 | 6016 | NEAGSESCSMDLRVK | | Carbamidomethyl (C)[8] | Mascot |
| 1718.8894 | 1718.8324 | -0.057 | -33 | 11872 | 11885 | EEGTKHMLILYNVR | | Oxidation (M)[7] | Mascot |
| 1755.8582 | 1755.9348 | 0.0766 | 44 | 17130 | 17145 | TVSIPECHVTGLEEGK | | Carbamidomethyl (C)[7] | Mascot |
| 1797.9381 | 1797.9182 | -0.0199 | -11 | 22684 | 22700 | NSVTLTWEPPLLDGGAK | | | Mascot |
| 1853.8309 | 1853.8535 | 0.0226 | 12 | 18756 | 18771 | HPFDPPSEPLNCCVNK | | Carbamidomethyl (C)[12] | Mascot |
| 1959.952 | 1960.0217 | 0.0697 | 36 | 17483 | 17498 | DYSCTYADLVWIKPTK | | Carbamidomethyl (C)[4] | Mascot |
| 2192.1155 | 2192.1182 | 0.0027 | 1 | 21661 | 21680 | ILAENEYGVGLPVETMEAIK | | Oxidation (M)[16] | Mascot |
| 2192.1155 | 2192.1182 | 0.0027 | 1 | 21661 | 21680 | ILAENEYGVGLPVETMEAIK | | Oxidation (M)[16] | Mascot |
| 2207.0801 | 2207.0408 | -0.0393 | -18 | 26462 | 26480 | FTVSGLTQNESYEFRVMAK | | | Mascot |
| 2211.0713 | 2211.0518 | -0.0195 | -9 | 29957 | 29976 | TPLCPPEPPSFPPEIMDVTK | | Oxidation (M)[16] | Mascot |
| 2221.1248 | 2221.103 | -0.0218 | -10 | 29857 | 29876 | VIGRPDPPEGPLEFDDIQAR | | | Mascot |
| 2225.0801 | 2225.0647 | -0.0154 | -7 | 488 | 506 | IRKPMHVEGAQMEEEADVR | | | Mascot |
| 2225.0801 | 2225.0647 | -0.0154 | -7 | 488 | 506 | IRKPMHVEGAQMEEEADVR | | | Mascot |
| 2230.1106 | 2230.1079 | -0.0027 | -1 | 29738 | 29759 | FRVMACNAGGAGEPAEIPGVVK | | Carbamidomethyl (C)[6] | Mascot |
| 2232.9575 | 2233.03 | 0.0725 | 32 | 1525 | 1542 | MINEFGYCSLDYEVAYSR | | Carbamidomethyl (C)[8], Oxidation (M)[1] | Mascot |
| 2283.104 | 2283.1248 | 0.0208 | 9 | 2802 | 2823 | NVHFGDVGDYKFVAGSAETAAK | | | Mascot |
| 2283.1284 | 2283.1248 | -0.0036 | -2 | 13772 | 13793 | VRAVNVAGESEPSEPSDPVLCK | | | Mascot |
| 2284.1389 | 2284.1189 | -0.02 | -9 | 21385 | 21405 | ACDTIFQPGPPGNPRVLDTTK | | Carbamidomethyl (C)[2] | Mascot |
| 2297.1516 | 2297.0979 | -0.0537 | -23 | 7038 | 7058 | NLSVEMGKSLILECTYAGSPK | | Carbamidomethyl (C)[14] | Mascot |
| 2299.1233 | 2299.1296 | 0.0063 | 3 | 30037 | 30058 | VVAQNDIGQSEPGPVSESVVCK | | Carbamidomethyl (C)[21] | Mascot |
| 2306.0732 | 2306.1018 | 0.0286 | 12 | 17825 | 17842 | ETFFMVDKCVENHIYQFR | | | Mascot |
| 2501.1753 | 2501.1995 | 0.0242 | 10 | 856 | 879 | DETMSEISSVSVDTGAPAAPFFVK | | Oxidation (M)[4] | Mascot |
| 2584.2922 | 2584.1545 | -0.1377 | -53 | 26393 | 26416 | SSDPVLAIDACEPPNSVRVTDITK | | Carbamidomethyl (C)[11] | Mascot |
| 2872.4258 | 2872.3237 | -0.1021 | -36 | 28183 | 28208 | RLDVIDTTSTTASLVWMKPEHDGGSR | | | Mascot |
| 3337.4834 | 3337.6814 | 0.198 | 59 | 4869 | 4900 | DTGTYTCTAANEAGSSSCSSSVTVQEPPVFVK | | Carbamidomethyl (C)[7,18] | Mascot |

| 19 | PREDICTED: titin [Danio rerio] | gi\|189523699 | 3645668 | 6.16 | 70 | 38 | 0 |
|---|---|---|---|---|---|---|---|

Peptide Information

| Calc. Mass | Obsrv. Mass | ± da | ± ppm | Start Seq. | End Seq. | Sequence | Ion Score | C. I. % | Modification | Rank | Result Type |
|---|---|---|---|---|---|---|---|---|---|---|---|
| 842.4981 | 842.4905 | -0.0076 | -9 | 8812 | 8818 | EDILPKK | | | | | Mascot |
| 850.4814 | 850.5237 | 0.0423 | 50 | 1481 | 1487 | LTSVRMK | | | Oxidation (M)[6] | | Mascot |
| 850.5396 | 850.5237 | -0.0159 | -19 | 17926 | 17932 | FLLTKTK | | | | | Mascot |
| 864.4686 | 864.4717 | 0.0031 | 4 | 10287 | 10293 | VYAEARR | | | | | Mascot |
| 870.4203 | 870.5187 | 0.0984 | 113 | 6908 | 6915 | LSDSGTYK | | | | | Mascot |
| 882.4679 | 882.5462 | 0.0783 | 89 | 2876 | 2882 | YKVSTER | | | | | Mascot |
| 996.536 | 996.572 | 0.036 | 36 | 2979 | 2986 | LTITETYR | | | | | Mascot |
| 996.591 | 996.572 | -0.019 | -19 | 9064 | 9072 | MKAVPLPPK | | | Oxidation (M)[1] | | Mascot |
| 1042.5204 | 1042.557 | 0.0366 | 35 | 27162 | 27169 | ELSEVYFR | | | | | Mascot |
| 1045.5347 | 1045.5411 | 0.0064 | 6 | 7144 | 7153 | CQIGGTPEIK | | | | | Mascot |
| 1051.6259 | 1051.6859 | 0.06 | 57 | 32375 | 32384 | SPIASPPRVK | | | | | Mascot |
| 1062.6306 | 1062.6647 | 0.0341 | 32 | 28602 | 28610 | IVGYVVERK | | | | | Mascot |
| 1104.5419 | 1104.5546 | 0.0127 | 11 | 10607 | 10615 | EAEEKEEIK | | | | | Mascot |
| 1104.5419 | 1104.5546 | 0.0127 | 11 | 10607 | 10615 | EAEEKEEIK | | | | | Mascot |
| 1118.6205 | 1118.604 | -0.0165 | -15 | 24180 | 24188 | EKNSILWTK | | | | | Mascot |
| 1120.5481 | 1120.5374 | -0.0107 | -10 | 11575 | 11584 | GSQEIKTDDK | | | | | Mascot |
| 1165.6212 | 1165.7177 | 0.0965 | 83 | 8759 | 8768 | ETFSVQVVTR | | | | | Mascot |
| 1165.6212 | 1165.7177 | 0.0965 | 83 | 8759 | 8768 | ETFSVQVVTR | | | | | Mascot |
| 1179.6078 | 1179.5767 | -0.0311 | -26 | 28311 | 28319 | VTWKLEEMK | | | Oxidation (M)[8] | | Mascot |
| 1182.6187 | 1182.5671 | -0.0516 | -44 | 28547 | 28557 | VYAMNIAGLSK | | | Oxidation (M)[4] | | Mascot |
| 1182.6729 | 1182.5671 | -0.1058 | -89 | 725 | 736 | VPETAAIPSAVK | | | | | Mascot |
| 1190.6263 | 1190.6184 | -0.0079 | -7 | 29946 | 29956 | SLKSDESVTPK | | | | | Mascot |
| 1221.5304 | 1221.5538 | 0.0234 | 19 | 10260 | 10269 | DKDEDPEMVK | | | Oxidation (M)[8] | | Mascot |
| 1233.662 | 1233.5854 | -0.0766 | -62 | 32566 | 32576 | IQGGSEMVIKR | | | Oxidation (M)[7] | | Mascot |
| 1233.6626 | 1233.5854 | -0.0772 | -63 | 5108 | 5118 | GSAPITVQWFK | | | | | Mascot |
| 1277.7688 | 1277.6538 | -0.115 | -90 | 16200 | 16211 | EKIVVHAGGVIR | | | | | Mascot |
| 1279.7256 | 1279.7423 | 0.0167 | 13 | 26573 | 26583 | LNTGTYELKIK | | | | | Mascot |
| 1310.6587 | 1310.6559 | -0.0028 | -2 | 17534 | 17544 | VKGLTEDTEYR | | | | | Mascot |
| 1328.6627 | 1328.5773 | -0.0854 | -64 | 8734 | 8744 | QDISQTKPHMK | | | Oxidation (M)[10] | | Mascot |
| 1335.6208 | 1335.6929 | 0.0721 | 54 | 18202 | 18212 | DKVEMEGEQVR | | | Oxidation (M)[5] | | Mascot |
| 1391.6914 | 1391.7091 | 0.0177 | 13 | 2956 | 2967 | NGVEIQFSVDQR | | | | | Mascot |
| 1413.7737 | 1413.7682 | -0.0055 | -4 | 31447 | 31459 | VSGTPAPTLKWEK | | | | | Mascot |

| | | | | | | | | |
|---|---|---|---|---|---|---|---|---|
| 1443.7446 | 1443.6772 | -0.0674 | -47 | 16899 | 16912 | AMAGTHINIMAGIK | Oxidation (M)[2] | Mascot |
| 1457.7594 | 1457.7173 | -0.0421 | -29 | 1571 | 1583 | SLVEDSQLPEGRK | | Mascot |
| 1463.7264 | 1463.7169 | -0.0095 | -6 | 27984 | 27996 | LTSPEIDIDADFK | | Mascot |
| 1469.6866 | 1469.7172 | 0.0306 | 21 | 5242 | 5254 | GDSSEYKVEVSNR | | Mascot |
| 1475.7601 | 1475.7218 | -0.0383 | -26 | 30138 | 30150 | VFAENETGLSRPR | | Mascot |
| 1485.7155 | 1485.712 | -0.0035 | -2 | 19518 | 19530 | ITNLEAGQAYCFR | | Mascot |
| 1493.7166 | 1493.7106 | -0.006 | -4 | 28125 | 28136 | LSWQEVSERCTR | | Mascot |
| 1531.8115 | 1531.7145 | -0.097 | -63 | 24478 | 24490 | ETSRLSWTVVEPK | | Mascot |
| 1579.7897 | 1579.7042 | -0.0855 | -54 | 11668 | 11681 | LHPSKVVHMSEDGK | Oxidation (M)[9] | Mascot |
| 1616.806 | 1616.7039 | -0.1021 | -63 | 5165 | 5178 | QKCEATVTVQEPAR | Carbamidomethyl (C)[3] | Mascot |
| 1626.7209 | 1626.7155 | -0.0054 | -3 | 4784 | 4798 | ASNSVGTATCCTELR | Carbamidomethyl (C)[10,11] | Mascot |
| 1632.7687 | 1632.6975 | -0.0712 | -44 | 17019 | 17033 | DAASPQWVPICSSSK | Carbamidomethyl (C)[11] | Mascot |
| 1637.7628 | 1637.7745 | 0.0117 | 7 | 28440 | 28452 | YMEYTFRVSAENK | | Mascot |
| 1638.9061 | 1638.8115 | -0.0946 | -58 | 7730 | 7743 | DIDIVELLRNVDPK | | Mascot |
| 1638.9061 | 1638.8115 | -0.0946 | -58 | 7730 | 7743 | DIDIVELLRNVDPK | | Mascot |
| 1648.6432 | 1648.6963 | 0.0531 | 32 | 12151 | 12164 | SVFDDEAEYECDAR | | Mascot |
| 1651.8174 | 1651.7683 | -0.0491 | -30 | 25798 | 25813 | IGPSEPSPETDPQVAK | | Mascot |
| 1670.7955 | 1670.817 | 0.0215 | 13 | 19173 | 19187 | DHMTVTWKAPANDGK | | Mascot |
| 1682.7472 | 1682.7767 | 0.0295 | 18 | 6002 | 6016 | NEAGSESCSMDLRVK | Carbamidomethyl (C)[8] | Mascot |
| 1718.8894 | 1718.8324 | -0.057 | -33 | 11872 | 11885 | EEGTKHMLILYNVR | Oxidation (M)[7] | Mascot |
| 1755.8582 | 1755.9348 | 0.0766 | 44 | 17130 | 17145 | TVSIPECHVTGLEEGK | Carbamidomethyl (C)[7] | Mascot |
| 1797.9381 | 1797.9182 | -0.0199 | -11 | 22684 | 22700 | NSVTLTWEPPLLDGGAK | | Mascot |
| 1853.8309 | 1853.8535 | 0.0226 | 12 | 18756 | 18771 | HPFDPPSEPLNCCVNK | Carbamidomethyl (C)[12] | Mascot |
| 1959.952 | 1960.0217 | 0.0697 | 36 | 17483 | 17498 | DYSCTYADLVWIKPTK | Carbamidomethyl (C)[4] | Mascot |
| 2192.1155 | 2192.1182 | 0.0027 | 1 | 21661 | 21680 | ILAENEYGVGLPVETMEA IK | Oxidation (M)[16] | Mascot |
| 2192.1155 | 2192.1182 | 0.0027 | 1 | 21661 | 21680 | ILAENEYGVGLPVETMEA IK | Oxidation (M)[16] | Mascot |
| 2207.0801 | 2207.0408 | -0.0393 | -18 | 26462 | 26480 | FTVSGLTQNESYEFRVM AK | | Mascot |
| 2211.0713 | 2211.0518 | -0.0195 | -9 | 29957 | 29976 | TPLCPPEPPSFPPEIMDV TK | Oxidation (M)[16] | Mascot |
| 2221.1248 | 2221.103 | -0.0218 | -10 | 29857 | 29876 | VIGRPDPPEGPLEFDDIQ AR | | Mascot |
| 2225.0479 | 2225.0647 | 0.0168 | 8 | 18756 | 18774 | HPFDPPSEPLNCCVNKV SK | Carbamidomethyl (C)[12,13] | Mascot |
| 2225.0479 | 2225.0647 | 0.0168 | 8 | 18756 | 18774 | HPFDPPSEPLNCCVNKV SK | Carbamidomethyl (C)[12,13] | Mascot |
| 2230.1106 | 2230.1079 | -0.0027 | -1 | 29738 | 29759 | FRVMACNAGGAGEPAEI PGVVK | Carbamidomethyl (C)[6] | Mascot |
| 2232.9575 | 2233.03 | 0.0725 | 32 | 1525 | 1542 | MINEFGYCSLDYEVAYSR | Carbamidomethyl (C)[8], Oxidation (M)[1] | Mascot |
| 2283.104 | 2283.1248 | 0.0208 | 9 | 2802 | 2823 | NVHFGDVGDYKFVAGSA ETAAK | | Mascot |
| 2283.1284 | 2283.1248 | -0.0036 | -2 | 13772 | 13793 | VRAVNVAGESEPSEPSD | | Mascot |

| | | | | | | | | | | | |
|---|---|---|---|---|---|---|---|---|---|---|---|
| | | | | | | PVLCK | | | | | |
| 2284.1389 | 2284.1189 | -0.02 | -9 | 21385 | 21405 | ACDTIFQPGPPGNPRVLD TTK | | | Carbamidomethyl (C)[2] | | Mascot |
| 2297.1013 | 2297.0979 | -0.0034 | -1 | 488 | 506 | IRKPMHVEGPQMEEETD VR | | | Oxidation (M)[5] | | Mascot |
| 2299.1233 | 2299.1296 | 0.0063 | 3 | 30037 | 30058 | VVAQNDIGQSEPGPVSE SVVCK | | | Carbamidomethyl (C)[21] | | Mascot |
| 2306.0732 | 2306.1018 | 0.0286 | 12 | 17825 | 17842 | ETFFMVDKCVENHIYQFR | | | | | Mascot |
| 2501.1753 | 2501.1995 | 0.0242 | 10 | 856 | 879 | DETMSEISSVSVDTGAPA APFFVK | | | Oxidation (M)[4] | | Mascot |
| 2584.2922 | 2584.1545 | -0.1377 | -53 | 26393 | 26416 | SSDPVLAIDACEPPNSVR VTDITK | | | Carbamidomethyl (C)[11] | | Mascot |
| 2872.4258 | 2872.3237 | -0.1021 | -36 | 28183 | 28208 | RLDVIDTTSTTASLVWMK PEHDGGSR | | | | | Mascot |
| 3337.4834 | 3337.6814 | 0.198 | 59 | 4869 | 4900 | DTGTYTCTAANEAGSSS CSSSVTVQEPPVFVK | | | Carbamidomethyl (C)[7,18] | | Mascot |

| | | | | | | | |
|---|---|---|---|---|---|---|---|
| 20 | Glutathione peroxidase 1b [Danio rerio] | gi\|51859004 | 16361.4 | 7.68 | 6 | 37 | 0 |

Peptide Information

| Calc. Mass | Obsrv. Mass | ± da | ± ppm | Start Seq. | End Seq. | Sequence | Ion Score | C. I. % | Modification | Rank | Result Type |
|---|---|---|---|---|---|---|---|---|---|---|---|
| 1120.5972 | 1120.5374 | -0.0598 | -53 | 91 | 99 | FIIWSPVCR | | | | | Mascot |
| 1165.6099 | 1165.7177 | 0.1078 | 92 | 125 | 135 | FLTSGIDGDIK | | | | | Mascot |
| 1165.6099 | 1165.7177 | 0.1078 | 92 | 125 | 135 | FLTSGIDGDIK | | | | | Mascot |
| 1179.6409 | 1179.5767 | -0.0642 | -54 | 109 | 119 | FLIGSDGVPFK | | | | | Mascot |
| 1279.6429 | 1279.7423 | 0.0994 | 78 | 40 | 50 | YVRPGNGYEPK | | | | | Mascot |
| 1335.7419 | 1335.6929 | -0.049 | -37 | 109 | 120 | FLIGSDGVPFKR | | | | | Mascot |
| 2297.1602 | 2297.0979 | -0.0623 | -27 | 100 | 119 | NDIAWNFEKFLIGSDGVP FK | | | | | Mascot |

| | | | | | | | |
|---|---|---|---|---|---|---|---|
| 21 | tektin 2 [Danio rerio] | gi\|37682089 | 48740.9 | 5.63 | 9 | 36 | 0 |

Peptide Information

| Calc. Mass | Obsrv. Mass | ± da | ± ppm | Start Seq. | End Seq. | Sequence | Ion Score | C. I. % | Modification | Rank | Result Type |
|---|---|---|---|---|---|---|---|---|---|---|---|
| 1045.5789 | 1045.5411 | -0.0378 | -36 | 68 | 75 | INDITRWK | | | | | Mascot |
| 1190.5834 | 1190.6184 | 0.035 | 29 | 234 | 243 | EEMQASVQIR | | | | | Mascot |
| 1328.6943 | 1328.5773 | -0.117 | -88 | 127 | 138 | ELVSDPVEAELK | | | | | Mascot |
| 1579.8915 | 1579.7042 | -0.1873 | -119 | 357 | 370 | QKLAQAQHSLQSLK | | | | | Mascot |
| 1616.752 | 1616.7039 | -0.0481 | -30 | 371 | 383 | QHEAQMMEELSRK | | | | | Mascot |
| 1632.7468 | 1632.6975 | -0.0493 | -30 | 371 | 383 | QHEAQMMEELSRK | | | Oxidation (M)[6] | | Mascot |
| 1648.7417 | 1648.6963 | -0.0454 | -28 | 371 | 383 | QHEAQMMEELSRK | | | Oxidation (M)[6,7] | | Mascot |
| 1854.0219 | 1853.8535 | -0.1684 | -91 | 299 | 315 | DIIGLEEDLQAKTAPLK | | | | | Mascot |
| 2211.0261 | 2211.0518 | 0.0257 | 12 | 271 | 287 | RTHHEEQAHHDMQWQL K | | | | | Mascot |
| 2283.1365 | 2283.1248 | -0.0117 | -5 | 2 | 22 | ATLSSKPASRYSVSDWA | | | | | Mascot |

| Calc. Mass | Obsrv. Mass | ± da | ± ppm | Start Seq. | End Seq. | Sequence | Ion Score | C. I. % | Modification | Rank | Result Type |
|---|---|---|---|---|---|---|---|---|---|---|---|
| | | | | | | TNNK | | | | | |
| 2283.1365 | 2283.1248 | -0.0117 | -5 | 2 | 22 | ATLSSKPASRYSVSDWA TNNK | | | | | Mascot |
| 2584.1211 | 2584.1545 | 0.0334 | 13 | 80 | 101 | ACAQEVDEEMDALTLSK EEMER | | | Carbamidomethyl (C)[2] | | Mascot |

| | | | | | | | |
|---|---|---|---|---|---|---|---|
| 22 | PREDICTED: hypothetical protein LOC100007481 [Danio rerio] | gi\|326664865 | 157182.2 | 8.05 | 16 | 35 | 0 |

**Peptide Information**

| Calc. Mass | Obsrv. Mass | ± da | ± ppm | Start Seq. | End Seq. | Sequence | Ion Score | C. I. % | Modification | Rank | Result Type |
|---|---|---|---|---|---|---|---|---|---|---|---|
| 864.4607 | 864.4717 | 0.011 | 13 | 883 | 889 | VSVKEMR | | | Oxidation (M)[6] | | Mascot |
| 882.4719 | 882.5462 | 0.0743 | 84 | 684 | 690 | QFAEFLK | | | | | Mascot |
| 1045.5173 | 1045.5411 | 0.0238 | 23 | 874 | 882 | QGSQSFVHR | | | | | Mascot |
| 1118.551 | 1118.604 | 0.053 | 47 | 206 | 215 | CEDGLQVAVK | | | Carbamidomethyl (C)[1] | | Mascot |
| 1221.6296 | 1221.5538 | -0.0758 | -62 | 336 | 346 | LIDFGCGDLLR | | | | | Mascot |
| 1233.6222 | 1233.5854 | -0.0368 | -30 | 180 | 189 | DDIHSKYSIR | | | | | Mascot |
| 1233.6222 | 1233.5854 | -0.0368 | -30 | 180 | 189 | DDIHSKYSIR | | | | | Mascot |
| 1310.6812 | 1310.6559 | -0.0253 | -19 | 943 | 954 | SSNPPSLPNNKR | | | | | Mascot |
| 1335.6176 | 1335.6929 | 0.0753 | 56 | 347 | 358 | TSSYNTYSGTVR | | | | | Mascot |
| 1413.7557 | 1413.7682 | 0.0125 | 9 | 463 | 474 | RAIQEISNQQAR | | | | | Mascot |
| 1475.7682 | 1475.7218 | -0.0464 | -31 | 931 | 942 | EYGHFQLPLPFK | | | | | Mascot |
| 1616.8563 | 1616.7039 | -0.1524 | -94 | 322 | 335 | MPNLLVNTETLEVK | | | Oxidation (M)[1] | | Mascot |
| 1632.7906 | 1632.6975 | -0.0931 | -57 | 1074 | 1087 | SDIKEVHCCLVMAK | | | Carbamidomethyl (C)[8] | | Mascot |
| 1648.7855 | 1648.6963 | -0.0892 | -54 | 1074 | 1087 | SDIKEVHCCLVMAK | | | Carbamidomethyl (C)[8], Oxidation (M)[12] | | Mascot |
| 1755.7683 | 1755.9348 | 0.1665 | 95 | 359 | 373 | YCPPEFIDTGEYHGK | | | | | Mascot |
| 1853.8527 | 1853.8535 | 0.0008 | 0 | 1033 | 1047 | DFTVPRCYYPSSFSK | | | Carbamidomethyl (C)[7] | | Mascot |
| 2299.1062 | 2299.1296 | 0.0234 | 10 | 665 | 683 | WNRIVVEELDMTGDYPS FK | | | | | Mascot |
| 2501.1216 | 2501.1995 | 0.0779 | 31 | 192 | 215 | MGQGGCGSVYEGTRCE DGLQVAVK | | | Carbamidomethyl (C)[6] | | Mascot |

| | | | | | | | |
|---|---|---|---|---|---|---|---|
| 23 | PREDICTED: hypothetical protein LOC559994 [Danio rerio] | gi\|326677062 | 1030064.3 | 5.62 | 45 | 34 | 0 |

**Peptide Information**

| Calc. Mass | Obsrv. Mass | ± da | ± ppm | Start Seq. | End Seq. | Sequence | Ion Score | C. I. % | Modification | Rank | Result Type |
|---|---|---|---|---|---|---|---|---|---|---|---|
| 850.5032 | 850.5237 | 0.0205 | 24 | 247 | 253 | LKELYGK | | | | | Mascot |
| 850.5185 | 850.5237 | 0.0052 | 6 | 8569 | 8575 | IKPFAFK | | | | | Mascot |
| 864.4321 | 864.4717 | 0.0396 | 46 | 3272 | 3278 | EERAFGR | | | | | Mascot |
| 870.5559 | 870.5187 | -0.0372 | -43 | 1536 | 1542 | ALLLWVR | | | | | Mascot |

| | | | | | | | | |
|---|---|---|---|---|---|---|---|---|
| 882.4679 | 882.5462 | 0.0783 | 89 | 3102 | 3108 | NLHEELK | | Mascot |
| 1045.5636 | 1045.5411 | -0.0225 | -22 | 8277 | 8285 | DLEVRSAQK | | Mascot |
| 1062.536 | 1062.6647 | 0.1287 | 121 | 3153 | 3160 | TKCLEQQR | Carbamidomethyl (C)[3] | Mascot |
| 1118.5687 | 1118.604 | 0.0353 | 32 | 6531 | 6541 | ESGKSVASEPK | | Mascot |
| 1120.5238 | 1120.5374 | 0.0136 | 12 | 6138 | 6146 | QCVMEQVAR | Carbamidomethyl (C)[2] | Mascot |
| 1179.5674 | 1179.5767 | 0.0093 | 8 | 2826 | 2835 | DMDLLSQSVR | Oxidation (M)[2] | Mascot |
| 1182.6841 | 1182.5671 | -0.117 | -99 | 1064 | 1073 | VPERLQEALK | | Mascot |
| 1182.6841 | 1182.5671 | -0.117 | -99 | 1064 | 1073 | VPERLQEALK | | Mascot |
| 1190.573 | 1190.6184 | 0.0454 | 38 | 6663 | 6671 | HMLMELLCK | Carbamidomethyl (C)[8], Oxidation (M)[2] | Mascot |
| 1221.6144 | 1221.5538 | -0.0606 | -50 | 5301 | 5311 | TLQQGVTMSEK | | Mascot |
| 1279.6562 | 1279.7423 | 0.0861 | 67 | 5890 | 5900 | TLKQDVTMSEK | | Mascot |
| 1310.7175 | 1310.6559 | -0.0616 | -47 | 4148 | 4158 | TLNLHTEAKQR | | Mascot |
| 1328.6416 | 1328.5773 | -0.0643 | -48 | 1103 | 1113 | DGSIRFCIDFR | | Mascot |
| 1391.7351 | 1391.7091 | -0.026 | -19 | 8002 | 8013 | GGTWMLVIDQKK | Oxidation (M)[5] | Mascot |
| 1413.7526 | 1413.7682 | 0.0156 | 11 | 2698 | 2709 | TPIKHFFDSPPK | | Mascot |
| 1457.6794 | 1457.7173 | 0.0379 | 26 | 7671 | 7683 | SESAPALTEYDFK | | Mascot |
| 1463.6649 | 1463.7169 | 0.052 | 36 | 3192 | 3204 | TSDQSYSSVSFQK | | Mascot |
| 1475.7522 | 1475.7218 | -0.0304 | -21 | 6801 | 6813 | QEAKILECSVAER | | Mascot |
| 1485.705 | 1485.712 | 0.007 | 5 | 3155 | 3165 | CLEQQRHCVQK | Carbamidomethyl (C)[1,8] | Mascot |
| 1616.8391 | 1616.7039 | -0.1352 | -84 | 3905 | 3919 | ISHTSIYHGSSKTAK | | Mascot |
| 1632.8447 | 1632.6975 | -0.1472 | -90 | 5443 | 5457 | LTEKSMPAVASPVMR | Oxidation (M)[6] | Mascot |
| 1637.7874 | 1637.7745 | -0.0129 | -8 | 6757 | 6771 | MACQETADKIEAISK | | Mascot |
| 1638.9438 | 1638.8115 | -0.1323 | -81 | 3297 | 3310 | HVLKEPTLPALQHR | | Mascot |
| 1638.9438 | 1638.8115 | -0.1323 | -81 | 3297 | 3310 | HVLKEPTLPALQHR | | Mascot |
| 1648.7847 | 1648.6963 | -0.0884 | -54 | 7781 | 7796 | MPDTESAKPGKDSGTK | | Mascot |
| 1651.7719 | 1651.7683 | -0.0036 | -2 | 605 | 616 | ERFCMYQGLLYR | Carbamidomethyl (C)[4], Oxidation (M)[5] | Mascot |
| 1670.8141 | 1670.817 | 0.0029 | 2 | 7083 | 7096 | CSAPVDLMLGHLWR | Carbamidomethyl (C)[1], Oxidation (M)[8] | Mascot |
| 1682.8088 | 1682.7767 | -0.0321 | -19 | 4188 | 4202 | LTEKSMATEASPVMR | Oxidation (M)[6,14] | Mascot |
| 1755.9785 | 1755.9348 | -0.0437 | -25 | 1231 | 1247 | LQAAGLTVNPQKCTIAK | | Mascot |
| 2191.8423 | 2192.1182 | 0.2759 | 126 | 7839 | 7857 | MSESGDEKEEPMNGEM STR | Oxidation (M)[1,12,16] | Mascot |
| 2191.8423 | 2192.1182 | 0.2759 | 126 | 7839 | 7857 | MSESGDEKEEPMNGEM STR | Oxidation (M)[1,12,16] | Mascot |
| 2207.207 | 2207.0408 | -0.1662 | -75 | 6697 | 6714 | IHLLELQAKEHEEYLTLK | | Mascot |
| 2211.126 | 2211.0518 | -0.0742 | -34 | 8508 | 8526 | MSAAELQTTGVIYRLELC R | Carbamidomethyl (C)[18] | Mascot |
| 2221.1406 | 2221.103 | -0.0376 | -17 | 3041 | 3057 | HQLIQQWREQQICVQAR | Carbamidomethyl (C)[13] | Mascot |
| 2224.9873 | 2225.0647 | 0.0774 | 35 | 4312 | 4333 | VTDAETSSQIGACAEDNE SGIK | | Mascot |
| 2224.9873 | 2225.0647 | 0.0774 | 35 | 4312 | 4333 | VTDAETSSQIGACAEDNE | | Mascot |

| Calc. Mass | Obsrv. Mass | ± da | ± ppm | Start Seq. | End Seq. | Sequence | Ion Score | C. I. % | Modification | Rank | Result Type |
|---|---|---|---|---|---|---|---|---|---|---|---|
| | | | | | | SGIK | | | | | |
| 2230.1643 | 2230.1079 | -0.0564 | -25 | 6653 | 6671 | DSLLALESVKHMLMELLCK | | | Carbamidomethyl (C)[18] | | Mascot |
| 2233.0012 | 2233.03 | 0.0288 | 13 | 8235 | 8252 | DSTELEEWMRCAHEQLQK | | | | | Mascot |
| 2283.1333 | 2283.1248 | -0.0085 | -4 | 7195 | 7214 | ESPQICQDLQSLLGHCRSLR | | | | | Mascot |
| 2283.1333 | 2283.1248 | -0.0085 | -4 | 7195 | 7214 | ESPQICQDLQSLLGHCRSLR | | | | | Mascot |
| 2284.022 | 2284.1189 | 0.0969 | 42 | 3439 | 3458 | RVLDCTDAPFESEADTLMDR | | | | | Mascot |
| 2297.1455 | 2297.0979 | -0.0476 | -21 | 8621 | 8639 | VLLNQHWERLGELMNSSDR | | | | | Mascot |
| 2299.0984 | 2299.1296 | 0.0312 | 14 | 2881 | 2901 | VEDIQHLTVSSSDLAPCQNSR | | | | | Mascot |
| 2306.0176 | 2306.1018 | 0.0842 | 37 | 8235 | 8252 | DSTELEEWMRCAHEQLQK | | | Carbamidomethyl (C)[11], Oxidation (M)[9] | | Mascot |
| 2501.1218 | 2501.1995 | 0.0777 | 31 | 2814 | 2835 | GMGDFCSDAQLRDMDLLSQSVR | | | Carbamidomethyl (C)[6] | | Mascot |
| 2584.248 | 2584.1545 | -0.0935 | -36 | 4168 | 4191 | CASIEATEMSLGTGDIVQSKLTEK | | | Carbamidomethyl (C)[1], Oxidation (M)[9] | | Mascot |
| 2872.3003 | 2872.3237 | 0.0234 | 8 | 659 | 681 | HFYWPGLNSVVAQYCKTCPECQK | | | Carbamidomethyl (C)[15,18,21] | | Mascot |

24 tektin 2 [Danio rerio] gi|310689065 48533.7 5.63 9 34 0

Peptide Information

| Calc. Mass | Obsrv. Mass | ± da | ± ppm | Start Seq. | End Seq. | Sequence | Ion Score | C. I. % | Modification | Rank | Result Type |
|---|---|---|---|---|---|---|---|---|---|---|---|
| 1045.5789 | 1045.5411 | -0.0378 | -36 | 68 | 75 | INDITRWK | | | | | Mascot |
| 1190.5834 | 1190.6184 | 0.035 | 29 | 231 | 240 | EEMQASVQIR | | | | | Mascot |
| 1328.6943 | 1328.5773 | -0.117 | -88 | 127 | 138 | ELVSDPVEAELK | | | | | Mascot |
| 1579.8915 | 1579.7042 | -0.1873 | -119 | 354 | 367 | QKLAQAQHSLQSLK | | | | | Mascot |
| 1616.752 | 1616.7039 | -0.0481 | -30 | 368 | 380 | QHEAQMMEELSRK | | | | | Mascot |
| 1632.7468 | 1632.6975 | -0.0493 | -30 | 368 | 380 | QHEAQMMEELSRK | | | Oxidation (M)[6] | | Mascot |
| 1648.7417 | 1648.6963 | -0.0454 | -28 | 368 | 380 | QHEAQMMEELSRK | | | Oxidation (M)[6,7] | | Mascot |
| 1854.0219 | 1853.8535 | -0.1684 | -91 | 296 | 312 | DIIGLEEDLQAKTAPLK | | | | | Mascot |
| 2211.0261 | 2211.0518 | 0.0257 | 12 | 268 | 284 | RTHHEEQAHHDMQWQLK | | | | | Mascot |
| 2283.1365 | 2283.1248 | -0.0117 | -5 | 2 | 22 | ATLSSKPASRYSVSDWATNNK | | | | | Mascot |
| 2283.1365 | 2283.1248 | -0.0117 | -5 | 2 | 22 | ATLSSKPASRYSVSDWATNNK | | | | | Mascot |
| 2584.1211 | 2584.1545 | 0.0334 | 13 | 80 | 101 | ACAQEVDEEMDALTLSKEEMER | | | Carbamidomethyl (C)[2] | | Mascot |

25 steroid hormone receptor ERR1 [Danio rerio] gi|47086129 47615.2 8.16 9 33 0

Peptide Information

| Calc. Mass | Obsrv. Mass | ± da | ± ppm | Start Seq. | End Seq. | Sequence | Ion Score | C. I. % | Modification | Rank | Result Type |
|---|---|---|---|---|---|---|---|---|---|---|---|

| | | | | | | | | | | | |
|---|---|---|---|---|---|---|---|---|---|---|---|
| 1051.7238 | 1051.6859 | -0.0379 | -36 | 392 | 400 | LLLTLPLLR | | | | | Mascot |
| 1221.6144 | 1221.5538 | -0.0606 | -50 | 239 | 249 | TLTTLCDLADR | | | | | Mascot |
| 1277.6888 | 1277.6538 | -0.035 | -27 | 55 | 65 | YSPPLYTPALR | | | | | Mascot |
| 1328.5825 | 1328.5773 | -0.0052 | -4 | 14 | 29 | AEPSSPEGGGGGGGGR | | | | | Mascot |
| 1335.8834 | 1335.6929 | -0.1905 | -143 | 389 | 400 | AGRLLLTLPLLR | | | | | Mascot |
| 1626.9174 | 1626.7155 | -0.2019 | -124 | 314 | 329 | VAGLTELNAAISQLAR | | | | | Mascot |
| 1638.6949 | 1638.8115 | 0.1166 | 71 | 30 | 48 | TSPGGASSDSSQSGGG GSR | | | | | Mascot |
| 1638.6949 | 1638.8115 | 0.1166 | 71 | 30 | 48 | TSPGGASSDSSQSGGG GSR | | | | | Mascot |
| 1853.7566 | 1853.8535 | 0.0969 | 52 | 70 | 89 | EEGADGAEEGSTGSGG GRCK | | | | | Mascot |
| 2233.0525 | 2233.03 | -0.0225 | -10 | 417 | 436 | GGVPMHKLFLEMLEAMM DSP | | | | | Mascot |
| 2297.032 | 2297.0979 | 0.0659 | 29 | 417 | 436 | GGVPMHKLFLEMLEAMM DSP | | | Oxidation (M)[5,12,16,17] | | Mascot |

| | | | | | | | |
|---|---|---|---|---|---|---|---|
| 26 | PREDICTED: pleckstrin homology domain-containing family H member 2 [Danio rerio] | gi\|326672868 | 159281.4 | 6.64 | 17 | 33 | 0 |

**Peptide Information**

| Calc. Mass | Obsrv. Mass | ± da | ± ppm | Start Seq. | End Seq. | Sequence | Ion Score | C. I. % | Modification | Rank | Result Type |
|---|---|---|---|---|---|---|---|---|---|---|---|
| 842.4195 | 842.4905 | 0.071 | 84 | 1269 | 1275 | WSFFGAK | | | | | Mascot |
| 864.4396 | 864.4717 | 0.0321 | 37 | 131 | 136 | EWVMRK | | | Oxidation (M)[4] | | Mascot |
| 996.4236 | 996.572 | 0.1484 | 149 | 1073 | 1080 | LNQDMGMR | | | Oxidation (M)[5,7] | | Mascot |
| 996.4236 | 996.572 | 0.1484 | 149 | 1073 | 1080 | LNQDMGMR | | | Oxidation (M)[5,7] | | Mascot |
| 1165.7191 | 1165.7177 | -0.0014 | -1 | 358 | 368 | SPILTPVLTPK | | | | | Mascot |
| 1165.7191 | 1165.7177 | -0.0014 | -1 | 358 | 368 | SPILTPVLTPK | | | | | Mascot |
| 1179.6521 | 1179.5767 | -0.0754 | -64 | 1217 | 1225 | QVLERFYPK | | | | | Mascot |
| 1190.576 | 1190.6184 | 0.0424 | 36 | 1147 | 1156 | QVSGETERER | | | | | Mascot |
| 1221.6321 | 1221.5538 | -0.0783 | -64 | 1332 | 1344 | LVVAESSGSGTSK | | | | | Mascot |
| 1279.6012 | 1279.7423 | 0.1411 | 110 | 213 | 224 | SISTLQEDSGDK | | | | | Mascot |
| 1310.5317 | 1310.6559 | 0.1242 | 95 | 2 | 13 | AECDESTGGAWK | | | Carbamidomethyl (C)[3] | | Mascot |
| 1443.6686 | 1443.6772 | 0.0086 | 6 | 787 | 797 | MWEARVEEEHK | | | | | Mascot |
| 1457.5671 | 1457.7173 | 0.1502 | 103 | 1 | 13 | MAECDESTGGAWK | | | Carbamidomethyl (C)[4], Oxidation (M)[1] | | Mascot |
| 1463.7965 | 1463.7169 | -0.0796 | -54 | 151 | 162 | HQQLEAEIQVLR | | | | | Mascot |
| 1475.7827 | 1475.7218 | -0.0609 | -41 | 780 | 791 | FPLGQIKMWEAR | | | | | Mascot |
| 1797.8282 | 1797.9182 | 0.09 | 50 | 254 | 271 | LSCGASSVLSSTASEGE R | | | Carbamidomethyl (C)[3] | | Mascot |
| 1853.9537 | 1853.8535 | -0.1002 | -54 | 1252 | 1267 | GQTSSECIRIYLTVAR | | | Carbamidomethyl (C)[7] | | Mascot |
| 2230.1497 | 2230.1079 | -0.0418 | -19 | 676 | 695 | KPLGQIELNACSTITRGD EK | | | Carbamidomethyl (C)[11] | | Mascot |
| 3337.6892 | 3337.6814 | -0.0078 | -2 | 1369 | 1399 | STQQHMAAAHHLSAPAL | | | | | Mascot |

LLALSAEMKNNEPR

| | | | | | | | |
|---|---|---|---|---|---|---|---|
| 27 | amylo-1, 6-glucosidase, 4-alpha-glucanotransferase [Danio rerio] | gi\|289547621 | 173383.7 | 6.08 | 17 | 33 | 0 |

Peptide Information

| Calc. Mass | Obsrv. Mass | ± da | ± ppm | Start Seq. | End Seq. | Sequence | Ion Score | C. I. % | Modification | Rank | Result Type |
|---|---|---|---|---|---|---|---|---|---|---|---|
| 850.5257 | 850.5237 | -0.002 | -2 | 422 | 428 | KHPLVTR | | | | | Mascot |
| 850.5257 | 850.5237 | -0.002 | -2 | 422 | 428 | KHPLVTR | | | | | Mascot |
| 1104.6047 | 1104.5546 | -0.0501 | -45 | 306 | 314 | IEALVEQFR | | | | | Mascot |
| 1104.6047 | 1104.5546 | -0.0501 | -45 | 306 | 314 | IEALVEQFR | | | | | Mascot |
| 1182.6113 | 1182.5671 | -0.0442 | -37 | 781 | 790 | TVERQTGTYK | | | | | Mascot |
| 1182.6113 | 1182.5671 | -0.0442 | -37 | 781 | 790 | TVERQTGTYK | | | | | Mascot |
| 1190.6085 | 1190.6184 | 0.0099 | 8 | 1280 | 1291 | DGSAVEIVGLCK | | | | | Mascot |
| 1328.6117 | 1328.5773 | -0.0344 | -26 | 45 | 55 | VYTNYPTEGER | | | | | Mascot |
| 1391.5492 | 1391.7091 | 0.1599 | 115 | 579 | 591 | EAMSAGDSHEEGR | | | Oxidation (M)[3] | | Mascot |
| 1413.722 | 1413.7682 | 0.0462 | 33 | 876 | 889 | AGSVPDSNTPDILK | | | | | Mascot |
| 1443.8358 | 1443.6772 | -0.1586 | -110 | 286 | 297 | GVLWQEVFPKIK | | | | | Mascot |
| 1457.6591 | 1457.7173 | 0.0582 | 40 | 950 | 961 | NDLGHPFCDNLR | | | Carbamidomethyl (C)[8] | | Mascot |
| 1463.7345 | 1463.7169 | -0.0176 | -12 | 5 | 16 | QTRVMVLNDMEK | | | | | Mascot |
| 1475.7675 | 1475.7218 | -0.0457 | -31 | 483 | 494 | ELICWGDSVKLR | | | Carbamidomethyl (C)[4] | | Mascot |
| 1616.7849 | 1616.7039 | -0.081 | -50 | 136 | 148 | CLGPLDEWSDRLR | | | Carbamidomethyl (C)[1] | | Mascot |
| 1637.7635 | 1637.7745 | 0.011 | 7 | 735 | 748 | HCPSTHQSVVAVCR | | | Carbamidomethyl (C)[2,13] | | Mascot |
| 2210.9812 | 2211.0518 | 0.0706 | 32 | 1334 | 1351 | NFEKMFYVSHDPNDPNEK | | | | | Mascot |
| 2297.0801 | 2297.0979 | 0.0178 | 8 | 525 | 544 | LDNCHSTPLHVAEFMLDAAR | | | Carbamidomethyl (C)[4] | | Mascot |
| 2306.0461 | 2306.1018 | 0.0557 | 24 | 636 | 658 | SVYDSLPSSAIVSMACCATGSTR | | | | | Mascot |
| 2584.3379 | 2584.1545 | -0.1834 | -71 | 974 | 996 | LVSRGGPLAEVGQWFQAMFAYLK | | | Oxidation (M)[18] | | Mascot |

| | | | | | | | |
|---|---|---|---|---|---|---|---|
| 28 | ryanodine receptor 1b (skeletal) [Danio rerio] | gi\|169146264 | 308125.7 | 4.98 | 23 | 33 | 0 |

Peptide Information

| Calc. Mass | Obsrv. Mass | ± da | ± ppm | Start Seq. | End Seq. | Sequence | Ion Score | C. I. % | Modification | Rank | Result Type |
|---|---|---|---|---|---|---|---|---|---|---|---|
| 864.4495 | 864.4717 | 0.0222 | 26 | 1832 | 1839 | IEIMGASK | | | Oxidation (M)[4] | | Mascot |
| 1042.5527 | 1042.557 | 0.0043 | 4 | 1592 | 1600 | DIIDEPGKR | | | | | Mascot |
| 1045.5425 | 1045.5411 | -0.0014 | -1 | 300 | 307 | LLTNHYER | | | | | Mascot |
| 1051.5605 | 1051.6859 | 0.1254 | 119 | 157 | 165 | ASMVLFLDR | | | | | Mascot |
| 1104.587 | 1104.5546 | -0.0324 | -29 | 1291 | 1299 | MTPLYNIPR | | | | | Mascot |

| | | | | | | | | | | | |
|---|---|---|---|---|---|---|---|---|---|---|---|
| 1104.62 | 1104.5546 | -0.0654 | -59 | 460 | 467 | EIYRWPIK | | | | | Mascot |
| 1118.5299 | 1118.604 | 0.0741 | 66 | 147 | 156 | MSASFVPDHK | | | | | Mascot |
| 1120.5819 | 1120.5374 | -0.0445 | -40 | 1291 | 1299 | MTPLYNIPR | | | Oxidation (M)[1] | | Mascot |
| 1413.7419 | 1413.7682 | 0.0263 | 19 | 1291 | 1301 | MTPLYNIPRHR | | | Oxidation (M)[1] | | Mascot |
| 1443.7994 | 1443.6772 | -0.1222 | -85 | 1953 | 1964 | SIVNFLSLFTFR | | | | | Mascot |
| 1457.7094 | 1457.7173 | 0.0079 | 5 | 1224 | 1235 | WQMALYKETSGK | | | Oxidation (M)[3] | | Mascot |
| 1463.5872 | 1463.7169 | 0.1297 | 89 | 2692 | 2703 | CWDFFPAGDCFR | | | | | Mascot |
| 1531.7322 | 1531.7145 | -0.0177 | -12 | 1854 | 1865 | NQWEMPQVRESK | | | | | Mascot |
| 1579.7971 | 1579.7042 | -0.0929 | -59 | 233 | 245 | AIMIDSMLHTIYR | | | Oxidation (M)[3] | | Mascot |
| 1648.7035 | 1648.6963 | -0.0072 | -4 | 2692 | 2704 | CWDFFPAGDCFRK | | | Carbamidomethyl (C)[1] | | Mascot |
| 1651.7228 | 1651.7683 | 0.0455 | 28 | 2619 | 2631 | DQQEQVREDMETK | | | Oxidation (M)[10] | | Mascot |
| 1670.882 | 1670.817 | -0.065 | -39 | 1457 | 1472 | LGISILNGGNSDVQQR | | | | | Mascot |
| 1682.9635 | 1682.7767 | -0.1868 | -111 | 270 | 282 | YLRPSMLQHLLRR | | | | | Mascot |
| 1755.8547 | 1755.9348 | 0.0801 | 46 | 749 | 764 | AGLRSFFESAADDIEK | | | | | Mascot |
| 1853.9027 | 1853.8535 | -0.0492 | -27 | 1866 | 1881 | RQFIFDVVNEGGESEK | | | | | Mascot |
| 2229.8894 | 2230.1079 | 0.2185 | 98 | 2550 | 2566 | CDDMMTCYLFHMYVGVR | | | Carbamidomethyl (C)[1,7], Oxidation (M)[4,5] | | Mascot |
| 2283.2205 | 2283.1248 | -0.0957 | -42 | 1050 | 1068 | DEFAVLCRDLYALYPLLIR | | | | | Mascot |
| 2283.2205 | 2283.1248 | -0.0957 | -42 | 1050 | 1068 | DEFAVLCRDLYALYPLLIR | | | | | Mascot |
| 2872.5247 | 2872.3237 | -0.201 | -70 | 258 | 281 | DVIEDCLMSLVKYLRPSM LQHLLR | | | | | Mascot |
| 3337.5386 | 3337.6814 | 0.1428 | 43 | 946 | 975 | GPENFPEQEGCLCTDVT SEQLNQLLGSIMK | | | Carbamidomethyl (C)[11] | | Mascot |

29 Ppp1r3b protein [Danio rerio] gi|190337192 33606.4 5.74 8 33 0

**Peptide Information**

| Calc. Mass | Obsrv. Mass | ± da | ± ppm | Start Seq. | End Seq. | Sequence | Ion Score | C. I. % | Modification | Rank | Result Type |
|---|---|---|---|---|---|---|---|---|---|---|---|
| 1051.5782 | 1051.6859 | 0.1077 | 102 | 157 | 165 | NISFEKSVK | | | | | Mascot |
| 1179.6521 | 1179.5767 | -0.0754 | -64 | 166 | 174 | LRITFDTWK | | | | | Mascot |
| 1233.7062 | 1233.5854 | -0.1208 | -98 | 233 | 242 | NYRIIQSALR | | | | | Mascot |
| 1233.7062 | 1233.5854 | -0.1208 | -98 | 233 | 242 | NYRIIQSALR | | | | | Mascot |
| 1335.5997 | 1335.6929 | 0.0932 | 70 | 175 | 185 | NHTDVECQYVK | | | | | Mascot |
| 1797.8081 | 1797.9182 | 0.1101 | 61 | 132 | 145 | IENDHVCLEHCMLK | | | Carbamidomethyl (C)[7,11] | | Mascot |
| 2211.1001 | 2211.0518 | -0.0483 | -22 | 2 | 20 | PIELAMSLYLSSDEFLNP R | | | Oxidation (M)[6] | | Mascot |
| 2229.9829 | 2230.1079 | 0.125 | 56 | 175 | 193 | NHTDVECQYVKDTYTGS NR | | | | | Mascot |
| 2284.0703 | 2284.1189 | 0.0486 | 21 | 168 | 185 | ITFDTWKNHTDVECQYVK | | | Carbamidomethyl (C)[14] | | Mascot |

30 PREDICTED: hypothetical protein LOC562472 [Danio rerio] gi|68362780 171051.4 8.98 19 32 0

Peptide Information

| Calc. Mass | Obsrv. Mass | ± da | ± ppm | Start Seq. | End Seq. | Sequence | Ion Score | C. I. % | Modification | Rank | Result Type |
|---|---|---|---|---|---|---|---|---|---|---|---|
| 882.5229 | 882.5462 | 0.0233 | 26 | 758 | 764 | LMQLLHK | | | | | Mascot |
| 996.5109 | 996.572 | 0.0611 | 61 | 375 | 382 | DKVTYTNR | | | | | Mascot |
| 996.5109 | 996.572 | 0.0611 | 61 | 375 | 382 | DKVTYTNR | | | | | Mascot |
| 1042.4663 | 1042.557 | 0.0907 | 87 | 1111 | 1118 | FDTYPMPR | | | Oxidation (M)[6] | | Mascot |
| 1051.5605 | 1051.6859 | 0.1254 | 119 | 361 | 369 | LTSLCYVPR | | | | | Mascot |
| 1062.5612 | 1062.6647 | 0.1035 | 97 | 206 | 214 | DLREALMAK | | | Oxidation (M)[7] | | Mascot |
| 1233.6949 | 1233.5854 | -0.1095 | -89 | 1342 | 1352 | KFLVQVDASAR | | | | | Mascot |
| 1233.6949 | 1233.5854 | -0.1095 | -89 | 1342 | 1352 | KFLVQVDASAR | | | | | Mascot |
| 1277.6848 | 1277.6538 | -0.031 | -24 | 75 | 84 | REEGLIYELR | | | | | Mascot |
| 1310.6012 | 1310.6559 | 0.0547 | 42 | 798 | 808 | FVSDNGKDWDK | | | | | Mascot |
| 1335.7518 | 1335.6929 | -0.0589 | -44 | 1383 | 1394 | YATIEKEGLAIK | | | | | Mascot |
| 1463.7642 | 1463.7169 | -0.0473 | -32 | 1140 | 1151 | GYWQVPLEKTSR | | | | | Mascot |
| 1485.7656 | 1485.712 | -0.0536 | -36 | 118 | 131 | GPSVTQNPTLSTQR | | | | | Mascot |
| 1531.8049 | 1531.7145 | -0.0904 | -59 | 1225 | 1238 | AGLTLNVAKCEWAR | | | | | Mascot |
| 1579.8148 | 1579.7042 | -0.1106 | -70 | 361 | 374 | LTSLCYVPREGDVK | | | | | Mascot |
| 1632.8163 | 1632.6975 | -0.1188 | -73 | 132 | 146 | VADSPPPPPMRPESR | | | | | Mascot |
| 1638.8308 | 1638.8115 | -0.0193 | -12 | 1105 | 1118 | LNAVSKFDTYPMPR | | | | | Mascot |
| 1638.8308 | 1638.8115 | -0.0193 | -12 | 1105 | 1118 | LNAVSKFDTYPMPR | | | | | Mascot |
| 1648.8112 | 1648.6963 | -0.1149 | -70 | 132 | 146 | VADSPPPPPMRPESR | | | Oxidation (M)[10] | | Mascot |
| 1797.8112 | 1797.9182 | 0.107 | 60 | 147 | 161 | MPAFQEGEDVENYLR | | | | | Mascot |
| 2297.1328 | 2297.0979 | -0.0349 | -15 | 500 | 521 | ADLEPLPAMDDSLIQGEG GKPK | | | Oxidation (M)[9] | | Mascot |
| 2501.2903 | 2501.1995 | -0.0908 | -36 | 1167 | 1188 | TMPFGLHGAPATFQRLM NQVLR | | | Oxidation (M)[2] | | Mascot |
| 2584.2712 | 2584.1545 | -0.1167 | -45 | 848 | 870 | SWEASSTTAEGETNIVKF VLQMR | | | | | Mascot |

| | | | | | | | | |
|---|---|---|---|---|---|---|---|---|
| 31 | PREDICTED: hypothetical protein LOC560599 [Danio rerio] | gi\|125820806 | 23741.5 | 4.38 | 6 | 32 | 0 | |

Peptide Information

| Calc. Mass | Obsrv. Mass | ± da | ± ppm | Start Seq. | End Seq. | Sequence | Ion Score | C. I. % | Modification | Rank | Result Type |
|---|---|---|---|---|---|---|---|---|---|---|---|
| 1120.5004 | 1120.5374 | 0.037 | 33 | 156 | 164 | KEEEVEDDK | | | | | Mascot |
| 1469.7819 | 1469.7172 | -0.0647 | -44 | 55 | 67 | DQLRQGVGVVENR | | | | | Mascot |
| 1475.7787 | 1475.7218 | -0.0569 | -39 | 46 | 58 | MVFAPGGLRDQLR | | | Oxidation (M)[1] | | Mascot |
| 1651.8398 | 1651.7683 | -0.0715 | -43 | 32 | 45 | KLNTETNGQYTVQR | | | | | Mascot |

| | | | | | | | | | | | | |
|---|---|---|---|---|---|---|---|---|---|---|---|---|
| 2233.1624 | 2233.03 | -0.1324 | -59 | 59 | 77 | QGVGVVENRFDVHIWPQPR | | | | | | Mascot |
| 2298.8535 | 2299.1296 | 0.2761 | 120 | 87 | 105 | DEDDENDQDKQNDTCTEER | | | | | | Mascot |

32 glutathione peroxidase 1b [Danio rerio] gi|169636422 21638 7.66 6 32 0

Peptide Information

| Calc. Mass | Obsrv. Mass | ± da | ± ppm | Start Seq. | End Seq. | Sequence | Ion Score | C. I. % | Modification | Rank | Result Type |
|---|---|---|---|---|---|---|---|---|---|---|---|
| 1120.5972 | 1120.5374 | -0.0598 | -53 | 139 | 147 | FIIWSPVCR | | | | | Mascot |
| 1165.6099 | 1165.7177 | 0.1078 | 92 | 173 | 183 | FLTSGIDGDIK | | | | | Mascot |
| 1165.6099 | 1165.7177 | 0.1078 | 92 | 173 | 183 | FLTSGIDGDIK | | | | | Mascot |
| 1179.6409 | 1179.5767 | -0.0642 | -54 | 157 | 167 | FLIGSDGVPFK | | | | | Mascot |
| 1279.6429 | 1279.7423 | 0.0994 | 78 | 88 | 98 | YVRPGNGYEPK | | | | | Mascot |
| 1335.7419 | 1335.6929 | -0.049 | -37 | 157 | 168 | FLIGSDGVPFKR | | | | | Mascot |
| 2297.1602 | 2297.0979 | -0.0623 | -27 | 148 | 167 | NDIAWNFEKFLIGSDGVPFK | | | | | Mascot |

33 unnamed protein product [Danio rerio] gi|157676717 68416.5 6.64 10 32 0

Peptide Information

| Calc. Mass | Obsrv. Mass | ± da | ± ppm | Start Seq. | End Seq. | Sequence | Ion Score | C. I. % | Modification | Rank | Result Type |
|---|---|---|---|---|---|---|---|---|---|---|---|
| 1104.616 | 1104.5546 | -0.0614 | -56 | 415 | 424 | GVFANLTNLR | | | | | Mascot |
| 1104.616 | 1104.5546 | -0.0614 | -56 | 415 | 424 | GVFANLTNLR | | | | | Mascot |
| 1165.5959 | 1165.7177 | 0.1218 | 104 | 323 | 332 | NGQIVSYQTR | | | | | Mascot |
| 1165.5959 | 1165.7177 | 0.1218 | 104 | 323 | 332 | NGQIVSYQTR | | | | | Mascot |
| 1277.6228 | 1277.6538 | 0.031 | 24 | 569 | 578 | ELKNEIMCPK | | | Carbamidomethyl (C)[8], Oxidation (M)[7] | | Mascot |
| 1443.6793 | 1443.6772 | -0.0021 | -1 | 333 | 345 | IPPLSCPQPCTCK | | | Carbamidomethyl (C)[6] | | Mascot |
| 1463.7311 | 1463.7169 | -0.0142 | -10 | 556 | 568 | CASPVQFSNIELR | | | | | Mascot |
| 1616.9006 | 1616.7039 | -0.1967 | -122 | 95 | 110 | LQNIEGGAFVGLSALK | | | | | Mascot |
| 1632.7434 | 1632.6975 | -0.0459 | -28 | 346 | 360 | AHPSDFGISVSCQER | | | | | Mascot |
| 1670.7148 | 1670.817 | 0.1022 | 61 | 259 | 273 | QELCPMGTGSDFDVR | | | Oxidation (M)[6] | | Mascot |
| 1718.98 | 1718.8324 | -0.1476 | -86 | 361 | 376 | NIQSLADLIPKPPNAK | | | | | Mascot |
| 2584.0491 | 2584.1545 | 0.1054 | 41 | 24 | 45 | AETCSACSCMSIENVLYVNCEK | | | Carbamidomethyl (C)[4,7,9], Oxidation (M)[10] | | Mascot |

34 adenylyl cyclase-associated protein 2 [Danio rerio] gi|41387192 51394.1 5.94 9 32 0

Peptide Information

| Calc. Mass | Obsrv. Mass | ± da | ± ppm | Start Seq. | End Seq. | Sequence | Ion Score | C. I. % | Modification | Rank | Result Type |
|---|---|---|---|---|---|---|---|---|---|---|---|
| 842.5206 | 842.4905 | -0.0301 | -36 | 8 | 14 | LERAVVR | | | | | Mascot |

| | | | | | | | | | | | |
|---|---|---|---|---|---|---|---|---|---|---|---|
| 1042.6003 | 1042.557 | -0.0433 | -42 | 2 | 10 | EALVGRLER | | | | | Mascot |
| 1104.5718 | 1104.5546 | -0.0172 | -16 | 360 | 369 | VNSIIVDNCK | | | | | Mascot |
| 1104.5718 | 1104.5546 | -0.0172 | -16 | 360 | 369 | VNSIIVDNCK | | | | | Mascot |
| 1118.6204 | 1118.604 | -0.0164 | -15 | 311 | 320 | HPPVLELEGK | | | | | Mascot |
| 1120.5674 | 1120.5374 | -0.03 | -27 | 443 | 451 | EFPVPEQFK | | | | | Mascot |
| 1179.5562 | 1179.5767 | 0.0205 | 17 | 416 | 426 | DSLDCEIVSAK | | | | | Mascot |
| 1310.631 | 1310.6559 | 0.0249 | 19 | 201 | 211 | EHHCTGLIWSK | | | | | Mascot |
| 1853.8221 | 1853.8535 | 0.0314 | 17 | 427 | 442 | SSEMNILVPEGEDDYR | | | | | Mascot |
| 2297.0789 | 2297.0979 | 0.019 | 8 | 406 | 426 | TEGCHVYLSKDSLDCEIV SAK | | | | | Mascot |

| | | | | | | | |
|---|---|---|---|---|---|---|---|
| 35 | caspase-2 [Danio rerio] | gi\|111607486 | 49545.4 | 6.17 | 8 | 32 | 0 |

**Peptide Information**

| Calc. Mass | Obsrv. Mass | ± da | ± ppm | Start Seq. | End Seq. | Sequence | Ion Score | C. I. % | Modification | Rank | Result Type |
|---|---|---|---|---|---|---|---|---|---|---|---|
| 996.507 | 996.572 | 0.065 | 65 | 93 | 100 | LLMDFTEK | | | | | Mascot |
| 996.507 | 996.572 | 0.065 | 65 | 93 | 100 | LLMDFTEK | | | | | Mascot |
| 1042.4366 | 1042.557 | 0.1204 | 115 | 1 | 9 | MLGECGMTK | | | Carbamidomethyl (C)[5], Oxidation (M)[1] | | Mascot |
| 1062.5361 | 1062.6647 | 0.1286 | 121 | 213 | 221 | DLTAEAMRR | | | | | Mascot |
| 1104.5167 | 1104.5546 | 0.0379 | 34 | 189 | 198 | GGEVDEETLR | | | | | Mascot |
| 1104.5167 | 1104.5546 | 0.0379 | 34 | 189 | 198 | GGEVDEETLR | 10 | 0 | | | Mascot |
| 1277.5388 | 1277.6538 | 0.115 | 90 | 413 | 423 | EMSEFTSSLCK | | | Oxidation (M)[2] | | Mascot |
| 1469.6703 | 1469.7172 | 0.0469 | 32 | 400 | 412 | EGYAPGSAHHRCK | | | Carbamidomethyl (C)[12] | | Mascot |
| 2207.189 | 2207.0408 | -0.1482 | -67 | 380 | 399 | ANNTHLSDILVQVNGQIK SR | | | | | Mascot |
| 2211.0349 | 2211.0518 | 0.0169 | 8 | 413 | 430 | EMSEFTSSLCKDLYLFPK | | | Carbamidomethyl (C)[10], Oxidation (M)[2] | | Mascot |

| | | | | | | | | | | |
|---|---|---|---|---|---|---|---|---|---|---|
| 36 | alpha-1,3-mannosyl-glycoprotein 4-beta-N-acetylglucosaminyltransferase C [Danio rerio] | gi\|55925363 | 52220.8 | 7.19 | 6 | 32 | 0 | 17 | 0 |

**Peptide Information**

| Calc. Mass | Obsrv. Mass | ± da | ± ppm | Start Seq. | End Seq. | Sequence | Ion Score | C. I. % | Modification | Rank | Result Type |
|---|---|---|---|---|---|---|---|---|---|---|---|
| 1104.5181 | 1104.5546 | 0.0365 | 33 | 161 | 169 | RNFNDAPDR | | | | | Mascot |
| 1104.5181 | 1104.5546 | 0.0365 | 33 | 161 | 169 | RNFNDAPDR | 17 | 0 | | | Mascot |
| 1120.6949 | 1120.5374 | -0.1575 | -141 | 141 | 150 | GRLLVIHANK | | | | | Mascot |
| 1277.7212 | 1277.6538 | -0.0674 | -53 | 130 | 140 | ISREFAVQLSK | | | | | Mascot |
| 1310.7216 | 1310.6559 | -0.0657 | -50 | 151 | 161 | EHYPPLTGLKR | | | | | Mascot |
| 1469.811 | 1469.7172 | -0.0938 | -64 | 430 | 442 | VTAAQPEWVIISR | | | | | Mascot |
| 2297.1367 | 2297.0979 | -0.0388 | -17 | 364 | 385 | VQIHTGSDGKDELASADV | | | | | Mascot |

ELGR

| | | | | | | | |
|---|---|---|---|---|---|---|---|
| 37 | arf-GAP with coiled-coil, ANK repeat and PH domain-containing protein 3 [Danio rerio] | gi\|131889644 | 94940.4 | 5.88 | 13 | 32 | 0 |

Peptide Information

| Calc. Mass | Obsrv. Mass | ± da | ± ppm | Start Seq. | End Seq. | Sequence | Ion Score | C. I. % | Modification | Rank | Result Type |
|---|---|---|---|---|---|---|---|---|---|---|---|
| 842.4478 | 842.4905 | 0.0427 | 51 | 494 | 501 | KPGPNSSR | | | | | Mascot |
| 1062.5135 | 1062.6647 | 0.1512 | 142 | 130 | 138 | EDMEIAQVK | | | | | Mascot |
| 1118.6245 | 1118.604 | -0.0205 | -18 | 837 | 845 | TWLIYTPPK | | | | | Mascot |
| 1165.5453 | 1165.7177 | 0.1724 | 148 | 789 | 797 | LARMNEEMR | | | Oxidation (M)[4] | | Mascot |
| 1165.5453 | 1165.7177 | 0.1724 | 148 | 789 | 797 | LARMNEEMR | | | Oxidation (M)[4] | | Mascot |
| 1179.6368 | 1179.5767 | -0.0601 | -51 | 664 | 673 | ELDPNTLLHK | | | | | Mascot |
| 1182.5129 | 1182.5671 | 0.0542 | 46 | 340 | 349 | SCMLQAESEK | | | Carbamidomethyl (C)[2] | | Mascot |
| 1182.5129 | 1182.5671 | 0.0542 | 46 | 340 | 349 | SCMLQAESEK | | | Carbamidomethyl (C)[2] | | Mascot |
| 1279.5769 | 1279.7423 | 0.1654 | 129 | 521 | 532 | MCGSEALLEGGR | | | Carbamidomethyl (C)[2] | | Mascot |
| 1391.6658 | 1391.7091 | 0.0433 | 31 | 317 | 328 | LCSVKPCEDIER | | | | | Mascot |
| 1485.7809 | 1485.712 | -0.0689 | -46 | 105 | 116 | QQLHSFVKEDVR | | | | | Mascot |
| 1493.8071 | 1493.7106 | -0.0965 | -65 | 664 | 676 | ELDPNTLLHKASR | | | | | Mascot |
| 1638.8533 | 1638.8115 | -0.0418 | -26 | 56 | 69 | LFVNGIRDLSHHCK | | | | | Mascot |
| 1638.8533 | 1638.8115 | -0.0418 | -26 | 56 | 69 | LFVNGIRDLSHHCK | | | | | Mascot |
| 1755.8516 | 1755.9348 | 0.0832 | 47 | 39 | 55 | LCSGMIEAGRAYATANK | | | | | Mascot |
| 2284.1431 | 2284.1189 | -0.0242 | -11 | 259 | 278 | VEFNVDAPNGVVMEGYL FKR | | | | | Mascot |

| | | | | | | | |
|---|---|---|---|---|---|---|---|
| 38 | Zgc:153795 protein [Danio rerio] | gi\|92097819 | 42352.1 | 5.79 | 10 | 31 | 0 |

Peptide Information

| Calc. Mass | Obsrv. Mass | ± da | ± ppm | Start Seq. | End Seq. | Sequence | Ion Score | C. I. % | Modification | Rank | Result Type |
|---|---|---|---|---|---|---|---|---|---|---|---|
| 1233.535 | 1233.5854 | 0.0504 | 41 | 253 | 263 | CVVPSDSGPCR | | | Carbamidomethyl (C)[1,10] | | Mascot |
| 1233.535 | 1233.5854 | 0.0504 | 41 | 253 | 263 | CVVPSDSGPCR | | | Carbamidomethyl (C)[1,10] | | Mascot |
| 1277.71 | 1277.6538 | -0.0562 | -44 | 233 | 244 | EQEGPVIPAIPK | | | | | Mascot |
| 1632.8003 | 1632.6975 | -0.1028 | -63 | 362 | 375 | EELLPAEEPVSYEK | | | | | Mascot |
| 1637.7522 | 1637.7745 | 0.0223 | 14 | 253 | 267 | CVVPSDSGPCRASFR | | | Carbamidomethyl (C)[1] | | Mascot |
| 1638.692 | 1638.8115 | 0.1195 | 73 | 26 | 40 | TADGPQECNLVNCMK | | | Oxidation (M)[14] | | Mascot |
| 1638.692 | 1638.8115 | 0.1195 | 73 | 26 | 40 | TADGPQECNLVNCMK | | | Oxidation (M)[14] | | Mascot |
| 1648.7893 | 1648.6963 | -0.093 | -56 | 177 | 191 | CQSPAVTGNCRASIK | | | Carbamidomethyl (C)[1,10] | | Mascot |
| 1651.76 | 1651.7683 | 0.0083 | 5 | 11 | 25 | ACCEREDCQLALIGK | | | | | Mascot |
| 1682.6342 | 1682.7767 | 0.1425 | 85 | 293 | 306 | YGSEEECMSVCSTK | | | Carbamidomethyl (C)[7,11], Oxidation (M)[8] | | Mascot |

| Calc. Mass | Obsrv. Mass | ± da | ± ppm | Start Seq. | End Seq. | Sequence | Ion Score | C. I. % | Modification | Rank | Result Type |
|---|---|---|---|---|---|---|---|---|---|---|---|
| 2211.0122 | 2211.0518 | 0.0396 | 18 | 158 | 176 | NDVDPKALTEMTSEEFQEK | | | | | Mascot |
| 2220.9319 | 2221.103 | 0.1711 | 77 | 288 | 306 | GNLNRYGSEEECMSVCSTK | | | Carbamidomethyl (C)[12,16] | | Mascot |

| | | | | | | | | | | |
|---|---|---|---|---|---|---|---|---|---|---|
| 39 | PREDICTED: zinc finger protein 271-like [Danio rerio] | gi\|326666322 | 31744.7 | 9.68 | 10 | 31 | 0 | | | |

Peptide Information

| Calc. Mass | Obsrv. Mass | ± da | ± ppm | Start Seq. | End Seq. | Sequence | Ion Score | C. I. % | Modification | Rank | Result Type |
|---|---|---|---|---|---|---|---|---|---|---|---|
| 996.5359 | 996.572 | 0.0361 | 36 | 138 | 146 | SFTQVSSLK | | | | | Mascot |
| 996.5359 | 996.572 | 0.0361 | 36 | 138 | 146 | SFTQVSSLK | | | | | Mascot |
| 1062.5803 | 1062.6647 | 0.0844 | 79 | 259 | 267 | AHERIHTAK | | | | | Mascot |
| 1118.551 | 1118.604 | 0.053 | 47 | 7 | 16 | ISIMSHTGEK | | | Oxidation (M)[4] | | Mascot |
| 1182.4594 | 1182.5671 | 0.1077 | 91 | 157 | 164 | EYFCFECK | | | Carbamidomethyl (C)[4,7] | | Mascot |
| 1182.4594 | 1182.5671 | 0.1077 | 91 | 157 | 164 | EYFCFECK | | | Carbamidomethyl (C)[4,7] | | Mascot |
| 1190.6389 | 1190.6184 | -0.0205 | -17 | 147 | 156 | NHEKIHTGVR | | | | | Mascot |
| 1310.5543 | 1310.6559 | 0.1016 | 78 | 157 | 165 | EYFCFECKK | | | Carbamidomethyl (C)[4,7] | | Mascot |
| 1718.7626 | 1718.8324 | 0.0698 | 41 | 235 | 249 | IHTGEKPYTCSHCDK | | | | | Mascot |
| 2207.012 | 2207.0408 | 0.0288 | 13 | 207 | 224 | IHTGEKPYACSHCDKTFR | | | Carbamidomethyl (C)[10,13] | | Mascot |
| 2224.9976 | 2225.0647 | 0.0671 | 30 | 204 | 221 | HERIHTGEKPYACSHCDK | | | Carbamidomethyl (C)[13,16] | | Mascot |
| 2224.9976 | 2225.0647 | 0.0671 | 30 | 204 | 221 | HERIHTGEKPYACSHCDK | | | Carbamidomethyl (C)[13,16] | | Mascot |
| 2283.0757 | 2283.1248 | 0.0491 | 22 | 38 | 57 | RIHTGEKPFSCTQCGQSFTR | | | | | Mascot |
| 2283.0757 | 2283.1248 | 0.0491 | 22 | 38 | 57 | RIHTGEKPFSCTQCGQSFTR | | | | | Mascot |

| | | | | | | | | | | |
|---|---|---|---|---|---|---|---|---|---|---|
| 40 | ectodermal-neural cortex (with BTB-like domain) [Danio rerio] | gi\|47777321 | 31766.1 | 6.05 | 5 | 31 | 0 | 15 | 0 | |

Peptide Information

| Calc. Mass | Obsrv. Mass | ± da | ± ppm | Start Seq. | End Seq. | Sequence | Ion Score | C. I. % | Modification | Rank | Result Type |
|---|---|---|---|---|---|---|---|---|---|---|---|
| 864.421 | 864.4717 | 0.0507 | 59 | 254 | 260 | SLSWDTR | | | | | Mascot |
| 1104.6523 | 1104.5546 | -0.0977 | -88 | 43 | 51 | RVFTDVVLR | | | | | Mascot |
| 1104.6523 | 1104.5546 | -0.0977 | -88 | 43 | 51 | RVFTDVVLR | 15 | 0 | | | Mascot |
| 1120.6514 | 1120.5374 | -0.114 | -102 | 265 | 273 | LWKTQFVAK | | | | | Mascot |
| 1179.5952 | 1179.5767 | -0.0185 | -16 | 52 | 61 | AGHRVFPCHR | | | | | Mascot |
| 1637.7628 | 1637.7745 | 0.0117 | 7 | 70 | 83 | YFEAMFSGGLKESR | | | Oxidation (M)[5] | | Mascot |

| | | | | | | | | | | |
|---|---|---|---|---|---|---|---|---|---|---|
| 41 | PREDICTED: u2 small nuclear ribonucleoprotein auxiliary factor 35 kDa subunit-related protein 2 [Da | gi\|189525066 | 74762.6 | 9.52 | 12 | 31 | 0 | | | |

Peptide Information

| Calc. Mass | Obsrv. Mass | ± da | ± ppm | Start Seq. | End Seq. | Sequence | Ion Score | C. I. % | Modification | Rank | Result Type |
|---|---|---|---|---|---|---|---|---|---|---|---|
| 996.4971 | 996.572 | 0.0749 | 75 | 183 | 190 | ANCPFFLK | | | Carbamidomethyl (C)[3] | | Mascot |
| 996.4971 | 996.572 | 0.0749 | 75 | 183 | 190 | ANCPFFLK | | | Carbamidomethyl (C)[3] | | Mascot |
| 1104.5531 | 1104.5546 | 0.0015 | 1 | 76 | 84 | LKVDEESER | | | | | Mascot |
| 1104.5531 | 1104.5546 | 0.0015 | 1 | 76 | 84 | LKVDEESER | | | | | Mascot |
| 1182.5975 | 1182.5671 | -0.0304 | -26 | 181 | 190 | DKANCPFFLK | | | | | Mascot |
| 1182.5975 | 1182.5671 | -0.0304 | -26 | 181 | 190 | DKANCPFFLK | | | | | Mascot |
| 1391.6082 | 1391.7091 | 0.1009 | 73 | 217 | 228 | GMFVSFGMEQSR | | | Oxidation (M)[2] | | Mascot |
| 1413.6692 | 1413.7682 | 0.099 | 70 | 85 | 94 | LHQEWMERER | | | | | Mascot |
| 1463.6267 | 1463.7169 | 0.0902 | 62 | 373 | 384 | HMSGRDGMWSQR | | | Oxidation (M)[2] | | Mascot |
| 1469.7343 | 1469.7172 | -0.0171 | -12 | 500 | 513 | SNSPEHVSKNGSVK | | | | | Mascot |
| 1531.7144 | 1531.7145 | 0.0001 | 0 | 217 | 229 | GMFVSFGMEQSRR | | | | | Mascot |
| 1638.7839 | 1638.8115 | 0.0276 | 17 | 203 | 216 | KHDHPASSCTLMVR | | | Carbamidomethyl (C)[9] | | Mascot |
| 1638.7839 | 1638.8115 | 0.0276 | 17 | 203 | 216 | KHDHPASSCTLMVR | | | Carbamidomethyl (C)[9] | | Mascot |
| 1670.6744 | 1670.817 | 0.1426 | 85 | 5 | 19 | GCCSIQTETQDSENR | | | | | Mascot |
| 2232.9165 | 2233.03 | 0.1135 | 51 | 1 | 19 | MTEKGCCSIQTETQDSE NR | | | Carbamidomethyl (C)[6], Oxidation (M)[1] | | Mascot |
| 2872.3027 | 2872.3237 | 0.021 | 7 | 271 | 294 | VSCNFEPHLRGNVYVQY ETEEQCK | | | | | Mascot |

42 hypothetical protein LOC436894 [Danio rerio] gi|50540310 28008.1 8.18 8 31 0

**Peptide Information**

| Calc. Mass | Obsrv. Mass | ± da | ± ppm | Start Seq. | End Seq. | Sequence | Ion Score | C. I. % | Modification | Rank | Result Type |
|---|---|---|---|---|---|---|---|---|---|---|---|
| 864.4495 | 864.4717 | 0.0222 | 26 | 184 | 190 | LETMNLK | | | Oxidation (M)[4] | | Mascot |
| 996.5433 | 996.572 | 0.0287 | 29 | 114 | 121 | MLLELFSK | | | Oxidation (M)[1] | | Mascot |
| 996.5433 | 996.572 | 0.0287 | 29 | 114 | 121 | MLLELFSK | | | Oxidation (M)[1] | | Mascot |
| 1182.5836 | 1182.5671 | -0.0165 | -14 | 30 | 38 | FCPFAQRTR | | | Carbamidomethyl (C)[2] | | Mascot |
| 1182.5836 | 1182.5671 | -0.0165 | -14 | 30 | 38 | FCPFAQRTR | | | Carbamidomethyl (C)[2] | | Mascot |
| 1190.6099 | 1190.6184 | 0.0085 | 7 | 21 | 29 | DHIRLYSMR | | | | | Mascot |
| 1391.7893 | 1391.7091 | -0.0802 | -58 | 45 | 56 | GIKYDTININLK | | | | | Mascot |
| 1463.804 | 1463.7169 | -0.0871 | -60 | 110 | 121 | AQQRMLLELFSK | | | | | Mascot |
| 1616.8544 | 1616.7039 | -0.1505 | -93 | 101 | 113 | LLPFDPFERAQQR | | | | | Mascot |
| 1718.7804 | 1718.8324 | 0.052 | 30 | 214 | 227 | ATMFSTETYMVFYK | | | | | Mascot |

43 PREDICTED: tRNA-dihydrouridine synthase 2-like [Danio rerio] gi|292628574 56676.6 6.04 11 31 0

**Peptide Information**

| Calc. Mass | Obsrv. Mass | ± da | ± ppm | Start Seq. | End Seq. | Sequence | Ion Score | C. I. % | Modification | Rank | Result Type |
|---|---|---|---|---|---|---|---|---|---|---|---|
| 842.473 | 842.4905 | 0.0175 | 21 | 436 | 443 | TLGLPEGR | | | | | Mascot |
| 1045.4628 | 1045.5411 | 0.0783 | 75 | 288 | 295 | YCLCQMLR | | | Oxidation (M)[6] | | Mascot |
| 1051.6007 | 1051.6859 | 0.0852 | 81 | 180 | 190 | TGVAAIAVHGR | | | | | Mascot |
| 1062.5538 | 1062.6647 | 0.1109 | 104 | 328 | 336 | TTAELESRR | | | | | Mascot |
| 1182.6113 | 1182.5671 | -0.0442 | -37 | 444 | 454 | IGEEHSGLVNK | | | | | Mascot |
| 1182.6113 | 1182.5671 | -0.0442 | -37 | 444 | 454 | IGEEHSGLVNK | | | | | Mascot |
| 1277.6041 | 1277.6538 | 0.0497 | 39 | 129 | 141 | GGMGSALLSDPEK | | | Oxidation (M)[3] | | Mascot |
| 1310.7361 | 1310.6559 | -0.0802 | -61 | 180 | 192 | TGVAAIAVHGRMK | | | | | Mascot |
| 1391.6802 | 1391.7091 | 0.0289 | 21 | 386 | 396 | LEQPVYETEQR | | | | | Mascot |
| 1648.8176 | 1648.6963 | -0.1213 | -74 | 384 | 396 | EKLEQPVYETEQR | | | | | Mascot |
| 1959.9658 | 1960.0217 | 0.0559 | 29 | 60 | 76 | VENDVLQTIDFVAPDER | | | | | Mascot |
| 2299.1387 | 2299.1296 | -0.0091 | -4 | 259 | 278 | QQGALSVDQVMQEYITY AVR | | | | | Mascot |

| | | | | | | | | |
|---|---|---|---|---|---|---|---|---|
| 44 | conserved oligomeric Golgi complex subunit 2 [Danio rerio] | gi\|47087011 | 82297.4 | 6.05 | 12 | 31 | 0 | |

Peptide Information

| Calc. Mass | Obsrv. Mass | ± da | ± ppm | Start Seq. | End Seq. | Sequence | Ion Score | C. I. % | Modification | Rank | Result Type |
|---|---|---|---|---|---|---|---|---|---|---|---|
| 864.4495 | 864.4717 | 0.0222 | 26 | 649 | 655 | KMEESLK | | | | | Mascot |
| 1045.5598 | 1045.5411 | -0.0187 | -18 | 517 | 525 | IPDISEMIK | | | | | Mascot |
| 1120.5527 | 1120.5374 | -0.0153 | -14 | 352 | 361 | QCGSQASVKR | | | Carbamidomethyl (C)[2] | | Mascot |
| 1310.6772 | 1310.6559 | -0.0213 | -16 | 262 | 273 | SSPNGLKVMYAK | | | Oxidation (M)[9] | | Mascot |
| 1335.7208 | 1335.6929 | -0.0279 | -21 | 375 | 384 | WNLPVYFQLR | | | | | Mascot |
| 1469.7634 | 1469.7172 | -0.0462 | -31 | 43 | 53 | EDLEQYYRLLK | | | | | Mascot |
| 1632.9155 | 1632.6975 | -0.218 | -134 | 274 | 286 | LLEFVPHHCRLLR | | | | | Mascot |
| 1638.7866 | 1638.8115 | 0.0249 | 15 | 2 | 15 | MTLPKAPDSLCFDK | | | Carbamidomethyl (C)[11], Oxidation (M)[1] | | Mascot |
| 1638.7866 | 1638.8115 | 0.0249 | 15 | 2 | 15 | MTLPKAPDSLCFDK | | | Carbamidomethyl (C)[11], Oxidation (M)[1] | | Mascot |
| 1718.74 | 1718.8324 | 0.0924 | 54 | 7 | 20 | APDSLCFDKDEFMK | | | Carbamidomethyl (C)[6], Oxidation (M)[13] | | Mascot |
| 1755.8635 | 1755.9348 | 0.0713 | 41 | 424 | 437 | CWTDQVYLPPLAHR | | | Carbamidomethyl (C)[1] | | Mascot |
| 2230.1284 | 2230.1079 | -0.0205 | -9 | 594 | 613 | ASAYMDNALRPLHQLVT DSK | | | | | Mascot |
| 2584.3188 | 2584.1545 | -0.1643 | -64 | 614 | 635 | NVVKDSIIQEWLQVTLSD CTHR | | | | | Mascot |

| | | | | | | | | |
|---|---|---|---|---|---|---|---|---|
| 45 | protein-associating with the carboxyl-terminal domain of ezrin [Danio rerio] | gi\|41054469 | 85270.2 | 5.3 | 11 | 30 | 0 | |

Peptide Information

| Calc. Mass | Obsrv. Mass | ± da | ± ppm | Start Seq. | End Seq. | Sequence | Ion Score | C. I. % | Modification | Rank | Result Type |
|---|---|---|---|---|---|---|---|---|---|---|---|
| 882.4427 | 882.5462 | 0.1035 | 117 | 349 | 355 | VNEEHVR | | | | | Mascot |
| 1045.5823 | 1045.5411 | -0.0412 | -39 | 622 | 630 | ALRLNPSMK | | | Oxidation (M)[8] | | Mascot |
| 1165.6622 | 1165.7177 | 0.0555 | 48 | 59 | 67 | TLRHPCLLR | | | Carbamidomethyl (C)[6] | | Mascot |
| 1165.6622 | 1165.7177 | 0.0555 | 48 | 59 | 67 | TLRHPCLLR | | | Carbamidomethyl (C)[6] | | Mascot |
| 1179.6885 | 1179.5767 | -0.1118 | -95 | 308 | 317 | SFLPHLLQPK | | | | | Mascot |
| 1190.6263 | 1190.6184 | -0.0079 | -7 | 258 | 267 | SLTLKNEEEK | | | | | Mascot |
| 1328.6997 | 1328.5773 | -0.1224 | -92 | 268 | 277 | NEFFKFLLDR | | | | | Mascot |
| 1531.8552 | 1531.7145 | -0.1407 | -92 | 294 | 307 | LLNSLVFAEPMAVK | | | | | Mascot |
| 1797.9705 | 1797.9182 | -0.0523 | -29 | 604 | 621 | STALSLSEPSAGPLRPSK | | | | | Mascot |
| 2283.0889 | 2283.1248 | 0.0359 | 16 | 688 | 706 | KFEPDPELDFFADMIPDIK | | | Oxidation (M)[14] | | Mascot |
| 2283.0889 | 2283.1248 | 0.0359 | 16 | 688 | 706 | KFEPDPELDFFADMIPDIK | | | Oxidation (M)[14] | | Mascot |
| 2297.1189 | 2297.0979 | -0.021 | -9 | 318 | 337 | QDSSVNAGEECLLSLSLY RR | | | Carbamidomethyl (C)[11] | | Mascot |
| 2872.1187 | 2872.3237 | 0.205 | 71 | 568 | 592 | EDDGEEPWDDFEDSEVT SDQSPSTR | | | | | Mascot |

46 hypothetical protein LOC562009 [Danio rerio] gi|116235420 47967.8 5.46 10 30 0

Peptide Information

| Calc. Mass | Obsrv. Mass | ± da | ± ppm | Start Seq. | End Seq. | Sequence | Ion Score | C. I. % | Modification | Rank | Result Type |
|---|---|---|---|---|---|---|---|---|---|---|---|
| 1233.535 | 1233.5854 | 0.0504 | 41 | 304 | 314 | CVVPSDSGPCR | | | Carbamidomethyl (C)[1,10] | | Mascot |
| 1233.535 | 1233.5854 | 0.0504 | 41 | 304 | 314 | CVVPSDSGPCR | | | Carbamidomethyl (C)[1,10] | | Mascot |
| 1277.71 | 1277.6538 | -0.0562 | -44 | 284 | 295 | EQEGPVIPAIPK | | | | | Mascot |
| 1632.8003 | 1632.6975 | -0.1028 | -63 | 413 | 426 | EELLPAEEPVSYEK | | | | | Mascot |
| 1637.7522 | 1637.7745 | 0.0223 | 14 | 304 | 318 | CVVPSDSGPCRASFR | | | Carbamidomethyl (C)[1] | | Mascot |
| 1638.692 | 1638.8115 | 0.1195 | 73 | 77 | 91 | TADGPQECNLVNCMK | | | Oxidation (M)[14] | | Mascot |
| 1638.692 | 1638.8115 | 0.1195 | 73 | 77 | 91 | TADGPQECNLVNCMK | | | Oxidation (M)[14] | | Mascot |
| 1648.7893 | 1648.6963 | -0.093 | -56 | 228 | 242 | CQSPAVTGNCRASIK | | | Carbamidomethyl (C)[1,10] | | Mascot |
| 1651.76 | 1651.7683 | 0.0083 | 5 | 62 | 76 | ACCEREDCQLALIGK | | | | | Mascot |
| 1682.6342 | 1682.7767 | 0.1425 | 85 | 344 | 357 | YGSEEECMSVCSTK | | | Carbamidomethyl (C)[7,11], Oxidation (M)[8] | | Mascot |
| 2211.0122 | 2211.0518 | 0.0396 | 18 | 209 | 227 | NDVDPKALTEMTSEEFQ EK | | | | | Mascot |
| 2220.9319 | 2221.103 | 0.1711 | 77 | 339 | 357 | GNLNRYGSEEECMSVCS TK | | | Carbamidomethyl (C)[12,16] | | Mascot |

47 tropomyosin alpha-4 chain isoform 2 [Danio rerio] gi|47085929 28484.4 4.63 8 30 0

Peptide Information

| Calc. Mass | Obsrv. Mass | ± da | ± ppm | Start Seq. | End Seq. | Sequence | Ion Score | C. I. % | Modification | Rank | Result Type |
|---|---|---|---|---|---|---|---|---|---|---|---|

| Calc. Mass | Obsrv. Mass | ± da | ± ppm | Start Seq. | End Seq. | Sequence | Ion Score | C. I. % | Modification | Rank | Result Type |
|---|---|---|---|---|---|---|---|---|---|---|---|
| 850.3975 | 850.5237 | 0.1262 | 148 | 98 | 104 | AMKDEEK | | | | | Mascot |
| 850.3975 | 850.5237 | 0.1262 | 148 | 98 | 104 | AMKDEEK | | | | | Mascot |
| 1165.5592 | 1165.7177 | 0.1585 | 136 | 105 | 113 | MEIQEMQLK | | | Oxidation (M)[1] | | Mascot |
| 1165.5592 | 1165.7177 | 0.1585 | 136 | 105 | 113 | MEIQEMQLK | | | Oxidation (M)[1] | | Mascot |
| 1182.5525 | 1182.5671 | 0.0146 | 12 | 182 | 190 | EDKYEEEIK | | | | | Mascot |
| 1182.5525 | 1182.5671 | 0.0146 | 12 | 182 | 190 | EDKYEEEIK | | | | | Mascot |
| 1277.6882 | 1277.6538 | -0.0344 | -27 | 1 | 12 | MTGVTSLDAVKR | | | | | Mascot |
| 1413.7445 | 1413.7682 | 0.0237 | 17 | 28 | 39 | ALGLQRELDNER | | | | | Mascot |
| 1493.7338 | 1493.7106 | -0.0232 | -16 | 105 | 116 | MEIQEMQLKEAK | | | Oxidation (M)[1] | | Mascot |
| 1632.7283 | 1632.6975 | -0.0308 | -19 | 14 | 27 | IQCLQQQADDAEDR | | | | | Mascot |
| 1682.7611 | 1682.7767 | 0.0156 | 9 | 101 | 113 | DEEKMEIQEMQLK | | | Oxidation (M)[5,10] | | Mascot |

48 novel protein similar to human titin (TTN) [Danio rerio] gi|29561775 2113153 6.46 59 30 0

**Peptide Information**

| Calc. Mass | Obsrv. Mass | ± da | ± ppm | Start Seq. | End Seq. | Sequence | Ion Score | C. I. % | Modification | Rank | Result Type |
|---|---|---|---|---|---|---|---|---|---|---|---|
| 842.5094 | 842.4905 | -0.0189 | -22 | 1522 | 1529 | LSIPGVTR | | | | | Mascot |
| 850.5396 | 850.5237 | -0.0159 | -19 | 4200 | 4206 | FLLTKTK | | | | | Mascot |
| 850.5396 | 850.5237 | -0.0159 | -19 | 4200 | 4206 | FLLTKTK | | | | | Mascot |
| 864.4937 | 864.4717 | -0.022 | -25 | 6550 | 6556 | GYIVERK | | | | | Mascot |
| 882.4315 | 882.5462 | 0.1147 | 130 | 12144 | 12151 | DGSSFTLR | | | | | Mascot |
| 996.5373 | 996.572 | 0.0347 | 35 | 670 | 676 | QYQFRVR | | | | | Mascot |
| 996.5373 | 996.572 | 0.0347 | 35 | 670 | 676 | QYQFRVR | | | | | Mascot |
| 1042.5204 | 1042.557 | 0.0366 | 35 | 13471 | 13478 | ELSEVYFR | | | | | Mascot |
| 1045.6404 | 1045.5411 | -0.0993 | -95 | 2435 | 2444 | GTKFVVPGLK | | | | | Mascot |
| 1051.6259 | 1051.6859 | 0.06 | 57 | 18684 | 18693 | SPIASPPRVK | | | | | Mascot |
| 1062.6306 | 1062.6647 | 0.0341 | 32 | 14911 | 14919 | IVGYVVERK | | | | | Mascot |
| 1104.5684 | 1104.5546 | -0.0138 | -12 | 148 | 157 | GWQAVDTTVK | | | | | Mascot |
| 1104.5684 | 1104.5546 | -0.0138 | -12 | 148 | 157 | GWQAVDTTVK | | | | | Mascot |
| 1118.6205 | 1118.604 | -0.0165 | -15 | 10489 | 10497 | EKNSILWTK | | | | | Mascot |
| 1120.6361 | 1120.5374 | -0.0987 | -88 | 2970 | 2980 | VPDAPQAPVVK | | | | | Mascot |
| 1165.6211 | 1165.7177 | 0.0966 | 83 | 14756 | 14766 | VSAENKFGVSK | | | | | Mascot |
| 1165.6211 | 1165.7177 | 0.0966 | 83 | 14756 | 14766 | VSAENKFGVSK | | | | | Mascot |
| 1179.6078 | 1179.5767 | -0.0311 | -26 | 14620 | 14628 | VTWKLEEMK | | | Oxidation (M)[8] | | Mascot |
| 1182.6187 | 1182.5671 | -0.0516 | -44 | 14856 | 14866 | VYAMNIAGLSK | | | Oxidation (M)[4] | | Mascot |
| 1182.6187 | 1182.5671 | -0.0516 | -44 | 14856 | 14866 | VYAMNIAGLSK | | | Oxidation (M)[4] | | Mascot |

| | | | | | | | | | |
|---|---|---|---|---|---|---|---|---|---|
| 1190.6263 | 1190.6184 | -0.0079 | -7 | 16255 | 16265 | SLKSDESVTPK | | | Mascot |
| 1221.6626 | 1221.5538 | -0.1088 | -89 | 10810 | 10819 | LLPGNEYIFR | | | Mascot |
| 1233.662 | 1233.5854 | -0.0766 | -62 | 18875 | 18885 | IQGGSEMVIKR | | Oxidation (M)[7] | Mascot |
| 1233.7201 | 1233.5854 | -0.1347 | -109 | 9620 | 9630 | NTDFKAVLVVK | | | Mascot |
| 1277.7688 | 1277.6538 | -0.115 | -90 | 2490 | 2501 | EKIVVHAGGVIR | | | Mascot |
| 1279.7256 | 1279.7423 | 0.0167 | 13 | 12882 | 12892 | LNTGTYELKIK | | | Mascot |
| 1310.6587 | 1310.6559 | -0.0028 | -2 | 3808 | 3818 | VKGLTEDTEYR | | | Mascot |
| 1335.6208 | 1335.6929 | 0.0721 | 54 | 4476 | 4486 | DKVEMEGEQVR | | Oxidation (M)[5] | Mascot |
| 1391.6624 | 1391.7091 | 0.0467 | 34 | 8975 | 8986 | VLDTPDMPANFR | | Oxidation (M)[7] | Mascot |
| 1413.7737 | 1413.7682 | -0.0055 | -4 | 17756 | 17768 | VSGTPAPTLKWEK | | | Mascot |
| 1443.7446 | 1443.6772 | -0.0674 | -47 | 3189 | 3202 | AMAGTHINIMAGIK | | Oxidation (M)[2] | Mascot |
| 1457.6147 | 1457.7173 | 0.1026 | 70 | 3989 | 4000 | TWAMISSDCTNR | | Carbamidomethyl (C)[9], Oxidation (M)[4] | Mascot |
| 1463.7264 | 1463.7169 | -0.0095 | -6 | 14293 | 14305 | LTSPEIDIDADFK | | | Mascot |
| 1469.8032 | 1469.7172 | -0.086 | -59 | 15675 | 15687 | EMPKIDLTGIPQK | | | Mascot |
| 1475.7601 | 1475.7218 | -0.0383 | -26 | 16447 | 16459 | VFAENETGLSRPR | | | Mascot |
| 1485.7155 | 1485.712 | -0.0035 | -2 | 5792 | 5804 | ITNLEAGQAYCFR | | | Mascot |
| 1493.7166 | 1493.7106 | -0.006 | -4 | 14434 | 14445 | LSWQEVSERCTR | | | Mascot |
| 1531.8115 | 1531.7145 | -0.097 | -63 | 10787 | 10799 | ETSRLSWTVVEPK | | | Mascot |
| 1579.8148 | 1579.7042 | -0.1106 | -70 | 12951 | 12963 | ETSRIMWSIISEK | | | Mascot |
| 1616.8101 | 1616.7039 | -0.1062 | -66 | 8743 | 8755 | VTGLLEMRSYEFR | | Oxidation (M)[7] | Mascot |
| 1626.7693 | 1626.7155 | -0.0538 | -33 | 7694 | 7708 | ACDTIFQPGPPGNPR | | Carbamidomethyl (C)[2] | Mascot |
| 1632.7687 | 1632.6975 | -0.0712 | -44 | 3309 | 3323 | DAASPQWVPICSSSK | | Carbamidomethyl (C)[11] | Mascot |
| 1637.7628 | 1637.7745 | 0.0117 | 7 | 14749 | 14761 | YMEYTFRVSAENK | | | Mascot |
| 1651.8174 | 1651.7683 | -0.0491 | -30 | 12107 | 12122 | IGPSEPSPETDPQVAK | | | Mascot |
| 1670.7955 | 1670.817 | 0.0215 | 13 | 5447 | 5461 | DHMTVTWKAPANDGK | | | Mascot |
| 1682.8265 | 1682.7767 | -0.0498 | -30 | 3222 | 3236 | AEIETSGTTTKLEMR | | Oxidation (M)[14] | Mascot |
| 1718.9006 | 1718.8324 | -0.0682 | -40 | 2542 | 2556 | SCLRQHQGIYTLTAK | | | Mascot |
| 1755.8582 | 1755.9348 | 0.0766 | 44 | 3404 | 3419 | TVSIPECHVTGLEEGK | | Carbamidomethyl (C)[7] | Mascot |
| 1797.9381 | 1797.9182 | -0.0199 | -11 | 8993 | 9009 | NSVTLTWEPPLLDGGAK | | | Mascot |
| 1853.8309 | 1853.8535 | 0.0226 | 12 | 5030 | 5045 | HPFDPPSEPLNCCVNK | | Carbamidomethyl (C)[12] | Mascot |
| 1959.952 | 1960.0217 | 0.0697 | 36 | 3757 | 3772 | DYSCTYADLVWIKPTK | | Carbamidomethyl (C)[4] | Mascot |
| 2192.1155 | 2192.1182 | 0.0027 | 1 | 7970 | 7989 | ILAENEYGVGLPVETMEA IK | | Oxidation (M)[16] | Mascot |
| 2192.1155 | 2192.1182 | 0.0027 | 1 | 7970 | 7989 | ILAENEYGVGLPVETMEA IK | | Oxidation (M)[16] | Mascot |
| 2207.0801 | 2207.0408 | -0.0393 | -18 | 12771 | 12789 | FTVSGLTQNESYEFRVM AK | | | Mascot |
| 2211.0713 | 2211.0518 | -0.0195 | -9 | 16266 | 16285 | TPLCPPEPPSFPPEIMDV TK | | Oxidation (M)[16] | Mascot |

| Calc. Mass | Obsrv. Mass | ± da | ± ppm | Start Seq. | End Seq. | Sequence | Ion Score | C. I. % | Modification | Rank | Result Type |
|---|---|---|---|---|---|---|---|---|---|---|---|
| 2221.1248 | 2221.103 | -0.0218 | -10 | 16166 | 16185 | VIGRPDPPEGPLEFDDIQAR | | | | | Mascot |
| 2225.0479 | 2225.0647 | 0.0168 | 8 | 5030 | 5048 | HPFDPPSEPLNCCVNKVSK | | | Carbamidomethyl (C)[12,13] | | Mascot |
| 2225.0479 | 2225.0647 | 0.0168 | 8 | 5030 | 5048 | HPFDPPSEPLNCCVNKVSK | | | Carbamidomethyl (C)[12,13] | | Mascot |
| 2230.1106 | 2230.1079 | -0.0027 | -1 | 16047 | 16068 | FRVMACNAGGAGEPAEIPGVVK | | | Carbamidomethyl (C)[6] | | Mascot |
| 2283.1284 | 2283.1248 | -0.0036 | -2 | 76 | 97 | VRAVNVAGESEPSEPSDPVLCK | | | | | Mascot |
| 2283.1284 | 2283.1248 | -0.0036 | -2 | 76 | 97 | VRAVNVAGESEPSEPSDPVLCK | | | | | Mascot |
| 2284.1389 | 2284.1189 | -0.02 | -9 | 7694 | 7714 | ACDTIFQPGPPGNPRVLDTTK | | | Carbamidomethyl (C)[2] | | Mascot |
| 2299.1233 | 2299.1296 | 0.0063 | 3 | 16346 | 16367 | VVAQNDIGQSEPGPVSESVVCK | | | Carbamidomethyl (C)[21] | | Mascot |
| 2306.0732 | 2306.1018 | 0.0286 | 12 | 4099 | 4116 | ETFFMVDKCVENHIYQFR | | | | | Mascot |
| 2501.3545 | 2501.1995 | -0.155 | -62 | 18126 | 18146 | SLSPTYIELMRPVSELIRQPR | | | Oxidation (M)[10] | | Mascot |
| 2584.2922 | 2584.1545 | -0.1377 | -53 | 12702 | 12725 | SSDPVLAIDACEPPNSVRVTDITK | | | Carbamidomethyl (C)[11] | | Mascot |
| 2872.4258 | 2872.3237 | -0.1021 | -36 | 14492 | 14517 | RLDVIDTTSTTASLVWMKPEHDGGSR | | | | | Mascot |

| | | | | | | | | |
|---|---|---|---|---|---|---|---|---|
| 49 | PREDICTED: sterol 26-hydroxylase, mitochondrial [Danio rerio] | gi\|326670761 | 63520.1 | 8.95 | 9 | 30 | 0 | |

**Peptide Information**

| Calc. Mass | Obsrv. Mass | ± da | ± ppm | Start Seq. | End Seq. | Sequence | Ion Score | C. I. % | Modification | Rank | Result Type |
|---|---|---|---|---|---|---|---|---|---|---|---|
| 864.4243 | 864.4717 | 0.0474 | 55 | 318 | 324 | MEAIQTR | | | Oxidation (M)[1] | | Mascot |
| 1051.5969 | 1051.6859 | 0.089 | 85 | 309 | 317 | FGTKMINLK | | | | | Mascot |
| 1104.5466 | 1104.5546 | 0.008 | 7 | 1 | 9 | MNESVIQQR | | | | | Mascot |
| 1104.5466 | 1104.5546 | 0.008 | 7 | 1 | 9 | MNESVIQQR | | | | | Mascot |
| 1120.5415 | 1120.5374 | -0.0041 | -4 | 1 | 9 | MNESVIQQR | | | Oxidation (M)[1] | | Mascot |
| 1413.6532 | 1413.7682 | 0.115 | 81 | 169 | 181 | GISYGPFTEEGEK | | | | | Mascot |
| 1463.7709 | 1463.7169 | -0.054 | -37 | 313 | 324 | MINLKMEAIQTR | | | Oxidation (M)[1] | | Mascot |
| 1670.8418 | 1670.817 | -0.0248 | -15 | 31 | 46 | SVSIMAVSFALSSAER | | | Oxidation (M)[5] | | Mascot |
| 1797.8654 | 1797.9182 | 0.0528 | 29 | 166 | 181 | DLRGISYGPFTEEGEK | | | | | Mascot |
| 1853.9479 | 1853.8535 | -0.0944 | -51 | 47 | 62 | LGWCFLRPTTAATGFR | | | Carbamidomethyl (C)[4] | | Mascot |
| 2283.2053 | 2283.1248 | -0.0805 | -35 | 240 | 259 | FSLEGIASILFETRIGCLEK | | | Carbamidomethyl (C)[17] | | Mascot |
| 2283.2053 | 2283.1248 | -0.0805 | -35 | 240 | 259 | FSLEGIASILFETRIGCLEK | | | Carbamidomethyl (C)[17] | | Mascot |

| | | | | | | | | |
|---|---|---|---|---|---|---|---|---|
| 50 | PREDICTED: sacsin [Danio rerio] | gi\|326670429 | 254713.7 | 7.13 | 1 | | 20 | 0 |

**Peptide Information**

| Calc. Mass | Obsrv. Mass | ± da | ± ppm | Start Seq. | End Seq. | Sequence | Ion Score | C. I. % | Modification | Rank | Result Type |
|---|---|---|---|---|---|---|---|---|---|---|---|

| | | | | | | | | | | | |
|---|---|---|---|---|---|---|---|---|---|---|---|
| 1104.6234 | 1104.5546 | -0.0688 | -62 | 306 | 314 | WLVTTCLLR | 20 | 0 | | 2 | Mascot |

| Gel Idx/Pos | 182/H9 | Instr./Gel Origin | BA2060/110707A | Process Status | Analysis Succeeded |
|---|---|---|---|---|---|
| Plate [#] Name | [1] 31496 | Instrument Sample Name | | Spectra | 11 |

| Rank | Protein Name | Accession No. | Protein MW | Protein PI | Pep. Count | Protein Score | Protein Score C. I. % | Total Ion Score | Total Ion C. I. % |
|---|---|---|---|---|---|---|---|---|---|
| 1 | protein disulfide-isomerase A4 [Danio rerio] | gi\|41054259 | 72669.4 | 4.99 | 24 | 603 | 100 | 441 | 100 |

Peptide Information

| Calc. Mass | Obsrv. Mass | ± da | ± ppm | Start Seq. | End Seq. | Sequence | Ion Score | C. I. % | Modification | Rank | Result Type |
|---|---|---|---|---|---|---|---|---|---|---|---|
| 829.4276 | 829.5081 | 0.0805 | 97 | 500 | 506 | SFVMAFK | | | | | Mascot |
| 882.577 | 882.5466 | -0.0304 | -34 | 525 | 533 | GPVKVVVGK | | | | | Mascot |
| 885.5152 | 885.4941 | -0.0211 | -24 | 295 | 301 | QVQELLR | | | | | Mascot |
| 907.4784 | 907.4562 | -0.0222 | -24 | 429 | 435 | VATQFWR | 49 | 99.638 | | | Mascot |
| 907.4784 | 907.4562 | -0.0222 | -24 | 429 | 435 | VATQFWR | | | | | Mascot |
| 939.4318 | 939.4149 | -0.0169 | -18 | 263 | 270 | AFDYNGPR | | | | | Mascot |
| 939.4318 | 939.4149 | -0.0169 | -18 | 263 | 270 | AFDYNGPR | 49 | 99.691 | | | Mascot |
| 940.4846 | 940.4044 | -0.0802 | -85 | 156 | 163 | SEHAIVER | | | | | Mascot |
| 1011.4781 | 1011.454 | -0.0241 | -24 | 96 | 103 | QFAPEYEK | | | | | Mascot |
| 1041.6316 | 1041.6078 | -0.0238 | -23 | 392 | 400 | HILPLVGHR | | | | | Mascot |
| 1068.5724 | 1068.5441 | -0.0283 | -26 | 247 | 256 | FGVSGYPTLK | | | | | Mascot |
| 1140.5935 | 1140.5654 | -0.0281 | -25 | 132 | 141 | FEVSGYPTIK | | | | | Mascot |
| 1248.6067 | 1248.5758 | -0.0309 | -25 | 235 | 246 | VDATAESDLATR | | | | | Mascot |
| 1470.6747 | 1470.6367 | -0.038 | -26 | 378 | 390 | DSTPASEVQDFFK | | | | | Mascot |
| 1598.7697 | 1598.7292 | -0.0405 | -25 | 378 | 391 | DSTPASEVQDFFKK | | | | | Mascot |
| 1683.8376 | 1683.7931 | -0.0445 | -26 | 597 | 611 | VEGFPTIYFAPSNNK | | | | | Mascot |
| 1683.8376 | 1683.7931 | -0.0445 | -26 | 597 | 611 | VEGFPTIYFAPSNNK | 71 | 99.998 | | | Mascot |
| 1700.7472 | 1700.7043 | -0.0429 | -25 | 486 | 499 | YAMEPEEFDSDVLR | | | | | Mascot |
| 1700.7472 | 1700.7043 | -0.0429 | -25 | 486 | 499 | YAMEPEEFDSDVLR | 119 | 100 | | | Mascot |
| 1711.7996 | 1711.7498 | -0.0498 | -29 | 273 | 288 | FGIVDYMSDQAGPPSK | | | | | Mascot |
| 1716.7421 | 1716.6989 | -0.0432 | -25 | 486 | 499 | YAMEPEEFDSDVLR | | | Oxidation (M)[3] | | Mascot |
| 1716.7421 | 1716.6989 | -0.0432 | -25 | 486 | 499 | YAMEPEEFDSDVLR | 69 | 99.997 | Oxidation (M)[3] | | Mascot |
| 1727.7944 | 1727.7474 | -0.047 | -27 | 273 | 288 | FGIVDYMSDQAGPPSK | | | Oxidation (M)[7] | | Mascot |
| 1828.8422 | 1828.7953 | -0.0469 | -26 | 485 | 499 | KYAMEPEEFDSDVLR | | | | | Mascot |
| 1844.837 | 1844.792 | -0.045 | -24 | 485 | 499 | KYAMEPEEFDSDVLR | | | Oxidation (M)[4] | | Mascot |
| 2017.0083 | 2016.9615 | -0.0468 | -23 | 464 | 484 | SLGLSESGEEVNVGIVGE | | | | | Mascot |

| Calc. Mass | Obsrv. Mass | ± da | ± ppm | Start Seq. | End Seq. | Sequence | Ion Score | C. I. % | Modification | Rank | Result Type |
|---|---|---|---|---|---|---|---|---|---|---|---|
| | | | | | | GGK | | | | | |
| 2119.1433 | 2119.0906 | -0.0527 | -25 | 166 | 184 | EVAQPDWKPPPEATLVLTK | | | | | Mascot |
| 2145.1033 | 2145.0481 | -0.0552 | -26 | 464 | 485 | SLGLSESGEEVNVGIVGEGGKK | | | | | Mascot |
| 2211.0815 | 2211.0491 | -0.0324 | -15 | 60 | 79 | EENGVLVLTDANFDTFIEGK | | | | | Mascot |
| 2346.3066 | 2346.2458 | -0.0608 | -26 | 164 | 184 | VKEVAQPDWKPPPEATLVLTK | | | | | Mascot |
| 2381.0344 | 2380.978 | -0.0564 | -24 | 444 | 463 | DFPEYTFAIADEEDYADELK | 153 | 100 | | | Mascot |
| 2381.0344 | 2380.978 | -0.0564 | -24 | 444 | 463 | DFPEYTFAIADEEDYADELK | | | | | Mascot |
| 3308.4536 | 3308.3765 | -0.0771 | -23 | 302 | 331 | DGDDAVIVGVFSSDEDAAYEIYQEACNSLR | | | Carbamidomethyl (C)[26] | | Mascot |

2 Protein disulfide isomerase associated 4 [Danio rerio] gi|39645929 72766.5 4.99 24 601 100 441 100

**Peptide Information**

| Calc. Mass | Obsrv. Mass | ± da | ± ppm | Start Seq. | End Seq. | Sequence | Ion Score | C. I. % | Modification | Rank | Result Type |
|---|---|---|---|---|---|---|---|---|---|---|---|
| 829.4276 | 829.5081 | 0.0805 | 97 | 500 | 506 | SFVMAFK | | | | | Mascot |
| 882.577 | 882.5466 | -0.0304 | -34 | 525 | 533 | GPVKVVVGK | | | | | Mascot |
| 885.5152 | 885.4941 | -0.0211 | -24 | 295 | 301 | QVQELLR | | | | | Mascot |
| 907.4784 | 907.4562 | -0.0222 | -24 | 429 | 435 | VATQFWR | 49 | 99.638 | | | Mascot |
| 907.4784 | 907.4562 | -0.0222 | -24 | 429 | 435 | VATQFWR | | | | | Mascot |
| 939.4318 | 939.4149 | -0.0169 | -18 | 263 | 270 | AFDYNGPR | | | | | Mascot |
| 939.4318 | 939.4149 | -0.0169 | -18 | 263 | 270 | AFDYNGPR | 49 | 99.691 | | | Mascot |
| 940.4846 | 940.4044 | -0.0802 | -85 | 156 | 163 | SEHAIVER | | | | | Mascot |
| 1011.4781 | 1011.454 | -0.0241 | -24 | 96 | 103 | QFAPEYEK | | | | | Mascot |
| 1041.6316 | 1041.6078 | -0.0238 | -23 | 392 | 400 | HILPLVGHR | | | | | Mascot |
| 1068.5724 | 1068.5441 | -0.0283 | -26 | 247 | 256 | FGVSGYPTLK | | | | | Mascot |
| 1140.5935 | 1140.5654 | -0.0281 | -25 | 132 | 141 | FEVSGYPTIK | | | | | Mascot |
| 1248.6067 | 1248.5758 | -0.0309 | -25 | 235 | 246 | VDATAESDLATR | | | | | Mascot |
| 1470.6747 | 1470.6367 | -0.038 | -26 | 378 | 390 | DSTPASEVQDFFK | | | | | Mascot |
| 1598.7697 | 1598.7292 | -0.0405 | -25 | 378 | 391 | DSTPASEVQDFFKK | | | | | Mascot |
| 1683.8376 | 1683.7931 | -0.0445 | -26 | 597 | 611 | VEGFPTIYFAPSNNK | | | | | Mascot |
| 1683.8376 | 1683.7931 | -0.0445 | -26 | 597 | 611 | VEGFPTIYFAPSNNK | 71 | 99.998 | | | Mascot |
| 1700.7472 | 1700.7043 | -0.0429 | -25 | 486 | 499 | YAMEPEEFDSDVLR | | | | | Mascot |
| 1700.7472 | 1700.7043 | -0.0429 | -25 | 486 | 499 | YAMEPEEFDSDVLR | 119 | 100 | | | Mascot |
| 1711.7996 | 1711.7498 | -0.0498 | -29 | 273 | 288 | FGIVDYMSDQAGPPSK | | | | | Mascot |
| 1716.7421 | 1716.6989 | -0.0432 | -25 | 486 | 499 | YAMEPEEFDSDVLR | | | Oxidation (M)[3] | | Mascot |
| 1716.7421 | 1716.6989 | -0.0432 | -25 | 486 | 499 | YAMEPEEFDSDVLR | 69 | 99.997 | Oxidation (M)[3] | | Mascot |
| 1727.7944 | 1727.7474 | -0.047 | -27 | 273 | 288 | FGIVDYMSDQAGPPSK | | | Oxidation (M)[7] | | Mascot |

| | | | | | | | | | | | |
|---|---|---|---|---|---|---|---|---|---|---|---|
| 1828.8422 | 1828.7953 | -0.0469 | -26 | 485 | 499 | KYAMEPEEFDSDVLR | | | | | Mascot |
| 1844.837 | 1844.792 | -0.045 | -24 | 485 | 499 | KYAMEPEEFDSDVLR | | | Oxidation (M)[4] | | Mascot |
| 2017.0083 | 2016.9615 | -0.0468 | -23 | 464 | 484 | SLGLSESGEEVNVGIVGE GGK | | | | | Mascot |
| 2119.1433 | 2119.0906 | -0.0527 | -25 | 166 | 184 | EVAQPDWKPPPEATLVL TK | | | | | Mascot |
| 2145.1033 | 2145.0481 | -0.0552 | -26 | 464 | 485 | SLGLSESGEEVNVGIVGE GGKK | | | | | Mascot |
| 2211.0815 | 2211.0491 | -0.0324 | -15 | 60 | 79 | EENGVLVLTDANFDTFIE GK | | | | | Mascot |
| 2346.3066 | 2346.2458 | -0.0608 | -26 | 164 | 184 | VKEVAQPDWKPPPEATL VLTK | | | | | Mascot |
| 2381.0344 | 2380.978 | -0.0564 | -24 | 444 | 463 | DFPEYTFAIADEEDYADE LK | 153 | 100 | | | Mascot |
| 2381.0344 | 2380.978 | -0.0564 | -24 | 444 | 463 | DFPEYTFAIADEEDYADE LK | | | | | Mascot |
| 3308.4536 | 3308.3765 | -0.0771 | -23 | 302 | 331 | DGDDAVIVGVFSSDEDA AYEIYQEACNSLR | | | Carbamidomethyl (C)[26] | | Mascot |

| | | | | | | | | | | |
|---|---|---|---|---|---|---|---|---|---|---|
| 3 | Pdia4 protein [Danio rerio] | gi\|108742052 | 72421.4 | 5.06 | 24 | 600 | 100 | 441 | 100 | |

**Peptide Information**

| Calc. Mass | Obsrv. Mass | ± da | ± ppm | Start Seq. | End Seq. | Sequence | Ion Score | C. I. % | Modification | Rank | Result Type |
|---|---|---|---|---|---|---|---|---|---|---|---|
| 829.4276 | 829.5081 | 0.0805 | 97 | 497 | 503 | SFVMAFK | | | | | Mascot |
| 882.577 | 882.5466 | -0.0304 | -34 | 522 | 530 | GPVKVVVGK | | | | | Mascot |
| 885.5152 | 885.4941 | -0.0211 | -24 | 292 | 298 | QVQELLR | | | | | Mascot |
| 907.4784 | 907.4562 | -0.0222 | -24 | 426 | 432 | VATQFWR | 49 | 99.638 | | | Mascot |
| 907.4784 | 907.4562 | -0.0222 | -24 | 426 | 432 | VATQFWR | | | | | Mascot |
| 939.4318 | 939.4149 | -0.0169 | -18 | 260 | 267 | AFDYNGPR | | | | | Mascot |
| 939.4318 | 939.4149 | -0.0169 | -18 | 260 | 267 | AFDYNGPR | 49 | 99.691 | | | Mascot |
| 940.4846 | 940.4044 | -0.0802 | -85 | 153 | 160 | SEHAIVER | | | | | Mascot |
| 1011.4781 | 1011.454 | -0.0241 | -24 | 93 | 100 | QFAPEYEK | | | | | Mascot |
| 1041.6316 | 1041.6078 | -0.0238 | -23 | 389 | 397 | HILPLVGHR | | | | | Mascot |
| 1068.5724 | 1068.5441 | -0.0283 | -26 | 244 | 253 | FGVSGYPTLK | | | | | Mascot |
| 1140.5935 | 1140.5654 | -0.0281 | -25 | 129 | 138 | FEVSGYPTIK | | | | | Mascot |
| 1248.6067 | 1248.5758 | -0.0309 | -25 | 232 | 243 | VDATAESDLATR | | | | | Mascot |
| 1470.6747 | 1470.6367 | -0.038 | -26 | 375 | 387 | DSTPASEVQDFFK | | | | | Mascot |
| 1598.7697 | 1598.7292 | -0.0405 | -25 | 375 | 388 | DSTPASEVQDFFKK | | | | | Mascot |
| 1683.8376 | 1683.7931 | -0.0445 | -26 | 594 | 608 | VEGFPTIYFAPSNNK | | | | | Mascot |
| 1683.8376 | 1683.7931 | -0.0445 | -26 | 594 | 608 | VEGFPTIYFAPSNNK | 71 | 99.998 | | | Mascot |
| 1700.7472 | 1700.7043 | -0.0429 | -25 | 483 | 496 | YAMEPEEFDSDVLR | | | | | Mascot |
| 1700.7472 | 1700.7043 | -0.0429 | -25 | 483 | 496 | YAMEPEEFDSDVLR | 119 | 100 | | | Mascot |
| 1711.7996 | 1711.7498 | -0.0498 | -29 | 270 | 285 | FGIVDYMSDQAGPPSK | | | | | Mascot |

| | | | | | | | | | | | |
|---|---|---|---|---|---|---|---|---|---|---|---|
| 1716.7421 | 1716.6989 | -0.0432 | -25 | 483 | 496 | YAMEPEEFDSDVLR | | | Oxidation (M)[3] | | Mascot |
| 1716.7421 | 1716.6989 | -0.0432 | -25 | 483 | 496 | YAMEPEEFDSDVLR | 69 | 99.997 | Oxidation (M)[3] | | Mascot |
| 1727.7944 | 1727.7474 | -0.047 | -27 | 270 | 285 | FGIVDYMSDQAGPPSK | | | Oxidation (M)[7] | | Mascot |
| 1828.8422 | 1828.7953 | -0.0469 | -26 | 482 | 496 | KYAMEPEEFDSDVLR | | | | | Mascot |
| 1844.837 | 1844.792 | -0.045 | -24 | 482 | 496 | KYAMEPEEFDSDVLR | | | Oxidation (M)[4] | | Mascot |
| 2017.0083 | 2016.9615 | -0.0468 | -23 | 461 | 481 | SLGLSESGEEVNVGIVGE GGK | | | | | Mascot |
| 2119.1433 | 2119.0906 | -0.0527 | -25 | 163 | 181 | EVAQPDWKPPPEATLVL TK | | | | | Mascot |
| 2145.1033 | 2145.0481 | -0.0552 | -26 | 461 | 482 | SLGLSESGEEVNVGIVGE GGKK | | | | | Mascot |
| 2211.0815 | 2211.0491 | -0.0324 | -15 | 57 | 76 | EENGVLVLTDANFDTFIE GK | | | | | Mascot |
| 2346.3066 | 2346.2458 | -0.0608 | -26 | 161 | 181 | VKEVAQPDWKPPPEATL VLTK | | | | | Mascot |
| 2381.0344 | 2380.978 | -0.0564 | -24 | 441 | 460 | DFPEYTFAIADEEDYADE LK | 153 | 100 | | | Mascot |
| 2381.0344 | 2380.978 | -0.0564 | -24 | 441 | 460 | DFPEYTFAIADEEDYADE LK | | | | | Mascot |
| 3308.4536 | 3308.3765 | -0.0771 | -23 | 299 | 328 | DGDDAVIVGVFSSDEDA AYEIYQEACNSLR | | | Carbamidomethyl (C)[26] | | Mascot |

| | | | | | | | | | |
|---|---|---|---|---|---|---|---|---|---|
| 4 | Pdia4 protein [Danio rerio] | gi\|66911899 | 25713.2 | 6.13 | 5 | 208 | 100 | 190 | 100 |

**Peptide Information**

| Calc. Mass | Obsrv. Mass | ± da | ± ppm | Start Seq. | End Seq. | Sequence | Ion Score | C. I. % | Modification | Rank | Result Type |
|---|---|---|---|---|---|---|---|---|---|---|---|
| 829.4276 | 829.5081 | 0.0805 | 97 | 83 | 89 | SFVMAFK | | | | | Mascot |
| 882.577 | 882.5466 | -0.0304 | -34 | 108 | 116 | GPVKVVVGK | | | | | Mascot |
| 1683.8376 | 1683.7931 | -0.0445 | -26 | 180 | 194 | VEGFPTIYFAPSNNK | | | | | Mascot |
| 1683.8376 | 1683.7931 | -0.0445 | -26 | 180 | 194 | VEGFPTIYFAPSNNK | 71 | 99.998 | | | Mascot |
| 1700.7472 | 1700.7043 | -0.0429 | -25 | 69 | 82 | YAMEPEEFDSDVLR | | | | | Mascot |
| 1700.7472 | 1700.7043 | -0.0429 | -25 | 69 | 82 | YAMEPEEFDSDVLR | 119 | 100 | | | Mascot |
| 1716.7421 | 1716.6989 | -0.0432 | -25 | 69 | 82 | YAMEPEEFDSDVLR | | | Oxidation (M)[3] | | Mascot |
| 1716.7421 | 1716.6989 | -0.0432 | -25 | 69 | 82 | YAMEPEEFDSDVLR | 69 | 99.997 | Oxidation (M)[3] | | Mascot |
| 1828.8422 | 1828.7953 | -0.0469 | -26 | 68 | 82 | KYAMEPEEFDSDVLR | | | | | Mascot |
| 1844.837 | 1844.792 | -0.045 | -24 | 68 | 82 | KYAMEPEEFDSDVLR | | | Oxidation (M)[4] | | Mascot |

| | | | | | | | | | |
|---|---|---|---|---|---|---|---|---|---|
| 5 | phenylalanyl-tRNA synthetase beta chain [Danio rerio] | gi\|56090156 | 66112.6 | 5.06 | 10 | 58 | 92.783 | 23 | 0 |

**Peptide Information**

| Calc. Mass | Obsrv. Mass | ± da | ± ppm | Start Seq. | End Seq. | Sequence | Ion Score | C. I. % | Modification | Rank | Result Type |
|---|---|---|---|---|---|---|---|---|---|---|---|
| 842.5168 | 842.4906 | -0.0262 | -31 | 469 | 475 | KMPLPLK | | | Oxidation (M)[2] | | Mascot |
| 940.4781 | 940.4044 | -0.0737 | -78 | 148 | 154 | LHQNICR | | | Carbamidomethyl (C)[6] | | Mascot |

| Calc. Mass | Obsrv. Mass | ± da | ± ppm | Start Seq. | End Seq. | Sequence | Ion Score | C. I. % | Modification | Rank | Result Type |
|---|---|---|---|---|---|---|---|---|---|---|---|
| 1011.5516 | 1011.454 | -0.0976 | -96 | 148 | 155 | LHQNICRK | | | | | Mascot |
| 1068.5731 | 1068.5441 | -0.029 | -27 | 148 | 155 | LHQNICRK | | | Carbamidomethyl (C)[6] | | Mascot |
| 1350.7086 | 1350.6696 | -0.039 | -29 | 72 | 82 | YDLLCLEGLVR | | | Carbamidomethyl (C)[5] | | Mascot |
| 1599.8965 | 1599.8459 | -0.0506 | -32 | 558 | 572 | SIGHLGVLHPDVISR | | | | | Mascot |
| 1700.9177 | 1700.7043 | -0.2134 | -125 | 320 | 335 | VGINESAESIAQLLTR | | | | | Mascot |
| 1700.9177 | 1700.7043 | -0.2134 | -125 | 320 | 335 | VGINESAESIAQLLTR | 23 | 0 | | | Mascot |
| 1829.0127 | 1828.7953 | -0.2174 | -119 | 319 | 335 | KVGINESAESIAQLLTR | | | | | Mascot |
| 1940.9559 | 1940.9021 | -0.0538 | -28 | 340 | 357 | SEVSGEDGQIQVEIPPTR | | | | | Mascot |
| 2156.2437 | 2156.1914 | -0.0523 | -24 | 110 | 129 | LLITEETASVRPFAVAAVL R | | | | | Mascot |
| 2168.1267 | 2167.981 | -0.1457 | -67 | 246 | 264 | ISLNTKNVFIECTATDLTK | | | Carbamidomethyl (C)[12] | | Mascot |

| | | | | | | | |
|---|---|---|---|---|---|---|---|
| 6 | Ppp1r3b protein [Danio rerio] | gi\|190337192 | 33606.4 | 5.74 | 9 | 45 | 0 |

**Peptide Information**

| Calc. Mass | Obsrv. Mass | ± da | ± ppm | Start Seq. | End Seq. | Sequence | Ion Score | C. I. % | Modification | Rank | Result Type |
|---|---|---|---|---|---|---|---|---|---|---|---|
| 940.4635 | 940.4044 | -0.0591 | -63 | 67 | 74 | HVSFADHK | | | | | Mascot |
| 1051.5782 | 1051.6881 | 0.1099 | 105 | 157 | 165 | NISFEKSVK | | | | | Mascot |
| 1068.5585 | 1068.5441 | -0.0144 | -13 | 66 | 74 | KHVSFADHK | | | | | Mascot |
| 1179.6521 | 1179.5751 | -0.077 | -65 | 166 | 174 | LRITFDTWK | | | | | Mascot |
| 1683.7651 | 1683.7931 | 0.028 | 17 | 132 | 145 | IENDHVCLEHCMLK | | | | | Mascot |
| 1683.7651 | 1683.7931 | 0.028 | 17 | 132 | 145 | IENDHVCLEHCMLK | | | | | Mascot |
| 1940.9027 | 1940.9021 | -0.0006 | 0 | 132 | 147 | IENDHVCLEHCMLKEK | | | | | Mascot |
| 1997.9242 | 1997.839 | -0.0852 | -43 | 132 | 147 | IENDHVCLEHCMLKEK | | | Carbamidomethyl (C)[7] | | Mascot |
| 1997.9242 | 1997.839 | -0.0852 | -43 | 132 | 147 | IENDHVCLEHCMLKEK | | | Carbamidomethyl (C)[7] | | Mascot |
| 2038.9685 | 2038.8834 | -0.0851 | -42 | 21 | 39 | ASKYSGPLQPCLNQCQS SK | | | | | Mascot |
| 2211.1001 | 2211.0491 | -0.051 | -23 | 2 | 20 | PIELAMSLYLSSDEFLNP R | | | Oxidation (M)[6] | | Mascot |
| 2284.0703 | 2284.1143 | 0.044 | 19 | 168 | 185 | ITFDTWKNHTDVECQYVK | | | Carbamidomethyl (C)[14] | | Mascot |

| | | | | | | | |
|---|---|---|---|---|---|---|---|
| 7 | RecName: Full=Adenosine deaminase; AltName: Full=Adenosine aminohydrolase | gi\|81167689 | 40817.5 | 6.13 | 10 | 41 | 0 |

**Peptide Information**

| Calc. Mass | Obsrv. Mass | ± da | ± ppm | Start Seq. | End Seq. | Sequence | Ion Score | C. I. % | Modification | Rank | Result Type |
|---|---|---|---|---|---|---|---|---|---|---|---|
| 939.4208 | 939.4149 | -0.0059 | -6 | 150 | 156 | SILCCMR | | | Carbamidomethyl (C)[4,5] | | Mascot |
| 939.4208 | 939.4149 | -0.0059 | -6 | 150 | 156 | SILCCMR | | | Carbamidomethyl (C)[4,5] | | Mascot |
| 1068.5111 | 1068.5441 | 0.033 | 31 | 148 | 156 | ARSILCCMR | | | Oxidation (M)[8] | | Mascot |
| 1234.679 | 1234.6483 | -0.0307 | -25 | 91 | 101 | AKEGVIYVEAR | | | | | Mascot |

| | | | | | | | | | | | |
|---|---|---|---|---|---|---|---|---|---|---|---|
| 1248.6405 | 1248.5758 | -0.0647 | -52 | 1 | 11 | MNGKPAFDKPK | | | Oxidation (M)[1] | | Mascot |
| 1470.741 | 1470.6367 | -0.1043 | -71 | 343 | 354 | LLNQLYEAYGMR | | | | | Mascot |
| 1598.8359 | 1598.7292 | -0.1067 | -67 | 343 | 355 | LLNQLYEAYGMRK | | | | | Mascot |
| 1599.9329 | 1599.8459 | -0.087 | -54 | 12 | 25 | VELHVHLDGAIRLK | | | | | Mascot |
| 1727.8785 | 1727.7474 | -0.1311 | -76 | 341 | 354 | EKLLNQLYEAYGMR | | | | | Mascot |
| 1887.8914 | 1887.9225 | 0.0311 | 16 | 157 | 171 | HMPNWSMEVVELCKK | | | Carbamidomethyl (C)[13] | | Mascot |
| 3308.4956 | 3308.3765 | -0.1191 | -36 | 250 | 277 | QLLHQNMHFEMCPVSSR LTGACEPDFTK | | | Carbamidomethyl (C)[12], Oxidation (M)[7,11] | | Mascot |

| | | | | | | | |
|---|---|---|---|---|---|---|---|
| 8 | PREDICTED: si:dkey-170m15.2 [Danio rerio] | gi\|292624792 | 132327.3 | 7.37 | 15 | 41 | 0 |

**Peptide Information**

| Calc. Mass | Obsrv. Mass | ± da | ± ppm | Start Seq. | End Seq. | Sequence | Ion Score | C. I. % | Modification | Rank | Result Type |
|---|---|---|---|---|---|---|---|---|---|---|---|
| 885.4788 | 885.4941 | 0.0153 | 17 | 919 | 926 | STPKSTHK | | | | | Mascot |
| 939.4968 | 939.4149 | -0.0819 | -87 | 75 | 83 | DCPLVAPPK | | | | | Mascot |
| 939.4968 | 939.4149 | -0.0819 | -87 | 75 | 83 | DCPLVAPPK | | | | | Mascot |
| 996.5182 | 996.5735 | 0.0553 | 55 | 75 | 83 | DCPLVAPPK | | | Carbamidomethyl (C)[2] | | Mascot |
| 1011.4411 | 1011.454 | 0.0129 | 13 | 48 | 56 | MADSSESLR | | | Oxidation (M)[1] | | Mascot |
| 1045.516 | 1045.5391 | 0.0231 | 22 | 98 | 106 | QTNITDEPK | | | | | Mascot |
| 1165.6034 | 1165.7177 | 0.1143 | 98 | 440 | 449 | FCEQAVTIVR | | | | | Mascot |
| 1434.879 | 1434.7368 | -0.1422 | -99 | 724 | 736 | LIGSREPLPLLAR | | | | | Mascot |
| 1700.7942 | 1700.7043 | -0.0899 | -53 | 42 | 56 | TSMLTRMADSSESLR | | | Oxidation (M)[3] | | Mascot |
| 1700.7942 | 1700.7043 | -0.0899 | -53 | 42 | 56 | TSMLTRMADSSESLR | 0 | 0 | Oxidation (M)[7] | | Mascot |
| 1707.8846 | 1707.7374 | -0.1472 | -86 | 436 | 449 | ESLRFCEQAVTIVR | | | Carbamidomethyl (C)[6] | | Mascot |
| 1711.8506 | 1711.7498 | -0.1008 | -59 | 1122 | 1135 | MLEAREAFQSCILK | | | Carbamidomethyl (C)[11], Oxidation (M)[1] | | Mascot |
| 1716.7891 | 1716.6989 | -0.0902 | -53 | 42 | 56 | TSMLTRMADSSESLR | | | Oxidation (M)[3,7] | | Mascot |
| 1716.7891 | 1716.6989 | -0.0902 | -53 | 42 | 56 | TSMLTRMADSSESLR | 3 | 0 | Oxidation (M)[3,7] | | Mascot |
| 1727.8633 | 1727.7474 | -0.1159 | -67 | 773 | 787 | ATVQCQTSKSDVYIK | | | Carbamidomethyl (C)[5] | | Mascot |
| 1829.0565 | 1828.7953 | -0.2612 | -143 | 372 | 388 | SIISKLPVVQIQTTACK | | | | | Mascot |
| 1887.9196 | 1887.9225 | 0.0029 | 2 | 525 | 541 | LLEARSADETPEGWTGR | | | | | Mascot |
| 1940.7823 | 1940.9021 | 0.1198 | 62 | 1104 | 1119 | QMETCIGFQDTMDAYR | | | Oxidation (M)[2,12] | | Mascot |
| 1997.8038 | 1997.839 | 0.0352 | 18 | 1104 | 1119 | QMETCIGFQDTMDAYR | | | Carbamidomethyl (C)[5], Oxidation (M)[2,12] | | Mascot |
| 1997.8038 | 1997.839 | 0.0352 | 18 | 1104 | 1119 | QMETCIGFQDTMDAYR | | | Carbamidomethyl (C)[5], Oxidation (M)[2,12] | | Mascot |
| 2168.0098 | 2167.981 | -0.0288 | -13 | 1141 | 1158 | KMEEKPMETVEQVETES K | | | Oxidation (M)[2] | | Mascot |
| 2283.1875 | 2283.1204 | -0.0671 | -29 | 663 | 682 | MFSPIPVCILHVINNDTLE K | | | | | Mascot |

| | | | | | | | |
|---|---|---|---|---|---|---|---|
| 9 | exosome component 10 [Danio rerio] | gi\|210147550 | 102307.7 | 8.64 | 14 | 41 | 0 |

Peptide Information

| Calc. Mass | Obsrv. Mass | ± da | ± ppm | Start Seq. | End Seq. | Sequence | Ion Score | C. I. % | Modification | Rank | Result Type |
|---|---|---|---|---|---|---|---|---|---|---|---|
| 882.4389 | 882.5466 | 0.1077 | 122 | 880 | 887 | SMSYVPAK | | | | | Mascot |
| 939.5258 | 939.4149 | -0.1109 | -118 | 399 | 406 | NSLDHLLK | | | | | Mascot |
| 939.5258 | 939.4149 | -0.1109 | -118 | 399 | 406 | NSLDHLLK | | | | | Mascot |
| 996.5948 | 996.5735 | -0.0213 | -21 | 179 | 186 | NIQRPQLK | | | | | Mascot |
| 1041.5377 | 1041.6078 | 0.0701 | 67 | 891 | 898 | GFRHNWPK | | | | | Mascot |
| 1045.5636 | 1045.5391 | -0.0245 | -23 | 105 | 113 | NKLTGLEDR | | | | | Mascot |
| 1570.7972 | 1570.736 | -0.0612 | -39 | 597 | 610 | KPQSTLFGPHDTSR | | | | | Mascot |
| 1652.89 | 1652.7042 | -0.1858 | -112 | 393 | 406 | CLNLGRNSLDHLLK | | | Carbamidomethyl (C)[1] | | Mascot |
| 1844.9348 | 1844.792 | -0.1428 | -77 | 779 | 795 | ERAASDVSEDSLKPNVK | | | | | Mascot |
| 1888.0022 | 1887.9225 | -0.0797 | -42 | 781 | 798 | AASDVSEDSLKPNVKLSK | | | | | Mascot |
| 2017.0138 | 2016.9615 | -0.0523 | -26 | 302 | 318 | LAKTTEFAVDLEHHSYR | | | | | Mascot |
| 2119.0542 | 2119.0906 | 0.0364 | 17 | 499 | 515 | SFNTQQLAAFRLMYAWR | | | Oxidation (M)[13] | | Mascot |
| 2283.1079 | 2283.1204 | 0.0125 | 5 | 830 | 848 | QDFKPYDYSQSNFKLFAGK | | | | | Mascot |
| 2346.2188 | 2346.2458 | 0.027 | 12 | 107 | 127 | LTGLEDRFDLVVDANDAILEK | | | | | Mascot |
| 2381.0935 | 2380.978 | -0.1155 | -49 | 518 | 537 | LAREEDESTGYILPNHMMMK | | | Oxidation (M)[17] | | Mascot |
| 2381.0935 | 2380.978 | -0.1155 | -49 | 518 | 537 | LAREEDESTGYILPNHMMMK | | | Oxidation (M)[17] | | Mascot |

| | | | | | | | |
|---|---|---|---|---|---|---|---|
| 10 | PREDICTED: calcium-dependent secretion activator 1 [Danio rerio] | gi\|326668736 | 141178 | 5.62 | 16 | 38 | 0 |

Peptide Information

| Calc. Mass | Obsrv. Mass | ± da | ± ppm | Start Seq. | End Seq. | Sequence | Ion Score | C. I. % | Modification | Rank | Result Type |
|---|---|---|---|---|---|---|---|---|---|---|---|
| 868.4635 | 868.4709 | 0.0074 | 9 | 1125 | 1131 | HSQDILR | | | | | Mascot |
| 1041.5364 | 1041.6078 | 0.0714 | 69 | 123 | 130 | DRFESFLK | | | | | Mascot |
| 1045.5459 | 1045.5391 | -0.0068 | -7 | 280 | 288 | LQMADQIAR | | | | | Mascot |
| 1179.6343 | 1179.5751 | -0.0592 | -50 | 578 | 586 | ILWVQAMYR | | | | | Mascot |
| 1350.6504 | 1350.6696 | 0.0192 | 14 | 161 | 173 | MVQTGGLSALDCR | | | | | Mascot |
| 1493.7166 | 1493.697 | -0.0196 | -13 | 789 | 800 | CLEQAAQINYQR | | | Carbamidomethyl (C)[1] | | Mascot |
| 1599.7791 | 1599.8459 | 0.0668 | 42 | 982 | 995 | MKLMSSDMIETSVK | | | | | Mascot |
| 1700.7288 | 1700.7043 | -0.0245 | -14 | 1032 | 1046 | DQSAKLCAMEMGQEK | | | Oxidation (M)[9,11] | | Mascot |
| 1700.7288 | 1700.7043 | -0.0245 | -14 | 1032 | 1046 | DQSAKLCAMEMGQEK | | | Oxidation (M)[9,11] | | Mascot |
| 1838.9213 | 1838.8722 | -0.0491 | -27 | 1016 | 1031 | IPLSLCTMFNVMVDAK | | | Carbamidomethyl (C)[6] | | Mascot |
| 1910.9463 | 1910.8079 | -0.1384 | -72 | 161 | 177 | MVQTGGLSALDCREVFK | | | Carbamidomethyl (C)[12] | | Mascot |

| Calc. Mass | Obsrv. Mass | ± da | ± ppm | Start Seq. | End Seq. | Sequence | Ion Score | C. I. % | Modification | Rank | Result Type |
|---|---|---|---|---|---|---|---|---|---|---|---|
| 1910.9463 | 1910.8079 | -0.1384 | -72 | 161 | 177 | MVQTGGLSALDCREVFK | | | Carbamidomethyl (C)[12] | | Mascot |
| 2145.1775 | 2145.0481 | -0.1294 | -60 | 587 | 606 | ATGQSHKPVPPTQVQKLNSK | | | | | Mascot |
| 2156.0554 | 2156.1914 | 0.136 | 63 | 789 | 806 | CLEQAAQINYQRITDYAR | | | | | Mascot |
| 2168.0474 | 2167.981 | -0.0664 | -31 | 377 | 395 | SLAPNRIVYCTMEVEGGQK | | | Carbamidomethyl (C)[10], Oxidation (M)[12] | | Mascot |
| 2224.9397 | 2225.0632 | 0.1235 | 56 | 1 | 20 | MLDPSSSEEESDGIVEEESR | | | | | Mascot |
| 2224.9397 | 2225.0632 | 0.1235 | 56 | 1 | 20 | MLDPSSSEEESDGIVEEESR | | | | | Mascot |
| 2233.166 | 2233.0325 | -0.1335 | -60 | 84 | 101 | LQLYVFVMRCIAYPFNAK | | | Carbamidomethyl (C)[10] | | Mascot |
| 2346.1055 | 2346.2458 | 0.1403 | 60 | 50 | 72 | GSSARPTSPSPSAASEEKEDVEK | | | | | Mascot |

11 CTAGE family, member 5 [Danio rerio] gi|41053471 77796.7 5.5 13 38 0

**Peptide Information**

| Calc. Mass | Obsrv. Mass | ± da | ± ppm | Start Seq. | End Seq. | Sequence | Ion Score | C. I. % | Modification | Rank | Result Type |
|---|---|---|---|---|---|---|---|---|---|---|---|
| 850.4417 | 850.5214 | 0.0797 | 94 | 404 | 410 | LTDAQFR | | | | | Mascot |
| 940.4734 | 940.4044 | -0.069 | -73 | 264 | 271 | EDLHEGIK | | | | | Mascot |
| 1011.5217 | 1011.454 | -0.0677 | -67 | 117 | 124 | IHEINTER | | | | | Mascot |
| 1051.567 | 1051.6881 | 0.1211 | 115 | 15 | 23 | VLETFSEVK | | | | | Mascot |
| 1068.5659 | 1068.5441 | -0.0218 | -20 | 634 | 643 | GPLPPGMFPR | | | | | Mascot |
| 1350.5236 | 1350.6696 | 0.146 | 108 | 170 | 181 | SHAGMMEDCANK | | | Carbamidomethyl (C)[9] | | Mascot |
| 1683.7386 | 1683.7931 | 0.0545 | 32 | 157 | 169 | LSELEEEMKMCER | | | Carbamidomethyl (C)[11] | | Mascot |
| 1683.7386 | 1683.7931 | 0.0545 | 32 | 157 | 169 | LSELEEEMKMCER | | | Carbamidomethyl (C)[11] | | Mascot |
| 1711.6469 | 1711.7498 | 0.1029 | 60 | 170 | 184 | SHAGMMEDCANKDDR | | | Oxidation (M)[5,6] | | Mascot |
| 1819.8966 | 1819.7543 | -0.1423 | -78 | 29 | 45 | LESTLQNNGMSARAAEK | | | | | Mascot |
| 1828.6903 | 1828.7953 | 0.105 | 57 | 166 | 181 | MCERSHAGMMEDCANK | | | Oxidation (M)[1] | | Mascot |
| 1844.6853 | 1844.792 | 0.1067 | 58 | 166 | 181 | MCERSHAGMMEDCANK | | | Oxidation (M)[1,9] | | Mascot |
| 2020.0016 | 2019.8184 | -0.1832 | -91 | 46 | 63 | DTLEVASQKLEQSNAQMK | | | | | Mascot |
| 2233.0725 | 2233.0325 | -0.04 | -18 | 605 | 623 | DPHFPHRGPFGPPDYFPPR | | | | | Mascot |
| 2283.136 | 2283.1204 | -0.0156 | -7 | 294 | 311 | LQQKLQIMTEMYQENELK | | | Oxidation (M)[8] | | Mascot |

12 Unknown (protein for IMAGE:7251921) [Danio rerio] gi|148744663 44771.3 6.74 10 38 0

**Peptide Information**

| Calc. Mass | Obsrv. Mass | ± da | ± ppm | Start Seq. | End Seq. | Sequence | Ion Score | C. I. % | Modification | Rank | Result Type |
|---|---|---|---|---|---|---|---|---|---|---|---|
| 907.3751 | 907.4562 | 0.0811 | 89 | 91 | 98 | SESDADQR | | | | | Mascot |
| 907.3751 | 907.4562 | 0.0811 | 89 | 91 | 98 | SESDADQR | | | | | Mascot |

| | | | | | | | | | | | |
|---|---|---|---|---|---|---|---|---|---|---|---|
| 1570.7361 | 1570.736 | -0.0001 | 0 | 188 | 201 | RCIAGVCVCQMPYK | | | | | Mascot |
| 1599.7432 | 1599.8459 | 0.1027 | 64 | 241 | 253 | TCKTDENVQTTFR | | | Carbamidomethyl (C)[2] | | Mascot |
| 1700.7739 | 1700.7043 | -0.0696 | -41 | 188 | 201 | RCIAGVCVCQMPYK | | | Carbamidomethyl (C)[2,7], Oxidation (M)[11] | | Mascot |
| 1700.7739 | 1700.7043 | -0.0696 | -41 | 188 | 201 | RCIAGVCVCQMPYK | | | Carbamidomethyl (C)[2,7], Oxidation (M)[11] | | Mascot |
| 1711.7882 | 1711.7498 | -0.0384 | -22 | 244 | 258 | TDENVQTTFRNSGDK | | | | | Mascot |
| 1998.0802 | 1997.839 | -0.2412 | -121 | 3 | 20 | ILCAFIFICVLQSASLEK | | | | | Mascot |
| 1998.0802 | 1997.839 | -0.2412 | -121 | 3 | 20 | ILCAFIFICVLQSASLEK | | | | | Mascot |
| 2144.969 | 2145.0481 | 0.0791 | 37 | 111 | 130 | STTESPNTNSQQNPANTPEK | | | | | Mascot |
| 2211.2029 | 2211.0491 | -0.1538 | -70 | 2 | 20 | RILCAFIFICVLQSASLEK | | | Carbamidomethyl (C)[4] | | Mascot |
| 2283.083 | 2283.1204 | 0.0374 | 16 | 268 | 287 | GKMLVCGSEWNIAAANVFCR | | | Carbamidomethyl (C)[6,19] | | Mascot |
| 2283.9502 | 2284.1143 | 0.1641 | 72 | 209 | 229 | ACTLDGAEFYSMCQTNAIACR | | | Oxidation (M)[12] | | Mascot |
| 2403.03 | 2402.9583 | -0.0717 | -30 | 300 | 319 | ASKMQYVELEGEDWPDQCMK | | | Oxidation (M)[4] | | Mascot |

13 Ppp4r2a protein [Danio rerio] gi|38173867 48758.5 4.37 9 36 0

**Peptide Information**

| Calc. Mass | Obsrv. Mass | ± da | ± ppm | Start Seq. | End Seq. | Sequence | Ion Score | C. I. % | Modification | Rank | Result Type |
|---|---|---|---|---|---|---|---|---|---|---|---|
| 850.4199 | 850.5214 | 0.1015 | 119 | 165 | 172 | KGSSCVNR | | | | | Mascot |
| 907.4413 | 907.4562 | 0.0149 | 16 | 165 | 172 | KGSSCVNR | 3 | 0 | Carbamidomethyl (C)[5] | | Mascot |
| 907.4413 | 907.4562 | 0.0149 | 16 | 165 | 172 | KGSSCVNR | | | Carbamidomethyl (C)[5] | | Mascot |
| 1045.5598 | 1045.5391 | -0.0207 | -20 | 127 | 135 | LCELLTEPK | | | | | Mascot |
| 1140.5605 | 1140.5654 | 0.0049 | 4 | 76 | 84 | LEKVMDDFK | | | Oxidation (M)[5] | | Mascot |
| 1598.6937 | 1598.7292 | 0.0355 | 22 | 79 | 91 | VMDDFKASCPEQR | | | Carbamidomethyl (C)[9], Oxidation (M)[2] | | Mascot |
| 1683.8444 | 1683.7931 | -0.0513 | -30 | 151 | 165 | NVMVVSCVYPTSEKK | | | | | Mascot |
| 1683.8444 | 1683.7931 | -0.0513 | -30 | 151 | 165 | NVMVVSCVYPTSEKK | | | | | Mascot |
| 1707.837 | 1707.7374 | -0.0996 | -58 | 20 | 34 | HTSTMEIDTLLGAFR | | | Oxidation (M)[5] | | Mascot |
| 2119.0276 | 2119.0906 | 0.063 | 30 | 92 | 109 | GPANPNVEYIPFEEMKQR | | | | | Mascot |
| 2186.0798 | 2185.9915 | -0.0883 | -40 | 1 | 19 | SLLPSFCFQLDSERLESSK | | | | | Mascot |
| 2186.0798 | 2185.9915 | -0.0883 | -40 | 1 | 19 | SLLPSFCFQLDSERLESSK | | | | | Mascot |
| 2211.075 | 2211.0491 | -0.0259 | -12 | 20 | 38 | HTSTMEIDTLLGAFRDFEK | | | | | Mascot |

14 PREDICTED: calcium-dependent secretion activator 1 [Danio rerio] gi|326668734 142952 5.72 16 36 0

**Peptide Information**

| Calc. Mass | Obsrv. Mass | ± da | ± ppm | Start Seq. | End Seq. | Sequence | Ion Score | C. I. % | Modification | Rank | Result Type |
|---|---|---|---|---|---|---|---|---|---|---|---|

| | | | | | | | | | | | |
|---|---|---|---|---|---|---|---|---|---|---|---|
| 868.4635 | 868.4709 | 0.0074 | 9 | 1138 | 1144 | HSQDILR | | | | | Mascot |
| 1041.5364 | 1041.6078 | 0.0714 | 69 | 123 | 130 | DRFESFLK | | | | | Mascot |
| 1045.5459 | 1045.5391 | -0.0068 | -7 | 280 | 288 | LQMADQIAR | | | | | Mascot |
| 1179.6343 | 1179.5751 | -0.0592 | -50 | 578 | 586 | ILWVQAMYR | | | | | Mascot |
| 1350.6504 | 1350.6696 | 0.0192 | 14 | 161 | 173 | MVQTGGLSALDCR | | | | | Mascot |
| 1493.7166 | 1493.697 | -0.0196 | -13 | 802 | 813 | CLEQAAQINYQR | | | Carbamidomethyl (C)[1] | | Mascot |
| 1599.7791 | 1599.8459 | 0.0668 | 42 | 995 | 1008 | MKLMSSDMIETSVK | | | | | Mascot |
| 1700.7288 | 1700.7043 | -0.0245 | -14 | 1045 | 1059 | DQSAKLCAMEMGQEK | | | Oxidation (M)[9,11] | | Mascot |
| 1700.7288 | 1700.7043 | -0.0245 | -14 | 1045 | 1059 | DQSAKLCAMEMGQEK | | | Oxidation (M)[9,11] | | Mascot |
| 1838.9213 | 1838.8722 | -0.0491 | -27 | 1029 | 1044 | IPLSLCTMFNVMVDAK | | | Carbamidomethyl (C)[6] | | Mascot |
| 1910.9463 | 1910.8079 | -0.1384 | -72 | 161 | 177 | MVQTGGLSALDCREVFK | | | Carbamidomethyl (C)[12] | | Mascot |
| 1910.9463 | 1910.8079 | -0.1384 | -72 | 161 | 177 | MVQTGGLSALDCREVFK | | | Carbamidomethyl (C)[12] | | Mascot |
| 2145.1775 | 2145.0481 | -0.1294 | -60 | 587 | 606 | ATGQSHKPVPPTQVQKL NSK | | | | | Mascot |
| 2156.0554 | 2156.1914 | 0.136 | 63 | 802 | 819 | CLEQAAQINYQRITDYAR | | | | | Mascot |
| 2168.0474 | 2167.981 | -0.0664 | -31 | 377 | 395 | SLAPNRIVYCTMEVEGG QK | | | Carbamidomethyl (C)[10], Oxidation (M)[12] | | Mascot |
| 2224.9397 | 2225.0632 | 0.1235 | 56 | 1 | 20 | MLDPSSSEEESDGIVEEE SR | | | | | Mascot |
| 2224.9397 | 2225.0632 | 0.1235 | 56 | 1 | 20 | MLDPSSSEEESDGIVEEE SR | | | | | Mascot |
| 2233.166 | 2233.0325 | -0.1335 | -60 | 84 | 101 | LQLYVFVMRCIAYPFNAK | | | Carbamidomethyl (C)[10] | | Mascot |
| 2346.1055 | 2346.2458 | 0.1403 | 60 | 50 | 72 | GSSARPTSPSPSAASEE KEDVEK | | | | | Mascot |

| | | | | | | | |
|---|---|---|---|---|---|---|---|
| 15 | novel protein [Danio rerio] | gi\|94733367 | 48029.7 | 9.11 | 12 | 36 | 0 |

**Peptide Information**

| Calc. Mass | Obsrv. Mass | ± da | ± ppm | Start Seq. | End Seq. | Sequence | Ion Score | C. I. % | Modification | Rank | Result Type |
|---|---|---|---|---|---|---|---|---|---|---|---|
| 885.4901 | 885.4941 | 0.004 | 5 | 1 | 7 | PISERQR | | | | | Mascot |
| 1045.5789 | 1045.5391 | -0.0398 | -38 | 282 | 290 | SFAQKSHLK | | | | | Mascot |
| 1068.5507 | 1068.5441 | -0.0066 | -6 | 310 | 318 | SFMKATDLR | | | | | Mascot |
| 1248.5712 | 1248.5758 | 0.0046 | 4 | 17 | 26 | MSDPEPCRIK | | | Carbamidomethyl (C)[7], Oxidation (M)[1] | | Mascot |
| 1707.7941 | 1707.7374 | -0.0567 | -33 | 295 | 309 | IHTGVREFVCSDCGK | | | Carbamidomethyl (C)[10] | | Mascot |
| 1819.9358 | 1819.7543 | -0.1815 | -100 | 25 | 39 | IKQEETEELIDVMVK | | | Oxidation (M)[13] | | Mascot |
| 1910.8557 | 1910.8079 | -0.0478 | -25 | 210 | 225 | MIHTGEKPFTCTQCGK | | | Carbamidomethyl (C)[11,14], Oxidation (M)[1] | | Mascot |
| 1910.8557 | 1910.8079 | -0.0478 | -25 | 210 | 225 | MIHTGEKPFTCTQCGK | | | Carbamidomethyl (C)[11,14], Oxidation (M)[1] | | Mascot |
| 2016.963 | 2016.9615 | -0.0015 | -1 | 264 | 281 | HLIGHTKEKPYSCSECGK | | | | | Mascot |
| 2156.0125 | 2156.1914 | 0.1789 | 83 | 123 | 141 | RHLGVHSTERPYSCPEC GK | | | | | Mascot |
| 2225.03 | 2225.0632 | 0.0332 | 15 | 207 | 225 | YHKMIHTGEKPFTCTQC GK | | | Oxidation (M)[4] | | Mascot |

| | | | | | | | | | | | | |
|---|---|---|---|---|---|---|---|---|---|---|---|---|
| 2225.03 | 2225.0632 | 0.0332 | 15 | 207 | 225 | YHKMIHTGEKPFTCTQC GK | | | Oxidation (M)[4] | | Mascot |
| 2232.9883 | 2233.0325 | 0.0442 | 20 | 95 | 113 | HHMMIHAGEKPHKCDQC SK | | | Oxidation (M)[3] | | Mascot |
| 2283.0757 | 2283.1204 | 0.0447 | 20 | 351 | 369 | IHTGEKPFTCTQCGRSFT R | | | Carbamidomethyl (C)[10,13] | | Mascot |

| | | | | | | | |
|---|---|---|---|---|---|---|---|
| 16 | novel protein similar to vertebrate tropomyosin 4 (TPM4) (zgc:63909) [Danio rerio] | gi\|94732583 | 23433.8 | 4.6 | 8 | 35 | 0 |

**Peptide Information**

| Calc. Mass | Obsrv. Mass | ± da | ± ppm | Start Seq. | End Seq. | Sequence | Ion Score | C. I. % | Modification | Rank | Result Type |
|---|---|---|---|---|---|---|---|---|---|---|---|
| 850.3975 | 850.5214 | 0.1239 | 146 | 54 | 60 | AMKDEEK | | | | | Mascot |
| 940.4482 | 940.4044 | -0.0438 | -47 | 73 | 80 | HIAEEADR | | | | | Mascot |
| 1068.5432 | 1068.5441 | 0.0009 | 1 | 73 | 81 | HIAEEADRK | | | | | Mascot |
| 1165.5592 | 1165.7177 | 0.1585 | 136 | 61 | 69 | MEIQEMQLK | | | Oxidation (M)[1] | | Mascot |
| 1234.5732 | 1234.6483 | 0.0751 | 61 | 99 | 109 | AEERAEVAECK | | | | | Mascot |
| 1434.7322 | 1434.7368 | 0.0046 | 3 | 172 | 184 | SIDDLEEKLSSAK | | | | | Mascot |
| 1493.7338 | 1493.697 | -0.0368 | -25 | 61 | 72 | MEIQEMQLKEAK | | | Oxidation (M)[1] | | Mascot |
| 1727.8922 | 1727.7474 | -0.1448 | -84 | 12 | 25 | IQLVEEELDRAQER | | | | | Mascot |

| | | | | | | | |
|---|---|---|---|---|---|---|---|
| 17 | PREDICTED: metabotropic glutamate receptor 5-like [Danio rerio] | gi\|292611331 | 130054.1 | 8.37 | 16 | 35 | 0 |

**Peptide Information**

| Calc. Mass | Obsrv. Mass | ± da | ± ppm | Start Seq. | End Seq. | Sequence | Ion Score | C. I. % | Modification | Rank | Result Type |
|---|---|---|---|---|---|---|---|---|---|---|---|
| 850.5257 | 850.5214 | -0.0043 | -5 | 270 | 276 | LRSHLPK | | | | | Mascot |
| 1051.5684 | 1051.6881 | 0.1197 | 114 | 950 | 957 | YPVTYRPR | | | | | Mascot |
| 1068.5684 | 1068.5441 | -0.0243 | -23 | 841 | 850 | SAFTTSTVVR | | | | | Mascot |
| 1140.4659 | 1140.5654 | 0.0995 | 87 | 134 | 144 | CSTEGGGTPMK | | | Carbamidomethyl (C)[1], Oxidation (M)[10] | | Mascot |
| 1234.5555 | 1234.6483 | 0.0928 | 75 | 74 | 83 | VEAMMHTLDR | | | Oxidation (M)[4,5] | | Mascot |
| 1475.7238 | 1475.7256 | 0.0018 | 1 | 391 | 402 | ESLRHQYAQDTK | | | | | Mascot |
| 1598.76 | 1598.7292 | -0.0308 | -19 | 277 | 290 | ARVVACFCEGMTVR | | | Carbamidomethyl (C)[6] | | Mascot |
| 1683.8271 | 1683.7931 | -0.034 | -20 | 193 | 206 | YFMRVVPSDAQQAR | | | Oxidation (M)[3] | | Mascot |
| 1683.8271 | 1683.7931 | -0.034 | -20 | 193 | 206 | YFMRVVPSDAQQAR | | | Oxidation (M)[3] | | Mascot |
| 1707.9349 | 1707.7374 | -0.1975 | -116 | 655 | 671 | LGIGLSPAMSYSALVTK | | | | | Mascot |
| 1933.8596 | 1933.9635 | 0.1039 | 54 | 1096 | 1113 | ASPASLFDSTYDSSCAVR | | | Carbamidomethyl (C)[15] | | Mascot |
| 1940.9712 | 1940.9021 | -0.0691 | -36 | 317 | 334 | YDVTDGFQREAAGGITIK | | | | | Mascot |
| 2118.9988 | 2119.0906 | 0.0918 | 43 | 279 | 297 | VVACFCEGMTVRGILMA MR | | | Oxidation (M)[9,16] | | Mascot |
| 2149.0938 | 2148.9441 | -0.1497 | -70 | 888 | 904 | TVTWAQNERSYRPNLWK | | | | | Mascot |

| | | | | | | | | | | | |
|---|---|---|---|---|---|---|---|---|---|---|---|
| 2167.9932 | 2167.981 | -0.0122 | -6 | 423 | 442 | SLCPGYAGLCEAMRPID GGK | | | Carbamidomethyl (C)[3,10], Oxidation (M)[13] | | Mascot |
| 2233.0417 | 2233.0325 | -0.0092 | -4 | 279 | 297 | VVACFCEGMTVRGILMA MR | | | Carbamidomethyl (C)[4,6], Oxidation (M)[9,16] | | Mascot |
| 2283.0522 | 2283.1204 | 0.0682 | 30 | 450 | 471 | TNFSGVSGEVVLFDENG DSPGR | | | | | Mascot |
| 2498.1873 | 2498.2664 | 0.0791 | 32 | 805 | 827 | IITMCFSVSLSATTALCCM FVPK | | | Oxidation (M)[4,19] | | Mascot |

18 integrin-linked protein kinase [Danio rerio] gi|41055748 51030.6 7.26 9 35 0

**Peptide Information**

| Calc. Mass | Obsrv. Mass | ± da | ± ppm | Start Seq. | End Seq. | Sequence | Ion Score | C. I. % | Modification | Rank | Result Type |
|---|---|---|---|---|---|---|---|---|---|---|---|
| 885.4788 | 885.4941 | 0.0153 | 17 | 364 | 370 | KPEEINR | | | | | Mascot |
| 907.4454 | 907.4562 | 0.0108 | 12 | 342 | 349 | LSFQCPGR | | | | | Mascot |
| 907.4454 | 907.4562 | 0.0108 | 12 | 342 | 349 | LSFQCPGR | | | | | Mascot |
| 940.408 | 940.4044 | -0.0036 | -4 | 132 | 139 | YGETPMDK | | | | | Mascot |
| 1041.58 | 1041.6078 | 0.0278 | 27 | 364 | 371 | KPEEINRR | | | | | Mascot |
| 1234.679 | 1234.6483 | -0.0307 | -25 | 155 | 165 | QGQSLSKVPYK | | | | | Mascot |
| 1711.9312 | 1711.7498 | -0.1814 | -106 | 212 | 225 | WQGTEIVVKMLHVR | | | Oxidation (M)[10] | | Mascot |
| 1940.8953 | 1940.9021 | 0.0068 | 4 | 1 | 17 | MDDIFTQCREGNAVAVR | | | Oxidation (M)[1] | | Mascot |
| 1997.9167 | 1997.839 | -0.0777 | -39 | 1 | 17 | MDDIFTQCREGNAVAVR | | | Carbamidomethyl (C)[8], Oxidation (M)[1] | | Mascot |
| 1997.9167 | 1997.839 | -0.0777 | -39 | 1 | 17 | MDDIFTQCREGNAVAVR | | | Carbamidomethyl (C)[8], Oxidation (M)[1] | | Mascot |
| 2168.0918 | 2167.981 | -0.1108 | -51 | 192 | 209 | QLNMLNKINENHSGELW K | | | | | Mascot |
| 2346.1807 | 2346.2458 | 0.0651 | 28 | 297 | 317 | FALDIACGMAFLHTLEPMI PR | | | | | Mascot |
| 2403.2021 | 2402.9583 | -0.2438 | -101 | 297 | 317 | FALDIACGMAFLHTLEPMI PR | | | Carbamidomethyl (C)[7] | | Mascot |

19 inositol monophosphatase 1 [Danio rerio] gi|57526522 30559.3 4.86 7 35 0

**Peptide Information**

| Calc. Mass | Obsrv. Mass | ± da | ± ppm | Start Seq. | End Seq. | Sequence | Ion Score | C. I. % | Modification | Rank | Result Type |
|---|---|---|---|---|---|---|---|---|---|---|---|
| 868.4709 | 868.4709 | 0 | 0 | 175 | 181 | IFSSMRK | | | | | Mascot |
| 885.5264 | 885.4941 | -0.0323 | -36 | 249 | 256 | RVLAANNK | | | | | Mascot |
| 1248.7609 | 1248.5758 | -0.1851 | -148 | 181 | 191 | KILCLPVHGIR | | | | | Mascot |
| 1838.8854 | 1838.8722 | -0.0132 | -7 | 2 | 17 | PDLWQDAMDHAVTLAR | | | | | Mascot |
| 1844.9963 | 1844.792 | -0.2043 | -111 | 46 | 61 | TDQNVEQLIITSVKEK | | | | | Mascot |
| 2225.0618 | 2225.0632 | 0.0014 | 1 | 116 | 134 | TLEFGVVYSCIEDKMYTA R | | | | | Mascot |
| 2225.0618 | 2225.0632 | 0.0014 | 1 | 116 | 134 | TLEFGVVYSCIEDKMYTA R | | | | | Mascot |
| 3308.5964 | 3308.3765 | -0.2199 | -66 | 138 | 167 | GAFCNGQPLQVSDQKEI NQSIIATEFGSNR | | | Carbamidomethyl (C)[4] | | Mascot |

| | | | | | | | |
|---|---|---|---|---|---|---|---|
| 20 | hypothetical protein LOC100170532 [Xenopus (Silurana) tropicalis] | gi\|194332586 | 43090.5 | 8.65 | 8 | 34 | 0 |

Peptide Information

| Calc. Mass | Obsrv. Mass | ± da | ± ppm | Start Seq. | End Seq. | Sequence | Ion Score | C. I. % | Modification | Rank | Result Type |
|---|---|---|---|---|---|---|---|---|---|---|---|
| 907.46 | 907.4562 | -0.0038 | -4 | 1 | 8 | MAACAIRR | | | Oxidation (M)[1] | | Mascot |
| 907.46 | 907.4562 | -0.0038 | -4 | 1 | 8 | MAACAIRR | | | Oxidation (M)[1] | | Mascot |
| 939.4642 | 939.4149 | -0.0493 | -52 | 150 | 157 | DSPHISQR | | | | | Mascot |
| 939.4642 | 939.4149 | -0.0493 | -52 | 150 | 157 | DSPHISQR | | | | | Mascot |
| 1011.508 | 1011.454 | -0.054 | -53 | 208 | 215 | FLMFNPSR | | | | | Mascot |
| 1140.6735 | 1140.5654 | -0.1081 | -95 | 308 | 318 | GVVGKEPVLSR | | | | | Mascot |
| 1475.6815 | 1475.7256 | 0.0441 | 30 | 262 | 272 | EAQNSHVYWWR | | | | | Mascot |
| 2145.0625 | 2145.0481 | -0.0144 | -7 | 227 | 243 | AWQEKNHPWLELSDVHR | | | | | Mascot |
| 2233.1511 | 2233.0325 | -0.1186 | -53 | 81 | 99 | QHGKYETGQLFLHSVLGYR | | | | | Mascot |
| 2346.1584 | 2346.2458 | 0.0874 | 37 | 232 | 250 | NHPWLELSDVHRETTENIR | | | | | Mascot |

| | | | | | | | |
|---|---|---|---|---|---|---|---|
| 21 | PREDICTED: cytosolic carboxypeptidase 1-like [Danio rerio] | gi\|326680138 | 40420.7 | 5.5 | 8 | 34 | 0 |

Peptide Information

| Calc. Mass | Obsrv. Mass | ± da | ± ppm | Start Seq. | End Seq. | Sequence | Ion Score | C. I. % | Modification | Rank | Result Type |
|---|---|---|---|---|---|---|---|---|---|---|---|
| 829.489 | 829.5081 | 0.0191 | 23 | 88 | 94 | DRQIGLK | | | | | Mascot |
| 885.5516 | 885.4941 | -0.0575 | -65 | 90 | 97 | QIGLKAQK | | | | | Mascot |
| 1011.5833 | 1011.454 | -0.1293 | -128 | 271 | 279 | IPLPLTSDR | | | | | Mascot |
| 1320.7206 | 1320.5513 | -0.1693 | -128 | 150 | 160 | LMSPHTTKHLR | | | | | Mascot |
| 1683.9462 | 1683.7931 | -0.1531 | -91 | 252 | 266 | GLESLVEPAIQLMRK | | | | | Mascot |
| 1683.9462 | 1683.7931 | -0.1531 | -91 | 252 | 266 | GLESLVEPAIQLMRK | | | | | Mascot |
| 1700.8346 | 1700.7043 | -0.1303 | -77 | 161 | 176 | TVKAAADAFTAMLCSK | | | Carbamidomethyl (C)[14], Oxidation (M)[12] | | Mascot |
| 1700.9153 | 1700.7043 | -0.211 | -124 | 143 | 157 | GLDVVFRLMSPHTTK | | | | | Mascot |
| 1716.9102 | 1716.6989 | -0.2113 | -123 | 143 | 157 | GLDVVFRLMSPHTTK | | | Oxidation (M)[9] | | Mascot |
| 1716.9102 | 1716.6989 | -0.2113 | -123 | 143 | 157 | GLDVVFRLMSPHTTK | | | Oxidation (M)[9] | | Mascot |
| 1819.7958 | 1819.7543 | -0.0415 | -23 | 164 | 180 | AAADAFTAMLCSKMNCR | | | Oxidation (M)[9] | | Mascot |
| 1933.8387 | 1933.9635 | 0.1248 | 65 | 164 | 180 | AAADAFTAMLCSKMNCR | | | Carbamidomethyl (C)[11,16], Oxidation (M)[9] | | Mascot |

| | | | | | | | |
|---|---|---|---|---|---|---|---|
| 22 | PREDICTED: protein C20orf11 homolog isoform 1 [Danio rerio] | gi\|189535879 | 26855.6 | 4.85 | 7 | 34 | 0 |

Peptide Information

| Calc. Mass | Obsrv. Mass | ± da | ± ppm | Start Seq. | End Seq. | Sequence | Ion Score | C. I. % | Modification | Rank | Result Type |
|---|---|---|---|---|---|---|---|---|---|---|---|
| 850.4417 | 850.5214 | 0.0797 | 94 | 45 | 51 | EAAEKFR | | | | | Mascot |
| 1434.7145 | 1434.7368 | 0.0223 | 16 | 217 | 229 | MTDLSKGTIEDPK | | | | | Mascot |
| 1470.7952 | 1470.6367 | -0.1585 | -108 | 199 | 210 | LLLWAQNELDQK | | | | | Mascot |
| 1570.724 | 1570.736 | 0.012 | 8 | 1 | 13 | MMSYAEKPEDITR | | | | | Mascot |
| 1598.89 | 1598.7292 | -0.1608 | -101 | 199 | 211 | LLLWAQNELDQKK | | | | | Mascot |
| 2149.033 | 2148.9441 | -0.0889 | -41 | 52 | 70 | MESGIEPSVDLDSLDERIK | | | Oxidation (M)[1] | | Mascot |
| 2211.0234 | 2211.0491 | 0.0257 | 12 | 50 | 68 | FRMESGIEPSVDLDSLDER | | | Oxidation (M)[3] | | Mascot |

| | | | | | | | | | | |
|---|---|---|---|---|---|---|---|---|---|---|
| 23 | PREDICTED: dystonin [Danio rerio] | gi\|326672751 | 766703.7 | 5.12 | 35 | 34 | 0 | 16 | 0 | |

**Peptide Information**

| Calc. Mass | Obsrv. Mass | ± da | ± ppm | Start Seq. | End Seq. | Sequence | Ion Score | C. I. % | Modification | Rank | Result Type |
|---|---|---|---|---|---|---|---|---|---|---|---|
| 829.4413 | 829.5081 | 0.0668 | 81 | 6329 | 6335 | KQPDVDK | | | | | Mascot |
| 842.5168 | 842.4906 | -0.0262 | -31 | 1683 | 1689 | LCLLPVK | | | Carbamidomethyl (C)[2] | | Mascot |
| 850.4087 | 850.5214 | 0.1127 | 133 | 3487 | 3493 | MEALSQR | | | Oxidation (M)[1] | | Mascot |
| 907.4189 | 907.4562 | 0.0373 | 41 | 4813 | 4820 | TLGCVDDK | 2 | 0 | Carbamidomethyl (C)[4] | | Mascot |
| 907.4731 | 907.4562 | -0.0169 | -19 | 542 | 549 | TLTSEQTK | | | | | Mascot |
| 939.5006 | 939.4149 | -0.0857 | -91 | 849 | 856 | SLQDAIHR | | | | | Mascot |
| 939.5006 | 939.4149 | -0.0857 | -91 | 849 | 856 | SLQDAIHR | 13 | 0 | | | Mascot |
| 940.4482 | 940.4044 | -0.0438 | -47 | 3787 | 3795 | HPSGNDSVK | | | | | Mascot |
| 996.5108 | 996.5735 | 0.0627 | 63 | 3265 | 3273 | LDSAGSRYK | | | | | Mascot |
| 1011.4741 | 1011.454 | -0.0201 | -20 | 5260 | 5267 | EKYETADR | | | | | Mascot |
| 1041.5826 | 1041.6078 | 0.0252 | 24 | 2558 | 2566 | DPDILLDLK | | | | | Mascot |
| 1051.6047 | 1051.6881 | 0.0834 | 79 | 97 | 104 | WINQHLLK | | | | | Mascot |
| 1140.6047 | 1140.5654 | -0.0393 | -34 | 351 | 360 | HFPSSPVELK | | | | | Mascot |
| 1234.7629 | 1234.6483 | -0.1146 | -93 | 5570 | 5580 | LNHQAGLLLKK | | | | | Mascot |
| 1248.5637 | 1248.5758 | 0.0121 | 10 | 446 | 456 | VQRDCAAGEDK | | | Carbamidomethyl (C)[5] | | Mascot |
| 1434.7271 | 1434.7368 | 0.0097 | 7 | 4627 | 4639 | SAQLHEALLHCGR | | | | | Mascot |
| 1493.7338 | 1493.697 | -0.0368 | -25 | 4037 | 4049 | LKEAECICAELSK | | | Carbamidomethyl (C)[6] | | Mascot |
| 1598.7656 | 1598.7292 | -0.0364 | -23 | 310 | 325 | VPEGGDGINANDVDVK | | | | | Mascot |
| 1652.8499 | 1652.7042 | -0.1457 | -88 | 1400 | 1413 | LLNWMSSMKTNLSK | | | | | Mascot |
| 1683.8661 | 1683.7931 | -0.073 | -43 | 163 | 177 | LVNIRNDDITDGNPK | | | | | Mascot |
| 1683.8661 | 1683.7931 | -0.073 | -43 | 163 | 177 | LVNIRNDDITDGNPK | 3 | 0 | | | Mascot |
| 1700.8966 | 1700.7043 | -0.1923 | -113 | 4328 | 4343 | VLDVHSSLGVQAFSNK | | | | | Mascot |

| | | | | | | | | | | | |
|---|---|---|---|---|---|---|---|---|---|---|---|
| 1700.8966 | 1700.7043 | -0.1923 | -113 | 4328 | 4343 | VLDVHSSLGVQAFSNK | 0 | 0 | | | Mascot |
| 1711.865 | 1711.7498 | -0.1152 | -67 | 4967 | 4980 | LQSSHEELNVWLEK | | | | | Mascot |
| 1819.9436 | 1819.7543 | -0.1893 | -104 | 3656 | 3671 | TDLKTVNDLLDAWTSK | | | | | Mascot |
| 1828.9916 | 1828.7953 | -0.1963 | -107 | 4327 | 4343 | KVLDVHSSLGVQAFSNK | | | | | Mascot |
| 1844.98 | 1844.792 | -0.188 | -102 | 5225 | 5239 | QMRELIAEHKPHIDK | | | | | Mascot |
| 1887.991 | 1887.9225 | -0.0685 | -36 | 6269 | 6285 | LSSALDELQATQELLEK | | | | | Mascot |
| 1933.9647 | 1933.9635 | -0.0012 | -1 | 4019 | 4034 | QIQATNEMLSQLREEK | | | Oxidation (M)[8] | | Mascot |
| 1940.9688 | 1940.9021 | -0.0667 | -34 | 3838 | 3853 | WKHMLAQIDAGWEEVK | | | | | Mascot |
| 2144.9214 | 2145.0481 | 0.1267 | 59 | 6646 | 6666 | SSDSFDSQGSSNESTPIQ GSK | | | | | Mascot |
| 2155.928 | 2156.1914 | 0.2634 | 122 | 3034 | 3052 | EMGGMQKENGNMPEFT PMK | | | | | Mascot |
| 2168.1604 | 2167.981 | -0.1794 | -83 | 428 | 445 | SLRPEVDRLEMLQQIANR | | | | | Mascot |
| 2186.0723 | 2185.9915 | -0.0808 | -37 | 2957 | 2974 | TVGDLQQYQQEHQLEEL K | | | | | Mascot |
| 2186.0723 | 2185.9915 | -0.0808 | -37 | 2957 | 2974 | TVGDLQQYQQEHQLEEL K | | | | | Mascot |
| 2211.1301 | 2211.0491 | -0.081 | -37 | 6571 | 6590 | STVMVRVGGGWMALDE FLVK | | | Oxidation (M)[4] | | Mascot |
| 2225.1548 | 2225.0632 | -0.0916 | -41 | 5419 | 5438 | DTQELLKELEEPGVDPSV VK | | | | | Mascot |
| 2225.1548 | 2225.0632 | -0.0916 | -41 | 5419 | 5438 | DTQELLKELEEPGVDPSV VK | | | | | Mascot |
| 2346.0952 | 2346.2458 | 0.1506 | 64 | 2505 | 2524 | SYLNELIQMKMDGASLDE TR | | | Oxidation (M)[9,11] | | Mascot |
| 3308.5596 | 3308.3765 | -0.1831 | -55 | 950 | 980 | VISTSGNEAMVPSVCFSV PPPNKEATEMASR | | | Carbamidomethyl (C)[15], Oxidation (M)[10] | | Mascot |

24 nucleolar MIF4G domain-containing protein 1 [Danio rerio] gi|148540048 55892.4 8.35 9 34 0

**Peptide Information**

| Calc. Mass | Obsrv. Mass | ± da | ± ppm | Start Seq. | End Seq. | Sequence | Ion Score | C. I. % | Modification | Rank | Result Type |
|---|---|---|---|---|---|---|---|---|---|---|---|
| 868.4774 | 868.4709 | -0.0065 | -7 | 115 | 122 | LVTSFSSK | | | | | Mascot |
| 907.4414 | 907.4562 | 0.0148 | 16 | 277 | 283 | MNTDVRR | | | Oxidation (M)[1] | | Mascot |
| 907.4414 | 907.4562 | 0.0148 | 16 | 277 | 283 | MNTDVRR | | | Oxidation (M)[1] | | Mascot |
| 940.5137 | 940.4044 | -0.1093 | -116 | 332 | 339 | FYAVLAEK | | | | | Mascot |
| 996.5724 | 996.5735 | 0.0011 | 1 | 114 | 122 | KLVTSFSSK | | | | | Mascot |
| 1140.5419 | 1140.5654 | 0.0235 | 21 | 76 | 85 | IYSELDGTDK | | | | | Mascot |
| 1570.8547 | 1570.736 | -0.1187 | -76 | 220 | 233 | VSLDNLLEAERVGR | | | | | Mascot |
| 1716.8118 | 1716.6989 | -0.1129 | -66 | 314 | 327 | EIVHVLMDCCLQEK | | | Carbamidomethyl (C)[9] | | Mascot |
| 1716.8118 | 1716.6989 | -0.1129 | -66 | 314 | 327 | EIVHVLMDCCLQEK | | | Carbamidomethyl (C)[9] | | Mascot |
| 1828.8745 | 1828.7953 | -0.0792 | -43 | 2 | 17 | ASISSQLEELYMNTSR | | | | | Mascot |
| 1844.8527 | 1844.792 | -0.0607 | -33 | 172 | 186 | FMLETMMALKNNDMR | | | | | Mascot |

25 PREDICTED: zinc finger protein 227-like [Danio rerio] gi|125847909 86097.2 8.77 14 33 0

**Peptide Information**

| Calc. Mass | Obsrv. Mass | ± da | ± ppm | Start Seq. | End Seq. | Sequence | Ion Score | C. I. % | Modification | Rank | Result Type |
|---|---|---|---|---|---|---|---|---|---|---|---|
| 850.4127 | 850.5214 | 0.1087 | 128 | 181 | 187 | QEPFVCK | | | | | Mascot |
| 882.4944 | 882.5466 | 0.0522 | 59 | 352 | 358 | AHVKHYK | | | | | Mascot |
| 907.4341 | 907.4562 | 0.0221 | 24 | 181 | 187 | QEPFVCK | 1 | 0 | Carbamidomethyl (C)[6] | | Mascot |
| 907.4341 | 907.4562 | 0.0221 | 24 | 181 | 187 | QEPFVCK | | | Carbamidomethyl (C)[6] | | Mascot |
| 1140.4958 | 1140.5654 | 0.0696 | 61 | 608 | 617 | RDCVCSICNK | | | | | Mascot |
| 1165.6221 | 1165.7177 | 0.0956 | 82 | 552 | 561 | LYGCPICLKR | | | | | Mascot |
| 1234.5092 | 1234.6483 | 0.1391 | 113 | 356 | 364 | HYKCSHCDK | | | Carbamidomethyl (C)[4,7] | | Mascot |
| 1470.811 | 1470.6367 | -0.1743 | -119 | 510 | 521 | KHLQAHMLVHTR | | | | | Mascot |
| 1475.6406 | 1475.7256 | 0.085 | 58 | 686 | 697 | FGYSSSIQMHMR | | | Oxidation (M)[9,11] | | Mascot |
| 1599.8536 | 1599.8459 | -0.0077 | -5 | 511 | 523 | HLQAHMLVHTREK | | | | | Mascot |
| 1707.8304 | 1707.7374 | -0.093 | -54 | 412 | 426 | TVTCPNCQKVFPGTR | | | Carbamidomethyl (C)[4] | | Mascot |
| 1716.8923 | 1716.6989 | -0.1934 | -113 | 545 | 560 | AGHTGVKLYGCPICLK | | | Carbamidomethyl (C)[11] | | Mascot |
| 1716.8923 | 1716.6989 | -0.1934 | -113 | 545 | 560 | AGHTGVKLYGCPICLK | | | Carbamidomethyl (C)[11] | | Mascot |
| 1911.0017 | 1910.8079 | -0.1938 | -101 | 370 | 386 | SCLIRHAATHTGAQLFK | | | Carbamidomethyl (C)[2] | | Mascot |
| 1911.0017 | 1910.8079 | -0.1938 | -101 | 370 | 386 | SCLIRHAATHTGAQLFK | | | Carbamidomethyl (C)[2] | | Mascot |
| 2145.0969 | 2145.0481 | -0.0488 | -23 | 62 | 80 | EFAAFMELLSKATVETMVK | | | | | Mascot |
| 2156.0957 | 2156.1914 | 0.0957 | 44 | 618 | 636 | AFIFPSSLNQHMLVHTGEK | | | | | Mascot |
| 3308.5317 | 3308.3765 | -0.1552 | -47 | 609 | 636 | DCVCSICNKAFIFPSSLNQHMLVHTGEK | | | Carbamidomethyl (C)[2,4,7], Oxidation (M)[21] | | Mascot |

26 LOC100001129 protein [Danio rerio] gi|160774064 68472.7 6.3 11 33 0

**Peptide Information**

| Calc. Mass | Obsrv. Mass | ± da | ± ppm | Start Seq. | End Seq. | Sequence | Ion Score | C. I. % | Modification | Rank | Result Type |
|---|---|---|---|---|---|---|---|---|---|---|---|
| 868.529 | 868.4709 | -0.0581 | -67 | 292 | 298 | FLKFVSK | | | | | Mascot |
| 1041.5364 | 1041.6078 | 0.0714 | 69 | 123 | 130 | DRFESFLK | | | | | Mascot |
| 1045.5459 | 1045.5391 | -0.0068 | -7 | 280 | 288 | LQMADQIAR | | | | | Mascot |
| 1179.6343 | 1179.5751 | -0.0592 | -50 | 578 | 586 | ILWVQAMYR | | | | | Mascot |
| 1350.6504 | 1350.6696 | 0.0192 | 14 | 161 | 173 | MVQTGGLSALDCR | | | | | Mascot |
| 1910.9463 | 1910.8079 | -0.1384 | -72 | 161 | 177 | MVQTGGLSALDCREVFK | | | Carbamidomethyl (C)[12] | | Mascot |
| 1910.9463 | 1910.8079 | -0.1384 | -72 | 161 | 177 | MVQTGGLSALDCREVFK | | | Carbamidomethyl (C)[12] | | Mascot |
| 2017.0825 | 2016.9615 | -0.121 | -60 | 587 | 605 | ATGQSHKPVPPTQVQKLNS | | | | | Mascot |

| Calc. Mass | Obsrv. Mass | ± da | ± ppm | Start Seq. | End Seq. | Sequence | Ion Score | C. I. % | Modification | Rank | Result Type |
|---|---|---|---|---|---|---|---|---|---|---|---|
| 2168.0474 | 2167.981 | -0.0664 | -31 | 377 | 395 | SLAPNRIVYCTMEVEGGQK | | | Carbamidomethyl (C)[10], Oxidation (M)[12] | | Mascot |
| 2224.9397 | 2225.0632 | 0.1235 | 56 | 1 | 20 | MLDPSSSEEESDGIVEEESR | | | | | Mascot |
| 2224.9397 | 2225.0632 | 0.1235 | 56 | 1 | 20 | MLDPSSSEEESDGIVEEESR | | | | | Mascot |
| 2233.166 | 2233.0325 | -0.1335 | -60 | 84 | 101 | LQLYVFVMRCIAYPFNAK | | | Carbamidomethyl (C)[10] | | Mascot |
| 2346.1055 | 2346.2458 | 0.1403 | 60 | 50 | 72 | GSSARPTSPSPSAASEEKEDVEK | | | | | Mascot |

27 novel protein similar to vertebrate N-glycanase 1 (NGLY1) [Danio rerio] gi|126632448 72396.7 6 8 33 0

**Peptide Information**

| Calc. Mass | Obsrv. Mass | ± da | ± ppm | Start Seq. | End Seq. | Sequence | Ion Score | C. I. % | Modification | Rank | Result Type |
|---|---|---|---|---|---|---|---|---|---|---|---|
| 907.4454 | 907.4562 | 0.0108 | 12 | 444 | 451 | MSGSLAWR | 14 | 0 | | | Mascot |
| 907.4454 | 907.4562 | 0.0108 | 12 | 444 | 451 | MSGSLAWR | | | | | Mascot |
| 1011.4386 | 1011.454 | 0.0154 | 15 | 545 | 553 | FNCAPAGMK | | | Carbamidomethyl (C)[3], Oxidation (M)[8] | | Mascot |
| 1041.5979 | 1041.6078 | 0.0099 | 10 | 358 | 366 | LSYILAFSK | | | | | Mascot |
| 1320.7271 | 1320.5513 | -0.1758 | -133 | 43 | 54 | SIRIGNPTFSTK | | | | | Mascot |
| 1350.7198 | 1350.6696 | -0.0502 | -37 | 180 | 190 | ALECIPHELLR | | | Carbamidomethyl (C)[4] | | Mascot |
| 1470.6691 | 1470.6367 | -0.0324 | -22 | 541 | 553 | MSWKFNCAPAGMK | | | | | Mascot |
| 1570.7318 | 1570.736 | 0.0042 | 3 | 367 | 379 | DQVADVTWRYSCK | | | | | Mascot |
| 2283.083 | 2283.1204 | 0.0374 | 16 | 294 | 313 | CGEWANCFTLLCRALGLEAR | | | Carbamidomethyl (C)[1] | | Mascot |

28 novel protein similar to vertebrate cytoplasmic linker associated protein 1 (CLASP1) [Danio rerio] gi|159569348 164674.4 8.99 17 33 0

**Peptide Information**

| Calc. Mass | Obsrv. Mass | ± da | ± ppm | Start Seq. | End Seq. | Sequence | Ion Score | C. I. % | Modification | Rank | Result Type |
|---|---|---|---|---|---|---|---|---|---|---|---|
| 829.5002 | 829.5081 | 0.0079 | 10 | 719 | 726 | LGIGRGTR | | | | | Mascot |
| 1165.6801 | 1165.7177 | 0.0376 | 32 | 1313 | 1322 | DHTIRALALR | | | | | Mascot |
| 1434.6715 | 1434.7368 | 0.0653 | 46 | 1240 | 1251 | QDCGENKMVLPK | | | Carbamidomethyl (C)[3], Oxidation (M)[8] | | Mascot |
| 1470.6715 | 1470.6367 | -0.0348 | -24 | 185 | 196 | DAAMNCLVEIYR | | | Carbamidomethyl (C)[6], Oxidation (M)[4] | | Mascot |
| 1475.6802 | 1475.7256 | 0.0454 | 31 | 1288 | 1299 | EDSLAVWDEHFK | | | | | Mascot |
| 1570.9462 | 1570.736 | -0.2102 | -134 | 421 | 435 | VMATSGVAAIRLILR | | | | | Mascot |
| 1598.6752 | 1598.7292 | 0.054 | 34 | 989 | 1002 | QMDPGDFVNSSETR | | | Oxidation (M)[2] | | Mascot |
| 1683.7649 | 1683.7931 | 0.0282 | 17 | 266 | 282 | GGSMGSMRRPSSASGSR | | | Oxidation (M)[4] | | Mascot |
| 1683.7649 | 1683.7931 | 0.0282 | 17 | 266 | 282 | GGSMGSMRRPSSASGSR | | | Oxidation (M)[4] | | Mascot |
| 1700.9078 | 1700.7043 | -0.2035 | -120 | 1186 | 1201 | TSLLNTPSPRSFAGPR | | | | | Mascot |

| Calc. Mass | Obsrv. Mass | ± da | ± ppm | Start Seq. | End Seq. | Sequence | Ion Score | C. I. % | Modification | Rank | Result Type |
|---|---|---|---|---|---|---|---|---|---|---|---|
| 1700.9078 | 1700.7043 | -0.2035 | -120 | 1186 | 1201 | TSLLNTPSPRSFAGPR | | | | | Mascot |
| 1711.803 | 1711.7498 | -0.0532 | -31 | 2 | 15 | EPNMEYCLTQVLQK | | | Oxidation (M)[4] | | Mascot |
| 1727.9287 | 1727.7474 | -0.1813 | -105 | 1180 | 1195 | TALDNKTSLLNTPSPR | | | | | Mascot |
| 2017.0171 | 2016.9615 | -0.0556 | -28 | 347 | 364 | SLLMAGATEHEGFIQQLR | | | Oxidation (M)[4] | | Mascot |
| 2168.1306 | 2167.981 | -0.1496 | -69 | 532 | 551 | ALQSHLKSSDSIVSLPQSDR | | | | | Mascot |
| 2225.207 | 2225.0632 | -0.1438 | -65 | 379 | 399 | SQVVREACITLGHLSSLLGNK | | | | | Mascot |
| 2225.207 | 2225.0632 | -0.1438 | -65 | 379 | 399 | SQVVREACITLGHLSSLLGNK | | | | | Mascot |
| 2284.0769 | 2284.1143 | 0.0374 | 16 | 1 | 19 | MEPNMEYCLTQVLQKDVAR | | | Oxidation (M)[1] | | Mascot |
| 2403.0994 | 2402.9583 | -0.1411 | -59 | 835 | 855 | QTEDVAEVLNHCASSNWSERK | | | | | Mascot |
| 2498.2629 | 2498.2664 | 0.0035 | 1 | 145 | 167 | EGVCLCLISTLNTYGAQGLTLSK | | | Carbamidomethyl (C)[4,6] | | Mascot |

29 Pbx2 [Danio rerio] gi|7861936 18895.6 9.08 6 33 0

**Peptide Information**

| Calc. Mass | Obsrv. Mass | ± da | ± ppm | Start Seq. | End Seq. | Sequence | Ion Score | C. I. % | Modification | Rank | Result Type |
|---|---|---|---|---|---|---|---|---|---|---|---|
| 1051.5782 | 1051.6881 | 0.1099 | 105 | 133 | 141 | FSSIQTQLK | | | | | Mascot |
| 1179.6442 | 1179.5751 | -0.0691 | -59 | 1 | 10 | ALFSVLCEIK | | | Carbamidomethyl (C)[7] | | Mascot |
| 1493.7563 | 1493.697 | -0.0593 | -40 | 142 | 154 | QSTCEAVMILRSR | | | | | Mascot |
| 1997.9055 | 1997.839 | -0.0665 | -33 | 93 | 109 | YEQACSESTTHVMNLLR | | | Oxidation (M)[13] | | Mascot |
| 1997.9055 | 1997.839 | -0.0665 | -33 | 93 | 109 | YEQACSESTTHVMNLLR | | | Oxidation (M)[13] | | Mascot |
| 2038.9321 | 2038.8834 | -0.0487 | -24 | 93 | 109 | YEQACSESTTHVMNLLR | | | Carbamidomethyl (C)[5] | | Mascot |
| 2283.1836 | 2283.1204 | -0.0632 | -28 | 133 | 152 | FSSIQTQLKQSTCEAVMILR | | | | | Mascot |
| 2498.1399 | 2498.2664 | 0.1265 | 51 | 93 | 113 | YEQACSESTTHVMNLLREQSR | | | Oxidation (M)[13] | | Mascot |

30 peptide-N(4)-(N-acetyl-beta-glucosaminyl)asparagine amidase [Danio rerio] gi|66472548 72442.7 6 8 33 0

**Peptide Information**

| Calc. Mass | Obsrv. Mass | ± da | ± ppm | Start Seq. | End Seq. | Sequence | Ion Score | C. I. % | Modification | Rank | Result Type |
|---|---|---|---|---|---|---|---|---|---|---|---|
| 907.4454 | 907.4562 | 0.0108 | 12 | 444 | 451 | MSGSLAWR | 14 | 0 | | | Mascot |
| 907.4454 | 907.4562 | 0.0108 | 12 | 444 | 451 | MSGSLAWR | | | | | Mascot |
| 1011.4386 | 1011.454 | 0.0154 | 15 | 545 | 553 | FNCAPAGMK | | | Carbamidomethyl (C)[3], Oxidation (M)[8] | | Mascot |
| 1041.5979 | 1041.6078 | 0.0099 | 10 | 358 | 366 | LSYILAFSK | | | | | Mascot |
| 1320.7271 | 1320.5513 | -0.1758 | -133 | 43 | 54 | SIRIGNPTFSTK | | | | | Mascot |
| 1350.7198 | 1350.6696 | -0.0502 | -37 | 180 | 190 | ALECIPHELLR | | | Carbamidomethyl (C)[4] | | Mascot |
| 1470.6691 | 1470.6367 | -0.0324 | -22 | 541 | 553 | MSWKFNCAPAGMK | | | | | Mascot |

| | | | | | | | | | | | | |
|---|---|---|---|---|---|---|---|---|---|---|---|---|
| 1570.7318 | 1570.736 | 0.0042 | 3 | 367 | 379 | DQVADVTWRYSCK | | | | | Mascot |
| 2283.083 | 2283.1204 | 0.0374 | 16 | 294 | 313 | CGEWANCFTLLCRALGL EAR | | | Carbamidomethyl (C)[1] | | Mascot |

31 adenosine deaminase [Danio rerio] gi|50540360 41148.6 6.04 9 32 0

Peptide Information

| Calc. Mass | Obsrv. Mass | ± da | ± ppm | Start Seq. | End Seq. | Sequence | Ion Score | C. I. % | Modification | Rank | Result Type |
|---|---|---|---|---|---|---|---|---|---|---|---|
| 939.4208 | 939.4149 | -0.0059 | -6 | 153 | 159 | SILCCMR | | | Carbamidomethyl (C)[4,5] | | Mascot |
| 939.4208 | 939.4149 | -0.0059 | -6 | 153 | 159 | SILCCMR | | | Carbamidomethyl (C)[4,5] | | Mascot |
| 1068.5111 | 1068.5441 | 0.033 | 31 | 151 | 159 | ARSILCCMR | | | Oxidation (M)[8] | | Mascot |
| 1234.679 | 1234.6483 | -0.0307 | -25 | 94 | 104 | AKEGVIYVEAR | | | | | Mascot |
| 1470.741 | 1470.6367 | -0.1043 | -71 | 346 | 357 | LLNQLYEAYGMR | | | | | Mascot |
| 1598.8359 | 1598.7292 | -0.1067 | -67 | 346 | 358 | LLNQLYEAYGMRK | | | | | Mascot |
| 1599.9329 | 1599.8459 | -0.087 | -54 | 15 | 28 | VELHVHLDGAIRLK | | | | | Mascot |
| 1727.8785 | 1727.7474 | -0.1311 | -76 | 344 | 357 | EKLLNQLYEAYGMR | | | | | Mascot |
| 1887.8914 | 1887.9225 | 0.0311 | 16 | 160 | 174 | HMPNWSMEVVELCKK | | | Carbamidomethyl (C)[13] | | Mascot |
| 3308.4956 | 3308.3765 | -0.1191 | -36 | 253 | 280 | QLLHQNMHFEMCPVSSR LTGACEPDFTK | | | Carbamidomethyl (C)[12], Oxidation (M)[7,11] | | Mascot |

32 novel protein similar to vertebrate microtubule-actin crosslinking factor 1 (MACF1) [Danio rerio] gi|94732207 181610.2 5.27 16 32 0 3 0

Peptide Information

| Calc. Mass | Obsrv. Mass | ± da | ± ppm | Start Seq. | End Seq. | Sequence | Ion Score | C. I. % | Modification | Rank | Result Type |
|---|---|---|---|---|---|---|---|---|---|---|---|
| 885.377 | 885.4941 | 0.1171 | 132 | 803 | 809 | DCESYLR | | | | | Mascot |
| 939.4716 | 939.4149 | -0.0567 | -60 | 522 | 529 | LQCASVYR | | | | | Mascot |
| 939.4716 | 939.4149 | -0.0567 | -60 | 522 | 529 | LQCASVYR | | | | | Mascot |
| 996.493 | 996.5735 | 0.0805 | 81 | 522 | 529 | LQCASVYR | | | Carbamidomethyl (C)[3] | | Mascot |
| 1011.4854 | 1011.454 | -0.0314 | -31 | 644 | 651 | EARTYESR | | | | | Mascot |
| 1068.4891 | 1068.5441 | 0.055 | 51 | 725 | 732 | REQYADMR | | | | | Mascot |
| 1140.4659 | 1140.5654 | 0.0995 | 87 | 1337 | 1345 | LDECQTCSK | | | Carbamidomethyl (C)[4,7] | | Mascot |
| 1234.7267 | 1234.6483 | -0.0784 | -63 | 487 | 496 | QLHLDLQVLR | | | | | Mascot |
| 1248.5637 | 1248.5758 | 0.0121 | 10 | 1023 | 1032 | ELEREAENCR | | | | | Mascot |
| 1320.61 | 1320.5513 | -0.0587 | -44 | 437 | 447 | IQNTALDCEEK | | | Carbamidomethyl (C)[8] | | Mascot |
| 1470.7621 | 1470.6367 | -0.1254 | -85 | 1480 | 1492 | STQVFLMSKQASK | | | Oxidation (M)[7] | | Mascot |
| 1652.8677 | 1652.7042 | -0.1635 | -99 | 881 | 894 | AICDYRQIEITISK | | | | | Mascot |
| 1683.8661 | 1683.7931 | -0.073 | -43 | 154 | 168 | LVNIRNDDITDGNPK | | | | | Mascot |
| 1683.8661 | 1683.7931 | -0.073 | -43 | 154 | 168 | LVNIRNDDITDGNPK | 3 | 0 | | | Mascot |

| Calc. Mass | Obsrv. Mass | ± da | ± ppm | Start Seq. | End Seq. | Sequence | Ion Score | C. I. % | Modification | Rank | Result Type |
|---|---|---|---|---|---|---|---|---|---|---|---|
| 1711.832 | 1711.7498 | -0.0822 | -48 | 803 | 816 | DCESYLRQLQDTIK | | | | | Mascot |
| 1828.8606 | 1828.7953 | -0.0653 | -36 | 22 | 40 | SERSGGSLSPCAHSTAGPK | | | | | Mascot |
| 1887.8575 | 1887.9225 | 0.065 | 34 | 1337 | 1352 | LDECQTCSKQYSAAVK | | | Carbamidomethyl (C)[4,7] | | Mascot |
| 2168.1167 | 2167.981 | -0.1357 | -63 | 1119 | 1137 | RCVSFFEEKPTSSSVPVLR | | | | | Mascot |
| 2225.1382 | 2225.0632 | -0.075 | -34 | 1119 | 1137 | RCVSFFEEKPTSSSVPVLR | | | Carbamidomethyl (C)[2] | | Mascot |
| 2225.1382 | 2225.0632 | -0.075 | -34 | 1119 | 1137 | RCVSFFEEKPTSSSVPVLR | | | Carbamidomethyl (C)[2] | | Mascot |
| 2381.1541 | 2380.978 | -0.1761 | -74 | 830 | 848 | LEDLLQDSMEEKEQLIEYR | | | | | Mascot |
| 2381.1541 | 2380.978 | -0.1761 | -74 | 830 | 848 | LEDLLQDSMEEKEQLIEYR | | | | | Mascot |

33 Tnfrsfa protein [Danio rerio] gi|190336943 48606.4 5.31 9 32 0

**Peptide Information**

| Calc. Mass | Obsrv. Mass | ± da | ± ppm | Start Seq. | End Seq. | Sequence | Ion Score | C. I. % | Modification | Rank | Result Type |
|---|---|---|---|---|---|---|---|---|---|---|---|
| 939.4274 | 939.4149 | -0.0125 | -13 | 1 | 8 | MDKSEAMK | | | | | Mascot |
| 939.4274 | 939.4149 | -0.0125 | -13 | 1 | 8 | MDKSEAMK | | | | | Mascot |
| 1011.5158 | 1011.454 | -0.0618 | -61 | 360 | 366 | YHNKFFR | | | | | Mascot |
| 1041.4669 | 1041.6078 | 0.1409 | 135 | 59 | 67 | NCEAGTYVK | | | Carbamidomethyl (C)[2] | | Mascot |
| 1475.6973 | 1475.7256 | 0.0283 | 19 | 214 | 226 | QQSETNGNLEEVK | | | | | Mascot |
| 1727.762 | 1727.7474 | -0.0146 | -8 | 321 | 334 | DQTESWPREDDPPR | | | | | Mascot |
| 2016.8927 | 2016.9615 | 0.0688 | 34 | 227 | 243 | VPIDECPRSEEEQENSR | | | | | Mascot |
| 2211.0537 | 2211.0491 | -0.0046 | -2 | 310 | 328 | SSPPAQRPTDRDQTESWPR | | | | | Mascot |
| 2283.9038 | 2284.1143 | 0.2105 | 92 | 41 | 58 | QMTCLENHEYPHNGFCCK | | | Carbamidomethyl (C)[4,16], Oxidation (M)[2] | | Mascot |
| 2498.0469 | 2498.2664 | 0.2195 | 88 | 38 | 58 | TARQMTCLENHEYPHNGFCCK | | | Oxidation (M)[5] | | Mascot |

34 MHC class II antigen [Danio rerio] gi|297179855 8402.9 5.07 4 32 0

**Peptide Information**

| Calc. Mass | Obsrv. Mass | ± da | ± ppm | Start Seq. | End Seq. | Sequence | Ion Score | C. I. % | Modification | Rank | Result Type |
|---|---|---|---|---|---|---|---|---|---|---|---|
| 940.4193 | 940.4044 | -0.0149 | -16 | 57 | 64 | AEVDTFCR | | | | | Mascot |
| 1041.4749 | 1041.6078 | 0.1329 | 128 | 65 | 73 | HNAQIWDSA | | | | | Mascot |
| 1068.5143 | 1068.5441 | 0.0298 | 28 | 56 | 64 | KAEVDTFCR | | | | | Mascot |
| 2019.8977 | 2019.8184 | -0.0793 | -39 | 57 | 73 | AEVDTFCRHNAQIWDSA | | | Carbamidomethyl (C)[7] | | Mascot |

35 Synbl protein [Danio rerio] gi|141796272 31226.2 5.37 7 32 0

**Peptide Information**

| Calc. Mass | Obsrv. Mass | ± da | ± ppm | Start Seq. | End Seq. | Sequence | Ion Score | C. I. % | Modification | Rank | Result Type |
|---|---|---|---|---|---|---|---|---|---|---|---|
| 842.5709 | 842.4906 | -0.0803 | -95 | 18 | 24 | VEKLLLK | | | | | Mascot |
| 868.4999 | 868.4709 | -0.029 | -33 | 251 | 257 | DSLHKLR | | | | | Mascot |
| 1045.6769 | 1045.5391 | -0.1378 | -132 | 171 | 179 | LLFLLSALR | | | | | Mascot |
| 1350.7375 | 1350.6696 | -0.0679 | -50 | 21 | 31 | LLLKYNTENSR | | | | | Mascot |
| 1470.6603 | 1470.6367 | -0.0236 | -16 | 238 | 250 | ALFNLTMSDPCDK | | | Oxidation (M)[7] | | Mascot |
| 1838.8688 | 1838.8722 | 0.0034 | 2 | 1 | 17 | MSLSDILSEIDGGDDKK | | | Oxidation (M)[1] | | Mascot |
| 2283.0193 | 2283.1204 | 0.1011 | 44 | 98 | 118 | ICVAGHEGEPLEEAQSAE EER | | | | | Mascot |

| | | | | | | | | |
|---|---|---|---|---|---|---|---|---|
| 36 | PREDICTED: hypothetical protein LOC100002065 [Danio rerio] | gi\|189530999 | 48002.4 | 8.91 | 8 | 32 | 0 | |

Peptide Information

| Calc. Mass | Obsrv. Mass | ± da | ± ppm | Start Seq. | End Seq. | Sequence | Ion Score | C. I. % | Modification | Rank | Result Type |
|---|---|---|---|---|---|---|---|---|---|---|---|
| 1011.5945 | 1011.454 | -0.1405 | -139 | 179 | 187 | TALHSLLTR | | | | | Mascot |
| 1179.7208 | 1179.5751 | -0.1457 | -124 | 188 | 197 | LNNLKHLSLK | | | | | Mascot |
| 1320.6876 | 1320.5513 | -0.1363 | -103 | 260 | 271 | CIFCPLACPLIK | | | | | Mascot |
| 1434.7306 | 1434.7368 | 0.0062 | 4 | 260 | 271 | CIFCPLACPLIK | | | Carbamidomethyl (C)[1,4] | | Mascot |
| 1470.7303 | 1470.6367 | -0.0936 | -64 | 137 | 148 | NLTHLCLCGVQR | | | Carbamidomethyl (C)[6,8] | | Mascot |
| 1475.825 | 1475.7256 | -0.0994 | -67 | 304 | 317 | LSLMGVKADLASVR | | | Oxidation (M)[4] | | Mascot |
| 1716.8091 | 1716.6989 | -0.1102 | -64 | 114 | 126 | HLDRLVICQCSCR | | | Carbamidomethyl (C)[8,10,12] | | Mascot |
| 1716.8091 | 1716.6989 | -0.1102 | -64 | 114 | 126 | HLDRLVICQCSCR | | | Carbamidomethyl (C)[8,10,12] | | Mascot |
| 2149.137 | 2148.9441 | -0.1929 | -90 | 253 | 271 | LSVHYTKCIFCPLACPLIK | | | | | Mascot |
| 3308.5852 | 3308.3765 | -0.2087 | -63 | 272 | 299 | WLEELCVLSELNISYGYS LEMYAGSIPR | | | Carbamidomethyl (C)[6], Oxidation (M)[21] | | Mascot |

| | | | | | | | | |
|---|---|---|---|---|---|---|---|---|
| 37 | PREDICTED: disks large homolog 3 [Danio rerio] | gi\|326673729 | 89938 | 6.56 | 10 | 32 | 0 | |

Peptide Information

| Calc. Mass | Obsrv. Mass | ± da | ± ppm | Start Seq. | End Seq. | Sequence | Ion Score | C. I. % | Modification | Rank | Result Type |
|---|---|---|---|---|---|---|---|---|---|---|---|
| 907.4301 | 907.4562 | 0.0261 | 29 | 584 | 591 | TGMIESNR | 9 | 0 | | | Mascot |
| 907.4301 | 907.4562 | 0.0261 | 29 | 584 | 591 | TGMIESNR | | | | | Mascot |
| 1045.5459 | 1045.5391 | -0.0068 | -7 | 178 | 186 | LGVNDCVLR | | | Carbamidomethyl (C)[6] | | Mascot |
| 1234.5521 | 1234.6483 | 0.0962 | 78 | 373 | 382 | HMLGEEDFTR | | | | | Mascot |
| 1434.6907 | 1434.7368 | 0.0461 | 32 | 580 | 591 | FHARTGMIESNR | | | Oxidation (M)[7] | | Mascot |
| 1470.7223 | 1470.6367 | -0.0856 | -58 | 758 | 769 | QTYEQANKVFDK | | | | | Mascot |
| 1570.8019 | 1570.736 | -0.0659 | -42 | 652 | 664 | FGSCVPHTTRPRR | | | Carbamidomethyl (C)[4] | | Mascot |

| | | | | | | | | | | | |
|---|---|---|---|---|---|---|---|---|---|---|---|
| 1844.7448 | 1844.792 | 0.0472 | 26 | 4 | 18 | HHHCCKCPECYEVTR | | | | | Mascot |
| 1911.052 | 1910.8079 | -0.2441 | -128 | 622 | 637 | QEIHYTRPVIILGPMK | | | Oxidation (M)[15] | | Mascot |
| 1911.052 | 1910.8079 | -0.2441 | -128 | 622 | 637 | QEIHYTRPVIILGPMK | | | Oxidation (M)[15] | | Mascot |
| 1933.9225 | 1933.9635 | 0.041 | 21 | 367 | 382 | YSPVPRHMLGEEDFTR | | | | | Mascot |
| 2186.1743 | 2185.9915 | -0.1828 | -84 | 799 | 817 | LIIEEQSGPYIWIPSAEKL | | | | | Mascot |
| 2186.1743 | 2185.9915 | -0.1828 | -84 | 799 | 817 | LIIEEQSGPYIWIPSAEKL | | | | | Mascot |

| | | | | | | | | | | |
|---|---|---|---|---|---|---|---|---|---|---|
| 38 | Excision repair cross-complementing rodent repair deficiency, complementation group 8 [Danio rerio] | gi\|158253610 | 44643 | 6.49 | 5 | 31 | 0 | 19 | 0 | |

**Peptide Information**

| Calc. Mass | Obsrv. Mass | ± da | ± ppm | Start Seq. | End Seq. | Sequence | Ion Score | C. I. % | Modification | Rank | Result Type |
|---|---|---|---|---|---|---|---|---|---|---|---|
| 850.4642 | 850.5214 | 0.0572 | 67 | 93 | 99 | SSRHVHK | | | | | Mascot |
| 907.5359 | 907.4562 | -0.0797 | -88 | 295 | 302 | GLKFTVSR | 19 | 0 | | | Mascot |
| 907.5359 | 907.4562 | -0.0797 | -88 | 295 | 302 | GLKFTVSR | | | | | Mascot |
| 1350.76 | 1350.6696 | -0.0904 | -67 | 26 | 36 | RVLSLELNHNR | | | | | Mascot |
| 1728.0027 | 1727.7474 | -0.2553 | -148 | 179 | 193 | IHILQGHRGEILSVR | | | | | Mascot |
| 1998.0114 | 1997.839 | -0.1724 | -86 | 1 | 17 | MLSFLYARQTGLDDPVR | | | Oxidation (M)[1] | | Mascot |
| 1998.0114 | 1997.839 | -0.1724 | -86 | 1 | 17 | MLSFLYARQTGLDDPVR | | | Oxidation (M)[1] | | Mascot |

| | | | | | | | | |
|---|---|---|---|---|---|---|---|---|
| 39 | Protein phosphatase 4 (formerly X), catalytic subunit [Danio rerio] | gi\|29436930 | 35407.5 | 5.02 | 7 | 31 | 0 | |

**Peptide Information**

| Calc. Mass | Obsrv. Mass | ± da | ± ppm | Start Seq. | End Seq. | Sequence | Ion Score | C. I. % | Modification | Rank | Result Type |
|---|---|---|---|---|---|---|---|---|---|---|---|
| 939.4682 | 939.4149 | -0.0533 | -57 | 138 | 145 | YGSATVWR | | | | | Mascot |
| 939.4682 | 939.4149 | -0.0533 | -57 | 138 | 145 | YGSATVWR | | | | | Mascot |
| 1434.7991 | 1434.7368 | -0.0623 | -43 | 299 | 311 | GLPSKKPVADYFL | | | | | Mascot |
| 1716.7911 | 1716.6989 | -0.0922 | -54 | 123 | 136 | QITQVHGFFDECHR | | | | | Mascot |
| 1716.7911 | 1716.6989 | -0.0922 | -54 | 123 | 136 | QITQVHGFFDECHR | | | | | Mascot |
| 1844.886 | 1844.792 | -0.094 | -51 | 123 | 137 | QITQVHGFFDECHRK | | | | | Mascot |
| 2144.9956 | 2145.0481 | 0.0525 | 24 | 72 | 90 | VGGEVPETNYLFMGDFV DR | | | | | Mascot |
| 2168.0474 | 2167.981 | -0.0664 | -31 | 2 | 19 | CVIMGDFTDLDRQIEQLR | | | Oxidation (M)[4] | | Mascot |
| 2225.0688 | 2225.0632 | -0.0056 | -3 | 2 | 19 | CVIMGDFTDLDRQIEQLR | | | Carbamidomethyl (C)[1], Oxidation (M)[4] | | Mascot |
| 2225.0688 | 2225.0632 | -0.0056 | -3 | 2 | 19 | CVIMGDFTDLDRQIEQLR | | | Carbamidomethyl (C)[1], Oxidation (M)[4] | | Mascot |
| 2283.093 | 2283.1204 | 0.0274 | 12 | 1 | 19 | MCVIMGDFTDLDRQIEQL R | | | | | Mascot |

| | | | | | | | |
|---|---|---|---|---|---|---|---|
| 40 | PREDICTED: disks large homolog 3-like [Danio rerio] | gi\|326673702 | 101905.9 | 6.73 | 10 | 31 | 0 |

Peptide Information

| Calc. Mass | Obsrv. Mass | ± da | ± ppm | Start Seq. | End Seq. | Sequence | Ion Score | C. I. % | Modification | Rank | Result Type |
|---|---|---|---|---|---|---|---|---|---|---|---|
| 829.4606 | 829.5081 | 0.0475 | 57 | 671 | 676 | KFPFYK | | | | | Mascot |
| 907.4301 | 907.4562 | 0.0261 | 29 | 645 | 652 | TGMIESNR | 9 | 0 | | | Mascot |
| 907.4301 | 907.4562 | 0.0261 | 29 | 645 | 652 | TGMIESNR | | | | | Mascot |
| 1045.5459 | 1045.5391 | -0.0068 | -7 | 239 | 247 | LGVNDCVLR | | | Carbamidomethyl (C)[6] | | Mascot |
| 1234.5521 | 1234.6483 | 0.0962 | 78 | 434 | 443 | HMLGEEDFTR | | | | | Mascot |
| 1434.6907 | 1434.7368 | 0.0461 | 32 | 641 | 652 | FHARTGMIESNR | | | Oxidation (M)[7] | | Mascot |
| 1470.7223 | 1470.6367 | -0.0856 | -58 | 855 | 866 | QTYEQANKVFDK | | | | | Mascot |
| 1570.8019 | 1570.736 | -0.0659 | -42 | 749 | 761 | FGSCVPHTTRPRR | | | Carbamidomethyl (C)[4] | | Mascot |
| 1911.052 | 1910.8079 | -0.2441 | -128 | 719 | 734 | QEIHYTRPVIILGPMK | | | Oxidation (M)[15] | | Mascot |
| 1911.052 | 1910.8079 | -0.2441 | -128 | 719 | 734 | QEIHYTRPVIILGPMK | | | Oxidation (M)[15] | | Mascot |
| 1933.9225 | 1933.9635 | 0.041 | 21 | 428 | 443 | YSPVPRHMLGEEDFTR | | | | | Mascot |
| 2186.1743 | 2185.9915 | -0.1828 | -84 | 896 | 914 | LIIEEQSGPYIWIPSAEKL | | | | | Mascot |
| 2186.1743 | 2185.9915 | -0.1828 | -84 | 896 | 914 | LIIEEQSGPYIWIPSAEKL | | | | | Mascot |

| | | | | | | | | |
|---|---|---|---|---|---|---|---|---|
| 41 | PREDICTED: hypothetical protein LOC563653 [Danio rerio] | gi\|292621547 | 110431.7 | 9.74 | 13 | 31 | 0 | |

Peptide Information

| Calc. Mass | Obsrv. Mass | ± da | ± ppm | Start Seq. | End Seq. | Sequence | Ion Score | C. I. % | Modification | Rank | Result Type |
|---|---|---|---|---|---|---|---|---|---|---|---|
| 842.4366 | 842.4906 | 0.054 | 64 | 593 | 599 | ENQVPQK | | | | | Mascot |
| 1011.533 | 1011.454 | -0.079 | -78 | 573 | 581 | QASHVRDAK | | | | | Mascot |
| 1045.5823 | 1045.5391 | -0.0432 | -41 | 247 | 256 | VVSPKMAGTR | | | | | Mascot |
| 1140.6484 | 1140.5654 | -0.083 | -73 | 523 | 532 | SHRTSQALLK | | | | | Mascot |
| 1320.6444 | 1320.5513 | -0.0931 | -70 | 912 | 921 | YRENHDAFLR | | | | | Mascot |
| 1493.7893 | 1493.697 | -0.0923 | -62 | 896 | 908 | GVYLTVPASMQRR | | | Oxidation (M)[10] | | Mascot |
| 1570.8911 | 1570.736 | -0.1551 | -99 | 193 | 206 | LSVLSEERLQAAVR | | | | | Mascot |
| 1598.6421 | 1598.7292 | 0.0871 | 54 | 795 | 806 | MEEMERDQDEVR | | | Oxidation (M)[1,4] | | Mascot |
| 1700.8271 | 1700.7043 | -0.1228 | -72 | 321 | 334 | ECVVEALEPNEKQR | | | Carbamidomethyl (C)[2] | | Mascot |
| 1700.8271 | 1700.7043 | -0.1228 | -72 | 321 | 334 | ECVVEALEPNEKQR | | | Carbamidomethyl (C)[2] | | Mascot |
| 1707.9427 | 1707.7374 | -0.2053 | -120 | 75 | 89 | TNYSNPVVVVIFSLR | | | | | Mascot |
| 1828.9399 | 1828.7953 | -0.1446 | -79 | 286 | 301 | NTIVNSSEPQLTQEIR | | | | | Mascot |
| 2211.0454 | 2211.0491 | 0.0037 | 2 | 783 | 800 | LQGPTLESMLLRMEEME R | | | Oxidation (M)[9,13,16] | | Mascot |
| 2498.2278 | 2498.2664 | 0.0386 | 15 | 394 | 415 | AMQVFINQLSDPAENRV PTHCK | | | | | Mascot |

| 42 | inositol(myo)-1(or 4)-monophosphatase 1 [Danio rerio] | gi\|169153986 | 25887.8 | 4.8 | 6 | 31 | 0 |
|---|---|---|---|---|---|---|---|

Peptide Information

| Calc. Mass | Obsrv. Mass | ± da | ± ppm | Start Seq. | End Seq. | Sequence | Ion Score | C. I. % | Modification | Rank | Result Type |
|---|---|---|---|---|---|---|---|---|---|---|---|
| 868.4709 | 868.4709 | 0 | 0 | 175 | 181 | IFSSMRK | | | | | Mascot |
| 1248.7609 | 1248.5758 | -0.1851 | -148 | 181 | 191 | KILCLPVHGIR | | | | | Mascot |
| 1838.8854 | 1838.8722 | -0.0132 | -7 | 2 | 17 | PDLWQDAMDHAVTLAR | | | | | Mascot |
| 1844.9963 | 1844.792 | -0.2043 | -111 | 46 | 61 | TDQNVEQLIITSVKEK | | | | | Mascot |
| 2225.0618 | 2225.0632 | 0.0014 | 1 | 116 | 134 | TLEFGVVYSCIEDKMYTAR | | | | | Mascot |
| 2225.0618 | 2225.0632 | 0.0014 | 1 | 116 | 134 | TLEFGVVYSCIEDKMYTAR | | | | | Mascot |
| 3308.5964 | 3308.3765 | -0.2199 | -66 | 138 | 167 | GAFCNGQPLQVSDQKEINQSIIATEFGSNR | | | Carbamidomethyl (C)[4] | | Mascot |

| 43 | PREDICTED: serine/arginine repetitive matrix protein 4 [Danio rerio] | gi\|189518857 | 64253.1 | 11.33 | 9 | 31 | 0 |
|---|---|---|---|---|---|---|---|

Peptide Information

| Calc. Mass | Obsrv. Mass | ± da | ± ppm | Start Seq. | End Seq. | Sequence | Ion Score | C. I. % | Modification | Rank | Result Type |
|---|---|---|---|---|---|---|---|---|---|---|---|
| 996.5836 | 996.5735 | -0.0101 | -10 | 59 | 67 | RLETPVGPK | | | | | Mascot |
| 1011.4815 | 1011.454 | -0.0275 | -27 | 288 | 296 | FSCLTSPEK | | | | | Mascot |
| 1041.5952 | 1041.6078 | 0.0126 | 12 | 49 | 56 | FEARLLHR | | | | | Mascot |
| 1068.5031 | 1068.5441 | 0.041 | 38 | 288 | 296 | FSCLTSPEK | | | Carbamidomethyl (C)[3] | | Mascot |
| 1248.6219 | 1248.5758 | -0.0461 | -37 | 325 | 335 | ENTLHSSVFSK | | | | | Mascot |
| 1599.6349 | 1599.8459 | 0.211 | 132 | 222 | 235 | CCCHFSESTVSPSR | | | Carbamidomethyl (C)[1] | | Mascot |
| 1700.955 | 1700.7043 | -0.2507 | -147 | 1 | 15 | MEELICRLLAGILAR | | | | | Mascot |
| 1700.955 | 1700.7043 | -0.2507 | -147 | 1 | 15 | MEELICRLLAGILAR | | | | | Mascot |
| 1716.9498 | 1716.6989 | -0.2509 | -146 | 1 | 15 | MEELICRLLAGILAR | | | Oxidation (M)[1] | | Mascot |
| 1716.9498 | 1716.6989 | -0.2509 | -146 | 1 | 15 | MEELICRLLAGILAR | | | Oxidation (M)[1] | | Mascot |
| 2019.9263 | 2019.8184 | -0.1079 | -53 | 29 | 48 | GSGPFAAGCETAGMRISTYK | | | Oxidation (M)[14] | | Mascot |
| 2119.0876 | 2119.0906 | 0.003 | 1 | 236 | 256 | SGVEGLSKVVIVQNSESSDGK | | | | | Mascot |
| 2155.9739 | 2156.1914 | 0.2175 | 101 | 259 | 279 | ADYDSGNDTSSPPSSKTGITR | | | | | Mascot |

| 44 | 39S ribosomal protein L17, mitochondrial [Danio rerio] | gi\|52219200 | 19088.2 | 9.81 | 7 | 31 | 0 |
|---|---|---|---|---|---|---|---|

Peptide Information

| Calc. Mass | Obsrv. Mass | ± da | ± ppm | Start Seq. | End Seq. | Sequence | Ion Score | C. I. % | Modification | Rank | Result Type |
|---|---|---|---|---|---|---|---|---|---|---|---|
| 850.5032 | 850.5214 | 0.0182 | 21 | 59 | 65 | LIDYAKK | | | | | Mascot |

| | | | | | | | | | | | |
|---|---|---|---|---|---|---|---|---|---|---|---|
| 885.5516 | 885.4941 | -0.0575 | -65 | 31 | 38 | NLLTGLVR | | | | | Mascot |
| 1011.5179 | 1011.454 | -0.0639 | -63 | 119 | 127 | AAMAVLEYK | | | Oxidation (M)[3] | | Mascot |
| 1140.5394 | 1140.5654 | 0.026 | 23 | 75 | 83 | MADFWLTEK | | | | | Mascot |
| 1179.6521 | 1179.5751 | -0.077 | -65 | 128 | 138 | GNPFPPLNPVK | | | | | Mascot |
| 1470.712 | 1470.6367 | -0.0753 | -51 | 72 | 83 | AMKMADFWLTEK | | | | | Mascot |
| 2156.1572 | 2156.1914 | 0.0342 | 16 | 119 | 138 | AAMAVLEYKGNPFPPLN PVK | | | | | Mascot |

45 PREDICTED: calcium-dependent secretion activator 1 [Danio rerio] gi|326668730 145894.4 5.69 16 31 0

**Peptide Information**

| Calc. Mass | Obsrv. Mass | ± da | ± ppm | Start Seq. | End Seq. | Sequence | Ion Score | C. I. % | Modification | Rank | Result Type |
|---|---|---|---|---|---|---|---|---|---|---|---|
| 868.4635 | 868.4709 | 0.0074 | 9 | 1165 | 1171 | HSQDILR | | | | | Mascot |
| 1041.5364 | 1041.6078 | 0.0714 | 69 | 123 | 130 | DRFESFLK | | | | | Mascot |
| 1045.5459 | 1045.5391 | -0.0068 | -7 | 280 | 288 | LQMADQIAR | | | | | Mascot |
| 1179.6343 | 1179.5751 | -0.0592 | -50 | 578 | 586 | ILWVQAMYR | | | | | Mascot |
| 1350.6504 | 1350.6696 | 0.0192 | 14 | 161 | 173 | MVQTGGLSALDCR | | | | | Mascot |
| 1493.7166 | 1493.697 | -0.0196 | -13 | 806 | 817 | CLEQAAQINYQR | | | Carbamidomethyl (C)[1] | | Mascot |
| 1599.7791 | 1599.8459 | 0.0668 | 42 | 1022 | 1035 | MKLMSSDMIETSVK | | | | | Mascot |
| 1700.7288 | 1700.7043 | -0.0245 | -14 | 1072 | 1086 | DQSAKLCAMEMGQEK | | | Oxidation (M)[9,11] | | Mascot |
| 1700.7288 | 1700.7043 | -0.0245 | -14 | 1072 | 1086 | DQSAKLCAMEMGQEK | | | Oxidation (M)[9,11] | | Mascot |
| 1838.9213 | 1838.8722 | -0.0491 | -27 | 1056 | 1071 | IPLSLCTMFNVMVDAK | | | Carbamidomethyl (C)[6] | | Mascot |
| 1910.9463 | 1910.8079 | -0.1384 | -72 | 161 | 177 | MVQTGGLSALDCREVFK | | | Carbamidomethyl (C)[12] | | Mascot |
| 1910.9463 | 1910.8079 | -0.1384 | -72 | 161 | 177 | MVQTGGLSALDCREVFK | | | Carbamidomethyl (C)[12] | | Mascot |
| 2145.1775 | 2145.0481 | -0.1294 | -60 | 587 | 606 | ATGQSHKPVPPTQVQKL NSK | | | | | Mascot |
| 2156.0554 | 2156.1914 | 0.136 | 63 | 806 | 823 | CLEQAAQINYQRITDYAR | | | | | Mascot |
| 2168.0474 | 2167.981 | -0.0664 | -31 | 377 | 395 | SLAPNRIVYCTMEVEGG QK | | | Carbamidomethyl (C)[10], Oxidation (M)[12] | | Mascot |
| 2224.9397 | 2225.0632 | 0.1235 | 56 | 1 | 20 | MLDPSSSEEESDGIVEEE SR | | | | | Mascot |
| 2224.9397 | 2225.0632 | 0.1235 | 56 | 1 | 20 | MLDPSSSEEESDGIVEEE SR | | | | | Mascot |
| 2233.166 | 2233.0325 | -0.1335 | -60 | 84 | 101 | LQLYVFVMRCIAYPFNAK | | | Carbamidomethyl (C)[10] | | Mascot |
| 2346.1055 | 2346.2458 | 0.1403 | 60 | 50 | 72 | GSSARPTSPSPSAASEE KEDVEK | | | | | Mascot |

46 Ppp4r2a protein [Danio rerio] gi|156230689 49479.9 4.37 8 30 0

**Peptide Information**

| Calc. Mass | Obsrv. Mass | ± da | ± ppm | Start Seq. | End Seq. | Sequence | Ion Score | C. I. % | Modification | Rank | Result Type |
|---|---|---|---|---|---|---|---|---|---|---|---|

| Calc. Mass | Obsrv. Mass | ± da | ± ppm | Start Seq. | End Seq. | Sequence | Ion Score | C. I. % | Modification | Rank | Result Type |
|---|---|---|---|---|---|---|---|---|---|---|---|
| 850.4199 | 850.5214 | 0.1015 | 119 | 172 | 179 | KGSSCVNR | | | | | Mascot |
| 907.4413 | 907.4562 | 0.0149 | 16 | 172 | 179 | KGSSCVNR | 3 | 0 | Carbamidomethyl (C)[5] | | Mascot |
| 907.4413 | 907.4562 | 0.0149 | 16 | 172 | 179 | KGSSCVNR | | | Carbamidomethyl (C)[5] | | Mascot |
| 1045.5598 | 1045.5391 | -0.0207 | -20 | 134 | 142 | LCELLTEPK | | | | | Mascot |
| 1140.5605 | 1140.5654 | 0.0049 | 4 | 83 | 91 | LEKVMDDFK | | | Oxidation (M)[5] | | Mascot |
| 1598.6937 | 1598.7292 | 0.0355 | 22 | 86 | 98 | VMDDFKASCPEQR | | | Carbamidomethyl (C)[9], Oxidation (M)[2] | | Mascot |
| 1683.8444 | 1683.7931 | -0.0513 | -30 | 158 | 172 | NVMVVSCVYPTSEKK | | | | | Mascot |
| 1683.8444 | 1683.7931 | -0.0513 | -30 | 158 | 172 | NVMVVSCVYPTSEKK | | | | | Mascot |
| 1707.837 | 1707.7374 | -0.0996 | -58 | 27 | 41 | HTSTMEIDTLLGAFR | | | Oxidation (M)[5] | | Mascot |
| 2119.0276 | 2119.0906 | 0.063 | 30 | 99 | 116 | GPANPNVEYIPFEEMKQR | | | | | Mascot |
| 2211.075 | 2211.0491 | -0.0259 | -12 | 27 | 45 | HTSTMEIDTLLGAFRDFEK | | | | | Mascot |

| | | | | | | | | |
|---|---|---|---|---|---|---|---|---|
| 47 | bcl-2-related ovarian killer protein homolog B [Danio rerio] | gi\|41055421 | 23818.6 | 7.71 | 6 | 30 | 0 | |

Peptide Information

| Calc. Mass | Obsrv. Mass | ± da | ± ppm | Start Seq. | End Seq. | Sequence | Ion Score | C. I. % | Modification | Rank | Result Type |
|---|---|---|---|---|---|---|---|---|---|---|---|
| 842.5134 | 842.4906 | -0.0228 | -27 | 161 | 167 | SLVPWLK | | | | | Mascot |
| 850.4668 | 850.5214 | 0.0546 | 64 | 26 | 32 | ELVFQSK | | | | | Mascot |
| 939.4022 | 939.4149 | 0.0127 | 14 | 178 | 185 | CVVNMDSR | | | Oxidation (M)[5] | | Mascot |
| 939.4022 | 939.4149 | 0.0127 | 14 | 178 | 185 | CVVNMDSR | | | Oxidation (M)[5] | | Mascot |
| 2149.0593 | 2148.9441 | -0.1152 | -54 | 7 | 25 | SSVLAAEMIDVFDRTHTEK | | | | | Mascot |
| 2156.0803 | 2156.1914 | 0.1111 | 52 | 2 | 20 | NVFARSSVLAAEMIDVFDR | | | Oxidation (M)[13] | | Mascot |
| 2186.0732 | 2185.9915 | -0.0817 | -37 | 74 | 91 | LGDELECMRPYVYRNIAK | | | Oxidation (M)[8] | | Mascot |
| 2186.0732 | 2185.9915 | -0.0817 | -37 | 74 | 91 | LGDELECMRPYVYRNIAK | | | Oxidation (M)[8] | | Mascot |

| | | | | | | | | | | |
|---|---|---|---|---|---|---|---|---|---|---|
| 48 | novel protein [Danio rerio] | gi\|169154274 | 85664.7 | 5.94 | 11 | 30 | 0 | 5 | 0 | |

Peptide Information

| Calc. Mass | Obsrv. Mass | ± da | ± ppm | Start Seq. | End Seq. | Sequence | Ion Score | C. I. % | Modification | Rank | Result Type |
|---|---|---|---|---|---|---|---|---|---|---|---|
| 940.5396 | 940.4044 | -0.1352 | -144 | 440 | 447 | NPLCLPKR | | | | | Mascot |
| 1165.5881 | 1165.7177 | 0.1296 | 111 | 1 | 10 | MLESSVNNKK | | | Oxidation (M)[1] | | Mascot |
| 1348.7075 | 1348.6322 | -0.0753 | -56 | 11 | 22 | RCIPSSLTQLCK | | | | | Mascot |
| 1599.8893 | 1599.8459 | -0.0434 | -27 | 366 | 378 | VPDWVFPETLKLR | | | | | Mascot |
| 1700.9364 | 1700.7043 | -0.2321 | -136 | 118 | 133 | LAAQLAVDCTSLLNIR | | | | | Mascot |
| 1700.9364 | 1700.7043 | -0.2321 | -136 | 118 | 133 | LAAQLAVDCTSLLNIR | 5 | 0 | | | Mascot |

| Calc. Mass | Obsrv. Mass | ± da | ± ppm | Start Seq. | End Seq. | Sequence | Ion Score | C. I. % | Modification | Rank | Result Type |
|---|---|---|---|---|---|---|---|---|---|---|---|
| 1819.9371 | 1819.7543 | -0.1828 | -100 | 344 | 360 | AQGDVVSVAFTPQTIMR | | | | | Mascot |
| 1828.9956 | 1828.7953 | -0.2003 | -110 | 361 | 376 | TAAVRVPDWVFPETLK | | | | | Mascot |
| 2020.032 | 2019.8184 | -0.2136 | -106 | 80 | 95 | QTFSEVLHICEQLFLR | | | Carbamidomethyl (C)[10] | | Mascot |
| 2168.0806 | 2167.981 | -0.0996 | -46 | 641 | 659 | DHLQEAIAAWEQATCLIQK | | | | | Mascot |
| 2225.1018 | 2225.0632 | -0.0386 | -17 | 641 | 659 | DHLQEAIAAWEQATCLIQK | | | Carbamidomethyl (C)[15] | | Mascot |
| 2225.1018 | 2225.0632 | -0.0386 | -17 | 641 | 659 | DHLQEAIAAWEQATCLIQK | | | Carbamidomethyl (C)[15] | | Mascot |
| 2233.1758 | 2233.0325 | -0.1433 | -64 | 341 | 360 | QLRAQGDVVSVAFTPQTIMR | | | Oxidation (M)[19] | | Mascot |
| 2381.2031 | 2380.978 | -0.2251 | -95 | 641 | 660 | DHLQEAIAAWEQATCLIQKR | | | Carbamidomethyl (C)[15] | | Mascot |
| 2381.2031 | 2380.978 | -0.2251 | -95 | 641 | 660 | DHLQEAIAAWEQATCLIQKR | | | Carbamidomethyl (C)[15] | | Mascot |

| Rank | Protein Name | Accession No. | Protein MW | Protein PI | Pep. Count | Protein Score | Protein Score C. I. % | Total Ion Score | Total Ion C. I. % |
|---|---|---|---|---|---|---|---|---|---|
| 49 | novel protein similar to vertebrate transient receptor potential cation channel, subfamily V, member 4 (TRPV4) [Danio rerio] | gi\|123227302 | 97398.1 | 8.04 | 1 | | | 25 | 24.383 |

Peptide Information

| Calc. Mass | Obsrv. Mass | ± da | ± ppm | Start Seq. | End Seq. | Sequence | Ion Score | C. I. % | Modification | Rank | Result Type |
|---|---|---|---|---|---|---|---|---|---|---|---|
| 907.4896 | 907.4562 | -0.0334 | -37 | 277 | 283 | GRFFQPR | 25 | 24.383 | | 2 | Mascot |

| Rank | Protein Name | Accession No. | Protein MW | Protein PI | Pep. Count | Protein Score | Protein Score C. I. % | Total Ion Score | Total Ion C. I. % |
|---|---|---|---|---|---|---|---|---|---|
| 50 | PREDICTED: poly(A) RNA polymerase, mitochondrial-like [Danio rerio] | gi\|326672376 | 63999.1 | 9.2 | 1 | | | 19 | 0 |

Peptide Information

| Calc. Mass | Obsrv. Mass | ± da | ± ppm | Start Seq. | End Seq. | Sequence | Ion Score | C. I. % | Modification | Rank | Result Type |
|---|---|---|---|---|---|---|---|---|---|---|---|
| 907.4488 | 907.4562 | 0.0074 | 8 | 1 | 9 | MAASLGVCR | 19 | 0 | | 3 | Mascot |

| | | | | | |
|---|---|---|---|---|---|
| Gel Idx/Pos | 183/H10 | Instr./Gel Origin | BA2060/110707A | Process Status | Analysis Succeeded |
| Plate [#] Name | [1] 31496 | Instrument Sample Name | | Spectra | 11 |

| Rank | Protein Name | Accession No. | Protein MW | Protein PI | Pep. Count | Protein Score | Protein Score C. I. % | Total Ion Score | Total Ion C. I. % |
|---|---|---|---|---|---|---|---|---|---|
| 1 | thimet oligopeptidase [Danio rerio] | gi\|50539778 | 77928.1 | 5.51 | 28 | 1,100 | 100 | 909 | 100 |

Peptide Information

| Calc. Mass | Obsrv. Mass | ± da | ± ppm | Start Seq. | End Seq. | Sequence | Ion Score | C. I. % | Modification | Rank | Result Type |
|---|---|---|---|---|---|---|---|---|---|---|---|
| 809.3787 | 809.3594 | -0.0193 | -24 | 113 | 118 | EDVYQR | | | | | Mascot |
| 908.4294 | 908.4072 | -0.0222 | -24 | 228 | 234 | CFVPETR | | | Carbamidomethyl (C)[1] | | Mascot |
| 965.4686 | 965.4443 | -0.0243 | -25 | 237 | 244 | LEEAFNSR | | | | | Mascot |

| | | | | | | | | | | |
|---|---|---|---|---|---|---|---|---|---|---|
| 1093.5636 | 1093.5359 | -0.0277 | -25 | 236 | 244 | KLEEAFNSR | | | | Mascot |
| 1118.6317 | 1118.6045 | -0.0272 | -24 | 541 | 550 | LANTGLFNLR | | | | Mascot |
| 1118.6317 | 1118.6045 | -0.0272 | -24 | 541 | 550 | LANTGLFNLR | 90 | 100 | | Mascot |
| 1183.5605 | 1183.5306 | -0.0299 | -25 | 218 | 226 | YPHYFPTMK | | | | Mascot |
| 1194.5823 | 1194.553 | -0.0293 | -25 | 624 | 633 | FKQEGVMDPK | | | Oxidation (M)[7] | Mascot |
| 1199.5554 | 1199.5233 | -0.0321 | -27 | 218 | 226 | YPHYFPTMK | | | Oxidation (M)[8] | Mascot |
| 1221.5746 | 1221.5453 | -0.0293 | -24 | 565 | 575 | TGLDPAEEYAR | | | | Mascot |
| 1221.5746 | 1221.5453 | -0.0293 | -24 | 565 | 575 | TGLDPAEEYAR | 95 | 100 | | Mascot |
| 1242.6075 | 1242.5757 | -0.0318 | -26 | 355 | 364 | EYFPMEVVTK | | | | Mascot |
| 1258.6024 | 1258.5682 | -0.0342 | -27 | 355 | 364 | EYFPMEVVTK | | | Oxidation (M)[5] | Mascot |
| 1325.6947 | 1325.6576 | -0.0371 | -28 | 524 | 535 | TGNPIPEELLDK | | | | Mascot |
| 1343.597 | 1343.5637 | -0.0333 | -25 | 102 | 112 | LSEFDVEMSMR | 70 | 99.997 | | Mascot |
| 1343.597 | 1343.5637 | -0.0333 | -25 | 102 | 112 | LSEFDVEMSMR | | | | Mascot |
| 1359.5919 | 1359.5563 | -0.0356 | -26 | 102 | 112 | LSEFDVEMSMR | | | Oxidation (M)[8] | Mascot |
| 1375.5868 | 1375.5514 | -0.0354 | -26 | 102 | 112 | LSEFDVEMSMR | | | Oxidation (M)[8,10] | Mascot |
| 1492.7642 | 1492.7264 | -0.0378 | -25 | 64 | 76 | ALADVEVEYTVQR | 90 | 100 | | Mascot |
| 1492.7642 | 1492.7264 | -0.0378 | -25 | 64 | 76 | ALADVEVEYTVQR | | | | Mascot |
| 1629.7438 | 1629.7004 | -0.0434 | -27 | 178 | 190 | HLNEDTTCLHFSR | | | Carbamidomethyl (C)[8] | Mascot |
| 1629.7438 | 1629.7004 | -0.0434 | -27 | 178 | 190 | HLNEDTTCLHFSR | 91 | 100 | Carbamidomethyl (C)[8] | Mascot |
| 1682.8173 | 1682.7738 | -0.0435 | -26 | 322 | 335 | NLPFTGELYAWDTR | | | | Mascot |
| 1682.8173 | 1682.7738 | -0.0435 | -26 | 322 | 335 | NLPFTGELYAWDTR | 118 | 100 | | Mascot |
| 1797.9534 | 1797.9053 | -0.0481 | -27 | 400 | 415 | SSGLVVGQFYLDLFPR | 141 | 100 | | Mascot |
| 1797.9534 | 1797.9053 | -0.0481 | -27 | 400 | 415 | SSGLVVGQFYLDLFPR | | | | Mascot |
| 1910.048 | 1909.9938 | -0.0542 | -28 | 119 | 136 | IVALEEKLPEGSLSPEAK | | | | Mascot |
| 2107.9792 | 2107.9185 | -0.0607 | -29 | 496 | 512 | DFVEAPSQMLENWVWE K | | | | Mascot |
| 2123.9744 | 2123.9202 | -0.0542 | -26 | 496 | 512 | DFVEAPSQMLENWVWE K | | | Oxidation (M)[9] | Mascot |
| 2141.1125 | 2141.0518 | -0.0607 | -28 | 23 | 40 | WDLTPEQINQLTEELIAK | | | | Mascot |
| 2165.9478 | 2165.895 | -0.0528 | -24 | 102 | 118 | LSEFDVEMSMREDVYQR | | | Oxidation (M)[8,10] | Mascot |
| 2189.0378 | 2188.9788 | -0.059 | -27 | 419 | 438 | YGHAACFGLQPGCLLPN GTR | | | Carbamidomethyl (C)[6,13] | Mascot |
| 2229.092 | 2229.0354 | -0.0566 | -25 | 44 | 63 | VYDTVGALDLDSVNYEN TLK | 51 | 99.799 | | Mascot |
| 2229.092 | 2229.0354 | -0.0566 | -25 | 44 | 63 | VYDTVGALDLDSVNYEN TLK | | | | Mascot |
| 2283.0632 | 2283.1169 | 0.0537 | 24 | 665 | 686 | QDAFLLSKGLTADVESGT PCAC | | | Carbamidomethyl (C)[20] | Mascot |
| 2306.1033 | 2306.0447 | -0.0586 | -25 | 191 | 211 | EELGGLPEDFLNSLEKDE SGK | | | | Mascot |
| 2351.0859 | 2351.0234 | -0.0625 | -27 | 336 | 354 | YYMTQVEETQYAVDQNQ LK | | | | Mascot |
| 2357.187 | 2357.124 | -0.063 | -27 | 43 | 63 | KVYDTVGALDLDSVNYE | | | | Mascot |

| | | | | | | | | | | | |
|---|---|---|---|---|---|---|---|---|---|---|---|
| | | | | | | NTLK | | | | | |
| 2367.0808 | 2367.0222 | -0.0586 | -25 | 336 | 354 | YYMTQVEETQYAVDQNQLK | | | Oxidation (M)[3] | | Mascot |
| 2370.2551 | 2370.1721 | -0.083 | -35 | 23 | 42 | WDLTPEQINQLTEELIAKTK | | | | | Mascot |
| 2410.2976 | 2410.2312 | -0.0664 | -28 | 21 | 40 | LRWDLTPEQINQLTEELIAK | | | | | Mascot |
| 2731.3184 | 2731.2441 | -0.0743 | -27 | 496 | 517 | DFVEAPSQMLENWVWEKEPIQR | | | | | Mascot |
| 2731.3184 | 2731.2441 | -0.0743 | -27 | 496 | 517 | DFVEAPSQMLENWVWEKEPIQR | 163 | 100 | | | Mascot |
| 2747.3132 | 2747.2437 | -0.0695 | -25 | 496 | 517 | DFVEAPSQMLENWVWEKEPIQR | | | Oxidation (M)[9] | | Mascot |

| | | | | | | | | |
|---|---|---|---|---|---|---|---|---|
| 2 | novel protein similar to H.sapiens TTN, titin (TTN) [Danio rerio] | gi\|169154588 | 2451841.5 | 6.24 | 65 | 60 | 96.034 | |

**Peptide Information**

| Calc. Mass | Obsrv. Mass | ± da | ± ppm | Start Seq. | End Seq. | Sequence | Ion Score | C. I. % | Modification | Rank | Result Type |
|---|---|---|---|---|---|---|---|---|---|---|---|
| 809.3675 | 809.3594 | -0.0081 | -10 | 21789 | 21795 | AEAFEDK | | | | | Mascot |
| 842.5094 | 842.4899 | -0.0195 | -23 | 4439 | 4446 | LSIPGVTR | | | | | Mascot |
| 882.4315 | 882.5447 | 0.1132 | 128 | 15166 | 15173 | DGSSFTLR | | | | | Mascot |
| 891.4166 | 891.3801 | -0.0365 | -41 | 15504 | 15512 | ESVASDAGR | | | | | Mascot |
| 907.4705 | 907.4323 | -0.0382 | -42 | 16765 | 16771 | NSLLWMK | | | Oxidation (M)[6] | | Mascot |
| 908.4294 | 908.4072 | -0.0222 | -24 | 8865 | 8872 | NSCTLGWK | | | | | Mascot |
| 965.4509 | 965.4443 | -0.0066 | -7 | 8865 | 8872 | NSCTLGWK | | | Carbamidomethyl (C)[3] | | Mascot |
| 996.5373 | 996.5723 | 0.035 | 35 | 3587 | 3593 | QYQFRVR | | | | | Mascot |
| 1042.5204 | 1042.557 | 0.0366 | 35 | 16493 | 16500 | ELSEVYFR | | | | | Mascot |
| 1045.6404 | 1045.5388 | -0.1016 | -97 | 5457 | 5466 | GTKFVVPGLK | | | | | Mascot |
| 1093.5095 | 1093.5359 | 0.0264 | 24 | 5041 | 5048 | ETWKQCNK | | | Carbamidomethyl (C)[6] | | Mascot |
| 1118.6205 | 1118.6045 | -0.016 | -14 | 13511 | 13519 | EKNSILWTK | | | | | Mascot |
| 1118.6681 | 1118.6045 | -0.0636 | -57 | 2058 | 2066 | ILAYVVERR | | | | | Mascot |
| 1123.4871 | 1123.5106 | 0.0235 | 21 | 9489 | 9497 | LAENVMDCR | | | Carbamidomethyl (C)[8], Oxidation (M)[6] | | Mascot |
| 1157.7252 | 1157.6738 | -0.0514 | -44 | 11658 | 11667 | VQTLNLKITK | | | | | Mascot |
| 1165.6211 | 1165.7151 | 0.094 | 81 | 17778 | 17788 | VSAENKFGVSK | | | | | Mascot |
| 1183.6028 | 1183.5306 | -0.0722 | -61 | 1355 | 1364 | KYEMISEGVK | | | | | Mascot |
| 1194.5923 | 1194.553 | -0.0393 | -33 | 156 | 165 | TLEEEMSVIK | | | Oxidation (M)[6] | | Mascot |
| 1199.5977 | 1199.5233 | -0.0744 | -62 | 1355 | 1364 | KYEMISEGVK | | | Oxidation (M)[4] | | Mascot |
| 1221.6051 | 1221.5453 | -0.0598 | -49 | 268 | 276 | DTPNWKWFK | | | | | Mascot |
| 1221.6051 | 1221.5453 | -0.0598 | -49 | 268 | 276 | DTPNWKWFK | | | | | Mascot |
| 1242.6113 | 1242.5757 | -0.0356 | -29 | 14517 | 14526 | LTQGTEYQFR | | | | | Mascot |
| 1258.6976 | 1258.5682 | -0.1294 | -103 | 19126 | 19136 | GKPLPTCKWTK | | | | | Mascot |

| | | | | | | | | | | |
|---|---|---|---|---|---|---|---|---|---|---|
| 1325.6888 | 1325.6576 | -0.0312 | -24 | 19425 | 19434 | EILGYWIEFR | | | | Mascot |
| 1343.6776 | 1343.5637 | -0.1139 | -85 | 4252 | 4264 | YGCGPPVDLGPIR | | | | Mascot |
| 1343.6776 | 1343.5637 | -0.1139 | -85 | 4252 | 4264 | YGCGPPVDLGPIR | | | | Mascot |
| 1359.6328 | 1359.5563 | -0.0765 | -56 | 20560 | 20570 | NTEVTWYFGSR | | | | Mascot |
| 1367.6412 | 1367.5828 | -0.0584 | -43 | 7028 | 7037 | LMENHEYLFR | | | Oxidation (M)[2] | Mascot |
| 1375.5769 | 1375.5514 | -0.0255 | -19 | 20550 | 20559 | FVCCIENYDR | | | Carbamidomethyl (C)[3,4] | Mascot |
| 1391.6624 | 1391.7058 | 0.0434 | 31 | 11997 | 12008 | VLDTPDMPANFR | | | Oxidation (M)[7] | Mascot |
| 1492.7166 | 1492.7264 | 0.0098 | 7 | 18002 | 18014 | DEYTPPKAELDSK | | | | Mascot |
| 1492.7465 | 1492.7264 | -0.0201 | -13 | 14627 | 14640 | VYAENIAGIGPCTK | | | Carbamidomethyl (C)[12] | Mascot |
| 1626.7693 | 1626.7129 | -0.0564 | -35 | 10716 | 10730 | ACDTIFQPGPPGNPR | | | Carbamidomethyl (C)[2] | Mascot |
| 1629.7809 | 1629.7004 | -0.0805 | -49 | 6389 | 6400 | TEVWLQWNWPDR | | | | Mascot |
| 1629.9104 | 1629.7004 | -0.21 | -129 | 5809 | 5822 | LVVRVGETCIIQSR | | | Carbamidomethyl (C)[9] | Mascot |
| 1682.8265 | 1682.7738 | -0.0527 | -31 | 6244 | 6258 | AEIETSGTTTKLEMR | | | Oxidation (M)[14] | Mascot |
| 1682.8265 | 1682.7738 | -0.0527 | -31 | 6244 | 6258 | AEIETSGTTTKLEMR | | | Oxidation (M)[14] | Mascot |
| 1698.8367 | 1698.765 | -0.0717 | -42 | 11047 | 11061 | ITGYIVEMQGKNSDK | | | Oxidation (M)[8] | Mascot |
| 1714.7965 | 1714.7611 | -0.0354 | -21 | 11274 | 11287 | TGCEYQFRIAAENR | | | Carbamidomethyl (C)[3] | Mascot |
| 1797.8734 | 1797.9053 | 0.0319 | 18 | 14649 | 14664 | DPCAPPSQPRVMNITR | | | Oxidation (M)[12] | Mascot |
| 1797.9713 | 1797.9053 | -0.066 | -37 | 6208 | 6224 | ELKAMAGTHINIMAGIK | 1 | 0 | | Mascot |
| 1820.1255 | 1819.8815 | -0.244 | -134 | 15462 | 15478 | IEIPLTGKPLPKVSLSK | | | | Mascot |
| 1842.8293 | 1842.7349 | -0.0944 | -51 | 1063 | 1077 | FETEISEEDVHGHWK | | | | Mascot |
| 1909.9728 | 1909.9938 | 0.021 | 11 | 1419 | 1434 | LICEVSKPSAEVTWYK | | | Carbamidomethyl (C)[3] | Mascot |
| 1970.877 | 1970.8958 | 0.0188 | 10 | 5938 | 5953 | DLWMPVTESCTRTSCK | | | Carbamidomethyl (C)[10,15] | Mascot |
| 2099.0227 | 2098.9607 | -0.062 | -30 | 2364 | 2382 | WTFGENALTAGDRVSMVTK | | | Oxidation (M)[16] | Mascot |
| 2108.1069 | 2107.9185 | -0.1884 | -89 | 2167 | 2184 | DRTSVVWAPVMVTKPHER | | | | Mascot |
| 2124.1018 | 2123.9202 | -0.1816 | -85 | 2167 | 2184 | DRTSVVWAPVMVTKPHER | | | Oxidation (M)[11] | Mascot |
| 2140.989 | 2141.0518 | 0.0628 | 29 | 1278 | 1296 | LVIHDCTLDDSKTYTCDAK | | | | Mascot |
| 2166.0825 | 2165.895 | -0.1875 | -87 | 13016 | 13034 | GRPIPEINWTKDDGDLPDK | | | | Mascot |
| 2189.0066 | 2188.9788 | -0.0278 | -13 | 19865 | 19884 | CENIGGESDWSEISEPVIPK | | | | Mascot |
| 2190.0205 | 2189.9678 | -0.0527 | -24 | 16484 | 16500 | CQCEVTELKELSEVYFR | | | Carbamidomethyl (C)[1,3] | Mascot |
| 2190.2029 | 2189.9678 | -0.2351 | -107 | 10448 | 10467 | LDAHVTGRPIPSIVWTKDGK | | | | Mascot |
| 2194.0583 | 2194.0261 | -0.0322 | -15 | 13155 | 13174 | VMAENEFGIGLPVETEESVK | | | Oxidation (M)[2] | Mascot |
| 2211.021 | 2211.0452 | 0.0242 | 11 | 7302 | 7320 | MVWSTYSASLVTNYCNVTR | | | Oxidation (M)[1] | Mascot |
| 2215.1111 | 2215.0227 | -0.0884 | -40 | 20741 | 20759 | LNVAPHAVAEDTMRPMFKR | | | Oxidation (M)[13,16] | Mascot |
| 2221.1248 | 2221.0967 | -0.0281 | -13 | 19188 | 19207 | VIGRPDPPEGPLEFDDIQAR | | | | Mascot |

| | | | | | | | | | | | |
|---|---|---|---|---|---|---|---|---|---|---|---|
| 2225.0479 | 2225.0618 | 0.0139 | 6 | 8052 | 8070 | HPFDPPSEPLNCCVNKVSK | | | Carbamidomethyl (C)[12,13] | | Mascot |
| 2229.1736 | 2229.0354 | -0.1382 | -62 | 7725 | 7743 | ITNLTPGIEYYFRVMAVNK | | | | | Mascot |
| 2229.1736 | 2229.0354 | -0.1382 | -62 | 7725 | 7743 | ITNLTPGIEYYFRVMAVNK | | | | | Mascot |
| 2283.1284 | 2283.1169 | -0.0115 | -5 | 2993 | 3014 | VRAVNVAGESEPSEPSDPVLCK | | | | | Mascot |
| 2284.1389 | 2284.1128 | -0.0261 | -11 | 10716 | 10736 | ACDTIFQPGPPGNPRVLDTTK | | | Carbamidomethyl (C)[2] | | Mascot |
| 2306.0732 | 2306.0447 | -0.0285 | -12 | 7121 | 7138 | ETFFMVDKCVENHIYQFR | | | | | Mascot |
| 2351.1272 | 2351.0234 | -0.1038 | -44 | 7301 | 7320 | RMVWSTYSASLVTNYCNVTR | | | | | Mascot |
| 2357.0979 | 2357.124 | 0.0261 | 11 | 5253 | 5270 | ECQYIVQRLTEGHEYEFR | | | Carbamidomethyl (C)[2] | | Mascot |
| 2367.1221 | 2367.0222 | -0.0999 | -42 | 7301 | 7320 | RMVWSTYSASLVTNYCNVTR | | | Oxidation (M)[2] | | Mascot |
| 2370.124 | 2370.1721 | 0.0481 | 20 | 2995 | 3016 | AVNVAGESEPSEPSDPVLCKER | | | Carbamidomethyl (C)[19] | | Mascot |
| 2410.0762 | 2410.2312 | 0.155 | 64 | 1463 | 1483 | MDDAGEYHCKLPTSQTTGNLR | | | Carbamidomethyl (C)[9], Oxidation (M)[1] | | Mascot |
| 2474.1868 | 2474.0852 | -0.1016 | -41 | 8873 | 8895 | KPISDGGSHVVAYALEICEGEDK | | | Carbamidomethyl (C)[18] | | Mascot |
| 2683.2014 | 2683.2458 | 0.0444 | 17 | 4189 | 4212 | LMMLNWSDPDDDGGSDVTGFIIER | | | | | Mascot |
| 2731.3396 | 2731.2441 | -0.0955 | -35 | 8873 | 8897 | KPISDGGSHVVAYALEICEGEDKWK | | | | | Mascot |
| 2731.3396 | 2731.2441 | -0.0955 | -35 | 8873 | 8897 | KPISDGGSHVVAYALEICEGEDKWK | | | | | Mascot |
| 2747.4556 | 2747.2437 | -0.2119 | -77 | 21863 | 21887 | TSFSFVGQVFQAIYTKVTAFVAAHK | | | | | Mascot |
| 2763.4238 | 2763.2476 | -0.1762 | -64 | 16346 | 16370 | GKLTLSWEPPYIDGGSPITNYVVEK | | | | | Mascot |

3 PREDICTED: NACHT, LRR and PYD domains-containing protein 1 [Danio rerio] gi|326674401 10256.3 7.63 7 55 86.561

**Peptide Information**

| Calc. Mass | Obsrv. Mass | ± da | ± ppm | Start Seq. | End Seq. | Sequence | Ion Score | C. I. % | Modification | Rank | Result Type |
|---|---|---|---|---|---|---|---|---|---|---|---|
| 1042.5779 | 1042.557 | -0.0209 | -20 | 86 | 94 | ELDLLVNNL | | | | | Mascot |
| 1258.7001 | 1258.5682 | -0.1319 | -105 | 72 | 83 | ALTEALKSNPSK | | | | | Mascot |
| 1325.6453 | 1325.6576 | 0.0123 | 9 | 44 | 54 | LCVGLQNPHCK | | | Carbamidomethyl (C)[2,10] | | Mascot |
| 1797.8357 | 1797.9053 | 0.0696 | 39 | 6 | 23 | CGLTEESCAALASALSSK | 2 | 0 | Carbamidomethyl (C)[1] | | Mascot |
| 1797.8357 | 1797.9053 | 0.0696 | 39 | 6 | 23 | CGLTEESCAALASALSSK | | | Carbamidomethyl (C)[1] | | Mascot |
| 2141.1018 | 2141.0518 | -0.05 | -23 | 60 | 78 | LSNCNVTHEELRALTEALK | | | | | Mascot |
| 2166.1145 | 2165.895 | -0.2195 | -101 | 2 | 23 | ILAKCGLTEESCAALASALSSK | | | | | Mascot |
| 2370.1714 | 2370.1721 | 0.0007 | 0 | 1 | 23 | MILAKCGLTEESCAALASALSSK | | | Carbamidomethyl (C)[6], Oxidation (M)[1] | | Mascot |

4 PREDICTED: BDNF/NT-3 growth factors receptor [Danio rerio] gi|326670340 87835.2 6.08 14 48 22.666

Peptide Information

| Calc. Mass | Obsrv. Mass | ± da | ± ppm | Start Seq. | End Seq. | Sequence | Ion Score | C. I. % | Modification | Rank | Result Type |
|---|---|---|---|---|---|---|---|---|---|---|---|
| 809.4225 | 809.3594 | -0.0631 | -78 | 45 | 51 | SIMAFPK | | | Oxidation (M)[3] | | Mascot |
| 842.4042 | 842.4899 | 0.0857 | 102 | 79 | 84 | DLEFYR | | | | | Mascot |
| 882.4138 | 882.5447 | 0.1309 | 148 | 650 | 657 | IGDFGMSR | | | | | Mascot |
| 907.4519 | 907.4323 | -0.0196 | -22 | 499 | 507 | ELGEGAFGK | | | | | Mascot |
| 965.5315 | 965.4443 | -0.0872 | -90 | 123 | 129 | LSWRTFR | | | | | Mascot |
| 1118.5801 | 1118.6045 | 0.0244 | 22 | 529 | 538 | TLKEASENAR | | | | | Mascot |
| 1118.5801 | 1118.6045 | 0.0244 | 22 | 529 | 538 | TLKEASENAR | | | | | Mascot |
| 1258.6824 | 1258.5682 | -0.1142 | -91 | 639 | 649 | NCLVGENLLVK | | | Carbamidomethyl (C)[2] | | Mascot |
| 1325.6017 | 1325.6576 | 0.0559 | 42 | 677 | 686 | WMPPESIMYR | | | Oxidation (M)[2] | | Mascot |
| 1375.7368 | 1375.5514 | -0.1854 | -135 | 464 | 474 | IPVIENPQYFR | | | | | Mascot |
| 1682.824 | 1682.7738 | -0.0502 | -30 | 37 | 51 | ISCVDAERSIMAFPK | | | Oxidation (M)[11] | | Mascot |
| 1682.824 | 1682.7738 | -0.0502 | -30 | 37 | 51 | ISCVDAERSIMAFPK | | | Oxidation (M)[11] | | Mascot |
| 2190.022 | 2189.9678 | -0.0542 | -25 | 739 | 755 | EVYDLMLGCWQREPHTR | | | Carbamidomethyl (C)[9] | | Mascot |
| 2190.022 | 2189.9678 | -0.0542 | -25 | 739 | 755 | EVYDLMLGCWQREPHTR | | | Carbamidomethyl (C)[9] | | Mascot |
| 2306.3118 | 2306.0447 | -0.2671 | -116 | 760 | 780 | EIHNLLLNLAKASPVYLDI LG | | | | | Mascot |
| 2410.2686 | 2410.2312 | -0.0374 | -16 | 508 | 528 | VFLAECYNLSADQEKILV AVK | | | Carbamidomethyl (C)[6] | | Mascot |
| 2683.2524 | 2683.2458 | -0.0066 | -2 | 200 | 223 | GLNVTLTCNTSGMPTPN LTWTTCK | | | Carbamidomethyl (C)[8,23], Oxidation (M)[13] | | Mascot |

| | | | | | | | | |
|---|---|---|---|---|---|---|---|---|
| 5 | PREDICTED: inactive ubiquitin carboxyl-terminal hydrolase 53-like [Danio rerio] | gi\|326663976 | 107397.4 | 8.94 | 16 | 41 | 0 | |

Peptide Information

| Calc. Mass | Obsrv. Mass | ± da | ± ppm | Start Seq. | End Seq. | Sequence | Ion Score | C. I. % | Modification | Rank | Result Type |
|---|---|---|---|---|---|---|---|---|---|---|---|
| 891.4716 | 891.3801 | -0.0915 | -103 | 298 | 305 | DVASKCIR | | | | | Mascot |
| 996.4965 | 996.5723 | 0.0758 | 76 | 586 | 593 | QMMVTRSK | | | Oxidation (M)[2] | | Mascot |
| 1042.5099 | 1042.557 | 0.0471 | 45 | 697 | 705 | LRYSSCGTR | | | | | Mascot |
| 1093.5055 | 1093.5359 | 0.0304 | 28 | 477 | 487 | SNQGMASGVSR | | | | | Mascot |
| 1157.6273 | 1157.6738 | 0.0465 | 40 | 57 | 66 | ERALPSDTLR | | | | | Mascot |
| 1194.5784 | 1194.553 | -0.0254 | -21 | 346 | 356 | GTDTQLSGCKK | | | Carbamidomethyl (C)[9] | | Mascot |
| 1199.5876 | 1199.5233 | -0.0643 | -54 | 532 | 540 | QQQQQQQQR | | | | | Mascot |
| 1325.6478 | 1325.6576 | 0.0098 | 7 | 1 | 14 | MSGTGARSGTGLSK | | | Oxidation (M)[1] | | Mascot |
| 1359.6838 | 1359.5563 | -0.1275 | -94 | 662 | 673 | MEAARSPLYHGK | | | | | Mascot |
| 1375.6787 | 1375.5514 | -0.1273 | -93 | 662 | 673 | MEAARSPLYHGK | | | Oxidation (M)[1] | | Mascot |
| 1391.7139 | 1391.7058 | -0.0081 | -6 | 458 | 469 | VSRTEHSSVHPR | | | | | Mascot |

| | | | | | | | | | | | |
|---|---|---|---|---|---|---|---|---|---|---|---|
| 1626.8281 | 1626.7129 | -0.1152 | -71 | 445 | 457 | FHCEQRPRTLPSR | | | | | Mascot |
| 1698.731 | 1698.765 | 0.034 | 20 | 852 | 867 | MEINGTCPSSSDVSSK | | | Carbamidomethyl (C)[7] | | Mascot |
| 1714.7258 | 1714.7611 | 0.0353 | 21 | 852 | 867 | MEINGTCPSSSDVSSK | | | Carbamidomethyl (C)[7], Oxidation (M)[1] | | Mascot |
| 1797.8105 | 1797.9053 | 0.0948 | 53 | 851 | 867 | RMEINGTCPSSSDVSSK | | | | | Mascot |
| 1797.8105 | 1797.9053 | 0.0948 | 53 | 851 | 867 | RMEINGTCPSSSDVSSK | | | | | Mascot |
| 2189.1396 | 2188.9788 | -0.1608 | -73 | 918 | 936 | YHHQRPLVLSMPEPRGA GK | | | Oxidation (M)[11] | | Mascot |
| 2306.0505 | 2306.0447 | -0.0058 | -3 | 642 | 661 | LSQDAQHLRDQGHNNQ DDSK | | | | | Mascot |
| 2474.0405 | 2474.0852 | 0.0447 | 18 | 852 | 874 | MEINGTCPSSSDVSSKS GSDQER | | | Carbamidomethyl (C)[7], Oxidation (M)[1] | | Mascot |

| | | | | | | | |
|---|---|---|---|---|---|---|---|
| 6 | PREDICTED: interferon-induced very large GTPase 1 [Danio rerio] | gi\|326665713 | 165664.4 | 5.71 | 19 | 40 | 0 |

Peptide Information

| Calc. Mass | Obsrv. Mass | ± da | ± ppm | Start Seq. | End Seq. | Sequence | Ion Score | C. I. % | Modification | Rank | Result Type |
|---|---|---|---|---|---|---|---|---|---|---|---|
| 908.4359 | 908.4072 | -0.0287 | -32 | 1377 | 1383 | FQEDLEK | | | | | Mascot |
| 996.4268 | 996.5723 | 0.1455 | 146 | 760 | 767 | DEVYDAER | | | | | Mascot |
| 1042.5316 | 1042.557 | 0.0254 | 24 | 381 | 389 | ESFLPHQGK | | | | | Mascot |
| 1045.6404 | 1045.5388 | -0.1016 | -97 | 1160 | 1168 | KFNISVLPK | | | | | Mascot |
| 1093.4691 | 1093.5359 | 0.0668 | 61 | 1105 | 1114 | SNCESLNGNR | | | | | Mascot |
| 1118.6165 | 1118.6045 | -0.012 | -11 | 662 | 671 | SLELSGKQTR | | | | | Mascot |
| 1118.6165 | 1118.6045 | -0.012 | -11 | 662 | 671 | SLELSGKQTR | | | | | Mascot |
| 1183.6074 | 1183.5306 | -0.0768 | -65 | 31 | 39 | LLMMNYRAR | | | Oxidation (M)[3] | | Mascot |
| 1199.6024 | 1199.5233 | -0.0791 | -66 | 31 | 39 | LLMMNYRAR | | | Oxidation (M)[3,4] | | Mascot |
| 1221.6222 | 1221.5453 | -0.0769 | -63 | 969 | 978 | THHENTLLEK | | | | | Mascot |
| 1221.6395 | 1221.5453 | -0.0942 | -77 | 637 | 646 | VSEEMIKEIK | | | Oxidation (M)[5] | | Mascot |
| 1258.6426 | 1258.5682 | -0.0744 | -59 | 832 | 841 | DLWEALLNER | | | | | Mascot |
| 1626.885 | 1626.7129 | -0.1721 | -106 | 376 | 389 | LTEIKESFLPHQGK | | | | | Mascot |
| 1682.8325 | 1682.7738 | -0.0587 | -35 | 1372 | 1383 | WFVWRFQEDLEK | | | | | Mascot |
| 1682.8325 | 1682.7738 | -0.0587 | -35 | 1372 | 1383 | WFVWRFQEDLEK | | | | | Mascot |
| 1698.8441 | 1698.765 | -0.0791 | -47 | 1023 | 1036 | IMTDVSEILCNLYK | | | Carbamidomethyl (C)[10] | | Mascot |
| 1714.839 | 1714.7611 | -0.0779 | -45 | 1023 | 1036 | IMTDVSEILCNLYK | | | Carbamidomethyl (C)[10], Oxidation (M)[2] | | Mascot |
| 2108.0667 | 2107.9185 | -0.1482 | -70 | 206 | 224 | SRILMDGVVEIAWFCPSG K | | | | | Mascot |
| 2124.0615 | 2123.9202 | -0.1413 | -67 | 206 | 224 | SRILMDGVVEIAWFCPSG K | | | Oxidation (M)[5] | | Mascot |
| 2189.0339 | 2188.9788 | -0.0551 | -25 | 87 | 105 | IHPMDVQMAVFHCADAF LK | | | Oxidation (M)[4] | | Mascot |
| 2306.1775 | 2306.0447 | -0.1328 | -58 | 459 | 478 | ILLDEHTSADLSALHHKY DK | | | | | Mascot |

| Calc. Mass | Obsrv. Mass | ± da | ± ppm | Start Seq. | End Seq. | Sequence | Ion Score | C. I. % | Modification | Rank | Result Type |
|---|---|---|---|---|---|---|---|---|---|---|---|
| 2357.1443 | 2357.124 | -0.0203 | -9 | 1239 | 1259 | QELPDIMSQTSTGFNKTFGQK | | | | | Mascot |
| 2410.2244 | 2410.2312 | 0.0068 | 3 | 282 | 302 | TAKPLICLFTEDESPLTEMKK | | | Oxidation (M)[19] | | Mascot |
| 2731.3184 | 2731.2441 | -0.0743 | -27 | 150 | 172 | DNEIISQSQPVYNAQTPMVFFFR | | | | | Mascot |
| 2731.3184 | 2731.2441 | -0.0743 | -27 | 150 | 172 | DNEIISQSQPVYNAQTPMVFFFR | | | | | Mascot |
| 2747.3132 | 2747.2437 | -0.0695 | -25 | 150 | 172 | DNEIISQSQPVYNAQTPMVFFFR | | | Oxidation (M)[18] | | Mascot |

7 novel protein similar to H.sapiens NTRK2, neurotrophic tyrosine kinase, receptor, type 2 (NTRK2) [D gi|220673329 44252.6 6.45 9 40 0

**Peptide Information**

| Calc. Mass | Obsrv. Mass | ± da | ± ppm | Start Seq. | End Seq. | Sequence | Ion Score | C. I. % | Modification | Rank | Result Type |
|---|---|---|---|---|---|---|---|---|---|---|---|
| 882.4138 | 882.5447 | 0.1309 | 148 | 262 | 269 | IGDFGMSR | | | | | Mascot |
| 907.4519 | 907.4323 | -0.0196 | -22 | 111 | 119 | ELGEGAFGK | | | | | Mascot |
| 1118.5801 | 1118.6045 | 0.0244 | 22 | 141 | 150 | TLKEASENAR | | | | | Mascot |
| 1118.5801 | 1118.6045 | 0.0244 | 22 | 141 | 150 | TLKEASENAR | | | | | Mascot |
| 1258.6824 | 1258.5682 | -0.1142 | -91 | 251 | 261 | NCLVGENLLVK | | | Carbamidomethyl (C)[2] | | Mascot |
| 1325.6017 | 1325.6576 | 0.0559 | 42 | 289 | 298 | WMPPESIMYR | | | Oxidation (M)[2] | | Mascot |
| 1375.7368 | 1375.5514 | -0.1854 | -135 | 76 | 86 | IPVIENPQYFR | | | | | Mascot |
| 2190.022 | 2189.9678 | -0.0542 | -25 | 351 | 367 | EVYDLMLGCWQREPHTR | | | Carbamidomethyl (C)[9] | | Mascot |
| 2190.022 | 2189.9678 | -0.0542 | -25 | 351 | 367 | EVYDLMLGCWQREPHTR | | | Carbamidomethyl (C)[9] | | Mascot |
| 2306.3118 | 2306.0447 | -0.2671 | -116 | 372 | 392 | EIHNLLLNLAKASPVYLDILG | | | | | Mascot |
| 2410.2686 | 2410.2312 | -0.0374 | -16 | 120 | 140 | VFLAECYNLSADQEKILVAVK | | | Carbamidomethyl (C)[6] | | Mascot |

8 PREDICTED: telomerase-binding protein EST1A-like [Danio rerio] gi|326671361 172765.9 8.16 19 39 0

**Peptide Information**

| Calc. Mass | Obsrv. Mass | ± da | ± ppm | Start Seq. | End Seq. | Sequence | Ion Score | C. I. % | Modification | Rank | Result Type |
|---|---|---|---|---|---|---|---|---|---|---|---|
| 842.4114 | 842.4899 | 0.0785 | 93 | 267 | 273 | HSEEGRK | | | | | Mascot |
| 908.4909 | 908.4072 | -0.0837 | -92 | 1439 | 1446 | AAVMFLEK | | | | | Mascot |
| 996.5512 | 996.5723 | 0.0211 | 21 | 809 | 816 | LQSVFQFK | | | | | Mascot |
| 1042.4735 | 1042.557 | 0.0835 | 80 | 376 | 383 | RYSQSDMR | | | | | Mascot |
| 1093.4392 | 1093.5359 | 0.0967 | 88 | 257 | 266 | DGQDDSNQSK | | | | | Mascot |
| 1118.5623 | 1118.6045 | 0.0422 | 38 | 1 | 9 | MADELERVR | | | | | Mascot |
| 1118.5623 | 1118.6045 | 0.0422 | 38 | 1 | 9 | MADELERVR | | | | | Mascot |
| 1183.5226 | 1183.5306 | 0.008 | 7 | 857 | 867 | EQASESANYGK | | | | | Mascot |

| | | | | | | | | | | | |
|---|---|---|---|---|---|---|---|---|---|---|---|
| 1194.6226 | 1194.553 | -0.0696 | -58 | 320 | 330 | ASEAPHLEGRK | | | | | Mascot |
| 1492.7502 | 1492.7264 | -0.0238 | -16 | 412 | 424 | AEGQQFSARTLER | | | | | Mascot |
| 1492.7502 | 1492.7264 | -0.0238 | -16 | 412 | 424 | AEGQQFSARTLER | | | | | Mascot |
| 1698.9021 | 1698.765 | -0.1371 | -81 | 774 | 788 | QLLKDQNSDTAPQIK | | | | | Mascot |
| 1970.9932 | 1970.8958 | -0.0974 | -49 | 1262 | 1278 | YFLEALCGQEEPLLAFK | | | | | Mascot |
| 2107.9084 | 2107.9185 | 0.0101 | 5 | 389 | 409 | TYSTSSASSGTSMDGLA EAER | | | | | Mascot |
| 2123.9033 | 2123.9202 | 0.0169 | 8 | 389 | 409 | TYSTSSASSGTSMDGLA EAER | | | Oxidation (M)[13] | | Mascot |
| 2210.9263 | 2211.0452 | 0.1189 | 54 | 1480 | 1497 | GNNDDVILSCCLHYCQD K | | | Carbamidomethyl (C)[10,11,15] | | Mascot |
| 2215.1614 | 2215.0227 | -0.1387 | -63 | 1056 | 1073 | MLQIITINMFTIYNAQIR | | | Oxidation (M)[1,9] | | Mascot |
| 2221.2451 | 2221.0967 | -0.1484 | -67 | 1036 | 1055 | VLLEFRALLQHSPSPLGS TR | | | | | Mascot |
| 2229.1257 | 2229.0354 | -0.0903 | -41 | 10 | 30 | ISAAELRAQASSFNIHGD DVK | | | | | Mascot |
| 2229.1257 | 2229.0354 | -0.0903 | -41 | 10 | 30 | ISAAELRAQASSFNIHGD DVK | | | | | Mascot |
| 2283.1301 | 2283.1169 | -0.0132 | -6 | 1138 | 1156 | ELLPSMKVWSDWMLGH PEK | | | | | Mascot |
| 2351.1765 | 2351.0234 | -0.1531 | -65 | 743 | 762 | VILTDIEFSDSQNVDQTL WK | | | | | Mascot |
| 2410.0583 | 2410.2312 | 0.1729 | 72 | 1480 | 1499 | GNNDDVILSCCLHYCQD KAK | | | Carbamidomethyl (C)[10,11,15] | | Mascot |

| | | | | | | | |
|---|---|---|---|---|---|---|---|
| 9 | PREDICTED: retrotransposable element Tf2 155 kDa protein type 1-like [Danio rerio] | gi\|326675725 | 129547 | 9.45 | 15 | 38 | 0 |

**Peptide Information**

| Calc. Mass | Obsrv. Mass | ± da | ± ppm | Start Seq. | End Seq. | Sequence | Ion Score | C. I. % | Modification | Rank | Result Type |
|---|---|---|---|---|---|---|---|---|---|---|---|
| 809.4039 | 809.3594 | -0.0445 | -55 | 127 | 133 | AFSDELK | | | | | Mascot |
| 908.4584 | 908.4072 | -0.0512 | -56 | 765 | 771 | LFDRSDR | | | | | Mascot |
| 1157.646 | 1157.6738 | 0.0278 | 24 | 1114 | 1123 | NIPLRTVCNK | | | | | Mascot |
| 1343.7529 | 1343.5637 | -0.1892 | -141 | 595 | 607 | QLAAPLTNLTSSK | | | | | Mascot |
| 1343.7529 | 1343.5637 | -0.1892 | -141 | 595 | 607 | QLAAPLTNLTSSK | | | | | Mascot |
| 1375.7004 | 1375.5514 | -0.149 | -108 | 107 | 118 | AALWGTTVWENK | | | | | Mascot |
| 1492.7502 | 1492.7264 | -0.0238 | -16 | 134 | 147 | VVFDQAASGREASR | | | | | Mascot |
| 1492.7502 | 1492.7264 | -0.0238 | -16 | 134 | 147 | VVFDQAASGREASR | | | | | Mascot |
| 1626.9148 | 1626.7129 | -0.2019 | -124 | 833 | 847 | VACHPGVSRTLFVIK | | | | | Mascot |
| 1682.8285 | 1682.7738 | -0.0547 | -33 | 608 | 622 | TPFRWSNAAEAAFSK | | | | | Mascot |
| 1682.9547 | 1682.7738 | -0.1809 | -107 | 204 | 218 | TLEGLINLAVRVDNR | | | | | Mascot |
| 1797.8622 | 1797.9053 | 0.0431 | 24 | 858 | 873 | DVRDFVLACSVCAASK | | | Carbamidomethyl (C)[9,12] | | Mascot |
| 1797.8622 | 1797.9053 | 0.0431 | 24 | 858 | 873 | DVRDFVLACSVCAASK | | | Carbamidomethyl (C)[9,12] | | Mascot |
| 2099.0227 | 2098.9607 | -0.062 | -30 | 556 | 573 | MDPEKIQAVVNWPTPDS R | | | Oxidation (M)[1] | | Mascot |

| | | | | | | | | | | | | |
|---|---|---|---|---|---|---|---|---|---|---|---|---|
| | 2221.0818 | 2221.0967 | 0.0149 | 7 | 53 | 72 | SSEPRLPPPQSYAGEPHLCR | | | | | Mascot |
| | 2225.0122 | 2225.0618 | 0.0496 | 22 | 269 | 289 | AGGLCMYCGVGGHFVALCPAK | | | Carbamidomethyl (C)[5,8,18] | | Mascot |
| | 2283.0652 | 2283.1169 | 0.0517 | 23 | 268 | 289 | RAGGLCMYCGVGGHFVALCPAK | | | Carbamidomethyl (C)[6], Oxidation (M)[7] | | Mascot |
| | 2370.1006 | 2370.1721 | 0.0715 | 30 | 107 | 126 | AALWGTTVWENKSPCCNTFK | | | Carbamidomethyl (C)[15,16] | | Mascot |

| | | | | | | | | | |
|---|---|---|---|---|---|---|---|---|---|
| 10 | vitamin D3 receptor B [Danio rerio] | gi\|229606097 | 47632.7 | 6.61 | 7 | 38 | 0 | 20 | 0 |

Peptide Information

| Calc. Mass | Obsrv. Mass | ± da | ± ppm | Start Seq. | End Seq. | Sequence | Ion Score | C. I. % | Modification | Rank | Result Type |
|---|---|---|---|---|---|---|---|---|---|---|---|
| 908.3964 | 908.4072 | 0.0108 | 12 | 22 | 29 | ICGVCGDK | | | Carbamidomethyl (C)[2,5] | | Mascot |
| 1118.6238 | 1118.6045 | -0.0193 | -17 | 262 | 271 | SSAIEVIMLR | | | | | Mascot |
| 1118.6238 | 1118.6045 | -0.0193 | -17 | 262 | 271 | SSAIEVIMLR | 20 | 0 | | | Mascot |
| 1123.5421 | 1123.5106 | -0.0315 | -28 | 82 | 90 | RCLDIGMMK | | | Carbamidomethyl (C)[2] | | Mascot |
| 1343.7417 | 1343.5637 | -0.178 | -132 | 250 | 261 | ELTAEDQIALLK | | | | | Mascot |
| 1343.7417 | 1343.5637 | -0.178 | -132 | 250 | 261 | ELTAEDQIALLK | | | | | Mascot |
| 1375.7329 | 1375.5514 | -0.1815 | -132 | 129 | 140 | NIIDTLVDAHHK | | | | | Mascot |
| 2107.9832 | 2107.9185 | -0.0647 | -31 | 141 | 157 | TYDDSYSDFSRFRPPVR | | | | | Mascot |
| 2123.9248 | 2123.9202 | -0.0046 | -2 | 30 | 48 | ATGFHFNAMTCEGCKGFFR | | | | | Mascot |

| | | | | | | | |
|---|---|---|---|---|---|---|---|
| 11 | hyaluronan-binding protein 2 [Danio rerio] | gi\|160333545 | 55971.4 | 8.88 | 10 | 38 | 0 |

Peptide Information

| Calc. Mass | Obsrv. Mass | ± da | ± ppm | Start Seq. | End Seq. | Sequence | Ion Score | C. I. % | Modification | Rank | Result Type |
|---|---|---|---|---|---|---|---|---|---|---|---|
| 908.4334 | 908.4072 | -0.0262 | -29 | 201 | 207 | EPWCFVK | | | | | Mascot |
| 965.4549 | 965.4443 | -0.0106 | -11 | 201 | 207 | EPWCFVK | | | Carbamidomethyl (C)[4] | | Mascot |
| 1093.5248 | 1093.5359 | 0.0111 | 10 | 92 | 100 | CWPPYRGSK | | | | | Mascot |
| 1165.571 | 1165.7151 | 0.1441 | 124 | 201 | 209 | EPWCFVKEK | | | | | Mascot |
| 1199.5408 | 1199.5233 | -0.0175 | -15 | 416 | 425 | CMSRNVYGNR | | | | | Mascot |
| 1367.6776 | 1367.5828 | -0.0948 | -69 | 486 | 495 | FIDWINEKMR | | | Oxidation (M)[9] | | Mascot |
| 1820.05 | 1819.8815 | -0.1685 | -93 | 46 | 63 | AAASNASVHRPILAKSVK | | | | | Mascot |
| 1842.8877 | 1842.7349 | -0.1528 | -83 | 237 | 253 | LEFSDCGKAAFSMIAPR | | | | | Mascot |
| 2141.012 | 2141.0518 | 0.0398 | 19 | 456 | 474 | DNIHYIYGVVSWGDSCGKK | | | | | Mascot |
| 2210.9487 | 2211.0452 | 0.0965 | 44 | 103 | 122 | NAYACNPSPCQNGGTCVKGR | | | Carbamidomethyl (C)[5,10,16] | | Mascot |
| 2228.9304 | 2229.0354 | 0.105 | 47 | 101 | 120 | CKNAYACNPSPCQNGGTCVK | | | Carbamidomethyl (C)[1,7,12] | | Mascot |
| 2228.9304 | 2229.0354 | 0.105 | 47 | 101 | 120 | CKNAYACNPSPCQNGGTCVK | | | Carbamidomethyl (C)[1,7,12] | | Mascot |

12 intraflagellar transport protein 74 homolog [Danio rerio] gi|50539838 57170.8 5.07 10 37 0

Peptide Information

| Calc. Mass | Obsrv. Mass | ± da | ± ppm | Start Seq. | End Seq. | Sequence | Ion Score | C. I. % | Modification | Rank | Result Type |
|---|---|---|---|---|---|---|---|---|---|---|---|
| 891.4166 | 891.3801 | -0.0365 | -41 | 332 | 339 | GLNSESER | | | | | Mascot |
| 1042.6003 | 1042.557 | -0.0433 | -42 | 104 | 112 | RAAQDIIQK | | | | | Mascot |
| 1045.5385 | 1045.5388 | 0.0003 | 0 | 179 | 187 | RASLQAEDR | | | | | Mascot |
| 1118.5874 | 1118.6045 | 0.0171 | 15 | 224 | 232 | IIQLSEEMR | | | | | Mascot |
| 1118.5874 | 1118.6045 | 0.0171 | 15 | 224 | 232 | IIQLSEEMR | | | | | Mascot |
| 1157.6161 | 1157.6738 | 0.0577 | 50 | 94 | 103 | AVEEQVVQQK | | | | | Mascot |
| 1367.6471 | 1367.5828 | -0.0643 | -47 | 320 | 331 | ETEMQKSQSTAK | | | | | Mascot |
| 1492.7312 | 1492.7264 | -0.0048 | -3 | 105 | 117 | AAQDIIQKMSEDK | | | Oxidation (M)[9] | | Mascot |
| 1492.7312 | 1492.7264 | -0.0048 | -3 | 105 | 117 | AAQDIIQKMSEDK | | | Oxidation (M)[9] | | Mascot |
| 2099.084 | 2098.9607 | -0.1233 | -59 | 279 | 297 | DSQSSIVALLEHSSRNIS R | | | | | Mascot |
| 2221.0408 | 2221.0967 | 0.0559 | 25 | 9 | 26 | DIDTFNQENSVYLSYEKR | | | | | Mascot |
| 2731.2727 | 2731.2441 | -0.0286 | -10 | 126 | 148 | SSNEELLQELETHQEELD TLMSR | | | | | Mascot |
| 2731.2727 | 2731.2441 | -0.0286 | -10 | 126 | 148 | SSNEELLQELETHQEELD TLMSR | | | | | Mascot |
| 2747.2676 | 2747.2437 | -0.0239 | -9 | 126 | 148 | SSNEELLQELETHQEELD TLMSR | | | Oxidation (M)[21] | | Mascot |

13 beta-adrenergic receptor kinase 2 [Danio rerio] gi|197927112 79453.5 7.57 13 37 0

Peptide Information

| Calc. Mass | Obsrv. Mass | ± da | ± ppm | Start Seq. | End Seq. | Sequence | Ion Score | C. I. % | Modification | Rank | Result Type |
|---|---|---|---|---|---|---|---|---|---|---|---|
| 1093.5649 | 1093.5359 | -0.029 | -27 | 387 | 395 | GHSPFRQHK | | | | | Mascot |
| 1118.5043 | 1118.6045 | 0.1002 | 90 | 216 | 224 | MYAMKCLDK | | | Oxidation (M)[1] | | Mascot |
| 1118.5043 | 1118.6045 | 0.1002 | 90 | 216 | 224 | MYAMKCLDK | | | Oxidation (M)[1] | | Mascot |
| 1165.7303 | 1165.7151 | -0.0152 | -13 | 31 | 40 | KIILPEPSIR | | | | | Mascot |
| 1242.5316 | 1242.5757 | 0.0441 | 35 | 558 | 567 | DCIMHGYMLK | | | Oxidation (M)[4,8] | | Mascot |
| 1325.6406 | 1325.6576 | 0.017 | 13 | 333 | 344 | ISDLGLACDFSK | | | Carbamidomethyl (C)[8] | | Mascot |
| 1359.7168 | 1359.5563 | -0.1605 | -118 | 568 | 578 | LGNPFLTQWQR | | | | | Mascot |
| 1375.7039 | 1375.5514 | -0.1525 | -111 | 116 | 127 | ELLSCSHPFSKK | | | | | Mascot |
| 1626.9247 | 1626.7129 | -0.2118 | -130 | 32 | 45 | IILPEPSIRSVMQK | | | Oxidation (M)[12] | | Mascot |
| 2098.9248 | 2098.9607 | 0.0359 | 17 | 365 | 383 | GTAYDSSADWFSLGCML FK | | | | | Mascot |
| 2194.1438 | 2194.0261 | -0.1177 | -54 | 345 | 364 | KKPHASVGTHGYMAPEV LQK | | | Oxidation (M)[13] | | Mascot |
| 2225.0696 | 2225.0618 | -0.0078 | -4 | 626 | 644 | GGKQFVLQCESDPEFVQ | | | | | Mascot |

| Calc. Mass | Obsrv. Mass | ± da | ± ppm | Start Seq. | End Seq. | Sequence | Ion Score | C. I. % | Modification | Rank | Result Type |
|---|---|---|---|---|---|---|---|---|---|---|---|
| | | | | | | WK | | | | | |
| 2474.2095 | 2474.0852 | -0.1243 | -50 | 107 | 126 | QIYDVYIMKELLSCSHPFSK | | | Carbamidomethyl (C)[14], Oxidation (M)[8] | | Mascot |
| 2731.3232 | 2731.2441 | -0.0791 | -29 | 174 | 195 | FCQWKNVELNIHLTMNDFSVHR | | | | | Mascot |
| 2731.3232 | 2731.2441 | -0.0791 | -29 | 174 | 195 | FCQWKNVELNIHLTMNDFSVHR | | | | | Mascot |
| 2747.3181 | 2747.2437 | -0.0744 | -27 | 174 | 195 | FCQWKNVELNIHLTMNDFSVHR | | | Oxidation (M)[15] | | Mascot |

14 PREDICTED: zinc finger protein 420 isoform 2 [Danio rerio] gi|326678086 70371 9.08 15 36 0

**Peptide Information**

| Calc. Mass | Obsrv. Mass | ± da | ± ppm | Start Seq. | End Seq. | Sequence | Ion Score | C. I. % | Modification | Rank | Result Type |
|---|---|---|---|---|---|---|---|---|---|---|---|
| 842.5206 | 842.4899 | -0.0307 | -36 | 25 | 31 | QRQGLLK | | | | | Mascot |
| 908.3171 | 908.4072 | 0.0901 | 99 | 127 | 133 | CSHCDMR | | | Carbamidomethyl (C)[1] | | Mascot |
| 965.3386 | 965.4443 | 0.1057 | 109 | 127 | 133 | CSHCDMR | | | Carbamidomethyl (C)[1,4] | | Mascot |
| 1045.536 | 1045.5388 | 0.0028 | 3 | 134 | 141 | FHCARNLK | | | Carbamidomethyl (C)[3] | | Mascot |
| 1093.4531 | 1093.5359 | 0.0828 | 76 | 62 | 70 | EDLSEDEEK | | | | | Mascot |
| 1118.4645 | 1118.6045 | 0.14 | 125 | 348 | 356 | GFVCFECEK | | | Carbamidomethyl (C)[4] | | Mascot |
| 1118.4645 | 1118.6045 | 0.14 | 125 | 208 | 216 | EFVCFECGK | | | Carbamidomethyl (C)[4] | | Mascot |
| 1183.6443 | 1183.5306 | -0.1137 | -96 | 478 | 487 | LHQHVHTGVR | | | | | Mascot |
| 1242.6073 | 1242.5757 | -0.0316 | -25 | 71 | 80 | HQVKSEEETR | | | | | Mascot |
| 1629.8716 | 1629.7004 | -0.1712 | -105 | 295 | 309 | CGHCGKTFLRPAVLK | | | | | Mascot |
| 1629.8716 | 1629.7004 | -0.1712 | -105 | 295 | 309 | CGHCGKTFLRPAVLK | | | | | Mascot |
| 1819.9358 | 1819.8815 | -0.0543 | -30 | 44 | 58 | IKQEETEELIDVMVK | | | Oxidation (M)[13] | | Mascot |
| 2189.0378 | 2188.9788 | -0.059 | -27 | 339 | 356 | HQQIHTAVRGFVCFECEK | | | Carbamidomethyl (C)[13] | | Mascot |
| 2211.0798 | 2211.0452 | -0.0346 | -16 | 450 | 468 | IHLRVHTNEKPYSCPECGK | | | | | Mascot |
| 2214.9841 | 2215.0227 | 0.0386 | 17 | 506 | 524 | QHQMIHTGEKPYTCSHCSK | | | | | Mascot |
| 2221.0139 | 2221.0967 | 0.0828 | 37 | 239 | 257 | CSHCDLRFSHSGHLTVHER | | | | | Mascot |
| 2283.0579 | 2283.1169 | 0.059 | 26 | 227 | 245 | HQMIHTGEKPYKCSHCDLR | | | | | Mascot |

15 PREDICTED: von Willebrand factor D and EGF domain-containing protein-like, partial [Danio rerio] gi|292626323 108459.6 6.25 14 36 0

**Peptide Information**

| Calc. Mass | Obsrv. Mass | ± da | ± ppm | Start Seq. | End Seq. | Sequence | Ion Score | C. I. % | Modification | Rank | Result Type |
|---|---|---|---|---|---|---|---|---|---|---|---|
| 907.4917 | 907.4323 | -0.0594 | -65 | 620 | 627 | ASELTKMK | | | | | Mascot |
| 908.3679 | 908.4072 | 0.0393 | 43 | 394 | 400 | QWDCGSR | | | Carbamidomethyl (C)[4] | | Mascot |
| 1093.5775 | 1093.5359 | -0.0416 | -38 | 648 | 656 | ELELDSLFK | | | | | Mascot |

| | | | | | | | | | | | |
|---|---|---|---|---|---|---|---|---|---|---|---|
| 1343.6624 | 1343.5637 | -0.0987 | -73 | 190 | 200 | YSSNTMKVEIR | | | Oxidation (M)[6] | | Mascot |
| 1343.6624 | 1343.5637 | -0.0987 | -73 | 190 | 200 | YSSNTMKVEIR | | | Oxidation (M)[6] | | Mascot |
| 1359.7089 | 1359.5563 | -0.1526 | -112 | 73 | 84 | CGTQAPVWLSLK | | | Carbamidomethyl (C)[1] | | Mascot |
| 1629.7477 | 1629.7004 | -0.0473 | -29 | 502 | 516 | NSHNDLHHPAGTSSR | | | | | Mascot |
| 1629.7477 | 1629.7004 | -0.0473 | -29 | 502 | 516 | NSHNDLHHPAGTSSR | | | | | Mascot |
| 1714.9296 | 1714.7611 | -0.1685 | -98 | 752 | 766 | LEEAVDLCILDLQLK | | | | | Mascot |
| 1970.884 | 1970.8958 | 0.0118 | 6 | 580 | 595 | CLSYDNTDYTSLFPFK | | | Carbamidomethyl (C)[1] | | Mascot |
| 2108.0085 | 2107.9185 | -0.09 | -43 | 67 | 84 | CVEMNRCGTQAPVWLSLK | | | Carbamidomethyl (C)[1], Oxidation (M)[4] | | Mascot |
| 2166.199 | 2165.895 | -0.304 | -140 | 868 | 887 | VFGLGFIDSSDLACLVIRLK | | | | | Mascot |
| 2210.9641 | 2211.0452 | 0.0811 | 37 | 394 | 413 | QWDCGSRHYAVACTCGVAAR | | | Carbamidomethyl (C)[4] | | Mascot |
| 2215.0171 | 2215.0227 | 0.0056 | 3 | 549 | 566 | WNFCACQKGYSLSLHSTR | | | Carbamidomethyl (C)[4,6] | | Mascot |
| 2351.2534 | 2351.0234 | -0.23 | -98 | 954 | 975 | ILTLYDGICQICVAPSGICKLK | | | | | Mascot |
| 2763.3657 | 2763.2476 | -0.1181 | -43 | 344 | 368 | ITVQDVPTATCYSLTDPHIITFDGR | | | | | Mascot |

| | | | | | | | | | | |
|---|---|---|---|---|---|---|---|---|---|---|
| 16 | VDR-B [Danio rerio] | gi\|68132044 | 47285.6 | 6.81 | 7 | 35 | 0 | 20 | 0 |

**Peptide Information**

| Calc. Mass | Obsrv. Mass | ± da | ± ppm | Start Seq. | End Seq. | Sequence | Ion Score | C. I. % | Modification | Rank | Result Type |
|---|---|---|---|---|---|---|---|---|---|---|---|
| 908.3964 | 908.4072 | 0.0108 | 12 | 19 | 26 | ICGVCGDK | | | Carbamidomethyl (C)[2,5] | | Mascot |
| 1118.6238 | 1118.6045 | -0.0193 | -17 | 259 | 268 | SSAIEVIMLR | | | | | Mascot |
| 1118.6238 | 1118.6045 | -0.0193 | -17 | 259 | 268 | SSAIEVIMLR | 20 | 0 | | | Mascot |
| 1123.5421 | 1123.5106 | -0.0315 | -28 | 79 | 87 | RCLDIGMMK | | | Carbamidomethyl (C)[2] | | Mascot |
| 1343.7417 | 1343.5637 | -0.178 | -132 | 247 | 258 | ELTAEDQIALLK | | | | | Mascot |
| 1343.7417 | 1343.5637 | -0.178 | -132 | 247 | 258 | ELTAEDQIALLK | | | | | Mascot |
| 1375.7329 | 1375.5514 | -0.1815 | -132 | 126 | 137 | NIIDTLVDAHHK | | | | | Mascot |
| 2107.9832 | 2107.9185 | -0.0647 | -31 | 138 | 154 | TYDDSYSDFSRFRPPVR | | | | | Mascot |
| 2123.9248 | 2123.9202 | -0.0046 | -2 | 27 | 45 | ATGFHFNAMTCEGCKGFFR | | | | | Mascot |

| | | | | | | | |
|---|---|---|---|---|---|---|---|
| 17 | PREDICTED: retrotransposable element Tf2 155 kDa protein type 1-like [Danio rerio] | gi\|326663868 | 132660.5 | 9.38 | 15 | 35 | 0 |

**Peptide Information**

| Calc. Mass | Obsrv. Mass | ± da | ± ppm | Start Seq. | End Seq. | Sequence | Ion Score | C. I. % | Modification | Rank | Result Type |
|---|---|---|---|---|---|---|---|---|---|---|---|
| 809.4039 | 809.3594 | -0.0445 | -55 | 153 | 159 | AFSDELK | | | | | Mascot |
| 908.4584 | 908.4072 | -0.0512 | -56 | 791 | 797 | LFDRSDR | | | | | Mascot |
| 1157.646 | 1157.6738 | 0.0278 | 24 | 1140 | 1149 | NIPLRTVCNK | | | | | Mascot |

| Calc. Mass | Obsrv. Mass | ± da | ± ppm | Start Seq. | End Seq. | Sequence | Ion Score | C. I. % | Modification | Rank | Result Type |
|---|---|---|---|---|---|---|---|---|---|---|---|
| 1375.7004 | 1375.5514 | -0.149 | -108 | 133 | 144 | AALWGTTVWENK | | | | | Mascot |
| 1492.7502 | 1492.7264 | -0.0238 | -16 | 160 | 173 | VVFDQAASGREASR | | | | | Mascot |
| 1492.7502 | 1492.7264 | -0.0238 | -16 | 160 | 173 | VVFDQAASGREASR | | | | | Mascot |
| 1626.9148 | 1626.7129 | -0.2019 | -124 | 859 | 873 | VACHPGVSRTLFVIK | | | | | Mascot |
| 1682.8285 | 1682.7738 | -0.0547 | -33 | 634 | 648 | TPFRWSNAAEAAFSK | | | | | Mascot |
| 1682.9547 | 1682.7738 | -0.1809 | -107 | 230 | 244 | TLEGLINLAVRVDNR | | | | | Mascot |
| 1797.8622 | 1797.9053 | 0.0431 | 24 | 884 | 899 | DVRDFVLACSVCAASK | | | Carbamidomethyl (C)[9,12] | | Mascot |
| 1797.8622 | 1797.9053 | 0.0431 | 24 | 884 | 899 | DVRDFVLACSVCAASK | | | Carbamidomethyl (C)[9,12] | | Mascot |
| 2099.0227 | 2098.9607 | -0.062 | -30 | 582 | 599 | MDPEKIQAVVNWPTPDS R | | | Oxidation (M)[1] | | Mascot |
| 2108.1499 | 2107.9185 | -0.2314 | -110 | 615 | 633 | FIRNFSQLAAPLTNLTSSK | | | | | Mascot |
| 2221.0818 | 2221.0967 | 0.0149 | 7 | 79 | 98 | SSEPRLPPPQSYAGEPH LCR | | | | | Mascot |
| 2225.0122 | 2225.0618 | 0.0496 | 22 | 295 | 315 | AGGLCMYCGVGGHFVAL CPAK | | | Carbamidomethyl (C)[5,8,18] | | Mascot |
| 2283.0652 | 2283.1169 | 0.0517 | 23 | 294 | 315 | RAGGLCMYCGVGGHFV ALCPAK | | | Carbamidomethyl (C)[6], Oxidation (M)[7] | | Mascot |
| 2370.1006 | 2370.1721 | 0.0715 | 30 | 133 | 152 | AALWGTTVWENKSPCCN TFK | | | Carbamidomethyl (C)[15,16] | | Mascot |

| | | | | | | | |
|---|---|---|---|---|---|---|---|
| 18 | novel protein similar to human adrenergic receptor kinase [Danio rerio] | gi\|29561781 | 68278.7 | 8.24 | 12 | 35 | 0 |

**Peptide Information**

| Calc. Mass | Obsrv. Mass | ± da | ± ppm | Start Seq. | End Seq. | Sequence | Ion Score | C. I. % | Modification | Rank | Result Type |
|---|---|---|---|---|---|---|---|---|---|---|---|
| 809.4403 | 809.3594 | -0.0809 | -100 | 1 | 6 | IKEYEK | | | | | Mascot |
| 1093.5649 | 1093.5359 | -0.029 | -27 | 299 | 307 | GHSPFRQHK | | | | | Mascot |
| 1118.5043 | 1118.6045 | 0.1002 | 90 | 128 | 136 | MYAMKCLDK | | | Oxidation (M)[1] | | Mascot |
| 1118.5043 | 1118.6045 | 0.1002 | 90 | 128 | 136 | MYAMKCLDK | | | Oxidation (M)[1] | | Mascot |
| 1242.5316 | 1242.5757 | 0.0441 | 35 | 461 | 470 | DCIMHGYMLK | | | Oxidation (M)[4,8] | | Mascot |
| 1325.6406 | 1325.6576 | 0.017 | 13 | 245 | 256 | ISDLGLACDFSK | | | Carbamidomethyl (C)[8] | | Mascot |
| 1359.7168 | 1359.5563 | -0.1605 | -118 | 471 | 481 | LGNPFLTQWQR | | | | | Mascot |
| 1375.7039 | 1375.5514 | -0.1525 | -111 | 28 | 39 | ELLSCSHPFSKK | | | | | Mascot |
| 2098.9248 | 2098.9607 | 0.0359 | 17 | 277 | 295 | GTAYDSSADWFSLGCML FK | | | | | Mascot |
| 2194.1438 | 2194.0261 | -0.1177 | -54 | 257 | 276 | KKPHASVGTHGYMAPEV LQK | | | Oxidation (M)[13] | | Mascot |
| 2225.0696 | 2225.0618 | -0.0078 | -4 | 529 | 547 | GGKQFVLQCESDPEFVQ WK | | | | | Mascot |
| 2474.2095 | 2474.0852 | -0.1243 | -50 | 19 | 38 | QIYDVYIMKELLSCSHPF SK | | | Carbamidomethyl (C)[14], Oxidation (M)[8] | | Mascot |
| 2731.3232 | 2731.2441 | -0.0791 | -29 | 86 | 107 | FCQWKNVELNIHLTMND FSVHR | | | | | Mascot |
| 2731.3232 | 2731.2441 | -0.0791 | -29 | 86 | 107 | FCQWKNVELNIHLTMND FSVHR | | | | | Mascot |
| 2747.3181 | 2747.2437 | -0.0744 | -27 | 86 | 107 | FCQWKNVELNIHLTMND | | | Oxidation (M)[15] | | Mascot |

FSVHR

| | | | | | | | |
|---|---|---|---|---|---|---|---|
| 19 | PREDICTED: retrotransposable element Tf2 155 kDa protein type 1-like [Danio rerio] | gi\|326673018 | 132510.4 | 9.38 | 15 | 34 | 0 |

Peptide Information

| Calc. Mass | Obsrv. Mass | ± da | ± ppm | Start Seq. | End Seq. | Sequence | Ion Score | C. I. % | Modification | Rank | Result Type |
|---|---|---|---|---|---|---|---|---|---|---|---|
| 809.4039 | 809.3594 | -0.0445 | -55 | 153 | 159 | AFSDELK | | | | | Mascot |
| 908.4584 | 908.4072 | -0.0512 | -56 | 791 | 797 | LFDRSDR | | | | | Mascot |
| 1157.646 | 1157.6738 | 0.0278 | 24 | 1140 | 1149 | NIPLRTVCNK | | | | | Mascot |
| 1375.7004 | 1375.5514 | -0.149 | -108 | 133 | 144 | AALWGTTVWENK | | | | | Mascot |
| 1492.7502 | 1492.7264 | -0.0238 | -16 | 160 | 173 | VVFDQAASGREASR | | | | | Mascot |
| 1492.7502 | 1492.7264 | -0.0238 | -16 | 160 | 173 | VVFDQAASGREASR | | | | | Mascot |
| 1626.9148 | 1626.7129 | -0.2019 | -124 | 859 | 873 | VACHPGVSRTLFVIK | | | | | Mascot |
| 1682.8285 | 1682.7738 | -0.0547 | -33 | 634 | 648 | TPFRWSNAAEAAFSK | | | | | Mascot |
| 1682.9547 | 1682.7738 | -0.1809 | -107 | 230 | 244 | TLEGLINLAVRVDNR | | | | | Mascot |
| 1797.8622 | 1797.9053 | 0.0431 | 24 | 884 | 899 | DVRDFVLACSVCAASK | | | Carbamidomethyl (C)[9,12] | | Mascot |
| 1797.8622 | 1797.9053 | 0.0431 | 24 | 884 | 899 | DVRDFVLACSVCAASK | | | Carbamidomethyl (C)[9,12] | | Mascot |
| 2099.0227 | 2098.9607 | -0.062 | -30 | 582 | 599 | MDPEKIQAVVNWPTPDSR | | | Oxidation (M)[1] | | Mascot |
| 2108.1499 | 2107.9185 | -0.2314 | -110 | 615 | 633 | FIRNFSQLAAPLTNLTSSK | | | | | Mascot |
| 2221.0818 | 2221.0967 | 0.0149 | 7 | 79 | 98 | SSEPRLPPPQSYAGEPHLCR | | | | | Mascot |
| 2225.0122 | 2225.0618 | 0.0496 | 22 | 295 | 315 | AGGLCMYCGVGGHFVALCPAK | | | Carbamidomethyl (C)[5,8,18] | | Mascot |
| 2283.0652 | 2283.1169 | 0.0517 | 23 | 294 | 315 | RAGGLCMYCGVGGHFVALCPAK | | | Carbamidomethyl (C)[6], Oxidation (M)[7] | | Mascot |
| 2370.1006 | 2370.1721 | 0.0715 | 30 | 133 | 152 | AALWGTTVWENKSPCCNTFK | | | Carbamidomethyl (C)[15,16] | | Mascot |

| | | | | | | | |
|---|---|---|---|---|---|---|---|
| 20 | ATP-binding cassette, sub-family B (MDR/TAP), member 3 like 1 [Danio rerio] | gi\|54633310 | 81688.4 | 8.72 | 12 | 34 | 0 |

Peptide Information

| Calc. Mass | Obsrv. Mass | ± da | ± ppm | Start Seq. | End Seq. | Sequence | Ion Score | C. I. % | Modification | Rank | Result Type |
|---|---|---|---|---|---|---|---|---|---|---|---|
| 809.3895 | 809.3594 | -0.0301 | -37 | 383 | 389 | MTELGMK | | | | | Mascot |
| 965.4907 | 965.4443 | -0.0464 | -48 | 382 | 389 | RMTELGMK | | | | | Mascot |
| 996.6676 | 996.5723 | -0.0953 | -96 | 616 | 624 | IAIARALIR | | | | | Mascot |
| 1242.6624 | 1242.5757 | -0.0867 | -70 | 360 | 369 | LMETHNLKTR | | | | | Mascot |
| 1258.6572 | 1258.5682 | -0.089 | -71 | 360 | 369 | LMETHNLKTR | | | Oxidation (M)[2] | | Mascot |
| 1492.7213 | 1492.7264 | 0.0051 | 3 | 356 | 367 | YDGRLMETHNLK | | | Oxidation (M)[6] | | Mascot |
| 1492.7213 | 1492.7264 | 0.0051 | 3 | 356 | 367 | YDGRLMETHNLK | | | Oxidation (M)[6] | | Mascot |

| | | | | | | | | | | | |
|---|---|---|---|---|---|---|---|---|---|---|---|
| 1629.6996 | 1629.7004 | 0.0008 | 0 | 563 | 576 | DNIAYGLQGCSMER | | | Carbamidomethyl (C)[10], Oxidation (M)[12] | | Mascot |
| 1629.6996 | 1629.7004 | 0.0008 | 0 | 563 | 576 | DNIAYGLQGCSMER | | | Carbamidomethyl (C)[10], Oxidation (M)[12] | | Mascot |
| 1819.8651 | 1819.8815 | 0.0164 | 9 | 212 | 227 | GGLFMCAINSFTCRVK | | | Carbamidomethyl (C)[6], Oxidation (M)[5] | | Mascot |
| 1842.9973 | 1842.7349 | -0.2624 | -142 | 474 | 488 | LTFSYPRRPDHNVLK | | | | | Mascot |
| 2099.1643 | 2098.9607 | -0.2036 | -97 | 275 | 292 | TLVKTLGMLYLMVSLSWK | | | Oxidation (M)[8] | | Mascot |
| 2108.0303 | 2107.9185 | -0.1118 | -53 | 192 | 211 | SAIIFMGLFSLGSSFSAGCR | | | Carbamidomethyl (C)[19] | | Mascot |
| 2124.0251 | 2123.9202 | -0.1049 | -49 | 192 | 211 | SAIIFMGLFSLGSSFSAGCR | | | Carbamidomethyl (C)[19], Oxidation (M)[6] | | Mascot |
| 2211.2173 | 2211.0452 | -0.1721 | -78 | 228 | 246 | VQLFGSLIRQDIGFFETIK | | | | | Mascot |
| 2221.1533 | 2221.0967 | -0.0566 | -25 | 489 | 510 | DFSLELKPGQITALVGMSGGGK | | | Oxidation (M)[17] | | Mascot |

21 PREDICTED: zinc finger protein 658B [Danio rerio] gi|326667382 85432.5 9.09 16 34 0

**Peptide Information**

| Calc. Mass | Obsrv. Mass | ± da | ± ppm | Start Seq. | End Seq. | Sequence | Ion Score | C. I. % | Modification | Rank | Result Type |
|---|---|---|---|---|---|---|---|---|---|---|---|
| 809.4264 | 809.3594 | -0.067 | -83 | 452 | 457 | FTERTR | | | | | Mascot |
| 891.4053 | 891.3801 | -0.0252 | -28 | 6 | 12 | EESEELR | | | | | Mascot |
| 1045.5282 | 1045.5388 | 0.0106 | 10 | 459 | 467 | LHMAKQACK | | | Oxidation (M)[3] | | Mascot |
| 1118.5687 | 1118.6045 | 0.0358 | 32 | 486 | 494 | EESEDLRIK | | | | | Mascot |
| 1118.5687 | 1118.6045 | 0.0358 | 32 | 484 | 492 | IKEESEDLR | 11 | 0 | | | Mascot |
| 1221.6521 | 1221.5453 | -0.1068 | -87 | 584 | 593 | IHMRIHTGEK | | | | | Mascot |
| 1221.6521 | 1221.5453 | -0.1068 | -87 | 584 | 593 | IHMRIHTGEK | | | | | Mascot |
| 1359.6686 | 1359.5563 | -0.1123 | -83 | 99 | 110 | IHTGEKLSTCDR | | | | | Mascot |
| 1698.7838 | 1698.765 | -0.0188 | -11 | 155 | 169 | IHSGEKPFACQHCGK | | | Carbamidomethyl (C)[10] | | Mascot |
| 1714.7676 | 1714.7611 | -0.0065 | -4 | 594 | 607 | LFTCDQCGKSFSHK | | | Carbamidomethyl (C)[4,7] | | Mascot |
| 1797.8635 | 1797.9053 | 0.0418 | 23 | 155 | 170 | IHSGEKPFACQHCGKR | 3 | 0 | | | Mascot |
| 1797.8635 | 1797.9053 | 0.0418 | 23 | 155 | 170 | IHSGEKPFACQHCGKR | | | | | Mascot |
| 1819.8318 | 1819.8815 | 0.0497 | 27 | 468 | 483 | SGHNNNPQDLPNEDLR | | | | | Mascot |
| 2189.0161 | 2188.9788 | -0.0373 | -17 | 349 | 367 | VHMRVHTGERPYTCAQCGK | | | Oxidation (M)[3] | | Mascot |
| 2220.9695 | 2221.0967 | 0.1272 | 57 | 291 | 308 | HMRVHTGERPYSCTECGK | | | Carbamidomethyl (C)[13,16], Oxidation (M)[2] | | Mascot |
| 2225.0591 | 2225.0618 | 0.0027 | 1 | 207 | 225 | SHIIRHTGEKPFPCDQCEK | | | | | Mascot |
| 2228.9998 | 2229.0354 | 0.0356 | 16 | 644 | 663 | VHSGERPYTCQQCGKSFACK | | | | | Mascot |
| 2228.9998 | 2229.0354 | 0.0356 | 16 | 644 | 663 | VHSGERPYTCQQCGKSFACK | | | | | Mascot |
| 2284.0598 | 2284.1128 | 0.053 | 23 | 183 | 202 | VHTGERPYTCQQCGKSFSQK | | | | | Mascot |

22 PREDICTED: MICAL-like 1 [Danio rerio] gi|326668603 96087.1 6.54 12 34 0

Peptide Information

| Calc. Mass | Obsrv. Mass | ± da | ± ppm | Start Seq. | End Seq. | Sequence | Ion Score | C. I. % | Modification | Rank | Result Type |
|---|---|---|---|---|---|---|---|---|---|---|---|
| 842.4301 | 842.4899 | 0.0598 | 71 | 376 | 382 | MHSPARK | | | Oxidation (M)[1] | | Mascot |
| 891.4141 | 891.3801 | -0.034 | -38 | 6 | 12 | ALQDWCR | | | | | Mascot |
| 1045.4697 | 1045.5388 | 0.0691 | 66 | 827 | 835 | DFHGQGEQK | | | | | Mascot |
| 1165.5684 | 1165.7151 | 0.1467 | 126 | 180 | 187 | LYHRNCFR | | | Carbamidomethyl (C)[6] | | Mascot |
| 1183.6793 | 1183.5306 | -0.1487 | -126 | 606 | 617 | SARPPSLSAGLK | | | | | Mascot |
| 1258.5402 | 1258.5682 | 0.028 | 22 | 155 | 166 | STLSSTCSACGK | | | Carbamidomethyl (C)[7,10] | | Mascot |
| 1492.7754 | 1492.7264 | -0.049 | -33 | 642 | 655 | TTTSANSFVSRPPK | | | | | Mascot |
| 1492.7754 | 1492.7264 | -0.049 | -33 | 642 | 655 | TTTSANSFVSRPPK | | | | | Mascot |
| 1819.8894 | 1819.8815 | -0.0079 | -4 | 767 | 781 | CLLNKPEKEWTSEDK | | | | | Mascot |
| 2189.9978 | 2189.9678 | -0.03 | -14 | 236 | 255 | LTTSAEDPLCGNDSEISP QR | | | Carbamidomethyl (C)[10] | | Mascot |
| 2189.9978 | 2189.9678 | -0.03 | -14 | 236 | 255 | LTTSAEDPLCGNDSEISP QR | | | Carbamidomethyl (C)[10] | | Mascot |
| 2410.2573 | 2410.2312 | -0.0261 | -11 | 557 | 579 | SVSEPSINTPASALSQKA ELQPR | | | | | Mascot |
| 2474.1755 | 2474.0852 | -0.0903 | -36 | 676 | 696 | VQSDQFIPVEDIHVEMSD LEK | | | Oxidation (M)[16] | | Mascot |
| 2747.3611 | 2747.2437 | -0.1174 | -43 | 194 | 217 | TLLPGSYKFTEDPGALVC THHFTR | | | Carbamidomethyl (C)[18] | | Mascot |

23 Transferrin-a [Danio rerio] gi|159155968 56844.4 5.78 7 34 0 18 0

Peptide Information

| Calc. Mass | Obsrv. Mass | ± da | ± ppm | Start Seq. | End Seq. | Sequence | Ion Score | C. I. % | Modification | Rank | Result Type |
|---|---|---|---|---|---|---|---|---|---|---|---|
| 1221.5793 | 1221.5453 | -0.034 | -28 | 93 | 102 | TCNFAREPAR | | | Carbamidomethyl (C)[2] | | Mascot |
| 1221.5793 | 1221.5453 | -0.034 | -28 | 93 | 102 | TCNFAREPAR | 18 | 0 | Carbamidomethyl (C)[2] | | Mascot |
| 1359.6586 | 1359.5563 | -0.1023 | -75 | 289 | 301 | SCHTGLGRSAGWK | | | | | Mascot |
| 1492.8005 | 1492.7264 | -0.0741 | -50 | 498 | 510 | TYKTSQNVPDLVK | | | | | Mascot |
| 1492.8005 | 1492.7264 | -0.0741 | -50 | 498 | 510 | TYKTSQNVPDLVK | | | | | Mascot |
| 1629.736 | 1629.7004 | -0.0356 | -22 | 74 | 86 | QNYELLCMDGSRK | | | Carbamidomethyl (C)[7], Oxidation (M)[8] | | Mascot |
| 1629.736 | 1629.7004 | -0.0356 | -22 | 74 | 86 | QNYELLCMDGSRK | | | Carbamidomethyl (C)[7], Oxidation (M)[8] | | Mascot |
| 2189.9124 | 2189.9678 | 0.0554 | 25 | 319 | 339 | FFSEGCAPGADPTSNMC KPCK | | | | | Mascot |
| 2189.9124 | 2189.9678 | 0.0554 | 25 | 319 | 339 | FFSEGCAPGADPTSNMC KPCK | | | | | Mascot |
| 2357.1223 | 2357.124 | 0.0017 | 1 | 173 | 195 | DGNPSAPTSQTKLAMCTI GHAEK | | | | | Mascot |
| 2370.084 | 2370.1721 | 0.0881 | 37 | 398 | 417 | DLKSEDFELICPNTPDTT MK | | | Carbamidomethyl (C)[11], Oxidation (M)[19] | | Mascot |

24 novel protein [Danio rerio] gi|220679558 45035 4.99 8 34 0

Peptide Information

| Calc. Mass | Obsrv. Mass | ± da | ± ppm | Start Seq. | End Seq. | Sequence | Ion Score | C. I. % | Modification | Rank | Result Type |
|---|---|---|---|---|---|---|---|---|---|---|---|
| 842.473 | 842.4899 | 0.0169 | 20 | 382 | 388 | DPKDVLR | | | | | Mascot |
| 882.4791 | 882.5447 | 0.0656 | 74 | 1 | 7 | VSTYTRR | | | | | Mascot |
| 1045.5889 | 1045.5388 | -0.0501 | -48 | 54 | 62 | VIDTLESLR | | | | | Mascot |
| 1242.5541 | 1242.5757 | 0.0216 | 17 | 7 | 16 | RCPGCQMIYR | | | Oxidation (M)[7] | | Mascot |
| 2098.9644 | 2098.9607 | -0.0037 | -2 | 220 | 237 | DCNVDSNGSAFDMILRL R | | | Carbamidomethyl (C)[2], Oxidation (M)[13] | | Mascot |
| 2214.9939 | 2215.0227 | 0.0288 | 13 | 217 | 235 | LCKDCNVDSNGSAFDMIL R | | | Carbamidomethyl (C)[2,5] | | Mascot |
| 2370.0708 | 2370.1721 | 0.1013 | 43 | 253 | 274 | ICGSSGGCSVVMCPHGV VYSLK | | | Carbamidomethyl (C)[2,8,13], Oxidation (M)[12] | | Mascot |
| 2731.3074 | 2731.2441 | -0.0633 | -23 | 249 | 274 | IYQKICGSSGGCSVVMCP HGVVYSLK | | | Oxidation (M)[16] | | Mascot |
| 2731.3074 | 2731.2441 | -0.0633 | -23 | 249 | 274 | IYQKICGSSGGCSVVMCP HGVVYSLK | | | Oxidation (M)[16] | | Mascot |

| | | | | | | | | | | |
|---|---|---|---|---|---|---|---|---|---|---|
| 25 | Transferrin-a [Danio rerio] | gi\|68085326 | 56868.3 | 5.58 | 7 | 33 | 0 | 18 | 0 | |

Peptide Information

| Calc. Mass | Obsrv. Mass | ± da | ± ppm | Start Seq. | End Seq. | Sequence | Ion Score | C. I. % | Modification | Rank | Result Type |
|---|---|---|---|---|---|---|---|---|---|---|---|
| 1221.5793 | 1221.5453 | -0.034 | -28 | 93 | 102 | TCNFAREPAR | | | Carbamidomethyl (C)[2] | | Mascot |
| 1221.5793 | 1221.5453 | -0.034 | -28 | 93 | 102 | TCNFAREPAR | 18 | 0 | Carbamidomethyl (C)[2] | | Mascot |
| 1359.6586 | 1359.5563 | -0.1023 | -75 | 289 | 301 | SCHTGLGRSAGWK | | | | | Mascot |
| 1492.8005 | 1492.7264 | -0.0741 | -50 | 498 | 510 | TYKTSQNVPDLVK | | | | | Mascot |
| 1492.8005 | 1492.7264 | -0.0741 | -50 | 498 | 510 | TYKTSQNVPDLVK | | | | | Mascot |
| 1629.736 | 1629.7004 | -0.0356 | -22 | 74 | 86 | QNYELLCMDGSRK | | | Carbamidomethyl (C)[7], Oxidation (M)[8] | | Mascot |
| 1629.736 | 1629.7004 | -0.0356 | -22 | 74 | 86 | QNYELLCMDGSRK | | | Carbamidomethyl (C)[7], Oxidation (M)[8] | | Mascot |
| 2141.0002 | 2141.0518 | 0.0516 | 24 | 501 | 520 | TSQNVPDLVKACTFGNCI SS | | | Carbamidomethyl (C)[12] | | Mascot |
| 2357.1223 | 2357.124 | 0.0017 | 1 | 173 | 195 | DGNPSAPTSQTKLAMCTI GHAEK | | | | | Mascot |
| 2370.084 | 2370.1721 | 0.0881 | 37 | 398 | 417 | DLKSEDFELICPNTPDTT MK | | | Carbamidomethyl (C)[11], Oxidation (M)[19] | | Mascot |

| | | | | | | | | |
|---|---|---|---|---|---|---|---|---|
| 26 | PREDICTED: cytoplasmic FMR1-interacting protein 2-like [Danio rerio] | gi\|326673718 | 62081 | 7.56 | 10 | 33 | 0 | |

Peptide Information

| Calc. Mass | Obsrv. Mass | ± da | ± ppm | Start Seq. | End Seq. | Sequence | Ion Score | C. I. % | Modification | Rank | Result Type |
|---|---|---|---|---|---|---|---|---|---|---|---|
| 965.505 | 965.4443 | -0.0607 | -63 | 276 | 283 | TDVFQSLR | | | | | Mascot |
| 1042.5469 | 1042.557 | 0.0101 | 10 | 192 | 200 | NFVGPPHFK | | | | | Mascot |
| 1045.5524 | 1045.5388 | -0.0136 | -13 | 475 | 483 | QDGKDEIIK | | | | | Mascot |

| Calc. Mass | Obsrv. Mass | ± da | ± ppm | Start Seq. | End Seq. | Sequence | Ion Score | C. I. % | Modification | Rank | Result Type |
|---|---|---|---|---|---|---|---|---|---|---|---|
| 1093.5775 | 1093.5359 | -0.0416 | -38 | 267 | 275 | DIIEYAELK | | | | | Mascot |
| 1165.5895 | 1165.7151 | 0.1256 | 108 | 25 | 34 | CGVQARFTAR | | | Carbamidomethyl (C)[1] | | Mascot |
| 1359.7487 | 1359.5563 | -0.1924 | -141 | 236 | 246 | TLIEVMPKICR | | | Carbamidomethyl (C)[10] | | Mascot |
| 1375.7435 | 1375.5514 | -0.1921 | -140 | 236 | 246 | TLIEVMPKICR | | | Carbamidomethyl (C)[10], Oxidation (M)[6] | | Mascot |
| 1626.871 | 1626.7129 | -0.1581 | -97 | 61 | 74 | FVHERSVIAALNDR | | | | | Mascot |
| 1698.8375 | 1698.765 | -0.0725 | -43 | 372 | 385 | LCCGLSMFEVILTR | | | Carbamidomethyl (C)[2,3] | | Mascot |
| 1714.8324 | 1714.7611 | -0.0713 | -42 | 372 | 385 | LCCGLSMFEVILTR | | | Carbamidomethyl (C)[2,3], Oxidation (M)[7] | | Mascot |
| 2190.0859 | 2189.9678 | -0.1181 | -54 | 35 | 55 | GGVSEIECPELDFAAARA LNK | | | | | Mascot |
| 2190.0859 | 2189.9678 | -0.1181 | -54 | 35 | 55 | GGVSEIECPELDFAAARA LNK | | | | | Mascot |
| 2306.261 | 2306.0447 | -0.2163 | -94 | 201 | 220 | TICRLLGYQGIAVVMEELL K | | | Carbamidomethyl (C)[3] | | Mascot |

| | | | | | | | | | | |
|---|---|---|---|---|---|---|---|---|---|---|
| 27 | Transferrin-a [Danio rerio] | gi\|112180541 | 56852.3 | 5.58 | 7 | 33 | 0 | 18 | 0 | |

**Peptide Information**

| Calc. Mass | Obsrv. Mass | ± da | ± ppm | Start Seq. | End Seq. | Sequence | Ion Score | C. I. % | Modification | Rank | Result Type |
|---|---|---|---|---|---|---|---|---|---|---|---|
| 1221.5793 | 1221.5453 | -0.034 | -28 | 93 | 102 | TCNFAREPAR | | | Carbamidomethyl (C)[2] | | Mascot |
| 1221.5793 | 1221.5453 | -0.034 | -28 | 93 | 102 | TCNFAREPAR | 18 | 0 | Carbamidomethyl (C)[2] | | Mascot |
| 1359.6586 | 1359.5563 | -0.1023 | -75 | 289 | 301 | SCHTGLGRSAGWK | | | | | Mascot |
| 1492.8005 | 1492.7264 | -0.0741 | -50 | 498 | 510 | TYKTSQNVPDLVK | | | | | Mascot |
| 1492.8005 | 1492.7264 | -0.0741 | -50 | 498 | 510 | TYKTSQNVPDLVK | | | | | Mascot |
| 1629.736 | 1629.7004 | -0.0356 | -22 | 74 | 86 | QNYELLCMDGSRK | | | Carbamidomethyl (C)[7], Oxidation (M)[8] | | Mascot |
| 1629.736 | 1629.7004 | -0.0356 | -22 | 74 | 86 | QNYELLCMDGSRK | | | Carbamidomethyl (C)[7], Oxidation (M)[8] | | Mascot |
| 2141.0002 | 2141.0518 | 0.0516 | 24 | 501 | 520 | TSQNVPDLVKACTFGNCI SS | | | Carbamidomethyl (C)[12] | | Mascot |
| 2357.1223 | 2357.124 | 0.0017 | 1 | 173 | 195 | DGNPSAPTSQTKLAMCTI GHAEK | | | | | Mascot |
| 2370.084 | 2370.1721 | 0.0881 | 37 | 398 | 417 | DLKSEDFELICPNTPDTT MK | | | Carbamidomethyl (C)[11], Oxidation (M)[19] | | Mascot |

| | | | | | | | |
|---|---|---|---|---|---|---|---|
| 28 | PREDICTED: retrotransposable element Tf2 155 kDa protein type 1-like [Danio rerio] | gi\|326680732 | 128413.4 | 9.38 | 14 | 33 | 0 |

**Peptide Information**

| Calc. Mass | Obsrv. Mass | ± da | ± ppm | Start Seq. | End Seq. | Sequence | Ion Score | C. I. % | Modification | Rank | Result Type |
|---|---|---|---|---|---|---|---|---|---|---|---|
| 809.4039 | 809.3594 | -0.0445 | -55 | 115 | 121 | AFSDELK | | | | | Mascot |
| 908.4584 | 908.4072 | -0.0512 | -56 | 753 | 759 | LFDRSDR | | | | | Mascot |
| 1157.646 | 1157.6738 | 0.0278 | 24 | 1102 | 1111 | NIPLRTVCNK | | | | | Mascot |
| 1375.7004 | 1375.5514 | -0.149 | -108 | 95 | 106 | AALWGTTVWENK | | | | | Mascot |
| 1492.7502 | 1492.7264 | -0.0238 | -16 | 122 | 135 | VVFDQAASGREASR | | | | | Mascot |

| | | | | | | | | | | | | |
|---|---|---|---|---|---|---|---|---|---|---|---|---|
| 1492.7502 | 1492.7264 | -0.0238 | -16 | 122 | 135 | VVFDQAASGREASR | | | | | | Mascot |
| 1626.9148 | 1626.7129 | -0.2019 | -124 | 821 | 835 | VACHPGVSRTLFVIK | | | | | | Mascot |
| 1682.8285 | 1682.7738 | -0.0547 | -33 | 596 | 610 | TPFRWSNAAEAAFSK | | | | | | Mascot |
| 1682.9547 | 1682.7738 | -0.1809 | -107 | 192 | 206 | TLEGLINLAVRVDNR | | | | | | Mascot |
| 1797.8622 | 1797.9053 | 0.0431 | 24 | 846 | 861 | DVRDFVLACSVCAASK | | | Carbamidomethyl (C)[9,12] | | | Mascot |
| 1797.8622 | 1797.9053 | 0.0431 | 24 | 846 | 861 | DVRDFVLACSVCAASK | | | Carbamidomethyl (C)[9,12] | | | Mascot |
| 2099.0227 | 2098.9607 | -0.062 | -30 | 544 | 561 | MDPEKIQAVVNWPTPDS R | | | Oxidation (M)[1] | | | Mascot |
| 2108.1499 | 2107.9185 | -0.2314 | -110 | 577 | 595 | FIRNFSQLAAPLTNLTSSK | | | | | | Mascot |
| 2225.0122 | 2225.0618 | 0.0496 | 22 | 257 | 277 | AGGLCMYCGVGGHFVAL CPAK | | | Carbamidomethyl (C)[5,8,18] | | | Mascot |
| 2283.0652 | 2283.1169 | 0.0517 | 23 | 256 | 277 | RAGGLCMYCGVGGHFV ALCPAK | | | Carbamidomethyl (C)[6], Oxidation (M)[7] | | | Mascot |
| 2370.1006 | 2370.1721 | 0.0715 | 30 | 95 | 114 | AALWGTTVWENKSPCCN TFK | | | Carbamidomethyl (C)[15,16] | | | Mascot |

| | | | | | | | |
|---|---|---|---|---|---|---|---|
| 29 | PREDICTED: transposon TX1 uncharacterized 149 kDa protein-like, partial [Danio rerio] | gi\|326664779 | 131029.7 | 9.39 | 16 | 32 | 0 |

**Peptide Information**

| Calc. Mass | Obsrv. Mass | ± da | ± ppm | Start Seq. | End Seq. | Sequence | Ion Score | C. I. % | Modification | Rank | Result Type |
|---|---|---|---|---|---|---|---|---|---|---|---|
| 809.3973 | 809.3594 | -0.0379 | -47 | 179 | 184 | LVDCFR | | | Carbamidomethyl (C)[4] | | Mascot |
| 882.4468 | 882.5447 | 0.0979 | 111 | 50 | 55 | YTKWER | | | | | Mascot |
| 1157.6313 | 1157.6738 | 0.0425 | 37 | 752 | 761 | DDHFKVLGVK | | | | | Mascot |
| 1199.5878 | 1199.5233 | -0.0645 | -54 | 176 | 184 | DFKLVDCFR | | | Carbamidomethyl (C)[7] | | Mascot |
| 1325.6995 | 1325.6576 | -0.0419 | -32 | 1110 | 1120 | RMIATTLQGYR | | | Oxidation (M)[2] | | Mascot |
| 1343.6677 | 1343.5637 | -0.104 | -77 | 283 | 292 | AHWWEMVKTR | | | | | Mascot |
| 1343.6677 | 1343.5637 | -0.104 | -77 | 283 | 292 | AHWWEMVKTR | | | | | Mascot |
| 1359.6626 | 1359.5563 | -0.1063 | -78 | 283 | 292 | AHWWEMVKTR | | | Oxidation (M)[6] | | Mascot |
| 1367.6525 | 1367.5828 | -0.0697 | -51 | 708 | 718 | RAMDLTDWFGR | | | | | Mascot |
| 1375.6899 | 1375.5514 | -0.1385 | -101 | 179 | 189 | LVDCFRTTHQR | | | | | Mascot |
| 1797.8264 | 1797.9053 | 0.0789 | 44 | 124 | 137 | YELFEDFQPHMLGR | | | Oxidation (M)[11] | | Mascot |
| 1797.8264 | 1797.9053 | 0.0789 | 44 | 124 | 137 | YELFEDFQPHMLGR | | | Oxidation (M)[11] | | Mascot |
| 1842.9974 | 1842.7349 | -0.2625 | -142 | 609 | 624 | MGFPGKFIAWVGLLYK | | | Oxidation (M)[1] | | Mascot |
| 1909.9324 | 1909.9938 | 0.0614 | 32 | 251 | 266 | LNCSLLEDKEIAGEYR | | | Carbamidomethyl (C)[3] | | Mascot |
| 1971.1169 | 1970.8958 | -0.2211 | -112 | 802 | 819 | AVILPLMLLVCSVFSPPR | | | Oxidation (M)[7] | | Mascot |
| 2108.0779 | 2107.9185 | -0.1594 | -76 | 519 | 535 | NWRPITLLNCDCKLFSK | | | Carbamidomethyl (C)[10] | | Mascot |
| 2194.1941 | 2194.0261 | -0.168 | -77 | 31 | 49 | TFRSDVLLIQECALPFLTK | | | | | Mascot |
| 2225.051 | 2225.0618 | 0.0108 | 5 | 541 | 560 | MSMVLEDVIHPDQACAV PGR | | | Carbamidomethyl (C)[15] | | Mascot |
| 2229.1445 | 2229.0354 | -0.1091 | -49 | 953 | 971 | ETVSRVPGLTLCEAQQV | | | Carbamidomethyl (C)[12] | | Mascot |

| | | | | | | | | | | | |
|---|---|---|---|---|---|---|---|---|---|---|---|
| 2229.1445 | 2229.0354 | -0.1091 | -49 | 953 | 971 | WR ETVSRVPGLTLCEAQQV WR | | | Carbamidomethyl (C)[12] | | Mascot |

30 inosine triphosphate pyrophosphatase [Danio rerio] gi|153791470 22854.6 5.89 6 32 0

Peptide Information

| Calc. Mass | Obsrv. Mass | ± da | ± ppm | Start Seq. | End Seq. | Sequence | Ion Score | C. I. % | Modification | Rank | Result Type |
|---|---|---|---|---|---|---|---|---|---|---|---|
| 1157.6565 | 1157.6738 | 0.0173 | 15 | 78 | 88 | ALEGLPGPYIK | | | | | Mascot |
| 1242.694 | 1242.5757 | -0.1183 | -95 | 19 | 29 | LEEVVQILGDK | | | | | Mascot |
| 2215.0559 | 2215.0227 | -0.0332 | -15 | 179 | 198 | ALAALSEHFCQDNGAPE TKR | | | Carbamidomethyl (C)[10] | | Mascot |
| 2221.074 | 2221.0967 | 0.0227 | 10 | 58 | 77 | EAARQVDGPVLVEDTCL CFR | | | | | Mascot |
| 2367.0969 | 2367.0222 | -0.0747 | -32 | 102 | 123 | MLAGFEDKSAWALCTFA FCAGK | | | | | Mascot |
| 2474.1995 | 2474.0852 | -0.1143 | -46 | 110 | 131 | SAWALCTFAFCAGKEEP VQLFR | | | | | Mascot |

31 PREDICTED: perforin-1-like [Danio rerio] gi|292624265 63633.9 8.25 11 32 0

Peptide Information

| Calc. Mass | Obsrv. Mass | ± da | ± ppm | Start Seq. | End Seq. | Sequence | Ion Score | C. I. % | Modification | Rank | Result Type |
|---|---|---|---|---|---|---|---|---|---|---|---|
| 882.577 | 882.5447 | -0.0323 | -37 | 85 | 92 | QKLPVAVK | | | | | Mascot |
| 907.4739 | 907.4323 | -0.0416 | -46 | 96 | 103 | TLPKCSMK | | | | | Mascot |
| 908.4472 | 908.4072 | -0.04 | -44 | 192 | 199 | SLPASYDR | | | | | Mascot |
| 1123.5057 | 1123.5106 | 0.0049 | 4 | 364 | 373 | ICSEPCKMGR | | | | | Mascot |
| 1367.6742 | 1367.5828 | -0.0914 | -67 | 449 | 459 | WRDTFDFGPVK | | | | | Mascot |
| 1375.6423 | 1375.5514 | -0.0909 | -66 | 263 | 273 | AEAHFCKEQQK | | | Carbamidomethyl (C)[6] | | Mascot |
| 1682.8384 | 1682.7738 | -0.0646 | -38 | 40 | 54 | YNLAGEGFDVVTLER | | | | | Mascot |
| 1682.8384 | 1682.7738 | -0.0646 | -38 | 40 | 54 | YNLAGEGFDVVTLER | | | | | Mascot |
| 2188.9856 | 2188.9788 | -0.0068 | -3 | 466 | 484 | LCFEVYDADSNWNSDLL GK | | | | | Mascot |
| 2283.1069 | 2283.1169 | 0.01 | 4 | 351 | 370 | AVEEYIIENALMKICSEPC K | | | | | Mascot |
| 2357.25 | 2357.124 | -0.126 | -53 | 204 | 224 | SLIYTYGTHYTTNVKLGG QIK | | | | | Mascot |
| 2763.3486 | 2763.2476 | -0.101 | -37 | 489 | 512 | LRSGVVTDACVFNYGTFF FTYEVK | | | | | Mascot |

32 dnaJ homolog subfamily C member 4 [Danio rerio] gi|47086683 28185 9.24 7 32 0

Peptide Information

| Calc. Mass | Obsrv. Mass | ± da | ± ppm | Start Seq. | End Seq. | Sequence | Ion Score | C. I. % | Modification | Rank | Result Type |
|---|---|---|---|---|---|---|---|---|---|---|---|
| 809.3788 | 809.3594 | -0.0194 | -24 | 94 | 99 | QDYDLR | | | | | Mascot |

| | | | | | | | | | | | |
|---|---|---|---|---|---|---|---|---|---|---|---|
| 1042.6003 | 1042.557 | -0.0433 | -42 | 213 | 220 | QQEILRQK | | | | | Mascot |
| 1157.6062 | 1157.6738 | 0.0676 | 58 | 219 | 227 | QKHAEFLER | | | | | Mascot |
| 1194.6729 | 1194.553 | -0.1199 | -100 | 193 | 202 | VITKIYNESK | | | | | Mascot |
| 1970.8145 | 1970.8958 | 0.0813 | 41 | 112 | 129 | TSSSSSNNPSWEANESMR | | | | | Mascot |
| 2357.0525 | 2357.124 | 0.0715 | 30 | 2 | 18 | QWEAHIRLCQTCFWYCR | | | Carbamidomethyl (C)[9,12] | | Mascot |
| 2410.25 | 2410.2312 | -0.0188 | -8 | 32 | 52 | SQTNYYELLGVKPDATLEQIK | | | | | Mascot |

33 hypothetical protein LOC572032 [Danio rerio] gi|130501553 75267.4 9.23 10 31 0

**Peptide Information**

| Calc. Mass | Obsrv. Mass | ± da | ± ppm | Start Seq. | End Seq. | Sequence | Ion Score | C. I. % | Modification | Rank | Result Type |
|---|---|---|---|---|---|---|---|---|---|---|---|
| 965.5162 | 965.4443 | -0.0719 | -74 | 68 | 75 | VRADYSVR | | | | | Mascot |
| 1042.5211 | 1042.557 | 0.0359 | 34 | 202 | 210 | NRHGCTLNK | | | | | Mascot |
| 1118.6204 | 1118.6045 | -0.0159 | -14 | 382 | 392 | SFIGVLSPNGK | | | | | Mascot |
| 1118.6204 | 1118.6045 | -0.0159 | -14 | 382 | 392 | SFIGVLSPNGK | | | | | Mascot |
| 1221.6045 | 1221.5453 | -0.0592 | -48 | 227 | 237 | NIGNCFKGSPK | | | Carbamidomethyl (C)[5] | | Mascot |
| 1221.6045 | 1221.5453 | -0.0592 | -48 | 227 | 237 | NIGNCFKGSPK | | | Carbamidomethyl (C)[5] | | Mascot |
| 1842.8802 | 1842.7349 | -0.1453 | -79 | 11 | 25 | NNDVFRNTMEAIFQK | | | Oxidation (M)[9] | | Mascot |
| 1909.9865 | 1909.9938 | 0.0073 | 4 | 48 | 65 | GPVPIDSAEGERELEALK | | | | | Mascot |
| 2221.0342 | 2221.0967 | 0.0625 | 28 | 248 | 266 | SSPLNISYESSACFYSPRR | | | Carbamidomethyl (C)[13] | | Mascot |
| 2233.1096 | 2233.0288 | -0.0808 | -36 | 267 | 287 | NSVDVSGVTDQSAWIDVALTR | | | | | Mascot |
| 2306.1121 | 2306.0447 | -0.0674 | -29 | 246 | 265 | LKSSPLNISYESSACFYSPR | | | Carbamidomethyl (C)[15] | | Mascot |
| 2731.3242 | 2731.2441 | -0.0801 | -29 | 145 | 169 | SLSSNGSTLLDVYPSMLNQIGEAYR | | | Oxidation (M)[16] | | Mascot |
| 2731.3242 | 2731.2441 | -0.0801 | -29 | 145 | 169 | SLSSNGSTLLDVYPSMLNQIGEAYR | | | Oxidation (M)[16] | | Mascot |

34 novel protein (zgc:114168) [Danio rerio] gi|220673162 33328.8 5.18 7 31 0

**Peptide Information**

| Calc. Mass | Obsrv. Mass | ± da | ± ppm | Start Seq. | End Seq. | Sequence | Ion Score | C. I. % | Modification | Rank | Result Type |
|---|---|---|---|---|---|---|---|---|---|---|---|
| 965.4549 | 965.4443 | -0.0106 | -11 | 276 | 283 | YCPGVLQW | | | | | Mascot |
| 1045.516 | 1045.5388 | 0.0228 | 22 | 256 | 264 | STELPDVER | | | | | Mascot |
| 1367.757 | 1367.5828 | -0.1742 | -127 | 265 | 275 | LKPYYQSLIDK | | | | | Mascot |
| 1626.8638 | 1626.7129 | -0.1509 | -93 | 112 | 125 | DWFQLVGLIHDVGK | | | | | Mascot |
| 2166.0171 | 2165.895 | -0.1221 | -56 | 167 | 185 | NPALNTEFGIYEPQCGLDK | | | Carbamidomethyl (C)[15] | | Mascot |
| 2215.0271 | 2215.0227 | -0.0044 | -2 | 3 | 21 | VVNMGPDPSLAYRPECH | | | Carbamidomethyl (C)[16], Oxidation (M)[4] | | Mascot |

| | | | | | | | | | | | |
|---|---|---|---|---|---|---|---|---|---|---|---|
| 2233.1399 | 2233.0288 | -0.1111 | -50 | 107 | 125 | EK EHPDKDWFQLVGLIHDV GK | | | | | Mascot |

35 PREDICTED: titin [Danio rerio] gi|189523699 3645668 6.16 68 31 0

Peptide Information

| Calc. Mass | Obsrv. Mass | ± da | ± ppm | Start Seq. | End Seq. | Sequence | Ion Score | C. I. % | Modification | Rank | Result Type |
|---|---|---|---|---|---|---|---|---|---|---|---|
| 809.3675 | 809.3594 | -0.0081 | -10 | 32458 | 32464 | AEAFEDK | | | | | Mascot |
| 842.4981 | 842.4899 | -0.0082 | -10 | 8812 | 8818 | EDILPKK | | | | | Mascot |
| 882.4679 | 882.5447 | 0.0768 | 87 | 2876 | 2882 | YKVSTER | | | | | Mascot |
| 891.3624 | 891.3801 | 0.0177 | 20 | 4693 | 4701 | HGSDSCSAK | | | | | Mascot |
| 907.4301 | 907.4323 | 0.0022 | 2 | 1543 | 1550 | DSGVITCR | | | Carbamidomethyl (C)[7] | | Mascot |
| 908.389 | 908.4072 | 0.0182 | 20 | 1240 | 1247 | CSEGQTAR | | | Carbamidomethyl (C)[1] | | Mascot |
| 965.4509 | 965.4443 | -0.0066 | -7 | 19569 | 19576 | NSCTLGWK | | | Carbamidomethyl (C)[3] | | Mascot |
| 996.591 | 996.5723 | -0.0187 | -19 | 9064 | 9072 | MKAVPLPPK | | | Oxidation (M)[1] | | Mascot |
| 1042.5204 | 1042.557 | 0.0366 | 35 | 27162 | 27169 | ELSEVYFR | | | | | Mascot |
| 1045.5347 | 1045.5388 | 0.0041 | 4 | 7144 | 7153 | CQIGGTPEIK | | | | | Mascot |
| 1093.5524 | 1093.5359 | -0.0165 | -15 | 9592 | 9600 | EDGIEFQKK | | | | | Mascot |
| 1118.6205 | 1118.6045 | -0.016 | -14 | 24180 | 24188 | EKNSILWTK | | | | | Mascot |
| 1118.6238 | 1118.6045 | -0.0193 | -17 | 4857 | 4866 | IACLDIQKSK | 4 | 0 | | | Mascot |
| 1123.4871 | 1123.5106 | 0.0235 | 21 | 20158 | 20166 | LAENVMDCR | | | Carbamidomethyl (C)[8], Oxidation (M)[6] | | Mascot |
| 1157.6525 | 1157.6738 | 0.0213 | 18 | 8082 | 8091 | VPIEESVTRK | | | | | Mascot |
| 1165.6212 | 1165.7151 | 0.0939 | 81 | 8759 | 8768 | ETFSVQVVTR | | | | | Mascot |
| 1183.5697 | 1183.5306 | -0.0391 | -33 | 9922 | 9931 | EMGSLLKMDK | | | Oxidation (M)[2,8] | | Mascot |
| 1194.5923 | 1194.553 | -0.0393 | -33 | 10919 | 10928 | TLEEEMSVIK | | | Oxidation (M)[6] | | Mascot |
| 1199.5613 | 1199.5233 | -0.038 | -32 | 1797 | 1806 | DESYEGMLKK | | | | | Mascot |
| 1221.5304 | 1221.5453 | 0.0149 | 12 | 10260 | 10269 | DKDEDPEMVK | | | Oxidation (M)[8] | | Mascot |
| 1221.6409 | 1221.5453 | -0.0956 | -78 | 4906 | 4915 | ILWKQGMSSR | | | Oxidation (M)[7] | | Mascot |
| 1242.5419 | 1242.5757 | 0.0338 | 27 | 5340 | 5350 | YTCQVSNDAGK | | | Carbamidomethyl (C)[3] | | Mascot |
| 1258.595 | 1258.5682 | -0.0268 | -21 | 2987 | 2998 | SDAGEYTFIAGK | | | | | Mascot |
| 1325.6888 | 1325.6576 | -0.0312 | -24 | 30094 | 30103 | EILGYWIEFR | | | | | Mascot |
| 1343.6698 | 1343.5637 | -0.1061 | -79 | 5309 | 5320 | EMLTGGTCFIKK | | | Oxidation (M)[2] | | Mascot |
| 1343.6698 | 1343.5637 | -0.1061 | -79 | 5309 | 5320 | EMLTGGTCFIKK | | | Oxidation (M)[2] | | Mascot |
| 1359.6328 | 1359.5563 | -0.0765 | -56 | 31229 | 31239 | NTEVTWYFGSR | | | | | Mascot |
| 1367.6406 | 1367.5828 | -0.0578 | -42 | 6380 | 6392 | AGSCECSGTLRVK | | | Carbamidomethyl (C)[4] | | Mascot |
| 1375.5769 | 1375.5514 | -0.0255 | -19 | 31219 | 31228 | FVCCIENYDR | | | Carbamidomethyl (C)[3,4] | | Mascot |
| 1391.6914 | 1391.7058 | 0.0144 | 10 | 2956 | 2967 | NGVEIQFSVDQR | | | | | Mascot |

| | | | | | | | | | | |
|---|---|---|---|---|---|---|---|---|---|---|
| 1492.7061 | 1492.7264 | 0.0203 | 14 | 6635 | 6649 | ATNNVGSDLCQGSVK | | | | Mascot |
| 1492.7166 | 1492.7264 | 0.0098 | 7 | 28671 | 28683 | DEYTPPKAELDSK | | | | Mascot |
| 1626.7209 | 1626.7129 | -0.008 | -5 | 4784 | 4798 | ASNSVGTATCCTELR | | | Carbamidomethyl (C)[10,11] | Mascot |
| 1629.7809 | 1629.7004 | -0.0805 | -49 | 17093 | 17104 | TEVWLQWNWPDR | | | | Mascot |
| 1629.9421 | 1629.7004 | -0.2417 | -148 | 3489 | 3503 | TILKVELSQLSAAEK | | | | Mascot |
| 1682.7472 | 1682.7738 | 0.0266 | 16 | 6002 | 6016 | NEAGSESCSMDLRVK | | | Carbamidomethyl (C)[8] | Mascot |
| 1682.7472 | 1682.7738 | 0.0266 | 16 | 6002 | 6016 | NEAGSESCSMDLRVK | | | Carbamidomethyl (C)[8] | Mascot |
| 1698.7421 | 1698.765 | 0.0229 | 13 | 6002 | 6016 | NEAGSESCSMDLRVK | | | Carbamidomethyl (C)[8], Oxidation (M)[10] | Mascot |
| 1714.7965 | 1714.7611 | -0.0354 | -21 | 21943 | 21956 | TGCEYQFRIAAENR | | | Carbamidomethyl (C)[3] | Mascot |
| 1797.8774 | 1797.9053 | 0.0279 | 16 | 1083 | 1097 | SYRILEGMGVTFHCK | | | Carbamidomethyl (C)[14] | Mascot |
| 1797.9713 | 1797.9053 | -0.066 | -37 | 16896 | 16912 | ELKAMAGTHINIMAGIK | 1 | 0 | | Mascot |
| 1819.8684 | 1819.8815 | 0.0131 | 7 | 1661 | 1675 | LHYDGIHYLEITDCK | | | | Mascot |
| 1842.8293 | 1842.7349 | -0.0944 | -51 | 11826 | 11840 | FETEISEEDVHGHWK | | | | Mascot |
| 1909.9728 | 1909.9938 | 0.021 | 11 | 12198 | 12213 | LICEVSKPSAEVTWYK | | | Carbamidomethyl (C)[3] | Mascot |
| 1970.877 | 1970.8958 | 0.0188 | 10 | 16626 | 16641 | DLWMPVTESCTRTSCK | | | Carbamidomethyl (C)[10,15] | Mascot |
| 2099.0227 | 2098.9607 | -0.062 | -30 | 13143 | 13161 | WTFGENALTAGDRVSMVTK | | | Oxidation (M)[16] | Mascot |
| 2108.1069 | 2107.9185 | -0.1884 | -89 | 12946 | 12963 | DRTSVVWAPVMVTKPHER | | | | Mascot |
| 2124.1018 | 2123.9202 | -0.1816 | -85 | 12946 | 12963 | DRTSVVWAPVMVTKPHER | | | Oxidation (M)[11] | Mascot |
| 2140.989 | 2141.0518 | 0.0628 | 29 | 12057 | 12075 | LVIHDCTLDDSKTYTCDAK | | | | Mascot |
| 2166.0825 | 2165.895 | -0.1875 | -87 | 23685 | 23703 | GRPIPEINWTKDDGDLPDK | | | | Mascot |
| 2189.0066 | 2188.9788 | -0.0278 | -13 | 30534 | 30553 | CENIGGESDWSEISEPVIPK | | | | Mascot |
| 2190.0093 | 2189.9678 | -0.0415 | -19 | 1962 | 1979 | DTMVTFECETNEPFVKVK | | | Carbamidomethyl (C)[8], Oxidation (M)[3] | Mascot |
| 2190.0093 | 2189.9678 | -0.0415 | -19 | 1962 | 1979 | DTMVTFECETNEPFVKVK | | | Carbamidomethyl (C)[8], Oxidation (M)[3] | Mascot |
| 2194.0583 | 2194.0261 | -0.0322 | -15 | 23824 | 23843 | VMAENEFGIGLPVETEESVK | | | Oxidation (M)[2] | Mascot |
| 2211.021 | 2211.0452 | 0.0242 | 11 | 18006 | 18024 | MVWSTYSASLVTNYCNVTR | | | Oxidation (M)[1] | Mascot |
| 2215.1111 | 2215.0227 | -0.0884 | -40 | 31410 | 31428 | LNVAPHAVAEDTMRPMFKR | | | Oxidation (M)[13,16] | Mascot |
| 2221.0742 | 2221.0967 | 0.0225 | 10 | 8387 | 8403 | ELFLKQCMFFEVLMEEK | | | Carbamidomethyl (C)[7] | Mascot |
| 2225.0479 | 2225.0618 | 0.0139 | 6 | 18756 | 18774 | HPFDPPSEPLNCCVNKVSK | | | Carbamidomethyl (C)[12,13] | Mascot |
| 2229.1736 | 2229.0354 | -0.1382 | -62 | 18429 | 18447 | ITNLTPGIEYYFRVMAVNK | | | | Mascot |
| 2229.239 | 2229.0354 | -0.2036 | -91 | 588 | 606 | VKVLLDWPAQSPDIHIIER | | | | Mascot |
| 2232.9575 | 2233.0288 | 0.0713 | 32 | 1525 | 1542 | MINEFGYCSLDYEVAYSR | | | Carbamidomethyl (C)[8], Oxidation (M)[1] | Mascot |
| 2283.1284 | 2283.1169 | -0.0115 | -5 | 13772 | 13793 | VRAVNVAGESEPSEPSDPVLCK | | | | Mascot |
| 2284.1389 | 2284.1128 | -0.0261 | -11 | 21385 | 21405 | ACDTIFQPGPPGNPRVLDTTK | | | Carbamidomethyl (C)[2] | Mascot |
| 2306.0732 | 2306.0447 | -0.0285 | -12 | 17825 | 17842 | ETFFMVDKCVENHIYQFR | | | | Mascot |

| | | | | | | | | | | | |
|---|---|---|---|---|---|---|---|---|---|---|---|
| 2351.1006 | 2351.0234 | -0.0772 | -33 | 4822 | 4841 | LECQVDEDTGVSIIWMRDGK | | | Carbamidomethyl (C)[3] | | Mascot |
| 2357.0979 | 2357.124 | 0.0261 | 11 | 16046 | 16063 | ECQYIVQRLTEGHEYEFR | | | Carbamidomethyl (C)[2] | | Mascot |
| 2367.0955 | 2367.0222 | -0.0733 | -31 | 4822 | 4841 | LECQVDEDTGVSIIWMRDGK | | | Carbamidomethyl (C)[3], Oxidation (M)[16] | | Mascot |
| 2370.124 | 2370.1721 | 0.0481 | 20 | 13774 | 13795 | AVNVAGESEPSEPSDPVLCKER | | | Carbamidomethyl (C)[19] | | Mascot |
| 2410.0762 | 2410.2312 | 0.155 | 64 | 12242 | 12262 | MDDAGEYHCKLPTSQTTGNLR | | | Carbamidomethyl (C)[9], Oxidation (M)[1] | | Mascot |
| 2474.1028 | 2474.0852 | -0.0176 | -7 | 2045 | 2065 | NLESVEVPETYSGEFECELSR | | | Carbamidomethyl (C)[17] | | Mascot |
| 2683.2014 | 2683.2458 | 0.0444 | 17 | 14982 | 15005 | LMMLNWSDPDDDGGSDVTGFIIER | | | | | Mascot |
| 2731.22 | 2731.2441 | 0.0241 | 9 | 5004 | 5027 | GCTAHLECEMTGTAPFEITWFKDK | | | Oxidation (M)[10] | | Mascot |
| 2731.22 | 2731.2441 | 0.0241 | 9 | 5004 | 5027 | GCTAHLECEMTGTAPFEITWFKDK | | | Oxidation (M)[10] | | Mascot |
| 2747.397 | 2747.2437 | -0.1533 | -56 | 8392 | 8414 | QCMFFEVLMEEKKPKPAIQEPPK | | | | | Mascot |
| 2763.2195 | 2763.2476 | 0.0281 | 10 | 5801 | 5827 | LDSSDSGNYTCIAKNQAGSDQCSGTLK | | | | | Mascot |

| | | | | | | | |
|---|---|---|---|---|---|---|---|
| 36 | PREDICTED: p2Y purinoceptor 4-like [Danio rerio] | gi\|326669372 | 48084.4 | 9.33 | 9 | 31 | 0 |

Peptide Information

| Calc. Mass | Obsrv. Mass | ± da | ± ppm | Start Seq. | End Seq. | Sequence | Ion Score | C. I. % | Modification | Rank | Result Type |
|---|---|---|---|---|---|---|---|---|---|---|---|
| 891.4794 | 891.3801 | -0.0993 | -111 | 284 | 290 | SPYVNRR | | | | | Mascot |
| 1118.5486 | 1118.6045 | 0.0559 | 50 | 144 | 152 | YLGICHPMK | | | Carbamidomethyl (C)[5] | | Mascot |
| 1118.5486 | 1118.6045 | 0.0559 | 50 | 144 | 152 | YLGICHPMK | | | Carbamidomethyl (C)[5] | | Mascot |
| 1123.5895 | 1123.5106 | -0.0789 | -70 | 183 | 191 | FAQTQYLPR | | | | | Mascot |
| 1698.7947 | 1698.765 | -0.0297 | -17 | 327 | 340 | VQCHTMRMVSICYK | | | | | Mascot |
| 1714.7896 | 1714.7611 | -0.0285 | -17 | 327 | 340 | VQCHTMRMVSICYK | | | Oxidation (M)[6] | | Mascot |
| 2124.0754 | 2123.9202 | -0.1552 | -73 | 6 | 24 | SLRTMTTSWIETVTGVDAR | | | | | Mascot |
| 2166.1602 | 2165.895 | -0.2652 | -122 | 164 | 182 | GTCVLVWIAVFALTCPIFR | | | Carbamidomethyl (C)[3] | | Mascot |
| 2284.1226 | 2284.1128 | -0.0098 | -4 | 364 | 383 | NGPCCQKPEHAQHGLLWPLR | | | | | Mascot |
| 2410.3354 | 2410.2312 | -0.1042 | -43 | 296 | 315 | TIITITLLFALCFFPFHVTR | | | Carbamidomethyl (C)[12] | | Mascot |
| 2747.3513 | 2747.2437 | -0.1076 | -39 | 123 | 143 | FLFYFNLYCSIFFLTCISVHR | | | Carbamidomethyl (C)[9,16] | | Mascot |

| | | | | | | | |
|---|---|---|---|---|---|---|---|
| 37 | PREDICTED: NACHT, LRR and PYD domains-containing protein 12, partial [Danio rerio] | gi\|326666704 | 119811 | 6.35 | 13 | 31 | 0 |

Peptide Information

| Calc. Mass | Obsrv. Mass | ± da | ± ppm | Start Seq. | End Seq. | Sequence | Ion Score | C. I. % | Modification | Rank | Result Type |
|---|---|---|---|---|---|---|---|---|---|---|---|
| 891.4604 | 891.3801 | -0.0803 | -90 | 278 | 284 | EMNLKEK | | | | | Mascot |
| 907.4553 | 907.4323 | -0.023 | -25 | 278 | 284 | EMNLKEK | | | Oxidation (M)[2] | | Mascot |

| | | | | | | | | | | | |
|---|---|---|---|---|---|---|---|---|---|---|---|
| 1045.5272 | 1045.5388 | 0.0116 | 11 | 634 | 642 | GLLTQQDDR | | | | | Mascot |
| 1157.5367 | 1157.6738 | 0.1371 | 118 | 862 | 871 | LNLDHGGEMR | | | Oxidation (M)[9] | | Mascot |
| 1343.6082 | 1343.5637 | -0.0445 | -33 | 1 | 11 | FSCSMAEERVK | | | Carbamidomethyl (C)[3] | | Mascot |
| 1343.6082 | 1343.5637 | -0.0445 | -33 | 1 | 11 | FSCSMAEERVK | | | Carbamidomethyl (C)[3] | | Mascot |
| 1359.6031 | 1359.5563 | -0.0468 | -34 | 1 | 11 | FSCSMAEERVK | | | Carbamidomethyl (C)[3], Oxidation (M)[5] | | Mascot |
| 1391.6736 | 1391.7058 | 0.0322 | 23 | 325 | 336 | LPLNFAGNETCR | | | Carbamidomethyl (C)[11] | | Mascot |
| 1629.8231 | 1629.7004 | -0.1227 | -75 | 605 | 618 | ALESENGHLDLYLR | | | | | Mascot |
| 1629.8231 | 1629.7004 | -0.1227 | -75 | 605 | 618 | ALESENGHLDLYLR | | | | | Mascot |
| 2141.0947 | 2141.0518 | -0.0429 | -20 | 666 | 683 | SINLFYCLNELNDQTLLK | | | | | Mascot |
| 2190.1917 | 2189.9678 | -0.2239 | -102 | 69 | 87 | THKSFTGNLLLIFQNLESK | | | | | Mascot |
| 2190.1917 | 2189.9678 | -0.2239 | -102 | 69 | 87 | THKSFTGNLLLIFQNLESK | | | | | Mascot |
| 2215.124 | 2215.0227 | -0.1013 | -46 | 832 | 851 | ELDLSYNHPGDSGVKLLTEK | | | | | Mascot |
| 2220.9517 | 2221.0967 | 0.145 | 65 | 905 | 921 | CVWENQSYPDHPDRFDR | | | Carbamidomethyl (C)[1] | | Mascot |
| 2306.0466 | 2306.0447 | -0.0019 | -1 | 18 | 38 | HSERSGPCVSSSVSLNSDWSK | | | Carbamidomethyl (C)[8] | | Mascot |
| 2370.1265 | 2370.1721 | 0.0456 | 19 | 464 | 481 | CLTQMYTHFLRCQIQQSR | | | Carbamidomethyl (C)[1,12] | | Mascot |
| 2683.3284 | 2683.2458 | -0.0826 | -31 | 139 | 161 | QDQAAETLEDELFFINQLKCSLK | | | | | Mascot |

38 titin a [Danio rerio] gi|110005909 3646149.2 6.18 67 30 0

**Peptide Information**

| Calc. Mass | Obsrv. Mass | ± da | ± ppm | Start Seq. | End Seq. | Sequence | Ion Score | C. I. % | Modification | Rank | Result Type |
|---|---|---|---|---|---|---|---|---|---|---|---|
| 809.3675 | 809.3594 | -0.0081 | -10 | 32458 | 32464 | AEAFEDK | | | | | Mascot |
| 842.4981 | 842.4899 | -0.0082 | -10 | 8812 | 8818 | EDILPKK | | | | | Mascot |
| 882.4679 | 882.5447 | 0.0768 | 87 | 2876 | 2882 | YKVSTER | | | | | Mascot |
| 891.3624 | 891.3801 | 0.0177 | 20 | 4693 | 4701 | HGSDSCSAK | | | | | Mascot |
| 907.4301 | 907.4323 | 0.0022 | 2 | 1543 | 1550 | DSGVITCR | | | Carbamidomethyl (C)[7] | | Mascot |
| 908.389 | 908.4072 | 0.0182 | 20 | 1240 | 1247 | CSEGQTAR | | | Carbamidomethyl (C)[1] | | Mascot |
| 965.4509 | 965.4443 | -0.0066 | -7 | 19569 | 19576 | NSCTLGWK | | | Carbamidomethyl (C)[3] | | Mascot |
| 996.591 | 996.5723 | -0.0187 | -19 | 9064 | 9072 | MKAVPLPPK | | | Oxidation (M)[1] | | Mascot |
| 1042.5204 | 1042.557 | 0.0366 | 35 | 27162 | 27169 | ELSEVYFR | | | | | Mascot |
| 1045.5347 | 1045.5388 | 0.0041 | 4 | 7144 | 7153 | CQIGGTPEIK | | | | | Mascot |
| 1093.5524 | 1093.5359 | -0.0165 | -15 | 9592 | 9600 | EDGIEFQKK | | | | | Mascot |
| 1118.6205 | 1118.6045 | -0.016 | -14 | 24180 | 24188 | EKNSILWTK | | | | | Mascot |
| 1118.6238 | 1118.6045 | -0.0193 | -17 | 4857 | 4866 | IACLDIQKSK | 4 | 0 | | | Mascot |
| 1123.5266 | 1123.5106 | -0.016 | -14 | 9802 | 9810 | EIFTNQDEK | | | | | Mascot |

| | | | | | | | | | | |
|---|---|---|---|---|---|---|---|---|---|---|
| 1157.6385 | 1157.6738 | 0.0353 | 30 | 117 | 126 | QGSQVRLDVR | | | | Mascot |
| 1165.6211 | 1165.7151 | 0.094 | 81 | 28447 | 28457 | VSAENKFGVSK | | | | Mascot |
| 1183.5962 | 1183.5306 | -0.0656 | -55 | 6129 | 6137 | MVEFKCVVR | | | Carbamidomethyl (C)[6], Oxidation (M)[1] | Mascot |
| 1194.5923 | 1194.553 | -0.0393 | -33 | 10919 | 10928 | TLEEEMSVIK | | | Oxidation (M)[6] | Mascot |
| 1199.5613 | 1199.5233 | -0.038 | -32 | 1797 | 1806 | DESYEGMLKK | | | | Mascot |
| 1221.5304 | 1221.5453 | 0.0149 | 12 | 10260 | 10269 | DKDEDPEMVK | | | Oxidation (M)[8] | Mascot |
| 1221.6409 | 1221.5453 | -0.0956 | -78 | 4906 | 4915 | ILWKQGMSSR | | | Oxidation (M)[7] | Mascot |
| 1242.5419 | 1242.5757 | 0.0338 | 27 | 5340 | 5350 | YTCQVSNDAGK | | | Carbamidomethyl (C)[3] | Mascot |
| 1258.595 | 1258.5682 | -0.0268 | -21 | 2987 | 2998 | SDAGEYTFIAGK | | | | Mascot |
| 1325.6888 | 1325.6576 | -0.0312 | -24 | 30094 | 30103 | EILGYWIEFR | | | | Mascot |
| 1343.6698 | 1343.5637 | -0.1061 | -79 | 5309 | 5320 | EMLTGGTCFIKK | | | Oxidation (M)[2] | Mascot |
| 1343.6698 | 1343.5637 | -0.1061 | -79 | 5309 | 5320 | EMLTGGTCFIKK | | | Oxidation (M)[2] | Mascot |
| 1359.6328 | 1359.5563 | -0.0765 | -56 | 31229 | 31239 | NTEVTWYFGSR | | | | Mascot |
| 1367.6406 | 1367.5828 | -0.0578 | -42 | 6380 | 6392 | AGSCECSGTLRVK | | | Carbamidomethyl (C)[4] | Mascot |
| 1375.5769 | 1375.5514 | -0.0255 | -19 | 31219 | 31228 | FVCCIENYDR | | | Carbamidomethyl (C)[3,4] | Mascot |
| 1391.6914 | 1391.7058 | 0.0144 | 10 | 2956 | 2967 | NGVEIQFSVDQR | | | | Mascot |
| 1492.7061 | 1492.7264 | 0.0203 | 14 | 6635 | 6649 | ATNNVGSDLCQGSVK | | | | Mascot |
| 1492.7166 | 1492.7264 | 0.0098 | 7 | 28671 | 28683 | DEYTPPKAELDSK | | | | Mascot |
| 1626.7209 | 1626.7129 | -0.008 | -5 | 4784 | 4798 | ASNSVGTATCCTELR | | | Carbamidomethyl (C)[10,11] | Mascot |
| 1629.7809 | 1629.7004 | -0.0805 | -49 | 17093 | 17104 | TEVWLQWNWPDR | | | | Mascot |
| 1629.9421 | 1629.7004 | -0.2417 | -148 | 3489 | 3503 | TILKVELSQLSAAEK | | | | Mascot |
| 1682.7472 | 1682.7738 | 0.0266 | 16 | 6002 | 6016 | NEAGSESCSMDLRVK | | | Carbamidomethyl (C)[8] | Mascot |
| 1682.7472 | 1682.7738 | 0.0266 | 16 | 6002 | 6016 | NEAGSESCSMDLRVK | | | Carbamidomethyl (C)[8] | Mascot |
| 1698.7421 | 1698.765 | 0.0229 | 13 | 6002 | 6016 | NEAGSESCSMDLRVK | | | Carbamidomethyl (C)[8], Oxidation (M)[10] | Mascot |
| 1714.7965 | 1714.7611 | -0.0354 | -21 | 21943 | 21956 | TGCEYQFRIAAENR | | | Carbamidomethyl (C)[3] | Mascot |
| 1797.8774 | 1797.9053 | 0.0279 | 16 | 1083 | 1097 | SYRILEGMGVTFHCK | | | Carbamidomethyl (C)[14] | Mascot |
| 1797.9713 | 1797.9053 | -0.066 | -37 | 16896 | 16912 | ELKAMAGTHINIMAGIK | 1 | 0 | | Mascot |
| 1819.8684 | 1819.8815 | 0.0131 | 7 | 1661 | 1675 | LHYDGIHYLEITDCK | | | | Mascot |
| 1842.8293 | 1842.7349 | -0.0944 | -51 | 11826 | 11840 | FETEISEEDVHGHWK | | | | Mascot |
| 1909.9728 | 1909.9938 | 0.021 | 11 | 12198 | 12213 | LICEVSKPSAEVTWYK | | | Carbamidomethyl (C)[3] | Mascot |
| 1970.877 | 1970.8958 | 0.0188 | 10 | 16626 | 16641 | DLWMPVTESCTRTSCK | | | Carbamidomethyl (C)[10,15] | Mascot |
| 2099.0227 | 2098.9607 | -0.062 | -30 | 13143 | 13161 | WTFGENALTAGDRVSMVTK | | | Oxidation (M)[16] | Mascot |
| 2108.1069 | 2107.9185 | -0.1884 | -89 | 12946 | 12963 | DRTSVVWAPVMVTKPHER | | | | Mascot |
| 2124.1018 | 2123.9202 | -0.1816 | -85 | 12946 | 12963 | DRTSVVWAPVMVTKPHER | | | Oxidation (M)[11] | Mascot |
| 2140.989 | 2141.0518 | 0.0628 | 29 | 12057 | 12075 | LVIHDCTLDDSKTYTCDAK | | | | Mascot |
| 2166.0825 | 2165.895 | -0.1875 | -87 | 23685 | 23703 | GRPIPEINWTKDDGDLPD | | | | Mascot |

| | | | | | | | | | | | |
|---|---|---|---|---|---|---|---|---|---|---|---|
| | | | | | | K | | | | | |
| 2189.0066 | 2188.9788 | -0.0278 | -13 | 30534 | 30553 | CENIGGESDWSEISEPVIPK | | | | | Mascot |
| 2190.0093 | 2189.9678 | -0.0415 | -19 | 1962 | 1979 | DTMVTFECETNEPFVKVK | | | Carbamidomethyl (C)[8], Oxidation (M)[3] | | Mascot |
| 2190.0093 | 2189.9678 | -0.0415 | -19 | 1962 | 1979 | DTMVTFECETNEPFVKVK | | | Carbamidomethyl (C)[8], Oxidation (M)[3] | | Mascot |
| 2194.0583 | 2194.0261 | -0.0322 | -15 | 23824 | 23843 | VMAENEFGIGLPVETEESVK | | | Oxidation (M)[2] | | Mascot |
| 2211.021 | 2211.0452 | 0.0242 | 11 | 18006 | 18024 | MVWSTYSASLVTNYCNVTR | | | Oxidation (M)[1] | | Mascot |
| 2215.1111 | 2215.0227 | -0.0884 | -40 | 31410 | 31428 | LNVAPHAVAEDTMRPMFKR | | | Oxidation (M)[13,16] | | Mascot |
| 2221.1248 | 2221.0967 | -0.0281 | -13 | 29857 | 29876 | VIGRPDPPEGPLEFDDIQAR | | | | | Mascot |
| 2225.0479 | 2225.0618 | 0.0139 | 6 | 18756 | 18774 | HPFDPPSEPLNCCVNKVSK | | | Carbamidomethyl (C)[12,13] | | Mascot |
| 2229.1736 | 2229.0354 | -0.1382 | -62 | 18429 | 18447 | ITNLTPGIEYYFRVMAVNK | | | | | Mascot |
| 2229.1736 | 2229.0354 | -0.1382 | -62 | 18429 | 18447 | ITNLTPGIEYYFRVMAVNK | | | | | Mascot |
| 2232.9575 | 2233.0288 | 0.0713 | 32 | 1525 | 1542 | MINEFGYCSLDYEVAYSR | | | Carbamidomethyl (C)[8], Oxidation (M)[1] | | Mascot |
| 2283.1284 | 2283.1169 | -0.0115 | -5 | 13772 | 13793 | VRAVNVAGESEPSEPSDPVLCK | | | | | Mascot |
| 2284.1389 | 2284.1128 | -0.0261 | -11 | 21385 | 21405 | ACDTIFQPGPPGNPRVLDTTK | | | Carbamidomethyl (C)[2] | | Mascot |
| 2306.0732 | 2306.0447 | -0.0285 | -12 | 17825 | 17842 | ETFFMVDKCVENHIYQFR | | | | | Mascot |
| 2351.1006 | 2351.0234 | -0.0772 | -33 | 4822 | 4841 | LECQVDEDTGVSIIWMRDGK | | | Carbamidomethyl (C)[3] | | Mascot |
| 2357.0979 | 2357.124 | 0.0261 | 11 | 16046 | 16063 | ECQYIVQRLTEGHEYEFR | | | Carbamidomethyl (C)[2] | | Mascot |
| 2367.0955 | 2367.0222 | -0.0733 | -31 | 4822 | 4841 | LECQVDEDTGVSIIWMRDGK | | | Carbamidomethyl (C)[3], Oxidation (M)[16] | | Mascot |
| 2370.124 | 2370.1721 | 0.0481 | 20 | 13774 | 13795 | AVNVAGESEPSEPSDPVLCKER | | | Carbamidomethyl (C)[19] | | Mascot |
| 2410.0762 | 2410.2312 | 0.155 | 64 | 12242 | 12262 | MDDAGEYHCKLPTSQTTGNLR | | | Carbamidomethyl (C)[9], Oxidation (M)[1] | | Mascot |
| 2474.1028 | 2474.0852 | -0.0176 | -7 | 2045 | 2065 | NLESVEVPETYSGEFECELSR | | | Carbamidomethyl (C)[17] | | Mascot |
| 2683.2014 | 2683.2458 | 0.0444 | 17 | 14982 | 15005 | LMMLNWSDPDDDGGSDVTGFIIER | | | | | Mascot |
| 2731.22 | 2731.2441 | 0.0241 | 9 | 5004 | 5027 | GCTAHLECEMTGTAPFEITWFKDK | | | Oxidation (M)[10] | | Mascot |
| 2731.22 | 2731.2441 | 0.0241 | 9 | 5004 | 5027 | GCTAHLECEMTGTAPFEITWFKDK | | | Oxidation (M)[10] | | Mascot |
| 2747.4556 | 2747.2437 | -0.2119 | -77 | 32532 | 32556 | TSFSFVGQVFQAIYTKVTAFVAAHK | | | | | Mascot |
| 2763.2195 | 2763.2476 | 0.0281 | 10 | 5801 | 5827 | LDSSDSGNYTCIAKNQAGSDQCSGTLK | | | | | Mascot |

| | | | | | | | | |
|---|---|---|---|---|---|---|---|---|
| 39 | novel protein similar to human titin (TTN) [Danio rerio] | gi\|29561775 | 2113153 | 6.46 | 60 | 30 | 0 | |

**Peptide Information**

| Calc. Mass | Obsrv. Mass | ± da | ± ppm | Start Seq. | End Seq. | Sequence | Ion Score | C. I. % | Modification | Rank | Result Type |
|---|---|---|---|---|---|---|---|---|---|---|---|
| 809.3675 | 809.3594 | -0.0081 | -10 | 18767 | 18773 | AEAFEDK | | | | | Mascot |
| 842.5094 | 842.4899 | -0.0195 | -23 | 1522 | 1529 | LSIPGVTR | | | | | Mascot |

| | | | | | | | | |
|---|---|---|---|---|---|---|---|---|
| 882.4315 | 882.5447 | 0.1132 | 128 | 12144 | 12151 | DGSSFTLR | | Mascot |
| 891.4166 | 891.3801 | -0.0365 | -41 | 12482 | 12490 | ESVASDAGR | | Mascot |
| 907.4705 | 907.4323 | -0.0382 | -42 | 13743 | 13749 | NSLLWMK | Oxidation (M)[6] | Mascot |
| 908.4294 | 908.4072 | -0.0222 | -24 | 5843 | 5850 | NSCTLGWK | | Mascot |
| 965.4509 | 965.4443 | -0.0066 | -7 | 5843 | 5850 | NSCTLGWK | Carbamidomethyl (C)[3] | Mascot |
| 996.5373 | 996.5723 | 0.035 | 35 | 670 | 676 | QYQFRVR | | Mascot |
| 1042.5204 | 1042.557 | 0.0366 | 35 | 13471 | 13478 | ELSEVYFR | | Mascot |
| 1045.6404 | 1045.5388 | -0.1016 | -97 | 2435 | 2444 | GTKFVVPGLK | | Mascot |
| 1093.5095 | 1093.5359 | 0.0264 | 24 | 2124 | 2131 | ETWKQCNK | Carbamidomethyl (C)[6] | Mascot |
| 1118.5477 | 1118.6045 | 0.0568 | 51 | 2114 | 2123 | LSWDTSGPQK | | Mascot |
| 1118.6205 | 1118.6045 | -0.016 | -14 | 10489 | 10497 | EKNSILWTK | | Mascot |
| 1123.4871 | 1123.5106 | 0.0235 | 21 | 6467 | 6475 | LAENVMDCR | Carbamidomethyl (C)[8], Oxidation (M)[6] | Mascot |
| 1157.7252 | 1157.6738 | -0.0514 | -44 | 8636 | 8645 | VQTLNLKITK | | Mascot |
| 1165.6211 | 1165.7151 | 0.094 | 81 | 14756 | 14766 | VSAENKFGVSK | | Mascot |
| 1194.6035 | 1194.553 | -0.0505 | -42 | 4241 | 4251 | DMKISGISTDK | | Mascot |
| 1199.6531 | 1199.5233 | -0.1298 | -108 | 1118 | 1127 | TRTFTVYGVR | | Mascot |
| 1221.6626 | 1221.5453 | -0.1173 | -96 | 10810 | 10819 | LLPGNEYIFR | | Mascot |
| 1221.6626 | 1221.5453 | -0.1173 | -96 | 8646 | 8655 | LLPGNEYIFR | | Mascot |
| 1242.6113 | 1242.5757 | -0.0356 | -29 | 11495 | 11504 | LTQGTEYQFR | | Mascot |
| 1258.6976 | 1258.5682 | -0.1294 | -103 | 16104 | 16114 | GKPLPTCKWTK | | Mascot |
| 1325.6888 | 1325.6576 | -0.0312 | -24 | 16403 | 16412 | EILGYWIEFR | | Mascot |
| 1343.6776 | 1343.5637 | -0.1139 | -85 | 1335 | 1347 | YGCGPPVDLGPIR | | Mascot |
| 1343.6776 | 1343.5637 | -0.1139 | -85 | 1335 | 1347 | YGCGPPVDLGPIR | | Mascot |
| 1359.6328 | 1359.5563 | -0.0765 | -56 | 17538 | 17548 | NTEVTWYFGSR | | Mascot |
| 1367.6412 | 1367.5828 | -0.0584 | -43 | 4006 | 4015 | LMENHEYLFR | Oxidation (M)[2] | Mascot |
| 1375.5769 | 1375.5514 | -0.0255 | -19 | 17528 | 17537 | FVCCIENYDR | Carbamidomethyl (C)[3,4] | Mascot |
| 1391.6624 | 1391.7058 | 0.0434 | 31 | 8975 | 8986 | VLDTPDMPANFR | Oxidation (M)[7] | Mascot |
| 1492.7166 | 1492.7264 | 0.0098 | 7 | 14980 | 14992 | DEYTPPKAELDSK | | Mascot |
| 1492.827 | 1492.7264 | -0.1006 | -67 | 2373 | 2386 | ARNLFTPPGPPTPK | | Mascot |
| 1626.7693 | 1626.7129 | -0.0564 | -35 | 7694 | 7708 | ACDTIFQPGPPGNPR | Carbamidomethyl (C)[2] | Mascot |
| 1629.7809 | 1629.7004 | -0.0805 | -49 | 3367 | 3378 | TEVWLQWNWPDR | | Mascot |
| 1629.9104 | 1629.7004 | -0.21 | -129 | 2787 | 2800 | LVVRVGETCIIQSR | Carbamidomethyl (C)[9] | Mascot |
| 1682.8265 | 1682.7738 | -0.0527 | -31 | 3222 | 3236 | AEIETSGTTTKLEMR | Oxidation (M)[14] | Mascot |
| 1682.8265 | 1682.7738 | -0.0527 | -31 | 3222 | 3236 | AEIETSGTTTKLEMR | Oxidation (M)[14] | Mascot |
| 1698.8367 | 1698.765 | -0.0717 | -42 | 8025 | 8039 | ITGYIVEMQGKNSDK | Oxidation (M)[8] | Mascot |
| 1714.7965 | 1714.7611 | -0.0354 | -21 | 8252 | 8265 | TGCEYQFRIAAENR | Carbamidomethyl (C)[3] | Mascot |
| 1797.8734 | 1797.9053 | 0.0319 | 18 | 11627 | 11642 | DPCAPPSQPRVMNITR | Oxidation (M)[12] | Mascot |

| | | | | | | | | | | | |
|---|---|---|---|---|---|---|---|---|---|---|---|
| 1797.9713 | 1797.9053 | -0.066 | -37 | 3186 | 3202 | ELKAMAGTHINIMAGIK | 1 | 0 | | | Mascot |
| 1820.1255 | 1819.8815 | -0.244 | -134 | 12440 | 12456 | IEIPLTGKPLPKVSLSK | | | | | Mascot |
| 1842.9232 | 1842.7349 | -0.1883 | -102 | 2216 | 2231 | APQDKPDDKVPVQYDK | | | | | Mascot |
| 1970.877 | 1970.8958 | 0.0188 | 10 | 2916 | 2931 | DLWMPVTESCTRTSCK | | | Carbamidomethyl (C)[10,15] | | Mascot |
| 2166.0825 | 2165.895 | -0.1875 | -87 | 9994 | 10012 | GRPIPEINWTKDDGDLPDK | | | | | Mascot |
| 2189.0066 | 2188.9788 | -0.0278 | -13 | 16843 | 16862 | CENIGGESDWSEISEPVIPK | | | | | Mascot |
| 2190.0205 | 2189.9678 | -0.0527 | -24 | 13462 | 13478 | CQCEVTELKELSEVYFR | | | Carbamidomethyl (C)[1,3] | | Mascot |
| 2190.2029 | 2189.9678 | -0.2351 | -107 | 7426 | 7445 | LDAHVTGRPIPSIVWTKDGK | | | | | Mascot |
| 2194.0583 | 2194.0261 | -0.0322 | -15 | 10133 | 10152 | VMAENEFGIGLPVETEESVK | | | Oxidation (M)[2] | | Mascot |
| 2211.021 | 2211.0452 | 0.0242 | 11 | 4280 | 4298 | MVWSTYSASLVTNYCNVTR | | | Oxidation (M)[1] | | Mascot |
| 2215.1111 | 2215.0227 | -0.0884 | -40 | 17719 | 17737 | LNVAPHAVAEDTMRPMFKR | | | Oxidation (M)[13,16] | | Mascot |
| 2221.1248 | 2221.0967 | -0.0281 | -13 | 16166 | 16185 | VIGRPDPPEGPLEFDDIQAR | | | | | Mascot |
| 2225.0479 | 2225.0618 | 0.0139 | 6 | 5030 | 5048 | HPFDPPSEPLNCCVNKVSK | | | Carbamidomethyl (C)[12,13] | | Mascot |
| 2229.1736 | 2229.0354 | -0.1382 | -62 | 4703 | 4721 | ITNLTPGIEYYFRVMAVNK | | | | | Mascot |
| 2229.1736 | 2229.0354 | -0.1382 | -62 | 4703 | 4721 | ITNLTPGIEYYFRVMAVNK | | | | | Mascot |
| 2283.1284 | 2283.1169 | -0.0115 | -5 | 76 | 97 | VRAVNVAGESEPSEPSDPVLCK | | | | | Mascot |
| 2284.1389 | 2284.1128 | -0.0261 | -11 | 7694 | 7714 | ACDTIFQPGPPGNPRVLDTTK | | | Carbamidomethyl (C)[2] | | Mascot |
| 2306.0732 | 2306.0447 | -0.0285 | -12 | 4099 | 4116 | ETFFMVDKCVENHIYQFR | | | | | Mascot |
| 2351.1272 | 2351.0234 | -0.1038 | -44 | 4279 | 4298 | RMVWSTYSASLVTNYCNVTR | | | | | Mascot |
| 2357.0979 | 2357.124 | 0.0261 | 11 | 2336 | 2353 | ECQYIVQRLTEGHEYEFR | | | Carbamidomethyl (C)[2] | | Mascot |
| 2367.1221 | 2367.0222 | -0.0999 | -42 | 4279 | 4298 | RMVWSTYSASLVTNYCNVTR | | | Oxidation (M)[2] | | Mascot |
| 2370.124 | 2370.1721 | 0.0481 | 20 | 78 | 99 | AVNVAGESEPSEPSDPVLCKER | | | Carbamidomethyl (C)[19] | | Mascot |
| 2474.1868 | 2474.0852 | -0.1016 | -41 | 5851 | 5873 | KPISDGGSHVVAYALEICEGEDK | | | Carbamidomethyl (C)[18] | | Mascot |
| 2683.2014 | 2683.2458 | 0.0444 | 17 | 1272 | 1295 | LMMLNWSDPDDDGGSDVTGFIIER | | | | | Mascot |
| 2731.3396 | 2731.2441 | -0.0955 | -35 | 5851 | 5875 | KPISDGGSHVVAYALEICEGEDKWK | | | | | Mascot |
| 2731.3396 | 2731.2441 | -0.0955 | -35 | 5851 | 5875 | KPISDGGSHVVAYALEICEGEDKWK | | | | | Mascot |
| 2747.4556 | 2747.2437 | -0.2119 | -77 | 18841 | 18865 | TSFSFVGQVFQAIYTKVTAFVAAHK | | | | | Mascot |
| 2763.4238 | 2763.2476 | -0.1762 | -64 | 13324 | 13348 | GKLTLSWEPPYIDGGSPITNYVVEK | | | | | Mascot |

| | | | | | | | |
|---|---|---|---|---|---|---|---|
| 40 | Zgc:162551 [Danio rerio] | gi\|134024880 | 35020.5 | 8.35 | 9 | 30 | 0 |

**Peptide Information**

| Calc. Mass | Obsrv. Mass | ± da | ± ppm | Start Seq. | End Seq. | Sequence | Ion Score | C. I. % | Modification | Rank | Result Type |
|---|---|---|---|---|---|---|---|---|---|---|---|

| | | | | | | | | | | | | |
|---|---|---|---|---|---|---|---|---|---|---|---|---|
| 891.3447 | 891.3801 | 0.0354 | 40 | 162 | 169 | HTCNECGK | | | | | | Mascot |
| 907.4666 | 907.4323 | -0.0343 | -38 | 186 | 193 | SLDSKMAR | | | | | | Mascot |
| 908.3964 | 908.4072 | 0.0108 | 12 | 194 | 200 | KCPTCDK | | | | Carbamidomethyl (C)[2,5] | | Mascot |
| 996.4818 | 996.5723 | 0.0905 | 91 | 48 | 55 | LCENGYIK | | | | Carbamidomethyl (C)[2] | | Mascot |
| 1157.5521 | 1157.6738 | 0.1217 | 105 | 264 | 273 | AHMQTHSAFK | | | | | | Mascot |
| 1629.7261 | 1629.7004 | -0.0257 | -16 | 240 | 254 | SHTGEKPFACAHCGK | | | | Carbamidomethyl (C)[10] | | Mascot |
| 1629.7261 | 1629.7004 | -0.0257 | -16 | 240 | 254 | SHTGEKPFACAHCGK | | | | Carbamidomethyl (C)[10] | | Mascot |
| 2189.9968 | 2189.9678 | -0.029 | -13 | 240 | 259 | SHTGEKPFACAHCGKAF ADR | | | | Carbamidomethyl (C)[10] | | Mascot |
| 2189.9968 | 2189.9678 | -0.029 | -13 | 240 | 259 | SHTGEKPFACAHCGKAF ADR | | | | Carbamidomethyl (C)[10] | | Mascot |
| 2232.8369 | 2233.0288 | 0.1919 | 86 | 293 | 312 | HYESACFRGSGDEDESG SEN | | | | Carbamidomethyl (C)[6] | | Mascot |
| 2370.1528 | 2370.1721 | 0.0193 | 8 | 221 | 239 | CHVCSKAFSRPWLLQGH MR | | | | Carbamidomethyl (C)[1,4] | | Mascot |

| | | | | | | | | |
|---|---|---|---|---|---|---|---|---|
| 41 | mediator of RNA polymerase II transcription subunit 22 [Danio rerio] | gi\|50344944 | 16398.5 | 5.15 | 5 | 30 | 0 | |

Peptide Information

| Calc. Mass | Obsrv. Mass | ± da | ± ppm | Start Seq. | End Seq. | Sequence | Ion Score | C. I. % | Modification | Rank | Result Type |
|---|---|---|---|---|---|---|---|---|---|---|---|
| 965.4244 | 965.4443 | 0.0199 | 21 | 107 | 114 | TLQEECDK | | | | | Mascot |
| 1093.5194 | 1093.5359 | 0.0165 | 15 | 107 | 115 | TLQEECDKK | | | | | Mascot |
| 1359.7413 | 1359.5563 | -0.185 | -136 | 65 | 77 | AANIVRAGESLMK | | | | | Mascot |
| 1375.7362 | 1375.5514 | -0.1848 | -134 | 65 | 77 | AANIVRAGESLMK | | | Oxidation (M)[12] | | Mascot |
| 1698.7653 | 1698.765 | -0.0003 | 0 | 51 | 64 | ATQAEQDHFEMHVR | | | | | Mascot |
| 1714.7603 | 1714.7611 | 0.0008 | 0 | 51 | 64 | ATQAEQDHFEMHVR | | | Oxidation (M)[11] | | Mascot |
| 2731.5029 | 2731.2441 | -0.2588 | -95 | 78 | 101 | LVSDLKQFLILNDFPSVN EAISLR | | | | | Mascot |
| 2731.5029 | 2731.2441 | -0.2588 | -95 | 78 | 101 | LVSDLKQFLILNDFPSVN EAISLR | | | | | Mascot |

| | | | | | | | | |
|---|---|---|---|---|---|---|---|---|
| 42 | transitional endoplasmic reticulum ATPase-like [Danio rerio] | gi\|90093334 | 89437.9 | 5.44 | 11 | 30 | 0 | |

Peptide Information

| Calc. Mass | Obsrv. Mass | ± da | ± ppm | Start Seq. | End Seq. | Sequence | Ion Score | C. I. % | Modification | Rank | Result Type |
|---|---|---|---|---|---|---|---|---|---|---|---|
| 1183.6293 | 1183.5306 | -0.0987 | -83 | 505 | 514 | FLKFGMTPSR | | | | | Mascot |
| 1194.5975 | 1194.553 | -0.0445 | -37 | 515 | 526 | GVLFYGPPGCGK | | | | | Mascot |
| 1199.6241 | 1199.5233 | -0.1008 | -84 | 505 | 514 | FLKFGMTPSR | | | Oxidation (M)[6] | | Mascot |
| 1258.5883 | 1258.5682 | -0.0201 | -16 | 587 | 601 | ARGGGAGDAGGAADR | | | | | Mascot |
| 1492.7828 | 1492.7264 | -0.0564 | -38 | 98 | 111 | LGDVISIHPCPDVK | | | | | Mascot |

| Calc. Mass | Obsrv. Mass | ± da | ± ppm | Start Seq. | End Seq. | Sequence | Ion Score | C. I. % | Modification | Rank | Result Type |
|---|---|---|---|---|---|---|---|---|---|---|---|
| 1492.7828 | 1492.7264 | -0.0564 | -38 | 98 | 111 | LGDVISIHPCPDVK | | | | | Mascot |
| 1629.8054 | 1629.7004 | -0.105 | -64 | 756 | 768 | KYEMFAQTLQQSR | | | | | Mascot |
| 1629.8054 | 1629.7004 | -0.105 | -64 | 756 | 768 | KYEMFAQTLQQSR | | | | | Mascot |
| 1714.9884 | 1714.7611 | -0.2273 | -133 | 326 | 340 | IVSQLLTLMDGLKQR | | | | | Mascot |
| 1909.8484 | 1909.9938 | 0.1454 | 76 | 720 | 735 | ETAMDDDYDPVPEIRK | | | Oxidation (M)[4] | | Mascot |
| 1970.9615 | 1970.8958 | -0.0657 | -33 | 508 | 526 | FGMTPSRGVLFYGPPGCGK | | | | | Mascot |
| 2098.9895 | 2098.9607 | -0.0288 | -14 | 680 | 698 | ITEGFSGADLTEICQRACK | | | Carbamidomethyl (C)[14] | | Mascot |
| 2108.0845 | 2107.9185 | -0.166 | -79 | 570 | 588 | QAAPCILFFDELDSIAKAR | | | | | Mascot |
| 2190.0957 | 2189.9678 | -0.1279 | -58 | 28 | 47 | LIVDEATNEDNSIVCLSQVK | | | | | Mascot |
| 2190.0957 | 2189.9678 | -0.1279 | -58 | 28 | 47 | LIVDEATNEDNSIVCLSQVK | | | | | Mascot |

| | | | | | | | | |
|---|---|---|---|---|---|---|---|---|
| 43 | tafazzin [Danio rerio] | gi\|49227272 | 30524.4 | 8.94 | 7 | 30 | 0 | |

Peptide Information

| Calc. Mass | Obsrv. Mass | ± da | ± ppm | Start Seq. | End Seq. | Sequence | Ion Score | C. I. % | Modification | Rank | Result Type |
|---|---|---|---|---|---|---|---|---|---|---|---|
| 996.4818 | 996.5723 | 0.0905 | 91 | 131 | 138 | GMDFLLER | | | Oxidation (M)[2] | | Mascot |
| 1343.6445 | 1343.5637 | -0.0808 | -60 | 153 | 163 | VNMSGEFMRIK | | | Oxidation (M)[3,8] | | Mascot |
| 1343.6445 | 1343.5637 | -0.0808 | -60 | 153 | 163 | VNMSGEFMRIK | | | Oxidation (M)[3,8] | | Mascot |
| 1367.6412 | 1367.5828 | -0.0584 | -43 | 95 | 106 | WTPAASDICFTR | | | | | Mascot |
| 1626.8097 | 1626.7129 | -0.0968 | -60 | 2 | 14 | PLEVTWPFPQCPR | | | Carbamidomethyl (C)[11] | | Mascot |
| 1714.8646 | 1714.7611 | -0.1035 | -60 | 235 | 248 | TVTDYIQDEFRSLK | | | | | Mascot |
| 2229.1096 | 2229.0354 | -0.0742 | -33 | 1 | 18 | MPLEVTWPFPQCPRLGWR | | | Oxidation (M)[1] | | Mascot |
| 2229.1096 | 2229.0354 | -0.0742 | -33 | 1 | 18 | MPLEVTWPFPQCPRLGWR | | | Oxidation (M)[1] | | Mascot |
| 2747.3181 | 2747.2437 | -0.0744 | -27 | 139 | 161 | LNQGEWIHIFPEGRVNMSGEFMR | | | | | Mascot |
| 2763.313 | 2763.2476 | -0.0654 | -24 | 139 | 161 | LNQGEWIHIFPEGRVNMSGEFMR | | | Oxidation (M)[17] | | Mascot |

| | | | | | | | | | |
|---|---|---|---|---|---|---|---|---|---|
| 44 | transferrin-a [Danio rerio] | gi\|220672954 | 73234.7 | 6.61 | 7 | 30 | 0 | 18 | 0 |

Peptide Information

| Calc. Mass | Obsrv. Mass | ± da | ± ppm | Start Seq. | End Seq. | Sequence | Ion Score | C. I. % | Modification | Rank | Result Type |
|---|---|---|---|---|---|---|---|---|---|---|---|
| 1221.5793 | 1221.5453 | -0.034 | -28 | 246 | 255 | TCNFAREPAR | | | Carbamidomethyl (C)[2] | | Mascot |
| 1221.5793 | 1221.5453 | -0.034 | -28 | 246 | 255 | TCNFAREPAR | 18 | 0 | Carbamidomethyl (C)[2] | | Mascot |
| 1359.6586 | 1359.5563 | -0.1023 | -75 | 442 | 454 | SCHTGLGRSAGWK | | | | | Mascot |
| 1492.8005 | 1492.7264 | -0.0741 | -50 | 651 | 663 | TYKTSQNVPDLVK | | | | | Mascot |
| 1492.8005 | 1492.7264 | -0.0741 | -50 | 651 | 663 | TYKTSQNVPDLVK | | | | | Mascot |

| Calc. Mass | Obsrv. Mass | ± da | ± ppm | Start Seq. | End Seq. | Sequence | Ion Score | C. I. % | Modification | Rank | Result Type |
|---|---|---|---|---|---|---|---|---|---|---|---|
| 1629.736 | 1629.7004 | -0.0356 | -22 | 227 | 239 | QNYELLCMDGSRK | | | Carbamidomethyl (C)[7], Oxidation (M)[8] | | Mascot |
| 1629.736 | 1629.7004 | -0.0356 | -22 | 227 | 239 | QNYELLCMDGSRK | | | Carbamidomethyl (C)[7], Oxidation (M)[8] | | Mascot |
| 2141.0002 | 2141.0518 | 0.0516 | 24 | 654 | 673 | TSQNVPDLVKACTFGNCISS | | | Carbamidomethyl (C)[12] | | Mascot |
| 2357.1223 | 2357.124 | 0.0017 | 1 | 326 | 348 | DGNPSAPTSQTKLAMCTIGHAEK | | | | | Mascot |
| 2370.084 | 2370.1721 | 0.0881 | 37 | 551 | 570 | DLKSEDFELICPNTPDTTMK | | | Carbamidomethyl (C)[11], Oxidation (M)[19] | | Mascot |

| | | | | | | | | | | |
|---|---|---|---|---|---|---|---|---|---|---|
| 45 | TPA_exp: transferrin [Danio rerio] | gi\|47264590 | 73483.9 | 6.81 | 7 | 29 | 0 | 18 | 0 |

**Peptide Information**

| Calc. Mass | Obsrv. Mass | ± da | ± ppm | Start Seq. | End Seq. | Sequence | Ion Score | C. I. % | Modification | Rank | Result Type |
|---|---|---|---|---|---|---|---|---|---|---|---|
| 1221.5793 | 1221.5453 | -0.034 | -28 | 246 | 255 | TCNFAREPAR | | | Carbamidomethyl (C)[2] | | Mascot |
| 1221.5793 | 1221.5453 | -0.034 | -28 | 246 | 255 | TCNFAREPAR | 18 | 0 | Carbamidomethyl (C)[2] | | Mascot |
| 1359.6586 | 1359.5563 | -0.1023 | -75 | 442 | 454 | SCHTGLGRSAGWK | | | | | Mascot |
| 1492.8005 | 1492.7264 | -0.0741 | -50 | 653 | 665 | TYKTSQNVPDLVK | | | | | Mascot |
| 1492.8005 | 1492.7264 | -0.0741 | -50 | 653 | 665 | TYKTSQNVPDLVK | | | | | Mascot |
| 1629.736 | 1629.7004 | -0.0356 | -22 | 227 | 239 | QNYELLCMDGSRK | | | Carbamidomethyl (C)[7], Oxidation (M)[8] | | Mascot |
| 1629.736 | 1629.7004 | -0.0356 | -22 | 227 | 239 | QNYELLCMDGSRK | | | Carbamidomethyl (C)[7], Oxidation (M)[8] | | Mascot |
| 2141.0002 | 2141.0518 | 0.0516 | 24 | 656 | 675 | TSQNVPDLVKACTFGNCISS | | | Carbamidomethyl (C)[12] | | Mascot |
| 2357.1223 | 2357.124 | 0.0017 | 1 | 326 | 348 | DGNPSAPTSQTKLAMCTIGHAEK | | | | | Mascot |
| 2370.084 | 2370.1721 | 0.0881 | 37 | 553 | 572 | DLKSEDFELICPNTPDTTMK | | | Carbamidomethyl (C)[11], Oxidation (M)[19] | | Mascot |

| | | | | | | | | | | |
|---|---|---|---|---|---|---|---|---|---|---|
| 46 | serotransferrin [Danio rerio] | gi\|62632717 | 56808.3 | 5.58 | 6 | 29 | 0 | 18 | 0 |

**Peptide Information**

| Calc. Mass | Obsrv. Mass | ± da | ± ppm | Start Seq. | End Seq. | Sequence | Ion Score | C. I. % | Modification | Rank | Result Type |
|---|---|---|---|---|---|---|---|---|---|---|---|
| 1221.5793 | 1221.5453 | -0.034 | -28 | 93 | 102 | TCNFAREPAR | | | Carbamidomethyl (C)[2] | | Mascot |
| 1221.5793 | 1221.5453 | -0.034 | -28 | 93 | 102 | TCNFAREPAR | 18 | 0 | Carbamidomethyl (C)[2] | | Mascot |
| 1359.6586 | 1359.5563 | -0.1023 | -75 | 289 | 301 | SCHTGLGRSAGWK | | | | | Mascot |
| 1492.8005 | 1492.7264 | -0.0741 | -50 | 498 | 510 | TYKTSQNVPDLVK | | | | | Mascot |
| 1492.8005 | 1492.7264 | -0.0741 | -50 | 498 | 510 | TYKTSQNVPDLVK | | | | | Mascot |
| 1629.736 | 1629.7004 | -0.0356 | -22 | 74 | 86 | QNYELLCMDGSRK | | | Carbamidomethyl (C)[7], Oxidation (M)[8] | | Mascot |
| 1629.736 | 1629.7004 | -0.0356 | -22 | 74 | 86 | QNYELLCMDGSRK | | | Carbamidomethyl (C)[7], Oxidation (M)[8] | | Mascot |
| 2357.1223 | 2357.124 | 0.0017 | 1 | 173 | 195 | DGNPSAPTSQTKLAMCTIGHAEK | | | | | Mascot |
| 2370.084 | 2370.1721 | 0.0881 | 37 | 398 | 417 | DLKSEDFELICPNTPDTTMK | | | Carbamidomethyl (C)[11], Oxidation (M)[19] | | Mascot |

| | | | | | | | | | | |
|---|---|---|---|---|---|---|---|---|---|---|
| 47 | DCN1-like protein 3 [Danio rerio] | gi\|156739269 | 33276.1 | 4.93 | 5 | 29 | 0 | 13 | 0 |

Peptide Information

| Calc. Mass | Obsrv. Mass | ± da | ± ppm | Start Seq. | End Seq. | Sequence | Ion Score | C. I. % | Modification | Rank | Result Type |
|---|---|---|---|---|---|---|---|---|---|---|---|
| 1118.6051 | 1118.6045 | -0.0006 | -1 | 43 | 53 | SSAEVIINGTK | | | | | Mascot |
| 1118.6051 | 1118.6045 | -0.0006 | -1 | 43 | 53 | SSAEVIINGTK | 13 | 0 | | | Mascot |
| 2166.0317 | 2165.895 | -0.1367 | -63 | 144 | 163 | EFVDGCKAIQADSIPGICSR | | | Carbamidomethyl (C)[6] | | Mascot |
| 2367.1875 | 2367.0222 | -0.1653 | -70 | 112 | 131 | FCNDLCVDPAEFKVLVLAWK | | | Carbamidomethyl (C)[2] | | Mascot |
| 2410.3857 | 2410.2312 | -0.1545 | -64 | 201 | 221 | SIAIALWRLVFTLDTPPVLER | | | | | Mascot |
| 2474.0671 | 2474.0852 | 0.0181 | 7 | 92 | 111 | MFLCYKDEHEDSILEEGMER | | | | | Mascot |

48 chloride intracellular channel protein 4 [Danio rerio] gi|41393129 28352.6 5.71 6 29 0

Peptide Information

| Calc. Mass | Obsrv. Mass | ± da | ± ppm | Start Seq. | End Seq. | Sequence | Ion Score | C. I. % | Modification | Rank | Result Type |
|---|---|---|---|---|---|---|---|---|---|---|---|
| 1042.5891 | 1042.557 | -0.0321 | -31 | 2 | 11 | SLSVPQNGLK | | | | | Mascot |
| 1221.6361 | 1221.5453 | -0.0908 | -74 | 238 | 248 | EIEIAYADVAK | | | | | Mascot |
| 1221.6361 | 1221.5453 | -0.0908 | -74 | 238 | 248 | EIEIAYADVAK | | | | | Mascot |
| 1343.6549 | 1343.5637 | -0.0912 | -68 | 130 | 141 | NSKPDANEALER | | | | | Mascot |
| 1343.6549 | 1343.5637 | -0.0912 | -68 | 130 | 141 | NSKPDANEALER | | | | | Mascot |
| 1359.7155 | 1359.5563 | -0.1592 | -117 | 12 | 23 | GDNEPVIELFVK | | | | | Mascot |
| 2211.0903 | 2211.0452 | -0.0451 | -20 | 110 | 129 | HPESNTAGMDIFAKFSAFIK | | | | | Mascot |
| 2747.4626 | 2747.2437 | -0.2189 | -80 | 60 | 84 | RKPADLQNLAPGTHPPFITFNGEVK | | | | | Mascot |

49 novel protein similar to vertebrate SLC3A1 solute carrier family 3 (cystine, dibasic and neutral am gi|220679491 74156.2 5.06 8 29 0

Peptide Information

| Calc. Mass | Obsrv. Mass | ± da | ± ppm | Start Seq. | End Seq. | Sequence | Ion Score | C. I. % | Modification | Rank | Result Type |
|---|---|---|---|---|---|---|---|---|---|---|---|
| 1093.6404 | 1093.5359 | -0.1045 | -96 | 549 | 558 | GFLVVLNFGK | | | | | Mascot |
| 1118.5072 | 1118.6045 | 0.0973 | 87 | 86 | 96 | DSNADGVGDLR | | | | | Mascot |
| 1118.5072 | 1118.6045 | 0.0973 | 87 | 86 | 96 | DSNADGVGDLR | 8 | 0 | | | Mascot |
| 1123.5677 | 1123.5106 | -0.0571 | -51 | 267 | 275 | HMLEATHLR | | | Oxidation (M)[2] | | Mascot |
| 1194.5935 | 1194.553 | -0.0405 | -34 | 256 | 266 | GVDGFRMDAVK | | | | | Mascot |
| 1367.6412 | 1367.5828 | -0.0584 | -43 | 343 | 353 | TMRYYGTNYAK | | | | | Mascot |
| 2225.2151 | 2225.0618 | -0.1533 | -69 | 238 | 255 | NPLVLQEMTDIIHFWLKK | | | | | Mascot |
| 2357.113 | 2357.124 | 0.011 | 5 | 220 | 237 | QQCYFHQFLKEQPDLNY | | | | | Mascot |

| | | | | | | | | | | | |
|---|---|---|---|---|---|---|---|---|---|---|---|
| 2370.1533 | 2370.1721 | 0.0188 | 8 | 3 | 23 | R VSSLPEESEYTQIKPYAG MPK | | | Oxidation (M)[19] | | Mascot |

| Rank | Protein Name | Accession No. | Protein MW | Protein PI | Pep. Count | Protein Score | Protein Score C. I. % | Total Ion Score | Total Ion C. I. % |
|---|---|---|---|---|---|---|---|---|---|
| 50 | PREDICTED: hypothetical protein LOC100535483 [Danio rerio] | gi\|326664199 | 24331.5 | 8.85 | 1 | | | 18 | 0 |

Peptide Information

| Calc. Mass | Obsrv. Mass | ± da | ± ppm | Start Seq. | End Seq. | Sequence | Ion Score | C. I. % | Modification | Rank | Result Type |
|---|---|---|---|---|---|---|---|---|---|---|---|
| 1118.635 | 1118.6045 | -0.0305 | -27 | 134 | 143 | GLKMSNVTLR | 18 | 0 | | 3 | Mascot |

| Gel Idx/Pos | 184/H11 | Instr./Gel Origin | BA2060/110707A | Process Status | Analysis Succeeded |
|---|---|---|---|---|---|
| Plate [#] Name | [1] 31496 | Instrument Sample Name | | Spectra | 11 |

| Rank | Protein Name | Accession No. | Protein MW | Protein PI | Pep. Count | Protein Score | Protein Score C. I. % | Total Ion Score | Total Ion C. I. % |
|---|---|---|---|---|---|---|---|---|---|
| 1 | succinate dehydrogenase [ubiquinone] flavoprotein subunit, mitochondrial precursor [Danio rerio] | gi\|41053873 | 72014.6 | 6.2 | 20 | 1,190 | 100 | 1070 | 100 |

Peptide Information

| Calc. Mass | Obsrv. Mass | ± da | ± ppm | Start Seq. | End Seq. | Sequence | Ion Score | C. I. % | Modification | Rank | Result Type |
|---|---|---|---|---|---|---|---|---|---|---|---|
| 1045.6616 | 1045.5433 | -0.1183 | -113 | 10 | 19 | VLGTKILSSK | | | | | Mascot |
| 1095.5259 | 1095.5037 | -0.0222 | -20 | 118 | 125 | WHFYDTVK | | | | | Mascot |
| 1108.5746 | 1108.5502 | -0.0244 | -22 | 248 | 258 | NTVIATGGYGR | | | | | Mascot |
| 1108.5746 | 1108.5502 | -0.0244 | -22 | 248 | 258 | NTVIATGGYGR | 82 | 100 | | | Mascot |
| 1127.5745 | 1127.5524 | -0.0221 | -20 | 613 | 620 | KPFEQHWR | 51 | 99.81 | | | Mascot |
| 1127.5745 | 1127.5524 | -0.0221 | -20 | 613 | 620 | KPFEQHWR | | | | | Mascot |
| 1330.6597 | 1330.6335 | -0.0262 | -20 | 310 | 322 | GEGGILINSEGER | | | | | Mascot |
| 1354.6709 | 1354.6934 | 0.0225 | 17 | 498 | 509 | YANGSTRTSEIR | | | | | Mascot |
| 1354.7227 | 1354.6934 | -0.0293 | -22 | 193 | 204 | TGHSLLHTLYGR | 73 | 99.999 | | | Mascot |
| 1473.8424 | 1473.8102 | -0.0322 | -22 | 449 | 462 | LGANSLLDLVVFGR | | | | | Mascot |
| 1473.8424 | 1473.8102 | -0.0322 | -22 | 449 | 462 | LGANSLLDLVVFGR | 132 | 100 | | | Mascot |
| 1557.7512 | 1557.7168 | -0.0344 | -22 | 230 | 243 | GVIALCMEDGSIHR | | | Carbamidomethyl (C)[6] | | Mascot |
| 1573.7461 | 1573.7124 | -0.0337 | -21 | 230 | 243 | GVIALCMEDGSIHR | | | Carbamidomethyl (C)[6], Oxidation (M)[7] | | Mascot |
| 1743.837 | 1743.7939 | -0.0431 | -25 | 73 | 89 | AAFGLSEAGFNTACVTK | | | Carbamidomethyl (C)[14] | | Mascot |
| 1768.8752 | 1768.832 | -0.0432 | -24 | 598 | 612 | VDEYDYSKPLQGQVK | | | | | Mascot |
| 2040.0033 | 2039.9558 | -0.0475 | -23 | 596 | 612 | DRVDEYDYSKPLQGQVK | | | | | Mascot |
| 2110.1416 | 2110.0537 | -0.0879 | -42 | 359 | 376 | DHVHLQLHHLPPQQLAA R | | | | | Mascot |
| 2247.9758 | 2247.9194 | -0.0564 | -25 | 259 | 279 | TFFSCTSAHTSTGDGNA | | | Carbamidomethyl (C)[5] | | Mascot |

| | | | | | | | | | | | |
|---|---|---|---|---|---|---|---|---|---|---|---|
| | | | | | | MVTR | | | | | |
| 2263.9707 | 2263.9163 | -0.0544 | -24 | 259 | 279 | TFFSCTSAHTSTGDGNA MVTR | | | Carbamidomethyl (C)[5], Oxidation (M)[18] | | Mascot |
| 2663.4277 | 2663.3643 | -0.0634 | -24 | 353 | 376 | GVGPDKDHVHLQLHHLP PQQLAAR | | | | | Mascot |
| 2663.4277 | 2663.3643 | -0.0634 | -24 | 353 | 376 | GVGPDKDHVHLQLHHLP PQQLAAR | 152 | 100 | | | Mascot |
| 2697.1892 | 2697.1252 | -0.064 | -24 | 423 | 448 | DGQDHVVPGLYACGEAG CASVHGANR | | | Carbamidomethyl (C)[13,18] | | Mascot |
| 2697.1892 | 2697.1252 | -0.064 | -24 | 423 | 448 | DGQDHVVPGLYACGEAG CASVHGANR | 143 | 100 | Carbamidomethyl (C)[13,18] | | Mascot |
| 2871.4556 | 2871.394 | -0.0616 | -21 | 634 | 659 | VTLEYRPVIDSSLDAEDC AAIPPAIR | 73 | 99.999 | Carbamidomethyl (C)[18] | | Mascot |
| 2871.4556 | 2871.394 | -0.0616 | -21 | 634 | 659 | VTLEYRPVIDSSLDAEDC AAIPPAIR | | | Carbamidomethyl (C)[18] | | Mascot |
| 2928.4485 | 2928.3816 | -0.0669 | -23 | 44 | 72 | ISDGVSNQYPVVDHEFD AVVVGAGGAGLR | 215 | 100 | | | Mascot |
| 2928.4485 | 2928.3816 | -0.0669 | -23 | 44 | 72 | ISDGVSNQYPVVDHEFD AVVVGAGGAGLR | | | | | Mascot |
| 3337.5325 | 3337.6738 | 0.1413 | 42 | 248 | 279 | NTVIATGGYGRTFFSCTS AHTSTGDGNAMVTR | | | Carbamidomethyl (C)[16] | | Mascot |
| 3366.5703 | 3366.4951 | -0.0752 | -22 | 280 | 309 | AGLPCQDLEFVQFHPTGI YGAGCLITEGCR | | | Carbamidomethyl (C)[5,23,29] | | Mascot |
| 3366.5703 | 3366.4951 | -0.0752 | -22 | 280 | 309 | AGLPCQDLEFVQFHPTGI YGAGCLITEGCR | 150 | 100 | Carbamidomethyl (C)[5,23,29] | | Mascot |

| | | | | | | | | | | |
|---|---|---|---|---|---|---|---|---|---|---|
| 2 | RecName: Full=Succinate dehydrogenase [ubiquinone] flavoprotein subunit, mitochondrial; AltName: Fu | gi\|205831554 | 72031.6 | 6.14 | 20 | 1,190 | 100 | 1070 | 100 | |

**Peptide Information**

| Calc. Mass | Obsrv. Mass | ± da | ± ppm | Start Seq. | End Seq. | Sequence | Ion Score | C. I. % | Modification | Rank | Result Type |
|---|---|---|---|---|---|---|---|---|---|---|---|
| 1095.5259 | 1095.5037 | -0.0222 | -20 | 118 | 125 | WHFYDTVK | | | | | Mascot |
| 1108.5746 | 1108.5502 | -0.0244 | -22 | 248 | 258 | NTVIATGGYGR | | | | | Mascot |
| 1108.5746 | 1108.5502 | -0.0244 | -22 | 248 | 258 | NTVIATGGYGR | 82 | 100 | | | Mascot |
| 1118.6602 | 1118.634 | -0.0262 | -23 | 10 | 19 | VLGTKILSCK | | | Carbamidomethyl (C)[9] | | Mascot |
| 1127.5745 | 1127.5524 | -0.0221 | -20 | 613 | 620 | KPFEQHWR | 51 | 99.81 | | | Mascot |
| 1127.5745 | 1127.5524 | -0.0221 | -20 | 613 | 620 | KPFEQHWR | | | | | Mascot |
| 1330.6597 | 1330.6335 | -0.0262 | -20 | 310 | 322 | GEGGILINSEGER | | | | | Mascot |
| 1354.6709 | 1354.6934 | 0.0225 | 17 | 498 | 509 | YANGSTRTSEIR | | | | | Mascot |
| 1354.7227 | 1354.6934 | -0.0293 | -22 | 193 | 204 | TGHSLLHTLYGR | 73 | 99.999 | | | Mascot |
| 1473.8424 | 1473.8102 | -0.0322 | -22 | 449 | 462 | LGANSLLDLVVFGR | | | | | Mascot |
| 1473.8424 | 1473.8102 | -0.0322 | -22 | 449 | 462 | LGANSLLDLVVFGR | 132 | 100 | | | Mascot |
| 1557.7512 | 1557.7168 | -0.0344 | -22 | 230 | 243 | GVIALCMEDGSIHR | | | Carbamidomethyl (C)[6] | | Mascot |
| 1573.7461 | 1573.7124 | -0.0337 | -21 | 230 | 243 | GVIALCMEDGSIHR | | | Carbamidomethyl (C)[6], Oxidation (M)[7] | | Mascot |
| 1743.837 | 1743.7939 | -0.0431 | -25 | 73 | 89 | AAFGLSEAGFNTACVTK | | | Carbamidomethyl (C)[14] | | Mascot |
| 1768.8752 | 1768.832 | -0.0432 | -24 | 598 | 612 | VDEYDYSKPLQGQVK | | | | | Mascot |
| 2040.0033 | 2039.9558 | -0.0475 | -23 | 596 | 612 | DRVDEYDYSKPLQGQVK | | | | | Mascot |

| | | | | | | | | | | | |
|---|---|---|---|---|---|---|---|---|---|---|---|
| 2110.1416 | 2110.0537 | -0.0879 | -42 | 359 | 376 | DHVHLQLHHLPPQQLAAR | | | | | Mascot |
| 2247.9758 | 2247.9194 | -0.0564 | -25 | 259 | 279 | TFFSCTSAHTSTGDGNAMVTR | | | Carbamidomethyl (C)[5] | | Mascot |
| 2263.9707 | 2263.9163 | -0.0544 | -24 | 259 | 279 | TFFSCTSAHTSTGDGNAMVTR | | | Carbamidomethyl (C)[5], Oxidation (M)[18] | | Mascot |
| 2663.4277 | 2663.3643 | -0.0634 | -24 | 353 | 376 | GVGPDKDHVHLQLHHLPPQQLAAR | | | | | Mascot |
| 2663.4277 | 2663.3643 | -0.0634 | -24 | 353 | 376 | GVGPDKDHVHLQLHHLPPQQLAAR | 152 | 100 | | | Mascot |
| 2697.1892 | 2697.1252 | -0.064 | -24 | 423 | 448 | DGQDHVVPGLYACGEAGCASVHGANR | | | Carbamidomethyl (C)[13,18] | | Mascot |
| 2697.1892 | 2697.1252 | -0.064 | -24 | 423 | 448 | DGQDHVVPGLYACGEAGCASVHGANR | 143 | 100 | Carbamidomethyl (C)[13,18] | | Mascot |
| 2871.4556 | 2871.394 | -0.0616 | -21 | 634 | 659 | VTLEYRPVIDSSLDAEDCAAIPPAIR | 73 | 99.999 | Carbamidomethyl (C)[18] | | Mascot |
| 2871.4556 | 2871.394 | -0.0616 | -21 | 634 | 659 | VTLEYRPVIDSSLDAEDCAAIPPAIR | | | Carbamidomethyl (C)[18] | | Mascot |
| 2928.4485 | 2928.3816 | -0.0669 | -23 | 44 | 72 | ISDGVSNQYPVVDHEFDAVVVGAGGAGLR | 215 | 100 | | | Mascot |
| 2928.4485 | 2928.3816 | -0.0669 | -23 | 44 | 72 | ISDGVSNQYPVVDHEFDAVVVGAGGAGLR | | | | | Mascot |
| 3337.5325 | 3337.6738 | 0.1413 | 42 | 248 | 279 | NTVIATGGYGRTFFSCTSAHTSTGDGNAMVTR | | | Carbamidomethyl (C)[16] | | Mascot |
| 3366.5703 | 3366.4951 | -0.0752 | -22 | 280 | 309 | AGLPCQDLEFVQFHPTGIYGAGCLITEGCR | | | Carbamidomethyl (C)[5,23,29] | | Mascot |
| 3366.5703 | 3366.4951 | -0.0752 | -22 | 280 | 309 | AGLPCQDLEFVQFHPTGIYGAGCLITEGCR | 150 | 100 | Carbamidomethyl (C)[5,23,29] | | Mascot |

| | | | | | | | | | |
|---|---|---|---|---|---|---|---|---|---|
| 3 | succinate dehydrogenase complex, subunit A, flavoprotein (Fp) [Danio rerio] | gi\|37362284 | 72611.9 | 6.1 | 15 | 906 | 100 | 838 | 100 |

**Peptide Information**

| Calc. Mass | Obsrv. Mass | ± da | ± ppm | Start Seq. | End Seq. | Sequence | Ion Score | C. I. % | Modification | Rank | Result Type |
|---|---|---|---|---|---|---|---|---|---|---|---|
| 1095.5259 | 1095.5037 | -0.0222 | -20 | 118 | 125 | WHFYDTVK | | | | | Mascot |
| 1118.6602 | 1118.634 | -0.0262 | -23 | 10 | 19 | VLGTKILSCK | | | Carbamidomethyl (C)[9] | | Mascot |
| 1127.5745 | 1127.5524 | -0.0221 | -20 | 615 | 622 | KPFEQHWR | 51 | 99.81 | | | Mascot |
| 1127.5745 | 1127.5524 | -0.0221 | -20 | 615 | 622 | KPFEQHWR | | | | | Mascot |
| 1330.6597 | 1330.6335 | -0.0262 | -20 | 312 | 324 | GEGGILINSEGER | | | | | Mascot |
| 1354.6709 | 1354.6934 | 0.0225 | 17 | 500 | 511 | YANGSTRTSEIR | | | | | Mascot |
| 1354.7227 | 1354.6934 | -0.0293 | -22 | 193 | 204 | TGHSLLHTLYGR | 73 | 99.999 | | | Mascot |
| 1473.8424 | 1473.8102 | -0.0322 | -22 | 451 | 464 | LGANSLLDLVVFGR | | | | | Mascot |
| 1473.8424 | 1473.8102 | -0.0322 | -22 | 451 | 464 | LGANSLLDLVVFGR | 132 | 100 | | | Mascot |
| 1743.837 | 1743.7939 | -0.0431 | -25 | 73 | 89 | AAFGLSEAGFNTACVTK | | | Carbamidomethyl (C)[14] | | Mascot |
| 1768.8752 | 1768.832 | -0.0432 | -24 | 600 | 614 | VDEYDYSKPLQGQVK | | | | | Mascot |
| 2040.0033 | 2039.9558 | -0.0475 | -23 | 598 | 614 | DRVDEYDYSKPLQGQVK | | | | | Mascot |
| 2110.1416 | 2110.0537 | -0.0879 | -42 | 361 | 378 | DHVHLQLHHLPPQQLAAR | | | | | Mascot |
| 2663.4277 | 2663.3643 | -0.0634 | -24 | 355 | 378 | GVGPDKDHVHLQLHHLPPQQLAAR | | | | | Mascot |

| | | | | | | | | | | | |
|---|---|---|---|---|---|---|---|---|---|---|---|
| 2663.4277 | 2663.3643 | -0.0634 | -24 | 355 | 378 | GVGPDKDHVHLQLHHLP PQQLAAR | 152 | 100 | | | Mascot |
| 2697.1892 | 2697.1252 | -0.064 | -24 | 425 | 450 | DGQDHVVPGLYACGEAG CASVHGANR | | | Carbamidomethyl (C)[13,18] | | Mascot |
| 2697.1892 | 2697.1252 | -0.064 | -24 | 425 | 450 | DGQDHVVPGLYACGEAG CASVHGANR | 143 | 100 | Carbamidomethyl (C)[13,18] | | Mascot |
| 2871.4556 | 2871.394 | -0.0616 | -21 | 636 | 661 | VTLEYRPVIDSSLDAEDC AAIPPAIR | 73 | 99.999 | Carbamidomethyl (C)[18] | | Mascot |
| 2871.4556 | 2871.394 | -0.0616 | -21 | 636 | 661 | VTLEYRPVIDSSLDAEDC AAIPPAIR | | | Carbamidomethyl (C)[18] | | Mascot |
| 2928.4485 | 2928.3816 | -0.0669 | -23 | 44 | 72 | ISDGVSNQYPVVDHEFD AVVVGAGGAGLR | 215 | 100 | | | Mascot |
| 2928.4485 | 2928.3816 | -0.0669 | -23 | 44 | 72 | ISDGVSNQYPVVDHEFD AVVVGAGGAGLR | | | | | Mascot |

4 Wu:fj82b07 protein [Danio rerio] gi|58701969 62105.9 6.04 10 48 20.865

**Peptide Information**

| Calc. Mass | Obsrv. Mass | ± da | ± ppm | Start Seq. | End Seq. | Sequence | Ion Score | C. I. % | Modification | Rank | Result Type |
|---|---|---|---|---|---|---|---|---|---|---|---|
| 996.5876 | 996.5761 | -0.0115 | -12 | 528 | 535 | LLGQFYKK | | | | | Mascot |
| 1127.7035 | 1127.5524 | -0.1511 | -134 | 376 | 385 | ALEVLQLTLK | 11 | 0 | | | Mascot |
| 1127.7035 | 1127.5524 | -0.1511 | -134 | 376 | 385 | ALEVLQLTLK | | | | | Mascot |
| 1174.5851 | 1174.553 | -0.0321 | -27 | 224 | 232 | EVWQEQTVR | | | | | Mascot |
| 1330.6862 | 1330.6335 | -0.0527 | -40 | 224 | 233 | EVWQEQTVRR | | | | | Mascot |
| 1720.8655 | 1720.9137 | 0.0482 | 28 | 112 | 125 | ALAVRLCQVLMDCR | | | Carbamidomethyl (C)[7,13], Oxidation (M)[11] | | Mascot |
| 2022.0742 | 2021.9862 | -0.088 | -44 | 334 | 349 | LLLFHVLEKHYGHMER | | | | | Mascot |
| 2211.1326 | 2211.053 | -0.0796 | -36 | 203 | 223 | LDLTGLVGSSPDECLPPS VVR | | | Carbamidomethyl (C)[14] | | Mascot |
| 2680.3213 | 2680.2212 | -0.1001 | -37 | 252 | 275 | IVSPPSRGSNNEPDLLYT SSYLDR | | | | | Mascot |
| 2697.2283 | 2697.1252 | -0.1031 | -38 | 80 | 102 | THENCFLGCEAVDVLQD YLTQNK | | | Carbamidomethyl (C)[5] | | Mascot |
| 2697.2283 | 2697.1252 | -0.1031 | -38 | 80 | 102 | THENCFLGCEAVDVLQD YLTQNK | | | Carbamidomethyl (C)[5] | | Mascot |
| 3366.6997 | 3366.4951 | -0.2046 | -61 | 203 | 232 | LDLTGLVGSSPDECLPPS VVREVWQEQTVR | | | Carbamidomethyl (C)[14] | | Mascot |
| 3366.6997 | 3366.4951 | -0.2046 | -61 | 203 | 232 | LDLTGLVGSSPDECLPPS VVREVWQEQTVR | | | Carbamidomethyl (C)[14] | | Mascot |

5 zinc finger protein 432-like [Danio rerio] gi|71834484 50250.7 8.61 14 46 0 6 0

**Peptide Information**

| Calc. Mass | Obsrv. Mass | ± da | ± ppm | Start Seq. | End Seq. | Sequence | Ion Score | C. I. % | Modification | Rank | Result Type |
|---|---|---|---|---|---|---|---|---|---|---|---|
| 987.4676 | 987.6115 | 0.1439 | 146 | 314 | 321 | ENLTSHMR | | | | | Mascot |
| 1095.5112 | 1095.5037 | -0.0075 | -7 | 231 | 238 | DHLDRHMR | | | Oxidation (M)[7] | | Mascot |
| 1564.7537 | 1564.8866 | 0.1329 | 85 | 309 | 321 | SFSQKENLTSHMR | | | | | Mascot |
| 1638.725 | 1638.6833 | -0.0417 | -25 | 294 | 308 | VHTGEKPYSCTECGK | | | | | Mascot |

| Calc. Mass | Obsrv. Mass | ± da | ± ppm | Start Seq. | End Seq. | Sequence | Ion Score | C. I. % | Modification | Rank | Result Type |
|---|---|---|---|---|---|---|---|---|---|---|---|
| 1720.829 | 1720.9137 | 0.0847 | 49 | 422 | 436 | DLDVKFPQPTHEASH | | | | | Mascot |
| 1768.7563 | 1768.832 | 0.0757 | 43 | 378 | 392 | THTGERPYTCMQCGK | | | Carbamidomethyl (C)[10] | | Mascot |
| 2086.9104 | 2086.842 | -0.0684 | -33 | 95 | 113 | VHMGIHTGENLSTCDQC GK | | | Carbamidomethyl (C)[14] | | Mascot |
| 2216.011 | 2215.9321 | -0.0789 | -36 | 294 | 313 | VHTGEKPYSCTECGKSF SQK | | | | | Mascot |
| 2247.9844 | 2247.9194 | -0.065 | -29 | 124 | 141 | HMKTHSGEKPFWCTECG R | | | Carbamidomethyl (C)[13,16] | | Mascot |
| 2263.9792 | 2263.9163 | -0.0629 | -28 | 124 | 141 | HMKTHSGEKPFWCTECG R | | | Carbamidomethyl (C)[13,16], Oxidation (M)[2] | | Mascot |
| 2283.0393 | 2283.125 | 0.0857 | 38 | 403 | 421 | HMKTHHTGTKPFACDDV ER | | | Carbamidomethyl (C)[14], Oxidation (M)[2] | | Mascot |
| 2384.1299 | 2383.8892 | -0.2407 | -101 | 406 | 426 | THHTGTKPFACDDVERD LDVK | | | | | Mascot |
| 2559.1748 | 2559.1748 | 0 | 0 | 91 | 113 | QSLKVHMGIHTGENLSTC DQCGK | 6 | 0 | Carbamidomethyl (C)[21], Oxidation (M)[7] | | Mascot |
| 2559.1748 | 2559.1748 | 0 | 0 | 91 | 113 | QSLKVHMGIHTGENLSTC DQCGK | | | Carbamidomethyl (C)[18], Oxidation (M)[7] | | Mascot |
| 2680.1912 | 2680.2212 | 0.03 | 11 | 95 | 118 | VHMGIHTGENLSTCDQC GKSFNTK | | | Carbamidomethyl (C)[14], Oxidation (M)[3] | | Mascot |
| 2853.2751 | 2853.3992 | 0.1241 | 43 | 314 | 336 | ENLTSHMRIHTGEKPFAC EYCEK | | | Carbamidomethyl (C)[18,21], Oxidation (M)[7] | | Mascot |

6 PREDICTED: gastrula zinc finger protein XlCGF57.1-like [Danio rerio] gi|326667039 42016.4 9.37 13 45 0

**Peptide Information**

| Calc. Mass | Obsrv. Mass | ± da | ± ppm | Start Seq. | End Seq. | Sequence | Ion Score | C. I. % | Modification | Rank | Result Type |
|---|---|---|---|---|---|---|---|---|---|---|---|
| 900.4282 | 900.4632 | 0.035 | 39 | 327 | 333 | THSREDR | | | | | Mascot |
| 987.4676 | 987.6115 | 0.1439 | 146 | 95 | 102 | SNLDVHMR | | | Oxidation (M)[7] | | Mascot |
| 996.5446 | 996.5761 | 0.0315 | 32 | 67 | 74 | TSSHRRPR | | | | | Mascot |
| 1174.4979 | 1174.553 | 0.0551 | 47 | 76 | 85 | SKSGCNFSCK | | | Carbamidomethyl (C)[5,9] | | Mascot |
| 1548.7588 | 1548.9131 | 0.1543 | 100 | 90 | 102 | SFSQKSNLDVHMR | | | | | Mascot |
| 1564.7537 | 1564.8866 | 0.1329 | 85 | 90 | 102 | SFSQKSNLDVHMR | | | Oxidation (M)[12] | | Mascot |
| 1605.7015 | 1605.7881 | 0.0866 | 54 | 174 | 186 | SFYHAGHFAEHMR | | | Oxidation (M)[12] | | Mascot |
| 1707.7941 | 1707.7482 | -0.0459 | -27 | 103 | 117 | THTGEKPFSCKQCGK | | | Carbamidomethyl (C)[10] | | Mascot |
| 1720.8258 | 1720.9137 | 0.0879 | 51 | 159 | 173 | IHTGERPYTCQKCGK | | | | | Mascot |
| 1974.9087 | 1975.0669 | 0.1582 | 80 | 348 | 364 | SLNTHMKLHNGEESPQH | | | Oxidation (M)[6] | | Mascot |
| 2211.0684 | 2211.053 | -0.0154 | -7 | 271 | 290 | IHTGEKPFACAQCGKSFT TK | | | Carbamidomethyl (C)[10] | | Mascot |
| 2536.1489 | 2536.1211 | -0.0278 | -11 | 295 | 317 | NHMNGHTGTIVFTCDQC GKSLTR | | | Oxidation (M)[3] | | Mascot |
| 2606.1909 | 2606.1165 | -0.0744 | -29 | 291 | 313 | TSLKNHMNGHTGTIVFTC DQCGK | | | Carbamidomethyl (C)[18,21] | | Mascot |
| 2853.3115 | 2853.3992 | 0.0877 | 31 | 235 | 257 | QDLYIHMRIHTGEKPYTC TECGK | | | Carbamidomethyl (C)[18,21], Oxidation (M)[7] | | Mascot |

7 PREDICTED: zinc finger protein 180-like [Danio rerio] gi|292613226 44120.8 9.2 12 43 0

**Peptide Information**

| Calc. Mass | Obsrv. Mass | ± da | ± ppm | Start Seq. | End Seq. | Sequence | Ion Score | C. I. % | Modification | Rank | Result Type |
|---|---|---|---|---|---|---|---|---|---|---|---|
| 1108.5382 | 1108.5502 | 0.012 | 11 | 374 | 383 | LHNGEQSPLN | | | | | Mascot |
| 1108.5382 | 1108.5502 | 0.012 | 11 | 374 | 383 | LHNGEQSPLN | | | | | Mascot |
| 1127.4642 | 1127.5524 | 0.0882 | 78 | 268 | 276 | ICTCTQCGK | | | Carbamidomethyl (C)[2,4,7] | | Mascot |
| 1127.4642 | 1127.5524 | 0.0882 | 78 | 268 | 276 | ICTCTQCGK | | | Carbamidomethyl (C)[2,4,7] | | Mascot |
| 1165.6259 | 1165.7202 | 0.0943 | 81 | 281 | 289 | KQSLHIHMR | | | Oxidation (M)[8] | | Mascot |
| 1174.4802 | 1174.553 | 0.0728 | 62 | 71 | 80 | CNFSCKQCGK | | | Carbamidomethyl (C)[1] | | Mascot |
| 1534.7794 | 1534.6222 | -0.1572 | -102 | 165 | 177 | SFKQNGTLTVHMR | | | Oxidation (M)[12] | | Mascot |
| 1605.7512 | 1605.7881 | 0.0369 | 23 | 318 | 332 | THTGEKPFACVQCGK | | | | | Mascot |
| 1620.6749 | 1620.7225 | 0.0476 | 29 | 268 | 280 | ICTCTQCGKCFSR | | | Carbamidomethyl (C)[2,4,7] | | Mascot |
| 1720.8146 | 1720.9137 | 0.0991 | 58 | 234 | 248 | IHTGEKPYSCLQCGK | | | Carbamidomethyl (C)[10] | | Mascot |
| 1768.8488 | 1768.832 | -0.0168 | -9 | 6 | 20 | EESEDVKIEDTFTVK | | | | | Mascot |
| 1974.9636 | 1975.0669 | 0.1033 | 52 | 77 | 93 | QCGKSFSQKPNLDVHMR | | | | | Mascot |
| 3337.4573 | 3337.6738 | 0.2165 | 65 | 178 | 205 | THTGERPYTCQQCGKSF YTSENFTQHMR | | | | | Mascot |
| 3366.5103 | 3366.4951 | -0.0152 | -5 | 122 | 149 | IHTGERPYTCQQCGQTF YHVGNFAAHMR | | | Carbamidomethyl (C)[10,13] | | Mascot |
| 3366.5103 | 3366.4951 | -0.0152 | -5 | 122 | 149 | IHTGERPYTCQQCGQTF YHVGNFAAHMR | | | Carbamidomethyl (C)[10,13] | | Mascot |

8 synaptonemal complex protein 3 [Danio rerio] gi|94536667 27983.3 8.55 8 42 0

**Peptide Information**

| Calc. Mass | Obsrv. Mass | ± da | ± ppm | Start Seq. | End Seq. | Sequence | Ion Score | C. I. % | Modification | Rank | Result Type |
|---|---|---|---|---|---|---|---|---|---|---|---|
| 850.4127 | 850.527 | 0.1143 | 134 | 111 | 116 | LEQMWK | | | Oxidation (M)[4] | | Mascot |
| 1045.5049 | 1045.5433 | 0.0384 | 37 | 44 | 52 | DDETPIIDK | | | | | Mascot |
| 1605.7802 | 1605.7881 | 0.0079 | 5 | 111 | 122 | LEQMWKTQQNQR | | | Oxidation (M)[4] | | Mascot |
| 2021.8782 | 2021.9862 | 0.108 | 53 | 35 | 52 | ESGSDEDTRDDETPIIDK | | | | | Mascot |
| 2233.0806 | 2233.0376 | -0.043 | -19 | 178 | 195 | TINDLHEQFVKNIEEMEK | | | Oxidation (M)[16] | | Mascot |
| 2264.1445 | 2263.9163 | -0.2282 | -101 | 14 | 33 | HTDGVVTDLKAFDFTVLE EK | | | | | Mascot |
| 2536.1807 | 2536.1211 | -0.0596 | -23 | 189 | 209 | NIEEMEKSHEAFLQGTQ MELR | | | Oxidation (M)[5] | | Mascot |
| 2871.4636 | 2871.394 | -0.0696 | -24 | 123 | 146 | QKLTQDHSQQVFSVLQQ WESDVLK | | | | | Mascot |
| 2871.4636 | 2871.394 | -0.0696 | -24 | 123 | 146 | QKLTQDHSQQVFSVLQQ WESDVLK | | | | | Mascot |

9 Zgc:55673 [Danio rerio] gi|28278370 41830.1 9.58 9 41 0

**Peptide Information**

| Calc. Mass | Obsrv. Mass | ± da | ± ppm | Start | End | Sequence | Ion | C. I. % | Modification | Rank | Result Type |
|---|---|---|---|---|---|---|---|---|---|---|---|

| Calc. Mass | Obsrv. Mass | ± da | ± ppm | Start Seq. | End Seq. | Sequence | Ion Score | C. I. % | Modification | Rank | Result Type |
|---|---|---|---|---|---|---|---|---|---|---|---|
| 939.5158 | 939.5073 | -0.0085 | -9 | 351 | 358 | YLRAGGFR | | | | | Mascot |
| 996.4996 | 996.5761 | 0.0765 | 77 | 2 | 11 | SSGTPYIGSK | | | | | Mascot |
| 1127.5402 | 1127.5524 | 0.0122 | 11 | 1 | 11 | MSSGTPYIGSK | | | | | Mascot |
| 1127.5402 | 1127.5524 | 0.0122 | 11 | 1 | 11 | MSSGTPYIGSK | | | | | Mascot |
| 1190.6012 | 1190.5504 | -0.0508 | -43 | 194 | 205 | GDSSSAQQAVLK | | | | | Mascot |
| 1557.7656 | 1557.7168 | -0.0488 | -31 | 318 | 330 | SFFDNISSELRSR | | | | | Mascot |
| 1620.8856 | 1620.7225 | -0.1631 | -101 | 340 | 353 | LNVETFGVPGRYLR | | | | | Mascot |
| 1768.9513 | 1768.832 | -0.1193 | -67 | 1 | 17 | MSSGTPYIGSKISLISK | | | | | Mascot |
| 2087.0542 | 2086.842 | -0.2122 | -102 | 22 | 40 | YEGILYTIDTDNSTVALAK | | | | | Mascot |
| 2853.416 | 2853.3992 | -0.0168 | -6 | 150 | 174 | GPMVEQAVQTVPAEKTQ QQQQQQQK | | | Oxidation (M)[3] | | Mascot |

| | | | | | | | |
|---|---|---|---|---|---|---|---|
| 10 | PREDICTED: DEP domain-containing protein 7 [Danio rerio] | gi\|292628040 | 69437.4 | 5.37 | 9 | 40 | 0 |

**Peptide Information**

| Calc. Mass | Obsrv. Mass | ± da | ± ppm | Start Seq. | End Seq. | Sequence | Ion Score | C. I. % | Modification | Rank | Result Type |
|---|---|---|---|---|---|---|---|---|---|---|---|
| 1127.7035 | 1127.5524 | -0.1511 | -134 | 424 | 433 | ALEVLQLTLK | 11 | 0 | | | Mascot |
| 1127.7035 | 1127.5524 | -0.1511 | -134 | 424 | 433 | ALEVLQLTLK | | | | | Mascot |
| 1174.5851 | 1174.553 | -0.0321 | -27 | 272 | 280 | EVWQEQTVR | | | | | Mascot |
| 1330.6862 | 1330.6335 | -0.0527 | -40 | 272 | 281 | EVWQEQTVRR | | | | | Mascot |
| 1720.8655 | 1720.9137 | 0.0482 | 28 | 160 | 173 | ALAVRLCQVLMDCR | | | Carbamidomethyl (C)[7,13], Oxidation (M)[11] | | Mascot |
| 2022.0742 | 2021.9862 | -0.088 | -44 | 382 | 397 | LLLFHVLEKHYGHMER | | | | | Mascot |
| 2211.1326 | 2211.053 | -0.0796 | -36 | 251 | 271 | LDLTGLVGSSPDECLPPS VVR | | | Carbamidomethyl (C)[14] | | Mascot |
| 2680.3213 | 2680.2212 | -0.1001 | -37 | 300 | 323 | IVSPPSRGSNNEPDLLYT SSYLDR | | | | | Mascot |
| 2697.2283 | 2697.1252 | -0.1031 | -38 | 128 | 150 | THENCFLGCEAVDVLQD YLTQNK | | | Carbamidomethyl (C)[5] | | Mascot |
| 2697.2283 | 2697.1252 | -0.1031 | -38 | 128 | 150 | THENCFLGCEAVDVLQD YLTQNK | | | Carbamidomethyl (C)[5] | | Mascot |
| 3366.6997 | 3366.4951 | -0.2046 | -61 | 251 | 280 | LDLTGLVGSSPDECLPPS VVREVWQEQTVR | | | Carbamidomethyl (C)[14] | | Mascot |
| 3366.6997 | 3366.4951 | -0.2046 | -61 | 251 | 280 | LDLTGLVGSSPDECLPPS VVREVWQEQTVR | | | Carbamidomethyl (C)[14] | | Mascot |

| | | | | | | | |
|---|---|---|---|---|---|---|---|
| 11 | PREDICTED: apolipoprotein B-100 [Danio rerio] | gi\|326675669 | 478034.2 | 5.42 | 30 | 38 | 0 |

**Peptide Information**

| Calc. Mass | Obsrv. Mass | ± da | ± ppm | Start Seq. | End Seq. | Sequence | Ion Score | C. I. % | Modification | Rank | Result Type |
|---|---|---|---|---|---|---|---|---|---|---|---|
| 900.4097 | 900.4632 | 0.0535 | 59 | 2163 | 2169 | AFDEQYK | | | | | Mascot |

| | | | | | | | | | | |
|---|---|---|---|---|---|---|---|---|---|---|
| 1045.5347 | 1045.5433 | 0.0086 | 8 | 931 | 939 | IVPSMVEDR | | | | Mascot |
| 1051.5756 | 1051.6887 | 0.1131 | 108 | 3778 | 3785 | HDLRQNLR | | | | Mascot |
| 1095.481 | 1095.5037 | 0.0227 | 21 | 522 | 530 | LMEMDSDVR | | | | Mascot |
| 1118.6052 | 1118.634 | 0.0288 | 26 | 274 | 283 | QSLTLQDSVK | | | | Mascot |
| 1127.4707 | 1127.5524 | 0.0817 | 72 | 522 | 530 | LMEMDSDVR | 2 | 0 | Oxidation (M)[2,4] | Mascot |
| 1127.4707 | 1127.5524 | 0.0817 | 72 | 522 | 530 | LMEMDSDVR | | | Oxidation (M)[2,4] | Mascot |
| 1330.6936 | 1330.6335 | -0.0601 | -45 | 713 | 723 | WISPLNGERMK | | | | Mascot |
| 1354.7114 | 1354.6934 | -0.018 | -13 | 1250 | 1260 | SDALLRYQFNK | | | | Mascot |
| 1354.7114 | 1354.6934 | -0.018 | -13 | 1250 | 1260 | SDALLRYQFNK | | | | Mascot |
| 1363.6998 | 1363.6482 | -0.0516 | -38 | 3206 | 3217 | RNMEASLTNLAK | | | Oxidation (M)[3] | Mascot |
| 1380.7369 | 1380.7235 | -0.0134 | -10 | 2448 | 2459 | NIKDVIDTTAYK | | | | Mascot |
| 1473.693 | 1473.8102 | 0.1172 | 80 | 2425 | 2436 | EMQATVFDYLEK | | | | Mascot |
| 1473.693 | 1473.8102 | 0.1172 | 80 | 2425 | 2436 | EMQATVFDYLEK | | | | Mascot |
| 1534.7708 | 1534.6222 | -0.1486 | -97 | 1657 | 1671 | GSSQDNTIELSAKGK | | | | Mascot |
| 1548.6998 | 1548.9131 | 0.2133 | 138 | 733 | 744 | SMEEYISEFKNR | | | Oxidation (M)[2] | Mascot |
| 1557.7573 | 1557.7168 | -0.0405 | -26 | 2345 | 2357 | MMEIMDSIVSLLK | | | Oxidation (M)[1,2,5] | Mascot |
| 1620.7906 | 1620.7225 | -0.0681 | -42 | 3220 | 3232 | CPVLMMDLNQVLR | | | Carbamidomethyl (C)[1], Oxidation (M)[5,6] | Mascot |
| 1638.7832 | 1638.6833 | -0.0999 | -61 | 605 | 619 | AVQGDVSFPPMDFTK | | | | Mascot |
| 1652.8563 | 1652.8976 | 0.0413 | 25 | 3450 | 3464 | VEMSESVEGKVAFLK | | | | Mascot |
| 1707.9388 | 1707.7482 | -0.1906 | -112 | 1517 | 1531 | TNAQNDVLKHVVELK | | | | Mascot |
| 1768.8972 | 1768.832 | -0.0652 | -37 | 4149 | 4164 | VSGIYKEAGNLEVMMK | | | | Mascot |
| 1974.9478 | 1975.0669 | 0.1191 | 60 | 2612 | 2628 | QIEMPEVDPEAIFGDLR | | | Oxidation (M)[4] | Mascot |
| 2110.0203 | 2110.0537 | 0.0334 | 16 | 1694 | 1712 | NTMNIGINKQGLSMSNTMR | | | | Mascot |
| 2211.1113 | 2211.053 | -0.0583 | -26 | 770 | 788 | TSDLKHVTQYINLCAETFK | | | | Mascot |
| 2216.0176 | 2215.9321 | -0.0855 | -39 | 1161 | 1178 | SDINSEVEKLFPNMEEYR | | | Oxidation (M)[14] | Mascot |
| 2248.0994 | 2247.9194 | -0.18 | -80 | 798 | 815 | LISSLENEFFVHYMFLDK | | | Oxidation (M)[14] | Mascot |
| 2264.0903 | 2263.9163 | -0.174 | -77 | 4112 | 4129 | SEEVFEDLMNYLKVYTQR | | | | Mascot |
| 2284.1416 | 2284.1194 | -0.0222 | -10 | 2731 | 2750 | SSLEFLDYTLDAMLQIEAPK | | | | Mascot |
| 2559.1853 | 2559.1748 | -0.0105 | -4 | 2971 | 2993 | VSGSISNTCDFLAQPFEVTLDCR | | | Carbamidomethyl (C)[9] | Mascot |
| 2559.1853 | 2559.1748 | -0.0105 | -4 | 2971 | 2993 | VSGSISNTCDFLAQPFEVTLDCR | | | Carbamidomethyl (C)[9] | Mascot |
| 2697.3994 | 2697.1252 | -0.2742 | -102 | 2044 | 2066 | FESLQNYLNSLDLNALIGQFRNK | | | | Mascot |
| 2697.3994 | 2697.1252 | -0.2742 | -102 | 2044 | 2066 | FESLQNYLNSLDLNALIGQFRNK | | | | Mascot |
| 2853.3835 | 2853.3992 | 0.0157 | 6 | 3351 | 3377 | IYATMAYTSSNAASIASFSTNGKQNVR | | | | Mascot |
| 2871.3286 | 2871.394 | 0.0654 | 23 | 3707 | 3732 | MASPIYNNTWTGNVENTEAGTVCTLK | | | Carbamidomethyl (C)[23] | Mascot |
| 2871.3286 | 2871.394 | 0.0654 | 23 | 3707 | 3732 | MASPIYNNTWTGNVENT | | | Carbamidomethyl (C)[23] | Mascot |

| | | | | | | | | | | | |
|---|---|---|---|---|---|---|---|---|---|---|---|
| 3337.7654 | 3337.6738 | -0.0916 | -27 | 3516 | 3544 | EAGTVCTLK LFENGVSFNIPQITFGVP DLFKTIPQMIR | | | Oxidation (M)[27] | | Mascot |

12 S-methyl-5'-thioadenosine phosphorylase [Danio rerio] gi|41054629 30827.4 5.99 8 37 0

**Peptide Information**

| Calc. Mass | Obsrv. Mass | ± da | ± ppm | Start Seq. | End Seq. | Sequence | Ion Score | C. I. % | Modification | Rank | Result Type |
|---|---|---|---|---|---|---|---|---|---|---|---|
| 1330.6572 | 1330.6335 | -0.0237 | -18 | 174 | 184 | FSSRAESLMFR | | | | | Mascot |
| 1354.7939 | 1354.6934 | -0.1005 | -74 | 147 | 159 | EVLLEVAQGLGVK | | | | | Mascot |
| 1354.7939 | 1354.6934 | -0.1005 | -74 | 147 | 159 | EVLLEVAQGLGVK | | | | | Mascot |
| 1363.6344 | 1363.6482 | 0.0138 | 10 | 269 | 280 | SMSQSSVMLPKH | | | Oxidation (M)[2,8] | | Mascot |
| 1557.7625 | 1557.7168 | -0.0457 | -29 | 160 | 173 | CHTRGTMVTIEGPR | | | | | Mascot |
| 1573.7573 | 1573.7124 | -0.0449 | -29 | 160 | 173 | CHTRGTMVTIEGPR | | | Oxidation (M)[7] | | Mascot |
| 1903.9 | 1903.8071 | -0.0929 | -49 | 80 | 96 | EEGCTHLLVTTACGSLR | | | Carbamidomethyl (C)[4,13] | | Mascot |
| 2087.0842 | 2086.842 | -0.2422 | -116 | 185 | 203 | QWGADVINMTTVPEVVL AK | | | Oxidation (M)[9] | | Mascot |
| 2224.9348 | 2225.0657 | 0.1309 | 59 | 204 | 222 | EAGLCYASIAMATDYDC WK | | | Carbamidomethyl (C)[5,17] | | Mascot |
| 2697.3511 | 2697.1252 | -0.2259 | -84 | 1 | 26 | MTSNSHVKIGIIGGSGLD DPDILEGR | | | Oxidation (M)[1] | | Mascot |
| 2697.3511 | 2697.1252 | -0.2259 | -84 | 1 | 26 | MTSNSHVKIGIIGGSGLD DPDILEGR | | | Oxidation (M)[1] | | Mascot |

13 LOC100002738 protein [Danio rerio] gi|134026300 92988.4 5.6 10 37 0

**Peptide Information**

| Calc. Mass | Obsrv. Mass | ± da | ± ppm | Start Seq. | End Seq. | Sequence | Ion Score | C. I. % | Modification | Rank | Result Type |
|---|---|---|---|---|---|---|---|---|---|---|---|
| 842.4842 | 842.4929 | 0.0087 | 10 | 602 | 608 | IVDPSRR | | | | | Mascot |
| 1127.5051 | 1127.5524 | 0.0473 | 42 | 614 | 622 | CHYEPHSVR | 11 | 0 | | | Mascot |
| 1127.5051 | 1127.5524 | 0.0473 | 42 | 614 | 622 | CHYEPHSVR | | | | | Mascot |
| 1190.5834 | 1190.5504 | -0.033 | -28 | 429 | 439 | CSVDRGDPITK | | | | | Mascot |
| 1354.6685 | 1354.6934 | 0.0249 | 18 | 614 | 624 | CHYEPHSVRVK | | | | | Mascot |
| 1354.6685 | 1354.6934 | 0.0249 | 18 | 614 | 624 | CHYEPHSVRVK | | | | | Mascot |
| 1363.7004 | 1363.6482 | -0.0522 | -38 | 402 | 413 | IPADNPFVHEPK | | | | | Mascot |
| 1548.7335 | 1548.9131 | 0.1796 | 116 | 225 | 238 | NPLAMVHANDGTHR | | | Oxidation (M)[5] | | Mascot |
| 2225.1641 | 2225.0657 | -0.0984 | -44 | 239 | 257 | FFVAEQVGLVWVYLPDQ SK | | | | | Mascot |
| 2233.259 | 2233.0376 | -0.2214 | -99 | 625 | 644 | SNIIPFEPKETLIGVHIPTK | | | | | Mascot |
| 2559.3049 | 2559.1748 | -0.1301 | -51 | 672 | 694 | EEDGQITTTPFTPTTPKP VRTSR | | | | | Mascot |
| 2559.3049 | 2559.1748 | -0.1301 | -51 | 672 | 694 | EEDGQITTTPFTPTTPKP VRTSR | | | | | Mascot |
| 3338.5549 | 3338.6826 | 0.1277 | 38 | 1 | 28 | LHNFYFHVSEVGSQALS | | | | | Mascot |

VMWYFCDVFGR

| 14 | PREDICTED: zinc finger protein 135-like [Danio rerio] | gi\|326666826 | 49481.4 | 9.54 | 12 | 36 | 0 |
|---|---|---|---|---|---|---|---|

Peptide Information

| Calc. Mass | Obsrv. Mass | ± da | ± ppm | Start Seq. | End Seq. | Sequence | Ion Score | C. I. % | Modification | Rank | Result Type |
|---|---|---|---|---|---|---|---|---|---|---|---|
| 1174.5739 | 1174.553 | -0.0209 | -18 | 325 | 334 | SFSQSTYLNK | | | | | Mascot |
| 1548.77 | 1548.9131 | 0.1431 | 92 | 269 | 281 | SFNRSSSLNLHMR | | | | | Mascot |
| 1557.759 | 1557.7168 | -0.0422 | -27 | 45 | 57 | SFNQPSNLNLHMR | | | | | Mascot |
| 1564.7649 | 1564.8866 | 0.1217 | 78 | 269 | 281 | SFNRSSSLNLHMR | | | Oxidation (M)[12] | | Mascot |
| 1573.754 | 1573.7124 | -0.0416 | -26 | 45 | 57 | SFNQPSNLNLHMR | | | Oxidation (M)[12] | | Mascot |
| 1638.8057 | 1638.6833 | -0.1224 | -75 | 241 | 253 | SFNQLSYLNLHMR | | | Oxidation (M)[12] | | Mascot |
| 1743.8054 | 1743.7939 | -0.0115 | -7 | 170 | 184 | IHTGEKPFTCTHCGR | | | Carbamidomethyl (C)[10] | | Mascot |
| 2086.9731 | 2086.842 | -0.1311 | -63 | 1 | 17 | MIHTGEKPFTCHQCGKR | | | Carbamidomethyl (C)[11,14] | | Mascot |
| 2211.0435 | 2211.053 | 0.0095 | 4 | 282 | 300 | IHTGEKPFTCTQCGKSFN R | | | Carbamidomethyl (C)[10] | | Mascot |
| 2248.0498 | 2247.9194 | -0.1304 | -58 | 170 | 188 | IHTGEKPFTCTHCGRSFN R | | | Carbamidomethyl (C)[10] | | Mascot |
| 2871.3401 | 2871.394 | 0.0539 | 19 | 338 | 362 | IHTGEKPFTCTQCEKSFN QSSSLNK | | | Carbamidomethyl (C)[10] | | Mascot |
| 2871.3401 | 2871.394 | 0.0539 | 19 | 338 | 362 | IHTGEKPFTCTQCEKSFN QSSSLNK | | | Carbamidomethyl (C)[10] | | Mascot |
| 2928.3613 | 2928.3816 | 0.0203 | 7 | 338 | 362 | IHTGEKPFTCTQCEKSFN QSSSLNK | | | Carbamidomethyl (C)[10,13] | | Mascot |
| 2928.3613 | 2928.3816 | 0.0203 | 7 | 338 | 362 | IHTGEKPFTCTQCEKSFN QSSSLNK | | | Carbamidomethyl (C)[10,13] | | Mascot |
| 3337.6179 | 3337.6738 | 0.0559 | 17 | 157 | 184 | IFSNLFSFTIHMRIHTGEK PFTCTHCGR | | | Carbamidomethyl (C)[23] | | Mascot |
| 3338.5979 | 3338.6826 | 0.0847 | 25 | 241 | 268 | SFNQLSYLNLHMRIHTGE KPFTCTQCGR | | | Carbamidomethyl (C)[23] | | Mascot |
| 3366.5676 | 3366.4951 | -0.0725 | -22 | 58 | 85 | IHTGEKPFTCTQCGKNFI HSSHLNEHMR | | | Carbamidomethyl (C)[10,13] | | Mascot |
| 3366.5676 | 3366.4951 | -0.0725 | -22 | 58 | 85 | IHTGEKPFTCTQCGKNFI HSSHLNEHMR | | | Carbamidomethyl (C)[10,13] | | Mascot |

| 15 | Zgc:56530 [Danio rerio] | gi\|29124454 | 11140.5 | 5.91 | 5 | 36 | 0 |
|---|---|---|---|---|---|---|---|

Peptide Information

| Calc. Mass | Obsrv. Mass | ± da | ± ppm | Start Seq. | End Seq. | Sequence | Ion Score | C. I. % | Modification | Rank | Result Type |
|---|---|---|---|---|---|---|---|---|---|---|---|
| 1118.551 | 1118.634 | 0.083 | 74 | 2 | 12 | PLVCGGTSEAK | | | Carbamidomethyl (C)[4] | | Mascot |
| 1380.6134 | 1380.7235 | 0.1101 | 80 | 21 | 32 | ICDQMKADAEEK | | | | | Mascot |
| 1688.9006 | 1688.8339 | -0.0667 | -39 | 44 | 58 | SFKTQVVAGTNYFVK | | | | | Mascot |
| 1707.7677 | 1707.7482 | -0.0195 | -11 | 13 | 26 | DANEEVQKICDQMK | | | Carbamidomethyl (C)[10] | | Mascot |
| 1974.9436 | 1975.0669 | 0.1233 | 62 | 2 | 20 | PLVCGGTSEAKDANEEV QK | | | | | Mascot |

| 16 | PREDICTED: zinc finger protein 420 isoform 2 [Danio | gi\|326678086 | 70371 | 9.08 | 13 | 36 | 0 |
|---|---|---|---|---|---|---|---|

rerio]

Peptide Information

| Calc. Mass | Obsrv. Mass | ± da | ± ppm | Start Seq. | End Seq. | Sequence | Ion Score | C. I. % | Modification | Rank | Result Type |
|---|---|---|---|---|---|---|---|---|---|---|---|
| 842.5206 | 842.4929 | -0.0277 | -33 | 25 | 31 | QRQGLLK | | | | | Mascot |
| 987.5621 | 987.6115 | 0.0494 | 50 | 497 | 505 | TFITAGHLK | | | | | Mascot |
| 1045.536 | 1045.5433 | 0.0073 | 7 | 134 | 141 | FHCARNLK | | | Carbamidomethyl (C)[3] | | Mascot |
| 1108.5997 | 1108.5502 | -0.0495 | -45 | 357 | 366 | TFITAAELSR | | | | | Mascot |
| 1108.5997 | 1108.5502 | -0.0495 | -45 | 357 | 366 | TFITAAELSR | | | | | Mascot |
| 1190.4856 | 1190.5504 | 0.0648 | 54 | 488 | 496 | EFVCFECEK | | | Carbamidomethyl (C)[4] | | Mascot |
| 1534.757 | 1534.6222 | -0.1348 | -88 | 83 | 95 | IDHNFLMETTAVK | | | Oxidation (M)[7] | | Mascot |
| 1743.833 | 1743.7939 | -0.0391 | -22 | 556 | 571 | VSSSLNSHMLTHTGEK | | | Oxidation (M)[9] | | Mascot |
| 2086.991 | 2086.842 | -0.149 | -71 | 367 | 384 | HQRIHTGEKPYTCTQCG K | | | | | Mascot |
| 2110.0347 | 2110.0537 | 0.019 | 9 | 556 | 574 | VSSSLNSHMLTHTGEKT HK | | | Oxidation (M)[9] | | Mascot |
| 2211.0798 | 2211.053 | -0.0268 | -12 | 450 | 468 | IHLRVHTNEKPYSCPECG K | | | | | Mascot |
| 2216.0515 | 2215.9321 | -0.1194 | -54 | 488 | 505 | EFVCFECEKTFITAGHLK | | | Carbamidomethyl (C)[4,7] | | Mascot |
| 2283.0579 | 2283.125 | 0.0671 | 29 | 227 | 245 | HQMIHTGEKPYKCSHCD LR | | | | | Mascot |
| 2559.1677 | 2559.1748 | 0.0071 | 3 | 83 | 104 | IDHNFLMETTAVKDFTCT QCGK | | | Carbamidomethyl (C)[17] | | Mascot |
| 2559.1677 | 2559.1748 | 0.0071 | 3 | 83 | 104 | IDHNFLMETTAVKDFTCT QCGK | | | Carbamidomethyl (C)[17] | | Mascot |

| | | | | | | | |
|---|---|---|---|---|---|---|---|
| 17 | ATP-binding cassette sub-family D member 3 [Danio rerio] | gi\|47087309 | 74945 | 9.47 | 12 | 34 | 0 |

Peptide Information

| Calc. Mass | Obsrv. Mass | ± da | ± ppm | Start Seq. | End Seq. | Sequence | Ion Score | C. I. % | Modification | Rank | Result Type |
|---|---|---|---|---|---|---|---|---|---|---|---|
| 850.4716 | 850.527 | 0.0554 | 65 | 151 | 156 | LCFRVR | | | Carbamidomethyl (C)[2] | | Mascot |
| 987.5833 | 987.6115 | 0.0282 | 29 | 396 | 404 | VLKELNSGK | | | | | Mascot |
| 1051.6411 | 1051.6887 | 0.0476 | 45 | 61 | 68 | LFFIRISR | | | | | Mascot |
| 1095.5428 | 1095.5037 | -0.0391 | -36 | 399 | 407 | ELNSGKYER | | | | | Mascot |
| 1118.6602 | 1118.634 | -0.0262 | -23 | 390 | 398 | ITELMRVLK | | | Oxidation (M)[5] | | Mascot |
| 1330.6381 | 1330.6335 | -0.0046 | -3 | 310 | 321 | FSMGMVDSIIAK | | | Oxidation (M)[3,5] | | Mascot |
| 1473.6328 | 1473.8102 | 0.1774 | 120 | 629 | 639 | HHEYYLHMDGR | | | Oxidation (M)[8] | | Mascot |
| 1473.6328 | 1473.8102 | 0.1774 | 120 | 629 | 639 | HHEYYLHMDGR | | | Oxidation (M)[8] | | Mascot |
| 1557.8325 | 1557.7168 | -0.1157 | -74 | 298 | 309 | LVDHLHNFIFFR | | | | | Mascot |
| 2110.0168 | 2110.0537 | 0.0369 | 17 | 462 | 481 | SGTNVLVCGPNGCGKSS LFR | | | Carbamidomethyl (C)[8,13] | | Mascot |

| Calc. Mass | Obsrv. Mass | ± da | ± ppm | Start Seq. | End Seq. | Sequence | Ion Score | C. I. % | Modification | Rank | Result Type |
|---|---|---|---|---|---|---|---|---|---|---|---|
| 2211.2283 | 2211.053 | -0.1753 | -79 | 482 | 501 | VLGELWPLFGGNLTKPE RGK | | | | | Mascot |
| 2297.0723 | 2297.1318 | 0.0595 | 26 | 455 | 476 | DLCFEVKSGTNVLVCGP NGCGK | | | Carbamidomethyl (C)[3] | | Mascot |
| 2853.4578 | 2853.3992 | -0.0586 | -21 | 298 | 321 | LVDHLHNFIFFRFSMGMV DSIIAK | | | Oxidation (M)[15] | | Mascot |

18 hypothetical protein LOC100003708 [Danio rerio] gi|324120910 46837.6 7.94 8 34 0

**Peptide Information**

| Calc. Mass | Obsrv. Mass | ± da | ± ppm | Start Seq. | End Seq. | Sequence | Ion Score | C. I. % | Modification | Rank | Result Type |
|---|---|---|---|---|---|---|---|---|---|---|---|
| 1095.5304 | 1095.5037 | -0.0267 | -24 | 352 | 359 | HHTRHHDR | | | | | Mascot |
| 1354.6658 | 1354.6934 | 0.0276 | 20 | 325 | 335 | AIMHSHHGHRR | | | Oxidation (M)[3] | | Mascot |
| 1354.6658 | 1354.6934 | 0.0276 | 20 | 325 | 335 | AIMHSHHGHRR | | | Oxidation (M)[3] | | Mascot |
| 1473.8358 | 1473.8102 | -0.0256 | -17 | 213 | 225 | LGLVMPGFLQRSR | | | | | Mascot |
| 1473.8358 | 1473.8102 | -0.0256 | -17 | 213 | 225 | LGLVMPGFLQRSR | | | | | Mascot |
| 1638.7871 | 1638.6833 | -0.1038 | -63 | 369 | 381 | HHHGHRPHHHGHR | | | | | Mascot |
| 2110.1226 | 2110.0537 | -0.0689 | -33 | 6 | 24 | AFAFLCLVVSVGAQRTQ SR | | | Carbamidomethyl (C)[6] | | Mascot |
| 2233.2261 | 2233.0376 | -0.1885 | -84 | 141 | 160 | SIKGVFEEEMRPLLLSAS VK | | | | | Mascot |
| 2559.3374 | 2559.1748 | -0.1626 | -64 | 93 | 115 | LMSANEASVGSFIQTLLT YLKEK | | | Oxidation (M)[2] | | Mascot |
| 2559.3374 | 2559.1748 | -0.1626 | -64 | 93 | 115 | LMSANEASVGSFIQTLLT YLKEK | | | Oxidation (M)[2] | | Mascot |
| 3366.6531 | 3366.4951 | -0.158 | -47 | 286 | 316 | EVLQQGFGGIGVVLIDLD VFGNSMCPNMTQK | | | Carbamidomethyl (C)[25] | | Mascot |
| 3366.6531 | 3366.4951 | -0.158 | -47 | 286 | 316 | EVLQQGFGGIGVVLIDLD VFGNSMCPNMTQK | | | Carbamidomethyl (C)[25] | | Mascot |

19 Zgc:63504 protein [Danio rerio] gi|39644985 175143.5 5.66 19 34 0

**Peptide Information**

| Calc. Mass | Obsrv. Mass | ± da | ± ppm | Start Seq. | End Seq. | Sequence | Ion Score | C. I. % | Modification | Rank | Result Type |
|---|---|---|---|---|---|---|---|---|---|---|---|
| 842.409 | 842.4929 | 0.0839 | 100 | 991 | 996 | MYHVHR | | | | | Mascot |
| 900.5148 | 900.4632 | -0.0516 | -57 | 1007 | 1015 | IPGAKTADK | | | | | Mascot |
| 987.5833 | 987.6115 | 0.0282 | 29 | 644 | 652 | LQTNISALK | | | | | Mascot |
| 1165.5735 | 1165.7202 | 0.1467 | 126 | 718 | 727 | LFEEATDVNK | | | | | Mascot |
| 1174.6249 | 1174.553 | -0.0719 | -61 | 707 | 717 | ALEGIMEGAKR | | | | | Mascot |
| 1190.5221 | 1190.5504 | 0.0283 | 24 | 1459 | 1468 | EFMGLTDFCK | | | | | Mascot |
| 1557.8132 | 1557.7168 | -0.0964 | -62 | 181 | 193 | NNLANKPQDFQIR | | | | | Mascot |
| 1564.8304 | 1564.8866 | 0.0562 | 36 | 1318 | 1330 | KCPNFQGNVLFLK | | | Carbamidomethyl (C)[2] | | Mascot |
| 1573.8771 | 1573.7124 | -0.1647 | -105 | 728 | 740 | CLTIIEGWLEKLR | | | | | Mascot |
| 1620.8083 | 1620.7225 | -0.0858 | -53 | 1193 | 1206 | DEMLGRSTCVPLVK | | | Carbamidomethyl (C)[9], Oxidation (M)[3] | | Mascot |

| | | | | | | | | | | | |
|---|---|---|---|---|---|---|---|---|---|---|---|
| 1688.7506 | 1688.8339 | 0.0833 | 49 | 1372 | 1386 | CDPYVTVSEVAMSSK | | | Carbamidomethyl (C)[1], Oxidation (M)[12] | | Mascot |
| 1720.9056 | 1720.9137 | 0.0081 | 5 | 384 | 398 | NLVDPFVEAWFAGKK | | | | | Mascot |
| 1743.9275 | 1743.7939 | -0.1336 | -77 | 17 | 32 | LGSPDPIASLVFRDEK | | | | | Mascot |
| 1975.0229 | 1975.0669 | 0.044 | 22 | 527 | 544 | ADKSVEEISSDDILVVQK | | | | | Mascot |
| 2039.9929 | 2039.9558 | -0.0371 | -18 | 273 | 289 | LDIGYIYDEQAHCIMKK | | | | | Mascot |
| 2225.0398 | 2225.0657 | 0.0259 | 12 | 1288 | 1307 | MYQLAPVTSPCLVVECGGER | | | Carbamidomethyl (C)[11], Oxidation (M)[1] | | Mascot |
| 2384.1938 | 2383.8892 | -0.3046 | -128 | 326 | 347 | EQDSDKDDVESNLLLPAGVALR | | | | | Mascot |
| 2680.2407 | 2680.2212 | -0.0195 | -7 | 358 | 382 | AEDIPQMDDSITQSLKEAFGGEGNK | | | | | Mascot |
| 2871.4919 | 2871.394 | -0.0979 | -34 | 1514 | 1538 | ELPDSVSQECVVRIYVVQGIDLQPK | | | Carbamidomethyl (C)[10] | | Mascot |
| 2871.4919 | 2871.394 | -0.0979 | -34 | 1514 | 1538 | ELPDSVSQECVVRIYVVQGIDLQPK | | | Carbamidomethyl (C)[10] | | Mascot |

| | | | | | | | | | | |
|---|---|---|---|---|---|---|---|---|---|---|
| 20 | PRKCA-binding protein [Danio rerio] | gi\|116268065 | 45868.4 | 5.48 | 7 | 33 | 0 | 16 | 0 |

Peptide Information

| Calc. Mass | Obsrv. Mass | ± da | ± ppm | Start Seq. | End Seq. | Sequence | Ion Score | C. I. % | Modification | Rank | Result Type |
|---|---|---|---|---|---|---|---|---|---|---|---|
| 987.504 | 987.6115 | 0.1075 | 109 | 165 | 172 | GLMEHTKR | | | Oxidation (M)[3] | | Mascot |
| 1045.571 | 1045.5433 | -0.0277 | -26 | 142 | 151 | AILCNDGLVK | | | | | Mascot |
| 1127.6783 | 1127.5524 | -0.1259 | -112 | 242 | 251 | AIPDTKLTIR | 16 | 0 | | | Mascot |
| 1127.6783 | 1127.5524 | -0.1259 | -112 | 242 | 251 | AIPDTKLTIR | | | | | Mascot |
| 1473.6564 | 1473.8102 | 0.1538 | 104 | 389 | 401 | AQNQQQEENGAEK | | | | | Mascot |
| 1473.6564 | 1473.8102 | 0.1538 | 104 | 389 | 401 | AQNQQQEENGAEK | | | | | Mascot |
| 1534.6254 | 1534.6222 | -0.0032 | -2 | 2 | 13 | FTDMDYELEEDK | | | | | Mascot |
| 1720.8534 | 1720.9137 | 0.0603 | 35 | 125 | 141 | LVENMSSGTADALGLSR | | | | | Mascot |
| 1768.8978 | 1768.832 | -0.0658 | -37 | 253 | 266 | YLDVKFGYLSYCLK | | | Carbamidomethyl (C)[12] | | Mascot |

| | | | | | | | |
|---|---|---|---|---|---|---|---|
| 21 | PREDICTED: collagen alpha-1(VI) chain [Danio rerio] | gi\|125829720 | 106609.7 | 5.87 | 12 | 33 | 0 |

Peptide Information

| Calc. Mass | Obsrv. Mass | ± da | ± ppm | Start Seq. | End Seq. | Sequence | Ion Score | C. I. % | Modification | Rank | Result Type |
|---|---|---|---|---|---|---|---|---|---|---|---|
| 900.5148 | 900.4632 | -0.0516 | -57 | 132 | 139 | EGIAELLR | | | | | Mascot |
| 939.4564 | 939.5073 | 0.0509 | 54 | 218 | 225 | STQMSTIR | | | Oxidation (M)[4] | | Mascot |
| 1174.6367 | 1174.553 | -0.0837 | -71 | 971 | 979 | HLFKVPDYR | | | | | Mascot |
| 1354.7617 | 1354.6934 | -0.0683 | -50 | 61 | 71 | TFTKLFIDELK | | | | | Mascot |
| 1354.7617 | 1354.6934 | -0.0683 | -50 | 61 | 71 | TFTKLFIDELK | | | | | Mascot |
| 1380.7555 | 1380.7235 | -0.032 | -23 | 621 | 631 | EFIVTIMDRLK | | | Oxidation (M)[7] | | Mascot |
| 2021.9821 | 2021.9862 | 0.0041 | 2 | 164 | 182 | EPCGGIQEAANEARQHAIK | | | | | Mascot |

| | | | | | | | | | | | |
|---|---|---|---|---|---|---|---|---|---|---|---|
| 2087.092 | 2086.842 | -0.25 | -120 | 975 | 992 | VPDYRSLVTGVFYQTVSR | | | | | Mascot |
| 2297.1785 | 2297.1318 | -0.0467 | -20 | 881 | 900 | FQNAGTQVTNALNFAIERFR | | | | | Mascot |
| 2663.2153 | 2663.3643 | 0.149 | 56 | 266 | 292 | GDFGAQGNPGDPGPVGYQGMKGDQGIK | | | Oxidation (M)[20] | | Mascot |
| 2663.2153 | 2663.3643 | 0.149 | 56 | 266 | 292 | GDFGAQGNPGDPGPVGYQGMKGDQGIK | | | Oxidation (M)[20] | | Mascot |
| 2697.3892 | 2697.1252 | -0.264 | -98 | 1 | 25 | MALNGYILTALCALWAGIFAQDVSR | | | | | Mascot |
| 2697.3892 | 2697.1252 | -0.264 | -98 | 1 | 25 | MALNGYILTALCALWAGIFAQDVSR | | | | | Mascot |
| 2871.2681 | 2871.394 | 0.1259 | 44 | 226 | 253 | SIIHMICCSFDCIADGGPQGPPGDPGQR | | | | | Mascot |
| 2871.2681 | 2871.394 | 0.1259 | 44 | 226 | 253 | SIIHMICCSFDCIADGGPQGPPGDPGQR | | | | | Mascot |
| 2928.2896 | 2928.3816 | 0.092 | 31 | 226 | 253 | SIIHMICCSFDCIADGGPQGPPGDPGQR | | | Carbamidomethyl (C)[7] | | Mascot |
| 2928.2896 | 2928.3816 | 0.092 | 31 | 226 | 253 | SIIHMICCSFDCIADGGPQGPPGDPGQR | | | Carbamidomethyl (C)[7] | | Mascot |
| 3338.5046 | 3338.6826 | 0.178 | 53 | 793 | 822 | KCPTYTCPISFNDNTDVLIMMDSSASVGAK | | | Carbamidomethyl (C)[2,7], Oxidation (M)[20] | | Mascot |

| | | | | | | | |
|---|---|---|---|---|---|---|---|
| 22 | PREDICTED: hypothetical protein LOC100320667 [Danio rerio] | gi\|326678190 | 55450.3 | 5.16 | 9 | 33 | 0 |

**Peptide Information**

| Calc. Mass | Obsrv. Mass | ± da | ± ppm | Start Seq. | End Seq. | Sequence | Ion Score | C. I. % | Modification | Rank | Result Type |
|---|---|---|---|---|---|---|---|---|---|---|---|
| 1534.8071 | 1534.6222 | -0.1849 | -120 | 304 | 317 | LDNKTGSLTITDTR | | | | | Mascot |
| 1548.8115 | 1548.9131 | 0.1016 | 66 | 145 | 159 | VLEENSVTLSSGVSK | | | | | Mascot |
| 1620.7963 | 1620.7225 | -0.0738 | -46 | 247 | 261 | EGDSVTLNSDLTELK | | | | | Mascot |
| 1768.8613 | 1768.832 | -0.0293 | -17 | 183 | 197 | QTNRFSVFDDVLDGR | | | | | Mascot |
| 2040.0496 | 2039.9558 | -0.0938 | -46 | 207 | 225 | KTGSLTITDIITENAGDYK | | | | | Mascot |
| 2225.063 | 2225.0657 | 0.0027 | 1 | 2 | 18 | FRHFQSCHLELFSELMK | | | Carbamidomethyl (C)[7], Oxidation (M)[16] | | Mascot |
| 2283.0872 | 2283.125 | 0.0378 | 17 | 1 | 18 | MFRHFQSCHLELFSELMK | | | | | Mascot |
| 2680.3577 | 2680.2212 | -0.1365 | -51 | 74 | 97 | ETVIAEINVTANRFSVFDDVLDGR | | | | | Mascot |
| 2697.3374 | 2697.1252 | -0.2122 | -79 | 24 | 46 | LVLLCLCRLVGGFDDDYQVQEGK | | | Carbamidomethyl (C)[5,7] | | Mascot |
| 2697.3374 | 2697.1252 | -0.2122 | -79 | 24 | 46 | LVLLCLCRLVGGFDDDYQVQEGK | | | Carbamidomethyl (C)[5,7] | | Mascot |

| | | | | | | | |
|---|---|---|---|---|---|---|---|
| 23 | novel protein [Danio rerio] | gi\|220679541 | 12846.6 | 6.84 | 5 | 33 | 0 |

**Peptide Information**

| Calc. Mass | Obsrv. Mass | ± da | ± ppm | Start Seq. | End Seq. | Sequence | Ion Score | C. I. % | Modification | Rank | Result Type |
|---|---|---|---|---|---|---|---|---|---|---|---|
| 850.4338 | 850.527 | 0.0932 | 110 | 73 | 79 | TIDVMQK | | | Oxidation (M)[5] | | Mascot |
| 1688.8602 | 1688.8339 | -0.0263 | -16 | 101 | 116 | TGLGSHVTDGFSIREL | | | | | Mascot |

| | | | | | | | | | | | |
|---|---|---|---|---|---|---|---|---|---|---|---|
| 1768.8357 | 1768.832 | -0.0037 | -2 | 82 | 96 | LMHSLSLSYMADTQR | | | Oxidation (M)[2] | | Mascot |
| 2022.026 | 2021.9862 | -0.0398 | -20 | 80 | 96 | IRLMHSLSLSYMADTQR | | | | | Mascot |
| 2871.4961 | 2871.394 | -0.1021 | -36 | 38 | 62 | ELKVPEFSALQSTFLEEA NPMIHLK | | | | | Mascot |
| 2871.4961 | 2871.394 | -0.1021 | -36 | 38 | 62 | ELKVPEFSALQSTFLEEA NPMIHLK | | | | | Mascot |

24 LSM14 homolog B-like [Danio rerio] gi|239050742 41515 9.51 8 33 0

Peptide Information

| Calc. Mass | Obsrv. Mass | ± da | ± ppm | Start Seq. | End Seq. | Sequence | Ion Score | C. I. % | Modification | Rank | Result Type |
|---|---|---|---|---|---|---|---|---|---|---|---|
| 939.5158 | 939.5073 | -0.0085 | -9 | 349 | 356 | YLRAGGFR | | | | | Mascot |
| 996.4996 | 996.5761 | 0.0765 | 77 | 2 | 11 | SSGTPYIGSK | | | | | Mascot |
| 1127.5402 | 1127.5524 | 0.0122 | 11 | 1 | 11 | MSSGTPYIGSK | | | | | Mascot |
| 1127.5402 | 1127.5524 | 0.0122 | 11 | 1 | 11 | MSSGTPYIGSK | | | | | Mascot |
| 1190.6012 | 1190.5504 | -0.0508 | -43 | 192 | 203 | GDSSSAQQAVLK | | | | | Mascot |
| 1557.7656 | 1557.7168 | -0.0488 | -31 | 316 | 328 | SFFDNISSELRSR | | | | | Mascot |
| 1620.8856 | 1620.7225 | -0.1631 | -101 | 338 | 351 | LNVETFGVPGRYLR | | | | | Mascot |
| 1768.9513 | 1768.832 | -0.1193 | -67 | 1 | 17 | MSSGTPYIGSKISLISK | | | | | Mascot |
| 2087.0542 | 2086.842 | -0.2122 | -102 | 22 | 40 | YEGILYTIDTDNSTVALAK | | | | | Mascot |

25 F-actin-capping protein subunit beta [Danio rerio] gi|41053959 30723.6 5.7 7 33 0

Peptide Information

| Calc. Mass | Obsrv. Mass | ± da | ± ppm | Start Seq. | End Seq. | Sequence | Ion Score | C. I. % | Modification | Rank | Result Type |
|---|---|---|---|---|---|---|---|---|---|---|---|
| 1108.6473 | 1108.5502 | -0.0971 | -88 | 15 | 23 | RLPPQQIEK | | | | | Mascot |
| 1108.6473 | 1108.5502 | -0.0971 | -88 | 15 | 23 | RLPPQQIEK | | | | | Mascot |
| 1174.5198 | 1174.553 | 0.0332 | 28 | 58 | 66 | DYLLCDYNR | | | | | Mascot |
| 1534.6842 | 1534.6222 | -0.062 | -40 | 79 | 92 | YEPPIDDGAMPSAR | | | Oxidation (M)[10] | | Mascot |
| 1548.7145 | 1548.9131 | 0.1986 | 128 | 2 | 14 | NEQQLDCALDLMR | | | | | Mascot |
| 1557.7729 | 1557.7168 | -0.0561 | -36 | 54 | 66 | VVGKDYLLCDYNR | | | | | Mascot |
| 1564.7094 | 1564.8866 | 0.1772 | 113 | 2 | 14 | NEQQLDCALDLMR | | | Oxidation (M)[12] | | Mascot |
| 1605.736 | 1605.7881 | 0.0521 | 32 | 2 | 14 | NEQQLDCALDLMR | | | Carbamidomethyl (C)[7] | | Mascot |
| 1720.8105 | 1720.9137 | 0.1032 | 60 | 2 | 15 | NEQQLDCALDLMRR | | | Oxidation (M)[12] | | Mascot |
| 1768.7662 | 1768.832 | 0.0658 | 37 | 1 | 14 | MNEQQLDCALDLMR | | | Carbamidomethyl (C)[8], Oxidation (M)[1,13] | | Mascot |

26 Zgc:171493 protein [Danio rerio] gi|158253797 23832.2 8.19 7 32 0

Peptide Information

| Calc. Mass | Obsrv. Mass | ± da | ± ppm | Start Seq. | End Seq. | Sequence | Ion Score | C. I. % | Modification | Rank | Result Type |
|---|---|---|---|---|---|---|---|---|---|---|---|

| Calc. Mass | Obsrv. Mass | ± da | ± ppm | Start Seq. | End Seq. | Sequence | Ion Score | C. I. % | Modification | Rank | Result Type |
|---|---|---|---|---|---|---|---|---|---|---|---|
| 900.5009 | 900.4632 | -0.0377 | -42 | 107 | 113 | LQERQAR | | | | | Mascot |
| 987.5469 | 987.6115 | 0.0646 | 65 | 2 | 10 | IPSSDSLLR | | | | | Mascot |
| 1118.5874 | 1118.634 | 0.0466 | 42 | 1 | 10 | MIPSSDSLLR | | | | | Mascot |
| 1473.7406 | 1473.8102 | 0.0696 | 47 | 197 | 209 | YSLVFGNCTIKTQ | | | | | Mascot |
| 1473.7406 | 1473.8102 | 0.0696 | 47 | 197 | 209 | YSLVFGNCTIKTQ | | | | | Mascot |
| 1557.7288 | 1557.7168 | -0.012 | -8 | 184 | 196 | SDFENKVMCDILK | | | Oxidation (M)[8] | | Mascot |
| 1564.8694 | 1564.8866 | 0.0172 | 11 | 94 | 106 | VDVIHLQEQLDKK | | | | | Mascot |
| 2283.1147 | 2283.125 | 0.0103 | 5 | 156 | 175 | MTIAAYQTLYESSVAFGM RK | | | Oxidation (M)[1] | | Mascot |

27 Zgc:110331 [Danio rerio] gi|62202266 17382.1 8.3 6 32 0

**Peptide Information**

| Calc. Mass | Obsrv. Mass | ± da | ± ppm | Start Seq. | End Seq. | Sequence | Ion Score | C. I. % | Modification | Rank | Result Type |
|---|---|---|---|---|---|---|---|---|---|---|---|
| 882.5294 | 882.5476 | 0.0182 | 21 | 122 | 129 | ELSLPPVK | | | | | Mascot |
| 900.4971 | 900.4632 | -0.0339 | -38 | 9 | 16 | CAAPQILK | | | Carbamidomethyl (C)[1] | | Mascot |
| 1045.5636 | 1045.5433 | -0.0203 | -19 | 42 | 51 | NVGSLDKNAK | | | | | Mascot |
| 1330.6494 | 1330.6335 | -0.0159 | -12 | 130 | 141 | LHCSMLAEDAIK | | | | | Mascot |
| 1974.9735 | 1975.0669 | 0.0934 | 47 | 49 | 68 | NAKNVGTGLVGAPACGD VMK | | | Carbamidomethyl (C)[15], Oxidation (M)[19] | | Mascot |
| 2194.1611 | 2194.0354 | -0.1257 | -57 | 122 | 141 | ELSLPPVKLHCSMLAEDA IK | | | | | Mascot |

28 novel protein similar to vertebrate (PIWIL2) [Danio rerio] gi|123232882 22624.7 6.37 6 32 0

**Peptide Information**

| Calc. Mass | Obsrv. Mass | ± da | ± ppm | Start Seq. | End Seq. | Sequence | Ion Score | C. I. % | Modification | Rank | Result Type |
|---|---|---|---|---|---|---|---|---|---|---|---|
| 1108.5092 | 1108.5502 | 0.041 | 37 | 88 | 97 | LPMEEGGFGR | | | Oxidation (M)[3] | | Mascot |
| 1108.5092 | 1108.5502 | 0.041 | 37 | 88 | 97 | LPMEEGGFGR | | | Oxidation (M)[3] | | Mascot |
| 1330.7477 | 1330.6335 | -0.1142 | -86 | 176 | 188 | GAAPVGVTFKDLR | | | | | Mascot |
| 1620.889 | 1620.7225 | -0.1665 | -103 | 40 | 54 | GLIMPIDEPLPGRGR | | | | | Mascot |
| 1743.8119 | 1743.7939 | -0.018 | -10 | 199 | 214 | DGVQQAIWGQICSQNP | | | | | Mascot |
| 2233.1646 | 2233.0376 | -0.127 | -57 | 149 | 171 | VGSPGTGSSLVSMFRGL GIEPGK | | | | | Mascot |
| 2384.1477 | 2383.8892 | -0.2585 | -108 | 17 | 37 | APWQQSTEDQSQLLDQP SLGR | | | | | Mascot |

29 novel protein similar to H.sapien ARHGAP23, Rho GTPase activating protein 23 (ARHGAP23) [Danio reri gi|220679183 43224.2 9.03 10 32 0

**Peptide Information**

| Calc. Mass | Obsrv. Mass | ± da | ± ppm | Start | End | Sequence | Ion | C. I. % | Modification | Rank | Result Type |
|---|---|---|---|---|---|---|---|---|---|---|---|

| | | | | Seq. | Seq. | | Score | | | | |
|---|---|---|---|---|---|---|---|---|---|---|---|
| 939.5509 | 939.5073 | -0.0436 | -46 | 369 | 376 | ITETLILH | | | | | Mascot |
| 1095.4962 | 1095.5037 | 0.0075 | 7 | 89 | 96 | DDMLMWIR | | | Oxidation (M)[3] | | Mascot |
| 1127.5116 | 1127.5524 | 0.0408 | 36 | 282 | 290 | YNDFIDANR | | | | | Mascot |
| 1127.5116 | 1127.5524 | 0.0408 | 36 | 282 | 290 | YNDFIDANR | | | | | Mascot |
| 1174.599 | 1174.553 | -0.046 | -39 | 272 | 281 | LPEPLFTDDK | | | | | Mascot |
| 1638.9214 | 1638.6833 | -0.2381 | -145 | 257 | 270 | DLNVISSLLKSFFR | | | | | Mascot |
| 1652.941 | 1652.8976 | -0.0434 | -26 | 12 | 25 | VFSVLHSSLLFLYK | | | | | Mascot |
| 1720.8687 | 1720.9137 | 0.045 | 26 | 187 | 202 | TFGVRLEDCLPAASNK | | | | | Mascot |
| 1974.8936 | 1975.0669 | 0.1733 | 88 | 73 | 88 | LMTQDFCEYLLQAEDR | | | | | Mascot |
| 2211.1213 | 2211.053 | -0.0683 | -31 | 47 | 66 | DIQTISIQGCLIDIAYSETK | | | | | Mascot |
| 2283.0928 | 2283.125 | 0.0322 | 14 | 272 | 290 | LPEPLFTDDKYNDFIDAN R | | | | | Mascot |

30 TPA_exp: transferrin [Danio rerio] gi|47264590 73483.9 6.81 11 32 0

**Peptide Information**

| Calc. Mass | Obsrv. Mass | ± da | ± ppm | Start Seq. | End Seq. | Sequence | Ion Score | C. I. % | Modification | Rank | Result Type |
|---|---|---|---|---|---|---|---|---|---|---|---|
| 939.4604 | 939.5073 | 0.0469 | 50 | 646 | 652 | YIDMIER | | | | | Mascot |
| 1108.6361 | 1108.5502 | -0.0859 | -77 | 306 | 314 | RTDSLLYLK | | | | | Mascot |
| 1108.6361 | 1108.5502 | -0.0859 | -77 | 306 | 314 | RTDSLLYLK | | | | | Mascot |
| 1127.4357 | 1127.5524 | 0.1167 | 104 | 121 | 129 | SSCHSCYQR | 8 | 0 | Carbamidomethyl (C)[6] | | Mascot |
| 1127.4357 | 1127.5524 | 0.1167 | 104 | 121 | 129 | SSCHSCYQR | | | Carbamidomethyl (C)[3] | | Mascot |
| 1363.5988 | 1363.6482 | 0.0494 | 36 | 196 | 207 | YFGDDGAFQCLK | | | | | Mascot |
| 1380.7369 | 1380.7235 | -0.0134 | -10 | 597 | 609 | SAVVSFLSDIQSK | | | | | Mascot |
| 1557.7366 | 1557.7168 | -0.0198 | -13 | 146 | 158 | ITWEGPNEMPVER | | | | | Mascot |
| 1573.7314 | 1573.7124 | -0.019 | -12 | 146 | 158 | ITWEGPNEMPVER | | | Oxidation (M)[9] | | Mascot |
| 1638.7349 | 1638.6833 | -0.0516 | -31 | 360 | 373 | SCILEASVDDCIEK | | | Carbamidomethyl (C)[2,11] | | Mascot |
| 1652.8279 | 1652.8976 | 0.0697 | 42 | 274 | 289 | QIPASDLFSSQAFGGK | | | | | Mascot |
| 2297.0676 | 2297.1318 | 0.0642 | 28 | 553 | 572 | DLKSEDFELICPNTPDTT MK | | | | | Mascot |
| 2536.061 | 2536.1211 | 0.0601 | 24 | 467 | 489 | CTLDKFFSEGCAPGADP TSNMCK | | | Carbamidomethyl (C)[1,11] | | Mascot |
| 3338.5085 | 3338.6826 | 0.1741 | 52 | 395 | 427 | CGLVPVMAEQSNSQSCS SGSGGTASYYAVAVVR | | | Carbamidomethyl (C)[1], Oxidation (M)[7] | | Mascot |

31 stromal interaction molecule 1 [Danio rerio] gi|113677986 76089.1 5.98 10 32 0

**Peptide Information**

| Calc. Mass | Obsrv. Mass | ± da | ± ppm | Start Seq. | End Seq. | Sequence | Ion Score | C. I. % | Modification | Rank | Result Type |
|---|---|---|---|---|---|---|---|---|---|---|---|

| Calc. Mass | Obsrv. Mass | ± da | ± ppm | Start Seq. | End Seq. | Sequence | Ion Score | C. I. % | Modification | Rank | Result Type |
|---|---|---|---|---|---|---|---|---|---|---|---|
| 850.5032 | 850.527 | 0.0238 | 28 | 583 | 589 | KLYGIEK | | | | | Mascot |
| 882.425 | 882.5476 | 0.1226 | 139 | 230 | 236 | HSKDHMK | | | | | Mascot |
| 900.5513 | 900.4632 | -0.0881 | -98 | 636 | 643 | IPQISSKK | | | | | Mascot |
| 1095.627 | 1095.5037 | -0.1233 | -113 | 175 | 183 | ILDRSHVQK | | | | | Mascot |
| 1652.8285 | 1652.8976 | 0.0691 | 42 | 492 | 505 | QRHIDPQMALGSQR | | | Oxidation (M)[8] | | Mascot |
| 2233.0806 | 2233.0376 | -0.043 | -19 | 45 | 63 | IDEPLCQDENAILSFEAIR | | | Carbamidomethyl (C)[6] | | Mascot |
| 2283.967 | 2284.1194 | 0.1524 | 67 | 68 | 87 | QMDDDDNGNVDVLETD GFLR | | | Oxidation (M)[2] | | Mascot |
| 2559.1687 | 2559.1748 | 0.0061 | 2 | 99 | 119 | HNTFHGDDQFISVEDLW NAWK | | | | | Mascot |
| 2559.1687 | 2559.1748 | 0.0061 | 2 | 99 | 119 | HNTFHGDDQFISVEDLW NAWK | | | | | Mascot |
| 2663.3181 | 2663.3643 | 0.0462 | 17 | 2 | 24 | EFSGLVTFWIACICFLLQC RADK | | | | | Mascot |
| 2663.3181 | 2663.3643 | 0.0462 | 17 | 2 | 24 | EFSGLVTFWIACICFLLQC RADK | | | | | Mascot |
| 3337.6431 | 3337.6738 | 0.0307 | 9 | 202 | 229 | HNHLKDFMLVVSIVIGMG GCWFAYIQNR | | | Carbamidomethyl (C)[20], Oxidation (M)[8,17] | | Mascot |

32 PREDICTED: phospholipase DDHD1-like [Danio rerio] gi|326675812 90094.3 5.88 11 31 0

**Peptide Information**

| Calc. Mass | Obsrv. Mass | ± da | ± ppm | Start Seq. | End Seq. | Sequence | Ion Score | C. I. % | Modification | Rank | Result Type |
|---|---|---|---|---|---|---|---|---|---|---|---|
| 987.5833 | 987.6115 | 0.0282 | 29 | 673 | 683 | ASILGAASLGK | | | | | Mascot |
| 1045.5135 | 1045.5433 | 0.0298 | 29 | 446 | 453 | LYSLFCSR | | | Carbamidomethyl (C)[6] | | Mascot |
| 1108.6361 | 1108.5502 | -0.0859 | -77 | 303 | 312 | TVTQKLGFSK | | | | | Mascot |
| 1108.6361 | 1108.5502 | -0.0859 | -77 | 303 | 312 | TVTQKLGFSK | | | | | Mascot |
| 1573.7955 | 1573.7124 | -0.0831 | -53 | 394 | 408 | LALDGDTVESITPDK | | | | | Mascot |
| 2021.9081 | 2021.9862 | 0.0781 | 39 | 641 | 660 | DTASDMENGSSPSTSPV LAR | | | | | Mascot |
| 2087.0273 | 2086.842 | -0.1853 | -89 | 548 | 565 | VENFFCMGCPLAVFLALR | | | Carbamidomethyl (C)[6] | | Mascot |
| 2194.0042 | 2194.0354 | 0.0312 | 14 | 641 | 661 | DTASDMENGSSPSTSPV LARR | | | Oxidation (M)[6] | | Mascot |
| 2211.0474 | 2211.053 | 0.0056 | 3 | 203 | 219 | ECYPVYWNQQERIPVMR | | | | | Mascot |
| 2233.1077 | 2233.0376 | -0.0701 | -31 | 566 | 585 | GIRPGNTGHQDHIMPKSI CR | | | Oxidation (M)[14] | | Mascot |
| 2284.0637 | 2284.1194 | 0.0557 | 24 | 203 | 219 | ECYPVYWNQQERIPVMR | | | Carbamidomethyl (C)[2], Oxidation (M)[16] | | Mascot |
| 2606.1729 | 2606.1165 | -0.0564 | -22 | 18 | 39 | LSFGNRDWNDAFFSLCD ESIGR | | | Carbamidomethyl (C)[16] | | Mascot |
| 2871.3428 | 2871.394 | 0.0512 | 18 | 414 | 437 | DLLNSSAMDIMYYTSPLY RDEITK | | | Oxidation (M)[8,11] | | Mascot |
| 2871.3428 | 2871.394 | 0.0512 | 18 | 414 | 437 | DLLNSSAMDIMYYTSPLY RDEITK | | | Oxidation (M)[8,11] | | Mascot |

33 nitrogen fixation cluster-like [Danio rerio] gi|221307563 17420.2 8.83 6 31 0

**Peptide Information**

| Calc. Mass | Obsrv. Mass | ± da | ± ppm | Start Seq. | End Seq. | Sequence | Ion Score | C. I. % | Modification | Rank | Result Type |
|---|---|---|---|---|---|---|---|---|---|---|---|
| 882.5294 | 882.5476 | 0.0182 | 21 | 122 | 129 | ELSLPPVK | | | | | Mascot |
| 900.4971 | 900.4632 | -0.0339 | -38 | 9 | 16 | CAAPQILK | | | Carbamidomethyl (C)[1] | | Mascot |
| 1045.5636 | 1045.5433 | -0.0203 | -19 | 42 | 51 | NVGSLDKNAK | | | | | Mascot |
| 1330.6494 | 1330.6335 | -0.0159 | -12 | 130 | 141 | LHCSMLAEDAIK | | | | | Mascot |
| 1974.9735 | 1975.0669 | 0.0934 | 47 | 49 | 68 | NAKNVGTGLVGAPACGD VMK | | | Carbamidomethyl (C)[15], Oxidation (M)[19] | | Mascot |
| 2194.1611 | 2194.0354 | -0.1257 | -57 | 122 | 141 | ELSLPPVKLHCSMLAEDA IK | | | | | Mascot |

| | | | | | | | | | |
|---|---|---|---|---|---|---|---|---|---|
| 34 | novel protein similar to vertebrate golgi associated, gamma adaptin ear containing, ARF binding pro | gi\|169158149 | 76674.1 | 5.35 | 5 | 31 | 0 | 21 | 0 |

**Peptide Information**

| Calc. Mass | Obsrv. Mass | ± da | ± ppm | Start Seq. | End Seq. | Sequence | Ion Score | C. I. % | Modification | Rank | Result Type |
|---|---|---|---|---|---|---|---|---|---|---|---|
| 842.5458 | 842.4929 | -0.0529 | -63 | 177 | 183 | RLAELLK | | | | | Mascot |
| 1127.6055 | 1127.5524 | -0.0531 | -47 | 41 | 50 | ELEGPQISVR | 21 | 0 | | | Mascot |
| 1127.6055 | 1127.5524 | -0.0531 | -47 | 41 | 50 | ELEGPQISVR | | | | | Mascot |
| 2384.2126 | 2383.8892 | -0.3234 | -136 | 296 | 318 | IVEGQTIVIDVPASTPSGC INDR | | | | | Mascot |
| 2559.1692 | 2559.1748 | 0.0056 | 2 | 265 | 288 | LATETEDDDSALGDILHA SDDLSR | | | | | Mascot |
| 2559.2144 | 2559.1748 | -0.0396 | -15 | 2 | 24 | IMADPEGESLESWLNKAT NPSNR | | | | | Mascot |

| | | | | | | | |
|---|---|---|---|---|---|---|---|
| 35 | PREDICTED: hypothetical protein LOC100534893 [Danio rerio] | gi\|326669436 | 168844.3 | 8.85 | 16 | 31 | 0 |

**Peptide Information**

| Calc. Mass | Obsrv. Mass | ± da | ± ppm | Start Seq. | End Seq. | Sequence | Ion Score | C. I. % | Modification | Rank | Result Type |
|---|---|---|---|---|---|---|---|---|---|---|---|
| 842.4227 | 842.4929 | 0.0702 | 83 | 299 | 305 | ENRPNGR | | | | | Mascot |
| 1045.5411 | 1045.5433 | 0.0022 | 2 | 1154 | 1162 | DEVEIAEIK | | | | | Mascot |
| 1118.5688 | 1118.634 | 0.0652 | 58 | 929 | 940 | LTAGDPSAASTK | | | | | Mascot |
| 1330.7438 | 1330.6335 | -0.1103 | -83 | 360 | 372 | AQGAQIASTIKSR | | | | | Mascot |
| 1354.6208 | 1354.6934 | 0.0726 | 54 | 1430 | 1441 | GYQGFPCEANLR | | | | | Mascot |
| 1354.7325 | 1354.6934 | -0.0391 | -29 | 1466 | 1477 | TPETLQTPPRSK | | | | | Mascot |
| 1473.7908 | 1473.8102 | 0.0194 | 13 | 573 | 586 | NAQDAKIASVIESK | | | | | Mascot |
| 1473.7908 | 1473.8102 | 0.0194 | 13 | 573 | 586 | NAQDAKIASVIESK | | | | | Mascot |
| 1557.7444 | 1557.7168 | -0.0276 | -18 | 1234 | 1248 | GLSDAGGWGSKFYGR | | | | | Mascot |
| 1573.8367 | 1573.7124 | -0.1243 | -79 | 1163 | 1177 | RSAGDEAQVCLAIIK | | | | | Mascot |
| 1605.7153 | 1605.7881 | 0.0728 | 45 | 825 | 837 | HADNKHVDFDDHR | | | | | Mascot |

| Calc. Mass | Obsrv. Mass | ± da | ± ppm | Start Seq. | End Seq. | Sequence | Ion Score | C. I. % | Modification | Rank | Result Type |
|---|---|---|---|---|---|---|---|---|---|---|---|
| 1688.845 | 1688.8339 | -0.0111 | -7 | 467 | 481 | TDIKQTENPSVNTNK | | | | | Mascot |
| 1720.8357 | 1720.9137 | 0.078 | 45 | 452 | 466 | VQDVPMTSSRNIMDK | | | | | Mascot |
| 2110.0344 | 2110.0537 | 0.0193 | 9 | 846 | 865 | VKILPHSCGGNDVNVDT GER | | | | | Mascot |
| 2536.1653 | 2536.1211 | -0.0442 | -17 | 523 | 545 | CASEELEISQSAKITSAM EHGNK | | | Carbamidomethyl (C)[1], Oxidation (M)[18] | | Mascot |
| 2871.3232 | 2871.394 | 0.0708 | 25 | 307 | 331 | EEMVIESGTSVKETEVQ MVSETEQR | | | Oxidation (M)[3] | | Mascot |
| 2871.3232 | 2871.394 | 0.0708 | 25 | 307 | 331 | EEMVIESGTSVKETEVQ MVSETEQR | | | Oxidation (M)[3] | | Mascot |
| 2928.4646 | 2928.3816 | -0.083 | -28 | 1442 | 1465 | VFEQFQIQHQCNYFCGLL NLRSLK | | | | | Mascot |
| 2928.4646 | 2928.3816 | -0.083 | -28 | 1442 | 1465 | VFEQFQIQHQCNYFCGLL NLRSLK | | | | | Mascot |

36 PREDICTED: si:dkey-170m15.2 [Danio rerio] gi|292624792 132327.3 7.37 14 31 0

**Peptide Information**

| Calc. Mass | Obsrv. Mass | ± da | ± ppm | Start Seq. | End Seq. | Sequence | Ion Score | C. I. % | Modification | Rank | Result Type |
|---|---|---|---|---|---|---|---|---|---|---|---|
| 939.4968 | 939.5073 | 0.0105 | 11 | 75 | 83 | DCPLVAPPK | | | | | Mascot |
| 996.5182 | 996.5761 | 0.0579 | 58 | 75 | 83 | DCPLVAPPK | | | Carbamidomethyl (C)[2] | | Mascot |
| 1045.516 | 1045.5433 | 0.0273 | 26 | 98 | 106 | QTNITDEPK | | | | | Mascot |
| 1095.5503 | 1095.5037 | -0.0466 | -43 | 1127 | 1135 | EAFQSCILK | | | Carbamidomethyl (C)[6] | | Mascot |
| 1165.6034 | 1165.7202 | 0.1168 | 100 | 440 | 449 | FCEQAVTIVR | | | | | Mascot |
| 1548.7838 | 1548.9131 | 0.1293 | 83 | 1127 | 1139 | EAFQSCILKEQPR | | | | | Mascot |
| 1605.8053 | 1605.7881 | -0.0172 | -11 | 1127 | 1139 | EAFQSCILKEQPR | | | Carbamidomethyl (C)[6] | | Mascot |
| 1638.7507 | 1638.6833 | -0.0674 | -41 | 847 | 860 | EGNDQHPTDTLFHK | | | | | Mascot |
| 1707.8846 | 1707.7482 | -0.1364 | -80 | 436 | 449 | ESLRFCEQAVTIVR | | | Carbamidomethyl (C)[6] | | Mascot |
| 1974.9919 | 1975.0669 | 0.075 | 38 | 700 | 719 | GGYTFVAEAHTGDVPVIG GK | | | | | Mascot |
| 2039.9148 | 2039.9558 | 0.041 | 20 | 1142 | 1158 | MEEKPMETVEQVETESK | | | Oxidation (M)[1] | | Mascot |
| 2264.208 | 2263.9163 | -0.2917 | -129 | 399 | 418 | HVLQVHTNASLAVHLHLC SK | | | Carbamidomethyl (C)[18] | | Mascot |
| 2283.1875 | 2283.125 | -0.0625 | -27 | 663 | 682 | MFSPIPVCILHVINNDTLE K | | | | | Mascot |
| 2536.1013 | 2536.1211 | 0.0198 | 8 | 605 | 626 | HMVTNGEGIAELYPCRE DECNR | | | | | Mascot |
| 2680.2454 | 2680.2212 | -0.0242 | -9 | 891 | 914 | NEMRVFGDILDGTSMPV GTDQQNR | | | | | Mascot |
| 3275.7019 | 3275.4758 | -0.2261 | -69 | 274 | 305 | TSHFTNSISSRLSAAALS YTVASTHSVAALPK | | | | | Mascot |

37 Ppp4r2a protein [Danio rerio] gi|156230689 49479.9 4.37 8 30 0

**Peptide Information**

| Calc. Mass | Obsrv. Mass | ± da | ± ppm | Start Seq. | End Seq. | Sequence | Ion Score | C. I. % | Modification | Rank | Result Type |
|---|---|---|---|---|---|---|---|---|---|---|---|

| | | | | | | | | | | | |
|---|---|---|---|---|---|---|---|---|---|---|---|
| 850.4199 | 850.527 | 0.1071 | 126 | 172 | 179 | KGSSCVNR | | | | | Mascot |
| 1045.5598 | 1045.5433 | -0.0165 | -16 | 134 | 142 | LCELLTEPK | | | | | Mascot |
| 1174.6506 | 1174.553 | -0.0976 | -83 | 77 | 85 | SYFLFKLEK | | | | | Mascot |
| 1707.837 | 1707.7482 | -0.0888 | -52 | 27 | 41 | HTSTMEIDTLLGAFR | | | Oxidation (M)[5] | | Mascot |
| 1743.8734 | 1743.7939 | -0.0795 | -46 | 50 | 64 | ESCPILDQFLSHVAK | | | Carbamidomethyl (C)[3] | | Mascot |
| 2211.075 | 2211.053 | -0.022 | -10 | 27 | 45 | HTSTMEIDTLLGAFRDFE K | | | | | Mascot |
| 2606.2014 | 2606.1165 | -0.0849 | -33 | 92 | 114 | ASCPEQRGPANPNVEYI PFEEMK | | | | | Mascot |
| 2663.2229 | 2663.3643 | 0.1414 | 53 | 92 | 114 | ASCPEQRGPANPNVEYI PFEEMK | | | Carbamidomethyl (C)[3] | | Mascot |
| 2663.2229 | 2663.3643 | 0.1414 | 53 | 92 | 114 | ASCPEQRGPANPNVEYI PFEEMK | | | Carbamidomethyl (C)[3] | | Mascot |
| 3338.6633 | 3338.6826 | 0.0193 | 6 | 180 | 210 | MNGVMFPGNTSAFPDRN VNGPGTPRPLNRPK | | | | | Mascot |

| | | | | | | | | |
|---|---|---|---|---|---|---|---|---|
| 38 | PREDICTED: interferon-induced very large GTPase 1-like [Danio rerio] | gi\|326665761 | 191677.2 | 6.42 | 17 | 30 | 0 | |

**Peptide Information**

| Calc. Mass | Obsrv. Mass | ± da | ± ppm | Start Seq. | End Seq. | Sequence | Ion Score | C. I. % | Modification | Rank | Result Type |
|---|---|---|---|---|---|---|---|---|---|---|---|
| 842.473 | 842.4929 | 0.0199 | 24 | 920 | 927 | SIISHASK | | | | | Mascot |
| 1045.5887 | 1045.5433 | -0.0454 | -43 | 613 | 621 | QAELEKISK | | | | | Mascot |
| 1051.5419 | 1051.6887 | 0.1468 | 140 | 1623 | 1630 | NLEQKYEK | | | | | Mascot |
| 1108.5746 | 1108.5502 | -0.0244 | -22 | 1390 | 1398 | SFIRDEVSR | | | | | Mascot |
| 1108.5746 | 1108.5502 | -0.0244 | -22 | 1390 | 1398 | SFIRDEVSR | | | | | Mascot |
| 1118.4823 | 1118.634 | 0.1517 | 136 | 1020 | 1028 | NSMSEFFEK | | | | | Mascot |
| 1127.6532 | 1127.5524 | -0.1008 | -89 | 1060 | 1068 | KLNDILQQR | 4 | 0 | | | Mascot |
| 1127.6532 | 1127.5524 | -0.1008 | -89 | 1060 | 1068 | KLNDILQQR | | | | | Mascot |
| 1380.5332 | 1380.7235 | 0.1903 | 138 | 460 | 470 | VDEEDDEDCRR | | | | | Mascot |
| 2039.9895 | 2039.9558 | -0.0337 | -17 | 275 | 292 | AETPMVFFFRFGSVSSS K | | | Oxidation (M)[5] | | Mascot |
| 2110.0459 | 2110.0537 | 0.0078 | 4 | 318 | 336 | SRVLMDGVVEIAWFCPS GK | | | Oxidation (M)[5] | | Mascot |
| 2194.0129 | 2194.0354 | 0.0225 | 10 | 198 | 216 | IDPMDVQMAVFHCADGF LK | | | Carbamidomethyl (C)[13] | | Mascot |
| 2225.1033 | 2225.0657 | -0.0376 | -17 | 293 | 310 | SHLMNSLINEKHNTFFHR | | | | | Mascot |
| 2264.1016 | 2263.9163 | -0.1853 | -82 | 829 | 848 | LYPSCVFVHQNVSDITAG EK | | | Carbamidomethyl (C)[5] | | Mascot |
| 2536.2791 | 2536.1211 | -0.158 | -62 | 1255 | 1275 | YEFKNEFFVELVLSICNG SIK | | | Carbamidomethyl (C)[16] | | Mascot |
| 2606.1333 | 2606.1165 | -0.0168 | -6 | 341 | 362 | FNNCVAFCNLHGDAGEH EKQMK | | | Carbamidomethyl (C)[4,8] | | Mascot |
| 2871.3372 | 2871.394 | 0.0568 | 20 | 198 | 222 | IDPMDVQMAVFHCADGF LKQMMVTK | | | Oxidation (M)[4] | | Mascot |
| 2871.3372 | 2871.394 | 0.0568 | 20 | 198 | 222 | IDPMDVQMAVFHCADGF LKQMMVTK | | | Oxidation (M)[4] | | Mascot |
| 2928.3584 | 2928.3816 | 0.0232 | 8 | 198 | 222 | IDPMDVQMAVFHCADGF | | | Carbamidomethyl (C)[13], Oxidation (M)[4] | | Mascot |

| Calc. Mass | Obsrv. Mass | ± da | ± ppm | Start Seq. | End Seq. | Sequence | Ion Score | C. I. % | Modification | Rank | Result Type |
|---|---|---|---|---|---|---|---|---|---|---|---|
| | | | | | | LKQMMVTK | | | | | |
| 2928.3584 | 2928.3816 | 0.0232 | 8 | 198 | 222 | IDPMDVQMAVFHCADGF LKQMMVTK | | | Carbamidomethyl (C)[13], Oxidation (M)[4] | | Mascot |
| 3337.7024 | 3337.6738 | -0.0286 | -9 | 387 | 414 | IIQELFTNPKPLICLFTEDD SNIFELER | | | | | Mascot |
| 3366.6306 | 3366.4951 | -0.1355 | -40 | 128 | 155 | YTLQTVESCAEEELVPTF LQKLLMMDYR | | | Oxidation (M)[24] | | Mascot |
| 3366.6306 | 3366.4951 | -0.1355 | -40 | 128 | 155 | YTLQTVESCAEEELVPTF LQKLLMMDYR | | | Oxidation (M)[24] | | Mascot |

| | | | | | | | |
|---|---|---|---|---|---|---|---|
| 39 | PREDICTED: coiled-coil domain-containing protein 42 like-2-like [Danio rerio] | gi\|326667730 | 29260.3 | 8.67 | 6 | 29 | 0 |

**Peptide Information**

| Calc. Mass | Obsrv. Mass | ± da | ± ppm | Start Seq. | End Seq. | Sequence | Ion Score | C. I. % | Modification | Rank | Result Type |
|---|---|---|---|---|---|---|---|---|---|---|---|
| 1380.7556 | 1380.7235 | -0.0321 | -23 | 123 | 133 | TKLELMQYINK | | | | | Mascot |
| 1564.8304 | 1564.8866 | 0.0562 | 36 | 189 | 200 | NIYQMIITHYIR | | | | | Mascot |
| 1720.9315 | 1720.9137 | -0.0178 | -10 | 189 | 201 | NIYQMIITHYIRR | | | | | Mascot |
| 2022.0953 | 2021.9862 | -0.1091 | -54 | 185 | 200 | TTVRNIYQMIITHYIR | | | | | Mascot |
| 2216.1492 | 2215.9321 | -0.2171 | -98 | 89 | 107 | FENPCQVIDRIQALQSINK | | | | | Mascot |
| 2928.3542 | 2928.3816 | 0.0274 | 9 | 1 | 26 | MDNVFSGLVFGSTLMSF SPYKENDAR | | | Oxidation (M)[1] | | Mascot |
| 2928.3542 | 2928.3816 | 0.0274 | 9 | 1 | 26 | MDNVFSGLVFGSTLMSF SPYKENDAR | | | Oxidation (M)[1] | | Mascot |

| | | | | | | | |
|---|---|---|---|---|---|---|---|
| 40 | Ppp4r2a protein [Danio rerio] | gi\|38173867 | 48758.5 | 4.37 | 8 | 29 | 0 |

**Peptide Information**

| Calc. Mass | Obsrv. Mass | ± da | ± ppm | Start Seq. | End Seq. | Sequence | Ion Score | C. I. % | Modification | Rank | Result Type |
|---|---|---|---|---|---|---|---|---|---|---|---|
| 850.4199 | 850.527 | 0.1071 | 126 | 165 | 172 | KGSSCVNR | | | | | Mascot |
| 1045.5598 | 1045.5433 | -0.0165 | -16 | 127 | 135 | LCELLTEPK | | | | | Mascot |
| 1174.6506 | 1174.553 | -0.0976 | -83 | 70 | 78 | SYFLFKLEK | | | | | Mascot |
| 1707.837 | 1707.7482 | -0.0888 | -52 | 20 | 34 | HTSTMEIDTLLGAFR | | | Oxidation (M)[5] | | Mascot |
| 1743.8734 | 1743.7939 | -0.0795 | -46 | 43 | 57 | ESCPILDQFLSHVAK | | | Carbamidomethyl (C)[3] | | Mascot |
| 2211.075 | 2211.053 | -0.022 | -10 | 20 | 38 | HTSTMEIDTLLGAFRDFE K | | | | | Mascot |
| 2606.2014 | 2606.1165 | -0.0849 | -33 | 85 | 107 | ASCPEQRGPANPNVEYI PFEEMK | | | | | Mascot |
| 2663.2229 | 2663.3643 | 0.1414 | 53 | 85 | 107 | ASCPEQRGPANPNVEYI PFEEMK | | | Carbamidomethyl (C)[3] | | Mascot |
| 2663.2229 | 2663.3643 | 0.1414 | 53 | 85 | 107 | ASCPEQRGPANPNVEYI PFEEMK | | | Carbamidomethyl (C)[3] | | Mascot |
| 3338.6633 | 3338.6826 | 0.0193 | 6 | 173 | 203 | MNGVMFPGNTSAFPDRN VNGPGTPRPLNRPK | | | | | Mascot |

| | | | | | | | |
|---|---|---|---|---|---|---|---|
| 41 | PREDICTED: patatin-like phospholipase domain-containing protein 4-like [Danio rerio] | gi\|326664037 | 11777 | 9.03 | 4 | 29 | 0 |

Peptide Information

| Calc. Mass | Obsrv. Mass | ± da | ± ppm | Start Seq. | End Seq. | Sequence | Ion Score | C. I. % | Modification | Rank | Result Type |
|---|---|---|---|---|---|---|---|---|---|---|---|
| 1974.9987 | 1975.0669 | 0.0682 | 35 | 37 | 57 | ACAGASAGALVAAMIITA PDK | | | Carbamidomethyl (C)[2], Oxidation (M)[14] | | Mascot |
| 2087.0378 | 2086.842 | -0.1958 | -94 | 74 | 91 | SQRFGAVTPGYDFMLEL R | | | | | Mascot |
| 2559.3884 | 2559.1748 | -0.2136 | -83 | 31 | 57 | LLSSLKACAGASAGALVA AMIITAPDK | | | Oxidation (M)[20] | | Mascot |
| 2559.3884 | 2559.1748 | -0.2136 | -83 | 31 | 57 | LLSSLKACAGASAGALVA AMIITAPDK | | | Oxidation (M)[20] | | Mascot |
| 2663.4741 | 2663.3643 | -0.1098 | -41 | 1 | 26 | MVVNVSFAAAGFLGAYH LGVAKALLK | | | Oxidation (M)[1] | | Mascot |
| 2663.4741 | 2663.3643 | -0.1098 | -41 | 1 | 26 | MVVNVSFAAAGFLGAYH LGVAKALLK | | | Oxidation (M)[1] | | Mascot |

42 Zgc:55395 protein [Danio rerio] gi|159155715 38358.6 8.55 8 29 0

Peptide Information

| Calc. Mass | Obsrv. Mass | ± da | ± ppm | Start Seq. | End Seq. | Sequence | Ion Score | C. I. % | Modification | Rank | Result Type |
|---|---|---|---|---|---|---|---|---|---|---|---|
| 882.577 | 882.5476 | -0.0294 | -33 | 308 | 316 | GLPKLGIGK | | | | | Mascot |
| 1380.5704 | 1380.7235 | 0.1531 | 111 | 1 | 11 | MPVQCSNCEQK | | | Carbamidomethyl (C)[5,8] | | Mascot |
| 1548.8632 | 1548.9131 | 0.0499 | 32 | 83 | 96 | LLLLSVDEGITGYR | | | | | Mascot |
| 1564.7272 | 1564.8866 | 0.1594 | 102 | 191 | 206 | CTAISTSSEGDGAIPR | | | | | Mascot |
| 1707.9752 | 1707.7482 | -0.227 | -133 | 62 | 76 | DSTVLAHVIKLLNER | | | | | Mascot |
| 1720.8282 | 1720.9137 | 0.0855 | 50 | 190 | 206 | RCTAISTSSEGDGAIPR | | | | | Mascot |
| 2110.1501 | 2110.0537 | -0.0964 | -46 | 50 | 71 | GETVAIGASGGKDSTVLA HVIK | | | | | Mascot |
| 2248.106 | 2247.9194 | -0.1866 | -83 | 191 | 211 | CTAISTSSEGDGAIPRCK PLK | | | Carbamidomethyl (C)[1,17] | | Mascot |

43 PREDICTED: zinc finger protein 135-like, partial [Danio rerio] gi|326668043 50492.5 9.56 11 29 0

Peptide Information

| Calc. Mass | Obsrv. Mass | ± da | ± ppm | Start Seq. | End Seq. | Sequence | Ion Score | C. I. % | Modification | Rank | Result Type |
|---|---|---|---|---|---|---|---|---|---|---|---|
| 1330.5588 | 1330.6335 | 0.0747 | 56 | 402 | 412 | EKPFTCTCCGK | | | Carbamidomethyl (C)[6,8] | | Mascot |
| 1557.759 | 1557.7168 | -0.0422 | -27 | 245 | 257 | SFNQPSNLNLHMR | | | | | Mascot |
| 1573.754 | 1573.7124 | -0.0416 | -26 | 245 | 257 | SFNQPSNLNLHMR | | | Oxidation (M)[12] | | Mascot |
| 1688.7883 | 1688.8339 | 0.0456 | 27 | 202 | 216 | VHTGEKPFTCPQCGK | | | Carbamidomethyl (C)[10] | | Mascot |
| 1720.7604 | 1720.9137 | 0.1533 | 89 | 402 | 415 | EKPFTCTCCGKSFR | | | Carbamidomethyl (C)[6,8] | | Mascot |
| 2039.979 | 2039.9558 | -0.0232 | -11 | 370 | 387 | IHTGEKPFTCTQCGKSFR | | | | | Mascot |
| 2086.9731 | 2086.842 | -0.1311 | -63 | 227 | 244 | HMRIHTGEKPFTCNQCG K | | | | | Mascot |

| | | | | | | | | | | | |
|---|---|---|---|---|---|---|---|---|---|---|---|
| 2663.2122 | 2663.3643 | 0.1521 | 57 | 109 | 132 | SSSLNLHIMSHNGEKPFT CTQCGR | | | Oxidation (M)[9] | | Mascot |
| 2663.2122 | 2663.3643 | 0.1521 | 57 | 109 | 132 | SSSLNLHIMSHNGEKPFT CTQCGR | | | Oxidation (M)[9] | | Mascot |
| 2680.2542 | 2680.2212 | -0.033 | -12 | 87 | 108 | HMRIHTGEKPFTCTQCG TSFIR | | | Carbamidomethyl (C)[13,16], Oxidation (M)[2] | | Mascot |
| 2697.1714 | 2697.1252 | -0.0462 | -17 | 11 | 33 | KPFTCTQCGNSFNSSSH LNQHMR | | | Carbamidomethyl (C)[5], Oxidation (M)[22] | | Mascot |
| 2697.1714 | 2697.1252 | -0.0462 | -17 | 11 | 33 | KPFTCTQCGNSFNSSSH LNQHMR | | | Carbamidomethyl (C)[5], Oxidation (M)[22] | | Mascot |
| 3337.5525 | 3337.6738 | 0.1213 | 36 | 273 | 300 | NFIHSSHLNEHMRIHTGE KPFTCTQCGR | | | Carbamidomethyl (C)[23] | | Mascot |
| 3366.5676 | 3366.4951 | -0.0725 | -22 | 258 | 285 | IHTGEKPFTCTQCGKNFI HSSHLNEHMR | | | Carbamidomethyl (C)[10,13] | | Mascot |
| 3366.5676 | 3366.4951 | -0.0725 | -22 | 258 | 285 | IHTGEKPFTCTQCGKNFI HSSHLNEHMR | | | Carbamidomethyl (C)[10,13] | | Mascot |

| | | | | | | | | | | |
|---|---|---|---|---|---|---|---|---|---|---|
| 44 | Golgi associated, gamma adaptin ear containing, ARF binding protein 3 [Danio rerio] | gi\|190340128 | 76353 | 5.35 | 4 | 29 | 0 | 21 | 0 |

**Peptide Information**

| Calc. Mass | Obsrv. Mass | ± da | ± ppm | Start Seq. | End Seq. | Sequence | Ion Score | C. I. % | Modification | Rank | Result Type |
|---|---|---|---|---|---|---|---|---|---|---|---|
| 842.5458 | 842.4929 | -0.0529 | -63 | 175 | 181 | RLAELLK | | | | | Mascot |
| 1127.6055 | 1127.5524 | -0.0531 | -47 | 39 | 48 | ELEGPQISVR | 21 | 0 | | | Mascot |
| 1127.6055 | 1127.5524 | -0.0531 | -47 | 39 | 48 | ELEGPQISVR | | | | | Mascot |
| 2384.2126 | 2383.8892 | -0.3234 | -136 | 294 | 316 | IVEGQTIVIDVPASTPSGC INDR | | | | | Mascot |
| 2559.1692 | 2559.1748 | 0.0056 | 2 | 263 | 286 | LATETEDDDSALGDILHA SDDLSR | | | | | Mascot |
| 2559.1692 | 2559.1748 | 0.0056 | 2 | 263 | 286 | LATETEDDDSALGDILHA SDDLSR | | | | | Mascot |

| | | | | | | | |
|---|---|---|---|---|---|---|---|
| 45 | novel protein [Danio rerio] | gi\|94733564 | 88236.8 | 8.75 | 10 | 29 | 0 |

**Peptide Information**

| Calc. Mass | Obsrv. Mass | ± da | ± ppm | Start Seq. | End Seq. | Sequence | Ion Score | C. I. % | Modification | Rank | Result Type |
|---|---|---|---|---|---|---|---|---|---|---|---|
| 842.4842 | 842.4929 | 0.0087 | 10 | 556 | 562 | IVDPSRR | | | | | Mascot |
| 900.4825 | 900.4632 | -0.0193 | -21 | 517 | 523 | LINSYYK | | | | | Mascot |
| 1473.6501 | 1473.8102 | 0.1601 | 109 | 499 | 510 | WSYKEICMGNDK | | | | | Mascot |
| 1473.6501 | 1473.8102 | 0.1601 | 109 | 499 | 510 | WSYKEICMGNDK | | | | | Mascot |
| 1534.7179 | 1534.6222 | -0.0957 | -62 | 179 | 192 | NPVAMVHANDGTHR | | | Oxidation (M)[5] | | Mascot |
| 1620.8091 | 1620.7225 | -0.0866 | -53 | 511 | 523 | TCSFPKLINSYYK | | | Carbamidomethyl (C)[2] | | Mascot |
| 2233.0151 | 2233.0376 | 0.0225 | 10 | 399 | 417 | MFCGDVGQNKYEEVDLI EK | | | Oxidation (M)[1] | | Mascot |
| 2283.0222 | 2283.125 | 0.1028 | 45 | 43 | 61 | RFHHIMDYFDHTGFTAC GK | | | | | Mascot |
| 2559.1697 | 2559.1748 | 0.0051 | 2 | 44 | 64 | FHHIMDYFDHTGFTACGK YIR | | | | | Mascot |
| 2559.1697 | 2559.1748 | 0.0051 | 2 | 44 | 64 | FHHIMDYFDHTGFTACGK | | | | | Mascot |

| Calc. Mass | Obsrv. Mass | ± da | ± ppm | Start Seq. | End Seq. | Sequence | Ion Score | C. I. % | Modification | Rank | Result Type |
|---|---|---|---|---|---|---|---|---|---|---|---|
| | | | | | | YIR | | | | | |
| 2871.2383 | 2871.394 | 0.1557 | 54 | 65 | 89 | SILCQECSPYSAHLYDAE DANTPMR | | | Carbamidomethyl (C)[4] | | Mascot |
| 2871.2383 | 2871.394 | 0.1557 | 54 | 65 | 89 | SILCQECSPYSAHLYDAE DANTPMR | | | Carbamidomethyl (C)[4] | | Mascot |
| 2928.2598 | 2928.3816 | 0.1218 | 42 | 65 | 89 | SILCQECSPYSAHLYDAE DANTPMR | | | Carbamidomethyl (C)[4,7] | | Mascot |
| 2928.2598 | 2928.3816 | 0.1218 | 42 | 65 | 89 | SILCQECSPYSAHLYDAE DANTPMR | | | Carbamidomethyl (C)[4,7] | | Mascot |
| 3366.6179 | 3366.4951 | -0.1228 | -36 | 708 | 737 | GEWGTVCDDLFNIQAAA VVCRQMGFPVAVR | | | Carbamidomethyl (C)[7,20] | | Mascot |
| 3366.6179 | 3366.4951 | -0.1228 | -36 | 708 | 737 | GEWGTVCDDLFNIQAAA VVCRQMGFPVAVR | | | Carbamidomethyl (C)[7,20] | | Mascot |

| 46 | SH3 domain-binding protein 4-A [Danio rerio] | gi|45387913 | 104947.5 | 7.73 | 12 | 29 | 0 |
|---|---|---|---|---|---|---|---|

**Peptide Information**

| Calc. Mass | Obsrv. Mass | ± da | ± ppm | Start Seq. | End Seq. | Sequence | Ion Score | C. I. % | Modification | Rank | Result Type |
|---|---|---|---|---|---|---|---|---|---|---|---|
| 842.5822 | 842.4929 | -0.0893 | -106 | 634 | 640 | LIKTVIR | | | | | Mascot |
| 850.4338 | 850.527 | 0.0932 | 110 | 358 | 364 | LSNMEIK | | | Oxidation (M)[4] | | Mascot |
| 1045.4796 | 1045.5433 | 0.0637 | 61 | 762 | 770 | AELDSEPER | | | | | Mascot |
| 1380.6498 | 1380.7235 | 0.0737 | 53 | 778 | 788 | LKEDCLNMETK | | | Carbamidomethyl (C)[5] | | Mascot |
| 1620.8591 | 1620.7225 | -0.1366 | -84 | 554 | 567 | ADEISDFTLRVQVK | | | | | Mascot |
| 1720.8037 | 1720.9137 | 0.11 | 64 | 247 | 260 | APSPEQFQNREDFR | | | | | Mascot |
| 1904.0166 | 1903.8071 | -0.2095 | -110 | 796 | 812 | ELMMALLKIDCQGLVAR | | | | | Mascot |
| 2039.9922 | 2039.9558 | -0.0364 | -18 | 373 | 390 | VSVMTRTESPMAEIVCVR | | | Oxidation (M)[4,11] | | Mascot |
| 2284.2368 | 2284.1194 | -0.1174 | -51 | 463 | 484 | TVVAIFGHDCAPKTLLVS EVGK | | | | | Mascot |
| 2697.3374 | 2697.1252 | -0.2122 | -79 | 564 | 587 | VQVKDDQDCILAQFCVQ TPQPPPK | | | | | Mascot |
| 2697.3374 | 2697.1252 | -0.2122 | -79 | 564 | 587 | VQVKDDQDCILAQFCVQ TPQPPPK | | | | | Mascot |
| 3337.6052 | 3337.6738 | 0.0686 | 21 | 269 | 298 | LARSCHDLESLGQNPGW GQTQPVETNIVCK | | | Carbamidomethyl (C)[5] | | Mascot |
| 3338.5496 | 3338.6826 | 0.133 | 40 | 856 | 884 | TGMVDSEAMWKPAYDFL LTWAAQIGDSYR | | | Oxidation (M)[3] | | Mascot |

| 47 | PREDICTED: myosin-Ib isoform 1 [Danio rerio] | gi|326670574 | 124410.6 | 9.28 | 14 | 29 | 0 |
|---|---|---|---|---|---|---|---|

**Peptide Information**

| Calc. Mass | Obsrv. Mass | ± da | ± ppm | Start Seq. | End Seq. | Sequence | Ion Score | C. I. % | Modification | Rank | Result Type |
|---|---|---|---|---|---|---|---|---|---|---|---|
| 842.4263 | 842.4929 | 0.0666 | 79 | 642 | 647 | YKMLCK | | | Carbamidomethyl (C)[5] | | Mascot |
| 996.4996 | 996.5761 | 0.0765 | 77 | 867 | 874 | SQFTEEKK | | | | | Mascot |
| 1095.4952 | 1095.5037 | 0.0085 | 8 | 419 | 426 | EEQEEYIR | | | | | Mascot |
| 1108.5092 | 1108.5502 | 0.041 | 37 | 633 | 641 | QAYEPCLER | | | | | Mascot |
| 1108.5092 | 1108.5502 | 0.041 | 37 | 633 | 641 | QAYEPCLER | | | | | Mascot |

| | | | | | | | | | | | |
|---|---|---|---|---|---|---|---|---|---|---|---|
| 1363.7018 | 1363.6482 | -0.0536 | -39 | 648 | 658 | QTWPHWKGPAR | | | | | Mascot |
| 1473.713 | 1473.8102 | 0.0972 | 66 | 644 | 654 | MLCKQTWPHWK | | | Oxidation (M)[1] | | Mascot |
| 1473.713 | 1473.8102 | 0.0972 | 66 | 644 | 654 | MLCKQTWPHWK | | | Oxidation (M)[1] | | Mascot |
| 1548.8679 | 1548.9131 | 0.0452 | 29 | 747 | 759 | IRCSALVVQSFIR | | | Carbamidomethyl (C)[3] | | Mascot |
| 1557.8417 | 1557.7168 | -0.1249 | -80 | 698 | 710 | RQCLEDLAALIQK | | | Carbamidomethyl (C)[3] | | Mascot |
| 2194.0886 | 2194.0354 | -0.0532 | -24 | 278 | 296 | LGNIEFKPESRVNGFDESR | | | | | Mascot |
| 2264.0876 | 2263.9163 | -0.1713 | -76 | 776 | 794 | CEEAVTTISAYWHGTQVRR | | | Carbamidomethyl (C)[1] | | Mascot |
| 2384.1716 | 2383.8892 | -0.2824 | -118 | 496 | 515 | FLTDHSLPHNCFRIQHYAGK | | | | | Mascot |
| 2536.293 | 2536.1211 | -0.1719 | -68 | 659 | 681 | EGVEVLLSDLQVPAEEFAYGRSK | | | | | Mascot |
| 2853.3645 | 2853.3992 | 0.0347 | 12 | 451 | 475 | NGILAMLDEECLRPGTVTDETFLDK | | | Carbamidomethyl (C)[11], Oxidation (M)[6] | | Mascot |
| 3366.7058 | 3366.4951 | -0.2107 | -63 | 5 | 34 | TSLLDNMIGVGDMVLLEPLSEESFIENLKK | | | Oxidation (M)[7,13] | | Mascot |
| 3366.7058 | 3366.4951 | -0.2107 | -63 | 5 | 34 | TSLLDNMIGVGDMVLLEPLSEESFIENLKK | | | Oxidation (M)[7,13] | | Mascot |

| | | | | | | | | | |
|---|---|---|---|---|---|---|---|---|---|
| 48 | protein phosphatase 1 regulatory subunit 7 [Danio rerio] | gi\|82658210 | 39360.8 | 4.94 | 1 | | 19 | 0 | |

**Peptide Information**

| Calc. Mass | Obsrv. Mass | ± da | ± ppm | Start Seq. | End Seq. | Sequence | Ion Score | C. I. % | Modification | Rank | Result Type |
|---|---|---|---|---|---|---|---|---|---|---|---|
| 1127.6969 | 1127.5524 | -0.1445 | -128 | 327 | 336 | KIMLALPSVR | 19 | 0 | | 3 | Mascot |

| | | | | | | | | | |
|---|---|---|---|---|---|---|---|---|---|
| 49 | PREDICTED: sushi, nidogen and EGF-like domains 1 [Danio rerio] | gi\|326668662 | 146125.5 | 5.54 | 1 | | 19 | 0 | |

**Peptide Information**

| Calc. Mass | Obsrv. Mass | ± da | ± ppm | Start Seq. | End Seq. | Sequence | Ion Score | C. I. % | Modification | Rank | Result Type |
|---|---|---|---|---|---|---|---|---|---|---|---|
| 1127.5084 | 1127.5524 | 0.044 | 39 | 1270 | 1277 | RCELYCQR | 19 | 0 | Carbamidomethyl (C)[6] | 4 | Mascot |

| | | | | | | | | | |
|---|---|---|---|---|---|---|---|---|---|
| 50 | exosome component 10 [Danio rerio] | gi\|210147550 | 102307.6 | 8.64 | 1 | | 17 | 0 | |

**Peptide Information**

| Calc. Mass | Obsrv. Mass | ± da | ± ppm | Start Seq. | End Seq. | Sequence | Ion Score | C. I. % | Modification | Rank | Result Type |
|---|---|---|---|---|---|---|---|---|---|---|---|
| 1108.6473 | 1108.5502 | -0.0971 | -88 | 645 | 655 | KSVHGLTAPAK | 17 | 0 | | 2 | Mascot |

| | | | | | |
|---|---|---|---|---|---|
| **Gel Idx/Pos** | 185/H12 | **Instr./Gel Origin** | BA2060/110707A | **Process Status** | Analysis Succeeded |
| **Plate [#] Name** | [1] 31496 | **Instrument Sample Name** | | **Spectra** | 11 |

| Rank | Protein Name | Accession No. | Protein MW | Protein PI | Pep. Count | Protein Score | Protein Score C. I. % | Total Ion Score | Total Ion C. I. % |
|---|---|---|---|---|---|---|---|---|---|

| 1 | hypothetical protein LOC792190 [Danio rerio] | gi\|224495957 | 78018.4 | 6.21 | 20 | 962 | 100 | 845 | 100 |
|---|---|---|---|---|---|---|---|---|---|

**Peptide Information**

| Calc. Mass | Obsrv. Mass | ± da | ± ppm | Start Seq. | End Seq. | Sequence | Ion Score | C. I. % | Modification | Rank | Result Type |
|---|---|---|---|---|---|---|---|---|---|---|---|
| 803.3794 | 803.3616 | -0.0178 | -22 | 366 | 372 | GHSFEAR | | | | | Mascot |
| 902.4875 | 902.4664 | -0.0211 | -23 | 532 | 538 | LNVLQCR | | | Carbamidomethyl (C)[6] | | Mascot |
| 973.5101 | 973.4905 | -0.0196 | -20 | 424 | 431 | LVVWGEDR | | | | | Mascot |
| 973.5101 | 973.4905 | -0.0196 | -20 | 424 | 431 | LVVWGEDR | 46 | 99.31 | | | Mascot |
| 1058.5887 | 1058.5662 | -0.0225 | -21 | 531 | 538 | RLNVLQCR | | | Carbamidomethyl (C)[7] | | Mascot |
| 1082.5112 | 1082.4855 | -0.0257 | -24 | 62 | 71 | SVAVYSDADR | | | | | Mascot |
| 1325.7423 | 1325.7146 | -0.0277 | -21 | 355 | 365 | LPLSQEQIELR | 52 | 99.854 | | | Mascot |
| 1325.7423 | 1325.7146 | -0.0277 | -21 | 355 | 365 | LPLSQEQIELR | | | | | Mascot |
| 1563.8376 | 1563.8031 | -0.0345 | -22 | 272 | 286 | IIEEAPGPGISPEVR | | | | | Mascot |
| 1563.8376 | 1563.8031 | -0.0345 | -22 | 272 | 286 | IIEEAPGPGISPEVR | 102 | 100 | | | Mascot |
| 1584.8744 | 1584.8403 | -0.0341 | -22 | 131 | 145 | QEGIIFIGPPSSAIR | 130 | 100 | | | Mascot |
| 1584.8744 | 1584.8403 | -0.0341 | -22 | 131 | 145 | QEGIIFIGPPSSAIR | | | | | Mascot |
| 1728.7823 | 1728.7454 | -0.0369 | -21 | 515 | 530 | EQTSDVFSPFASSNGR | | | | | Mascot |
| 1844.8773 | 1844.8348 | -0.0425 | -23 | 204 | 219 | IAQSEAEFHEQLESAR | | | | | Mascot |
| 1844.8773 | 1844.8348 | -0.0425 | -23 | 204 | 219 | IAQSEAEFHEQLESAR | 119 | 100 | | | Mascot |
| 1881.0439 | 1880.9956 | -0.0483 | -26 | 349 | 365 | VAAGEKLPLSQEQIELR | | | | | Mascot |
| 2000.9784 | 2000.9335 | -0.0449 | -22 | 204 | 220 | IAQSEAEFHEQLESARR | | | | | Mascot |
| 2047.9468 | 2047.9043 | -0.0425 | -21 | 513 | 530 | YREQTSDVFSPFASSNG R | | | | | Mascot |
| 2117.9807 | 2117.9324 | -0.0483 | -23 | 549 | 568 | VAVSITYNPDGTYSMETG GR | | | | | Mascot |
| 2133.9756 | 2133.9275 | -0.0481 | -23 | 549 | 568 | VAVSITYNPDGTYSMETG GR | | | Oxidation (M)[15] | | Mascot |
| 2371.1353 | 2371.0811 | -0.0542 | -23 | 242 | 261 | HVEVQVFGDQYGDAVYL FER | 149 | 100 | | | Mascot |
| 2371.1353 | 2371.0811 | -0.0542 | -23 | 242 | 261 | HVEVQVFGDQYGDAVYL FER | | | | | Mascot |
| 2439.1357 | 2439.0789 | -0.0568 | -23 | 155 | 177 | HIMSAAGVPIIEGYHGDD QSDAR | | | | | Mascot |
| 2455.1306 | 2455.083 | -0.0476 | -19 | 155 | 177 | HIMSAAGVPIIEGYHGDD QSDAR | | | Oxidation (M)[3] | | Mascot |
| 2580.3127 | 2580.2556 | -0.0571 | -22 | 327 | 348 | LQVEHPVSEMITGTDLVE WQLR | | | | | Mascot |
| 2580.3127 | 2580.2556 | -0.0571 | -22 | 327 | 348 | LQVEHPVSEMITGTDLVE WQLR | 112 | 100 | | | Mascot |
| 2596.3076 | 2596.2539 | -0.0537 | -21 | 327 | 348 | LQVEHPVSEMITGTDLVE WQLR | 51 | 99.805 | Oxidation (M)[10] | | Mascot |
| 2596.3076 | 2596.2539 | -0.0537 | -21 | 327 | 348 | LQVEHPVSEMITGTDLVE WQLR | | | Oxidation (M)[10] | | Mascot |
| 2657.1936 | 2657.1265 | -0.0671 | -25 | 107 | 130 | SSAQAVHPGYGFLSENT EFAEQCK | | | Carbamidomethyl (C)[23] | | Mascot |

| | | | | | | | | | | | |
|---|---|---|---|---|---|---|---|---|---|---|---|
| 3218.5093 | 3218.5154 | 0.0061 | 2 | 539 | 568 | NMSLQLGDNKVAVSITYN PDGTYSMETGGR | | | | | Mascot |
| 3218.5864 | 3218.5154 | -0.071 | -22 | 373 | 402 | IYAEDPNNNFLPGAGPLL HLSTPPGDQNTR | 137 | 100 | | | Mascot |

| | | | | | | | | | | | |
|---|---|---|---|---|---|---|---|---|---|---|---|
| 2 | hypothetical protein LOC777632 [Danio rerio] | gi\|118150458 | 77993.3 | 6.12 | 18 | 943 | 100 | 847 | 100 | | |

**Peptide Information**

| Calc. Mass | Obsrv. Mass | ± da | ± ppm | Start Seq. | End Seq. | Sequence | Ion Score | C. I. % | Modification | Rank | Result Type |
|---|---|---|---|---|---|---|---|---|---|---|---|
| 803.3794 | 803.3616 | -0.0178 | -22 | 366 | 372 | GHSFEAR | | | | | Mascot |
| 902.4875 | 902.4664 | -0.0211 | -23 | 532 | 538 | LNVLQCR | | | Carbamidomethyl (C)[6] | | Mascot |
| 973.5101 | 973.4905 | -0.0196 | -20 | 424 | 431 | LVVWGEDR | | | | | Mascot |
| 973.5101 | 973.4905 | -0.0196 | -20 | 424 | 431 | LVVWGEDR | 46 | 99.31 | | | Mascot |
| 1058.5887 | 1058.5662 | -0.0225 | -21 | 531 | 538 | RLNVLQCR | | | Carbamidomethyl (C)[7] | | Mascot |
| 1082.5112 | 1082.4855 | -0.0257 | -24 | 62 | 71 | SVAVYSDADR | | | | | Mascot |
| 1325.7423 | 1325.7146 | -0.0277 | -21 | 355 | 365 | LPLSQEQIELR | 52 | 99.854 | | | Mascot |
| 1325.7423 | 1325.7146 | -0.0277 | -21 | 355 | 365 | LPLSQEQIELR | | | | | Mascot |
| 1563.8376 | 1563.8031 | -0.0345 | -22 | 272 | 286 | IIEEAPGPGISPEVR | | | | | Mascot |
| 1563.8376 | 1563.8031 | -0.0345 | -22 | 272 | 286 | IIEEAPGPGISPEVR | 102 | 100 | | | Mascot |
| 1584.8744 | 1584.8403 | -0.0341 | -22 | 131 | 145 | QEGIIFIGPPSSAIR | 130 | 100 | | | Mascot |
| 1584.8744 | 1584.8403 | -0.0341 | -22 | 131 | 145 | QEGIIFIGPPSSAIR | | | | | Mascot |
| 1728.7823 | 1728.7454 | -0.0369 | -21 | 515 | 530 | EQTSDVFSPFASSNGR | | | | | Mascot |
| 1844.8773 | 1844.8348 | -0.0425 | -23 | 204 | 219 | IAQSEAEFHEQLESAR | | | | | Mascot |
| 1844.8773 | 1844.8348 | -0.0425 | -23 | 204 | 219 | IAQSEAEFHEQLESAR | 119 | 100 | | | Mascot |
| 1881.0439 | 1880.9956 | -0.0483 | -26 | 349 | 365 | VAAGEKLPLSQEQIELR | | | | | Mascot |
| 2000.9784 | 2000.9335 | -0.0449 | -22 | 204 | 220 | IAQSEAEFHEQLESARR | | | | | Mascot |
| 2047.9468 | 2047.9043 | -0.0425 | -21 | 513 | 530 | YREQTSDVFSPFASSNG R | | | | | Mascot |
| 2371.1353 | 2371.0811 | -0.0542 | -23 | 242 | 261 | HVEVQVFGDQYGDAVYL FER | 149 | 100 | | | Mascot |
| 2371.1353 | 2371.0811 | -0.0542 | -23 | 242 | 261 | HVEVQVFGDQYGDAVYL FER | | | | | Mascot |
| 2439.1357 | 2439.0789 | -0.0568 | -23 | 155 | 177 | HIMSAAGVPIIEGYHGDD QSDAR | | | | | Mascot |
| 2455.1306 | 2455.083 | -0.0476 | -19 | 155 | 177 | HIMSAAGVPIIEGYHGDD QSDAR | | | Oxidation (M)[3] | | Mascot |
| 2580.3127 | 2580.2556 | -0.0571 | -22 | 327 | 348 | LQVEHPVSEMITGTDLVE WQLR | | | | | Mascot |
| 2580.3127 | 2580.2556 | -0.0571 | -22 | 327 | 348 | LQVEHPVSEMITGTDLVE WQLR | 112 | 100 | | | Mascot |
| 2596.3076 | 2596.2539 | -0.0537 | -21 | 327 | 348 | LQVEHPVSEMITGTDLVE WQLR | 51 | 99.805 | Oxidation (M)[10] | | Mascot |
| 2596.3076 | 2596.2539 | -0.0537 | -21 | 327 | 348 | LQVEHPVSEMITGTDLVE WQLR | | | Oxidation (M)[10] | | Mascot |
| 2657.1936 | 2657.1265 | -0.0671 | -25 | 107 | 130 | SSAQAVHPGYGFLSENT EFAEQCK | | | Carbamidomethyl (C)[23] | | Mascot |

| | | | | | | | | | | | |
|---|---|---|---|---|---|---|---|---|---|---|---|
| 3218.5864 | 3218.5154 | -0.071 | -22 | 373 | 402 | IYAEDPNNNFLPGAGPLL HLSTPPGDQNTR | 137 | 100 | | | Mascot |
| 3218.5864 | 3218.5154 | -0.071 | -22 | 373 | 402 | IYAEDPNNNFLPGAGPLL HLSTPPGDQNTR | | | | | Mascot |

| | | | | | | | | | | |
|---|---|---|---|---|---|---|---|---|---|---|
| 3 | hypothetical protein LOC559969 [Danio rerio] | gi\|113678532 | 77970.3 | 6.17 | 20 | 828 | 100 | 710 | 100 | |

**Peptide Information**

| Calc. Mass | Obsrv. Mass | ± da | ± ppm | Start Seq. | End Seq. | Sequence | Ion Score | C. I. % | Modification | Rank | Result Type |
|---|---|---|---|---|---|---|---|---|---|---|---|
| 803.3794 | 803.3616 | -0.0178 | -22 | 366 | 372 | GHSFEAR | | | | | Mascot |
| 902.4875 | 902.4664 | -0.0211 | -23 | 532 | 538 | LNVLQCR | | | Carbamidomethyl (C)[6] | | Mascot |
| 973.5101 | 973.4905 | -0.0196 | -20 | 424 | 431 | LVVWGEDR | | | | | Mascot |
| 973.5101 | 973.4905 | -0.0196 | -20 | 424 | 431 | LVVWGEDR | 46 | 99.31 | | | Mascot |
| 1058.5887 | 1058.5662 | -0.0225 | -21 | 531 | 538 | RLNVLQCR | | | Carbamidomethyl (C)[7] | | Mascot |
| 1082.5112 | 1082.4855 | -0.0257 | -24 | 62 | 71 | SVAVYSDADR | | | | | Mascot |
| 1325.7423 | 1325.7146 | -0.0277 | -21 | 355 | 365 | LPLSQEQIELR | 52 | 99.854 | | | Mascot |
| 1325.7423 | 1325.7146 | -0.0277 | -21 | 355 | 365 | LPLSQEQIELR | | | | | Mascot |
| 1563.8376 | 1563.8031 | -0.0345 | -22 | 272 | 286 | IIEEAPGPGISPEVR | | | | | Mascot |
| 1563.8376 | 1563.8031 | -0.0345 | -22 | 272 | 286 | IIEEAPGPGISPEVR | 102 | 100 | | | Mascot |
| 1584.8744 | 1584.8403 | -0.0341 | -22 | 131 | 145 | QEGIIFIGPPSSAIR | 130 | 100 | | | Mascot |
| 1584.8744 | 1584.8403 | -0.0341 | -22 | 131 | 145 | QEGIIFIGPPSSAIR | | | | | Mascot |
| 1728.7823 | 1728.7454 | -0.0369 | -21 | 515 | 530 | EQTSDVFSPFASSNGR | | | | | Mascot |
| 1844.8773 | 1844.8348 | -0.0425 | -23 | 204 | 219 | IAQSEAEFHEQLESAR | | | | | Mascot |
| 1844.8773 | 1844.8348 | -0.0425 | -23 | 204 | 219 | IAQSEAEFHEQLESAR | 119 | 100 | | | Mascot |
| 1881.0439 | 1880.9956 | -0.0483 | -26 | 349 | 365 | VAAGEKLPLSQEQIELR | | | | | Mascot |
| 2000.9784 | 2000.9335 | -0.0449 | -22 | 204 | 220 | IAQSEAEFHEQLESARR | | | | | Mascot |
| 2047.9468 | 2047.9043 | -0.0425 | -21 | 513 | 530 | YREQTSDVFSPFASSNG R | | | | | Mascot |
| 2117.9807 | 2117.9324 | -0.0483 | -23 | 549 | 568 | VAVSITYNPDGTYSMETG GR | | | | | Mascot |
| 2133.9756 | 2133.9275 | -0.0481 | -23 | 549 | 568 | VAVSITYNPDGTYSMETG GR | | | Oxidation (M)[15] | | Mascot |
| 2371.1353 | 2371.0811 | -0.0542 | -23 | 242 | 261 | HVEVQVFGDQYGDAVYL FER | 149 | 100 | | | Mascot |
| 2371.1353 | 2371.0811 | -0.0542 | -23 | 242 | 261 | HVEVQVFGDQYGDAVYL FER | | | | | Mascot |
| 2439.1357 | 2439.0789 | -0.0568 | -23 | 155 | 177 | HIMSAAGVPIIEGYHGDD QSDAR | | | | | Mascot |
| 2455.1306 | 2455.083 | -0.0476 | -19 | 155 | 177 | HIMSAAGVPIIEGYHGDD QSDAR | | | Oxidation (M)[3] | | Mascot |
| 2580.3127 | 2580.2556 | -0.0571 | -22 | 327 | 348 | LQVEHPVSEMITGTDLVE WQLR | | | | | Mascot |
| 2580.3127 | 2580.2556 | -0.0571 | -22 | 327 | 348 | LQVEHPVSEMITGTDLVE WQLR | 112 | 100 | | | Mascot |
| 2596.3076 | 2596.2539 | -0.0537 | -21 | 327 | 348 | LQVEHPVSEMITGTDLVE WQLR | 51 | 99.805 | Oxidation (M)[10] | | Mascot |

| Calc. Mass | Obsrv. Mass | ± da | ± ppm | Start Seq. | End Seq. | Sequence | Ion Score | C. I. % | Modification | Rank | Result Type |
|---|---|---|---|---|---|---|---|---|---|---|---|
| 2596.3076 | 2596.2539 | -0.0537 | -21 | 327 | 348 | LQVEHPVSEMITGTDLVE WQLR | | | Oxidation (M)[10] | | Mascot |
| 2657.1936 | 2657.1265 | -0.0671 | -25 | 107 | 130 | SSAQAVHPGYGFLSENT EFAEQCK | | | Carbamidomethyl (C)[23] | | Mascot |
| 3218.5093 | 3218.5154 | 0.0061 | 2 | 539 | 568 | NMSLQLGDNKVAVSITYN PDGTYSMETGGR | | | | | Mascot |
| 3218.5093 | 3218.5154 | 0.0061 | 2 | 539 | 568 | NMSLQLGDNKVAVSITYN PDGTYSMETGGR | | | | | Mascot |
| 3227.5869 | 3227.5264 | -0.0605 | -19 | 373 | 402 | IYAEDPNNNFLPGAGPLL HLSTPPGDHNTR | | | | | Mascot |

| | | | | | | | |
|---|---|---|---|---|---|---|---|
| 4 | PREDICTED: serine/threonine-protein kinase pim-2-like [Danio rerio] | gi\|326675052 | 52407.5 | 9.24 | 10 | 43 | 0 |

**Peptide Information**

| Calc. Mass | Obsrv. Mass | ± da | ± ppm | Start Seq. | End Seq. | Sequence | Ion Score | C. I. % | Modification | Rank | Result Type |
|---|---|---|---|---|---|---|---|---|---|---|---|
| 864.4971 | 864.4924 | -0.0047 | -5 | 1 | 7 | MLRTTVK | | | Oxidation (M)[1] | | Mascot |
| 1325.6089 | 1325.7146 | 0.1057 | 80 | 159 | 170 | QAVFAAQTCCQR | | | | | Mascot |
| 1325.6089 | 1325.7146 | 0.1057 | 80 | 159 | 170 | QAVFAAQTCCQR | | | | | Mascot |
| 1347.7267 | 1347.6968 | -0.0299 | -22 | 143 | 154 | SYVGPIKEDAIR | | | | | Mascot |
| 1563.8013 | 1563.8031 | 0.0018 | 1 | 299 | 312 | EDLSWTKNSTALAK | | | | | Mascot |
| 1563.8013 | 1563.8031 | 0.0018 | 1 | 299 | 312 | EDLSWTKNSTALAK | | | | | Mascot |
| 1567.7468 | 1567.8163 | 0.0695 | 44 | 159 | 171 | QAVFAAQTCCQRK | | | Carbamidomethyl (C)[9,10] | | Mascot |
| 1844.8969 | 1844.8348 | -0.0621 | -34 | 155 | 170 | FIMKQAVFAAQTCCQR | | | | | Mascot |
| 1844.8969 | 1844.8348 | -0.0621 | -34 | 155 | 170 | FIMKQAVFAAQTCCQR | | | | | Mascot |
| 2192.9331 | 2193.052 | 0.1189 | 54 | 258 | 275 | IMTKDIEGLSEECCDFMR | | | Carbamidomethyl (C)[13], Oxidation (M)[2] | | Mascot |
| 2247.0928 | 2247.0203 | -0.0725 | -32 | 43 | 63 | KYEIGEELGEGGFGTVYA ATR | | | | | Mascot |
| 2283.2529 | 2283.1292 | -0.1237 | -54 | 8 | 27 | SSILPALHQPFKSQTMLQ LK | | | Oxidation (M)[16] | | Mascot |
| 2657.3328 | 2657.1265 | -0.2063 | -78 | 393 | 416 | KALCITADATHPMHSFFS LLPSGR | | | Carbamidomethyl (C)[4] | | Mascot |

| | | | | | | | | | | |
|---|---|---|---|---|---|---|---|---|---|---|
| 5 | RNA 3'-terminal phosphate cyclase [Danio rerio] | gi\|41054721 | 38740.9 | 8.3 | 7 | 41 | 0 | 16 | 0 |

**Peptide Information**

| Calc. Mass | Obsrv. Mass | ± da | ± ppm | Start Seq. | End Seq. | Sequence | Ion Score | C. I. % | Modification | Rank | Result Type |
|---|---|---|---|---|---|---|---|---|---|---|---|
| 864.4244 | 864.4924 | 0.068 | 79 | 209 | 216 | DMSTAAIR | | | | | Mascot |
| 1234.6572 | 1234.6538 | -0.0034 | -3 | 209 | 219 | DMSTAAIRTIR | | | | | Mascot |
| 1347.73 | 1347.6968 | -0.0332 | -25 | 22 | 35 | VSAALSCIQGASIK | | | | | Mascot |
| 1584.889 | 1584.8403 | -0.0487 | -31 | 272 | 285 | VGIEAAEMLLRNIR | 16 | 0 | | | Mascot |
| 1584.889 | 1584.8403 | -0.0487 | -31 | 272 | 285 | VGIEAAEMLLRNIR | | | | | Mascot |
| 2225.1536 | 2225.0662 | -0.0874 | -39 | 139 | 156 | VFKPIVERFGVQFDCDLR | | | Carbamidomethyl (C)[15] | | Mascot |
| 2225.1536 | 2225.0662 | -0.0874 | -39 | 139 | 156 | VFKPIVERFGVQFDCDLR | | | Carbamidomethyl (C)[15] | | Mascot |

| Calc. Mass | Obsrv. Mass | ± da | ± ppm | Start Seq. | End Seq. | Sequence | Ion Score | C. I. % | Modification | Rank | Result Type |
|---|---|---|---|---|---|---|---|---|---|---|---|
| 2232.9934 | 2233.0288 | 0.0354 | 16 | 1 | 21 | MAATTFEMDGSVMEGG GQILR | | | Oxidation (M)[1,8] | | Mascot |
| 2367.0803 | 2367.2065 | 0.1262 | 53 | 61 | 83 | DMCNGNLEGATVGSSEI TLTPGK | | | Carbamidomethyl (C)[3], Oxidation (M)[2] | | Mascot |

| | | | | | | | | | | |
|---|---|---|---|---|---|---|---|---|---|---|
| 6 | COMM domain-containing protein 1 [Danio rerio] | gi\|113674991 | 21219.7 | 5.48 | 4 | 40 | 0 | 23 | 0 | |

Peptide Information

| Calc. Mass | Obsrv. Mass | ± da | ± ppm | Start Seq. | End Seq. | Sequence | Ion Score | C. I. % | Modification | Rank | Result Type |
|---|---|---|---|---|---|---|---|---|---|---|---|
| 870.4614 | 870.5187 | 0.0573 | 66 | 48 | 54 | ALHDKMR | | | | | Mascot |
| 1844.8807 | 1844.8348 | -0.0459 | -25 | 174 | 189 | NMAELQESIDSIVHRS | | | Oxidation (M)[2] | | Mascot |
| 1844.8807 | 1844.8348 | -0.0459 | -25 | 174 | 189 | NMAELQESIDSIVHRS | 23 | 0 | Oxidation (M)[2] | | Mascot |
| 2211.0022 | 2211.0537 | 0.0515 | 23 | 35 | 52 | NELYAEMTEDEFRALHD K | | | | | Mascot |
| 2283.092 | 2283.1292 | 0.0372 | 16 | 59 | 79 | SIASADMDQAQLEAFLTA QTR | | | Oxidation (M)[7] | | Mascot |

| | | | | | | | | |
|---|---|---|---|---|---|---|---|---|
| 7 | novel odorant receptor [Danio rerio] | gi\|220679255 | 94990.8 | 8.66 | 13 | 40 | 0 | |

Peptide Information

| Calc. Mass | Obsrv. Mass | ± da | ± ppm | Start Seq. | End Seq. | Sequence | Ion Score | C. I. % | Modification | Rank | Result Type |
|---|---|---|---|---|---|---|---|---|---|---|---|
| 864.4243 | 864.4924 | 0.0681 | 79 | 25 | 33 | VSVGNASCK | | | | | Mascot |
| 973.4295 | 973.4905 | 0.061 | 63 | 114 | 121 | IYDTCSSK | | | Carbamidomethyl (C)[5] | | Mascot |
| 973.4295 | 973.4905 | 0.061 | 63 | 114 | 121 | IYDTCSSK | | | Carbamidomethyl (C)[5] | | Mascot |
| 1051.6219 | 1051.6892 | 0.0673 | 64 | 666 | 674 | TLLVLMAFK | | | Oxidation (M)[6] | | Mascot |
| 1104.5031 | 1104.5543 | 0.0512 | 46 | 720 | 727 | NMKYYEEK | | | | | Mascot |
| 1213.5405 | 1213.5719 | 0.0314 | 26 | 244 | 253 | EGICVEYSEK | | | Carbamidomethyl (C)[4] | | Mascot |
| 1434.8202 | 1434.7355 | -0.0847 | -59 | 101 | 113 | SESLLPKLSLGYK | | | | | Mascot |
| 1567.869 | 1567.8163 | -0.0527 | -34 | 420 | 433 | DLLTQPQQVVDALK | | | | | Mascot |
| 1729.8359 | 1729.7338 | -0.1021 | -59 | 173 | 188 | IPVISHAASCECLSSR | | | Carbamidomethyl (C)[10] | | Mascot |
| 2048.1646 | 2047.9043 | -0.2603 | -127 | 666 | 684 | TLLVLMAFKATLPGSNIM K | | | | | Mascot |
| 2279.1853 | 2278.9968 | -0.1885 | -83 | 361 | 379 | NDSWVILNCSRYQDLLVL K | | | | | Mascot |
| 2283.2239 | 2283.1292 | -0.0947 | -41 | 824 | 842 | CYIILLKPEQNTKQYMLG K | | | | | Mascot |
| 2371.1809 | 2371.0811 | -0.0998 | -42 | 413 | 433 | EQEGCEKDLLTQPQQVV DALK | | | | | Mascot |
| 2371.1809 | 2371.0811 | -0.0998 | -42 | 413 | 433 | EQEGCEKDLLTQPQQVV DALK | | | | | Mascot |
| 3337.5483 | 3337.6785 | 0.1302 | 39 | 214 | 243 | HLGWSWVGAVNSDNDY GNYGMAIFLNTAHK | | | | | Mascot |

| | | | | | | | | |
|---|---|---|---|---|---|---|---|---|
| 8 | novel protein similar to Janus kinase family (a protein tyrosine kinase, leukocyte) [Danio rerio] | gi\|169145296 | 125997.7 | 6.83 | 15 | 40 | 0 | |

Peptide Information

| Calc. Mass | Obsrv. Mass | ± da | ± ppm | Start Seq. | End Seq. | Sequence | Ion Score | C. I. % | Modification | Rank | Result Type |
|---|---|---|---|---|---|---|---|---|---|---|---|
| 803.437 | 803.3616 | -0.0754 | -94 | 538 | 544 | GSKIDQR | | | | | Mascot |
| 850.4603 | 850.5243 | 0.064 | 75 | 399 | 405 | NGMFLLR | | | | | Mascot |
| 856.3981 | 856.5068 | 0.1087 | 127 | 882 | 889 | GICYSTGR | | | | | Mascot |
| 864.4825 | 864.4924 | 0.0099 | 11 | 959 | 966 | IADFGLTK | | | | | Mascot |
| 1058.5629 | 1058.5662 | 0.0033 | 3 | 115 | 123 | ASYRFSLSK | | | | | Mascot |
| 1179.5277 | 1179.5769 | 0.0492 | 42 | 806 | 814 | QQEDVFEER | | | | | Mascot |
| 1316.6692 | 1316.6663 | -0.0029 | -2 | 1088 | 1098 | DLIENSLLDER | | | | | Mascot |
| 1475.7094 | 1475.7168 | 0.0074 | 5 | 308 | 319 | RLCQEQMPLEGR | | | Oxidation (M)[7] | | Mascot |
| 2001.0685 | 2000.9335 | -0.135 | -67 | 666 | 684 | LSDPGVSMSLLGKDVVL DR | | | | | Mascot |
| 2118.1416 | 2117.9324 | -0.2092 | -99 | 597 | 614 | NIMVQEFVKHGALDLYLK | | | | | Mascot |
| 2134.1365 | 2133.9275 | -0.209 | -98 | 597 | 614 | NIMVQEFVKHGALDLYLK | | | Oxidation (M)[3] | | Mascot |
| 2193.1108 | 2193.052 | -0.0588 | -27 | 658 | 678 | EGDSPFIKLSDPGVSMSL LGK | | | Oxidation (M)[16] | | Mascot |
| 2211.0791 | 2211.0537 | -0.0254 | -11 | 290 | 307 | QEWQTFCDFPQIIDISIK | | | | | Mascot |
| 2225.0115 | 2225.0662 | 0.0547 | 25 | 564 | 582 | NCWESLFEAASLMSQIS HR | | | Oxidation (M)[13] | | Mascot |
| 2225.0115 | 2225.0662 | 0.0547 | 25 | 564 | 582 | NCWESLFEAASLMSQIS HR | | | Oxidation (M)[13] | | Mascot |
| 2247.1333 | 2247.0203 | -0.113 | -50 | 920 | 938 | LLLFASQICKGMEYLQSM R | | | Oxidation (M)[12] | | Mascot |
| 2263.1284 | 2263.0032 | -0.1252 | -55 | 920 | 938 | LLLFASQICKGMEYLQSM R | | | Oxidation (M)[12,18] | | Mascot |
| 2367.1802 | 2367.2065 | 0.0263 | 11 | 290 | 308 | QEWQTFCDFPQIIDISIKR | | | | | Mascot |

9 BSD domain-containing protein 1 isoform 2 [Danio rerio] gi|147900977 25132 4.28 7 40 0

Peptide Information

| Calc. Mass | Obsrv. Mass | ± da | ± ppm | Start Seq. | End Seq. | Sequence | Ion Score | C. I. % | Modification | Rank | Result Type |
|---|---|---|---|---|---|---|---|---|---|---|---|
| 1213.6211 | 1213.5719 | -0.0492 | -41 | 24 | 33 | SAEAYEFIKR | | | | | Mascot |
| 1563.7068 | 1563.8031 | 0.0963 | 62 | 153 | 165 | EETAREDMAQDLR | | | | | Mascot |
| 1563.7068 | 1563.8031 | 0.0963 | 62 | 153 | 165 | EETAREDMAQDLR | | | | | Mascot |
| 1880.8984 | 1880.9956 | 0.0972 | 52 | 166 | 183 | VFELNSDSGKSTPSNNG K | | | | | Mascot |
| 2000.8827 | 2000.9335 | 0.0508 | 25 | 197 | 213 | DFDLDMTEEEVQMALSK | | | | | Mascot |
| 2053.9495 | 2053.9241 | -0.0254 | -12 | 158 | 175 | EDMAQDLRVFELNSDSG K | | | | | Mascot |
| 2439.1362 | 2439.0789 | -0.0573 | -23 | 2 | 23 | ADGEGGWWGGWLTQSF QSVKDK | | | | | Mascot |
| 3337.4246 | 3337.6785 | 0.2539 | 76 | 185 | 213 | GSSTDVSEDWEKDFDLD MTEEEVQMALSK | | | Oxidation (M)[18] | | Mascot |

| | | | | | | | |
|---|---|---|---|---|---|---|---|
| 10 | PREDICTED: LOW QUALITY PROTEIN: tyrosine-protein kinase JAK2 [Danio rerio] | gi\|326669956 | 130087.7 | 6.55 | 15 | 39 | 0 |

Peptide Information

| Calc. Mass | Obsrv. Mass | ± da | ± ppm | Start Seq. | End Seq. | Sequence | Ion Score | C. I. % | Modification | Rank | Result Type |
|---|---|---|---|---|---|---|---|---|---|---|---|
| 803.437 | 803.3616 | -0.0754 | -94 | 538 | 544 | GSKIDQR | | | | | Mascot |
| 850.4603 | 850.5243 | 0.064 | 75 | 399 | 405 | NGMFLLR | | | | | Mascot |
| 856.3981 | 856.5068 | 0.1087 | 127 | 882 | 889 | GICYSTGR | | | | | Mascot |
| 864.4825 | 864.4924 | 0.0099 | 11 | 959 | 966 | IADFGLTK | | | | | Mascot |
| 1058.5629 | 1058.5662 | 0.0033 | 3 | 115 | 123 | ASYRFSLSK | | | | | Mascot |
| 1179.5277 | 1179.5769 | 0.0492 | 42 | 806 | 814 | QQEDVFEER | | | | | Mascot |
| 1316.6692 | 1316.6663 | -0.0029 | -2 | 1093 | 1103 | DLIENSLLDER | | | | | Mascot |
| 1475.7094 | 1475.7168 | 0.0074 | 5 | 308 | 319 | RLCQEQMPLEGR | | | Oxidation (M)[7] | | Mascot |
| 2001.0685 | 2000.9335 | -0.135 | -67 | 666 | 684 | LSDPGVSMSLLGKDVVL DR | | | | | Mascot |
| 2118.1416 | 2117.9324 | -0.2092 | -99 | 597 | 614 | NIMVQEFVKHGALDLYLK | | | | | Mascot |
| 2134.1365 | 2133.9275 | -0.209 | -98 | 597 | 614 | NIMVQEFVKHGALDLYLK | | | Oxidation (M)[3] | | Mascot |
| 2193.1108 | 2193.052 | -0.0588 | -27 | 658 | 678 | EGDSPFIKLSDPGVSMSL LGK | | | Oxidation (M)[16] | | Mascot |
| 2211.0791 | 2211.0537 | -0.0254 | -11 | 290 | 307 | QEWQTFCDFPQIIDISIK | | | | | Mascot |
| 2225.0115 | 2225.0662 | 0.0547 | 25 | 564 | 582 | NCWESLFEAASLMSQIS HR | | | Oxidation (M)[13] | | Mascot |
| 2225.0115 | 2225.0662 | 0.0547 | 25 | 564 | 582 | NCWESLFEAASLMSQIS HR | | | Oxidation (M)[13] | | Mascot |
| 2247.1333 | 2247.0203 | -0.113 | -50 | 920 | 938 | LLLFASQICKGMEYLQSM R | | | Oxidation (M)[12] | | Mascot |
| 2263.1284 | 2263.0032 | -0.1252 | -55 | 920 | 938 | LLLFASQICKGMEYLQSM R | | | Oxidation (M)[12,18] | | Mascot |
| 2367.1802 | 2367.2065 | 0.0263 | 11 | 290 | 308 | QEWQTFCDFPQIIDISIKR | | | | | Mascot |

| | | | | | | | |
|---|---|---|---|---|---|---|---|
| 11 | PREDICTED: paxillin-like [Danio rerio] | gi\|326669829 | 58290.7 | 5.95 | 10 | 37 | 0 |

Peptide Information

| Calc. Mass | Obsrv. Mass | ± da | ± ppm | Start Seq. | End Seq. | Sequence | Ion Score | C. I. % | Modification | Rank | Result Type |
|---|---|---|---|---|---|---|---|---|---|---|---|
| 1584.7284 | 1584.8403 | 0.1119 | 71 | 244 | 256 | ELEELMACLSDFK | | | Carbamidomethyl (C)[8] | | Mascot |
| 1584.7284 | 1584.8403 | 0.1119 | 71 | 244 | 256 | ELEELMACLSDFK | | | Carbamidomethyl (C)[8] | | Mascot |
| 1729.7672 | 1729.7338 | -0.0334 | -19 | 516 | 529 | EQNDKPYCQGCFIK | | | Carbamidomethyl (C)[8] | | Mascot |
| 1880.9396 | 1880.9956 | 0.056 | 30 | 257 | 274 | VQSNIMAQGKGPHGQAN K | | | Oxidation (M)[6] | | Mascot |
| 2118.041 | 2117.9324 | -0.1086 | -51 | 495 | 511 | FHPEHFVCAFCLKQLNK | | | Carbamidomethyl (C)[8] | | Mascot |
| 2133.9558 | 2133.9275 | -0.0283 | -13 | 341 | 357 | EGQPYCERDYHHLFSPR | | | | | Mascot |
| 2225.0657 | 2225.0662 | 0.0005 | 0 | 473 | 493 | GSLCSGCQKPITGRCITA MGK | | | Carbamidomethyl (C)[4,7] | | Mascot |

| | | | | | | | | | | | |
|---|---|---|---|---|---|---|---|---|---|---|---|
| 2225.0657 | 2225.0662 | 0.0005 | 0 | 473 | 493 | GSLCSGCQKPITGRCITA MGK | | | Carbamidomethyl (C)[4,7] | | Mascot |
| 2263.0261 | 2263.0032 | -0.0229 | -10 | 86 | 105 | DSSPPASRTEEDHVYSF PNK | | | | | Mascot |
| 2297.1118 | 2297.1343 | 0.0225 | 10 | 3 | 23 | FAFCTDALLADLESSTAHI SK | | | Carbamidomethyl (C)[4] | | Mascot |
| 2298.082 | 2298.1377 | 0.0557 | 24 | 473 | 493 | GSLCSGCQKPITGRCITA MGK | | | Carbamidomethyl (C)[4,7,15], Oxidation (M)[19] | | Mascot |
| 2367.27 | 2367.2065 | -0.0635 | -27 | 275 | 296 | LDNMLGSLQSDLHKLGV QTVAK | | | | | Mascot |
| 2657.262 | 2657.1265 | -0.1355 | -51 | 244 | 266 | ELEELMACLSDFKVQSNI MAQGK | | | Carbamidomethyl (C)[8], Oxidation (M)[6] | | Mascot |

| | | | | | | | |
|---|---|---|---|---|---|---|---|
| 12 | PREDICTED: collagen alpha-4(VI) chain-like [Danio rerio] | gi\|326673140 | 38361.2 | 5.51 | 9 | 37 | 0 |

Peptide Information

| Calc. Mass | Obsrv. Mass | ± da | ± ppm | Start Seq. | End Seq. | Sequence | Ion Score | C. I. % | Modification | Rank | Result Type |
|---|---|---|---|---|---|---|---|---|---|---|---|
| 864.3879 | 864.4924 | 0.1045 | 121 | 340 | 346 | RAMQVDE | | | Oxidation (M)[3] | | Mascot |
| 870.4791 | 870.5187 | 0.0396 | 45 | 114 | 122 | GSSGIKGHK | | | | | Mascot |
| 1213.4871 | 1213.5719 | 0.0848 | 70 | 171 | 182 | KNCACCSGNGTR | | | | | Mascot |
| 1316.7169 | 1316.6663 | -0.0506 | -38 | 254 | 265 | QSLLEALNTTAR | | | | | Mascot |
| 1584.6451 | 1584.8403 | 0.1952 | 123 | 84 | 95 | MEEMDMDRLDQR | | | Oxidation (M)[1] | | Mascot |
| 1584.6451 | 1584.8403 | 0.1952 | 123 | 84 | 95 | MEEMDMDRLDQR | | | Oxidation (M)[1] | | Mascot |
| 1729.8789 | 1729.7338 | -0.1451 | -84 | 232 | 246 | VSVMSYSNESKTLIR | | | Oxidation (M)[4] | | Mascot |
| 2047.9827 | 2047.9043 | -0.0784 | -38 | 153 | 170 | GVPGISPMPECDLVEYIR | | | Carbamidomethyl (C)[11], Oxidation (M)[8] | | Mascot |
| 2118.1062 | 2117.9324 | -0.1738 | -82 | 120 | 140 | GHKGYQGRPGNAGRPG NPGIK | | | | | Mascot |
| 2371.1235 | 2371.0811 | -0.0424 | -18 | 210 | 231 | SAAYSLLEDIFISDTNCPQ GAR | | | | | Mascot |
| 2371.1235 | 2371.0811 | -0.0424 | -18 | 210 | 231 | SAAYSLLEDIFISDTNCPQ GAR | | | | | Mascot |

| | | | | | | | |
|---|---|---|---|---|---|---|---|
| 13 | DNA mismatch repair protein Msh3 [Danio rerio] | gi\|157841276 | 121710.5 | 6.91 | 15 | 36 | 0 |

Peptide Information

| Calc. Mass | Obsrv. Mass | ± da | ± ppm | Start Seq. | End Seq. | Sequence | Ion Score | C. I. % | Modification | Rank | Result Type |
|---|---|---|---|---|---|---|---|---|---|---|---|
| 842.4981 | 842.4913 | -0.0068 | -8 | 150 | 156 | EEPIKVK | | | | | Mascot |
| 870.4727 | 870.5187 | 0.046 | 53 | 1 | 7 | MHTAVRR | | | | | Mascot |
| 902.5458 | 902.4664 | -0.0794 | -88 | 1060 | 1066 | LVTQWKK | | | | | Mascot |
| 973.5465 | 973.4905 | -0.056 | -58 | 288 | 295 | QLHALYTK | | | | | Mascot |
| 973.5465 | 973.4905 | -0.056 | -58 | 288 | 295 | QLHALYTK | | | | | Mascot |
| 1106.5437 | 1106.5333 | -0.0104 | -9 | 55 | 65 | LSDGGASTDKR | | | | | Mascot |
| 1179.5535 | 1179.5769 | 0.0234 | 20 | 895 | 905 | MGASDNISRGR | | | Oxidation (M)[1] | | Mascot |
| 1234.5739 | 1234.6538 | 0.0799 | 65 | 157 | 166 | QEFSFSQYAK | | | | | Mascot |

| | | | | | | | | | | | |
|---|---|---|---|---|---|---|---|---|---|---|---|
| 1325.7899 | 1325.7146 | -0.0753 | -57 | 95 | 106 | VSISHATLTRLK | | | | | Mascot |
| 1325.7899 | 1325.7146 | -0.0753 | -57 | 95 | 106 | VSISHATLTRLK | | | | | Mascot |
| 1475.7562 | 1475.7168 | -0.0394 | -27 | 713 | 725 | NSMLSIVPADWVK | | | Oxidation (M)[3] | | Mascot |
| 1728.8473 | 1728.7454 | -0.1019 | -59 | 672 | 685 | EEIESVLLDIMEHR | | | Oxidation (M)[11] | | Mascot |
| 2001.1127 | 2000.9335 | -0.1792 | -90 | 1021 | 1038 | SYGLNVARLAEIPESILR | | | | | Mascot |
| 2263.1396 | 2263.0032 | -0.1364 | -60 | 778 | 797 | KAVCHLATMDCLFSLAQ VAK | | | Carbamidomethyl (C)[4,11] | | Mascot |
| 2279.1345 | 2278.9968 | -0.1377 | -60 | 778 | 797 | KAVCHLATMDCLFSLAQ VAK | | | Carbamidomethyl (C)[4,11], Oxidation (M)[9] | | Mascot |
| 2297.1157 | 2297.1343 | 0.0186 | 8 | 415 | 433 | ESAMFEYPTALSIIKDFYR | | | Oxidation (M)[4] | | Mascot |
| 2439.1968 | 2439.0789 | -0.1179 | -48 | 340 | 361 | ELTVGMVVVQPSIGDVM VDCFK | | | Carbamidomethyl (C)[20], Oxidation (M)[6] | | Mascot |
| 2455.1917 | 2455.083 | -0.1087 | -44 | 340 | 361 | ELTVGMVVVQPSIGDVM VDCFK | | | Carbamidomethyl (C)[20], Oxidation (M)[6,17] | | Mascot |
| 3218.7056 | 3218.5154 | -0.1902 | -59 | 926 | 955 | SLVILDELGRGTSTHDGIA IAYATLESFIR | | | | | Mascot |
| 3218.7056 | 3218.5154 | -0.1902 | -59 | 926 | 955 | SLVILDELGRGTSTHDGIA IAYATLESFIR | | | | | Mascot |

| | | | | | | | |
|---|---|---|---|---|---|---|---|
| 14 | PREDICTED: microtubule-associated protein 6-like [Danio rerio] | gi\|292621423 | 42669 | 9.6 | 9 | 36 | 0 |

**Peptide Information**

| Calc. Mass | Obsrv. Mass | ± da | ± ppm | Start Seq. | End Seq. | Sequence | Ion Score | C. I. % | Modification | Rank | Result Type |
|---|---|---|---|---|---|---|---|---|---|---|---|
| 864.421 | 864.4924 | 0.0714 | 83 | 113 | 119 | NQYQPSK | | | | | Mascot |
| 973.5173 | 973.4905 | -0.0268 | -28 | 220 | 228 | RSAADALNR | | | | | Mascot |
| 973.5173 | 973.4905 | -0.0268 | -28 | 220 | 228 | RSAADALNR | | | | | Mascot |
| 1165.7052 | 1165.7196 | 0.0144 | 12 | 317 | 326 | HNLSLTKPKK | | | | | Mascot |
| 1179.6368 | 1179.5769 | -0.0599 | -51 | 300 | 309 | IRSLYSEPSK | | | | | Mascot |
| 1234.5984 | 1234.6538 | 0.0554 | 45 | 201 | 211 | EPVSEASKMEK | | | | | Mascot |
| 1316.644 | 1316.6663 | 0.0223 | 17 | 282 | 293 | GEQAKPQTTDNK | | | | | Mascot |
| 1563.7341 | 1563.8031 | 0.069 | 44 | 2 | 14 | AWPCITRACCINR | | | Carbamidomethyl (C)[4] | | Mascot |
| 1563.7341 | 1563.8031 | 0.069 | 44 | 2 | 14 | AWPCITRACCINR | | | Carbamidomethyl (C)[4] | | Mascot |
| 1584.7297 | 1584.8403 | 0.1106 | 70 | 9 | 21 | ACCINRFWSELDK | | | | | Mascot |
| 1584.7297 | 1584.8403 | 0.1106 | 70 | 9 | 21 | ACCINRFWSELDK | | | | | Mascot |
| 2001.0538 | 2000.9335 | -0.1203 | -60 | 264 | 281 | LLVGEKTNLETSYSATFK | | | | | Mascot |

| | | | | | | | |
|---|---|---|---|---|---|---|---|
| 15 | PREDICTED: hypothetical protein LOC559994 [Danio rerio] | gi\|326677062 | 1030064.3 | 5.62 | 46 | 36 | 0 |

**Peptide Information**

| Calc. Mass | Obsrv. Mass | ± da | ± ppm | Start Seq. | End Seq. | Sequence | Ion Score | C. I. % | Modification | Rank | Result Type |
|---|---|---|---|---|---|---|---|---|---|---|---|

| | | | | | | | | | | |
|---|---|---|---|---|---|---|---|---|---|---|
| 850.5185 | 850.5243 | 0.0058 | 7 | 8569 | 8575 | IKPFAFK | | | | Mascot |
| 856.5138 | 856.5068 | -0.007 | -8 | 1082 | 1089 | LGIIEPSK | | | | Mascot |
| 864.4321 | 864.4924 | 0.0603 | 70 | 3272 | 3278 | EERAFGR | | | | Mascot |
| 870.5559 | 870.5187 | -0.0372 | -43 | 1536 | 1542 | ALLLWVR | | | | Mascot |
| 882.4679 | 882.5473 | 0.0794 | 90 | 3102 | 3108 | NLHEELK | | | | Mascot |
| 902.4512 | 902.4664 | 0.0152 | 17 | 4824 | 4831 | TVSISHCR | | | | Mascot |
| 973.5312 | 973.4905 | -0.0407 | -42 | 3727 | 3735 | AKDAALEQK | | | | Mascot |
| 973.5789 | 973.4905 | -0.0884 | -91 | 8219 | 8226 | IQSTQKIR | 1 | 0 | | Mascot |
| 1045.5636 | 1045.5409 | -0.0227 | -22 | 8277 | 8285 | DLEVRSAQK | | | | Mascot |
| 1058.584 | 1058.5662 | -0.0178 | -17 | 6340 | 6349 | AADLAVLTER | | | | Mascot |
| 1082.5551 | 1082.4855 | -0.0696 | -64 | 8651 | 8659 | MQYISALEK | | | | Mascot |
| 1179.5674 | 1179.5769 | 0.0095 | 8 | 2826 | 2835 | DMDLLSQSVR | | | Oxidation (M)[2] | Mascot |
| 1213.6648 | 1213.5719 | -0.0929 | -77 | 69 | 78 | IAELAQQERR | | | | Mascot |
| 1234.6348 | 1234.6538 | 0.019 | 15 | 3360 | 3370 | EQCASLTELLK | | | | Mascot |
| 1316.6593 | 1316.6663 | 0.007 | 5 | 3905 | 3916 | ISHTSIYHGSSK | | | | Mascot |
| 1325.7899 | 1325.7146 | -0.0753 | -57 | 4060 | 4071 | AKTLQKPQNVAK | | | | Mascot |
| 1325.7899 | 1325.7146 | -0.0753 | -57 | 4060 | 4071 | AKTLQKPQNVAK | | | | Mascot |
| 1347.7123 | 1347.6968 | -0.0155 | -12 | 7311 | 7322 | LAMVMEKSGPLR | | | Oxidation (M)[3] | Mascot |
| 1434.8467 | 1434.7355 | -0.1112 | -77 | 8569 | 8580 | IKPFAFKLLSDR | | | | Mascot |
| 1475.7522 | 1475.7168 | -0.0354 | -24 | 6801 | 6813 | QEAKILECSVAER | | | | Mascot |
| 1638.9438 | 1638.8247 | -0.1191 | -73 | 3297 | 3310 | HVLKEPTLPALQHR | | | | Mascot |
| 1648.7847 | 1648.7839 | -0.0008 | 0 | 7781 | 7796 | MPDTESAKPGKDSGTK | | | | Mascot |
| 1728.7395 | 1728.7454 | 0.0059 | 3 | 8383 | 8396 | IHDVEDQWQACGDR | | | Carbamidomethyl (C)[11] | Mascot |
| 1729.869 | 1729.7338 | -0.1352 | -78 | 718 | 732 | SGNRFMLVVTDYATR | | | | Mascot |
| 2000.9818 | 2000.9335 | -0.0483 | -24 | 61 | 77 | MEAQEAQRIAELAQQER | | | | Mascot |
| 2047.9866 | 2047.9043 | -0.0823 | -40 | 7014 | 7030 | DQTVHLSHHEEMSQLLK | | | Oxidation (M)[12] | Mascot |
| 2054.0037 | 2053.9241 | -0.0796 | -39 | 7370 | 7387 | IAELEGEVQDLAEQQPER | | | | Mascot |
| 2118.0496 | 2117.9324 | -0.1172 | -55 | 3987 | 4005 | SSSSKTQEALQHNISEIMK | | | | Mascot |
| 2134.0444 | 2133.9275 | -0.1169 | -55 | 3987 | 4005 | SSSSKTQEALQHNISEIMK | | | Oxidation (M)[18] | Mascot |
| 2193.0247 | 2193.052 | 0.0273 | 12 | 8962 | 8978 | DFEWTEEQLQLHEGLYR | | | | Mascot |
| 2211.126 | 2211.0537 | -0.0723 | -33 | 8508 | 8526 | MSAAELQTTGVIYRLELCR | | | Carbamidomethyl (C)[18] | Mascot |
| 2224.9873 | 2225.0662 | 0.0789 | 35 | 4312 | 4333 | VTDAETSSQIGACAEDNESGIK | | | | Mascot |
| 2224.9873 | 2225.0662 | 0.0789 | 35 | 4312 | 4333 | VTDAETSSQIGACAEDNESGIK | | | | Mascot |
| 2233.0012 | 2233.0288 | 0.0276 | 12 | 8235 | 8252 | DSTELEEWMRCAHEQLQK | | | | Mascot |
| 2239.1262 | 2239.0825 | -0.0437 | -20 | 8399 | 8415 | LLRCIHSWISQHMEWVK | | | Carbamidomethyl (C)[4], Oxidation (M)[13] | Mascot |

| | | | | | | | | | | | |
|---|---|---|---|---|---|---|---|---|---|---|---|
| 2247.0305 | 2247.0203 | -0.0102 | -5 | 8357 | 8376 | RLEDESSGVHCALDSSE LSR | | | Carbamidomethyl (C)[11] | | Mascot |
| 2263.124 | 2263.0032 | -0.1208 | -53 | 150 | 168 | LEKLSDGDDVEHFLITFE R | | | | | Mascot |
| 2283.1333 | 2283.1292 | -0.0041 | -2 | 7195 | 7214 | ESPQICQDLQSLLGHCRS LR | | | | | Mascot |
| 2284.022 | 2284.1206 | 0.0986 | 43 | 3439 | 3458 | RVLDCTDAPFESEADTL MDR | | | | | Mascot |
| 2297.1455 | 2297.1343 | -0.0112 | -5 | 8621 | 8639 | VLLNQHWERLGELMNSS DR | | | | | Mascot |
| 2298.2559 | 2298.1377 | -0.1182 | -51 | 8671 | 8691 | VMEASKLLIGQMQPQSA PLIK | | | Oxidation (M)[2] | | Mascot |
| 2300.0168 | 2300.126 | 0.1092 | 47 | 3439 | 3458 | RVLDCTDAPFESEADTL MDR | | | Oxidation (M)[18] | | Mascot |
| 2367.1311 | 2367.2065 | 0.0754 | 32 | 1497 | 1520 | SSSLDSLSSLDSFSGSPA SSPVPR | | | | | Mascot |
| 2439.1489 | 2439.0789 | -0.07 | -29 | 8630 | 8650 | LGELMNSSDREENTVEM LISR | | | Oxidation (M)[5] | | Mascot |
| 2455.1438 | 2455.083 | -0.0608 | -25 | 8630 | 8650 | LGELMNSSDREENTVEM LISR | | | Oxidation (M)[5,17] | | Mascot |
| 2532.1858 | 2532.2136 | 0.0278 | 11 | 8164 | 8183 | LHKILEDTCSQVWETEDC LR | | | Carbamidomethyl (C)[9,18] | | Mascot |
| 2580.2974 | 2580.2556 | -0.0418 | -16 | 6048 | 6071 | AECITQTPTVIPSDLQLSH AEAQK | | | | | Mascot |
| 2580.2974 | 2580.2556 | -0.0418 | -16 | 6048 | 6071 | AECITQTPTVIPSDLQLSH AEAQK | | | | | Mascot |
| 2596.187 | 2596.2539 | 0.0669 | 26 | 4718 | 4742 | HTSAESSNEANALQNPP NGGEDTKK | | | | | Mascot |
| 2596.2888 | 2596.2539 | -0.0349 | -13 | 6613 | 6634 | WVIEELLHSQESSAEEQN VIQK | | | | | Mascot |
| 2657.3967 | 2657.1265 | -0.2702 | -102 | 570 | 592 | LPVNIVQMQQEDASLVPY LERAK | | | Oxidation (M)[8] | | Mascot |
| 3227.575 | 3227.5264 | -0.0486 | -15 | 2132 | 2160 | AASLLEGRLAELENWSTE AQEVCQHLSSR | | | | | Mascot |

| | | | | | | | |
|---|---|---|---|---|---|---|---|
| 16 | septin 5a [Danio rerio] | gi\|41152396 | 43011.6 | 5.85 | 10 | 36 | 0 |

**Peptide Information**

| Calc. Mass | Obsrv. Mass | ± da | ± ppm | Start Seq. | End Seq. | Sequence | Ion Score | C. I. % | Modification | Rank | Result Type |
|---|---|---|---|---|---|---|---|---|---|---|---|
| 996.6451 | 996.5773 | -0.0678 | -68 | 180 | 188 | VNIIPLISK | | | | | Mascot |
| 1045.5889 | 1045.5409 | -0.048 | -46 | 80 | 88 | INQTVEITK | | | | | Mascot |
| 1106.584 | 1106.5333 | -0.0507 | -46 | 206 | 214 | DEIERFGIK | | | | | Mascot |
| 1844.8741 | 1844.8348 | -0.0393 | -21 | 307 | 322 | AHCIQEMTSKLAQDNR | | | | | Mascot |
| 1844.8741 | 1844.8348 | -0.0393 | -21 | 307 | 322 | AHCIQEMTSKLAQDNR | | | | | Mascot |
| 1993.8008 | 1993.9235 | 0.1227 | 62 | 215 | 230 | VYQFPECDSDEEEEFK | | | | | Mascot |
| 2054.1565 | 2053.9241 | -0.2324 | -113 | 180 | 198 | VNIIPLISKADCLTPTEVK | | | | | Mascot |
| 2134.0708 | 2133.9275 | -0.1433 | -67 | 238 | 257 | ECTPFAVIGSNTVVEARG QR | | | | | Mascot |
| 2263.0852 | 2263.0032 | -0.082 | -36 | 262 | 280 | LYPWGIVEVENQSHCDF VK | | | | | Mascot |
| 2439.0696 | 2439.0789 | 0.0093 | 4 | 211 | 230 | FGIKVYQFPECDSDEEEE FK | | | | | Mascot |
| 2532.2703 | 2532.2136 | -0.0567 | -22 | 262 | 282 | LYPWGIVEVENQSHCDF | | | | | Mascot |

VKLR

| | | | | | | | |
|---|---|---|---|---|---|---|---|
| 17 | growth/differentiation factor 9 [Danio rerio] | gi\|59933270 | 48701.4 | 8.77 | 10 | 35 | 0 |

Peptide Information

| Calc. Mass | Obsrv. Mass | ± da | ± ppm | Start Seq. | End Seq. | Sequence | Ion Score | C. I. % | Modification | Rank | Result Type |
|---|---|---|---|---|---|---|---|---|---|---|---|
| 850.5032 | 850.5243 | 0.0211 | 25 | 324 | 330 | VSFKELK | | | | | Mascot |
| 1082.5122 | 1082.4855 | -0.0267 | -25 | 8 | 16 | CSALCYLPR | | | Carbamidomethyl (C)[1] | | Mascot |
| 1993.9696 | 1993.9235 | -0.0461 | -23 | 1 | 16 | MATLFFKCSALCYLPR | | | Carbamidomethyl (C)[8,12], Oxidation (M)[1] | | Mascot |
| 2047.9655 | 2047.9043 | -0.0612 | -30 | 116 | 131 | QNREFFMQDISYSLNR | | | | | Mascot |
| 2225.156 | 2225.0662 | -0.0898 | -40 | 246 | 264 | SPSLLLYLNDTSEVAYQR R | | | | | Mascot |
| 2225.156 | 2225.0662 | -0.0898 | -40 | 246 | 264 | SPSLLLYLNDTSEVAYQR R | | | | | Mascot |
| 2247.2285 | 2247.0203 | -0.2082 | -93 | 195 | 213 | WVEVDVTSFLHPLIQAHK K | | | | | Mascot |
| 2298.1118 | 2298.1377 | 0.0259 | 11 | 171 | 190 | DQMCSNHLGIQHSVPLL SFR | | | Oxidation (M)[3] | | Mascot |
| 2299.9885 | 2300.126 | 0.1375 | 60 | 306 | 323 | LVPMYEFTTDDCDLYDFR | | | Carbamidomethyl (C)[12] | | Mascot |
| 2657.2922 | 2657.1265 | -0.1657 | -62 | 168 | 190 | SSKDQMCSNHLGIQHSV PLLSFR | | | Carbamidomethyl (C)[7], Oxidation (M)[6] | | Mascot |
| 3227.5903 | 3227.5264 | -0.0639 | -20 | 141 | 167 | SVLLYSFDHNHLSPFSLL CYLDMKEQK | | | | | Mascot |

| | | | | | | | |
|---|---|---|---|---|---|---|---|
| 18 | nucleoside diphosphate-linked moiety X motif 6 [Danio rerio] | gi\|148237304 | 36698.5 | 7.22 | 8 | 35 | 0 |

Peptide Information

| Calc. Mass | Obsrv. Mass | ± da | ± ppm | Start Seq. | End Seq. | Sequence | Ion Score | C. I. % | Modification | Rank | Result Type |
|---|---|---|---|---|---|---|---|---|---|---|---|
| 850.4893 | 850.5243 | 0.035 | 41 | 243 | 249 | LQPLSHR | | | | | Mascot |
| 1325.6882 | 1325.7146 | 0.0264 | 20 | 59 | 70 | LCPPLLAGDVDR | | | Carbamidomethyl (C)[2] | | Mascot |
| 1325.6882 | 1325.7146 | 0.0264 | 20 | 59 | 70 | LCPPLLAGDVDR | | | Carbamidomethyl (C)[2] | | Mascot |
| 1584.7653 | 1584.8403 | 0.075 | 47 | 205 | 217 | EVFEETGVRSEFR | | | | | Mascot |
| 1584.7653 | 1584.8403 | 0.075 | 47 | 205 | 217 | EVFEETGVRSEFR | | | | | Mascot |
| 1728.7792 | 1728.7454 | -0.0338 | -20 | 43 | 58 | LVSAGSARFCSGCEER | | | Carbamidomethyl (C)[10] | | Mascot |
| 1880.88 | 1880.9956 | 0.1156 | 61 | 78 | 93 | DFPPDISEEEFSDLLK | | | | | Mascot |
| 2000.9867 | 2000.9335 | -0.0532 | -27 | 2 | 19 | VLWIMSAGFCRVSSGLC R | | | Oxidation (M)[5] | | Mascot |
| 2247.0435 | 2247.0203 | -0.0232 | -10 | 224 | 242 | QQHTHPGAFGMSDLYLI CR | | | Carbamidomethyl (C)[18], Oxidation (M)[11] | | Mascot |
| 2297.0735 | 2297.1343 | 0.0608 | 26 | 250 | 266 | IHICTHECLRCDWLDLR | | | Carbamidomethyl (C)[4,8,11] | | Mascot |

| | | | | | | | |
|---|---|---|---|---|---|---|---|
| 19 | transcription elongation factor B polypeptide 1 [Danio rerio] | gi\|73088652 | 12495 | 4.74 | 5 | 35 | 0 |

Peptide Information

| Calc. Mass | Obsrv. Mass | ± da | ± ppm | Start Seq. | End Seq. | Sequence | Ion Score | C. I. % | Modification | Rank | Result Type |
|---|---|---|---|---|---|---|---|---|---|---|---|
| 1325.6381 | 1325.7146 | 0.0765 | 58 | 73 | 82 | VCMYFTYKVR | | | Oxidation (M)[3] | | Mascot |
| 1325.6381 | 1325.7146 | 0.0765 | 58 | 73 | 82 | VCMYFTYKVR | | | Oxidation (M)[3] | | Mascot |
| 1563.6454 | 1563.8031 | 0.1577 | 101 | 7 | 20 | TYGGCEGPDAMYVK | | | Carbamidomethyl (C)[5], Oxidation (M)[11] | | Mascot |
| 1563.6454 | 1563.8031 | 0.1577 | 101 | 7 | 20 | TYGGCEGPDAMYVK | | | Carbamidomethyl (C)[5], Oxidation (M)[11] | | Mascot |
| 2118.0398 | 2117.9324 | -0.1074 | -51 | 64 | 80 | EIPSHVLSKVCMYFTYK | | | Carbamidomethyl (C)[11], Oxidation (M)[12] | | Mascot |
| 2211.0134 | 2211.0537 | 0.0403 | 18 | 44 | 63 | AMLSGPGQFAENETNEV NFR | | | | | Mascot |
| 2282.925 | 2283.1292 | 0.2042 | 89 | 1 | 20 | MDSEEKTYGGCEGPDA MYVK | | | Carbamidomethyl (C)[11], Oxidation (M)[1] | | Mascot |

| | | | | | | | |
|---|---|---|---|---|---|---|---|
| 20 | PREDICTED: DNA mismatch repair protein Msh3-like [Danio rerio] | gi\|326668008 | 121301.1 | 6.7 | 14 | 34 | 0 |

Peptide Information

| Calc. Mass | Obsrv. Mass | ± da | ± ppm | Start Seq. | End Seq. | Sequence | Ion Score | C. I. % | Modification | Rank | Result Type |
|---|---|---|---|---|---|---|---|---|---|---|---|
| 870.4727 | 870.5187 | 0.046 | 53 | 1 | 7 | MHTAVRR | | | | | Mascot |
| 902.5458 | 902.4664 | -0.0794 | -88 | 1060 | 1066 | LVTQWKK | | | | | Mascot |
| 973.5465 | 973.4905 | -0.056 | -58 | 288 | 295 | QLHALYTK | | | | | Mascot |
| 973.5465 | 973.4905 | -0.056 | -58 | 288 | 295 | QLHALYTK | | | | | Mascot |
| 1106.5437 | 1106.5333 | -0.0104 | -9 | 55 | 65 | LSDGGASTDKR | | | | | Mascot |
| 1179.5535 | 1179.5769 | 0.0234 | 20 | 895 | 905 | MGASDNISRGR | | | Oxidation (M)[1] | | Mascot |
| 1234.5739 | 1234.6538 | 0.0799 | 65 | 157 | 166 | QEFSFSQYAK | | | | | Mascot |
| 1325.7899 | 1325.7146 | -0.0753 | -57 | 95 | 106 | VSISHATLTRLK | | | | | Mascot |
| 1325.7899 | 1325.7146 | -0.0753 | -57 | 95 | 106 | VSISHATLTRLK | | | | | Mascot |
| 1475.7529 | 1475.7168 | -0.0361 | -24 | 155 | 166 | IKQEFSFSQYAK | | | | | Mascot |
| 1728.8473 | 1728.7454 | -0.1019 | -59 | 672 | 685 | EEIESVLLDIMEHR | | | Oxidation (M)[11] | | Mascot |
| 2001.1127 | 2000.9335 | -0.1792 | -90 | 1021 | 1038 | SYGLNVARLAEIPESILR | | | | | Mascot |
| 2263.1396 | 2263.0032 | -0.1364 | -60 | 778 | 797 | KAVCHLATMDCLFSLAQ VAK | | | Carbamidomethyl (C)[4,11] | | Mascot |
| 2279.1345 | 2278.9968 | -0.1377 | -60 | 778 | 797 | KAVCHLATMDCLFSLAQ VAK | | | Carbamidomethyl (C)[4,11], Oxidation (M)[9] | | Mascot |
| 2297.1157 | 2297.1343 | 0.0186 | 8 | 415 | 433 | ESAMFEYPTALSIIKDFYR | | | Oxidation (M)[4] | | Mascot |
| 2439.1968 | 2439.0789 | -0.1179 | -48 | 340 | 361 | ELTVGMVVVQPSIGDVM VDCFK | | | Carbamidomethyl (C)[20], Oxidation (M)[6] | | Mascot |
| 2455.1917 | 2455.083 | -0.1087 | -44 | 340 | 361 | ELTVGMVVVQPSIGDVM VDCFK | | | Carbamidomethyl (C)[20], Oxidation (M)[6,17] | | Mascot |
| 3218.7056 | 3218.5154 | -0.1902 | -59 | 926 | 955 | SLVILDELGRGTSTHDGIA IAYATLESFIR | | | | | Mascot |
| 3218.7056 | 3218.5154 | -0.1902 | -59 | 926 | 955 | SLVILDELGRGTSTHDGIA IAYATLESFIR | | | | | Mascot |

| | | | | | | | |
|---|---|---|---|---|---|---|---|
| 21 | MCM6 minichromosome maintenance deficient 6, like | gi\|70887711 | 93152.1 | 5.23 | 12 | 34 | 0 |

[Danio rerio]

**Peptide Information**

| Calc. Mass | Obsrv. Mass | ± da | ± ppm | Start Seq. | End Seq. | Sequence | Ion Score | C. I. % | Modification | Rank | Result Type |
|---|---|---|---|---|---|---|---|---|---|---|---|
| 856.49 | 856.5068 | 0.0168 | 20 | 87 | 93 | AVRHFAR | | | | | Mascot |
| 973.5789 | 973.4905 | -0.0884 | -91 | 117 | 124 | IRELSTVR | | | | | Mascot |
| 973.5789 | 973.4905 | -0.0884 | -91 | 117 | 124 | IRELSTVR | | | | | Mascot |
| 996.5261 | 996.5773 | 0.0512 | 51 | 194 | 201 | FVDFQKGR | | | | | Mascot |
| 1165.6252 | 1165.7196 | 0.0944 | 81 | 747 | 755 | SELINWYLK | | | | | Mascot |
| 1234.7228 | 1234.6538 | -0.069 | -56 | 361 | 372 | GILLMLFGGVAK | | | Oxidation (M)[5] | | Mascot |
| 1325.7899 | 1325.7146 | -0.0753 | -57 | 212 | 223 | GSIPRSVEVILR | | | | | Mascot |
| 1325.7899 | 1325.7146 | -0.0753 | -57 | 212 | 223 | GSIPRSVEVILR | | | | | Mascot |
| 1347.6508 | 1347.6968 | 0.046 | 34 | 512 | 523 | QNVNMSAPIMSR | | | | | Mascot |
| 1475.7502 | 1475.7168 | -0.0334 | -23 | 90 | 102 | HFARDHGNIPPSK | | | | | Mascot |
| 2211.1875 | 2211.0537 | -0.1338 | -61 | 361 | 381 | GILLMLFGGVAKTTMEGT SLR | | | Oxidation (M)[5] | | Mascot |
| 2239.1096 | 2239.0825 | -0.0271 | -12 | 236 | 257 | CDFTGTLVVVPDVSAMA LAGTR | | | Oxidation (M)[16] | | Mascot |
| 2297.1812 | 2297.1343 | -0.0469 | -20 | 575 | 593 | QFQPKITVEAQEFVVDQY K | | | | | Mascot |
| 2580.2949 | 2580.2556 | -0.0393 | -15 | 138 | 160 | THPVHPELVSGTFLCLDC QSVIK | | | Carbamidomethyl (C)[15] | | Mascot |
| 2580.2949 | 2580.2556 | -0.0393 | -15 | 138 | 160 | THPVHPELVSGTFLCLDC QSVIK | | | Carbamidomethyl (C)[15] | | Mascot |

22 Mbd1 protein [Danio rerio] gi|146187002 109196.7 5.19 13 34 0

**Peptide Information**

| Calc. Mass | Obsrv. Mass | ± da | ± ppm | Start Seq. | End Seq. | Sequence | Ion Score | C. I. % | Modification | Rank | Result Type |
|---|---|---|---|---|---|---|---|---|---|---|---|
| 864.476 | 864.4924 | 0.0164 | 19 | 456 | 462 | LFGVCIR | | | Carbamidomethyl (C)[5] | | Mascot |
| 902.4664 | 902.4664 | 0 | 0 | 494 | 500 | KWVPCGR | | | Carbamidomethyl (C)[5] | | Mascot |
| 973.5135 | 973.4905 | -0.023 | -24 | 874 | 881 | LQPLQCSK | | | Carbamidomethyl (C)[6] | | Mascot |
| 973.5135 | 973.4905 | -0.023 | -24 | 874 | 881 | LQPLQCSK | | | Carbamidomethyl (C)[6] | | Mascot |
| 1045.5524 | 1045.5409 | -0.0115 | -11 | 70 | 78 | KDEEAVVQK | | | | | Mascot |
| 1165.5453 | 1165.7196 | 0.1743 | 150 | 971 | 979 | CEKCQTLQR | | | Carbamidomethyl (C)[1] | | Mascot |
| 1563.5946 | 1563.8031 | 0.2085 | 133 | 626 | 639 | TDCGTCDFCMDKPK | | | | | Mascot |
| 1563.5946 | 1563.8031 | 0.2085 | 133 | 626 | 639 | TDCGTCDFCMDKPK | | | | | Mascot |
| 1728.9629 | 1728.7454 | -0.2175 | -126 | 921 | 936 | LTSAAEIIALIEDLEK | | | | | Mascot |
| 1880.8153 | 1880.9956 | 0.1803 | 96 | 463 | 477 | CNKTYSYEEGQTLCK | | | Carbamidomethyl (C)[1,14] | | Mascot |
| 1993.9574 | 1993.9235 | -0.0339 | -17 | 366 | 385 | SDSADVQRPASSAGFAT AQK | | | | | Mascot |

| | | | | | | | | | | | |
|---|---|---|---|---|---|---|---|---|---|---|---|
| 2000.9672 | 2000.9335 | -0.0337 | -17 | 789 | 807 | RPDVPPTTQFDSTGNPG SK | | | | | Mascot |
| 2053.8235 | 2053.9241 | 0.1006 | 49 | 626 | 643 | TDCGTCDFCMDKPKFGG R | | | Carbamidomethyl (C)[3], Oxidation (M)[10] | | Mascot |
| 2296.9382 | 2297.1343 | 0.1961 | 85 | 695 | 712 | KMWGFEMSDDMPEQYM QK | | | Oxidation (M)[2] | | Mascot |
| 2580.2949 | 2580.2556 | -0.0393 | -15 | 658 | 679 | EAMKHLLPMDEAELLAQ GWLPR | | | Oxidation (M)[3,9] | | Mascot |
| 2580.2949 | 2580.2556 | -0.0393 | -15 | 658 | 679 | EAMKHLLPMDEAELLAQ GWLPR | | | Oxidation (M)[3,9] | | Mascot |

23 hypothetical protein FLJ22318 [Danio rerio] gi|37595352 44472.4 6.24 9 34 0

**Peptide Information**

| Calc. Mass | Obsrv. Mass | ± da | ± ppm | Start Seq. | End Seq. | Sequence | Ion Score | C. I. % | Modification | Rank | Result Type |
|---|---|---|---|---|---|---|---|---|---|---|---|
| 842.4366 | 842.4913 | 0.0547 | 65 | 80 | 87 | DIHGSVSK | | | | | Mascot |
| 1045.4884 | 1045.5409 | 0.0525 | 50 | 306 | 314 | QCSGVWTHK | | | | | Mascot |
| 1106.559 | 1106.5333 | -0.0257 | -23 | 209 | 217 | QQEALQYAR | | | | | Mascot |
| 1567.6913 | 1567.8163 | 0.125 | 80 | 1 | 13 | MEQCACVERELEK | | | | | Mascot |
| 1880.834 | 1880.9956 | 0.1616 | 86 | 371 | 386 | LKCPYCPMEQNPSDAK | | | Carbamidomethyl (C)[3] | | Mascot |
| 2133.908 | 2133.9275 | 0.0195 | 9 | 373 | 390 | CPYCPMEQNPSDAKQIY F | | | | | Mascot |
| 2225.1165 | 2225.0662 | -0.0503 | -23 | 340 | 359 | QQTSESNPPMKLICGHVI SR | | | | | Mascot |
| 2225.1165 | 2225.0662 | -0.0503 | -23 | 340 | 359 | QQTSESNPPMKLICGHVI SR | | | | | Mascot |
| 2298.1328 | 2298.1377 | 0.0049 | 2 | 340 | 359 | QQTSESNPPMKLICGHVI SR | | | Carbamidomethyl (C)[14], Oxidation (M)[10] | | Mascot |
| 2580.2256 | 2580.2556 | 0.03 | 12 | 43 | 65 | LLAYGVQDADLSVLHQT MAQCCK | | | Carbamidomethyl (C)[21], Oxidation (M)[18] | | Mascot |
| 2580.2256 | 2580.2556 | 0.03 | 12 | 43 | 65 | LLAYGVQDADLSVLHQT MAQCCK | | | Carbamidomethyl (C)[21], Oxidation (M)[18] | | Mascot |
| 3338.6252 | 3338.6836 | 0.0584 | 17 | 129 | 158 | QGMLSVAEDLCQSGVVI DMSMKQPFLELNR | | | | | Mascot |

24 PREDICTED: gastrula zinc finger protein XlCGF57.1-like [Danio rerio] gi|326667403 40716.1 9.31 11 34 0

**Peptide Information**

| Calc. Mass | Obsrv. Mass | ± da | ± ppm | Start Seq. | End Seq. | Sequence | Ion Score | C. I. % | Modification | Rank | Result Type |
|---|---|---|---|---|---|---|---|---|---|---|---|
| 803.408 | 803.3616 | -0.0464 | -58 | 340 | 346 | TLSSHMK | | | | | Mascot |
| 1347.6072 | 1347.6968 | 0.0896 | 66 | 76 | 86 | CFECEKVFQSK | | | | | Mascot |
| 1567.8162 | 1567.8163 | 0.0001 | 0 | 250 | 262 | VFSHSLNLNIHMR | | | | | Mascot |
| 1729.8512 | 1729.7338 | -0.1174 | -68 | 325 | 338 | LFTCQQCGVSFRVK | | | Carbamidomethyl (C)[4,7] | | Mascot |
| 1844.8782 | 1844.8348 | -0.0434 | -24 | 123 | 137 | VHTVEKPFSCQQCPK | | | Carbamidomethyl (C)[10,13] | | Mascot |
| 1844.8782 | 1844.8348 | -0.0434 | -24 | 123 | 137 | VHTVEKPFSCQQCPK | | | Carbamidomethyl (C)[10,13] | | Mascot |
| 1880.9963 | 1880.9956 | -0.0007 | 0 | 19 | 34 | VKQEDPQEQTDLLLPK | | | | | Mascot |

| Calc. Mass | Obsrv. Mass | ± da | ± ppm | Start Seq. | End Seq. | Sequence | Ion Score | C. I. % | Modification | Rank | Result Type |
|---|---|---|---|---|---|---|---|---|---|---|---|
| 2225.0227 | 2225.0662 | 0.0435 | 20 | 179 | 197 | VHTGEKPFTCQQCGTTFAR | | | Carbamidomethyl (C)[10,13] | | Mascot |
| 2225.0227 | 2225.0662 | 0.0435 | 20 | 179 | 197 | VHTGEKPFTCQQCGTTFAR | | | Carbamidomethyl (C)[10,13] | | Mascot |
| 2279.0696 | 2278.9968 | -0.0728 | -32 | 151 | 169 | VHTGEKPFSCEQCPKTFAR | | | Carbamidomethyl (C)[10,13] | | Mascot |
| 2300.0845 | 2300.126 | 0.0415 | 18 | 231 | 249 | VHMRIHSGEKPFTCQQCGK | | | Carbamidomethyl (C)[14,17] | | Mascot |
| 2371.0198 | 2371.0811 | 0.0613 | 26 | 91 | 110 | EHMLTHFTDGCFTCQLCGRR | | | Oxidation (M)[3] | | Mascot |
| 2371.0198 | 2371.0811 | 0.0613 | 26 | 91 | 110 | EHMLTHFTDGCFTCQLCGRR | | | Oxidation (M)[3] | | Mascot |
| 3227.5659 | 3227.5264 | -0.0395 | -12 | 235 | 262 | IHSGEKPFTCQQCGKVFSHSLNLNIHMR | | | Oxidation (M)[27] | | Mascot |

25 protein RMD5 homolog B [Danio rerio] gi|41152122 44610.5 6.03 9 34 0

**Peptide Information**

| Calc. Mass | Obsrv. Mass | ± da | ± ppm | Start Seq. | End Seq. | Sequence | Ion Score | C. I. % | Modification | Rank | Result Type |
|---|---|---|---|---|---|---|---|---|---|---|---|
| 842.4366 | 842.4913 | 0.0547 | 65 | 80 | 87 | DIHGSVSK | | | | | Mascot |
| 1045.4884 | 1045.5409 | 0.0525 | 50 | 307 | 315 | QCSGVWTHK | | | | | Mascot |
| 1106.559 | 1106.5333 | -0.0257 | -23 | 210 | 218 | QQEALQYAR | | | | | Mascot |
| 1567.6913 | 1567.8163 | 0.125 | 80 | 1 | 13 | MEQCACVERELEK | | | | | Mascot |
| 1880.834 | 1880.9956 | 0.1616 | 86 | 372 | 387 | LKCPYCPMEQNPSDAK | | | Carbamidomethyl (C)[3] | | Mascot |
| 2054.0889 | 2053.9241 | -0.1648 | -80 | 166 | 182 | TQDLRPALEWAVTNRQR | | | | | Mascot |
| 2133.908 | 2133.9275 | 0.0195 | 9 | 374 | 391 | CPYCPMEQNPSDAKQIYF | | | | | Mascot |
| 2225.1165 | 2225.0662 | -0.0503 | -23 | 341 | 360 | QQTSESNPPMKLICGHVISR | | | | | Mascot |
| 2225.1165 | 2225.0662 | -0.0503 | -23 | 341 | 360 | QQTSESNPPMKLICGHVISR | | | | | Mascot |
| 2298.1328 | 2298.1377 | 0.0049 | 2 | 341 | 360 | QQTSESNPPMKLICGHVISR | | | Carbamidomethyl (C)[14], Oxidation (M)[10] | | Mascot |
| 2580.2256 | 2580.2556 | 0.03 | 12 | 43 | 65 | LLAYGVQDADLSVLHQTMAQCCK | | | Carbamidomethyl (C)[21], Oxidation (M)[18] | | Mascot |
| 2580.2256 | 2580.2556 | 0.03 | 12 | 43 | 65 | LLAYGVQDADLSVLHQTMAQCCK | | | Carbamidomethyl (C)[21], Oxidation (M)[18] | | Mascot |

26 CLIP-associating protein 1 [Danio rerio] gi|169646251 168891.5 9.05 19 33 0

**Peptide Information**

| Calc. Mass | Obsrv. Mass | ± da | ± ppm | Start Seq. | End Seq. | Sequence | Ion Score | C. I. % | Modification | Rank | Result Type |
|---|---|---|---|---|---|---|---|---|---|---|---|
| 803.437 | 803.3616 | -0.0754 | -94 | 666 | 672 | VVSQSQR | | | | | Mascot |
| 856.5502 | 856.5068 | -0.0434 | -51 | 1319 | 1326 | GALIELLK | | | | | Mascot |
| 870.577 | 870.5187 | -0.0583 | -67 | 1365 | 1371 | VLKEILR | | | | | Mascot |
| 1082.5551 | 1082.4855 | -0.0696 | -64 | 1379 | 1387 | NYAELTIMK | | | | | Mascot |
| 1104.5255 | 1104.5543 | 0.0288 | 26 | 894 | 902 | MFADPHSKR | | | Oxidation (M)[1] | | Mascot |

| | | | | | | | | | | | |
|---|---|---|---|---|---|---|---|---|---|---|---|
| 1165.6801 | 1165.7196 | 0.0395 | 34 | 1355 | 1364 | DHTIRALALR | | | | | Mascot |
| 1213.6648 | 1213.5719 | -0.0929 | -77 | 724 | 734 | ETSPNRLGIAR | | | | | Mascot |
| 1325.524 | 1325.7146 | 0.1906 | 144 | 241 | 252 | NFDDEDSVDGGR | | | | | Mascot |
| 1325.8151 | 1325.7146 | -0.1005 | -76 | 863 | 874 | KEGLLGLQNLLK | | | | | Mascot |
| 1475.6802 | 1475.7168 | 0.0366 | 25 | 1330 | 1341 | EDSLAVWDEHFK | | | | | Mascot |
| 1648.8007 | 1648.7839 | -0.0168 | -10 | 735 | 749 | SSRIPRPSMSQGCSR | | | | | Mascot |
| 1728.9126 | 1728.7454 | -0.1672 | -97 | 788 | 804 | VLNTGTEVEAAVADALR | | | | | Mascot |
| 2001.0222 | 2000.9335 | -0.0887 | -44 | 349 | 366 | SLLMAGATEHEGFIQQLR | | | | | Mascot |
| 2053.9502 | 2053.9241 | -0.0261 | -13 | 1257 | 1274 | SREFAPYGYGDTITAYDK | | | | | Mascot |
| 2225.207 | 2225.0662 | -0.1408 | -63 | 381 | 401 | SQVVREACITLGHLSSLL GNK | | | | | Mascot |
| 2225.207 | 2225.0662 | -0.1408 | -63 | 381 | 401 | SQVVREACITLGHLSSLL GNK | | | | | Mascot |
| 2284.0769 | 2284.1206 | 0.0437 | 19 | 1 | 19 | MEPNMEYCLTQVLQKDV AR | | | Oxidation (M)[1] | | Mascot |
| 2300.072 | 2300.126 | 0.054 | 23 | 1 | 19 | MEPNMEYCLTQVLQKDV AR | | | Oxidation (M)[1,5] | | Mascot |
| 2371.1572 | 2371.0811 | -0.0761 | -32 | 118 | 137 | IMEQSATPQYIWDRMLG GFK | | | | | Mascot |
| 2371.1572 | 2371.0811 | -0.0761 | -32 | 118 | 137 | IMEQSATPQYIWDRMLG GFK | | | | | Mascot |
| 3218.4617 | 3218.5154 | 0.0537 | 17 | 37 | 65 | CQDLESDQTALDKMVDG IATSWVNSSNFK | | | Oxidation (M)[14] | | Mascot |
| 3218.4617 | 3218.5154 | 0.0537 | 17 | 37 | 65 | CQDLESDQTALDKMVDG IATSWVNSSNFK | | | Oxidation (M)[14] | | Mascot |
| 3338.7485 | 3338.6836 | -0.0649 | -19 | 50 | 80 | MVDGIATSWVNSSNFKL ALLGMDLLSALVTR | | | Oxidation (M)[1] | | Mascot |

27 X-prolyl aminopeptidase (aminopeptidase P) 3, putative [Danio rerio] gi|158253582 56493.5 7.08 9 33 0

**Peptide Information**

| Calc. Mass | Obsrv. Mass | ± da | ± ppm | Start Seq. | End Seq. | Sequence | Ion Score | C. I. % | Modification | Rank | Result Type |
|---|---|---|---|---|---|---|---|---|---|---|---|
| 803.2988 | 803.3616 | 0.0628 | 78 | 504 | 510 | TCAHAED | | | Carbamidomethyl (C)[2] | | Mascot |
| 856.4015 | 856.5068 | 0.1053 | 123 | 268 | 274 | KTMAMSR | | | Oxidation (M)[3,5] | | Mascot |
| 1179.6957 | 1179.5769 | -0.1188 | -101 | 235 | 244 | SLRPLTHSLR | | | | | Mascot |
| 1584.6722 | 1584.8403 | 0.1681 | 106 | 19 | 31 | SYWSPGSVWCPCR | | | Carbamidomethyl (C)[10] | | Mascot |
| 1584.6722 | 1584.8403 | 0.1681 | 106 | 19 | 31 | SYWSPGSVWCPCR | | | Carbamidomethyl (C)[10] | | Mascot |
| 1638.9425 | 1638.8247 | -0.1178 | -72 | 188 | 202 | VHSTEELGVVLKSIK | | | | | Mascot |
| 1993.9695 | 1993.9235 | -0.046 | -23 | 1 | 18 | MFHSSSGLLRAAVQCAS R | | | Carbamidomethyl (C)[15], Oxidation (M)[1] | | Mascot |
| 2054.0586 | 2053.9241 | -0.1345 | -65 | 395 | 413 | QLKELGILPSHASDTDAM K | | | | | Mascot |
| 2371.0527 | 2371.0811 | 0.0284 | 12 | 11 | 31 | AAVQCASRSYWSPGSV WCPCR | | | Carbamidomethyl (C)[5] | | Mascot |
| 2371.0527 | 2371.0811 | 0.0284 | 12 | 11 | 31 | AAVQCASRSYWSPGSV WCPCR | | | Carbamidomethyl (C)[5] | | Mascot |
| 2439.0842 | 2439.0789 | -0.0053 | -2 | 324 | 345 | DGEMVLLDGGCEYFGYV | | | | | Mascot |

| | | | | | | | | | | | |
|---|---|---|---|---|---|---|---|---|---|---|---|
| 2455.0791 | 2455.083 | 0.0039 | 2 | 324 | 345 | SDITR DGEMVLLDGGCEYFGYV SDITR | | | Oxidation (M)[4] | | Mascot |

28 SH3 domain-binding protein 4-A [Danio rerio] gi|45387913 104947.5 7.73 13 33 0

**Peptide Information**

| Calc. Mass | Obsrv. Mass | ± da | ± ppm | Start Seq. | End Seq. | Sequence | Ion Score | C. I. % | Modification | Rank | Result Type |
|---|---|---|---|---|---|---|---|---|---|---|---|
| 842.5822 | 842.4913 | -0.0909 | -108 | 634 | 640 | LIKTVIR | | | | | Mascot |
| 850.4338 | 850.5243 | 0.0905 | 106 | 358 | 364 | LSNMEIK | | | Oxidation (M)[4] | | Mascot |
| 870.4726 | 870.5187 | 0.0461 | 53 | 1 | 7 | MAAHRIR | | | Oxidation (M)[1] | | Mascot |
| 973.5829 | 973.4905 | -0.0924 | -95 | 666 | 673 | LKGQLWTK | | | | | Mascot |
| 973.5829 | 973.4905 | -0.0924 | -95 | 666 | 673 | LKGQLWTK | | | | | Mascot |
| 1045.4796 | 1045.5409 | 0.0613 | 59 | 762 | 770 | AELDSEPER | | | | | Mascot |
| 1082.4493 | 1082.4855 | 0.0362 | 33 | 780 | 788 | EDCLNMETK | | | | | Mascot |
| 1434.7158 | 1434.7355 | 0.0197 | 14 | 885 | 896 | DVIHELHMGLDR | | | | | Mascot |
| 2118.1958 | 2117.9324 | -0.2634 | -124 | 607 | 624 | IILSPLAITTKYPQFQER | | | | | Mascot |
| 2284.2368 | 2284.1206 | -0.1162 | -51 | 463 | 484 | TVVAIFGHDCAPKTLLVS EVGK | | | | | Mascot |
| 2300.0686 | 2300.126 | 0.0574 | 25 | 568 | 587 | DDQDCILAQFCVQTPQP PPK | | | Carbamidomethyl (C)[5] | | Mascot |
| 3227.677 | 3227.5264 | -0.1506 | -47 | 907 | 936 | HLTGTLILVNCLDLLRSSA FSPAPQDDFAI | | | | | Mascot |
| 3337.6052 | 3337.6785 | 0.0733 | 22 | 269 | 298 | LARSCHDLESLGQNPGW GQTQPVETNIVCK | | | Carbamidomethyl (C)[5] | | Mascot |
| 3338.5496 | 3338.6836 | 0.134 | 40 | 856 | 884 | TGMVDSEAMWKPAYDFL LTWAAQIGDSYR | | | Oxidation (M)[3] | | Mascot |

29 PREDICTED: tripartite motif-containing protein 39-like [Danio rerio] gi|326676388 71379.4 6.07 11 33 0

**Peptide Information**

| Calc. Mass | Obsrv. Mass | ± da | ± ppm | Start Seq. | End Seq. | Sequence | Ion Score | C. I. % | Modification | Rank | Result Type |
|---|---|---|---|---|---|---|---|---|---|---|---|
| 803.4257 | 803.3616 | -0.0641 | -80 | 268 | 274 | AAEKQEK | | | | | Mascot |
| 850.4967 | 850.5243 | 0.0276 | 32 | 578 | 584 | FLRVMGK | | | | | Mascot |
| 1082.5009 | 1082.4855 | -0.0154 | -14 | 402 | 411 | FDTCPCVLGK | | | | | Mascot |
| 1234.6427 | 1234.6538 | 0.0111 | 9 | 77 | 86 | ETFTQRPDLK | | | | | Mascot |
| 1316.674 | 1316.6663 | -0.0077 | -6 | 17 | 27 | RNINEPMSLSR | | | | | Mascot |
| 1584.7515 | 1584.8403 | 0.0888 | 56 | 503 | 516 | LFPYFGPCLNDGGK | | | Carbamidomethyl (C)[8] | | Mascot |
| 1584.7515 | 1584.8403 | 0.0888 | 56 | 503 | 516 | LFPYFGPCLNDGGK | | | Carbamidomethyl (C)[8] | | Mascot |
| 2133.8643 | 2133.9275 | 0.0632 | 30 | 58 | 76 | SCLNTCWNKTQTCSCPN CK | | | | | Mascot |
| 2210.9595 | 2211.0537 | 0.0942 | 43 | 176 | 194 | DDQTGVCLYCSFTDHKN HK | | | | | Mascot |

| Calc. Mass | Obsrv. Mass | ± da | ± ppm | Start Seq. | End Seq. | Sequence | Ion Score | C. I. % | Modification | Rank | Result Type |
|---|---|---|---|---|---|---|---|---|---|---|---|
| 2225.0247 | 2225.0662 | 0.0415 | 19 | 250 | 267 | SIERCQTELLEMMEEQQK | | | | | Mascot |
| 2225.0247 | 2225.0662 | 0.0415 | 19 | 250 | 267 | SIERCQTELLEMMEEQQK | | | | | Mascot |
| 2298.041 | 2298.1377 | 0.0967 | 42 | 250 | 267 | SIERCQTELLEMMEEQQK | | | Carbamidomethyl (C)[5], Oxidation (M)[12] | | Mascot |
| 2300.0469 | 2300.126 | 0.0791 | 34 | 67 | 86 | TQTCSCPNCKETFTQRPDLK | | | | | Mascot |
| 3338.52 | 3338.6836 | 0.1636 | 49 | 28 | 57 | VPLSEDLQCSICLEVFTDPVSTPCGHNFCK | | | Carbamidomethyl (C)[9] | | Mascot |

| | | | | | | | |
|---|---|---|---|---|---|---|---|
| 30 | BSD domain containing 1 [Danio rerio] | gi\|51480446 | 45281.8 | 4.28 | 8 | 33 | 0 |

**Peptide Information**

| Calc. Mass | Obsrv. Mass | ± da | ± ppm | Start Seq. | End Seq. | Sequence | Ion Score | C. I. % | Modification | Rank | Result Type |
|---|---|---|---|---|---|---|---|---|---|---|---|
| 1213.6211 | 1213.5719 | -0.0492 | -41 | 24 | 33 | SAEAYEFIKR | | | | | Mascot |
| 1563.7068 | 1563.8031 | 0.0963 | 62 | 336 | 348 | EETAREDMAQDLR | | | | | Mascot |
| 1563.7068 | 1563.8031 | 0.0963 | 62 | 336 | 348 | EETAREDMAQDLR | | | | | Mascot |
| 1880.8984 | 1880.9956 | 0.0972 | 52 | 349 | 366 | VFELNSDSGKSTPSNNGK | | | | | Mascot |
| 2000.8827 | 2000.9335 | 0.0508 | 25 | 380 | 396 | DFDLDMTEEEVQMALSK | | | | | Mascot |
| 2053.9495 | 2053.9241 | -0.0254 | -12 | 341 | 358 | EDMAQDLRVFELNSDSGK | | | | | Mascot |
| 2439.1362 | 2439.0789 | -0.0573 | -23 | 2 | 23 | ADGEGGWWGGWLTQSFQSVKDK | | | | | Mascot |
| 2657.3164 | 2657.1265 | -0.1899 | -71 | 318 | 340 | LSDTGLQETFPEERPVPREETAR | | | | | Mascot |
| 3337.4246 | 3337.6785 | 0.2539 | 76 | 368 | 396 | GSSTDVSEDWEKDFDLDMTEEEVQMALSK | | | Oxidation (M)[18] | | Mascot |

| | | | | | | | |
|---|---|---|---|---|---|---|---|
| 31 | pyruvate dehydrogenase kinase, isozyme 3b [Danio rerio] | gi\|123702577 | 21003.7 | 8.49 | 7 | 33 | 0 |

**Peptide Information**

| Calc. Mass | Obsrv. Mass | ± da | ± ppm | Start Seq. | End Seq. | Sequence | Ion Score | C. I. % | Modification | Rank | Result Type |
|---|---|---|---|---|---|---|---|---|---|---|---|
| 882.4468 | 882.5473 | 0.1005 | 114 | 30 | 36 | QFLDFGR | | | | | Mascot |
| 1045.5499 | 1045.5409 | -0.009 | -9 | 43 | 50 | TSYMFLRK | | | | | Mascot |
| 1165.6836 | 1165.7196 | 0.036 | 31 | 1 | 9 | MKLFICLLK | | | Carbamidomethyl (C)[6] | | Mascot |
| 1325.6631 | 1325.7146 | 0.0515 | 39 | 162 | 172 | MLINQHSETPR | | | | | Mascot |
| 1325.6631 | 1325.7146 | 0.0515 | 39 | 162 | 172 | MLINQHSETPR | | | | | Mascot |
| 1648.7457 | 1648.7839 | 0.0382 | 23 | 37 | 49 | ENACEKTSYMFLR | | | Carbamidomethyl (C)[4] | | Mascot |
| 1844.9436 | 1844.8348 | -0.1088 | -59 | 158 | 172 | ISFRMLINQHSETPR | | | Oxidation (M)[5] | | Mascot |
| 1844.9436 | 1844.8348 | -0.1088 | -59 | 158 | 172 | ISFRMLINQHSETPR | | | Oxidation (M)[5] | | Mascot |
| 2284.1641 | 2284.1206 | -0.0435 | -19 | 97 | 115 | SPEDPHTLNDFLEMLIKIR | | | Oxidation (M)[14] | | Mascot |

| | | | | | | | |
|---|---|---|---|---|---|---|---|
| 32 | adipocyte plasma membrane-associated protein [Danio rerio] | gi\|47086817 | 46714 | 6.81 | 8 | 32 | 0 |

Peptide Information

| Calc. Mass | Obsrv. Mass | ± da | ± ppm | Start Seq. | End Seq. | Sequence | Ion Score | C. I. % | Modification | Rank | Result Type |
|---|---|---|---|---|---|---|---|---|---|---|---|
| 1234.5919 | 1234.6538 | 0.0619 | 50 | 247 | 256 | EVNVMMENLR | | | | | Mascot |
| 1563.8893 | 1563.8031 | -0.0862 | -55 | 347 | 359 | LFSQDTLLKFVPR | | | | | Mascot |
| 1563.8893 | 1563.8031 | -0.0862 | -55 | 347 | 359 | LFSQDTLLKFVPR | | | | | Mascot |
| 1638.7251 | 1638.8247 | 0.0996 | 61 | 224 | 237 | DFMHLIMEATADGR | | | Oxidation (M)[3,7] | | Mascot |
| 1648.854 | 1648.7839 | -0.0701 | -43 | 196 | 210 | LGFVNDLDVTQDGKK | | | | | Mascot |
| 2118.1599 | 2117.9324 | -0.2275 | -107 | 123 | 142 | IEGRNIHVLATIGKPPCGS R | | | | | Mascot |
| 2225.1382 | 2225.0662 | -0.072 | -32 | 356 | 374 | FVPRYSLVVELQSDGTC VR | | | Carbamidomethyl (C)[17] | | Mascot |
| 2225.1382 | 2225.0662 | -0.072 | -32 | 356 | 374 | FVPRYSLVVELQSDGTC VR | | | Carbamidomethyl (C)[17] | | Mascot |
| 2247.1628 | 2247.0203 | -0.1425 | -63 | 11 | 28 | RLNRPHIITDETHEPQYK | | | | | Mascot |
| 2580.2649 | 2580.2556 | -0.0093 | -4 | 257 | 279 | FPNGIQLFPDEESVLVAE TTMAR | | | Oxidation (M)[21] | | Mascot |
| 2580.2649 | 2580.2556 | -0.0093 | -4 | 257 | 279 | FPNGIQLFPDEESVLVAE TTMAR | | | Oxidation (M)[21] | | Mascot |

33 PREDICTED: zinc finger protein 567-like [Danio rerio] gi|326666771 48963.9 9.43 10 32 0

Peptide Information

| Calc. Mass | Obsrv. Mass | ± da | ± ppm | Start Seq. | End Seq. | Sequence | Ion Score | C. I. % | Modification | Rank | Result Type |
|---|---|---|---|---|---|---|---|---|---|---|---|
| 856.4457 | 856.5068 | 0.0611 | 71 | 147 | 153 | NLAVHMR | | | Oxidation (M)[6] | | Mascot |
| 1082.4796 | 1082.4855 | 0.0059 | 5 | 347 | 355 | HMQTHSGER | | | | | Mascot |
| 1584.7839 | 1584.8403 | 0.0564 | 36 | 85 | 97 | SFSQKPNLDVYMR | | | | | Mascot |
| 1584.7839 | 1584.8403 | 0.0564 | 36 | 85 | 97 | SFSQKPNLDVYMR | | | | | Mascot |
| 1728.8738 | 1728.7454 | -0.1284 | -74 | 84 | 97 | KSFSQKPNLDVYMR | | | Oxidation (M)[13] | | Mascot |
| 2000.9355 | 2000.9335 | -0.002 | -1 | 172 | 187 | QNYNLEVHMRTHTGER | | | Oxidation (M)[9] | | Mascot |
| 2284.085 | 2284.1206 | 0.0356 | 16 | 266 | 284 | IHTGEKPYTCTECGKSFI R | | | Carbamidomethyl (C)[10,13] | | Mascot |
| 2367.1799 | 2367.2065 | 0.0266 | 11 | 377 | 397 | LHNSEQSPQHLGLVHTN AEQK | | | | | Mascot |
| 2580.1462 | 2580.2556 | 0.1094 | 42 | 314 | 336 | LSLMNHMNGHTGTIVFTC DQCGK | | | Carbamidomethyl (C)[18], Oxidation (M)[4] | | Mascot |
| 2580.1462 | 2580.2556 | 0.1094 | 42 | 314 | 336 | LSLMNHMNGHTGTIVFTC DQCGK | | | Carbamidomethyl (C)[18], Oxidation (M)[4] | | Mascot |
| 2596.1411 | 2596.2539 | 0.1128 | 43 | 314 | 336 | LSLMNHMNGHTGTIVFTC DQCGK | 3 | 0 | Carbamidomethyl (C)[18], Oxidation (M)[4,7] | | Mascot |
| 2596.1411 | 2596.2539 | 0.1128 | 43 | 314 | 336 | LSLMNHMNGHTGTIVFTC DQCGK | | | Carbamidomethyl (C)[18], Oxidation (M)[4,7] | | Mascot |
| 2657.238 | 2657.1265 | -0.1115 | -42 | 147 | 168 | NLAVHMRVHTGEKPYSC PQCEK | | | Carbamidomethyl (C)[17,20], Oxidation (M)[6] | | Mascot |
| 3218.4463 | 3218.5154 | 0.0691 | 21 | 21 | 46 | QEDLQEQTDLMMLKEET HQQNEVEEK | | | Oxidation (M)[11] | | Mascot |

| | | | | | | | | | | | |
|---|---|---|---|---|---|---|---|---|---|---|---|
| 3218.4463 | 3218.5154 | 0.0691 | 21 | 21 | 46 | QEDLQEQTDLMMLKEETHQQNEVEEK | | | Oxidation (M)[11] | | Mascot |

| | | | | | | | |
|---|---|---|---|---|---|---|---|
| 34 | PREDICTED: hypothetical protein LOC100004944 [Danio rerio] | gi\|125854141 | 69244.2 | 6.33 | 10 | 32 | 0 |

Peptide Information

| Calc. Mass | Obsrv. Mass | ± da | ± ppm | Start Seq. | End Seq. | Sequence | Ion Score | C. I. % | Modification | Rank | Result Type |
|---|---|---|---|---|---|---|---|---|---|---|---|
| 1045.452 | 1045.5409 | 0.0889 | 85 | 472 | 479 | CHYSDHVK | | | Carbamidomethyl (C)[1] | | Mascot |
| 1316.6052 | 1316.6663 | 0.0611 | 46 | 472 | 482 | CHYSDHVKEAK | | | | | Mascot |
| 1567.873 | 1567.8163 | -0.0567 | -36 | 259 | 272 | LIYITDAVQSLFGK | | | | | Mascot |
| 1729.9265 | 1729.7338 | -0.1927 | -111 | 361 | 375 | MTQDVLQIQRELGAK | | | | | Mascot |
| 2048.1321 | 2047.9043 | -0.2278 | -111 | 343 | 360 | GMTGLTQFINNIKPRTLK | | | Oxidation (M)[2] | | Mascot |
| 2211.1147 | 2211.0537 | -0.061 | -28 | 153 | 171 | TTLVNVMVNYMLGVEREDK | | | | | Mascot |
| 2239.2485 | 2239.0825 | -0.166 | -74 | 278 | 298 | IVLLFTHSDGFPPSNALIAVK | | | | | Mascot |
| 2283.2595 | 2283.1292 | -0.1303 | -57 | 544 | 563 | IALNTDSLNTLLHIDFLIEK | | | | | Mascot |
| 2367.2085 | 2367.2065 | -0.002 | -1 | 234 | 254 | IDDLTHEINAVCLVNKATQNR | | | | | Mascot |
| 2371.0627 | 2371.0811 | 0.0184 | 8 | 35 | 53 | ECLQQFWATTKTQDCPVCR | | | Carbamidomethyl (C)[2,15] | | Mascot |
| 2371.0627 | 2371.0811 | 0.0184 | 8 | 35 | 53 | ECLQQFWATTKTQDCPVCR | | | Carbamidomethyl (C)[2,15] | | Mascot |

| | | | | | | | |
|---|---|---|---|---|---|---|---|
| 35 | PREDICTED: transposon TX1 uncharacterized 149 kDa protein-like [Danio rerio] | gi\|326667829 | 148918.2 | 8.57 | 15 | 31 | 0 |

Peptide Information

| Calc. Mass | Obsrv. Mass | ± da | ± ppm | Start Seq. | End Seq. | Sequence | Ion Score | C. I. % | Modification | Rank | Result Type |
|---|---|---|---|---|---|---|---|---|---|---|---|
| 850.5621 | 850.5243 | -0.0378 | -44 | 387 | 393 | RLIHALK | | | | | Mascot |
| 902.4941 | 902.4664 | -0.0277 | -31 | 517 | 524 | KGDLNDIK | | | | | Mascot |
| 1325.7463 | 1325.7146 | -0.0317 | -24 | 21 | 30 | YQIYEIIKQK | | | | | Mascot |
| 1325.7463 | 1325.7146 | -0.0317 | -24 | 21 | 30 | YQIYEIIKQK | | | | | Mascot |
| 1347.7452 | 1347.6968 | -0.0484 | -36 | 1214 | 1225 | CQLINFLLGQAK | | | | | Mascot |
| 1563.9409 | 1563.8031 | -0.1378 | -88 | 842 | 854 | LHWVPKAVLYLPK | | | | | Mascot |
| 1563.9409 | 1563.8031 | -0.1378 | -88 | 842 | 854 | LHWVPKAVLYLPK | | | | | Mascot |
| 1844.9865 | 1844.8348 | -0.1517 | -82 | 1015 | 1029 | LLEQWRASLTTEEIR | | | | | Mascot |
| 1845.0592 | 1844.8348 | -0.2244 | -122 | 344 | 360 | NILGKFLDTVTQGALVR | | | | | Mascot |
| 1880.9653 | 1880.9956 | 0.0303 | 16 | 740 | 755 | WLNGEPNLPDGLLWTR | | | | | Mascot |
| 1993.9457 | 1993.9235 | -0.0222 | -11 | 449 | 466 | ALTLEELEEALQSMECGK | | | | | Mascot |
| 2193.1697 | 2193.052 | -0.1177 | -54 | 296 | 314 | QLCQQYTLNVTGDILRSIK | | | | | Mascot |

| | | | | | | | | | | | |
|---|---|---|---|---|---|---|---|---|---|---|---|
| 2239.1865 | 2239.0825 | -0.104 | -46 | 654 | 673 | QGCALSGMLYVLAIEPFLI K | | | Carbamidomethyl (C)[3], Oxidation (M)[8] | | Mascot |
| 2298.1509 | 2298.1377 | -0.0132 | -6 | 627 | 647 | ALYCDVESILKVNGDLCA PFK | | | | | Mascot |
| 2439.2917 | 2439.0789 | -0.2128 | -87 | 760 | 781 | YLGVFLGNEAVVQKNFE GVVEK | | | | | Mascot |
| 2580.2002 | 2580.2556 | 0.0554 | 21 | 220 | 241 | SCSICPVSFSDHSLLQCVI SQR | | | Carbamidomethyl (C)[2,5,17] | | Mascot |
| 2580.2002 | 2580.2556 | 0.0554 | 21 | 220 | 241 | SCSICPVSFSDHSLLQCVI SQR | | | Carbamidomethyl (C)[2,5,17] | | Mascot |
| 3218.6602 | 3218.5154 | -0.1448 | -45 | 1136 | 1164 | ILHGAVAVNAFISILNPDV VHDCPFCLQR | | | Carbamidomethyl (C)[23] | | Mascot |
| 3218.6602 | 3218.5154 | -0.1448 | -45 | 1136 | 1164 | ILHGAVAVNAFISILNPDV VHDCPFCLQR | | | Carbamidomethyl (C)[23] | | Mascot |

| | | | | | | | | |
|---|---|---|---|---|---|---|---|---|
| 36 | lactase-like [Danio rerio] | gi\|57526567 | 63841.3 | 7.96 | 10 | 31 | 0 | |

**Peptide Information**

| Calc. Mass | Obsrv. Mass | ± da | ± ppm | Start Seq. | End Seq. | Sequence | Ion Score | C. I. % | Modification | Rank | Result Type |
|---|---|---|---|---|---|---|---|---|---|---|---|
| 803.3893 | 803.3616 | -0.0277 | -34 | 277 | 283 | DIEAAER | | | | | Mascot |
| 842.4954 | 842.4913 | -0.0041 | -5 | 2 | 8 | QRNGVLR | | | | | Mascot |
| 882.4203 | 882.5473 | 0.127 | 144 | 126 | 132 | TDYINEK | | | | | Mascot |
| 902.5206 | 902.4664 | -0.0542 | -60 | 484 | 490 | NKPRYPK | | | | | Mascot |
| 973.5359 | 973.4905 | -0.0454 | -47 | 1 | 8 | MQRNGVLR | | | | | Mascot |
| 973.5359 | 973.4905 | -0.0454 | -47 | 1 | 8 | MQRNGVLR | | | | | Mascot |
| 1058.4935 | 1058.5662 | 0.0727 | 69 | 536 | 544 | SKEHADMPK | | | Oxidation (M)[7] | | Mascot |
| 1475.5422 | 1475.7168 | 0.1746 | 118 | 420 | 430 | MMCTDLCDEWR | | | Carbamidomethyl (C)[3], Oxidation (M)[1] | | Mascot |
| 1993.9106 | 1993.9235 | 0.0129 | 6 | 133 | 148 | GIEHYDNMINMLLENR | | | Oxidation (M)[8,11] | | Mascot |
| 2054.0413 | 2053.9241 | -0.1172 | -57 | 313 | 331 | SAQQGLSSSRLPAFNPH EK | | | | | Mascot |
| 2247.071 | 2247.0203 | -0.0507 | -23 | 256 | 276 | GMVGISLSADWGEPVDV TNQR | | | Oxidation (M)[2] | | Mascot |

| | | | | | | | | |
|---|---|---|---|---|---|---|---|---|
| 37 | PREDICTED: bromodomain adjacent to zinc finger domain protein 1A, partial [Danio rerio] | gi\|125841384 | 81560.8 | 6.03 | 12 | 31 | 0 | |

**Peptide Information**

| Calc. Mass | Obsrv. Mass | ± da | ± ppm | Start Seq. | End Seq. | Sequence | Ion Score | C. I. % | Modification | Rank | Result Type |
|---|---|---|---|---|---|---|---|---|---|---|---|
| 803.4145 | 803.3616 | -0.0529 | -66 | 316 | 322 | DDLIEAK | | | | | Mascot |
| 902.5669 | 902.4664 | -0.1005 | -111 | 604 | 611 | LLTLVSTR | | | | | Mascot |
| 1045.3999 | 1045.5409 | 0.141 | 135 | 303 | 310 | EQMMSMEK | | | Oxidation (M)[3,4] | | Mascot |
| 1106.4789 | 1106.5333 | 0.0544 | 49 | 36 | 43 | TYDEFFER | | | | | Mascot |
| 1234.7782 | 1234.6538 | -0.1244 | -101 | 2 | 11 | PLLHKKPFVR | | | | | Mascot |
| 1316.5796 | 1316.6663 | 0.0867 | 66 | 303 | 312 | EQMMSMEKFR | | | | | Mascot |
| 1567.7461 | 1567.8163 | 0.0702 | 45 | 101 | 113 | LHEICDDVYAYVK | | | | | Mascot |

| Calc. Mass | Obsrv. Mass | ± da | ± ppm | Start Seq. | End Seq. | Sequence | Ion Score | C. I. % | Modification | Rank | Result Type |
|---|---|---|---|---|---|---|---|---|---|---|---|
| 1845.0084 | 1844.8348 | -0.1736 | -94 | 596 | 611 | ILHSMCGKLLTLVSTR | | | Carbamidomethyl (C)[6], Oxidation (M)[5] | | Mascot |
| 1845.0084 | 1844.8348 | -0.1736 | -94 | 596 | 611 | ILHSMCGKLLTLVSTR | 2 | 0 | Carbamidomethyl (C)[6], Oxidation (M)[5] | | Mascot |
| 1880.8517 | 1880.9956 | 0.1439 | 77 | 378 | 392 | LWSKPREDMECDDLK | | | Oxidation (M)[9] | | Mascot |
| 1993.9912 | 1993.9235 | -0.0677 | -34 | 535 | 552 | LHILSSGADCGFTNAKFR | | | Carbamidomethyl (C)[10] | | Mascot |
| 2283.1836 | 2283.1292 | -0.0544 | -24 | 195 | 215 | VQPVSPEACEPVIVKVAQ MSR | | | Oxidation (M)[19] | | Mascot |
| 3218.6958 | 3218.5154 | -0.1804 | -56 | 402 | 429 | TRLPPELFGDALMVLEFL HAFGELFDLK | | | | | Mascot |
| 3218.6958 | 3218.5154 | -0.1804 | -56 | 402 | 429 | TRLPPELFGDALMVLEFL HAFGELFDLK | | | | | Mascot |

| | | | | | | | |
|---|---|---|---|---|---|---|---|
| 38 | PREDICTED: hypothetical protein LOC798191 [Danio rerio] | gi\|189522448 | 41942 | 8.11 | 8 | 31 | 0 |

Peptide Information

| Calc. Mass | Obsrv. Mass | ± da | ± ppm | Start Seq. | End Seq. | Sequence | Ion Score | C. I. % | Modification | Rank | Result Type |
|---|---|---|---|---|---|---|---|---|---|---|---|
| 864.4726 | 864.4924 | 0.0198 | 23 | 322 | 328 | FFGQPLR | | | | | Mascot |
| 996.4489 | 996.5773 | 0.1284 | 129 | 213 | 221 | QSPSCTMVK | | | Oxidation (M)[7] | | Mascot |
| 1082.4797 | 1082.4855 | 0.0058 | 5 | 358 | 366 | CCPPAFYPK | | | Carbamidomethyl (C)[1] | | Mascot |
| 1316.6528 | 1316.6663 | 0.0135 | 10 | 124 | 134 | HSRCQPGQYIK | | | | | Mascot |
| 1563.6825 | 1563.8031 | 0.1206 | 77 | 213 | 225 | QSPSCTMVKNHCR | | | Carbamidomethyl (C)[5], Oxidation (M)[7] | | Mascot |
| 1563.6825 | 1563.8031 | 0.1206 | 77 | 213 | 225 | QSPSCTMVKNHCR | | | Carbamidomethyl (C)[5], Oxidation (M)[7] | | Mascot |
| 2053.947 | 2053.9241 | -0.0229 | -11 | 97 | 114 | SSNTVCEPLAGFYCIHQK | | | Carbamidomethyl (C)[6] | | Mascot |
| 2239.0635 | 2239.0825 | 0.019 | 8 | 97 | 115 | SSNTVCEPLAGFYCIHQK K | | | Carbamidomethyl (C)[6,14] | | Mascot |
| 3337.4883 | 3337.6785 | 0.1902 | 57 | 50 | 78 | CTIDTVTTCVPCPPLTYT DEPNGLYTCFR | | | Carbamidomethyl (C)[1,9] | | Mascot |

| | | | | | | | |
|---|---|---|---|---|---|---|---|
| 39 | Si:ch211-215a10.4 protein [Danio rerio] | gi\|126631961 | 20359.9 | 9.75 | 5 | 30 | 0 |

Peptide Information

| Calc. Mass | Obsrv. Mass | ± da | ± ppm | Start Seq. | End Seq. | Sequence | Ion Score | C. I. % | Modification | Rank | Result Type |
|---|---|---|---|---|---|---|---|---|---|---|---|
| 882.5519 | 882.5473 | -0.0046 | -5 | 5 | 11 | LRVELPR | | | | | Mascot |
| 1567.8671 | 1567.8163 | -0.0508 | -32 | 85 | 97 | GLPFHSILVFFYK | | | | | Mascot |
| 2367.1152 | 2367.2065 | 0.0913 | 39 | 101 | 120 | NHLYRTYDAFSTFYIGAD GR | | | | | Mascot |
| 3337.7615 | 3337.6785 | -0.083 | -25 | 41 | 67 | VVYQLTLTLWKLMCLFYY ADAQLEVLK | | | Carbamidomethyl (C)[14], Oxidation (M)[13] | | Mascot |
| 3338.9343 | 3338.6836 | -0.2507 | -75 | 140 | 171 | VASMLGGALVALGIQEHR PALNLLPLLLSSLR | | | Oxidation (M)[4] | | Mascot |

| | | | | | | | |
|---|---|---|---|---|---|---|---|
| 40 | PREDICTED: protein NLRC3-like [Danio rerio] | gi\|326667285 | 62976.7 | 6.15 | 9 | 30 | 0 |

Peptide Information

| Calc. Mass | Obsrv. Mass | ± da | ± ppm | Start | End | Sequence | Ion | C. I. % | Modification | Rank | Result Type |
|---|---|---|---|---|---|---|---|---|---|---|---|

| Calc. Mass | Obsrv. Mass | ± da | ± ppm | Start Seq. | End Seq. | Sequence | Ion Score | C. I. % | Modification | Rank | Result Type |
|---|---|---|---|---|---|---|---|---|---|---|---|
| 864.3767 | 864.4924 | 0.1157 | 134 | 363 | 370 | DCGIDVDK | | | | | Mascot |
| 902.44 | 902.4664 | 0.0264 | 29 | 230 | 237 | IPADCIDR | | | | | Mascot |
| 973.5789 | 973.4905 | -0.0884 | -91 | 528 | 536 | KSVTALQAR | | | | | Mascot |
| 973.5789 | 973.4905 | -0.0884 | -91 | 528 | 536 | KSVTALQAR | | | | | Mascot |
| 1045.5272 | 1045.5409 | 0.0137 | 13 | 423 | 431 | GLLTQQDDR | | | | | Mascot |
| 1234.7041 | 1234.6538 | -0.0503 | -41 | 436 | 445 | EEIIAYIKQK | | | | | Mascot |
| 1434.6755 | 1434.7355 | 0.06 | 42 | 371 | 383 | ASVYSGMCTQIFK | | | | | Mascot |
| 2134.1326 | 2133.9275 | -0.2051 | -96 | 1 | 17 | MEIYELLFIYQLKCSLK | | | | | Mascot |
| 2298.1812 | 2298.1377 | -0.0435 | -19 | 303 | 320 | TLTQMYTHFLRFQIQQSR | | | | | Mascot |
| 2580.2576 | 2580.2556 | -0.002 | -1 | 39 | 60 | IYTDLYITQGASEQVNTE HEIR | | | | | Mascot |
| 2580.2576 | 2580.2556 | -0.002 | -1 | 39 | 60 | IYTDLYITQGASEQVNTE HEIR | | | | | Mascot |

| | | | | | | | |
|---|---|---|---|---|---|---|---|
| 41 | PREDICTED: hypothetical protein LOC100001326 [Danio rerio] | gi\|125846403 | 17013.6 | 6.06 | 5 | 30 | 0 |

**Peptide Information**

| Calc. Mass | Obsrv. Mass | ± da | ± ppm | Start Seq. | End Seq. | Sequence | Ion Score | C. I. % | Modification | Rank | Result Type |
|---|---|---|---|---|---|---|---|---|---|---|---|
| 1058.5339 | 1058.5662 | 0.0323 | 31 | 128 | 136 | VCAFNYTIK | | | | | Mascot |
| 1213.5956 | 1213.5719 | -0.0237 | -20 | 142 | 152 | ACFIMNTVTVV | | | Oxidation (M)[5] | | Mascot |
| 1584.8387 | 1584.8403 | 0.0016 | 1 | 106 | 118 | CQIIANQLNRQAR | | | Carbamidomethyl (C)[1] | | Mascot |
| 1584.8387 | 1584.8403 | 0.0016 | 1 | 106 | 118 | CQIIANQLNRQAR | | | Carbamidomethyl (C)[1] | | Mascot |
| 2054.0627 | 2053.9241 | -0.1386 | -67 | 119 | 136 | ELAEPGTLKVCAFNYTIK | | | Carbamidomethyl (C)[11] | | Mascot |
| 2284.1416 | 2284.1206 | -0.021 | -9 | 42 | 62 | QQVIEVSESSESVQSPAL NPR | | | | | Mascot |

| | | | | | | | |
|---|---|---|---|---|---|---|---|
| 42 | estrogen receptor alpha long isoform [Danio rerio] | gi\|154850277 | 68248.4 | 8.6 | 11 | 30 | 0 |

**Peptide Information**

| Calc. Mass | Obsrv. Mass | ± da | ± ppm | Start Seq. | End Seq. | Sequence | Ion Score | C. I. % | Modification | Rank | Result Type |
|---|---|---|---|---|---|---|---|---|---|---|---|
| 902.469 | 902.4664 | -0.0026 | -3 | 310 | 317 | TTGPQDKR | | | | | Mascot |
| 1104.4886 | 1104.5543 | 0.0657 | 59 | 263 | 271 | KCYEVGMMK | | | Oxidation (M)[7] | | Mascot |
| 1213.6398 | 1213.5719 | -0.0679 | -56 | 373 | 382 | ELVHMIAWAK | | | Oxidation (M)[5] | | Mascot |
| 1316.7573 | 1316.6663 | -0.091 | -69 | 422 | 432 | LIFAQDLILDR | | | | | Mascot |
| 1325.7399 | 1325.7146 | -0.0253 | -19 | 373 | 383 | ELVHMIAWAKK | | | | | Mascot |
| 1325.7399 | 1325.7146 | -0.0253 | -19 | 373 | 383 | ELVHMIAWAKK | | | | | Mascot |
| 2239.0667 | 2239.0825 | 0.0158 | 7 | 2 | 24 | LAACVFPMVMSGGQTSG EAVGAR | | | | | Mascot |

| | | | | | | | | | | | |
|---|---|---|---|---|---|---|---|---|---|---|---|
| 2279.1377 | 2278.9968 | -0.1409 | -62 | 36 | 56 | EALEEPSSPPAAHKLSPMYPK | | | | | Mascot |
| 2371.0979 | 2371.0811 | -0.0168 | -7 | 433 | 454 | SEGECVEGMAEIFDMLLATVAR | | | | | Mascot |
| 2371.1091 | 2371.0811 | -0.028 | -12 | 353 | 372 | HSRPYTEITMMSLLTNMADK | | | Oxidation (M)[10,11] | | Mascot |
| 2439.2588 | 2439.0789 | -0.1799 | -74 | 554 | 574 | VPLYDLLLEMLDAQRFQSSGK | | | Oxidation (M)[10] | | Mascot |
| 2580.2478 | 2580.2556 | 0.0078 | 3 | 2 | 26 | LAACVFPMVMSGGQTSGEAVGARQR | | | Carbamidomethyl (C)[4] | | Mascot |
| 2580.2478 | 2580.2556 | 0.0078 | 3 | 2 | 26 | LAACVFPMVMSGGQTSGEAVGARQR | | | Carbamidomethyl (C)[4] | | Mascot |
| 2596.2429 | 2596.2539 | 0.011 | 4 | 2 | 26 | LAACVFPMVMSGGQTSGEAVGARQR | | | Carbamidomethyl (C)[4], Oxidation (M)[8] | | Mascot |
| 2596.2429 | 2596.2539 | 0.011 | 4 | 2 | 26 | LAACVFPMVMSGGQTSGEAVGARQR | | | Carbamidomethyl (C)[4], Oxidation (M)[8] | | Mascot |

43 PREDICTED: interferon-induced very large GTPase 1-like [Danio rerio] gi|326676497 194760.8 7 13 30 0 24 6.699

**Peptide Information**

| Calc. Mass | Obsrv. Mass | ± da | ± ppm | Start Seq. | End Seq. | Sequence | Ion Score | C. I. % | Modification | Rank | Result Type |
|---|---|---|---|---|---|---|---|---|---|---|---|
| 864.4178 | 864.4924 | 0.0746 | 86 | 551 | 556 | EMCRLR | | | Carbamidomethyl (C)[3] | | Mascot |
| 1082.5841 | 1082.4855 | -0.0986 | -91 | 1097 | 1105 | EVQTDIHIK | | | | | Mascot |
| 1106.5146 | 1106.5333 | 0.0187 | 17 | 1049 | 1058 | CEADNELGKK | | | | | Mascot |
| 1234.5708 | 1234.6538 | 0.083 | 67 | 1011 | 1019 | KHMIDWCTK | | | Carbamidomethyl (C)[7], Oxidation (M)[3] | | Mascot |
| 1347.6539 | 1347.6968 | 0.0429 | 32 | 621 | 631 | QHLSSLDEQYK | | | | | Mascot |
| 1475.7522 | 1475.7168 | -0.0354 | -24 | 428 | 440 | LVLVCNTNDLESR | | | | | Mascot |
| 1584.8124 | 1584.8403 | 0.0279 | 18 | 588 | 602 | QVISLFISGLCCSSK | 26 | 36.67 | | | Mascot |
| 1584.8124 | 1584.8403 | 0.0279 | 18 | 588 | 602 | QVISLFISGLCCSSK | | | | | Mascot |
| 1648.8481 | 1648.7839 | -0.0642 | -39 | 958 | 971 | VPAQDFVAFAEWIR | | | | | Mascot |
| 2225.0828 | 2225.0662 | -0.0166 | -7 | 464 | 482 | MNDDDFVAKLQMTVDEILK | | | | | Mascot |
| 2225.0828 | 2225.0662 | -0.0166 | -7 | 464 | 482 | MNDDDFVAKLQMTVDEILK | | | | | Mascot |
| 2284.0913 | 2284.1206 | 0.0293 | 13 | 297 | 316 | DCSGENSIVQTDFPLISFVR | | | Carbamidomethyl (C)[2] | | Mascot |
| 2297.0974 | 2297.1343 | 0.0369 | 16 | 156 | 174 | TNINMEYMLMELGLQSHLK | | | Oxidation (M)[5,8] | | Mascot |
| 2300.0759 | 2300.126 | 0.0501 | 22 | 356 | 375 | ICNGLVEMTWYLPCGKETDK | | | | | Mascot |
| 2371.1672 | 2371.0811 | -0.0861 | -36 | 1199 | 1220 | EMLSTVTSLDQCGIGPFFAVAK | | | Carbamidomethyl (C)[12] | | Mascot |
| 2371.1672 | 2371.0811 | -0.0861 | -36 | 1199 | 1220 | EMLSTVTSLDQCGIGPFFAVAK | | | Carbamidomethyl (C)[12] | | Mascot |

44 talin-1 [Danio rerio] gi|57222259 270469.6 5.86 21 30 0

**Peptide Information**

| Calc. Mass | Obsrv. Mass | ± da | ± ppm | Start Seq. | End Seq. | Sequence | Ion Score | C. I. % | Modification | Rank | Result Type |
|---|---|---|---|---|---|---|---|---|---|---|---|

| Calc. Mass | Obsrv. Mass | ± da | ± ppm | Start Seq. | End Seq. | Sequence | Ion Score | C. I. % | Modification | Rank | Result Type |
|---|---|---|---|---|---|---|---|---|---|---|---|
| 803.4257 | 803.3616 | -0.0641 | -80 | 1944 | 1950 | ELIDSAR | | | | | Mascot |
| 856.408 | 856.5068 | 0.0988 | 115 | 1303 | 1310 | TISMSSSK | | | Oxidation (M)[4] | | Mascot |
| 902.5053 | 902.4664 | -0.0389 | -43 | 2460 | 2467 | RASDNLVK | | | | | Mascot |
| 973.4883 | 973.4905 | 0.0022 | 2 | 1195 | 1203 | SVNCLPGQR | | | | | Mascot |
| 973.4883 | 973.4905 | 0.0022 | 2 | 1195 | 1203 | SVNCLPGQR | | | | | Mascot |
| 1082.4783 | 1082.4855 | 0.0072 | 7 | 1083 | 1092 | CSQDLGSSTK | | | Carbamidomethyl (C)[1] | | Mascot |
| 1165.6576 | 1165.7196 | 0.062 | 53 | 1615 | 1625 | SLAVNPKDPPK | | | | | Mascot |
| 1179.5422 | 1179.5769 | 0.0347 | 29 | 2441 | 2450 | ADQDSQTMKR | | | | | Mascot |
| 1213.6171 | 1213.5719 | -0.0452 | -37 | 1133 | 1144 | GVAANTEDPLAR | | | | | Mascot |
| 1234.5811 | 1234.6538 | 0.0727 | 59 | 1541 | 1551 | ALDGAFNQENR | | | | | Mascot |
| 1316.6804 | 1316.6663 | -0.0141 | -11 | 2193 | 2204 | QEDVIATANLSR | | | | | Mascot |
| 1325.7787 | 1325.7146 | -0.0641 | -48 | 438 | 450 | VETGSVALPAIIR | | | | | Mascot |
| 1325.7787 | 1325.7146 | -0.0641 | -48 | 438 | 450 | VETGSVALPAIIR | | | | | Mascot |
| 1347.705 | 1347.6968 | -0.0082 | -6 | 896 | 908 | MATNAAAQNAIKK | | | Oxidation (M)[1] | | Mascot |
| 1475.7886 | 1475.7168 | -0.0718 | -49 | 859 | 872 | LLADATARMVEAAK | | | Oxidation (M)[9] | | Mascot |
| 1638.8077 | 1638.8247 | 0.017 | 10 | 92 | 106 | MLDGTVKTVMVDDSK | | | | | Mascot |
| 2117.9768 | 2117.9324 | -0.0444 | -21 | 867 | 886 | MVEAAKGAAANPDSEEQQQK | | | Oxidation (M)[1] | | Mascot |
| 2211.1865 | 2211.0537 | -0.1328 | -60 | 2059 | 2080 | LGAASLGSEDPETQVVLINAVK | | | | | Mascot |
| 2225.1846 | 2225.0662 | -0.1184 | -53 | 718 | 738 | VVVAPTISSPVCQEQLIEAGK | | | Carbamidomethyl (C)[12] | | Mascot |
| 2225.1846 | 2225.0662 | -0.1184 | -53 | 718 | 738 | VVVAPTISSPVCQEQLIEAGK | | | Carbamidomethyl (C)[12] | | Mascot |
| 2233.0952 | 2233.0288 | -0.0664 | -30 | 1337 | 1357 | AVTDSINQLISMCTQQAPGQK | | | | | Mascot |
| 2263.1387 | 2263.0032 | -0.1355 | -60 | 1763 | 1783 | TLAESALQMLYTAKETGGNHK | | | | | Mascot |
| 2279.1335 | 2278.9968 | -0.1367 | -60 | 1763 | 1783 | TLAESALQMLYTAKETGGNHK | | | Oxidation (M)[9] | | Mascot |
| 2371.0825 | 2371.0811 | -0.0014 | -1 | 1145 | 1166 | NAMLDCTGDVMDKSASLIEETK | | | | | Mascot |
| 2371.0825 | 2371.0811 | -0.0014 | -1 | 1145 | 1166 | NAMLDCTGDVMDKSASLIEETK | | | | | Mascot |
| 2532.1453 | 2532.2136 | 0.0683 | 27 | 2394 | 2418 | MVAAATNNLCEAANSAVQGHASEEK | | | Oxidation (M)[1] | | Mascot |

| | | | | | | | |
|---|---|---|---|---|---|---|---|
| 45 | ATP-dependent chromatin remodeling protein SNF2-related protein [Danio rerio] | gi\|21105437 | 7534.1 | 10.57 | 4 | 30 | 0 |

**Peptide Information**

| Calc. Mass | Obsrv. Mass | ± da | ± ppm | Start Seq. | End Seq. | Sequence | Ion Score | C. I. % | Modification | Rank | Result Type |
|---|---|---|---|---|---|---|---|---|---|---|---|
| 864.4243 | 864.4924 | 0.0681 | 79 | 4 | 10 | TAMELQR | | | Oxidation (M)[3] | | Mascot |
| 973.4697 | 973.4905 | 0.0208 | 21 | 48 | 56 | KQDGTPDGR | | | | | Mascot |

| Calc. Mass | Obsrv. Mass | ± da | ± ppm | Start Seq. | End Seq. | Sequence | Ion Score | C. I. % | Modification | Rank | Result Type |
|---|---|---|---|---|---|---|---|---|---|---|---|
| 973.4697 | 973.4905 | 0.0208 | 21 | 48 | 56 | KQDGTPDGR | | | | | Mascot |
| 1058.4973 | 1058.5662 | 0.0689 | 65 | 49 | 58 | QDGTPDGRGR | | | | | Mascot |
| 2263.1057 | 2263.0032 | -0.1025 | -45 | 12 | 29 | CNTLITLIERENMELEER | | | Carbamidomethyl (C)[1] | | Mascot |
| 2279.1006 | 2278.9968 | -0.1038 | -46 | 12 | 29 | CNTLITLIERENMELEER | | | Carbamidomethyl (C)[1], Oxidation (M)[13] | | Mascot |

| | | | | | | | |
|---|---|---|---|---|---|---|---|
| 46 | novel protein similar to vertebrate cytoplasmic linker associated protein 1 (CLASP1) [Danio rerio] | gi\|159569348 | 164674.4 | 8.99 | 17 | 30 | 0 |

**Peptide Information**

| Calc. Mass | Obsrv. Mass | ± da | ± ppm | Start Seq. | End Seq. | Sequence | Ion Score | C. I. % | Modification | Rank | Result Type |
|---|---|---|---|---|---|---|---|---|---|---|---|
| 856.5502 | 856.5068 | -0.0434 | -51 | 1277 | 1284 | GALIELLK | | | | | Mascot |
| 870.577 | 870.5187 | -0.0583 | -67 | 1323 | 1329 | VLKEILR | | | | | Mascot |
| 1082.5551 | 1082.4855 | -0.0696 | -64 | 1337 | 1345 | NYAELTIMK | | | | | Mascot |
| 1165.6801 | 1165.7196 | 0.0395 | 34 | 1313 | 1322 | DHTIRALALR | | | | | Mascot |
| 1325.524 | 1325.7146 | 0.1906 | 144 | 239 | 250 | NFDDEDSVDGGR | | | | | Mascot |
| 1325.8151 | 1325.7146 | -0.1005 | -76 | 855 | 866 | KEGLLGLQNLLK | | | | | Mascot |
| 1434.6715 | 1434.7355 | 0.064 | 45 | 1240 | 1251 | QDCGENKMVLPK | | | Carbamidomethyl (C)[3], Oxidation (M)[8] | | Mascot |
| 1475.6802 | 1475.7168 | 0.0366 | 25 | 1288 | 1299 | EDSLAVWDEHFK | | | | | Mascot |
| 1648.8007 | 1648.7839 | -0.0168 | -10 | 727 | 741 | SSRIPRPSMSQGCSR | | | | | Mascot |
| 1728.9126 | 1728.7454 | -0.1672 | -97 | 780 | 796 | VLNTGTEVEAAVADALR | | | | | Mascot |
| 2001.0222 | 2000.9335 | -0.0887 | -44 | 347 | 364 | SLLMAGATEHEGFIQQLR | | | | | Mascot |
| 2053.9502 | 2053.9241 | -0.0261 | -13 | 1202 | 1219 | SREFAPYGYGDTITAYDK | | | | | Mascot |
| 2225.207 | 2225.0662 | -0.1408 | -63 | 379 | 399 | SQVVREACITLGHLSSLL GNK | | | | | Mascot |
| 2225.207 | 2225.0662 | -0.1408 | -63 | 379 | 399 | SQVVREACITLGHLSSLL GNK | | | | | Mascot |
| 2284.0769 | 2284.1206 | 0.0437 | 19 | 1 | 19 | MEPNMEYCLTQVLQKDV AR | | | Oxidation (M)[1] | | Mascot |
| 2300.072 | 2300.126 | 0.054 | 23 | 1 | 19 | MEPNMEYCLTQVLQKDV AR | | | Oxidation (M)[1,5] | | Mascot |
| 2371.1572 | 2371.0811 | -0.0761 | -32 | 118 | 137 | IMEQSATPQYIWDRMLG GFK | | | | | Mascot |
| 2371.1572 | 2371.0811 | -0.0761 | -32 | 118 | 137 | IMEQSATPQYIWDRMLG GFK | | | | | Mascot |
| 3218.4617 | 3218.5154 | 0.0537 | 17 | 37 | 65 | CQDLESDQTALDKMVDG IATSWVNSSNFK | | | Oxidation (M)[14] | | Mascot |
| 3218.4617 | 3218.5154 | 0.0537 | 17 | 37 | 65 | CQDLESDQTALDKMVDG IATSWVNSSNFK | | | Oxidation (M)[14] | | Mascot |
| 3338.7485 | 3338.6836 | -0.0649 | -19 | 50 | 80 | MVDGIATSWVNSSNFKL ALLGMDLLSALVTR | | | Oxidation (M)[1] | | Mascot |

| | | | | | | | | | |
|---|---|---|---|---|---|---|---|---|---|
| 47 | PREDICTED: inositol 1,4,5-trisphosphate receptor type 2, partial [Danio rerio] | gi\|326676026 | 226480.1 | 6.12 | 17 | 30 | 0 | 15 | 0 |

**Peptide Information**

| Calc. Mass | Obsrv. Mass | ± da | ± ppm | Start Seq. | End Seq. | Sequence | Ion Score | C. I. % | Modification | Rank | Result Type |
|---|---|---|---|---|---|---|---|---|---|---|---|

| Calc. Mass | Obsrv. Mass | ± da | ± ppm | Start Seq. | End Seq. | Sequence | Ion Score | C. I. % | Modification | Rank | Result Type |
|---|---|---|---|---|---|---|---|---|---|---|---|
| 803.4443 | 803.3616 | -0.0827 | -103 | 1188 | 1194 | LCSPAKK | | | Carbamidomethyl (C)[2] | | Mascot |
| 902.5557 | 902.4664 | -0.0893 | -99 | 1180 | 1187 | EILLSLSK | | | | | Mascot |
| 973.5353 | 973.4905 | -0.0448 | -46 | 807 | 814 | LWTEIPSK | | | | | Mascot |
| 973.5353 | 973.4905 | -0.0448 | -46 | 807 | 814 | LWTEIPSK | | | | | Mascot |
| 1213.6113 | 1213.5719 | -0.0394 | -32 | 66 | 74 | YSAQRQFWK | | | | | Mascot |
| 1234.5554 | 1234.6538 | 0.0984 | 80 | 422 | 431 | IGTCHMKEDK | | | Carbamidomethyl (C)[4], Oxidation (M)[6] | | Mascot |
| 1325.6154 | 1325.7146 | 0.0992 | 75 | 1675 | 1685 | EMFDEKGNALR | 17 | 0 | Oxidation (M)[2] | | Mascot |
| 1325.6163 | 1325.7146 | 0.0983 | 74 | 55 | 65 | DCLFKVCPMNR | | | | | Mascot |
| 1434.6536 | 1434.7355 | 0.0819 | 57 | 815 | 825 | ISVNEYDYEFR | | | | | Mascot |
| 1475.7498 | 1475.7168 | -0.033 | -22 | 556 | 566 | YMLQLCYRTLR | | | Oxidation (M)[2] | | Mascot |
| 1584.8129 | 1584.8403 | 0.0274 | 17 | 236 | 249 | GGDVVRLFHAEQEK | | | | | Mascot |
| 1584.8129 | 1584.8403 | 0.0274 | 17 | 236 | 249 | GGDVVRLFHAEQEK | | | | | Mascot |
| 1648.7812 | 1648.7839 | 0.0027 | 2 | 1043 | 1057 | SPVDLDDEGGQTFLR | | | | | Mascot |
| 1844.8969 | 1844.8348 | -0.0621 | -34 | 940 | 956 | GLMMPHCLPDAQPIHGK | | | | | Mascot |
| 1844.8969 | 1844.8348 | -0.0621 | -34 | 940 | 956 | GLMMPHCLPDAQPIHGK | | | | | Mascot |
| 2000.9423 | 2000.9335 | -0.0088 | -4 | 242 | 257 | LFHAEQEKFLTCDEYK | | | | | Mascot |
| 2053.875 | 2053.9241 | 0.0491 | 24 | 768 | 784 | CMFDDCLPYNLRASFCR | | | | | Mascot |
| 2224.9395 | 2225.0662 | 0.1267 | 57 | 768 | 784 | CMFDDCLPYNLRASFCR | | | Carbamidomethyl (C)[1,6,16] | | Mascot |
| 2224.9395 | 2225.0662 | 0.1267 | 57 | 768 | 784 | CMFDDCLPYNLRASFCR | | | Carbamidomethyl (C)[1,6,16] | | Mascot |
| 2247.1477 | 2247.0203 | -0.1274 | -57 | 726 | 743 | LDTDIITYYRCQLNLFAK | | | Carbamidomethyl (C)[11] | | Mascot |
| 2367.0024 | 2367.2065 | 0.2041 | 86 | 1269 | 1287 | TMCYIFMNNYHLCNEISD R | | | | | Mascot |
| 2455.2107 | 2455.083 | -0.1277 | -52 | 1512 | 1531 | LYNCTWLNSLQKTNIEGC IK | | | Carbamidomethyl (C)[4,18] | | Mascot |

| | | | | | | | |
|---|---|---|---|---|---|---|---|
| 48 | PREDICTED: tripartite motif-containing protein 35-like [Danio rerio] | gi\|326678845 | 52995.6 | 8.51 | 10 | 30 | 0 |

Peptide Information

| Calc. Mass | Obsrv. Mass | ± da | ± ppm | Start Seq. | End Seq. | Sequence | Ion Score | C. I. % | Modification | Rank | Result Type |
|---|---|---|---|---|---|---|---|---|---|---|---|
| 856.4523 | 856.5068 | 0.0545 | 64 | 111 | 117 | DSEKHLK | | | | | Mascot |
| 1045.5887 | 1045.5409 | -0.0478 | -46 | 131 | 139 | KEELNTALK | | | | | Mascot |
| 1082.4976 | 1082.4855 | -0.0121 | -11 | 33 | 40 | ECLQEFWK | | | | | Mascot |
| 1106.4718 | 1106.5333 | 0.0615 | 56 | 43 | 51 | NTQECPVCR | | | Carbamidomethyl (C)[5] | | Mascot |
| 1347.6508 | 1347.6968 | 0.046 | 34 | 41 | 51 | IKNTQECPVCR | | | Carbamidomethyl (C)[7] | | Mascot |
| 2211.1372 | 2211.0537 | -0.0835 | -38 | 52 | 71 | RPSMADPPVNLALKNLC ESR | | | | | Mascot |
| 2284.1536 | 2284.1206 | -0.033 | -14 | 52 | 71 | RPSMADPPVNLALKNLC ESR | | | Carbamidomethyl (C)[17], Oxidation (M)[4] | | Mascot |

| Calc. Mass | Obsrv. Mass | ± da | ± ppm | Start Seq. | End Seq. | Sequence | Ion Score | C. I. % | Modification | Rank | Result Type |
|---|---|---|---|---|---|---|---|---|---|---|---|
| 2296.9922 | 2297.1343 | 0.1421 | 62 | 1 | 19 | MASSAEYDYICPVCQDIFK | | | Carbamidomethyl (C)[11,14] | | Mascot |
| 2455.1606 | 2455.083 | -0.0776 | -32 | 20 | 40 | TPVILSCGHSFCKECLQEFWK | | | | | Mascot |
| 2596.2793 | 2596.2539 | -0.0254 | -10 | 43 | 65 | NTQECPVCRRPSMADPPVNLALK | | | Carbamidomethyl (C)[5] | | Mascot |
| 2596.2793 | 2596.2539 | -0.0254 | -10 | 43 | 65 | NTQECPVCRRPSMADPPVNLALK | | | Carbamidomethyl (C)[5] | | Mascot |
| 3338.6572 | 3338.6836 | 0.0264 | 8 | 281 | 309 | MQDIVQYTPVILNPNTAHPELELSDDLTR | | | Oxidation (M)[1] | | Mascot |

| | | | | | | | | | |
|---|---|---|---|---|---|---|---|---|---|
| 49 | Arginyl-tRNA synthetase [Danio rerio] | gi\|53734470 | 75716.9 | 6.22 | 12 | 30 | 0 | | |

**Peptide Information**

| Calc. Mass | Obsrv. Mass | ± da | ± ppm | Start Seq. | End Seq. | Sequence | Ion Score | C. I. % | Modification | Rank | Result Type |
|---|---|---|---|---|---|---|---|---|---|---|---|
| 973.4948 | 973.4905 | -0.0043 | -4 | 354 | 362 | GLVEVDEGR | | | | | Mascot |
| 973.4948 | 973.4905 | -0.0043 | -4 | 354 | 362 | GLVEVDEGR | | | | | Mascot |
| 1058.4313 | 1058.5662 | 0.1349 | 127 | 281 | 288 | FDEDEEFK | | | | | Mascot |
| 1082.4969 | 1082.4855 | -0.0114 | -11 | 215 | 224 | STIIGDSMCR | | | | | Mascot |
| 1316.7242 | 1316.6663 | -0.0579 | -44 | 463 | 473 | LMDLLEEGLKR | | | | | Mascot |
| 1475.7675 | 1475.7168 | -0.0507 | -34 | 649 | 661 | GFDILGINPVQRM | | | Oxidation (M)[13] | | Mascot |
| 1584.838 | 1584.8403 | 0.0023 | 1 | 437 | 451 | VEHAGFGVVLGEDKK | | | | | Mascot |
| 1584.838 | 1584.8403 | 0.0023 | 1 | 437 | 451 | VEHAGFGVVLGEDKK | | | | | Mascot |
| 1638.7904 | 1638.8247 | 0.0343 | 21 | 1 | 14 | MGDPVTEYASRLQR | | | Oxidation (M)[1] | | Mascot |
| 1729.9384 | 1729.7338 | -0.2046 | -118 | 529 | 543 | GNTAAYLLYAFTRIR | | | | | Mascot |
| 2047.7687 | 2047.9043 | 0.1356 | 66 | 111 | 127 | FGDYQCNSAMAMSQMMK | | | Carbamidomethyl (C)[6], Oxidation (M)[10,12,15] | | Mascot |
| 2054.0054 | 2053.9241 | -0.0813 | -40 | 631 | 648 | VNMWRMLLCEATAAVMAK | | | Oxidation (M)[3] | | Mascot |
| 2246.9006 | 2247.0203 | 0.1197 | 53 | 111 | 129 | FGDYQCNSAMAMSQMMKAK | | | Carbamidomethyl (C)[6], Oxidation (M)[10,12,15] | | Mascot |
| 2262.8955 | 2263.0032 | 0.1077 | 48 | 111 | 129 | FGDYQCNSAMAMSQMMKAK | | | Carbamidomethyl (C)[6], Oxidation (M)[10,12,15,16] | | Mascot |
| 2284.0149 | 2284.1206 | 0.1057 | 46 | 335 | 353 | GESYYQDMMTAVVKEFEEK | | | | | Mascot |
| 2300.0098 | 2300.126 | 0.1162 | 51 | 335 | 353 | GESYYQDMMTAVVKEFEEK | | | Oxidation (M)[8] | | Mascot |

| | | | | | | | | | |
|---|---|---|---|---|---|---|---|---|---|
| 50 | si:dkey-189g17.2 [Danio rerio] | gi\|190570242 | 106521.3 | 5.47 | 1 | | | 26 | 41.033 |

**Peptide Information**

| Calc. Mass | Obsrv. Mass | ± da | ± ppm | Start Seq. | End Seq. | Sequence | Ion Score | C. I. % | Modification | Rank | Result Type |
|---|---|---|---|---|---|---|---|---|---|---|---|
| 1325.7899 | 1325.7146 | -0.0753 | -57 | 914 | 924 | QRIVDALLELR | 26 | 41.033 | | 2 | Mascot |

| Gel Idx/Pos | 186/H13 | Instr./Gel Origin | BA2060/110707A | Process Status | Analysis Succeeded |
|---|---|---|---|---|---|
| Plate [#] Name | [1] 31496 | Instrument Sample Name | | Spectra | 11 |

| Rank | Protein Name | Accession No. | Protein MW | Protein PI | Pep. Count | Protein Score | Protein Score C. I. % | Total Ion Score | Total Ion C. I. % |
|---|---|---|---|---|---|---|---|---|---|
| 1 | hypothetical protein LOC559969 [Danio rerio] | gi\|113678532 | 77970.3 | 6.17 | 12 | 419 | 100 | 375 | 100 |

Peptide Information

| Calc. Mass | Obsrv. Mass | ± da | ± ppm | Start Seq. | End Seq. | Sequence | Ion Score | C. I. % | Modification | Rank | Result Type |
|---|---|---|---|---|---|---|---|---|---|---|---|
| 973.5101 | 973.4948 | -0.0153 | -16 | 424 | 431 | LVVWGEDR | | | | | Mascot |
| 973.5101 | 973.4948 | -0.0153 | -16 | 424 | 431 | LVVWGEDR | 23 | 0 | | | Mascot |
| 1058.5887 | 1058.5728 | -0.0159 | -15 | 531 | 538 | RLNVLQCR | | | Carbamidomethyl (C)[7] | | Mascot |
| 1325.7423 | 1325.719 | -0.0233 | -18 | 355 | 365 | LPLSQEQIELR | 39 | 96.544 | | | Mascot |
| 1325.7423 | 1325.719 | -0.0233 | -18 | 355 | 365 | LPLSQEQIELR | | | | | Mascot |
| 1563.8376 | 1563.8088 | -0.0288 | -18 | 272 | 286 | IIEEAPGPGISPEVR | | | | | Mascot |
| 1563.8376 | 1563.8088 | -0.0288 | -18 | 272 | 286 | IIEEAPGPGISPEVR | 68 | 99.996 | | | Mascot |
| 1584.8744 | 1584.8456 | -0.0288 | -18 | 131 | 145 | QEGIIFIGPPSSAIR | 51 | 99.799 | | | Mascot |
| 1584.8744 | 1584.8456 | -0.0288 | -18 | 131 | 145 | QEGIIFIGPPSSAIR | | | | | Mascot |
| 1773.968 | 1773.7863 | -0.1817 | -102 | 1 | 16 | MAAVFLKTHINGTINK | | | Oxidation (M)[1] | | Mascot |
| 1844.8773 | 1844.845 | -0.0323 | -18 | 204 | 219 | IAQSEAEFHEQLESAR | 75 | 100 | | | Mascot |
| 1844.8773 | 1844.845 | -0.0323 | -18 | 204 | 219 | IAQSEAEFHEQLESAR | | | | | Mascot |
| 1881.0439 | 1881.0011 | -0.0428 | -23 | 349 | 365 | VAAGEKLPLSQEQIELR | | | | | Mascot |
| 2117.9807 | 2117.9473 | -0.0334 | -16 | 549 | 568 | VAVSITYNPDGTYSMETG GR | | | | | Mascot |
| 2371.1353 | 2371.0925 | -0.0428 | -18 | 242 | 261 | HVEVQVFGDQYGDAVYL FER | | | | | Mascot |
| 2371.1353 | 2371.0925 | -0.0428 | -18 | 242 | 261 | HVEVQVFGDQYGDAVYL FER | 118 | 100 | | | Mascot |
| 2580.3127 | 2580.2661 | -0.0466 | -18 | 327 | 348 | LQVEHPVSEMITGTDLVE WQLR | | | | | Mascot |
| 2596.3076 | 2596.2666 | -0.041 | -16 | 327 | 348 | LQVEHPVSEMITGTDLVE WQLR | | | Oxidation (M)[10] | | Mascot |
| 3227.5869 | 3227.5364 | -0.0505 | -16 | 373 | 402 | IYAEDPNNNFLPGAGPLL HLSTPPGDHNTR | | | | | Mascot |

| Rank | Protein Name | Accession No. | Protein MW | Protein PI | Pep. Count | Protein Score | Protein Score C. I. % | Total Ion Score | Total Ion C. I. % |
|---|---|---|---|---|---|---|---|---|---|
| 2 | hypothetical protein LOC792190 [Danio rerio] | gi\|224495957 | 78018.4 | 6.21 | 11 | 412 | 100 | 375 | 100 |

Peptide Information

| Calc. Mass | Obsrv. Mass | ± da | ± ppm | Start Seq. | End Seq. | Sequence | Ion Score | C. I. % | Modification | Rank | Result Type |
|---|---|---|---|---|---|---|---|---|---|---|---|
| 973.5101 | 973.4948 | -0.0153 | -16 | 424 | 431 | LVVWGEDR | | | | | Mascot |
| 973.5101 | 973.4948 | -0.0153 | -16 | 424 | 431 | LVVWGEDR | 23 | 0 | | | Mascot |

| | | | | | | | | | | | |
|---|---|---|---|---|---|---|---|---|---|---|---|
| 1058.5887 | 1058.5728 | -0.0159 | -15 | 531 | 538 | RLNVLQCR | | | Carbamidomethyl (C)[7] | | Mascot |
| 1325.7423 | 1325.719 | -0.0233 | -18 | 355 | 365 | LPLSQEQIELR | 39 | 96.544 | | | Mascot |
| 1325.7423 | 1325.719 | -0.0233 | -18 | 355 | 365 | LPLSQEQIELR | | | | | Mascot |
| 1563.8376 | 1563.8088 | -0.0288 | -18 | 272 | 286 | IIEEAPGPGISPEVR | | | | | Mascot |
| 1563.8376 | 1563.8088 | -0.0288 | -18 | 272 | 286 | IIEEAPGPGISPEVR | 68 | 99.996 | | | Mascot |
| 1584.8744 | 1584.8456 | -0.0288 | -18 | 131 | 145 | QEGIIFIGPPSSAIR | 51 | 99.799 | | | Mascot |
| 1584.8744 | 1584.8456 | -0.0288 | -18 | 131 | 145 | QEGIIFIGPPSSAIR | | | | | Mascot |
| 1773.968 | 1773.7863 | -0.1817 | -102 | 1 | 16 | MAAVFLKTHINGTINK | | | Oxidation (M)[1] | | Mascot |
| 1844.8773 | 1844.845 | -0.0323 | -18 | 204 | 219 | IAQSEAEFHEQLESAR | 75 | 100 | | | Mascot |
| 1844.8773 | 1844.845 | -0.0323 | -18 | 204 | 219 | IAQSEAEFHEQLESAR | | | | | Mascot |
| 1881.0439 | 1881.0011 | -0.0428 | -23 | 349 | 365 | VAAGEKLPLSQEQIELR | | | | | Mascot |
| 2117.9807 | 2117.9473 | -0.0334 | -16 | 549 | 568 | VAVSITYNPDGTYSMETGGR | | | | | Mascot |
| 2371.1353 | 2371.0925 | -0.0428 | -18 | 242 | 261 | HVEVQVFGDQYGDAVYLFER | | | | | Mascot |
| 2371.1353 | 2371.0925 | -0.0428 | -18 | 242 | 261 | HVEVQVFGDQYGDAVYLFER | 118 | 100 | | | Mascot |
| 2580.3127 | 2580.2661 | -0.0466 | -18 | 327 | 348 | LQVEHPVSEMITGTDLVEWQLR | | | | | Mascot |
| 2596.3076 | 2596.2666 | -0.041 | -16 | 327 | 348 | LQVEHPVSEMITGTDLVEWQLR | | | Oxidation (M)[10] | | Mascot |

3 hypothetical protein LOC777632 [Danio rerio] gi|118150458 77993.3 6.12 10 406 100 375 100

**Peptide Information**

| Calc. Mass | Obsrv. Mass | ± da | ± ppm | Start Seq. | End Seq. | Sequence | Ion Score | C. I. % | Modification | Rank | Result Type |
|---|---|---|---|---|---|---|---|---|---|---|---|
| 973.5101 | 973.4948 | -0.0153 | -16 | 424 | 431 | LVVWGEDR | | | | | Mascot |
| 973.5101 | 973.4948 | -0.0153 | -16 | 424 | 431 | LVVWGEDR | 23 | 0 | | | Mascot |
| 1058.5887 | 1058.5728 | -0.0159 | -15 | 531 | 538 | RLNVLQCR | | | Carbamidomethyl (C)[7] | | Mascot |
| 1325.7423 | 1325.719 | -0.0233 | -18 | 355 | 365 | LPLSQEQIELR | 39 | 96.544 | | | Mascot |
| 1325.7423 | 1325.719 | -0.0233 | -18 | 355 | 365 | LPLSQEQIELR | | | | | Mascot |
| 1563.8376 | 1563.8088 | -0.0288 | -18 | 272 | 286 | IIEEAPGPGISPEVR | | | | | Mascot |
| 1563.8376 | 1563.8088 | -0.0288 | -18 | 272 | 286 | IIEEAPGPGISPEVR | 68 | 99.996 | | | Mascot |
| 1584.8744 | 1584.8456 | -0.0288 | -18 | 131 | 145 | QEGIIFIGPPSSAIR | 51 | 99.799 | | | Mascot |
| 1584.8744 | 1584.8456 | -0.0288 | -18 | 131 | 145 | QEGIIFIGPPSSAIR | | | | | Mascot |
| 1773.968 | 1773.7863 | -0.1817 | -102 | 1 | 16 | MAAVFLKTHINGTINK | | | Oxidation (M)[1] | | Mascot |
| 1844.8773 | 1844.845 | -0.0323 | -18 | 204 | 219 | IAQSEAEFHEQLESAR | 75 | 100 | | | Mascot |
| 1844.8773 | 1844.845 | -0.0323 | -18 | 204 | 219 | IAQSEAEFHEQLESAR | | | | | Mascot |
| 1881.0439 | 1881.0011 | -0.0428 | -23 | 349 | 365 | VAAGEKLPLSQEQIELR | | | | | Mascot |
| 2371.1353 | 2371.0925 | -0.0428 | -18 | 242 | 261 | HVEVQVFGDQYGDAVYLFER | | | | | Mascot |
| 2371.1353 | 2371.0925 | -0.0428 | -18 | 242 | 261 | HVEVQVFGDQYGDAVYL | 118 | 100 | | | Mascot |

| Calc. Mass | Obsrv. Mass | ± da | ± ppm | Start Seq. | End Seq. | Sequence | Ion Score | C. I. % | Modification | Rank | Result Type |
|---|---|---|---|---|---|---|---|---|---|---|---|
| | | | | | | FER | | | | | |
| 2580.3127 | 2580.2661 | -0.0466 | -18 | 327 | 348 | LQVEHPVSEMITGTDLVE WQLR | | | | | Mascot |
| 2596.3076 | 2596.2666 | -0.041 | -16 | 327 | 348 | LQVEHPVSEMITGTDLVE WQLR | | | Oxidation (M)[10] | | Mascot |

4 SH3 domain-binding protein 4-A [Danio rerio] gi|45387913 104947.5 7.73 14 40 0

Peptide Information

| Calc. Mass | Obsrv. Mass | ± da | ± ppm | Start Seq. | End Seq. | Sequence | Ion Score | C. I. % | Modification | Rank | Result Type |
|---|---|---|---|---|---|---|---|---|---|---|---|
| 842.5822 | 842.495 | -0.0872 | -103 | 634 | 640 | LIKTVIR | | | | | Mascot |
| 850.4338 | 850.5255 | 0.0917 | 108 | 358 | 364 | LSNMEIK | | | Oxidation (M)[4] | | Mascot |
| 870.4726 | 870.5257 | 0.0531 | 61 | 1 | 7 | MAAHRIR | | | Oxidation (M)[1] | | Mascot |
| 973.5829 | 973.4948 | -0.0881 | -90 | 666 | 673 | LKGQLWTK | | | | | Mascot |
| 973.5829 | 973.4948 | -0.0881 | -90 | 666 | 673 | LKGQLWTK | | | | | Mascot |
| 1045.4796 | 1045.5449 | 0.0653 | 62 | 762 | 770 | AELDSEPER | | | | | Mascot |
| 1434.7158 | 1434.745 | 0.0292 | 20 | 885 | 896 | DVIHELHMGLDR | | | | | Mascot |
| 1657.8651 | 1657.7596 | -0.1055 | -64 | 365 | 378 | SFITLEMKVSVMTR | | | Oxidation (M)[7] | | Mascot |
| 2118.1958 | 2117.9473 | -0.2485 | -117 | 607 | 624 | IILSPLAITTKYPQFQER | | | | | Mascot |
| 2230.2417 | 2230.1536 | -0.0881 | -40 | 813 | 831 | LIMDFVLLTTAVEVAPRW R | | | | | Mascot |
| 2284.2368 | 2284.1323 | -0.1045 | -46 | 463 | 484 | TVVAIFGHDCAPKTLLVS EVGK | | | | | Mascot |
| 2300.0686 | 2300.1367 | 0.0681 | 30 | 568 | 587 | DDQDCILAQFCVQTPQP PPK | | | Carbamidomethyl (C)[5] | | Mascot |
| 3227.677 | 3227.5364 | -0.1406 | -44 | 907 | 936 | HLTGTLILVNCLDLLRSSA FSPAPQDDFAI | | | | | Mascot |
| 3337.6052 | 3337.6936 | 0.0884 | 26 | 269 | 298 | LARSCHDLESLGQNPGW GQTQPVETNIVCK | | | Carbamidomethyl (C)[5] | | Mascot |
| 3338.5496 | 3338.7061 | 0.1565 | 47 | 856 | 884 | TGMVDSEAMWKPAYDFL LTWAAQIGDSYR | | | Oxidation (M)[3] | | Mascot |

5 PREDICTED: bromodomain adjacent to zinc finger domain protein 1A, partial [Danio rerio] gi|125841384 81560.8 6.03 12 40 0

Peptide Information

| Calc. Mass | Obsrv. Mass | ± da | ± ppm | Start Seq. | End Seq. | Sequence | Ion Score | C. I. % | Modification | Rank | Result Type |
|---|---|---|---|---|---|---|---|---|---|---|---|
| 1045.3999 | 1045.5449 | 0.145 | 139 | 303 | 310 | EQMMSMEK | | | Oxidation (M)[3,4] | | Mascot |
| 1106.4789 | 1106.5375 | 0.0586 | 53 | 36 | 43 | TYDEFFER | | | | | Mascot |
| 1234.7782 | 1234.6344 | -0.1438 | -116 | 2 | 11 | PLLHKKPFVR | | | | | Mascot |
| 1277.7939 | 1277.6868 | -0.1071 | -84 | 393 | 403 | ELPKPIPVKTR | | | | | Mascot |
| 1316.5796 | 1316.6753 | 0.0957 | 73 | 303 | 312 | EQMMSMEKFR | | | | | Mascot |
| 1633.8003 | 1633.802 | 0.0017 | 1 | 535 | 550 | LHILSSGADCGFTNAK | | | | | Mascot |
| 1657.7417 | 1657.7596 | 0.0179 | 11 | 298 | 310 | LMQQKEQMMSMEK | | | Oxidation (M)[2] | | Mascot |

| | | | | | | | | | | | |
|---|---|---|---|---|---|---|---|---|---|---|---|
| 1838.8741 | 1838.8802 | 0.0061 | 3 | 101 | 115 | LHEICDDVYAYVKDR | | | | | Mascot |
| 1845.0084 | 1844.845 | -0.1634 | -89 | 596 | 611 | ILHSMCGKLLTLVSTR | | | Carbamidomethyl (C)[6], Oxidation (M)[5] | | Mascot |
| 1845.0084 | 1844.845 | -0.1634 | -89 | 596 | 611 | ILHSMCGKLLTLVSTR | | | Carbamidomethyl (C)[6], Oxidation (M)[5] | | Mascot |
| 1864.8568 | 1864.9264 | 0.0696 | 37 | 378 | 392 | LWSKPREDMECDDLK | | | | | Mascot |
| 1880.8517 | 1881.0011 | 0.1494 | 79 | 378 | 392 | LWSKPREDMECDDLK | | | Oxidation (M)[9] | | Mascot |
| 1993.9912 | 1993.9388 | -0.0524 | -26 | 535 | 552 | LHILSSGADCGFTNAKFR | | | Carbamidomethyl (C)[10] | | Mascot |
| 2283.1836 | 2283.1423 | -0.0413 | -18 | 195 | 215 | VQPVSPEACEPVIVKVAQ MSR | | | Oxidation (M)[19] | | Mascot |
| 2283.1836 | 2283.1423 | -0.0413 | -18 | 195 | 215 | VQPVSPEACEPVIVKVAQ MSR | | | Oxidation (M)[19] | | Mascot |

| | | | | | | | |
|---|---|---|---|---|---|---|---|
| 6 | MCM6 minichromosome maintenance deficient 6, like [Danio rerio] | gi\|70887711 | 93152.1 | 5.23 | 13 | 39 | 0 |

Peptide Information

| Calc. Mass | Obsrv. Mass | ± da | ± ppm | Start Seq. | End Seq. | Sequence | Ion Score | C. I. % | Modification | Rank | Result Type |
|---|---|---|---|---|---|---|---|---|---|---|---|
| 856.49 | 856.5079 | 0.0179 | 21 | 87 | 93 | AVRHFAR | | | | | Mascot |
| 973.5789 | 973.4948 | -0.0841 | -86 | 117 | 124 | IRELSTVR | | | | | Mascot |
| 973.5789 | 973.4948 | -0.0841 | -86 | 117 | 124 | IRELSTVR | | | | | Mascot |
| 996.5261 | 996.58 | 0.0539 | 54 | 194 | 201 | FVDFQKGR | | | | | Mascot |
| 996.5261 | 996.58 | 0.0539 | 54 | 194 | 201 | FVDFQKGR | | | | | Mascot |
| 1165.6252 | 1165.724 | 0.0988 | 85 | 747 | 755 | SELINWYLK | | | | | Mascot |
| 1165.6252 | 1165.724 | 0.0988 | 85 | 747 | 755 | SELINWYLK | | | | | Mascot |
| 1234.7228 | 1234.6344 | -0.0884 | -72 | 361 | 372 | GILLMLFGGVAK | | | Oxidation (M)[5] | | Mascot |
| 1277.6195 | 1277.6868 | 0.0673 | 53 | 321 | 330 | NQMTVLEWEK | | | | | Mascot |
| 1325.7899 | 1325.719 | -0.0709 | -53 | 212 | 223 | GSIPRSVEVILR | | | | | Mascot |
| 1325.7899 | 1325.719 | -0.0709 | -53 | 212 | 223 | GSIPRSVEVILR | | | | | Mascot |
| 1347.6508 | 1347.6898 | 0.039 | 29 | 512 | 523 | QNVNMSAPIMSR | | | | | Mascot |
| 1475.7502 | 1475.7279 | -0.0223 | -15 | 90 | 102 | HFARDHGNIPPSK | | | | | Mascot |
| 1707.8306 | 1707.7456 | -0.085 | -50 | 168 | 182 | YTQPTICKNPVCANR | | | | | Mascot |
| 2211.1875 | 2211.0635 | -0.124 | -56 | 361 | 381 | GILLMLFGGVAKTTMEGT SLR | | | Oxidation (M)[5] | | Mascot |
| 2297.1812 | 2297.1516 | -0.0296 | -13 | 575 | 593 | QFQPKITVEAQEFVVDQY K | | | | | Mascot |
| 2580.2949 | 2580.2661 | -0.0288 | -11 | 138 | 160 | THPVHPELVSGTFLCLDC QSVIK | | | Carbamidomethyl (C)[15] | | Mascot |

| | | | | | | | |
|---|---|---|---|---|---|---|---|
| 7 | Synbl protein [Danio rerio] | gi\|141796272 | 31226.2 | 5.37 | 8 | 38 | 0 |

Peptide Information

| Calc. Mass | Obsrv. Mass | ± da | ± ppm | Start Seq. | End Seq. | Sequence | Ion Score | C. I. % | Modification | Rank | Result Type |
|---|---|---|---|---|---|---|---|---|---|---|---|

| | | | | | | | | | | | |
|---|---|---|---|---|---|---|---|---|---|---|---|
| 842.5709 | 842.495 | -0.0759 | -90 | 18 | 24 | VEKLLLK | | | | | Mascot |
| 1045.6769 | 1045.5449 | -0.132 | -126 | 171 | 179 | LLFLLSALR | | | | | Mascot |
| 1058.6503 | 1058.5728 | -0.0775 | -73 | 256 | 264 | LRLIAGIMR | | | Oxidation (M)[8] | | Mascot |
| 1328.7831 | 1328.7145 | -0.0686 | -52 | 46 | 56 | LCQGLLTVLRR | | | Carbamidomethyl (C)[2] | | Mascot |
| 1495.7937 | 1495.8158 | 0.0221 | 15 | 265 | 276 | HLLMLKTHTEEK | | | Oxidation (M)[4] | | Mascot |
| 1563.7384 | 1563.8088 | 0.0704 | 45 | 2 | 16 | SLSDILSEIDGGDDK | | | | | Mascot |
| 1563.7384 | 1563.8088 | 0.0704 | 45 | 2 | 16 | SLSDILSEIDGGDDK | | | | | Mascot |
| 1838.8688 | 1838.8802 | 0.0114 | 6 | 1 | 17 | MSLSDILSEIDGGDDKK | | | Oxidation (M)[1] | | Mascot |
| 2283.0193 | 2283.1423 | 0.123 | 54 | 98 | 118 | ICVAGHEGEPLEEAQSAE EER | | | | | Mascot |
| 2283.0193 | 2283.1423 | 0.123 | 54 | 98 | 118 | ICVAGHEGEPLEEAQSAE EER | | | | | Mascot |

| | | | | | | | |
|---|---|---|---|---|---|---|---|
| 8 | PREDICTED: chromodomain-helicase-DNA-binding protein 9-like [Danio rerio] | gi\|326669481 | 278654.9 | 8.67 | 23 | 38 | 0 |

**Peptide Information**

| Calc. Mass | Obsrv. Mass | ± da | ± ppm | Start Seq. | End Seq. | Sequence | Ion Score | C. I. % | Modification | Rank | Result Type |
|---|---|---|---|---|---|---|---|---|---|---|---|
| 842.4665 | 842.495 | 0.0285 | 34 | 1477 | 1483 | AMARLHK | | | Oxidation (M)[2] | | Mascot |
| 870.5043 | 870.5257 | 0.0214 | 25 | 1807 | 1815 | NASVVPGVK | | | | | Mascot |
| 1051.5604 | 1051.6952 | 0.1348 | 128 | 603 | 611 | NCKLLEGFK | | | | | Mascot |
| 1104.5895 | 1104.561 | -0.0285 | -26 | 2345 | 2355 | SSALNLSLDGK | | | | | Mascot |
| 1106.5437 | 1106.5375 | -0.0062 | -6 | 2356 | 2365 | AERTEAQSSK | | | | | Mascot |
| 1179.6481 | 1179.5756 | -0.0725 | -61 | 1046 | 1055 | KQTRPFSSTK | | | | | Mascot |
| 1234.6249 | 1234.6344 | 0.0095 | 8 | 1294 | 1303 | ADPKLCFLDR | | | Carbamidomethyl (C)[6] | | Mascot |
| 1279.6021 | 1279.7494 | 0.1473 | 115 | 477 | 488 | NCILADEMGLGK | | | Oxidation (M)[8] | | Mascot |
| 1320.5897 | 1320.5619 | -0.0278 | -21 | 1492 | 1501 | YLCAFIAMCR | | | Carbamidomethyl (C)[3,9], Oxidation (M)[8] | | Mascot |
| 1347.675 | 1347.6898 | 0.0148 | 11 | 1695 | 1706 | DDILKVGESESR | | | | | Mascot |
| 1460.6959 | 1460.7313 | 0.0354 | 24 | 1492 | 1502 | YLCAFIAMCRR | | | Carbamidomethyl (C)[3,9] | | Mascot |
| 1493.7668 | 1493.7123 | -0.0545 | -36 | 807 | 818 | CLDILEDYLIQR | | | | | Mascot |
| 1699.861 | 1699.9062 | 0.0452 | 27 | 2137 | 2151 | NPNKIDINSLTGEER | | | | | Mascot |
| 1773.9718 | 1773.7863 | -0.1855 | -105 | 1161 | 1176 | ELQNHLGLSTPVPRGR | | | | | Mascot |
| 2211.0322 | 2211.0635 | 0.0313 | 14 | 1589 | 1606 | TDYHILRDPELCYMAAQR | | | Oxidation (M)[14] | | Mascot |
| 2230.0715 | 2230.1536 | 0.0821 | 37 | 459 | 475 | DYQLEGVNWLLFNWYNR | | | | | Mascot |
| 2282.9988 | 2283.1423 | 0.1435 | 63 | 356 | 374 | AHFFADEDPFNPDYVEV DR | | | | | Mascot |
| 2282.9988 | 2283.1423 | 0.1435 | 63 | 356 | 374 | AHFFADEDPFNPDYVEV DR | | | | | Mascot |
| 2297.3015 | 2297.1516 | -0.1499 | -65 | 502 | 522 | TGIKGPFLIIAPLSTIANWE R | | | | | Mascot |
| 2298.146 | 2298.1445 | -0.0015 | -1 | 1901 | 1922 | LLDNASTLPEDPSDSPQ GSKVK | | | | | Mascot |

| Calc. Mass | Obsrv. Mass | ± da | ± ppm | Start Seq. | End Seq. | Sequence | Ion Score | C. I. % | Modification | Rank | Result Type |
|---|---|---|---|---|---|---|---|---|---|---|---|
| 2371.22 | 2371.0925 | -0.1275 | -54 | 424 | 443 | LQAAKPHTNRMERPPASHWK | | | Oxidation (M)[11] | | Mascot |
| 2371.22 | 2371.0925 | -0.1275 | -54 | 424 | 443 | LQAAKPHTNRMERPPASHWK | | | Oxidation (M)[11] | | Mascot |
| 2384.2014 | 2383.9158 | -0.2856 | -120 | 1506 | 1527 | LPAIEGDMVDSSLAIQPITEER | | | | | Mascot |
| 2705.2483 | 2705.1128 | -0.1355 | -50 | 1262 | 1283 | SEMPAMWWDMLSDKCLLLGVYK | | | Carbamidomethyl (C)[15], Oxidation (M)[3,6] | | Mascot |
| 3338.5801 | 3338.7061 | 0.126 | 38 | 1548 | 1575 | HPQLYEHLALCQPGPDLPVWWETGSHDR | | | Carbamidomethyl (C)[11] | | Mascot |

| | | | | | | | | |
|---|---|---|---|---|---|---|---|---|
| 9 | NADH dehydrogenase (ubiquinone) 1 alpha subcomplex, 12 [Danio rerio] | gi\|74315939 | 16958.4 | 9.39 | 6 | 36 | 0 | |

Peptide Information

| Calc. Mass | Obsrv. Mass | ± da | ± ppm | Start Seq. | End Seq. | Sequence | Ion Score | C. I. % | Modification | Rank | Result Type |
|---|---|---|---|---|---|---|---|---|---|---|---|
| 882.4501 | 882.5508 | 0.1007 | 114 | 2 | 9 | AGYATLMR | | | | | Mascot |
| 1045.4805 | 1045.5449 | 0.0644 | 62 | 1 | 9 | MAGYATLMR | | | Oxidation (M)[1,8] | | Mascot |
| 1234.6215 | 1234.6344 | 0.0129 | 10 | 11 | 22 | FVGEFTGHGGVK | | | | | Mascot |
| 1460.7955 | 1460.7313 | -0.0642 | -44 | 31 | 44 | VQETKTGALIGTDK | | | | | Mascot |
| 2117.9419 | 2117.9473 | 0.0054 | 3 | 90 | 107 | WLHCMTDDPPTTHPPEPK | | | Oxidation (M)[5] | | Mascot |
| 2230.042 | 2230.1536 | 0.1116 | 50 | 90 | 108 | WLHCMTDDPPTTHPPEPKK | | | | | Mascot |

| | | | | | | | | |
|---|---|---|---|---|---|---|---|---|
| 10 | PREDICTED: microtubule-associated protein 6-like [Danio rerio] | gi\|292621423 | 42669 | 9.6 | 9 | 36 | 0 | |

Peptide Information

| Calc. Mass | Obsrv. Mass | ± da | ± ppm | Start Seq. | End Seq. | Sequence | Ion Score | C. I. % | Modification | Rank | Result Type |
|---|---|---|---|---|---|---|---|---|---|---|---|
| 864.421 | 864.4794 | 0.0584 | 68 | 113 | 119 | NQYQPSK | | | | | Mascot |
| 973.5173 | 973.4948 | -0.0225 | -23 | 220 | 228 | RSAADALNR | | | | | Mascot |
| 973.5173 | 973.4948 | -0.0225 | -23 | 220 | 228 | RSAADALNR | | | | | Mascot |
| 1165.7052 | 1165.724 | 0.0188 | 16 | 317 | 326 | HNLSLTKPKK | | | | | Mascot |
| 1165.7052 | 1165.724 | 0.0188 | 16 | 317 | 326 | HNLSLTKPKK | | | | | Mascot |
| 1179.6368 | 1179.5756 | -0.0612 | -52 | 300 | 309 | IRSLYSEPSK | | | | | Mascot |
| 1234.5984 | 1234.6344 | 0.036 | 29 | 201 | 211 | EPVSEASKMEK | | | | | Mascot |
| 1316.644 | 1316.6753 | 0.0313 | 24 | 282 | 293 | GEQAKPQTTDNK | | | | | Mascot |
| 1563.7341 | 1563.8088 | 0.0747 | 48 | 2 | 14 | AWPCITRACCINR | | | Carbamidomethyl (C)[4] | | Mascot |
| 1563.7341 | 1563.8088 | 0.0747 | 48 | 2 | 14 | AWPCITRACCINR | | | Carbamidomethyl (C)[4] | | Mascot |
| 1584.7297 | 1584.8456 | 0.1159 | 73 | 9 | 21 | ACCINRFWSELDK | | | | | Mascot |
| 1584.7297 | 1584.8456 | 0.1159 | 73 | 9 | 21 | ACCINRFWSELDK | | | | | Mascot |
| 1657.8391 | 1657.7596 | -0.0795 | -48 | 352 | 367 | TSVSNQEPKPEGGVTK | | | | | Mascot |

| 11 | ATPase, Ca++ transporting, plasma membrane 3a [Danio rerio] | gi\|49903091 | 79409.9 | 5.25 | 13 | 36 | 0 |
|---|---|---|---|---|---|---|---|

Peptide Information

| Calc. Mass | Obsrv. Mass | ± da | ± ppm | Start Seq. | End Seq. | Sequence | Ion Score | C. I. % | Modification | Rank | Result Type |
|---|---|---|---|---|---|---|---|---|---|---|---|
| 864.4131 | 864.4794 | 0.0663 | 77 | 626 | 632 | DKDEMVK | | | | | Mascot |
| 882.4501 | 882.5508 | 0.1007 | 114 | 609 | 615 | CSFILSR | | | Carbamidomethyl (C)[1] | | Mascot |
| 973.604 | 973.4948 | -0.1092 | -112 | 358 | 366 | SVLQGKLTK | | | | | Mascot |
| 973.604 | 973.4948 | -0.1092 | -112 | 358 | 366 | SVLQGKLTK | | | | | Mascot |
| 1165.6074 | 1165.724 | 0.1166 | 100 | 84 | 93 | VFGMNFIPPK | | | Oxidation (M)[4] | | Mascot |
| 1165.6074 | 1165.724 | 0.1166 | 100 | 84 | 93 | VFGMNFIPPK | | | Oxidation (M)[4] | | Mascot |
| 1277.7074 | 1277.6868 | -0.0206 | -16 | 83 | 93 | KVFGMNFIPPK | | | | | Mascot |
| 1347.6759 | 1347.6898 | 0.0139 | 10 | 633 | 644 | KVIEPMACDGLR | | | Oxidation (M)[6] | | Mascot |
| 1633.7924 | 1633.802 | 0.0096 | 6 | 322 | 335 | KQDEAVAMEMQPLK | | | Oxidation (M)[8] | | Mascot |
| 1699.8142 | 1699.9062 | 0.092 | 54 | 581 | 595 | KSMSTVIQMPDGSFR | | | Oxidation (M)[3] | | Mascot |
| 1993.8412 | 1993.9388 | 0.0976 | 49 | 457 | 474 | HLDACETMGNATAICSDK | | | Carbamidomethyl (C)[5,15] | | Mascot |
| 2118.0205 | 2117.9473 | -0.0732 | -35 | 254 | 273 | SIDKDPMLLSGTHVMEG SGK | | | Oxidation (M)[7] | | Mascot |
| 2211.2053 | 2211.0635 | -0.1418 | -64 | 200 | 220 | NSTVIQIPVAEMVVGDIA QVK | | | | | Mascot |
| 2596.3359 | 2596.2666 | -0.0693 | -27 | 508 | 531 | TLELISSAIAVNCAYTSKI MPADK | | | Carbamidomethyl (C)[13] | | Mascot |
| 2705.2075 | 2705.1128 | -0.0947 | -35 | 451 | 474 | DNNLVRHLDACETMGNA TAICSDK | | | Carbamidomethyl (C)[11,21] | | Mascot |

| 12 | Arginyl-tRNA synthetase [Danio rerio] | gi\|53734470 | 75716.9 | 6.22 | 13 | 36 | 0 |
|---|---|---|---|---|---|---|---|

Peptide Information

| Calc. Mass | Obsrv. Mass | ± da | ± ppm | Start Seq. | End Seq. | Sequence | Ion Score | C. I. % | Modification | Rank | Result Type |
|---|---|---|---|---|---|---|---|---|---|---|---|
| 973.4948 | 973.4948 | 0 | 0 | 354 | 362 | GLVEVDEGR | | | | | Mascot |
| 973.4948 | 973.4948 | 0 | 0 | 354 | 362 | GLVEVDEGR | 2 | 0 | | | Mascot |
| 1058.4313 | 1058.5728 | 0.1415 | 134 | 281 | 288 | FDEDEEFK | | | | | Mascot |
| 1316.7242 | 1316.6753 | -0.0489 | -37 | 463 | 473 | LMDLLEEGLKR | | | | | Mascot |
| 1328.7321 | 1328.7145 | -0.0176 | -13 | 649 | 660 | GFDILGINPVQR | | | | | Mascot |
| 1385.7463 | 1385.7235 | -0.0228 | -16 | 225 | 235 | LFEFLGYEVLR | | | | | Mascot |
| 1460.7532 | 1460.7313 | -0.0219 | -15 | 529 | 541 | GNTAAYLLYAFTR | | | | | Mascot |
| 1475.7675 | 1475.7279 | -0.0396 | -27 | 649 | 661 | GFDILGINPVQRM | | | Oxidation (M)[13] | | Mascot |
| 1495.8267 | 1495.8158 | -0.0109 | -7 | 155 | 168 | TEIAGPGFINVHLK | | | | | Mascot |
| 1584.838 | 1584.8456 | 0.0076 | 5 | 437 | 451 | VEHAGFGVVLGEDKK | | | | | Mascot |
| 1584.838 | 1584.8456 | 0.0076 | 5 | 437 | 451 | VEHAGFGVVLGEDKK | | | | | Mascot |

| | | | | | | | | | | | |
|---|---|---|---|---|---|---|---|---|---|---|---|
| 1638.7904 | 1638.8326 | 0.0422 | 26 | 1 | 14 | MGDPVTEYASRLQR | | | Oxidation (M)[1] | | Mascot |
| 1699.8723 | 1699.9062 | 0.0339 | 20 | 319 | 332 | EFQKVYNCLDISIK | | | | | Mascot |
| 2246.9006 | 2247.063 | 0.1624 | 72 | 111 | 129 | FGDYQCNSAMAMSQMMKAK | | | Carbamidomethyl (C)[6], Oxidation (M)[10,12,15] | | Mascot |
| 2284.0149 | 2284.1323 | 0.1174 | 51 | 335 | 353 | GESYYQDMMTAVVKEFEEK | | | | | Mascot |
| 2300.0098 | 2300.1367 | 0.1269 | 55 | 335 | 353 | GESYYQDMMTAVVKEFEEK | | | Oxidation (M)[8] | | Mascot |

| | | | | | | | |
|---|---|---|---|---|---|---|---|
| 13 | 60S ribosomal protein L28 [Danio rerio] | gi\|41055030 | 15629.8 | 12.11 | 6 | 35 | 0 |

Peptide Information

| Calc. Mass | Obsrv. Mass | ± da | ± ppm | Start Seq. | End Seq. | Sequence | Ion Score | C. I. % | Modification | Rank | Result Type |
|---|---|---|---|---|---|---|---|---|---|---|---|
| 856.4787 | 856.5079 | 0.0292 | 34 | 41 | 47 | FNGLIHR | | | | | Mascot |
| 1051.5895 | 1051.6952 | 0.1057 | 101 | 13 | 21 | NSSSFLIKR | | | | | Mascot |
| 1213.7375 | 1213.5818 | -0.1557 | -128 | 89 | 99 | ATLASVRNIIR | | | | | Mascot |
| 1279.6753 | 1279.7494 | 0.0741 | 58 | 69 | 80 | SGQRKPATSYGK | | | | | Mascot |
| 1639.0013 | 1638.8326 | -0.1687 | -103 | 114 | 128 | RVSAILSSQKPVVVR | | | | | Mascot |
| 2230.1802 | 2230.1536 | -0.0266 | -12 | 2 | 20 | ASPHLQWMVIRNSSSFLIK | | | Oxidation (M)[8] | | Mascot |

| | | | | | | | |
|---|---|---|---|---|---|---|---|
| 14 | novel protein similar to human and mouse myeloid/lymphoid or mixed-lineage leukemia 3 (MLL3) [Danio | gi\|157885936 | 82601.8 | 9.38 | 14 | 35 | 0 |

Peptide Information

| Calc. Mass | Obsrv. Mass | ± da | ± ppm | Start Seq. | End Seq. | Sequence | Ion Score | C. I. % | Modification | Rank | Result Type |
|---|---|---|---|---|---|---|---|---|---|---|---|
| 864.5189 | 864.4794 | -0.0395 | -46 | 636 | 642 | ELLYKAK | | | | | Mascot |
| 870.4679 | 870.5257 | 0.0578 | 66 | 140 | 147 | SISEGHLK | | | | | Mascot |
| 971.4879 | 971.525 | 0.0371 | 38 | 343 | 349 | CFSVFRR | | | Carbamidomethyl (C)[1] | | Mascot |
| 996.5948 | 996.58 | -0.0148 | -15 | 462 | 470 | VLGPVAERR | | | | | Mascot |
| 996.5948 | 996.58 | -0.0148 | -15 | 462 | 470 | VLGPVAERR | | | | | Mascot |
| 1045.6152 | 1045.5449 | -0.0703 | -67 | 602 | 611 | IQGLGLFAAR | | | | | Mascot |
| 1106.5323 | 1106.5375 | 0.0052 | 5 | 35 | 43 | ITKDEEESR | | | | | Mascot |
| 1325.6089 | 1325.719 | 0.1101 | 83 | 275 | 285 | GQTVVCAFCQR | | | Carbamidomethyl (C)[6,9] | | Mascot |
| 1325.6089 | 1325.719 | 0.1101 | 83 | 275 | 285 | GQTVVCAFCQR | | | Carbamidomethyl (C)[6,9] | | Mascot |
| 1460.6863 | 1460.7313 | 0.045 | 31 | 188 | 200 | VAPQSSESELEER | | | | | Mascot |
| 1495.7257 | 1495.8158 | 0.0901 | 60 | 273 | 285 | QRGQTVVCAFCQR | | | | | Mascot |
| 1584.776 | 1584.8456 | 0.0696 | 44 | 616 | 628 | QTMVIEYMGDILR | | | Oxidation (M)[3] | | Mascot |
| 1584.776 | 1584.8456 | 0.0696 | 44 | 616 | 628 | QTMVIEYMGDILR | | | Oxidation (M)[3] | | Mascot |
| 1707.8483 | 1707.7456 | -0.1027 | -60 | 499 | 513 | IAESLPGVEACSRYR | | | Carbamidomethyl (C)[11] | | Mascot |

| Calc. Mass | Obsrv. Mass | ± da | ± ppm | Start Seq. | End Seq. | Sequence | Ion Score | C. I. % | Modification | Rank | Result Type |
|---|---|---|---|---|---|---|---|---|---|---|---|
| 1880.9487 | 1881.0011 | 0.0524 | 28 | 440 | 456 | VIEQGYEDLVLTDTSAK | | | | | Mascot |
| 2225.0112 | 2225.0808 | 0.0696 | 31 | 668 | 686 | YINHSCSPNCVAEVVTFER | | | Carbamidomethyl (C)[6] | | Mascot |
| 2225.0112 | 2225.0808 | 0.0696 | 31 | 668 | 686 | YINHSCSPNCVAEVVTFER | | | Carbamidomethyl (C)[6] | | Mascot |
| 2283.1562 | 2283.1423 | -0.0139 | -6 | 328 | 348 | GAGAAAGLHVEHQLRCFSVFR | | | Carbamidomethyl (C)[16] | | Mascot |
| 2283.1562 | 2283.1423 | -0.0139 | -6 | 328 | 348 | GAGAAAGLHVEHQLRCFSVFR | | | Carbamidomethyl (C)[16] | | Mascot |

15 transcription elongation factor B polypeptide 1 [Danio rerio] gi|73088652 12495 4.74 5 35 0

**Peptide Information**

| Calc. Mass | Obsrv. Mass | ± da | ± ppm | Start Seq. | End Seq. | Sequence | Ion Score | C. I. % | Modification | Rank | Result Type |
|---|---|---|---|---|---|---|---|---|---|---|---|
| 1325.6381 | 1325.719 | 0.0809 | 61 | 73 | 82 | VCMYFTYKVR | | | Oxidation (M)[3] | | Mascot |
| 1325.6381 | 1325.719 | 0.0809 | 61 | 73 | 82 | VCMYFTYKVR | | | Oxidation (M)[3] | | Mascot |
| 1563.6454 | 1563.8088 | 0.1634 | 104 | 7 | 20 | TYGGCEGPDAMYVK | | | Carbamidomethyl (C)[5], Oxidation (M)[11] | | Mascot |
| 1563.6454 | 1563.8088 | 0.1634 | 104 | 7 | 20 | TYGGCEGPDAMYVK | | | Carbamidomethyl (C)[5], Oxidation (M)[11] | | Mascot |
| 2118.0398 | 2117.9473 | -0.0925 | -44 | 64 | 80 | EIPSHVLSKVCMYFTYK | | | Carbamidomethyl (C)[11], Oxidation (M)[12] | | Mascot |
| 2211.0134 | 2211.0635 | 0.0501 | 23 | 44 | 63 | AMLSGPGQFAENETNEVNFR | | | | | Mascot |
| 2282.925 | 2283.1423 | 0.2173 | 95 | 1 | 20 | MDSEEKTYGGCEGPDAMYVK | | | Carbamidomethyl (C)[11], Oxidation (M)[1] | | Mascot |
| 2282.925 | 2283.1423 | 0.2173 | 95 | 1 | 20 | MDSEEKTYGGCEGPDAMYVK | | | Carbamidomethyl (C)[11], Oxidation (M)[1] | | Mascot |

16 plasma membrane calcium ATPase 3a [Danio rerio] gi|313482836 129460.6 5.41 16 34 0

**Peptide Information**

| Calc. Mass | Obsrv. Mass | ± da | ± ppm | Start Seq. | End Seq. | Sequence | Ion Score | C. I. % | Modification | Rank | Result Type |
|---|---|---|---|---|---|---|---|---|---|---|---|
| 864.4131 | 864.4794 | 0.0663 | 77 | 626 | 632 | DKDEMVK | | | | | Mascot |
| 882.4501 | 882.5508 | 0.1007 | 114 | 609 | 615 | CSFILSR | | | Carbamidomethyl (C)[1] | | Mascot |
| 973.604 | 973.4948 | -0.1092 | -112 | 358 | 366 | SVLQGKLTK | | | | | Mascot |
| 973.604 | 973.4948 | -0.1092 | -112 | 358 | 366 | SVLQGKLTK | | | | | Mascot |
| 1165.6074 | 1165.724 | 0.1166 | 100 | 84 | 93 | VFGMNFIPPK | | | Oxidation (M)[4] | | Mascot |
| 1165.6074 | 1165.724 | 0.1166 | 100 | 84 | 93 | VFGMNFIPPK | | | Oxidation (M)[4] | | Mascot |
| 1277.7074 | 1277.6868 | -0.0206 | -16 | 83 | 93 | KVFGMNFIPPK | | | | | Mascot |
| 1320.6763 | 1320.5619 | -0.1144 | -87 | 917 | 927 | NNPLISRTMMK | | | Oxidation (M)[9] | | Mascot |
| 1347.6759 | 1347.6898 | 0.0139 | 10 | 633 | 644 | KVIEPMACDGLR | | | Oxidation (M)[6] | | Mascot |
| 1633.7924 | 1633.802 | 0.0096 | 6 | 322 | 335 | KQDEAVAMEMQPLK | | | Oxidation (M)[8] | | Mascot |
| 1699.8142 | 1699.9062 | 0.092 | 54 | 581 | 595 | KSMSTVIQMPDGSFR | | | Oxidation (M)[3] | | Mascot |
| 1864.8026 | 1864.9264 | 0.1238 | 66 | 719 | 734 | CGIIHPGDDFLCMEGK | | | Carbamidomethyl (C)[1,12], Oxidation (M)[13] | | Mascot |

| | | | | | | | | | | | |
|---|---|---|---|---|---|---|---|---|---|---|---|
| 1993.8412 | 1993.9388 | 0.0976 | 49 | 457 | 474 | HLDACETMGNATAICSDK | | | Carbamidomethyl (C)[5,15] | | Mascot |
| 2118.0205 | 2117.9473 | -0.0732 | -35 | 254 | 273 | SIDKDPMLLSGTHVMEG SGK | | | Oxidation (M)[7] | | Mascot |
| 2211.2053 | 2211.0635 | -0.1418 | -64 | 200 | 220 | NSTVIQIPVAEMVVGDIA QVK | | | | | Mascot |
| 2282.999 | 2283.1423 | 0.1433 | 63 | 719 | 738 | CGIIHPGDDFLCMEGKDF NR | | | Oxidation (M)[13] | | Mascot |
| 2282.999 | 2283.1423 | 0.1433 | 63 | 719 | 738 | CGIIHPGDDFLCMEGKDF NR | | | Oxidation (M)[13] | | Mascot |
| 2596.3359 | 2596.2666 | -0.0693 | -27 | 508 | 531 | TLELISSAIAVNCAYTSKI MPADK | | | Carbamidomethyl (C)[13] | | Mascot |
| 2705.2075 | 2705.1128 | -0.0947 | -35 | 451 | 474 | DNNLVRHLDACETMGNA TAICSDK | | | Carbamidomethyl (C)[11,21] | | Mascot |

17 Synembryn-like [Danio rerio] gi|94574123 59482.8 5.37 10 34 0

**Peptide Information**

| Calc. Mass | Obsrv. Mass | ± da | ± ppm | Start Seq. | End Seq. | Sequence | Ion Score | C. I. % | Modification | Rank | Result Type |
|---|---|---|---|---|---|---|---|---|---|---|---|
| 842.5709 | 842.495 | -0.0759 | -90 | 18 | 24 | VEKLLLK | | | | | Mascot |
| 1045.6769 | 1045.5449 | -0.132 | -126 | 171 | 179 | LLFLLSALR | | | | | Mascot |
| 1058.6503 | 1058.5728 | -0.0775 | -73 | 256 | 264 | LRLIAGIMR | | | Oxidation (M)[8] | | Mascot |
| 1325.6921 | 1325.719 | 0.0269 | 20 | 399 | 410 | QGAAEFLFVLCK | | | | | Mascot |
| 1325.6921 | 1325.719 | 0.0269 | 20 | 399 | 410 | QGAAEFLFVLCK | | | | | Mascot |
| 1328.7831 | 1328.7145 | -0.0686 | -52 | 46 | 56 | LCQGLLTVLRR | | | Carbamidomethyl (C)[2] | | Mascot |
| 1460.6396 | 1460.7313 | 0.0917 | 63 | 238 | 250 | ALFNLTMSDSCDK | | | Oxidation (M)[7] | | Mascot |
| 1563.7384 | 1563.8088 | 0.0704 | 45 | 2 | 16 | SLSDILSEIDGGDDK | | | | | Mascot |
| 1563.7384 | 1563.8088 | 0.0704 | 45 | 2 | 16 | SLSDILSEIDGGDDK | | | | | Mascot |
| 1838.8688 | 1838.8802 | 0.0114 | 6 | 1 | 17 | MSLSDILSEIDGGDDKK | | | Oxidation (M)[1] | | Mascot |
| 1865.0023 | 1864.9264 | -0.0759 | -41 | 313 | 328 | GKNMDTVQILLNFMIK | | | | | Mascot |
| 1880.9972 | 1881.0011 | 0.0039 | 2 | 313 | 328 | GKNMDTVQILLNFMIK | | | Oxidation (M)[4] | | Mascot |
| 2283.0193 | 2283.1423 | 0.123 | 54 | 98 | 118 | ICVAGHEGEPLEEAQSAE EER | | | | | Mascot |
| 2283.0193 | 2283.1423 | 0.123 | 54 | 98 | 118 | ICVAGHEGEPLEEAQSAE EER | | | | | Mascot |

18 pyruvate dehydrogenase kinase, isozyme 3b [Danio rerio] gi|123702577 21003.7 8.49 7 34 0

**Peptide Information**

| Calc. Mass | Obsrv. Mass | ± da | ± ppm | Start Seq. | End Seq. | Sequence | Ion Score | C. I. % | Modification | Rank | Result Type |
|---|---|---|---|---|---|---|---|---|---|---|---|
| 882.4468 | 882.5508 | 0.104 | 118 | 30 | 36 | QFLDFGR | | | | | Mascot |
| 1045.5499 | 1045.5449 | -0.005 | -5 | 43 | 50 | TSYMFLRK | | | | | Mascot |
| 1165.6836 | 1165.724 | 0.0404 | 35 | 1 | 9 | MKLFICLLK | | | Carbamidomethyl (C)[6] | | Mascot |

| Calc. Mass | Obsrv. Mass | ± da | ± ppm | Start Seq. | End Seq. | Sequence | Ion Score | C. I. % | Modification | Rank | Result Type |
|---|---|---|---|---|---|---|---|---|---|---|---|
| 1165.6836 | 1165.724 | 0.0404 | 35 | 1 | 9 | MKLFICLLK | | | Carbamidomethyl (C)[6] | | Mascot |
| 1325.6631 | 1325.719 | 0.0559 | 42 | 162 | 172 | MLINQHSETPR | | | | | Mascot |
| 1325.6631 | 1325.719 | 0.0559 | 42 | 162 | 172 | MLINQHSETPR | | | | | Mascot |
| 1838.8636 | 1838.8802 | 0.0166 | 9 | 162 | 176 | MLINQHSETPRNHVM | | | Oxidation (M)[1,15] | | Mascot |
| 1844.9436 | 1844.845 | -0.0986 | -53 | 158 | 172 | ISFRMLINQHSETPR | | | Oxidation (M)[5] | | Mascot |
| 1844.9436 | 1844.845 | -0.0986 | -53 | 158 | 172 | ISFRMLINQHSETPR | | | Oxidation (M)[5] | | Mascot |
| 2284.1641 | 2284.1323 | -0.0318 | -14 | 97 | 115 | SPEDPHTLNDFLEMLIKIR | | | Oxidation (M)[14] | | Mascot |

| | | | | | | | |
|---|---|---|---|---|---|---|---|
| 19 | hypothetical protein LOC393702 [Danio rerio] | gi\|41152266 | 20567.3 | 5.44 | 6 | 34 | 0 |

Peptide Information

| Calc. Mass | Obsrv. Mass | ± da | ± ppm | Start Seq. | End Seq. | Sequence | Ion Score | C. I. % | Modification | Rank | Result Type |
|---|---|---|---|---|---|---|---|---|---|---|---|
| 864.4131 | 864.4794 | 0.0663 | 77 | 128 | 135 | VISCEEGK | | | | | Mascot |
| 971.5883 | 971.525 | -0.0633 | -65 | 162 | 169 | RIILEAEK | | | | | Mascot |
| 1213.6973 | 1213.5818 | -0.1155 | -95 | 110 | 120 | VQVPIMLVGNK | | | Oxidation (M)[6] | | Mascot |
| 1320.6907 | 1320.5619 | -0.1288 | -98 | 152 | 162 | ENQTAVEVFKR | | | | | Mascot |
| 1325.7974 | 1325.719 | -0.0784 | -59 | 110 | 121 | VQVPIMLVGNKK | | | | | Mascot |
| 1325.7974 | 1325.719 | -0.0784 | -59 | 110 | 121 | VQVPIMLVGNKK | | | | | Mascot |
| 3337.6475 | 3337.6936 | 0.0461 | 14 | 16 | 45 | SVGKSSLTIQFVEGQFVD SYDPTIENTFTK | | | | | Mascot |

| | | | | | | | |
|---|---|---|---|---|---|---|---|
| 20 | protein tyrosine phosphatase, receptor type, N polypeptide 2 [Danio rerio] | gi\|126540834 | 32113.7 | 10.05 | 7 | 34 | 0 |

Peptide Information

| Calc. Mass | Obsrv. Mass | ± da | ± ppm | Start Seq. | End Seq. | Sequence | Ion Score | C. I. % | Modification | Rank | Result Type |
|---|---|---|---|---|---|---|---|---|---|---|---|
| 842.473 | 842.495 | 0.022 | 26 | 119 | 125 | ELSQLPR | | | | | Mascot |
| 1051.6371 | 1051.6952 | 0.0581 | 55 | 95 | 103 | SVLQKLAHR | | | | | Mascot |
| 1316.6125 | 1316.6753 | 0.0628 | 48 | 28 | 38 | RFGCLFEDVCK | | | | | Mascot |
| 1633.8909 | 1633.802 | -0.0889 | -54 | 51 | 64 | DFIKITTLTSTTHR | | | | | Mascot |
| 1844.9324 | 1844.845 | -0.0874 | -47 | 232 | 248 | SGVPLAELQQYQAGPMR | | | | | Mascot |
| 1844.9324 | 1844.845 | -0.0874 | -47 | 232 | 248 | SGVPLAELQQYQAGPMR | | | | | Mascot |
| 2225.135 | 2225.0808 | -0.0542 | -24 | 159 | 178 | FLQGFLPSDVSSFKQSP QGR | | | | | Mascot |
| 2225.135 | 2225.0808 | -0.0542 | -24 | 159 | 178 | FLQGFLPSDVSSFKQSP QGR | | | | | Mascot |
| 2297.1707 | 2297.1516 | -0.0191 | -8 | 55 | 74 | ITTLTSTTHREYAVFGQC LR | | | | | Mascot |

| | | | | | | | |
|---|---|---|---|---|---|---|---|
| 21 | PREDICTED: hypothetical protein LOC569436 [Danio rerio] | gi\|326678363 | 352771.2 | 7.92 | 27 | 34 | 0 |

## Peptide Information

| Calc. Mass | Obsrv. Mass | ± da | ± ppm | Start Seq. | End Seq. | Sequence | Ion Score | C. I. % | Modification | Rank | Result Type |
|---|---|---|---|---|---|---|---|---|---|---|---|
| 842.4743 | 842.495 | 0.0207 | 25 | 2664 | 2669 | HFRINR | | | | | Mascot |
| 856.4311 | 856.5079 | 0.0768 | 90 | 1282 | 1287 | ENYFRK | | | | | Mascot |
| 864.4131 | 864.4794 | 0.0663 | 77 | 603 | 610 | LGAEMSEK | | | | | Mascot |
| 971.4904 | 971.525 | 0.0346 | 36 | 617 | 624 | QESPNLQR | | | | | Mascot |
| 973.5676 | 973.4948 | -0.0728 | -75 | 1952 | 1959 | LKELDLSR | | | | | Mascot |
| 973.5676 | 973.4948 | -0.0728 | -75 | 1952 | 1959 | LKELDLSR | | | | | Mascot |
| 1058.588 | 1058.5728 | -0.0152 | -14 | 312 | 320 | SFSYKVSLK | | | | | Mascot |
| 1179.6256 | 1179.5756 | -0.05 | -42 | 2297 | 2306 | LSELTLDFNK | | | | | Mascot |
| 1320.6616 | 1320.5619 | -0.0997 | -75 | 3017 | 3027 | LHLYNCSITEK | | | | | Mascot |
| 1347.7454 | 1347.6898 | -0.0556 | -41 | 2092 | 2102 | HCKISTLTFLK | | | Carbamidomethyl (C)[2] | | Mascot |
| 1475.647 | 1475.7279 | 0.0809 | 55 | 1333 | 1345 | RNNENNQADDASK | | | | | Mascot |
| 1495.7859 | 1495.8158 | 0.0299 | 20 | 1690 | 1702 | STMCKLEILGLSK | | | Carbamidomethyl (C)[4], Oxidation (M)[3] | | Mascot |
| 1584.7257 | 1584.8456 | 0.1199 | 76 | 1 | 13 | MHSAHQEEQKCIK | | | Oxidation (M)[1] | | Mascot |
| 1584.7257 | 1584.8456 | 0.1199 | 76 | 1 | 13 | MHSAHQEEQKCIK | | | Oxidation (M)[1] | | Mascot |
| 1699.9086 | 1699.9062 | -0.0024 | -1 | 2183 | 2197 | ELNLNNSRLLDSGVR | | | | | Mascot |
| 1707.8007 | 1707.7456 | -0.0551 | -32 | 1474 | 1487 | KNVFMDESPELENR | | | | | Mascot |
| 1839.0586 | 1838.8802 | -0.1784 | -97 | 2648 | 2663 | LDLEKVNQLAVLLQDK | | | | | Mascot |
| 1881.0116 | 1881.0011 | -0.0105 | -6 | 2408 | 2423 | SIIPYLFEEQGKTSLR | | | | | Mascot |
| 1993.9246 | 1993.9388 | 0.0142 | 7 | 1066 | 1082 | LLNDVYTDLYMTQCASK | | | Oxidation (M)[11] | | Mascot |
| 2118.0713 | 2117.9473 | -0.124 | -59 | 1024 | 1041 | EDEVANALQDELVLIYQR | | | | | Mascot |
| 2193.8933 | 2194.0452 | 0.1519 | 69 | 238 | 255 | HMCIHSGEKPYACDQCG K | | | Carbamidomethyl (C)[3,13,16], Oxidation (M)[2] | | Mascot |
| 2224.9797 | 2225.0808 | 0.1011 | 45 | 321 | 339 | HHMSLHNEEKPYVCGQC GR | | | | | Mascot |
| 2224.9797 | 2225.0808 | 0.1011 | 45 | 321 | 339 | HHMSLHNEEKPYVCGQC GR | | | | | Mascot |
| 2232.8818 | 2233.0427 | 0.1609 | 72 | 181 | 199 | YHMKMHSDETAFSCEEC GK | | | | | Mascot |
| 2265.9949 | 2266.0667 | 0.0718 | 32 | 131 | 149 | HCGNAQHICDLCGKSFS FK | | | Carbamidomethyl (C)[2,9,12] | | Mascot |
| 2283.2068 | 2283.1423 | -0.0645 | -28 | 1358 | 1375 | TLTQMYTHFLRFQILQSR | | | | | Mascot |
| 2283.2068 | 2283.1423 | -0.0645 | -28 | 1358 | 1375 | TLTQMYTHFLRFQILQSR | | | | | Mascot |
| 2297.144 | 2297.1516 | 0.0076 | 3 | 2728 | 2748 | LNLSDCSISEEGYKALAS ALR | | | Carbamidomethyl (C)[6] | | Mascot |
| 2297.9961 | 2298.1445 | 0.1484 | 65 | 321 | 339 | HHMSLHNEEKPYVCGQC GR | | | Carbamidomethyl (C)[14], Oxidation (M)[3] | | Mascot |
| 2371.1301 | 2371.0925 | -0.0376 | -16 | 2158 | 2178 | MNNCELTEKSCSVLATVL SSK | | | Carbamidomethyl (C)[4,11] | | Mascot |
| 2371.1301 | 2371.0925 | -0.0376 | -16 | 2158 | 2178 | MNNCELTEKSCSVLATVL SSK | | | Carbamidomethyl (C)[4,11] | | Mascot |
| 2705.1799 | 2705.1128 | -0.0671 | -25 | 233 | 255 | ASVNRHMCIHSGEKPYA | | | Carbamidomethyl (C)[8,18,21] | | Mascot |

| Calc. Mass | Obsrv. Mass | ± da | ± ppm | Start Seq. | End Seq. | Sequence | Ion Score | C. I. % | Modification | Rank | Result Type |
|---|---|---|---|---|---|---|---|---|---|---|---|
| 3227.6946 | 3227.5364 | -0.1582 | -49 | 1212 | 1240 | CDQCGK LPLNFDGNETWRDVSSP ASLDVLLTNLIK | | | | | Mascot |

| | | | | | | | |
|---|---|---|---|---|---|---|---|
| 22 | PREDICTED: zinc finger protein 84-like [Danio rerio] | gi\|326666743 | 48665.5 | 9.41 | 13 | 34 | 0 |

Peptide Information

| Calc. Mass | Obsrv. Mass | ± da | ± ppm | Start Seq. | End Seq. | Sequence | Ion Score | C. I. % | Modification | Rank | Result Type |
|---|---|---|---|---|---|---|---|---|---|---|---|
| 856.4457 | 856.5079 | 0.0622 | 73 | 236 | 242 | NLAVHMR | | | Oxidation (M)[6] | | Mascot |
| 971.4727 | 971.525 | 0.0523 | 54 | 179 | 186 | SNLDVHMR | | | | | Mascot |
| 1058.4393 | 1058.5728 | 0.1335 | 126 | 390 | 397 | FRCSECEK | | | Carbamidomethyl (C)[3] | | Mascot |
| 1234.5885 | 1234.6344 | 0.0459 | 37 | 243 | 253 | THTGEKPFSCK | | | | | Mascot |
| 1279.6688 | 1279.7494 | 0.0806 | 63 | 97 | 106 | LDVHMRVHTR | | | Oxidation (M)[5] | | Mascot |
| 1347.5602 | 1347.6898 | 0.1296 | 96 | 221 | 232 | SCTCQQCGKSFR | | | | | Mascot |
| 1460.8267 | 1460.7313 | -0.0954 | -65 | 263 | 274 | LNLIAHMRVHTR | | | | | Mascot |
| 1475.6609 | 1475.7279 | 0.067 | 45 | 35 | 46 | EQTNQQNEIDEK | | | | | Mascot |
| 1638.8057 | 1638.8326 | 0.0269 | 16 | 286 | 298 | SFGQKQDLYIHMR | | | Oxidation (M)[12] | | Mascot |
| 1707.736 | 1707.7456 | 0.0096 | 6 | 215 | 229 | IHTGERSCTCQQCGK | | | Carbamidomethyl (C)[8] | | Mascot |
| 2300.0435 | 2300.1367 | 0.0932 | 41 | 327 | 346 | THTGEKPFACDQCGKSF TTK | | | Carbamidomethyl (C)[10,13] | | Mascot |
| 2371.0806 | 2371.0925 | 0.0119 | 5 | 187 | 206 | VHTGEKPYTCEQCGKSF SQK | | | Carbamidomethyl (C)[10,13] | | Mascot |
| 2371.0806 | 2371.0925 | 0.0119 | 5 | 187 | 206 | VHTGEKPYTCEQCGKSF SQK | | | Carbamidomethyl (C)[10,13] | | Mascot |
| 2705.2229 | 2705.1128 | -0.1101 | -41 | 179 | 201 | SNLDVHMRVHTGEKPYT CEQCGK | | | Carbamidomethyl (C)[18], Oxidation (M)[7] | | Mascot |

| | | | | | | | |
|---|---|---|---|---|---|---|---|
| 23 | PREDICTED: tRNA-dihydrouridine synthase 2-like [Danio rerio] | gi\|292628574 | 56676.6 | 6.04 | 11 | 33 | 0 |

Peptide Information

| Calc. Mass | Obsrv. Mass | ± da | ± ppm | Start Seq. | End Seq. | Sequence | Ion Score | C. I. % | Modification | Rank | Result Type |
|---|---|---|---|---|---|---|---|---|---|---|---|
| 842.473 | 842.495 | 0.022 | 26 | 436 | 443 | TLGLPEGR | | | | | Mascot |
| 971.4801 | 971.525 | 0.0449 | 46 | 77 | 84 | VMFRTCSK | | | | | Mascot |
| 1045.4628 | 1045.5449 | 0.0821 | 79 | 288 | 295 | YCLCQMLR | | | Oxidation (M)[6] | | Mascot |
| 1051.6007 | 1051.6952 | 0.0945 | 90 | 180 | 190 | TGVAAIAVHGR | | | | | Mascot |
| 1058.5518 | 1058.5728 | 0.021 | 20 | 366 | 374 | DYPPQITPK | | | | | Mascot |
| 1277.6041 | 1277.6868 | 0.0827 | 65 | 129 | 141 | GGMGSALLSDPEK | | | Oxidation (M)[3] | | Mascot |
| 1316.5908 | 1316.6753 | 0.0845 | 64 | 288 | 297 | YCLCQMLRDR | | | Oxidation (M)[6] | | Mascot |
| 1699.9346 | 1699.9062 | -0.0284 | -17 | 19 | 33 | NILAPMVRVGTLPMR | | | Oxidation (M)[6,14] | | Mascot |
| 1773.851 | 1773.7863 | -0.0647 | -36 | 107 | 123 | LVENDVAAVDVNMGCPK | | | | | Mascot |
| 2384.1484 | 2383.9158 | -0.2326 | -98 | 237 | 258 | ESTGASSVMLARAAMWN | | | Oxidation (M)[9] | | Mascot |

| Calc. Mass | Obsrv. Mass | ± da | ± ppm | Start Seq. | End Seq. | Sequence | Ion Score | C. I. % | Modification | Rank | Result Type |
|---|---|---|---|---|---|---|---|---|---|---|---|
| | | | | | | PSVFR | | | | | |
| 2580.2222 | 2580.2661 | 0.0439 | 17 | 306 | 327 | LHAAQTNQEICEVFGMTE LFNK | | | Carbamidomethyl (C)[11] | | Mascot |
| 2596.217 | 2596.2666 | 0.0496 | 19 | 306 | 327 | LHAAQTNQEICEVFGMTE LFNK | | | Carbamidomethyl (C)[11], Oxidation (M)[16] | | Mascot |

24 novel protein similar to MCM6 minichromosome maintenance deficient 6, mitotin (S. cerevisiae) (mcm6 gi|169158286 93177.2 5.26 12 33 0

Peptide Information

| Calc. Mass | Obsrv. Mass | ± da | ± ppm | Start Seq. | End Seq. | Sequence | Ion Score | C. I. % | Modification | Rank | Result Type |
|---|---|---|---|---|---|---|---|---|---|---|---|
| 856.49 | 856.5079 | 0.0179 | 21 | 87 | 93 | AVRHFAR | | | | | Mascot |
| 973.5789 | 973.4948 | -0.0841 | -86 | 117 | 124 | IRELSTVR | | | | | Mascot |
| 973.5789 | 973.4948 | -0.0841 | -86 | 117 | 124 | IRELSTVR | | | | | Mascot |
| 1165.6252 | 1165.724 | 0.0988 | 85 | 747 | 755 | SELINWYLK | | | | | Mascot |
| 1165.6252 | 1165.724 | 0.0988 | 85 | 747 | 755 | SELINWYLK | | | | | Mascot |
| 1234.7228 | 1234.6344 | -0.0884 | -72 | 361 | 372 | GILLMLFGGVAK | | | Oxidation (M)[5] | | Mascot |
| 1277.6195 | 1277.6868 | 0.0673 | 53 | 321 | 330 | NQMTVLEWEK | | | | | Mascot |
| 1325.7899 | 1325.719 | -0.0709 | -53 | 212 | 223 | GSIPRSVEVILR | | | | | Mascot |
| 1325.7899 | 1325.719 | -0.0709 | -53 | 212 | 223 | GSIPRSVEVILR | | | | | Mascot |
| 1347.6508 | 1347.6898 | 0.039 | 29 | 512 | 523 | QNVNMSAPIMSR | | | | | Mascot |
| 1475.7502 | 1475.7279 | -0.0223 | -15 | 90 | 102 | HFARDHGNIPPSK | | | | | Mascot |
| 1707.8306 | 1707.7456 | -0.085 | -50 | 168 | 182 | YTQPTICKNPVCANR | | | | | Mascot |
| 2211.1875 | 2211.0635 | -0.124 | -56 | 361 | 381 | GILLMLFGGVAKTTMEGT SLR | | | Oxidation (M)[5] | | Mascot |
| 2297.1812 | 2297.1516 | -0.0296 | -13 | 575 | 593 | QFQPKITVEAQEFVVDQY K | | | | | Mascot |
| 2580.2949 | 2580.2661 | -0.0288 | -11 | 138 | 160 | THPVHPELVSGTFLCLDC QSVIK | | | Carbamidomethyl (C)[15] | | Mascot |

25 PREDICTED: transposon TX1 uncharacterized 149 kDa protein-like [Danio rerio] gi|326674296 180436.7 7.83 20 33 0

Peptide Information

| Calc. Mass | Obsrv. Mass | ± da | ± ppm | Start Seq. | End Seq. | Sequence | Ion Score | C. I. % | Modification | Rank | Result Type |
|---|---|---|---|---|---|---|---|---|---|---|---|
| 850.4087 | 850.5255 | 0.1168 | 137 | 595 | 601 | DITQAMR | | | Oxidation (M)[6] | | Mascot |
| 996.493 | 996.58 | 0.087 | 87 | 564 | 571 | KQCFSDLR | | | | | Mascot |
| 996.493 | 996.58 | 0.087 | 87 | 564 | 571 | KQCFSDLR | | | | | Mascot |
| 1045.5717 | 1045.5449 | -0.0268 | -26 | 662 | 669 | FFFSLEKK | | | | | Mascot |
| 1058.6609 | 1058.5728 | -0.0881 | -83 | 1405 | 1413 | VLYKPPLTK | | | | | Mascot |
| 1104.5585 | 1104.561 | 0.0025 | 2 | 476 | 483 | QYTWLHTR | | | | | Mascot |
| 1179.6455 | 1179.5756 | -0.0699 | -59 | 1218 | 1226 | VWRLFSLCR | | | | | Mascot |

| | | | | | | | | | | | | |
|---|---|---|---|---|---|---|---|---|---|---|---|---|
| 1320.5571 | 1320.5619 | 0.0048 | 4 | 87 | 97 | CFGCGQEGHLR | | | Carbamidomethyl (C)[1,4] | | Mascot |
| 1325.6484 | 1325.719 | 0.0706 | 53 | 257 | 267 | NVQVVDFFSDR | | | | | Mascot |
| 1325.6484 | 1325.719 | 0.0706 | 53 | 257 | 267 | NVQVVDFFSDR | | | | | Mascot |
| 1460.7783 | 1460.7313 | -0.047 | -32 | 899 | 911 | TLESFGFGSKFIK | | | | | Mascot |
| 1475.71 | 1475.7279 | 0.0179 | 12 | 583 | 594 | LFCQQYAFNVSR | | | | | Mascot |
| 1495.6715 | 1495.8158 | 0.1443 | 96 | 551 | 561 | EVFCFFWDQFK | | | | | Mascot |
| 1638.7904 | 1638.8326 | 0.0422 | 26 | 648 | 661 | SRYQNLTQMDAPSK | | | | | Mascot |
| 1657.9132 | 1657.7596 | -0.1536 | -93 | 926 | 941 | VNGGLSAPFNISRGIR | | | | | Mascot |
| 1707.775 | 1707.7456 | -0.0294 | -17 | 1357 | 1370 | DVNLTMMQGKTMYK | | | Oxidation (M)[6,7,12] | | Mascot |
| 1838.8265 | 1838.8802 | 0.0537 | 29 | 71 | 86 | VDGYDYVVFASSETMR | | | | | Mascot |
| 1994.0642 | 1993.9388 | -0.1254 | -63 | 276 | 291 | IVFVFCYTLFLMGVIR | | | Carbamidomethyl (C)[6], Oxidation (M)[12] | | Mascot |
| 2230.1299 | 2230.1536 | 0.0237 | 11 | 1202 | 1220 | NICLNGLPSFYCGLFKVWR | | | | | Mascot |
| 2297.25 | 2297.1516 | -0.0984 | -43 | 1422 | 1443 | ILHGIVAVNAFISVVNSSVDDK | | | | | Mascot |
| 2298.0818 | 2298.1445 | 0.0627 | 27 | 2 | 23 | AAAMETPASAFSGDAPFERLSR | | | Oxidation (M)[4] | | Mascot |
| 3227.645 | 3227.5364 | -0.1086 | -34 | 1422 | 1450 | ILHGIVAVNAFISVVNSSVDDKCIFCGHR | | | Carbamidomethyl (C)[23,26] | | Mascot |

26 hypothetical protein LOC492329 [Danio rerio] gi|55925514 119622.9 8.99 15 33 0

**Peptide Information**

| Calc. Mass | Obsrv. Mass | ± da | ± ppm | Start Seq. | End Seq. | Sequence | Ion Score | C. I. % | Modification | Rank | Result Type |
|---|---|---|---|---|---|---|---|---|---|---|---|
| 842.4366 | 842.495 | 0.0584 | 69 | 90 | 96 | NTTLEHK | | | | | Mascot |
| 1058.4645 | 1058.5728 | 0.1083 | 102 | 694 | 701 | KEACEMFK | | | Carbamidomethyl (C)[4], Oxidation (M)[6] | | Mascot |
| 1325.6519 | 1325.719 | 0.0671 | 51 | 394 | 404 | CHPKEPTSLEK | | | Carbamidomethyl (C)[1] | | Mascot |
| 1325.6519 | 1325.719 | 0.0671 | 51 | 394 | 404 | CHPKEPTSLEK | | | Carbamidomethyl (C)[1] | | Mascot |
| 1434.7522 | 1434.745 | -0.0072 | -5 | 666 | 677 | KVSIANMLSWNR | | | Oxidation (M)[7] | | Mascot |
| 1493.6941 | 1493.7123 | 0.0182 | 12 | 345 | 358 | NPSDYSPAGLECIK | | | | | Mascot |
| 1563.8376 | 1563.8088 | -0.0288 | -18 | 794 | 806 | ITQFILDQQDSKK | | | | | Mascot |
| 1563.8376 | 1563.8088 | -0.0288 | -18 | 793 | 805 | KITQFILDQQDSK | | | | | Mascot |
| 1633.8405 | 1633.802 | -0.0385 | -24 | 198 | 212 | GTSLHLPQSKASDHR | | | | | Mascot |
| 1657.7527 | 1657.7596 | 0.0069 | 4 | 639 | 653 | QPPPPDTSMTDWASK | | | | | Mascot |
| 1844.896 | 1844.845 | -0.051 | -28 | 542 | 558 | TNNNPSMAFTPLDIPGR | | | | | Mascot |
| 1844.896 | 1844.845 | -0.051 | -28 | 542 | 558 | TNNNPSMAFTPLDIPGR | | | | | Mascot |
| 1864.9875 | 1864.9264 | -0.0611 | -33 | 146 | 161 | QKPDIHVSDINKDSLR | | | | | Mascot |
| 1880.8485 | 1881.0011 | 0.1526 | 81 | 1016 | 1031 | MDVNNLAMVMAPNCLR | | | Carbamidomethyl (C)[14], Oxidation (M)[1,8] | | Mascot |
| 1993.9713 | 1993.9388 | -0.0325 | -16 | 816 | 833 | KQNSDEEAEALPISTYAK | | | | | Mascot |
| 2225.1133 | 2225.0808 | -0.0325 | -15 | 753 | 773 | SLAAGWELMAVCLAFFAPSPK | | | Oxidation (M)[9] | | Mascot |

| | | | | | | | | | | | |
|---|---|---|---|---|---|---|---|---|---|---|---|
| 2225.1133 | 2225.0808 | -0.0325 | -15 | 753 | 773 | SLAAGWELMAVCLAFFA PSPK | | | Oxidation (M)[9] | | Mascot |
| 2247.0354 | 2247.063 | 0.0276 | 12 | 695 | 713 | EACEMFKLVQAYMGDRP SR | | | Oxidation (M)[5] | | Mascot |
| 2266.1399 | 2266.0667 | -0.0732 | -32 | 753 | 773 | SLAAGWELMAVCLAFFA PSPK | | | Carbamidomethyl (C)[12] | | Mascot |

27 novel protein [Danio rerio] gi|94733078 59497.8 5.33 10 33 0

**Peptide Information**

| Calc. Mass | Obsrv. Mass | ± da | ± ppm | Start Seq. | End Seq. | Sequence | Ion Score | C. I. % | Modification | Rank | Result Type |
|---|---|---|---|---|---|---|---|---|---|---|---|
| 842.5709 | 842.495 | -0.0759 | -90 | 18 | 24 | VEKLLLK | | | | | Mascot |
| 1045.6769 | 1045.5449 | -0.132 | -126 | 171 | 179 | LLFLLSALR | | | | | Mascot |
| 1058.6503 | 1058.5728 | -0.0775 | -73 | 256 | 264 | LRLIAGIMR | | | Oxidation (M)[8] | | Mascot |
| 1325.6921 | 1325.719 | 0.0269 | 20 | 399 | 410 | QGAAEFLFVLCK | | | | | Mascot |
| 1325.6921 | 1325.719 | 0.0269 | 20 | 399 | 410 | QGAAEFLFVLCK | | | | | Mascot |
| 1328.7831 | 1328.7145 | -0.0686 | -52 | 46 | 56 | LCQGLLTVLRR | | | Carbamidomethyl (C)[2] | | Mascot |
| 1460.6396 | 1460.7313 | 0.0917 | 63 | 238 | 250 | ALFNLTMSDSCDK | | | Oxidation (M)[7] | | Mascot |
| 1495.7937 | 1495.8158 | 0.0221 | 15 | 265 | 276 | HLLMLKTHTEEK | | | Oxidation (M)[4] | | Mascot |
| 1563.7384 | 1563.8088 | 0.0704 | 45 | 2 | 16 | SLSDILSEIDGGDDK | | | | | Mascot |
| 1563.7384 | 1563.8088 | 0.0704 | 45 | 2 | 16 | SLSDILSEIDGGDDK | | | | | Mascot |
| 1838.8688 | 1838.8802 | 0.0114 | 6 | 1 | 17 | MSLSDILSEIDGGDDKK | | | Oxidation (M)[1] | | Mascot |
| 2283.0193 | 2283.1423 | 0.123 | 54 | 98 | 118 | ICVAGHEGEPLEEAQSAE EER | | | | | Mascot |
| 2283.0193 | 2283.1423 | 0.123 | 54 | 98 | 118 | ICVAGHEGEPLEEAQSAE EER | | | | | Mascot |

28 RecName: Full=SH3 domain-binding protein 4-A gi|123888628 108383 7.89 14 33 0

**Peptide Information**

| Calc. Mass | Obsrv. Mass | ± da | ± ppm | Start Seq. | End Seq. | Sequence | Ion Score | C. I. % | Modification | Rank | Result Type |
|---|---|---|---|---|---|---|---|---|---|---|---|
| 842.5822 | 842.495 | -0.0872 | -103 | 663 | 669 | LIKTVIR | | | | | Mascot |
| 850.4338 | 850.5255 | 0.0917 | 108 | 387 | 393 | LSNMEIK | | | Oxidation (M)[4] | | Mascot |
| 870.4726 | 870.5257 | 0.0531 | 61 | 1 | 7 | MAAHRIR | | | Oxidation (M)[1] | | Mascot |
| 973.5829 | 973.4948 | -0.0881 | -90 | 695 | 702 | LKGQLWTK | | | | | Mascot |
| 973.5829 | 973.4948 | -0.0881 | -90 | 695 | 702 | LKGQLWTK | | | | | Mascot |
| 1045.4796 | 1045.5449 | 0.0653 | 62 | 791 | 799 | AELDSEPER | | | | | Mascot |
| 1434.7158 | 1434.745 | 0.0292 | 20 | 914 | 925 | DVIHELHMGLDR | | | | | Mascot |
| 1657.8651 | 1657.7596 | -0.1055 | -64 | 394 | 407 | SFITLEMKVSVMTR | | | Oxidation (M)[7] | | Mascot |
| 2118.1958 | 2117.9473 | -0.2485 | -117 | 636 | 653 | IILSPLAITTKYPQFQER | | | | | Mascot |
| 2230.2417 | 2230.1536 | -0.0881 | -40 | 842 | 860 | LIMDFVLLTTAVEVAPRW | | | | | Mascot |

| | | | | | | | | | | | |
|---|---|---|---|---|---|---|---|---|---|---|---|
| | | | | | | R | | | | | |
| 2284.2368 | 2284.1323 | -0.1045 | -46 | 492 | 513 | TVVAIFGHDCAPKTLLVS EVGK | | | | | Mascot |
| 2300.0686 | 2300.1367 | 0.0681 | 30 | 597 | 616 | DDQDCILAQFCVQTPQP PPK | | | Carbamidomethyl (C)[5] | | Mascot |
| 3227.677 | 3227.5364 | -0.1406 | -44 | 936 | 965 | HLTGTLILVNCLDLLRSSA FSPAPQDDFAI | | | | | Mascot |
| 3337.6052 | 3337.6936 | 0.0884 | 26 | 298 | 327 | LARSCHDLESLGQNPGW GQTQPVETNIVCK | | | Carbamidomethyl (C)[5] | | Mascot |
| 3338.5496 | 3338.7061 | 0.1565 | 47 | 885 | 913 | TGMVDSEAMWKPAYDFL LTWAAQIGDSYR | | | Oxidation (M)[3] | | Mascot |

29 LOC100005466 protein [Danio rerio] gi|141795779 69366.9 9.49 9 33 0 12 0

**Peptide Information**

| Calc. Mass | Obsrv. Mass | ± da | ± ppm | Start Seq. | End Seq. | Sequence | Ion Score | C. I. % | Modification | Rank | Result Type |
|---|---|---|---|---|---|---|---|---|---|---|---|
| 973.5214 | 973.4948 | -0.0266 | -27 | 31 | 38 | DKGITFHR | | | | | Mascot |
| 973.5214 | 973.4948 | -0.0266 | -27 | 31 | 38 | DKGITFHR | 12 | 0 | | | Mascot |
| 996.5836 | 996.58 | -0.0036 | -4 | 395 | 403 | TLASIHNLK | | | | | Mascot |
| 996.5836 | 996.58 | -0.0036 | -4 | 395 | 403 | TLASIHNLK | | | | | Mascot |
| 1385.63 | 1385.7235 | 0.0935 | 67 | 383 | 394 | NRNTFICTECGK | | | | | Mascot |
| 1493.7971 | 1493.7123 | -0.0848 | -57 | 517 | 528 | HQRIHTGEKPYK | | | | | Mascot |
| 1563.6025 | 1563.8088 | 0.2063 | 132 | 12 | 25 | MPDFCAAYGCSNER | | | | | Mascot |
| 1563.6025 | 1563.8088 | 0.2063 | 132 | 12 | 25 | MPDFCAAYGCSNER | | | | | Mascot |
| 2224.9788 | 2225.0808 | 0.102 | 46 | 601 | 619 | HTSAHEEKNCTHSLQFN NA | | | Carbamidomethyl (C)[10] | | Mascot |
| 2224.9788 | 2225.0808 | 0.102 | 46 | 601 | 619 | HTSAHEEKNCTHSLQFN NA | | | Carbamidomethyl (C)[10] | | Mascot |
| 2266.0479 | 2266.0667 | 0.0188 | 8 | 468 | 487 | EKPYSCSVCGKSFSQSS SLK | | | Carbamidomethyl (C)[6,9] | | Mascot |
| 2283.0105 | 2283.1423 | 0.1318 | 58 | 426 | 444 | QPSDHMQHMMTHTGEK PYK | | | | | Mascot |
| 2283.0105 | 2283.1423 | 0.1318 | 58 | 426 | 444 | QPSDHMQHMMTHTGEK PYK | | | | | Mascot |
| 2705.2017 | 2705.1128 | -0.0889 | -33 | 423 | 444 | SFRQPSDHMQHMMTHT GEKPYK | | | Oxidation (M)[9,12] | | Mascot |

30 PREDICTED: serine/threonine-protein kinase pim-2 [Danio rerio] gi|326676649 47149.7 5.18 8 32 0

**Peptide Information**

| Calc. Mass | Obsrv. Mass | ± da | ± ppm | Start Seq. | End Seq. | Sequence | Ion Score | C. I. % | Modification | Rank | Result Type |
|---|---|---|---|---|---|---|---|---|---|---|---|
| 971.5421 | 971.525 | -0.0171 | -18 | 281 | 288 | GVFHRDIK | | | | | Mascot |
| 1277.6406 | 1277.6868 | 0.0462 | 36 | 118 | 128 | TDSPEKLLSCK | | | Carbamidomethyl (C)[10] | | Mascot |
| 1584.7651 | 1584.8456 | 0.0805 | 51 | 164 | 178 | EIGEGGFGSVYEGKR | | | | | Mascot |
| 1584.7651 | 1584.8456 | 0.0805 | 51 | 164 | 178 | EIGEGGFGSVYEGKR | | | | | Mascot |
| 1657.7164 | 1657.7596 | 0.0432 | 26 | 266 | 279 | IMEQATTAGLMCCR | | | Carbamidomethyl (C)[12,13], Oxidation (M)[2] | | Mascot |

| Calc. Mass | Obsrv. Mass | ± da | ± ppm | Start Seq. | End Seq. | Sequence | Ion Score | C. I. % | Modification | Rank | Result Type |
|---|---|---|---|---|---|---|---|---|---|---|---|
| 1699.7747 | 1699.9062 | 0.1315 | 77 | 266 | 280 | IMEQATTAGLMCCRR | | | Oxidation (M)[2] | | Mascot |
| 1844.8621 | 1844.845 | -0.0171 | -9 | 132 | 148 | SSSSSSQEDQTPVQIHK | | | | | Mascot |
| 1844.8621 | 1844.845 | -0.0171 | -9 | 132 | 148 | SSSSSSQEDQTPVQIHK | | | | | Mascot |
| 2300.3853 | 2300.1367 | -0.2486 | -108 | 198 | 218 | YINIPLPLEIGLLILAHRGG K | | | | | Mascot |
| 3337.5293 | 3337.6936 | 0.1643 | 49 | 52 | 78 | WESFEKPLSLYAEDDNQ WMTFLCGQKR | | | Oxidation (M)[19] | | Mascot |

31 novel protein similar to H.sapiens ATPase, Ca++ transporting, plasma membrane (zgc:92885) [Danio re gi|169145692 138055 5.51 16 32 0

**Peptide Information**

| Calc. Mass | Obsrv. Mass | ± da | ± ppm | Start Seq. | End Seq. | Sequence | Ion Score | C. I. % | Modification | Rank | Result Type |
|---|---|---|---|---|---|---|---|---|---|---|---|
| 864.4131 | 864.4794 | 0.0663 | 77 | 626 | 632 | DKDEMVK | | | | | Mascot |
| 882.4501 | 882.5508 | 0.1007 | 114 | 609 | 615 | CSFILSR | | | Carbamidomethyl (C)[1] | | Mascot |
| 973.604 | 973.4948 | -0.1092 | -112 | 358 | 366 | SVLQGKLTK | | | | | Mascot |
| 973.604 | 973.4948 | -0.1092 | -112 | 358 | 366 | SVLQGKLTK | | | | | Mascot |
| 1165.6074 | 1165.724 | 0.1166 | 100 | 84 | 93 | VFGMNFIPPK | | | Oxidation (M)[4] | | Mascot |
| 1165.6074 | 1165.724 | 0.1166 | 100 | 84 | 93 | VFGMNFIPPK | | | Oxidation (M)[4] | | Mascot |
| 1277.7074 | 1277.6868 | -0.0206 | -16 | 83 | 93 | KVFGMNFIPPK | | | | | Mascot |
| 1320.6763 | 1320.5619 | -0.1144 | -87 | 917 | 927 | NNPLISRTMMK | | | Oxidation (M)[9] | | Mascot |
| 1347.6759 | 1347.6898 | 0.0139 | 10 | 633 | 644 | KVIEPMACDGLR | | | Oxidation (M)[6] | | Mascot |
| 1633.7924 | 1633.802 | 0.0096 | 6 | 322 | 335 | KQDEAVAMEMQPLK | | | Oxidation (M)[8] | | Mascot |
| 1699.8142 | 1699.9062 | 0.092 | 54 | 581 | 595 | KSMSTVIQMPDGSFR | | | Oxidation (M)[3] | | Mascot |
| 1864.8026 | 1864.9264 | 0.1238 | 66 | 719 | 734 | CGIIHPGDDFLCMEGK | | | Carbamidomethyl (C)[1,12], Oxidation (M)[13] | | Mascot |
| 1993.8412 | 1993.9388 | 0.0976 | 49 | 457 | 474 | HLDACETMGNATAICSDK | | | Carbamidomethyl (C)[5,15] | | Mascot |
| 2118.0205 | 2117.9473 | -0.0732 | -35 | 254 | 273 | SIDKDPMLLSGTHVMEG SGK | | | Oxidation (M)[7] | | Mascot |
| 2211.2053 | 2211.0635 | -0.1418 | -64 | 200 | 220 | NSTVIQIPVAEMVVGDIA QVK | | | | | Mascot |
| 2282.999 | 2283.1423 | 0.1433 | 63 | 719 | 738 | CGIIHPGDDFLCMEGKDF NR | | | Oxidation (M)[13] | | Mascot |
| 2282.999 | 2283.1423 | 0.1433 | 63 | 719 | 738 | CGIIHPGDDFLCMEGKDF NR | | | Oxidation (M)[13] | | Mascot |
| 2596.3359 | 2596.2666 | -0.0693 | -27 | 508 | 531 | TLELISSAIAVNCAYTSKI MPADK | | | Carbamidomethyl (C)[13] | | Mascot |
| 2705.2075 | 2705.1128 | -0.0947 | -35 | 451 | 474 | DNNLVRHLDACETMGNA TAICSDK | | | Carbamidomethyl (C)[11,21] | | Mascot |

32 TCR-alpha V segment II-35 [Danio rerio] gi|21322357 13424.7 5.37 5 32 0

**Peptide Information**

| Calc. Mass | Obsrv. Mass | ± da | ± ppm | Start Seq. | End Seq. | Sequence | Ion Score | C. I. % | Modification | Rank | Result Type |
|---|---|---|---|---|---|---|---|---|---|---|---|

| Calc. Mass | Obsrv. Mass | ± da | ± ppm | Start Seq. | End Seq. | Sequence | Ion Score | C. I. % | Modification | Rank | Result Type |
|---|---|---|---|---|---|---|---|---|---|---|---|
| 1385.7134 | 1385.7235 | 0.0101 | 7 | 65 | 77 | SPTFMLSAFTVGK | | | | | Mascot |
| 1994.0527 | 1993.9388 | -0.1139 | -57 | 60 | 77 | QLPNRSPTFMLSAFTVGK | | | | | Mascot |
| 2297.2395 | 2297.1516 | -0.0879 | -38 | 2 | 20 | MEKLLIVALFITAQEFVCR | | | Carbamidomethyl (C)[18], Oxidation (M)[1] | | Mascot |
| 2371.2585 | 2371.0925 | -0.166 | -70 | 1 | 20 | MMEKLLIVALFITAQEFVCR | | | Oxidation (M)[1] | | Mascot |
| 2371.2585 | 2371.0925 | -0.166 | -70 | 1 | 20 | MMEKLLIVALFITAQEFVCR | | | Oxidation (M)[1] | | Mascot |
| 3338.5232 | 3338.7061 | 0.1829 | 55 | 31 | 59 | EMTADKGDTVTLSCNYSVTTTNAYLFWYK | | | Oxidation (M)[2] | | Mascot |

| | | | | | | | |
|---|---|---|---|---|---|---|---|
| 33 | novel protein [Danio rerio] | gi\|123231159 | 11826.2 | 9.18 | 5 | 32 | 0 |

Peptide Information

| Calc. Mass | Obsrv. Mass | ± da | ± ppm | Start Seq. | End Seq. | Sequence | Ion Score | C. I. % | Modification | Rank | Result Type |
|---|---|---|---|---|---|---|---|---|---|---|---|
| 864.472 | 864.4794 | 0.0074 | 9 | 15 | 21 | RTSMLTR | | | | | Mascot |
| 996.5182 | 996.58 | 0.0618 | 62 | 49 | 57 | DCPLVAPPK | | | Carbamidomethyl (C)[2] | | Mascot |
| 996.5182 | 996.58 | 0.0618 | 62 | 49 | 57 | DCPLVAPPK | | | Carbamidomethyl (C)[2] | | Mascot |
| 1045.516 | 1045.5449 | 0.0289 | 28 | 72 | 80 | QTNITDEPK | | | | | Mascot |
| 1633.8076 | 1633.802 | -0.0056 | -3 | 1 | 14 | VVCASYQPLHLMEK | | | Oxidation (M)[12] | | Mascot |
| 1773.9138 | 1773.7863 | -0.1275 | -72 | 1 | 15 | VVCASYQPLHLMEKR | | | | | Mascot |

| | | | | | | | |
|---|---|---|---|---|---|---|---|
| 34 | Ewing tumor-associated antigen 1 [Danio rerio] | gi\|162139038 | 71948.4 | 6.84 | 10 | 32 | 0 |

Peptide Information

| Calc. Mass | Obsrv. Mass | ± da | ± ppm | Start Seq. | End Seq. | Sequence | Ion Score | C. I. % | Modification | Rank | Result Type |
|---|---|---|---|---|---|---|---|---|---|---|---|
| 973.4196 | 973.4948 | 0.0752 | 77 | 159 | 165 | QFDFNMR | | | Oxidation (M)[6] | | Mascot |
| 973.4196 | 973.4948 | 0.0752 | 77 | 159 | 165 | QFDFNMR | | | Oxidation (M)[6] | | Mascot |
| 996.5836 | 996.58 | -0.0036 | -4 | 28 | 37 | SPKAAAPLNK | | | | | Mascot |
| 996.5836 | 996.58 | -0.0036 | -4 | 28 | 37 | SPKAAAPLNK | | | | | Mascot |
| 1460.6951 | 1460.7313 | 0.0362 | 25 | 398 | 409 | LSELNNACHFQK | | | Carbamidomethyl (C)[8] | | Mascot |
| 1475.7085 | 1475.7279 | 0.0194 | 13 | 2 | 14 | ESRGSQEQNEIAK | | | | | Mascot |
| 1584.8452 | 1584.8456 | 0.0004 | 0 | 91 | 105 | AANVRAVDISDIANR | | | | | Mascot |
| 1584.8452 | 1584.8456 | 0.0004 | 0 | 91 | 105 | AANVRAVDISDIANR | | | | | Mascot |
| 1880.9573 | 1881.0011 | 0.0438 | 23 | 575 | 591 | GVLAVIQSEARDTDNHR | | | | | Mascot |
| 1994.0526 | 1993.9388 | -0.1138 | -57 | 334 | 351 | TKVSQNNSQIQINGGLHR | | | | | Mascot |
| 2194.1187 | 2194.0452 | -0.0735 | -33 | 391 | 409 | AQHVVQKLSELNNACHFQK | | | | | Mascot |
| 2211.155 | 2211.0635 | -0.0915 | -41 | 413 | 432 | NAIKPSQSKPSSMHQNLISK | | | Oxidation (M)[13] | | Mascot |
| 2384.0354 | 2383.9158 | -0.1196 | -50 | 524 | 548 | AAGPGCNVSCDAARPSGSSSNGAYK | | | Carbamidomethyl (C)[6] | | Mascot |

| 35 | lactosylceramide alpha-2,3-sialyltransferase [Danio rerio] | gi\|117606180 | 41829.7 | 9.18 | 8 | 32 | 0 | 6 | 0 |
|---|---|---|---|---|---|---|---|---|---|

Peptide Information

| Calc. Mass | Obsrv. Mass | ± da | ± ppm | Start Seq. | End Seq. | Sequence | Ion Score | C. I. % | Modification | Rank | Result Type |
|---|---|---|---|---|---|---|---|---|---|---|---|
| 973.5101 | 973.4948 | -0.0153 | -16 | 226 | 233 | TPVSLWDR | | | | | Mascot |
| 973.5101 | 973.4948 | -0.0153 | -16 | 226 | 233 | TPVSLWDR | 6 | 0 | | | Mascot |
| 1058.6027 | 1058.5728 | -0.0299 | -28 | 140 | 150 | CVVVGNGGILK | | | | | Mascot |
| 1563.7948 | 1563.8088 | 0.014 | 9 | 61 | 73 | EILDKECRPSFAR | | | | | Mascot |
| 1563.7948 | 1563.8088 | 0.014 | 9 | 61 | 73 | EILDKECRPSFAR | | | | | Mascot |
| 1633.8268 | 1633.802 | -0.0248 | -15 | 213 | 225 | SVDFHWLRAMISR | | | Oxidation (M)[10] | | Mascot |
| 1707.8927 | 1707.7456 | -0.1471 | -86 | 234 | 247 | LFFWQNVPMSVPVK | | | Oxidation (M)[9] | | Mascot |
| 1880.8881 | 1881.0011 | 0.113 | 60 | 346 | 364 | LVASGSITDLTGGIHCSF C | | | | | Mascot |
| 1993.9939 | 1993.9388 | -0.0551 | -28 | 314 | 330 | EAPLHYYDSVPMTTILK | | | Oxidation (M)[12] | | Mascot |
| 2297.01 | 2297.1516 | 0.1416 | 62 | 187 | 205 | MSYPESCPKVWEDTDPD LK | | | Carbamidomethyl (C)[7] | | Mascot |

| 36 | Ssfa2 protein [Danio rerio] | gi\|115313851 | 31579.7 | 5.91 | 7 | 31 | 0 |
|---|---|---|---|---|---|---|---|

Peptide Information

| Calc. Mass | Obsrv. Mass | ± da | ± ppm | Start Seq. | End Seq. | Sequence | Ion Score | C. I. % | Modification | Rank | Result Type |
|---|---|---|---|---|---|---|---|---|---|---|---|
| 1475.6907 | 1475.7279 | 0.0372 | 25 | 93 | 106 | GHSMESLSNSSALR | | | | | Mascot |
| 1563.7319 | 1563.8088 | 0.0769 | 49 | 190 | 203 | MESNPTPTSLEVSR | | | Oxidation (M)[1] | | Mascot |
| 1563.7319 | 1563.8088 | 0.0769 | 49 | 190 | 203 | MESNPTPTSLEVSR | | | Oxidation (M)[1] | | Mascot |
| 1707.9136 | 1707.7456 | -0.168 | -98 | 230 | 244 | SRGNGDLQHLIQEIK | | | | | Mascot |
| 2117.9504 | 2117.9473 | -0.0031 | -1 | 76 | 92 | NSSQQLGFYRHHMGMH R | | | Oxidation (M)[13,15] | | Mascot |
| 2247.0557 | 2247.063 | 0.0073 | 3 | 184 | 203 | DVSPERMESNPTPTSLE VSR | | | Oxidation (M)[7] | | Mascot |
| 2284.1501 | 2284.1323 | -0.0178 | -8 | 67 | 85 | LLSINEQMRNSSQQLGFY R | | | | | Mascot |
| 2297.2725 | 2297.1516 | -0.1209 | -53 | 253 | 273 | QEIVNELLAAVSPHRSPL PAR | | | | | Mascot |
| 2300.1453 | 2300.1367 | -0.0086 | -4 | 67 | 85 | LLSINEQMRNSSQQLGFY R | | | Oxidation (M)[8] | | Mascot |

| 37 | PREDICTED: fragaceatoxin C [Danio rerio] | gi\|326665980 | 20858.2 | 6.31 | 6 | 31 | 0 |
|---|---|---|---|---|---|---|---|

Peptide Information

| Calc. Mass | Obsrv. Mass | ± da | ± ppm | Start Seq. | End Seq. | Sequence | Ion Score | C. I. % | Modification | Rank | Result Type |
|---|---|---|---|---|---|---|---|---|---|---|---|
| 971.4502 | 971.525 | 0.0748 | 77 | 56 | 63 | TEVCTFSK | | | Carbamidomethyl (C)[4] | | Mascot |

| | | | | | | | | | | | |
|---|---|---|---|---|---|---|---|---|---|---|---|
| 1277.6527 | 1277.6868 | 0.0341 | 27 | 157 | 168 | ATMCPMGRAIVK | | | | | Mascot |
| 1493.7021 | 1493.7123 | 0.0102 | 7 | 152 | 164 | NLDIRATMCPMGR | | | Oxidation (M)[8] | | Mascot |
| 1699.8009 | 1699.9062 | 0.1053 | 62 | 126 | 138 | EMYYQKNQHGFVR | | | | | Mascot |
| 1864.8643 | 1864.9264 | 0.0621 | 33 | 169 | 184 | VEVWDKLLSPMAQMDC | | | | | Mascot |
| 1880.8591 | 1881.0011 | 0.142 | 75 | 169 | 184 | VEVWDKLLSPMAQMDC | | | Oxidation (M)[11] | | Mascot |
| 1993.9977 | 1993.9388 | -0.0589 | -30 | 64 | 82 | SAAHATGSVGVLTYDLFER | | | | | Mascot |

| | | | | | | | |
|---|---|---|---|---|---|---|---|
| 38 | cytoplasmic dynein 1 heavy chain 1 [Danio rerio] | gi\|112363126 | 532854.1 | 6.06 | 32 | 31 | 0 |

Peptide Information

| Calc. Mass | Obsrv. Mass | ± da | ± ppm | Start Seq. | End Seq. | Sequence | Ion Score | C. I. % | Modification | Rank | Result Type |
|---|---|---|---|---|---|---|---|---|---|---|---|
| 850.4087 | 850.5255 | 0.1168 | 137 | 59 | 65 | SALEQMR | | | Oxidation (M)[6] | | Mascot |
| 870.4389 | 870.5257 | 0.0868 | 100 | 656 | 662 | QLTAYMK | | | Oxidation (M)[6] | | Mascot |
| 971.5632 | 971.525 | -0.0382 | -39 | 283 | 290 | ALNRIQEK | | | | | Mascot |
| 1045.5272 | 1045.5449 | 0.0177 | 17 | 2836 | 2843 | LVEDEERR | | | | | Mascot |
| 1058.6278 | 1058.5728 | -0.055 | -52 | 1396 | 1404 | VNMLVIELK | | | | | Mascot |
| 1104.5354 | 1104.561 | 0.0256 | 23 | 3429 | 3437 | AEEVEQMIR | | | | | Mascot |
| 1106.5952 | 1106.5375 | -0.0577 | -52 | 2511 | 2519 | AELGEYIRR | | | | | Mascot |
| 1165.6212 | 1165.724 | 0.1028 | 88 | 3438 | 3447 | DLEASIARYK | | | | | Mascot |
| 1165.6212 | 1165.724 | 0.1028 | 88 | 3438 | 3447 | DLEASIARYK | | | | | Mascot |
| 1179.5286 | 1179.5756 | 0.047 | 40 | 2442 | 2450 | MEHIMDFTR | | | | | Mascot |
| 1213.6212 | 1213.5818 | -0.0394 | -32 | 3695 | 3704 | ETFVNFTVTR | | | | | Mascot |
| 1277.6598 | 1277.6868 | 0.027 | 21 | 4143 | 4153 | IFVFEPPPGMK | | | Oxidation (M)[10] | | Mascot |
| 1320.6793 | 1320.5619 | -0.1174 | -89 | 223 | 234 | GEKPKVTDFGDK | | | | | Mascot |
| 1325.6736 | 1325.719 | 0.0454 | 34 | 835 | 845 | LAETVFNFQEK | | | | | Mascot |
| 1325.6736 | 1325.719 | 0.0454 | 34 | 835 | 845 | LAETVFNFQEK | | | | | Mascot |
| 1328.6917 | 1328.7145 | 0.0228 | 17 | 1242 | 1252 | IVQEDRAVENR | | | | | Mascot |
| 1434.7886 | 1434.745 | -0.0436 | -30 | 2034 | 2045 | LFRSLAMTKPDR | | | | | Mascot |
| 1475.6835 | 1475.7279 | 0.0444 | 30 | 3682 | 3694 | DPTVEFPPDLCSR | | | | | Mascot |
| 1563.8264 | 1563.8088 | -0.0176 | -11 | 3448 | 3461 | EEYAVLISEAQAIK | | | | | Mascot |
| 1563.8264 | 1563.8088 | -0.0176 | -11 | 3448 | 3461 | EEYAVLISEAQAIK | | | | | Mascot |
| 1584.8704 | 1584.8456 | -0.0248 | -16 | 3847 | 3860 | SVSDHTQRLSLITK | | | | | Mascot |
| 1584.8704 | 1584.8456 | -0.0248 | -16 | 3847 | 3860 | SVSDHTQRLSLITK | | | | | Mascot |
| 1633.8326 | 1633.802 | -0.0306 | -19 | 3925 | 3939 | GLTGEQAEAMVRLSR | | | Oxidation (M)[10] | | Mascot |
| 1707.7867 | 1707.7456 | -0.0411 | -24 | 4362 | 4376 | QRSTSSSDAQPAWMR | | | | | Mascot |
| 1845.0215 | 1844.845 | -0.1765 | -96 | 3759 | 3774 | ILDDDTIITTLENLKK | | | | | Mascot |
| 1845.0215 | 1844.845 | -0.1765 | -96 | 3759 | 3774 | ILDDDTIITTLENLKK | | | | | Mascot |

| | | | | | | | | | | | |
|---|---|---|---|---|---|---|---|---|---|---|---|
| 2194.0828 | 2194.0452 | -0.0376 | -17 | 3174 | 3190 | HYLDFINHYANLFNEKR | | | | | Mascot |
| 2210.9944 | 2211.0635 | 0.0691 | 31 | 531 | 550 | EVDGLDVSKEGMEAWEA AMK | | | Oxidation (M)[12] | | Mascot |
| 2230.1689 | 2230.1536 | -0.0153 | -7 | 2943 | 2961 | TTLSRFVAWMNGLSVYQI K | | | Oxidation (M)[10] | | Mascot |
| 2233.0627 | 2233.0427 | -0.02 | -9 | 3561 | 3580 | WQANSLPADDLCTENAI MLK | | | | | Mascot |
| 2247.1074 | 2247.063 | -0.0444 | -20 | 1020 | 1040 | NALTRMPDGPSALEEAY NAVK | | | | | Mascot |
| 2266.1689 | 2266.0667 | -0.1022 | -45 | 3121 | 3139 | VPDYMPIVYDKLPQPPTH R | | | | | Mascot |
| 2283.1624 | 2283.1423 | -0.0201 | -9 | 2004 | 2024 | QVKVSAEMAIFITMNPGY AGR | | | | | Mascot |
| 2283.1624 | 2283.1423 | -0.0201 | -9 | 2004 | 2024 | QVKVSAEMAIFITMNPGY AGR | | | | | Mascot |
| 2284.2561 | 2284.1323 | -0.1238 | -54 | 4504 | 4523 | NIHVNLGRLFVPEAYITAT R | | | | | Mascot |
| 2371.218 | 2371.0925 | -0.1255 | -53 | 1629 | 1649 | FYFVGDEDLLEIIGNSKNV AK | | | | | Mascot |
| 2371.218 | 2371.0925 | -0.1255 | -53 | 1629 | 1649 | FYFVGDEDLLEIIGNSKNV AK | | | | | Mascot |
| 2384.2642 | 2383.9158 | -0.3484 | -146 | 2046 | 2066 | QLIAQVMLYSQGFRTAET LAK | | | Oxidation (M)[7] | | Mascot |
| 2580.3337 | 2580.2661 | -0.0676 | -26 | 1656 | 1679 | KMFAGVSSILLNEDNTEV LGISSR | | | | | Mascot |
| 2596.3286 | 2596.2666 | -0.062 | -24 | 1656 | 1679 | KMFAGVSSILLNEDNTEV LGISSR | | | Oxidation (M)[2] | | Mascot |

| | | | | | | | |
|---|---|---|---|---|---|---|---|
| 39 | Rho guanine exchange factor betaPix B [Danio rerio] | gi\|109745507 | 90350.1 | 8.42 | 11 | 31 | 0 |

Peptide Information

| Calc. Mass | Obsrv. Mass | ± da | ± ppm | Start Seq. | End Seq. | Sequence | Ion Score | C. I. % | Modification | Rank | Result Type |
|---|---|---|---|---|---|---|---|---|---|---|---|
| 1104.6664 | 1104.561 | -0.1054 | -95 | 388 | 396 | LEKYPTLLK | | | | | Mascot |
| 1279.6715 | 1279.7494 | 0.0779 | 61 | 391 | 400 | YPTLLKEMER | | | | | Mascot |
| 1347.7631 | 1347.6898 | -0.0733 | -54 | 267 | 277 | ELQNLLSVYLR | | | | | Mascot |
| 1633.8102 | 1633.802 | -0.0082 | -5 | 154 | 168 | SLDMSENSAPQVLVK | | | Oxidation (M)[4] | | Mascot |
| 1638.8711 | 1638.8326 | -0.0385 | -23 | 48 | 61 | LRPGSVHTIYQDPR | | | | | Mascot |
| 1657.7891 | 1657.7596 | -0.0295 | -18 | 711 | 724 | KESGPHMLFPEEEK | | | | | Mascot |
| 1844.9535 | 1844.845 | -0.1085 | -59 | 154 | 170 | SLDMSENSAPQVLVKAR | | | | | Mascot |
| 1844.9535 | 1844.845 | -0.1085 | -59 | 154 | 170 | SLDMSENSAPQVLVKAR | | | | | Mascot |
| 2283.1914 | 2283.1423 | -0.0491 | -22 | 317 | 336 | VSDSQQRIGGFFLSLLTQ MR | | | | | Mascot |
| 2283.1914 | 2283.1423 | -0.0491 | -22 | 317 | 336 | VSDSQQRIGGFFLSLLTQ MR | | | | | Mascot |
| 2297.0325 | 2297.1516 | 0.1191 | 52 | 397 | 414 | EMERHMEPLHPDYPDIQ K | | | Oxidation (M)[2,6] | | Mascot |
| 2580.2505 | 2580.2661 | 0.0156 | 6 | 98 | 122 | VLNCLVALNKATSESGG GSNSVCSR | | | Carbamidomethyl (C)[4,23] | | Mascot |
| 2596.25 | 2596.2666 | 0.0166 | 6 | 75 | 97 | GCALYHIEVFEANDLLQG QNFSK | | | | | Mascot |

| | | | | | | | |
|---|---|---|---|---|---|---|---|
| 40 | PREDICTED: estrogen-related receptor gamma, partial [Danio rerio] | gi\|326675851 | 25552.3 | 5.28 | 6 | 30 | 0 |

Peptide Information

| Calc. Mass | Obsrv. Mass | ± da | ± ppm | Start Seq. | End Seq. | Sequence | Ion Score | C. I. % | Modification | Rank | Result Type |
|---|---|---|---|---|---|---|---|---|---|---|---|
| 1106.5994 | 1106.5375 | -0.0619 | -56 | 197 | 205 | AIQHFYSIK | | | | | Mascot |
| 1325.8337 | 1325.719 | -0.1147 | -87 | 180 | 191 | AGKLLMTLPLLR | | | | | Mascot |
| 1325.8337 | 1325.719 | -0.1147 | -87 | 180 | 191 | AGKLLMTLPLLR | | | | | Mascot |
| 1563.9104 | 1563.8088 | -0.1016 | -65 | 1 | 14 | TKIVSHLLVAEPEK | | | | | Mascot |
| 1563.9104 | 1563.8088 | -0.1016 | -65 | 1 | 14 | TKIVSHLLVAEPEK | | | | | Mascot |
| 1707.7969 | 1707.7456 | -0.0513 | -30 | 15 | 29 | IYAMPDPTMPESDIK | | | | | Mascot |
| 2300.2683 | 2300.1367 | -0.1316 | -57 | 30 | 50 | ALTTLCDLADRELVVIIGW AK | | | | | Mascot |
| 2705.156 | 2705.1128 | -0.0432 | -16 | 83 | 104 | SLPYEDELVYAEDYVMD EEHSR | | | Oxidation (M)[16] | | Mascot |

| | | | | | | | |
|---|---|---|---|---|---|---|---|
| 41 | Baz1a protein [Danio rerio] | gi\|120538654 | 37393.3 | 9.36 | 8 | 30 | 0 |

Peptide Information

| Calc. Mass | Obsrv. Mass | ± da | ± ppm | Start Seq. | End Seq. | Sequence | Ion Score | C. I. % | Modification | Rank | Result Type |
|---|---|---|---|---|---|---|---|---|---|---|---|
| 1045.3999 | 1045.5449 | 0.145 | 139 | 303 | 310 | EQMMSMEK | | | Oxidation (M)[3,4] | | Mascot |
| 1106.4789 | 1106.5375 | 0.0586 | 53 | 36 | 43 | TYDEFFER | | | | | Mascot |
| 1234.7782 | 1234.6344 | -0.1438 | -116 | 2 | 11 | PLLHKKPFVR | | | | | Mascot |
| 1316.5796 | 1316.6753 | 0.0957 | 73 | 303 | 312 | EQMMSMEKFR | | | | | Mascot |
| 1657.7417 | 1657.7596 | 0.0179 | 11 | 298 | 310 | LMQQKEQMMSMEK | | | Oxidation (M)[2] | | Mascot |
| 1838.8741 | 1838.8802 | 0.0061 | 3 | 101 | 115 | LHEICDDVYAYVKDR | | | | | Mascot |
| 2266.0518 | 2266.0667 | 0.0149 | 7 | 276 | 295 | GRGRPPNNASSTEMNYI EEK | | | Oxidation (M)[14] | | Mascot |
| 2283.1836 | 2283.1423 | -0.0413 | -18 | 195 | 215 | VQPVSPEACEPVIVKVAQ MSR | | | Oxidation (M)[19] | | Mascot |
| 2283.1836 | 2283.1423 | -0.0413 | -18 | 195 | 215 | VQPVSPEACEPVIVKVAQ MSR | | | Oxidation (M)[19] | | Mascot |

| | | | | | | | |
|---|---|---|---|---|---|---|---|
| 42 | Tnfrsfa protein [Danio rerio] | gi\|190336943 | 48606.4 | 5.31 | 9 | 30 | 0 |

Peptide Information

| Calc. Mass | Obsrv. Mass | ± da | ± ppm | Start Seq. | End Seq. | Sequence | Ion Score | C. I. % | Modification | Rank | Result Type |
|---|---|---|---|---|---|---|---|---|---|---|---|
| 856.4159 | 856.5079 | 0.092 | 107 | 329 | 335 | EDDPPRK | | | | | Mascot |
| 971.4172 | 971.525 | 0.1078 | 111 | 1 | 8 | MDKSEAMK | | | Oxidation (M)[1,7] | | Mascot |
| 1460.7491 | 1460.7313 | -0.0178 | -12 | 418 | 430 | YSAEHIASKAVER | | | | | Mascot |
| 1475.6973 | 1475.7279 | 0.0306 | 21 | 214 | 226 | QQSETNGNLEEVK | | | | | Mascot |

| | | | | | | | | | | | |
|---|---|---|---|---|---|---|---|---|---|---|---|
| 2193.8782 | 2194.0452 | 0.167 | 76 | 121 | 139 | CDCKFGTFCLPDEPCEV CK | | | Carbamidomethyl (C)[1] | | Mascot |
| 2211.0537 | 2211.0635 | 0.0098 | 4 | 310 | 328 | SSPPAQRPTDRDQTESW PR | | | | | Mascot |
| 2266.2515 | 2266.0667 | -0.1848 | -82 | 398 | 417 | EGLRANINTLLQALLDLD QR | | | | | Mascot |
| 2283.9038 | 2284.1323 | 0.2285 | 100 | 41 | 58 | QMTCLENHEYPHNGFCC K | | | Carbamidomethyl (C)[4,16], Oxidation (M)[2] | | Mascot |
| 2596.0947 | 2596.2666 | 0.1719 | 66 | 38 | 58 | TARQMTCLENHEYPHNG FCCK | | | Carbamidomethyl (C)[7,19] | | Mascot |

| | | | | | | | |
|---|---|---|---|---|---|---|---|
| 43 | PREDICTED: zinc finger protein 64 homolog, isoforms 1 and 2 [Danio rerio] | gi\|292627186 | 71408.1 | 8.74 | 11 | 30 | 0 |

**Peptide Information**

| Calc. Mass | Obsrv. Mass | ± da | ± ppm | Start Seq. | End Seq. | Sequence | Ion Score | C. I. % | Modification | Rank | Result Type |
|---|---|---|---|---|---|---|---|---|---|---|---|
| 971.4904 | 971.525 | 0.0346 | 36 | 394 | 401 | EDSLRSHK | | | | | Mascot |
| 973.4117 | 973.4948 | 0.0831 | 85 | 323 | 331 | CDLCSFASK | | | | | Mascot |
| 973.4117 | 973.4948 | 0.0831 | 85 | 323 | 331 | CDLCSFASK | | | | | Mascot |
| 1045.4077 | 1045.5449 | 0.1372 | 131 | 270 | 277 | CDFQCSTK | | | Carbamidomethyl (C)[1,5] | | Mascot |
| 1277.6783 | 1277.6868 | 0.0085 | 7 | 145 | 155 | LHTGEKPHKCK | | | | | Mascot |
| 1320.5204 | 1320.5619 | 0.0415 | 31 | 238 | 248 | CDLCDYRCAMK | | | | | Mascot |
| 1434.5632 | 1434.745 | 0.1818 | 127 | 238 | 248 | CDLCDYRCAMK | | | Carbamidomethyl (C)[1,4] | | Mascot |
| 1699.8003 | 1699.9062 | 0.1059 | 62 | 41 | 54 | QNCCQIPSTHISVR | | | Carbamidomethyl (C)[3,4] | | Mascot |
| 2117.8474 | 2117.9473 | 0.0999 | 47 | 259 | 277 | HSASDSFHCSKCDFQCS TK | | | | | Mascot |
| 2210.9263 | 2211.0635 | 0.1372 | 62 | 286 | 305 | QHQPAQPLQCSECSYSC SSK | | | | | Mascot |
| 2233.0964 | 2233.0427 | -0.0537 | -24 | 182 | 200 | CQICPYASRNSSQLTVHL R | | | Carbamidomethyl (C)[1] | | Mascot |
| 2283.0798 | 2283.1423 | 0.0625 | 27 | 173 | 190 | IHYDERPFKCQICPYASR | | | Carbamidomethyl (C)[10] | | Mascot |
| 2283.0798 | 2283.1423 | 0.0625 | 27 | 173 | 190 | IHYDERPFKCQICPYASR | | | Carbamidomethyl (C)[10] | | Mascot |
| 2580.1638 | 2580.2661 | 0.1023 | 40 | 286 | 309 | QHQPAQPLQCSECSYSC SSKGALK | | | | | Mascot |

| | | | | | | | |
|---|---|---|---|---|---|---|---|
| 44 | Rars protein [Danio rerio] | gi\|182892034 | 75755 | 6.48 | 12 | 30 | 0 |

**Peptide Information**

| Calc. Mass | Obsrv. Mass | ± da | ± ppm | Start Seq. | End Seq. | Sequence | Ion Score | C. I. % | Modification | Rank | Result Type |
|---|---|---|---|---|---|---|---|---|---|---|---|
| 973.4948 | 973.4948 | 0 | 0 | 354 | 362 | GLVEVDEGR | | | | | Mascot |
| 973.4948 | 973.4948 | 0 | 0 | 354 | 362 | GLVEVDEGR | 2 | 0 | | | Mascot |
| 1058.4313 | 1058.5728 | 0.1415 | 134 | 281 | 288 | FDEDEEFK | | | | | Mascot |
| 1316.7242 | 1316.6753 | -0.0489 | -37 | 463 | 473 | LMDLLEEGLKR | | | | | Mascot |
| 1328.7321 | 1328.7145 | -0.0176 | -13 | 649 | 660 | GFDILGINPVQR | | | | | Mascot |

| | | | | | | | | | | | | |
|---|---|---|---|---|---|---|---|---|---|---|---|---|
| 1385.7463 | 1385.7235 | -0.0228 | -16 | 225 | 235 | LFEFLGYEVLR | | | | | | Mascot |
| 1460.7532 | 1460.7313 | -0.0219 | -15 | 529 | 541 | GNTAAYLLYAFTR | | | | | | Mascot |
| 1475.7675 | 1475.7279 | -0.0396 | -27 | 649 | 661 | GFDILGINPVQRM | | | Oxidation (M)[13] | | | Mascot |
| 1495.8267 | 1495.8158 | -0.0109 | -7 | 155 | 168 | TEIAGPGFINVHLK | | | | | | Mascot |
| 1584.838 | 1584.8456 | 0.0076 | 5 | 437 | 451 | VEHAGFGVVLGEDKK | | | | | | Mascot |
| 1584.838 | 1584.8456 | 0.0076 | 5 | 437 | 451 | VEHAGFGVVLGEDKK | | | | | | Mascot |
| 1699.8723 | 1699.9062 | 0.0339 | 20 | 319 | 332 | EFQKVYNCLDISIK | | | | | | Mascot |
| 2246.9006 | 2247.063 | 0.1624 | 72 | 111 | 129 | FGDYQCNSAMAMSQMMKAK | | | Carbamidomethyl (C)[6], Oxidation (M)[10,12,15] | | | Mascot |
| 2284.0149 | 2284.1323 | 0.1174 | 51 | 335 | 353 | GESYYQDMMTAVVKEFEEK | | | | | | Mascot |
| 2300.0098 | 2300.1367 | 0.1269 | 55 | 335 | 353 | GESYYQDMMTAVVKEFEEK | | | Oxidation (M)[8] | | | Mascot |

| | | | | | | | | |
|---|---|---|---|---|---|---|---|---|
| 45 | PREDICTED: serine/threonine-protein kinase pim-2-like [Danio rerio] | gi\|326675052 | 52407.5 | 9.24 | 8 | 30 | 0 | |

**Peptide Information**

| Calc. Mass | Obsrv. Mass | ± da | ± ppm | Start Seq. | End Seq. | Sequence | Ion Score | C. I. % | Modification | Rank | Result Type |
|---|---|---|---|---|---|---|---|---|---|---|---|
| 864.4971 | 864.4794 | -0.0177 | -20 | 1 | 7 | MLRTTVK | | | Oxidation (M)[1] | | Mascot |
| 1325.6089 | 1325.719 | 0.1101 | 83 | 159 | 170 | QAVFAAQTCCQR | | | | | Mascot |
| 1325.6089 | 1325.719 | 0.1101 | 83 | 159 | 170 | QAVFAAQTCCQR | | | | | Mascot |
| 1347.7267 | 1347.6898 | -0.0369 | -27 | 143 | 154 | SYVGPIKEDAIR | | | | | Mascot |
| 1563.8013 | 1563.8088 | 0.0075 | 5 | 299 | 312 | EDLSWTKNSTALAK | | | | | Mascot |
| 1563.8013 | 1563.8088 | 0.0075 | 5 | 299 | 312 | EDLSWTKNSTALAK | | | | | Mascot |
| 1844.8969 | 1844.845 | -0.0519 | -28 | 155 | 170 | FIMKQAVFAAQTCCQR | | | | | Mascot |
| 1844.8969 | 1844.845 | -0.0519 | -28 | 155 | 170 | FIMKQAVFAAQTCCQR | | | | | Mascot |
| 2247.0928 | 2247.063 | -0.0298 | -13 | 43 | 63 | KYEIGEELGEGGFGTVYAATR | | | | | Mascot |
| 2265.9495 | 2266.0667 | 0.1172 | 52 | 258 | 275 | IMTKDIEGLSEECCDFMR | | | Carbamidomethyl (C)[13,14], Oxidation (M)[2,17] | | Mascot |
| 2283.2529 | 2283.1423 | -0.1106 | -48 | 8 | 27 | SSILPALHQPFKSQTMLQLK | | | Oxidation (M)[16] | | Mascot |
| 2283.2529 | 2283.1423 | -0.1106 | -48 | 8 | 27 | SSILPALHQPFKSQTMLQLK | | | Oxidation (M)[16] | | Mascot |

| | | | | | | | | |
|---|---|---|---|---|---|---|---|---|
| 46 | novel protein (wu:fj53d02) [Danio rerio] | gi\|122891311 | 95823.2 | 6.41 | 14 | 30 | 0 | |

**Peptide Information**

| Calc. Mass | Obsrv. Mass | ± da | ± ppm | Start Seq. | End Seq. | Sequence | Ion Score | C. I. % | Modification | Rank | Result Type |
|---|---|---|---|---|---|---|---|---|---|---|---|
| 850.4781 | 850.5255 | 0.0474 | 56 | 635 | 641 | ARYLAEK | | | | | Mascot |
| 971.6135 | 971.525 | -0.0885 | -91 | 318 | 325 | LIEKLEVK | | | | | Mascot |
| 973.4083 | 973.4948 | 0.0865 | 89 | 268 | 275 | YFDPSCGK | | | Carbamidomethyl (C)[6] | | Mascot |

| Calc. Mass | Obsrv. Mass | ± da | ± ppm | Start Seq. | End Seq. | Sequence | Ion Score | C. I. % | Modification | Rank | Result Type |
|---|---|---|---|---|---|---|---|---|---|---|---|
| 973.5002 | 973.4948 | -0.0054 | -6 | 411 | 418 | GRFYAFGR | | | | | Mascot |
| 1234.6096 | 1234.6344 | 0.0248 | 20 | 62 | 71 | DEQERCITIK | | | | | Mascot |
| 1316.7144 | 1316.6753 | -0.0391 | -30 | 430 | 441 | VRIMGPNFTPGK | | | | | Mascot |
| 1475.7896 | 1475.7279 | -0.0617 | -42 | 151 | 162 | IKPVLMMNKMDR | | | | | Mascot |
| 1493.6835 | 1493.7123 | 0.0288 | 19 | 692 | 704 | EGALCEENMRAVR | | | Oxidation (M)[9] | | Mascot |
| 1699.7852 | 1699.9062 | 0.121 | 71 | 390 | 403 | NCDPKAPLMMYISK | | | Carbamidomethyl (C)[2], Oxidation (M)[9,10] | | Mascot |
| 1707.8734 | 1707.7456 | -0.1278 | -75 | 2 | 15 | VNFTVDQIREIMDK | | | | | Mascot |
| 1773.9713 | 1773.7863 | -0.185 | -104 | 145 | 159 | QAIAERIKPVLMMNK | | | Oxidation (M)[12,13] | | Mascot |
| 1838.9139 | 1838.8802 | -0.0337 | -18 | 1 | 15 | MVNFTVDQIREIMDK | | | | | Mascot |
| 2230.0154 | 2230.1536 | 0.1382 | 62 | 370 | 389 | YRCELLYEGPGDDEAAM GIK | | | | | Mascot |
| 2371.1743 | 2371.0925 | -0.0818 | -34 | 21 | 42 | NMSVIAHVDHGKSTLTDS LVCK | | | Oxidation (M)[2] | | Mascot |
| 2371.1743 | 2371.0925 | -0.0818 | -34 | 21 | 42 | NMSVIAHVDHGKSTLTDS LVCK | | | Oxidation (M)[2] | | Mascot |

47 PREDICTED: tankyrase-1 [Danio rerio] gi|125847900 134832 6.42 14 29 0

**Peptide Information**

| Calc. Mass | Obsrv. Mass | ± da | ± ppm | Start Seq. | End Seq. | Sequence | Ion Score | C. I. % | Modification | Rank | Result Type |
|---|---|---|---|---|---|---|---|---|---|---|---|
| 870.396 | 870.5257 | 0.1297 | 149 | 1173 | 1178 | QMLFCR | | | Carbamidomethyl (C)[5], Oxidation (M)[2] | | Mascot |
| 971.5883 | 971.525 | -0.0633 | -65 | 464 | 471 | QVTELLLR | | | | | Mascot |
| 973.506 | 973.4948 | -0.0112 | -12 | 473 | 481 | GANVNEKNK | | | | | Mascot |
| 973.506 | 973.4948 | -0.0112 | -12 | 473 | 481 | GANVNEKNK | | | | | Mascot |
| 1058.584 | 1058.5728 | -0.0112 | -11 | 126 | 135 | LVDSVNVNAK | | | | | Mascot |
| 1179.6805 | 1179.5756 | -0.1049 | -89 | 688 | 696 | YEICKLLLK | | | Carbamidomethyl (C)[4] | | Mascot |
| 1279.7004 | 1279.7494 | 0.049 | 38 | 494 | 504 | AHNDILEVLQK | | | | | Mascot |
| 1493.7594 | 1493.7123 | -0.0471 | -32 | 572 | 584 | NSDVDYRLLEAAK | | | | | Mascot |
| 1495.6958 | 1495.8158 | 0.12 | 80 | 110 | 122 | ELFEACRNGDVSR | | | | | Mascot |
| 1584.8492 | 1584.8456 | -0.0036 | -2 | 962 | 974 | SLGLEHLRDIFER | | | | | Mascot |
| 1584.8492 | 1584.8456 | -0.0036 | -2 | 962 | 974 | SLGLEHLRDIFER | | | | | Mascot |
| 1633.8148 | 1633.802 | -0.0128 | -8 | 744 | 756 | VQKLCSLENINCR | | | Carbamidomethyl (C)[5,12] | | Mascot |
| 1773.7804 | 1773.7863 | 0.0059 | 3 | 1166 | 1178 | SCYICHRQMLFCR | | | Carbamidomethyl (C)[2,5] | | Mascot |
| 1838.849 | 1838.8802 | 0.0312 | 17 | 1146 | 1163 | SNQYVYGIGGGTGCPTH K | | | | | Mascot |
| 2118.0066 | 2117.9473 | -0.0593 | -28 | 585 | 603 | AGDLDTVKQLCSPQNVN CR | | | Carbamidomethyl (C)[11] | | Mascot |
| 2705.3723 | 2705.1128 | -0.2595 | -96 | 509 | 533 | MNAVDTLGQTALHRAAL AGHLQTCR | | | Carbamidomethyl (C)[24] | | Mascot |

48 PREDICTED: zinc finger protein 629 [Danio rerio] gi|292614670 69451.9 9.49 1 12 0

**Peptide Information**

| Calc. Mass | Obsrv. Mass | ± da | ± ppm | Start Seq. | End Seq. | Sequence | Ion Score | C. I. % | Modification | Rank | Result Type |
|---|---|---|---|---|---|---|---|---|---|---|---|
| 973.5214 | 973.4948 | -0.0266 | -27 | 31 | 38 | DKGITFHR | 12 | 0 | | 2 | Mascot |

| | | | | | | | | |
|---|---|---|---|---|---|---|---|---|
| 49 | PREDICTED: inositol 1,4,5-trisphosphate receptor type 2, partial [Danio rerio] | gi\|326676026 | 226478.9 | 6.12 | 1 | | 11 | 0 |

Peptide Information

| Calc. Mass | Obsrv. Mass | ± da | ± ppm | Start Seq. | End Seq. | Sequence | Ion Score | C. I. % | Modification | Rank | Result Type |
|---|---|---|---|---|---|---|---|---|---|---|---|
| 1325.6154 | 1325.719 | 0.1036 | 78 | 1675 | 1685 | EMFDEKGNALR | 13 | 0 | Oxidation (M)[2] | 2 | Mascot |

| | | | | | | | | |
|---|---|---|---|---|---|---|---|---|
| 50 | COMM domain-containing protein 1 [Danio rerio] | gi\|113674991 | 21219.7 | 5.48 | 1 | | 11 | 0 |

Peptide Information

| Calc. Mass | Obsrv. Mass | ± da | ± ppm | Start Seq. | End Seq. | Sequence | Ion Score | C. I. % | Modification | Rank | Result Type |
|---|---|---|---|---|---|---|---|---|---|---|---|
| 1844.8807 | 1844.845 | -0.0357 | -19 | 174 | 189 | NMAELQESIDSIVHRS | 11 | 0 | Oxidation (M)[2] | 2 | Mascot |

| | | | | | |
|---|---|---|---|---|---|
| **Gel Idx/Pos** | 187/H14 | **Instr./Gel Origin** | BA2060/110707A | **Process Status** | Analysis Succeeded |
| **Plate [#] Name** | [1] 31496 | **Instrument Sample Name** | | **Spectra** | 11 |

| Rank | Protein Name | Accession No. | Protein MW | Protein PI | Pep. Count | Protein Score | Protein Score C. I. % | Total Ion Score | Total Ion C. I. % |
|---|---|---|---|---|---|---|---|---|---|
| 1 | transferrin-a [Danio rerio] | gi\|220672954 | 73234.7 | 6.61 | 17 | 166 | 100 | 103 | 100 |

Peptide Information

| Calc. Mass | Obsrv. Mass | ± da | ± ppm | Start Seq. | End Seq. | Sequence | Ion Score | C. I. % | Modification | Rank | Result Type |
|---|---|---|---|---|---|---|---|---|---|---|---|
| 1149.5898 | 1149.5762 | -0.0136 | -12 | 108 | 118 | AGSGFNINELK | | | | | Mascot |
| 1184.4572 | 1184.4415 | -0.0157 | -13 | 121 | 129 | SSCHSCYQR | | | Carbamidomethyl (C)[3,6] | | Mascot |
| 1420.6201 | 1420.5984 | -0.0217 | -15 | 196 | 207 | YFGDDGAFQCLK | | | Carbamidomethyl (C)[10] | | Mascot |
| 1485.6461 | 1485.6229 | -0.0232 | -16 | 227 | 238 | QNYELLCMDGSR | | | Carbamidomethyl (C)[7] | | Mascot |
| 1557.7366 | 1557.7135 | -0.0231 | -15 | 146 | 158 | ITWEGPNEMPVER | | | | | Mascot |
| 1572.7792 | 1572.7548 | -0.0244 | -16 | 261 | 273 | TDTDLQYVYDVLK | | | | | Mascot |
| 1573.7314 | 1573.7151 | -0.0163 | -10 | 146 | 158 | ITWEGPNEMPVER | | | Oxidation (M)[9] | | Mascot |
| 1573.7314 | 1573.7151 | -0.0163 | -10 | 146 | 158 | ITWEGPNEMPVER | 10 | 0 | Oxidation (M)[9] | | Mascot |
| 1577.858 | 1577.8296 | -0.0284 | -18 | 577 | 590 | CNLAQVPVHAVITR | | | Carbamidomethyl (C)[1] | | Mascot |
| 1638.7349 | 1638.8279 | 0.093 | 57 | 360 | 373 | SCILEASVDDCIEK | | | Carbamidomethyl (C)[2,11] | | Mascot |
| 1652.787 | 1652.8027 | 0.0157 | 9 | 359 | 373 | KSCILEASVDDCIEK | | | | | Mascot |

| | | | | | | | | | | | |
|---|---|---|---|---|---|---|---|---|---|---|---|
| 1752.8802 | 1752.8513 | -0.0289 | -16 | 378 | 394 | EADFLAVDGGQVYIAGK | | | | | Mascot |
| 1912.9473 | 1912.9166 | -0.0307 | -16 | 159 | 176 | AVSEFFSSSCVPGVSKP K | | | Carbamidomethyl (C)[10] | | Mascot |
| 2028.9299 | 2028.8933 | -0.0366 | -18 | 45 | 61 | AADIECHLQPTVIDCMR | | | Carbamidomethyl (C)[6,15] | | Mascot |
| 2040.8756 | 2040.8425 | -0.0331 | -16 | 504 | 520 | CKPSAEEQYYGYDGAFR | | | Carbamidomethyl (C)[1] | | Mascot |
| 2040.8756 | 2040.8425 | -0.0331 | -16 | 504 | 520 | CKPSAEEQYYGYDGAFR | 93 | 100 | Carbamidomethyl (C)[1] | | Mascot |
| 2044.9249 | 2044.9039 | -0.021 | -10 | 45 | 61 | AADIECHLQPTVIDCMR | | | Carbamidomethyl (C)[6,15], Oxidation (M)[16] | | Mascot |
| 2179.9937 | 2179.9604 | -0.0333 | -15 | 208 | 226 | NDNGQVAFVCHHAIPES ER | | | Carbamidomethyl (C)[10] | | Mascot |
| 2300.1768 | 2300.1436 | -0.0332 | -14 | 595 | 615 | SAVVSFLSDIQSKNNDLF TSK | | | | | Mascot |
| 3338.5085 | 3338.7083 | 0.1998 | 60 | 395 | 427 | CGLVPVMAEQSNSQSCS SGSGGTASYYAVAVVR | | | Carbamidomethyl (C)[1], Oxidation (M)[7] | | Mascot |
| 3459.8481 | 3459.7966 | -0.0515 | -15 | 62 | 95 | SIAAGGTDIVTVDGANVF TGGLNNYLLRPIIAEK | | | | | Mascot |

| | | | | | | | | | | |
|---|---|---|---|---|---|---|---|---|---|---|
| 2 | Transferrin-a [Danio rerio] | gi\|112180541 | 56852.3 | 5.58 | 12 | 135 | 100 | 93 | 100 | |

Peptide Information

| Calc. Mass | Obsrv. Mass | ± da | ± ppm | Start Seq. | End Seq. | Sequence | Ion Score | C. I. % | Modification | Rank | Result Type |
|---|---|---|---|---|---|---|---|---|---|---|---|
| 1420.6201 | 1420.5984 | -0.0217 | -15 | 43 | 54 | YFGDDGAFQCLK | | | Carbamidomethyl (C)[10] | | Mascot |
| 1485.6461 | 1485.6229 | -0.0232 | -16 | 74 | 85 | QNYELLCMDGSR | | | Carbamidomethyl (C)[7] | | Mascot |
| 1572.7792 | 1572.7548 | -0.0244 | -16 | 108 | 120 | TDTDLQYVYDVLK | | | | | Mascot |
| 1577.858 | 1577.8296 | -0.0284 | -18 | 424 | 437 | CNLAQVPVHAVITR | | | Carbamidomethyl (C)[1] | | Mascot |
| 1638.7349 | 1638.8279 | 0.093 | 57 | 207 | 220 | SCILEASVDDCIEK | | | Carbamidomethyl (C)[2,11] | | Mascot |
| 1652.787 | 1652.8027 | 0.0157 | 9 | 206 | 220 | KSCILEASVDDCIEK | | | | | Mascot |
| 1752.8802 | 1752.8513 | -0.0289 | -16 | 225 | 241 | EADFLAVDGGQVYIAGK | | | | | Mascot |
| 1912.9473 | 1912.9166 | -0.0307 | -16 | 6 | 23 | AVSEFFSSSCVPGVSKP K | | | Carbamidomethyl (C)[10] | | Mascot |
| 2040.8756 | 2040.8425 | -0.0331 | -16 | 351 | 367 | CKPSAEEQYYGYDGAFR | | | Carbamidomethyl (C)[1] | | Mascot |
| 2040.8756 | 2040.8425 | -0.0331 | -16 | 351 | 367 | CKPSAEEQYYGYDGAFR | 93 | 100 | Carbamidomethyl (C)[1] | | Mascot |
| 2179.9937 | 2179.9604 | -0.0333 | -15 | 55 | 73 | NDNGQVAFVCHHAIPES ER | | | Carbamidomethyl (C)[10] | | Mascot |
| 2300.1768 | 2300.1436 | -0.0332 | -14 | 442 | 462 | SAVVSFLSDIQSKNNDLF TSK | | | | | Mascot |
| 3338.5085 | 3338.7083 | 0.1998 | 60 | 242 | 274 | CGLVPVMAEQSNSQSCS SGSGGTASYYAVAVVR | | | Carbamidomethyl (C)[1], Oxidation (M)[7] | | Mascot |

| | | | | | | | | | | |
|---|---|---|---|---|---|---|---|---|---|---|
| 3 | Transferrin-a [Danio rerio] | gi\|159155968 | 56844.4 | 5.78 | 12 | 133 | 100 | 93 | 100 | |

Peptide Information

| Calc. Mass | Obsrv. Mass | ± da | ± ppm | Start Seq. | End Seq. | Sequence | Ion Score | C. I. % | Modification | Rank | Result Type |
|---|---|---|---|---|---|---|---|---|---|---|---|
| 1420.6201 | 1420.5984 | -0.0217 | -15 | 43 | 54 | YFGDDGAFQCLK | | | Carbamidomethyl (C)[10] | | Mascot |
| 1485.6461 | 1485.6229 | -0.0232 | -16 | 74 | 85 | QNYELLCMDGSR | | | Carbamidomethyl (C)[7] | | Mascot |

| Calc. Mass | Obsrv. Mass | ± da | ± ppm | Start Seq. | End Seq. | Sequence | Ion Score | C. I. % | Modification | Rank | Result Type |
|---|---|---|---|---|---|---|---|---|---|---|---|
| 1572.7145 | 1572.7548 | 0.0403 | 26 | 74 | 86 | QNYELLCMDGSRK | | | Oxidation (M)[8] | | Mascot |
| 1577.858 | 1577.8296 | -0.0284 | -18 | 424 | 437 | CNLAQVPVHAVITR | | | Carbamidomethyl (C)[1] | | Mascot |
| 1638.7349 | 1638.8279 | 0.093 | 57 | 207 | 220 | SCILEASVDDCIEK | | | Carbamidomethyl (C)[2,11] | | Mascot |
| 1652.787 | 1652.8027 | 0.0157 | 9 | 206 | 220 | KSCILEASVDDCIEK | | | | | Mascot |
| 1752.8802 | 1752.8513 | -0.0289 | -16 | 225 | 241 | EADFLAVDGGQVYIAGK | | | | | Mascot |
| 1912.9473 | 1912.9166 | -0.0307 | -16 | 6 | 23 | AVSEFFSSSCVPGVSKPK | | | Carbamidomethyl (C)[10] | | Mascot |
| 2040.8756 | 2040.8425 | -0.0331 | -16 | 351 | 367 | CKPSAEEQYYGYDGAFR | | | Carbamidomethyl (C)[1] | | Mascot |
| 2040.8756 | 2040.8425 | -0.0331 | -16 | 351 | 367 | CKPSAEEQYYGYDGAFR | 93 | 100 | Carbamidomethyl (C)[1] | | Mascot |
| 2179.9937 | 2179.9604 | -0.0333 | -15 | 55 | 73 | NDNGQVAFVCHHAIPESER | | | Carbamidomethyl (C)[10] | | Mascot |
| 2246.9338 | 2247.0632 | 0.1294 | 58 | 319 | 339 | FFSEGCAPGADPTSNMCKPCK | | | Carbamidomethyl (C)[6] | | Mascot |
| 2300.1768 | 2300.1436 | -0.0332 | -14 | 442 | 462 | SAVVSFLSDIQSKNNDLFTSK | | | | | Mascot |

4 echinoderm microtubule-associated protein-like 2 [Danio rerio] gi|71834606 88474.1 6.51 17 118 100 59 99.973

Peptide Information

| Calc. Mass | Obsrv. Mass | ± da | ± ppm | Start Seq. | End Seq. | Sequence | Ion Score | C. I. % | Modification | Rank | Result Type |
|---|---|---|---|---|---|---|---|---|---|---|---|
| 882.4791 | 882.5546 | 0.0755 | 86 | 39 | 45 | LSHLEQR | | | | | Mascot |
| 1045.4467 | 1045.5497 | 0.103 | 99 | 150 | 160 | TTSSSSSTCGK | | | | | Mascot |
| 1084.5575 | 1084.543 | -0.0145 | -13 | 213 | 220 | LQWVYGYR | 35 | 93.294 | | | Mascot |
| 1084.5575 | 1084.543 | -0.0145 | -13 | 213 | 220 | LQWVYGYR | | | | | Mascot |
| 1118.5841 | 1118.5657 | -0.0184 | -16 | 598 | 606 | WLVLDTDTR | | | | | Mascot |
| 1216.611 | 1216.5925 | -0.0185 | -15 | 474 | 482 | VLLWDHDYR | | | | | Mascot |
| 1344.7059 | 1344.6853 | -0.0206 | -15 | 474 | 483 | VLLWDHDYRK | | | | | Mascot |
| 1441.7004 | 1441.679 | -0.0214 | -15 | 753 | 764 | VHLFSNPCSQPR | | | Carbamidomethyl (C)[8] | | Mascot |
| 1446.5953 | 1446.574 | -0.0213 | -15 | 167 | 178 | ECSFNADEGYVR | | | Carbamidomethyl (C)[2] | | Mascot |
| 1463.6835 | 1463.6587 | -0.0248 | -17 | 485 | 497 | SEMEVPEAFGPVR | | | Oxidation (M)[3] | | Mascot |
| 1591.7784 | 1591.7767 | -0.0017 | -1 | 484 | 497 | KSEMEVPEAFGPVR | | | Oxidation (M)[4] | | Mascot |
| 1675.738 | 1675.7169 | -0.0211 | -13 | 165 | 178 | TKECSFNADEGYVR | | | Carbamidomethyl (C)[4] | | Mascot |
| 1719.8701 | 1719.842 | -0.0281 | -16 | 383 | 397 | SHINFWTVEGSTLTK | | | | | Mascot |
| 1752.8949 | 1752.8513 | -0.0436 | -25 | 5 | 20 | VASCGNLYDTNTLLLR | | | | | Mascot |
| 1772.9363 | 1772.9076 | -0.0287 | -16 | 444 | 460 | VVSGAHEGGIFSLCVLK | | | Carbamidomethyl (C)[14] | | Mascot |
| 1993.8894 | 1993.9467 | 0.0573 | 29 | 167 | 182 | ECSFNADEGYVRMFLR | | | Carbamidomethyl (C)[2] | | Mascot |
| 2185.1399 | 2185.105 | -0.0349 | -16 | 297 | 316 | VWDSVSLNTLHVIGAGVFDR | 24 | 5.268 | | | Mascot |
| 2185.1399 | 2185.105 | -0.0349 | -16 | 297 | 316 | VWDSVSLNTLHVIGAGVFDR | | | | | Mascot |
| 2808.2795 | 2808.3599 | 0.0804 | 29 | 326 | 350 | SNGGAHLCAVDDANDHILSVWDWQK | | | Carbamidomethyl (C)[8] | | Mascot |

| 5 | TPA_exp: transferrin [Danio rerio] | gi\|47264590 | 73483.9 | 6.81 | 16 | 65 | 98.593 | 10 | 0 |
|---|---|---|---|---|---|---|---|---|---|

Peptide Information

| Calc. Mass | Obsrv. Mass | ± da | ± ppm | Start Seq. | End Seq. | Sequence | Ion Score | C. I. % | Modification | Rank | Result Type |
|---|---|---|---|---|---|---|---|---|---|---|---|
| 1149.5898 | 1149.5762 | -0.0136 | -12 | 108 | 118 | AGSGFNINELK | | | | | Mascot |
| 1184.4572 | 1184.4415 | -0.0157 | -13 | 121 | 129 | SSCHSCYQR | | | Carbamidomethyl (C)[3,6] | | Mascot |
| 1420.6201 | 1420.5984 | -0.0217 | -15 | 196 | 207 | YFGDDGAFQCLK | | | Carbamidomethyl (C)[10] | | Mascot |
| 1485.6461 | 1485.6229 | -0.0232 | -16 | 227 | 238 | QNYELLCMDGSR | | | Carbamidomethyl (C)[7] | | Mascot |
| 1557.7366 | 1557.7135 | -0.0231 | -15 | 146 | 158 | ITWEGPNEMPVER | | | | | Mascot |
| 1572.7792 | 1572.7548 | -0.0244 | -16 | 261 | 273 | TDTDLQYVYDVLK | | | | | Mascot |
| 1573.7314 | 1573.7151 | -0.0163 | -10 | 146 | 158 | ITWEGPNEMPVER | | | Oxidation (M)[9] | | Mascot |
| 1573.7314 | 1573.7151 | -0.0163 | -10 | 146 | 158 | ITWEGPNEMPVER | 10 | 0 | Oxidation (M)[9] | | Mascot |
| 1577.858 | 1577.8296 | -0.0284 | -18 | 579 | 592 | CNLAQVPVHAVITR | | | Carbamidomethyl (C)[1] | | Mascot |
| 1638.7349 | 1638.8279 | 0.093 | 57 | 360 | 373 | SCILEASVDDCIEK | | | Carbamidomethyl (C)[2,11] | | Mascot |
| 1652.787 | 1652.8027 | 0.0157 | 9 | 359 | 373 | KSCILEASVDDCIEK | | | | | Mascot |
| 1752.8802 | 1752.8513 | -0.0289 | -16 | 378 | 394 | EADFLAVDGGQVYIAGK | | | | | Mascot |
| 1912.9473 | 1912.9166 | -0.0307 | -16 | 159 | 176 | AVSEFFSSSCVPGVSKP K | | | Carbamidomethyl (C)[10] | | Mascot |
| 2028.9299 | 2028.8933 | -0.0366 | -18 | 45 | 61 | AADIECHLQPTVIDCMR | | | Carbamidomethyl (C)[6,15] | | Mascot |
| 2044.9249 | 2044.9039 | -0.021 | -10 | 45 | 61 | AADIECHLQPTVIDCMR | | | Carbamidomethyl (C)[6,15], Oxidation (M)[16] | | Mascot |
| 2179.9937 | 2179.9604 | -0.0333 | -15 | 208 | 226 | NDNGQVAFVCHHAIPES ER | | | Carbamidomethyl (C)[10] | | Mascot |
| 2300.1768 | 2300.1436 | -0.0332 | -14 | 597 | 617 | SAVVSFLSDIQSKNNDLF TSK | | | | | Mascot |
| 3338.5085 | 3338.7083 | 0.1998 | 60 | 395 | 427 | CGLVPVMAEQSNSQSCS SGSGGTASYYAVAVVR | | | Carbamidomethyl (C)[1], Oxidation (M)[7] | | Mascot |
| 3459.8481 | 3459.7966 | -0.0515 | -15 | 62 | 95 | SIAAGGTDIVTVDGANVF TGGLNNYLLRPIIAEK | | | | | Mascot |

| 6 | transferrin-a [Danio rerio] | gi\|220672955 | 31328.3 | 7.83 | 10 | 61 | 96.383 | 10 | 0 |
|---|---|---|---|---|---|---|---|---|---|

Peptide Information

| Calc. Mass | Obsrv. Mass | ± da | ± ppm | Start Seq. | End Seq. | Sequence | Ion Score | C. I. % | Modification | Rank | Result Type |
|---|---|---|---|---|---|---|---|---|---|---|---|
| 1149.5898 | 1149.5762 | -0.0136 | -12 | 118 | 128 | AGSGFNINELK | | | | | Mascot |
| 1184.4572 | 1184.4415 | -0.0157 | -13 | 131 | 139 | SSCHSCYQR | | | Carbamidomethyl (C)[3,6] | | Mascot |
| 1420.6201 | 1420.5984 | -0.0217 | -15 | 206 | 217 | YFGDDGAFQCLK | | | Carbamidomethyl (C)[10] | | Mascot |
| 1485.6461 | 1485.6229 | -0.0232 | -16 | 237 | 248 | QNYELLCMDGSR | | | Carbamidomethyl (C)[7] | | Mascot |
| 1557.7366 | 1557.7135 | -0.0231 | -15 | 156 | 168 | ITWEGPNEMPVER | | | | | Mascot |
| 1572.7792 | 1572.7548 | -0.0244 | -16 | 271 | 283 | TDTDLQYVYDVLK | | | | | Mascot |
| 1573.7314 | 1573.7151 | -0.0163 | -10 | 156 | 168 | ITWEGPNEMPVER | | | Oxidation (M)[9] | | Mascot |

| Calc. Mass | Obsrv. Mass | ± da | ± ppm | Start Seq. | End Seq. | Sequence | Ion Score | C. I. % | Modification | Rank | Result Type |
|---|---|---|---|---|---|---|---|---|---|---|---|
| 1573.7314 | 1573.7151 | -0.0163 | -10 | 156 | 168 | ITWEGPNEMPVER | 10 | 0 | Oxidation (M)[9] | | Mascot |
| 1912.9473 | 1912.9166 | -0.0307 | -16 | 169 | 186 | AVSEFFSSSCVPGVSKPK | | | Carbamidomethyl (C)[10] | | Mascot |
| 2028.9299 | 2028.8933 | -0.0366 | -18 | 55 | 71 | AADIECHLQPTVIDCMR | | | Carbamidomethyl (C)[6,15] | | Mascot |
| 2044.9249 | 2044.9039 | -0.021 | -10 | 55 | 71 | AADIECHLQPTVIDCMR | | | Carbamidomethyl (C)[6,15], Oxidation (M)[16] | | Mascot |
| 2179.9937 | 2179.9604 | -0.0333 | -15 | 218 | 236 | NDNGQVAFVCHHAIPESER | | | Carbamidomethyl (C)[10] | | Mascot |
| 3459.8481 | 3459.7966 | -0.0515 | -15 | 72 | 105 | SIAAGGTDIVTVDGANVFTGGLNNYLLRPIIAEK | | | | | Mascot |

7 mutant transferrin [Danio rerio] gi|183583908 32529.2 8.44 10 56 90.037 10 0

**Peptide Information**

| Calc. Mass | Obsrv. Mass | ± da | ± ppm | Start Seq. | End Seq. | Sequence | Ion Score | C. I. % | Modification | Rank | Result Type |
|---|---|---|---|---|---|---|---|---|---|---|---|
| 1149.5898 | 1149.5762 | -0.0136 | -12 | 108 | 118 | AGSGFNINELK | | | | | Mascot |
| 1184.4572 | 1184.4415 | -0.0157 | -13 | 121 | 129 | SSCHSCYQR | | | Carbamidomethyl (C)[3,6] | | Mascot |
| 1420.6201 | 1420.5984 | -0.0217 | -15 | 196 | 207 | YFGDDGAFQCLK | | | Carbamidomethyl (C)[10] | | Mascot |
| 1485.6461 | 1485.6229 | -0.0232 | -16 | 227 | 238 | QNYELLCMDGSR | | | Carbamidomethyl (C)[7] | | Mascot |
| 1557.7366 | 1557.7135 | -0.0231 | -15 | 146 | 158 | ITWEGPNEMPVER | | | | | Mascot |
| 1572.7792 | 1572.7548 | -0.0244 | -16 | 261 | 273 | TDTDLQYVYDVLK | | | | | Mascot |
| 1573.7314 | 1573.7151 | -0.0163 | -10 | 146 | 158 | ITWEGPNEMPVER | | | Oxidation (M)[9] | | Mascot |
| 1573.7314 | 1573.7151 | -0.0163 | -10 | 146 | 158 | ITWEGPNEMPVER | 10 | 0 | Oxidation (M)[9] | | Mascot |
| 1912.9473 | 1912.9166 | -0.0307 | -16 | 159 | 176 | AVSEFFSSSCVPGVSKPK | | | Carbamidomethyl (C)[10] | | Mascot |
| 2028.9299 | 2028.8933 | -0.0366 | -18 | 45 | 61 | AADIECHLQPTVIDCMR | | | Carbamidomethyl (C)[6,15] | | Mascot |
| 2044.9249 | 2044.9039 | -0.021 | -10 | 45 | 61 | AADIECHLQPTVIDCMR | | | Carbamidomethyl (C)[6,15], Oxidation (M)[16] | | Mascot |
| 2179.9937 | 2179.9604 | -0.0333 | -15 | 208 | 226 | NDNGQVAFVCHHAIPESER | | | Carbamidomethyl (C)[10] | | Mascot |
| 3459.8481 | 3459.7966 | -0.0515 | -15 | 62 | 95 | SIAAGGTDIVTVDGANVFTGGLNNYLLRPIIAEK | | | | | Mascot |

8 PREDICTED: hypothetical protein LOC100537957 [Danio rerio] gi|326680154 13500.6 8.77 8 50 53.402

**Peptide Information**

| Calc. Mass | Obsrv. Mass | ± da | ± ppm | Start Seq. | End Seq. | Sequence | Ion Score | C. I. % | Modification | Rank | Result Type |
|---|---|---|---|---|---|---|---|---|---|---|---|
| 1084.5634 | 1084.543 | -0.0204 | -19 | 5 | 14 | DQAEVNAIPK | | | | | Mascot |
| 1084.5634 | 1084.543 | -0.0204 | -19 | 5 | 14 | DQAEVNAIPK | | | | | Mascot |
| 1184.4976 | 1184.4415 | -0.0561 | -47 | 75 | 83 | AEMFHCQFR | | | Oxidation (M)[3] | | Mascot |
| 1441.6351 | 1441.679 | 0.0439 | 30 | 73 | 83 | EKAEMFHCQFR | | | Oxidation (M)[5] | | Mascot |
| 1475.7377 | 1475.7318 | -0.0059 | -4 | 35 | 48 | WLSISESGVSGPEK | | | | | Mascot |
| 1557.709 | 1557.7135 | 0.0045 | 3 | 75 | 86 | AEMFHCQFRNFK | | | | | Mascot |

| Calc. Mass | Obsrv. Mass | ± da | ± ppm | Start Seq. | End Seq. | Sequence | Ion Score | C. I. % | Modification | Rank | Result Type |
|---|---|---|---|---|---|---|---|---|---|---|---|
| 1573.7039 | 1573.7151 | 0.0112 | 7 | 75 | 86 | AEMFHCQFRNFK | | | Oxidation (M)[3] | | Mascot |
| 1573.7891 | 1573.7151 | -0.074 | -47 | 1 | 14 | MVDKDQAEVNAIPK | | | Oxidation (M)[1] | | Mascot |
| 2225.0344 | 2225.085 | 0.0506 | 23 | 94 | 111 | NNWETIGHLWSNFGRCYK | | | | | Mascot |
| 2225.0344 | 2225.085 | 0.0506 | 23 | 94 | 111 | NNWETIGHLWSNFGRCYK | | | | | Mascot |
| 3459.7366 | 3459.7966 | 0.06 | 17 | 5 | 34 | DQAEVNAIPKVFNESDILLCWYHVTQAVTR | | | | | Mascot |

9 IGF-IRa tyrosine kinase receptor [Danio rerio] gi|20302782 14771.6 8.66 7 46 0

**Peptide Information**

| Calc. Mass | Obsrv. Mass | ± da | ± ppm | Start Seq. | End Seq. | Sequence | Ion Score | C. I. % | Modification | Rank | Result Type |
|---|---|---|---|---|---|---|---|---|---|---|---|
| 1084.6011 | 1084.543 | -0.0581 | -54 | 115 | 123 | FVHRDLAAR | | | | | Mascot |
| 1084.6011 | 1084.543 | -0.0581 | -54 | 115 | 123 | FVHRDLAAR | | | | | Mascot |
| 1485.7908 | 1485.6229 | -0.1679 | -113 | 82 | 95 | EQGSSSQSLPPLKK | | | | | Mascot |
| 1572.8228 | 1572.7548 | -0.068 | -43 | 80 | 94 | SKEQGSSSQSLPPLK | | | | | Mascot |
| 2040.9551 | 2040.8425 | -0.1126 | -55 | 96 | 114 | MIQMAGEIADGMAYLNANK | | | | | Mascot |
| 2040.9551 | 2040.8425 | -0.1126 | -55 | 96 | 114 | MIQMAGEIADGMAYLNANK | | | | | Mascot |
| 2185.0449 | 2185.105 | 0.0601 | 28 | 95 | 114 | KMIQMAGEIADGMAYLNANK | | | Oxidation (M)[2] | | Mascot |
| 2185.2083 | 2185.105 | -0.1033 | -47 | 49 | 68 | LLGVVSQGQPTLVIMELMTR | | | | | Mascot |
| 3338.717 | 3338.7083 | -0.0087 | -3 | 40 | 68 | EFNCHHVVRLLGVVSQGQPTLVIMELMTR | | | Oxidation (M)[24,27] | | Mascot |

10 PREDICTED: homeobox protein Hox-C11a isoform 2 [Danio rerio] gi|68437229 34998.9 9.1 9 43 0

**Peptide Information**

| Calc. Mass | Obsrv. Mass | ± da | ± ppm | Start Seq. | End Seq. | Sequence | Ion Score | C. I. % | Modification | Rank | Result Type |
|---|---|---|---|---|---|---|---|---|---|---|---|
| 842.4114 | 842.4988 | 0.0874 | 104 | 226 | 233 | SGHSTTPR | | | | | Mascot |
| 1216.6208 | 1216.5925 | -0.0283 | -23 | 74 | 84 | DLPFELNPSGK | | | | | Mascot |
| 1320.6622 | 1320.6135 | -0.0487 | -37 | 252 | 261 | EFFFNVYINK | | | | | Mascot |
| 1441.8485 | 1441.679 | -0.1695 | -118 | 265 | 276 | LQLSRILNLTDR | | | | | Mascot |
| 1572.7223 | 1572.7548 | 0.0325 | 21 | 2 | 15 | FNSVNIGNFCSQSR | | | | | Mascot |
| 1577.7998 | 1577.8296 | 0.0298 | 19 | 252 | 263 | EFFFNVYINKEK | | | | | Mascot |
| 1675.861 | 1675.7169 | -0.1441 | -86 | 176 | 192 | GSSGKPESASQVSSVLR | | | | | Mascot |
| 1719.7577 | 1719.842 | 0.0843 | 49 | 1 | 15 | MFNSVNIGNFCSQSR | | | Oxidation (M)[1] | | Mascot |
| 1772.7573 | 1772.9076 | 0.1503 | 85 | 104 | 118 | DCLPPSATMTEMLMK | | | Carbamidomethyl (C)[2], Oxidation (M)[9,12,14] | | Mascot |

11 novel protein similar to vertebrate copine IV (CPNE4) [Danio rerio] gi|94732437 25404.6 5.47 8 42 0

Peptide Information

| Calc. Mass | Obsrv. Mass | ± da | ± ppm | Start Seq. | End Seq. | Sequence | Ion Score | C. I. % | Modification | Rank | Result Type |
|---|---|---|---|---|---|---|---|---|---|---|---|
| 996.4819 | 996.5818 | 0.0999 | 100 | 66 | 74 | SCINPTFSK | | | | | Mascot |
| 996.4819 | 996.5818 | 0.0999 | 100 | 66 | 74 | SCINPTFSK | | | | | Mascot |
| 1441.7719 | 1441.679 | -0.0929 | -64 | 36 | 48 | DALSKPDPCVVIK | | | Carbamidomethyl (C)[9] | | Mascot |
| 1475.6543 | 1475.7318 | 0.0775 | 53 | 209 | 221 | TSLNSLCSGDHER | | | Carbamidomethyl (C)[7] | | Mascot |
| 1485.6903 | 1485.6229 | -0.0674 | -45 | 49 | 60 | MQSHGQWLEVDR | | | | | Mascot |
| 1772.896 | 1772.9076 | 0.0116 | 7 | 209 | 224 | TSLNSLCSGDHERILK | | | | | Mascot |
| 1913.016 | 1912.9166 | -0.0994 | -52 | 31 | 48 | GISDRDALSKPDPCVVIK | | | | | Mascot |
| 2023.9246 | 2023.8156 | -0.109 | -54 | 104 | 122 | EADCLGAMECTLGQIVS QR | | | | | Mascot |
| 2023.9246 | 2023.8156 | -0.109 | -54 | 104 | 122 | EADCLGAMECTLGQIVS QR | | | | | Mascot |
| 2225.0359 | 2225.085 | 0.0491 | 22 | 104 | 123 | EADCLGAMECTLGQIVS QRK | | | Carbamidomethyl (C)[4], Oxidation (M)[8] | | Mascot |
| 2225.0359 | 2225.085 | 0.0491 | 22 | 104 | 123 | EADCLGAMECTLGQIVS QRK | | | Carbamidomethyl (C)[4], Oxidation (M)[8] | | Mascot |

| | | | | | | | | |
|---|---|---|---|---|---|---|---|---|
| 12 | T-cell receptor alpha variable region [Danio rerio] | gi\|11139444 | 6844.6 | 9.67 | 5 | 42 | 0 | |

Peptide Information

| Calc. Mass | Obsrv. Mass | ± da | ± ppm | Start Seq. | End Seq. | Sequence | Ion Score | C. I. % | Modification | Rank | Result Type |
|---|---|---|---|---|---|---|---|---|---|---|---|
| 1084.6262 | 1084.543 | -0.0832 | -77 | 30 | 38 | ERFHAALIK | | | | | Mascot |
| 1084.6262 | 1084.543 | -0.0832 | -77 | 30 | 38 | ERFHAALIK | | | | | Mascot |
| 1320.7092 | 1320.6135 | -0.0957 | -72 | 6 | 16 | INGVPRYMLNK | | | Oxidation (M)[8] | | Mascot |
| 1420.6414 | 1420.5984 | -0.043 | -30 | 47 | 58 | DLRVSDSTVYYC | | | | | Mascot |
| 1638.9941 | 1638.8279 | -0.1662 | -101 | 32 | 46 | FHAALIKTSVPLTIK | | | | | Mascot |
| 1752.8585 | 1752.8513 | -0.0072 | -4 | 12 | 26 | YMLNKITNTSGHTEK | | | Oxidation (M)[2] | | Mascot |

| | | | | | | | | |
|---|---|---|---|---|---|---|---|---|
| 13 | PREDICTED: hypothetical protein LOC325902 [Danio rerio] | gi\|292621312 | 43251.8 | 5.91 | 9 | 39 | 0 | |

Peptide Information

| Calc. Mass | Obsrv. Mass | ± da | ± ppm | Start Seq. | End Seq. | Sequence | Ion Score | C. I. % | Modification | Rank | Result Type |
|---|---|---|---|---|---|---|---|---|---|---|---|
| 850.5145 | 850.5313 | 0.0168 | 20 | 77 | 83 | IKLASYR | | | | | Mascot |
| 1084.611 | 1084.543 | -0.068 | -63 | 2 | 10 | NLNEKKPNK | | | | | Mascot |
| 1084.611 | 1084.543 | -0.068 | -63 | 2 | 10 | NLNEKKPNK | | | | | Mascot |
| 1441.8022 | 1441.679 | -0.1232 | -85 | 55 | 66 | GIHFTVTQIRNR | | | | | Mascot |
| 1446.5632 | 1446.574 | 0.0108 | 7 | 256 | 267 | AFHCSMLGMMDR | | | Oxidation (M)[6,9,10] | | Mascot |

| Calc. Mass | Obsrv. Mass | ± da | ± ppm | Start Seq. | End Seq. | Sequence | Ion Score | C. I. % | Modification | Rank | Result Type |
|---|---|---|---|---|---|---|---|---|---|---|---|
| 1493.7028 | 1493.7094 | 0.0066 | 4 | 97 | 107 | YFHMLDHMLVR | | | Oxidation (M)[4,8] | | Mascot |
| 1719.7854 | 1719.842 | 0.0566 | 33 | 200 | 215 | TEPPSLCTPNSTESTR | | | | | Mascot |
| 1772.9388 | 1772.9076 | -0.0312 | -18 | 354 | 370 | NKNASSPPSLEISSLTK | | | | | Mascot |
| 2180.157 | 2179.9604 | -0.1966 | -90 | 268 | 285 | QIQVLEQLSNFTRNHQPK | | | | | Mascot |
| 2233.04 | 2233.0535 | 0.0135 | 6 | 335 | 353 | RSPSEPTIENEISQTMEE R | | | | | Mascot |

| | | | | | | | |
|---|---|---|---|---|---|---|---|
| 14 | PREDICTED: disco-interacting protein 2 homolog C [Danio rerio] | gi\|189536803 | 170745 | 7.39 | 17 | 38 | 0 |

**Peptide Information**

| Calc. Mass | Obsrv. Mass | ± da | ± ppm | Start Seq. | End Seq. | Sequence | Ion Score | C. I. % | Modification | Rank | Result Type |
|---|---|---|---|---|---|---|---|---|---|---|---|
| 882.4501 | 882.5546 | 0.1045 | 118 | 581 | 587 | VCQYKGK | | | Carbamidomethyl (C)[2] | | Mascot |
| 996.4852 | 996.5818 | 0.0966 | 97 | 1014 | 1021 | IATMLMER | | | Oxidation (M)[4,6] | | Mascot |
| 996.4852 | 996.5818 | 0.0966 | 97 | 1014 | 1021 | IATMLMER | | | Oxidation (M)[4,6] | | Mascot |
| 1045.5887 | 1045.5497 | -0.039 | -37 | 1242 | 1251 | GLGLQTESLK | | | | | Mascot |
| 1084.615 | 1084.543 | -0.072 | -66 | 572 | 580 | VNPLSWIQK | | | | | Mascot |
| 1084.615 | 1084.543 | -0.072 | -66 | 572 | 580 | VNPLSWIQK | | | | | Mascot |
| 1165.567 | 1165.7288 | 0.1618 | 139 | 1166 | 1174 | LQCELYPSR | | | Carbamidomethyl (C)[3] | | Mascot |
| 1165.567 | 1165.7288 | 0.1618 | 139 | 1166 | 1174 | LQCELYPSR | | | Carbamidomethyl (C)[3] | | Mascot |
| 1184.5841 | 1184.4415 | -0.1426 | -120 | 374 | 383 | QEPMVRPGDR | | | | | Mascot |
| 1316.6917 | 1316.6779 | -0.0138 | -10 | 605 | 615 | DQRDINLSSLR | | | | | Mascot |
| 1441.8737 | 1441.679 | -0.1947 | -135 | 254 | 266 | VSAKIQQLVNTLK | | | | | Mascot |
| 1485.806 | 1485.6229 | -0.1831 | -123 | 827 | 839 | IAVFSVTVLHDER | | | | | Mascot |
| 1493.7781 | 1493.7094 | -0.0687 | -46 | 1163 | 1174 | SIKLQCELYPSR | | | Carbamidomethyl (C)[6] | | Mascot |
| 1772.886 | 1772.9076 | 0.0216 | 12 | 1285 | 1300 | DLGLHPRAVSTSFGCR | | | Carbamidomethyl (C)[15] | | Mascot |
| 2055.0903 | 2054.8594 | -0.2309 | -112 | 928 | 947 | QKQPEIGPASVMVGNLV SGK | | | Oxidation (M)[12] | | Mascot |
| 2185.1025 | 2185.105 | 0.0025 | 1 | 1074 | 1092 | MIVEVSRSACVMTTQIISK | | | Carbamidomethyl (C)[10], Oxidation (M)[1,12] | | Mascot |
| 2185.1025 | 2185.105 | 0.0025 | 1 | 1074 | 1092 | MIVEVSRSACVMTTQIISK | | | Carbamidomethyl (C)[10], Oxidation (M)[1,12] | | Mascot |
| 2211.1074 | 2211.0718 | -0.0356 | -16 | 676 | 695 | GVLSMQGLSYGVIRVDS EER | | | Oxidation (M)[5] | | Mascot |
| 2283.092 | 2283.144 | 0.052 | 23 | 236 | 257 | YGNAELMETGDGVPVSS RVSAK | | | Oxidation (M)[7] | | Mascot |
| 2283.092 | 2283.144 | 0.052 | 23 | 236 | 257 | YGNAELMETGDGVPVSS RVSAK | | | Oxidation (M)[7] | | Mascot |
| 2514.1792 | 2514.1936 | 0.0144 | 6 | 1536 | 1556 | MHLRDGFLADQLDPIYVA YNM | | | Oxidation (M)[1,21] | | Mascot |
| 3338.7341 | 3338.7083 | -0.0258 | -8 | 1121 | 1151 | KPPQLYKPSNPDTLAYLD FSVSTTGMLAGVK | | | | | Mascot |

| | | | | | | | |
|---|---|---|---|---|---|---|---|
| 15 | methyltransferase-like protein 13 [Danio rerio] | gi\|217416336 | 75976.5 | 5.99 | 11 | 38 | 0 |

**Peptide Information**

| Calc. Mass | Obsrv. Mass | ± da | ± ppm | Start Seq. | End Seq. | Sequence | Ion Score | C. I. % | Modification | Rank | Result Type |
|---|---|---|---|---|---|---|---|---|---|---|---|
| 864.4032 | 864.5068 | 0.1036 | 120 | 504 | 510 | DFVPCAR | | | Carbamidomethyl (C)[5] | | Mascot |
| 1062.6306 | 1062.6775 | 0.0469 | 44 | 589 | 597 | KVYSLLSPR | | | | | Mascot |
| 1165.6221 | 1165.7288 | 0.1067 | 92 | 598 | 607 | GLFMLNLVCR | | | | | Mascot |
| 1165.6221 | 1165.7288 | 0.1067 | 92 | 598 | 607 | GLFMLNLVCR | | | | | Mascot |
| 1485.796 | 1485.6229 | -0.1731 | -117 | 291 | 303 | SNHFAIFIVPQGR | | | | | Mascot |
| 1533.74 | 1533.8055 | 0.0655 | 43 | 630 | 643 | GIEGEVNEVLLCCR | | | | | Mascot |
| 1573.8407 | 1573.7151 | -0.1256 | -80 | 153 | 165 | YVCITLAQEHVIK | | | Carbamidomethyl (C)[3] | | Mascot |
| 1573.8407 | 1573.7151 | -0.1256 | -80 | 153 | 165 | YVCITLAQEHVIK | | | Carbamidomethyl (C)[3] | | Mascot |
| 2040.9086 | 2040.8425 | -0.0661 | -32 | 7 | 22 | TAEEFSSADYWERFFR | | | | | Mascot |
| 2040.9086 | 2040.8425 | -0.0661 | -32 | 7 | 22 | TAEEFSSADYWERFFR | | | | | Mascot |
| 2180.0613 | 2179.9604 | -0.1009 | -46 | 569 | 588 | DTTLGMSCPPPAFVETSL LK | | | Carbamidomethyl (C)[8], Oxidation (M)[6] | | Mascot |
| 2283.1826 | 2283.144 | -0.0386 | -17 | 536 | 556 | LKVTLGDGLDHITTLESE GER | | | | | Mascot |
| 2283.1826 | 2283.144 | -0.0386 | -17 | 536 | 556 | LKVTLGDGLDHITTLESE GER | | | | | Mascot |
| 2300.1226 | 2300.1436 | 0.021 | 9 | 52 | 72 | VLVVGCGNSELSEQLYD VGYR | | | | | Mascot |
| 3337.5254 | 3337.7178 | 0.1924 | 58 | 453 | 482 | SVDRGFLCCTHHEVMVA GLAMLGMDAINNK | | | Carbamidomethyl (C)[8], Oxidation (M)[15,21,24] | | Mascot |

16 ras-related protein Rab-40C [Danio rerio] gi|94536665 31226.9 9.41 8 37 0

**Peptide Information**

| Calc. Mass | Obsrv. Mass | ± da | ± ppm | Start Seq. | End Seq. | Sequence | Ion Score | C. I. % | Modification | Rank | Result Type |
|---|---|---|---|---|---|---|---|---|---|---|---|
| 850.576 | 850.5313 | -0.0447 | -53 | 212 | 219 | LPLPVAIK | | | | | Mascot |
| 1084.5858 | 1084.543 | -0.0428 | -39 | 134 | 142 | RQVPTEQAR | | | | | Mascot |
| 1084.5858 | 1084.543 | -0.0428 | -39 | 134 | 142 | RQVPTEQAR | | | | | Mascot |
| 1149.615 | 1149.5762 | -0.0388 | -34 | 17 | 27 | FLLVGDSDVGK | | | | | Mascot |
| 1279.6252 | 1279.7557 | 0.1305 | 102 | 76 | 85 | FCTIFRSYSR | | | | | Mascot |
| 1772.7335 | 1772.9076 | 0.1741 | 98 | 224 | 239 | SFSMANGMNAVMMHGR | | | Oxidation (M)[4,8] | | Mascot |
| 2028.996 | 2028.8933 | -0.1027 | -51 | 65 | 81 | LELWDTSGQGRFCTIFR | | | | | Mascot |
| 2284.3462 | 2284.1384 | -0.2078 | -91 | 199 | 219 | AIVSCTPVHLIDKLPLPVAI K | | | Carbamidomethyl (C)[5] | | Mascot |
| 3459.6235 | 3459.7966 | 0.1731 | 50 | 143 | 172 | AYAEKNGMTFFEVSPLC NFNVIESFTELSR | | | Oxidation (M)[8] | | Mascot |

17 PREDICTED: zinc finger protein 658-like [Danio rerio] gi|326666995 54907.7 9.53 14 37 0

**Peptide Information**

| Calc. Mass | Obsrv. Mass | ± da | ± ppm | Start Seq. | End Seq. | Sequence | Ion Score | C. I. % | Modification | Rank | Result Type |
|---|---|---|---|---|---|---|---|---|---|---|---|

| Calc. Mass | Obsrv. Mass | ± da | ± ppm | Start Seq. | End Seq. | Sequence | Ion Score | C. I. % | Modification | Rank | Result Type |
|---|---|---|---|---|---|---|---|---|---|---|---|
| 1441.7183 | 1441.679 | -0.0393 | -27 | 185 | 196 | SFTHKNTLDNHK | | | | | Mascot |
| 1475.7139 | 1475.7318 | 0.0179 | 12 | 157 | 168 | SFTHKSTFNNHR | | | | | Mascot |
| 1533.6863 | 1533.8055 | 0.1192 | 78 | 465 | 477 | SFSQSSSLNHHMR | | | Oxidation (M)[12] | | Mascot |
| 1549.6635 | 1549.8024 | 0.1389 | 90 | 17 | 29 | SFSCSSHLNQHMR | | | Oxidation (M)[12] | | Mascot |
| 1557.7267 | 1557.7135 | -0.0132 | -8 | 214 | 225 | FPYKSTFNNHMR | | | Oxidation (M)[11] | | Mascot |
| 1577.7198 | 1577.8296 | 0.1098 | 70 | 226 | 240 | THTGEKPFACAQCGK | | | | | Mascot |
| 1719.7611 | 1719.842 | 0.0809 | 47 | 394 | 408 | IHTGEKPCTCTQCGK | | | Carbamidomethyl (C)[8,10] | | Mascot |
| 1752.7792 | 1752.8513 | 0.0721 | 41 | 198 | 212 | THTGEKPYRCTDCGK | | | Carbamidomethyl (C)[10] | | Mascot |
| 2054.946 | 2054.8594 | -0.0866 | -42 | 465 | 482 | SFSQSSSLNHHMRIHTGE | | | | | Mascot |
| 2181.0579 | 2180.9521 | -0.1058 | -49 | 86 | 105 | IHTGEKPFTCAQCGQSFAKK | | | | | Mascot |
| 2211.0435 | 2211.0718 | 0.0283 | 13 | 310 | 328 | IHTGEKPFTCTQCGKSFNR | | | Carbamidomethyl (C)[10] | | Mascot |
| 2808.3191 | 2808.3599 | 0.0408 | 15 | 30 | 54 | VHTGEKPFTCTQCGKSFSQSSHLNK | | | Carbamidomethyl (C)[10] | | Mascot |
| 3337.4717 | 3337.7178 | 0.2461 | 74 | 17 | 44 | SFSCSSHLNQHMRVHTGEKPFTCTQCGK | | | Carbamidomethyl (C)[4,23,26], Oxidation (M)[12] | | Mascot |
| 3338.4558 | 3338.7083 | 0.2525 | 76 | 2 | 29 | SHTGEKPFLCTECGKSFSCSSHLNQHMR | | | Carbamidomethyl (C)[10,13,19], Oxidation (M)[27] | | Mascot |

18 PREDICTED: copine-4 [Danio rerio] gi|326675160 19667.7 5.46 7 37 0

**Peptide Information**

| Calc. Mass | Obsrv. Mass | ± da | ± ppm | Start Seq. | End Seq. | Sequence | Ion Score | C. I. % | Modification | Rank | Result Type |
|---|---|---|---|---|---|---|---|---|---|---|---|
| 996.4819 | 996.5818 | 0.0999 | 100 | 66 | 74 | SCINPTFSK | | | | | Mascot |
| 996.4819 | 996.5818 | 0.0999 | 100 | 66 | 74 | SCINPTFSK | | | | | Mascot |
| 1441.7719 | 1441.679 | -0.0929 | -64 | 36 | 48 | DALSKPDPCVVIK | | | Carbamidomethyl (C)[9] | | Mascot |
| 1485.6903 | 1485.6229 | -0.0674 | -45 | 49 | 60 | MQSHGQWLEVDR | | | | | Mascot |
| 1675.7454 | 1675.7169 | -0.0285 | -17 | 163 | 176 | LDDKNGCILTYGFC | | | Carbamidomethyl (C)[7,14] | | Mascot |
| 1913.016 | 1912.9166 | -0.0994 | -52 | 31 | 48 | GISDRDALSKPDPCVVIK | | | | | Mascot |
| 2023.9246 | 2023.8156 | -0.109 | -54 | 104 | 122 | EADCLGAMECTLGQIVSQR | | | | | Mascot |
| 2023.9246 | 2023.8156 | -0.109 | -54 | 104 | 122 | EADCLGAMECTLGQIVSQR | | | | | Mascot |
| 2225.0359 | 2225.085 | 0.0491 | 22 | 104 | 123 | EADCLGAMECTLGQIVSQRK | | | Carbamidomethyl (C)[4], Oxidation (M)[8] | | Mascot |
| 2225.0359 | 2225.085 | 0.0491 | 22 | 104 | 123 | EADCLGAMECTLGQIVSQRK | | | Carbamidomethyl (C)[4], Oxidation (M)[8] | | Mascot |

19 PREDICTED: zinc finger protein 569-like [Danio rerio] gi|326667380 66620.4 9.28 16 36 0

**Peptide Information**

| Calc. Mass | Obsrv. Mass | ± da | ± ppm | Start Seq. | End Seq. | Sequence | Ion Score | C. I. % | Modification | Rank | Result Type |
|---|---|---|---|---|---|---|---|---|---|---|---|

| | | | | | | | | | | | |
|---|---|---|---|---|---|---|---|---|---|---|---|
| 870.4614 | 870.531 | 0.0696 | 80 | 501 | 507 | SQVIHMR | | | | | Mascot |
| 1045.5282 | 1045.5497 | 0.0215 | 21 | 558 | 566 | LHMAKQACK | | | Oxidation (M)[3] | | Mascot |
| 1084.572 | 1084.543 | -0.029 | -27 | 301 | 308 | YVCKHHLK | | | Carbamidomethyl (C)[3] | | Mascot |
| 1084.572 | 1084.543 | -0.029 | -27 | 301 | 308 | YVCKHHLK | | | Carbamidomethyl (C)[3] | | Mascot |
| 1320.6292 | 1320.6135 | -0.0157 | -12 | 567 | 578 | SGHNNNPQDLPK | | | | | Mascot |
| 1485.6903 | 1485.6229 | -0.0674 | -45 | 132 | 144 | SFSAKSGFNTHMR | | | Oxidation (M)[12] | | Mascot |
| 1533.6838 | 1533.8055 | 0.1217 | 79 | 95 | 107 | HFPCHQCGKSFSR | | | | | Mascot |
| 1549.7474 | 1549.8024 | 0.055 | 35 | 383 | 395 | SCSHKHNLTVHMR | | | | | Mascot |
| 1572.8856 | 1572.7548 | -0.1308 | -83 | 272 | 285 | SFAQKSTLTAHIIR | | | | | Mascot |
| 1591.7268 | 1591.7767 | 0.0499 | 31 | 57 | 69 | ELNEIEEKDQCGK | | | Carbamidomethyl (C)[11] | | Mascot |
| 1638.7655 | 1638.8279 | 0.0624 | 38 | 319 | 332 | YFICDQCGSSFKLK | | | | | Mascot |
| 1652.7632 | 1652.8027 | 0.0395 | 24 | 257 | 271 | VHTGEKPHTCQQCGK | | | | | Mascot |
| 1675.7203 | 1675.7169 | -0.0034 | -2 | 286 | 299 | HTGEKPFPCDQCEK | | | Carbamidomethyl (C)[9] | | Mascot |
| 1719.7617 | 1719.842 | 0.0803 | 47 | 317 | 330 | HKYFICDQCGSSFK | | | Carbamidomethyl (C)[6] | | Mascot |
| 1752.8083 | 1752.8513 | 0.043 | 25 | 319 | 332 | YFICDQCGSSFKLK | | | Carbamidomethyl (C)[4,7] | | Mascot |
| 2180.0237 | 2179.9604 | -0.0633 | -29 | 253 | 271 | SHFRVHTGEKPHTCQQC GK | | | | | Mascot |
| 2180.9521 | 2180.9521 | 0 | 0 | 368 | 387 | VHTGEKPYSCTECGKSC SHK | | | | | Mascot |
| 2501.0676 | 2501.21 | 0.1424 | 57 | 346 | 367 | SFICDQCGRNCISESGLE SHMK | | | Carbamidomethyl (C)[4] | | Mascot |

| | | | | | | | | |
|---|---|---|---|---|---|---|---|---|
| 20 | hypothetical protein LOC790927 [Danio rerio] | gi\|121583715 | 57741.7 | 6.08 | 11 | 36 | 0 | |

**Peptide Information**

| Calc. Mass | Obsrv. Mass | ± da | ± ppm | Start Seq. | End Seq. | Sequence | Ion Score | C. I. % | Modification | Rank | Result Type |
|---|---|---|---|---|---|---|---|---|---|---|---|
| 1045.5524 | 1045.5497 | -0.0027 | -3 | 212 | 219 | IEEEIERK | | | | | Mascot |
| 1084.5316 | 1084.543 | 0.0114 | 11 | 230 | 238 | ETRSHPAMR | | | | | Mascot |
| 1084.5316 | 1084.543 | 0.0114 | 11 | 230 | 238 | ETRSHPAMR | | | | | Mascot |
| 1118.5212 | 1118.5657 | 0.0445 | 40 | 46 | 55 | DDVDGEIDLK | | | | | Mascot |
| 1420.7036 | 1420.5984 | -0.1052 | -74 | 403 | 413 | LMDTQMEQLRR | | | | | Mascot |
| 1463.7158 | 1463.6587 | -0.0571 | -39 | 416 | 427 | QDLQARLTDEMK | | | Oxidation (M)[11] | | Mascot |
| 1493.7385 | 1493.7094 | -0.0291 | -19 | 448 | 459 | SCCEMQVLLRVR | | | Carbamidomethyl (C)[2] | | Mascot |
| 1912.9835 | 1912.9166 | -0.0669 | -35 | 130 | 147 | LRSQSEASSAIINSAHNK | | | | | Mascot |
| 2024.0117 | 2023.8156 | -0.1961 | -97 | 372 | 388 | ELQALSERYSQQCVELK | | | | | Mascot |
| 2024.0117 | 2023.8156 | -0.1961 | -97 | 372 | 388 | ELQALSERYSQQCVELK | | | | | Mascot |
| 2185.0667 | 2185.105 | 0.0383 | 18 | 352 | 370 | ALDSHTLNKQQQDEVCS LR | | | | | Mascot |
| 2185.0667 | 2185.105 | 0.0383 | 18 | 352 | 370 | ALDSHTLNKQQQDEVCS LR | | | | | Mascot |
| 2211.0017 | 2211.0718 | 0.0701 | 32 | 395 | 412 | DDSDTQIRLMDTQMEQL | | | Oxidation (M)[10] | | Mascot |

| Calc. Mass | Obsrv. Mass | ± da | ± ppm | Start Seq. | End Seq. | Sequence | Ion Score | C. I. % | Modification | Rank | Result Type |
|---|---|---|---|---|---|---|---|---|---|---|---|
| 2514.2478 | 2514.1936 | -0.0542 | -22 | 475 | 496 | R NQLHFLTTEKSSVCVCVS SYLR | | | | | Mascot |

| | | | | | | | |
|---|---|---|---|---|---|---|---|
| 21 | novel protein similar to H.sapiens OLFM1, olfactomedin 1 (OLFM1, zgc:92039) [Danio rerio] | gi\|220676963 | 17247.8 | 9.07 | 6 | 35 | 0 |

Peptide Information

| Calc. Mass | Obsrv. Mass | ± da | ± ppm | Start Seq. | End Seq. | Sequence | Ion Score | C. I. % | Modification | Rank | Result Type |
|---|---|---|---|---|---|---|---|---|---|---|---|
| 850.4781 | 850.5313 | 0.0532 | 63 | 131 | 137 | GLETKFR | | | | | Mascot |
| 864.4825 | 864.5068 | 0.0243 | 28 | 118 | 124 | DLQYVVK | | | | | Mascot |
| 1652.8312 | 1652.8027 | -0.0285 | -17 | 118 | 130 | DLQYVVKMEDQLR | | | Oxidation (M)[8] | | Mascot |
| 1675.8431 | 1675.7169 | -0.1262 | -75 | 101 | 114 | VQNMTQSIQVLDQR | | | Oxidation (M)[4] | | Mascot |
| 2040.9082 | 2040.8425 | -0.0657 | -32 | 73 | 90 | CVCTVVAPQQTMCSRDA R | | | Carbamidomethyl (C)[1], Oxidation (M)[12] | | Mascot |
| 2040.9082 | 2040.8425 | -0.0657 | -32 | 73 | 90 | CVCTVVAPQQTMCSRDA R | | | Carbamidomethyl (C)[1], Oxidation (M)[12] | | Mascot |
| 2045.0557 | 2044.9039 | -0.1518 | -74 | 101 | 117 | VQNMTQSIQVLDQRTQR | | | | | Mascot |

| | | | | | | | |
|---|---|---|---|---|---|---|---|
| 22 | PREDICTED: pericentrin [Danio rerio] | gi\|326668478 | 491858 | 4.98 | 31 | 34 | 0 |

Peptide Information

| Calc. Mass | Obsrv. Mass | ± da | ± ppm | Start Seq. | End Seq. | Sequence | Ion Score | C. I. % | Modification | Rank | Result Type |
|---|---|---|---|---|---|---|---|---|---|---|---|
| 842.4995 | 842.4988 | -0.0007 | -1 | 15 | 21 | AKLAHFR | | | | | Mascot |
| 850.4087 | 850.5313 | 0.1226 | 144 | 2129 | 2136 | TPSSMTAR | | | | | Mascot |
| 882.5155 | 882.5546 | 0.0391 | 44 | 3606 | 3612 | SEIHKLR | | | | | Mascot |
| 1045.4908 | 1045.5497 | 0.0589 | 56 | 1472 | 1480 | QAEAEVNER | | | | | Mascot |
| 1084.6361 | 1084.543 | -0.0931 | -86 | 1333 | 1341 | EEIALLQLR | | | | | Mascot |
| 1084.6361 | 1084.543 | -0.0931 | -86 | 1333 | 1341 | EEIALLQLR | | | | | Mascot |
| 1118.551 | 1118.5657 | 0.0147 | 13 | 224 | 232 | EQECLLAQK | | | Carbamidomethyl (C)[4] | | Mascot |
| 1184.5178 | 1184.4415 | -0.0763 | -64 | 4217 | 4226 | SFSSTQDQER | | | | | Mascot |
| 1316.6692 | 1316.6779 | 0.0087 | 7 | 2033 | 2045 | AEVSGLQGELEGK | | | | | Mascot |
| 1320.6365 | 1320.6135 | -0.023 | -17 | 1062 | 1072 | WAQESAMLQTR | | | | | Mascot |
| 1344.7118 | 1344.6853 | -0.0265 | -20 | 3904 | 3914 | QLQQTLTDLER | | | | | Mascot |
| 1475.7271 | 1475.7318 | 0.0047 | 3 | 2723 | 2734 | SLVERLQCDQER | | | | | Mascot |
| 1485.7366 | 1485.6229 | -0.1137 | -77 | 2046 | 2058 | GAEMETLESLLHR | | | | | Mascot |
| 1533.7941 | 1533.8055 | 0.0114 | 7 | 2298 | 2310 | LAEELESARMQLK | | | Oxidation (M)[10] | | Mascot |
| 1557.7213 | 1557.7135 | -0.0078 | -5 | 919 | 930 | QHEEEQKMLEEK | | | | | Mascot |
| 1572.7435 | 1572.7548 | 0.0113 | 7 | 1515 | 1526 | EQLRQMEETHQK | | | Oxidation (M)[6] | | Mascot |
| 1573.7162 | 1573.7151 | -0.0011 | -1 | 919 | 930 | QHEEEQKMLEEK | | | Oxidation (M)[8] | | Mascot |

| Calc. Mass | Obsrv. Mass | ± da | ± ppm | Start Seq. | End Seq. | Sequence | Ion Score | C. I. % | Modification | Rank | Result Type |
|---|---|---|---|---|---|---|---|---|---|---|---|
| 1573.7162 | 1573.7151 | -0.0011 | -1 | 919 | 930 | QHEEEQKMLEEK | | | Oxidation (M)[8] | | Mascot |
| 1577.8281 | 1577.8296 | 0.0015 | 1 | 1139 | 1151 | IKTEFQSELNNVR | | | | | Mascot |
| 1591.8915 | 1591.7767 | -0.1148 | -72 | 367 | 379 | LDNLVKELQNHLR | | | | | Mascot |
| 1638.881 | 1638.8279 | -0.0531 | -32 | 2528 | 2541 | TVTQLEQELQKGHK | | | | | Mascot |
| 1652.7809 | 1652.8027 | 0.0218 | 13 | 1501 | 1514 | MHSEELEKAASQHR | | | | | Mascot |
| 1719.8547 | 1719.842 | -0.0127 | -7 | 3592 | 3605 | TELQSSYKHQEELK | | | | | Mascot |
| 1772.9402 | 1772.9076 | -0.0326 | -18 | 3094 | 3108 | DRALADLEIHNQHLK | | | | | Mascot |
| 1993.9937 | 1993.9467 | -0.047 | -24 | 3014 | 3031 | ELHEQQEAAVAKQGEAQK | | | | | Mascot |
| 2041.14 | 2040.8425 | -0.2975 | -146 | 148 | 165 | NEIISQLSKNLQVALQSR | | | | | Mascot |
| 2041.14 | 2040.8425 | -0.2975 | -146 | 148 | 165 | NEIISQLSKNLQVALQSR | | | | | Mascot |
| 2045.0648 | 2044.9039 | -0.1609 | -79 | 711 | 728 | TSIPSSTELLLQELQEEK | | | | | Mascot |
| 2180.1128 | 2179.9604 | -0.1524 | -70 | 1206 | 1224 | AVEELVACHKVALQEQQER | | | | | Mascot |
| 2181.0745 | 2180.9521 | -0.1224 | -56 | 1579 | 1596 | LEQAFLEVQKQLDMESEK | | | Oxidation (M)[14] | | Mascot |
| 2184.9858 | 2185.105 | 0.1192 | 55 | 2116 | 2136 | LGSCVSGGQSEEKTPSSMTAR | | | Carbamidomethyl (C)[4], Oxidation (M)[18] | | Mascot |
| 2184.9858 | 2185.105 | 0.1192 | 55 | 2116 | 2136 | LGSCVSGGQSEEKTPSSMTAR | | | Carbamidomethyl (C)[4], Oxidation (M)[18] | | Mascot |
| 2247.1404 | 2247.0632 | -0.0772 | -34 | 3304 | 3321 | LELENTERLDAHFVEYLR | | | | | Mascot |
| 2284.1013 | 2284.1384 | 0.0371 | 16 | 3955 | 3974 | ENSEFSECLAALSEEKISLK | | | Carbamidomethyl (C)[8] | | Mascot |
| 2300.0935 | 2300.1436 | 0.0501 | 22 | 3545 | 3563 | LQQEQTQSHDLRCSLEAEK | | | Carbamidomethyl (C)[13] | | Mascot |
| 2501.2075 | 2501.21 | 0.0025 | 1 | 2956 | 2977 | LEELEEHVQADMVNLSALETSK | | | Oxidation (M)[12] | | Mascot |

| | | | | | | | | |
|---|---|---|---|---|---|---|---|---|
| 23 | outer dense fiber of sperm tails 3-like 2 [Danio rerio] | gi\|194578833 | 27402.8 | 10.1 | 7 | 34 | 0 | |

Peptide Information

| Calc. Mass | Obsrv. Mass | ± da | ± ppm | Start Seq. | End Seq. | Sequence | Ion Score | C. I. % | Modification | Rank | Result Type |
|---|---|---|---|---|---|---|---|---|---|---|---|
| 1084.5013 | 1084.543 | 0.0417 | 38 | 1 | 9 | MTMSETEKK | | | | | Mascot |
| 1084.5013 | 1084.543 | 0.0417 | 38 | 1 | 9 | MTMSETEKK | | | | | Mascot |
| 1279.6099 | 1279.7557 | 0.1458 | 114 | 167 | 178 | CKSGGLAENWSK | | | | | Mascot |
| 1533.7556 | 1533.8055 | 0.0499 | 33 | 76 | 89 | FGRDGTPAYSLHGR | | | | | Mascot |
| 1557.7703 | 1557.7135 | -0.0568 | -36 | 197 | 210 | QPAFSMLGRHANGR | | | Oxidation (M)[6] | | Mascot |
| 1560.7904 | 1560.8088 | 0.0184 | 12 | 94 | 109 | GGILSTPGPGTYSPEK | | | | | Mascot |
| 1719.7867 | 1719.842 | 0.0553 | 32 | 79 | 93 | DGTPAYSLHGRMENR | | | Oxidation (M)[12] | | Mascot |
| 2055.0076 | 2054.8594 | -0.1482 | -72 | 110 | 127 | ASLSSTHRKPPSYTMGYR | | | Oxidation (M)[15] | | Mascot |

| | | | | | | | | |
|---|---|---|---|---|---|---|---|---|
| 24 | novel protein [Danio rerio] | gi\|94734532 | 73379.5 | 8.71 | 11 | 34 | 0 | |

Peptide Information

| Calc. Mass | Obsrv. Mass | ± da | ± ppm | Start | End | Sequence | Ion | C. I. % | Modification | Rank | Result Type |
|---|---|---|---|---|---|---|---|---|---|---|---|

| | | | | Seq. | Seq. | | Score | | | | |
|---|---|---|---|---|---|---|---|---|---|---|---|
| 870.5267 | 870.531 | 0.0043 | 5 | 562 | 570 | RIAAGGVAR | | | | | Mascot |
| 1084.4979 | 1084.543 | 0.0451 | 42 | 402 | 410 | TAFLDTCEK | | | Carbamidomethyl (C)[7] | | Mascot |
| 1084.4979 | 1084.543 | 0.0451 | 42 | 402 | 410 | TAFLDTCEK | | | Carbamidomethyl (C)[7] | | Mascot |
| 1118.6066 | 1118.5657 | -0.0409 | -37 | 175 | 183 | SHHDQLLLR | | | | | Mascot |
| 1279.7382 | 1279.7557 | 0.0175 | 14 | 188 | 198 | LVGQHFPRVAR | | | | | Mascot |
| 1316.6627 | 1316.6779 | 0.0152 | 12 | 274 | 286 | ASTEGMGGPLKPR | | | Oxidation (M)[6] | | Mascot |
| 1320.7018 | 1320.6135 | -0.0883 | -67 | 574 | 585 | RIYVLGGENGSR | | | | | Mascot |
| 1475.7345 | 1475.7318 | -0.0027 | -2 | 144 | 155 | LQVMPLQDLCSR | | | Carbamidomethyl (C)[10], Oxidation (M)[4] | | Mascot |
| 1557.7002 | 1557.7135 | 0.0133 | 9 | 311 | 323 | DFTVDCFDPRTGK | | | Carbamidomethyl (C)[6] | | Mascot |
| 1772.9146 | 1772.9076 | -0.007 | -4 | 327 | 344 | LAALGSLLCPGCTAVGG R | | | Carbamidomethyl (C)[9,12] | | Mascot |
| 2029.0382 | 2028.8933 | -0.1449 | -71 | 294 | 310 | IVCVDLKPREDESLQER | | | | | Mascot |
| 3338.678 | 3338.7083 | 0.0303 | 9 | 371 | 401 | WISRPLMAEPRAMLGLL GCGGFLYALGGCNR | | | Oxidation (M)[7] | | Mascot |

25 coiled-coil domain-containing protein 123, mitochondrial precursor [Danio rerio] gi|41053710 79256.9 5.96 11 34 0

**Peptide Information**

| Calc. Mass | Obsrv. Mass | ± da | ± ppm | Start Seq. | End Seq. | Sequence | Ion Score | C. I. % | Modification | Rank | Result Type |
|---|---|---|---|---|---|---|---|---|---|---|---|
| 1118.5082 | 1118.5657 | 0.0575 | 51 | 304 | 312 | CLQQSQHMK | | | Oxidation (M)[8] | | Mascot |
| 1216.6143 | 1216.5925 | -0.0218 | -18 | 1 | 10 | MSSKFNFSLR | | | | | Mascot |
| 1279.7269 | 1279.7557 | 0.0288 | 23 | 366 | 375 | YQARFRPLTK | | | | | Mascot |
| 1475.7522 | 1475.7318 | -0.0204 | -14 | 564 | 575 | VKTCEVEDLQVR | | | Carbamidomethyl (C)[4] | | Mascot |
| 1533.8192 | 1533.8055 | -0.0137 | -9 | 503 | 515 | VMLLEAEKQSLEK | | | Oxidation (M)[2] | | Mascot |
| 1577.6901 | 1577.8296 | 0.1395 | 88 | 226 | 239 | SMASPELDDWSSPR | | | | | Mascot |
| 1638.8077 | 1638.8279 | 0.0202 | 12 | 654 | 667 | DQLMLMASTLEKDK | | | Oxidation (M)[4] | | Mascot |
| 2211.0823 | 2211.0718 | -0.0105 | -5 | 340 | 358 | QQAQELVDENDGLKMTV HR | | | | | Mascot |
| 2233.2188 | 2233.0535 | -0.1653 | -74 | 536 | 555 | LALEHSLSLAEHQTVTDK LK | | | | | Mascot |
| 2247.2092 | 2247.0632 | -0.146 | -65 | 534 | 553 | VRLALEHSLSLAEHQTVT DK | | | | | Mascot |
| 2501.1707 | 2501.21 | 0.0393 | 16 | 304 | 323 | CLQQSQHMKHLQQELCH THR | | | Oxidation (M)[8] | | Mascot |

26 novel protein [Danio rerio] gi|220679558 45035 4.99 8 33 0

**Peptide Information**

| Calc. Mass | Obsrv. Mass | ± da | ± ppm | Start Seq. | End Seq. | Sequence | Ion Score | C. I. % | Modification | Rank | Result Type |
|---|---|---|---|---|---|---|---|---|---|---|---|
| 842.473 | 842.4988 | 0.0258 | 31 | 382 | 388 | DPKDVLR | | | | | Mascot |

| | | | | | | | | | | | |
|---|---|---|---|---|---|---|---|---|---|---|---|
| 882.4791 | 882.5546 | 0.0755 | 86 | 1 | 7 | VSTYTRR | | | | | Mascot |
| 1045.5889 | 1045.5497 | -0.0392 | -37 | 54 | 62 | VIDTLESLR | | | | | Mascot |
| 1084.6401 | 1084.543 | -0.0971 | -90 | 343 | 351 | VSLPWLLEK | | | | | Mascot |
| 1084.6401 | 1084.543 | -0.0971 | -90 | 343 | 351 | VSLPWLLEK | | | | | Mascot |
| 1184.501 | 1184.4415 | -0.0595 | -50 | 8 | 16 | CPGCQMIYR | | | Carbamidomethyl (C)[1,4] | | Mascot |
| 1772.7578 | 1772.9076 | 0.1498 | 85 | 220 | 235 | DCNVDSNGSAFDMILR | | | Oxidation (M)[13] | | Mascot |
| 2023.9971 | 2023.8156 | -0.1815 | -90 | 108 | 126 | NGVFSTAASDLKEPPSEFK | | | | | Mascot |
| 2023.9971 | 2023.8156 | -0.1815 | -90 | 108 | 126 | NGVFSTAASDLKEPPSEFK | | | | | Mascot |
| 2247.2205 | 2247.0632 | -0.1573 | -70 | 43 | 62 | HSLQSHVGLSRVIDTLESLR | | | | | Mascot |

27 si:ch1073-126c3.2 [Danio rerio] gi|221307488 24754.2 6.97 6 33 0

Peptide Information

| Calc. Mass | Obsrv. Mass | ± da | ± ppm | Start Seq. | End Seq. | Sequence | Ion Score | C. I. % | Modification | Rank | Result Type |
|---|---|---|---|---|---|---|---|---|---|---|---|
| 864.4243 | 864.5068 | 0.0825 | 95 | 1 | 8 | MESATIGR | | | | | Mascot |
| 1638.8069 | 1638.8279 | 0.021 | 13 | 199 | 213 | SELAKVETTESESTK | | | | | Mascot |
| 2054.9285 | 2054.8594 | -0.0691 | -34 | 114 | 130 | IYCKPMCNAGYDFNFLR | | | | | Mascot |
| 2184.9663 | 2185.105 | 0.1387 | 63 | 114 | 130 | IYCKPMCNAGYDFNFLR | | | Carbamidomethyl (C)[3,7], Oxidation (M)[6] | | Mascot |
| 2184.9663 | 2185.105 | 0.1387 | 63 | 114 | 130 | IYCKPMCNAGYDFNFLR | | | Carbamidomethyl (C)[3,7], Oxidation (M)[6] | | Mascot |
| 2211.0295 | 2211.0718 | 0.0423 | 19 | 114 | 131 | IYCKPMCNAGYDFNFLRR | | | | | Mascot |
| 2284.0459 | 2284.1384 | 0.0925 | 40 | 114 | 131 | IYCKPMCNAGYDFNFLRR | | | Carbamidomethyl (C)[3], Oxidation (M)[6] | | Mascot |
| 2298.0503 | 2298.1475 | 0.0972 | 42 | 112 | 130 | DKIYCKPMCNAGYDFNFLR | | | | | Mascot |
| 2808.4785 | 2808.3599 | -0.1186 | -42 | 85 | 111 | AFQKLLPENCPAPAVPENGGLLCLSVK | | | | | Mascot |

28 hypothetical protein LOC100191015 [Danio rerio] gi|209863095 44412.9 9.4 8 33 0

Peptide Information

| Calc. Mass | Obsrv. Mass | ± da | ± ppm | Start Seq. | End Seq. | Sequence | Ion Score | C. I. % | Modification | Rank | Result Type |
|---|---|---|---|---|---|---|---|---|---|---|---|
| 996.5373 | 996.5818 | 0.0445 | 45 | 282 | 288 | QRIFNYR | | | | | Mascot |
| 996.5373 | 996.5818 | 0.0445 | 45 | 282 | 288 | QRIFNYR | | | | | Mascot |
| 1475.6802 | 1475.7318 | 0.0516 | 35 | 176 | 188 | APDNSGSLFYNYK | | | | | Mascot |
| 1493.8335 | 1493.7094 | -0.1241 | -83 | 22 | 33 | RSVWVHEILQAR | | | | | Mascot |
| 1752.9075 | 1752.8513 | -0.0562 | -32 | 267 | 281 | RDLMRPFPGHNLSGR | | | | | Mascot |
| 1993.9834 | 1993.9467 | -0.0367 | -18 | 309 | 326 | MYRGAIEISPANVDACVK | | | Carbamidomethyl (C)[16] | | Mascot |
| 2055.0183 | 2054.8594 | -0.1589 | -77 | 1 | 17 | MEDQVVACCVALLYLRR | | | Carbamidomethyl (C)[8], Oxidation (M)[1] | | Mascot |
| 2211.1743 | 2211.0718 | -0.1025 | -46 | 294 | 311 | LIVENTFGILTAQWRMYR | | | | | Mascot |

| | | | | | | | | | | | | |
|---|---|---|---|---|---|---|---|---|---|---|---|---|
| 2514.2795 | 2514.1936 | -0.0859 | -34 | 337 | 361 | STTSTRIPLPSAADGDAA GLQEVTR | | | | | Mascot |

29 PREDICTED: zinc finger protein 567-like [Danio rerio] gi|189517588 45312.1 9.54 11 33 0

Peptide Information

| Calc. Mass | Obsrv. Mass | ± da | ± ppm | Start Seq. | End Seq. | Sequence | Ion Score | C. I. % | Modification | Rank | Result Type |
|---|---|---|---|---|---|---|---|---|---|---|---|
| 1051.6119 | 1051.6962 | 0.0843 | 80 | 66 | 74 | KTSLHGRPR | | | | | Mascot |
| 1051.6119 | 1051.6962 | 0.0843 | 80 | 67 | 75 | TSLHGRPRK | | | | | Mascot |
| 1441.6879 | 1441.679 | -0.0089 | -6 | 1 | 12 | MAFIKEESEDVK | | | Oxidation (M)[1] | | Mascot |
| 1572.7952 | 1572.7548 | -0.0404 | -26 | 146 | 158 | SFSQKPNLDIHMR | | | | | Mascot |
| 1577.7198 | 1577.8296 | 0.1098 | 70 | 299 | 313 | THTGEKPFACAQCGK | | | | | Mascot |
| 1719.8304 | 1719.842 | 0.0116 | 7 | 131 | 145 | IHTGEKPFSCKQCGK | | | Carbamidomethyl (C)[10] | | Mascot |
| 2180.0586 | 2179.9604 | -0.0982 | -45 | 327 | 345 | VHTGTIVFTCDQCGKSLT R | | | Carbamidomethyl (C)[10,13] | | Mascot |
| 2185.0164 | 2185.105 | 0.0886 | 41 | 299 | 318 | THTGEKPFACAQCGKSF STK | | | Carbamidomethyl (C)[10] | | Mascot |
| 2185.0164 | 2185.105 | 0.0886 | 41 | 299 | 318 | THTGEKPFACAQCGKSF STK | | | Carbamidomethyl (C)[10] | | Mascot |
| 2233.031 | 2233.0535 | 0.0225 | 10 | 323 | 341 | NHMKVHTGTIVFTCDQC GK | | | Carbamidomethyl (C)[14,17] | | Mascot |
| 2300.0435 | 2300.1436 | 0.1001 | 44 | 159 | 178 | VHTGEKPYSCEQCGKSF SQK | | | Carbamidomethyl (C)[10] | | Mascot |
| 3337.5146 | 3337.7178 | 0.2032 | 61 | 95 | 122 | SNLDVHMRVHTGEKPYT CEQCAQSFSQK | | | Carbamidomethyl (C)[18,21] | | Mascot |

30 Zgc:101621 protein [Danio rerio] gi|160773250 15314.9 9.35 5 33 0

Peptide Information

| Calc. Mass | Obsrv. Mass | ± da | ± ppm | Start Seq. | End Seq. | Sequence | Ion Score | C. I. % | Modification | Rank | Result Type |
|---|---|---|---|---|---|---|---|---|---|---|---|
| 1320.6793 | 1320.6135 | -0.0658 | -50 | 39 | 49 | LQEDGEQFVKK | | | | | Mascot |
| 1577.8533 | 1577.8296 | -0.0237 | -15 | 12 | 27 | VQAVLTSASAGLEGFK | | | | | Mascot |
| 2040.9287 | 2040.8425 | -0.0862 | -42 | 88 | 106 | ADCTIAMADSDLLDLMTG K | | | Carbamidomethyl (C)[3] | | Mascot |
| 2040.9287 | 2040.8425 | -0.0862 | -42 | 88 | 106 | ADCTIAMADSDLLDLMTG K | | | Carbamidomethyl (C)[3] | | Mascot |
| 2185.0186 | 2185.105 | 0.0864 | 40 | 87 | 106 | KADCTIAMADSDLLDLMT GK | | | Carbamidomethyl (C)[4], Oxidation (M)[8] | | Mascot |
| 2185.0186 | 2185.105 | 0.0864 | 40 | 87 | 106 | KADCTIAMADSDLLDLMT GK | | | Carbamidomethyl (C)[4], Oxidation (M)[8] | | Mascot |
| 3338.5046 | 3338.7083 | 0.2037 | 61 | 88 | 117 | ADCTIAMADSDLLDLMTG KMNPQTAFFQGK | | | Carbamidomethyl (C)[3], Oxidation (M)[7,16,20] | | Mascot |

31 PREDICTED: outer dense fiber protein 2 [Danio rerio] gi|326677656 95646.7 6.54 13 33 0

Peptide Information

| Calc. Mass | Obsrv. Mass | ± da | ± ppm | Start Seq. | End Seq. | Sequence | Ion Score | C. I. % | Modification | Rank | Result Type |
|---|---|---|---|---|---|---|---|---|---|---|---|

| | | | | | | | | | | | |
|---|---|---|---|---|---|---|---|---|---|---|---|
| 850.4338 | 850.5313 | 0.0975 | 115 | 714 | 720 | ECSILTK | | | Carbamidomethyl (C)[2] | | Mascot |
| 1051.6259 | 1051.6962 | 0.0703 | 67 | 46 | 55 | TGLHPTAKVK | | | | | Mascot |
| 1051.6259 | 1051.6962 | 0.0703 | 67 | 46 | 55 | TGLHPTAKVK | | | | | Mascot |
| 1062.5612 | 1062.6775 | 0.1163 | 109 | 34 | 43 | CSPTKTAQVK | | | | | Mascot |
| 1118.551 | 1118.5657 | 0.0147 | 13 | 659 | 667 | NMLLQEENK | | | | | Mascot |
| 1165.5881 | 1165.7288 | 0.1407 | 121 | 247 | 257 | MSGSDSTLLVR | | | | | Mascot |
| 1165.5881 | 1165.7288 | 0.1407 | 121 | 247 | 257 | MSGSDSTLLVR | | | | | Mascot |
| 1320.6139 | 1320.6135 | -0.0004 | 0 | 788 | 798 | DRLEQSDSTNR | | | | | Mascot |
| 1441.8009 | 1441.679 | -0.1219 | -85 | 463 | 475 | GQLEVEIALLNSR | | | | | Mascot |
| 1463.741 | 1463.6587 | -0.0823 | -56 | 213 | 226 | KLLEAEMDSSAAAK | | | | | Mascot |
| 1485.6849 | 1485.6229 | -0.062 | -42 | 233 | 245 | ETVSQMSRSTSEK | | | Oxidation (M)[6] | | Mascot |
| 1573.8367 | 1573.7151 | -0.1216 | -77 | 643 | 656 | IKAEQQGSLAEMLR | | | | | Mascot |
| 1573.8367 | 1573.7151 | -0.1216 | -77 | 643 | 656 | IKAEQQGSLAEMLR | 2 | 0 | | | Mascot |
| 1591.822 | 1591.7767 | -0.0453 | -28 | 227 | 240 | QVSALRETVSQMSR | | | | | Mascot |
| 1752.9061 | 1752.8513 | -0.0548 | -31 | 659 | 672 | NMLLQEENKQLQHK | | | | | Mascot |
| 3459.7236 | 3459.7966 | 0.073 | 21 | 423 | 454 | SEDTVGQLSGQLLEIEKQ LAEAVTAAENWSSR | | | | | Mascot |

32 olfactomedin 1b [Danio rerio] gi|51011063 14508.5 8.86 6 32 0

**Peptide Information**

| Calc. Mass | Obsrv. Mass | ± da | ± ppm | Start Seq. | End Seq. | Sequence | Ion Score | C. I. % | Modification | Rank | Result Type |
|---|---|---|---|---|---|---|---|---|---|---|---|
| 850.4781 | 850.5313 | 0.0532 | 63 | 103 | 109 | GLETKFR | | | | | Mascot |
| 864.4825 | 864.5068 | 0.0243 | 28 | 90 | 96 | DLQYVVK | | | | | Mascot |
| 1652.8312 | 1652.8027 | -0.0285 | -17 | 90 | 102 | DLQYVVKMEDQLR | | | Oxidation (M)[8] | | Mascot |
| 1675.8431 | 1675.7169 | -0.1262 | -75 | 73 | 86 | VQNMTQSIQVLDQR | | | Oxidation (M)[4] | | Mascot |
| 2040.9082 | 2040.8425 | -0.0657 | -32 | 45 | 62 | CVCTVVAPQQTMCSRDA R | | | Carbamidomethyl (C)[1], Oxidation (M)[12] | | Mascot |
| 2040.9082 | 2040.8425 | -0.0657 | -32 | 45 | 62 | CVCTVVAPQQTMCSRDA R | | | Carbamidomethyl (C)[1], Oxidation (M)[12] | | Mascot |
| 2045.0557 | 2044.9039 | -0.1518 | -74 | 73 | 89 | VQNMTQSIQVLDQRTQR | | | | | Mascot |

33 Ift81 protein [Danio rerio] gi|145337899 29908.3 5.76 8 32 0

**Peptide Information**

| Calc. Mass | Obsrv. Mass | ± da | ± ppm | Start Seq. | End Seq. | Sequence | Ion Score | C. I. % | Modification | Rank | Result Type |
|---|---|---|---|---|---|---|---|---|---|---|---|
| 870.5519 | 870.531 | -0.0209 | -24 | 200 | 206 | VLRQQVK | | | | | Mascot |
| 1045.5636 | 1045.5497 | -0.0139 | -13 | 14 | 21 | TEEILRER | | | | | Mascot |

| | | | | | | | | | | | |
|---|---|---|---|---|---|---|---|---|---|---|---|
| 1184.5173 | 1184.4415 | -0.0758 | -64 | 65 | 74 | TLDDMSEMVK | | | Oxidation (M)[5] | | Mascot |
| 1420.6307 | 1420.5984 | -0.0323 | -23 | 210 | 220 | ENQEPNMRQMK | | | Oxidation (M)[7] | | Mascot |
| 1572.7177 | 1572.7548 | 0.0371 | 24 | 99 | 111 | QEHAELAPEYEEK | | | | | Mascot |
| 1912.9399 | 1912.9166 | -0.0233 | -12 | 96 | 111 | ALRQEHAELAPEYEEK | | | | | Mascot |
| 2225.0879 | 2225.085 | -0.0029 | -1 | 147 | 163 | YHHINCEREIIESQIQR | | | Carbamidomethyl (C)[6] | | Mascot |
| 2225.0879 | 2225.085 | -0.0029 | -1 | 147 | 163 | YHHINCEREIIESQIQR | | | Carbamidomethyl (C)[6] | | Mascot |
| 2283.1914 | 2283.144 | -0.0474 | -21 | 234 | 254 | QCYLKAQSQAPIGHVIQD VGK | | | | | Mascot |
| 2283.1914 | 2283.144 | -0.0474 | -21 | 234 | 254 | QCYLKAQSQAPIGHVIQD VGK | | | | | Mascot |

| | | | | | | | |
|---|---|---|---|---|---|---|---|
| 34 | novel protein similar to H.sapiens OLFM1, olfactomedin 1 (OLFM1, zgc:92039) [Danio rerio] | gi\|220676962 | 55200.4 | 8.33 | 9 | 32 | 0 |

**Peptide Information**

| Calc. Mass | Obsrv. Mass | ± da | ± ppm | Start Seq. | End Seq. | Sequence | Ion Score | C. I. % | Modification | Rank | Result Type |
|---|---|---|---|---|---|---|---|---|---|---|---|
| 850.4781 | 850.5313 | 0.0532 | 63 | 131 | 137 | GLETKFR | | | | | Mascot |
| 864.4825 | 864.5068 | 0.0243 | 28 | 118 | 124 | DLQYVVK | | | | | Mascot |
| 996.5182 | 996.5818 | 0.0636 | 64 | 315 | 322 | FQSNMIIK | | | Oxidation (M)[5] | | Mascot |
| 996.5182 | 996.5818 | 0.0636 | 64 | 315 | 322 | FQSNMIIK | | | Oxidation (M)[5] | | Mascot |
| 1533.777 | 1533.8055 | 0.0285 | 19 | 315 | 326 | FQSNMIIKFDFK | | | Oxidation (M)[5] | | Mascot |
| 1652.8312 | 1652.8027 | -0.0285 | -17 | 118 | 130 | DLQYVVKMEDQLR | | | Oxidation (M)[8] | | Mascot |
| 1675.8431 | 1675.7169 | -0.1262 | -75 | 101 | 114 | VQNMTQSIQVLDQR | | | Oxidation (M)[4] | | Mascot |
| 2040.9082 | 2040.8425 | -0.0657 | -32 | 73 | 90 | CVCTVVAPQQTMCSRDA R | | | Carbamidomethyl (C)[1], Oxidation (M)[12] | | Mascot |
| 2040.9082 | 2040.8425 | -0.0657 | -32 | 73 | 90 | CVCTVVAPQQTMCSRDA R | | | Carbamidomethyl (C)[1], Oxidation (M)[12] | | Mascot |
| 2045.0557 | 2044.9039 | -0.1518 | -74 | 101 | 117 | VQNMTQSIQVLDQRTQR | | | | | Mascot |
| 2284.0298 | 2284.1384 | 0.1086 | 48 | 241 | 261 | TSGSRFGSWMTDPLAPE GDTR | | | Oxidation (M)[10] | | Mascot |

| | | | | | | | |
|---|---|---|---|---|---|---|---|
| 35 | LOC100002738 protein [Danio rerio] | gi\|134026300 | 92988.4 | 5.6 | 11 | 31 | 0 |

**Peptide Information**

| Calc. Mass | Obsrv. Mass | ± da | ± ppm | Start Seq. | End Seq. | Sequence | Ion Score | C. I. % | Modification | Rank | Result Type |
|---|---|---|---|---|---|---|---|---|---|---|---|
| 842.4842 | 842.4988 | 0.0146 | 17 | 602 | 608 | IVDPSRR | | | | | Mascot |
| 1184.5265 | 1184.4415 | -0.085 | -72 | 614 | 622 | CHYEPHSVR | | | Carbamidomethyl (C)[1] | | Mascot |
| 1560.9108 | 1560.8088 | -0.102 | -65 | 634 | 647 | ETLIGVHIPTKQPK | | | | | Mascot |
| 1577.7642 | 1577.8296 | 0.0654 | 41 | 609 | 622 | APPGKCHYEPHSVR | | | | | Mascot |
| 2185.0481 | 2185.105 | 0.0569 | 26 | 445 | 463 | IFCGDVGQNKYEEVDIVE K | | | | | Mascot |
| 2185.0481 | 2185.105 | 0.0569 | 26 | 445 | 463 | IFCGDVGQNKYEEVDIVE K | | | | | Mascot |

| | | | | | | | | | | | |
|---|---|---|---|---|---|---|---|---|---|---|---|
| 2225.1641 | 2225.085 | -0.0791 | -36 | 239 | 257 | FFVAEQVGLVWVYLPDQSK | | | | | Mascot |
| 2225.1641 | 2225.085 | -0.0791 | -36 | 239 | 257 | FFVAEQVGLVWVYLPDQSK | | | | | Mascot |
| 2233.259 | 2233.0535 | -0.2055 | -92 | 625 | 644 | SNIIPFEPKETLIGVHIPTK | | | | | Mascot |
| 2247.1477 | 2247.0632 | -0.0845 | -38 | 481 | 500 | KLCANSSLDDVLPIYAYPHK | | | | | Mascot |
| 2306.075 | 2306.1121 | 0.0371 | 16 | 204 | 224 | TAADSEGCLQLCLEEVANGLR | | | Carbamidomethyl (C)[8,12] | | Mascot |
| 2808.3152 | 2808.3599 | 0.0447 | 16 | 513 | 537 | GCENPNLNGLYIFGDFMSGRLMSLK | | | Oxidation (M)[17,22] | | Mascot |
| 3338.5549 | 3338.7083 | 0.1534 | 46 | 1 | 28 | LHNFYFHVSEVGSQALSVMWYFCDVFGR | | | | | Mascot |

36 PREDICTED: obscurin [Danio rerio] gi|326669989 741504.6 6.35 39 31 0

**Peptide Information**

| Calc. Mass | Obsrv. Mass | ± da | ± ppm | Start Seq. | End Seq. | Sequence | Ion Score | C. I. % | Modification | Rank | Result Type |
|---|---|---|---|---|---|---|---|---|---|---|---|
| 842.473 | 842.4988 | 0.0258 | 31 | 5613 | 5620 | DVAITAPR | | | | | Mascot |
| 864.4573 | 864.5068 | 0.0495 | 57 | 5295 | 5301 | EVEFGKR | | | | | Mascot |
| 870.577 | 870.531 | -0.046 | -53 | 1721 | 1727 | LELVIRK | | | | | Mascot |
| 1045.5248 | 1045.5497 | 0.0249 | 24 | 1960 | 1968 | AGCPVEWRK | | | | | Mascot |
| 1051.5895 | 1051.6962 | 0.1067 | 101 | 3572 | 3581 | SKAVPEAPPR | | | | | Mascot |
| 1051.5895 | 1051.6962 | 0.1067 | 101 | 3574 | 3583 | AVPEAPPRSK | | | | | Mascot |
| 1062.5248 | 1062.6775 | 0.1527 | 144 | 1791 | 1800 | GAEMLLAGER | | | Oxidation (M)[4] | | Mascot |
| 1084.5382 | 1084.543 | 0.0048 | 4 | 4601 | 4609 | DEHSLIQSR | | | | | Mascot |
| 1084.5997 | 1084.543 | -0.0567 | -52 | 2327 | 2336 | NNVTLLPGEK | | | | | Mascot |
| 1118.5874 | 1118.5657 | -0.0217 | -19 | 791 | 800 | GIVVLECETR | | | | | Mascot |
| 1149.5721 | 1149.5762 | 0.0041 | 4 | 6343 | 6351 | MTPYKPEQR | | | | | Mascot |
| 1165.567 | 1165.7288 | 0.1618 | 139 | 6343 | 6351 | MTPYKPEQR | | | Oxidation (M)[1] | | Mascot |
| 1165.567 | 1165.7288 | 0.1618 | 139 | 6343 | 6351 | MTPYKPEQR | | | Oxidation (M)[1] | | Mascot |
| 1316.7936 | 1316.6779 | -0.1157 | -88 | 1391 | 1403 | ATIKIVGAPATFK | | | | | Mascot |
| 1344.7668 | 1344.6853 | -0.0815 | -61 | 3951 | 3962 | TLTTIPVRGMQK | | | | | Mascot |
| 1441.7621 | 1441.679 | -0.0831 | -58 | 5364 | 5375 | LACLLDFFSTRR | | | | | Mascot |
| 1446.7709 | 1446.574 | -0.1969 | -136 | 2779 | 2790 | GMMLLRPGHKYK | | | Oxidation (M)[2] | | Mascot |
| 1463.692 | 1463.6587 | -0.0333 | -23 | 4306 | 4318 | RHTITHNPDGGCR | | | | | Mascot |
| 1475.7021 | 1475.7318 | 0.0297 | 20 | 4659 | 4671 | DIASFHECCILPK | | | | | Mascot |
| 1485.7076 | 1485.6229 | -0.0847 | -57 | 3539 | 3551 | ICDLKCQDSGIYK | | | | | Mascot |
| 1549.7891 | 1549.8024 | 0.0133 | 9 | 82 | 96 | AEGIETSAMLTVTAR | | | | | Mascot |
| 1560.7687 | 1560.8088 | 0.0401 | 26 | 3586 | 3599 | NIMDEALSGQVQQK | | | | | Mascot |
| 1573.8068 | 1573.7151 | -0.0917 | -58 | 4376 | 4389 | TVVPTVNTEEESLR | | | | | Mascot |
| 1573.8068 | 1573.7151 | -0.0917 | -58 | 4376 | 4389 | TVVPTVNTEEESLR | | | | | Mascot |

| | | | | | | | | | | | |
|---|---|---|---|---|---|---|---|---|---|---|---|
| 1652.7948 | 1652.8027 | 0.0079 | 5 | 1702 | 1715 | GEEVLTPGEKYQMR | | | Oxidation (M)[13] | | Mascot |
| 1675.8398 | 1675.7169 | -0.1229 | -73 | 4575 | 4588 | TREIFSLAHESETR | | | | | Mascot |
| 1719.7101 | 1719.842 | 0.1319 | 77 | 5445 | 5458 | ICDFGFCQEVDSSR | | | Carbamidomethyl (C)[2,7] | | Mascot |
| 1772.9153 | 1772.9076 | -0.0077 | -4 | 274 | 288 | WTLNGQILFPCPDIR | | | | | Mascot |
| 1993.9899 | 1993.9467 | -0.0432 | -22 | 784 | 800 | DVFAEEKGIVVLECETR | | | Carbamidomethyl (C)[14] | | Mascot |
| 2024.0117 | 2023.8156 | -0.1961 | -97 | 1545 | 1563 | LAINNLVEGDAGNYTCKT K | | | | | Mascot |
| 2024.0117 | 2023.8156 | -0.1961 | -97 | 1545 | 1563 | LAINNLVEGDAGNYTCKT K | | | | | Mascot |
| 2040.9219 | 2040.8425 | -0.0794 | -39 | 113 | 129 | ESLTFICEVNWEDVDGK | | | Carbamidomethyl (C)[7] | | Mascot |
| 2040.9219 | 2040.8425 | -0.0794 | -39 | 113 | 129 | ESLTFICEVNWEDVDGK | | | Carbamidomethyl (C)[7] | | Mascot |
| 2045.0549 | 2044.9039 | -0.151 | -74 | 543 | 560 | DGEILNSTEKYEILHVGK | | | | | Mascot |
| 2181.0757 | 2180.9521 | -0.1236 | -57 | 5033 | 5051 | GQDVQFKCSTQSVPFPSI R | | | Carbamidomethyl (C)[8] | | Mascot |
| 2184.9424 | 2185.105 | 0.1626 | 74 | 1193 | 1212 | NPVPEDSGTYICVCEDQ STK | | | | | Mascot |
| 2184.9424 | 2185.105 | 0.1626 | 74 | 1193 | 1212 | NPVPEDSGTYICVCEDQ STK | | | | | Mascot |
| 2210.9956 | 2211.0718 | 0.0762 | 34 | 4509 | 4527 | ETFDIYVAKTDCHPMGG NR | | | Carbamidomethyl (C)[12] | | Mascot |
| 2247.2166 | 2247.0632 | -0.1534 | -68 | 62 | 81 | NHTLTLTRVTLADMGIISF K | | | Oxidation (M)[14] | | Mascot |
| 2284.0979 | 2284.1384 | 0.0405 | 18 | 3166 | 3184 | EKLLFFSEELQNQESEEG K | | | | | Mascot |
| 2306.104 | 2306.1121 | 0.0081 | 4 | 1586 | 1607 | SQEVQEGNSVTLNCELS KAGGR | | | | | Mascot |
| 2501.2085 | 2501.21 | 0.0015 | 1 | 2793 | 2814 | QDGCKMQLLILELSCQDS GIYK | | | Oxidation (M)[6] | | Mascot |
| 2808.3225 | 2808.3599 | 0.0374 | 13 | 1095 | 1118 | KSCISLAIHNVKPDDSGN YTCICR | | | Carbamidomethyl (C)[3,21,23] | | Mascot |
| 3338.4536 | 3338.7083 | 0.2547 | 76 | 5965 | 5995 | SHHDAFVIAGSSGKTSAC SLLQDSEDEDMER | | | Oxidation (M)[29] | | Mascot |
| 3459.521 | 3459.7966 | 0.2756 | 80 | 2430 | 2460 | QDGCELQLQINDLTSQD SGAYRCCADGIETR | | | Carbamidomethyl (C)[4] | | Mascot |

| | | | | | | | | |
|---|---|---|---|---|---|---|---|---|
| 37 | PREDICTED: probable polyketide synthase 1 [Danio rerio] | gi\|189536864 | 232039.4 | 6.77 | 19 | 31 | 0 | |

**Peptide Information**

| Calc. Mass | Obsrv. Mass | ± da | ± ppm | Start Seq. | End Seq. | Sequence | Ion Score | C. I. % | Modification | Rank | Result Type |
|---|---|---|---|---|---|---|---|---|---|---|---|
| 1045.571 | 1045.5497 | -0.0213 | -20 | 237 | 246 | GEGCGIVLLK | | | Carbamidomethyl (C)[4] | | Mascot |
| 1051.5895 | 1051.6962 | 0.1067 | 101 | 1148 | 1156 | DGHVLIELR | | | | | Mascot |
| 1051.5895 | 1051.6962 | 0.1067 | 101 | 1148 | 1156 | DGHVLIELR | | | | | Mascot |
| 1149.635 | 1149.5762 | -0.0588 | -51 | 1666 | 1674 | WLKGMHLHK | | | | | Mascot |
| 1165.6299 | 1165.7288 | 0.0989 | 85 | 1666 | 1674 | WLKGMHLHK | | | Oxidation (M)[5] | | Mascot |
| 1165.6299 | 1165.7288 | 0.0989 | 85 | 1666 | 1674 | WLKGMHLHK | | | Oxidation (M)[5] | | Mascot |
| 1216.6685 | 1216.5925 | -0.076 | -62 | 777 | 786 | KPVEFEQAIR | | | | | Mascot |

| | | | | | | | | | | | |
|---|---|---|---|---|---|---|---|---|---|---|---|
| 1316.7606 | 1316.6779 | -0.0827 | -63 | 237 | 249 | GEGCGIVLLKSLK | | | | | Mascot |
| 1420.7617 | 1420.5984 | -0.1633 | -115 | 1922 | 1933 | LGLLMNKDNFQK | | | | | Mascot |
| 1441.6495 | 1441.679 | 0.0295 | 20 | 762 | 773 | QGDFVNGEYWAR | | | | | Mascot |
| 1446.6868 | 1446.574 | -0.1128 | -78 | 1486 | 1497 | VGDHVICCYPVK | | | Carbamidomethyl (C)[7,8] | | Mascot |
| 1533.7689 | 1533.8055 | 0.0366 | 24 | 1763 | 1778 | GGGCIATLSRSAPSEK | | | | | Mascot |
| 1573.8229 | 1573.7151 | -0.1078 | -68 | 1484 | 1497 | LKVGDHVICCYPVK | | | | | Mascot |
| 1573.8229 | 1573.7151 | -0.1078 | -68 | 1484 | 1497 | LKVGDHVICCYPVK | | | | | Mascot |
| 1577.9149 | 1577.8296 | -0.0853 | -54 | 496 | 510 | VFAASSLSELISLLK | | | | | Mascot |
| 1591.8816 | 1591.7767 | -0.1049 | -66 | 1157 | 1170 | DVRITFLGAHGHIR | | | | | Mascot |
| 1719.8445 | 1719.842 | -0.0025 | -1 | 1640 | 1653 | TCFQTLEMSKIFQK | | | Oxidation (M)[8] | | Mascot |
| 1752.801 | 1752.8513 | 0.0503 | 29 | 907 | 921 | CNLSASTNSYLWEHK | | | | | Mascot |
| 1772.6964 | 1772.9076 | 0.2112 | 119 | 1133 | 1147 | QMSDYFEVCGCFTDK | | | | | Mascot |
| 2023.955 | 2023.8156 | -0.1394 | -69 | 526 | 542 | LVFVFCGNGVTYRGMCK | | | Carbamidomethyl (C)[6,16], Oxidation (M)[15] | | Mascot |
| 2023.955 | 2023.8156 | -0.1394 | -69 | 526 | 542 | LVFVFCGNGVTYRGMCK | | | Carbamidomethyl (C)[6,16], Oxidation (M)[15] | | Mascot |
| 2180.9724 | 2180.9521 | -0.0203 | -9 | 1998 | 2017 | NKDAEDSGISVQSSVDD CVR | | | Carbamidomethyl (C)[18] | | Mascot |
| 2298.1797 | 2298.1475 | -0.0322 | -14 | 829 | 848 | DQQTMFNTVSKLFELGV NVK | | | | | Mascot |
| 3338.7415 | 3338.7083 | -0.0332 | -10 | 2054 | 2082 | FFQEMGVNIPLVALLDPN CTLLSLTEYLK | | | Carbamidomethyl (C)[19] | | Mascot |

38 tetratricopeptide repeat protein 5 [Danio rerio] gi|62955285 49632.1 6.49 9 31 0

**Peptide Information**

| Calc. Mass | Obsrv. Mass | ± da | ± ppm | Start Seq. | End Seq. | Sequence | Ion Score | C. I. % | Modification | Rank | Result Type |
|---|---|---|---|---|---|---|---|---|---|---|---|
| 864.4798 | 864.5068 | 0.027 | 31 | 399 | 405 | RHSVTHK | | | | | Mascot |
| 1084.6262 | 1084.543 | -0.0832 | -77 | 71 | 79 | HSAQFLLLR | | | | | Mascot |
| 1084.6262 | 1084.543 | -0.0832 | -77 | 71 | 79 | HSAQFLLLR | | | | | Mascot |
| 1149.5205 | 1149.5762 | 0.0557 | 48 | 48 | 57 | QNDVAQEMAK | | | Oxidation (M)[8] | | Mascot |
| 1475.7522 | 1475.7318 | -0.0204 | -14 | 48 | 60 | QNDVAQEMAKTLK | | | | | Mascot |
| 1549.6976 | 1549.8024 | 0.1048 | 68 | 2 | 15 | AEVDNDGEQKTTDK | | | | | Mascot |
| 2023.9211 | 2023.8156 | -0.1055 | -52 | 82 | 99 | CLNVAPEFSQAAEECLS R | | | Carbamidomethyl (C)[1] | | Mascot |
| 2023.9211 | 2023.8156 | -0.1055 | -52 | 82 | 99 | CLNVAPEFSQAAEECLS R | | | Carbamidomethyl (C)[1] | | Mascot |
| 2029.0059 | 2028.8933 | -0.1126 | -55 | 429 | 446 | QVMKSQSAAFVTYKPQS E | | | | | Mascot |
| 2045.0007 | 2044.9039 | -0.0968 | -47 | 429 | 446 | QVMKSQSAAFVTYKPQS E | | | Oxidation (M)[3] | | Mascot |
| 2180.0222 | 2179.9604 | -0.0618 | -28 | 80 | 99 | GRCLNVAPEFSQAAEEC LSR | | | | | Mascot |
| 2247.1443 | 2247.0632 | -0.0811 | -36 | 103 | 121 | LEPGLVEGWNTLGEQYW KK | | | | | Mascot |

39 novel protein similar to H.sapiens OLFM1, olfactomedin gi|220676964 52461.1 8.07 9 31 0

1 (OLFM1, zgc:92039) [Danio rerio]

Peptide Information

| Calc. Mass | Obsrv. Mass | ± da | ± ppm | Start Seq. | End Seq. | Sequence | Ion Score | C. I. % | Modification | Rank | Result Type |
|---|---|---|---|---|---|---|---|---|---|---|---|
| 850.4781 | 850.5313 | 0.0532 | 63 | 103 | 109 | GLETKFR | | | | | Mascot |
| 864.4825 | 864.5068 | 0.0243 | 28 | 90 | 96 | DLQYVVK | | | | | Mascot |
| 996.5182 | 996.5818 | 0.0636 | 64 | 287 | 294 | FQSNMIIK | | | Oxidation (M)[5] | | Mascot |
| 996.5182 | 996.5818 | 0.0636 | 64 | 287 | 294 | FQSNMIIK | | | Oxidation (M)[5] | | Mascot |
| 1533.777 | 1533.8055 | 0.0285 | 19 | 287 | 298 | FQSNMIIKFDFK | | | Oxidation (M)[5] | | Mascot |
| 1652.8312 | 1652.8027 | -0.0285 | -17 | 90 | 102 | DLQYVVKMEDQLR | | | Oxidation (M)[8] | | Mascot |
| 1675.8431 | 1675.7169 | -0.1262 | -75 | 73 | 86 | VQNMTQSIQVLDQR | | | Oxidation (M)[4] | | Mascot |
| 2040.9082 | 2040.8425 | -0.0657 | -32 | 45 | 62 | CVCTVVAPQQTMCSRDA R | | | Carbamidomethyl (C)[1], Oxidation (M)[12] | | Mascot |
| 2040.9082 | 2040.8425 | -0.0657 | -32 | 45 | 62 | CVCTVVAPQQTMCSRDA R | | | Carbamidomethyl (C)[1], Oxidation (M)[12] | | Mascot |
| 2045.0557 | 2044.9039 | -0.1518 | -74 | 73 | 89 | VQNMTQSIQVLDQRTQR | | | | | Mascot |
| 2284.0298 | 2284.1384 | 0.1086 | 48 | 213 | 233 | TSGSRFGSWMTDPLAPE GDTR | | | Oxidation (M)[10] | | Mascot |

| | | | | | | | |
|---|---|---|---|---|---|---|---|
| 40 | Pbx2 [Danio rerio] | gi\|7861936 | 18895.6 | 9.08 | 6 | 30 | 0 |

Peptide Information

| Calc. Mass | Obsrv. Mass | ± da | ± ppm | Start Seq. | End Seq. | Sequence | Ion Score | C. I. % | Modification | Rank | Result Type |
|---|---|---|---|---|---|---|---|---|---|---|---|
| 864.4686 | 864.5068 | 0.0382 | 44 | 153 | 159 | SRFLDAR | | | | | Mascot |
| 1051.5782 | 1051.6962 | 0.118 | 112 | 133 | 141 | FSSIQTQLK | | | | | Mascot |
| 1051.5782 | 1051.6962 | 0.118 | 112 | 133 | 141 | FSSIQTQLK | | | | | Mascot |
| 1493.7563 | 1493.7094 | -0.0469 | -31 | 142 | 154 | QSTCEAVMILRSR | | | | | Mascot |
| 1572.7937 | 1572.7548 | -0.0389 | -25 | 33 | 47 | LDNMLLAEGVAGPEK | | | Oxidation (M)[4] | | Mascot |
| 2054.927 | 2054.8594 | -0.0676 | -33 | 93 | 109 | YEQACSESTTHVMNLLR | | | Carbamidomethyl (C)[5], Oxidation (M)[13] | | Mascot |
| 2283.1836 | 2283.144 | -0.0396 | -17 | 133 | 152 | FSSIQTQLKQSTCEAVMI LR | | | | | Mascot |
| 2283.1836 | 2283.144 | -0.0396 | -17 | 133 | 152 | FSSIQTQLKQSTCEAVMI LR | | | | | Mascot |

| | | | | | | | |
|---|---|---|---|---|---|---|---|
| 41 | PREDICTED: zinc finger protein 850-like [Danio rerio] | gi\|326666761 | 93003 | 9.53 | 17 | 30 | 0 |

Peptide Information

| Calc. Mass | Obsrv. Mass | ± da | ± ppm | Start Seq. | End Seq. | Sequence | Ion Score | C. I. % | Modification | Rank | Result Type |
|---|---|---|---|---|---|---|---|---|---|---|---|
| 870.4719 | 870.531 | 0.0591 | 68 | 361 | 367 | LFTQFSK | | | | | Mascot |
| 1045.4441 | 1045.5497 | 0.1056 | 101 | 407 | 415 | TFTCTQCGK | | | Carbamidomethyl (C)[4] | | Mascot |

| Calc. Mass | Obsrv. Mass | ± da | ± ppm | Start Seq. | End Seq. | Sequence | Ion Score | C. I. % | Modification | Rank | Result Type |
|---|---|---|---|---|---|---|---|---|---|---|---|
| 1084.5898 | 1084.543 | -0.0468 | -43 | 429 | 437 | IHTGEKPFR | | | | | Mascot |
| 1084.5898 | 1084.543 | -0.0468 | -43 | 429 | 437 | IHTGEKPFR | | | | | Mascot |
| 1149.6626 | 1149.5762 | -0.0864 | -75 | 639 | 648 | TFITAAELKR | | | | | Mascot |
| 1184.5476 | 1184.4415 | -0.1061 | -90 | 73 | 83 | SACNFGSKQSR | | | | | Mascot |
| 1216.5562 | 1216.5925 | 0.0363 | 30 | 295 | 304 | KPHTCTQCGK | | | Carbamidomethyl (C)[5,8] | | Mascot |
| 1420.7478 | 1420.5984 | -0.1494 | -105 | 142 | 153 | ANLDVHMRVHTK | | | | | Mascot |
| 1493.7166 | 1493.7094 | -0.0072 | -5 | 500 | 512 | SFSQSSSLNLHMR | | | | | Mascot |
| 1591.7355 | 1591.7767 | 0.0412 | 26 | 686 | 698 | LFTCTQCGKSFSR | | | Carbamidomethyl (C)[4,7] | | Mascot |
| 1638.8203 | 1638.8279 | 0.0076 | 5 | 290 | 304 | IHTGKKPHTCTQCGK | | | | | Mascot |
| 1719.769 | 1719.842 | 0.073 | 42 | 262 | 276 | THTGDRSFICTHCGK | | | Carbamidomethyl (C)[10] | | Mascot |
| 1752.8632 | 1752.8513 | -0.0119 | -7 | 290 | 304 | IHTGKKPHTCTQCGK | | | Carbamidomethyl (C)[10,13] | | Mascot |
| 2055.0188 | 2054.8594 | -0.1594 | -78 | 310 | 326 | TSLDNHMRIHTGEKPYR | | | | | Mascot |
| 2184.9849 | 2185.105 | 0.1201 | 55 | 342 | 360 | HHMRTHTGEKPFACTQCGK | | | Oxidation (M)[3] | | Mascot |
| 2184.9912 | 2185.105 | 0.1138 | 52 | 745 | 763 | STQCGKSFSLSSNFNPHMR | | | Carbamidomethyl (C)[4] | | Mascot |
| 2283.0327 | 2283.144 | 0.1113 | 49 | 342 | 360 | HHMRTHTGEKPFACTQCGK | | | Carbamidomethyl (C)[14,17] | | Mascot |
| 2283.0327 | 2283.144 | 0.1113 | 49 | 342 | 360 | HHMRTHTGEKPFACTQCGK | | | Carbamidomethyl (C)[14,17] | | Mascot |
| 2808.2935 | 2808.3599 | 0.0664 | 24 | 699 | 722 | LSSLNLHMMIHTGEKPFTCSQCGK | | | Carbamidomethyl (C)[19,22], Oxidation (M)[8,9] | | Mascot |
| 3337.52 | 3337.7178 | 0.1978 | 59 | 193 | 220 | SFYHPGNFAVHMRIHTGERPYTCQQCGK | | | Carbamidomethyl (C)[23], Oxidation (M)[12] | | Mascot |
| 3338.4268 | 3338.7083 | 0.2815 | 84 | 444 | 471 | SFNCSSHLNEHMMIHTGEKPFTCTQCGK | | | Carbamidomethyl (C)[4,23,26] | | Mascot |

42 Ppp4r2a protein [Danio rerio] gi|156230689 49479.9 4.37 8 30 0

Peptide Information

| Calc. Mass | Obsrv. Mass | ± da | ± ppm | Start Seq. | End Seq. | Sequence | Ion Score | C. I. % | Modification | Rank | Result Type |
|---|---|---|---|---|---|---|---|---|---|---|---|
| 850.4199 | 850.5313 | 0.1114 | 131 | 172 | 179 | KGSSCVNR | | | | | Mascot |
| 1045.5598 | 1045.5497 | -0.0101 | -10 | 134 | 142 | LCELLTEPK | | | | | Mascot |
| 1184.6058 | 1184.4415 | -0.1643 | -139 | 144 | 153 | NYAGTDKFLR | | | | | Mascot |
| 1591.8591 | 1591.7767 | -0.0824 | -52 | 120 | 133 | IVNGYNGIPFTIQR | | | | | Mascot |
| 1772.7731 | 1772.9076 | 0.1345 | 76 | 180 | 195 | MNGVMFPGNTSAFPDR | | | Oxidation (M)[1,5] | | Mascot |
| 2211.075 | 2211.0718 | -0.0032 | -1 | 27 | 45 | HTSTMEIDTLLGAFRDFEK | | | | | Mascot |
| 2501.1118 | 2501.21 | 0.0982 | 39 | 173 | 195 | GSSCVNRMNGVMFPGNTSAFPDR | | | Carbamidomethyl (C)[4] | | Mascot |
| 3338.6633 | 3338.7083 | 0.045 | 13 | 180 | 210 | MNGVMFPGNTSAFPDRNVNGPGTPRPLNRPK | | | | | Mascot |

43 novel protein similar to H.sapiens C1orf57, chromosome 1 open reading frame 57 (C1orf57, zgc:92420) gi|220672935 20390.7 9.12 6 30 0

Peptide Information

| Calc. Mass | Obsrv. Mass | ± da | ± ppm | Start Seq. | End Seq. | Sequence | Ion Score | C. I. % | Modification | Rank | Result Type |
|---|---|---|---|---|---|---|---|---|---|---|---|
| 1165.5922 | 1165.7288 | 0.1366 | 117 | 103 | 112 | QLFVMDEVGK | | | | | Mascot |
| 1165.5922 | 1165.7288 | 0.1366 | 117 | 103 | 112 | QLFVMDEVGK | | | | | Mascot |
| 1463.7601 | 1463.6587 | -0.1014 | -69 | 45 | 58 | VGFDVVTVSGDRGR | | | | | Mascot |
| 1485.8246 | 1485.6229 | -0.2017 | -136 | 1 | 14 | MKHVFLTGVPGVGK | | | Oxidation (M)[1] | | Mascot |
| 1753.037 | 1752.8513 | -0.1857 | -106 | 3 | 19 | HVFLTGVPGVGKTTLVK | | | | | Mascot |
| 2028.9364 | 2028.8933 | -0.0431 | -21 | 95 | 112 | NMQEGSGKQLFVMDEV GK | | | Oxidation (M)[2,13] | | Mascot |
| 2247.0962 | 2247.0632 | -0.033 | -15 | 20 | 39 | KVCDTLSGLSVSGFYTEE VR | | | Carbamidomethyl (C)[3] | | Mascot |

| | | | | | | | |
|---|---|---|---|---|---|---|---|
| 44 | hypothetical protein LOC100145413 [Xenopus (Silurana) tropicalis] | gi\|187607099 | 39415.2 | 9.16 | 10 | 30 | 0 |

Peptide Information

| Calc. Mass | Obsrv. Mass | ± da | ± ppm | Start Seq. | End Seq. | Sequence | Ion Score | C. I. % | Modification | Rank | Result Type |
|---|---|---|---|---|---|---|---|---|---|---|---|
| 1118.6429 | 1118.5657 | -0.0772 | -69 | 249 | 258 | THQKIHAGVK | | | | | Mascot |
| 1344.5558 | 1344.6853 | 0.1295 | 96 | 229 | 239 | EKPYSCSECGK | | | Carbamidomethyl (C)[6,9] | | Mascot |
| 1446.6287 | 1446.574 | -0.0547 | -38 | 62 | 74 | NCFTCSQCGKSLR | | | | | Mascot |
| 1533.7955 | 1533.8055 | 0.01 | 7 | 100 | 112 | SFRTSSSLKPHMR | | | | | Mascot |
| 1549.7904 | 1549.8024 | 0.012 | 8 | 100 | 112 | SFRTSSSLKPHMR | | | Oxidation (M)[12] | | Mascot |
| 1560.6715 | 1560.8088 | 0.1373 | 88 | 62 | 74 | NCFTCSQCGKSLR | | | Carbamidomethyl (C)[2,5] | | Mascot |
| 1719.8284 | 1719.842 | 0.0136 | 8 | 30 | 43 | QEETEEQTDLKEIK | | | | | Mascot |
| 1752.8043 | 1752.8513 | 0.047 | 27 | 225 | 239 | VHTKEKPYSCSECGK | | | Carbamidomethyl (C)[10] | | Mascot |
| 2179.9392 | 2179.9604 | 0.0212 | 10 | 166 | 183 | HMMIHTGEKPFTCTECG K | | | Carbamidomethyl (C)[13,16], Oxidation (M)[2] | | Mascot |
| 2211.1702 | 2211.0718 | -0.0984 | -45 | 184 | 202 | SFIQLSTLNLHMRIHAGE K | | | Oxidation (M)[12] | | Mascot |
| 2501.1548 | 2501.21 | 0.0552 | 22 | 113 | 134 | IHTGEKPYTCTQCGKSFG QSSK | | | Carbamidomethyl (C)[10,13] | | Mascot |
| 2808.2056 | 2808.3599 | 0.1543 | 55 | 47 | 71 | LGDTEGIISMQSGNKNCF TCSQCGK | | | Carbamidomethyl (C)[17,20,23], Oxidation (M)[10] | | Mascot |

| | | | | | | | |
|---|---|---|---|---|---|---|---|
| 45 | hypothetical protein LOC751722 [Danio rerio] | gi\|113679753 | 31904.4 | 6.69 | 8 | 30 | 0 |

Peptide Information

| Calc. Mass | Obsrv. Mass | ± da | ± ppm | Start Seq. | End Seq. | Sequence | Ion Score | C. I. % | Modification | Rank | Result Type |
|---|---|---|---|---|---|---|---|---|---|---|---|
| 870.577 | 870.531 | -0.046 | -53 | 190 | 197 | NLLLKAAK | | | | | Mascot |
| 1062.5248 | 1062.6775 | 0.1527 | 144 | 182 | 189 | EQRLEMEK | | | | | Mascot |
| 1216.5449 | 1216.5925 | 0.0476 | 39 | 219 | 228 | HCADLLNMDK | | | Carbamidomethyl (C)[2] | | Mascot |

| Calc. Mass | Obsrv. Mass | ± da | ± ppm | Start Seq. | End Seq. | Sequence | Ion Score | C. I. % | Modification | Rank | Result Type |
|---|---|---|---|---|---|---|---|---|---|---|---|
| 1279.6616 | 1279.7557 | 0.0941 | 74 | 231 | 240 | NGNLMWLYLR | | | | | Mascot |
| 1557.7689 | 1557.7135 | -0.0554 | -36 | 103 | 115 | ALISHSQQLMEER | | | Oxidation (M)[10] | | Mascot |
| 1573.7097 | 1573.7151 | 0.0054 | 3 | 216 | 228 | NDKHCADLLNMDK | | | Carbamidomethyl (C)[5] | | Mascot |
| 1573.7097 | 1573.7151 | 0.0054 | 3 | 216 | 228 | NDKHCADLLNMDK | | | Carbamidomethyl (C)[5] | | Mascot |
| 1652.7875 | 1652.8027 | 0.0152 | 9 | 117 | 129 | HLQQEQQDLEQEK | | | | | Mascot |
| 2225.1409 | 2225.085 | -0.0559 | -25 | 74 | 92 | ETLTENQRTAAHLELELE K | | | | | Mascot |
| 2225.1409 | 2225.085 | -0.0559 | -25 | 74 | 92 | ETLTENQRTAAHLELELE K | | | | | Mascot |

46 novel protein [Danio rerio] gi|94732554 10949.6 4.74 4 30 0

**Peptide Information**

| Calc. Mass | Obsrv. Mass | ± da | ± ppm | Start Seq. | End Seq. | Sequence | Ion Score | C. I. % | Modification | Rank | Result Type |
|---|---|---|---|---|---|---|---|---|---|---|---|
| 850.4087 | 850.5313 | 0.1226 | 144 | 1 | 8 | MAALDASR | | | Oxidation (M)[1] | | Mascot |
| 2029.0713 | 2028.8933 | -0.178 | -88 | 44 | 61 | LPVSVKINPDAYSQDNIR | | | | | Mascot |
| 2045.0258 | 2044.9039 | -0.1219 | -60 | 9 | 27 | NVTEAEDAARLLSDAINS R | | | | | Mascot |
| 3338.7009 | 3338.7083 | 0.0074 | 2 | 64 | 93 | VGVADAESEHHIPITVMV PVYMTISELKEK | | | Oxidation (M)[17] | | Mascot |

47 toll-like receptor 6 [Danio rerio] gi|194578857 91036.5 6.38 11 30 0

**Peptide Information**

| Calc. Mass | Obsrv. Mass | ± da | ± ppm | Start Seq. | End Seq. | Sequence | Ion Score | C. I. % | Modification | Rank | Result Type |
|---|---|---|---|---|---|---|---|---|---|---|---|
| 850.4053 | 850.5313 | 0.126 | 148 | 65 | 71 | DFNTTPR | | | | | Mascot |
| 1279.6245 | 1279.7557 | 0.1312 | 103 | 228 | 237 | MEVVQQMRSR | | | Oxidation (M)[1] | | Mascot |
| 1320.6439 | 1320.6135 | -0.0304 | -23 | 749 | 758 | YYQLKTMMSR | | | | | Mascot |
| 1463.743 | 1463.6587 | -0.0843 | -58 | 640 | 651 | LHFHAFVSYSQK | | | | | Mascot |
| 1485.6726 | 1485.6229 | -0.0497 | -33 | 672 | 683 | MCHHERDFIPGK | | | Oxidation (M)[1] | | Mascot |
| 1493.7767 | 1493.7094 | -0.0673 | -45 | 271 | 283 | ELSLTDLTLTEMK | | | | | Mascot |
| 1560.7437 | 1560.8088 | 0.0651 | 42 | 131 | 144 | FSAMALGEEFSMLK | | | | | Mascot |
| 1577.7958 | 1577.8296 | 0.0338 | 21 | 760 | 772 | TYLEWPQEGAKQK | | | | | Mascot |
| 1652.9006 | 1652.8027 | -0.0979 | -59 | 534 | 548 | DFASLINAQGIKTFK | | | | | Mascot |
| 2024.0443 | 2023.8156 | -0.2287 | -113 | 326 | 344 | VSLTESPIIFMTCPGTISK | | | | | Mascot |
| 2024.0443 | 2023.8156 | -0.2287 | -113 | 326 | 344 | VSLTESPIIFMTCPGTISK | | | | | Mascot |
| 2225.1555 | 2225.085 | -0.0705 | -32 | 325 | 344 | KVSLTESPIIFMTCPGTIS K | | | Carbamidomethyl (C)[14], Oxidation (M)[12] | | Mascot |
| 2225.1555 | 2225.085 | -0.0705 | -32 | 325 | 344 | KVSLTESPIIFMTCPGTIS K | | | Carbamidomethyl (C)[14], Oxidation (M)[12] | | Mascot |

48 disks large homolog 5 [Danio rerio] gi|224496092 217705.3 6.7 1 21 0

Peptide Information

| Calc. Mass | Obsrv. Mass | ± da | ± ppm | Start Seq. | End Seq. | Sequence | Ion Score | C. I. % | Modification | Rank | Result Type |
|---|---|---|---|---|---|---|---|---|---|---|---|
| 1084.6473 | 1084.543 | -0.1043 | -96 | 327 | 335 | QIDALLKQR | 21 | 0 | | 2 | Mascot |

| | | | | | | | | |
|---|---|---|---|---|---|---|---|---|
| 49 | PREDICTED: Down syndrome cell adhesion molecule like 1 [Danio rerio] | gi\|292621329 | 232717.7 | 8.83 | 1 | | 13 | 0 |

Peptide Information

| Calc. Mass | Obsrv. Mass | ± da | ± ppm | Start Seq. | End Seq. | Sequence | Ion Score | C. I. % | Modification | Rank | Result Type |
|---|---|---|---|---|---|---|---|---|---|---|---|
| 1165.5782 | 1165.7288 | 0.1506 | 129 | 2 | 11 | TVPRMGGYER | 13 | 0 | | 1 | Mascot |

| | | | | | | | | |
|---|---|---|---|---|---|---|---|---|
| 50 | E3 ubiquitin-protein ligase NEDD4 [Danio rerio] | gi\|77681805 | 105816.1 | 6.16 | 1 | | 11 | 0 |

Peptide Information

| Calc. Mass | Obsrv. Mass | ± da | ± ppm | Start Seq. | End Seq. | Sequence | Ion Score | C. I. % | Modification | Rank | Result Type |
|---|---|---|---|---|---|---|---|---|---|---|---|
| 1165.7052 | 1165.7288 | 0.0236 | 20 | 543 | 552 | LKKPAEIPNR | 11 | 0 | | 2 | Mascot |

| | | | | | |
|---|---|---|---|---|---|
| **Gel Idx/Pos** | 188/H15 | **Instr./Gel Origin** | BA2060/110707A | **Process Status** | Analysis Succeeded |
| **Plate [#] Name** | [1] 31496 | **Instrument Sample Name** | | **Spectra** | 11 |

| Rank | Protein Name | Accession No. | Protein MW | Protein PI | Pep. Count | Protein Score | Protein Score C. I. % | Total Ion Score | Total Ion C. I. % |
|---|---|---|---|---|---|---|---|---|---|
| 1 | bifunctional purine biosynthesis protein PURH [Danio rerio] | gi\|195539484 | 64003.9 | 6.31 | 22 | 1,170 | 100 | 1019 | 100 |

Peptide Information

| Calc. Mass | Obsrv. Mass | ± da | ± ppm | Start Seq. | End Seq. | Sequence | Ion Score | C. I. % | Modification | Rank | Result Type |
|---|---|---|---|---|---|---|---|---|---|---|---|
| 869.5203 | 869.5105 | -0.0098 | -11 | 199 | 206 | GVSQLPLR | | | | | Mascot |
| 869.5203 | 869.5105 | -0.0098 | -11 | 199 | 206 | GVSQLPLR | 38 | 95.43 | | | Mascot |
| 892.4886 | 892.4758 | -0.0128 | -14 | 14 | 21 | TGLVEFAR | | | | | Mascot |
| 892.4886 | 892.4758 | -0.0128 | -14 | 14 | 21 | TGLVEFAR | 33 | 84.938 | | | Mascot |
| 926.5822 | 926.5717 | -0.0105 | -11 | 379 | 386 | VLFGLHLK | | | | | Mascot |
| 960.4686 | 960.4583 | -0.0103 | -11 | 460 | 466 | ADNWWLR | 42 | 98.302 | | | Mascot |
| 960.4686 | 960.4583 | -0.0103 | -11 | 460 | 466 | ADNWWLR | | | | | Mascot |
| 1033.536 | 1033.5251 | -0.0109 | -11 | 467 | 475 | HHPGVLSMR | | | | | Mascot |
| 1049.5309 | 1049.5206 | -0.0103 | -10 | 467 | 475 | HHPGVLSMR | | | Oxidation (M)[8] | | Mascot |
| 1075.6146 | 1075.6029 | -0.0117 | -11 | 255 | 265 | ALGLPAATSFK | | | | | Mascot |

| | | | | | | | | | | | |
|---|---|---|---|---|---|---|---|---|---|---|---|
| 1320.5889 | 1320.6573 | 0.0684 | 52 | 425 | 435 | YTQSNSVCYAK | | | Carbamidomethyl (C)[8] | | Mascot |
| 1341.7749 | 1341.761 | -0.0139 | -10 | 66 | 78 | TLHPAVHGGILAR | | | | | Mascot |
| 1341.7749 | 1341.761 | -0.0139 | -10 | 66 | 78 | TLHPAVHGGILAR | 92 | 100 | | | Mascot |
| 1385.7131 | 1385.6997 | -0.0134 | -10 | 436 | 449 | DGQVIGIGAGQQSR | 123 | 100 | | | Mascot |
| 1385.7131 | 1385.6997 | -0.0134 | -10 | 436 | 449 | DGQVIGIGAGQQSR | | | | | Mascot |
| 1444.7332 | 1444.7188 | -0.0144 | -10 | 455 | 466 | LAGDKADNWWLR | | | | | Mascot |
| 1607.7733 | 1607.7588 | -0.0145 | -9 | 49 | 63 | DVSEITGFPEMLGGR | | | | | Mascot |
| 1623.7683 | 1623.7515 | -0.0168 | -10 | 49 | 63 | DVSEITGFPEMLGGR | 38 | 95.409 | Oxidation (M)[11] | | Mascot |
| 1623.7683 | 1623.7515 | -0.0168 | -10 | 49 | 63 | DVSEITGFPEMLGGR | | | Oxidation (M)[11] | | Mascot |
| 1637.6636 | 1637.6448 | -0.0188 | -11 | 366 | 378 | MDPEYEPDEEEVR | | | | | Mascot |
| 1653.6584 | 1653.6389 | -0.0195 | -12 | 366 | 378 | MDPEYEPDEEEVR | | | Oxidation (M)[1] | | Mascot |
| 1665.8152 | 1665.8595 | 0.0443 | 27 | 141 | 155 | VTVVCDPSDYNVVAK | | | Carbamidomethyl (C)[5] | | Mascot |
| 1701.8582 | 1701.8429 | -0.0153 | -9 | 508 | 522 | GLFEEVPEPLSEVEK | | | | | Mascot |
| 1710.8955 | 1710.8761 | -0.0194 | -11 | 572 | 586 | ACNELGITLVHTNIR | | | Carbamidomethyl (C)[2] | | Mascot |
| 1710.8955 | 1710.8761 | -0.0194 | -11 | 572 | 586 | ACNELGITLVHTNIR | 102 | 100 | Carbamidomethyl (C)[2] | | Mascot |
| 1809.7629 | 1809.7441 | -0.0188 | -10 | 156 | 170 | EMETSEIHDTTMETR | | | | | Mascot |
| 1819.8708 | 1819.8468 | -0.024 | -13 | 335 | 351 | EVSDGIVAPGYEEDALR | | | | | Mascot |
| 1819.8708 | 1819.8468 | -0.024 | -13 | 335 | 351 | EVSDGIVAPGYEEDALR | 136 | 100 | | | Mascot |
| 1825.7579 | 1825.7393 | -0.0186 | -10 | 156 | 170 | EMETSEIHDTTMETR | | | Oxidation (M)[2] | | Mascot |
| 1829.9531 | 1829.9308 | -0.0223 | -12 | 508 | 523 | GLFEEVPEPLSEVEKK | | | | | Mascot |
| 1833.9341 | 1833.9143 | -0.0198 | -11 | 266 | 284 | HVSPAGAAVGVPLSEDE AK | | | | | Mascot |
| 1841.7528 | 1841.7614 | 0.0086 | 5 | 156 | 170 | EMETSEIHDTTMETR | | | Oxidation (M)[2,12] | | Mascot |
| 2034.9192 | 2034.8927 | -0.0265 | -13 | 177 | 193 | AFTHTAQYDEAISDYFR | | | | | Mascot |
| 2034.9192 | 2034.8927 | -0.0265 | -13 | 177 | 193 | AFTHTAQYDEAISDYFR | 150 | 100 | | | Mascot |
| 2225.0764 | 2225.084 | 0.0076 | 3 | 285 | 304 | VCMVNDMLQDLTPLATA YAR | | | | | Mascot |
| 2298.0928 | 2298.0984 | 0.0056 | 2 | 285 | 304 | VCMVNDMLQDLTPLATA YAR | | | Carbamidomethyl (C)[2], Oxidation (M)[3] | | Mascot |
| 2555.3562 | 2555.322 | -0.0342 | -13 | 108 | 132 | TVSSPGVTVEDAVEQIDI GGVTLLR | | | | | Mascot |
| 2555.3562 | 2555.322 | -0.0342 | -13 | 108 | 132 | TVSSPGVTVEDAVEQIDI GGVTLLR | 266 | 100 | | | Mascot |

2 PREDICTED: kinase suppressor of Ras 1 [Danio rerio] gi|326674294 98801.7 8.24 17 50 56.512

**Peptide Information**

| Calc. Mass | Obsrv. Mass | ± da | ± ppm | Start Seq. | End Seq. | Sequence | Ion Score | C. I. % | Modification | Rank | Result Type |
|---|---|---|---|---|---|---|---|---|---|---|---|
| 842.3898 | 842.4998 | 0.11 | 131 | 368 | 374 | NMMFGVK | | | Oxidation (M)[2] | | Mascot |
| 850.5396 | 850.5322 | -0.0074 | -9 | 83 | 89 | LVKYISK | | | | | Mascot |
| 892.4345 | 892.4758 | 0.0413 | 46 | 705 | 712 | GMGYLHAK | | | Oxidation (M)[2] | | Mascot |

| | | | | | | | | | | | | |
|---|---|---|---|---|---|---|---|---|---|---|---|---|
| | 892.4345 | 892.4758 | 0.0413 | 46 | 705 | 712 | GMGYLHAK | | | Oxidation (M)[2] | | Mascot |
| | 954.4899 | 954.4235 | -0.0664 | -70 | 367 | 374 | KNMMFGVK | | | | | Mascot |
| | 976.5057 | 976.4554 | -0.0503 | -52 | 583 | 591 | ASQTSVDLR | | | | | Mascot |
| | 982.4509 | 982.4392 | -0.0117 | -12 | 10 | 19 | GMETSSVSGK | | | | | Mascot |
| | 998.4458 | 998.4122 | -0.0336 | -34 | 10 | 19 | GMETSSVSGK | | | Oxidation (M)[2] | | Mascot |
| | 1051.5571 | 1051.7004 | 0.1433 | 136 | 395 | 403 | ISFAQFPNK | | | | | Mascot |
| | 1073.494 | 1073.4951 | 0.0011 | 1 | 368 | 376 | NMMFGVKCK | | | Oxidation (M)[2] | | Mascot |
| | 1075.521 | 1075.6029 | 0.0819 | 76 | 1 | 9 | MPLKPCQCR | | | | | Mascot |
| | 1102.6718 | 1102.6154 | -0.0564 | -51 | 50 | 59 | LIEISISSLK | | | | | Mascot |
| | 1118.5913 | 1118.6117 | 0.0204 | 18 | 310 | 319 | SKSQSQLANR | | | | | Mascot |
| | 1148.5374 | 1148.5958 | 0.0584 | 51 | 1 | 9 | MPLKPCQCR | | | Carbamidomethyl (C)[6], Oxidation (M)[1] | | Mascot |
| | 1320.7423 | 1320.6573 | -0.085 | -64 | 395 | 405 | ISFAQFPNKIR | | | | | Mascot |
| | 1607.8944 | 1607.7588 | -0.1356 | -84 | 759 | 771 | GWIYYLAPEIVRK | | | | | Mascot |
| | 1710.8553 | 1710.8761 | 0.0208 | 12 | 328 | 342 | MEYALSEGSPLVMRK | | | | | Mascot |
| | 1710.8553 | 1710.8761 | 0.0208 | 12 | 328 | 342 | MEYALSEGSPLVMRK | | | | | Mascot |
| | 2096.9507 | 2096.8096 | -0.1411 | -67 | 484 | 501 | GSADSRFHFPAACYFQH R | | | | | Mascot |
| | 2225.1382 | 2225.084 | -0.0542 | -24 | 731 | 750 | VVITDFGLFGMSGVVQE DRR | | | | | Mascot |
| | 2249.1265 | 2249.0305 | -0.096 | -43 | 133 | 152 | LSLDALLQMSSGQVEEN MKR | | | | | Mascot |

3 PREDICTED: zinc finger protein 420 isoform 2 [Danio rerio] gi|326678086 70371 9.08 16 48 27.828

**Peptide Information**

| Calc. Mass | Obsrv. Mass | ± da | ± ppm | Start Seq. | End Seq. | Sequence | Ion Score | C. I. % | Modification | Rank | Result Type |
|---|---|---|---|---|---|---|---|---|---|---|---|
| 842.5206 | 842.4998 | -0.0208 | -25 | 25 | 31 | QRQGLLK | | | | | Mascot |
| 864.4798 | 864.4897 | 0.0099 | 11 | 591 | 597 | HRSVHTK | | | | | Mascot |
| 998.4407 | 998.4122 | -0.0285 | -29 | 292 | 300 | THRCGHCGK | | | | | Mascot |
| 1045.536 | 1045.5522 | 0.0162 | 15 | 134 | 141 | FHCARNLK | | | Carbamidomethyl (C)[3] | | Mascot |
| 1073.5408 | 1073.4951 | -0.0457 | -43 | 598 | 606 | EKPPSCSLR | | | Carbamidomethyl (C)[6] | | Mascot |
| 1118.4645 | 1118.6117 | 0.1472 | 132 | 348 | 356 | GFVCFECEK | | | Carbamidomethyl (C)[4] | | Mascot |
| 1623.806 | 1623.7515 | -0.0545 | -34 | 105 | 117 | SFSQKYNLNVHMR | | | | | Mascot |
| 1623.806 | 1623.7515 | -0.0545 | -34 | 105 | 117 | SFSQKYNLNVHMR | | | | | Mascot |
| 1665.8087 | 1665.8595 | 0.0508 | 30 | 174 | 188 | VHTKEKPYSCSVCGK | | | | | Mascot |
| 1724.8247 | 1724.8828 | 0.0581 | 34 | 202 | 216 | IHTGVREFVCFECGK | | | | | Mascot |
| 1748.7731 | 1748.8676 | 0.0945 | 54 | 454 | 468 | VHTNEKPYSCPECGK | | | Carbamidomethyl (C)[10] | | Mascot |
| 1819.9358 | 1819.8468 | -0.089 | -49 | 44 | 58 | IKQEETEELIDVMVK | | | Oxidation (M)[13] | | Mascot |

| Calc. Mass | Obsrv. Mass | ± da | ± ppm | Start Seq. | End Seq. | Sequence | Ion Score | C. I. % | Modification | Rank | Result Type |
|---|---|---|---|---|---|---|---|---|---|---|---|
| 1819.9358 | 1819.8468 | -0.089 | -49 | 44 | 58 | IKQEETEELIDVMVK | | | Oxidation (M)[13] | | Mascot |
| 1847.8527 | 1847.8706 | 0.0179 | 10 | 454 | 469 | VHTNEKPYSCPECGKR | | | | | Mascot |
| 2034.8579 | 2034.8927 | 0.0348 | 17 | 118 | 133 | IHTGEQPYKCSHCDMR | | | Carbamidomethyl (C)[10,13], Oxidation (M)[15] | | Mascot |
| 2034.8579 | 2034.8927 | 0.0348 | 17 | 118 | 133 | IHTGEQPYKCSHCDMR | | | Carbamidomethyl (C)[10,13], Oxidation (M)[15] | | Mascot |
| 2211.0798 | 2211.0767 | -0.0031 | -1 | 450 | 468 | IHLRVHTNEKPYSCPECGK | | | | | Mascot |
| 2283.0579 | 2283.1169 | 0.059 | 26 | 227 | 245 | HQMIHTGEKPYKCSHCDLR | | | | | Mascot |
| 2298.0906 | 2298.0984 | 0.0078 | 3 | 478 | 496 | LHQHVHTGVREFVCFECEK | | | | | Mascot |

4 Synbl protein [Danio rerio] gi|141796272 31226.2 5.37 9 48 24.426

**Peptide Information**

| Calc. Mass | Obsrv. Mass | ± da | ± ppm | Start Seq. | End Seq. | Sequence | Ion Score | C. I. % | Modification | Rank | Result Type |
|---|---|---|---|---|---|---|---|---|---|---|---|
| 842.5709 | 842.4998 | -0.0711 | -84 | 18 | 24 | VEKLLLK | | | | | Mascot |
| 1045.6769 | 1045.5522 | -0.1247 | -119 | 171 | 179 | LLFLLSALR | | | | | Mascot |
| 1131.619 | 1131.5668 | -0.0522 | -46 | 87 | 97 | EGMLTLAGLAR | | | | | Mascot |
| 1541.901 | 1541.775 | -0.126 | -82 | 195 | 207 | LLTNVLERTLDVR | | | | | Mascot |
| 1678.8138 | 1678.85 | 0.0362 | 22 | 57 | 71 | SDLPLCQSTCLETLR | | | | | Mascot |
| 1710.7738 | 1710.8761 | 0.1023 | 60 | 1 | 16 | MSLSDILSEIDGGDDK | | | Oxidation (M)[1] | | Mascot |
| 1710.7738 | 1710.8761 | 0.1023 | 60 | 1 | 16 | MSLSDILSEIDGGDDK | | | Oxidation (M)[1] | | Mascot |
| 2034.9623 | 2034.8927 | -0.0696 | -34 | 238 | 255 | ALFNLTMSDPCDKDSLHK | | | | | Mascot |
| 2034.9623 | 2034.8927 | -0.0696 | -34 | 238 | 255 | ALFNLTMSDPCDKDSLHK | | | | | Mascot |
| 2249.0105 | 2249.0305 | 0.02 | 9 | 25 | 43 | YNTENSRTFAFDLEEEGAR | | | | | Mascot |
| 2283.0193 | 2283.1169 | 0.0976 | 43 | 98 | 118 | ICVAGHEGEPLEEAQSAEEER | | | | | Mascot |

5 Synbl protein [Danio rerio] gi|63101126 33515.4 5.82 9 46 0

**Peptide Information**

| Calc. Mass | Obsrv. Mass | ± da | ± ppm | Start Seq. | End Seq. | Sequence | Ion Score | C. I. % | Modification | Rank | Result Type |
|---|---|---|---|---|---|---|---|---|---|---|---|
| 842.5709 | 842.4998 | -0.0711 | -84 | 41 | 47 | VEKLLLK | | | | | Mascot |
| 1045.6769 | 1045.5522 | -0.1247 | -119 | 194 | 202 | LLFLLSALR | | | | | Mascot |
| 1131.619 | 1131.5668 | -0.0522 | -46 | 110 | 120 | EGMLTLAGLAR | | | | | Mascot |
| 1444.6447 | 1444.7188 | 0.0741 | 51 | 261 | 273 | ALFNLTMSDSCDK | | | | | Mascot |
| 1541.901 | 1541.775 | -0.126 | -82 | 218 | 230 | LLTNVLERTLDVR | | | | | Mascot |
| 1678.8138 | 1678.85 | 0.0362 | 22 | 80 | 94 | SDLPLCQSTCLETLR | | | | | Mascot |
| 1841.9829 | 1841.7614 | -0.2215 | -120 | 1 | 17 | YSLLLCAEGLTVFLSGR | | | | | Mascot |

| | | | | | | | | | | | |
|---|---|---|---|---|---|---|---|---|---|---|---|
| 2249.0105 | 2249.0305 | 0.02 | 9 | 48 | 66 | YNTENSRTFAFDLEEEGA R | | | | | Mascot |
| 2283.0193 | 2283.1169 | 0.0976 | 43 | 121 | 141 | ICVAGHEGEPLEEAQSAE EER | | | | | Mascot |

6 PREDICTED: myosin-9, partial [Danio rerio] gi|326665652 196608.4 5.46 21 45 0

**Peptide Information**

| Calc. Mass | Obsrv. Mass | ± da | ± ppm | Start Seq. | End Seq. | Sequence | Ion Score | C. I. % | Modification | Rank | Result Type |
|---|---|---|---|---|---|---|---|---|---|---|---|
| 892.4233 | 892.4758 | 0.0525 | 59 | 586 | 592 | ADEWLMK | | | | | Mascot |
| 892.4808 | 892.4758 | -0.005 | -6 | 1617 | 1623 | KMELDLK | | | Oxidation (M)[2] | | Mascot |
| 960.493 | 960.4583 | -0.0347 | -36 | 697 | 705 | CNGVLEGIR | 15 | 0 | | | Mascot |
| 960.493 | 960.4583 | -0.0347 | -36 | 697 | 705 | CNGVLEGIR | | | | | Mascot |
| 1033.5571 | 1033.5251 | -0.032 | -31 | 1607 | 1615 | QRTLAMAAR | | | Oxidation (M)[6] | | Mascot |
| 1045.5887 | 1045.5522 | -0.0365 | -35 | 1169 | 1177 | ETEVAQLKK | | | | | Mascot |
| 1051.5717 | 1051.7004 | 0.1287 | 122 | 818 | 826 | NCAAYLKLR | | | | | Mascot |
| 1073.5197 | 1073.4951 | -0.0246 | -23 | 425 | 432 | ATYERMFR | | | | | Mascot |
| 1075.608 | 1075.6029 | -0.0051 | -5 | 433 | 440 | WLVMRINK | | | Oxidation (M)[4] | | Mascot |
| 1118.5801 | 1118.6117 | 0.0316 | 28 | 1656 | 1664 | ELEDTRLSR | | | | | Mascot |
| 1131.6051 | 1131.5668 | -0.0383 | -34 | 805 | 813 | RQQQLTAMR | | | | | Mascot |
| 1341.6644 | 1341.761 | 0.0966 | 72 | 1517 | 1528 | DDVGKSVHDLEK | | | | | Mascot |
| 1341.6644 | 1341.761 | 0.0966 | 72 | 1517 | 1528 | DDVGKSVHDLEK | | | | | Mascot |
| 1558.8588 | 1558.8331 | -0.0257 | -16 | 722 | 734 | QRYEILTPNSIPK | | | | | Mascot |
| 1623.744 | 1623.7515 | 0.0075 | 5 | 379 | 392 | VCHLMGMNVTDFTR | 3 | 0 | | | Mascot |
| 1623.744 | 1623.7515 | 0.0075 | 5 | 379 | 392 | VCHLMGMNVTDFTR | | | | | Mascot |
| 1637.8546 | 1637.6448 | -0.2098 | -128 | 709 | 721 | QGFPNRIVFQEFR | | | | | Mascot |
| 1710.8302 | 1710.8761 | 0.0459 | 27 | 1561 | 1574 | LEVNMQAMKAQYER | | | | | Mascot |
| 1710.8302 | 1710.8761 | 0.0459 | 27 | 1561 | 1574 | LEVNMQAMKAQYER | | | | | Mascot |
| 1772.9442 | 1772.8203 | -0.1239 | -70 | 764 | 778 | VFFRAGVLAHLEEER | | | | | Mascot |
| 1809.8339 | 1809.7441 | -0.0898 | -50 | 278 | 292 | TFHMFYYMLTGVGDK | | | | | Mascot |
| 1825.8287 | 1825.7393 | -0.0894 | -49 | 278 | 292 | TFHMFYYMLTGVGDK | | | Oxidation (M)[4] | | Mascot |
| 1841.8237 | 1841.7614 | -0.0623 | -34 | 278 | 292 | TFHMFYYMLTGVGDK | | | Oxidation (M)[4,8] | | Mascot |
| 1847.9935 | 1847.8706 | -0.1229 | -67 | 623 | 640 | IVGLDKVAGMSELPGAFK | | | Oxidation (M)[10] | | Mascot |
| 2225.0979 | 2225.084 | -0.0139 | -6 | 1419 | 1436 | TRLQQELDDMLVDQDHL R | | | | | Mascot |
| 2298.0806 | 2298.0984 | 0.0178 | 8 | 999 | 1017 | LMEERIAEFTTNLAEEEE K | | | Oxidation (M)[2] | | Mascot |
| 2299.9951 | 2300.115 | 0.1199 | 52 | 158 | 178 | SMMQDREDQSILCTGES GAGK | | | Carbamidomethyl (C)[13] | | Mascot |

7 zinc finger protein Eos [Danio rerio] gi|294489252 53711.7 7.09 13 45 0

Peptide Information

| Calc. Mass | Obsrv. Mass | ± da | ± ppm | Start Seq. | End Seq. | Sequence | Ion Score | C. I. % | Modification | Rank | Result Type |
|---|---|---|---|---|---|---|---|---|---|---|---|
| 882.4791 | 882.5579 | 0.0788 | 89 | 196 | 203 | DALTGHLR | | | | | Mascot |
| 902.5417 | 902.5221 | -0.0196 | -22 | 283 | 290 | LANSITKR | | | | | Mascot |
| 1033.5248 | 1033.5251 | 0.0003 | 0 | 298 | 305 | FLGEKHMR | | | Oxidation (M)[7] | | Mascot |
| 1403.5765 | 1403.6858 | 0.1093 | 78 | 185 | 194 | CPFCNYACRR | | | Carbamidomethyl (C)[1,4,8] | | Mascot |
| 1558.7642 | 1558.8331 | 0.0689 | 44 | 417 | 430 | EAMQVVDGEGRPVR | | | Oxidation (M)[3] | | Mascot |
| 1623.7874 | 1623.7515 | -0.0359 | -22 | 469 | 481 | SQDRYEFSSHIVR | | | | | Mascot |
| 1623.7874 | 1623.7515 | -0.0359 | -22 | 469 | 481 | SQDRYEFSSHIVR | | | | | Mascot |
| 1724.7656 | 1724.8828 | 0.1172 | 68 | 382 | 396 | HSPSHAKEMDIEGDR | | | Oxidation (M)[9] | | Mascot |
| 1833.8865 | 1833.9143 | 0.0278 | 15 | 54 | 70 | VELYSDEEAGGAPLVER | | | | | Mascot |
| 2034.9302 | 2034.8927 | -0.0375 | -18 | 129 | 146 | CDICGMICIGPNVLMVHK | | | Carbamidomethyl (C)[1], Oxidation (M)[6,15] | | Mascot |
| 2034.9302 | 2034.8927 | -0.0375 | -18 | 129 | 146 | CDICGMICIGPNVLMVHK | | | Carbamidomethyl (C)[1], Oxidation (M)[6,15] | | Mascot |
| 2097.0869 | 2096.8096 | -0.2773 | -132 | 412 | 430 | SPLTREAMQVVDGEGRPVR | | | | | Mascot |
| 2225.0657 | 2225.084 | 0.0183 | 8 | 303 | 321 | HMRLNISEAPYDLDPNPDK | | | | | Mascot |
| 2283.0029 | 2283.1169 | 0.114 | 50 | 148 | 167 | SHTGERPFQCNQCGASFTQK | | | Carbamidomethyl (C)[10] | | Mascot |
| 2555.1978 | 2555.322 | 0.1242 | 49 | 100 | 126 | GQAGSGCGEMASSEPASPGPIRLPNGK | | | | | Mascot |
| 2555.1978 | 2555.322 | 0.1242 | 49 | 100 | 126 | GQAGSGCGEMASSEPASPGPIRLPNGK | | | | | Mascot |

8 calbindin 2a [Danio rerio] gi|41152295 31105.5 4.99 6 44 0 21 0

Peptide Information

| Calc. Mass | Obsrv. Mass | ± da | ± ppm | Start Seq. | End Seq. | Sequence | Ion Score | C. I. % | Modification | Rank | Result Type |
|---|---|---|---|---|---|---|---|---|---|---|---|
| 892.4556 | 892.4758 | 0.0202 | 23 | 171 | 178 | LGLSEMAR | | | Oxidation (M)[6] | | Mascot |
| 892.4556 | 892.4758 | 0.0202 | 23 | 171 | 178 | LGLSEMAR | 23 | 0 | Oxidation (M)[6] | | Mascot |
| 996.4454 | 996.5897 | 0.1443 | 145 | 162 | 170 | MFDLNGDGK | | | | | Mascot |
| 1118.5874 | 1118.6117 | 0.0243 | 22 | 259 | 267 | SELEIVLCR | | | Carbamidomethyl (C)[8] | | Mascot |
| 1320.5963 | 1320.6573 | 0.061 | 46 | 68 | 77 | MTEFMQKFDK | | | Oxidation (M)[1] | | Mascot |
| 1809.8694 | 1809.7441 | -0.1253 | -69 | 195 | 209 | LSAEQFNAIFTYYDK | | | | | Mascot |
| 2555.3293 | 2555.322 | -0.0073 | -3 | 5 | 26 | GQQPPYLHLAELTASQFLDIWK | | | | | Mascot |
| 2555.3293 | 2555.322 | -0.0073 | -3 | 5 | 26 | GQQPPYLHLAELTASQFLDIWK | | | | | Mascot |

9 PREDICTED: LOW QUALITY PROTEIN: ryanodine receptor 2 [Danio rerio] gi|189530774 552356.6 5.83 32 44 0

Peptide Information

| Calc. Mass | Obsrv. Mass | ± da | ± ppm | Start Seq. | End Seq. | Sequence | Ion Score | C. I. % | Modification | Rank | Result Type |
|---|---|---|---|---|---|---|---|---|---|---|---|
| 869.4111 | 869.5105 | 0.0994 | 114 | 1354 | 1360 | DELNNHK | | | | | Mascot |
| 869.4111 | 869.5105 | 0.0994 | 114 | 1354 | 1360 | DELNNHK | | | | | Mascot |
| 892.529 | 892.4758 | -0.0532 | -60 | 1525 | 1532 | LFPAVFAK | | | | | Mascot |
| 892.529 | 892.4758 | -0.0532 | -60 | 1525 | 1532 | LFPAVFAK | 10 | 0 | | | Mascot |
| 902.5557 | 902.5221 | -0.0336 | -37 | 590 | 597 | TIISLLDK | | | | | Mascot |
| 954.4526 | 954.4235 | -0.0291 | -30 | 659 | 666 | DDSAQYKK | | | | | Mascot |
| 960.4203 | 960.4583 | 0.038 | 40 | 2667 | 2674 | HSVMDSER | | | | | Mascot |
| 960.4203 | 960.4583 | 0.038 | 40 | 2667 | 2674 | HSVMDSER | | | | | Mascot |
| 976.4152 | 976.4554 | 0.0402 | 41 | 2667 | 2674 | HSVMDSER | | | Oxidation (M)[4] | | Mascot |
| 996.5624 | 996.5897 | 0.0273 | 27 | 3510 | 3516 | LEIYFRR | | | | | Mascot |
| 1045.5459 | 1045.5522 | 0.0063 | 6 | 75 | 83 | ALQEMLANR | | | | | Mascot |
| 1049.5626 | 1049.5206 | -0.042 | -40 | 4247 | 4255 | LTDLFGDLR | | | | | Mascot |
| 1051.5604 | 1051.7004 | 0.14 | 133 | 2440 | 2448 | AAMVLFLDR | | | Oxidation (M)[3] | | Mascot |
| 1073.5006 | 1073.4951 | -0.0055 | -5 | 2977 | 2985 | EMVTSLFCK | | | Oxidation (M)[2] | | Mascot |
| 1131.467 | 1131.5668 | 0.0998 | 88 | 23 | 32 | CCSSGPHQQK | | | Carbamidomethyl (C)[1] | | Mascot |
| 1165.567 | 1165.7366 | 0.1696 | 146 | 33 | 43 | LCLAAEGFGER | | | | | Mascot |
| 1320.6517 | 1320.6573 | 0.0056 | 4 | 2130 | 2139 | VFYQHPNLMR | | | Oxidation (M)[9] | | Mascot |
| 1341.6038 | 1341.761 | 0.1572 | 117 | 3404 | 3414 | MNKGGVFCQER | | | Carbamidomethyl (C)[8], Oxidation (M)[1] | | Mascot |
| 1341.6403 | 1341.761 | 0.1207 | 90 | 4175 | 4185 | MTRDLNCFSVR | | | | | Mascot |
| 1385.6478 | 1385.6997 | 0.0519 | 37 | 1585 | 1596 | VPDHTQSVEMSR | | | | | Mascot |
| 1385.6478 | 1385.6997 | 0.0519 | 37 | 1585 | 1596 | VPDHTQSVEMSR | | | | | Mascot |
| 1403.6663 | 1403.6858 | 0.0195 | 14 | 1349 | 1360 | YAGSRDELNNHK | | | | | Mascot |
| 1444.7253 | 1444.7188 | -0.0065 | -4 | 4570 | 4582 | VMAKYGELYGSAR | | | | | Mascot |
| 1558.7496 | 1558.8331 | 0.0835 | 54 | 3571 | 3583 | VWLKAENDSPEDR | | | | | Mascot |
| 1607.7218 | 1607.7588 | 0.037 | 23 | 4790 | 4802 | DQQEQVKEDMETK | | | | | Mascot |
| 1619.7854 | 1619.8684 | 0.083 | 51 | 2162 | 2174 | EIIFPKMVAHCCR | | | Carbamidomethyl (C)[11], Oxidation (M)[7] | | Mascot |
| 1623.7167 | 1623.7515 | 0.0348 | 21 | 4790 | 4802 | DQQEQVKEDMETK | | | Oxidation (M)[10] | | Mascot |
| 1623.7167 | 1623.7515 | 0.0348 | 21 | 4790 | 4802 | DQQEQVKEDMETK | | | Oxidation (M)[10] | | Mascot |
| 1653.6544 | 1653.6389 | -0.0155 | -9 | 3636 | 3649 | SCQSEEEQDEDGLK | | | Carbamidomethyl (C)[2] | | Mascot |
| 1665.7788 | 1665.8595 | 0.0807 | 48 | 1 | 14 | MDIGEAEDEIQFLR | | | | | Mascot |
| 1678.7708 | 1678.85 | 0.0792 | 47 | 4844 | 4857 | DVTEHTGQESYVWK | | | | | Mascot |
| 1772.7948 | 1772.8203 | 0.0255 | 14 | 515 | 529 | EAGECWEGVLNAFYK | | | Carbamidomethyl (C)[5] | | Mascot |
| 1841.8521 | 1841.7614 | -0.0907 | -49 | 3089 | 3105 | QSGAGLILDEVQSCCYR | | | | | Mascot |
| 1847.8998 | 1847.8706 | -0.0292 | -16 | 604 | 620 | ILDVLCSLCVCHGMAVR | | | Oxidation (M)[14] | | Mascot |

| Calc. Mass | Obsrv. Mass | ± da | ± ppm | Start Seq. | End Seq. | Sequence | Ion Score | C. I. % | Modification | Rank | Result Type |
|---|---|---|---|---|---|---|---|---|---|---|---|
| 2211.1018 | 2211.0767 | -0.0251 | -11 | 601 | 620 | NHKILDVLCSLCVCHGMAVR | | | | | Mascot |
| 2233.0884 | 2233.0576 | -0.0308 | -14 | 1898 | 1917 | VEAVISFSGDFVGHLQEDQR | | | | | Mascot |
| 2249.2024 | 2249.0305 | -0.1719 | -76 | 2517 | 2538 | SGPVDELLQGVYSLSKASGLTK | | | | | Mascot |
| 2283.2324 | 2283.1169 | -0.1155 | -51 | 3360 | 3378 | LFSLVAEVFIFWAKSHNFK | | | | | Mascot |
| 2300.2068 | 2300.115 | -0.0918 | -40 | 3106 | 3125 | ILSSLYILGTCSGSFIERQR | | | Carbamidomethyl (C)[11] | | Mascot |

10 RecName: Full=Tudor domain-containing protein 7B gi|334351210 121757 8.62 15 43 0

**Peptide Information**

| Calc. Mass | Obsrv. Mass | ± da | ± ppm | Start Seq. | End Seq. | Sequence | Ion Score | C. I. % | Modification | Rank | Result Type |
|---|---|---|---|---|---|---|---|---|---|---|---|
| 864.4495 | 864.4897 | 0.0402 | 47 | 939 | 945 | MEEKALK | | | Oxidation (M)[1] | | Mascot |
| 892.4774 | 892.4758 | -0.0016 | -2 | 544 | 550 | LFELNEK | | | | | Mascot |
| 892.4774 | 892.4758 | -0.0016 | -2 | 544 | 550 | LFELNEK | | | | | Mascot |
| 998.4901 | 998.4122 | -0.0779 | -78 | 876 | 885 | STPAGSPPER | | | | | Mascot |
| 1118.635 | 1118.6117 | -0.0233 | -21 | 10 | 19 | MVRAVLQSSK | | | | | Mascot |
| 1148.6198 | 1148.5958 | -0.024 | -21 | 33 | 42 | DLTGELIPYK | | | | | Mascot |
| 1320.665 | 1320.6573 | -0.0077 | -6 | 516 | 527 | AQVCEVMADKVK | | | | | Mascot |
| 1403.626 | 1403.6858 | 0.0598 | 43 | 477 | 486 | LEDEMREFYR | | | Oxidation (M)[5] | | Mascot |
| 1619.8639 | 1619.8684 | 0.0045 | 3 | 563 | 577 | LAGLESFSQEQAVLK | | | | | Mascot |
| 1710.873 | 1710.8761 | 0.0031 | 2 | 1053 | 1066 | KVMIYLVDTSQEER | | | | | Mascot |
| 1710.873 | 1710.8761 | 0.0031 | 2 | 1053 | 1066 | KVMIYLVDTSQEER | | | | | Mascot |
| 1809.9745 | 1809.7441 | -0.2304 | -127 | 967 | 983 | GVLTNGLVSVFQLDYGK | | | | | Mascot |
| 1842.0331 | 1841.7614 | -0.2717 | -148 | 837 | 854 | GPLKSLNVPTATQTSSLK | | | | | Mascot |
| 2225.1013 | 2225.084 | -0.0173 | -8 | 925 | 942 | LVVLMGEMILHYNKMEEK | | | Oxidation (M)[5,8,15] | | Mascot |
| 2249.1482 | 2249.0305 | -0.1177 | -52 | 445 | 464 | VEYPSVLVVDASDTNCVILR | | | Carbamidomethyl (C)[16] | | Mascot |
| 2283.0308 | 2283.1169 | 0.0861 | 38 | 1067 | 1085 | DIWLHDIMAEFTDEMTKTA | | | Oxidation (M)[8] | | Mascot |
| 2555.2769 | 2555.322 | 0.0451 | 18 | 492 | 515 | MALRSPSPGQLTAVGAEEEDDIIR | | | | | Mascot |
| 2555.2769 | 2555.322 | 0.0451 | 18 | 492 | 515 | MALRSPSPGQLTAVGAEEEDDIIR | 3 | 0 | | | Mascot |

11 Transferrin receptor 1a [Danio rerio] gi|190337022 84943 6.38 10 43 0

**Peptide Information**

| Calc. Mass | Obsrv. Mass | ± da | ± ppm | Start Seq. | End Seq. | Sequence | Ion Score | C. I. % | Modification | Rank | Result Type |
|---|---|---|---|---|---|---|---|---|---|---|---|
| 842.4842 | 842.4998 | 0.0156 | 19 | 66 | 72 | LNQRSPK | | | | | Mascot |
| 856.4747 | 856.5146 | 0.0399 | 47 | 344 | 351 | LGGRNSPR | | | | | Mascot |

| Calc. Mass | Obsrv. Mass | ± da | ± ppm | Start Seq. | End Seq. | Sequence | Ion Score | C. I. % | Modification | Rank | Result Type |
|---|---|---|---|---|---|---|---|---|---|---|---|
| 960.5472 | 960.4583 | -0.0889 | -93 | 662 | 671 | AASALTSAIR | 15 | 0 | | | Mascot |
| 960.5472 | 960.4583 | -0.0889 | -93 | 662 | 671 | AASALTSAIR | | | | | Mascot |
| 976.4634 | 976.4554 | -0.008 | -8 | 409 | 417 | DAWGPGFAR | | | | | Mascot |
| 1045.5935 | 1045.5522 | -0.0413 | -39 | 683 | 690 | IINNRIMR | | | Oxidation (M)[7] | | Mascot |
| 1049.5156 | 1049.5206 | 0.005 | 5 | 634 | 642 | VESRQMSGR | | | | | Mascot |
| 1403.7529 | 1403.6858 | -0.0671 | -48 | 691 | 703 | VEGSLLSPYVSPR | | | | | Mascot |
| 1607.8462 | 1607.7588 | -0.0874 | -54 | 431 | 444 | TITDMIKNDGFKPK | | | | | Mascot |
| 1623.8411 | 1623.7515 | -0.0896 | -55 | 431 | 444 | TITDMIKNDGFKPK | | | Oxidation (M)[5] | | Mascot |
| 1623.8411 | 1623.7515 | -0.0896 | -55 | 431 | 444 | TITDMIKNDGFKPK | | | Oxidation (M)[5] | | Mascot |
| 1819.9734 | 1819.8468 | -0.1266 | -70 | 688 | 703 | IMRVEGSLLSPYVSPR | | | Oxidation (M)[2] | | Mascot |
| 1819.9734 | 1819.8468 | -0.1266 | -70 | 688 | 703 | IMRVEGSLLSPYVSPR | | | Oxidation (M)[2] | | Mascot |
| 2283.0339 | 2283.1169 | 0.083 | 36 | 662 | 682 | AASALTSAIRNSDLDDME QCR | | | Oxidation (M)[17] | | Mascot |

| | | | | | | | | | | |
|---|---|---|---|---|---|---|---|---|---|---|
| 12 | Zgc:152741 protein [Danio rerio] | gi\|66267405 | 41026.8 | 5.7 | 5 | 42 | 0 | 26 | 24.512 |

**Peptide Information**

| Calc. Mass | Obsrv. Mass | ± da | ± ppm | Start Seq. | End Seq. | Sequence | Ion Score | C. I. % | Modification | Rank | Result Type |
|---|---|---|---|---|---|---|---|---|---|---|---|
| 869.5315 | 869.5105 | -0.021 | -24 | 319 | 326 | GASIRIPR | | | | | Mascot |
| 869.5315 | 869.5105 | -0.021 | -24 | 319 | 326 | GASIRIPR | 26 | 24.512 | | | Mascot |
| 976.5071 | 976.4554 | -0.0517 | -53 | 279 | 285 | RHEEHIR | | | | | Mascot |
| 1049.472 | 1049.5206 | 0.0486 | 46 | 173 | 180 | DIVDCHYK | | | Carbamidomethyl (C)[5] | | Mascot |
| 1558.6954 | 1558.8331 | 0.1377 | 88 | 327 | 339 | QVGQEKCGYFEDR | | | | | Mascot |
| 1653.833 | 1653.6389 | -0.1941 | -117 | 262 | 275 | EEEGIEHIEKAIEK | | | | | Mascot |

| | | | | | | | | |
|---|---|---|---|---|---|---|---|---|
| 13 | PREDICTED: zinc-binding protein A33-like [Danio rerio] | gi\|292610346 | 58802.6 | 7.49 | 10 | 42 | 0 |

**Peptide Information**

| Calc. Mass | Obsrv. Mass | ± da | ± ppm | Start Seq. | End Seq. | Sequence | Ion Score | C. I. % | Modification | Rank | Result Type |
|---|---|---|---|---|---|---|---|---|---|---|---|
| 869.4951 | 869.5105 | 0.0154 | 18 | 398 | 404 | RNPEVVR | | | | | Mascot |
| 869.4951 | 869.5105 | 0.0154 | 18 | 398 | 404 | RNPEVVR | 15 | 0 | | | Mascot |
| 892.5137 | 892.4758 | -0.0379 | -42 | 173 | 179 | IKVEFEK | | | | | Mascot |
| 892.5137 | 892.4758 | -0.0379 | -42 | 173 | 179 | IKVEFEK | | | | | Mascot |
| 960.4277 | 960.4583 | 0.0306 | 32 | 473 | 479 | KMSFMER | | | Oxidation (M)[2,5] | | Mascot |
| 960.4277 | 960.4583 | 0.0306 | 32 | 473 | 479 | KMSFMER | | | Oxidation (M)[2,5] | | Mascot |
| 1341.6984 | 1341.761 | 0.0626 | 47 | 350 | 360 | HWSLGVCLELK | | | Carbamidomethyl (C)[7] | | Mascot |
| 1341.6984 | 1341.761 | 0.0626 | 47 | 350 | 360 | HWSLGVCLELK | | | Carbamidomethyl (C)[7] | | Mascot |
| 1403.6559 | 1403.6858 | 0.0299 | 21 | 18 | 30 | DPVLLGCGHSFCR | | | | | Mascot |

| | | | | | | | | | | | |
|---|---|---|---|---|---|---|---|---|---|---|---|
| 1558.7206 | 1558.8331 | 0.1125 | 72 | 423 | 436 | VSFFDASCDAVIER | | | | | Mascot |
| 1772.783 | 1772.8203 | 0.0373 | 21 | 2 | 17 | SLEDDLSCAVCTDVFR | | | | | Mascot |
| 1829.8044 | 1829.9308 | 0.1264 | 69 | 2 | 17 | SLEDDLSCAVCTDVFR | | | Carbamidomethyl (C)[8] | | Mascot |
| 2225.0259 | 2225.084 | 0.0581 | 26 | 95 | 111 | IELFCQNDEQLLCARCR | | | Carbamidomethyl (C)[5,13,16] | | Mascot |
| 2233.0085 | 2233.0576 | 0.0491 | 22 | 380 | 397 | ERYMYHLMTAEGCEINIK | | | Oxidation (M)[4,8] | | Mascot |
| 2282.9851 | 2283.1169 | 0.1318 | 58 | 31 | 49 | QCIYDHWSSSGTRNCPICR | | | Carbamidomethyl (C)[2] | | Mascot |

| | | | | | | | | |
|---|---|---|---|---|---|---|---|---|
| 14 | PREDICTED: rapamycin-insensitive companion of mTOR [Danio rerio] | gi\|189533864 | 193500.8 | 8.2 | 18 | 41 | 0 | |

**Peptide Information**

| Calc. Mass | Obsrv. Mass | ± da | ± ppm | Start Seq. | End Seq. | Sequence | Ion Score | C. I. % | Modification | Rank | Result Type |
|---|---|---|---|---|---|---|---|---|---|---|---|
| 864.5189 | 864.4897 | -0.0292 | -34 | 1102 | 1109 | FLISLSGK | | | | | Mascot |
| 892.492 | 892.4758 | -0.0162 | -18 | 286 | 293 | MSIVASLR | | | Oxidation (M)[1] | | Mascot |
| 892.492 | 892.4758 | -0.0162 | -18 | 286 | 293 | MSIVASLR | 5 | 0 | Oxidation (M)[1] | | Mascot |
| 954.5002 | 954.4235 | -0.0767 | -80 | 534 | 541 | DSQILNHK | | | | | Mascot |
| 1045.5887 | 1045.5522 | -0.0365 | -35 | 1652 | 1661 | GNETGLLTIK | | | | | Mascot |
| 1049.5599 | 1049.5206 | -0.0393 | -37 | 509 | 516 | DQHLRPQR | | | | | Mascot |
| 1051.5782 | 1051.7004 | 0.1222 | 116 | 822 | 830 | GYVSKQLEK | | | | | Mascot |
| 1385.7134 | 1385.6997 | -0.0137 | -10 | 326 | 336 | GLLEVMYEIFR | | | Oxidation (M)[6] | | Mascot |
| 1385.7134 | 1385.6997 | -0.0137 | -10 | 326 | 336 | GLLEVMYEIFR | | | Oxidation (M)[6] | | Mascot |
| 1541.8145 | 1541.775 | -0.0395 | -26 | 325 | 336 | RGLLEVMYEIFR | | | Oxidation (M)[7] | | Mascot |
| 1558.7344 | 1558.8331 | 0.0987 | 63 | 19 | 32 | NDSGEENVPLDLTR | | | | | Mascot |
| 1623.8635 | 1623.7515 | -0.112 | -69 | 1621 | 1634 | NIPEDAPACRVLLR | | | Carbamidomethyl (C)[9] | | Mascot |
| 1623.8635 | 1623.7515 | -0.112 | -69 | 1621 | 1634 | NIPEDAPACRVLLR | | | Carbamidomethyl (C)[9] | | Mascot |
| 1653.8629 | 1653.6389 | -0.224 | -135 | 720 | 734 | ILTAATDNCRLYATK | | | | | Mascot |
| 1710.8744 | 1710.8761 | 0.0017 | 1 | 980 | 994 | QGCEVLKLHGWDAVR | | | | | Mascot |
| 1710.8843 | 1710.8761 | -0.0082 | -5 | 720 | 734 | ILTAATDNCRLYATK | | | Carbamidomethyl (C)[9] | | Mascot |
| 1819.8629 | 1819.8468 | -0.0161 | -9 | 765 | 781 | AVSVEALDILDEACEDK | | | | | Mascot |
| 1819.8629 | 1819.8468 | -0.0161 | -9 | 765 | 781 | AVSVEALDILDEACEDK | | | | | Mascot |
| 2034.99 | 2034.8927 | -0.0973 | -48 | 763 | 781 | SKAVSVEALDILDEACEDK | | | | | Mascot |
| 2035.0826 | 2034.8927 | -0.1899 | -93 | 286 | 303 | MSIVASLRSWSGIINLCK | | | Carbamidomethyl (C)[17] | | Mascot |
| 2249.1003 | 2249.0305 | -0.0698 | -31 | 1181 | 1200 | TLDTQDSTSSIGEQEVRLNR | | | | | Mascot |
| 2283.22 | 2283.1169 | -0.1031 | -45 | 186 | 205 | ACIAIICELALENPEIVAKR | | | Carbamidomethyl (C)[2,7] | | Mascot |
| 2298.2312 | 2298.0984 | -0.1328 | -58 | 1358 | 1378 | LQQQRIHPSLSHSEALASPAK | | | | | Mascot |

| | | | | | | | | |
|---|---|---|---|---|---|---|---|---|
| 15 | PREDICTED: protein QN1 homolog [Danio rerio] | gi\|68385147 | 155728.8 | 5.89 | 17 | 40 | 0 | |

Peptide Information

| Calc. Mass | Obsrv. Mass | ± da | ± ppm | Start Seq. | End Seq. | Sequence | Ion Score | C. I. % | Modification | Rank | Result Type |
|---|---|---|---|---|---|---|---|---|---|---|---|
| 850.5145 | 850.5322 | 0.0177 | 21 | 81 | 87 | SFLKSLR | | | | | Mascot |
| 856.4821 | 856.5146 | 0.0325 | 38 | 1 | 7 | MAHRLTK | | | | | Mascot |
| 902.5053 | 902.5221 | 0.0168 | 19 | 595 | 601 | ERELSLR | | | | | Mascot |
| 926.44 | 926.5717 | 0.1317 | 142 | 1285 | 1291 | IQSMEFR | | | Oxidation (M)[4] | | Mascot |
| 990.4785 | 990.4245 | -0.054 | -55 | 713 | 720 | MNTNNIQR | | | | | Mascot |
| 1049.599 | 1049.5206 | -0.0784 | -75 | 702 | 710 | LLTELSFAR | | | | | Mascot |
| 1131.619 | 1131.5668 | -0.0522 | -46 | 777 | 786 | DLAKVQAVCK | | | Carbamidomethyl (C)[9] | | Mascot |
| 1320.7271 | 1320.6573 | -0.0698 | -53 | 702 | 712 | LLTELSFARDR | | | | | Mascot |
| 1444.7002 | 1444.7188 | 0.0186 | 13 | 1285 | 1295 | IQSMEFRYSQR | | | | | Mascot |
| 1710.9133 | 1710.8761 | -0.0372 | -22 | 166 | 180 | INREQELSAPTSPLR | | | | | Mascot |
| 1710.9133 | 1710.8761 | -0.0372 | -22 | 166 | 180 | INREQELSAPTSPLR | | | | | Mascot |
| 1748.9766 | 1748.8676 | -0.109 | -62 | 1161 | 1176 | VSLQAAVTHAQNQLLR | | | | | Mascot |
| 1809.8549 | 1809.7441 | -0.1108 | -61 | 1084 | 1099 | TMLSGPSHERPNNEPK | | | Oxidation (M)[2] | | Mascot |
| 1819.8239 | 1819.8468 | 0.0229 | 13 | 296 | 313 | QISGSAGVCEDGQVEAG R | | | Carbamidomethyl (C)[9] | | Mascot |
| 1819.8239 | 1819.8468 | 0.0229 | 13 | 296 | 313 | QISGSAGVCEDGQVEAG R | | | Carbamidomethyl (C)[9] | | Mascot |
| 1829.979 | 1829.9308 | -0.0482 | -26 | 1337 | 1351 | MELDSILDVLRELQR | | | | | Mascot |
| 1847.8625 | 1847.8706 | 0.0081 | 4 | 570 | 586 | GMQEKMDGGPELASLER | | | | | Mascot |
| 1863.8575 | 1863.8599 | 0.0024 | 1 | 570 | 586 | GMQEKMDGGPELASLER | | | Oxidation (M)[2] | | Mascot |
| 2225.123 | 2225.084 | -0.039 | -18 | 423 | 441 | RLMQEQDTSSQEPPPLK PK | | | Oxidation (M)[3] | | Mascot |
| 2555.2559 | 2555.322 | 0.0661 | 26 | 690 | 710 | QNEEALFMENQRLLTELS FAR | | | Oxidation (M)[8] | | Mascot |
| 2555.2559 | 2555.322 | 0.0661 | 26 | 690 | 710 | QNEEALFMENQRLLTELS FAR | | | Oxidation (M)[8] | | Mascot |

16 Zgc:103631 protein [Danio rerio] gi|158253732 37789.7 8.19 9 40 0

Peptide Information

| Calc. Mass | Obsrv. Mass | ± da | ± ppm | Start Seq. | End Seq. | Sequence | Ion Score | C. I. % | Modification | Rank | Result Type |
|---|---|---|---|---|---|---|---|---|---|---|---|
| 864.4066 | 864.4897 | 0.0831 | 96 | 100 | 107 | CLQAGMNK | | | | | Mascot |
| 869.4259 | 869.5105 | 0.0846 | 97 | 337 | 343 | LLCDMFK | | | | | Mascot |
| 869.4259 | 869.5105 | 0.0846 | 97 | 337 | 343 | LLCDMFK | | | | | Mascot |
| 926.4474 | 926.5717 | 0.1243 | 134 | 337 | 343 | LLCDMFK | | | Carbamidomethyl (C)[3] | | Mascot |
| 960.5546 | 960.4583 | -0.0963 | -100 | 32 | 40 | VLNTGLLCK | | | | | Mascot |
| 960.5546 | 960.4583 | -0.0963 | -100 | 32 | 40 | VLNTGLLCK | | | | | Mascot |
| 990.4924 | 990.4245 | -0.0679 | -69 | 328 | 336 | TIGNTPMEK | | | | | Mascot |

| Calc. Mass | Obsrv. Mass | ± da | ± ppm | Start Seq. | End Seq. | Sequence | Ion Score | C. I. % | Modification | Rank | Result Type |
|---|---|---|---|---|---|---|---|---|---|---|---|
| 1049.5231 | 1049.5206 | -0.0025 | -2 | 99 | 107 | KCLQAGMNK | | | Carbamidomethyl (C)[2] | | Mascot |
| 1131.5892 | 1131.5668 | -0.0224 | -20 | 124 | 133 | LDALDVDKDK | | | | | Mascot |
| 1403.6368 | 1403.6858 | 0.049 | 35 | 182 | 194 | FMASLMTAETCAK | | | | | Mascot |
| 1558.6876 | 1558.8331 | 0.1455 | 93 | 1 | 14 | MSMVPFSSPTESSR | | | Oxidation (M)[1] | | Mascot |
| 1833.9453 | 1833.9143 | -0.031 | -17 | 227 | 243 | GSPSASDVRVLQEVFSR | | | | | Mascot |

17 RecName: Full=Probable tRNA threonylcarbamoyladenosine biosynthesis protein osgepl1; AltName: Full= gi|160013234 43929.2 8.7 9 39 0

**Peptide Information**

| Calc. Mass | Obsrv. Mass | ± da | ± ppm | Start Seq. | End Seq. | Sequence | Ion Score | C. I. % | Modification | Rank | Result Type |
|---|---|---|---|---|---|---|---|---|---|---|---|
| 1073.6313 | 1073.4951 | -0.1362 | -127 | 246 | 255 | TQITGAINKK | | | | | Mascot |
| 1148.5881 | 1148.5958 | 0.0077 | 7 | 2 | 11 | FQSCLPGALR | | | Carbamidomethyl (C)[4] | | Mascot |
| 1607.8112 | 1607.7588 | -0.0524 | -33 | 2 | 15 | FQSCLPGALRSWSR | | | | | Mascot |
| 1619.85 | 1619.8684 | 0.0184 | 11 | 272 | 287 | DIAAASQHTVASHLAK | | | | | Mascot |
| 1623.835 | 1623.7515 | -0.0835 | -51 | 12 | 25 | SWSRGVFSTSTRPR | | | | | Mascot |
| 1623.835 | 1623.7515 | -0.0835 | -51 | 12 | 25 | SWSRGVFSTSTRPR | | | | | Mascot |
| 1678.9197 | 1678.85 | -0.0697 | -42 | 327 | 342 | IITDATGLHLLCPPSK | | | | | Mascot |
| 1809.8687 | 1809.7441 | -0.1246 | -69 | 256 | 271 | EKEEGVEAGQFLSCVK | | | Carbamidomethyl (C)[14] | | Mascot |
| 1825.8359 | 1825.7393 | -0.0966 | -53 | 343 | 358 | FCTDNGVMIAWNGIER | | | | | Mascot |
| 1841.8309 | 1841.7614 | -0.0695 | -38 | 343 | 358 | FCTDNGVMIAWNGIER | | | Oxidation (M)[8] | | Mascot |
| 2249.0964 | 2249.0305 | -0.0659 | -29 | 26 | 47 | LVLGIETSCDETGAAVLD ETGR | | | | | Mascot |

18 afadin- and alpha-actinin-binding protein [Danio rerio] gi|41387198 66121.4 6.93 13 39 0 4 0

**Peptide Information**

| Calc. Mass | Obsrv. Mass | ± da | ± ppm | Start Seq. | End Seq. | Sequence | Ion Score | C. I. % | Modification | Rank | Result Type |
|---|---|---|---|---|---|---|---|---|---|---|---|
| 850.4165 | 850.5322 | 0.1157 | 136 | 432 | 437 | NFERER | | | | | Mascot |
| 960.5109 | 960.4583 | -0.0526 | -55 | 323 | 330 | EQLTNSIR | | | | | Mascot |
| 960.5109 | 960.4583 | -0.0526 | -55 | 323 | 330 | EQLTNSIR | | | | | Mascot |
| 976.4945 | 976.4554 | -0.0391 | -40 | 162 | 169 | SEKEEVQK | | | | | Mascot |
| 990.5288 | 990.4245 | -0.1043 | -105 | 199 | 206 | LNQLLMDK | | | Oxidation (M)[6] | | Mascot |
| 1033.4871 | 1033.5251 | 0.038 | 37 | 1 | 9 | MPDPTLDTK | | | Oxidation (M)[1] | | Mascot |
| 1049.5739 | 1049.5206 | -0.0533 | -51 | 438 | 446 | KNFTEAAIR | | | | | Mascot |
| 1131.6442 | 1131.5668 | -0.0774 | -68 | 273 | 282 | KDMIAILSPK | | | Oxidation (M)[3] | | Mascot |
| 1148.5914 | 1148.5958 | 0.0044 | 4 | 373 | 381 | MRLELQQCK | | | | | Mascot |

| | | | | | | | | | | | |
|---|---|---|---|---|---|---|---|---|---|---|---|
| 1341.6389 | 1341.761 | 0.1221 | 91 | 256 | 266 | SLMMENSELKK | | | Oxidation (M)[3,4] | | Mascot |
| 1341.6389 | 1341.761 | 0.1221 | 91 | 256 | 266 | SLMMENSELKK | | | Oxidation (M)[3,4] | | Mascot |
| 1403.7125 | 1403.6858 | -0.0267 | -19 | 343 | 355 | LDSQASLVASQER | | | | | Mascot |
| 1558.8695 | 1558.8331 | -0.0364 | -23 | 274 | 287 | DMIAILSPKKPCVK | | | Oxidation (M)[2] | | Mascot |
| 1623.8336 | 1623.7515 | -0.0821 | -51 | 211 | 225 | LSIEVSNYVGRSDGK | | | | | Mascot |
| 1623.8336 | 1623.7515 | -0.0821 | -51 | 211 | 225 | LSIEVSNYVGRSDGK | | | | | Mascot |
| 2555.0322 | 2555.322 | 0.2898 | 113 | 288 | 311 | AELADDSLEQAGSECED EAGESSR | | | Carbamidomethyl (C)[15] | | Mascot |
| 2555.0322 | 2555.322 | 0.2898 | 113 | 288 | 311 | AELADDSLEQAGSECED EAGESSR | 4 | 0 | Carbamidomethyl (C)[15] | | Mascot |

| | | | | | | | |
|---|---|---|---|---|---|---|---|
| 19 | N(6)-adenine-specific DNA methyltransferase 2 [Danio rerio] | gi\|46309537 | 18902.4 | 5.21 | 5 | 38 | 0 |

**Peptide Information**

| Calc. Mass | Obsrv. Mass | ± da | ± ppm | Start Seq. | End Seq. | Sequence | Ion Score | C. I. % | Modification | Rank | Result Type |
|---|---|---|---|---|---|---|---|---|---|---|---|
| 869.4008 | 869.5105 | 0.1097 | 126 | 139 | 145 | MCSFLPR | | | Oxidation (M)[1] | | Mascot |
| 869.4008 | 869.5105 | 0.1097 | 126 | 139 | 145 | MCSFLPR | 9 | 0 | Oxidation (M)[1] | | Mascot |
| 1033.4408 | 1033.5251 | 0.0843 | 82 | 156 | 163 | CYVNYESR | | | | | Mascot |
| 1403.6624 | 1403.6858 | 0.0234 | 17 | 156 | 166 | CYVNYESRLLS | | | Carbamidomethyl (C)[1] | | Mascot |
| 1637.6901 | 1637.6448 | -0.0453 | -28 | 1 | 13 | MSQFWYSEETASR | | | Oxidation (M)[1] | | Mascot |
| 2225.2031 | 2225.084 | -0.1191 | -54 | 119 | 138 | ILLCTGAIMEEHAGKLLDL K | | | Carbamidomethyl (C)[4] | | Mascot |

| | | | | | | | |
|---|---|---|---|---|---|---|---|
| 20 | transferrin receptor 1a [Danio rerio] | gi\|57770576 | 84944.1 | 6.38 | 9 | 38 | 0 |

**Peptide Information**

| Calc. Mass | Obsrv. Mass | ± da | ± ppm | Start Seq. | End Seq. | Sequence | Ion Score | C. I. % | Modification | Rank | Result Type |
|---|---|---|---|---|---|---|---|---|---|---|---|
| 842.4842 | 842.4998 | 0.0156 | 19 | 66 | 72 | LNQRSPK | | | | | Mascot |
| 960.5472 | 960.4583 | -0.0889 | -93 | 662 | 671 | AASALTSAIR | 15 | 0 | | | Mascot |
| 960.5472 | 960.4583 | -0.0889 | -93 | 662 | 671 | AASALTSAIR | | | | | Mascot |
| 976.4634 | 976.4554 | -0.008 | -8 | 409 | 417 | DAWGPGFAR | | | | | Mascot |
| 1045.5935 | 1045.5522 | -0.0413 | -39 | 683 | 690 | IINNRIMR | | | Oxidation (M)[7] | | Mascot |
| 1049.5156 | 1049.5206 | 0.005 | 5 | 634 | 642 | VESRQMSGR | | | | | Mascot |
| 1403.7529 | 1403.6858 | -0.0671 | -48 | 691 | 703 | VEGSLLSPYVSPR | | | | | Mascot |
| 1607.8462 | 1607.7588 | -0.0874 | -54 | 431 | 444 | TITDMIKNDGFKPK | | | | | Mascot |
| 1623.8411 | 1623.7515 | -0.0896 | -55 | 431 | 444 | TITDMIKNDGFKPK | | | Oxidation (M)[5] | | Mascot |
| 1623.8411 | 1623.7515 | -0.0896 | -55 | 431 | 444 | TITDMIKNDGFKPK | | | Oxidation (M)[5] | | Mascot |
| 1819.9734 | 1819.8468 | -0.1266 | -70 | 688 | 703 | IMRVEGSLLSPYVSPR | | | Oxidation (M)[2] | | Mascot |
| 1819.9734 | 1819.8468 | -0.1266 | -70 | 688 | 703 | IMRVEGSLLSPYVSPR | | | Oxidation (M)[2] | | Mascot |

| Calc. Mass | Obsrv. Mass | ± da | ± ppm | Start Seq. | End Seq. | Sequence | Ion Score | C. I. % | Modification | Rank | Result Type |
|---|---|---|---|---|---|---|---|---|---|---|---|
| 2283.0339 | 2283.1169 | 0.083 | 36 | 662 | 682 | AASALTSAIRNSDLDDME QCR | | | Oxidation (M)[17] | | Mascot |

21 glutamine synthetase [Danio rerio] gi|115430097 41664.2 5.85 4 37 0 26 24.512

Peptide Information

| Calc. Mass | Obsrv. Mass | ± da | ± ppm | Start Seq. | End Seq. | Sequence | Ion Score | C. I. % | Modification | Rank | Result Type |
|---|---|---|---|---|---|---|---|---|---|---|---|
| 869.5315 | 869.5105 | -0.021 | -24 | 325 | 332 | GASIRIPR | | | | | Mascot |
| 869.5315 | 869.5105 | -0.021 | -24 | 325 | 332 | GASIRIPR | 26 | 24.512 | | | Mascot |
| 976.5071 | 976.4554 | -0.0517 | -53 | 285 | 291 | RHEEHIR | | | | | Mascot |
| 1049.472 | 1049.5206 | 0.0486 | 46 | 179 | 186 | DIVDCHYK | | | Carbamidomethyl (C)[5] | | Mascot |
| 1558.6954 | 1558.8331 | 0.1377 | 88 | 333 | 345 | QVGQEKCGYFEDR | | | | | Mascot |

22 hypothetical protein LOC553197 [Danio rerio] gi|66392211 58633.7 7.78 9 37 0

Peptide Information

| Calc. Mass | Obsrv. Mass | ± da | ± ppm | Start Seq. | End Seq. | Sequence | Ion Score | C. I. % | Modification | Rank | Result Type |
|---|---|---|---|---|---|---|---|---|---|---|---|
| 869.4951 | 869.5105 | 0.0154 | 18 | 398 | 404 | RNPEVVR | | | | | Mascot |
| 869.4951 | 869.5105 | 0.0154 | 18 | 398 | 404 | RNPEVVR | 15 | 0 | | | Mascot |
| 892.5137 | 892.4758 | -0.0379 | -42 | 173 | 179 | IKVEFEK | | | | | Mascot |
| 892.5137 | 892.4758 | -0.0379 | -42 | 173 | 179 | IKVEFEK | | | | | Mascot |
| 960.4277 | 960.4583 | 0.0306 | 32 | 473 | 479 | KMSFMER | | | Oxidation (M)[2,5] | | Mascot |
| 960.4277 | 960.4583 | 0.0306 | 32 | 473 | 479 | KMSFMER | | | Oxidation (M)[2,5] | | Mascot |
| 1341.6984 | 1341.761 | 0.0626 | 47 | 350 | 360 | HWSLGVCLELK | | | Carbamidomethyl (C)[7] | | Mascot |
| 1341.6984 | 1341.761 | 0.0626 | 47 | 350 | 360 | HWSLGVCLELK | | | Carbamidomethyl (C)[7] | | Mascot |
| 1403.6559 | 1403.6858 | 0.0299 | 21 | 18 | 30 | DPVLLGCGHSFCR | | | | | Mascot |
| 1558.7128 | 1558.8331 | 0.1203 | 77 | 405 | 417 | VKLDYVCDDTMEK | | | | | Mascot |
| 1772.783 | 1772.8203 | 0.0373 | 21 | 2 | 17 | SLEDDLSCAVCTDVFR | | | | | Mascot |
| 1829.8044 | 1829.9308 | 0.1264 | 69 | 2 | 17 | SLEDDLSCAVCTDVFR | | | Carbamidomethyl (C)[8] | | Mascot |
| 2225.0259 | 2225.084 | 0.0581 | 26 | 95 | 111 | IELFCQNDEQLLCARCR | | | Carbamidomethyl (C)[5,13,16] | | Mascot |
| 2282.9851 | 2283.1169 | 0.1318 | 58 | 31 | 49 | QCIYDHWSSSGTRNCPIC R | | | Carbamidomethyl (C)[2] | | Mascot |

23 PREDICTED: protein NLRC3-like [Danio rerio] gi|326667475 86681.6 6.22 12 37 0

Peptide Information

| Calc. Mass | Obsrv. Mass | ± da | ± ppm | Start Seq. | End Seq. | Sequence | Ion Score | C. I. % | Modification | Rank | Result Type |
|---|---|---|---|---|---|---|---|---|---|---|---|
| 892.4193 | 892.4758 | 0.0565 | 63 | 740 | 746 | SDECLIR | | | Carbamidomethyl (C)[4] | | Mascot |
| 892.4193 | 892.4758 | 0.0565 | 63 | 740 | 746 | SDECLIR | | | Carbamidomethyl (C)[4] | | Mascot |

| | | | | | | | | | | | |
|---|---|---|---|---|---|---|---|---|---|---|---|
| 902.44 | 902.5221 | 0.0821 | 91 | 395 | 402 | IPADCIDR | | | | | Mascot |
| 960.5472 | 960.4583 | -0.0889 | -93 | 45 | 53 | TPSSVKSVR | | | | | Mascot |
| 960.5472 | 960.4583 | -0.0889 | -93 | 45 | 53 | TPSSVKSVR | | | | | Mascot |
| 990.5366 | 990.4245 | -0.1121 | -113 | 520 | 527 | LAFQQLDR | | | | | Mascot |
| 1045.5272 | 1045.5522 | 0.025 | 24 | 645 | 653 | GLLTQQDDR | | | | | Mascot |
| 1320.614 | 1320.6573 | 0.0433 | 33 | 69 | 80 | SMGYPPPDLSEK | | | | | Mascot |
| 1341.6685 | 1341.761 | 0.0925 | 69 | 528 | 538 | NNVIFYESDLK | | | | | Mascot |
| 1341.6685 | 1341.761 | 0.0925 | 69 | 528 | 538 | NNVIFYESDLK | | | | | Mascot |
| 1678.7742 | 1678.85 | 0.0758 | 45 | 66 | 80 | SDRSMGYPPPDLSEK | | | | | Mascot |
| 1710.8486 | 1710.8761 | 0.0275 | 16 | 285 | 298 | ENQDISFTFPLPFR | | | | | Mascot |
| 1710.8486 | 1710.8761 | 0.0275 | 16 | 285 | 298 | ENQDISFTFPLPFR | | | | | Mascot |
| 1724.9039 | 1724.8828 | -0.0211 | -12 | 332 | 345 | FKVLFILDGLDECR | | | Carbamidomethyl (C)[13] | | Mascot |
| 2211.2092 | 2211.0767 | -0.1325 | -60 | 143 | 161 | SIITEAALDLTLFFLREMK | | | | | Mascot |
| 2298.1812 | 2298.0984 | -0.0828 | -36 | 479 | 496 | TLTQMYTHFLRFQIQQSR | | | | | Mascot |

| | | | | | | | | |
|---|---|---|---|---|---|---|---|---|
| 24 | PREDICTED: retrotransposable element Tf2 155 kDa protein type 1-like [Danio rerio] | gi\|326674161 | 152950.2 | 9.44 | 16 | 37 | 0 | |

**Peptide Information**

| Calc. Mass | Obsrv. Mass | ± da | ± ppm | Start Seq. | End Seq. | Sequence | Ion Score | C. I. % | Modification | Rank | Result Type |
|---|---|---|---|---|---|---|---|---|---|---|---|
| 882.4567 | 882.5579 | 0.1012 | 115 | 183 | 190 | SVADYSIK | | | | | Mascot |
| 960.5472 | 960.4583 | -0.0889 | -93 | 285 | 292 | SRLSLQEK | | | | | Mascot |
| 960.5472 | 960.4583 | -0.0889 | -93 | 285 | 292 | SRLSLQEK | | | | | Mascot |
| 982.4886 | 982.4392 | -0.0494 | -50 | 1012 | 1020 | VACHPGVSR | | | Carbamidomethyl (C)[3] | | Mascot |
| 998.5305 | 998.4122 | -0.1183 | -118 | 605 | 613 | LQGASFFTK | | | | | Mascot |
| 1118.6125 | 1118.6117 | -0.0008 | -1 | 327 | 336 | LLMGEISLDK | | | | | Mascot |
| 1131.6091 | 1131.5668 | -0.0423 | -37 | 618 | 626 | NAYHLVRMK | | | | | Mascot |
| 1341.6104 | 1341.761 | 0.1506 | 112 | 471 | 482 | SAVSCSVFQEER | | | | | Mascot |
| 1341.6104 | 1341.761 | 0.1506 | 112 | 471 | 482 | SAVSCSVFQEER | | | | | Mascot |
| 1385.7939 | 1385.6997 | -0.0942 | -68 | 1099 | 1110 | FSKATHFIPLPK | | | | | Mascot |
| 1385.7939 | 1385.6997 | -0.0942 | -68 | 1099 | 1110 | FSKATHFIPLPK | | | | | Mascot |
| 1403.6334 | 1403.6858 | 0.0524 | 37 | 144 | 155 | LPCCSSFDLFSK | | | Carbamidomethyl (C)[3] | | Mascot |
| 1607.7859 | 1607.7588 | -0.0271 | -17 | 1006 | 1020 | WGHSSKVACHPGVSR | | | | | Mascot |
| 1623.7935 | 1623.7515 | -0.042 | -26 | 483 | 496 | MDLSNVPSEYLDLK | | | | | Mascot |
| 1623.7935 | 1623.7515 | -0.042 | -26 | 483 | 496 | MDLSNVPSEYLDLK | | | | | Mascot |
| 1653.7876 | 1653.6389 | -0.1487 | -90 | 83 | 97 | LPPPTCYAGEPHLCR | | | | | Mascot |
| 1665.8516 | 1665.8595 | 0.0079 | 5 | 530 | 543 | LYSLSIPEREAMEK | | | | | Mascot |

| | | | | | | | | | | | |
|---|---|---|---|---|---|---|---|---|---|---|---|
| 1710.8091 | 1710.8761 | 0.067 | 39 | 83 | 97 | LPPPTCYAGEPHLCR | | | Carbamidomethyl (C)[6] | | Mascot |
| 1710.8091 | 1710.8761 | 0.067 | 39 | 83 | 97 | LPPPTCYAGEPHLCR | | | Carbamidomethyl (C)[6] | | Mascot |
| 1829.85 | 1829.9308 | 0.0808 | 44 | 840 | 855 | AASDDRIHPCAFFSHR | | | | | Mascot |
| 1847.9609 | 1847.8706 | -0.0903 | -49 | 935 | 950 | NIKPDALSRLFDSSER | | | | | Mascot |
| 1864.0902 | 1863.8599 | -0.2303 | -124 | 393 | 410 | ALSGLSLPTITHSTKPIK | | | | | Mascot |

25 PREDICTED: myosin-10-like [Danio rerio] gi|292622124 232610.3 5.52 17 37 0

Peptide Information

| Calc. Mass | Obsrv. Mass | ± da | ± ppm | Start Seq. | End Seq. | Sequence | Ion Score | C. I. % | Modification | Rank | Result Type |
|---|---|---|---|---|---|---|---|---|---|---|---|
| 864.4321 | 864.4897 | 0.0576 | 67 | 1663 | 1669 | GREEAFR | | | | | Mascot |
| 902.524 | 902.5221 | -0.0019 | -2 | 1862 | 1869 | AMANKLVR | | | | | Mascot |
| 960.493 | 960.4583 | -0.0347 | -36 | 725 | 733 | CNGVLEGIR | 15 | 0 | | | Mascot |
| 960.493 | 960.4583 | -0.0347 | -36 | 725 | 733 | CNGVLEGIR | | | | | Mascot |
| 976.5032 | 976.4554 | -0.0478 | -49 | 86 | 93 | MNPPRFSK | | | | | Mascot |
| 1045.5272 | 1045.5522 | 0.025 | 24 | 1515 | 1523 | ALQEATEQR | | | | | Mascot |
| 1051.5717 | 1051.7004 | 0.1287 | 122 | 846 | 854 | NCAAYLKLR | | | | | Mascot |
| 1075.6194 | 1075.6029 | -0.0165 | -15 | 583 | 590 | FMRPRQLK | | | | | Mascot |
| 1148.5979 | 1148.5958 | -0.0021 | -2 | 1825 | 1835 | LSEMEGAVKGK | | | | | Mascot |
| 1403.6947 | 1403.6858 | -0.0089 | -6 | 1877 | 1887 | EVLMQVEDERR | | | | | Mascot |
| 1619.8024 | 1619.8684 | 0.066 | 41 | 568 | 582 | SFVDKLSAEQGSHSK | | | | | Mascot |
| 1623.7795 | 1623.7515 | -0.028 | -17 | 1477 | 1490 | FDQCLAEEKAVSAR | 4 | 0 | Carbamidomethyl (C)[4] | | Mascot |
| 1623.7795 | 1623.7515 | -0.028 | -17 | 1477 | 1490 | FDQCLAEEKAVSAR | | | Carbamidomethyl (C)[4] | | Mascot |
| 1748.8483 | 1748.8676 | 0.0193 | 11 | 1451 | 1464 | EEIEDMTIALQRER | | | Oxidation (M)[6] | | Mascot |
| 1772.9442 | 1772.8203 | -0.1239 | -70 | 792 | 806 | VFFRAGVLAHLEEER | | | | | Mascot |
| 1819.8304 | 1819.8468 | 0.0164 | 9 | 59 | 74 | EERGDEVEVELTDSGR | | | | | Mascot |
| 1819.8304 | 1819.8468 | 0.0164 | 9 | 59 | 74 | EERGDEVEVELTDSGR | | | | | Mascot |
| 2225.0173 | 2225.084 | 0.0667 | 30 | 171 | 191 | SMLQDREDQSILCTGESGAGK | | | | | Mascot |
| 2249.2512 | 2249.0305 | -0.2207 | -98 | 198 | 218 | VIQYLAHVASSHKSGTLGRPK | | | | | Mascot |
| 2298.0337 | 2298.0984 | 0.0647 | 28 | 171 | 191 | SMLQDREDQSILCTGESGAGK | | | Carbamidomethyl (C)[13], Oxidation (M)[2] | | Mascot |
| 2300.1008 | 2300.115 | 0.0142 | 6 | 94 | 113 | VEDMADLTCLNEASVLHNLR | | | Carbamidomethyl (C)[9] | | Mascot |

26 novel protein [Danio rerio] gi|123230013 19821.7 8.29 6 36 0

Peptide Information

| Calc. Mass | Obsrv. Mass | ± da | ± ppm | Start Seq. | End Seq. | Sequence | Ion Score | C. I. % | Modification | Rank | Result Type |
|---|---|---|---|---|---|---|---|---|---|---|---|

| Calc. Mass | Obsrv. Mass | ± da | ± ppm | Start Seq. | End Seq. | Sequence | Ion Score | C. I. % | Modification | Rank | Result Type |
|---|---|---|---|---|---|---|---|---|---|---|---|
| 1102.6102 | 1102.6154 | 0.0052 | 5 | 60 | 70 | AGTDIDAALKK | | | | | Mascot |
| 1541.9414 | 1541.775 | -0.1664 | -108 | 161 | 174 | TIANLPIIGTFLIR | | | | | Mascot |
| 1748.7982 | 1748.8676 | 0.0694 | 40 | 105 | 118 | MNTLTVDCLFFDTR | | | Carbamidomethyl (C)[8], Oxidation (M)[1] | | Mascot |
| 1825.8823 | 1825.7393 | -0.143 | -78 | 122 | 137 | CNINDEALDKIVTCFK | | | | | Mascot |
| 1847.8778 | 1847.8706 | -0.0072 | -4 | 104 | 118 | RMNTLTVDCLFFDTR | | | Oxidation (M)[2] | | Mascot |
| 2555.4531 | 2555.322 | -0.1311 | -51 | 132 | 153 | IVTCFKHQTIANLPIIGTFLIR | | | Carbamidomethyl (C)[4] | | Mascot |
| 2555.4531 | 2555.322 | -0.1311 | -51 | 132 | 153 | IVTCFKHQTIANLPIIGTFLIR | | | Carbamidomethyl (C)[4] | | Mascot |

| | | | | | | | | | |
|---|---|---|---|---|---|---|---|---|---|
| 27 | PREDICTED: phosphatase and actin regulator 2 [Danio rerio] | gi\|125833212 | 58430.7 | 7.1 | 8 | 36 | 0 | 10 | 0 |

**Peptide Information**

| Calc. Mass | Obsrv. Mass | ± da | ± ppm | Start Seq. | End Seq. | Sequence | Ion Score | C. I. % | Modification | Rank | Result Type |
|---|---|---|---|---|---|---|---|---|---|---|---|
| 990.4486 | 990.4245 | -0.0241 | -24 | 295 | 303 | DAGKEEGER | | | | | Mascot |
| 1148.6157 | 1148.5958 | -0.0199 | -17 | 217 | 226 | KELTTSVNEK | | | | | Mascot |
| 1385.7495 | 1385.6997 | -0.0498 | -36 | 190 | 201 | TEPSRSQRPLSK | | | | | Mascot |
| 1385.7495 | 1385.6997 | -0.0498 | -36 | 190 | 201 | TEPSRSQRPLSK | | | | | Mascot |
| 1403.8409 | 1403.6858 | -0.1551 | -110 | 43 | 54 | LSSLGKIFKPWK | | | | | Mascot |
| 1665.7537 | 1665.8595 | 0.1058 | 64 | 504 | 516 | EMEVHEESKQFTR | | | Oxidation (M)[2] | | Mascot |
| 1710.9061 | 1710.8761 | -0.03 | -18 | 459 | 472 | ILRFNEYVEVTEAK | | | | | Mascot |
| 1710.9061 | 1710.8761 | -0.03 | -18 | 459 | 472 | ILRFNEYVEVTEAK | | | | | Mascot |
| 1819.8855 | 1819.8468 | -0.0387 | -21 | 21 | 38 | SSLANCDVVGSSQSPQLK | | | | | Mascot |
| 1819.8855 | 1819.8468 | -0.0387 | -21 | 21 | 38 | SSLANCDVVGSSQSPQLK | 10 | 0 | | | Mascot |
| 1829.8472 | 1829.9308 | 0.0836 | 46 | 156 | 174 | GSSQASGQSSSQTSSKTSK | | | | | Mascot |

| | | | | | | | | | |
|---|---|---|---|---|---|---|---|---|---|
| 28 | Flk1b [Danio rerio] | gi\|56548636 | 51362.5 | 6.09 | 5 | 36 | 0 | 28 | 55.754 |

**Peptide Information**

| Calc. Mass | Obsrv. Mass | ± da | ± ppm | Start Seq. | End Seq. | Sequence | Ion Score | C. I. % | Modification | Rank | Result Type |
|---|---|---|---|---|---|---|---|---|---|---|---|
| 869.5203 | 869.5105 | -0.0098 | -11 | 388 | 395 | GDARLPLK | | | | | Mascot |
| 869.5203 | 869.5105 | -0.0098 | -11 | 388 | 395 | GDARLPLK | 17 | 0 | | | Mascot |
| 892.4523 | 892.4758 | 0.0235 | 26 | 381 | 387 | DPDYVRK | | | | | Mascot |
| 892.4523 | 892.4758 | 0.0235 | 26 | 381 | 387 | DPDYVRK | | | | | Mascot |
| 926.4399 | 926.5717 | 0.1318 | 142 | 340 | 347 | GMEFLASR | | | Oxidation (M)[2] | | Mascot |
| 1051.5453 | 1051.7004 | 0.1551 | 147 | 182 | 191 | ATTCTTVAVK | | | Carbamidomethyl (C)[4] | | Mascot |
| 1341.6644 | 1341.761 | 0.0966 | 72 | 192 | 203 | XLKEGATSSEYR | 11 | 0 | | | Mascot |

29 PREDICTED: gelsolin-like [Danio rerio] gi|326668568 79933.9 6.95 10 35 0

Peptide Information

| Calc. Mass | Obsrv. Mass | ± da | ± ppm | Start Seq. | End Seq. | Sequence | Ion Score | C. I. % | Modification | Rank | Result Type |
|---|---|---|---|---|---|---|---|---|---|---|---|
| 869.4363 | 869.5105 | 0.0742 | 85 | 593 | 599 | KDYQTSK | | | | | Mascot |
| 869.4363 | 869.5105 | 0.0742 | 85 | 593 | 599 | KDYQTSK | | | | | Mascot |
| 892.4808 | 892.4758 | -0.005 | -6 | 21 | 27 | IEKMDLK | | | Oxidation (M)[4] | | Mascot |
| 892.4808 | 892.4758 | -0.005 | -6 | 21 | 27 | IEKMDLK | 6 | 0 | Oxidation (M)[4] | | Mascot |
| 998.5417 | 998.4122 | -0.1295 | -130 | 13 | 20 | EPGLQIWR | | | | | Mascot |
| 1148.5042 | 1148.5958 | 0.0916 | 80 | 707 | 715 | MWDTDPLDR | | | | | Mascot |
| 1701.8629 | 1701.8429 | -0.02 | -12 | 470 | 484 | VTQGQEPAHLMSLFK | | | Oxidation (M)[11] | | Mascot |
| 1772.9078 | 1772.8203 | -0.0875 | -49 | 6 | 20 | EFQNAGKEPGLQIWR | | | | | Mascot |
| 2233.1533 | 2233.0576 | -0.0957 | -43 | 522 | 541 | AVEVEPCASKLNTNDVFVLK | | | Carbamidomethyl (C)[7] | | Mascot |
| 2283.0823 | 2283.1169 | 0.0346 | 15 | 111 | 131 | YKQGGVSSGFNHVVSNEMNTK | | | | | Mascot |
| 2298.199 | 2298.0984 | -0.1006 | -44 | 532 | 550 | LNTNDVFVLKFPEGMFLWK | | | | | Mascot |
| 2555.1694 | 2555.322 | 0.1526 | 60 | 144 | 166 | ATEVNMSWASFNHGDCFILDLGK | | | | | Mascot |
| 2555.1694 | 2555.322 | 0.1526 | 60 | 144 | 166 | ATEVNMSWASFNHGDCFILDLGK | | | | | Mascot |

30 cullin 1-like [Danio rerio] gi|47087291 89546.8 8.18 14 35 0

Peptide Information

| Calc. Mass | Obsrv. Mass | ± da | ± ppm | Start Seq. | End Seq. | Sequence | Ion Score | C. I. % | Modification | Rank | Result Type |
|---|---|---|---|---|---|---|---|---|---|---|---|
| 864.4825 | 864.4897 | 0.0072 | 8 | 220 | 227 | GPTLSVYK | | | | | Mascot |
| 882.4501 | 882.5579 | 0.1078 | 122 | 320 | 326 | MYNLVSR | | | | | Mascot |
| 982.572 | 982.4392 | -0.1328 | -135 | 669 | 676 | LFLGYKNK | | | | | Mascot |
| 996.6564 | 996.5897 | -0.0667 | -67 | 707 | 715 | LLIQAAIVR | | | | | Mascot |
| 1051.5531 | 1051.7004 | 0.1473 | 140 | 26 | 34 | SGIQQVYTR | | | | | Mascot |
| 1073.6201 | 1073.4951 | -0.125 | -116 | 327 | 336 | ITDGLGELKK | | | | | Mascot |
| 1131.6442 | 1131.5668 | -0.0774 | -68 | 749 | 757 | KCIDILIEK | | | Carbamidomethyl (C)[2] | | Mascot |
| 1341.8099 | 1341.761 | -0.0489 | -36 | 176 | 187 | QVTNAVLKLVEK | | | | | Mascot |
| 1341.8099 | 1341.761 | -0.0489 | -36 | 176 | 187 | QVTNAVLKLVEK | | | | | Mascot |
| 1385.6709 | 1385.6997 | 0.0288 | 21 | 564 | 575 | FTAFYGSRHSGR | | | | | Mascot |
| 1385.6709 | 1385.6997 | 0.0288 | 21 | 564 | 575 | FTAFYGSRHSGR | | | | | Mascot |
| 1607.8462 | 1607.7588 | -0.0874 | -54 | 416 | 429 | SPELLARYCDSLLK | | | | | Mascot |
| 1711.0728 | 1710.8761 | -0.1967 | -115 | 631 | 645 | TDILVQVLQILLKSK | | | | | Mascot |

| | | | | | | | | | | | |
|---|---|---|---|---|---|---|---|---|---|---|---|
| 1711.0728 | 1710.8761 | -0.1967 | -115 | 631 | 645 | TDILVQVLQILLKSK | | | | | Mascot |
| 1772.9653 | 1772.8203 | -0.145 | -82 | 337 | 352 | LLESHIHNQGLAAIEK | | | | | Mascot |
| 1833.9857 | 1833.9143 | -0.0714 | -39 | 73 | 89 | TPTPGGAQFVGLELYKR | | | | | Mascot |
| 2283.1423 | 2283.1169 | -0.0254 | -11 | 96 | 115 | SYLTNLLKDGEDLMDESVLK | | | | | Mascot |

| | | | | | | | | | | |
|---|---|---|---|---|---|---|---|---|---|---|
| 31 | calbindin 2 [Danio rerio] | gi\|239596164 | 15235.8 | 5.73 | 3 | 35 | 0 | 23 | 0 | |

Peptide Information

| Calc. Mass | Obsrv. Mass | ± da | ± ppm | Start Seq. | End Seq. | Sequence | Ion Score | C. I. % | Modification | Rank | Result Type |
|---|---|---|---|---|---|---|---|---|---|---|---|
| 892.4556 | 892.4758 | 0.0202 | 23 | 32 | 39 | LGLSEMAR | | | Oxidation (M)[6] | | Mascot |
| 892.4556 | 892.4758 | 0.0202 | 23 | 32 | 39 | LGLSEMAR | 23 | 0 | Oxidation (M)[6] | | Mascot |
| 996.4454 | 996.5897 | 0.1443 | 145 | 23 | 31 | MFDLNGDGK | | | | | Mascot |
| 1829.8665 | 1829.9308 | 0.0643 | 35 | 71 | 86 | DGNGYIDEHELDALLR | | | | | Mascot |

| | | | | | | | | | | |
|---|---|---|---|---|---|---|---|---|---|---|
| 32 | PREDICTED: vigilin isoform 2 [Danio rerio] | gi\|292619547 | 142099.2 | 6.53 | 14 | 34 | 0 | 12 | 0 | |

Peptide Information

| Calc. Mass | Obsrv. Mass | ± da | ± ppm | Start Seq. | End Seq. | Sequence | Ion Score | C. I. % | Modification | Rank | Result Type |
|---|---|---|---|---|---|---|---|---|---|---|---|
| 882.4679 | 882.5579 | 0.09 | 102 | 899 | 905 | EHEVQIK | | | | | Mascot |
| 960.5988 | 960.4583 | -0.1405 | -146 | 1220 | 1228 | ALAKGFVVR | | | | | Mascot |
| 960.5988 | 960.4583 | -0.1405 | -146 | 1220 | 1228 | ALAKGFVVR | | | | | Mascot |
| 976.488 | 976.4554 | -0.0326 | -33 | 950 | 957 | AEKCELAR | | | Carbamidomethyl (C)[4] | | Mascot |
| 990.5214 | 990.4245 | -0.0969 | -98 | 604 | 611 | IREETNTK | | | | | Mascot |
| 1045.5095 | 1045.5522 | 0.0427 | 41 | 848 | 857 | GPRECVDAAK | | | | | Mascot |
| 1075.5994 | 1075.6029 | 0.0035 | 3 | 195 | 204 | ITGTKEGIEK | | | | | Mascot |
| 1102.531 | 1102.6154 | 0.0844 | 77 | 848 | 857 | GPRECVDAAK | | | Carbamidomethyl (C)[5] | | Mascot |
| 1131.6051 | 1131.5668 | -0.0383 | -34 | 890 | 898 | GCRIQQITR | | | Carbamidomethyl (C)[2] | | Mascot |
| 1148.598 | 1148.5958 | -0.0022 | -2 | 119 | 129 | DQGLSIMVTGK | | | | | Mascot |
| 1623.8013 | 1623.7515 | -0.0498 | -31 | 822 | 836 | ELAEEYGGVAVSFPR | 12 | 0 | | | Mascot |
| 1623.8013 | 1623.7515 | -0.0498 | -31 | 822 | 836 | ELAEEYGGVAVSFPR | | | | | Mascot |
| 1637.8606 | 1637.6448 | -0.2158 | -132 | 205 | 218 | ARHEIQLISAEQDK | | | | | Mascot |
| 1665.7496 | 1665.8595 | 0.1099 | 66 | 1208 | 1223 | TMGTGGNDEDNRALAK | | | Oxidation (M)[2] | | Mascot |
| 1701.921 | 1701.8429 | -0.0781 | -46 | 534 | 547 | FPEVIIIFPDQQQK | | | | | Mascot |
| 1724.933 | 1724.8828 | -0.0502 | -29 | 70 | 84 | ASVITQVFHVPLEER | | | | | Mascot |
| 2298.0376 | 2298.0984 | 0.0608 | 26 | 88 | 107 | DNSQFGEGEEAKVCLDIMQK | | | Carbamidomethyl (C)[14] | | Mascot |

| | | | | | | | | | | |
|---|---|---|---|---|---|---|---|---|---|---|
| 33 | PREDICTED: neurobeachin-like [Danio rerio] | gi\|189528934 | 320583.8 | 5.86 | 13 | 34 | 0 | 23 | 0 | |

Peptide Information

| Calc. Mass | Obsrv. Mass | ± da | ± ppm | Start Seq. | End Seq. | Sequence | Ion Score | C. I. % | Modification | Rank | Result Type |
|---|---|---|---|---|---|---|---|---|---|---|---|
| 856.4046 | 856.5146 | 0.11 | 128 | 2640 | 2647 | VYSSDTGK | | | | | Mascot |
| 960.5009 | 960.4583 | -0.0426 | -44 | 329 | 335 | WRNSEIR | 23 | 0 | | | Mascot |
| 960.5009 | 960.4583 | -0.0426 | -44 | 329 | 335 | WRNSEIR | | | | | Mascot |
| 976.5066 | 976.4554 | -0.0512 | -52 | 1445 | 1452 | LVCCVAVR | | | Carbamidomethyl (C)[3,4] | | Mascot |
| 982.5064 | 982.4392 | -0.0672 | -68 | 1925 | 1932 | QRAEDVHK | | | | | Mascot |
| 1320.6754 | 1320.6573 | -0.0181 | -14 | 1663 | 1675 | TASSISSINQANK | | | | | Mascot |
| 1444.7754 | 1444.7188 | -0.0566 | -39 | 142 | 154 | NLQTSSEVGLIQR | | | | | Mascot |
| 1541.8395 | 1541.775 | -0.0645 | -42 | 1250 | 1263 | KIQTTATTQSLHGR | | | | | Mascot |
| 1558.7834 | 1558.8331 | 0.0497 | 32 | 2143 | 2154 | WMFSEIRAVFTR | | | Oxidation (M)[2] | | Mascot |
| 1623.8523 | 1623.7515 | -0.1008 | -62 | 2737 | 2751 | EGPCLVHTITGDLLR | 3 | 0 | | | Mascot |
| 1623.8523 | 1623.7515 | -0.1008 | -62 | 2737 | 2751 | EGPCLVHTITGDLLR | | | | | Mascot |
| 1637.7743 | 1637.6448 | -0.1295 | -79 | 1445 | 1458 | LVCCVAVRNCLECR | | | Carbamidomethyl (C)[3] | | Mascot |
| 1724.894 | 1724.8828 | -0.0112 | -6 | 316 | 328 | KWYMVSIVHIYNR | | | Oxidation (M)[4] | | Mascot |
| 1825.7819 | 1825.7393 | -0.0426 | -23 | 1712 | 1726 | EAQAMMPMQFHSFDR | | | | | Mascot |
| 1829.8759 | 1829.9308 | 0.0549 | 30 | 243 | 256 | WPYQNGFTLNTWFR | | | | | Mascot |
| 1841.7767 | 1841.7614 | -0.0153 | -8 | 1712 | 1726 | EAQAMMPMQFHSFDR | | | Oxidation (M)[5] | | Mascot |

| | | | | | | | | |
|---|---|---|---|---|---|---|---|---|
| 34 | PREDICTED: myosin heavy chain, fast skeletal muscle-like [Danio rerio] | gi\|326678010 | 161130.5 | 6.2 | 16 | 34 | 0 | |

Peptide Information

| Calc. Mass | Obsrv. Mass | ± da | ± ppm | Start Seq. | End Seq. | Sequence | Ion Score | C. I. % | Modification | Rank | Result Type |
|---|---|---|---|---|---|---|---|---|---|---|---|
| 856.4886 | 856.5146 | 0.026 | 30 | 206 | 213 | KQEPVAGK | | | | | Mascot |
| 864.4495 | 864.4897 | 0.0402 | 47 | 862 | 868 | MKEDLTK | | | | | Mascot |
| 926.5053 | 926.5717 | 0.0664 | 72 | 972 | 979 | HATENKVK | | | | | Mascot |
| 960.4818 | 960.4583 | -0.0235 | -24 | 675 | 682 | CLIPNESK | | | Carbamidomethyl (C)[1] | | Mascot |
| 960.493 | 960.4583 | -0.0347 | -36 | 698 | 706 | CNGVLEGIR | 15 | 0 | | | Mascot |
| 1341.6025 | 1341.761 | 0.1585 | 118 | 845 | 856 | SAETEKEMAAMK | | | Oxidation (M)[8] | | Mascot |
| 1341.6025 | 1341.761 | 0.1585 | 118 | 845 | 856 | SAETEKEMAAMK | | | Oxidation (M)[8] | | Mascot |
| 1385.6115 | 1385.6997 | 0.0882 | 64 | 1375 | 1385 | VQHEMEEAQER | | | | | Mascot |
| 1385.6115 | 1385.6997 | 0.0882 | 64 | 1375 | 1385 | VQHEMEEAQER | | | | | Mascot |
| 1558.7933 | 1558.8331 | 0.0398 | 26 | 191 | 205 | VIQYFATVGAMSGAK | | | Oxidation (M)[11] | | Mascot |
| 1607.858 | 1607.7588 | -0.0992 | -62 | 130 | 143 | WLPVYDAVVVAGYR | | | | | Mascot |
| 1665.7637 | 1665.8595 | 0.0958 | 58 | 980 | 994 | NLTEEMASQDESIAK | | | | | Mascot |

| Calc. Mass | Obsrv. Mass | ± da | ± ppm | Start Seq. | End Seq. | Sequence | Ion Score | C. I. % | Modification | Rank | Result Type |
|---|---|---|---|---|---|---|---|---|---|---|---|
| 1701.8855 | 1701.8429 | -0.0426 | -25 | 432 | 444 | SVYEKMFLWMVIR | | | | | Mascot |
| 1710.9247 | 1710.8761 | -0.0486 | -28 | 766 | 780 | VFFKAGLLGTLEEMR | | | | | Mascot |
| 1710.9247 | 1710.8761 | -0.0486 | -28 | 766 | 780 | VFFKAGLLGTLEEMR | | | | | Mascot |
| 1724.9014 | 1724.8828 | -0.0186 | -11 | 661 | 674 | LMTNLRSTHPHFVR | | | Oxidation (M)[2] | | Mascot |
| 1819.8352 | 1819.8468 | 0.0116 | 6 | 1266 | 1280 | SDENIRQLNDMNAQR | | | Oxidation (M)[11] | | Mascot |
| 1819.8352 | 1819.8468 | 0.0116 | 6 | 1266 | 1280 | SDENIRQLNDMNAQR | | | Oxidation (M)[11] | | Mascot |
| 2035.0858 | 2034.8927 | -0.1931 | -95 | 725 | 742 | YKVLNASVIPEGQFIDNK | | | | | Mascot |
| 2035.0858 | 2034.8927 | -0.1931 | -95 | 725 | 742 | YKVLNASVIPEGQFIDNK | | | | | Mascot |
| 2300.1599 | 2300.115 | -0.0449 | -20 | 386 | 405 | IAYLMGLNSADMLKALCYPR | | | Carbamidomethyl (C)[17] | | Mascot |

35 glutamine synthetase [Danio rerio] gi|225579106 41854.1 5.98 3 34 0 26 24.512

**Peptide Information**

| Calc. Mass | Obsrv. Mass | ± da | ± ppm | Start Seq. | End Seq. | Sequence | Ion Score | C. I. % | Modification | Rank | Result Type |
|---|---|---|---|---|---|---|---|---|---|---|---|
| 869.5315 | 869.5105 | -0.021 | -24 | 320 | 327 | GASIRIPR | | | | | Mascot |
| 869.5315 | 869.5105 | -0.021 | -24 | 320 | 327 | GASIRIPR | 26 | 24.512 | | | Mascot |
| 902.5379 | 902.5221 | -0.0158 | -18 | 96 | 103 | LVLCDVLK | | | | | Mascot |
| 2283.2278 | 2283.1169 | -0.1109 | -49 | 96 | 114 | LVLCDVLKYNHKPAETNLR | | | Carbamidomethyl (C)[4] | | Mascot |

36 dehydrogenase/reductase SDR family member 1 [Danio rerio] gi|50345068 33868.3 8.39 7 34 0

**Peptide Information**

| Calc. Mass | Obsrv. Mass | ± da | ± ppm | Start Seq. | End Seq. | Sequence | Ion Score | C. I. % | Modification | Rank | Result Type |
|---|---|---|---|---|---|---|---|---|---|---|---|
| 1075.5815 | 1075.6029 | 0.0214 | 20 | 232 | 240 | CIVELAKDK | | | Carbamidomethyl (C)[1] | | Mascot |
| 1320.6399 | 1320.6573 | 0.0174 | 13 | 53 | 65 | GGRCLPVVCDSSK | | | | | Mascot |
| 1607.8032 | 1607.7588 | -0.0444 | -28 | 1 | 15 | MALSGWICVVTGASR | | | Carbamidomethyl (C)[8] | | Mascot |
| 1623.7981 | 1623.7515 | -0.0466 | -29 | 1 | 15 | MALSGWICVVTGASR | 3 | 0 | Carbamidomethyl (C)[8], Oxidation (M)[1] | | Mascot |
| 1623.7981 | 1623.7515 | -0.0466 | -29 | 1 | 15 | MALSGWICVVTGASR | | | Carbamidomethyl (C)[8], Oxidation (M)[1] | | Mascot |
| 1701.7061 | 1701.8429 | 0.1368 | 80 | 201 | 215 | QYMSQDEGPPGFDSK | | | Oxidation (M)[3] | | Mascot |
| 1772.8788 | 1772.8203 | -0.0585 | -33 | 155 | 170 | YLFNVPYGVGKAACDR | | | | | Mascot |
| 1819.9912 | 1819.8468 | -0.1444 | -79 | 20 | 37 | GIALQLSEAGATVYITGR | | | | | Mascot |
| 1819.9912 | 1819.8468 | -0.1444 | -79 | 20 | 37 | GIALQLSEAGATVYITGR | | | | | Mascot |
| 1829.9003 | 1829.9308 | 0.0305 | 17 | 155 | 170 | YLFNVPYGVGKAACDR | | | Carbamidomethyl (C)[14] | | Mascot |
| 2555.4053 | 2555.322 | -0.0833 | -33 | 142 | 165 | GLIVVISSMGGLRYLFNVPYGVGK | | | Oxidation (M)[9] | | Mascot |
| 2555.4053 | 2555.322 | -0.0833 | -33 | 142 | 165 | GLIVVISSMGGLRYLFNVPYGVGK | | | Oxidation (M)[9] | | Mascot |

37 glutamate-ammonia ligase [Danio rerio] gi|37682111 42018.3 6.38 3 34 0 26 24.512

Peptide Information

| Calc. Mass | Obsrv. Mass | ± da | ± ppm | Start Seq. | End Seq. | Sequence | Ion Score | C. I. % | Modification | Rank | Result Type |
|---|---|---|---|---|---|---|---|---|---|---|---|
| 869.5315 | 869.5105 | -0.021 | -24 | 320 | 327 | GASIRIPR | | | | | Mascot |
| 869.5315 | 869.5105 | -0.021 | -24 | 320 | 327 | GASIRIPR | 26 | 24.512 | | | Mascot |
| 1444.7577 | 1444.7188 | -0.0389 | -27 | 1 | 14 | MATSASSHLNKGIK | | | | | Mascot |
| 2097.0256 | 2096.8096 | -0.216 | -103 | 26 | 43 | VQAMYIWIDGTGEGLRC K | | | Carbamidomethyl (C)[17] | | Mascot |

| | | | | | | | | | | | |
|---|---|---|---|---|---|---|---|---|---|---|---|
| 38 | truncated RAF1 [Danio rerio] | gi\|41224402 | 32290.4 | 9.5 | 8 | 33 | 0 | | | | |

Peptide Information

| Calc. Mass | Obsrv. Mass | ± da | ± ppm | Start Seq. | End Seq. | Sequence | Ion Score | C. I. % | Modification | Rank | Result Type |
|---|---|---|---|---|---|---|---|---|---|---|---|
| 882.4791 | 882.5579 | 0.0788 | 89 | 215 | 222 | IPSSSVHR | | | | | Mascot |
| 892.4669 | 892.4758 | 0.0089 | 10 | 208 | 214 | MRESLTR | | | | | Mascot |
| 892.4669 | 892.4758 | 0.0089 | 10 | 208 | 214 | MRESLTR | | | | | Mascot |
| 902.4148 | 902.5221 | 0.1073 | 119 | 33 | 40 | SPAPCQSR | | | Carbamidomethyl (C)[5] | | Mascot |
| 982.4435 | 982.4392 | -0.0043 | -4 | 41 | 49 | SSDDSKTSR | | | | | Mascot |
| 1045.452 | 1045.5522 | 0.1002 | 96 | 165 | 172 | FHEHCSTK | | | Carbamidomethyl (C)[5] | | Mascot |
| 1118.5411 | 1118.6117 | 0.0706 | 63 | 1 | 9 | MEQLQGAWR | | | | | Mascot |
| 1841.8785 | 1841.7614 | -0.1171 | -64 | 83 | 99 | GLQPECCAVFSLHPGQR | | | | | Mascot |
| 2097.0481 | 2096.8096 | -0.2385 | -114 | 81 | 99 | VRGLQPECCAVFSLHPG QR | | | | | Mascot |
| 2211.0908 | 2211.0767 | -0.0141 | -6 | 81 | 99 | VRGLQPECCAVFSLHPG QR | | | Carbamidomethyl (C)[8,9] | | Mascot |

| | | | | | | | | | | | |
|---|---|---|---|---|---|---|---|---|---|---|---|
| 39 | Wu:fj64b09 protein [Danio rerio] | gi\|32766289 | 81939.8 | 6.7 | 6 | 33 | 0 | 28 | 49.432 | | |

Peptide Information

| Calc. Mass | Obsrv. Mass | ± da | ± ppm | Start Seq. | End Seq. | Sequence | Ion Score | C. I. % | Modification | Rank | Result Type |
|---|---|---|---|---|---|---|---|---|---|---|---|
| 869.4297 | 869.5105 | 0.0808 | 93 | 54 | 60 | NYKVCSR | | | | | Mascot |
| 869.4297 | 869.5105 | 0.0808 | 93 | 54 | 60 | NYKVCSR | | | | | Mascot |
| 882.4501 | 882.5579 | 0.1078 | 122 | 312 | 318 | LKDFTCR | | | | | Mascot |
| 892.4556 | 892.4758 | 0.0202 | 23 | 565 | 572 | VLGTEMAR | | | Oxidation (M)[6] | | Mascot |
| 892.4556 | 892.4758 | 0.0202 | 23 | 565 | 572 | VLGTEMAR | 28 | 49.432 | Oxidation (M)[6] | | Mascot |
| 926.4512 | 926.5717 | 0.1205 | 130 | 54 | 60 | NYKVCSR | | | Carbamidomethyl (C)[5] | | Mascot |
| 1045.6041 | 1045.5522 | -0.0519 | -50 | 377 | 385 | ITTLYGHIK | | | | | Mascot |
| 1131.5575 | 1131.5668 | 0.0093 | 8 | 38 | 46 | CNRPDLQTK | | | Carbamidomethyl (C)[1] | | Mascot |

| | | | | | | | | | | | |
|---|---|---|---|---|---|---|---|---|---|---|---|
| 1665.7723 | 1665.8595 | 0.0872 | 52 | 61 | 74 | HFETSMICQQSAVK | | | Carbamidomethyl (C)[8] | | Mascot |

| | | | | | | | |
|---|---|---|---|---|---|---|---|
| 40 | PREDICTED: hypothetical protein LOC100536945 [Danio rerio] | gi\|326665516 | 19714 | 5.96 | 4 | 33 | 0 |

**Peptide Information**

| Calc. Mass | Obsrv. Mass | ± da | ± ppm | Start Seq. | End Seq. | Sequence | Ion Score | C. I. % | Modification | Rank | Result Type |
|---|---|---|---|---|---|---|---|---|---|---|---|
| 856.4159 | 856.5146 | 0.0987 | 115 | 25 | 32 | SHDSPSVK | | | | | Mascot |
| 960.5261 | 960.4583 | -0.0678 | -71 | 2 | 9 | TWLTGNIR | 13 | 0 | | | Mascot |
| 960.5261 | 960.4583 | -0.0678 | -71 | 2 | 9 | TWLTGNIR | | | | | Mascot |
| 1385.6842 | 1385.6997 | 0.0155 | 11 | 21 | 32 | ELMRSHDSPSVK | | | | | Mascot |
| 1385.6842 | 1385.6997 | 0.0155 | 11 | 21 | 32 | ELMRSHDSPSVK | | | | | Mascot |
| 1637.7887 | 1637.6448 | -0.1439 | -88 | 1 | 13 | MTWLTGNIRENMR | | | Oxidation (M)[1] | | Mascot |
| 1653.7836 | 1653.6389 | -0.1447 | -87 | 1 | 13 | MTWLTGNIRENMR | | | Oxidation (M)[1,12] | | Mascot |

| | | | | | | | |
|---|---|---|---|---|---|---|---|
| 41 | photoreceptor-specific nuclear receptor [Danio rerio] | gi\|55925261 | 46425.1 | 7.1 | 9 | 33 | 0 |

**Peptide Information**

| Calc. Mass | Obsrv. Mass | ± da | ± ppm | Start Seq. | End Seq. | Sequence | Ion Score | C. I. % | Modification | Rank | Result Type |
|---|---|---|---|---|---|---|---|---|---|---|---|
| 864.4066 | 864.4897 | 0.0831 | 96 | 100 | 107 | CLQAGMNK | | | | | Mascot |
| 869.4259 | 869.5105 | 0.0846 | 97 | 412 | 418 | LLCDMFK | | | | | Mascot |
| 869.4259 | 869.5105 | 0.0846 | 97 | 412 | 418 | LLCDMFK | | | | | Mascot |
| 926.4474 | 926.5717 | 0.1243 | 134 | 412 | 418 | LLCDMFK | | | Carbamidomethyl (C)[3] | | Mascot |
| 960.5546 | 960.4583 | -0.0963 | -100 | 32 | 40 | VLNTGLLCK | | | | | Mascot |
| 960.5546 | 960.4583 | -0.0963 | -100 | 32 | 40 | VLNTGLLCK | | | | | Mascot |
| 990.4924 | 990.4245 | -0.0679 | -69 | 403 | 411 | TIGNTPMEK | | | | | Mascot |
| 1049.5231 | 1049.5206 | -0.0025 | -2 | 99 | 107 | KCLQAGMNK | | | Carbamidomethyl (C)[2] | | Mascot |
| 1131.5892 | 1131.5668 | -0.0224 | -20 | 124 | 133 | LDALDVDKDK | | | | | Mascot |
| 1403.6368 | 1403.6858 | 0.049 | 35 | 182 | 194 | FMASLMTAETCAK | | | | | Mascot |
| 1558.6876 | 1558.8331 | 0.1455 | 93 | 1 | 14 | MSMVPFSSPTESSR | | | Oxidation (M)[1] | | Mascot |
| 1833.9453 | 1833.9143 | -0.031 | -17 | 302 | 318 | GSPSASDVRVLQEVFSR | | | | | Mascot |

| | | | | | | | |
|---|---|---|---|---|---|---|---|
| 42 | keratin 18 [Danio rerio] | gi\|29335504 | 41247.4 | 5.07 | 9 | 32 | 0 |

**Peptide Information**

| Calc. Mass | Obsrv. Mass | ± da | ± ppm | Start Seq. | End Seq. | Sequence | Ion Score | C. I. % | Modification | Rank | Result Type |
|---|---|---|---|---|---|---|---|---|---|---|---|
| 864.4461 | 864.4897 | 0.0436 | 50 | 315 | 322 | LLDGGDFK | | | | | Mascot |
| 882.4753 | 882.5579 | 0.0826 | 94 | 188 | 194 | YEKMALK | | | | | Mascot |

| Calc. Mass | Obsrv. Mass | ± da | ± ppm | Start Seq. | End Seq. | Sequence | Ion Score | C. I. % | Modification | Rank | Result Type |
|---|---|---|---|---|---|---|---|---|---|---|---|
| 902.4941 | 902.5221 | 0.028 | 31 | 32 | 39 | ILEQANSK | | | | | Mascot |
| 996.4996 | 996.5897 | 0.0901 | 90 | 100 | 107 | YESELSIR | | | | | Mascot |
| 1045.4697 | 1045.5522 | 0.0825 | 79 | 52 | 60 | GPDVHDYSR | | | | | Mascot |
| 1073.5474 | 1073.4951 | -0.0523 | -49 | 323 | 331 | LQDALEEQK | | | | | Mascot |
| 1385.7998 | 1385.6997 | -0.1001 | -72 | 32 | 43 | ILEQANSKLELK | | | | | Mascot |
| 1385.7998 | 1385.6997 | -0.1001 | -72 | 32 | 43 | ILEQANSKLELK | | | | | Mascot |
| 1558.8323 | 1558.8331 | 0.0008 | 1 | 234 | 246 | QIQTLEIELESQK | | | | | Mascot |
| 1829.9868 | 1829.9308 | -0.056 | -31 | 82 | 97 | LVLQIDNARLAADDFR | | | | | Mascot |

43 TBC1 domain family member 7 [Danio rerio] gi|41152120 33966.3 6.35 8 32 0

**Peptide Information**

| Calc. Mass | Obsrv. Mass | ± da | ± ppm | Start Seq. | End Seq. | Sequence | Ion Score | C. I. % | Modification | Rank | Result Type |
|---|---|---|---|---|---|---|---|---|---|---|---|
| 882.4832 | 882.5579 | 0.0747 | 85 | 279 | 285 | AIDLWHK | | | | | Mascot |
| 1118.5337 | 1118.6117 | 0.078 | 70 | 2 | 10 | ADDPQRNFR | | | | | Mascot |
| 1385.6995 | 1385.6997 | 0.0002 | 0 | 111 | 121 | MFQLENHTLPR | | | | | Mascot |
| 1385.6995 | 1385.6997 | 0.0002 | 0 | 111 | 121 | MFQLENHTLPR | | | | | Mascot |
| 1541.8005 | 1541.775 | -0.0255 | -17 | 111 | 122 | MFQLENHTLPRR | | | | | Mascot |
| 1623.7729 | 1623.7515 | -0.0214 | -13 | 210 | 223 | RCFAGCLPESSLQR | | | Carbamidomethyl (C)[2] | | Mascot |
| 1623.7729 | 1623.7515 | -0.0214 | -13 | 210 | 223 | RCFAGCLPESSLQR | | | Carbamidomethyl (C)[2] | | Mascot |
| 1748.8813 | 1748.8676 | -0.0137 | -8 | 34 | 48 | DNPLDVEKLSTFSQR | | | | | Mascot |
| 1829.9366 | 1829.9308 | -0.0058 | -3 | 42 | 56 | LSTFSQRFPLPSMYR | | | | | Mascot |
| 2233.0376 | 2233.0576 | 0.02 | 9 | 79 | 96 | QYRVDQFEDVSCALTVM R | | | Carbamidomethyl (C)[12], Oxidation (M)[17] | | Mascot |

44 O-sialoglycoprotein endopeptidase-like 1 [Danio rerio] gi|169145701 44028.3 8.83 8 32 0

**Peptide Information**

| Calc. Mass | Obsrv. Mass | ± da | ± ppm | Start Seq. | End Seq. | Sequence | Ion Score | C. I. % | Modification | Rank | Result Type |
|---|---|---|---|---|---|---|---|---|---|---|---|
| 1073.6313 | 1073.4951 | -0.1362 | -127 | 246 | 255 | TQITGAINKK | | | | | Mascot |
| 1148.5881 | 1148.5958 | 0.0077 | 7 | 2 | 11 | FQSCLPGALR | | | Carbamidomethyl (C)[4] | | Mascot |
| 1607.8112 | 1607.7588 | -0.0524 | -33 | 2 | 15 | FQSCLPGALRSWSR | | | | | Mascot |
| 1619.85 | 1619.8684 | 0.0184 | 11 | 272 | 287 | DIAAASQHTVASHLAK | | | | | Mascot |
| 1678.9197 | 1678.85 | -0.0697 | -42 | 327 | 342 | IITDATGLHLLCPPSK | | | | | Mascot |
| 1809.8687 | 1809.7441 | -0.1246 | -69 | 256 | 271 | EKEEGVEAGQFLSCVK | | | Carbamidomethyl (C)[14] | | Mascot |
| 1825.8359 | 1825.7393 | -0.0966 | -53 | 343 | 358 | FCTDNGVMIAWNGIER | | | | | Mascot |
| 1841.8309 | 1841.7614 | -0.0695 | -38 | 343 | 358 | FCTDNGVMIAWNGIER | | | Oxidation (M)[8] | | Mascot |
| 2249.0964 | 2249.0305 | -0.0659 | -29 | 26 | 47 | LVLGIETSCDETGAAVLD | | | | | Mascot |

ETGR

| | | | | | | | | | |
|---|---|---|---|---|---|---|---|---|---|
| 45 | TPA_exp: glutamine synthetase [Danio rerio] | gi\|30141105 | 41549.8 | 5.57 | 2 | 32 | 0 | 26 | 24.512 |

**Peptide Information**

| Calc. Mass | Obsrv. Mass | ± da | ± ppm | Start Seq. | End Seq. | Sequence | Ion Score | C. I. % | Modification | Rank | Result Type |
|---|---|---|---|---|---|---|---|---|---|---|---|
| 869.5315 | 869.5105 | -0.021 | -24 | 320 | 327 | GASIRIPR | | | | | Mascot |
| 869.5315 | 869.5105 | -0.021 | -24 | 320 | 327 | GASIRIPR | 26 | 24.512 | | | Mascot |
| 902.5378 | 902.5221 | -0.0157 | -17 | 96 | 103 | LVLCEVVK | | | | | Mascot |

| | | | | | | | | |
|---|---|---|---|---|---|---|---|---|
| 46 | PREDICTED: BAH and coiled-coil domain-containing protein 1-like [Danio rerio] | gi\|326672508 | 287695.1 | 8.4 | 1 | | 31 | 78.429 |

**Peptide Information**

| Calc. Mass | Obsrv. Mass | ± da | ± ppm | Start Seq. | End Seq. | Sequence | Ion Score | C. I. % | Modification | Rank | Result Type |
|---|---|---|---|---|---|---|---|---|---|---|---|
| 892.4634 | 892.4758 | 0.0124 | 14 | 866 | 872 | DRVEFAR | 31 | 78.429 | | 2 | Mascot |

| | | | | | | | | |
|---|---|---|---|---|---|---|---|---|
| 47 | PREDICTED: hypothetical protein LOC402835 [Danio rerio] | gi\|326671330 | 402017.2 | 4.54 | 1 | | 28 | 49.432 |

**Peptide Information**

| Calc. Mass | Obsrv. Mass | ± da | ± ppm | Start Seq. | End Seq. | Sequence | Ion Score | C. I. % | Modification | Rank | Result Type |
|---|---|---|---|---|---|---|---|---|---|---|---|
| 892.4556 | 892.4758 | 0.0202 | 23 | 565 | 572 | VLGTEMAR | 28 | 49.432 | Oxidation (M)[6] | 3 | Mascot |

| | | | | | | | | |
|---|---|---|---|---|---|---|---|---|
| 48 | nuclear respiratory factor 1 [Danio rerio] | gi\|18859135 | 53552 | 5.02 | 1 | | 26 | 23.814 |

**Peptide Information**

| Calc. Mass | Obsrv. Mass | ± da | ± ppm | Start Seq. | End Seq. | Sequence | Ion Score | C. I. % | Modification | Rank | Result Type |
|---|---|---|---|---|---|---|---|---|---|---|---|
| 960.5261 | 960.4583 | -0.0678 | -71 | 257 | 264 | VSWTQALR | 26 | 23.814 | | 2 | Mascot |

| | | | | | | | | |
|---|---|---|---|---|---|---|---|---|
| 49 | novel protein similar to vertebrate scavenger receptor protein [Danio rerio] | gi\|56208005 | 121130 | 4.56 | 1 | | 24 | 0 |

**Peptide Information**

| Calc. Mass | Obsrv. Mass | ± da | ± ppm | Start Seq. | End Seq. | Sequence | Ion Score | C. I. % | Modification | Rank | Result Type |
|---|---|---|---|---|---|---|---|---|---|---|---|
| 960.493 | 960.4583 | -0.0347 | -36 | 725 | 733 | DAGVICGLR | 24 | 0 | Carbamidomethyl (C)[6] | 3 | Mascot |

| | | | | | | | | |
|---|---|---|---|---|---|---|---|---|
| 50 | PREDICTED: death-associated protein kinase 2 [Danio rerio] | gi\|326676536 | 41405.2 | 6.4 | 1 | | 23 | 0 |

**Peptide Information**

| Calc. Mass | Obsrv. Mass | ± da | ± ppm | Start Seq. | End Seq. | Sequence | Ion Score | C. I. % | Modification | Rank | Result Type |
|---|---|---|---|---|---|---|---|---|---|---|---|
| 960.4744 | 960.4583 | -0.0161 | -17 | 329 | 336 | GTEQQELR | 23 | 0 | | 4 | Mascot |

| Gel Idx/Pos | 189/H16 | Instr./Gel Origin | BA2060/110707A | Process Status | Analysis Succeeded |
|---|---|---|---|---|---|
| Plate [#] Name | [1] 31496 | Instrument Sample Name | | Spectra | 11 |

| Rank | Protein Name | Accession No. | Protein MW | Protein PI | Pep. Count | Protein Score | Protein Score C. I. % | Total Ion Score | Total Ion C. I. % |
|---|---|---|---|---|---|---|---|---|---|
| 1 | novel protein (zgc:85809) [Danio rerio] | gi\|148725394 | 69172 | 6.25 | 12 | 448 | 100 | 402 | 100 |

Peptide Information

| Calc. Mass | Obsrv. Mass | ± da | ± ppm | Start Seq. | End Seq. | Sequence | Ion Score | C. I. % | Modification | Rank | Result Type |
|---|---|---|---|---|---|---|---|---|---|---|---|
| 870.4389 | 870.5333 | 0.0944 | 108 | 519 | 525 | KMGEVYK | | | Oxidation (M)[2] | | Mascot |
| 908.5352 | 908.5312 | -0.004 | -4 | 566 | 572 | FTRPLFK | | | | | Mascot |
| 1102.6289 | 1102.6257 | -0.0032 | -3 | 165 | 174 | ELVALMSALR | | | | | Mascot |
| 1118.6238 | 1118.6157 | -0.0081 | -7 | 165 | 174 | ELVALMSALR | | | Oxidation (M)[6] | | Mascot |
| 1320.6696 | 1320.6624 | -0.0072 | -5 | 154 | 164 | HTYYAQVSVPR | 89 | 100 | | | Mascot |
| 1320.6696 | 1320.6624 | -0.0072 | -5 | 154 | 164 | HTYYAQVSVPR | | | | | Mascot |
| 1354.7365 | 1354.7288 | -0.0077 | -6 | 545 | 556 | AQWEEAVPLALK | | | | | Mascot |
| 1541.7859 | 1541.7784 | -0.0075 | -5 | 20 | 31 | HLNLIYHVDFDR | | | | | Mascot |
| 1541.7859 | 1541.7784 | -0.0075 | -5 | 20 | 31 | HLNLIYHVDFDR | 84 | 100 | | | Mascot |
| 1558.8475 | 1558.8386 | -0.0089 | -6 | 83 | 96 | GSPLEITLPFELSR | 98 | 100 | | | Mascot |
| 1558.8475 | 1558.8386 | -0.0089 | -6 | 83 | 96 | GSPLEITLPFELSR | | | | | Mascot |
| 1834.0109 | 1833.9923 | -0.0186 | -10 | 81 | 96 | FKGSPLEITLPFELSR | | | | | Mascot |
| 2194.1853 | 2194.1262 | -0.0591 | -27 | 38 | 57 | VALTVEVLEDKFSSLTLD SK | | | | | Mascot |
| 2217.0513 | 2217.0422 | -0.0091 | -4 | 310 | 326 | TWEHFWLNEGHTVYIER | | | | | Mascot |
| 2362.2261 | 2362.209 | -0.0171 | -7 | 288 | 309 | SLAGVIAHEISHSWTGNL VTNR | 130 | 100 | | | Mascot |
| 2362.2261 | 2362.209 | -0.0171 | -7 | 288 | 309 | SLAGVIAHEISHSWTGNL VTNR | | | | | Mascot |
| 2811.4563 | 2811.4519 | -0.0044 | -2 | 97 | 121 | GQHVIVEIEYETSPTATAL QWLTPK | | | | | Mascot |

| Rank | Protein Name | Accession No. | Protein MW | Protein PI | Pep. Count | Protein Score | Protein Score C. I. % | Total Ion Score | Total Ion C. I. % |
|---|---|---|---|---|---|---|---|---|---|
| 2 | leukotriene A-4 hydrolase [Danio rerio] | gi\|47086915 | 69145 | 6.17 | 11 | 443 | 100 | 402 | 100 |

Peptide Information

| Calc. Mass | Obsrv. Mass | ± da | ± ppm | Start Seq. | End Seq. | Sequence | Ion Score | C. I. % | Modification | Rank | Result Type |
|---|---|---|---|---|---|---|---|---|---|---|---|
| 870.4389 | 870.5333 | 0.0944 | 108 | 519 | 525 | KMGEVYK | | | Oxidation (M)[2] | | Mascot |

| Calc. Mass | Obsrv. Mass | ± da | ± ppm | Start Seq. | End Seq. | Sequence | Ion Score | C. I. % | Modification | Rank | Result Type |
|---|---|---|---|---|---|---|---|---|---|---|---|
| 1102.6289 | 1102.6257 | -0.0032 | -3 | 165 | 174 | ELVALMSALR | | | | | Mascot |
| 1118.6238 | 1118.6157 | -0.0081 | -7 | 165 | 174 | ELVALMSALR | | | Oxidation (M)[6] | | Mascot |
| 1320.6696 | 1320.6624 | -0.0072 | -5 | 154 | 164 | HTYYAQVSVPR | 89 | 100 | | | Mascot |
| 1320.6696 | 1320.6624 | -0.0072 | -5 | 154 | 164 | HTYYAQVSVPR | | | | | Mascot |
| 1354.7365 | 1354.7288 | -0.0077 | -6 | 545 | 556 | AQWEEAVPLALK | | | | | Mascot |
| 1541.7859 | 1541.7784 | -0.0075 | -5 | 20 | 31 | HLNLIYHVDFDR | | | | | Mascot |
| 1541.7859 | 1541.7784 | -0.0075 | -5 | 20 | 31 | HLNLIYHVDFDR | 84 | 100 | | | Mascot |
| 1558.8475 | 1558.8386 | -0.0089 | -6 | 83 | 96 | GSPLEITLPFELSR | 98 | 100 | | | Mascot |
| 1558.8475 | 1558.8386 | -0.0089 | -6 | 83 | 96 | GSPLEITLPFELSR | | | | | Mascot |
| 1834.0109 | 1833.9923 | -0.0186 | -10 | 81 | 96 | FKGSPLEITLPFELSR | | | | | Mascot |
| 2194.1853 | 2194.1262 | -0.0591 | -27 | 38 | 57 | VALTVEVLEDKFSSLTLD SK | | | | | Mascot |
| 2217.0513 | 2217.0422 | -0.0091 | -4 | 310 | 326 | TWEHFWLNEGHTVYIER | | | | | Mascot |
| 2362.2261 | 2362.209 | -0.0171 | -7 | 288 | 309 | SLAGVIAHEISHSWTGNL VTNR | 130 | 100 | | | Mascot |
| 2362.2261 | 2362.209 | -0.0171 | -7 | 288 | 309 | SLAGVIAHEISHSWTGNL VTNR | | | | | Mascot |
| 2811.4563 | 2811.4519 | -0.0044 | -2 | 97 | 121 | GQHVIVEIEYETSPTATAL QWLTPK | | | | | Mascot |

| | | | | | | | | | | |
|---|---|---|---|---|---|---|---|---|---|---|
| 3 | novel protein (zgc:85809) [Danio rerio] | gi\|55962574 | 22856.7 | 8.76 | 7 | 312 | 100 | 272 | 100 | |

**Peptide Information**

| Calc. Mass | Obsrv. Mass | ± da | ± ppm | Start Seq. | End Seq. | Sequence | Ion Score | C. I. % | Modification | Rank | Result Type |
|---|---|---|---|---|---|---|---|---|---|---|---|
| 1102.6289 | 1102.6257 | -0.0032 | -3 | 165 | 174 | ELVALMSALR | | | | | Mascot |
| 1118.6238 | 1118.6157 | -0.0081 | -7 | 165 | 174 | ELVALMSALR | | | Oxidation (M)[6] | | Mascot |
| 1320.6696 | 1320.6624 | -0.0072 | -5 | 154 | 164 | HTYYAQVSVPR | 89 | 100 | | | Mascot |
| 1320.6696 | 1320.6624 | -0.0072 | -5 | 154 | 164 | HTYYAQVSVPR | | | | | Mascot |
| 1541.7859 | 1541.7784 | -0.0075 | -5 | 20 | 31 | HLNLIYHVDFDR | | | | | Mascot |
| 1541.7859 | 1541.7784 | -0.0075 | -5 | 20 | 31 | HLNLIYHVDFDR | 84 | 100 | | | Mascot |
| 1558.8475 | 1558.8386 | -0.0089 | -6 | 83 | 96 | GSPLEITLPFELSR | 98 | 100 | | | Mascot |
| 1558.8475 | 1558.8386 | -0.0089 | -6 | 83 | 96 | GSPLEITLPFELSR | | | | | Mascot |
| 1834.0109 | 1833.9923 | -0.0186 | -10 | 81 | 96 | FKGSPLEITLPFELSR | | | | | Mascot |
| 2194.1853 | 2194.1262 | -0.0591 | -27 | 38 | 57 | VALTVEVLEDKFSSLTLD SK | | | | | Mascot |
| 2811.4563 | 2811.4519 | -0.0044 | -2 | 97 | 121 | GQHVIVEIEYETSPTATAL QWLTPK | | | | | Mascot |

| | | | | | | | | | | |
|---|---|---|---|---|---|---|---|---|---|---|
| 4 | 5-aminoimidazole-4-carboxamide ribonucleotide formyltransferase/IMP cyclohydrolase [Danio rerio] | gi\|122891203 | 64016.9 | 6.2 | 19 | 286 | 100 | 173 | 100 | |

**Peptide Information**

| Calc. Mass | Obsrv. Mass | ± da | ± ppm | Start Seq. | End Seq. | Sequence | Ion Score | C. I. % | Modification | Rank | Result Type |
|---|---|---|---|---|---|---|---|---|---|---|---|

| Calc. Mass | Obsrv. Mass | ± da | ± ppm | Start Seq. | End Seq. | Sequence | Ion Score | C. I. % | Modification | Rank | Result Type |
|---|---|---|---|---|---|---|---|---|---|---|---|
| 869.5203 | 869.5234 | 0.0031 | 4 | 199 | 206 | GVSQLPLR | | | | | Mascot |
| 892.4886 | 892.4858 | -0.0028 | -3 | 14 | 21 | TGLVEFAR | | | | | Mascot |
| 926.5822 | 926.5747 | -0.0075 | -8 | 379 | 386 | VLFGLHLK | | | | | Mascot |
| 960.4686 | 960.4664 | -0.0022 | -2 | 460 | 466 | ADNWWLR | | | | | Mascot |
| 960.4686 | 960.4664 | -0.0022 | -2 | 460 | 466 | ADNWWLR | 43 | 98.536 | | | Mascot |
| 1075.6146 | 1075.6244 | 0.0098 | 9 | 255 | 265 | ALGLPAATSFK | | | | | Mascot |
| 1320.5889 | 1320.6624 | 0.0735 | 56 | 425 | 435 | YTQSNSVCYAK | | | Carbamidomethyl (C)[8] | | Mascot |
| 1320.5889 | 1320.6624 | 0.0735 | 56 | 425 | 435 | YTQSNSVCYAK | | | Carbamidomethyl (C)[8] | | Mascot |
| 1341.7749 | 1341.7687 | -0.0062 | -5 | 66 | 78 | TLHPAVHGGILAR | | | | | Mascot |
| 1385.7131 | 1385.7086 | -0.0045 | -3 | 436 | 449 | DGQVIGIGAGQQSR | | | | | Mascot |
| 1607.7733 | 1607.7671 | -0.0062 | -4 | 49 | 63 | DVSEITGFPEMLGGR | | | | | Mascot |
| 1623.7683 | 1623.7631 | -0.0052 | -3 | 49 | 63 | DVSEITGFPEMLGGR | | | Oxidation (M)[11] | | Mascot |
| 1637.6636 | 1637.6639 | 0.0003 | 0 | 366 | 378 | MDPEYEPDEEEVR | | | | | Mascot |
| 1653.6584 | 1653.6521 | -0.0063 | -4 | 366 | 378 | MDPEYEPDEEEVR | | | Oxidation (M)[1] | | Mascot |
| 1665.8152 | 1665.8523 | 0.0371 | 22 | 141 | 155 | VTVVCDPSDYNVVAK | | | Carbamidomethyl (C)[5] | | Mascot |
| 1710.8955 | 1710.8851 | -0.0104 | -6 | 572 | 586 | ACNELGITLVHTNIR | | | Carbamidomethyl (C)[2] | | Mascot |
| 1819.8708 | 1819.86 | -0.0108 | -6 | 335 | 351 | EVSDGIVAPGYEEDALR | | | | | Mascot |
| 1829.9531 | 1829.9438 | -0.0093 | -5 | 508 | 523 | GLFEEVPEPLSEVEKK | | | | | Mascot |
| 1833.9341 | 1833.9923 | 0.0582 | 32 | 266 | 284 | HVSPAGAAVGVPLSEDE AK | | | | | Mascot |
| 2034.9192 | 2034.9102 | -0.009 | -4 | 177 | 193 | AFTHTAQYDEAISDYFR | | | | | Mascot |
| 2034.9192 | 2034.9102 | -0.009 | -4 | 177 | 193 | AFTHTAQYDEAISDYFR | 132 | 100 | | | Mascot |
| 2225.0764 | 2225.1055 | 0.0291 | 13 | 285 | 304 | VCMVNDMLQDLTPLATA YAR | | | | | Mascot |
| 2225.0764 | 2225.1055 | 0.0291 | 13 | 285 | 304 | VCMVNDMLQDLTPLATA YAR | | | | | Mascot |
| 2237.1846 | 2237.0667 | -0.1179 | -53 | 1 | 21 | MASEIALLSVSDKTGLVE FAR | | | | | Mascot |
| 2298.0928 | 2298.1726 | 0.0798 | 35 | 285 | 304 | VCMVNDMLQDLTPLATA YAR | | | Carbamidomethyl (C)[2], Oxidation (M)[3] | | Mascot |
| 2314.0876 | 2314.1418 | 0.0542 | 23 | 285 | 304 | VCMVNDMLQDLTPLATA YAR | | | Carbamidomethyl (C)[2], Oxidation (M)[3,7] | | Mascot |
| 2555.3562 | 2555.3406 | -0.0156 | -6 | 108 | 132 | TVSSPGVTVEDAVEQIDI GGVTLLR | | | | | Mascot |

| | | | | | | | | | | |
|---|---|---|---|---|---|---|---|---|---|---|
| 5 | bifunctional purine biosynthesis protein PURH [Danio rerio] | gi\|195539484 | 64003.9 | 6.31 | 19 | 285 | 100 | 173 | 100 | |

**Peptide Information**

| Calc. Mass | Obsrv. Mass | ± da | ± ppm | Start Seq. | End Seq. | Sequence | Ion Score | C. I. % | Modification | Rank | Result Type |
|---|---|---|---|---|---|---|---|---|---|---|---|
| 869.5203 | 869.5234 | 0.0031 | 4 | 199 | 206 | GVSQLPLR | | | | | Mascot |
| 892.4886 | 892.4858 | -0.0028 | -3 | 14 | 21 | TGLVEFAR | | | | | Mascot |

| | | | | | | | | | | | |
|---|---|---|---|---|---|---|---|---|---|---|---|
| 926.5822 | 926.5747 | -0.0075 | -8 | 379 | 386 | VLFGLHLK | | | | | Mascot |
| 960.4686 | 960.4664 | -0.0022 | -2 | 460 | 466 | ADNWWLR | | | | | Mascot |
| 960.4686 | 960.4664 | -0.0022 | -2 | 460 | 466 | ADNWWLR | 43 | 98.536 | | | Mascot |
| 1075.6146 | 1075.6244 | 0.0098 | 9 | 255 | 265 | ALGLPAATSFK | | | | | Mascot |
| 1320.5889 | 1320.6624 | 0.0735 | 56 | 425 | 435 | YTQSNSVCYAK | | | Carbamidomethyl (C)[8] | | Mascot |
| 1320.5889 | 1320.6624 | 0.0735 | 56 | 425 | 435 | YTQSNSVCYAK | | | Carbamidomethyl (C)[8] | | Mascot |
| 1341.7749 | 1341.7687 | -0.0062 | -5 | 66 | 78 | TLHPAVHGGILAR | | | | | Mascot |
| 1385.7131 | 1385.7086 | -0.0045 | -3 | 436 | 449 | DGQVIGIGAGQQSR | | | | | Mascot |
| 1607.7733 | 1607.7671 | -0.0062 | -4 | 49 | 63 | DVSEITGFPEMLGGR | | | | | Mascot |
| 1623.7683 | 1623.7631 | -0.0052 | -3 | 49 | 63 | DVSEITGFPEMLGGR | | | Oxidation (M)[11] | | Mascot |
| 1637.6636 | 1637.6639 | 0.0003 | 0 | 366 | 378 | MDPEYEPDEEEVR | | | | | Mascot |
| 1653.6584 | 1653.6521 | -0.0063 | -4 | 366 | 378 | MDPEYEPDEEEVR | | | Oxidation (M)[1] | | Mascot |
| 1665.8152 | 1665.8523 | 0.0371 | 22 | 141 | 155 | VTVVCDPSDYNVVAK | | | Carbamidomethyl (C)[5] | | Mascot |
| 1710.8955 | 1710.8851 | -0.0104 | -6 | 572 | 586 | ACNELGITLVHTNIR | | | Carbamidomethyl (C)[2] | | Mascot |
| 1819.8708 | 1819.86 | -0.0108 | -6 | 335 | 351 | EVSDGIVAPGYEEDALR | | | | | Mascot |
| 1829.9531 | 1829.9438 | -0.0093 | -5 | 508 | 523 | GLFEEVPEPLSEVEKK | | | | | Mascot |
| 1833.9341 | 1833.9923 | 0.0582 | 32 | 266 | 284 | HVSPAGAAVGVPLSEDEAK | | | | | Mascot |
| 2034.9192 | 2034.9102 | -0.009 | -4 | 177 | 193 | AFTHTAQYDEAISDYFR | | | | | Mascot |
| 2034.9192 | 2034.9102 | -0.009 | -4 | 177 | 193 | AFTHTAQYDEAISDYFR | 132 | 100 | | | Mascot |
| 2225.0764 | 2225.1055 | 0.0291 | 13 | 285 | 304 | VCMVNDMLQDLTPLATAYAR | | | | | Mascot |
| 2225.0764 | 2225.1055 | 0.0291 | 13 | 285 | 304 | VCMVNDMLQDLTPLATAYAR | | | | | Mascot |
| 2237.1846 | 2237.0667 | -0.1179 | -53 | 1 | 21 | MASEIALLSVSDKTGLVEFAR | | | | | Mascot |
| 2298.0928 | 2298.1726 | 0.0798 | 35 | 285 | 304 | VCMVNDMLQDLTPLATAYAR | | | Carbamidomethyl (C)[2], Oxidation (M)[3] | | Mascot |
| 2314.0876 | 2314.1418 | 0.0542 | 23 | 285 | 304 | VCMVNDMLQDLTPLATAYAR | | | Carbamidomethyl (C)[2], Oxidation (M)[3,7] | | Mascot |
| 2555.3562 | 2555.3406 | -0.0156 | -6 | 108 | 132 | TVSSPGVTVEDAVEQIDIGGVTLLR | | | | | Mascot |

6 PREDICTED: 39S ribosomal protein L2, mitochondrial isoform 2 [Danio rerio]   gi|326673218   31482.8   11.12   7   42   0

**Peptide Information**

| Calc. Mass | Obsrv. Mass | ± da | ± ppm | Start Seq. | End Seq. | Sequence | Ion Score | C. I. % | Modification | Rank | Result Type |
|---|---|---|---|---|---|---|---|---|---|---|---|
| 960.5294 | 960.4664 | -0.063 | -66 | 194 | 203 | AAGTCGVLLR | | | | | Mascot |
| 960.5294 | 960.4664 | -0.063 | -66 | 194 | 203 | AAGTCGVLLR | 10 | 0 | | | Mascot |
| 1128.6272 | 1128.6136 | -0.0136 | -12 | 250 | 259 | WLGIRPSSGR | | | | | Mascot |
| 1341.8101 | 1341.7687 | -0.0414 | -31 | 204 | 216 | KVSGTAIIQLPSK | | | | | Mascot |

| Calc. Mass | Obsrv. Mass | ± da | ± ppm | Start Seq. | End Seq. | Sequence | Ion Score | C. I. % | Modification | Rank | Result Type |
|---|---|---|---|---|---|---|---|---|---|---|---|
| 1653.874 | 1653.6521 | -0.2219 | -134 | 217 | 231 | HQIQVLETCVATVGR | | | | | Mascot |
| 1710.8955 | 1710.8851 | -0.0104 | -6 | 217 | 231 | HQIQVLETCVATVGR | | | Carbamidomethyl (C)[9] | | Mascot |
| 2034.9735 | 2034.9102 | -0.0633 | -31 | 135 | 152 | WIIASQNMEAGDLIQSCR | | | | | Mascot |
| 2034.9735 | 2034.9102 | -0.0633 | -31 | 135 | 152 | WIIASQNMEAGDLIQSCR | | | | | Mascot |
| 2115.1743 | 2115.0486 | -0.1257 | -59 | 271 | 289 | IRPLPAMKSYINLPTASAR | | | Oxidation (M)[7] | | Mascot |
| 2362.1753 | 2362.209 | 0.0337 | 14 | 12 | 35 | ALCLSGSRTWAGPSAAA SALVGCR | | | Carbamidomethyl (C)[3] | | Mascot |
| 2362.1753 | 2362.209 | 0.0337 | 14 | 12 | 35 | ALCLSGSRTWAGPSAAA SALVGCR | | | Carbamidomethyl (C)[3] | | Mascot |

7 PREDICTED: beta-1,3-galactosyl-O-glycosyl-glycoprotein beta-1,6-N-acetylglucosaminyltransferase-lik gi|292628442 49526.7 7.14 11 42 0

**Peptide Information**

| Calc. Mass | Obsrv. Mass | ± da | ± ppm | Start Seq. | End Seq. | Sequence | Ion Score | C. I. % | Modification | Rank | Result Type |
|---|---|---|---|---|---|---|---|---|---|---|---|
| 856.4675 | 856.5259 | 0.0584 | 68 | 414 | 420 | YKAVYGR | | | | | Mascot |
| 869.4008 | 869.5234 | 0.1226 | 141 | 407 | 413 | CMEAFLR | | | | | Mascot |
| 960.5374 | 960.4664 | -0.071 | -74 | 388 | 395 | QHHLLANK | | | | | Mascot |
| 960.5374 | 960.4664 | -0.071 | -74 | 388 | 395 | QHHLLANK | | | | | Mascot |
| 1118.6165 | 1118.6157 | -0.0008 | -1 | 221 | 230 | TNAETVRSLK | | | | | Mascot |
| 1541.7668 | 1541.7784 | 0.0116 | 8 | 208 | 220 | YLLNTCGTDFPIK | | | Carbamidomethyl (C)[6] | | Mascot |
| 1541.7668 | 1541.7784 | 0.0116 | 8 | 208 | 220 | YLLNTCGTDFPIK | | | Carbamidomethyl (C)[6] | | Mascot |
| 1637.8315 | 1637.6639 | -0.1676 | -102 | 336 | 349 | YEQSDMNAIARLVK | | | | | Mascot |
| 1653.8264 | 1653.6521 | -0.1743 | -105 | 336 | 349 | YEQSDMNAIARLVK | | | Oxidation (M)[6] | | Mascot |
| 1665.8608 | 1665.8523 | -0.0085 | -5 | 250 | 262 | WQFHHNITNTVIR | | | | | Mascot |
| 2230.1108 | 2230.1941 | 0.0833 | 37 | 189 | 207 | VQADVNCMQDLLKSPVQ WR | | | | | Mascot |
| 2233.1621 | 2233.0708 | -0.0913 | -41 | 135 | 153 | LLRAIYAPQNVYCVHVDM K | | | | | Mascot |
| 2238.1375 | 2238.0618 | -0.0757 | -34 | 202 | 220 | SPVQWRYLLNTCGTDFPI K | | | | | Mascot |
| 2299.1538 | 2299.1731 | 0.0193 | 8 | 267 | 287 | KSPPPINTSMFSGNAYFV VSR | | | | | Mascot |

8 PREDICTED: zinc finger protein 420 isoform 2 [Danio rerio] gi|326678086 70371 9.08 15 38 0

**Peptide Information**

| Calc. Mass | Obsrv. Mass | ± da | ± ppm | Start Seq. | End Seq. | Sequence | Ion Score | C. I. % | Modification | Rank | Result Type |
|---|---|---|---|---|---|---|---|---|---|---|---|
| 842.5206 | 842.5072 | -0.0134 | -16 | 25 | 31 | QRQGLLK | | | | | Mascot |
| 864.4798 | 864.5098 | 0.03 | 35 | 591 | 597 | HRSVHTK | | | | | Mascot |
| 1045.536 | 1045.5601 | 0.0241 | 23 | 134 | 141 | FHCARNLK | | | Carbamidomethyl (C)[3] | | Mascot |

| Calc. Mass | Obsrv. Mass | ± da | ± ppm | Start Seq. | End Seq. | Sequence | Ion Score | C. I. % | Modification | Rank | Result Type |
|---|---|---|---|---|---|---|---|---|---|---|---|
| 1118.4645 | 1118.6157 | 0.1512 | 135 | 348 | 356 | GFVCFECEK | | | Carbamidomethyl (C)[4] | | Mascot |
| 1623.806 | 1623.7631 | -0.0429 | -26 | 105 | 117 | SFSQKYNLNVHMR | | | | | Mascot |
| 1665.8087 | 1665.8523 | 0.0436 | 26 | 174 | 188 | VHTKEKPYSCSVCGK | | | | | Mascot |
| 1819.9358 | 1819.86 | -0.0758 | -42 | 44 | 58 | IKQEETEELIDVMVK | | | Oxidation (M)[13] | | Mascot |
| 2034.8579 | 2034.9102 | 0.0523 | 26 | 118 | 133 | IHTGEQPYKCSHCDMR | | | Carbamidomethyl (C)[10,13], Oxidation (M)[15] | | Mascot |
| 2034.8579 | 2034.9102 | 0.0523 | 26 | 118 | 133 | IHTGEQPYKCSHCDMR | | | Carbamidomethyl (C)[10,13], Oxidation (M)[15] | | Mascot |
| 2211.0798 | 2211.0938 | 0.014 | 6 | 450 | 468 | IHLRVHTNEKPYSCPECGK | | | | | Mascot |
| 2221.0139 | 2221.0764 | 0.0625 | 28 | 239 | 257 | CSHCDLRFSHSGHLTVHER | | | | | Mascot |
| 2223.9646 | 2224.0549 | 0.0903 | 41 | 318 | 337 | EKPYSCSECGSSFTQQSSLR | | | | | Mascot |
| 2230.0605 | 2230.1941 | 0.1336 | 60 | 276 | 294 | CSSHLNEHMIIHTGEKTHR | | | | | Mascot |
| 2255.0696 | 2255.0515 | -0.0181 | -8 | 273 | 291 | SFKCSSHLNEHMIIHTGEK | | | Carbamidomethyl (C)[4] | | Mascot |
| 2283.0579 | 2283.166 | 0.1081 | 47 | 227 | 245 | HQMIHTGEKPYKCSHCDLR | | | | | Mascot |
| 2283.0579 | 2283.166 | 0.1081 | 47 | 227 | 245 | HQMIHTGEKPYKCSHCDLR | | | | | Mascot |
| 2298.0906 | 2298.1726 | 0.082 | 36 | 478 | 496 | LHQHVHTGVREFVCFECEK | | | | | Mascot |
| 2299.053 | 2299.1731 | 0.1201 | 52 | 227 | 245 | HQMIHTGEKPYKCSHCDLR | | | Oxidation (M)[3] | | Mascot |

9 Zgc:110114 protein [Danio rerio] gi|63101268 70288.7 6.5 11 38 0

**Peptide Information**

| Calc. Mass | Obsrv. Mass | ± da | ± ppm | Start Seq. | End Seq. | Sequence | Ion Score | C. I. % | Modification | Rank | Result Type |
|---|---|---|---|---|---|---|---|---|---|---|---|
| 869.4661 | 869.5234 | 0.0573 | 66 | 76 | 82 | LSKHCPK | | | Carbamidomethyl (C)[5] | | Mascot |
| 892.4457 | 892.4858 | 0.0401 | 45 | 191 | 197 | HIPNMHK | | | Oxidation (M)[5] | | Mascot |
| 1541.7289 | 1541.7784 | 0.0495 | 32 | 483 | 497 | EVSSSTSSSSETVVR | | | | | Mascot |
| 1541.7289 | 1541.7784 | 0.0495 | 32 | 483 | 497 | EVSSSTSSSSETVVR | | | | | Mascot |
| 1607.7516 | 1607.7671 | 0.0155 | 10 | 500 | 513 | GQSIECLPQTTMAR | | | Carbamidomethyl (C)[6], Oxidation (M)[12] | | Mascot |
| 1665.7939 | 1665.8523 | 0.0584 | 35 | 360 | 376 | ASGSSAGSTSRPNPFSR | | | | | Mascot |
| 1829.9102 | 1829.9438 | 0.0336 | 18 | 198 | 213 | EQFACTASEAQLKYIK | | | | | Mascot |
| 2224.2163 | 2224.0549 | -0.1614 | -73 | 259 | 277 | QLLYDFPWTNVGKLVFVGK | | | | | Mascot |
| 2229.9966 | 2230.1941 | 0.1975 | 89 | 408 | 429 | THSSSNTSHASTHTSGIESSSK | | | | | Mascot |
| 2283.1008 | 2283.166 | 0.0652 | 29 | 191 | 210 | HIPNMHKEQFACTASEAQLK | | | | | Mascot |
| 2283.1008 | 2283.166 | 0.0652 | 29 | 191 | 210 | HIPNMHKEQFACTASEAQLK | | | | | Mascot |
| 2299.0957 | 2299.1731 | 0.0774 | 34 | 191 | 210 | HIPNMHKEQFACTASEAQLK | | | Oxidation (M)[5] | | Mascot |
| 2362.2256 | 2362.209 | -0.0166 | -7 | 16 | 35 | RCVCVFLPNDETLNVIVSVK | | | Carbamidomethyl (C)[2,4] | | Mascot |

| Calc. Mass | Obsrv. Mass | ± da | ± ppm | Start Seq. | End Seq. | Sequence | Ion Score | C. I. % | Modification | Rank | Result Type |
|---|---|---|---|---|---|---|---|---|---|---|---|
| 2362.2256 | 2362.209 | -0.0166 | -7 | 16 | 35 | RCVCVFLPNDETLNVIVSVK | | | Carbamidomethyl (C)[2,4] | | Mascot |
| 2555.1467 | 2555.3406 | 0.1939 | 76 | 337 | 357 | QYRESYIGDALEADMDHLEQR | | | Oxidation (M)[15] | | Mascot |

10 SH3 domain-binding protein 4-A [Danio rerio] gi|45387913 104947.5 7.73 13 38 0

Peptide Information

| Calc. Mass | Obsrv. Mass | ± da | ± ppm | Start Seq. | End Seq. | Sequence | Ion Score | C. I. % | Modification | Rank | Result Type |
|---|---|---|---|---|---|---|---|---|---|---|---|
| 842.5822 | 842.5072 | -0.075 | -89 | 634 | 640 | LIKTVIR | | | | | Mascot |
| 850.4338 | 850.5395 | 0.1057 | 124 | 358 | 364 | LSNMEIK | | | Oxidation (M)[4] | | Mascot |
| 850.4338 | 850.5395 | 0.1057 | 124 | 358 | 364 | LSNMEIK | | | Oxidation (M)[4] | | Mascot |
| 870.4726 | 870.5333 | 0.0607 | 70 | 1 | 7 | MAAHRIR | | | Oxidation (M)[1] | | Mascot |
| 1045.4796 | 1045.5601 | 0.0805 | 77 | 762 | 770 | AELDSEPER | | | | | Mascot |
| 1075.651 | 1075.6244 | -0.0266 | -25 | 446 | 455 | KVTLGLYGPK | | | | | Mascot |
| 1623.7723 | 1623.7631 | -0.0092 | -6 | 66 | 79 | DYCPSSFTTLKFSK | | | | | Mascot |
| 1946.9316 | 1946.9719 | 0.0403 | 21 | 101 | 116 | EMGYIPSTYVQPINYR | | | Oxidation (M)[2] | | Mascot |
| 2230.2417 | 2230.1941 | -0.0476 | -21 | 813 | 831 | LIMDFVLLTTAVEVAPRWR | | | | | Mascot |
| 2284.2368 | 2284.1619 | -0.0749 | -33 | 463 | 484 | TVVAIFGHDCAPKTLLVSEVGK | | | | | Mascot |
| 2300.0686 | 2300.1689 | 0.1003 | 44 | 568 | 587 | DDQDCILAQFCVQTPQPPPK | | | Carbamidomethyl (C)[5] | | Mascot |
| 2811.3804 | 2811.4519 | 0.0715 | 25 | 564 | 587 | VQVKDDQDCILAQFCVQTPQPPPK | | | Carbamidomethyl (C)[9,15] | | Mascot |
| 3337.6052 | 3337.7546 | 0.1494 | 45 | 269 | 298 | LARSCHDLESLGQNPGWGQTQPVETNIVCK | | | Carbamidomethyl (C)[5] | | Mascot |
| 3338.5496 | 3338.7439 | 0.1943 | 58 | 856 | 884 | TGMVDSEAMWKPAYDFLLTWAAQIGDSYR | | | Oxidation (M)[3] | | Mascot |

11 StAR-related lipid transfer domain containing 8 [Danio rerio] gi|187608279 120586.8 8.23 14 36 0

Peptide Information

| Calc. Mass | Obsrv. Mass | ± da | ± ppm | Start Seq. | End Seq. | Sequence | Ion Score | C. I. % | Modification | Rank | Result Type |
|---|---|---|---|---|---|---|---|---|---|---|---|
| 842.557 | 842.5072 | -0.0498 | -59 | 24 | 30 | ALTLLRR | | | | | Mascot |
| 864.4461 | 864.5098 | 0.0637 | 74 | 616 | 622 | VPDYKDK | | | | | Mascot |
| 892.3981 | 892.4858 | 0.0877 | 98 | 44 | 50 | DACEWLR | | | | | Mascot |
| 937.5213 | 937.4719 | -0.0494 | -53 | 800 | 808 | KGGAKPDHK | | | | | Mascot |
| 996.5043 | 996.5922 | 0.0879 | 88 | 906 | 914 | KVGDGHPMR | | | | | Mascot |
| 996.5043 | 996.5922 | 0.0879 | 88 | 906 | 914 | KVGDGHPMR | | | | | Mascot |
| 1279.6827 | 1279.7686 | 0.0859 | 67 | 653 | 663 | SQCLEKVGIFR | | | | | Mascot |
| 1341.6481 | 1341.7687 | 0.1206 | 90 | 299 | 309 | DPSWRAHTICR | | | | | Mascot |
| 1385.6444 | 1385.7086 | 0.0642 | 46 | 404 | 416 | SGSQDFLSGFGQR | | | | | Mascot |

| Calc. Mass | Obsrv. Mass | ± da | ± ppm | Start Seq. | End Seq. | Sequence | Ion Score | C. I. % | Modification | Rank | Result Type |
|---|---|---|---|---|---|---|---|---|---|---|---|
| 1541.7455 | 1541.7784 | 0.0329 | 21 | 404 | 417 | SGSQDFLSGFGQRR | | | | | Mascot |
| 1541.7455 | 1541.7784 | 0.0329 | 21 | 404 | 417 | SGSQDFLSGFGQRR | | | | | Mascot |
| 1665.7198 | 1665.8523 | 0.1325 | 80 | 220 | 235 | DENDDTGSSSSLPSVR | | | | | Mascot |
| 1830.0596 | 1829.9438 | -0.1158 | -63 | 572 | 587 | QSAAQLNLLQKFSLLR | | | | | Mascot |
| 2194.0818 | 2194.1262 | 0.0444 | 20 | 92 | 110 | LTTLNKCAAMSLEVHFQCK | | | Carbamidomethyl (C)[7] | | Mascot |
| 2297.1553 | 2297.1833 | 0.028 | 12 | 988 | 1009 | GVCVLVSSSVDHDNVQLEAGLR | | | | | Mascot |
| 2362.0659 | 2362.209 | 0.1431 | 61 | 809 | 829 | DLSENMAATHCLSHMITECKK | | | | | Mascot |
| 2362.0659 | 2362.209 | 0.1431 | 61 | 809 | 829 | DLSENMAATHCLSHMITECKK | | | | | Mascot |

| | | | | | | | | | |
|---|---|---|---|---|---|---|---|---|---|
| 12 | PREDICTED: GAS2-like protein 2-like [Danio rerio] | gi\|326668195 | 100432.5 | 9.58 | 13 | 36 | 0 | 3 | 0 |

**Peptide Information**

| Calc. Mass | Obsrv. Mass | ± da | ± ppm | Start Seq. | End Seq. | Sequence | Ion Score | C. I. % | Modification | Rank | Result Type |
|---|---|---|---|---|---|---|---|---|---|---|---|
| 856.3981 | 856.5259 | 0.1278 | 149 | 657 | 663 | IPSDHCK | | | Carbamidomethyl (C)[6] | | Mascot |
| 937.5577 | 937.4719 | -0.0858 | -92 | 871 | 878 | SHVPITRK | | | | | Mascot |
| 996.5883 | 996.5922 | 0.0039 | 4 | 808 | 815 | LRPCVRPR | | | | | Mascot |
| 996.5883 | 996.5922 | 0.0039 | 4 | 808 | 815 | LRPCVRPR | | | | | Mascot |
| 1045.5636 | 1045.5601 | -0.0035 | -3 | 668 | 677 | SASLDRATPK | | | | | Mascot |
| 1623.8489 | 1623.7631 | -0.0858 | -53 | 430 | 443 | NLGQTWTKSQFVSK | | | | | Mascot |
| 1653.7399 | 1653.6521 | -0.0878 | -53 | 324 | 337 | SSCSPDPEHQPTRR | | | Carbamidomethyl (C)[3] | | Mascot |
| 2115.1477 | 2115.0486 | -0.0991 | -47 | 183 | 200 | SLINIQNLDEMVQLLISR | | | Oxidation (M)[11] | | Mascot |
| 2194.0266 | 2194.1262 | 0.0996 | 45 | 349 | 367 | ERPGTPQMFTCPDSKDSLK | | | Carbamidomethyl (C)[11] | | Mascot |
| 2255.0298 | 2255.0515 | 0.0217 | 10 | 239 | 257 | VGGGWDTLEHYLDKHDPCR | | | Carbamidomethyl (C)[18] | | Mascot |
| 2283.1248 | 2283.166 | 0.0412 | 18 | 112 | 130 | KEMSIPDVLMFETDDLVLR | | | Oxidation (M)[3,10] | | Mascot |
| 2283.1248 | 2283.166 | 0.0412 | 18 | 113 | 131 | EMSIPDVLMFETDDLVLRK | | | Oxidation (M)[2,9] | | Mascot |
| 2362.303 | 2362.209 | -0.094 | -40 | 502 | 522 | TFSPFKGLIGAILEAQFNRPR | 3 | 0 | | | Mascot |
| 2362.303 | 2362.209 | -0.094 | -40 | 502 | 522 | TFSPFKGLIGAILEAQFNRPR | | | | | Mascot |
| 3338.6616 | 3338.7439 | 0.0823 | 25 | 183 | 211 | SLINIQNLDEMVQLLISRCSCPSQFPMVK | | | Oxidation (M)[11,27] | | Mascot |

| | | | | | | | |
|---|---|---|---|---|---|---|---|
| 13 | PREDICTED: hypothetical protein LOC100536945 [Danio rerio] | gi\|326665516 | 19714 | 5.96 | 4 | 36 | 0 |

**Peptide Information**

| Calc. Mass | Obsrv. Mass | ± da | ± ppm | Start Seq. | End Seq. | Sequence | Ion Score | C. I. % | Modification | Rank | Result Type |
|---|---|---|---|---|---|---|---|---|---|---|---|
| 856.4159 | 856.5259 | 0.11 | 128 | 25 | 32 | SHDSPSVK | | | | | Mascot |

| | | | | | | | | | | | |
|---|---|---|---|---|---|---|---|---|---|---|---|
| 960.5261 | 960.4664 | -0.0597 | -62 | 2 | 9 | TWLTGNIR | | | | | Mascot |
| 960.5261 | 960.4664 | -0.0597 | -62 | 2 | 9 | TWLTGNIR | 16 | 0 | | | Mascot |
| 1385.6842 | 1385.7086 | 0.0244 | 18 | 21 | 32 | ELMRSHDSPSVK | | | | | Mascot |
| 1637.7887 | 1637.6639 | -0.1248 | -76 | 1 | 13 | MTWLTGNIRENMR | | | Oxidation (M)[1] | | Mascot |
| 1653.7836 | 1653.6521 | -0.1315 | -80 | 1 | 13 | MTWLTGNIRENMR | | | Oxidation (M)[1,12] | | Mascot |

| | | | | | | | |
|---|---|---|---|---|---|---|---|
| 14 | PREDICTED: rapamycin-insensitive companion of mTOR, partial [Danio rerio] | gi\|326667603 | 180801.9 | 7.89 | 17 | 35 | 0 |

**Peptide Information**

| Calc. Mass | Obsrv. Mass | ± da | ± ppm | Start Seq. | End Seq. | Sequence | Ion Score | C. I. % | Modification | Rank | Result Type |
|---|---|---|---|---|---|---|---|---|---|---|---|
| 870.4832 | 870.5333 | 0.0501 | 58 | 493 | 499 | WPNVNLK | | | | | Mascot |
| 1051.5782 | 1051.708 | 0.1298 | 123 | 758 | 766 | GYVSKQLEK | | | | | Mascot |
| 1075.4836 | 1075.6244 | 0.1408 | 131 | 1512 | 1521 | AISEDGPACR | | | Carbamidomethyl (C)[9] | | Mascot |
| 1354.5978 | 1354.7288 | 0.131 | 97 | 459 | 469 | DTEEALMMNLR | | | Oxidation (M)[7,8] | | Mascot |
| 1541.7312 | 1541.7784 | 0.0472 | 31 | 1049 | 1060 | TPDELRLMHCNR | | | Carbamidomethyl (C)[10] | | Mascot |
| 1541.7312 | 1541.7784 | 0.0472 | 31 | 1049 | 1060 | TPDELRLMHCNR | | | Carbamidomethyl (C)[10] | | Mascot |
| 1558.7249 | 1558.8386 | 0.1137 | 73 | 615 | 628 | CGVFQCLLNLCSMK | | | | | Mascot |
| 1558.7249 | 1558.8386 | 0.1137 | 73 | 615 | 628 | CGVFQCLLNLCSMK | | | | | Mascot |
| 1607.6794 | 1607.7671 | 0.0877 | 55 | 1378 | 1391 | FNFGESDGFLESCR | | | | | Mascot |
| 1637.7878 | 1637.6639 | -0.1239 | -76 | 199 | 212 | HNPETAEAQIKEDR | | | | | Mascot |
| 1653.8629 | 1653.6521 | -0.2108 | -127 | 656 | 670 | ILTAATDNCRLYATK | | | | | Mascot |
| 1710.8843 | 1710.8851 | 0.0008 | 0 | 656 | 670 | ILTAATDNCRLYATK | | | Carbamidomethyl (C)[9] | | Mascot |
| 2211.1987 | 2211.0938 | -0.1049 | -47 | 121 | 140 | ACIAIICELALKNPEVVAQR | | | Carbamidomethyl (C)[2] | | Mascot |
| 2224.0583 | 2224.0549 | -0.0034 | -2 | 1164 | 1184 | EPAMDTDCASLNTVVSARTVK | | | Oxidation (M)[4] | | Mascot |
| 2230.1318 | 2230.1941 | 0.0623 | 28 | 239 | 259 | SGNSGVQSLIGLLCIPNMDVR | | | Carbamidomethyl (C)[14] | | Mascot |
| 2247.0461 | 2247.0908 | 0.0447 | 20 | 611 | 628 | MLEKCGVFQCLLNLCSMK | | | Carbamidomethyl (C)[5,10,15], Oxidation (M)[1] | | Mascot |
| 2298.2312 | 2298.1726 | -0.0586 | -25 | 1254 | 1274 | LQQQRIHPSLSHSEALASPAK | | | | | Mascot |
| 2362.1418 | 2362.209 | 0.0672 | 28 | 1223 | 1243 | SPSVATIKSLVDCSYMYTSPR | | | Carbamidomethyl (C)[13] | | Mascot |
| 2362.1418 | 2362.209 | 0.0672 | 28 | 1223 | 1243 | SPSVATIKSLVDCSYMYTSPR | | | Carbamidomethyl (C)[13] | | Mascot |
| 2555.3464 | 2555.3406 | -0.0058 | -2 | 1301 | 1323 | ALSFASLDKEDLLSPINHSTLQR | | | | | Mascot |
| 3337.5789 | 3337.7546 | 0.1757 | 53 | 1329 | 1358 | SMVSSATFGCSEDYIGLALPLDINNMFQIK | | | Carbamidomethyl (C)[10], Oxidation (M)[2] | | Mascot |

| | | | | | | | |
|---|---|---|---|---|---|---|---|
| 15 | cysteinyl-tRNA synthetase, cytoplasmic [Danio rerio] | gi\|169646833 | 93752.3 | 6.07 | 12 | 35 | 0 |

**Peptide Information**

| Calc. Mass | Obsrv. Mass | ± da | ± ppm | Start Seq. | End Seq. | Sequence | Ion Score | C. I. % | Modification | Rank | Result Type |
|---|---|---|---|---|---|---|---|---|---|---|---|
| 882.5155 | 882.5638 | 0.0483 | 55 | 612 | 619 | SAKLPPNR | | | | | Mascot |
| 1075.6146 | 1075.6244 | 0.0098 | 9 | 116 | 124 | TKEVFVPQK | | | | | Mascot |
| 1128.6259 | 1128.6136 | -0.0123 | -11 | 260 | 269 | QAQVLLEEAK | | | | | Mascot |
| 1558.7278 | 1558.8386 | 0.1108 | 71 | 577 | 590 | TSVHGALCDNIDTR | | | Carbamidomethyl (C)[8] | | Mascot |
| 1558.8622 | 1558.8386 | -0.0236 | -15 | 684 | 696 | KVTELLQLCDQLR | | | | | Mascot |
| 1637.817 | 1637.6639 | -0.1531 | -93 | 92 | 106 | VQPPWSPPEGTDISK | | | | | Mascot |
| 1665.9098 | 1665.8523 | -0.0575 | -35 | 319 | 332 | VSEYVPEIVEFVKK | | | | | Mascot |
| 2225.0505 | 2225.1055 | 0.055 | 25 | 165 | 182 | YDVFYCMNITDIDDKIIK | | | Oxidation (M)[7] | | Mascot |
| 2225.0505 | 2225.1055 | 0.055 | 25 | 165 | 182 | YDVFYCMNITDIDDKIIK | | | Oxidation (M)[7] | | Mascot |
| 2230.0591 | 2230.1941 | 0.135 | 61 | 591 | 610 | SALEEMRALVGQSNTYM AAR | | | Oxidation (M)[6,17] | | Mascot |
| 2299.1499 | 2299.1731 | 0.0232 | 10 | 70 | 90 | HIAFLKPQANDQSPECST KGK | | | | | Mascot |
| 2362.2874 | 2362.209 | -0.0784 | -33 | 615 | 634 | LPPNRMLLQSIALYLTDM LK | | | Oxidation (M)[6,18] | | Mascot |
| 2362.2874 | 2362.209 | -0.0784 | -33 | 615 | 634 | LPPNRMLLQSIALYLTDM LK | | | Oxidation (M)[6,18] | | Mascot |
| 2555.3496 | 2555.3406 | -0.009 | -4 | 228 | 251 | QMLERLDSAVDAALGPL QVAVQSK | | | Oxidation (M)[2] | | Mascot |

16 ZAR1-like protein [Danio rerio] gi|313661374 36462.9 9.07 7 35 0

**Peptide Information**

| Calc. Mass | Obsrv. Mass | ± da | ± ppm | Start Seq. | End Seq. | Sequence | Ion Score | C. I. % | Modification | Rank | Result Type |
|---|---|---|---|---|---|---|---|---|---|---|---|
| 960.4533 | 960.4664 | 0.0131 | 14 | 105 | 112 | GDKWDSPR | | | | | Mascot |
| 960.4533 | 960.4664 | 0.0131 | 14 | 105 | 112 | GDKWDSPR | 12 | 0 | | | Mascot |
| 1637.7556 | 1637.6639 | -0.0917 | -56 | 259 | 271 | VESIQCQVCCKAR | | | Carbamidomethyl (C)[6,9,10] | | Mascot |
| 1653.8748 | 1653.6521 | -0.2227 | -135 | 134 | 147 | FSRPVAIYSPVFDR | | | | | Mascot |
| 1665.8192 | 1665.8523 | 0.0331 | 20 | 299 | 312 | GLSCDTIYSFKYIV | | | Carbamidomethyl (C)[4] | | Mascot |
| 2237.0549 | 2237.0667 | 0.0118 | 5 | 35 | 53 | KGNNFYIAPDAPDYFDVY K | | | | | Mascot |
| 2246.8401 | 2247.0908 | 0.2507 | 112 | 170 | 188 | TDSETEDEDDDEFCKPD EK | | | | | Mascot |
| 2299.0449 | 2299.1731 | 0.1282 | 56 | 250 | 269 | CQAGLNPYRVESIQCQV CCK | | | Carbamidomethyl (C)[1] | | Mascot |

17 PREDICTED: MAX gene-associated protein-like [Danio rerio] gi|326677444 30618 6.1 8 35 0

**Peptide Information**

| Calc. Mass | Obsrv. Mass | ± da | ± ppm | Start Seq. | End Seq. | Sequence | Ion Score | C. I. % | Modification | Rank | Result Type |
|---|---|---|---|---|---|---|---|---|---|---|---|
| 856.5614 | 856.5259 | -0.0355 | -41 | 236 | 242 | LIQNKLK | | | | | Mascot |

| | | | | | | | | | | | |
|---|---|---|---|---|---|---|---|---|---|---|---|
| 960.4203 | 960.4664 | 0.0461 | 48 | 150 | 157 | LTHDSCER | | | | | Mascot |
| 960.4203 | 960.4664 | 0.0461 | 48 | 150 | 157 | LTHDSCER | | | | | Mascot |
| 1075.4634 | 1075.6244 | 0.161 | 150 | 73 | 80 | MFPCCRFR | | | Oxidation (M)[1] | | Mascot |
| 1279.7482 | 1279.7686 | 0.0204 | 16 | 243 | 254 | LALTPARTHTAK | | | | | Mascot |
| 1320.6405 | 1320.6624 | 0.0219 | 17 | 189 | 199 | SPNVHMFTFPK | | | Oxidation (M)[6] | | Mascot |
| 1320.698 | 1320.6624 | -0.0356 | -27 | 172 | 183 | LYVAPVSACLER | | | | | Mascot |
| 2114.9634 | 2115.0486 | 0.0852 | 40 | 218 | 235 | IDCNPFMLAFREDETSAR | | | | | Mascot |
| 2194.0491 | 2194.1262 | 0.0771 | 35 | 150 | 167 | LTHDSCEREGVLLQPMHR | | | Carbamidomethyl (C)[6], Oxidation (M)[16] | | Mascot |

18 PREDICTED: hypothetical protein LOC798582 [Danio rerio] gi|125831442 154710.2 6.38 15 35 0

Peptide Information

| Calc. Mass | Obsrv. Mass | ± da | ± ppm | Start Seq. | End Seq. | Sequence | Ion Score | C. I. % | Modification | Rank | Result Type |
|---|---|---|---|---|---|---|---|---|---|---|---|
| 842.4478 | 842.5072 | 0.0594 | 71 | 242 | 250 | KSPPGGGSR | | | | | Mascot |
| 870.5519 | 870.5333 | -0.0186 | -21 | 1252 | 1258 | DVVRLLR | | | | | Mascot |
| 892.4523 | 892.4858 | 0.0335 | 38 | 296 | 302 | ESNKWTK | | | | | Mascot |
| 960.5546 | 960.4664 | -0.0882 | -92 | 924 | 932 | LITKACSPK | | | | | Mascot |
| 960.5546 | 960.4664 | -0.0882 | -92 | 924 | 932 | LITKACSPK | 4 | 0 | | | Mascot |
| 1082.5299 | 1082.6895 | 0.1596 | 147 | 76 | 85 | MQQGAIFSGK | | | Oxidation (M)[1] | | Mascot |
| 1320.5435 | 1320.6624 | 0.1189 | 90 | 1378 | 1386 | KFCCWFCGR | | | Carbamidomethyl (C)[3,4,7] | | Mascot |
| 1320.5435 | 1320.6624 | 0.1189 | 90 | 1378 | 1386 | KFCCWFCGR | | | Carbamidomethyl (C)[3,4,7] | | Mascot |
| 1653.8555 | 1653.6521 | -0.2034 | -123 | 858 | 871 | HSDAIISQQLEKER | | | | | Mascot |
| 1833.9705 | 1833.9923 | 0.0218 | 12 | 279 | 294 | TLEDTQQFLERAVGVK | | | | | Mascot |
| 1946.9786 | 1946.9719 | -0.0067 | -3 | 870 | 886 | ERMMSSAPVQETVRPTK | | | | | Mascot |
| 2194.0208 | 2194.1262 | 0.1054 | 48 | 1390 | 1407 | NQEDWIGHGQRHLMEATR | | | Oxidation (M)[14] | | Mascot |
| 2237.0222 | 2237.0667 | 0.0445 | 20 | 76 | 95 | MQQGAIFSGKILSFCCSECK | | | Carbamidomethyl (C)[15] | | Mascot |
| 2247.0498 | 2247.0908 | 0.041 | 18 | 490 | 509 | SNGRSSPPPHEMSFLYPSEK | | | | | Mascot |
| 2255.0869 | 2255.0515 | -0.0354 | -16 | 349 | 368 | ISIPPNCTTQVMGFKMVDGK | | | Carbamidomethyl (C)[7], Oxidation (M)[12,16] | | Mascot |
| 2298.1472 | 2298.1726 | 0.0254 | 11 | 973 | 994 | GEASVQTAQSGNRYFVNASALK | | | | | Mascot |
| 2362.2361 | 2362.209 | -0.0271 | -11 | 830 | 853 | KPEGPAVANENTAPSLNNAAVLGK | | | | | Mascot |
| 2362.2361 | 2362.209 | -0.0271 | -11 | 830 | 853 | KPEGPAVANENTAPSLNNAAVLGK | | | | | Mascot |

19 nucleoside diphosphate-linked moiety X motif 6 [Danio rerio] gi|148237304 36698.5 7.22 8 34 0

Peptide Information

| Calc. Mass | Obsrv. Mass | ± da | ± ppm | Start Seq. | End Seq. | Sequence | Ion Score | C. I. % | Modification | Rank | Result Type |
|---|---|---|---|---|---|---|---|---|---|---|---|
| 850.4893 | 850.5395 | 0.0502 | 59 | 243 | 249 | LQPLSHR | | | | | Mascot |
| 850.4893 | 850.5395 | 0.0502 | 59 | 243 | 249 | LQPLSHR | | | | | Mascot |
| 1354.6975 | 1354.7288 | 0.0313 | 23 | 94 | 104 | VSLHQWRSEGR | | | | | Mascot |
| 1558.7794 | 1558.8386 | 0.0592 | 38 | 13 | 26 | VSSGLCRVSSGFFR | | | Carbamidomethyl (C)[6] | | Mascot |
| 1558.7794 | 1558.8386 | 0.0592 | 38 | 13 | 26 | VSSGLCRVSSGFFR | | | Carbamidomethyl (C)[6] | | Mascot |
| 2115.0295 | 2115.0486 | 0.0191 | 9 | 2 | 19 | VLWIMSAGFCRVSSGLC R | | | Carbamidomethyl (C)[10,17], Oxidation (M)[5] | | Mascot |
| 2230.0752 | 2230.1941 | 0.1189 | 53 | 1 | 19 | MVLWIMSAGFCRVSSGL CR | | | Carbamidomethyl (C)[11,18] | | Mascot |
| 2237.0146 | 2237.0667 | 0.0521 | 23 | 51 | 70 | FCSGCEERLCPPLLAGD VDR | | | Carbamidomethyl (C)[2] | | Mascot |
| 2247.0435 | 2247.0908 | 0.0473 | 21 | 224 | 242 | QQHTHPGAFGMSDLYLI CR | | | Carbamidomethyl (C)[18], Oxidation (M)[11] | | Mascot |
| 2297.0735 | 2297.1833 | 0.1098 | 48 | 250 | 266 | IHICTHECLRCDWLDLR | | | Carbamidomethyl (C)[4,8,11] | | Mascot |

| | | | | | | | |
|---|---|---|---|---|---|---|---|
| 20 | novel protein similar to vertebrate actin filament associated protein 1-like 1 (AFAP1L1, zgc:114084 | gi\|220679093 | 84272.2 | 8.16 | 11 | 34 | 0 |

**Peptide Information**

| Calc. Mass | Obsrv. Mass | ± da | ± ppm | Start Seq. | End Seq. | Sequence | Ion Score | C. I. % | Modification | Rank | Result Type |
|---|---|---|---|---|---|---|---|---|---|---|---|
| 850.5145 | 850.5395 | 0.025 | 29 | 383 | 389 | IISFSKR | | | | | Mascot |
| 850.5145 | 850.5395 | 0.025 | 29 | 383 | 389 | IISFSKR | | | | | Mascot |
| 892.4787 | 892.4858 | 0.0071 | 8 | 226 | 232 | RFGQWAK | | | | | Mascot |
| 960.4169 | 960.4664 | 0.0495 | 52 | 399 | 406 | SSFDHDPR | | | | | Mascot |
| 960.5658 | 960.4664 | -0.0994 | -103 | 36 | 43 | MAVKNLLR | | | Oxidation (M)[1] | | Mascot |
| 996.4819 | 996.5922 | 0.1103 | 111 | 133 | 140 | MPEYITSR | | | | | Mascot |
| 996.4819 | 996.5922 | 0.1103 | 111 | 133 | 140 | MPEYITSR | | | | | Mascot |
| 1128.6483 | 1128.6136 | -0.0347 | -31 | 233 | 241 | QLTVIRENR | | | | | Mascot |
| 1833.9791 | 1833.9923 | 0.0132 | 7 | 450 | 466 | GCEVVPGLGPKHPFALR | | | Carbamidomethyl (C)[2] | | Mascot |
| 2225.0979 | 2225.1055 | 0.0076 | 3 | 681 | 701 | SLAGGEMEAPTESKPAH KTQR | | | | | Mascot |
| 2225.0979 | 2225.1055 | 0.0076 | 3 | 681 | 701 | SLAGGEMEAPTESKPAH KTQR | | | | | Mascot |
| 2237.9512 | 2238.0618 | 0.1106 | 49 | 702 | 720 | TEAQYMESFLPVNCASE MR | | | Oxidation (M)[6,18] | | Mascot |
| 2247.1975 | 2247.0908 | -0.1067 | -47 | 2 | 20 | EINSKPMELLVTELNMLL K | | | Oxidation (M)[7,16] | | Mascot |
| 2362.2429 | 2362.209 | -0.0339 | -14 | 1 | 20 | MEINSKPMELLVTELNML LK | | | Oxidation (M)[1] | | Mascot |
| 2362.2429 | 2362.209 | -0.0339 | -14 | 1 | 20 | MEINSKPMELLVTELNML LK | | | Oxidation (M)[1] | | Mascot |

| | | | | | | | |
|---|---|---|---|---|---|---|---|
| 21 | TBC1 domain family member 7 [Danio rerio] | gi\|41152120 | 33966.3 | 6.35 | 8 | 34 | 0 |

Peptide Information

| Calc. Mass | Obsrv. Mass | ± da | ± ppm | Start Seq. | End Seq. | Sequence | Ion Score | C. I. % | Modification | Rank | Result Type |
|---|---|---|---|---|---|---|---|---|---|---|---|
| 882.4832 | 882.5638 | 0.0806 | 91 | 279 | 285 | AIDLWHK | | | | | Mascot |
| 1118.5337 | 1118.6157 | 0.082 | 73 | 2 | 10 | ADDPQRNFR | | | | | Mascot |
| 1385.6995 | 1385.7086 | 0.0091 | 7 | 111 | 121 | MFQLENHTLPR | | | | | Mascot |
| 1541.8005 | 1541.7784 | -0.0221 | -14 | 111 | 122 | MFQLENHTLPRR | | | | | Mascot |
| 1541.8005 | 1541.7784 | -0.0221 | -14 | 111 | 122 | MFQLENHTLPRR | | | | | Mascot |
| 1623.7729 | 1623.7631 | -0.0098 | -6 | 210 | 223 | RCFAGCLPESSLQR | | | Carbamidomethyl (C)[2] | | Mascot |
| 1829.9366 | 1829.9438 | 0.0072 | 4 | 42 | 56 | LSTFSQRFPLPSMYR | | | | | Mascot |
| 2217.0427 | 2217.0422 | -0.0005 | 0 | 79 | 96 | QYRVDQFEDVSCALTVMR | | | Carbamidomethyl (C)[12] | | Mascot |
| 2233.0376 | 2233.0708 | 0.0332 | 15 | 79 | 96 | QYRVDQFEDVSCALTVMR | | | Carbamidomethyl (C)[12], Oxidation (M)[17] | | Mascot |
| 2255.3196 | 2255.0515 | -0.2681 | -119 | 228 | 248 | VISGSCKILVFVAVEILLSHK | | | | | Mascot |

22 novel protein similar to vertebrate actin filament associated protein 1-like 1 (AFAP1L1, zgc:114084 gi|220678265 84328.3 8.16 11 34 0

Peptide Information

| Calc. Mass | Obsrv. Mass | ± da | ± ppm | Start Seq. | End Seq. | Sequence | Ion Score | C. I. % | Modification | Rank | Result Type |
|---|---|---|---|---|---|---|---|---|---|---|---|
| 850.5145 | 850.5395 | 0.025 | 29 | 383 | 389 | IISFSKR | | | | | Mascot |
| 850.5145 | 850.5395 | 0.025 | 29 | 383 | 389 | IISFSKR | | | | | Mascot |
| 892.4787 | 892.4858 | 0.0071 | 8 | 226 | 232 | RFGQWAK | | | | | Mascot |
| 960.4169 | 960.4664 | 0.0495 | 52 | 399 | 406 | SSFDHDPR | | | | | Mascot |
| 960.5658 | 960.4664 | -0.0994 | -103 | 36 | 43 | MAVKNLLR | | | Oxidation (M)[1] | | Mascot |
| 996.4819 | 996.5922 | 0.1103 | 111 | 133 | 140 | MPEYITSR | | | | | Mascot |
| 996.4819 | 996.5922 | 0.1103 | 111 | 133 | 140 | MPEYITSR | | | | | Mascot |
| 1128.6483 | 1128.6136 | -0.0347 | -31 | 233 | 241 | QLTVIRENR | | | | | Mascot |
| 1833.9791 | 1833.9923 | 0.0132 | 7 | 450 | 466 | GCEVVPGLGPKHPFALR | | | Carbamidomethyl (C)[2] | | Mascot |
| 2225.0979 | 2225.1055 | 0.0076 | 3 | 681 | 701 | SLAGGEMEAPTESKPAHKTQR | | | | | Mascot |
| 2225.0979 | 2225.1055 | 0.0076 | 3 | 681 | 701 | SLAGGEMEAPTESKPAHKTQR | | | | | Mascot |
| 2238.1362 | 2238.0618 | -0.0744 | -33 | 250 | 269 | DQSPYTDIPLSLCSVIYVPK | | | | | Mascot |
| 2247.1975 | 2247.0908 | -0.1067 | -47 | 2 | 20 | EINSKPMELLVTELNMLLK | | | Oxidation (M)[7,16] | | Mascot |
| 2362.2429 | 2362.209 | -0.0339 | -14 | 1 | 20 | MEINSKPMELLVTELNMLLK | | | Oxidation (M)[1] | | Mascot |
| 2362.2429 | 2362.209 | -0.0339 | -14 | 1 | 20 | MEINSKPMELLVTELNMLLK | | | Oxidation (M)[1] | | Mascot |

23 PREDICTED: amine sulfotransferase [Danio rerio] gi|68372820 34418.6 8.81 7 33 0

Peptide Information

| Calc. Mass | Obsrv. Mass | ± da | ± ppm | Start Seq. | End Seq. | Sequence | Ion Score | C. I. % | Modification | Rank | Result Type |
|---|---|---|---|---|---|---|---|---|---|---|---|
| 882.4355 | 882.5638 | 0.1283 | 145 | 171 | 176 | EWYKEK | | | | | Mascot |
| 900.4897 | 900.5101 | 0.0204 | 23 | 207 | 215 | NLDAAAVAR | | | | | Mascot |
| 1128.6047 | 1128.6136 | 0.0089 | 8 | 4 | 12 | VNNYLSKYK | | | | | Mascot |
| 2211.0288 | 2211.0938 | 0.065 | 29 | 70 | 86 | YPNNLEQMPWLEYREGR | | | Oxidation (M)[8] | | Mascot |
| 2223.9863 | 2224.0549 | 0.0686 | 31 | 257 | 274 | NTFTVSQSEMFDQIFEER | | | Oxidation (M)[10] | | Mascot |
| 2362.1958 | 2362.209 | 0.0132 | 6 | 47 | 67 | SGTIWVQNIINLVCEDSLT EK | | | | | Mascot |
| 2362.1958 | 2362.209 | 0.0132 | 6 | 47 | 67 | SGTIWVQNIINLVCEDSLT EK | | | | | Mascot |
| 3337.5588 | 3337.7546 | 0.1958 | 59 | 147 | 174 | SFEDFLQQYLAGNVGGS SWFDHVKEWYK | | | | | Mascot |

24 PREDICTED: rapamycin-insensitive companion of mTOR [Danio rerio] gi|189533864 193500.8 8.2 18 33 0

Peptide Information

| Calc. Mass | Obsrv. Mass | ± da | ± ppm | Start Seq. | End Seq. | Sequence | Ion Score | C. I. % | Modification | Rank | Result Type |
|---|---|---|---|---|---|---|---|---|---|---|---|
| 864.5189 | 864.5098 | -0.0091 | -11 | 1102 | 1109 | FLISLSGK | | | | | Mascot |
| 892.492 | 892.4858 | -0.0062 | -7 | 286 | 293 | MSIVASLR | | | Oxidation (M)[1] | | Mascot |
| 1045.5887 | 1045.5601 | -0.0286 | -27 | 1652 | 1661 | GNETGLLTIK | | | | | Mascot |
| 1051.5782 | 1051.708 | 0.1298 | 123 | 822 | 830 | GYVSKQLEK | | | | | Mascot |
| 1354.5978 | 1354.7288 | 0.131 | 97 | 523 | 533 | DTEEALMMNLR | | | Oxidation (M)[7,8] | | Mascot |
| 1385.7134 | 1385.7086 | -0.0048 | -3 | 326 | 336 | GLLEVMYEIFR | | | Oxidation (M)[6] | | Mascot |
| 1541.8145 | 1541.7784 | -0.0361 | -23 | 325 | 336 | RGLLEVMYEIFR | | | Oxidation (M)[7] | | Mascot |
| 1541.8145 | 1541.7784 | -0.0361 | -23 | 325 | 336 | RGLLEVMYEIFR | | | Oxidation (M)[7] | | Mascot |
| 1558.7344 | 1558.8386 | 0.1042 | 67 | 19 | 32 | NDSGEENVPLDLTR | | | | | Mascot |
| 1558.7344 | 1558.8386 | 0.1042 | 67 | 19 | 32 | NDSGEENVPLDLTR | | | | | Mascot |
| 1623.8635 | 1623.7631 | -0.1004 | -62 | 1621 | 1634 | NIPEDAPACRVLLR | | | Carbamidomethyl (C)[9] | | Mascot |
| 1653.8629 | 1653.6521 | -0.2108 | -127 | 720 | 734 | ILTAATDNCRLYATK | | | | | Mascot |
| 1710.8843 | 1710.8851 | 0.0008 | 0 | 720 | 734 | ILTAATDNCRLYATK | | | Carbamidomethyl (C)[9] | | Mascot |
| 1819.8629 | 1819.86 | -0.0029 | -2 | 765 | 781 | AVSVEALDILDEACEDK | | | | | Mascot |
| 2034.99 | 2034.9102 | -0.0798 | -39 | 763 | 781 | SKAVSVEALDILDEACED K | | | | | Mascot |
| 2035.0826 | 2034.9102 | -0.1724 | -85 | 286 | 303 | MSIVASLRSWSGIINLCK | | | Carbamidomethyl (C)[17] | | Mascot |
| 2217.1365 | 2217.0422 | -0.0943 | -43 | 304 | 324 | SGNSGIQSLIGLLCIPNME VR | | | Oxidation (M)[18] | | Mascot |
| 2224.2019 | 2224.0549 | -0.147 | -66 | 223 | 241 | INEALMTTILHLLNHPHTR | | | | | Mascot |
| 2237.9866 | 2238.0618 | 0.0752 | 34 | 1046 | 1064 | HNSESDSIQPTMYILDDE | | | Oxidation (M)[12] | | Mascot |

| Calc. Mass | Obsrv. Mass | ± da | ± ppm | Start Seq. | End Seq. | Sequence | Ion Score | C. I. % | Modification | Rank | Result Type |
|---|---|---|---|---|---|---|---|---|---|---|---|
| | | | | | | K | | | | | |
| 2283.22 | 2283.166 | -0.054 | -24 | 186 | 205 | ACIAIICELALENPEIVAKR | | | Carbamidomethyl (C)[2,7] | | Mascot |
| 2283.22 | 2283.166 | -0.054 | -24 | 186 | 205 | ACIAIICELALENPEIVAKR | | | Carbamidomethyl (C)[2,7] | | Mascot |
| 2298.2312 | 2298.1726 | -0.0586 | -25 | 1358 | 1378 | LQQQRIHPSLSHSEALASPAK | | | | | Mascot |

| | | | | | | | |
|---|---|---|---|---|---|---|---|
| 25 | novel protein similar to vertebrate fuse-binding protein-interacting repressor (SIAHBP1) [Danio rer | gi\|169154386 | 25703.1 | 7.75 | 6 | 33 | 0 |

**Peptide Information**

| Calc. Mass | Obsrv. Mass | ± da | ± ppm | Start Seq. | End Seq. | Sequence | Ion Score | C. I. % | Modification | Rank | Result Type |
|---|---|---|---|---|---|---|---|---|---|---|---|
| 850.4273 | 850.5395 | 0.1122 | 132 | 88 | 94 | ALAIMCR | | | Carbamidomethyl (C)[6], Oxidation (M)[5] | | Mascot |
| 850.4273 | 850.5395 | 0.1122 | 132 | 88 | 94 | ALAIMCR | | | Carbamidomethyl (C)[6], Oxidation (M)[5] | | Mascot |
| 1075.5935 | 1075.6244 | 0.0309 | 29 | 111 | 120 | QAFAPFGPIK | | | | | Mascot |
| 1118.5922 | 1118.6157 | 0.0235 | 21 | 86 | 94 | QRALAIMCR | | | Carbamidomethyl (C)[8] | | Mascot |
| 2362.2473 | 2362.209 | -0.0383 | -16 | 166 | 187 | VGRPSNIGQAQPIIDQLAEEAR | | | | | Mascot |
| 2362.2473 | 2362.209 | -0.0383 | -16 | 166 | 187 | VGRPSNIGQAQPIIDQLAEEAR | | | | | Mascot |
| 2811.4192 | 2811.4519 | 0.0327 | 12 | 2 | 27 | MENGQSTASKLGLPPLTPEQQEALQK | | | Oxidation (M)[1] | | Mascot |
| 3337.6343 | 3337.7546 | 0.1203 | 36 | 133 | 162 | HKGFAFVEYEVPEAAQLALEQMNSVMLGGR | | | Oxidation (M)[22] | | Mascot |

| | | | | | | | |
|---|---|---|---|---|---|---|---|
| 26 | novel protein similar to vertebrate fuse-binding protein-interacting repressor (SIAHBP1) [Danio rer | gi\|169154387 | 26020.2 | 7.75 | 6 | 33 | 0 |

**Peptide Information**

| Calc. Mass | Obsrv. Mass | ± da | ± ppm | Start Seq. | End Seq. | Sequence | Ion Score | C. I. % | Modification | Rank | Result Type |
|---|---|---|---|---|---|---|---|---|---|---|---|
| 850.4273 | 850.5395 | 0.1122 | 132 | 88 | 94 | ALAIMCR | | | Carbamidomethyl (C)[6], Oxidation (M)[5] | | Mascot |
| 850.4273 | 850.5395 | 0.1122 | 132 | 88 | 94 | ALAIMCR | | | Carbamidomethyl (C)[6], Oxidation (M)[5] | | Mascot |
| 1075.5935 | 1075.6244 | 0.0309 | 29 | 111 | 120 | QAFAPFGPIK | | | | | Mascot |
| 1118.5922 | 1118.6157 | 0.0235 | 21 | 86 | 94 | QRALAIMCR | | | Carbamidomethyl (C)[8] | | Mascot |
| 2362.2473 | 2362.209 | -0.0383 | -16 | 166 | 187 | VGRPSNIGQAQPIIDQLAEEAR | | | | | Mascot |
| 2362.2473 | 2362.209 | -0.0383 | -16 | 166 | 187 | VGRPSNIGQAQPIIDQLAEEAR | | | | | Mascot |
| 2811.4192 | 2811.4519 | 0.0327 | 12 | 2 | 27 | MENGQSTASKLGLPPLTPEQQEALQK | | | Oxidation (M)[1] | | Mascot |
| 3337.6343 | 3337.7546 | 0.1203 | 36 | 133 | 162 | HKGFAFVEYEVPEAAQLALEQMNSVMLGGR | | | Oxidation (M)[22] | | Mascot |

| | | | | | | | |
|---|---|---|---|---|---|---|---|
| 27 | Igf1rb protein [Danio rerio] | gi\|190337705 | 155838 | 5.87 | 16 | 32 | 0 |

**Peptide Information**

| Calc. Mass | Obsrv. Mass | ± da | ± ppm | Start Seq. | End Seq. | Sequence | Ion Score | C. I. % | Modification | Rank | Result Type |
|---|---|---|---|---|---|---|---|---|---|---|---|

| Calc. Mass | Obsrv. Mass | ± da | ± ppm | Start Seq. | End Seq. | Sequence | Ion Score | C. I. % | Modification | Rank | Result Type |
|---|---|---|---|---|---|---|---|---|---|---|---|
| 900.5373 | 900.5101 | -0.0272 | -30 | 136 | 143 | NITRGAIR | | | | | Mascot |
| 908.4359 | 908.5312 | 0.0953 | 105 | 1255 | 1262 | EDLEAGFK | | | | | Mascot |
| 937.4594 | 937.4719 | 0.0125 | 13 | 985 | 991 | EKITMCR | | | Carbamidomethyl (C)[6] | | Mascot |
| 1320.7158 | 1320.6624 | -0.0534 | -40 | 378 | 389 | SSHTLTSLSFLK | | | | | Mascot |
| 1320.7158 | 1320.6624 | -0.0534 | -40 | 378 | 389 | SSHTLTSLSFLK | | | | | Mascot |
| 1341.6177 | 1341.7687 | 0.151 | 113 | 1127 | 1137 | NCMVAEDFIVK | | | Carbamidomethyl (C)[2], Oxidation (M)[3] | | Mascot |
| 1385.6842 | 1385.7086 | 0.0244 | 18 | 1368 | 1380 | NERALPLPQSSAC | | | | | Mascot |
| 1541.7377 | 1541.7784 | 0.0407 | 26 | 858 | 870 | LGTEPEKHECVSR | | | Carbamidomethyl (C)[10] | | Mascot |
| 1541.7377 | 1541.7784 | 0.0407 | 26 | 858 | 870 | LGTEPEKHECVSR | | | Carbamidomethyl (C)[10] | | Mascot |
| 1653.8918 | 1653.6521 | -0.2397 | -145 | 1018 | 1032 | VAIKTVNESASLHER | | | | | Mascot |
| 1833.9263 | 1833.9923 | 0.066 | 36 | 321 | 337 | ICDEKVIDSVDAAQSLK | | | | | Mascot |
| 2114.9487 | 2115.0486 | 0.0999 | 47 | 1138 | 1154 | IGDFGMTRDIYETDYYR | | | | | Mascot |
| 2210.8821 | 2211.0938 | 0.2117 | 96 | 300 | 320 | DESHGVSVSMLCTACDG PCDK | | | Carbamidomethyl (C)[12] | | Mascot |
| 2217.0349 | 2217.0422 | 0.0073 | 3 | 1098 | 1117 | KMIQMAGEIADGMAYLN ANK | | | Oxidation (M)[2,5,13] | | Mascot |
| 2283.8984 | 2284.1619 | 0.2635 | 115 | 300 | 320 | DESHGVSVSMLCTACDG PCDK | | | Carbamidomethyl (C)[12,15], Oxidation (M)[10] | | Mascot |
| 2298.189 | 2298.1726 | -0.0164 | -7 | 710 | 728 | AFENFLHNAIFTPRPPDR R | | | | | Mascot |
| 2298.957 | 2299.1731 | 0.2161 | 94 | 175 | 195 | QSKECGNVCPGIMEDNS QCTR | | | | | Mascot |
| 2362.1899 | 2362.209 | 0.0191 | 8 | 839 | 857 | WPEPDKPNGLIMLYEIQY R | | | | | Mascot |
| 2362.1899 | 2362.209 | 0.0191 | 8 | 839 | 857 | WPEPDKPNGLIMLYEIQY R | | | | | Mascot |
| 3338.717 | 3338.7439 | 0.0269 | 8 | 1044 | 1072 | EFNCHHVVRLLGVVSQG QPTLVIMELMTR | | | Oxidation (M)[24,27] | | Mascot |

28 Zgc:110331 [Danio rerio] gi|62202266 17382.1 8.3 6 32 0

**Peptide Information**

| Calc. Mass | Obsrv. Mass | ± da | ± ppm | Start Seq. | End Seq. | Sequence | Ion Score | C. I. % | Modification | Rank | Result Type |
|---|---|---|---|---|---|---|---|---|---|---|---|
| 882.5294 | 882.5638 | 0.0344 | 39 | 122 | 129 | ELSLPPVK | | | | | Mascot |
| 900.4971 | 900.5101 | 0.013 | 14 | 9 | 16 | CAAPQILK | | | Carbamidomethyl (C)[1] | | Mascot |
| 1045.5636 | 1045.5601 | -0.0035 | -3 | 42 | 51 | NVGSLDKNAK | | | | | Mascot |
| 1128.5432 | 1128.6136 | 0.0704 | 62 | 33 | 41 | VVDHYENPR | | | | | Mascot |
| 2194.1611 | 2194.1262 | -0.0349 | -16 | 122 | 141 | ELSLPPVKLHCSMLAEDA IK | | | | | Mascot |
| 2247.1113 | 2247.0908 | -0.0205 | -9 | 84 | 104 | FKTFGCGSAIASSSLATE WVK | | | Carbamidomethyl (C)[6] | | Mascot |

29 PREDICTED: zinc-binding protein A33-like [Danio rerio] gi|292610346 58802.6 7.49 11 32 0

**Peptide Information**

| Calc. Mass | Obsrv. Mass | ± da | ± ppm | Start | End | Sequence | Ion | C. I. % | Modification | Rank | Result Type |
|---|---|---|---|---|---|---|---|---|---|---|---|

| | | | | Seq. | Seq. | | Score | | | | |
|---|---|---|---|---|---|---|---|---|---|---|---|
| 869.4951 | 869.5234 | 0.0283 | 33 | 398 | 404 | RNPEVVR | | | | | Mascot |
| 892.5137 | 892.4858 | -0.0279 | -31 | 173 | 179 | IKVEFEK | | | | | Mascot |
| 960.4277 | 960.4664 | 0.0387 | 40 | 473 | 479 | KMSFMER | | | Oxidation (M)[2,5] | | Mascot |
| 960.4277 | 960.4664 | 0.0387 | 40 | 473 | 479 | KMSFMER | | | Oxidation (M)[2,5] | | Mascot |
| 1341.6984 | 1341.7687 | 0.0703 | 52 | 350 | 360 | HWSLGVCLELK | | | Carbamidomethyl (C)[7] | | Mascot |
| 1558.7128 | 1558.8386 | 0.1258 | 81 | 405 | 417 | VKLDYVCDDTMEK | | | | | Mascot |
| 1558.7206 | 1558.8386 | 0.118 | 76 | 423 | 436 | VSFFDASCDAVIER | | | | | Mascot |
| 1829.8044 | 1829.9438 | 0.1394 | 76 | 2 | 17 | SLEDDLSCAVCTDVFR | | | Carbamidomethyl (C)[8] | | Mascot |
| 2217.0137 | 2217.0422 | 0.0285 | 13 | 380 | 397 | ERYMYHLMTAEGCEINIK | | | Oxidation (M)[4] | | Mascot |
| 2225.0259 | 2225.1055 | 0.0796 | 36 | 95 | 111 | IELFCQNDEQLLCARCR | | | Carbamidomethyl (C)[5,13,16] | | Mascot |
| 2225.0259 | 2225.1055 | 0.0796 | 36 | 95 | 111 | IELFCQNDEQLLCARCR | | | Carbamidomethyl (C)[5,13,16] | | Mascot |
| 2233.0085 | 2233.0708 | 0.0623 | 28 | 380 | 397 | ERYMYHLMTAEGCEINIK | | | Oxidation (M)[4,8] | | Mascot |
| 2247.0928 | 2247.0908 | -0.002 | -1 | 327 | 347 | VVLGSVGYSDGDHTWEI EVGK | | | | | Mascot |
| 2282.9851 | 2283.166 | 0.1809 | 79 | 31 | 49 | QCIYDHWSSSGTRNCPIC R | | | Carbamidomethyl (C)[2] | | Mascot |
| 2282.9851 | 2283.166 | 0.1809 | 79 | 31 | 49 | QCIYDHWSSSGTRNCPIC R | | | Carbamidomethyl (C)[2] | | Mascot |

30 RecName: Full=SH3 domain-binding protein 4-A gi|123888628 108383 7.89 13 32 0

Peptide Information

| Calc. Mass | Obsrv. Mass | ± da | ± ppm | Start Seq. | End Seq. | Sequence | Ion Score | C. I. % | Modification | Rank | Result Type |
|---|---|---|---|---|---|---|---|---|---|---|---|
| 842.5822 | 842.5072 | -0.075 | -89 | 663 | 669 | LIKTVIR | | | | | Mascot |
| 850.4338 | 850.5395 | 0.1057 | 124 | 387 | 393 | LSNMEIK | | | Oxidation (M)[4] | | Mascot |
| 850.4338 | 850.5395 | 0.1057 | 124 | 387 | 393 | LSNMEIK | | | Oxidation (M)[4] | | Mascot |
| 870.4726 | 870.5333 | 0.0607 | 70 | 1 | 7 | MAAHRIR | | | Oxidation (M)[1] | | Mascot |
| 1045.4796 | 1045.5601 | 0.0805 | 77 | 791 | 799 | AELDSEPER | | | | | Mascot |
| 1075.651 | 1075.6244 | -0.0266 | -25 | 475 | 484 | KVTLGLYGPK | | | | | Mascot |
| 1623.7723 | 1623.7631 | -0.0092 | -6 | 66 | 79 | DYCPSSFTTLKFSK | | | | | Mascot |
| 1946.9316 | 1946.9719 | 0.0403 | 21 | 101 | 116 | EMGYIPSTYVQPINYR | | | Oxidation (M)[2] | | Mascot |
| 2230.2417 | 2230.1941 | -0.0476 | -21 | 842 | 860 | LIMDFVLLTTAVEVAPRW R | | | | | Mascot |
| 2284.2368 | 2284.1619 | -0.0749 | -33 | 492 | 513 | TVVAIFGHDCAPKTLLVS EVGK | | | | | Mascot |
| 2300.0686 | 2300.1689 | 0.1003 | 44 | 597 | 616 | DDQDCILAQFCVQTPQP PPK | | | Carbamidomethyl (C)[5] | | Mascot |
| 2811.3804 | 2811.4519 | 0.0715 | 25 | 593 | 616 | VQVKDDQDCILAQFCVQ TPQPPPK | | | Carbamidomethyl (C)[9,15] | | Mascot |
| 3337.6052 | 3337.7546 | 0.1494 | 45 | 298 | 327 | LARSCHDLESLGQNPGW GQTQPVETNIVCK | | | Carbamidomethyl (C)[5] | | Mascot |
| 3338.5496 | 3338.7439 | 0.1943 | 58 | 885 | 913 | TGMVDSEAMWKPAYDFL | | | Oxidation (M)[3] | | Mascot |

LTWAAQIGDSYR

| 31 | PREDICTED: histone acetyltransferase KAT2A [Danio rerio] | gi\|189516875 | 90565.6 | 9.09 | 12 | 32 | 0 |
|---|---|---|---|---|---|---|---|

**Peptide Information**

| Calc. Mass | Obsrv. Mass | ± da | ± ppm | Start Seq. | End Seq. | Sequence | Ion Score | C. I. % | Modification | Rank | Result Type |
|---|---|---|---|---|---|---|---|---|---|---|---|
| 864.476 | 864.5098 | 0.0338 | 39 | 517 | 524 | VIGGICFR | | | | | Mascot |
| 937.485 | 937.4719 | -0.0131 | -14 | 85 | 93 | NPNPPTAAR | | | | | Mascot |
| 960.5373 | 960.4664 | -0.0709 | -74 | 52 | 59 | AQVRSFPR | | | | | Mascot |
| 960.5373 | 960.4664 | -0.0709 | -74 | 52 | 59 | AQVRSFPR | | | | | Mascot |
| 1102.4987 | 1102.6257 | 0.127 | 115 | 271 | 279 | YETCQVFGR | | | | | Mascot |
| 1118.5437 | 1118.6157 | 0.072 | 64 | 445 | 453 | DETARLEER | | | | | Mascot |
| 1341.6321 | 1341.7687 | 0.1366 | 102 | 718 | 728 | SEAPDYYEVIR | | | | | Mascot |
| 1607.835 | 1607.7671 | -0.0679 | -42 | 684 | 696 | ELKDPDLLYNMLK | | | Oxidation (M)[11] | | Mascot |
| 1947.0078 | 1946.9719 | -0.0359 | -18 | 132 | 148 | LLGMVVDVENLFMSVHK | | | Oxidation (M)[4] | | Mascot |
| 2233.1194 | 2233.0708 | -0.0486 | -22 | 368 | 391 | GSSASALGVTGLDVASS EPTIGEK | | | | | Mascot |
| 2284.1323 | 2284.1619 | 0.0296 | 13 | 563 | 581 | HGILYFLTYADEYAIGYFK | | | | | Mascot |
| 2362.1721 | 2362.209 | 0.0369 | 16 | 220 | 237 | MFLLCLNYWKLETPSQF R | | | Carbamidomethyl (C)[5], Oxidation (M)[1] | | Mascot |
| 2362.1721 | 2362.209 | 0.0369 | 16 | 220 | 237 | MFLLCLNYWKLETPSQF R | | | Carbamidomethyl (C)[5], Oxidation (M)[1] | | Mascot |
| 3337.6416 | 3337.7546 | 0.113 | 34 | 517 | 546 | VIGGICFRMFPTQGFTEIV FCAVTSNEQVK | | | Oxidation (M)[9] | | Mascot |

| 32 | Cars protein [Danio rerio] | gi\|28856175 | 73800.2 | 5.84 | 11 | 32 | 0 |
|---|---|---|---|---|---|---|---|

**Peptide Information**

| Calc. Mass | Obsrv. Mass | ± da | ± ppm | Start Seq. | End Seq. | Sequence | Ion Score | C. I. % | Modification | Rank | Result Type |
|---|---|---|---|---|---|---|---|---|---|---|---|
| 882.5155 | 882.5638 | 0.0483 | 55 | 533 | 540 | SAKLPPNR | | | | | Mascot |
| 1075.6146 | 1075.6244 | 0.0098 | 9 | 37 | 45 | TKEVFVPQK | | | | | Mascot |
| 1128.6259 | 1128.6136 | -0.0123 | -11 | 181 | 190 | QAQVLLEEAK | | | | | Mascot |
| 1558.7278 | 1558.8386 | 0.1108 | 71 | 498 | 511 | TSVHGALCDNIDTR | | | Carbamidomethyl (C)[8] | | Mascot |
| 1558.8622 | 1558.8386 | -0.0236 | -15 | 605 | 617 | KVTELLQLCDQLR | | | | | Mascot |
| 1637.817 | 1637.6639 | -0.1531 | -93 | 13 | 27 | VQPPWSPPEGTDISK | | | | | Mascot |
| 1665.9098 | 1665.8523 | -0.0575 | -35 | 240 | 253 | VSEYVPEIVEFVKK | | | | | Mascot |
| 2225.0505 | 2225.1055 | 0.055 | 25 | 86 | 103 | YDVFYCMNITDIDDKIIK | | | Oxidation (M)[7] | | Mascot |
| 2225.0505 | 2225.1055 | 0.055 | 25 | 86 | 103 | YDVFYCMNITDIDDKIIK | | | Oxidation (M)[7] | | Mascot |
| 2230.0591 | 2230.1941 | 0.135 | 61 | 512 | 531 | SALEEMRALVGQSNTYM AAR | | | Oxidation (M)[6,17] | | Mascot |
| 2362.2874 | 2362.209 | -0.0784 | -33 | 536 | 555 | LPPNRMLLQSIALYLTDM | | | Oxidation (M)[6,18] | | Mascot |

| | | | | | | | | | | | |
|---|---|---|---|---|---|---|---|---|---|---|---|
| | | | | | | LK | | | | | |
| 2362.2874 | 2362.209 | -0.0784 | -33 | 536 | 555 | LPPNRMLLQSIALYLTDM LK | | | Oxidation (M)[6,18] | | Mascot |
| 2555.3496 | 2555.3406 | -0.009 | -4 | 149 | 172 | QMLERLDSAVDAALGPL QVAVQSK | | | Oxidation (M)[2] | | Mascot |

33 insulin-like growth factor 1b receptor [Danio rerio] gi|169145927 155839.9 5.87 16 32 0

Peptide Information

| Calc. Mass | Obsrv. Mass | ± da | ± ppm | Start Seq. | End Seq. | Sequence | Ion Score | C. I. % | Modification | Rank | Result Type |
|---|---|---|---|---|---|---|---|---|---|---|---|
| 900.5373 | 900.5101 | -0.0272 | -30 | 136 | 143 | NITRGAIR | | | | | Mascot |
| 908.4359 | 908.5312 | 0.0953 | 105 | 1255 | 1262 | EDLEAGFK | | | | | Mascot |
| 937.4594 | 937.4719 | 0.0125 | 13 | 985 | 991 | EKITMCR | | | Carbamidomethyl (C)[6] | | Mascot |
| 1320.7158 | 1320.6624 | -0.0534 | -40 | 378 | 389 | SSHTLTSLSFLK | | | | | Mascot |
| 1320.7158 | 1320.6624 | -0.0534 | -40 | 378 | 389 | SSHTLTSLSFLK | | | | | Mascot |
| 1341.6177 | 1341.7687 | 0.151 | 113 | 1127 | 1137 | NCMVAEDFIVK | | | Carbamidomethyl (C)[2], Oxidation (M)[3] | | Mascot |
| 1385.6842 | 1385.7086 | 0.0244 | 18 | 1368 | 1380 | NERALPLPQSSAC | | | | | Mascot |
| 1541.7377 | 1541.7784 | 0.0407 | 26 | 858 | 870 | LGTEPEKHECVSR | | | Carbamidomethyl (C)[10] | | Mascot |
| 1541.7377 | 1541.7784 | 0.0407 | 26 | 858 | 870 | LGTEPEKHECVSR | | | Carbamidomethyl (C)[10] | | Mascot |
| 1653.8918 | 1653.6521 | -0.2397 | -145 | 1018 | 1032 | VAIKTVNESASLHER | | | | | Mascot |
| 1833.9263 | 1833.9923 | 0.066 | 36 | 321 | 337 | ICDEKVIDSVDAAQSLK | | | | | Mascot |
| 2114.9487 | 2115.0486 | 0.0999 | 47 | 1138 | 1154 | IGDFGMTRDIYETDYYR | | | | | Mascot |
| 2210.8821 | 2211.0938 | 0.2117 | 96 | 300 | 320 | DESHGVSVSMLCTACDG PCDK | | | Carbamidomethyl (C)[12] | | Mascot |
| 2217.0349 | 2217.0422 | 0.0073 | 3 | 1098 | 1117 | KMIQMAGEIADGMAYLN ANK | | | Oxidation (M)[2,5,13] | | Mascot |
| 2283.8984 | 2284.1619 | 0.2635 | 115 | 300 | 320 | DESHGVSVSMLCTACDG PCDK | | | Carbamidomethyl (C)[12,15], Oxidation (M)[10] | | Mascot |
| 2298.189 | 2298.1726 | -0.0164 | -7 | 710 | 728 | AFENFLHNAIFTPRPPDR R | | | | | Mascot |
| 2298.957 | 2299.1731 | 0.2161 | 94 | 175 | 195 | QSKECGNVCPGIMEDNS QCTR | | | | | Mascot |
| 2362.1899 | 2362.209 | 0.0191 | 8 | 839 | 857 | WPEPDKPNGLIMLYEIQY R | | | | | Mascot |
| 2362.1899 | 2362.209 | 0.0191 | 8 | 839 | 857 | WPEPDKPNGLIMLYEIQY R | | | | | Mascot |
| 3338.717 | 3338.7439 | 0.0269 | 8 | 1044 | 1072 | EFNCHHVVRLLGVVSQG QPTLVIMELMTR | | | Oxidation (M)[24,27] | | Mascot |

34 structural maintenance of chromosomes protein 1A [Danio rerio] gi|239052720 143054.8 6.64 15 32 0

Peptide Information

| Calc. Mass | Obsrv. Mass | ± da | ± ppm | Start Seq. | End Seq. | Sequence | Ion Score | C. I. % | Modification | Rank | Result Type |
|---|---|---|---|---|---|---|---|---|---|---|---|
| 882.4389 | 882.5638 | 0.1249 | 142 | 555 | 561 | DCIQYIK | | | | | Mascot |
| 892.4491 | 892.4858 | 0.0367 | 41 | 269 | 275 | ELGRMMR | | | | | Mascot |

| | | | | | | | | | | | |
|---|---|---|---|---|---|---|---|---|---|---|---|
| 908.444 | 908.5312 | 0.0872 | 96 | 269 | 275 | ELGRMMR | | | Oxidation (M)[5] | | Mascot |
| 926.4876 | 926.5747 | 0.0871 | 94 | 927 | 934 | HNLLQACK | | | | | Mascot |
| 960.4632 | 960.4664 | 0.0032 | 3 | 331 | 338 | ADIEELDR | | | | | Mascot |
| 960.4632 | 960.4664 | 0.0032 | 3 | 331 | 338 | ADIEELDR | 9 | 0 | | | Mascot |
| 1045.5272 | 1045.5601 | 0.0329 | 31 | 427 | 434 | EIEENQKR | | | | | Mascot |
| 1082.5476 | 1082.6895 | 0.1419 | 131 | 97 | 106 | AIIGSSSEYR | | | | | Mascot |
| 1279.6537 | 1279.7686 | 0.1149 | 90 | 835 | 844 | VMMWEQTVKK | | | | | Mascot |
| 1341.6692 | 1341.7687 | 0.0995 | 74 | 924 | 934 | SDRHNLLQACK | | | Carbamidomethyl (C)[10] | | Mascot |
| 1541.7927 | 1541.7784 | -0.0143 | -9 | 927 | 939 | HNLLQACKMQDIK | | | | | Mascot |
| 1541.7927 | 1541.7784 | -0.0143 | -9 | 927 | 939 | HNLLQACKMQDIK | | | | | Mascot |
| 1623.8748 | 1623.7631 | -0.1117 | -69 | 700 | 713 | QVQSQAHGLQMRLK | | | | | Mascot |
| 1653.8668 | 1653.6521 | -0.2147 | -130 | 132 | 146 | NFLVFQGAVESIAMK | | | | | Mascot |
| 2216.8933 | 2217.0422 | 0.1489 | 67 | 78 | 96 | AFVSMVYCEDNGEECTF TR | | | Oxidation (M)[5] | | Mascot |
| 2224.0483 | 2224.0549 | 0.0066 | 3 | 607 | 626 | KALQYACGNALVCENVE DAR | | | Carbamidomethyl (C)[7] | | Mascot |
| 2255.1699 | 2255.0515 | -0.1184 | -53 | 862 | 880 | IIDETMAQLQDLKNQHLT K | | | Oxidation (M)[6] | | Mascot |
| 3337.4446 | 3337.7546 | 0.31 | 93 | 78 | 106 | AFVSMVYCEDNGEECTF TRAIIGSSSEYR | | | Carbamidomethyl (C)[8], Oxidation (M)[5] | | Mascot |

| | | | | | | | |
|---|---|---|---|---|---|---|---|
| 35 | PREDICTED: hypothetical protein LOC569436 [Danio rerio] | gi\|326678363 | 352771.2 | 7.92 | 28 | 32 | 0 |

**Peptide Information**

| Calc. Mass | Obsrv. Mass | ± da | ± ppm | Start Seq. | End Seq. | Sequence | Ion Score | C. I. % | Modification | Rank | Result Type |
|---|---|---|---|---|---|---|---|---|---|---|---|
| 842.4743 | 842.5072 | 0.0329 | 39 | 2664 | 2669 | HFRINR | | | | | Mascot |
| 856.4311 | 856.5259 | 0.0948 | 111 | 1282 | 1287 | ENYFRK | | | | | Mascot |
| 864.4131 | 864.5098 | 0.0967 | 112 | 603 | 610 | LGAEMSEK | | | | | Mascot |
| 869.4661 | 869.5234 | 0.0573 | 66 | 368 | 374 | EKPHKCK | | | | | Mascot |
| 900.5624 | 900.5101 | -0.0523 | -58 | 2956 | 2962 | LETLRLR | | | | | Mascot |
| 908.5273 | 908.5312 | 0.0039 | 4 | 1516 | 1523 | FLLGMSLK | | | | | Mascot |
| 926.4875 | 926.5747 | 0.0872 | 94 | 368 | 374 | EKPHKCK | | | Carbamidomethyl (C)[6] | | Mascot |
| 937.3978 | 937.4719 | 0.0741 | 79 | 401 | 407 | CEQCGKR | | | Carbamidomethyl (C)[1,4] | | Mascot |
| 960.4819 | 960.4664 | -0.0155 | -16 | 412 | 419 | STLIDHMK | | | Oxidation (M)[7] | | Mascot |
| 960.4819 | 960.4664 | -0.0155 | -16 | 412 | 419 | STLIDHMK | | | Oxidation (M)[7] | | Mascot |
| 1075.5742 | 1075.6244 | 0.0502 | 47 | 2075 | 2083 | QLRDTTVDK | | | | | Mascot |
| 1082.6569 | 1082.6895 | 0.0326 | 30 | 2288 | 2296 | NPLLQKELK | | | | | Mascot |
| 1320.6616 | 1320.6624 | 0.0008 | 1 | 3017 | 3027 | LHLYNCSITEK | | | | | Mascot |
| 1320.6616 | 1320.6624 | 0.0008 | 1 | 3017 | 3027 | LHLYNCSITEK | | | | | Mascot |

| | | | | | | | | | | | | |
|---|---|---|---|---|---|---|---|---|---|---|---|---|
| 1354.6783 | 1354.7288 | 0.0505 | 37 | 2500 | 2511 | LSRCNITAEGYK | | | | | | Mascot |
| 1558.8621 | 1558.8386 | -0.0235 | -15 | 2191 | 2204 | LLDSGVRVICEGLK | | | Carbamidomethyl (C)[10] | | | Mascot |
| 1558.8687 | 1558.8386 | -0.0301 | -19 | 2078 | 2091 | DTTVDKLAVLLQDK | | | | | | Mascot |
| 1607.8461 | 1607.7671 | -0.079 | -49 | 1624 | 1636 | SDECLFRLLEVVK | | | Carbamidomethyl (C)[4] | | | Mascot |
| 1665.8667 | 1665.8523 | -0.0144 | -9 | 2864 | 2878 | SNPSHLRELNLSGNK | | | | | | Mascot |
| 1829.8422 | 1829.9438 | 0.1016 | 56 | 269 | 283 | IHSGEKLHFCEQCGK | | | Carbamidomethyl (C)[10,13] | | | Mascot |
| 1833.9132 | 1833.9923 | 0.0791 | 43 | 2483 | 2497 | NICCLLQNVQCRLEK | | | Carbamidomethyl (C)[3] | | | Mascot |
| 2034.951 | 2034.9102 | -0.0408 | -20 | 1066 | 1082 | LLNDVYTDLYMTQCASK | | | Carbamidomethyl (C)[14] | | | Mascot |
| 2034.951 | 2034.9102 | -0.0408 | -20 | 1066 | 1082 | LLNDVYTDLYMTQCASK | | | Carbamidomethyl (C)[14] | | | Mascot |
| 2114.9607 | 2115.0486 | 0.0879 | 42 | 448 | 465 | IHSGEKPHTCDQCGRSF R | | | Carbamidomethyl (C)[10] | | | Mascot |
| 2193.8933 | 2194.1262 | 0.2329 | 106 | 238 | 255 | HMCIHSGEKPYACDQCG K | | | Carbamidomethyl (C)[3,13,16], Oxidation (M)[2] | | | Mascot |
| 2220.968 | 2221.0764 | 0.1084 | 49 | 14 | 31 | TEFIEDYSENRSDSEPFR | | | | | | Mascot |
| 2224.9797 | 2225.1055 | 0.1258 | 57 | 321 | 339 | HHMSLHNEEKPYVCGQC GR | | | | | | Mascot |
| 2224.9797 | 2225.1055 | 0.1258 | 57 | 321 | 339 | HHMSLHNEEKPYVCGQC GR | | | | | | Mascot |
| 2232.8818 | 2233.0708 | 0.189 | 85 | 181 | 199 | YHMKMHSDETAFSCEEC GK | | | | | | Mascot |
| 2238.2832 | 2238.0618 | -0.2214 | -99 | 1516 | 1534 | FLLGMSLKYNRPLFQGLL K | | | | | | Mascot |
| 2283.2068 | 2283.166 | -0.0408 | -18 | 1358 | 1375 | TLTQMYTHFLRFQILQSR | | | | | | Mascot |
| 2283.2068 | 2283.166 | -0.0408 | -18 | 1358 | 1375 | TLTQMYTHFLRFQILQSR | | | | | | Mascot |
| 2297.144 | 2297.1833 | 0.0393 | 17 | 2728 | 2748 | LNLSDCSISEEGYKALAS ALR | | | Carbamidomethyl (C)[6] | | | Mascot |
| 2297.9961 | 2298.1726 | 0.1765 | 77 | 321 | 339 | HHMSLHNEEKPYVCGQC GR | | | Carbamidomethyl (C)[14], Oxidation (M)[3] | | | Mascot |
| 2299.2017 | 2299.1731 | -0.0286 | -12 | 1358 | 1375 | TLTQMYTHFLRFQILQSR | | | Oxidation (M)[5] | | | Mascot |
| 2314.1086 | 2314.1418 | 0.0332 | 14 | 2158 | 2178 | MNNCELTEKSCSVLATVL SSK | | | Carbamidomethyl (C)[4] | | | Mascot |

| | | | | | | | | | |
|---|---|---|---|---|---|---|---|---|---|
| 36 | cullin 1-like [Danio rerio] | gi\|47087291 | 89546.8 | 8.18 | 12 | 32 | 0 | 1 | 0 |

**Peptide Information**

| Calc. Mass | Obsrv. Mass | ± da | ± ppm | Start Seq. | End Seq. | Sequence | Ion Score | C. I. % | Modification | Rank | Result Type |
|---|---|---|---|---|---|---|---|---|---|---|---|
| 864.4825 | 864.5098 | 0.0273 | 32 | 220 | 227 | GPTLSVYK | | | | | Mascot |
| 882.4501 | 882.5638 | 0.1137 | 129 | 320 | 326 | MYNLVSR | | | | | Mascot |
| 996.6564 | 996.5922 | -0.0642 | -64 | 707 | 715 | LLIQAAIVR | | | | | Mascot |
| 996.6564 | 996.5922 | -0.0642 | -64 | 707 | 715 | LLIQAAIVR | | | | | Mascot |
| 1051.5531 | 1051.708 | 0.1549 | 147 | 26 | 34 | SGIQQVYTR | | | | | Mascot |
| 1341.8099 | 1341.7687 | -0.0412 | -31 | 176 | 187 | QVTNAVLKLVEK | | | | | Mascot |
| 1385.6709 | 1385.7086 | 0.0377 | 27 | 564 | 575 | FTAFYGSRHSGR | | | | | Mascot |
| 1607.8462 | 1607.7671 | -0.0791 | -49 | 416 | 429 | SPELLARYCDSLLK | | | | | Mascot |

| Calc. Mass | Obsrv. Mass | ± da | ± ppm | Start Seq. | End Seq. | Sequence | Ion Score | C. I. % | Modification | Rank | Result Type |
|---|---|---|---|---|---|---|---|---|---|---|---|
| 1711.0728 | 1710.8851 | -0.1877 | -110 | 631 | 645 | TDILVQVLQILLKSK | | | | | Mascot |
| 1833.9857 | 1833.9923 | 0.0066 | 4 | 73 | 89 | TPTPGGAQFVGLELYKR | | | | | Mascot |
| 2221.0693 | 2221.0764 | 0.0071 | 3 | 434 | 452 | NPEEAELEDTLNQVMVVFK | | | Oxidation (M)[15] | | Mascot |
| 2247.075 | 2247.0908 | 0.0158 | 7 | 375 | 395 | YNALVMSAFNNDAGFVAALDK | | | Oxidation (M)[6] | | Mascot |
| 2283.1423 | 2283.166 | 0.0237 | 10 | 96 | 115 | SYLTNLLKDGEDLMDESVLK | 1 | 0 | | | Mascot |
| 2283.1423 | 2283.166 | 0.0237 | 10 | 96 | 115 | SYLTNLLKDGEDLMDESVLK | | | | | Mascot |
| 2299.1375 | 2299.1731 | 0.0356 | 15 | 96 | 115 | SYLTNLLKDGEDLMDESVLK | | | Oxidation (M)[14] | | Mascot |

| | | | | | | | | |
|---|---|---|---|---|---|---|---|---|
| 37 | PREDICTED: ribonuclease inhibitor-like [Danio rerio] | gi\|326678374 | 93203.1 | 5.41 | 11 | 32 | 0 | |

**Peptide Information**

| Calc. Mass | Obsrv. Mass | ± da | ± ppm | Start Seq. | End Seq. | Sequence | Ion Score | C. I. % | Modification | Rank | Result Type |
|---|---|---|---|---|---|---|---|---|---|---|---|
| 869.4661 | 869.5234 | 0.0573 | 66 | 1 | 9 | MPAGPAGIR | | | | | Mascot |
| 1320.6803 | 1320.6624 | -0.0179 | -14 | 68 | 78 | HCTLNTLMFLK | | | | | Mascot |
| 1320.6803 | 1320.6624 | -0.0179 | -14 | 68 | 78 | HCTLNTLMFLK | | | | | Mascot |
| 1653.8914 | 1653.6521 | -0.2393 | -145 | 447 | 462 | SCSVLATCLSSKTILK | | | | | Mascot |
| 1710.9128 | 1710.8851 | -0.0277 | -16 | 447 | 462 | SCSVLATCLSSKTILK | | | Carbamidomethyl (C)[2] | | Mascot |
| 2034.9987 | 2034.9102 | -0.0885 | -43 | 495 | 513 | LLCPAAEEGCQFVTGVVGK | | | Carbamidomethyl (C)[3,10] | | Mascot |
| 2034.9987 | 2034.9102 | -0.0885 | -43 | 495 | 513 | LLCPAAEEGCQFVTGVVGK | | | Carbamidomethyl (C)[3,10] | | Mascot |
| 2217.0017 | 2217.0422 | 0.0405 | 18 | 778 | 798 | LRLSCCEMTDEACSAVASALK | | | Oxidation (M)[8] | | Mascot |
| 2225.094 | 2225.1055 | 0.0115 | 5 | 814 | 832 | LENSGVKNLSDLLMYPQCK | | | Carbamidomethyl (C)[18], Oxidation (M)[14] | | Mascot |
| 2225.094 | 2225.1055 | 0.0115 | 5 | 814 | 832 | LENSGVKNLSDLLMYPQCK | | | Carbamidomethyl (C)[18], Oxidation (M)[14] | | Mascot |
| 2297.0562 | 2297.1833 | 0.1271 | 55 | 571 | 591 | SDSSLKELDMSNNNLQDSGVK | | | Oxidation (M)[10] | | Mascot |
| 2298.1316 | 2298.1726 | 0.041 | 18 | 268 | 290 | GCGLTEESCSALATVLKSNSSLK | | | | | Mascot |
| 2300.093 | 2300.1689 | 0.0759 | 33 | 438 | 458 | LNNCELTEKSCSVLATCLSSK | | | Carbamidomethyl (C)[4] | | Mascot |
| 2811.5112 | 2811.4519 | -0.0593 | -21 | 323 | 347 | FLSNFADEVCQFVTGVVGKNLLLLK | | | Carbamidomethyl (C)[10] | | Mascot |
| 3338.6758 | 3338.7439 | 0.0681 | 20 | 197 | 227 | MNQTKSLLTPSQFLSPAAEEACQFVTGVVGK | | | Carbamidomethyl (C)[22] | | Mascot |

| | | | | | | | | | | |
|---|---|---|---|---|---|---|---|---|---|---|
| 38 | Jag2 protein [Danio rerio] | gi\|112418665 | 15637.6 | 6.08 | 1 | 32 | 0 | 27 | 48.285 | |

**Peptide Information**

| Calc. Mass | Obsrv. Mass | ± da | ± ppm | Start Seq. | End Seq. | Sequence | Ion Score | C. I. % | Modification | Rank | Result Type |
|---|---|---|---|---|---|---|---|---|---|---|---|
| 960.5196 | 960.4664 | -0.0532 | -55 | 2 | 8 | WNCIRIR | | | | | Mascot |
| 960.5196 | 960.4664 | -0.0532 | -55 | 2 | 8 | WNCIRIR | 27 | 48.285 | | | Mascot |

39 novel protein [Danio rerio] gi|126632667 13693 7.88 5 32 0

Peptide Information

| Calc. Mass | Obsrv. Mass | ± da | ± ppm | Start Seq. | End Seq. | Sequence | Ion Score | C. I. % | Modification | Rank | Result Type |
|---|---|---|---|---|---|---|---|---|---|---|---|
| 937.4407 | 937.4719 | 0.0312 | 33 | 54 | 62 | AEGTSKNCK | | | | | Mascot |
| 1045.5524 | 1045.5601 | 0.0077 | 7 | 44 | 53 | AGETIDAIQK | | | | | Mascot |
| 1165.6688 | 1165.7391 | 0.0703 | 60 | 119 | 128 | SQLHNITKKP | | | | | Mascot |
| 2254.9551 | 2255.0515 | 0.0964 | 43 | 23 | 43 | SENAKCEADEENAAMLG NTNK | | | Oxidation (M)[15] | | Mascot |
| 2314.1067 | 2314.1418 | 0.0351 | 15 | 1 | 19 | FLHLQIQAQIQEPTGDCC R | | | Carbamidomethyl (C)[17,18] | | Mascot |

40 Transferrin receptor 1a [Danio rerio] gi|190337022 84943 6.38 8 31 0

Peptide Information

| Calc. Mass | Obsrv. Mass | ± da | ± ppm | Start Seq. | End Seq. | Sequence | Ion Score | C. I. % | Modification | Rank | Result Type |
|---|---|---|---|---|---|---|---|---|---|---|---|
| 842.4842 | 842.5072 | 0.023 | 27 | 66 | 72 | LNQRSPK | | | | | Mascot |
| 856.4747 | 856.5259 | 0.0512 | 60 | 344 | 351 | LGGRNSPR | | | | | Mascot |
| 960.5472 | 960.4664 | -0.0808 | -84 | 662 | 671 | AASALTSAIR | | | | | Mascot |
| 960.5472 | 960.4664 | -0.0808 | -84 | 662 | 671 | AASALTSAIR | 14 | 0 | | | Mascot |
| 1045.5935 | 1045.5601 | -0.0334 | -32 | 683 | 690 | IINNRIMR | | | Oxidation (M)[7] | | Mascot |
| 1279.6165 | 1279.7686 | 0.1521 | 119 | 731 | 742 | GGLESADIDQFK | | | | | Mascot |
| 1607.8462 | 1607.7671 | -0.0791 | -49 | 431 | 444 | TITDMIKNDGFKPK | | | | | Mascot |
| 1623.8411 | 1623.7631 | -0.078 | -48 | 431 | 444 | TITDMIKNDGFKPK | | | Oxidation (M)[5] | | Mascot |
| 1819.9734 | 1819.86 | -0.1134 | -62 | 688 | 703 | IMRVEGSLLSPYVSPR | | | Oxidation (M)[2] | | Mascot |
| 2283.0339 | 2283.166 | 0.1321 | 58 | 662 | 682 | AASALTSAIRNSDLDDME QCR | | | Oxidation (M)[17] | | Mascot |
| 2283.0339 | 2283.166 | 0.1321 | 58 | 662 | 682 | AASALTSAIRNSDLDDME QCR | | | Oxidation (M)[17] | | Mascot |

41 nitrogen fixation cluster-like [Danio rerio] gi|221307563 17420.2 8.83 6 31 0

Peptide Information

| Calc. Mass | Obsrv. Mass | ± da | ± ppm | Start Seq. | End Seq. | Sequence | Ion Score | C. I. % | Modification | Rank | Result Type |
|---|---|---|---|---|---|---|---|---|---|---|---|
| 882.5294 | 882.5638 | 0.0344 | 39 | 122 | 129 | ELSLPPVK | | | | | Mascot |
| 900.4971 | 900.5101 | 0.013 | 14 | 9 | 16 | CAAPQILK | | | Carbamidomethyl (C)[1] | | Mascot |
| 1045.5636 | 1045.5601 | -0.0035 | -3 | 42 | 51 | NVGSLDKNAK | | | | | Mascot |
| 1128.5432 | 1128.6136 | 0.0704 | 62 | 33 | 41 | VVDHYENPR | | | | | Mascot |
| 2194.1611 | 2194.1262 | -0.0349 | -16 | 122 | 141 | ELSLPPVKLHCSMLAEDA IK | | | | | Mascot |

| | | | | | | | | | | | | |
|---|---|---|---|---|---|---|---|---|---|---|---|---|
| 2247.1113 | 2247.0908 | -0.0205 | -9 | 84 | 104 | FKTFGCGSAIASSSLATE WVK | | | Carbamidomethyl (C)[6] | | Mascot |

| | | | | | | | |
|---|---|---|---|---|---|---|---|
| 42 | NADH-ubiquinone oxidoreductase 75 kDa subunit, mitochondrial [Danio rerio] | gi\|56090150 | 79382.4 | 5.82 | 11 | 31 | 0 |

Peptide Information

| Calc. Mass | Obsrv. Mass | ± da | ± ppm | Start Seq. | End Seq. | Sequence | Ion Score | C. I. % | Modification | Rank | Result Type |
|---|---|---|---|---|---|---|---|---|---|---|---|
| 842.5093 | 842.5072 | -0.0021 | -2 | 330 | 338 | VAGALQGVK | | | | | Mascot |
| 850.5257 | 850.5395 | 0.0138 | 16 | 516 | 522 | VLNVLHR | | | | | Mascot |
| 850.5257 | 850.5395 | 0.0138 | 16 | 516 | 522 | VLNVLHR | | | | | Mascot |
| 1128.6306 | 1128.6136 | -0.017 | -15 | 304 | 312 | QRLTQPMVR | | | | | Mascot |
| 1320.7747 | 1320.6624 | -0.1123 | -85 | 4 | 16 | LPAVSRALAAHSK | | | | | Mascot |
| 1320.7747 | 1320.6624 | -0.1123 | -85 | 4 | 16 | LPAVSRALAAHSK | | | | | Mascot |
| 1341.6436 | 1341.7687 | 0.1251 | 93 | 175 | 185 | TIMTRCIQCTR | | | Oxidation (M)[3] | | Mascot |
| 1354.6307 | 1354.7288 | 0.0981 | 72 | 205 | 216 | GNDMQIGTYVEK | | | | | Mascot |
| 1710.9246 | 1710.8851 | -0.0395 | -23 | 10 | 26 | ALAAHSKGSIATTNNVR | | | | | Mascot |
| 1819.8564 | 1819.86 | 0.0036 | 2 | 714 | 731 | CVKAVTEGAAAVDEPSIC | | | Carbamidomethyl (C)[1] | | Mascot |
| 1946.8987 | 1946.9719 | 0.0732 | 38 | 697 | 713 | DFYMTDPISRASPTMAK | | | Oxidation (M)[4] | | Mascot |
| 2555.3748 | 2555.3406 | -0.0342 | -13 | 339 | 364 | GSEIGAIAGGMVDAEALV ALKDLLNK | | | | | Mascot |
| 2811.5283 | 2811.4519 | -0.0764 | -27 | 330 | 359 | VAGALQGVKGSEIGAIAG GMVDAEALVALK | | | Oxidation (M)[20] | | Mascot |

| | | | | | | | |
|---|---|---|---|---|---|---|---|
| 43 | kazrin [Danio rerio] | gi\|121583740 | 48879.4 | 6.17 | 9 | 31 | 0 |

Peptide Information

| Calc. Mass | Obsrv. Mass | ± da | ± ppm | Start Seq. | End Seq. | Sequence | Ion Score | C. I. % | Modification | Rank | Result Type |
|---|---|---|---|---|---|---|---|---|---|---|---|
| 850.4265 | 850.5395 | 0.113 | 133 | 28 | 35 | SELSATSR | | | | | Mascot |
| 850.4451 | 850.5395 | 0.0944 | 111 | 390 | 397 | MSLGSLSR | | | | | Mascot |
| 856.4094 | 856.5259 | 0.1165 | 136 | 333 | 339 | ISPCHSR | | | Carbamidomethyl (C)[4] | | Mascot |
| 960.4645 | 960.4664 | 0.0019 | 2 | 205 | 211 | NYDQHRK | | | | | Mascot |
| 960.4645 | 960.4664 | 0.0019 | 2 | 205 | 211 | NYDQHRK | | | | | Mascot |
| 996.4852 | 996.5922 | 0.107 | 107 | 120 | 127 | QMKDMLSK | | | Oxidation (M)[2] | | Mascot |
| 996.4852 | 996.5922 | 0.107 | 107 | 120 | 127 | QMKDMLSK | | | Oxidation (M)[2] | | Mascot |
| 1118.5623 | 1118.6157 | 0.0534 | 48 | 162 | 171 | EALQAMKGDR | | | | | Mascot |
| 1819.9219 | 1819.86 | -0.0619 | -34 | 240 | 255 | EATEQATVLRSQIDMK | | | | | Mascot |
| 2283.0781 | 2283.166 | 0.0879 | 39 | 333 | 354 | ISPCHSRQPSIISDASAAD GDR | | | | | Mascot |
| 2283.0781 | 2283.166 | 0.0879 | 39 | 333 | 354 | ISPCHSRQPSIISDASAAD GDR | | | | | Mascot |

| Calc. Mass | Obsrv. Mass | ± da | ± ppm | Start Seq. | End Seq. | Sequence | Ion Score | C. I. % | Modification | Rank | Result Type |
|---|---|---|---|---|---|---|---|---|---|---|---|
| 2298.2161 | 2298.1726 | -0.0435 | -19 | 7 | 27 | QLAQRIDGAIQSASQEVT NLR | | | | | Mascot |

| | | | | | | | |
|---|---|---|---|---|---|---|---|
| 44 | novel protein similar to vertebrate fuse-binding protein-interacting repressor (SIAHBP1) [Danio rer | gi\|169154385 | 17912.1 | 6.73 | 5 | 31 | 0 |

Peptide Information

| Calc. Mass | Obsrv. Mass | ± da | ± ppm | Start Seq. | End Seq. | Sequence | Ion Score | C. I. % | Modification | Rank | Result Type |
|---|---|---|---|---|---|---|---|---|---|---|---|
| 850.4273 | 850.5395 | 0.1122 | 132 | 88 | 94 | ALAIMCR | | | Carbamidomethyl (C)[6], Oxidation (M)[5] | | Mascot |
| 850.4273 | 850.5395 | 0.1122 | 132 | 88 | 94 | ALAIMCR | | | Carbamidomethyl (C)[6], Oxidation (M)[5] | | Mascot |
| 1075.5935 | 1075.6244 | 0.0309 | 29 | 111 | 120 | QAFAPFGPIK | | | | | Mascot |
| 1118.5922 | 1118.6157 | 0.0235 | 21 | 86 | 94 | QRALAIMCR | | | Carbamidomethyl (C)[8] | | Mascot |
| 2811.4192 | 2811.4519 | 0.0327 | 12 | 2 | 27 | MENGQSTASKLGLPPLT PEQQEALQK | | | Oxidation (M)[1] | | Mascot |
| 3337.6343 | 3337.7546 | 0.1203 | 36 | 133 | 162 | HKGFAFVEYEVPEAAQL ALEQMNSVMLGGR | | | Oxidation (M)[22] | | Mascot |

| | | | | | | | |
|---|---|---|---|---|---|---|---|
| 45 | insulin-like growth factor 1 receptor [Danio rerio] | gi\|22036200 | 48992.8 | 4.96 | 8 | 30 | 0 |

Peptide Information

| Calc. Mass | Obsrv. Mass | ± da | ± ppm | Start Seq. | End Seq. | Sequence | Ion Score | C. I. % | Modification | Rank | Result Type |
|---|---|---|---|---|---|---|---|---|---|---|---|
| 908.4359 | 908.5312 | 0.0953 | 105 | 314 | 321 | EDLEAGFK | | | | | Mascot |
| 937.4594 | 937.4719 | 0.0125 | 13 | 44 | 50 | EKITMCR | | | Carbamidomethyl (C)[6] | | Mascot |
| 1341.6177 | 1341.7687 | 0.151 | 113 | 186 | 196 | NCMVAEDFIVK | | | Carbamidomethyl (C)[2], Oxidation (M)[3] | | Mascot |
| 1385.6842 | 1385.7086 | 0.0244 | 18 | 427 | 439 | NERALPLPQSSAC | | | | | Mascot |
| 1653.8918 | 1653.6521 | -0.2397 | -145 | 77 | 91 | VAIKTVNESASLHER | | | | | Mascot |
| 2114.9487 | 2115.0486 | 0.0999 | 47 | 197 | 213 | IGDFGMTRDIYETDYYR | | | | | Mascot |
| 2217.0349 | 2217.0422 | 0.0073 | 3 | 157 | 176 | KMIQMAGEIADGMAYLN ANK | | | Oxidation (M)[2,5,13] | | Mascot |
| 3338.717 | 3338.7439 | 0.0269 | 8 | 103 | 131 | EFNCHHVVRLLGVVSQG QPTLVIMELMTR | | | Oxidation (M)[24,27] | | Mascot |

| | | | | | | | |
|---|---|---|---|---|---|---|---|
| 46 | PREDICTED: apolipoprotein B-100 [Danio rerio] | gi\|326675669 | 478034.2 | 5.42 | 28 | 30 | 0 |

Peptide Information

| Calc. Mass | Obsrv. Mass | ± da | ± ppm | Start Seq. | End Seq. | Sequence | Ion Score | C. I. % | Modification | Rank | Result Type |
|---|---|---|---|---|---|---|---|---|---|---|---|
| 864.3879 | 864.5098 | 0.1219 | 141 | 2950 | 2956 | AQMEDVR | | | Oxidation (M)[3] | | Mascot |
| 892.496 | 892.4858 | -0.0102 | -11 | 1783 | 1789 | LKMEPFK | | | | | Mascot |
| 900.4097 | 900.5101 | 0.1004 | 112 | 2163 | 2169 | AFDEQYK | | | | | Mascot |
| 908.4909 | 908.5312 | 0.0403 | 44 | 1783 | 1789 | LKMEPFK | | | Oxidation (M)[3] | | Mascot |
| 937.4447 | 937.4719 | 0.0272 | 29 | 4167 | 4173 | EWMKEAK | | | Oxidation (M)[3] | | Mascot |
| 1045.5823 | 1045.5601 | -0.0222 | -21 | 2442 | 2450 | IAEVCRNIK | | | | | Mascot |

| | | | | | | | | | | | |
|---|---|---|---|---|---|---|---|---|---|---|---|
| 1051.5756 | 1051.708 | 0.1324 | 126 | 3778 | 3785 | HDLRQNLR | | | | | Mascot |
| 1075.651 | 1075.6244 | -0.0266 | -25 | 3465 | 3473 | SIKLPVYQK | | | | | Mascot |
| 1082.6932 | 1082.6895 | -0.0037 | -3 | 2996 | 3005 | GNSKIILPLK | | | | | Mascot |
| 1102.6038 | 1102.6257 | 0.0219 | 20 | 2442 | 2450 | IAEVCRNIK | | | Carbamidomethyl (C)[5] | | Mascot |
| 1118.6052 | 1118.6157 | 0.0105 | 9 | 274 | 283 | QSLTLQDSVK | | | | | Mascot |
| 1341.7737 | 1341.7687 | -0.005 | -4 | 2463 | 2473 | LKIQDIIEDVR | | | | | Mascot |
| 1354.7114 | 1354.7288 | 0.0174 | 13 | 1250 | 1260 | SDALLRYQFNK | | | | | Mascot |
| 1385.7899 | 1385.7086 | -0.0813 | -59 | 336 | 347 | ASLFHKLVSEVR | | | | | Mascot |
| 1541.7806 | 1541.7784 | -0.0022 | -1 | 442 | 455 | QLPQDQATSTPEVK | | | | | Mascot |
| 1541.7806 | 1541.7784 | -0.0022 | -1 | 442 | 455 | QLPQDQATSTPEVK | | | | | Mascot |
| 1558.7781 | 1558.8386 | 0.0605 | 39 | 2957 | 2970 | MEIEISHSADLIGK | | | Oxidation (M)[1] | | Mascot |
| 1558.7781 | 1558.8386 | 0.0605 | 39 | 2957 | 2970 | MEIEISHSADLIGK | | | Oxidation (M)[1] | | Mascot |
| 1665.8737 | 1665.8523 | -0.0214 | -13 | 2345 | 2358 | MMEIMDSIVSLLKR | | | | | Mascot |
| 1710.8037 | 1710.8851 | 0.0814 | 48 | 348 | 363 | GLKNETLSGAADEMMK | | | Oxidation (M)[14] | | Mascot |
| 1819.8722 | 1819.86 | -0.0122 | -7 | 1957 | 1971 | LNNHAYNQDVNIYNK | | | | | Mascot |
| 1829.9799 | 1829.9438 | -0.0361 | -20 | 3218 | 3232 | IKCPVLMMDLNQVLR | | | Carbamidomethyl (C)[3] | | Mascot |
| 2034.9991 | 2034.9102 | -0.0889 | -44 | 1955 | 1971 | SKLNNHAYNQDVNIYNK | | | | | Mascot |
| 2034.9991 | 2034.9102 | -0.0889 | -44 | 1955 | 1971 | SKLNNHAYNQDVNIYNK | | | | | Mascot |
| 2114.96 | 2115.0486 | 0.0886 | 42 | 3987 | 4003 | FFSSANEFIRTYESNMR | | | Oxidation (M)[16] | | Mascot |
| 2211.1113 | 2211.0938 | -0.0175 | -8 | 770 | 788 | TSDLKHVTQYINLCAETFK | | | | | Mascot |
| 2230.1172 | 2230.1941 | 0.0769 | 34 | 2610 | 2628 | VRQIEMPEVDPEAIFGDLR | | | Oxidation (M)[6] | | Mascot |
| 2284.1416 | 2284.1619 | 0.0203 | 9 | 2731 | 2750 | SSLEFLDYTLDAMLQIEAPK | | | | | Mascot |
| 2299.1572 | 2299.1731 | 0.0159 | 7 | 1720 | 1738 | SECMQTLNITLWTLAFRSK | | | Carbamidomethyl (C)[3] | | Mascot |
| 2300.1367 | 2300.1689 | 0.0322 | 14 | 2731 | 2750 | SSLEFLDYTLDAMLQIEAPK | | | Oxidation (M)[13] | | Mascot |
| 2314.1504 | 2314.1418 | -0.0086 | -4 | 1718 | 1736 | MRSECMQTLNITLWTLAFR | | | | | Mascot |
| 2362.0989 | 2362.209 | 0.1101 | 47 | 940 | 959 | IDSTECNRIFPGMNYCTILR | | | Oxidation (M)[13] | | Mascot |
| 2362.0989 | 2362.209 | 0.1101 | 47 | 940 | 959 | IDSTECNRIFPGMNYCTILR | | | Oxidation (M)[13] | | Mascot |
| 2811.4348 | 2811.4519 | 0.0171 | 6 | 4203 | 4226 | LNDLLMAVESYIDSLFKSYLAFMK | | | | | Mascot |
| 3337.7654 | 3337.7546 | -0.0108 | -3 | 3516 | 3544 | LFENGVSFNIPQITFGVPDLFKTIPQMIR | | | Oxidation (M)[27] | | Mascot |

47 LOC562123 protein [Danio rerio] gi|81097651 53882.6 6.12 9 30 0

Peptide Information

| Calc. Mass | Obsrv. Mass | ± da | ± ppm | Start Seq. | End Seq. | Sequence | Ion Score | C. I. % | Modification | Rank | Result Type |
|---|---|---|---|---|---|---|---|---|---|---|---|

| Calc. Mass | Obsrv. Mass | ± da | ± ppm | Start Seq. | End Seq. | Sequence | Ion Score | C. I. % | Modification | Rank | Result Type |
|---|---|---|---|---|---|---|---|---|---|---|---|
| 937.5214 | 937.4719 | -0.0495 | -53 | 312 | 319 | AVQLDHVR | | | | | Mascot |
| 1118.559 | 1118.6157 | 0.0567 | 51 | 12 | 21 | ASEPPQQHPK | | | | | Mascot |
| 1320.6715 | 1320.6624 | -0.0091 | -7 | 320 | 331 | TVVVDEADTMLK | | | | | Mascot |
| 1320.6715 | 1320.6624 | -0.0091 | -7 | 320 | 331 | TVVVDEADTMLK | | | | | Mascot |
| 1354.5613 | 1354.7288 | 0.1675 | 124 | 92 | 102 | TDNDICSLECK | | | Carbamidomethyl (C)[6,10] | | Mascot |
| 1946.8669 | 1946.9719 | 0.105 | 54 | 67 | 83 | SQRWPEPGEPVCVMCGR | | | Oxidation (M)[14] | | Mascot |
| 2035.0342 | 2034.9102 | -0.124 | -61 | 137 | 153 | QDAFISELTEEQIERVK | | | | | Mascot |
| 2035.0342 | 2034.9102 | -0.124 | -61 | 137 | 153 | QDAFISELTEEQIERVK | | | | | Mascot |
| 2224.1543 | 2224.0549 | -0.0994 | -45 | 382 | 400 | NQPCANIRQIVLWVEEPSK | | | | | Mascot |
| 2225.1169 | 2225.1055 | -0.0114 | -5 | 429 | 447 | QAEKHTTHTQHLTPHTTTR | | | | | Mascot |
| 2225.1169 | 2225.1055 | -0.0114 | -5 | 429 | 447 | QAEKHTTHTQHLTPHTTTR | | | | | Mascot |
| 2255.1699 | 2255.0515 | -0.1184 | -53 | 312 | 331 | AVQLDHVRTVVVDEADTMLK | | | Oxidation (M)[18] | | Mascot |

| | | | | | | | | |
|---|---|---|---|---|---|---|---|---|
| 48 | dehydrogenase/reductase SDR family member 1 [Danio rerio] | gi\|50345068 | 33868.3 | 8.39 | 7 | 30 | 0 | |

**Peptide Information**

| Calc. Mass | Obsrv. Mass | ± da | ± ppm | Start Seq. | End Seq. | Sequence | Ion Score | C. I. % | Modification | Rank | Result Type |
|---|---|---|---|---|---|---|---|---|---|---|---|
| 1075.5815 | 1075.6244 | 0.0429 | 40 | 232 | 240 | CIVELAKDK | | | Carbamidomethyl (C)[1] | | Mascot |
| 1320.6399 | 1320.6624 | 0.0225 | 17 | 53 | 65 | GGRCLPVVCDSSK | | | | | Mascot |
| 1320.6399 | 1320.6624 | 0.0225 | 17 | 53 | 65 | GGRCLPVVCDSSK | | | | | Mascot |
| 1607.8032 | 1607.7671 | -0.0361 | -22 | 1 | 15 | MALSGWICVVTGASR | | | Carbamidomethyl (C)[8] | | Mascot |
| 1623.7981 | 1623.7631 | -0.035 | -22 | 1 | 15 | MALSGWICVVTGASR | | | Carbamidomethyl (C)[8], Oxidation (M)[1] | | Mascot |
| 1819.9912 | 1819.86 | -0.1312 | -72 | 20 | 37 | GIALQLSEAGATVYITGR | | | | | Mascot |
| 1829.9003 | 1829.9438 | 0.0435 | 24 | 155 | 170 | YLFNVPYGVGKAACDR | | | Carbamidomethyl (C)[14] | | Mascot |
| 2230.2078 | 2230.1941 | -0.0137 | -6 | 84 | 104 | LDILVNNAYAGVQAILDNVSK | | | | | Mascot |
| 2555.4053 | 2555.3406 | -0.0647 | -25 | 142 | 165 | GLIVVISSMGGLRYLFNVPYGVGK | | | Oxidation (M)[9] | | Mascot |

| | | | | | | | | | | |
|---|---|---|---|---|---|---|---|---|---|---|
| 49 | SH3-domain GRB2-like 3 [Danio rerio] | gi\|28278936 | 43568.5 | 8.64 | 4 | 30 | 0 | 20 | 0 | |

**Peptide Information**

| Calc. Mass | Obsrv. Mass | ± da | ± ppm | Start Seq. | End Seq. | Sequence | Ion Score | C. I. % | Modification | Rank | Result Type |
|---|---|---|---|---|---|---|---|---|---|---|---|
| 856.4886 | 856.5259 | 0.0373 | 44 | 39 | 45 | KTEITHK | | | | | Mascot |
| 960.5359 | 960.4664 | -0.0695 | -72 | 176 | 184 | GKIADSEIK | | | | | Mascot |
| 960.5359 | 960.4664 | -0.0695 | -72 | 176 | 184 | GKIADSEIK | 20 | 0 | | | Mascot |
| 2284.3064 | 2284.1619 | -0.1445 | -63 | 46 | 65 | LILDIVPKTIGYLQPNPAYR | | | | | Mascot |

| Calc. Mass | Obsrv. Mass | ± da | ± ppm | Start Seq. | End Seq. | Sequence | Ion Score | C. I. % | Modification | Rank | Result Type |
|---|---|---|---|---|---|---|---|---|---|---|---|
| 2362.2625 | 2362.209 | -0.0535 | -23 | 208 | 227 | DVQRLQHLSGLLEAVIDY HR | | | | | Mascot |
| 2362.2625 | 2362.209 | -0.0535 | -23 | 208 | 227 | DVQRLQHLSGLLEAVIDY HR | | | | | Mascot |

| Rank | Protein Name | Accession No. | Protein MW | Protein PI | Pep. Count | Protein Score | Protein Score C. I. % | Total Ion Score | Total Ion C. I. % |
|---|---|---|---|---|---|---|---|---|---|
| 50 | jagged2 [Danio rerio] | gi\|15799278 | 133526.6 | 5.41 | 1 | | | 27 | 48.285 |

**Peptide Information**

| Calc. Mass | Obsrv. Mass | ± da | ± ppm | Start Seq. | End Seq. | Sequence | Ion Score | C. I. % | Modification | Rank | Result Type |
|---|---|---|---|---|---|---|---|---|---|---|---|
| 960.5196 | 960.4664 | -0.0532 | -55 | 2 | 8 | WNCIRIR | 27 | 48.285 | | 2 | Mascot |

| | | | | | |
|---|---|---|---|---|---|
| **Gel Idx/Pos** | 190/H17 | **Instr./Gel Origin** | BA2060/110707A | **Process Status** | Analysis Succeeded |
| **Plate [#] Name** | [1] 31496 | **Instrument Sample Name** | | **Spectra** | 11 |

| Rank | Protein Name | Accession No. | Protein MW | Protein PI | Pep. Count | Protein Score | Protein Score C. I. % | Total Ion Score | Total Ion C. I. % |
|---|---|---|---|---|---|---|---|---|---|
| 1 | Tkt protein [Danio rerio] | gi\|182890016 | 67962.5 | 6.79 | 22 | 1,360 | 100 | 1212 | 100 |

**Peptide Information**

| Calc. Mass | Obsrv. Mass | ± da | ± ppm | Start Seq. | End Seq. | Sequence | Ion Score | C. I. % | Modification | Rank | Result Type |
|---|---|---|---|---|---|---|---|---|---|---|---|
| 864.421 | 864.4704 | 0.0494 | 57 | 57 | 63 | YKADDPR | | | | | Mascot |
| 959.5156 | 959.5134 | -0.0022 | -2 | 93 | 100 | ESDLLNLR | | | | | Mascot |
| 1044.5472 | 1044.4929 | -0.0543 | -52 | 2 | 9 | EYHKPDKK | | | | | Mascot |
| 1103.5731 | 1103.5682 | -0.0049 | -4 | 559 | 567 | VITVEDHYK | | | | | Mascot |
| 1112.5081 | 1112.5077 | -0.0004 | 0 | 226 | 234 | YMWEAEGVK | | | | | Mascot |
| 1354.7689 | 1354.7678 | -0.0011 | -1 | 174 | 185 | LDNLVAILDVNR | | | | | Mascot |
| 1354.7689 | 1354.7678 | -0.0011 | -1 | 174 | 185 | LDNLVAILDVNR | 108 | 100 | | | Mascot |
| 1436.738 | 1436.7465 | 0.0085 | 6 | 101 | 113 | KIDSDLEGHPTPK | | | | | Mascot |
| 1488.7845 | 1488.7856 | 0.0011 | 1 | 385 | 398 | TVSFASTFAAFLAR | 120 | 100 | | | Mascot |
| 1488.7845 | 1488.7856 | 0.0011 | 1 | 385 | 398 | TVSFASTFAAFLAR | | | | | Mascot |
| 1719.8547 | 1719.8544 | -0.0003 | 0 | 475 | 489 | TSRPDTAVIYNPEEK | | | | | Mascot |
| 1719.8547 | 1719.8544 | -0.0003 | 0 | 475 | 489 | TSRPDTAVIYNPEEK | 65 | 99.992 | | | Mascot |
| 1736.8887 | 1736.8861 | -0.0026 | -1 | 598 | 613 | SGKPQELLDMFGISAK | | | Oxidation (M)[10] | | Mascot |
| 1751.817 | 1751.814 | -0.003 | -2 | 252 | 266 | GIEDQDNWHGKPMPK | | | | | Mascot |
| 1767.8119 | 1767.8112 | -0.0007 | 0 | 252 | 266 | GIEDQDNWHGKPMPK | | | Oxidation (M)[13] | | Mascot |
| 1893.9817 | 1893.9828 | 0.0011 | 1 | 186 | 202 | LGQSEPAPLQHNVNVYK | | | | | Mascot |
| 1893.9817 | 1893.9828 | 0.0011 | 1 | 186 | 202 | LGQSEPAPLQHNVNVYK | 97 | 100 | | | Mascot |
| 1901.9908 | 1901.9904 | -0.0004 | 0 | 75 | 92 | GHAAPILYAAWAEAGYIK | | | | | Mascot |
| 1999.943 | 1999.9437 | 0.0007 | 0 | 442 | 459 | AIPTCTVFYPSDGVSTER | 127 | 100 | Carbamidomethyl (C)[5] | | Mascot |

| | | | | | | | | | | | |
|---|---|---|---|---|---|---|---|---|---|---|---|
| 1999.943 | 1999.9437 | 0.0007 | 0 | 442 | 459 | AIPTCTVFYPSDGVSTER | | | Carbamidomethyl (C)[5] | | Mascot |
| 2114.1855 | 2114.187 | 0.0015 | 1 | 534 | 553 | VIDPFTIKPLDASTIVASAR | 145 | 100 | | | Mascot |
| 2114.1855 | 2114.187 | 0.0015 | 1 | 534 | 553 | VIDPFTIKPLDASTIVASAR | | | | | Mascot |
| 2175.1404 | 2175.1401 | -0.0003 | 0 | 568 | 589 | EGGLGEAVLSAVGEEPGIVVHR | 208 | 100 | | | Mascot |
| 2175.1404 | 2175.1401 | -0.0003 | 0 | 568 | 589 | EGGLGEAVLSAVGEEPGIVVHR | | | | | Mascot |
| 2294.1663 | 2294.1646 | -0.0017 | -1 | 475 | 494 | TSRPDTAVIYNPEEKFEIGK | | | | | Mascot |
| 2294.1663 | 2294.1646 | -0.0017 | -1 | 475 | 494 | TSRPDTAVIYNPEEKFEIGK | 29 | 65.921 | | | Mascot |
| 2432.1406 | 2432.1375 | -0.0031 | -1 | 362 | 382 | YIECFIAEQNMVSVAIGCATR | | | Carbamidomethyl (C)[4,18] | | Mascot |
| 2432.1406 | 2432.1375 | -0.0031 | -1 | 362 | 382 | YIECFIAEQNMVSVAIGCATR | 181 | 100 | Carbamidomethyl (C)[4,18] | | Mascot |
| 2448.1355 | 2448.1428 | 0.0073 | 3 | 362 | 382 | YIECFIAEQNMVSVAIGCATR | | | Carbamidomethyl (C)[4,18], Oxidation (M)[11] | | Mascot |
| 2457.3096 | 2457.3127 | 0.0031 | 1 | 504 | 527 | DQVTVIGAGVTLHEALAAHDQLAK | | | | | Mascot |
| 2457.3096 | 2457.3127 | 0.0031 | 1 | 504 | 527 | DQVTVIGAGVTLHEALAAHDQLAK | 133 | 100 | | | Mascot |
| 2515.2319 | 2515.2405 | 0.0086 | 3 | 114 | 139 | LAFVDVATGSLGQGLGAACGMAYTGK | | | Carbamidomethyl (C)[19] | | Mascot |
| 2590.3398 | 2590.2161 | -0.1237 | -48 | 294 | 317 | EDAPPADLSPIHLLTAPEYKLGDK | | | | | Mascot |
| 3000.2307 | 3000.2456 | 0.0149 | 5 | 148 | 173 | VYCMLGDGECSEGSVWEAMAFASHYK | | | Carbamidomethyl (C)[3,10], Oxidation (M)[4] | | Mascot |

2 transketolase-like protein 2 [Danio rerio] gi|186288308 67795.3 6.81 21 1,350 100 1209 100

**Peptide Information**

| Calc. Mass | Obsrv. Mass | ± da | ± ppm | Start Seq. | End Seq. | Sequence | Ion Score | C. I. % | Modification | Rank | Result Type |
|---|---|---|---|---|---|---|---|---|---|---|---|
| 901.5101 | 901.5057 | -0.0044 | -5 | 93 | 100 | ASDLLNLR | | | | | Mascot |
| 1044.4932 | 1044.4929 | -0.0003 | 0 | 226 | 234 | AMWHAEGVK | | | Oxidation (M)[2] | | Mascot |
| 1103.5731 | 1103.5682 | -0.0049 | -4 | 559 | 567 | VITVEDHYK | | | | | Mascot |
| 1354.7689 | 1354.7678 | -0.0011 | -1 | 174 | 185 | LDNLVAILDVNR | | | | | Mascot |
| 1354.7689 | 1354.7678 | -0.0011 | -1 | 174 | 185 | LDNLVAILDVNR | 108 | 100 | | | Mascot |
| 1436.738 | 1436.7465 | 0.0085 | 6 | 101 | 113 | KIDSDLEGHPTPK | | | | | Mascot |
| 1488.7845 | 1488.7856 | 0.0011 | 1 | 385 | 398 | TVSFASTFAAFLAR | 120 | 100 | | | Mascot |
| 1488.7845 | 1488.7856 | 0.0011 | 1 | 385 | 398 | TVSFASTFAAFLAR | | | | | Mascot |
| 1719.8547 | 1719.8544 | -0.0003 | 0 | 475 | 489 | TSRPDTAVIYNPEEK | | | | | Mascot |
| 1719.8547 | 1719.8544 | -0.0003 | 0 | 475 | 489 | TSRPDTAVIYNPEEK | 65 | 99.992 | | | Mascot |
| 1736.8887 | 1736.8861 | -0.0026 | -1 | 598 | 613 | SGKPQELLDMFGISAK | | | Oxidation (M)[10] | | Mascot |
| 1751.817 | 1751.814 | -0.003 | -2 | 252 | 266 | GIEDQDNWHGKPMPK | | | | | Mascot |
| 1767.8119 | 1767.8112 | -0.0007 | 0 | 252 | 266 | GIEDQDNWHGKPMPK | | | Oxidation (M)[13] | | Mascot |
| 1893.9817 | 1893.9828 | 0.0011 | 1 | 186 | 202 | LGQSEPAPLQHNVNVYK | | | | | Mascot |

| | | | | | | | | | | | |
|---|---|---|---|---|---|---|---|---|---|---|---|
| 1893.9817 | 1893.9828 | 0.0011 | 1 | 186 | 202 | LGQSEPAPLQHNVNVYK | 97 | 100 | | | Mascot |
| 1901.9908 | 1901.9904 | -0.0004 | 0 | 75 | 92 | GHAAPILYAAWAEAGYIK | | | | | Mascot |
| 1999.943 | 1999.9437 | 0.0007 | 0 | 442 | 459 | AIPTCTVFYPSDGVSTER | 127 | 100 | Carbamidomethyl (C)[5] | | Mascot |
| 1999.943 | 1999.9437 | 0.0007 | 0 | 442 | 459 | AIPTCTVFYPSDGVSTER | | | Carbamidomethyl (C)[5] | | Mascot |
| 2114.1855 | 2114.187 | 0.0015 | 1 | 534 | 553 | VIDPFTIKPLDASTIVASA R | 145 | 100 | | | Mascot |
| 2114.1855 | 2114.187 | 0.0015 | 1 | 534 | 553 | VIDPFTIKPLDASTIVASA R | | | | | Mascot |
| 2175.1404 | 2175.1401 | -0.0003 | 0 | 568 | 589 | EGGLGEAVLSAVGEEPGI VVHR | 208 | 100 | | | Mascot |
| 2175.1404 | 2175.1401 | -0.0003 | 0 | 568 | 589 | EGGLGEAVLSAVGEEPGI VVHR | | | | | Mascot |
| 2294.1663 | 2294.1646 | -0.0017 | -1 | 475 | 494 | TSRPDTAVIYNPEEKFEIG K | | | | | Mascot |
| 2294.1663 | 2294.1646 | -0.0017 | -1 | 475 | 494 | TSRPDTAVIYNPEEKFEIG K | 29 | 65.921 | | | Mascot |
| 2432.1406 | 2432.1375 | -0.0031 | -1 | 362 | 382 | YIECFIAEQNMVSVAIGCA TR | | | Carbamidomethyl (C)[4,18] | | Mascot |
| 2432.1406 | 2432.1375 | -0.0031 | -1 | 362 | 382 | YIECFIAEQNMVSVAIGCA TR | 181 | 100 | Carbamidomethyl (C)[4,18] | | Mascot |
| 2448.1355 | 2448.1428 | 0.0073 | 3 | 362 | 382 | YIECFIAEQNMVSVAIGCA TR | | | Carbamidomethyl (C)[4,18], Oxidation (M)[11] | | Mascot |
| 2457.3096 | 2457.3127 | 0.0031 | 1 | 504 | 527 | DQVTVIGAGVTLHEALAA HDQLAK | | | | | Mascot |
| 2457.3096 | 2457.3127 | 0.0031 | 1 | 504 | 527 | DQVTVIGAGVTLHEALAA HDQLAK | 133 | 100 | | | Mascot |
| 2489.0747 | 2489.0876 | 0.0129 | 5 | 205 | 225 | CEAFGFNTYVVDGHDVE ELCK | | | Carbamidomethyl (C)[1,20] | | Mascot |
| 2515.2319 | 2515.2405 | 0.0086 | 3 | 114 | 139 | LAFVDVATGSLGQGLGA ACGMAYTGK | | | Carbamidomethyl (C)[19] | | Mascot |
| 2590.3398 | 2590.2161 | -0.1237 | -48 | 294 | 317 | EDAPPADLSPIHLLTAPE YKLGDK | | | | | Mascot |
| 3000.2307 | 3000.2456 | 0.0149 | 5 | 148 | 173 | VYCMLGDGECSEGSVW EAMAFASHYK | | | Carbamidomethyl (C)[3,10], Oxidation (M)[4] | | Mascot |

| | | | | | | | | | | |
|---|---|---|---|---|---|---|---|---|---|---|
| 3 | transketolase [Danio rerio] | gi\|31872040 | 67811.3 | 6.81 | 20 | 1,210 | 100 | 1082 | 100 | |

Peptide Information

| Calc. Mass | Obsrv. Mass | ± da | ± ppm | Start Seq. | End Seq. | Sequence | Ion Score | C. I. % | Modification | Rank | Result Type |
|---|---|---|---|---|---|---|---|---|---|---|---|
| 901.5101 | 901.5057 | -0.0044 | -5 | 93 | 100 | ASDLLNLR | | | | | Mascot |
| 1044.4932 | 1044.4929 | -0.0003 | 0 | 226 | 234 | AMWHAEGVK | | | Oxidation (M)[2] | | Mascot |
| 1103.5731 | 1103.5682 | -0.0049 | -4 | 559 | 567 | VITVEDHYK | | | | | Mascot |
| 1354.7689 | 1354.7678 | -0.0011 | -1 | 174 | 185 | LDNLVAILDVNR | | | | | Mascot |
| 1354.7689 | 1354.7678 | -0.0011 | -1 | 174 | 185 | LDNLVAILDVNR | 108 | 100 | | | Mascot |
| 1436.738 | 1436.7465 | 0.0085 | 6 | 101 | 113 | KIDSDLEGHPTPK | | | | | Mascot |
| 1488.7845 | 1488.7856 | 0.0011 | 1 | 385 | 398 | TVSFASTFAAFLAR | 120 | 100 | | | Mascot |
| 1488.7845 | 1488.7856 | 0.0011 | 1 | 385 | 398 | TVSFASTFAAFLAR | | | | | Mascot |
| 1719.8547 | 1719.8544 | -0.0003 | 0 | 475 | 489 | TSRPDTAVIYNPEEK | | | | | Mascot |

| | | | | | | | | | | | |
|---|---|---|---|---|---|---|---|---|---|---|---|
| 1719.8547 | 1719.8544 | -0.0003 | 0 | 475 | 489 | TSRPDTAVIYNPEEK | 65 | 99.992 | | | Mascot |
| 1736.8887 | 1736.8861 | -0.0026 | -1 | 598 | 613 | SGKPQELLDMFGISAK | | | Oxidation (M)[10] | | Mascot |
| 1751.817 | 1751.814 | -0.003 | -2 | 252 | 266 | GIEDQDNWHGKPMPK | | | | | Mascot |
| 1767.8119 | 1767.8112 | -0.0007 | 0 | 252 | 266 | GIEDQDNWHGKPMPK | | | Oxidation (M)[13] | | Mascot |
| 1893.9817 | 1893.9828 | 0.0011 | 1 | 186 | 202 | LGQSEPAPLQHNVNVYK | | | | | Mascot |
| 1893.9817 | 1893.9828 | 0.0011 | 1 | 186 | 202 | LGQSEPAPLQHNVNVYK | 97 | 100 | | | Mascot |
| 1901.9908 | 1901.9904 | -0.0004 | 0 | 75 | 92 | GHAAPILYAAWAEAGYIK | | | | | Mascot |
| 2114.1855 | 2114.187 | 0.0015 | 1 | 534 | 553 | VIDPFTIKPLDASTIVASAR | 145 | 100 | | | Mascot |
| 2114.1855 | 2114.187 | 0.0015 | 1 | 534 | 553 | VIDPFTIKPLDASTIVASAR | | | | | Mascot |
| 2175.1404 | 2175.1401 | -0.0003 | 0 | 568 | 589 | EGGLGEAVLSAVGEEPGIVVHR | 208 | 100 | | | Mascot |
| 2175.1404 | 2175.1401 | -0.0003 | 0 | 568 | 589 | EGGLGEAVLSAVGEEPGIVVHR | | | | | Mascot |
| 2294.1663 | 2294.1646 | -0.0017 | -1 | 475 | 494 | TSRPDTAVIYNPEEKFEIGK | | | | | Mascot |
| 2294.1663 | 2294.1646 | -0.0017 | -1 | 475 | 494 | TSRPDTAVIYNPEEKFEIGK | 29 | 65.921 | | | Mascot |
| 2432.1406 | 2432.1375 | -0.0031 | -1 | 362 | 382 | YIECFIAEQNMVSVAIGCATR | | | Carbamidomethyl (C)[4,18] | | Mascot |
| 2432.1406 | 2432.1375 | -0.0031 | -1 | 362 | 382 | YIECFIAEQNMVSVAIGCATR | 181 | 100 | Carbamidomethyl (C)[4,18] | | Mascot |
| 2448.1355 | 2448.1428 | 0.0073 | 3 | 362 | 382 | YIECFIAEQNMVSVAIGCATR | | | Carbamidomethyl (C)[4,18], Oxidation (M)[11] | | Mascot |
| 2457.3096 | 2457.3127 | 0.0031 | 1 | 504 | 527 | DQVTVIGAGVTLHEALAAHDQLAK | | | | | Mascot |
| 2457.3096 | 2457.3127 | 0.0031 | 1 | 504 | 527 | DQVTVIGAGVTLHEALAAHDQLAK | 133 | 100 | | | Mascot |
| 2489.0747 | 2489.0876 | 0.0129 | 5 | 205 | 225 | CEAFGFNTYVVDGHDVEELCK | | | Carbamidomethyl (C)[1,20] | | Mascot |
| 2515.2319 | 2515.2405 | 0.0086 | 3 | 114 | 139 | LAFVDVATGSLGQGLGAACGMAYTGK | | | Carbamidomethyl (C)[19] | | Mascot |
| 2590.3398 | 2590.2161 | -0.1237 | -48 | 294 | 317 | EDAPPADLSPIHLLTAPEYKLGDK | | | | | Mascot |
| 3000.2307 | 3000.2456 | 0.0149 | 5 | 148 | 173 | VYCMLGDGECSEGSVWEAMAFASHYK | | | Carbamidomethyl (C)[3,10], Oxidation (M)[4] | | Mascot |

4 PREDICTED: calcium/calmodulin-dependent 3',5'-cyclic nucleotide phosphodiesterase 1C-like, partial gi|326681376 45655.3 5.68 11 50 53.402

**Peptide Information**

| Calc. Mass | Obsrv. Mass | ± da | ± ppm | Start Seq. | End Seq. | Sequence | Ion Score | C. I. % | Modification | Rank | Result Type |
|---|---|---|---|---|---|---|---|---|---|---|---|
| 856.5515 | 856.5234 | -0.0281 | -33 | 27 | 32 | IWLRLR | | | | | Mascot |
| 864.4429 | 864.4704 | 0.0275 | 32 | 104 | 110 | QMGLMLR | | | Oxidation (M)[2] | | Mascot |
| 901.5651 | 901.5057 | -0.0594 | -66 | 33 | 40 | GLLRCLVK | | | | | Mascot |
| 1045.5823 | 1045.562 | -0.0203 | -19 | 37 | 44 | CLVKQLER | | | Carbamidomethyl (C)[1] | | Mascot |
| 1062.5691 | 1062.6919 | 0.1228 | 116 | 112 | 119 | TEEKPRFR | | | | | Mascot |
| 1908.9185 | 1908.9324 | 0.0139 | 7 | 2 | 17 | ESPTREIEEFENTSLK | | | | | Mascot |

| | | | | | | | | | | | |
|---|---|---|---|---|---|---|---|---|---|---|---|
| 1923.9368 | 1923.9685 | 0.0317 | 16 | 299 | 314 | LLQDDDEMNILYNLSK | | | | | Mascot |
| 2283.2634 | 2283.1833 | -0.0801 | -35 | 193 | 213 | FKIPVSALVSFVEALEVG YSK | | | | | Mascot |
| 2306.134 | 2306.1567 | 0.0227 | 10 | 322 | 341 | ALVVEMVLATDMSCHFQ QVK | | | Carbamidomethyl (C)[14] | | Mascot |
| 2489.2002 | 2489.0876 | -0.1126 | -45 | 73 | 94 | RLVDTEDELSDIQSDSVP SEVR | | | | | Mascot |
| 2590.2644 | 2590.2161 | -0.0483 | -19 | 276 | 298 | SDAAILYNDRSVLESHHV SASYR | | | | | Mascot |

| | | | | | | | |
|---|---|---|---|---|---|---|---|
| 5 | purine nucleoside phosphorylase 5b [Danio rerio] | gi\|52219108 | 32118.2 | 6.31 | 9 | 45 | 0 |

Peptide Information

| Calc. Mass | Obsrv. Mass | ± da | ± ppm | Start Seq. | End Seq. | Sequence | Ion Score | C. I. % | Modification | Rank | Result Type |
|---|---|---|---|---|---|---|---|---|---|---|---|
| 1151.5118 | 1151.6196 | 0.1078 | 94 | 79 | 88 | GKPCVCMQGR | | | Carbamidomethyl (C)[4], Oxidation (M)[7] | | Mascot |
| 1736.752 | 1736.8861 | 0.1341 | 77 | 159 | 172 | FGVRFPCMSDAYDR | | | Carbamidomethyl (C)[7], Oxidation (M)[8] | | Mascot |
| 1893.986 | 1893.9828 | -0.0032 | -2 | 72 | 88 | LVFGTLKGKPCVCMQGR | | | Carbamidomethyl (C)[11] | | Mascot |
| 1894.0577 | 1893.9828 | -0.0749 | -40 | 25 | 44 | APERPLVGIVCGSGLGGL AK | | | | | Mascot |
| 1999.9429 | 1999.9437 | 0.0008 | 0 | 193 | 211 | DGVYSVLGGPSFETIAEC R | | | | | Mascot |
| 1999.9429 | 1999.9437 | 0.0008 | 0 | 193 | 211 | DGVYSVLGGPSFETIAEC R | | | | | Mascot |
| 2113.9795 | 2114.187 | 0.2075 | 98 | 163 | 179 | FPCMSDAYDRDLQQLVR | | | Carbamidomethyl (C)[3] | | Mascot |
| 2113.9795 | 2114.187 | 0.2075 | 98 | 163 | 179 | FPCMSDAYDRDLQQLVR | | | Carbamidomethyl (C)[3] | | Mascot |
| 2207.1377 | 2207.1174 | -0.0203 | -9 | 109 | 129 | LMGVETVILTNAAGGLNQ DFK | | | Oxidation (M)[2] | | Mascot |
| 2293.2444 | 2293.0771 | -0.1673 | -73 | 212 | 233 | ALRQLGADAVGMSTVHE VIVAR | | | | | Mascot |
| 2479.0969 | 2479.2808 | 0.1839 | 74 | 2 | 23 | TPDSATGYSYEECTATA DWLLK | | | Carbamidomethyl (C)[13] | | Mascot |

| | | | | | | | |
|---|---|---|---|---|---|---|---|
| 6 | novel protein [Danio rerio] | gi\|148725299 | 82126.8 | 6.15 | 13 | 45 | 0 |

Peptide Information

| Calc. Mass | Obsrv. Mass | ± da | ± ppm | Start Seq. | End Seq. | Sequence | Ion Score | C. I. % | Modification | Rank | Result Type |
|---|---|---|---|---|---|---|---|---|---|---|---|
| 901.4737 | 901.5057 | 0.032 | 35 | 75 | 81 | ILQEENR | | | | | Mascot |
| 959.4614 | 959.5134 | 0.052 | 54 | 347 | 354 | IPADCIDR | | | Carbamidomethyl (C)[5] | | Mascot |
| 1045.5272 | 1045.562 | 0.0348 | 33 | 600 | 608 | GLLTQQDDR | | | | | Mascot |
| 1062.5803 | 1062.6919 | 0.1116 | 105 | 442 | 449 | FQIQQSRR | | | | | Mascot |
| 1210.6791 | 1210.5983 | -0.0808 | -67 | 382 | 391 | EIIDHIKQSK | | | | | Mascot |
| 1436.7631 | 1436.7465 | -0.0166 | -12 | 609 | 620 | DQSKEEIIAYIK | | | | | Mascot |
| 2114.0876 | 2114.187 | 0.0994 | 47 | 566 | 584 | AAVDKALESENGHLDLYL R | | | | | Mascot |
| 2114.0876 | 2114.187 | 0.0994 | 47 | 566 | 584 | AAVDKALESENGHLDLYL R | | | | | Mascot |
| 2136.072 | 2136.1672 | 0.0952 | 45 | 43 | 60 | HKSFTDNLQSIFQNLESK | | | | | Mascot |

| | | | | | | | | | | | | |
|---|---|---|---|---|---|---|---|---|---|---|---|---|
| 2211.2092 | 2211.1079 | -0.1013 | -46 | 93 | 111 | SIITEAALDLTLFFLREMK | | | | | Mascot |
| 2294.0864 | 2294.1646 | 0.0782 | 34 | 491 | 511 | DCGIDVYKASVYSGMCTQILK | | | | | Mascot |
| 2294.0864 | 2294.1646 | 0.0782 | 34 | 491 | 511 | DCGIDVYKASVYSGMCTQILK | | | | | Mascot |
| 2298.1812 | 2298.1887 | 0.0075 | 3 | 431 | 448 | TLTQMYTHFLRFQIQQSR | | | | | Mascot |
| 2306.1008 | 2306.1567 | 0.0559 | 24 | 480 | 498 | NNLIFYESDLKDCGIDVYK | | | Carbamidomethyl (C)[13] | | Mascot |
| 2341.2188 | 2341.1233 | -0.0955 | -41 | 472 | 490 | LAFQQLERNNLIFYESDLK | | | | | Mascot |

7 hypothetical protein LOC799147 [Danio rerio] gi|130487208 15157.6 7.64 6 43 0

Peptide Information

| Calc. Mass | Obsrv. Mass | ± da | ± ppm | Start Seq. | End Seq. | Sequence | Ion Score | C. I. % | Modification | Rank | Result Type |
|---|---|---|---|---|---|---|---|---|---|---|---|
| 842.477 | 842.5089 | 0.0319 | 38 | 124 | 130 | KGSLFYK | | | | | Mascot |
| 959.4879 | 959.5134 | 0.0255 | 27 | 1 | 8 | MAAFNHLR | | | | | Mascot |
| 2197.0991 | 2197.1208 | 0.0217 | 10 | 104 | 123 | LGTCTKTVEYGLSSVTCHLK | | | Carbamidomethyl (C)[4] | | Mascot |
| 2207.2104 | 2207.1174 | -0.093 | -42 | 9 | 29 | LVSLAVLMLASQLDFASASVR | | | Oxidation (M)[8] | | Mascot |
| 2225.0081 | 2225.1235 | 0.1154 | 52 | 68 | 86 | DNANPVWSAEFNFPNCKSK | | | Carbamidomethyl (C)[16] | | Mascot |
| 3000.645 | 3000.2456 | -0.3994 | -133 | 2 | 29 | AAFNHLRLVSLAVLMLASQLDFASASVR | | | | | Mascot |

8 Ighmbp2l protein [Danio rerio] gi|215539488 110340.2 9 14 40 0

Peptide Information

| Calc. Mass | Obsrv. Mass | ± da | ± ppm | Start Seq. | End Seq. | Sequence | Ion Score | C. I. % | Modification | Rank | Result Type |
|---|---|---|---|---|---|---|---|---|---|---|---|
| 882.4389 | 882.5677 | 0.1288 | 146 | 393 | 400 | CILAGDYK | | | | | Mascot |
| 901.5464 | 901.5057 | -0.0407 | -45 | 850 | 857 | SQSVKKPK | | | | | Mascot |
| 996.4818 | 996.5963 | 0.1145 | 115 | 893 | 901 | AESVCGFVK | | | Carbamidomethyl (C)[5] | | Mascot |
| 1210.5773 | 1210.5983 | 0.021 | 17 | 297 | 306 | DMDKAFNEIK | | | | | Mascot |
| 1436.7743 | 1436.7465 | -0.0278 | -19 | 944 | 957 | ISKEGVLYAGSGQK | | | | | Mascot |
| 1767.8879 | 1767.8112 | -0.0767 | -43 | 904 | 918 | ASVLTLGQLCIYCNR | | | Carbamidomethyl (C)[10,13] | | Mascot |
| 1901.9425 | 1901.9904 | 0.0479 | 25 | 129 | 144 | DGLYNLMKLANDVTYR | | | Oxidation (M)[7] | | Mascot |
| 1915.9364 | 1915.9589 | 0.0225 | 12 | 236 | 252 | VLCCAPSNVAVDNLVER | | | Carbamidomethyl (C)[3,4] | | Mascot |
| 2114.1096 | 2114.187 | 0.0774 | 37 | 236 | 255 | VLCCAPSNVAVDNLVERLAK | | | | | Mascot |
| 2114.1096 | 2114.187 | 0.0774 | 37 | 236 | 255 | VLCCAPSNVAVDNLVERLAK | | | | | Mascot |
| 2136.051 | 2136.1672 | 0.1162 | 54 | 41 | 59 | GVCLMKLQIGSQHTGMYGR | | | Carbamidomethyl (C)[3] | | Mascot |
| 2489.3721 | 2489.0876 | -0.2845 | -114 | 508 | 531 | GNQGEVDIVALHIKALTEAGVQVK | | | | | Mascot |

| | | | | | | | | | | | | |
|---|---|---|---|---|---|---|---|---|---|---|---|---|
| 2515.167 | 2515.2405 | 0.0735 | 29 | 631 | 651 | HGELRTAFEYLEDCVAQ NYTR | | | | | | Mascot |
| 2590.2375 | 2590.2161 | -0.0214 | -8 | 484 | 507 | IPLLLVDTAGCGLNEMEN TDEQSK | | | | | | Mascot |
| 3338.6208 | 3338.7615 | 0.1407 | 42 | 868 | 901 | AGATDIAAAANGDDDFD ALINAVVKAESVCGFVK | | | | | | Mascot |

| | | | | | | | |
|---|---|---|---|---|---|---|---|
| 9 | RecName: Full=Eukaryotic translation initiation factor 3 subunit D; Short=eIF3d; AltName: Full=Euka | gi\|82187444 | 64049.5 | 6.06 | 11 | 39 | 0 |

Peptide Information

| Calc. Mass | Obsrv. Mass | ± da | ± ppm | Start Seq. | End Seq. | Sequence | Ion Score | C. I. % | Modification | Rank | Result Type |
|---|---|---|---|---|---|---|---|---|---|---|---|
| 856.3981 | 856.5234 | 0.1253 | 146 | 333 | 339 | MGGERYK | | | Oxidation (M)[1] | | Mascot |
| 901.5352 | 901.5057 | -0.0295 | -33 | 421 | 429 | GAVLATELK | | | | | Mascot |
| 1103.6459 | 1103.5682 | -0.0777 | -70 | 509 | 517 | YLILKDPNK | | | | | Mascot |
| 1112.5082 | 1112.5077 | -0.0005 | 0 | 27 | 35 | DMPYQPFSK | | | | | Mascot |
| 1354.6273 | 1354.7678 | 0.1405 | 104 | 42 | 53 | VADWTGATYQDK | | | | | Mascot |
| 1354.6273 | 1354.7678 | 0.1405 | 104 | 42 | 53 | VADWTGATYQDK | | | | | Mascot |
| 1707.9098 | 1707.776 | -0.1338 | -78 | 113 | 127 | TLTQFNMQTLPKSAK | | | | | Mascot |
| 2233.1143 | 2233.0842 | -0.0301 | -13 | 438 | 457 | WTCCAILAGSEYLKLGYV SR | | | | | Mascot |
| 2306.1023 | 2306.1567 | 0.0544 | 24 | 4 | 24 | FHAPVIQDNPSGWGPCA VPEK | | | Carbamidomethyl (C)[16] | | Mascot |
| 2448.2129 | 2448.1428 | -0.0701 | -29 | 2 | 24 | AKFHAPVIQDNPSGWGP CAVPEK | | | | | Mascot |
| 2457.0408 | 2457.3127 | 0.2719 | 111 | 187 | 207 | YMDVADPLDIECCGALEH YDK | | | Carbamidomethyl (C)[12] | | Mascot |
| 2457.2297 | 2457.3127 | 0.083 | 34 | 241 | 263 | LAKTQGNVFATDAILATL MCCTR | | | Oxidation (M)[19] | | Mascot |

| | | | | | | | |
|---|---|---|---|---|---|---|---|
| 10 | eukaryotic translation initiation factor 3, subunit 7 zeta [Danio rerio] | gi\|123230120 | 63850.4 | 6.06 | 11 | 38 | 0 |

Peptide Information

| Calc. Mass | Obsrv. Mass | ± da | ± ppm | Start Seq. | End Seq. | Sequence | Ion Score | C. I. % | Modification | Rank | Result Type |
|---|---|---|---|---|---|---|---|---|---|---|---|
| 856.3981 | 856.5234 | 0.1253 | 146 | 331 | 337 | MGGERYK | | | Oxidation (M)[1] | | Mascot |
| 901.5352 | 901.5057 | -0.0295 | -33 | 419 | 427 | GAVLATELK | | | | | Mascot |
| 1103.6459 | 1103.5682 | -0.0777 | -70 | 507 | 515 | YLILKDPNK | | | | | Mascot |
| 1112.5082 | 1112.5077 | -0.0005 | 0 | 27 | 35 | DMPYQPFSK | | | | | Mascot |
| 1354.6273 | 1354.7678 | 0.1405 | 104 | 42 | 53 | VADWTGATYQDK | | | | | Mascot |
| 1354.6273 | 1354.7678 | 0.1405 | 104 | 42 | 53 | VADWTGATYQDK | | | | | Mascot |
| 1707.9098 | 1707.776 | -0.1338 | -78 | 113 | 127 | TLTQFNMQTLPKSAK | | | | | Mascot |
| 2233.1143 | 2233.0842 | -0.0301 | -13 | 436 | 455 | WTCCAILAGSEYLKLGYV SR | | | | | Mascot |
| 2306.1023 | 2306.1567 | 0.0544 | 24 | 4 | 24 | FHAPVIQDNPSGWGPCA | | | Carbamidomethyl (C)[16] | | Mascot |

| Calc. Mass | Obsrv. Mass | ± da | ± ppm | Start Seq. | End Seq. | Sequence | Ion Score | C. I. % | Modification | Rank | Result Type |
|---|---|---|---|---|---|---|---|---|---|---|---|
| | | | | | | VPEK | | | | | |
| 2448.2129 | 2448.1428 | -0.0701 | -29 | 2 | 24 | AKFHAPVIQDNPSGWGP CAVPEK | | | | | Mascot |
| 2457.0408 | 2457.3127 | 0.2719 | 111 | 185 | 205 | YMDVADPLDIECCGALEH YDK | | | Carbamidomethyl (C)[12] | | Mascot |
| 2457.2297 | 2457.3127 | 0.083 | 34 | 239 | 261 | LAKTQGNVFATDAILATL MCCTR | | | Oxidation (M)[19] | | Mascot |

11 PREDICTED: UDP-GlcNAc:betaGal beta-1,3-N-acetylglucosaminyltransferase 2 [Danio rerio] gi|125843963 48322.6 8.69 10 38 0

**Peptide Information**

| Calc. Mass | Obsrv. Mass | ± da | ± ppm | Start Seq. | End Seq. | Sequence | Ion Score | C. I. % | Modification | Rank | Result Type |
|---|---|---|---|---|---|---|---|---|---|---|---|
| 864.411 | 864.4704 | 0.0594 | 69 | 84 | 90 | NGAFWNR | | | | | Mascot |
| 1044.6047 | 1044.4929 | -0.1118 | -107 | 37 | 46 | ITSVTASIPR | | | | | Mascot |
| 1103.5215 | 1103.5682 | 0.0467 | 42 | 210 | 219 | SSLDDPNLDK | | | | | Mascot |
| 1165.5847 | 1165.7476 | 0.1629 | 140 | 193 | 202 | EGEYDGLKVR | | | | | Mascot |
| 1179.5753 | 1179.6022 | 0.0269 | 23 | 378 | 386 | TFDIREQDR | | | | | Mascot |
| 1354.6168 | 1354.7678 | 0.151 | 111 | 383 | 393 | EQDRENPCVHK | | | | | Mascot |
| 1354.6168 | 1354.7678 | 0.151 | 111 | 383 | 393 | EQDRENPCVHK | | | | | Mascot |
| 1436.6879 | 1436.7465 | 0.0586 | 41 | 112 | 123 | FHCSPESFELLK | | | | | Mascot |
| 1736.8763 | 1736.8861 | 0.0098 | 6 | 253 | 266 | WMLGHCPRVSFIFK | | | Oxidation (M)[2] | | Mascot |
| 1751.9486 | 1751.814 | -0.1346 | -77 | 387 | 400 | ENPCVHKHLLLVHR | | | Carbamidomethyl (C)[4] | | Mascot |
| 2457.3323 | 2457.3127 | -0.0196 | -8 | 5 | 25 | YMKFLIVALASSLCFILFY SK | | | | | Mascot |
| 2457.3323 | 2457.3127 | -0.0196 | -8 | 5 | 25 | YMKFLIVALASSLCFILFY SK | | | | | Mascot |

12 PREDICTED: protein NLRC3 isoform 1 [Danio rerio] gi|326667049 94863 6.12 13 37 0

**Peptide Information**

| Calc. Mass | Obsrv. Mass | ± da | ± ppm | Start Seq. | End Seq. | Sequence | Ion Score | C. I. % | Modification | Rank | Result Type |
|---|---|---|---|---|---|---|---|---|---|---|---|
| 901.4737 | 901.5057 | 0.032 | 35 | 135 | 141 | ILQEENR | | | | | Mascot |
| 959.4614 | 959.5134 | 0.052 | 54 | 407 | 414 | IPADCIDR | | | Carbamidomethyl (C)[5] | | Mascot |
| 1045.5272 | 1045.562 | 0.0348 | 33 | 660 | 668 | GLLTQQDDR | | | | | Mascot |
| 1062.5803 | 1062.6919 | 0.1116 | 105 | 502 | 509 | FQIQQSRR | | | | | Mascot |
| 1210.6791 | 1210.5983 | -0.0808 | -67 | 442 | 451 | EIIDHIKQSK | | | | | Mascot |
| 1436.7631 | 1436.7465 | -0.0166 | -12 | 669 | 680 | DQSKEEIIAYIK | | | | | Mascot |
| 2114.0876 | 2114.187 | 0.0994 | 47 | 626 | 644 | AAVDKALESENGHLDLYL R | | | | | Mascot |
| 2114.0876 | 2114.187 | 0.0994 | 47 | 626 | 644 | AAVDKALESENGHLDLYL R | | | | | Mascot |
| 2136.072 | 2136.1672 | 0.0952 | 45 | 103 | 120 | HKSFTDNLQSIFQNLESK | | | | | Mascot |

| Calc. Mass | Obsrv. Mass | ± da | ± ppm | Start Seq. | End Seq. | Sequence | Ion Score | C. I. % | Modification | Rank | Result Type |
|---|---|---|---|---|---|---|---|---|---|---|---|
| 2211.2092 | 2211.1079 | -0.1013 | -46 | 153 | 171 | SIITEAALDLTLFFLREMK | | | | | Mascot |
| 2294.0864 | 2294.1646 | 0.0782 | 34 | 551 | 571 | DCGIDVYKASVYSGMCTQILK | | | | | Mascot |
| 2294.0864 | 2294.1646 | 0.0782 | 34 | 551 | 571 | DCGIDVYKASVYSGMCTQILK | | | | | Mascot |
| 2298.1812 | 2298.1887 | 0.0075 | 3 | 491 | 508 | TLTQMYTHFLRFQIQQSR | | | | | Mascot |
| 2306.1008 | 2306.1567 | 0.0559 | 24 | 540 | 558 | NNLIFYESDLKDCGIDVYK | | | Carbamidomethyl (C)[13] | | Mascot |
| 2341.2188 | 2341.1233 | -0.0955 | -41 | 532 | 550 | LAFQQLERNNLIFYESDLK | | | | | Mascot |

13 protein BTG3 [Danio rerio] gi|55925203 18115.1 5.85 6 36 0

**Peptide Information**

| Calc. Mass | Obsrv. Mass | ± da | ± ppm | Start Seq. | End Seq. | Sequence | Ion Score | C. I. % | Modification | Rank | Result Type |
|---|---|---|---|---|---|---|---|---|---|---|---|
| 901.5353 | 901.5057 | -0.0296 | -33 | 34 | 41 | LTVALQEK | | | | | Mascot |
| 1112.504 | 1112.5077 | 0.0037 | 3 | 75 | 84 | ACAESGVQYK | | | Carbamidomethyl (C)[2] | | Mascot |
| 2096.978 | 2097.0769 | 0.0989 | 47 | 91 | 108 | ELTLWVDPGEVCCRYGEK | | | | | Mascot |
| 2211.0208 | 2211.1079 | 0.0871 | 39 | 91 | 108 | ELTLWVDPGEVCCRYGEK | | | Carbamidomethyl (C)[12,13] | | Mascot |
| 2224.9412 | 2225.1235 | 0.1823 | 82 | 139 | 158 | VTSDYHSGSSSDEDSMLVHR | | | Oxidation (M)[16] | | Mascot |
| 2300.1414 | 2300.1807 | 0.0393 | 17 | 85 | 104 | DLGLPKELTLWVDPGEVCCR | | | Carbamidomethyl (C)[18] | | Mascot |
| 2457.0688 | 2457.3127 | 0.2439 | 99 | 109 | 130 | NHGFTVATFSSEDDDDKEDVTK | | | | | Mascot |
| 2457.0688 | 2457.3127 | 0.2439 | 99 | 109 | 130 | NHGFTVATFSSEDDDDKEDVTK | | | | | Mascot |

14 SH3 domain-binding protein 4-A [Danio rerio] gi|45387913 104947.5 7.73 13 35 0

**Peptide Information**

| Calc. Mass | Obsrv. Mass | ± da | ± ppm | Start Seq. | End Seq. | Sequence | Ion Score | C. I. % | Modification | Rank | Result Type |
|---|---|---|---|---|---|---|---|---|---|---|---|
| 842.5822 | 842.5089 | -0.0733 | -87 | 634 | 640 | LIKTVIR | | | | | Mascot |
| 850.4338 | 850.5435 | 0.1097 | 129 | 358 | 364 | LSNMEIK | | | Oxidation (M)[4] | | Mascot |
| 870.4726 | 870.54 | 0.0674 | 77 | 1 | 7 | MAAHRIR | | | Oxidation (M)[1] | | Mascot |
| 1045.4796 | 1045.562 | 0.0824 | 79 | 762 | 770 | AELDSEPER | | | | | Mascot |
| 1210.679 | 1210.5983 | -0.0807 | -67 | 832 | 842 | DLAEKLAHVSK | | | | | Mascot |
| 1488.9624 | 1488.7856 | -0.1768 | -119 | 684 | 697 | IGLVHVKNVLVLGK | | | | | Mascot |
| 1488.9624 | 1488.7856 | -0.1768 | -119 | 684 | 697 | IGLVHVKNVLVLGK | | | | | Mascot |
| 1767.8074 | 1767.8112 | 0.0038 | 2 | 379 | 394 | TESPMAEIVCVRSDCK | | | | | Mascot |
| 2097.0137 | 2097.0769 | 0.0632 | 30 | 373 | 390 | VSVMTRTESPMAEIVCVR | | | Carbamidomethyl (C)[16], Oxidation (M)[4,11] | | Mascot |
| 2284.2368 | 2284.1746 | -0.0622 | -27 | 463 | 484 | TVVAIFGHDCAPKTLLVSEVGK | | | | | Mascot |

| Calc. Mass | Obsrv. Mass | ± da | ± ppm | Start Seq. | End Seq. | Sequence | Ion Score | C. I. % | Modification | Rank | Result Type |
|---|---|---|---|---|---|---|---|---|---|---|---|
| 2300.0686 | 2300.1807 | 0.1121 | 49 | 568 | 587 | DDQDCILAQFCVQTPQPPPK | | | Carbamidomethyl (C)[5] | | Mascot |
| 2341.2583 | 2341.1233 | -0.135 | -58 | 463 | 484 | TVVAIFGHDCAPKTLLVSEVGK | | | Carbamidomethyl (C)[10] | | Mascot |
| 3000.4343 | 3000.2456 | -0.1887 | -63 | 500 | 524 | HQFVLSRPQDLQIGMYSNMSNYEVK | | | Oxidation (M)[15] | | Mascot |
| 3337.6052 | 3337.7454 | 0.1402 | 42 | 269 | 298 | LARSCHDLESLGQNPGWGQTQPVETNIVCK | | | Carbamidomethyl (C)[5] | | Mascot |
| 3338.5496 | 3338.7615 | 0.2119 | 63 | 856 | 884 | TGMVDSEAMWKPAYDFLLTWAAQIGDSYR | | | Oxidation (M)[3] | | Mascot |

| | | | | | | | |
|---|---|---|---|---|---|---|---|
| 15 | PREDICTED: hypothetical protein LOC565734 [Danio rerio] | gi\|68402113 | 30582.9 | 10.05 | 7 | 35 | 0 |

**Peptide Information**

| Calc. Mass | Obsrv. Mass | ± da | ± ppm | Start Seq. | End Seq. | Sequence | Ion Score | C. I. % | Modification | Rank | Result Type |
|---|---|---|---|---|---|---|---|---|---|---|---|
| 1165.6324 | 1165.7476 | 0.1152 | 99 | 118 | 128 | VSPNATHAQIK | | | | | Mascot |
| 1436.7943 | 1436.7465 | -0.0478 | -33 | 80 | 91 | HWCLLLRPVGSR | | | | | Mascot |
| 1475.7634 | 1475.7533 | -0.0101 | -7 | 1 | 14 | MAEVTGSVRNAVNK | | | | | Mascot |
| 1902.0093 | 1901.9904 | -0.0189 | -10 | 20 | 36 | TLLSLHHHPHGPVVDSR | | | | | Mascot |
| 2284.0571 | 2284.1746 | 0.1175 | 51 | 41 | 60 | KSNVCVHWCAADCVWALHNK | | | | | Mascot |
| 2341.0786 | 2341.1233 | 0.0447 | 19 | 41 | 60 | KSNVCVHWCAADCVWALHNK | | | Carbamidomethyl (C)[5] | | Mascot |
| 2448.3259 | 2448.1428 | -0.1831 | -75 | 15 | 36 | LSAFKTLLSLHHHPHGPVVDSR | | | | | Mascot |
| 2479.5107 | 2479.2808 | -0.2299 | -93 | 246 | 269 | EQITGVSVILLLLLGGVIINSLKS | | | | | Mascot |

| | | | | | | | |
|---|---|---|---|---|---|---|---|
| 16 | PREDICTED: protein NLRC3 isoform 2 [Danio rerio] | gi\|326667051 | 87403.4 | 6.43 | 12 | 35 | 0 |

**Peptide Information**

| Calc. Mass | Obsrv. Mass | ± da | ± ppm | Start Seq. | End Seq. | Sequence | Ion Score | C. I. % | Modification | Rank | Result Type |
|---|---|---|---|---|---|---|---|---|---|---|---|
| 901.4737 | 901.5057 | 0.032 | 35 | 126 | 132 | ILQEENR | | | | | Mascot |
| 959.4614 | 959.5134 | 0.052 | 54 | 398 | 405 | IPADCIDR | | | Carbamidomethyl (C)[5] | | Mascot |
| 1045.5272 | 1045.562 | 0.0348 | 33 | 651 | 659 | GLLTQQDDR | | | | | Mascot |
| 1062.5803 | 1062.6919 | 0.1116 | 105 | 493 | 500 | FQIQQSRR | | | | | Mascot |
| 1210.6791 | 1210.5983 | -0.0808 | -67 | 433 | 442 | EIIDHIKQSK | | | | | Mascot |
| 1436.7631 | 1436.7465 | -0.0166 | -12 | 660 | 671 | DQSKEEIIAYIK | | | | | Mascot |
| 2114.0876 | 2114.187 | 0.0994 | 47 | 617 | 635 | AAVDKALESENGHLDLYLR | | | | | Mascot |
| 2114.0876 | 2114.187 | 0.0994 | 47 | 617 | 635 | AAVDKALESENGHLDLYLR | | | | | Mascot |
| 2211.2092 | 2211.1079 | -0.1013 | -46 | 144 | 162 | SIITEAALDLTLFFLREMK | | | | | Mascot |
| 2294.0864 | 2294.1646 | 0.0782 | 34 | 542 | 562 | DCGIDVYKASVYSGMCTQILK | | | | | Mascot |
| 2294.0864 | 2294.1646 | 0.0782 | 34 | 542 | 562 | DCGIDVYKASVYSGMCT | | | | | Mascot |

| Calc. Mass | Obsrv. Mass | ± da | ± ppm | Start Seq. | End Seq. | Sequence | Ion Score | C. I. % | Modification | Rank | Result Type |
|---|---|---|---|---|---|---|---|---|---|---|---|
| | | | | | | QILK | | | | | |
| 2298.1812 | 2298.1887 | 0.0075 | 3 | 482 | 499 | TLTQMYTHFLRFQIQQSR | | | | | Mascot |
| 2306.1008 | 2306.1567 | 0.0559 | 24 | 531 | 549 | NNLIFYESDLKDCGIDVYK | | | Carbamidomethyl (C)[13] | | Mascot |
| 2341.2188 | 2341.1233 | -0.0955 | -41 | 523 | 541 | LAFQQLERNNLIFYESDLK | | | | | Mascot |

17 hypothetical protein LOC100170820 [Danio rerio] gi|194578801 15546.9 9.84 7 35 0

**Peptide Information**

| Calc. Mass | Obsrv. Mass | ± da | ± ppm | Start Seq. | End Seq. | Sequence | Ion Score | C. I. % | Modification | Rank | Result Type |
|---|---|---|---|---|---|---|---|---|---|---|---|
| 870.4614 | 870.54 | 0.0786 | 90 | 55 | 61 | RNPPCVK | | | Carbamidomethyl (C)[5] | | Mascot |
| 1151.6089 | 1151.6196 | 0.0107 | 9 | 105 | 114 | MNSTLSTLLR | | | Oxidation (M)[1] | | Mascot |
| 1179.5796 | 1179.6022 | 0.0226 | 19 | 2 | 10 | QLNQKMMMR | | | | | Mascot |
| 2136.0427 | 2136.1672 | 0.1245 | 58 | 7 | 24 | MMMRLAAIAVIMSVMIMK | | | Oxidation (M)[1,2,3,12,15,17] | | Mascot |
| 2193.1553 | 2193.1125 | -0.0428 | -20 | 11 | 30 | LAAIAVIMSVMIMKTNGQNR | | | Oxidation (M)[8,11] | | Mascot |
| 2294.1982 | 2294.1646 | -0.0336 | -15 | 85 | 104 | IQELIISMEKMNSTVSTPLK | | | Oxidation (M)[8,11] | | Mascot |
| 2294.1982 | 2294.1646 | -0.0336 | -15 | 85 | 104 | IQELIISMEKMNSTVSTPLK | | | Oxidation (M)[8,11] | | Mascot |
| 2457.2024 | 2457.3127 | 0.1103 | 45 | 115 | 138 | INSTISTALQMNSTLSTPLSTSSS | | | Oxidation (M)[11] | | Mascot |
| 2457.2024 | 2457.3127 | 0.1103 | 45 | 115 | 138 | INSTISTALQMNSTLSTPLSTSSS | | | Oxidation (M)[11] | | Mascot |

18 hypothetical protein LOC567306 [Danio rerio] gi|148540014 33418.2 5.53 9 35 0

**Peptide Information**

| Calc. Mass | Obsrv. Mass | ± da | ± ppm | Start Seq. | End Seq. | Sequence | Ion Score | C. I. % | Modification | Rank | Result Type |
|---|---|---|---|---|---|---|---|---|---|---|---|
| 1112.5524 | 1112.5077 | -0.0447 | -40 | 200 | 208 | YGAKDHFFK | | | | | Mascot |
| 1179.5385 | 1179.6022 | 0.0637 | 54 | 9 | 19 | EAMAIMAEADK | | | | | Mascot |
| 1436.676 | 1436.7465 | 0.0705 | 49 | 7 | 19 | EKEAMAIMAEADK | | | | | Mascot |
| 1542.7223 | 1542.8818 | 0.1595 | 103 | 141 | 153 | AIAHYEQAADYYK | | | | | Mascot |
| 1908.8289 | 1908.9324 | 0.1035 | 54 | 38 | 53 | VEEACDMYVRAANMFK | | | Oxidation (M)[7,14] | | Mascot |
| 2000.0171 | 1999.9437 | -0.0734 | -37 | 57 | 74 | NWNAAGDAFCKAALLHLK | | | Carbamidomethyl (C)[10] | | Mascot |
| 2000.0171 | 1999.9437 | -0.0734 | -37 | 57 | 74 | NWNAAGDAFCKAALLHLK | | | Carbamidomethyl (C)[10] | | Mascot |
| 2157.0571 | 2157.1226 | 0.0655 | 30 | 246 | 264 | KLLDAHDEQNIDAYADAVR | | | | | Mascot |
| 2306.1025 | 2306.1567 | 0.0542 | 24 | 209 | 227 | AALCHFCVDMLNCRLAVQK | | | Carbamidomethyl (C)[4,7,13] | | Mascot |
| 2457.0718 | 2457.3127 | 0.2409 | 98 | 204 | 222 | DHFFKAALCHFCVDMLNCR | | | Carbamidomethyl (C)[9,12,18], Oxidation (M)[15] | | Mascot |
| 2457.0718 | 2457.3127 | 0.2409 | 98 | 204 | 222 | DHFFKAALCHFCVDMLNCR | | | Carbamidomethyl (C)[9,12,18], Oxidation (M)[15] | | Mascot |

19 DNA-binding protein SMUBP-2 [Danio rerio] gi|120952590 110816.5 8.97 13 35 0

**Peptide Information**

| Calc. Mass | Obsrv. Mass | ± da | ± ppm | Start Seq. | End Seq. | Sequence | Ion Score | C. I. % | Modification | Rank | Result Type |
|---|---|---|---|---|---|---|---|---|---|---|---|
| 882.4389 | 882.5677 | 0.1288 | 146 | 393 | 400 | CILAGDYK | | | | | Mascot |
| 996.4818 | 996.5963 | 0.1145 | 115 | 897 | 905 | AESVCGFVK | | | Carbamidomethyl (C)[5] | | Mascot |
| 1210.5773 | 1210.5983 | 0.021 | 17 | 297 | 306 | DMDKAFNEIK | | | | | Mascot |
| 1436.7743 | 1436.7465 | -0.0278 | -19 | 948 | 961 | ISKEGVLYAGSGQK | | | | | Mascot |
| 1767.8879 | 1767.8112 | -0.0767 | -43 | 908 | 922 | ASVLTLGQLCIYCNR | | | Carbamidomethyl (C)[10,13] | | Mascot |
| 1901.9425 | 1901.9904 | 0.0479 | 25 | 129 | 144 | DGLYNLMKLANDVTYR | | | Oxidation (M)[7] | | Mascot |
| 1915.9364 | 1915.9589 | 0.0225 | 12 | 236 | 252 | VLCCAPSNVAVDNLVER | | | Carbamidomethyl (C)[3,4] | | Mascot |
| 2114.1096 | 2114.187 | 0.0774 | 37 | 236 | 255 | VLCCAPSNVAVDNLVERL AK | | | | | Mascot |
| 2114.1096 | 2114.187 | 0.0774 | 37 | 236 | 255 | VLCCAPSNVAVDNLVERL AK | | | | | Mascot |
| 2136.051 | 2136.1672 | 0.1162 | 54 | 41 | 59 | GVCLMKLQIGSQHTGMY GR | | | Carbamidomethyl (C)[3] | | Mascot |
| 2489.3721 | 2489.0876 | -0.2845 | -114 | 508 | 531 | GNQGEVDIVALHIKALTE AGVQVK | | | | | Mascot |
| 2515.167 | 2515.2405 | 0.0735 | 29 | 631 | 651 | HGELRTAFEYLEDCVAQ NYTR | | | | | Mascot |
| 2590.2375 | 2590.2161 | -0.0214 | -8 | 484 | 507 | IPLLLVDTAGCGLNEMEN TDEQSK | | | | | Mascot |
| 3338.6208 | 3338.7615 | 0.1407 | 42 | 872 | 905 | AGATDIAAAANGDDDFD ALINAVVKAESVCGFVK | | | | | Mascot |

| | | | | | | | |
|---|---|---|---|---|---|---|---|
| 20 | PREDICTED: diacylglycerol kinase beta-like, partial [Danio rerio] | gi\|326674321 | 85995.9 | 6.78 | 12 | 34 | 0 |

**Peptide Information**

| Calc. Mass | Obsrv. Mass | ± da | ± ppm | Start Seq. | End Seq. | Sequence | Ion Score | C. I. % | Modification | Rank | Result Type |
|---|---|---|---|---|---|---|---|---|---|---|---|
| 842.4229 | 842.5089 | 0.086 | 102 | 153 | 158 | LEFMFR | | | | | Mascot |
| 864.4032 | 864.4704 | 0.0672 | 78 | 455 | 462 | SGPMPGYR | | | | | Mascot |
| 901.5353 | 901.5057 | -0.0296 | -33 | 678 | 685 | KTTIVDPK | | | | | Mascot |
| 1045.4264 | 1045.562 | 0.1356 | 130 | 270 | 278 | QGLCCSFCK | | | Carbamidomethyl (C)[4] | | Mascot |
| 1112.6497 | 1112.5077 | -0.142 | -128 | 112 | 121 | LIKGSCPPLK | | | Carbamidomethyl (C)[6] | | Mascot |
| 1210.5455 | 1210.5983 | 0.0528 | 44 | 455 | 465 | SGPMPGYRSCR | | | | | Mascot |
| 1707.8007 | 1707.776 | -0.0247 | -14 | 534 | 547 | DVQDSSEVMLDRWK | | | | | Mascot |
| 2157.0005 | 2157.1226 | 0.1221 | 57 | 302 | 319 | KNTEIMHHFWVEGNCPS K | | | | | Mascot |
| 2283.2488 | 2283.1833 | -0.0655 | -29 | 115 | 135 | GSCPPLKVTSQSRPLSTV QLK | | | Carbamidomethyl (C)[3] | | Mascot |
| 2284.0591 | 2284.1746 | 0.1155 | 51 | 153 | 171 | LEFMFRLYDTDGNGFLD SK | | | Oxidation (M)[4] | | Mascot |
| 2432.0581 | 2432.1375 | 0.0794 | 33 | 303 | 322 | NTEIMHHFWVEGNCPSK CDK | | | Carbamidomethyl (C)[14] | | Mascot |

| Calc. Mass | Obsrv. Mass | ± da | ± ppm | Start Seq. | End Seq. | Sequence | Ion Score | C. I. % | Modification | Rank | Result Type |
|---|---|---|---|---|---|---|---|---|---|---|---|
| 2432.0581 | 2432.1375 | 0.0794 | 33 | 303 | 322 | NTEIMHHFWVEGNCPSK CDK | | | Carbamidomethyl (C)[14] | | Mascot |
| 2448.053 | 2448.1428 | 0.0898 | 37 | 303 | 322 | NTEIMHHFWVEGNCPSK CDK | | | Carbamidomethyl (C)[14], Oxidation (M)[5] | | Mascot |
| 2479.3022 | 2479.2808 | -0.0214 | -9 | 248 | 269 | LKHFSKPAYCNLCLNMLI GLGK | | | Oxidation (M)[16] | | Mascot |
| 2489.0796 | 2489.0876 | 0.008 | 3 | 303 | 322 | NTEIMHHFWVEGNCPSK CDK | | | Carbamidomethyl (C)[14,18] | | Mascot |

| | | | | | | | | |
|---|---|---|---|---|---|---|---|---|
| 21 | PREDICTED: tetratricopeptide repeat protein 37-like [Danio rerio] | gi\|292614103 | 173677.3 | 6.37 | 17 | 34 | 0 | |

**Peptide Information**

| Calc. Mass | Obsrv. Mass | ± da | ± ppm | Start Seq. | End Seq. | Sequence | Ion Score | C. I. % | Modification | Rank | Result Type |
|---|---|---|---|---|---|---|---|---|---|---|---|
| 856.402 | 856.5234 | 0.1214 | 142 | 1242 | 1248 | HSGEDRR | | | | | Mascot |
| 864.4825 | 864.4704 | -0.0121 | -14 | 21 | 27 | EFKEALK | | | | | Mascot |
| 870.5519 | 870.54 | -0.0119 | -14 | 295 | 302 | NIGLGLKR | | | | | Mascot |
| 1103.5592 | 1103.5682 | 0.009 | 8 | 992 | 1000 | KQATEAYHR | | | | | Mascot |
| 1436.7896 | 1436.7465 | -0.0431 | -30 | 479 | 491 | AAKLDPYLGSVFR | | | | | Mascot |
| 1475.6761 | 1475.7533 | 0.0772 | 52 | 1024 | 1036 | ALSDDGQWEEAVR | | | | | Mascot |
| 1736.9734 | 1736.8861 | -0.0873 | -50 | 305 | 319 | FSIIGWYNLALAQLK | | | | | Mascot |
| 1901.9459 | 1901.9904 | 0.0445 | 23 | 728 | 745 | AKVLVPGALCGMDPSDQ K | | | Carbamidomethyl (C)[10], Oxidation (M)[12] | | Mascot |
| 1908.8834 | 1908.9324 | 0.049 | 26 | 1020 | 1036 | GYGRALSDDGQWEEAV R | | | | | Mascot |
| 2000.0885 | 1999.9437 | -0.1448 | -72 | 1445 | 1463 | LSLLALTPCLAGVGVSDW K | | | Carbamidomethyl (C)[9] | | Mascot |
| 2000.0885 | 1999.9437 | -0.1448 | -72 | 1445 | 1463 | LSLLALTPCLAGVGVSDW K | | | Carbamidomethyl (C)[9] | | Mascot |
| 2157.145 | 2157.1226 | -0.0224 | -10 | 264 | 282 | RLLELDPNNSYAHFGLGI K | | | | | Mascot |
| 2193.1218 | 2193.1125 | -0.0093 | -4 | 664 | 684 | GLGECLLALAKSALDDYL DGR | | | | | Mascot |
| 2294.269 | 2294.1646 | -0.1044 | -46 | 1083 | 1103 | AYILTALALIQHKLGSMDH AK | | | | | Mascot |
| 2294.269 | 2294.1646 | -0.1044 | -46 | 1083 | 1103 | AYILTALALIQHKLGSMDH AK | | | | | Mascot |
| 2306.1379 | 2306.1567 | 0.0188 | 8 | 702 | 721 | QRPDLSCLWKLLGDSCT ASR | | | Carbamidomethyl (C)[7] | | Mascot |
| 2489.2751 | 2489.0876 | -0.1875 | -75 | 1158 | 1180 | CLLTCTLLALQGNFSGVH REASR | | | | | Mascot |
| 3000.5095 | 3000.2456 | -0.2639 | -88 | 940 | 966 | SNRDSELYLYNIVQMNAV SAAQVALSK | | | Oxidation (M)[15] | | Mascot |
| 3337.738 | 3337.7454 | 0.0074 | 2 | 1114 | 1145 | EPVQESLQCLCALGLVH GDVTLASATVTELLK | | | | | Mascot |

| | | | | | | | | |
|---|---|---|---|---|---|---|---|---|
| 22 | PREDICTED: dyslexia-associated protein KIAA0319-like [Danio rerio] | gi\|326675082 | 112852.8 | 5 | 13 | 34 | 0 | |

**Peptide Information**

| Calc. Mass | Obsrv. Mass | ± da | ± ppm | Start Seq. | End Seq. | Sequence | Ion Score | C. I. % | Modification | Rank | Result Type |
|---|---|---|---|---|---|---|---|---|---|---|---|

| | | | | | | | | | | | |
|---|---|---|---|---|---|---|---|---|---|---|---|
| 864.4243 | 864.4704 | 0.0461 | 53 | 534 | 540 | VTMQDVR | | | Oxidation (M)[3] | | Mascot |
| 959.5156 | 959.5134 | -0.0022 | -2 | 150 | 157 | RAEDIDLK | | | | | Mascot |
| 1542.6741 | 1542.8818 | 0.2077 | 135 | 951 | 962 | YTILDNMDEQER | | | Oxidation (M)[7] | | Mascot |
| 1707.7288 | 1707.776 | 0.0472 | 28 | 885 | 898 | SCSCDPLWMENPIR | | | Carbamidomethyl (C)[2] | | Mascot |
| 1720.0004 | 1719.8544 | -0.146 | -85 | 798 | 813 | QLAALLHVLDSDIALK | | | | | Mascot |
| 1720.0004 | 1719.8544 | -0.146 | -85 | 798 | 813 | QLAALLHVLDSDIALK | | | | | Mascot |
| 1736.8499 | 1736.8861 | 0.0362 | 21 | 616 | 630 | YQWDVMSGPPGLKMK | | | | | Mascot |
| 1751.9802 | 1751.814 | -0.1662 | -95 | 827 | 843 | FSVQGPDGLIPGPKLAR | | | | | Mascot |
| 1923.8146 | 1923.9685 | 0.1539 | 80 | 58 | 72 | CYVLSCQHTENCQPK | | | Carbamidomethyl (C)[1,6,12] | | Mascot |
| 2097.126 | 2097.0769 | -0.0491 | -23 | 292 | 311 | ALTVSIEGPVEAMLPQNT VK | | | | | Mascot |
| 2432.2766 | 2432.1375 | -0.1391 | -57 | 651 | 673 | LTVVDEQGETDSAVLSIT VKEAK | | | | | Mascot |
| 2432.2766 | 2432.1375 | -0.1391 | -57 | 651 | 673 | LTVVDEQGETDSAVLSIT VKEAK | | | | | Mascot |
| 2448.2881 | 2448.1428 | -0.1453 | -59 | 365 | 387 | VIVKAHQAYGEGSVDLTV HPAEK | | | | | Mascot |
| 2515.1592 | 2515.2405 | 0.0813 | 32 | 1 | 22 | MISGQCWQAATYSESVV SPELR | | | Carbamidomethyl (C)[6], Oxidation (M)[1] | | Mascot |
| 3338.4485 | 3338.7615 | 0.313 | 94 | 29 | 57 | VPDVSSLAQCAGACCDL PGCDLAWFFEHR | | | Carbamidomethyl (C)[10,14,15,20] | | Mascot |

23 PREDICTED: NACHT, LRR and PYD domains-containing protein 12 [Danio rerio] gi|292621731 149261.8 5.29 15 33 0

**Peptide Information**

| Calc. Mass | Obsrv. Mass | ± da | ± ppm | Start Seq. | End Seq. | Sequence | Ion Score | C. I. % | Modification | Rank | Result Type |
|---|---|---|---|---|---|---|---|---|---|---|---|
| 842.4114 | 842.5089 | 0.0975 | 116 | 1214 | 1220 | SNSEHLR | | | | | Mascot |
| 864.5777 | 864.4704 | -0.1073 | -124 | 133 | 139 | KILVVHR | | | | | Mascot |
| 870.5593 | 870.54 | -0.0193 | -22 | 1011 | 1017 | KLLPMIR | | | | | Mascot |
| 901.5829 | 901.5057 | -0.0772 | -86 | 507 | 514 | AIRTVLTK | | | | | Mascot |
| 959.5156 | 959.5134 | -0.0022 | -2 | 872 | 880 | AVNEALQSK | | | | | Mascot |
| 1436.7744 | 1436.7465 | -0.0279 | -19 | 913 | 924 | SIQNNKTVEYIK | | | | | Mascot |
| 1488.7289 | 1488.7856 | 0.0567 | 38 | 375 | 388 | SGDTKSDLSSVHQK | | | | | Mascot |
| 1488.7289 | 1488.7856 | 0.0567 | 38 | 375 | 388 | SGDTKSDLSSVHQK | | | | | Mascot |
| 1916.0157 | 1915.9589 | -0.0568 | -30 | 1115 | 1132 | LGDSVTLLSAVLEDPRCK | | | | | Mascot |
| 2175.0896 | 2175.1401 | 0.0505 | 23 | 1250 | 1270 | LGLSNCGLTDEGCAALAL ALR | | | Carbamidomethyl (C)[6,13] | | Mascot |
| 2175.0896 | 2175.1401 | 0.0505 | 23 | 1250 | 1270 | LGLSNCGLTDEGCAALAL ALR | | | Carbamidomethyl (C)[6,13] | | Mascot |
| 2225.2839 | 2225.1235 | -0.1604 | -72 | 890 | 908 | FLLGLSVESHQILLQRLM K | | | | | Mascot |
| 2284.0181 | 2284.1746 | 0.1565 | 69 | 380 | 400 | SDLSSVHQKSTESESSC VSMR | | | | | Mascot |
| 2300.0129 | 2300.1807 | 0.1678 | 73 | 380 | 400 | SDLSSVHQKSTESESSC | | | Oxidation (M)[20] | | Mascot |

| Calc. Mass | Obsrv. Mass | ± da | ± ppm | Start Seq. | End Seq. | Sequence | Ion Score | C. I. % | Modification | Rank | Result Type |
|---|---|---|---|---|---|---|---|---|---|---|---|
| | | | | | | VSMR | | | | | |
| 2341.0396 | 2341.1233 | 0.0837 | 36 | 380 | 400 | SDLSSVHQKSTESESSC VSMR | | | Carbamidomethyl (C)[17] | | Mascot |
| 2448.2002 | 2448.1428 | -0.0574 | -23 | 414 | 435 | SGDTETDLSHEALNTFRS NLLK | | | | | Mascot |
| 2460.2954 | 2460.1133 | -0.1821 | -74 | 537 | 556 | ENQDVQLIFPLPFREINL MK | | | Oxidation (M)[19] | | Mascot |
| 2479.0752 | 2479.2808 | 0.2056 | 83 | 277 | 297 | KDFCSADQEELLETLDNY SCR | | | | | Mascot |
| 3338.6072 | 3338.7615 | 0.1543 | 46 | 780 | 809 | ECGIDVAEALVYSGLCTQ IFREEFGLYQGK | | | | | Mascot |

| | | | | | | | |
|---|---|---|---|---|---|---|---|
| 24 | immunoglobulin heavy chain variable segment 9-2 [Danio rerio] | gi\|56207380 | 12773.2 | 7.57 | 5 | 33 | 0 |

**Peptide Information**

| Calc. Mass | Obsrv. Mass | ± da | ± ppm | Start Seq. | End Seq. | Sequence | Ion Score | C. I. % | Modification | Rank | Result Type |
|---|---|---|---|---|---|---|---|---|---|---|---|
| 1112.5847 | 1112.5077 | -0.077 | -69 | 28 | 38 | SVNVAPGGFHK | | | | | Mascot |
| 1751.8163 | 1751.814 | -0.0023 | -1 | 63 | 76 | GLEWIIYYYSDSDK | | | | | Mascot |
| 2097.0435 | 2097.0769 | 0.0334 | 16 | 86 | 103 | FTASKDSSNLYLHMNQL K | | | | | Mascot |
| 2225.03 | 2225.1235 | 0.0935 | 42 | 39 | 57 | LSCACSGFTLRSTNMYWI R | | | Oxidation (M)[15] | | Mascot |
| 2233.0811 | 2233.0842 | 0.0031 | 1 | 58 | 76 | QAPGKGLEWIIYYYSDSD K | | | | | Mascot |

| | | | | | | | |
|---|---|---|---|---|---|---|---|
| 25 | procollagen-lysine,2-oxoglutarate 5-dioxygenase 3 [Danio rerio] | gi\|113195568 | 84856.6 | 6.17 | 11 | 32 | 0 |

**Peptide Information**

| Calc. Mass | Obsrv. Mass | ± da | ± ppm | Start Seq. | End Seq. | Sequence | Ion Score | C. I. % | Modification | Rank | Result Type |
|---|---|---|---|---|---|---|---|---|---|---|---|
| 901.5213 | 901.5057 | -0.0156 | -17 | 72 | 80 | TVGGGQKVR | | | | | Mascot |
| 996.4852 | 996.5963 | 0.1111 | 111 | 364 | 372 | TMAVEACKK | | | Oxidation (M)[2] | | Mascot |
| 1044.5697 | 1044.4929 | -0.0768 | -74 | 341 | 349 | HRSLFTGAR | | | | | Mascot |
| 1436.738 | 1436.7465 | 0.0085 | 6 | 432 | 443 | AEDYIDIVQSKR | | | | | Mascot |
| 1475.673 | 1475.7533 | 0.0803 | 54 | 359 | 371 | HDQARTMAVEACK | | | Oxidation (M)[7] | | Mascot |
| 1902.0331 | 1901.9904 | -0.0427 | -22 | 205 | 221 | SQIFQNLNGAIEEVVLK | | | | | Mascot |
| 2097.0222 | 2097.0769 | 0.0547 | 26 | 125 | 142 | VVFSAEGFCWPDQRLAS K | | | Carbamidomethyl (C)[9] | | Mascot |
| 2136.2283 | 2136.1672 | -0.0611 | -29 | 1 | 19 | MTPVPVILTVILAVIQPCR | | | Carbamidomethyl (C)[18], Oxidation (M)[1] | | Mascot |
| 2225.0344 | 2225.1235 | 0.0891 | 40 | 121 | 138 | FSHRVVFSAEGFCWPDQ R | | | Carbamidomethyl (C)[13] | | Mascot |
| 2232.9973 | 2233.0842 | 0.0869 | 39 | 467 | 486 | LAAVSLYQQEGMDPDMS FCK | | | | | Mascot |
| 2306.239 | 2306.1567 | -0.0823 | -36 | 205 | 224 | SQIFQNLNGAIEEVVLKFE K | | | | | Mascot |

| | | | | | | | |
|---|---|---|---|---|---|---|---|
| 26 | PREDICTED: gastrula zinc finger protein XlCGF57.1-like [Danio rerio] | gi\|326667410 | 37299.3 | 9.21 | 9 | 32 | 0 |

Peptide Information

| Calc. Mass | Obsrv. Mass | ± da | ± ppm | Start Seq. | End Seq. | Sequence | Ion Score | C. I. % | Modification | Rank | Result Type |
|---|---|---|---|---|---|---|---|---|---|---|---|
| 959.5091 | 959.5134 | 0.0043 | 4 | 313 | 320 | KTLSSHMR | | | | | Mascot |
| 1354.6532 | 1354.7678 | 0.1146 | 85 | 314 | 325 | TLSSHMRVHASE | | | | | Mascot |
| 1354.6532 | 1354.7678 | 0.1146 | 85 | 314 | 325 | TLSSHMRVHASE | | | | | Mascot |
| 1488.7991 | 1488.7856 | -0.0135 | -9 | 140 | 152 | SFVQQGVLKAHMK | | | Oxidation (M)[12] | | Mascot |
| 1488.7991 | 1488.7856 | -0.0135 | -9 | 140 | 152 | SFVQQGVLKAHMK | | | Oxidation (M)[12] | | Mascot |
| 1542.8573 | 1542.8818 | 0.0245 | 16 | 196 | 208 | SFVQQGLLKVHMR | | | | | Mascot |
| 1707.7577 | 1707.776 | 0.0183 | 11 | 265 | 279 | THTGEKPFSCQQCGK | | | Carbamidomethyl (C)[10] | | Mascot |
| 2097.0005 | 2097.0769 | 0.0764 | 36 | 293 | 310 | VHTGEKLFTCQQCGVSF R | | | Carbamidomethyl (C)[10] | | Mascot |
| 2196.9988 | 2197.1208 | 0.122 | 56 | 65 | 83 | IHMTVHTGEKPFSCEHCP K | | | Oxidation (M)[3] | | Mascot |
| 2284.042 | 2284.1746 | 0.1326 | 58 | 149 | 167 | AHMKTHTGEKPYICHEC GK | | | Carbamidomethyl (C)[14,17] | | Mascot |
| 2300.0369 | 2300.1807 | 0.1438 | 63 | 149 | 167 | AHMKTHTGEKPYICHEC GK | | | Carbamidomethyl (C)[14,17], Oxidation (M)[3] | | Mascot |
| 2479.2043 | 2479.2808 | 0.0765 | 31 | 63 | 83 | LKIHMTVHTGEKPFSCEH CPK | | | Carbamidomethyl (C)[16] | | Mascot |

27 PREDICTED: hypothetical protein LOC795590, partial [Danio rerio] gi|326667578 34784.3 5.29 7 32 0

Peptide Information

| Calc. Mass | Obsrv. Mass | ± da | ± ppm | Start Seq. | End Seq. | Sequence | Ion Score | C. I. % | Modification | Rank | Result Type |
|---|---|---|---|---|---|---|---|---|---|---|---|
| 842.4842 | 842.5089 | 0.0247 | 29 | 129 | 135 | LNQALQR | | | | | Mascot |
| 996.5359 | 996.5963 | 0.0604 | 61 | 307 | 316 | VLPPADAKDA | | | | | Mascot |
| 1767.8153 | 1767.8112 | -0.0041 | -2 | 8 | 23 | LSEAQTQVSGWMGSMR | | | | | Mascot |
| 1923.9164 | 1923.9685 | 0.0521 | 27 | 8 | 24 | LSEAQTQVSGWMGSMR R | | | | | Mascot |
| 2197.0842 | 2197.1208 | 0.0366 | 17 | 273 | 294 | RSAALDSDAQAEAATPP SVPSR | | | | | Mascot |
| 2460.1646 | 2460.1133 | -0.0513 | -21 | 2 | 23 | QFLGSKLSEAQTQVSGW MGSMR | | | Oxidation (M)[18,21] | | Mascot |
| 2479.1558 | 2479.2808 | 0.125 | 50 | 202 | 224 | AALPALTEGTNPDWRFC DSTDAK | | | | | Mascot |

28 PREDICTED: NACHT, LRR and PYD domains-containing protein 12-like [Danio rerio] gi|292610042 103784.1 7.26 13 32 0

Peptide Information

| Calc. Mass | Obsrv. Mass | ± da | ± ppm | Start Seq. | End Seq. | Sequence | Ion Score | C. I. % | Modification | Rank | Result Type |
|---|---|---|---|---|---|---|---|---|---|---|---|
| 870.577 | 870.54 | -0.037 | -43 | 660 | 666 | LEKLVLR | | | | | Mascot |

| | | | | | | | | | | | |
|---|---|---|---|---|---|---|---|---|---|---|---|
| 901.5829 | 901.5057 | -0.0772 | -86 | 92 | 99 | AIRTVLTK | | | | | Mascot |
| 1044.5836 | 1044.4929 | -0.0907 | -87 | 865 | 872 | ELNLKWNK | | | | | Mascot |
| 1179.6732 | 1179.6022 | -0.071 | -60 | 263 | 272 | LADEIIQHLK | | | | | Mascot |
| 1475.8719 | 1475.7533 | -0.1186 | -80 | 903 | 915 | SEVSVIVLLEFIK | | | | | Mascot |
| 1719.8507 | 1719.8544 | 0.0037 | 2 | 742 | 756 | SNSSNLRELELSENK | | | | | Mascot |
| 1719.8507 | 1719.8544 | 0.0037 | 2 | 684 | 698 | SNSSNLRELELSENK | | | | | Mascot |
| 1751.8236 | 1751.814 | -0.0096 | -5 | 667 | 683 | CCGVSVEGCAALASVLR | | | Carbamidomethyl (C)[1,2] | | Mascot |
| 2211.1047 | 2211.1079 | 0.0032 | 1 | 721 | 741 | LWLGYCGVSDKGCAALASALR | | | Carbamidomethyl (C)[6] | | Mascot |
| 2233.1614 | 2233.0842 | -0.0772 | -35 | 663 | 683 | LVLRCCGVSVEGCAALASVLR | | | Carbamidomethyl (C)[5,6] | | Mascot |
| 2460.2954 | 2460.1133 | -0.1821 | -74 | 122 | 141 | ENQDVQLIFPLPFREINLMK | | | Oxidation (M)[19] | | Mascot |
| 2479.3037 | 2479.2808 | -0.0229 | -9 | 474 | 495 | SNQTLLKNLLTQVGNISYSEEK | | | | | Mascot |
| 2489.2969 | 2489.0876 | -0.2093 | -84 | 309 | 328 | TLTEMYTHFLIIQTSIRHER | | | | | Mascot |
| 2515.2246 | 2515.2405 | 0.0159 | 6 | 70 | 91 | STTEDTPIQCRDIFTALPGQHK | | | Carbamidomethyl (C)[10] | | Mascot |

| | | | | | | | | |
|---|---|---|---|---|---|---|---|---|
| 29 | immunoglobulin mu heavy chain [Danio rerio] | gi\|51559357 | 23906.6 | 8.05 | 6 | 31 | 0 | |

**Peptide Information**

| Calc. Mass | Obsrv. Mass | ± da | ± ppm | Start Seq. | End Seq. | Sequence | Ion Score | C. I. % | Modification | Rank | Result Type |
|---|---|---|---|---|---|---|---|---|---|---|---|
| 864.4097 | 864.4704 | 0.0607 | 70 | 209 | 215 | SDWIDTK | | | | | Mascot |
| 1112.5847 | 1112.5077 | -0.077 | -69 | 28 | 38 | SVNVAPGGFHK | | | | | Mascot |
| 1751.8163 | 1751.814 | -0.0023 | -1 | 63 | 76 | GLEWIIYYYSDSDK | | | | | Mascot |
| 2097.0435 | 2097.0769 | 0.0334 | 16 | 86 | 103 | FTASKDSSNLYLHMNQLK | | | | | Mascot |
| 2225.03 | 2225.1235 | 0.0935 | 42 | 39 | 57 | LSCACSGFTLRSTNMYWIR | | | Oxidation (M)[15] | | Mascot |
| 2233.0811 | 2233.0842 | 0.0031 | 1 | 58 | 76 | QAPGKGLEWIIYYYSDSDK | | | | | Mascot |

| | | | | | | | | |
|---|---|---|---|---|---|---|---|---|
| 30 | novel protein [Danio rerio] | gi\|220679564 | 9886.7 | 8.37 | 5 | 31 | 0 | |

**Peptide Information**

| Calc. Mass | Obsrv. Mass | ± da | ± ppm | Start Seq. | End Seq. | Sequence | Ion Score | C. I. % | Modification | Rank | Result Type |
|---|---|---|---|---|---|---|---|---|---|---|---|
| 864.4218 | 864.4704 | 0.0486 | 56 | 33 | 38 | WLMVCR | | | Carbamidomethyl (C)[5] | | Mascot |
| 1103.5632 | 1103.5682 | 0.005 | 5 | 70 | 78 | TAHANWYLK | | | | | Mascot |
| 1736.8207 | 1736.8861 | 0.0654 | 38 | 24 | 38 | SAVPGSSDRWLMVCR | | | Carbamidomethyl (C)[14], Oxidation (M)[12] | | Mascot |
| 2456.99 | 2457.3127 | 0.3227 | 131 | 2 | 23 | IPEYSMSSAASSDMCPYMPVCR | | | Oxidation (M)[6,14] | | Mascot |
| 2456.99 | 2457.3127 | 0.3227 | 131 | 2 | 23 | IPEYSMSSAASSDMCPYMPVCR | | | Oxidation (M)[6,14] | | Mascot |
| 3338.4255 | 3338.7615 | 0.336 | 101 | 2 | 32 | IPEYSMSSAASSDMCPY | | | Carbamidomethyl (C)[15] | | Mascot |

MPVCRSAVPGSSDR

| 31 | replication factor C subunit 2 [Danio rerio] | gi\|61651808 | 38999 | 6.07 | 8 | 31 | 0 |
|---|---|---|---|---|---|---|---|

Peptide Information

| Calc. Mass | Obsrv. Mass | ± da | ± ppm | Start Seq. | End Seq. | Sequence | Ion Score | C. I. % | Modification | Rank | Result Type |
|---|---|---|---|---|---|---|---|---|---|---|---|
| 996.5294 | 996.5963 | 0.0669 | 67 | 180 | 187 | CAVLRYSK | | | Carbamidomethyl (C)[1] | | Mascot |
| 1736.8557 | 1736.8861 | 0.0304 | 18 | 190 | 203 | DEQIMMRLTEVVEK | | | Oxidation (M)[5] | | Mascot |
| 1893.8469 | 1893.9828 | 0.1359 | 72 | 260 | 275 | SMLEHCVNANIDEAYK | | | Carbamidomethyl (C)[6] | | Mascot |
| 1893.8469 | 1893.9828 | 0.1359 | 72 | 260 | 275 | SMLEHCVNANIDEAYK | | | Carbamidomethyl (C)[6] | | Mascot |
| 2114.0911 | 2114.187 | 0.0959 | 45 | 87 | 106 | ALLGPAMKDAVLELNASN DR | | | Oxidation (M)[7] | | Mascot |
| 2114.0911 | 2114.187 | 0.0959 | 45 | 87 | 106 | ALLGPAMKDAVLELNASN DR | | | Oxidation (M)[7] | | Mascot |
| 2157.1631 | 2157.1226 | -0.0405 | -19 | 322 | 341 | VAEGVNSLLQMAGLLGR LCR | | | Carbamidomethyl (C)[19] | | Mascot |
| 2175.1404 | 2175.1401 | -0.0003 | 0 | 42 | 60 | LNEIVGNEETVSRLEVFA R | | | | | Mascot |
| 2175.1404 | 2175.1401 | -0.0003 | 0 | 42 | 60 | LNEIVGNEETVSRLEVFA R | | | | | Mascot |
| 2341.1816 | 2341.1233 | -0.0583 | -25 | 130 | 150 | HKIIILDEADSMTDGAQQ ALR | | | Oxidation (M)[12] | | Mascot |
| 2460.1824 | 2460.1133 | -0.0691 | -28 | 204 | 225 | ENLHVTNDGLEAIIFTAQ GDMR | | | Oxidation (M)[21] | | Mascot |

| 32 | novel protein with a Viral A-type inclusion protein repeat domain [Danio rerio] | gi\|169146304 | 27380.3 | 5.44 | 7 | 31 | 0 |
|---|---|---|---|---|---|---|---|

Peptide Information

| Calc. Mass | Obsrv. Mass | ± da | ± ppm | Start Seq. | End Seq. | Sequence | Ion Score | C. I. % | Modification | Rank | Result Type |
|---|---|---|---|---|---|---|---|---|---|---|---|
| 901.4988 | 901.5057 | 0.0069 | 8 | 182 | 189 | AVEEVVQK | | | | | Mascot |
| 1044.5796 | 1044.4929 | -0.0867 | -83 | 14 | 22 | AELRAELSR | | | | | Mascot |
| 1103.515 | 1103.5682 | 0.0532 | 48 | 224 | 233 | QCHSLKTATD | | | | | Mascot |
| 1488.7329 | 1488.7856 | 0.0527 | 35 | 2 | 13 | ESELLIGWQEER | | | | | Mascot |
| 1488.7805 | 1488.7856 | 0.0051 | 3 | 198 | 210 | EVLTGYIGKEHSR | | | | | Mascot |
| 1915.929 | 1915.9589 | 0.0299 | 16 | 101 | 116 | CQALEQQAVGEERELR | | | Carbamidomethyl (C)[1] | | Mascot |
| 2157.1509 | 2157.1226 | -0.0283 | -13 | 137 | 155 | LEEEQQRSLGLSEVSALL R | | | | | Mascot |

| 33 | PREDICTED: Kv channel-interacting protein 4-like [Danio rerio] | gi\|326663989 | 23863.6 | 4.71 | 6 | 31 | 0 |
|---|---|---|---|---|---|---|---|

Peptide Information

| Calc. Mass | Obsrv. Mass | ± da | ± ppm | Start Seq. | End Seq. | Sequence | Ion Score | C. I. % | Modification | Rank | Result Type |
|---|---|---|---|---|---|---|---|---|---|---|---|
| 850.5257 | 850.5435 | 0.0178 | 21 | 148 | 154 | SIHPRLK | | | | | Mascot |

| Calc. Mass | Obsrv. Mass | ± da | ± ppm | Start Seq. | End Seq. | Sequence | Ion Score | C. I. % | Modification | Rank | Result Type |
|---|---|---|---|---|---|---|---|---|---|---|---|
| 1062.5942 | 1062.6919 | 0.0977 | 92 | 36 | 43 | QELQLLYR | | | | | Mascot |
| 1915.9214 | 1915.9589 | 0.0375 | 20 | 132 | 147 | EEMLDIMKSIYDLMGK | | | | | Mascot |
| 2113.9268 | 2114.187 | 0.2602 | 123 | 2 | 18 | RLNTYNEDEEQESTTER | | | | | Mascot |
| 2114.0049 | 2114.187 | 0.1821 | 86 | 19 | 35 | YHPENLEQLQSQTHFSR | | | | | Mascot |
| 2294.1697 | 2294.1646 | -0.0051 | -2 | 88 | 107 | DKNGSLSFEELVCDLSVLLR | | | Carbamidomethyl (C)[13] | | Mascot |
| 2294.1697 | 2294.1646 | -0.0051 | -2 | 88 | 107 | DKNGSLSFEELVCDLSVLLR | | | Carbamidomethyl (C)[13] | | Mascot |

34 RecName: Full=SH3 domain-binding protein 4-A gi|123888628 108383 7.89 13 30 0

**Peptide Information**

| Calc. Mass | Obsrv. Mass | ± da | ± ppm | Start Seq. | End Seq. | Sequence | Ion Score | C. I. % | Modification | Rank | Result Type |
|---|---|---|---|---|---|---|---|---|---|---|---|
| 842.5822 | 842.5089 | -0.0733 | -87 | 663 | 669 | LIKTVIR | | | | | Mascot |
| 850.4338 | 850.5435 | 0.1097 | 129 | 387 | 393 | LSNMEIK | | | Oxidation (M)[4] | | Mascot |
| 870.4726 | 870.54 | 0.0674 | 77 | 1 | 7 | MAAHRIR | | | Oxidation (M)[1] | | Mascot |
| 1045.4796 | 1045.562 | 0.0824 | 79 | 791 | 799 | AELDSEPER | | | | | Mascot |
| 1210.679 | 1210.5983 | -0.0807 | -67 | 861 | 871 | DLAEKLAHVSK | | | | | Mascot |
| 1488.9624 | 1488.7856 | -0.1768 | -119 | 713 | 726 | IGLVHVKNVLVLGK | | | | | Mascot |
| 1488.9624 | 1488.7856 | -0.1768 | -119 | 713 | 726 | IGLVHVKNVLVLGK | | | | | Mascot |
| 1767.8074 | 1767.8112 | 0.0038 | 2 | 408 | 423 | TESPMAEIVCVRSDCK | | | | | Mascot |
| 2097.0137 | 2097.0769 | 0.0632 | 30 | 402 | 419 | VSVMTRTESPMAEIVCVR | | | Carbamidomethyl (C)[16], Oxidation (M)[4,11] | | Mascot |
| 2284.2368 | 2284.1746 | -0.0622 | -27 | 492 | 513 | TVVAIFGHDCAPKTLLVSEVGK | | | | | Mascot |
| 2300.0686 | 2300.1807 | 0.1121 | 49 | 597 | 616 | DDQDCILAQFCVQTPQPPPK | | | Carbamidomethyl (C)[5] | | Mascot |
| 2341.2583 | 2341.1233 | -0.135 | -58 | 492 | 513 | TVVAIFGHDCAPKTLLVSEVGK | | | Carbamidomethyl (C)[10] | | Mascot |
| 3000.4343 | 3000.2456 | -0.1887 | -63 | 529 | 553 | HQFVLSRPQDLQIGMYSNMSNYEVK | | | Oxidation (M)[15] | | Mascot |
| 3337.6052 | 3337.7454 | 0.1402 | 42 | 298 | 327 | LARSCHDLESLGQNPGWGQTQPVETNIVCK | | | Carbamidomethyl (C)[5] | | Mascot |
| 3338.5496 | 3338.7615 | 0.2119 | 63 | 885 | 913 | TGMVDSEAMWKPAYDFLLTWAAQIGDSYR | | | Oxidation (M)[3] | | Mascot |

35 stromal interaction molecule 1b [Danio rerio] gi|321400099 72493.8 6.03 11 30 0

**Peptide Information**

| Calc. Mass | Obsrv. Mass | ± da | ± ppm | Start Seq. | End Seq. | Sequence | Ion Score | C. I. % | Modification | Rank | Result Type |
|---|---|---|---|---|---|---|---|---|---|---|---|
| 842.4076 | 842.5089 | 0.1013 | 120 | 555 | 561 | KSFGMEK | | | Oxidation (M)[5] | | Mascot |
| 901.4849 | 901.5057 | 0.0208 | 23 | 289 | 295 | EEAQRLR | | | | | Mascot |
| 959.4801 | 959.5134 | 0.0333 | 35 | 233 | 240 | DHMGKMIK | | | | | Mascot |
| 1051.5643 | 1051.7111 | 0.1468 | 140 | 175 | 183 | IPDRSHAQK | | | | | Mascot |

| | | | | | | | | | | | |
|---|---|---|---|---|---|---|---|---|---|---|---|
| 1062.536 | 1062.6919 | 0.1559 | 147 | 274 | 281 | VNLEREMR | | | Oxidation (M)[7] | | Mascot |
| 1179.5674 | 1179.6022 | 0.0348 | 30 | 279 | 288 | EMRSEIEAAK | | | Oxidation (M)[2] | | Mascot |
| 1751.942 | 1751.814 | -0.128 | -73 | 9 | 21 | HFIYITWLGFLWR | | | | | Mascot |
| 2196.9487 | 2197.1208 | 0.1721 | 78 | 608 | 629 | SLVDEDNGSTGEDTEGS GSGRK | | | | | Mascot |
| 2224.9548 | 2225.1235 | 0.1687 | 76 | 607 | 628 | RSLVDEDNGSTGEDTEG SGSGR | | | | | Mascot |
| 2341.011 | 2341.1233 | 0.1123 | 48 | 508 | 528 | DLNRSESDSSLSMSQYG DGHR | | | | | Mascot |
| 3000.5586 | 3000.2456 | -0.313 | -104 | 207 | 232 | DLMLVVSIIIGVGGCWFA YIQNRNYR | | | | | Mascot |

36 LOC565764 protein [Danio rerio] gi|112418812 25782.2 5.2 6 30 0

**Peptide Information**

| Calc. Mass | Obsrv. Mass | ± da | ± ppm | Start Seq. | End Seq. | Sequence | Ion Score | C. I. % | Modification | Rank | Result Type |
|---|---|---|---|---|---|---|---|---|---|---|---|
| 864.4355 | 864.4704 | 0.0349 | 40 | 77 | 83 | RSMDSIR | | | | | Mascot |
| 882.4389 | 882.5677 | 0.1288 | 146 | 11 | 17 | LEAMFQK | | | Oxidation (M)[4] | | Mascot |
| 901.4988 | 901.5057 | 0.0069 | 8 | 222 | 230 | GLVSLLAND | | | | | Mascot |
| 1112.5293 | 1112.5077 | -0.0216 | -19 | 2 | 10 | LFNMETVDK | | | Oxidation (M)[4] | | Mascot |
| 1767.8694 | 1767.8112 | -0.0582 | -33 | 61 | 76 | HAALCAQVEEIAAEQK | | | Carbamidomethyl (C)[5] | | Mascot |
| 1923.9705 | 1923.9685 | -0.002 | -1 | 61 | 77 | HAALCAQVEEIAAEQKR | | | Carbamidomethyl (C)[5] | | Mascot |

37 Syf2 protein [Danio rerio] gi|60688494 28206.2 7.68 6 30 0

**Peptide Information**

| Calc. Mass | Obsrv. Mass | ± da | ± ppm | Start Seq. | End Seq. | Sequence | Ion Score | C. I. % | Modification | Rank | Result Type |
|---|---|---|---|---|---|---|---|---|---|---|---|
| 870.4468 | 870.54 | 0.0932 | 107 | 217 | 223 | AERFYGK | | | | | Mascot |
| 1436.6686 | 1436.7465 | 0.0779 | 54 | 172 | 183 | DSINRMVDDVEK | | | Oxidation (M)[6] | | Mascot |
| 1736.8888 | 1736.8861 | -0.0027 | -2 | 133 | 146 | LTKQIKPDMENYEK | | | | | Mascot |
| 1999.8992 | 1999.9437 | 0.0445 | 22 | 196 | 212 | AYNDDADIDYINERNAK | | | | | Mascot |
| 1999.8992 | 1999.9437 | 0.0445 | 22 | 196 | 212 | AYNDDADIDYINERNAK | | | | | Mascot |
| 2197.042 | 2197.1208 | 0.0788 | 36 | 114 | 132 | NPDPGFSGYAEAQLRQY QR | | | | | Mascot |
| 2590.1514 | 2590.2161 | 0.0647 | 25 | 149 | 171 | EDCGEDFHPTSNSLIYGT HVPSK | | | Carbamidomethyl (C)[3] | | Mascot |

38 PREDICTED: si:dkey-170m15.2 [Danio rerio] gi|292624792 132327.3 7.37 14 30 0

**Peptide Information**

| Calc. Mass | Obsrv. Mass | ± da | ± ppm | Start Seq. | End Seq. | Sequence | Ion Score | C. I. % | Modification | Rank | Result Type |
|---|---|---|---|---|---|---|---|---|---|---|---|
| 864.472 | 864.4704 | -0.0016 | -2 | 41 | 47 | RTSMLTR | | | | | Mascot |

| Calc. Mass | Obsrv. Mass | ± da | ± ppm | Start Seq. | End Seq. | Sequence | Ion Score | C. I. % | Modification | Rank | Result Type |
|---|---|---|---|---|---|---|---|---|---|---|---|
| 996.5182 | 996.5963 | 0.0781 | 78 | 75 | 83 | DCPLVAPPK | | | Carbamidomethyl (C)[2] | | Mascot |
| 1045.516 | 1045.562 | 0.046 | 44 | 98 | 106 | QTNITDEPK | | | | | Mascot |
| 1103.6167 | 1103.5682 | -0.0485 | -44 | 1159 | 1168 | TTVVQQSGRK | | | | | Mascot |
| 1165.6034 | 1165.7476 | 0.1442 | 124 | 440 | 449 | FCEQAVTIVR | | | | | Mascot |
| 1707.8846 | 1707.776 | -0.1086 | -64 | 436 | 449 | ESLRFCEQAVTIVR | | | Carbamidomethyl (C)[6] | | Mascot |
| 1751.8157 | 1751.814 | -0.0017 | -1 | 583 | 597 | TLQEMWASVESDVEK | | | | | Mascot |
| 1767.8105 | 1767.8112 | 0.0007 | 0 | 583 | 597 | TLQEMWASVESDVEK | | | Oxidation (M)[5] | | Mascot |
| 1908.7925 | 1908.9324 | 0.1399 | 73 | 1104 | 1119 | QMETCIGFQDTMDAYR | | | | | Mascot |
| 2096.8835 | 2097.0769 | 0.1934 | 92 | 1103 | 1119 | RQMETCIGFQDTMDAYR | | | Oxidation (M)[3,13] | | Mascot |
| 2196.9358 | 2197.1208 | 0.185 | 84 | 1104 | 1121 | QMETCIGFQDTMDAYRQK | | | Oxidation (M)[2,12] | | Mascot |
| 2207.1865 | 2207.1174 | -0.0691 | -31 | 399 | 418 | HVLQVHTNASLAVHLHLCSK | | | | | Mascot |
| 2283.1875 | 2283.1833 | -0.0042 | -2 | 663 | 682 | MFSPIPVCILHVINNDTLEK | | | | | Mascot |
| 2460.281 | 2460.1133 | -0.1677 | -68 | 107 | 127 | EPPVQKPEQFIEVSSPFINFK | | | | | Mascot |
| 2489.2605 | 2489.0876 | -0.1729 | -69 | 804 | 827 | GHVIIPVYCFLASNGSSVAQAEAR | | | | | Mascot |

39 follistatin-like 1b [Danio rerio] gi|88900441 35185.6 5.16 8 30 0

**Peptide Information**

| Calc. Mass | Obsrv. Mass | ± da | ± ppm | Start Seq. | End Seq. | Sequence | Ion Score | C. I. % | Modification | Rank | Result Type |
|---|---|---|---|---|---|---|---|---|---|---|---|
| 870.4315 | 870.54 | 0.1085 | 125 | 296 | 302 | HQETVEK | | | | | Mascot |
| 1210.5455 | 1210.5983 | 0.0528 | 44 | 29 | 39 | VCANVFCGAGR | | | Carbamidomethyl (C)[2,7] | | Mascot |
| 1908.9419 | 1908.9324 | -0.0095 | -5 | 81 | 98 | DACLTGLKIQVAHDGHCK | | | | | Mascot |
| 2196.9392 | 2197.1208 | 0.1816 | 83 | 40 | 58 | ECAVNEKGEPSCLCIEQCK | | | Carbamidomethyl (C)[2,12] | | Mascot |
| 2225.0947 | 2225.1235 | 0.0288 | 13 | 212 | 230 | LSFDEFLNCLKPGFSPPEK | | | Carbamidomethyl (C)[9] | | Mascot |
| 2299.9661 | 2300.1807 | 0.2146 | 93 | 270 | 288 | TPVFDTEGDQEMTEEEWTR | | | | | Mascot |
| 2460.0288 | 2460.1133 | 0.0845 | 34 | 231 | 251 | KCALEDETYEDGAETQVECNR | | | Carbamidomethyl (C)[2] | | Mascot |
| 3000.4424 | 3000.2456 | -0.1968 | -66 | 1 | 26 | MMFRCLPVFFLLAVVCCHAEQEAQSK | | | | | Mascot |

40 PREDICTED: transposon TX1 uncharacterized 149 kDa protein-like [Danio rerio] gi|326664772 131122 9.55 15 29 0

**Peptide Information**

| Calc. Mass | Obsrv. Mass | ± da | ± ppm | Start Seq. | End Seq. | Sequence | Ion Score | C. I. % | Modification | Rank | Result Type |
|---|---|---|---|---|---|---|---|---|---|---|---|
| 1045.5499 | 1045.562 | 0.0121 | 12 | 913 | 921 | CPVAFNLPK | | | Carbamidomethyl (C)[1] | | Mascot |
| 1051.5935 | 1051.7111 | 0.1176 | 112 | 824 | 831 | FFLLNLER | | | | | Mascot |

| | | | | | | | | | | | |
|---|---|---|---|---|---|---|---|---|---|---|---|
| 1062.667 | 1062.6919 | 0.0249 | 23 | 537 | 545 | LFTKILATR | | | | | Mascot |
| 1103.5448 | 1103.5682 | 0.0234 | 21 | 1002 | 1011 | AVMHSRGMAK | | | Oxidation (M)[3] | | Mascot |
| 1475.8251 | 1475.7533 | -0.0718 | -49 | 168 | 180 | LDKTSVLLQGMVR | | | Oxidation (M)[11] | | Mascot |
| 1488.639 | 1488.7856 | 0.1466 | 98 | 195 | 207 | EEGFTWFSGDGTR | | | | | Mascot |
| 1488.8573 | 1488.7856 | -0.0717 | -48 | 602 | 613 | ISHQFLFKVLEK | | | | | Mascot |
| 1736.8734 | 1736.8861 | 0.0127 | 7 | 343 | 357 | EVKNEMSVLSEIQSK | | | Oxidation (M)[6] | | Mascot |
| 1751.7871 | 1751.814 | 0.0269 | 15 | 766 | 782 | FDEEGGGGGNWTDLLG K | | | | | Mascot |
| 2097.1086 | 2097.0769 | -0.0317 | -15 | 957 | 975 | EQVSRVPGLTLGEAQQA WK | | | | | Mascot |
| 2193.177 | 2193.1125 | -0.0645 | -29 | 1 | 19 | MSFTHILLMALTISTINVR | | | Oxidation (M)[1,9] | | Mascot |
| 2207.0366 | 2207.1174 | 0.0808 | 37 | 1008 | 1027 | GMAKTSICPRPGCSSPE TVR | | | Carbamidomethyl (C)[8,13], Oxidation (M)[2] | | Mascot |
| 2225.051 | 2225.1235 | 0.0725 | 33 | 546 | 565 | MSMVLEDVIHPDQACAV PGR | | | Carbamidomethyl (C)[15] | | Mascot |
| 2300.1445 | 2300.1807 | 0.0362 | 16 | 327 | 345 | YFNLLNEGLDFNDEIKEV K | | | | | Mascot |
| 2341.1821 | 2341.1233 | -0.0588 | -25 | 324 | 342 | LQRYFNLLNEGLDFNDEI K | | | | | Mascot |

| | | | | | | | |
|---|---|---|---|---|---|---|---|
| 41 | PREDICTED: collagen alpha-1(XXI) chain-like [Danio rerio] | gi\|292629362 | 17314.5 | 9.43 | 5 | 29 | 0 |

**Peptide Information**

| Calc. Mass | Obsrv. Mass | ± da | ± ppm | Start Seq. | End Seq. | Sequence | Ion Score | C. I. % | Modification | Rank | Result Type |
|---|---|---|---|---|---|---|---|---|---|---|---|
| 1112.4248 | 1112.5077 | 0.0829 | 75 | 32 | 39 | SCEHCYTR | | | Carbamidomethyl (C)[2,5] | | Mascot |
| 1165.651 | 1165.7476 | 0.0966 | 83 | 71 | 82 | GLPGRPGVPGMK | | | | | Mascot |
| 2300.2468 | 2300.1807 | -0.0661 | -29 | 12 | 31 | DILRSEIPALLLSSQHSQH R | | | | | Mascot |
| 2460.0676 | 2460.1133 | 0.0457 | 19 | 138 | 160 | GQSGEPGQPGVCHPAM CYGAMIR | | | Carbamidomethyl (C)[12,17] | | Mascot |
| 2515.1716 | 2515.2405 | 0.0689 | 27 | 32 | 55 | SCEHCYTRVGPPGPVGT PGPHGPR | | | Carbamidomethyl (C)[2] | | Mascot |

| | | | | | | | |
|---|---|---|---|---|---|---|---|
| 42 | zinc finger CCHC domain-containing protein 9 [Danio rerio] | gi\|38198649 | 28388.1 | 9.59 | 7 | 29 | 0 |

**Peptide Information**

| Calc. Mass | Obsrv. Mass | ± da | ± ppm | Start Seq. | End Seq. | Sequence | Ion Score | C. I. % | Modification | Rank | Result Type |
|---|---|---|---|---|---|---|---|---|---|---|---|
| 1103.4431 | 1103.5682 | 0.1251 | 113 | 113 | 121 | SNMICFNCR | | | Oxidation (M)[3] | | Mascot |
| 1475.6478 | 1475.7533 | 0.1055 | 71 | 147 | 158 | CGSTEHEIQRCR | | | Carbamidomethyl (C)[1] | | Mascot |
| 1751.7952 | 1751.814 | 0.0188 | 11 | 142 | 156 | GICYRCGSTEHEIQR | | | | | Mascot |
| 1915.9984 | 1915.9589 | -0.0395 | -21 | 228 | 244 | LSNRISADHEDVHVPVK | | | | | Mascot |
| 2196.9397 | 2197.1208 | 0.1811 | 82 | 122 | 141 | KPGHGLADCPEANNDEE MGR | | | Carbamidomethyl (C)[9] | | Mascot |
| 3000.3687 | 3000.2456 | -0.1231 | -41 | 202 | 227 | VCGSVEHFQKDCPEHQN | | | Carbamidomethyl (C)[2,12] | | Mascot |

| Calc. Mass | Obsrv. Mass | ± da | ± ppm | Start Seq. | End Seq. | Sequence | Ion Score | C. I. % | Modification | Rank | Result Type |
|---|---|---|---|---|---|---|---|---|---|---|---|
| 3338.3862 | 3338.7615 | 0.3753 | 112 | 113 | 141 | STNSITLGR SNMICFNCRKPGHGLAD CPEANNDEEMGR | | | Carbamidomethyl (C)[5,8], Oxidation (M)[3] | | Mascot |

43 novel protein similar to vertebrate complement component (3d/Epstein Barr virus) receptor 2 (CR2) [ gi|157885951 53513.7 6.77 10 29 0

**Peptide Information**

| Calc. Mass | Obsrv. Mass | ± da | ± ppm | Start Seq. | End Seq. | Sequence | Ion Score | C. I. % | Modification | Rank | Result Type |
|---|---|---|---|---|---|---|---|---|---|---|---|
| 856.5039 | 856.5234 | 0.0195 | 23 | 441 | 447 | KIFSPHK | | | | | Mascot |
| 996.5407 | 996.5963 | 0.0556 | 56 | 94 | 102 | LRHGVCSPK | | | | | Mascot |
| 1179.496 | 1179.6022 | 0.1062 | 90 | 147 | 156 | TCRDGGWDNR | | | | | Mascot |
| 1210.6136 | 1210.5983 | -0.0153 | -13 | 256 | 266 | ITFSCIGDGLK | | | Carbamidomethyl (C)[5] | | Mascot |
| 2207.0298 | 2207.1174 | 0.0876 | 40 | 269 | 287 | GQREITCQSNGQWSSPF PK | | | Carbamidomethyl (C)[7] | | Mascot |
| 2224.9883 | 2225.1235 | 0.1352 | 61 | 224 | 242 | CVEATCPLNTTMEDLNIE R | | | Carbamidomethyl (C)[1], Oxidation (M)[12] | | Mascot |
| 2341.0652 | 2341.1233 | 0.0581 | 25 | 203 | 223 | LEGESEITCQINGEWSSS FPK | | | | | Mascot |
| 2448.0771 | 2448.1428 | 0.0657 | 27 | 60 | 81 | DSYNPGDTIDFTCQHGFT SSKK | | | | | Mascot |
| 2460.0046 | 2460.1133 | 0.1087 | 44 | 131 | 149 | YICNEGYQMMTQTDTRT CR | | | Carbamidomethyl (C)[3,18], Oxidation (M)[9,10] | | Mascot |
| 3000.3899 | 3000.2456 | -0.1443 | -48 | 407 | 433 | NGEWTGNIYCLNPCSVT LDTMNEKGIK | | | | | Mascot |

44 guanine nucleotide binding protein (G protein), beta polypeptide 1, like [Danio rerio] gi|47087315 37279 5.6 7 29 0

**Peptide Information**

| Calc. Mass | Obsrv. Mass | ± da | ± ppm | Start Seq. | End Seq. | Sequence | Ion Score | C. I. % | Modification | Rank | Result Type |
|---|---|---|---|---|---|---|---|---|---|---|---|
| 1751.8315 | 1751.814 | -0.0175 | -10 | 135 | 150 | VSRELAGHTGYLSCCR | | | | | Mascot |
| 1908.9636 | 1908.9324 | -0.0312 | -16 | 198 | 214 | LFVSGACDASAKLWDIR | | | Carbamidomethyl (C)[7] | | Mascot |
| 1915.9542 | 1915.9589 | 0.0047 | 2 | 24 | 42 | ACADATLSQITANIDPVG R | | | | | Mascot |
| 2157.032 | 2157.1226 | 0.0906 | 42 | 284 | 301 | LLLAGYDDFNCNVWDTL K | | | Carbamidomethyl (C)[11] | | Mascot |
| 2432.1108 | 2432.1375 | 0.0267 | 11 | 315 | 337 | VSCLGVTDDGMAVATGS WDSFLK | | | Carbamidomethyl (C)[3], Oxidation (M)[11] | | Mascot |
| 2432.1108 | 2432.1375 | 0.0267 | 11 | 315 | 337 | VSCLGVTDDGMAVATGS WDSFLK | | | Carbamidomethyl (C)[3], Oxidation (M)[11] | | Mascot |
| 2457.1868 | 2457.3127 | 0.1259 | 51 | 281 | 301 | SGRLLLAGYDDFNCNVW DTLK | | | Carbamidomethyl (C)[14] | | Mascot |
| 2457.1868 | 2457.3127 | 0.1259 | 51 | 281 | 301 | SGRLLLAGYDDFNCNVW DTLK | | | Carbamidomethyl (C)[14] | | Mascot |
| 2460.2334 | 2460.1133 | -0.1201 | -49 | 24 | 46 | ACADATLSQITANIDPVG RIQMR | | | Oxidation (M)[22] | | Mascot |

45 novel protein similar to vertebrate protein kinase C, theta (PRKCQ) [Danio rerio] gi|55962227 81064.5 7.59 12 29 0

Peptide Information

| Calc. Mass | Obsrv. Mass | ± da | ± ppm | Start Seq. | End Seq. | Sequence | Ion Score | C. I. % | Modification | Rank | Result Type |
|---|---|---|---|---|---|---|---|---|---|---|---|
| 850.4781 | 850.5435 | 0.0654 | 77 | 31 | 37 | GQVYKQK | | | | | Mascot |
| 856.5362 | 856.5234 | -0.0128 | -15 | 623 | 630 | LGVKGNIR | | | | | Mascot |
| 901.4737 | 901.5057 | 0.032 | 35 | 529 | 536 | ENIIGEAR | | | | | Mascot |
| 1045.5459 | 1045.562 | 0.0161 | 15 | 272 | 281 | VANLCGVNQK | | | | | Mascot |
| 1751.9227 | 1751.814 | -0.1087 | -62 | 372 | 385 | EHHKFTIDSFILHK | | | | | Mascot |
| 2113.9199 | 2114.187 | 0.2671 | 126 | 415 | 431 | DVVLMDDDVECTMVERR | | | Carbamidomethyl (C)[11], Oxidation (M)[5,13] | | Mascot |
| 2113.9607 | 2114.187 | 0.2263 | 107 | 658 | 675 | SANDCSNFDKEFINEKPR | | | | | Mascot |
| 2157.0334 | 2157.1226 | 0.0892 | 41 | 38 | 55 | RPTMYPPWSSTFDAHVHK | | | | | Mascot |
| 2192.9602 | 2193.1125 | 0.1523 | 69 | 154 | 172 | CHEFTATFFPQPTFCSVCK | | | | | Mascot |
| 2197.2161 | 2197.1208 | -0.0953 | -43 | 92 | 109 | LEIWVELKPQGRLLMEAR | | | Oxidation (M)[15] | | Mascot |
| 2284.0989 | 2284.1746 | 0.0757 | 33 | 537 | 556 | TCTFCGTPDYIAPEILLGQK | | | Carbamidomethyl (C)[2,5] | | Mascot |
| 3337.5266 | 3337.7454 | 0.2188 | 66 | 154 | 180 | CHEFTATFFPQPTFCSVCKEFVWGLNK | | | Carbamidomethyl (C)[1,15,18] | | Mascot |

| | | | | | | | | |
|---|---|---|---|---|---|---|---|---|
| 46 | novel protein similar to vertebrate N-ethylmaleimide-sensitive factor attachment protein, alpha (NA | gi\|123233194 | 33390.2 | 5.53 | 8 | 28 | 0 | |

Peptide Information

| Calc. Mass | Obsrv. Mass | ± da | ± ppm | Start Seq. | End Seq. | Sequence | Ion Score | C. I. % | Modification | Rank | Result Type |
|---|---|---|---|---|---|---|---|---|---|---|---|
| 1112.5524 | 1112.5077 | -0.0447 | -40 | 200 | 208 | YGAKDHFFK | | | | | Mascot |
| 1179.5385 | 1179.6022 | 0.0637 | 54 | 9 | 19 | EAMAIMAEADK | | | | | Mascot |
| 1436.676 | 1436.7465 | 0.0705 | 49 | 7 | 19 | EKEAMAIMAEADK | | | | | Mascot |
| 1542.7223 | 1542.8818 | 0.1595 | 103 | 141 | 153 | AIAHYEQAADYYK | | | | | Mascot |
| 1908.8289 | 1908.9324 | 0.1035 | 54 | 38 | 53 | VEEACDMYVRAANMFK | | | Oxidation (M)[7,14] | | Mascot |
| 2000.0171 | 1999.9437 | -0.0734 | -37 | 57 | 74 | NWNAAGDAFCKAALLHLK | | | Carbamidomethyl (C)[10] | | Mascot |
| 2000.0171 | 1999.9437 | -0.0734 | -37 | 57 | 74 | NWNAAGDAFCKAALLHLK | | | Carbamidomethyl (C)[10] | | Mascot |
| 2157.0571 | 2157.1226 | 0.0655 | 30 | 246 | 264 | KLLDAHDEQNIDAYADAVR | | | | | Mascot |
| 2294.0911 | 2294.1646 | 0.0735 | 32 | 209 | 227 | AALCHFCVDMLNCKLAVQK | | | Carbamidomethyl (C)[4,7,13], Oxidation (M)[10] | | Mascot |
| 2294.0911 | 2294.1646 | 0.0735 | 32 | 209 | 227 | AALCHFCVDMLNCKLAVQK | | | Carbamidomethyl (C)[4,7,13], Oxidation (M)[10] | | Mascot |

| | | | | | | | | |
|---|---|---|---|---|---|---|---|---|
| 47 | plexin b2b [Danio rerio] | gi\|309243104 | 203041.6 | 6.62 | 17 | 28 | 0 | |

Peptide Information

| Calc. Mass | Obsrv. Mass | ± da | ± ppm | Start | End | Sequence | Ion | C. I. % | Modification | Rank | Result Type |
|---|---|---|---|---|---|---|---|---|---|---|---|

| Calc. Mass | Obsrv. Mass | ± da | ± ppm | Start Seq. | End Seq. | Sequence | Ion Score | C. I. % | Modification | Rank | Result Type |
|---|---|---|---|---|---|---|---|---|---|---|---|
| 850.4206 | 850.5435 | 0.1229 | 145 | 213 | 218 | YLHDFR | | | | | Mascot |
| 870.4791 | 870.54 | 0.0609 | 70 | 1699 | 1705 | TEHKLSR | | | | | Mascot |
| 996.522 | 996.5963 | 0.0743 | 75 | 369 | 376 | HDKDIVNR | | | | | Mascot |
| 1044.5684 | 1044.4929 | -0.0755 | -72 | 890 | 899 | SGVVEIEVGR | | | | | Mascot |
| 1051.5717 | 1051.7111 | 0.1394 | 133 | 479 | 487 | VPVQACHLK | | | Carbamidomethyl (C)[6] | | Mascot |
| 1103.5626 | 1103.5682 | 0.0056 | 5 | 857 | 866 | NITVAGVNCR | | | Carbamidomethyl (C)[9] | | Mascot |
| 1436.6144 | 1436.7465 | 0.1321 | 92 | 783 | 794 | EDCSLCKNADPK | | | Carbamidomethyl (C)[3,6] | | Mascot |
| 1542.8196 | 1542.8818 | 0.0622 | 40 | 1585 | 1597 | SMTKAITEIYLTR | | | Oxidation (M)[2] | | Mascot |
| 1719.8041 | 1719.8544 | 0.0503 | 29 | 72 | 87 | DAKGCTPPVSTCQDLK | | | Carbamidomethyl (C)[5] | | Mascot |
| 1719.8041 | 1719.8544 | 0.0503 | 29 | 72 | 87 | DAKGCTPPVSTCQDLK | | | Carbamidomethyl (C)[5] | | Mascot |
| 1901.8889 | 1901.9904 | 0.1015 | 53 | 1000 | 1016 | AAFQFSNNPSVSDHQPR | | | | | Mascot |
| 1908.9056 | 1908.9324 | 0.0268 | 14 | 1374 | 1388 | MLSNWMSICLYQYLK | | | Oxidation (M)[1] | | Mascot |
| 2136.1235 | 2136.1672 | 0.0437 | 20 | 908 | 925 | TVQFTYRDPVPLSVFPNR | | | | | Mascot |
| 2192.991 | 2193.1125 | 0.1215 | 55 | 75 | 94 | GCTPPVSTCQDLKDTNNTNK | | | Carbamidomethyl (C)[2] | | Mascot |
| 2300.0752 | 2300.1807 | 0.1055 | 46 | 286 | 305 | ELAQVMTDSGEYGTIKEWDK | | | | | Mascot |
| 2460.2036 | 2460.1133 | -0.0903 | -37 | 306 | 328 | VLFVVASSEEGATESALCMYPLK | | | Oxidation (M)[19] | | Mascot |
| 2590.1946 | 2590.2161 | 0.0215 | 8 | 767 | 789 | IDSSLNVTLYNCSLGREDCSLCK | | | Carbamidomethyl (C)[12] | | Mascot |
| 3337.5974 | 3337.7454 | 0.148 | 44 | 1086 | 1113 | TFVQLDNLQMELTPFAYYPNPTFDELSK | | | Oxidation (M)[10] | | Mascot |

| | | | | | | | |
|---|---|---|---|---|---|---|---|
| 48 | Mios protein [Danio rerio] | gi\|45767831 | 26550.6 | 8.45 | 8 | 28 | 0 |

**Peptide Information**

| Calc. Mass | Obsrv. Mass | ± da | ± ppm | Start Seq. | End Seq. | Sequence | Ion Score | C. I. % | Modification | Rank | Result Type |
|---|---|---|---|---|---|---|---|---|---|---|---|
| 1151.5295 | 1151.6196 | 0.0901 | 78 | 137 | 146 | SKVTSCPGCR | | | Carbamidomethyl (C)[6,9] | | Mascot |
| 1210.5674 | 1210.5983 | 0.0309 | 26 | 60 | 68 | VQCWIENYR | | | | | Mascot |
| 1436.5603 | 1436.7465 | 0.1862 | 130 | 212 | 224 | DHSECPVSACTCK | | | Carbamidomethyl (C)[5] | | Mascot |
| 1736.783 | 1736.8861 | 0.1031 | 59 | 225 | 240 | CMQLDTTGNLVPSDSV | | | Carbamidomethyl (C)[1] | | Mascot |
| 2136.0444 | 2136.1672 | 0.1228 | 57 | 60 | 75 | VQCWIENYRNLLDAWR | | | Carbamidomethyl (C)[3] | | Mascot |
| 2156.9265 | 2157.1226 | 0.1961 | 91 | 152 | 171 | CALCLMNMGTPVSSCPGTGK | | | Carbamidomethyl (C)[1,4,15], Oxidation (M)[6] | | Mascot |
| 2197.0779 | 2197.1208 | 0.0429 | 20 | 90 | 109 | LDPSSKPLAQVFVSCNFCGK | | | Carbamidomethyl (C)[15] | | Mascot |
| 2225.094 | 2225.1235 | 0.0295 | 13 | 36 | 56 | TGDVQTASFCMLKGSPGEVLK | | | Carbamidomethyl (C)[10] | | Mascot |

| | | | | | | | |
|---|---|---|---|---|---|---|---|
| 49 | fizzy/cell division cycle 20 related 1 (Drosophila) [Danio rerio] | gi\|169146644 | 9957.9 | 9.25 | 4 | 28 | 0 |

Peptide Information

| Calc. Mass | Obsrv. Mass | ± da | ± ppm | Start Seq. | End Seq. | Sequence | Ion Score | C. I. % | Modification | Rank | Result Type |
|---|---|---|---|---|---|---|---|---|---|---|---|
| 1112.479 | 1112.5077 | 0.0287 | 26 | 1 | 8 | MDQDYERR | | | | | Mascot |
| 1488.7112 | 1488.7856 | 0.0744 | 50 | 12 | 24 | QINVQNDNTSPMK | | | | | Mascot |
| 1488.7112 | 1488.7856 | 0.0744 | 50 | 12 | 24 | QINVQNDNTSPMK | | | | | Mascot |
| 1999.9733 | 1999.9437 | -0.0296 | -15 | 55 | 71 | AGANWSINFHRINENEK | | | | | Mascot |
| 1999.9733 | 1999.9437 | -0.0296 | -15 | 55 | 71 | AGANWSINFHRINENEK | | | | | Mascot |
| 2207.1377 | 2207.1174 | -0.0203 | -9 | 25 | 45 | AKDVICHLTPTPESPLSS PSK | | | | | Mascot |

| | | | | | | | | | |
|---|---|---|---|---|---|---|---|---|---|
| 50 | PREDICTED: zinc finger protein 217-like [Danio rerio] | gi\|326679281 | 105874.2 | 9.01 | 1 | | | 15 | 0 |

Peptide Information

| Calc. Mass | Obsrv. Mass | ± da | ± ppm | Start Seq. | End Seq. | Sequence | Ion Score | C. I. % | Modification | Rank | Result Type |
|---|---|---|---|---|---|---|---|---|---|---|---|
| 1354.7438 | 1354.7678 | 0.024 | 18 | 741 | 752 | GRELDATNLLPR | 15 | 0 | | 3 | Mascot |

| Gel Idx/Pos | 191/H18 | Instr./Gel Origin | BA2060/110707A | Process Status | Analysis Succeeded |
|---|---|---|---|---|---|
| Plate [#] Name | [1] 31496 | Instrument Sample Name | | Spectra | 11 |

| Rank | Protein Name | Accession No. | Protein MW | Protein PI | Pep. Count | Protein Score | Protein Score C. I. % | Total Ion Score | Total Ion C. I. % |
|---|---|---|---|---|---|---|---|---|---|
| 1 | Tkt protein [Danio rerio] | gi\|182890016 | 67962.5 | 6.79 | 25 | 1,310 | 100 | 1117 | 100 |

Peptide Information

| Calc. Mass | Obsrv. Mass | ± da | ± ppm | Start Seq. | End Seq. | Sequence | Ion Score | C. I. % | Modification | Rank | Result Type |
|---|---|---|---|---|---|---|---|---|---|---|---|
| 864.421 | 864.4503 | 0.0293 | 34 | 57 | 63 | YKADDPR | | | | | Mascot |
| 959.5156 | 959.5182 | 0.0026 | 3 | 93 | 100 | ESDLLNLR | | | | | Mascot |
| 1076.4717 | 1076.4761 | 0.0044 | 4 | 347 | 355 | NSTFADMFK | | | Oxidation (M)[7] | | Mascot |
| 1103.5731 | 1103.575 | 0.0019 | 2 | 559 | 567 | VITVEDHYK | | | | | Mascot |
| 1112.5081 | 1112.5112 | 0.0031 | 3 | 226 | 234 | YMWEAEGVK | | | | | Mascot |
| 1128.5031 | 1128.5063 | 0.0032 | 3 | 226 | 234 | YMWEAEGVK | | | Oxidation (M)[2] | | Mascot |
| 1354.7689 | 1354.7754 | 0.0065 | 5 | 174 | 185 | LDNLVAILDVNR | 111 | 100 | | | Mascot |
| 1354.7689 | 1354.7754 | 0.0065 | 5 | 174 | 185 | LDNLVAILDVNR | | | | | Mascot |
| 1436.738 | 1436.7466 | 0.0086 | 6 | 101 | 113 | KIDSDLEGHPTPK | | | | | Mascot |
| 1488.7845 | 1488.7906 | 0.0061 | 4 | 385 | 398 | TVSFASTFAAFLAR | 118 | 100 | | | Mascot |
| 1488.7845 | 1488.7906 | 0.0061 | 4 | 385 | 398 | TVSFASTFAAFLAR | | | | | Mascot |
| 1719.8547 | 1719.859 | 0.0043 | 3 | 475 | 489 | TSRPDTAVIYNPEEK | | | | | Mascot |

| | | | | | | | | | | |
|---|---|---|---|---|---|---|---|---|---|---|
| 1719.8547 | 1719.859 | 0.0043 | 3 | 475 | 489 | TSRPDTAVIYNPEEK | 59 | 99.97 | | Mascot |
| 1736.8887 | 1736.8944 | 0.0057 | 3 | 598 | 613 | SGKPQELLDMFGISAK | | | Oxidation (M)[10] | Mascot |
| 1751.817 | 1751.8234 | 0.0064 | 4 | 252 | 266 | GIEDQDNWHGKPMPK | | | | Mascot |
| 1767.8119 | 1767.8188 | 0.0069 | 4 | 252 | 266 | GIEDQDNWHGKPMPK | | | Oxidation (M)[13] | Mascot |
| 1893.9817 | 1893.9885 | 0.0068 | 4 | 186 | 202 | LGQSEPAPLQHNVNVYK | | | | Mascot |
| 1893.9817 | 1893.9885 | 0.0068 | 4 | 186 | 202 | LGQSEPAPLQHNVNVYK | 95 | 100 | | Mascot |
| 1901.9908 | 1901.9963 | 0.0055 | 3 | 75 | 92 | GHAAPILYAAWAEAGYIK | | | | Mascot |
| 1999.943 | 1999.9485 | 0.0055 | 3 | 442 | 459 | AIPTCTVFYPSDGVSTER | | | Carbamidomethyl (C)[5] | Mascot |
| 1999.943 | 1999.9485 | 0.0055 | 3 | 442 | 459 | AIPTCTVFYPSDGVSTER | 135 | 100 | Carbamidomethyl (C)[5] | Mascot |
| 2114.1855 | 2114.1943 | 0.0088 | 4 | 534 | 553 | VIDPFTIKPLDASTIVASA R | 148 | 100 | | Mascot |
| 2114.1855 | 2114.1943 | 0.0088 | 4 | 534 | 553 | VIDPFTIKPLDASTIVASA R | | | | Mascot |
| 2175.1404 | 2175.1472 | 0.0068 | 3 | 568 | 589 | EGGLGEAVLSAVGEEPGI VVHR | 216 | 100 | | Mascot |
| 2175.1404 | 2175.1472 | 0.0068 | 3 | 568 | 589 | EGGLGEAVLSAVGEEPGI VVHR | | | | Mascot |
| 2294.1663 | 2294.1719 | 0.0056 | 2 | 475 | 494 | TSRPDTAVIYNPEEKFEIG K | | | | Mascot |
| 2294.1663 | 2294.1719 | 0.0056 | 2 | 475 | 494 | TSRPDTAVIYNPEEKFEIG K | 52 | 99.858 | | Mascot |
| 2432.1406 | 2432.1443 | 0.0037 | 2 | 362 | 382 | YIECFIAEQNMVSVAIGCA TR | | | Carbamidomethyl (C)[4,18] | Mascot |
| 2432.1406 | 2432.1443 | 0.0037 | 2 | 362 | 382 | YIECFIAEQNMVSVAIGCA TR | 182 | 100 | Carbamidomethyl (C)[4,18] | Mascot |
| 2448.1355 | 2448.1497 | 0.0142 | 6 | 362 | 382 | YIECFIAEQNMVSVAIGCA TR | | | Carbamidomethyl (C)[4,18], Oxidation (M)[11] | Mascot |
| 2448.1355 | 2448.1497 | 0.0142 | 6 | 362 | 382 | YIECFIAEQNMVSVAIGCA TR | 50 | 99.764 | Carbamidomethyl (C)[4,18], Oxidation (M)[11] | Mascot |
| 2457.3096 | 2457.3203 | 0.0107 | 4 | 504 | 527 | DQVTVIGAGVTLHEALAA HDQLAK | | | | Mascot |
| 2459.1006 | 2459.1106 | 0.01 | 4 | 205 | 225 | CVAFGFNTYVVDGHDVE ELCK | | | Carbamidomethyl (C)[1,20] | Mascot |
| 2515.2319 | 2515.2476 | 0.0157 | 6 | 114 | 139 | LAFVDVATGSLGQGLGA ACGMAYTGK | | | Carbamidomethyl (C)[19] | Mascot |
| 2531.2268 | 2531.2427 | 0.0159 | 6 | 114 | 139 | LAFVDVATGSLGQGLGA ACGMAYTGK | | | Carbamidomethyl (C)[19], Oxidation (M)[21] | Mascot |
| 2984.2358 | 2984.2456 | 0.0098 | 3 | 148 | 173 | VYCMLGDGECSEGSVW EAMAFASHYK | | | Carbamidomethyl (C)[3,10] | Mascot |
| 3000.2307 | 3000.2488 | 0.0181 | 6 | 148 | 173 | VYCMLGDGECSEGSVW EAMAFASHYK | | | Carbamidomethyl (C)[3,10], Oxidation (M)[4] | Mascot |
| 3103.6423 | 3103.6494 | 0.0071 | 2 | 267 | 293 | DRAEELINDLLSQIQSPN KPLYPQPPK | | | | Mascot |
| 3287.4158 | 3287.4243 | 0.0085 | 3 | 28 | 56 | QTCASNSGHPTSCCSAA ELMSVLFFHTMR | | | Carbamidomethyl (C)[3,13,14] | Mascot |
| 3303.4109 | 3303.4224 | 0.0115 | 3 | 28 | 56 | QTCASNSGHPTSCCSAA ELMSVLFFHTMR | | | Carbamidomethyl (C)[3,13,14], Oxidation (M)[20] | Mascot |
| 3319.4058 | 3319.4426 | 0.0368 | 11 | 28 | 56 | QTCASNSGHPTSCCSAA ELMSVLFFHTMR | | | Carbamidomethyl (C)[3,13,14], Oxidation (M)[20,28] | Mascot |
| 3891.8132 | 3891.8274 | 0.0142 | 4 | 405 | 441 | MAAISQSNVNLVGSHCG VSIGEDGPSQMALEDLA MFR | | | Carbamidomethyl (C)[16] | Mascot |
| 3907.8081 | 3907.8484 | 0.0403 | 10 | 405 | 441 | MAAISQSNVNLVGSHCG VSIGEDGPSQMALEDLA | | | Carbamidomethyl (C)[16], Oxidation (M)[1] | Mascot |

| | | | | | | | | | | |
|---|---|---|---|---|---|---|---|---|---|---|
| | | | MFR | | | | | | | |
| 2 | transketolase-like protein 2 [Danio rerio] | | gi\|186288308 | 67795.3 | 6.81 | 23 | 1,280 | 100 | 1114 | 100 |

**Peptide Information**

| Calc. Mass | Obsrv. Mass | ± da | ± ppm | Start Seq. | End Seq. | Sequence | Ion Score | C. I. % | Modification | Rank | Result Type |
|---|---|---|---|---|---|---|---|---|---|---|---|
| 901.5101 | 901.5096 | -0.0005 | -1 | 93 | 100 | ASDLLNLR | | | | | Mascot |
| 1076.4717 | 1076.4761 | 0.0044 | 4 | 347 | 355 | NSTFADMFK | | | Oxidation (M)[7] | | Mascot |
| 1103.5731 | 1103.575 | 0.0019 | 2 | 559 | 567 | VITVEDHYK | | | | | Mascot |
| 1354.7689 | 1354.7754 | 0.0065 | 5 | 174 | 185 | LDNLVAILDVNR | 111 | 100 | | | Mascot |
| 1354.7689 | 1354.7754 | 0.0065 | 5 | 174 | 185 | LDNLVAILDVNR | | | | | Mascot |
| 1436.738 | 1436.7466 | 0.0086 | 6 | 101 | 113 | KIDSDLEGHPTPK | | | | | Mascot |
| 1488.7845 | 1488.7906 | 0.0061 | 4 | 385 | 398 | TVSFASTFAAFLAR | 118 | 100 | | | Mascot |
| 1488.7845 | 1488.7906 | 0.0061 | 4 | 385 | 398 | TVSFASTFAAFLAR | | | | | Mascot |
| 1719.8547 | 1719.859 | 0.0043 | 3 | 475 | 489 | TSRPDTAVIYNPEEK | | | | | Mascot |
| 1719.8547 | 1719.859 | 0.0043 | 3 | 475 | 489 | TSRPDTAVIYNPEEK | 59 | 99.97 | | | Mascot |
| 1736.8887 | 1736.8944 | 0.0057 | 3 | 598 | 613 | SGKPQELLDMFGISAK | | | Oxidation (M)[10] | | Mascot |
| 1751.817 | 1751.8234 | 0.0064 | 4 | 252 | 266 | GIEDQDNWHGKPMPK | | | | | Mascot |
| 1767.8119 | 1767.8188 | 0.0069 | 4 | 252 | 266 | GIEDQDNWHGKPMPK | | | Oxidation (M)[13] | | Mascot |
| 1893.9817 | 1893.9885 | 0.0068 | 4 | 186 | 202 | LGQSEPAPLQHNVNVYK | | | | | Mascot |
| 1893.9817 | 1893.9885 | 0.0068 | 4 | 186 | 202 | LGQSEPAPLQHNVNVYK | 95 | 100 | | | Mascot |
| 1901.9908 | 1901.9963 | 0.0055 | 3 | 75 | 92 | GHAAPILYAAWAEAGYIK | | | | | Mascot |
| 1999.943 | 1999.9485 | 0.0055 | 3 | 442 | 459 | AIPTCTVFYPSDGVSTER | | | Carbamidomethyl (C)[5] | | Mascot |
| 1999.943 | 1999.9485 | 0.0055 | 3 | 442 | 459 | AIPTCTVFYPSDGVSTER | 135 | 100 | Carbamidomethyl (C)[5] | | Mascot |
| 2114.1855 | 2114.1943 | 0.0088 | 4 | 534 | 553 | VIDPFTIKPLDASTIVASA R | 148 | 100 | | | Mascot |
| 2114.1855 | 2114.1943 | 0.0088 | 4 | 534 | 553 | VIDPFTIKPLDASTIVASA R | | | | | Mascot |
| 2175.1404 | 2175.1472 | 0.0068 | 3 | 568 | 589 | EGGLGEAVLSAVGEEPGI VVHR | 216 | 100 | | | Mascot |
| 2175.1404 | 2175.1472 | 0.0068 | 3 | 568 | 589 | EGGLGEAVLSAVGEEPGI VVHR | | | | | Mascot |
| 2294.1663 | 2294.1719 | 0.0056 | 2 | 475 | 494 | TSRPDTAVIYNPEEKFEIG K | | | | | Mascot |
| 2294.1663 | 2294.1719 | 0.0056 | 2 | 475 | 494 | TSRPDTAVIYNPEEKFEIG K | 52 | 99.858 | | | Mascot |
| 2432.1406 | 2432.1443 | 0.0037 | 2 | 362 | 382 | YIECFIAEQNMVSVAIGCA TR | | | Carbamidomethyl (C)[4,18] | | Mascot |
| 2432.1406 | 2432.1443 | 0.0037 | 2 | 362 | 382 | YIECFIAEQNMVSVAIGCA TR | 182 | 100 | Carbamidomethyl (C)[4,18] | | Mascot |
| 2448.1355 | 2448.1497 | 0.0142 | 6 | 362 | 382 | YIECFIAEQNMVSVAIGCA TR | | | Carbamidomethyl (C)[4,18], Oxidation (M)[11] | | Mascot |
| 2448.1355 | 2448.1497 | 0.0142 | 6 | 362 | 382 | YIECFIAEQNMVSVAIGCA TR | 50 | 99.764 | Carbamidomethyl (C)[4,18], Oxidation (M)[11] | | Mascot |
| 2457.3096 | 2457.3203 | 0.0107 | 4 | 504 | 527 | DQVTVIGAGVTLHEALAA HDQLAK | | | | | Mascot |

| Calc. Mass | Obsrv. Mass | ± da | ± ppm | Start Seq. | End Seq. | Sequence | Ion Score | C. I. % | Modification | Rank | Result Type |
|---|---|---|---|---|---|---|---|---|---|---|---|
| 2489.0747 | 2489.0869 | 0.0122 | 5 | 205 | 225 | CEAFGFNTYVVDGHDVE ELCK | | | Carbamidomethyl (C)[1,20] | | Mascot |
| 2515.2319 | 2515.2476 | 0.0157 | 6 | 114 | 139 | LAFVDVATGSLGQGLGA ACGMAYTGK | | | Carbamidomethyl (C)[19] | | Mascot |
| 2531.2268 | 2531.2427 | 0.0159 | 6 | 114 | 139 | LAFVDVATGSLGQGLGA ACGMAYTGK | | | Carbamidomethyl (C)[19], Oxidation (M)[21] | | Mascot |
| 2984.2358 | 2984.2456 | 0.0098 | 3 | 148 | 173 | VYCMLGDGECSEGSVW EAMAFASHYK | | | Carbamidomethyl (C)[3,10] | | Mascot |
| 3000.2307 | 3000.2488 | 0.0181 | 6 | 148 | 173 | VYCMLGDGECSEGSVW EAMAFASHYK | | | Carbamidomethyl (C)[3,10], Oxidation (M)[4] | | Mascot |
| 3103.6423 | 3103.6494 | 0.0071 | 2 | 267 | 293 | DRAEELINDLLSQIQSPN KPLYPQPPK | | | | | Mascot |
| 3287.4158 | 3287.4243 | 0.0085 | 3 | 28 | 56 | QTCASNSGHPTSCCSAA ELMSVLFFHTMR | | | Carbamidomethyl (C)[3,13,14] | | Mascot |
| 3303.4109 | 3303.4224 | 0.0115 | 3 | 28 | 56 | QTCASNSGHPTSCCSAA ELMSVLFFHTMR | | | Carbamidomethyl (C)[3,13,14], Oxidation (M)[20] | | Mascot |
| 3319.4058 | 3319.4426 | 0.0368 | 11 | 28 | 56 | QTCASNSGHPTSCCSAA ELMSVLFFHTMR | | | Carbamidomethyl (C)[3,13,14], Oxidation (M)[20,28] | | Mascot |
| 3891.8132 | 3891.8274 | 0.0142 | 4 | 405 | 441 | MAAISQSNVNLVGSHCG VSIGEDGPSQMALEDLA MFR | | | Carbamidomethyl (C)[16] | | Mascot |
| 3907.8081 | 3907.8484 | 0.0403 | 10 | 405 | 441 | MAAISQSNVNLVGSHCG VSIGEDGPSQMALEDLA MFR | | | Carbamidomethyl (C)[16], Oxidation (M)[1] | | Mascot |

| | | | | | | | | | | |
|---|---|---|---|---|---|---|---|---|---|---|
| 3 | transketolase [Danio rerio] | gi\|31872040 | 67811.3 | 6.81 | 22 | 1,140 | 100 | 978 | 100 | |

**Peptide Information**

| Calc. Mass | Obsrv. Mass | ± da | ± ppm | Start Seq. | End Seq. | Sequence | Ion Score | C. I. % | Modification | Rank | Result Type |
|---|---|---|---|---|---|---|---|---|---|---|---|
| 901.5101 | 901.5096 | -0.0005 | -1 | 93 | 100 | ASDLLNLR | | | | | Mascot |
| 1076.4717 | 1076.4761 | 0.0044 | 4 | 347 | 355 | NSTFADMFK | | | Oxidation (M)[7] | | Mascot |
| 1103.5731 | 1103.575 | 0.0019 | 2 | 559 | 567 | VITVEDHYK | | | | | Mascot |
| 1354.7689 | 1354.7754 | 0.0065 | 5 | 174 | 185 | LDNLVAILDVNR | 111 | 100 | | | Mascot |
| 1354.7689 | 1354.7754 | 0.0065 | 5 | 174 | 185 | LDNLVAILDVNR | | | | | Mascot |
| 1436.738 | 1436.7466 | 0.0086 | 6 | 101 | 113 | KIDSDLEGHPTPK | | | | | Mascot |
| 1488.7845 | 1488.7906 | 0.0061 | 4 | 385 | 398 | TVSFASTFAAFLAR | 118 | 100 | | | Mascot |
| 1488.7845 | 1488.7906 | 0.0061 | 4 | 385 | 398 | TVSFASTFAAFLAR | | | | | Mascot |
| 1719.8547 | 1719.859 | 0.0043 | 3 | 475 | 489 | TSRPDTAVIYNPEEK | | | | | Mascot |
| 1719.8547 | 1719.859 | 0.0043 | 3 | 475 | 489 | TSRPDTAVIYNPEEK | 59 | 99.97 | | | Mascot |
| 1736.8887 | 1736.8944 | 0.0057 | 3 | 598 | 613 | SGKPQELLDMFGISAK | | | Oxidation (M)[10] | | Mascot |
| 1751.817 | 1751.8234 | 0.0064 | 4 | 252 | 266 | GIEDQDNWHGKPMPK | | | | | Mascot |
| 1767.8119 | 1767.8188 | 0.0069 | 4 | 252 | 266 | GIEDQDNWHGKPMPK | | | Oxidation (M)[13] | | Mascot |
| 1893.9817 | 1893.9885 | 0.0068 | 4 | 186 | 202 | LGQSEPAPLQHNVNVYK | | | | | Mascot |
| 1893.9817 | 1893.9885 | 0.0068 | 4 | 186 | 202 | LGQSEPAPLQHNVNVYK | 95 | 100 | | | Mascot |
| 1901.9908 | 1901.9963 | 0.0055 | 3 | 75 | 92 | GHAAPILYAAWAEAGYIK | | | | | Mascot |
| 2114.1855 | 2114.1943 | 0.0088 | 4 | 534 | 553 | VIDPFTIKPLDASTIVASA R | 148 | 100 | | | Mascot |

| | | | | | | | | | | | |
|---|---|---|---|---|---|---|---|---|---|---|---|
| 2114.1855 | 2114.1943 | 0.0088 | 4 | 534 | 553 | VIDPFTIKPLDASTIVASA R | | | | | Mascot |
| 2175.1404 | 2175.1472 | 0.0068 | 3 | 568 | 589 | EGGLGEAVLSAVGEEPGI VVHR | 216 | 100 | | | Mascot |
| 2175.1404 | 2175.1472 | 0.0068 | 3 | 568 | 589 | EGGLGEAVLSAVGEEPGI VVHR | | | | | Mascot |
| 2294.1663 | 2294.1719 | 0.0056 | 2 | 475 | 494 | TSRPDTAVIYNPEEKFEIG K | | | | | Mascot |
| 2294.1663 | 2294.1719 | 0.0056 | 2 | 475 | 494 | TSRPDTAVIYNPEEKFEIG K | 52 | 99.858 | | | Mascot |
| 2432.1406 | 2432.1443 | 0.0037 | 2 | 362 | 382 | YIECFIAEQNMVSVAIGCA TR | | | Carbamidomethyl (C)[4,18] | | Mascot |
| 2432.1406 | 2432.1443 | 0.0037 | 2 | 362 | 382 | YIECFIAEQNMVSVAIGCA TR | 182 | 100 | Carbamidomethyl (C)[4,18] | | Mascot |
| 2448.1355 | 2448.1497 | 0.0142 | 6 | 362 | 382 | YIECFIAEQNMVSVAIGCA TR | | | Carbamidomethyl (C)[4,18], Oxidation (M)[11] | | Mascot |
| 2448.1355 | 2448.1497 | 0.0142 | 6 | 362 | 382 | YIECFIAEQNMVSVAIGCA TR | 50 | 99.764 | Carbamidomethyl (C)[4,18], Oxidation (M)[11] | | Mascot |
| 2457.3096 | 2457.3203 | 0.0107 | 4 | 504 | 527 | DQVTVIGAGVTLHEALAA HDQLAK | | | | | Mascot |
| 2489.0747 | 2489.0869 | 0.0122 | 5 | 205 | 225 | CEAFGFNTYVVDGHDVE ELCK | | | Carbamidomethyl (C)[1,20] | | Mascot |
| 2515.2319 | 2515.2476 | 0.0157 | 6 | 114 | 139 | LAFVDVATGSLGQGLGA ACGMAYTGK | | | Carbamidomethyl (C)[19] | | Mascot |
| 2531.2268 | 2531.2427 | 0.0159 | 6 | 114 | 139 | LAFVDVATGSLGQGLGA ACGMAYTGK | | | Carbamidomethyl (C)[19], Oxidation (M)[21] | | Mascot |
| 2984.2358 | 2984.2456 | 0.0098 | 3 | 148 | 173 | VYCMLGDGECSEGSVW EAMAFASHYK | | | Carbamidomethyl (C)[3,10] | | Mascot |
| 3000.2307 | 3000.2488 | 0.0181 | 6 | 148 | 173 | VYCMLGDGECSEGSVW EAMAFASHYK | | | Carbamidomethyl (C)[3,10], Oxidation (M)[4] | | Mascot |
| 3103.6423 | 3103.6494 | 0.0071 | 2 | 267 | 293 | DRAEELINDLLSQIQSPN KPLYPQPPK | | | | | Mascot |
| 3287.4158 | 3287.4243 | 0.0085 | 3 | 28 | 56 | QTCASNSGHPTSCCSAA ELMSVLFFHTMR | | | Carbamidomethyl (C)[3,13,14] | | Mascot |
| 3303.4109 | 3303.4224 | 0.0115 | 3 | 28 | 56 | QTCASNSGHPTSCCSAA ELMSVLFFHTMR | | | Carbamidomethyl (C)[3,13,14], Oxidation (M)[20] | | Mascot |
| 3319.4058 | 3319.4426 | 0.0368 | 11 | 28 | 56 | QTCASNSGHPTSCCSAA ELMSVLFFHTMR | | | Carbamidomethyl (C)[3,13,14], Oxidation (M)[20,28] | | Mascot |
| 3891.8132 | 3891.8274 | 0.0142 | 4 | 405 | 441 | MAAISQSNVNLVGSHCG VSIGEDGPSQMALEDLA MFR | | | Carbamidomethyl (C)[16] | | Mascot |
| 3907.8081 | 3907.8484 | 0.0403 | 10 | 405 | 441 | MAAISQSNVNLVGSHCG VSIGEDGPSQMALEDLA MFR | | | Carbamidomethyl (C)[16], Oxidation (M)[1] | | Mascot |

| | | | | | | | |
|---|---|---|---|---|---|---|---|
| 4 | PREDICTED: NACHT, LRR and PYD domains-containing protein 12 [Danio rerio] | gi\|292621731 | 149261.8 | 5.29 | 15 | 36 | 0 |

**Peptide Information**

| Calc. Mass | Obsrv. Mass | ± da | ± ppm | Start Seq. | End Seq. | Sequence | Ion Score | C. I. % | Modification | Rank | Result Type |
|---|---|---|---|---|---|---|---|---|---|---|---|
| 842.4114 | 842.5123 | 0.1009 | 120 | 1214 | 1220 | SNSEHLR | | | | | Mascot |
| 864.5777 | 864.4503 | -0.1274 | -147 | 133 | 139 | KILVVHR | | | | | Mascot |
| 901.5829 | 901.5096 | -0.0733 | -81 | 507 | 514 | AIRTVLTK | | | | | Mascot |
| 959.5156 | 959.5182 | 0.0026 | 3 | 872 | 880 | AVNEALQSK | | | | | Mascot |
| 1076.5007 | 1076.4761 | -0.0246 | -23 | 741 | 748 | HQKDYEEK | | | | | Mascot |

| | | | | | | | | | | | |
|---|---|---|---|---|---|---|---|---|---|---|---|
| 1436.7744 | 1436.7466 | -0.0278 | -19 | 913 | 924 | SIQNNKTVEYIK | | | | | Mascot |
| 1488.7289 | 1488.7906 | 0.0617 | 41 | 375 | 388 | SGDTKSDLSSVHQK | | | | | Mascot |
| 1488.7289 | 1488.7906 | 0.0617 | 41 | 375 | 388 | SGDTKSDLSSVHQK | | | | | Mascot |
| 1916.0157 | 1915.9642 | -0.0515 | -27 | 1115 | 1132 | LGDSVTLLSAVLEDPRCK | | | | | Mascot |
| 2175.0896 | 2175.1472 | 0.0576 | 26 | 1250 | 1270 | LGLSNCGLTDEGCAALAL ALR | | | Carbamidomethyl (C)[6,13] | | Mascot |
| 2175.0896 | 2175.1472 | 0.0576 | 26 | 1250 | 1270 | LGLSNCGLTDEGCAALAL ALR | | | Carbamidomethyl (C)[6,13] | | Mascot |
| 2225.2839 | 2225.126 | -0.1579 | -71 | 890 | 908 | FLLGLSVESHQILLQRLM K | | | | | Mascot |
| 2284.0181 | 2284.1846 | 0.1665 | 73 | 380 | 400 | SDLSSVHQKSTESESSC VSMR | | | | | Mascot |
| 2341.0396 | 2341.1333 | 0.0937 | 40 | 380 | 400 | SDLSSVHQKSTESESSC VSMR | | | Carbamidomethyl (C)[17] | | Mascot |
| 2357.0344 | 2357.1387 | 0.1043 | 44 | 380 | 400 | SDLSSVHQKSTESESSC VSMR | | | Carbamidomethyl (C)[17], Oxidation (M)[20] | | Mascot |
| 2448.2002 | 2448.1497 | -0.0505 | -21 | 414 | 435 | SGDTETDLSHEALNTFRS NLLK | | | | | Mascot |
| 2448.2002 | 2448.1497 | -0.0505 | -21 | 414 | 435 | SGDTETDLSHEALNTFRS NLLK | | | | | Mascot |
| 2459.2744 | 2459.1106 | -0.1638 | -67 | 1247 | 1270 | LERLGLSNCGLTDEGCA ALALALR | | | | | Mascot |
| 2479.0752 | 2479.3013 | 0.2261 | 91 | 277 | 297 | KDFCSADQEELLETLDNY SCR | | | | | Mascot |
| 3338.6072 | 3338.7676 | 0.1604 | 48 | 780 | 809 | ECGIDVAEALVYSGLCTQ IFREEFGLYQGK | | | | | Mascot |

| | | | | | | | |
|---|---|---|---|---|---|---|---|
| 5 | vitellogenin 2 isoform 2 [Danio rerio] | gi\|160333705 | 93050.4 | 8.22 | 12 | 35 | 0 |

Peptide Information

| Calc. Mass | Obsrv. Mass | ± da | ± ppm | Start Seq. | End Seq. | Sequence | Ion Score | C. I. % | Modification | Rank | Result Type |
|---|---|---|---|---|---|---|---|---|---|---|---|
| 850.4702 | 850.5433 | 0.0731 | 86 | 750 | 756 | MKLESVK | | | Oxidation (M)[1] | | Mascot |
| 864.3945 | 864.4503 | 0.0558 | 65 | 612 | 618 | KDEESEK | | | | | Mascot |
| 1112.5769 | 1112.5112 | -0.0657 | -59 | 322 | 330 | TLTKMEAFR | | | Oxidation (M)[5] | | Mascot |
| 1909.1117 | 1908.9408 | -0.1709 | -90 | 251 | 269 | TTASALPIGVQVDAILALR | | | | | Mascot |
| 1951.9187 | 1951.9611 | 0.0424 | 22 | 692 | 708 | IQVADWMKGQTCGLCGK | | | Carbamidomethyl (C)[12,15] | | Mascot |
| 2233.2446 | 2233.0996 | -0.145 | -65 | 171 | 191 | IATIPALREVVMLGYGSMI AK | | | | | Mascot |
| 2294.3706 | 2294.1719 | -0.1987 | -87 | 278 | 297 | LVQPVALQLVLERALHPE LR | | | | | Mascot |
| 2294.3706 | 2294.1719 | -0.1987 | -87 | 278 | 297 | LVQPVALQLVLERALHPE LR | | | | | Mascot |
| 2298.1655 | 2298.1902 | 0.0247 | 11 | 298 | 318 | MVACILLFETKPSVAAMS SSR | | | Carbamidomethyl (C)[4] | | Mascot |
| 2457.3235 | 2457.3203 | -0.0032 | -1 | 619 | 640 | THLNVKLVDIDIDLYTLGT DAK | | | | | Mascot |
| 2479.2974 | 2479.3013 | 0.0039 | 2 | 1 | 22 | MMEATQSLVFVEFEKAP VVPVK | | | | | Mascot |
| 3000.4878 | 3000.2488 | -0.239 | -80 | 586 | 611 | NEMPISCYQVLAQDCTS ELKFVALLK | | | Carbamidomethyl (C)[7] | | Mascot |
| 3891.77 | 3891.8274 | 0.0574 | 15 | 715 | 749 | QEYTTPSGYLTESSVSFA | | | | | Mascot |

HSWVLPAESCRNASQCR

| 6 | protein BTG3 [Danio rerio] | gi\|55925203 | 18115.1 | 5.85 | 6 | 34 | 0 |
|---|---|---|---|---|---|---|---|

Peptide Information

| Calc. Mass | Obsrv. Mass | ± da | ± ppm | Start Seq. | End Seq. | Sequence | Ion Score | C. I. % | Modification | Rank | Result Type |
|---|---|---|---|---|---|---|---|---|---|---|---|
| 901.5353 | 901.5096 | -0.0257 | -29 | 34 | 41 | LTVALQEK | | | | | Mascot |
| 1112.504 | 1112.5112 | 0.0072 | 6 | 75 | 84 | ACAESGVQYK | | | Carbamidomethyl (C)[2] | | Mascot |
| 2211.0208 | 2211.1123 | 0.0915 | 41 | 91 | 108 | ELTLWVDPGEVCCRYGE K | | | Carbamidomethyl (C)[12,13] | | Mascot |
| 2224.9412 | 2225.126 | 0.1848 | 83 | 139 | 158 | VTSDYHSGSSSDEDSML VHR | | | Oxidation (M)[16] | | Mascot |
| 2357.1628 | 2357.1387 | -0.0241 | -10 | 85 | 104 | DLGLPKELTLWVDPGEV CCR | | | Carbamidomethyl (C)[18,19] | | Mascot |
| 2457.0688 | 2457.3203 | 0.2515 | 102 | 109 | 130 | NHGFTVATFSSEDDDDK EDVTK | | | | | Mascot |

| 7 | Ighmbp2l protein [Danio rerio] | gi\|215539488 | 110340.2 | 9 | 13 | 34 | 0 |
|---|---|---|---|---|---|---|---|

Peptide Information

| Calc. Mass | Obsrv. Mass | ± da | ± ppm | Start Seq. | End Seq. | Sequence | Ion Score | C. I. % | Modification | Rank | Result Type |
|---|---|---|---|---|---|---|---|---|---|---|---|
| 882.4389 | 882.5708 | 0.1319 | 149 | 393 | 400 | CILAGDYK | | | | | Mascot |
| 901.5464 | 901.5096 | -0.0368 | -41 | 850 | 857 | SQSVKKPK | | | | | Mascot |
| 996.4818 | 996.6025 | 0.1207 | 121 | 893 | 901 | AESVCGFVK | | | Carbamidomethyl (C)[5] | | Mascot |
| 1210.5773 | 1210.6035 | 0.0262 | 22 | 297 | 306 | DMDKAFNEIK | | | | | Mascot |
| 1436.7743 | 1436.7466 | -0.0277 | -19 | 944 | 957 | ISKEGVLYAGSGQK | | | | | Mascot |
| 1767.8879 | 1767.8188 | -0.0691 | -39 | 904 | 918 | ASVLTLGQLCIYCNR | | | Carbamidomethyl (C)[10,13] | | Mascot |
| 1901.9425 | 1901.9963 | 0.0538 | 28 | 129 | 144 | DGLYNLMKLANDVTYR | | | Oxidation (M)[7] | | Mascot |
| 1915.9364 | 1915.9642 | 0.0278 | 15 | 236 | 252 | VLCCAPSNVAVDNLVER | | | Carbamidomethyl (C)[3,4] | | Mascot |
| 2114.1096 | 2114.1943 | 0.0847 | 40 | 236 | 255 | VLCCAPSNVAVDNLVERL AK | | | | | Mascot |
| 2114.1096 | 2114.1943 | 0.0847 | 40 | 236 | 255 | VLCCAPSNVAVDNLVERL AK | | | | | Mascot |
| 2136.051 | 2136.1777 | 0.1267 | 59 | 41 | 59 | GVCLMKLQIGSQHTGMY GR | | | Carbamidomethyl (C)[3] | | Mascot |
| 2489.3721 | 2489.0869 | -0.2852 | -115 | 508 | 531 | GNQGEVDIVALHIKALTE AGVQVK | | | | | Mascot |
| 2515.167 | 2515.2476 | 0.0806 | 32 | 631 | 651 | HGELRTAFEYLEDCVAQ NYTR | | | | | Mascot |
| 3338.6208 | 3338.7676 | 0.1468 | 44 | 868 | 901 | AGATDIAAAANGDDDFD ALINAVVKAESVCGFVK | | | | | Mascot |

| 8 | hypothetical protein LOC799147 [Danio rerio] | gi\|130487208 | 15157.6 | 7.64 | 5 | 33 | 0 |
|---|---|---|---|---|---|---|---|

Peptide Information

| Calc. Mass | Obsrv. Mass | ± da | ± ppm | Start Seq. | End Seq. | Sequence | Ion Score | C. I. % | Modification | Rank | Result Type |
|---|---|---|---|---|---|---|---|---|---|---|---|

| Calc. Mass | Obsrv. Mass | ± da | ± ppm | Start Seq. | End Seq. | Sequence | Ion Score | C. I. % | Modification | Rank | Result Type |
|---|---|---|---|---|---|---|---|---|---|---|---|
| 842.477 | 842.5123 | 0.0353 | 42 | 124 | 130 | KGSLFYK | | | | | Mascot |
| 959.4879 | 959.5182 | 0.0303 | 32 | 1 | 8 | MAAFNHLR | | | | | Mascot |
| 2197.0991 | 2197.1292 | 0.0301 | 14 | 104 | 123 | LGTCTKTVEYGLSSVTCH LK | | | Carbamidomethyl (C)[4] | | Mascot |
| 2225.0081 | 2225.126 | 0.1179 | 53 | 68 | 86 | DNANPVWSAEFNFPNCK SK | | | Carbamidomethyl (C)[16] | | Mascot |
| 3000.645 | 3000.2488 | -0.3962 | -132 | 2 | 29 | AAFNHLRLVSLAVLMLAS QLDFASASVR | | | | | Mascot |

9 si:ch1073-126c3.2 [Danio rerio] gi|221307488 24754.2 6.97 6 32 0

**Peptide Information**

| Calc. Mass | Obsrv. Mass | ± da | ± ppm | Start Seq. | End Seq. | Sequence | Ion Score | C. I. % | Modification | Rank | Result Type |
|---|---|---|---|---|---|---|---|---|---|---|---|
| 864.4243 | 864.4503 | 0.026 | 30 | 1 | 8 | MESATIGR | | | | | Mascot |
| 1128.5242 | 1128.5063 | -0.0179 | -16 | 134 | 143 | LFEECSTATK | | | | | Mascot |
| 1951.9277 | 1951.9611 | 0.0334 | 17 | 204 | 221 | VETTESESTKLLCGNINN | | | | | Mascot |
| 2211.0295 | 2211.1123 | 0.0828 | 37 | 114 | 131 | IYCKPMCNAGYDFNFLRR | | | | | Mascot |
| 2284.0459 | 2284.1846 | 0.1387 | 61 | 114 | 131 | IYCKPMCNAGYDFNFLRR | | | Carbamidomethyl (C)[3], Oxidation (M)[6] | | Mascot |
| 2298.0503 | 2298.1902 | 0.1399 | 61 | 112 | 130 | DKIYCKPMCNAGYDFNFL R | | | | | Mascot |
| 2341.0674 | 2341.1333 | 0.0659 | 28 | 114 | 131 | IYCKPMCNAGYDFNFLRR | | | Carbamidomethyl (C)[3,7], Oxidation (M)[6] | | Mascot |
| 2448.2625 | 2448.1497 | -0.1128 | -46 | 89 | 111 | LLPENCPAPAVPENGGLL CLSVK | | | Carbamidomethyl (C)[6,19] | | Mascot |
| 2448.2625 | 2448.1497 | -0.1128 | -46 | 89 | 111 | LLPENCPAPAVPENGGLL CLSVK | | | Carbamidomethyl (C)[6,19] | | Mascot |

10 stromal interaction molecule 1b [Danio rerio] gi|321400099 72493.8 6.03 10 32 0

**Peptide Information**

| Calc. Mass | Obsrv. Mass | ± da | ± ppm | Start Seq. | End Seq. | Sequence | Ion Score | C. I. % | Modification | Rank | Result Type |
|---|---|---|---|---|---|---|---|---|---|---|---|
| 842.4076 | 842.5123 | 0.1047 | 124 | 555 | 561 | KSFGMEK | | | Oxidation (M)[5] | | Mascot |
| 901.4849 | 901.5096 | 0.0247 | 27 | 289 | 295 | EEAQRLR | | | | | Mascot |
| 959.4801 | 959.5182 | 0.0381 | 40 | 233 | 240 | DHMGKMIK | | | | | Mascot |
| 1051.5643 | 1051.7158 | 0.1515 | 144 | 175 | 183 | IPDRSHAQK | | | | | Mascot |
| 1128.6735 | 1128.5063 | -0.1672 | -148 | 408 | 418 | KALGEVTAALR | | | | | Mascot |
| 1751.942 | 1751.8234 | -0.1186 | -68 | 9 | 21 | HFIYITWLGFLWR | | | | | Mascot |
| 2196.9487 | 2197.1292 | 0.1805 | 82 | 608 | 629 | SLVDEDNGSTGEDTEGS GSGRK | | | | | Mascot |
| 2224.9548 | 2225.126 | 0.1712 | 77 | 607 | 628 | RSLVDEDNGSTGEDTEG SGSGR | | | | | Mascot |
| 2341.011 | 2341.1333 | 0.1223 | 52 | 508 | 528 | DLNRSESDSSLSMSQYG DGHR | | | | | Mascot |
| 2357.0059 | 2357.1387 | 0.1328 | 56 | 508 | 528 | DLNRSESDSSLSMSQYG DGHR | | | Oxidation (M)[13] | | Mascot |
| 3000.5586 | 3000.2488 | -0.3098 | -103 | 207 | 232 | DLMLVVSIIIGVGGCWFA | | | | | Mascot |

YIQNRNYR

| 11 | novel protein [Danio rerio] | gi|148725299 | 82126.8 | 6.15 | 11 | 31 | 0 |
|---|---|---|---|---|---|---|---|

Peptide Information

| Calc. Mass | Obsrv. Mass | ± da | ± ppm | Start Seq. | End Seq. | Sequence | Ion Score | C. I. % | Modification | Rank | Result Type |
|---|---|---|---|---|---|---|---|---|---|---|---|
| 901.4737 | 901.5096 | 0.0359 | 40 | 75 | 81 | ILQEENR | | | | | Mascot |
| 959.4614 | 959.5182 | 0.0568 | 59 | 347 | 354 | IPADCIDR | | | Carbamidomethyl (C)[5] | | Mascot |
| 1045.5272 | 1045.5662 | 0.039 | 37 | 600 | 608 | GLLTQQDDR | | | | | Mascot |
| 1210.6791 | 1210.6035 | -0.0756 | -62 | 382 | 391 | EIIDHIKQSK | | | | | Mascot |
| 1436.7631 | 1436.7466 | -0.0165 | -11 | 609 | 620 | DQSKEEIIAYIK | | | | | Mascot |
| 2114.0876 | 2114.1943 | 0.1067 | 50 | 566 | 584 | AAVDKALESENGHLDLYL R | | | | | Mascot |
| 2114.0876 | 2114.1943 | 0.1067 | 50 | 566 | 584 | AAVDKALESENGHLDLYL R | | | | | Mascot |
| 2136.072 | 2136.1777 | 0.1057 | 49 | 43 | 60 | HKSFTDNLQSIFQNLESK | | | | | Mascot |
| 2211.2092 | 2211.1123 | -0.0969 | -44 | 93 | 111 | SIITEAALDLTLFFLREMK | | | | | Mascot |
| 2294.0864 | 2294.1719 | 0.0855 | 37 | 491 | 511 | DCGIDVYKASVYSGMCT QILK | | | | | Mascot |
| 2294.0864 | 2294.1719 | 0.0855 | 37 | 491 | 511 | DCGIDVYKASVYSGMCT QILK | | | | | Mascot |
| 2298.1812 | 2298.1902 | 0.009 | 4 | 431 | 448 | TLTQMYTHFLRFQIQQSR | | | | | Mascot |
| 2341.2188 | 2341.1333 | -0.0855 | -37 | 472 | 490 | LAFQQLERNNLIFYESDL K | | | | | Mascot |

| 12 | ryanodine receptor 1 [Danio rerio] | gi|156523287 | 574386.1 | 5.03 | 30 | 31 | 0 |
|---|---|---|---|---|---|---|---|

Peptide Information

| Calc. Mass | Obsrv. Mass | ± da | ± ppm | Start Seq. | End Seq. | Sequence | Ion Score | C. I. % | Modification | Rank | Result Type |
|---|---|---|---|---|---|---|---|---|---|---|---|
| 842.4155 | 842.5123 | 0.0968 | 115 | 313 | 319 | QSAFSFR | | | | | Mascot |
| 864.4495 | 864.4503 | 0.0008 | 1 | 4197 | 4204 | IEIMGASK | | | Oxidation (M)[4] | | Mascot |
| 901.4962 | 901.5096 | 0.0134 | 15 | 2264 | 2270 | ISRQNQR | | | | | Mascot |
| 1045.5425 | 1045.5662 | 0.0237 | 23 | 2665 | 2672 | LLTNHYER | | | | | Mascot |
| 1051.5605 | 1051.7158 | 0.1553 | 148 | 2522 | 2530 | ASMVLFLDR | | | | | Mascot |
| 1076.5106 | 1076.4761 | -0.0345 | -32 | 1861 | 1869 | EEGEEVKEK | | | | | Mascot |
| 1112.5768 | 1112.5112 | -0.0656 | -59 | 3966 | 3975 | NFSKAMTVAK | | | Oxidation (M)[6] | | Mascot |
| 1128.5432 | 1128.5063 | -0.0369 | -33 | 4766 | 4774 | YGDIYGRER | | | | | Mascot |
| 1354.6606 | 1354.7754 | 0.1148 | 85 | 2458 | 2469 | CAPEMHLIQAGK | | | Carbamidomethyl (C)[1] | | Mascot |
| 1354.6606 | 1354.7754 | 0.1148 | 85 | 2458 | 2469 | CAPEMHLIQAGK | | | Carbamidomethyl (C)[1] | | Mascot |
| 1436.5884 | 1436.7466 | 0.1582 | 110 | 3500 | 3514 | AGDGEGGGSDDDRTK | | | | | Mascot |
| 1488.7904 | 1488.7906 | 0.0002 | 0 | 164 | 177 | VGDDLILVSVSSER | 7 | 0 | | | Mascot |

| | | | | | | | | | | | |
|---|---|---|---|---|---|---|---|---|---|---|---|
| 1488.7904 | 1488.7906 | 0.0002 | 0 | 164 | 177 | VGDDLILVSVSSER | | | | | Mascot |
| 1542.8573 | 1542.8898 | 0.0325 | 21 | 2635 | 2646 | YLRPSMLQHLLR | | | Oxidation (M)[6] | | Mascot |
| 1719.8768 | 1719.859 | -0.0178 | -10 | 2620 | 2634 | AQRDVIEDCLMSLVK | | | | | Mascot |
| 1719.8768 | 1719.859 | -0.0178 | -10 | 2620 | 2634 | AQRDVIEDCLMSLVK | | | | | Mascot |
| 1751.7554 | 1751.8234 | 0.068 | 39 | 2424 | 2437 | RFMFGGEDQHEENR | | | | | Mascot |
| 1767.7504 | 1767.8188 | 0.0684 | 39 | 2424 | 2437 | RFMFGGEDQHEENR | | | Oxidation (M)[3] | | Mascot |
| 1893.8977 | 1893.9885 | 0.0908 | 48 | 2793 | 2808 | IQNNWTYGEVLDENAK | | | | | Mascot |
| 1893.8977 | 1893.9885 | 0.0908 | 48 | 2793 | 2808 | IQNNWTYGEVLDENAK | | | | | Mascot |
| 1923.9932 | 1923.9712 | -0.022 | -11 | 3649 | 3664 | AVVACFRMTPLYNIPR | | | Carbamidomethyl (C)[5], Oxidation (M)[8] | | Mascot |
| 1952.0092 | 1951.9611 | -0.0481 | -25 | 2598 | 2613 | AIMIDSMLHTIYRLSR | | | Oxidation (M)[3,7] | | Mascot |
| 1999.8783 | 1999.9485 | 0.0702 | 35 | 379 | 395 | VMLHQEGHMDDALTCSR | | | Carbamidomethyl (C)[15] | | Mascot |
| 1999.8783 | 1999.9485 | 0.0702 | 35 | 379 | 395 | VMLHQEGHMDDALTCSR | | | Carbamidomethyl (C)[15] | | Mascot |
| 2136.0793 | 2136.1777 | 0.0984 | 46 | 958 | 976 | TYVMSSGYKPAPLDLNH VK | | | Oxidation (M)[4] | | Mascot |
| 2156.873 | 2157.1394 | 0.2664 | 124 | 4915 | 4931 | CDDMMTCYLFHMYVGVR | | | Carbamidomethyl (C)[1], Oxidation (M)[4] | | Mascot |
| 2175.1743 | 2175.1472 | -0.0271 | -12 | 2140 | 2157 | LMFSLLHRQYDALGELIR | | | | | Mascot |
| 2175.1743 | 2175.1472 | -0.0271 | -12 | 2140 | 2157 | LMFSLLHRQYDALGELIR | | | | | Mascot |
| 2283.2205 | 2283.1875 | -0.033 | -14 | 3415 | 3433 | DEFAVLCRDLYALYPLLIR | | | | | Mascot |
| 2432.1206 | 2432.1443 | 0.0237 | 10 | 1938 | 1958 | EEEEEAPEEGLLQMKMP ESVK | | | | | Mascot |
| 2432.1206 | 2432.1443 | 0.0237 | 10 | 1938 | 1958 | EEEEEAPEEGLLQMKMP ESVK | | | | | Mascot |
| 2442.3213 | 2442.083 | -0.2383 | -98 | 1292 | 1313 | LSMPVEFHLTFKVPAGTT PLTR | | | | | Mascot |
| 2448.1155 | 2448.1497 | 0.0342 | 14 | 1938 | 1958 | EEEEEAPEEGLLQMKMP ESVK | | | Oxidation (M)[14] | | Mascot |
| 2448.1155 | 2448.1497 | 0.0342 | 14 | 1938 | 1958 | EEEEEAPEEGLLQMKMP ESVK | | | Oxidation (M)[14] | | Mascot |
| 2459.1086 | 2459.1106 | 0.002 | 1 | 1959 | 1976 | LQMCTLLQYFCDCELRH R | | | Carbamidomethyl (C)[4,11,13], Oxidation (M)[3] | | Mascot |
| 2489.3042 | 2489.0869 | -0.2173 | -87 | 2575 | 2597 | YLCSAVLPLLTKCAPLFA GTDHR | | | | | Mascot |
| 2531.3113 | 2531.2427 | -0.0686 | -27 | 977 | 998 | LTPNQNQLVEKLAENGH NVWAR | | | | | Mascot |
| 3103.3625 | 3103.6494 | 0.2869 | 92 | 379 | 405 | VMLHQEGHMDDALTCSR SQTEESQAAR | | | Carbamidomethyl (C)[15], Oxidation (M)[2] | | Mascot |
| 3287.8501 | 3287.4243 | -0.4258 | -130 | 2481 | 2511 | SLVPIEDLVGVISLPVQIP TFGKDGQIVEPK | | | | | Mascot |
| 3337.5386 | 3337.7583 | 0.2197 | 66 | 3311 | 3340 | GPENFPEQEGCLCTDVT SEQLNQLLGSIMK | | | Carbamidomethyl (C)[11] | | Mascot |

13 novel protein [Danio rerio] gi|220679564 9886.7 8.37 5 31 0

**Peptide Information**

| Calc. Mass | Obsrv. Mass | ± da | ± ppm | Start Seq. | End Seq. | Sequence | Ion Score | C. I. % | Modification | Rank | Result Type |
|---|---|---|---|---|---|---|---|---|---|---|---|
| 864.4218 | 864.4503 | 0.0285 | 33 | 33 | 38 | WLMVCR | | | Carbamidomethyl (C)[5] | | Mascot |

| Calc. Mass | Obsrv. Mass | ± da | ± ppm | Start Seq. | End Seq. | Sequence | Ion Score | C. I. % | Modification | Rank | Result Type |
|---|---|---|---|---|---|---|---|---|---|---|---|
| 1103.5632 | 1103.575 | 0.0118 | 11 | 70 | 78 | TAHANWYLK | | | | | Mascot |
| 1736.8207 | 1736.8944 | 0.0737 | 42 | 24 | 38 | SAVPGSSDRWLMVCR | | | Carbamidomethyl (C)[14], Oxidation (M)[12] | | Mascot |
| 2456.99 | 2457.3203 | 0.3303 | 134 | 2 | 23 | IPEYSMSSAASSDMCPY MPVCR | | | Oxidation (M)[6,14] | | Mascot |
| 3338.4255 | 3338.7676 | 0.3421 | 102 | 2 | 32 | IPEYSMSSAASSDMCPY MPVCRSAVPGSSDR | | | Carbamidomethyl (C)[15] | | Mascot |

| | | | | | | | | | | |
|---|---|---|---|---|---|---|---|---|---|---|
| 14 | PREDICTED: hypothetical protein LOC100535442 isoform 2 [Danio rerio] | gi\|326668097 | 11743 | 9.12 | 5 | 31 | 0 | | | |

**Peptide Information**

| Calc. Mass | Obsrv. Mass | ± da | ± ppm | Start Seq. | End Seq. | Sequence | Ion Score | C. I. % | Modification | Rank | Result Type |
|---|---|---|---|---|---|---|---|---|---|---|---|
| 901.5101 | 901.5096 | -0.0005 | -1 | 31 | 38 | TNDLLAVR | | | | | Mascot |
| 1128.6483 | 1128.5063 | -0.142 | -126 | 29 | 38 | ARTNDLLAVR | | | | | Mascot |
| 1436.7493 | 1436.7466 | -0.0027 | -2 | 18 | 30 | LHTETPTDPAKAR | | | | | Mascot |
| 1488.7589 | 1488.7906 | 0.0317 | 21 | 91 | 103 | LTNTCFVITFVCK | | | | | Mascot |
| 1488.7589 | 1488.7906 | 0.0317 | 21 | 91 | 103 | LTNTCFVITFVCK | | | | | Mascot |
| 1893.9601 | 1893.9885 | 0.0284 | 15 | 91 | 105 | LTNTCFVITFVCKYK | | | Carbamidomethyl (C)[5,12] | | Mascot |
| 1893.9601 | 1893.9885 | 0.0284 | 15 | 91 | 105 | LTNTCFVITFVCKYK | | | Carbamidomethyl (C)[5,12] | | Mascot |

| | | | | | | | | | | |
|---|---|---|---|---|---|---|---|---|---|---|
| 15 | novel protein (zgc:77021) [Danio rerio] | gi\|94732505 | 46122.4 | 8.52 | 7 | 31 | 0 | 9 | 0 | |

**Peptide Information**

| Calc. Mass | Obsrv. Mass | ± da | ± ppm | Start Seq. | End Seq. | Sequence | Ion Score | C. I. % | Modification | Rank | Result Type |
|---|---|---|---|---|---|---|---|---|---|---|---|
| 882.4753 | 882.5708 | 0.0955 | 108 | 107 | 114 | GYMLSIAK | | | | | Mascot |
| 901.5141 | 901.5096 | -0.0045 | -5 | 365 | 373 | ALVPPGYGK | | | | | Mascot |
| 1488.7839 | 1488.7906 | 0.0067 | 5 | 374 | 386 | KPLRSNSLSDMPK | 9 | 0 | Oxidation (M)[11] | | Mascot |
| 1488.7839 | 1488.7906 | 0.0067 | 5 | 374 | 386 | KPLRSNSLSDMPK | | | Oxidation (M)[11] | | Mascot |
| 1901.9326 | 1901.9963 | 0.0637 | 33 | 10 | 25 | GRAQLPPCYHEGFLEK | | | Carbamidomethyl (C)[8] | | Mascot |
| 1951.9258 | 1951.9611 | 0.0353 | 18 | 36 | 52 | LWTSLCGDSLFFFNSSK | | | | | Mascot |
| 2459.0239 | 2459.1106 | 0.0867 | 35 | 344 | 363 | HLPYQYSMPQETNTSSD CER | | | Carbamidomethyl (C)[18], Oxidation (M)[8] | | Mascot |
| 2515.4316 | 2515.2476 | -0.184 | -73 | 115 | 137 | LSVPVSLNLLPGQIHAMK EVIEK | | | | | Mascot |
| 2531.4265 | 2531.2427 | -0.1838 | -73 | 115 | 137 | LSVPVSLNLLPGQIHAMK EVIEK | | | Oxidation (M)[17] | | Mascot |

| | | | | | | | | | | |
|---|---|---|---|---|---|---|---|---|---|---|
| 16 | RecName: Full=Eukaryotic translation initiation factor 3 subunit D; Short=eIF3d; AltName: Full=Euka | gi\|82187444 | 64049.5 | 6.06 | 9 | 30 | 0 | | | |

**Peptide Information**

| Calc. Mass | Obsrv. Mass | ± da | ± ppm | Start Seq. | End Seq. | Sequence | Ion Score | C. I. % | Modification | Rank | Result Type |
|---|---|---|---|---|---|---|---|---|---|---|---|

| | | | | | | | | | | | |
|---|---|---|---|---|---|---|---|---|---|---|---|
| 901.5352 | 901.5096 | -0.0256 | -28 | 421 | 429 | GAVLATELK | | | | | Mascot |
| 1103.6459 | 1103.575 | -0.0709 | -64 | 509 | 517 | YLILKDPNK | | | | | Mascot |
| 1112.5082 | 1112.5112 | 0.003 | 3 | 27 | 35 | DMPYQPFSK | | | | | Mascot |
| 1128.5031 | 1128.5063 | 0.0032 | 3 | 27 | 35 | DMPYQPFSK | | | Oxidation (M)[2] | | Mascot |
| 1354.6273 | 1354.7754 | 0.1481 | 109 | 42 | 53 | VADWTGATYQDK | | | | | Mascot |
| 1354.6273 | 1354.7754 | 0.1481 | 109 | 42 | 53 | VADWTGATYQDK | | | | | Mascot |
| 2233.1143 | 2233.0996 | -0.0147 | -7 | 438 | 457 | WTCCAILAGSEYLKLGYV SR | | | | | Mascot |
| 2400.0193 | 2400.1211 | 0.1018 | 42 | 187 | 207 | YMDVADPLDIECCGALEH YDK | | | | | Mascot |
| 2448.2129 | 2448.1497 | -0.0632 | -26 | 2 | 24 | AKFHAPVIQDNPSGWGP CAVPEK | | | | | Mascot |
| 2448.2129 | 2448.1497 | -0.0632 | -26 | 2 | 24 | AKFHAPVIQDNPSGWGP CAVPEK | | | | | Mascot |
| 2457.2297 | 2457.3203 | 0.0906 | 37 | 241 | 263 | LAKTQGNVFATDAILATL MCCTR | | | Oxidation (M)[19] | | Mascot |
| 3287.6033 | 3287.4243 | -0.179 | -54 | 368 | 397 | LGEDIDLIVRCEHDGVMT GANGEVSFINVK | | | Carbamidomethyl (C)[11] | | Mascot |
| 3303.5984 | 3303.4224 | -0.176 | -53 | 368 | 397 | LGEDIDLIVRCEHDGVMT GANGEVSFINVK | | | Carbamidomethyl (C)[11], Oxidation (M)[17] | | Mascot |

17 novel NACHT domain containing protein [Danio rerio] gi|169158295 75440.9 5.59 10 30 0

Peptide Information

| Calc. Mass | Obsrv. Mass | ± da | ± ppm | Start Seq. | End Seq. | Sequence | Ion Score | C. I. % | Modification | Rank | Result Type |
|---|---|---|---|---|---|---|---|---|---|---|---|
| 901.4737 | 901.5096 | 0.0359 | 40 | 48 | 54 | ILQEENR | | | | | Mascot |
| 959.4614 | 959.5182 | 0.0568 | 59 | 320 | 327 | IPADCIDR | | | Carbamidomethyl (C)[5] | | Mascot |
| 1045.5272 | 1045.5662 | 0.039 | 37 | 570 | 578 | GLLTQQDDR | | | | | Mascot |
| 1210.6062 | 1210.6035 | -0.0027 | -2 | 393 | 403 | AHAETLQESPK | | | | | Mascot |
| 1436.7631 | 1436.7466 | -0.0165 | -11 | 579 | 590 | DQSKEEIIAYIK | | | | | Mascot |
| 2136.072 | 2136.1777 | 0.1057 | 49 | 16 | 33 | HKSFTDNLQSIFQNLESK | | | | | Mascot |
| 2211.2092 | 2211.1123 | -0.0969 | -44 | 66 | 84 | SIITEAALDLTLFFLREMK | | | | | Mascot |
| 2298.1812 | 2298.1902 | 0.009 | 4 | 404 | 421 | TLTQMYTHFLRFQIQQSR | | | | | Mascot |
| 2442.1138 | 2442.083 | -0.0308 | -13 | 464 | 484 | DCGIDVYKASVYSGMCT QIFK | | | Carbamidomethyl (C)[2,16] | | Mascot |
| 3908.0125 | 3907.8484 | -0.1641 | -42 | 485 | 517 | EETGIILGTMYCFLHLSIQ EFIAALYQHLILDR | | | Carbamidomethyl (C)[12] | | Mascot |

18 PREDICTED: diacylglycerol kinase beta-like, partial [Danio rerio] gi|326674321 85995.9 6.78 11 30 0

Peptide Information

| Calc. Mass | Obsrv. Mass | ± da | ± ppm | Start Seq. | End Seq. | Sequence | Ion Score | C. I. % | Modification | Rank | Result Type |
|---|---|---|---|---|---|---|---|---|---|---|---|
| 842.4229 | 842.5123 | 0.0894 | 106 | 153 | 158 | LEFMFR | | | | | Mascot |
| 864.4032 | 864.4503 | 0.0471 | 54 | 455 | 462 | SGPMPGYR | | | | | Mascot |

| | | | | | | | | | | | |
|---|---|---|---|---|---|---|---|---|---|---|---|
| 901.5353 | 901.5096 | -0.0257 | -29 | 678 | 685 | KTTIVDPK | | | | | Mascot |
| 1045.4264 | 1045.5662 | 0.1398 | 134 | 270 | 278 | QGLCCSFCK | | | Carbamidomethyl (C)[4] | | Mascot |
| 1112.6497 | 1112.5112 | -0.1385 | -124 | 112 | 121 | LIKGSCPPLK | | | Carbamidomethyl (C)[6] | | Mascot |
| 1210.5455 | 1210.6035 | 0.058 | 48 | 455 | 465 | SGPMPGYRSCR | | | | | Mascot |
| 2157.0005 | 2157.1394 | 0.1389 | 64 | 302 | 319 | KNTEIMHHFWVEGNCPSK | | | | | Mascot |
| 2283.2488 | 2283.1875 | -0.0613 | -27 | 115 | 135 | GSCPPLKVTSQSRPLSTVQLK | | | Carbamidomethyl (C)[3] | | Mascot |
| 2284.0591 | 2284.1846 | 0.1255 | 55 | 153 | 171 | LEFMFRLYDTDGNGFLDSK | | | Oxidation (M)[4] | | Mascot |
| 2432.0581 | 2432.1443 | 0.0862 | 35 | 303 | 322 | NTEIMHHFWVEGNCPSKCDK | | | Carbamidomethyl (C)[14] | | Mascot |
| 2432.0581 | 2432.1443 | 0.0862 | 35 | 303 | 322 | NTEIMHHFWVEGNCPSKCDK | | | Carbamidomethyl (C)[14] | | Mascot |
| 2448.053 | 2448.1497 | 0.0967 | 40 | 303 | 322 | NTEIMHHFWVEGNCPSKCDK | | | Carbamidomethyl (C)[14], Oxidation (M)[5] | | Mascot |
| 2448.053 | 2448.1497 | 0.0967 | 40 | 303 | 322 | NTEIMHHFWVEGNCPSKCDK | | | Carbamidomethyl (C)[14], Oxidation (M)[5] | | Mascot |
| 2479.3022 | 2479.3013 | -0.0009 | 0 | 248 | 269 | LKHFSKPAYCNLCLNMLIGLGK | | | Oxidation (M)[16] | | Mascot |
| 2489.0796 | 2489.0869 | 0.0073 | 3 | 303 | 322 | NTEIMHHFWVEGNCPSKCDK | | | Carbamidomethyl (C)[14,18] | | Mascot |

| | | | | | | | |
|---|---|---|---|---|---|---|---|
| 19 | LOC565764 protein [Danio rerio] | gi\|112418812 | 25782.2 | 5.2 | 6 | 30 | 0 |

**Peptide Information**

| Calc. Mass | Obsrv. Mass | ± da | ± ppm | Start Seq. | End Seq. | Sequence | Ion Score | C. I. % | Modification | Rank | Result Type |
|---|---|---|---|---|---|---|---|---|---|---|---|
| 864.4355 | 864.4503 | 0.0148 | 17 | 77 | 83 | RSMDSIR | | | | | Mascot |
| 882.4389 | 882.5708 | 0.1319 | 149 | 11 | 17 | LEAMFQK | | | Oxidation (M)[4] | | Mascot |
| 901.4988 | 901.5096 | 0.0108 | 12 | 222 | 230 | GLVSLLAND | | | | | Mascot |
| 1112.5293 | 1112.5112 | -0.0181 | -16 | 2 | 10 | LFNMETVDK | | | Oxidation (M)[4] | | Mascot |
| 1767.8694 | 1767.8188 | -0.0506 | -29 | 61 | 76 | HAALCAQVEEIAAEQK | | | Carbamidomethyl (C)[5] | | Mascot |
| 1923.9705 | 1923.9712 | 0.0007 | 0 | 61 | 77 | HAALCAQVEEIAAEQKR | | | Carbamidomethyl (C)[5] | | Mascot |

| | | | | | | | |
|---|---|---|---|---|---|---|---|
| 20 | PREDICTED: HEAT repeat-containing protein 1-like [Danio rerio] | gi\|189525800 | 241986.3 | 5.96 | 20 | 30 | 0 |

**Peptide Information**

| Calc. Mass | Obsrv. Mass | ± da | ± ppm | Start Seq. | End Seq. | Sequence | Ion Score | C. I. % | Modification | Rank | Result Type |
|---|---|---|---|---|---|---|---|---|---|---|---|
| 842.557 | 842.5123 | -0.0447 | -53 | 1792 | 1798 | LTLLARR | | | | | Mascot |
| 901.5101 | 901.5096 | -0.0005 | -1 | 1942 | 1949 | LADRIADK | | | | | Mascot |
| 959.4363 | 959.5182 | 0.0819 | 85 | 209 | 216 | DGNCPQLR | | | Carbamidomethyl (C)[4] | | Mascot |
| 996.4567 | 996.6025 | 0.1458 | 146 | 1853 | 1860 | EHISHMDK | | | | | Mascot |
| 1076.5768 | 1076.4761 | -0.1007 | -94 | 1618 | 1626 | KAMELLNNK | | | Oxidation (M)[3] | | Mascot |

| Calc. Mass | Obsrv. Mass | ± da | ± ppm | Start Seq. | End Seq. | Sequence | Ion Score | C. I. % | Modification | Rank | Result Type |
|---|---|---|---|---|---|---|---|---|---|---|---|
| 1103.5878 | 1103.575 | -0.0128 | -12 | 1799 | 1808 | LTSCPQLSVR | | | | | Mascot |
| 1128.5684 | 1128.5063 | -0.0621 | -55 | 977 | 985 | FYEEAVSRK | | | | | Mascot |
| 1736.9073 | 1736.8944 | -0.0129 | -7 | 1591 | 1605 | VNALLPMDTFITVMR | | | Oxidation (M)[7] | | Mascot |
| 1908.9768 | 1908.9408 | -0.036 | -19 | 1712 | 1729 | NVMGSALLCVAEVTSTLK | | | Carbamidomethyl (C)[9], Oxidation (M)[3] | | Mascot |
| 2114.1362 | 2114.1943 | 0.0581 | 27 | 2054 | 2072 | SRITTHLVPCIAQFAVAMR | | | | | Mascot |
| 2114.1362 | 2114.1943 | 0.0581 | 27 | 2054 | 2072 | SRITTHLVPCIAQFAVAMR | | | | | Mascot |
| 2136.1104 | 2136.1777 | 0.0673 | 32 | 647 | 666 | TSESDLLGVANEMLTTTLIK | | | | | Mascot |
| 2225.0286 | 2225.126 | 0.0974 | 44 | 254 | 273 | SNLSDYTAATYMIVCQMAVK | | | Oxidation (M)[12] | | Mascot |
| 2233.2219 | 2233.0996 | -0.1223 | -55 | 1799 | 1819 | LTSCPQLSVRLASLSSTLATK | | | Carbamidomethyl (C)[4] | | Mascot |
| 2283.1294 | 2283.1875 | 0.0581 | 25 | 1824 | 1843 | VLIPTITKCYCSMVDAQQNR | | | | | Mascot |
| 2298.0452 | 2298.1902 | 0.145 | 63 | 254 | 273 | SNLSDYTAATYMIVCQMAVK | | | Carbamidomethyl (C)[15], Oxidation (M)[12,17] | | Mascot |
| 2442.2043 | 2442.083 | -0.1213 | -50 | 991 | 1012 | LASVEQLLQCLQSPFCPSYTSK | | | | | Mascot |
| 2448.386 | 2448.1497 | -0.2363 | -97 | 881 | 903 | VSAILQTQALYVGKAFLSSQPVK | | | | | Mascot |
| 2448.386 | 2448.1497 | -0.2363 | -97 | 881 | 903 | VSAILQTQALYVGKAFLSSQPVK | | | | | Mascot |
| 2459.2791 | 2459.1106 | -0.1685 | -69 | 1510 | 1531 | HFKFISISFMAQLLASDGFVGK | | | Oxidation (M)[10] | | Mascot |
| 2531.1873 | 2531.2427 | 0.0554 | 22 | 1832 | 1852 | CYCSMVDAQQNRLSSLMNILK | | | Carbamidomethyl (C)[1,3] | | Mascot |
| 3000.5134 | 3000.2488 | -0.2646 | -88 | 209 | 235 | DGNCPQLRVIFSFYASTIVSALDAVEK | | | Carbamidomethyl (C)[4] | | Mascot |
| 3303.6599 | 3303.4224 | -0.2375 | -72 | 1136 | 1166 | SPACAQSINSVFKTIAVDCELVANELIPADK | | | Carbamidomethyl (C)[4] | | Mascot |

| | | | | | | | |
|---|---|---|---|---|---|---|---|
| 21 | PREDICTED: interferon-induced protein 44-like [Danio rerio] | gi\|189514429 | 38705.9 | 5.26 | 5 | 29 | 0 |

**Peptide Information**

| Calc. Mass | Obsrv. Mass | ± da | ± ppm | Start Seq. | End Seq. | Sequence | Ion Score | C. I. % | Modification | Rank | Result Type |
|---|---|---|---|---|---|---|---|---|---|---|---|
| 1045.6074 | 1045.5662 | -0.0412 | -39 | 214 | 223 | GIAQVVVMTK | | | | | Mascot |
| 1354.8052 | 1354.7754 | -0.0298 | -22 | 60 | 73 | DIKVLVAGQVGAGK | 5 | 0 | | | Mascot |
| 1354.8052 | 1354.7754 | -0.0298 | -22 | 60 | 73 | DIKVLVAGQVGAGK | | | | | Mascot |
| 1488.8455 | 1488.7906 | -0.0549 | -37 | 210 | 223 | ISDKGIAQVVVMTK | 6 | 0 | | | Mascot |
| 1488.8455 | 1488.7906 | -0.0549 | -37 | 210 | 223 | ISDKGIAQVVVMTK | | | | | Mascot |
| 2157.0234 | 2157.1394 | 0.116 | 54 | 308 | 325 | NYYNEIDTDDDVDVLILK | | | | | Mascot |
| 3319.6514 | 3319.4426 | -0.2088 | -63 | 126 | 156 | DIMGLEAEALLGSQTDDIINAVFGHVKDGYK | | | | | Mascot |

| | | | | | | | |
|---|---|---|---|---|---|---|---|
| 22 | PREDICTED: calcium/calmodulin-dependent 3',5'-cyclic nucleotide phosphodiesterase 1C-like, partial | gi\|326681376 | 45655.3 | 5.68 | 8 | 29 | 0 |

**Peptide Information**

| Calc. Mass | Obsrv. Mass | ± da | ± ppm | Start Seq. | End Seq. | Sequence | Ion Score | C. I. % | Modification | Rank | Result Type |
|---|---|---|---|---|---|---|---|---|---|---|---|
| 864.4429 | 864.4503 | 0.0074 | 9 | 104 | 110 | QMGLMLR | | | Oxidation (M)[2] | | Mascot |
| 901.5651 | 901.5096 | -0.0555 | -62 | 33 | 40 | GLLRCLVK | | | | | Mascot |
| 1045.5823 | 1045.5662 | -0.0161 | -15 | 37 | 44 | CLVKQLER | | | Carbamidomethyl (C)[1] | | Mascot |
| 1908.9185 | 1908.9408 | 0.0223 | 12 | 2 | 17 | ESPTREIEEFENTSLK | | | | | Mascot |
| 1923.9368 | 1923.9712 | 0.0344 | 18 | 299 | 314 | LLQDDDEMNILYNLSK | | | | | Mascot |
| 2283.2634 | 2283.1875 | -0.0759 | -33 | 193 | 213 | FKIPVSALVSFVEALEVG YSK | | | | | Mascot |
| 2459.2144 | 2459.1106 | -0.1038 | -42 | 374 | 392 | TWDLHHRWTTSLLEEFFL Q | | | | | Mascot |
| 2489.2002 | 2489.0869 | -0.1133 | -46 | 73 | 94 | RLVDTEDELSDIQSDSVP SEVR | | | | | Mascot |

23 eukaryotic translation initiation factor 3, subunit 7 zeta [Danio rerio] gi|123230120 63850.4 6.06 9 29 0

Peptide Information

| Calc. Mass | Obsrv. Mass | ± da | ± ppm | Start Seq. | End Seq. | Sequence | Ion Score | C. I. % | Modification | Rank | Result Type |
|---|---|---|---|---|---|---|---|---|---|---|---|
| 901.5352 | 901.5096 | -0.0256 | -28 | 419 | 427 | GAVLATELK | | | | | Mascot |
| 1103.6459 | 1103.575 | -0.0709 | -64 | 507 | 515 | YLILKDPNK | | | | | Mascot |
| 1112.5082 | 1112.5112 | 0.003 | 3 | 27 | 35 | DMPYQPFSK | | | | | Mascot |
| 1128.5031 | 1128.5063 | 0.0032 | 3 | 27 | 35 | DMPYQPFSK | | | Oxidation (M)[2] | | Mascot |
| 1354.6273 | 1354.7754 | 0.1481 | 109 | 42 | 53 | VADWTGATYQDK | | | | | Mascot |
| 1354.6273 | 1354.7754 | 0.1481 | 109 | 42 | 53 | VADWTGATYQDK | | | | | Mascot |
| 2233.1143 | 2233.0996 | -0.0147 | -7 | 436 | 455 | WTCCAILAGSEYLKLGYV SR | | | | | Mascot |
| 2400.0193 | 2400.1211 | 0.1018 | 42 | 185 | 205 | YMDVADPLDIECCGALEH YDK | | | | | Mascot |
| 2448.2129 | 2448.1497 | -0.0632 | -26 | 2 | 24 | AKFHAPVIQDNPSGWGP CAVPEK | | | | | Mascot |
| 2448.2129 | 2448.1497 | -0.0632 | -26 | 2 | 24 | AKFHAPVIQDNPSGWGP CAVPEK | | | | | Mascot |
| 2457.2297 | 2457.3203 | 0.0906 | 37 | 239 | 261 | LAKTQGNVFATDAILATL MCCTR | | | Oxidation (M)[19] | | Mascot |
| 3287.6033 | 3287.4243 | -0.179 | -54 | 366 | 395 | LGEDIDLIVRCEHDGVMT GANGEVSFINVK | | | Carbamidomethyl (C)[11] | | Mascot |
| 3303.5984 | 3303.4224 | -0.176 | -53 | 366 | 395 | LGEDIDLIVRCEHDGVMT GANGEVSFINVK | | | Carbamidomethyl (C)[11], Oxidation (M)[17] | | Mascot |

24 guanine nucleotide binding protein (G protein), beta polypeptide 1, like [Danio rerio] gi|47087315 37279 5.6 7 29 0

Peptide Information

| Calc. Mass | Obsrv. Mass | ± da | ± ppm | Start Seq. | End Seq. | Sequence | Ion Score | C. I. % | Modification | Rank | Result Type |
|---|---|---|---|---|---|---|---|---|---|---|---|

| Calc. Mass | Obsrv. Mass | ± da | ± ppm | Start Seq. | End Seq. | Sequence | Ion Score | C. I. % | Modification | Rank | Result Type |
|---|---|---|---|---|---|---|---|---|---|---|---|
| 1751.8315 | 1751.8234 | -0.0081 | -5 | 135 | 150 | VSRELAGHTGYLSCCR | | | | | Mascot |
| 1908.9636 | 1908.9408 | -0.0228 | -12 | 198 | 214 | LFVSGACDASAKLWDIR | | | Carbamidomethyl (C)[7] | | Mascot |
| 1915.9542 | 1915.9642 | 0.01 | 5 | 24 | 42 | ACADATLSQITANIDPVGR | | | | | Mascot |
| 2157.032 | 2157.1394 | 0.1074 | 50 | 284 | 301 | LLLAGYDDFNCNVWDTLK | | | Carbamidomethyl (C)[11] | | Mascot |
| 2400.1653 | 2400.1211 | -0.0442 | -18 | 281 | 301 | SGRLLLAGYDDFNCNVWDTLK | | | | | Mascot |
| 2432.1108 | 2432.1443 | 0.0335 | 14 | 315 | 337 | VSCLGVTDDGMAVATGSWDSFLK | | | Carbamidomethyl (C)[3], Oxidation (M)[11] | | Mascot |
| 2432.1108 | 2432.1443 | 0.0335 | 14 | 315 | 337 | VSCLGVTDDGMAVATGSWDSFLK | | | Carbamidomethyl (C)[3], Oxidation (M)[11] | | Mascot |
| 2442.1758 | 2442.083 | -0.0928 | -38 | 284 | 304 | LLLAGYDDFNCNVWDTLKADR | | | | | Mascot |
| 2457.1868 | 2457.3203 | 0.1335 | 54 | 281 | 301 | SGRLLLAGYDDFNCNVWDTLK | | | Carbamidomethyl (C)[14] | | Mascot |

| | | | | | | | |
|---|---|---|---|---|---|---|---|
| 25 | RecName: Full=Hematopoietic SH2 domain-containing protein homolog | gi\|82178338 | 41293.7 | 6.57 | 7 | 29 | 0 |

**Peptide Information**

| Calc. Mass | Obsrv. Mass | ± da | ± ppm | Start Seq. | End Seq. | Sequence | Ion Score | C. I. % | Modification | Rank | Result Type |
|---|---|---|---|---|---|---|---|---|---|---|---|
| 1354.671 | 1354.7754 | 0.1044 | 77 | 257 | 268 | QQITPNTPNEGR | | | | | Mascot |
| 1354.671 | 1354.7754 | 0.1044 | 77 | 257 | 268 | QQITPNTPNEGR | | | | | Mascot |
| 1719.8846 | 1719.859 | -0.0256 | -15 | 219 | 233 | TPAEETPPQLPPRMR | | | | | Mascot |
| 1719.8846 | 1719.859 | -0.0256 | -15 | 219 | 233 | TPAEETPPQLPPRMR | | | | | Mascot |
| 1999.9802 | 1999.9485 | -0.0317 | -16 | 43 | 59 | AAEEMLMCKPAGYFLIR | | | Carbamidomethyl (C)[8] | | Mascot |
| 1999.9802 | 1999.9485 | -0.0317 | -16 | 43 | 59 | AAEEMLMCKPAGYFLIR | | | Carbamidomethyl (C)[8] | | Mascot |
| 2284.1907 | 2284.1846 | -0.0061 | -3 | 23 | 42 | SCILENGVVPEWFHGIISRK | | | | | Mascot |
| 2341.2122 | 2341.1333 | -0.0789 | -34 | 23 | 42 | SCILENGVVPEWFHGIISRK | | | Carbamidomethyl (C)[2] | | Mascot |
| 2400.1255 | 2400.1211 | -0.0044 | -2 | 2 | 22 | AVPLPNDSSNAAFSWFTEYQR | | | | | Mascot |
| 2448.1985 | 2448.1497 | -0.0488 | -20 | 77 | 96 | CRHFMINVLPDNQFMIVGEK | | | Carbamidomethyl (C)[1] | | Mascot |
| 2448.1985 | 2448.1497 | -0.0488 | -20 | 77 | 96 | CRHFMINVLPDNQFMIVGEK | | | Carbamidomethyl (C)[1] | | Mascot |
| 2531.166 | 2531.2427 | 0.0767 | 30 | 1 | 22 | MAVPLPNDSSNAAFSWFTEYQR | | | | | Mascot |

| | | | | | | | |
|---|---|---|---|---|---|---|---|
| 26 | DNA-binding protein SMUBP-2 [Danio rerio] | gi\|120952590 | 110816.5 | 8.97 | 12 | 29 | 0 |

**Peptide Information**

| Calc. Mass | Obsrv. Mass | ± da | ± ppm | Start Seq. | End Seq. | Sequence | Ion Score | C. I. % | Modification | Rank | Result Type |
|---|---|---|---|---|---|---|---|---|---|---|---|
| 882.4389 | 882.5708 | 0.1319 | 149 | 393 | 400 | CILAGDYK | | | | | Mascot |
| 996.4818 | 996.6025 | 0.1207 | 121 | 897 | 905 | AESVCGFVK | | | Carbamidomethyl (C)[5] | | Mascot |
| 1210.5773 | 1210.6035 | 0.0262 | 22 | 297 | 306 | DMDKAFNEIK | | | | | Mascot |

| Calc. Mass | Obsrv. Mass | ± da | ± ppm | Start Seq. | End Seq. | Sequence | Ion Score | C. I. % | Modification | Rank | Result Type |
|---|---|---|---|---|---|---|---|---|---|---|---|
| 1436.7743 | 1436.7466 | -0.0277 | -19 | 948 | 961 | ISKEGVLYAGSGQK | | | | | Mascot |
| 1767.8879 | 1767.8188 | -0.0691 | -39 | 908 | 922 | ASVLTLGQLCIYCNR | | | Carbamidomethyl (C)[10,13] | | Mascot |
| 1901.9425 | 1901.9963 | 0.0538 | 28 | 129 | 144 | DGLYNLMKLANDVTYR | | | Oxidation (M)[7] | | Mascot |
| 1915.9364 | 1915.9642 | 0.0278 | 15 | 236 | 252 | VLCCAPSNVAVDNLVER | | | Carbamidomethyl (C)[3,4] | | Mascot |
| 2114.1096 | 2114.1943 | 0.0847 | 40 | 236 | 255 | VLCCAPSNVAVDNLVERLAK | | | | | Mascot |
| 2114.1096 | 2114.1943 | 0.0847 | 40 | 236 | 255 | VLCCAPSNVAVDNLVERLAK | | | | | Mascot |
| 2136.051 | 2136.1777 | 0.1267 | 59 | 41 | 59 | GVCLMKLQIGSQHTGMYGR | | | Carbamidomethyl (C)[3] | | Mascot |
| 2489.3721 | 2489.0869 | -0.2852 | -115 | 508 | 531 | GNQGEVDIVALHIKALTEAGVQVK | | | | | Mascot |
| 2515.167 | 2515.2476 | 0.0806 | 32 | 631 | 651 | HGELRTAFEYLEDCVAQNYTR | | | | | Mascot |
| 3338.6208 | 3338.7676 | 0.1468 | 44 | 872 | 905 | AGATDIAAAANGDDDFDALINAVVKAESVCGFVK | | | | | Mascot |

| | | | | | | | |
|---|---|---|---|---|---|---|---|
| 27 | ornithine decarboxylase [Danio rerio] | gi\|18859145 | 50756.2 | 5.08 | 8 | 29 | 0 |

Peptide Information

| Calc. Mass | Obsrv. Mass | ± da | ± ppm | Start Seq. | End Seq. | Sequence | Ion Score | C. I. % | Modification | Rank | Result Type |
|---|---|---|---|---|---|---|---|---|---|---|---|
| 842.5458 | 842.5123 | -0.0335 | -40 | 179 | 185 | LLLERAK | | | | | Mascot |
| 1767.8945 | 1767.8188 | -0.0757 | -43 | 75 | 92 | AVVTTLASLGAGFDCASK | | | Carbamidomethyl (C)[15] | | Mascot |
| 2196.9561 | 2197.1292 | 0.1731 | 79 | 295 | 313 | VIMKEQSASDEEEDVSNDR | | | Oxidation (M)[3] | | Mascot |
| 2357.1665 | 2357.1387 | -0.0278 | -12 | 423 | 445 | AQGIPALPLEEPSAGNVPSHCGR | | | Carbamidomethyl (C)[21] | | Mascot |
| 2400.1282 | 2400.1211 | -0.0071 | -3 | 70 | 92 | CNDSRAVVTTLASLGAGFDCASK | | | Carbamidomethyl (C)[1,20] | | Mascot |
| 2431.999 | 2432.1443 | 0.1453 | 60 | 1 | 21 | MTACTGSDFDFAFLEEGFCAR | | | Carbamidomethyl (C)[4,19] | | Mascot |
| 2431.999 | 2432.1443 | 0.1453 | 60 | 1 | 21 | MTACTGSDFDFAFLEEGFCAR | | | Carbamidomethyl (C)[4,19] | | Mascot |
| 2447.9941 | 2448.1497 | 0.1556 | 64 | 1 | 21 | MTACTGSDFDFAFLEEGFCAR | | | Carbamidomethyl (C)[4,19], Oxidation (M)[1] | | Mascot |
| 2447.9941 | 2448.1497 | 0.1556 | 64 | 1 | 21 | MTACTGSDFDFAFLEEGFCAR | | | Carbamidomethyl (C)[4,19], Oxidation (M)[1] | | Mascot |
| 3103.3481 | 3103.6494 | 0.3013 | 97 | 1 | 27 | MTACTGSDFDFAFLEEGFCARDIVEQK | | | Carbamidomethyl (C)[4], Oxidation (M)[1] | | Mascot |
| 3319.7202 | 3319.4426 | -0.2776 | -84 | 75 | 107 | AVVTTLASLGAGFDCASKTEIQIVQSVGVDPSR | | | | | Mascot |

| | | | | | | | |
|---|---|---|---|---|---|---|---|
| 28 | olfactory receptor [Danio rerio] | gi\|7211675 | 18109.8 | 9.4 | 5 | 28 | 0 |

Peptide Information

| Calc. Mass | Obsrv. Mass | ± da | ± ppm | Start Seq. | End Seq. | Sequence | Ion Score | C. I. % | Modification | Rank | Result Type |
|---|---|---|---|---|---|---|---|---|---|---|---|
| 901.4448 | 901.5096 | 0.0648 | 72 | 9 | 15 | YTTIMTR | | | Oxidation (M)[5] | | Mascot |
| 1076.5735 | 1076.4761 | -0.0974 | -90 | 140 | 148 | VSKFDPEVR | | | | | Mascot |
| 1751.9296 | 1751.8234 | -0.1062 | -61 | 1 | 15 | VAICNPLRYTTIMTR | | | | | Mascot |

| | | | | | | | | | | | |
|---|---|---|---|---|---|---|---|---|---|---|---|
| 1767.9244 | 1767.8188 | -0.1056 | -60 | 1 | 15 | VAICNPLRYTTIMTR | | | Oxidation (M)[13] | | Mascot |
| 2135.967 | 2136.1777 | 0.2107 | 99 | 53 | 71 | HMFCDHSSVLTLACVDTTR | | | | | Mascot |
| 3337.7476 | 3337.7583 | 0.0107 | 3 | 111 | 139 | LKAGATCVSHLIVVLISYCSAFFVYISYR | | | Carbamidomethyl (C)[7,19] | | Mascot |

| | | | | | | | | | |
|---|---|---|---|---|---|---|---|---|---|
| 29 | tudor and KH domain-containing protein [Danio rerio] | gi\|62122909 | 63641 | 4.89 | 7 | 28 | 0 | 12 | 0 |

**Peptide Information**

| Calc. Mass | Obsrv. Mass | ± da | ± ppm | Start Seq. | End Seq. | Sequence | Ion Score | C. I. % | Modification | Rank | Result Type |
|---|---|---|---|---|---|---|---|---|---|---|---|
| 864.3482 | 864.4503 | 0.1021 | 118 | 525 | 531 | GDWDESR | | | | | Mascot |
| 959.5156 | 959.5182 | 0.0026 | 3 | 223 | 230 | VIENETVR | | | | | Mascot |
| 1112.5344 | 1112.5112 | -0.0232 | -21 | 387 | 395 | DHGTWNRAR | | | | | Mascot |
| 1436.7124 | 1436.7466 | 0.0342 | 24 | 211 | 222 | EIQSAKEMIMEK | | | | | Mascot |
| 1488.8533 | 1488.7906 | -0.0627 | -42 | 85 | 97 | ISVPLDVYRSIAR | 12 | 0 | | | Mascot |
| 1488.8533 | 1488.7906 | -0.0627 | -42 | 85 | 97 | ISVPLDVYRSIAR | | | | | Mascot |
| 1736.8557 | 1736.8944 | 0.0387 | 22 | 217 | 230 | EMIMEKVIENETVR | | | Oxidation (M)[2] | | Mascot |
| 2459.064 | 2459.1106 | 0.0466 | 19 | 510 | 531 | LGHAVSCEDEGNGLRGDWDESR | | | Carbamidomethyl (C)[7] | | Mascot |

| | | | | | | | |
|---|---|---|---|---|---|---|---|
| 30 | PREDICTED: hypothetical protein LOC565734 [Danio rerio] | gi\|68402113 | 30582.9 | 10.05 | 6 | 28 | 0 |

**Peptide Information**

| Calc. Mass | Obsrv. Mass | ± da | ± ppm | Start Seq. | End Seq. | Sequence | Ion Score | C. I. % | Modification | Rank | Result Type |
|---|---|---|---|---|---|---|---|---|---|---|---|
| 1165.6324 | 1165.7515 | 0.1191 | 102 | 118 | 128 | VSPNATHAQIK | | | | | Mascot |
| 1436.7943 | 1436.7466 | -0.0477 | -33 | 80 | 91 | HWCLLLRPVGSR | | | | | Mascot |
| 1902.0093 | 1901.9963 | -0.013 | -7 | 20 | 36 | TLLSLHHHPHGPVVDSR | | | | | Mascot |
| 2284.0571 | 2284.1846 | 0.1275 | 56 | 41 | 60 | KSNVCVHWCAADCVWALHNK | | | | | Mascot |
| 2341.0786 | 2341.1333 | 0.0547 | 23 | 41 | 60 | KSNVCVHWCAADCVWALHNK | | | Carbamidomethyl (C)[5] | | Mascot |
| 2448.3259 | 2448.1497 | -0.1762 | -72 | 15 | 36 | LSAFKTLLSLHHHPHGPVVDSR | | | | | Mascot |
| 2448.3259 | 2448.1497 | -0.1762 | -72 | 15 | 36 | LSAFKTLLSLHHHPHGPVVDSR | | | | | Mascot |
| 2479.5107 | 2479.3013 | -0.2094 | -84 | 246 | 269 | EQITGVSVILLLLLGGVIINSLKS | | | | | Mascot |

| | | | | | | | |
|---|---|---|---|---|---|---|---|
| 31 | complement component c3b [Danio rerio] | gi\|220673392 | 182155.7 | 6.42 | 16 | 28 | 0 |

**Peptide Information**

| Calc. Mass | Obsrv. Mass | ± da | ± ppm | Start Seq. | End Seq. | Sequence | Ion Score | C. I. % | Modification | Rank | Result Type |
|---|---|---|---|---|---|---|---|---|---|---|---|
| 864.3998 | 864.4503 | 0.0505 | 58 | 1457 | 1462 | YYHPER | | | | | Mascot |

| | | | | | | | | | | | |
|---|---|---|---|---|---|---|---|---|---|---|---|
| 901.5352 | 901.5096 | -0.0256 | -28 | 1556 | 1564 | VSLGLVEGK | | | | | Mascot |
| 1045.4796 | 1045.5662 | 0.0866 | 83 | 1535 | 1544 | EGTDPDVQGK | | | | | Mascot |
| 1112.5443 | 1112.5112 | -0.0331 | -30 | 1488 | 1496 | KNHVNESER | | | | | Mascot |
| 1128.6259 | 1128.5063 | -0.1196 | -106 | 912 | 921 | TEKLELNPGK | | | | | Mascot |
| 1210.6613 | 1210.6035 | -0.0578 | -48 | 630 | 640 | TVPECPAPLKR | | | | | Mascot |
| 1436.7025 | 1436.7466 | 0.0441 | 31 | 37 | 48 | NFPAKNVEMMQK | | | | | Mascot |
| 1908.854 | 1908.9408 | 0.0868 | 45 | 1610 | 1627 | YIGMTAVENMFTTGGCLT | | | | | Mascot |
| 1915.9661 | 1915.9642 | -0.0019 | -1 | 1079 | 1094 | QLPDGSFKEDKPVYHR | | | | | Mascot |
| 2283.1504 | 2283.1875 | 0.0371 | 16 | 1149 | 1170 | LTNPYAAALTSYALANAD SLDK | | | | | Mascot |
| 2357.2029 | 2357.1387 | -0.0642 | -27 | 1535 | 1555 | EGTDPDVQGKVKPFLAR PSCR | | | Carbamidomethyl (C)[20] | | Mascot |
| 2448.0984 | 2448.1497 | 0.0513 | 21 | 606 | 629 | DSMGVFSDAGLIFESNTA GGTDTR | | | | | Mascot |
| 2448.0984 | 2448.1497 | 0.0513 | 21 | 606 | 629 | DSMGVFSDAGLIFESNTA GGTDTR | | | | | Mascot |
| 2457.2178 | 2457.3203 | 0.1025 | 42 | 302 | 323 | SIYVSVNLLTESGSEMVE AEKR | | | Oxidation (M)[16] | | Mascot |
| 2515.3113 | 2515.2476 | -0.0637 | -25 | 863 | 885 | DSSVAVSFVIVPMTLESQ HIEVK | | | | | Mascot |
| 2531.3062 | 2531.2427 | -0.0635 | -25 | 863 | 885 | DSSVAVSFVIVPMTLESQ HIEVK | | | Oxidation (M)[13] | | Mascot |
| 3000.4924 | 3000.2488 | -0.2436 | -81 | 1428 | 1453 | MHKTFNVGLLQPASVTIY EYYSPDAR | | | | | Mascot |
| 3891.9255 | 3891.8274 | -0.0981 | -25 | 1087 | 1121 | EDKPVYHREIMGGIEGQ NTDVSLTAFVLIAMQEGK | | | Oxidation (M)[11] | | Mascot |
| 3907.9204 | 3907.8484 | -0.072 | -18 | 1087 | 1121 | EDKPVYHREIMGGIEGQ NTDVSLTAFVLIAMQEGK | | | Oxidation (M)[11,31] | | Mascot |

| | | | | | | | | |
|---|---|---|---|---|---|---|---|---|
| 32 | PREDICTED: NACHT, LRR and PYD domains-containing protein 14-like [Danio rerio] | gi\|326678388 | 102092.3 | 5.48 | 11 | 28 | 0 | |

**Peptide Information**

| Calc. Mass | Obsrv. Mass | ± da | ± ppm | Start Seq. | End Seq. | Sequence | Ion Score | C. I. % | Modification | Rank | Result Type |
|---|---|---|---|---|---|---|---|---|---|---|---|
| 842.5458 | 842.5123 | -0.0335 | -40 | 835 | 841 | LNTLILR | | | | | Mascot |
| 1210.7089 | 1210.6035 | -0.1054 | -87 | 832 | 841 | HCKLNTLILR | | | | | Mascot |
| 1542.8024 | 1542.8898 | 0.0874 | 57 | 181 | 193 | HLFEASAQDKQIR | | | | | Mascot |
| 1736.9006 | 1736.8944 | -0.0062 | -4 | 221 | 234 | ENQEISFIFPLPFR | | | | | Mascot |
| 1893.8357 | 1893.9885 | 0.1528 | 81 | 497 | 512 | ASDYSGMCTQIFKEEK | | | Carbamidomethyl (C)[8] | | Mascot |
| 1893.8357 | 1893.9885 | 0.1528 | 81 | 497 | 512 | ASDYSGMCTQIFKEEK | | | Carbamidomethyl (C)[8] | | Mascot |
| 1999.9794 | 1999.9485 | -0.0309 | -15 | 121 | 138 | YQCVFEGIAQQGDSTLLK | | | | | Mascot |
| 1999.9794 | 1999.9485 | -0.0309 | -15 | 121 | 138 | YQCVFEGIAQQGDSTLLK | | | | | Mascot |
| 2114.0876 | 2114.1943 | 0.1067 | 50 | 568 | 586 | TAVDKALESDNGHLDLFL R | | | | | Mascot |
| 2114.0876 | 2114.1943 | 0.1067 | 50 | 568 | 586 | TAVDKALESDNGHLDLFL R | | | | | Mascot |

| Calc. Mass | Obsrv. Mass | ± da | ± ppm | Start Seq. | End Seq. | Sequence | Ion Score | C. I. % | Modification | Rank | Result Type |
|---|---|---|---|---|---|---|---|---|---|---|---|
| 2197.1169 | 2197.1292 | 0.0123 | 6 | 723 | 743 | ALLSDCSISEEGYKALAS ALR | | | | | Mascot |
| 2298.1812 | 2298.1902 | 0.009 | 4 | 429 | 446 | TLTQMYTHFLRFQIQQSR | | | | | Mascot |
| 2400.179 | 2400.1211 | -0.0579 | -24 | 612 | 631 | VDQQQAHNDQSKEEIVQ YIK | | | | | Mascot |
| 3303.7202 | 3303.4224 | -0.2978 | -90 | 376 | 402 | SLFIMCHIPVFCWISATVL QNILQEKR | | | Carbamidomethyl (C)[6,12] | | Mascot |
| 3319.7151 | 3319.4426 | -0.2725 | -82 | 376 | 402 | SLFIMCHIPVFCWISATVL QNILQEKR | | | Carbamidomethyl (C)[6,12], Oxidation (M)[5] | | Mascot |

| | | | | | | | |
|---|---|---|---|---|---|---|---|
| 33 | ATPase, H+ transporting, lysosomal V1 subunit B2 [Danio rerio] | gi\|133777410 | 56441.8 | 5.5 | 8 | 28 | 0 |

**Peptide Information**

| Calc. Mass | Obsrv. Mass | ± da | ± ppm | Start Seq. | End Seq. | Sequence | Ion Score | C. I. % | Modification | Rank | Result Type |
|---|---|---|---|---|---|---|---|---|---|---|---|
| 842.4478 | 842.5123 | 0.0645 | 77 | 314 | 320 | EEVPGRR | | | | | Mascot |
| 901.5101 | 901.5096 | -0.0005 | -1 | 29 | 36 | ENVLAVTR | | | | | Mascot |
| 1751.8962 | 1751.8234 | -0.0728 | -42 | 494 | 508 | IPQSTLAEFYPRDSK | | | | | Mascot |
| 2197.0383 | 2197.1292 | 0.0909 | 41 | 321 | 339 | GFPGYMYTDLATIYERAG R | | | Oxidation (M)[6] | | Mascot |
| 2341.1162 | 2341.1333 | 0.0171 | 7 | 109 | 129 | TTCEFTGDILRTPVSEDM LGR | | | | | Mascot |
| 2357.1111 | 2357.1387 | 0.0276 | 12 | 109 | 129 | TTCEFTGDILRTPVSEDM LGR | | | Oxidation (M)[18] | | Mascot |
| 2400.1687 | 2400.1211 | -0.0476 | -20 | 141 | 162 | GPAVLAEDYLDIMGQPIN PQCR | | | | | Mascot |
| 2457.1899 | 2457.3203 | 0.1304 | 53 | 141 | 162 | GPAVLAEDYLDIMGQPIN PQCR | | | Carbamidomethyl (C)[21] | | Mascot |
| 2531.3286 | 2531.2427 | -0.0859 | -34 | 291 | 313 | HVLVILTDMSSYAEALRE VSAAR | | | | | Mascot |
| 3000.335 | 3000.2488 | -0.0862 | -29 | 245 | 270 | SDFEENGSMDNVCLFLN LANDPTIER | | | Carbamidomethyl (C)[13] | | Mascot |

| | | | | | | | |
|---|---|---|---|---|---|---|---|
| 34 | hypothetical protein LOC570464 [Danio rerio] | gi\|139948991 | 19666.3 | 6.08 | 5 | 28 | 0 |

**Peptide Information**

| Calc. Mass | Obsrv. Mass | ± da | ± ppm | Start Seq. | End Seq. | Sequence | Ion Score | C. I. % | Modification | Rank | Result Type |
|---|---|---|---|---|---|---|---|---|---|---|---|
| 1045.4697 | 1045.5662 | 0.0965 | 92 | 96 | 104 | DVEFDHAGR | | | | | Mascot |
| 1128.5208 | 1128.5063 | -0.0145 | -13 | 78 | 87 | AESFPSEYAK | | | | | Mascot |
| 1916.0431 | 1915.9642 | -0.0789 | -41 | 2 | 17 | MQRCCAIFLLLHLIGK | | | Carbamidomethyl (C)[4] | | Mascot |
| 2136.0947 | 2136.1777 | 0.083 | 39 | 1 | 17 | MMQRCCAIFLLLHLIGK | | | Carbamidomethyl (C)[5,6], Oxidation (M)[1,2] | | Mascot |
| 2284.2297 | 2284.1846 | -0.0451 | -20 | 105 | 123 | YTCFFPELNVEKLVELLV K | | | | | Mascot |
| 2341.2512 | 2341.1333 | -0.1179 | -50 | 105 | 123 | YTCFFPELNVEKLVELLV K | | | Carbamidomethyl (C)[3] | | Mascot |

| | | | | | | | |
|---|---|---|---|---|---|---|---|
| 35 | calcium-binding mitochondrial carrier protein SCaMC-2-B [Danio rerio] | gi\|229608957 | 52678 | 8.35 | 8 | 28 | 0 |

Peptide Information

| Calc. Mass | Obsrv. Mass | ± da | ± ppm | Start Seq. | End Seq. | Sequence | Ion Score | C. I. % | Modification | Rank | Result Type |
|---|---|---|---|---|---|---|---|---|---|---|---|
| 850.4702 | 850.5433 | 0.0731 | 86 | 122 | 128 | ILKSMDK | | | Oxidation (M)[5] | | Mascot |
| 1045.5023 | 1045.5662 | 0.0639 | 61 | 261 | 268 | FMAYEQIK | | | Oxidation (M)[2] | | Mascot |
| 1542.8486 | 1542.8898 | 0.0412 | 27 | 270 | 283 | LIGSNQETLGILER | | | | | Mascot |
| 1999.8636 | 1999.9485 | 0.0849 | 42 | 125 | 140 | SMDKNGTMTIDWNEWR | | | Oxidation (M)[2] | | Mascot |
| 1999.8636 | 1999.9485 | 0.0849 | 42 | 125 | 140 | SMDKNGTMTIDWNEWR | | | Oxidation (M)[2] | | Mascot |
| 2114.0117 | 2114.1943 | 0.1826 | 86 | 221 | 240 | SNSMGIAGGFTQMIREG GLR | | | Oxidation (M)[4,13] | | Mascot |
| 2114.0117 | 2114.1943 | 0.1826 | 86 | 221 | 240 | SNSMGIAGGFTQMIREG GLR | | | Oxidation (M)[4,13] | | Mascot |
| 2232.9683 | 2233.0996 | 0.1313 | 59 | 405 | 424 | MQAQASQEGSPQMTMS GLFR | | | Oxidation (M)[1,13,15] | | Mascot |
| 2442.1323 | 2442.083 | -0.0493 | -20 | 403 | 424 | TRMQAQASQEGSPQMT MSGLFR | | | | | Mascot |
| 3891.9077 | 3891.8274 | -0.0803 | -21 | 368 | 404 | FATDSADPGVFVLLACGT MSSTCGQLASYPLALVR TR | | | Carbamidomethyl (C)[16], Oxidation (M)[19] | | Mascot |

36 hypothetical protein LOC100007529 [Danio rerio] gi|164414401 18354.1 6.11 5 27 0

Peptide Information

| Calc. Mass | Obsrv. Mass | ± da | ± ppm | Start Seq. | End Seq. | Sequence | Ion Score | C. I. % | Modification | Rank | Result Type |
|---|---|---|---|---|---|---|---|---|---|---|---|
| 882.4944 | 882.5708 | 0.0764 | 87 | 45 | 51 | LISAHWR | | | | | Mascot |
| 1952.0747 | 1951.9611 | -0.1136 | -58 | 147 | 163 | IVSQVLACFFAVILLCV | | | Carbamidomethyl (C)[8,16] | | Mascot |
| 2284.0378 | 2284.1846 | 0.1468 | 64 | 23 | 42 | TFEGFIGGSALFPCYYDP SR | | | Carbamidomethyl (C)[14] | | Mascot |
| 2442.2888 | 2442.083 | -0.2058 | -84 | 45 | 65 | LISAHWRLNDSLNVYDIL SGR | | | | | Mascot |
| 2479.2722 | 2479.3013 | 0.0291 | 12 | 1 | 22 | MFRSGFLICFLISLSSEAS VQK | | | Oxidation (M)[1] | | Mascot |

37 Zgc:65788 protein [Danio rerio] gi|28279226 55688.4 7.59 8 27 0

Peptide Information

| Calc. Mass | Obsrv. Mass | ± da | ± ppm | Start Seq. | End Seq. | Sequence | Ion Score | C. I. % | Modification | Rank | Result Type |
|---|---|---|---|---|---|---|---|---|---|---|---|
| 864.3491 | 864.4503 | 0.1012 | 117 | 495 | 500 | CCNWPK | | | Carbamidomethyl (C)[1,2] | | Mascot |
| 959.5672 | 959.5182 | -0.049 | -51 | 395 | 402 | YPLIGQLR | | | | | Mascot |
| 1045.6074 | 1045.5662 | -0.0412 | -39 | 198 | 208 | LMLTAAVAAGK | | | | | Mascot |
| 1112.6058 | 1112.5112 | -0.0946 | -85 | 272 | 281 | DQGAPVEKLR | | | | | Mascot |
| 1436.666 | 1436.7466 | 0.0806 | 56 | 483 | 494 | CPAKTVFDPNCK | | | Carbamidomethyl (C)[1,11] | | Mascot |
| 2442.2371 | 2442.083 | -0.1541 | -63 | 290 | 315 | TFRLSSAVSGVGAPASG AASAGTYTR | | | | | Mascot |
| 2489.1077 | 2489.0869 | -0.0208 | -8 | 251 | 271 | GSGDIGDKIYYNTDFAMT | | | Oxidation (M)[17] | | Mascot |

| | | | | | | | | | | | | |
|---|---|---|---|---|---|---|---|---|---|---|---|---|
| 3103.5007 | 3103.6494 | 0.1487 | 48 | 86 | 111 | YWR LTTYEWNDETLYQSFNG LKQSNPNLK | | | | | | Mascot |

| | | | | | | | |
|---|---|---|---|---|---|---|---|
| 38 | PREDICTED: hypothetical protein LOC799338 [Danio rerio] | gi\|326667977 | 158597.6 | 6.54 | 15 | 27 | 0 |

**Peptide Information**

| Calc. Mass | Obsrv. Mass | ± da | ± ppm | Start Seq. | End Seq. | Sequence | Ion Score | C. I. % | Modification | Rank | Result Type |
|---|---|---|---|---|---|---|---|---|---|---|---|
| 1076.5154 | 1076.4761 | -0.0393 | -37 | 1357 | 1365 | SKMSDSHLR | | | Oxidation (M)[3] | | Mascot |
| 1112.5558 | 1112.5112 | -0.0446 | -40 | 1321 | 1329 | FPEMRSFAK | | | | | Mascot |
| 1128.5507 | 1128.5063 | -0.0444 | -39 | 1321 | 1329 | FPEMRSFAK | | | Oxidation (M)[4] | | Mascot |
| 1165.5922 | 1165.7515 | 0.1593 | 137 | 1081 | 1090 | FDDVMKPVAK | | | Oxidation (M)[5] | | Mascot |
| 1210.5707 | 1210.6035 | 0.0328 | 27 | 926 | 935 | QEFMNVCLAR | | | | | Mascot |
| 1354.6605 | 1354.7754 | 0.1149 | 85 | 925 | 935 | KQEFMNVCLAR | | | Oxidation (M)[5] | | Mascot |
| 1354.6605 | 1354.7754 | 0.1149 | 85 | 925 | 935 | KQEFMNVCLAR | | | Oxidation (M)[5] | | Mascot |
| 1751.7793 | 1751.8234 | 0.0441 | 25 | 376 | 390 | AFLDDENQEDLMPAK | | | Oxidation (M)[12] | | Mascot |
| 1767.8629 | 1767.8188 | -0.0441 | -25 | 926 | 940 | QEFMNVCLARNTVAR | | | Oxidation (M)[4] | | Mascot |
| 1893.9263 | 1893.9885 | 0.0622 | 33 | 331 | 346 | LKVTFQDDQEDLMPAK | | | Oxidation (M)[13] | | Mascot |
| 1893.9263 | 1893.9885 | 0.0622 | 33 | 79 | 94 | LKVTFQDDQEDLMPAK | | | Oxidation (M)[13] | | Mascot |
| 1923.8389 | 1923.9712 | 0.1323 | 69 | 292 | 307 | AFQDDEDQEDLMPAKR | | | Oxidation (M)[12] | | Mascot |
| 2197.1545 | 2197.1292 | -0.0253 | -12 | 636 | 655 | VCGIRPVHFALEENVTSV AR | | | | | Mascot |
| 2294.0103 | 2294.1719 | 0.1616 | 70 | 264 | 283 | RKPSQFPDDNEEPSTSS GCR | | | Carbamidomethyl (C)[19] | | Mascot |
| 2294.0103 | 2294.1719 | 0.1616 | 70 | 264 | 283 | RKPSQFPDDNEEPSTSS GCR | | | Carbamidomethyl (C)[19] | | Mascot |
| 2340.9456 | 2341.1333 | 0.1877 | 80 | 179 | 199 | RCEPSQFPDDNEEPSTS SGCR | | | | | Mascot |
| 2489.2095 | 2489.0869 | -0.1226 | -49 | 1111 | 1130 | DIQAEYGDLIYYNDVRWL SR | | | | | Mascot |
| 3000.3589 | 3000.2488 | -0.1101 | -37 | 601 | 626 | LIDFGCSEYCQGHLKPHA GFDQIYCR | | | | | Mascot |
| 3891.8601 | 3891.8274 | -0.0327 | -8 | 1268 | 1301 | DMLLFSSPLSADPDNAPP RLQLELIELQCDYECR | | | | | Mascot |
| 3907.855 | 3907.8484 | -0.0066 | -2 | 1268 | 1301 | DMLLFSSPLSADPDNAPP RLQLELIELQCDYECR | | | Oxidation (M)[2] | | Mascot |

| | | | | | | | |
|---|---|---|---|---|---|---|---|
| 39 | novel protein similar to vertebrate aggrecan 1 (chondroitin sulfate proteoglycan 1, large aggregati | gi\|94732656 | 18946.2 | 8.77 | 5 | 27 | 0 |

**Peptide Information**

| Calc. Mass | Obsrv. Mass | ± da | ± ppm | Start Seq. | End Seq. | Sequence | Ion Score | C. I. % | Modification | Rank | Result Type |
|---|---|---|---|---|---|---|---|---|---|---|---|
| 959.5097 | 959.5182 | 0.0085 | 9 | 102 | 109 | AHLYAAWK | | | | | Mascot |
| 1436.6879 | 1436.7466 | 0.0587 | 41 | 91 | 101 | YDVYCYVDKLR | | | | | Mascot |

| Calc. Mass | Obsrv. Mass | ± da | ± ppm | Start Seq. | End Seq. | Sequence | Ion Score | C. I. % | Modification | Rank | Result Type |
|---|---|---|---|---|---|---|---|---|---|---|---|
| 1542.6788 | 1542.8898 | 0.211 | 137 | 67 | 80 | HQCSGDMEQVPGVR | | | | | Mascot |
| 2298.1514 | 2298.1902 | 0.0388 | 17 | 141 | 161 | TGVHTVYLFPNQTGSPD LHSK | | | | | Mascot |
| 2984.4431 | 2984.2456 | -0.1975 | -66 | 134 | 161 | QQCGGGRTGVHTVYLFP NQTGSPDLHSK | | | | | Mascot |

40 PREDICTED: paired box protein Pax-6-like [Danio rerio] gi|326680199 30178.1 9.33 6 27 0

**Peptide Information**

| Calc. Mass | Obsrv. Mass | ± da | ± ppm | Start Seq. | End Seq. | Sequence | Ion Score | C. I. % | Modification | Rank | Result Type |
|---|---|---|---|---|---|---|---|---|---|---|---|
| 996.5472 | 996.6025 | 0.0553 | 55 | 64 | 72 | ILQTHADAK | | | | | Mascot |
| 1542.7039 | 1542.8898 | 0.1859 | 121 | 168 | 180 | QQMGADGMYEKLR | | | Oxidation (M)[3] | | Mascot |
| 1736.8239 | 1736.8944 | 0.0705 | 41 | 212 | 227 | TNNINSGPPSSFSTWK | | | | | Mascot |
| 1915.8524 | 1915.9642 | 0.1118 | 58 | 162 | 178 | NLASEKQQMGADGMYE K | | | Oxidation (M)[9] | | Mascot |
| 2175.071 | 2175.1472 | 0.0762 | 35 | 139 | 158 | LLSEGVCTNDNIPSVSSIN R | | | Carbamidomethyl (C)[7] | | Mascot |
| 2175.071 | 2175.1472 | 0.0762 | 35 | 139 | 158 | LLSEGVCTNDNIPSVSSIN R | | | Carbamidomethyl (C)[7] | | Mascot |
| 3287.5649 | 3287.4243 | -0.1406 | -43 | 181 | 210 | MLNGQTGTWGTRPGWY PGTSVPGQPNQVER | | | Oxidation (M)[1] | | Mascot |

41 novel protein [Danio rerio] gi|94732798 158511.5 5.14 13 27 0

**Peptide Information**

| Calc. Mass | Obsrv. Mass | ± da | ± ppm | Start Seq. | End Seq. | Sequence | Ion Score | C. I. % | Modification | Rank | Result Type |
|---|---|---|---|---|---|---|---|---|---|---|---|
| 864.4607 | 864.4503 | -0.0104 | -12 | 959 | 966 | QSSALKCK | | | | | Mascot |
| 901.5618 | 901.5096 | -0.0522 | -58 | 1381 | 1387 | KVVLWTR | | | | | Mascot |
| 1488.8016 | 1488.7906 | -0.011 | -7 | 951 | 964 | AGQEQTLKQSSALK | | | | | Mascot |
| 1488.8016 | 1488.7906 | -0.011 | -7 | 951 | 964 | AGQEQTLKQSSALK | | | | | Mascot |
| 1719.8894 | 1719.859 | -0.0304 | -18 | 611 | 624 | HLRGTLHPLQMTCR | | | Carbamidomethyl (C)[13] | | Mascot |
| 1719.8894 | 1719.859 | -0.0304 | -18 | 611 | 624 | HLRGTLHPLQMTCR | | | Carbamidomethyl (C)[13] | | Mascot |
| 1736.8384 | 1736.8944 | 0.056 | 32 | 1202 | 1216 | ECGLLAEQQAFERSR | | | | | Mascot |
| 1751.9473 | 1751.8234 | -0.1239 | -71 | 433 | 449 | ALLESKGVSVPPACPQR | | | | | Mascot |
| 1767.8409 | 1767.8188 | -0.0221 | -13 | 1106 | 1120 | KTVLHNDASDHDPYR | | | | | Mascot |
| 2136.1018 | 2136.1777 | 0.0759 | 36 | 595 | 613 | GPFTSAEDCLVVLGHKHL R | | | Carbamidomethyl (C)[9] | | Mascot |
| 2459.0991 | 2459.1106 | 0.0115 | 5 | 1348 | 1371 | DSEASLGDSQQSLFNAT GSSVCAK | | | Carbamidomethyl (C)[22] | | Mascot |
| 3103.5774 | 3103.6494 | 0.072 | 23 | 56 | 82 | QHNLTSANVRSIIHEVITN EHVVAMMK | | | Oxidation (M)[25,26] | | Mascot |
| 3303.4583 | 3303.4224 | -0.0359 | -11 | 664 | 694 | TYNQASGNPQTTEATDP AGPGPHFCFPPGMR | | | Carbamidomethyl (C)[25] | | Mascot |
| 3319.4531 | 3319.4426 | -0.0105 | -3 | 664 | 694 | TYNQASGNPQTTEATDP AGPGPHFCFPPGMR | | | Carbamidomethyl (C)[25], Oxidation (M)[30] | | Mascot |
| 3338.636 | 3338.7676 | 0.1316 | 39 | 519 | 548 | AEHGFFTSIFNACNLQGA | | | Carbamidomethyl (C)[13] | | Mascot |

| Calc. Mass | Obsrv. Mass | ± da | ± ppm | Start Seq. | End Seq. | Sequence | Ion Score | C. I. % | Modification | Rank | Result Type |
|---|---|---|---|---|---|---|---|---|---|---|---|
| | | | | | | VSLLEELDQSLK | | | | | |
| 3891.78 | 3891.8274 | 0.0474 | 12 | 232 | 267 | LHAVDEELAFGTDCMDSYQSLGGASGEESLISFRTR | | | | | Mascot |
| 3907.7749 | 3907.8484 | 0.0735 | 19 | 232 | 267 | LHAVDEELAFGTDCMDSYQSLGGASGEESLISFRTR | | | Oxidation (M)[15] | | Mascot |

| | | | | | | | |
|---|---|---|---|---|---|---|---|
| 42 | Max protein [Danio rerio] | gi\|214997 | 17750.8 | 6.66 | 4 | 26 | 0 |

**Peptide Information**

| Calc. Mass | Obsrv. Mass | ± da | ± ppm | Start Seq. | End Seq. | Sequence | Ion Score | C. I. % | Modification | Rank | Result Type |
|---|---|---|---|---|---|---|---|---|---|---|---|
| 1103.5817 | 1103.575 | -0.0067 | -6 | 16 | 24 | RAHHNALER | | | | | Mascot |
| 1354.755 | 1354.7754 | 0.0204 | 15 | 86 | 96 | RQNALLEQQVR | 6 | 0 | | | Mascot |
| 1354.755 | 1354.7754 | 0.0204 | 15 | 86 | 96 | RQNALLEQQVR | | | | | Mascot |
| 2283.9595 | 2284.1846 | 0.2251 | 99 | 124 | 146 | GQAVSAFDGGSDSSSGSEPEEQR | | | | | Mascot |
| 2489.2153 | 2489.0869 | -0.1284 | -52 | 101 | 123 | VKGTTQLQANYSSSDSSLYTNPK | | | | | Mascot |

| | | | | | | | |
|---|---|---|---|---|---|---|---|
| 43 | PREDICTED: hypothetical protein LOC100537950 [Danio rerio] | gi\|326676836 | 45410.9 | 5.02 | 7 | 26 | 0 |

**Peptide Information**

| Calc. Mass | Obsrv. Mass | ± da | ± ppm | Start Seq. | End Seq. | Sequence | Ion Score | C. I. % | Modification | Rank | Result Type |
|---|---|---|---|---|---|---|---|---|---|---|---|
| 864.5301 | 864.4503 | -0.0798 | -92 | 352 | 358 | LFLRSTK | | | | | Mascot |
| 1901.8584 | 1901.9963 | 0.1379 | 73 | 192 | 209 | QEESNRPANVSDAANGDK | | | | | Mascot |
| 2136.2461 | 2136.1777 | -0.0684 | -32 | 71 | 90 | GIILEGVLTPVMPLSLPSKR | | | Oxidation (M)[12] | | Mascot |
| 2225.1487 | 2225.126 | -0.0227 | -10 | 362 | 380 | GVLVNEYFPDVEQFVDKVK | | | | | Mascot |
| 2442.335 | 2442.083 | -0.252 | -103 | 90 | 111 | RVTLSNVPPFISDEVLTQALSR | | | | | Mascot |
| 2457.1423 | 2457.3203 | 0.178 | 72 | 187 | 209 | ICPNKQEESNRPANVSDAANGDK | | | | | Mascot |
| 3338.4224 | 3338.7676 | 0.3452 | 103 | 314 | 343 | VDMEDELESESDSELSDSSLTLSQSEFSSR | | | | | Mascot |

| | | | | | | | |
|---|---|---|---|---|---|---|---|
| 44 | HEAT repeat-containing protein 1 [Danio rerio] | gi\|41054019 | 241895.3 | 5.94 | 19 | 26 | 0 |

**Peptide Information**

| Calc. Mass | Obsrv. Mass | ± da | ± ppm | Start Seq. | End Seq. | Sequence | Ion Score | C. I. % | Modification | Rank | Result Type |
|---|---|---|---|---|---|---|---|---|---|---|---|
| 842.557 | 842.5123 | -0.0447 | -53 | 1792 | 1798 | LTLLARR | | | | | Mascot |
| 901.5101 | 901.5096 | -0.0005 | -1 | 1942 | 1949 | LADRIADK | | | | | Mascot |
| 959.4363 | 959.5182 | 0.0819 | 85 | 209 | 216 | DGNCPQLR | | | Carbamidomethyl (C)[4] | | Mascot |
| 996.4567 | 996.6025 | 0.1458 | 146 | 1853 | 1860 | EHISHMDK | | | | | Mascot |

| | | | | | | | | | | | |
|---|---|---|---|---|---|---|---|---|---|---|---|
| 1076.5768 | 1076.4761 | -0.1007 | -94 | 1618 | 1626 | KAMELLNNK | | | Oxidation (M)[3] | | Mascot |
| 1103.5878 | 1103.575 | -0.0128 | -12 | 1799 | 1808 | LTSCPQLSVR | | | | | Mascot |
| 1128.5684 | 1128.5063 | -0.0621 | -55 | 977 | 985 | FYEEAVSRK | | | | | Mascot |
| 1736.9073 | 1736.8944 | -0.0129 | -7 | 1591 | 1605 | VNALLPMDTFITVMR | | | Oxidation (M)[7] | | Mascot |
| 1908.9768 | 1908.9408 | -0.036 | -19 | 1712 | 1729 | NVMGSALLCVAEVTSTLK | | | Carbamidomethyl (C)[9], Oxidation (M)[3] | | Mascot |
| 2114.1362 | 2114.1943 | 0.0581 | 27 | 2054 | 2072 | SRITTHLVPCIAQFAVAM R | | | | | Mascot |
| 2114.1362 | 2114.1943 | 0.0581 | 27 | 2054 | 2072 | SRITTHLVPCIAQFAVAM R | | | | | Mascot |
| 2136.1104 | 2136.1777 | 0.0673 | 32 | 647 | 666 | TSESDLLGVANEMLTTTLI K | | | | | Mascot |
| 2225.0286 | 2225.126 | 0.0974 | 44 | 254 | 273 | SNLSDYTAATYMIVCQMA VK | | | Oxidation (M)[12] | | Mascot |
| 2233.2219 | 2233.0996 | -0.1223 | -55 | 1799 | 1819 | LTSCPQLSVRLASLSSTL ATK | | | Carbamidomethyl (C)[4] | | Mascot |
| 2283.1294 | 2283.1875 | 0.0581 | 25 | 1824 | 1843 | VLIPTITKCYCSMVDAQQ NR | | | | | Mascot |
| 2298.0452 | 2298.1902 | 0.145 | 63 | 254 | 273 | SNLSDYTAATYMIVCQMA VK | | | Carbamidomethyl (C)[15], Oxidation (M)[12,17] | | Mascot |
| 2442.2043 | 2442.083 | -0.1213 | -50 | 991 | 1012 | LASVEQLLQCLQSPFCPS YTSK | | | | | Mascot |
| 2448.386 | 2448.1497 | -0.2363 | -97 | 881 | 903 | VSAILQTQALYVGKAFLS SQPVK | | | | | Mascot |
| 2448.386 | 2448.1497 | -0.2363 | -97 | 881 | 903 | VSAILQTQALYVGKAFLS SQPVK | | | | | Mascot |
| 2459.155 | 2459.1106 | -0.0444 | -18 | 1832 | 1852 | CYCSMVDAQQNRLSPLM NILK | | | Oxidation (M)[5,17] | | Mascot |
| 3000.5134 | 3000.2488 | -0.2646 | -88 | 209 | 235 | DGNCPQLRVIFSFYASTI VSALDAVEK | | | Carbamidomethyl (C)[4] | | Mascot |
| 3303.6599 | 3303.4224 | -0.2375 | -72 | 1136 | 1166 | SPACAQSINSVFKTIAVD CELVANELIPADK | | | Carbamidomethyl (C)[4] | | Mascot |

| | | | | | | | |
|---|---|---|---|---|---|---|---|
| 45 | PREDICTED: hypothetical protein LOC100538187 [Danio rerio] | gi\|326671902 | 13561.6 | 8.77 | 5 | 26 | 0 |

**Peptide Information**

| Calc. Mass | Obsrv. Mass | ± da | ± ppm | Start Seq. | End Seq. | Sequence | Ion Score | C. I. % | Modification | Rank | Result Type |
|---|---|---|---|---|---|---|---|---|---|---|---|
| 901.46 | 901.5096 | 0.0496 | 55 | 84 | 90 | NFKYVCK | | | | | Mascot |
| 1488.7474 | 1488.7906 | 0.0432 | 29 | 1 | 13 | MVDKDQAEVNAIR | | | | | Mascot |
| 1488.7474 | 1488.7906 | 0.0432 | 29 | 1 | 13 | MVDKDQAEVNAIR | | | | | Mascot |
| 1901.8376 | 1901.9963 | 0.1587 | 83 | 53 | 67 | AHIIQFMSEMKCCSK | | | Carbamidomethyl (C)[12,13], Oxidation (M)[7,10] | | Mascot |
| 2225.0344 | 2225.126 | 0.0916 | 41 | 94 | 111 | NNWETIGHLWSNFGRCY K | | | | | Mascot |
| 3907.9324 | 3907.8484 | -0.084 | -21 | 15 | 48 | VFNESDILLCWYHVTQAV TRWLSISESGVSGPEK | | | Carbamidomethyl (C)[10] | | Mascot |

| | | | | | | | |
|---|---|---|---|---|---|---|---|
| 46 | novel protein similar to human BAP28 [Danio rerio] | gi\|33284848 | 143432.7 | 7.69 | 15 | 26 | 0 |

**Peptide Information**

| Calc. Mass | Obsrv. Mass | ± da | ± ppm | Start | End | Sequence | Ion | C. I. % | Modification | Rank | Result Type |
|---|---|---|---|---|---|---|---|---|---|---|---|

| | | | | Seq. | Seq. | | Score | | | | |
|---|---|---|---|---|---|---|---|---|---|---|---|
| 842.557 | 842.5123 | -0.0447 | -53 | 991 | 997 | LTLLARR | | | | | Mascot |
| 901.5101 | 901.5096 | -0.0005 | -1 | 1141 | 1148 | LADRIADK | | | | | Mascot |
| 996.4567 | 996.6025 | 0.1458 | 146 | 1052 | 1059 | EHISHMDK | | | | | Mascot |
| 1076.5768 | 1076.4761 | -0.1007 | -94 | 817 | 825 | KAMELLNNK | | | Oxidation (M)[3] | | Mascot |
| 1103.5878 | 1103.575 | -0.0128 | -12 | 998 | 1007 | LTSCPQLSVR | | | | | Mascot |
| 1128.5684 | 1128.5063 | -0.0621 | -55 | 176 | 184 | FYEEAVSRK | | | | | Mascot |
| 1736.9073 | 1736.8944 | -0.0129 | -7 | 790 | 804 | VNALLPMDTFITVMR | | | Oxidation (M)[7] | | Mascot |
| 1908.9768 | 1908.9408 | -0.036 | -19 | 911 | 928 | NVMGSALLCVAEVTSTLK | | | Carbamidomethyl (C)[9], Oxidation (M)[3] | | Mascot |
| 2114.1362 | 2114.1943 | 0.0581 | 27 | 1231 | 1249 | SRITTHLVPCIAQFAVAM R | | | | | Mascot |
| 2114.1362 | 2114.1943 | 0.0581 | 27 | 1231 | 1249 | SRITTHLVPCIAQFAVAM R | | | | | Mascot |
| 2233.2219 | 2233.0996 | -0.1223 | -55 | 998 | 1018 | LTSCPQLSVRLASLSSTL ATK | | | Carbamidomethyl (C)[4] | | Mascot |
| 2283.1294 | 2283.1875 | 0.0581 | 25 | 1023 | 1042 | VLIPTITKCYCSMVDAQQ NR | | | | | Mascot |
| 2442.2043 | 2442.083 | -0.1213 | -50 | 190 | 211 | LASVEQLLQCLQSPFCPS YTSK | | | | | Mascot |
| 2448.386 | 2448.1497 | -0.2363 | -97 | 80 | 102 | VSAILQTQALYVGKAFLS SQPVK | | | | | Mascot |
| 2448.386 | 2448.1497 | -0.2363 | -97 | 80 | 102 | VSAILQTQALYVGKAFLS SQPVK | | | | | Mascot |
| 2459.155 | 2459.1106 | -0.0444 | -18 | 1031 | 1051 | CYCSMVDAQQNRLSPLM NILK | | | Oxidation (M)[5,17] | | Mascot |
| 3303.6599 | 3303.4224 | -0.2375 | -72 | 335 | 365 | SPACAQSINSVFKTIAVD CELVANELIPADK | | | Carbamidomethyl (C)[4] | | Mascot |

47 hypothetical protein LOC100137109 [Danio rerio] gi|167963466 21724.1 5.82 5 26 0

Peptide Information

| Calc. Mass | Obsrv. Mass | ± da | ± ppm | Start Seq. | End Seq. | Sequence | Ion Score | C. I. % | Modification | Rank | Result Type |
|---|---|---|---|---|---|---|---|---|---|---|---|
| 882.4389 | 882.5708 | 0.1319 | 149 | 1 | 8 | MGSPFSLK | | | Oxidation (M)[1] | | Mascot |
| 1103.6055 | 1103.575 | -0.0305 | -28 | 175 | 184 | TSESLNVVVR | | | | | Mascot |
| 2197.1282 | 2197.1292 | 0.001 | 0 | 1 | 21 | MGSPFSLKNTITSGIVSS AQR | | | Oxidation (M)[1] | | Mascot |
| 2384.2385 | 2384.1057 | -0.1328 | -56 | 103 | 122 | YIGVMMLTLTPSIIDELRM R | | | Oxidation (M)[5,6] | | Mascot |
| 2400.2334 | 2400.1211 | -0.1123 | -47 | 103 | 122 | YIGVMMLTLTPSIIDELRM R | | | Oxidation (M)[5,6,19] | | Mascot |
| 2479.2786 | 2479.3013 | 0.0227 | 9 | 163 | 184 | VNTSEEIYNAVRTSESLN VVVR | | | | | Mascot |

48 PREDICTED: protein NLRC3 isoform 1 [Danio rerio] gi|326667049 94863 6.12 11 26 0

Peptide Information

| Calc. Mass | Obsrv. Mass | ± da | ± ppm | Start Seq. | End Seq. | Sequence | Ion Score | C. I. % | Modification | Rank | Result Type |
|---|---|---|---|---|---|---|---|---|---|---|---|

| Calc. Mass | Obsrv. Mass | ± da | ± ppm | Start Seq. | End Seq. | Sequence | Ion Score | C. I. % | Modification | Rank | Result Type |
|---|---|---|---|---|---|---|---|---|---|---|---|
| 901.4737 | 901.5096 | 0.0359 | 40 | 135 | 141 | ILQEENR | | | | | Mascot |
| 959.4614 | 959.5182 | 0.0568 | 59 | 407 | 414 | IPADCIDR | | | Carbamidomethyl (C)[5] | | Mascot |
| 1045.5272 | 1045.5662 | 0.039 | 37 | 660 | 668 | GLLTQQDDR | | | | | Mascot |
| 1210.6791 | 1210.6035 | -0.0756 | -62 | 442 | 451 | EIIDHIKQSK | | | | | Mascot |
| 1436.7631 | 1436.7466 | -0.0165 | -11 | 669 | 680 | DQSKEEIIAYIK | | | | | Mascot |
| 2114.0876 | 2114.1943 | 0.1067 | 50 | 626 | 644 | AAVDKALESENGHLDLYLR | | | | | Mascot |
| 2114.0876 | 2114.1943 | 0.1067 | 50 | 626 | 644 | AAVDKALESENGHLDLYLR | | | | | Mascot |
| 2136.072 | 2136.1777 | 0.1057 | 49 | 103 | 120 | HKSFTDNLQSIFQNLESK | | | | | Mascot |
| 2211.2092 | 2211.1123 | -0.0969 | -44 | 153 | 171 | SIITEAALDLTLFFLREMK | | | | | Mascot |
| 2294.0864 | 2294.1719 | 0.0855 | 37 | 551 | 571 | DCGIDVYKASVYSGMCTQILK | | | | | Mascot |
| 2294.0864 | 2294.1719 | 0.0855 | 37 | 551 | 571 | DCGIDVYKASVYSGMCTQILK | | | | | Mascot |
| 2298.1812 | 2298.1902 | 0.009 | 4 | 491 | 508 | TLTQMYTHFLRFQIQQSR | | | | | Mascot |
| 2341.2188 | 2341.1333 | -0.0855 | -37 | 532 | 550 | LAFQQLERNNLIFYESDLK | | | | | Mascot |

49 PREDICTED: ryanodine receptor 2-like, partial [Danio rerio] gi|326672665 23609.3 4.4 5 26 0

**Peptide Information**

| Calc. Mass | Obsrv. Mass | ± da | ± ppm | Start Seq. | End Seq. | Sequence | Ion Score | C. I. % | Modification | Rank | Result Type |
|---|---|---|---|---|---|---|---|---|---|---|---|
| 1719.7406 | 1719.859 | 0.1184 | 69 | 182 | 194 | CWEFFPAGDCFRK | | | Carbamidomethyl (C)[1,10] | | Mascot |
| 1719.7406 | 1719.859 | 0.1184 | 69 | 182 | 194 | CWEFFPAGDCFRK | | | Carbamidomethyl (C)[1,10] | | Mascot |
| 2156.873 | 2157.1394 | 0.2664 | 124 | 40 | 56 | CDDMLTCYMFHMYVGVR | | | Carbamidomethyl (C)[1], Oxidation (M)[4] | | Mascot |
| 2224.9993 | 2225.126 | 0.1267 | 57 | 57 | 77 | AGGGIGDQIEDPAGDEYEIYR | | | | | Mascot |
| 2432.0459 | 2432.1443 | 0.0984 | 40 | 163 | 181 | DETEHTGQESYVWKMYQER | | | Oxidation (M)[15] | | Mascot |
| 2432.0459 | 2432.1443 | 0.0984 | 40 | 163 | 181 | DETEHTGQESYVWKMYQER | | | Oxidation (M)[15] | | Mascot |
| 3337.292 | 3337.7583 | 0.4663 | 140 | 30 | 56 | SEDGESPDMKCDDMLTCYMFHMYVGVR | | | Carbamidomethyl (C)[11,17], Oxidation (M)[9,14,19,22] | | Mascot |

50 PREDICTED: formin-like 2b [Danio rerio] gi|326668505 125459.5 6.66 1 16 0

**Peptide Information**

| Calc. Mass | Obsrv. Mass | ± da | ± ppm | Start Seq. | End Seq. | Sequence | Ion Score | C. I. % | Modification | Rank | Result Type |
|---|---|---|---|---|---|---|---|---|---|---|---|
| 1488.712 | 1488.7906 | 0.0786 | 53 | 218 | 229 | KDDVHVCIMCLR | 16 | 0 | Carbamidomethyl (C)[7] | 2 | Mascot |

| Gel Idx/Pos | 192/H19 | Instr./Gel Origin | BA2060/110707A | Process Status | Analysis Succeeded |
|---|---|---|---|---|---|
| Plate [#] Name | [1] 31496 | Instrument Sample Name | | Spectra | 11 |

| Rank | Protein Name | Accession No. | Protein MW | Protein PI | Pep. Count | Protein Score | Protein Score C. I. % | Total Ion Score | Total Ion C. I. % |
|---|---|---|---|---|---|---|---|---|---|
| 1 | transketolase-like protein 2 [Danio rerio] | gi\|186288308 | 67795.3 | 6.81 | 21 | 1,350 | 100 | 1208 | 100 |

Peptide Information

| Calc. Mass | Obsrv. Mass | ± da | ± ppm | Start Seq. | End Seq. | Sequence | Ion Score | C. I. % | Modification | Rank | Result Type |
|---|---|---|---|---|---|---|---|---|---|---|---|
| 901.5101 | 901.5135 | 0.0034 | 4 | 93 | 100 | ASDLLNLR | 54 | 99.889 | | | Mascot |
| 901.5101 | 901.5135 | 0.0034 | 4 | 93 | 100 | ASDLLNLR | | | | | Mascot |
| 1028.4982 | 1028.5013 | 0.0031 | 3 | 226 | 234 | AMWHAEGVK | | | | | Mascot |
| 1060.4768 | 1060.4849 | 0.0081 | 8 | 347 | 355 | NSTFADMFK | | | | | Mascot |
| 1076.4717 | 1076.4808 | 0.0091 | 8 | 347 | 355 | NSTFADMFK | | | Oxidation (M)[7] | | Mascot |
| 1103.5731 | 1103.5774 | 0.0043 | 4 | 559 | 567 | VITVEDHYK | | | | | Mascot |
| 1354.7689 | 1354.7772 | 0.0083 | 6 | 174 | 185 | LDNLVAILDVNR | 108 | 100 | | | Mascot |
| 1354.7689 | 1354.7772 | 0.0083 | 6 | 174 | 185 | LDNLVAILDVNR | | | | | Mascot |
| 1436.738 | 1436.7474 | 0.0094 | 7 | 101 | 113 | KIDSDLEGHPTPK | | | | | Mascot |
| 1488.7845 | 1488.793 | 0.0085 | 6 | 385 | 398 | TVSFASTFAAFLAR | | | | | Mascot |
| 1488.7845 | 1488.793 | 0.0085 | 6 | 385 | 398 | TVSFASTFAAFLAR | 123 | 100 | | | Mascot |
| 1719.8547 | 1719.8651 | 0.0104 | 6 | 475 | 489 | TSRPDTAVIYNPEEK | | | | | Mascot |
| 1719.8547 | 1719.8651 | 0.0104 | 6 | 475 | 489 | TSRPDTAVIYNPEEK | 67 | 99.994 | | | Mascot |
| 1736.8887 | 1736.8948 | 0.0061 | 4 | 598 | 613 | SGKPQELLDMFGISAK | | | Oxidation (M)[10] | | Mascot |
| 1751.817 | 1751.8267 | 0.0097 | 6 | 252 | 266 | GIEDQDNWHGKPMPK | | | | | Mascot |
| 1767.8119 | 1767.8225 | 0.0106 | 6 | 252 | 266 | GIEDQDNWHGKPMPK | | | Oxidation (M)[13] | | Mascot |
| 1893.9817 | 1893.9919 | 0.0102 | 5 | 186 | 202 | LGQSEPAPLQHNVNVYK | 83 | 100 | | | Mascot |
| 1893.9817 | 1893.9919 | 0.0102 | 5 | 186 | 202 | LGQSEPAPLQHNVNVYK | | | | | Mascot |
| 1901.9908 | 1902.0004 | 0.0096 | 5 | 75 | 92 | GHAAPILYAAWAEAGYIK | | | | | Mascot |
| 1999.943 | 1999.9546 | 0.0116 | 6 | 442 | 459 | AIPTCTVFYPSDGVSTER | 138 | 100 | Carbamidomethyl (C)[5] | | Mascot |
| 1999.943 | 1999.9546 | 0.0116 | 6 | 442 | 459 | AIPTCTVFYPSDGVSTER | | | Carbamidomethyl (C)[5] | | Mascot |
| 2114.1855 | 2114.1997 | 0.0142 | 7 | 534 | 553 | VIDPFTIKPLDASTIVASA R | | | | | Mascot |
| 2114.1855 | 2114.1997 | 0.0142 | 7 | 534 | 553 | VIDPFTIKPLDASTIVASA R | 138 | 100 | | | Mascot |
| 2175.1404 | 2175.1536 | 0.0132 | 6 | 568 | 589 | EGGLGEAVLSAVGEEPGI VVHR | 222 | 100 | | | Mascot |
| 2175.1404 | 2175.1536 | 0.0132 | 6 | 568 | 589 | EGGLGEAVLSAVGEEPGI VVHR | | | | | Mascot |
| 2294.1663 | 2294.178 | 0.0117 | 5 | 475 | 494 | TSRPDTAVIYNPEEKFEIG K | | | | | Mascot |

| Calc. Mass | Obsrv. Mass | ± da | ± ppm | Start Seq. | End Seq. | Sequence | Ion Score | C. I. % | Modification | Rank | Result Type |
|---|---|---|---|---|---|---|---|---|---|---|---|
| 2432.1406 | 2432.1536 | 0.013 | 5 | 362 | 382 | YIECFIAEQNMVSVAIGCATR | 160 | 100 | Carbamidomethyl (C)[4,18] | | Mascot |
| 2432.1406 | 2432.1536 | 0.013 | 5 | 362 | 382 | YIECFIAEQNMVSVAIGCATR | | | Carbamidomethyl (C)[4,18] | | Mascot |
| 2448.1355 | 2448.1565 | 0.021 | 9 | 362 | 382 | YIECFIAEQNMVSVAIGCATR | | | Carbamidomethyl (C)[4,18], Oxidation (M)[11] | | Mascot |
| 2457.3096 | 2457.3267 | 0.0171 | 7 | 504 | 527 | DQVTVIGAGVTLHEALAAHDQLAK | 120 | 100 | | | Mascot |
| 2457.3096 | 2457.3267 | 0.0171 | 7 | 504 | 527 | DQVTVIGAGVTLHEALAAHDQLAK | | | | | Mascot |
| 2489.0747 | 2489.1013 | 0.0266 | 11 | 205 | 225 | CEAFGFNTYVVDGHDVEELCK | | | Carbamidomethyl (C)[1,20] | | Mascot |
| 2515.2319 | 2515.2617 | 0.0298 | 12 | 114 | 139 | LAFVDVATGSLGQGLGAACGMAYTGK | | | Carbamidomethyl (C)[19] | | Mascot |
| 2531.2268 | 2531.2527 | 0.0259 | 10 | 114 | 139 | LAFVDVATGSLGQGLGAACGMAYTGK | | | Carbamidomethyl (C)[19], Oxidation (M)[21] | | Mascot |
| 3103.6423 | 3103.6582 | 0.0159 | 5 | 267 | 293 | DRAEELINDLLSQIQSPNKPLYPQPPK | | | | | Mascot |

2 Tkt protein [Danio rerio] gi|182890016 67962.5 6.79 22 1,310 100 1158 100

**Peptide Information**

| Calc. Mass | Obsrv. Mass | ± da | ± ppm | Start Seq. | End Seq. | Sequence | Ion Score | C. I. % | Modification | Rank | Result Type |
|---|---|---|---|---|---|---|---|---|---|---|---|
| 864.421 | 864.4554 | 0.0344 | 40 | 57 | 63 | YKADDPR | | | | | Mascot |
| 959.5156 | 959.5181 | 0.0025 | 3 | 93 | 100 | ESDLLNLR | | | | | Mascot |
| 1060.4768 | 1060.4849 | 0.0081 | 8 | 347 | 355 | NSTFADMFK | | | | | Mascot |
| 1076.4717 | 1076.4808 | 0.0091 | 8 | 347 | 355 | NSTFADMFK | | | Oxidation (M)[7] | | Mascot |
| 1103.5731 | 1103.5774 | 0.0043 | 4 | 559 | 567 | VITVEDHYK | | | | | Mascot |
| 1112.5081 | 1112.5123 | 0.0042 | 4 | 226 | 234 | YMWEAEGVK | | | | | Mascot |
| 1128.5031 | 1128.5116 | 0.0085 | 8 | 226 | 234 | YMWEAEGVK | | | Oxidation (M)[2] | | Mascot |
| 1354.7689 | 1354.7772 | 0.0083 | 6 | 174 | 185 | LDNLVAILDVNR | 108 | 100 | | | Mascot |
| 1354.7689 | 1354.7772 | 0.0083 | 6 | 174 | 185 | LDNLVAILDVNR | | | | | Mascot |
| 1436.738 | 1436.7474 | 0.0094 | 7 | 101 | 113 | KIDSDLEGHPTPK | | | | | Mascot |
| 1488.7845 | 1488.793 | 0.0085 | 6 | 385 | 398 | TVSFASTFAAFLAR | | | | | Mascot |
| 1488.7845 | 1488.793 | 0.0085 | 6 | 385 | 398 | TVSFASTFAAFLAR | 123 | 100 | | | Mascot |
| 1719.8547 | 1719.8651 | 0.0104 | 6 | 475 | 489 | TSRPDTAVIYNPEEK | | | | | Mascot |
| 1719.8547 | 1719.8651 | 0.0104 | 6 | 475 | 489 | TSRPDTAVIYNPEEK | 67 | 99.994 | | | Mascot |
| 1736.8887 | 1736.8948 | 0.0061 | 4 | 598 | 613 | SGKPQELLDMFGISAK | | | Oxidation (M)[10] | | Mascot |
| 1751.817 | 1751.8267 | 0.0097 | 6 | 252 | 266 | GIEDQDNWHGKPMPK | | | | | Mascot |
| 1767.8119 | 1767.8225 | 0.0106 | 6 | 252 | 266 | GIEDQDNWHGKPMPK | | | Oxidation (M)[13] | | Mascot |
| 1893.9817 | 1893.9919 | 0.0102 | 5 | 186 | 202 | LGQSEPAPLQHNVNVYK | 83 | 100 | | | Mascot |
| 1893.9817 | 1893.9919 | 0.0102 | 5 | 186 | 202 | LGQSEPAPLQHNVNVYK | | | | | Mascot |
| 1901.9908 | 1902.0004 | 0.0096 | 5 | 75 | 92 | GHAAPILYAAWAEAGYIK | | | | | Mascot |
| 1994.0415 | 1993.9897 | -0.0518 | -26 | 226 | 243 | YMWEAEGVKGKPTAIVA | | | Oxidation (M)[2] | | Mascot |

| | | | | | | | | | | | |
|---|---|---|---|---|---|---|---|---|---|---|---|
| | | | | | | K | | | | | |
| 1999.943 | 1999.9546 | 0.0116 | 6 | 442 | 459 | AIPTCTVFYPSDGVSTER | 138 | 100 | Carbamidomethyl (C)[5] | | Mascot |
| 1999.943 | 1999.9546 | 0.0116 | 6 | 442 | 459 | AIPTCTVFYPSDGVSTER | | | Carbamidomethyl (C)[5] | | Mascot |
| 2114.1855 | 2114.1997 | 0.0142 | 7 | 534 | 553 | VIDPFTIKPLDASTIVASA R | | | | | Mascot |
| 2114.1855 | 2114.1997 | 0.0142 | 7 | 534 | 553 | VIDPFTIKPLDASTIVASA R | 138 | 100 | | | Mascot |
| 2175.1404 | 2175.1536 | 0.0132 | 6 | 568 | 589 | EGGLGEAVLSAVGEEPGI VVHR | 222 | 100 | | | Mascot |
| 2175.1404 | 2175.1536 | 0.0132 | 6 | 568 | 589 | EGGLGEAVLSAVGEEPGI VVHR | | | | | Mascot |
| 2294.1663 | 2294.178 | 0.0117 | 5 | 475 | 494 | TSRPDTAVIYNPEEKFEIG K | | | | | Mascot |
| 2432.1406 | 2432.1536 | 0.013 | 5 | 362 | 382 | YIECFIAEQNMVSVAIGCA TR | 160 | 100 | Carbamidomethyl (C)[4,18] | | Mascot |
| 2432.1406 | 2432.1536 | 0.013 | 5 | 362 | 382 | YIECFIAEQNMVSVAIGCA TR | | | Carbamidomethyl (C)[4,18] | | Mascot |
| 2448.1355 | 2448.1565 | 0.021 | 9 | 362 | 382 | YIECFIAEQNMVSVAIGCA TR | | | Carbamidomethyl (C)[4,18], Oxidation (M)[11] | | Mascot |
| 2457.3096 | 2457.3267 | 0.0171 | 7 | 504 | 527 | DQVTVIGAGVTLHEALAA HDQLAK | 120 | 100 | | | Mascot |
| 2457.3096 | 2457.3267 | 0.0171 | 7 | 504 | 527 | DQVTVIGAGVTLHEALAA HDQLAK | | | | | Mascot |
| 2515.2319 | 2515.2617 | 0.0298 | 12 | 114 | 139 | LAFVDVATGSLGQGLGA ACGMAYTGK | | | Carbamidomethyl (C)[19] | | Mascot |
| 2531.2268 | 2531.2527 | 0.0259 | 10 | 114 | 139 | LAFVDVATGSLGQGLGA ACGMAYTGK | | | Carbamidomethyl (C)[19], Oxidation (M)[21] | | Mascot |
| 3103.6423 | 3103.6582 | 0.0159 | 5 | 267 | 293 | DRAEELINDLLSQIQSPN KPLYPQPPK | | | | | Mascot |

| | | | | | | | | | | |
|---|---|---|---|---|---|---|---|---|---|---|
| 3 | transketolase [Danio rerio] | gi\|31872040 | 67811.3 | 6.81 | 20 | 1,200 | 100 | 1070 | 100 | |

**Peptide Information**

| Calc. Mass | Obsrv. Mass | ± da | ± ppm | Start Seq. | End Seq. | Sequence | Ion Score | C. I. % | Modification | Rank | Result Type |
|---|---|---|---|---|---|---|---|---|---|---|---|
| 901.5101 | 901.5135 | 0.0034 | 4 | 93 | 100 | ASDLLNLR | 54 | 99.889 | | | Mascot |
| 901.5101 | 901.5135 | 0.0034 | 4 | 93 | 100 | ASDLLNLR | | | | | Mascot |
| 1028.4982 | 1028.5013 | 0.0031 | 3 | 226 | 234 | AMWHAEGVK | | | | | Mascot |
| 1060.4768 | 1060.4849 | 0.0081 | 8 | 347 | 355 | NSTFADMFK | | | | | Mascot |
| 1076.4717 | 1076.4808 | 0.0091 | 8 | 347 | 355 | NSTFADMFK | | | Oxidation (M)[7] | | Mascot |
| 1103.5731 | 1103.5774 | 0.0043 | 4 | 559 | 567 | VITVEDHYK | | | | | Mascot |
| 1354.7689 | 1354.7772 | 0.0083 | 6 | 174 | 185 | LDNLVAILDVNR | 108 | 100 | | | Mascot |
| 1354.7689 | 1354.7772 | 0.0083 | 6 | 174 | 185 | LDNLVAILDVNR | | | | | Mascot |
| 1436.738 | 1436.7474 | 0.0094 | 7 | 101 | 113 | KIDSDLEGHPTPK | | | | | Mascot |
| 1488.7845 | 1488.793 | 0.0085 | 6 | 385 | 398 | TVSFASTFAAFLAR | | | | | Mascot |
| 1488.7845 | 1488.793 | 0.0085 | 6 | 385 | 398 | TVSFASTFAAFLAR | 123 | 100 | | | Mascot |
| 1719.8547 | 1719.8651 | 0.0104 | 6 | 475 | 489 | TSRPDTAVIYNPEEK | | | | | Mascot |
| 1719.8547 | 1719.8651 | 0.0104 | 6 | 475 | 489 | TSRPDTAVIYNPEEK | 67 | 99.994 | | | Mascot |
| 1736.8887 | 1736.8948 | 0.0061 | 4 | 598 | 613 | SGKPQELLDMFGISAK | | | Oxidation (M)[10] | | Mascot |

| | | | | | | | | | | | |
|---|---|---|---|---|---|---|---|---|---|---|---|
| 1751.817 | 1751.8267 | 0.0097 | 6 | 252 | 266 | GIEDQDNWHGKPMPK | | | | | Mascot |
| 1767.8119 | 1767.8225 | 0.0106 | 6 | 252 | 266 | GIEDQDNWHGKPMPK | | | Oxidation (M)[13] | | Mascot |
| 1893.9817 | 1893.9919 | 0.0102 | 5 | 186 | 202 | LGQSEPAPLQHNVNVYK | 83 | 100 | | | Mascot |
| 1893.9817 | 1893.9919 | 0.0102 | 5 | 186 | 202 | LGQSEPAPLQHNVNVYK | | | | | Mascot |
| 1901.9908 | 1902.0004 | 0.0096 | 5 | 75 | 92 | GHAAPILYAAWAEAGYIK | | | | | Mascot |
| 2114.1855 | 2114.1997 | 0.0142 | 7 | 534 | 553 | VIDPFTIKPLDASTIVASA R | | | | | Mascot |
| 2114.1855 | 2114.1997 | 0.0142 | 7 | 534 | 553 | VIDPFTIKPLDASTIVASA R | 138 | 100 | | | Mascot |
| 2175.1404 | 2175.1536 | 0.0132 | 6 | 568 | 589 | EGGLGEAVLSAVGEEPGI VVHR | 222 | 100 | | | Mascot |
| 2175.1404 | 2175.1536 | 0.0132 | 6 | 568 | 589 | EGGLGEAVLSAVGEEPGI VVHR | | | | | Mascot |
| 2294.1663 | 2294.178 | 0.0117 | 5 | 475 | 494 | TSRPDTAVIYNPEEKFEIG K | | | | | Mascot |
| 2432.1406 | 2432.1536 | 0.013 | 5 | 362 | 382 | YIECFIAEQNMVSVAIGCA TR | 160 | 100 | Carbamidomethyl (C)[4,18] | | Mascot |
| 2432.1406 | 2432.1536 | 0.013 | 5 | 362 | 382 | YIECFIAEQNMVSVAIGCA TR | | | Carbamidomethyl (C)[4,18] | | Mascot |
| 2448.1355 | 2448.1565 | 0.021 | 9 | 362 | 382 | YIECFIAEQNMVSVAIGCA TR | | | Carbamidomethyl (C)[4,18], Oxidation (M)[11] | | Mascot |
| 2457.3096 | 2457.3267 | 0.0171 | 7 | 504 | 527 | DQVTVIGAGVTLHEALAA HDQLAK | 120 | 100 | | | Mascot |
| 2457.3096 | 2457.3267 | 0.0171 | 7 | 504 | 527 | DQVTVIGAGVTLHEALAA HDQLAK | | | | | Mascot |
| 2489.0747 | 2489.1013 | 0.0266 | 11 | 205 | 225 | CEAFGFNTYVVDGHDVE ELCK | | | Carbamidomethyl (C)[1,20] | | Mascot |
| 2515.2319 | 2515.2617 | 0.0298 | 12 | 114 | 139 | LAFVDVATGSLGQGLGA ACGMAYTGK | | | Carbamidomethyl (C)[19] | | Mascot |
| 2531.2268 | 2531.2527 | 0.0259 | 10 | 114 | 139 | LAFVDVATGSLGQGLGA ACGMAYTGK | | | Carbamidomethyl (C)[19], Oxidation (M)[21] | | Mascot |
| 3103.6423 | 3103.6582 | 0.0159 | 5 | 267 | 293 | DRAEELINDLLSQIQSPN KPLYPQPPK | | | | | Mascot |

4 novel protein similar to vertebrate pim oncogene family [Danio rerio] gi|55962882 32334.2 8.77 8 53 79.659

**Peptide Information**

| Calc. Mass | Obsrv. Mass | ± da | ± ppm | Start Seq. | End Seq. | Sequence | Ion Score | C. I. % | Modification | Rank | Result Type |
|---|---|---|---|---|---|---|---|---|---|---|---|
| 901.5464 | 901.5135 | -0.0329 | -36 | 49 | 56 | IVDSKALR | 15 | 0 | | | Mascot |
| 901.5464 | 901.5135 | -0.0329 | -36 | 49 | 56 | IVDSKALR | | | | | Mascot |
| 959.5342 | 959.5181 | -0.0161 | -17 | 106 | 113 | LIPSMNLR | | | Oxidation (M)[5] | | Mascot |
| 1342.7729 | 1342.6414 | -0.1315 | -98 | 83 | 93 | VKQIAELLDWK | | | | | Mascot |
| 1365.7446 | 1365.6426 | -0.102 | -75 | 168 | 179 | LIDFGCSVLLTK | | | Carbamidomethyl (C)[6] | | Mascot |
| 1475.8217 | 1475.7635 | -0.0582 | -39 | 94 | 105 | VEAERYIIVVER | | | | | Mascot |
| 1707.8127 | 1707.7886 | -0.0241 | -14 | 131 | 145 | IMFNVTIAAHTCCQR | | | | | Mascot |
| 1908.9241 | 1908.9456 | 0.0215 | 11 | 130 | 145 | KIMFNVTIAAHTCCQR | | | Carbamidomethyl (C)[13], Oxidation (M)[3] | | Mascot |
| 2432.3376 | 2432.1536 | -0.184 | -76 | 202 | 222 | YHGEPATVWSLGILQFLL | | | | | Mascot |

| | | | | | | | | | | | |
|---|---|---|---|---|---|---|---|---|---|---|---|
| | | | | | | LFK | | | | | |
| 2432.3376 | 2432.1536 | -0.184 | -76 | 202 | 222 | YHGEPATVWSLGILQFLL LFK | | | | | Mascot |

5 novel protein similar to vertebrate pim oncogene family [Danio rerio] gi|55962348 32151.9 6.97 8 53 79.185

**Peptide Information**

| Calc. Mass | Obsrv. Mass | ± da | ± ppm | Start Seq. | End Seq. | Sequence | Ion Score | C. I. % | Modification | Rank | Result Type |
|---|---|---|---|---|---|---|---|---|---|---|---|
| 901.5464 | 901.5135 | -0.0329 | -36 | 49 | 56 | IVDSKALR | 15 | 0 | | | Mascot |
| 901.5464 | 901.5135 | -0.0329 | -36 | 49 | 56 | IVDSKALR | | | | | Mascot |
| 959.5342 | 959.5181 | -0.0161 | -17 | 106 | 113 | LIPSMNLR | | | Oxidation (M)[5] | | Mascot |
| 1342.7729 | 1342.6414 | -0.1315 | -98 | 83 | 93 | VKQIAELLDWK | | | | | Mascot |
| 1365.7446 | 1365.6426 | -0.102 | -75 | 168 | 179 | LIDFGCSVLLTK | | | Carbamidomethyl (C)[6] | | Mascot |
| 1475.8217 | 1475.7635 | -0.0582 | -39 | 94 | 105 | VEAERYIIVVER | | | | | Mascot |
| 1707.8127 | 1707.7886 | -0.0241 | -14 | 131 | 145 | IMFNVTIAAHTCCQR | | | | | Mascot |
| 1908.9241 | 1908.9456 | 0.0215 | 11 | 130 | 145 | KIMFNVTIAAHTCCQR | | | Carbamidomethyl (C)[13], Oxidation (M)[3] | | Mascot |
| 2432.3376 | 2432.1536 | -0.184 | -76 | 202 | 222 | YHGEPATVWSLGILQFLL LFK | | | | | Mascot |
| 2432.3376 | 2432.1536 | -0.184 | -76 | 202 | 222 | YHGEPATVWSLGILQFLL LFK | | | | | Mascot |

6 Si:dkey-180p18.9 protein [Danio rerio] gi|63101286 38863 6.56 6 50 59.415 28 54.216

**Peptide Information**

| Calc. Mass | Obsrv. Mass | ± da | ± ppm | Start Seq. | End Seq. | Sequence | Ion Score | C. I. % | Modification | Rank | Result Type |
|---|---|---|---|---|---|---|---|---|---|---|---|
| 850.4781 | 850.5455 | 0.0674 | 79 | 318 | 324 | TILSFNR | | | | | Mascot |
| 901.5465 | 901.5135 | -0.033 | -37 | 274 | 281 | TTAQVLIR | 28 | 54.216 | | | Mascot |
| 901.5465 | 901.5135 | -0.033 | -37 | 274 | 281 | TTAQVLIR | | | | | Mascot |
| 1028.4805 | 1028.5013 | 0.0208 | 20 | 328 | 336 | ACPMHWGVK | | | | | Mascot |
| 1112.631 | 1112.5123 | -0.1187 | -107 | 53 | 63 | SPPGKVTEAVK | | | | | Mascot |
| 1179.6078 | 1179.6071 | -0.0007 | -1 | 11 | 21 | FCTEPGSVVLK | | | | | Mascot |
| 2531.2883 | 2531.2527 | -0.0356 | -14 | 11 | 33 | FCTEPGSVVLKSSVLNLS FTMSK | | | Carbamidomethyl (C)[2] | | Mascot |

7 hypothetical protein LOC553738 [Danio rerio] gi|66472320 27546.3 9.57 11 46 0.375

**Peptide Information**

| Calc. Mass | Obsrv. Mass | ± da | ± ppm | Start Seq. | End Seq. | Sequence | Ion Score | C. I. % | Modification | Rank | Result Type |
|---|---|---|---|---|---|---|---|---|---|---|---|
| 1128.4561 | 1128.5116 | 0.0555 | 49 | 132 | 141 | CDQCGQTFAR | | | | | Mascot |
| 1165.6212 | 1165.7528 | 0.1316 | 113 | 194 | 203 | TFVTADDLKR | | | | | Mascot |

| | | | | | | | | | | | |
|---|---|---|---|---|---|---|---|---|---|---|---|
| 1179.615 | 1179.6071 | -0.0079 | -7 | 166 | 175 | SCTRQSSLIK | | | Carbamidomethyl (C)[2] | | Mascot |
| 1365.6501 | 1365.6426 | -0.0075 | -5 | 13 | 25 | TGEKPITCTQCGK | | | | | Mascot |
| 1736.7843 | 1736.8948 | 0.1105 | 64 | 151 | 165 | VHTNERPYSCSVCGK | | | Carbamidomethyl (C)[10] | | Mascot |
| 1751.8601 | 1751.8267 | -0.0334 | -19 | 10 | 25 | MVRTGEKPITCTQCGK | | | | | Mascot |
| 1767.855 | 1767.8225 | -0.0325 | -18 | 10 | 25 | MVRTGEKPITCTQCGK | | | Oxidation (M)[1] | | Mascot |
| 1862.7982 | 1863.073 | 0.2748 | 148 | 207 | 221 | IHTGEKPYMCSHCDK | | | Carbamidomethyl (C)[10,13] | | Mascot |
| 2113.9568 | 2114.1997 | 0.2429 | 115 | 185 | 202 | EHVCFECEKTFVTADDLK | | | | | Mascot |
| 2113.9568 | 2114.1997 | 0.2429 | 115 | 185 | 202 | EHVCFECEKTFVTADDLK | | | | | Mascot |
| 2233.0752 | 2233.105 | 0.0298 | 13 | 147 | 165 | FHLRVHTNERPYSCSVCGK | | | | | Mascot |
| 2284.0168 | 2284.1877 | 0.1709 | 75 | 204 | 221 | HQRIHTGEKPYMCSHCDK | | | Carbamidomethyl (C)[13,16] | | Mascot |
| 2298.0171 | 2298.198 | 0.1809 | 79 | 151 | 169 | VHTNERPYSCSVCGKSCTR | | | Carbamidomethyl (C)[10,13,17] | | Mascot |

| | | | | | | | | | | |
|---|---|---|---|---|---|---|---|---|---|---|
| 8 | cutC copper transporter homolog [Danio rerio] | gi\|54400594 | 27229.9 | 5.63 | 6 | 46 | 0 | | | |

**Peptide Information**

| Calc. Mass | Obsrv. Mass | ± da | ± ppm | Start Seq. | End Seq. | Sequence | Ion Score | C. I. % | Modification | Rank | Result Type |
|---|---|---|---|---|---|---|---|---|---|---|---|
| 870.4138 | 870.5441 | 0.1303 | 150 | 209 | 215 | DSSMKFR | | | | | Mascot |
| 901.5464 | 901.5135 | -0.0329 | -36 | 234 | 241 | VADVTKIR | 16 | 0 | | | Mascot |
| 901.5464 | 901.5135 | -0.0329 | -36 | 234 | 241 | VADVTKIR | | | | | Mascot |
| 1028.6099 | 1028.5013 | -0.1086 | -106 | 242 | 251 | ALNAIAKNTL | | | | | Mascot |
| 2294.064 | 2294.178 | 0.114 | 50 | 2 | 22 | SDGLLMEVCVDSVESAINAER | | | Carbamidomethyl (C)[9] | | Mascot |
| 2384.0779 | 2384.0769 | -0.001 | 0 | 1 | 22 | MSDGLLMEVCVDSVESAINAER | | | Oxidation (M)[1] | | Mascot |
| 2457.0942 | 2457.3267 | 0.2325 | 95 | 1 | 22 | MSDGLLMEVCVDSVESAINAER | | | Carbamidomethyl (C)[10], Oxidation (M)[1,7] | | Mascot |
| 2457.0942 | 2457.3267 | 0.2325 | 95 | 1 | 22 | MSDGLLMEVCVDSVESAINAER | | | Carbamidomethyl (C)[10], Oxidation (M)[1,7] | | Mascot |
| 2489.2744 | 2489.1013 | -0.1731 | -70 | 126 | 147 | AFDMVHDPLVALETLISLGFER | | | Oxidation (M)[4] | | Mascot |

| | | | | | | | | | | |
|---|---|---|---|---|---|---|---|---|---|---|
| 9 | PREDICTED: polyphosphoinositide phosphatase [Danio rerio] | gi\|326681249 | 37784.5 | 8.99 | 3 | 45 | 0 | 37 | 94.223 | |

**Peptide Information**

| Calc. Mass | Obsrv. Mass | ± da | ± ppm | Start Seq. | End Seq. | Sequence | Ion Score | C. I. % | Modification | Rank | Result Type |
|---|---|---|---|---|---|---|---|---|---|---|---|
| 850.457 | 850.5455 | 0.0885 | 104 | 293 | 298 | YVWNLR | | | | | Mascot |
| 901.4559 | 901.5135 | 0.0576 | 64 | 109 | 115 | CLPENLR | 37 | 94.223 | Carbamidomethyl (C)[1] | | Mascot |
| 901.4559 | 901.5135 | 0.0576 | 64 | 109 | 115 | CLPENLR | | | Carbamidomethyl (C)[1] | | Mascot |
| 1342.6862 | 1342.6414 | -0.0448 | -33 | 211 | 221 | VTHPDEARYVR | | | | | Mascot |

| | | | | | | | | | | |
|---|---|---|---|---|---|---|---|---|---|---|
| 10 | protein BTG3 [Danio rerio] | gi\|55925203 | 18115.1 | 5.85 | 7 | 45 | 0 | | | |

Peptide Information

| Calc. Mass | Obsrv. Mass | ± da | ± ppm | Start Seq. | End Seq. | Sequence | Ion Score | C. I. % | Modification | Rank | Result Type |
|---|---|---|---|---|---|---|---|---|---|---|---|
| 901.5353 | 901.5135 | -0.0218 | -24 | 34 | 41 | LTVALQEK | | | | | Mascot |
| 901.5353 | 901.5135 | -0.0218 | -24 | 34 | 41 | LTVALQEK | | | | | Mascot |
| 1112.504 | 1112.5123 | 0.0083 | 7 | 75 | 84 | ACAESGVQYK | | | Carbamidomethyl (C)[2] | | Mascot |
| 1735.8683 | 1735.8551 | -0.0132 | -8 | 75 | 90 | ACAESGVQYKDLGLPK | | | Carbamidomethyl (C)[2] | | Mascot |
| 2211.0208 | 2211.1179 | 0.0971 | 44 | 91 | 108 | ELTLWVDPGEVCCRYGE K | | | Carbamidomethyl (C)[12,13] | | Mascot |
| 2224.9412 | 2225.1333 | 0.1921 | 86 | 139 | 158 | VTSDYHSGSSSDEDSML VHR | | | Oxidation (M)[16] | | Mascot |
| 2357.1628 | 2357.1431 | -0.0197 | -8 | 85 | 104 | DLGLPKELTLWVDPGEV CCR | | | Carbamidomethyl (C)[18,19] | | Mascot |
| 2457.0688 | 2457.3267 | 0.2579 | 105 | 109 | 130 | NHGFTVATFSSEDDDDK EDVTK | | | | | Mascot |
| 2457.0688 | 2457.3267 | 0.2579 | 105 | 109 | 130 | NHGFTVATFSSEDDDDK EDVTK | | | | | Mascot |

11 isobutyryl-CoA dehydrogenase, mitochondrial [Danio rerio] gi|41055941 45197.7 8.33 6 45 0

Peptide Information

| Calc. Mass | Obsrv. Mass | ± da | ± ppm | Start Seq. | End Seq. | Sequence | Ion Score | C. I. % | Modification | Rank | Result Type |
|---|---|---|---|---|---|---|---|---|---|---|---|
| 901.54 | 901.5135 | -0.0265 | -29 | 1 | 8 | MAARALIR | 27 | 52.278 | | | Mascot |
| 901.54 | 901.5135 | -0.0265 | -29 | 1 | 8 | MAARALIR | | | | | Mascot |
| 1103.548 | 1103.5774 | 0.0294 | 27 | 182 | 191 | GDHYILNGSK | | | | | Mascot |
| 1354.6671 | 1354.7772 | 0.1101 | 81 | 261 | 273 | LGTEGQGFSIAMK | | | Oxidation (M)[12] | | Mascot |
| 1354.6671 | 1354.7772 | 0.1101 | 81 | 261 | 273 | LGTEGQGFSIAMK | | | Oxidation (M)[12] | | Mascot |
| 1862.8411 | 1863.073 | 0.2319 | 124 | 192 | 208 | AFISGGGDTDVYVVMCR | | | Carbamidomethyl (C)[16], Oxidation (M)[15] | | Mascot |
| 1908.9596 | 1908.9456 | -0.014 | -7 | 261 | 279 | LGTEGQGFSIAMKGLNG GR | | | Oxidation (M)[12] | | Mascot |
| 1924.0432 | 1923.98 | -0.0632 | -33 | 280 | 298 | INIASCSLGAAHASVLLAR | | | Carbamidomethyl (C)[6] | | Mascot |

12 Ewing tumor-associated antigen 1 [Danio rerio] gi|162139038 71948.4 6.84 12 43 0

Peptide Information

| Calc. Mass | Obsrv. Mass | ± da | ± ppm | Start Seq. | End Seq. | Sequence | Ion Score | C. I. % | Modification | Rank | Result Type |
|---|---|---|---|---|---|---|---|---|---|---|---|
| 996.5836 | 996.6034 | 0.0198 | 20 | 28 | 37 | SPKAAAPLNK | | | | | Mascot |
| 1103.5327 | 1103.5774 | 0.0447 | 41 | 5 | 14 | GSQEQNEIAK | | | | | Mascot |
| 1210.7054 | 1210.6071 | -0.0983 | -81 | 592 | 601 | FKKPHGELVR | | | | | Mascot |
| 1475.7085 | 1475.7635 | 0.055 | 37 | 2 | 14 | ESRGSQEQNEIAK | | | | | Mascot |

| Calc. Mass | Obsrv. Mass | ± da | ± ppm | Start Seq. | End Seq. | Sequence | Ion Score | C. I. % | Modification | Rank | Result Type |
|---|---|---|---|---|---|---|---|---|---|---|---|
| 1488.7917 | 1488.793 | 0.0013 | 1 | 38 | 50 | NTAEFKTPTRPAR | | | | | Mascot |
| 1488.7917 | 1488.793 | 0.0013 | 1 | 38 | 50 | NTAEFKTPTRPAR | | | | | Mascot |
| 1862.9071 | 1863.073 | 0.1659 | 89 | 489 | 505 | SFATSENAVFAHPYPPK | | | | | Mascot |
| 1880.9573 | 1880.9967 | 0.0394 | 21 | 575 | 591 | GVLAVIQSEARDTDNHR | | | | | Mascot |
| 1994.0526 | 1993.9897 | -0.0629 | -32 | 334 | 351 | TKVSQNNSQIQINGGLHR | | | | | Mascot |
| 2197.1216 | 2197.137 | 0.0154 | 7 | 314 | 333 | NAVSVMTNKPCEAKNVG LHK | | | Carbamidomethyl (C)[11] | | Mascot |
| 2211.155 | 2211.1179 | -0.0371 | -17 | 413 | 432 | NAIKPSQSKPSSMHQNLI SK | | | Oxidation (M)[13] | | Mascot |
| 2384.0354 | 2384.0769 | 0.0415 | 17 | 524 | 548 | AAGPGCNVSCDAARPSG SSSNGAYK | | | Carbamidomethyl (C)[6] | | Mascot |
| 2479.3198 | 2479.3062 | -0.0136 | -5 | 413 | 434 | NAIKPSQSKPSSMHQNLI SKQR | | | | | Mascot |

13 PREDICTED: UDP-GlcNAc:betaGal beta-1,3-N-acetylglucosaminyltransferase 2 [Danio rerio] gi|125843963 48322.6 8.69 10 41 0

**Peptide Information**

| Calc. Mass | Obsrv. Mass | ± da | ± ppm | Start Seq. | End Seq. | Sequence | Ion Score | C. I. % | Modification | Rank | Result Type |
|---|---|---|---|---|---|---|---|---|---|---|---|
| 864.411 | 864.4554 | 0.0444 | 51 | 84 | 90 | NGAFWNR | | | | | Mascot |
| 1028.3846 | 1028.5013 | 0.1167 | 113 | 412 | 420 | SMHSSMLTC | | | Oxidation (M)[2,6] | | Mascot |
| 1103.5215 | 1103.5774 | 0.0559 | 51 | 210 | 219 | SSLDDPNLDK | | | | | Mascot |
| 1165.5847 | 1165.7528 | 0.1681 | 144 | 193 | 202 | EGEYDGLKVR | | | | | Mascot |
| 1179.5753 | 1179.6071 | 0.0318 | 27 | 378 | 386 | TFDIREQDR | | | | | Mascot |
| 1354.6168 | 1354.7772 | 0.1604 | 118 | 383 | 393 | EQDRENPCVHK | | | | | Mascot |
| 1354.6168 | 1354.7772 | 0.1604 | 118 | 383 | 393 | EQDRENPCVHK | | | | | Mascot |
| 1436.6879 | 1436.7474 | 0.0595 | 41 | 112 | 123 | FHCSPESFELLK | | | | | Mascot |
| 1736.8763 | 1736.8948 | 0.0185 | 11 | 253 | 266 | WMLGHCPRVSFIFK | | | Oxidation (M)[2] | | Mascot |
| 1751.9486 | 1751.8267 | -0.1219 | -70 | 387 | 400 | ENPCVHKHLLLVHR | | | Carbamidomethyl (C)[4] | | Mascot |
| 2457.3323 | 2457.3267 | -0.0056 | -2 | 5 | 25 | YMKFLIVALASSLCFILFY SK | | | | | Mascot |
| 2457.3323 | 2457.3267 | -0.0056 | -2 | 5 | 25 | YMKFLIVALASSLCFILFY SK | | | | | Mascot |

14 Si:dkey-180p18.9 protein [Danio rerio] gi|50604191 36662.8 6.44 4 40 0 28 54.216

**Peptide Information**

| Calc. Mass | Obsrv. Mass | ± da | ± ppm | Start Seq. | End Seq. | Sequence | Ion Score | C. I. % | Modification | Rank | Result Type |
|---|---|---|---|---|---|---|---|---|---|---|---|
| 850.4781 | 850.5455 | 0.0674 | 79 | 299 | 305 | TILSFNR | | | | | Mascot |
| 901.5465 | 901.5135 | -0.033 | -37 | 255 | 262 | TTAQVLIR | 28 | 54.216 | | | Mascot |
| 901.5465 | 901.5135 | -0.033 | -37 | 255 | 262 | TTAQVLIR | | | | | Mascot |
| 1028.4805 | 1028.5013 | 0.0208 | 20 | 309 | 317 | ACPMHWGVK | | | | | Mascot |

| | | | | | | | | | | | |
|---|---|---|---|---|---|---|---|---|---|---|---|
| 1112.631 | 1112.5123 | -0.1187 | -107 | 34 | 44 | SPPGKVTEAVK | | | | | Mascot |

| 15 | novel protein similar to vertebrate stabilin 2 (STAB2) [Danio rerio] | gi\|148725539 | 264800.5 | 6.26 | 16 | 40 | 0 | 30 | 73.04 |
|---|---|---|---|---|---|---|---|---|---|

**Peptide Information**

| Calc. Mass | Obsrv. Mass | ± da | ± ppm | Start Seq. | End Seq. | Sequence | Ion Score | C. I. % | Modification | Rank | Result Type |
|---|---|---|---|---|---|---|---|---|---|---|---|
| 901.5464 | 901.5135 | -0.0329 | -36 | 1587 | 1594 | ASIDLVKR | 30 | 73.04 | | | Mascot |
| 901.5464 | 901.5135 | -0.0329 | -36 | 1587 | 1594 | ASIDLVKR | | | | | Mascot |
| 1045.5789 | 1045.5679 | -0.011 | -11 | 1756 | 1763 | YHILRDTK | | | | | Mascot |
| 1103.472 | 1103.5774 | 0.1054 | 96 | 1437 | 1447 | NGGCSAHAVCK | | | Carbamidomethyl (C)[4] | | Mascot |
| 1179.6117 | 1179.6071 | -0.0046 | -4 | 1557 | 1565 | NRIYELDTR | | | | | Mascot |
| 1210.5885 | 1210.6071 | 0.0186 | 15 | 1837 | 1846 | QTIDFTVACR | | | Carbamidomethyl (C)[9] | | Mascot |
| 1342.6942 | 1342.6414 | -0.0528 | -39 | 2435 | 2444 | TKPFQFHYFK | | | | | Mascot |
| 1354.6783 | 1354.7772 | 0.0989 | 73 | 1259 | 1270 | CDKATSILFSGR | | | Carbamidomethyl (C)[1] | | Mascot |
| 1354.6783 | 1354.7772 | 0.0989 | 73 | 1259 | 1270 | CDKATSILFSGR | | | Carbamidomethyl (C)[1] | | Mascot |
| 1475.7059 | 1475.7635 | 0.0576 | 39 | 2208 | 2221 | CGSGHVGIVDYGVR | | | Carbamidomethyl (C)[1] | | Mascot |
| 1488.6896 | 1488.793 | 0.1034 | 69 | 662 | 674 | YMAPCVSCTLISK | | | Carbamidomethyl (C)[5], Oxidation (M)[2] | | Mascot |
| 1488.6896 | 1488.793 | 0.1034 | 69 | 662 | 674 | YMAPCVSCTLISK | | | Carbamidomethyl (C)[5], Oxidation (M)[2] | | Mascot |
| 1542.6675 | 1542.8931 | 0.2256 | 146 | 147 | 159 | TDLYGPTCSNVCR | | | Carbamidomethyl (C)[8,12] | | Mascot |
| 1736.9065 | 1736.8948 | -0.0117 | -7 | 1081 | 1097 | TLPVYSTNDTTIVAGGK | | | | | Mascot |
| 2114.0732 | 2114.1997 | 0.1265 | 60 | 695 | 714 | KQLGLSLPSIGCSHICND TK | | | | | Mascot |
| 2114.0732 | 2114.1997 | 0.1265 | 60 | 695 | 714 | KQLGLSLPSIGCSHICND TK | | | | | Mascot |
| 2156.9595 | 2157.1462 | 0.1867 | 87 | 1271 | 1288 | CMDCFQTIINPCPSGTKK | | | Carbamidomethyl (C)[1,4,12] | | Mascot |
| 2196.9504 | 2197.137 | 0.1866 | 85 | 267 | 287 | VCMPIDPCQTNLGNCTS GSTR | | | | | Mascot |
| 2284.0486 | 2284.1877 | 0.1391 | 61 | 1511 | 1528 | NCELINLCKEGSHLYYDR | | | Carbamidomethyl (C)[2,8] | | Mascot |
| 2384.0098 | 2384.0769 | 0.0671 | 28 | 267 | 287 | VCMPIDPCQTNLGNCTS GSTR | | | Carbamidomethyl (C)[2,8,15], Oxidation (M)[3] | | Mascot |
| 2531.3525 | 2531.2527 | -0.0998 | -39 | 494 | 516 | LMDAVPSTVISEKEESLL EILSK | | | | | Mascot |

| 16 | novel protein similar to vertebrate aldo-keto reductase family 1 [Danio rerio] | gi\|56207864 | 38502.8 | 6.73 | 4 | 40 | 0 | 28 | 54.216 |
|---|---|---|---|---|---|---|---|---|---|

**Peptide Information**

| Calc. Mass | Obsrv. Mass | ± da | ± ppm | Start Seq. | End Seq. | Sequence | Ion Score | C. I. % | Modification | Rank | Result Type |
|---|---|---|---|---|---|---|---|---|---|---|---|
| 850.4781 | 850.5455 | 0.0674 | 79 | 316 | 322 | TILSFNR | | | | | Mascot |
| 901.5465 | 901.5135 | -0.033 | -37 | 272 | 279 | TTAQVLIR | 28 | 54.216 | | | Mascot |

| | | | | | | | | | | | | |
|---|---|---|---|---|---|---|---|---|---|---|---|---|
| | 901.5465 | 901.5135 | -0.033 | -37 | 272 | 279 | TTAQVLIR | | | | | Mascot |
| | 1028.4805 | 1028.5013 | 0.0208 | 20 | 326 | 334 | ACPMHWGVK | | | | | Mascot |
| | 1112.631 | 1112.5123 | -0.1187 | -107 | 51 | 61 | SPPGKVTEAVK | | | | | Mascot |

| | | | | | | | | |
|---|---|---|---|---|---|---|---|---|
| 17 | PREDICTED: diacylglycerol kinase beta-like, partial [Danio rerio] | gi\|326674321 | 85995.9 | 6.78 | 13 | 39 | 0 | |

**Peptide Information**

| Calc. Mass | Obsrv. Mass | ± da | ± ppm | Start Seq. | End Seq. | Sequence | Ion Score | C. I. % | Modification | Rank | Result Type |
|---|---|---|---|---|---|---|---|---|---|---|---|
| 842.4229 | 842.5153 | 0.0924 | 110 | 153 | 158 | LEFMFR | | | | | Mascot |
| 864.4032 | 864.4554 | 0.0522 | 60 | 455 | 462 | SGPMPGYR | | | | | Mascot |
| 901.5353 | 901.5135 | -0.0218 | -24 | 678 | 685 | KTTIVDPK | | | | | Mascot |
| 901.5353 | 901.5135 | -0.0218 | -24 | 678 | 685 | KTTIVDPK | | | | | Mascot |
| 1045.4264 | 1045.5679 | 0.1415 | 135 | 270 | 278 | QGLCCSFCK | | | Carbamidomethyl (C)[4] | | Mascot |
| 1112.6497 | 1112.5123 | -0.1374 | -123 | 112 | 121 | LIKGSCPPLK | | | Carbamidomethyl (C)[6] | | Mascot |
| 1210.5455 | 1210.6071 | 0.0616 | 51 | 455 | 465 | SGPMPGYRSCR | | | | | Mascot |
| 1707.8007 | 1707.7886 | -0.0121 | -7 | 534 | 547 | DVQDSSEVMLDRWK | | | | | Mascot |
| 2157.0005 | 2157.1462 | 0.1457 | 68 | 302 | 319 | KNTEIMHHFWVEGNCPS K | | | | | Mascot |
| 2283.2488 | 2283.1914 | -0.0574 | -25 | 115 | 135 | GSCPPLKVTSQSRPLSTV QLK | | | Carbamidomethyl (C)[3] | | Mascot |
| 2284.0591 | 2284.1877 | 0.1286 | 56 | 153 | 171 | LEFMFRLYDTDGNGFLD SK | | | Oxidation (M)[4] | | Mascot |
| 2432.0581 | 2432.1536 | 0.0955 | 39 | 303 | 322 | NTEIMHHFWVEGNCPSK CDK | | | Carbamidomethyl (C)[14] | | Mascot |
| 2432.0581 | 2432.1536 | 0.0955 | 39 | 303 | 322 | NTEIMHHFWVEGNCPSK CDK | | | Carbamidomethyl (C)[14] | | Mascot |
| 2448.053 | 2448.1565 | 0.1035 | 42 | 303 | 322 | NTEIMHHFWVEGNCPSK CDK | | | Carbamidomethyl (C)[14], Oxidation (M)[5] | | Mascot |
| 2479.3022 | 2479.3062 | 0.004 | 2 | 248 | 269 | LKHFSKPAYCNLCLNMLI GLGK | | | Oxidation (M)[16] | | Mascot |
| 2488.1682 | 2488.1079 | -0.0603 | -24 | 329 | 348 | CYQGLTGLHCVWCQITL HNK | | | Carbamidomethyl (C)[1,10,13] | | Mascot |
| 2489.0796 | 2489.1013 | 0.0217 | 9 | 303 | 322 | NTEIMHHFWVEGNCPSK CDK | | | Carbamidomethyl (C)[14,18] | | Mascot |

| | | | | | | | | |
|---|---|---|---|---|---|---|---|---|
| 18 | PREDICTED: uncharacterized protein C11orf95 homolog [Danio rerio] | gi\|326669276 | 70864.2 | 5.62 | 11 | 38 | 0 | |

**Peptide Information**

| Calc. Mass | Obsrv. Mass | ± da | ± ppm | Start Seq. | End Seq. | Sequence | Ion Score | C. I. % | Modification | Rank | Result Type |
|---|---|---|---|---|---|---|---|---|---|---|---|
| 1045.4619 | 1045.5679 | 0.106 | 101 | 1 | 9 | MDEAESVHK | | | | | Mascot |
| 1112.5232 | 1112.5123 | -0.0109 | -10 | 167 | 175 | SGWENHLNR | | | | | Mascot |
| 1365.5272 | 1365.6426 | 0.1154 | 85 | 530 | 539 | MDFLMDYDCR | | | Carbamidomethyl (C)[9] | | Mascot |
| 1436.6007 | 1436.7474 | 0.1467 | 102 | 530 | 540 | MDFLMDYDCRK | | | | | Mascot |

| | | | | | | | | | | | |
|---|---|---|---|---|---|---|---|---|---|---|---|
| 1475.7166 | 1475.7635 | 0.0469 | 32 | 151 | 162 | HPYSLLWTESDK | | | | | Mascot |
| 1751.8494 | 1751.8267 | -0.0227 | -13 | 431 | 445 | KHIQECHPETSSLSR | | | | | Mascot |
| 1862.8185 | 1863.073 | 0.2545 | 137 | 1 | 16 | MDEAESVHKAEQTESR | | | Oxidation (M)[1] | | Mascot |
| 2114.0222 | 2114.1997 | 0.1775 | 84 | 291 | 308 | SHMLELHPNSLLYSAEEK | | | Oxidation (M)[3] | | Mascot |
| 2114.0222 | 2114.1997 | 0.1775 | 84 | 291 | 308 | SHMLELHPNSLLYSAEEK | | | Oxidation (M)[3] | | Mascot |
| 2175.1697 | 2175.1536 | -0.0161 | -7 | 541 | 561 | HGLICMVCGATLATLKVS TIK | | | Oxidation (M)[6] | | Mascot |
| 2175.1697 | 2175.1536 | -0.0161 | -7 | 541 | 561 | HGLICMVCGATLATLKVS TIK | | | Oxidation (M)[6] | | Mascot |
| 2197.1001 | 2197.137 | 0.0369 | 17 | 272 | 290 | LVCMVCSCLLPSLHLDHI K | | | Carbamidomethyl (C)[3], Oxidation (M)[4] | | Mascot |
| 2298.1687 | 2298.198 | 0.0293 | 13 | 124 | 144 | QGMICMVCGSSLATLKLS TIK | | | Carbamidomethyl (C)[5,8] | | Mascot |

| | | | | | | | | | |
|---|---|---|---|---|---|---|---|---|---|
| 19 | hypothetical protein LOC100142653 [Danio rerio] | gi|169790892 | 51963.8 | 6.07 | 2 | 38 | 0 | 38 | 96.076 |

**Peptide Information**

| Calc. Mass | Obsrv. Mass | ± da | ± ppm | Start Seq. | End Seq. | Sequence | Ion Score | C. I. % | Modification | Rank | Result Type |
|---|---|---|---|---|---|---|---|---|---|---|---|
| 901.5101 | 901.5135 | 0.0034 | 4 | 350 | 357 | SDVILQAR | 38 | 96.076 | | | Mascot |
| 901.5101 | 901.5135 | 0.0034 | 4 | 350 | 357 | SDVILQAR | | | | | Mascot |
| 2294.2463 | 2294.178 | -0.0683 | -30 | 350 | 371 | SDVILQARGSSTVLLADG NHLK | | | | | Mascot |

| | | | | | | | | | |
|---|---|---|---|---|---|---|---|---|---|
| 20 | novel protein containing an EMI domain [Danio rerio] | gi|148725612 | 46007.5 | 9.47 | 3 | 38 | 0 | 31 | 78.486 |

**Peptide Information**

| Calc. Mass | Obsrv. Mass | ± da | ± ppm | Start Seq. | End Seq. | Sequence | Ion Score | C. I. % | Modification | Rank | Result Type |
|---|---|---|---|---|---|---|---|---|---|---|---|
| 842.4188 | 842.5153 | 0.0965 | 115 | 425 | 431 | AKMSSFR | | | Oxidation (M)[3] | | Mascot |
| 901.456 | 901.5135 | 0.0575 | 64 | 9 | 15 | CLQIPDR | 31 | 78.486 | Carbamidomethyl (C)[1] | | Mascot |
| 901.456 | 901.5135 | 0.0575 | 64 | 9 | 15 | CLQIPDR | | | Carbamidomethyl (C)[1] | | Mascot |
| 1342.6709 | 1342.6414 | -0.0295 | -22 | 220 | 234 | GDVGTRGPSGAPGSK | | | | | Mascot |

| | | | | | | | | | |
|---|---|---|---|---|---|---|---|---|---|
| 21 | PREDICTED: hypothetical protein LOC100003636 [Danio rerio] | gi|292612849 | 55164.8 | 4.93 | 4 | 38 | 0 | 28 | 59.847 |

**Peptide Information**

| Calc. Mass | Obsrv. Mass | ± da | ± ppm | Start Seq. | End Seq. | Sequence | Ion Score | C. I. % | Modification | Rank | Result Type |
|---|---|---|---|---|---|---|---|---|---|---|---|
| 901.5213 | 901.5135 | -0.0078 | -9 | 34 | 41 | AEVRSALR | 28 | 59.847 | | | Mascot |
| 901.5213 | 901.5135 | -0.0078 | -9 | 34 | 41 | AEVRSALR | | | | | Mascot |
| 1028.516 | 1028.5013 | -0.0147 | -14 | 109 | 115 | EYYERLR | | | | | Mascot |
| 1365.6182 | 1365.6426 | 0.0244 | 18 | 104 | 113 | HESPREYYER | | | | | Mascot |
| 2531.271 | 2531.2527 | -0.0183 | -7 | 147 | 167 | QQLALFNIEKIWAMSEPD | | | | | Mascot |

| | | | | | | | | |
|---|---|---|---|---|---|---|---|---|
| | | QNR | | | | | | |
| 22 | PREDICTED: hypothetical protein LOC100331078 [Danio rerio] | gi\|326668546 | 57666.5 | 8.69 | 11 | 37 | 0 | |

Peptide Information

| Calc. Mass | Obsrv. Mass | ± da | ± ppm | Start Seq. | End Seq. | Sequence | Ion Score | C. I. % | Modification | Rank | Result Type |
|---|---|---|---|---|---|---|---|---|---|---|---|
| 959.5342 | 959.5181 | -0.0161 | -17 | 46 | 54 | DLIGCIIGR | | | | | Mascot |
| 1179.5211 | 1179.6071 | 0.086 | 73 | 389 | 398 | QMNWSGANQK | | | Oxidation (M)[2] | | Mascot |
| 1210.7153 | 1210.6071 | -0.1082 | -89 | 5 | 15 | HSGVVTLIKEK | | | | | Mascot |
| 1365.7485 | 1365.6426 | -0.1059 | -78 | 35 | 45 | RIEDDLHQVLK | | | | | Mascot |
| 1719.8469 | 1719.8651 | 0.0182 | 11 | 465 | 479 | KENGGDILEDLELMK | | | Oxidation (M)[14] | | Mascot |
| 1719.8469 | 1719.8651 | 0.0182 | 11 | 465 | 479 | KENGGDILEDLELMK | | | Oxidation (M)[14] | | Mascot |
| 1736.8319 | 1736.8948 | 0.0629 | 36 | 14 | 28 | EKAPNAVGTHCMLHR | | | Carbamidomethyl (C)[11], Oxidation (M)[12] | | Mascot |
| 1901.9347 | 1902.0004 | 0.0657 | 35 | 281 | 296 | TYCNSSCLSLLTELIK | | | Carbamidomethyl (C)[3,7] | | Mascot |
| 1993.943 | 1993.9897 | 0.0467 | 23 | 64 | 82 | QMSGAQIKIANAMEGSSDR | | | | | Mascot |
| 2196.9939 | 2197.137 | 0.1431 | 65 | 261 | 280 | SSTPRNIYSPCSSAVGDQDR | | | Carbamidomethyl (C)[11] | | Mascot |
| 2384.2788 | 2384.0769 | -0.2019 | -85 | 132 | 154 | ICSSSSLTLPRLIGCIIGHQGTK | | | | | Mascot |
| 2488.1694 | 2488.1079 | -0.0615 | -25 | 466 | 487 | ENGGDILEDLELMKQVGMEHSK | | | Oxidation (M)[13] | | Mascot |

| | | | | | | | |
|---|---|---|---|---|---|---|---|
| 23 | PREDICTED: pericentrin [Danio rerio] | gi\|326668478 | 491858 | 4.98 | 29 | 36 | 0 |

Peptide Information

| Calc. Mass | Obsrv. Mass | ± da | ± ppm | Start Seq. | End Seq. | Sequence | Ion Score | C. I. % | Modification | Rank | Result Type |
|---|---|---|---|---|---|---|---|---|---|---|---|
| 842.4995 | 842.5153 | 0.0158 | 19 | 15 | 21 | AKLAHFR | | | | | Mascot |
| 864.3581 | 864.4554 | 0.0973 | 113 | 1982 | 1988 | EESDQEK | | | | | Mascot |
| 882.5155 | 882.5723 | 0.0568 | 64 | 3606 | 3612 | SEIHKLR | | | | | Mascot |
| 901.5101 | 901.5135 | 0.0034 | 4 | 1827 | 1833 | KEEQVIR | 19 | 0 | | | Mascot |
| 901.5101 | 901.5135 | 0.0034 | 4 | 1827 | 1833 | KEEQVIR | | | | | Mascot |
| 959.4904 | 959.5181 | 0.0277 | 29 | 3810 | 3816 | EQEEIRR | | | | | Mascot |
| 1028.4604 | 1028.5013 | 0.0409 | 40 | 891 | 898 | AELEYMEK | | | Oxidation (M)[6] | | Mascot |
| 1045.4908 | 1045.5679 | 0.0771 | 74 | 1472 | 1480 | QAEAEVNER | | | | | Mascot |
| 1060.5634 | 1060.4849 | -0.0785 | -74 | 1834 | 1842 | DELRSELAK | | | | | Mascot |
| 1076.5331 | 1076.4808 | -0.0523 | -49 | 1285 | 1293 | SSRVDDIER | | | | | Mascot |
| 1103.5878 | 1103.5774 | -0.0104 | -9 | 1192 | 1200 | ELKQALMDR | | | | | Mascot |
| 1112.5847 | 1112.5123 | -0.0724 | -65 | 1318 | 1325 | TYFEQRLR | | | | | Mascot |
| 1128.5392 | 1128.5116 | -0.0276 | -24 | 3753 | 3762 | QESASAHDRK | | | | | Mascot |

| Calc. Mass | Obsrv. Mass | ± da | ± ppm | Start Seq. | End Seq. | Sequence | Ion Score | C. I. % | Modification | Rank | Result Type |
|---|---|---|---|---|---|---|---|---|---|---|---|
| 1179.5674 | 1179.6071 | 0.0397 | 34 | 1115 | 1124 | TSVEQLQSCK | | | Carbamidomethyl (C)[9] | | Mascot |
| 1210.5698 | 1210.6071 | 0.0373 | 31 | 1540 | 1549 | NRASEEFTEK | | | | | Mascot |
| 1342.6671 | 1342.6414 | -0.0257 | -19 | 3003 | 3013 | QEMTSKTNYIK | | | | | Mascot |
| 1475.7522 | 1475.7635 | 0.0113 | 8 | 3623 | 3635 | QTCLQAVEKEQAK | | | | | Mascot |
| 1488.838 | 1488.793 | -0.045 | -30 | 1488 | 1500 | VTQLESRLSQLSK | | | | | Mascot |
| 1488.838 | 1488.793 | -0.045 | -30 | 1488 | 1500 | VTQLESRLSQLSK | | | | | Mascot |
| 1719.8547 | 1719.8651 | 0.0104 | 6 | 3592 | 3605 | TELQSSYKHQEELK | | | | | Mascot |
| 1719.8547 | 1719.8651 | 0.0104 | 6 | 3592 | 3605 | TELQSSYKHQEELK | | | | | Mascot |
| 1767.8503 | 1767.8225 | -0.0278 | -16 | 828 | 842 | LTCLKENLTSMEEEK | | | | | Mascot |
| 1993.9937 | 1993.9897 | -0.004 | -2 | 3014 | 3031 | ELHEQQEAAVAKQGEAQK | | | | | Mascot |
| 1999.9791 | 1999.9546 | -0.0245 | -12 | 1463 | 1480 | LQSAQEAARQAEAEVNER | | | | | Mascot |
| 1999.9791 | 1999.9546 | -0.0245 | -12 | 1463 | 1480 | LQSAQEAARQAEAEVNER | | | | | Mascot |
| 2157.0969 | 2157.1462 | 0.0493 | 23 | 3936 | 3954 | HTNISQDSSTPSLIERLMK | | | | | Mascot |
| 2175.1438 | 2175.1536 | 0.0098 | 5 | 2014 | 2032 | MSQLTAQLQQLDTVVSQLR | | | Oxidation (M)[1] | | Mascot |
| 2175.1438 | 2175.1536 | 0.0098 | 5 | 2014 | 2032 | MSQLTAQLQQLDTVVSQLR | | | Oxidation (M)[1] | | Mascot |
| 2197.052 | 2197.137 | 0.085 | 39 | 1307 | 1323 | EQNEVELENLRTYFEQR | | | | | Mascot |
| 2284.1013 | 2284.1877 | 0.0864 | 38 | 3955 | 3974 | ENSEFSECLAALSEEKISLK | | | Carbamidomethyl (C)[8] | | Mascot |
| 2357.1255 | 2357.1431 | 0.0176 | 7 | 392 | 410 | YEADISNYDLRLQTLEEER | | | | | Mascot |
| 2457.1133 | 2457.3267 | 0.2134 | 87 | 3525 | 3544 | EQGMETQHQLHSDVKELEMR | | | Oxidation (M)[4,19] | | Mascot |
| 2457.1133 | 2457.3267 | 0.2134 | 87 | 3525 | 3544 | EQGMETQHQLHSDVKELEMR | | | Oxidation (M)[4,19] | | Mascot |
| 2488.3042 | 2488.1079 | -0.1963 | -79 | 3152 | 3174 | FLEGLSQDPSLSASLSSPEVLRR | | | | | Mascot |
| 2515.282 | 2515.2617 | -0.0203 | -8 | 1026 | 1047 | LSLTNLHTAQLELSQSNMQKDK | | | Oxidation (M)[18] | | Mascot |

| | | | | | | | | | | |
|---|---|---|---|---|---|---|---|---|---|---|
| 24 | hypothetical protein LOC553452 [Danio rerio] | gi\|116235416 | 35430.1 | 6.02 | 3 | 36 | 0 | 28 | 54.216 |

**Peptide Information**

| Calc. Mass | Obsrv. Mass | ± da | ± ppm | Start Seq. | End Seq. | Sequence | Ion Score | C. I. % | Modification | Rank | Result Type |
|---|---|---|---|---|---|---|---|---|---|---|---|
| 850.4781 | 850.5455 | 0.0674 | 79 | 288 | 294 | TILSFNR | | | | | Mascot |
| 901.5465 | 901.5135 | -0.033 | -37 | 244 | 251 | TTAQVLIR | 28 | 54.216 | | | Mascot |
| 901.5465 | 901.5135 | -0.033 | -37 | 244 | 251 | TTAQVLIR | | | | | Mascot |
| 1028.4805 | 1028.5013 | 0.0208 | 20 | 298 | 306 | ACPMHWGVK | | | | | Mascot |

| | | | | | | | | |
|---|---|---|---|---|---|---|---|---|
| 25 | PREDICTED: hypothetical protein LOC100006878 [Danio rerio] | gi\|292628291 | 6219.4 | 9.11 | 4 | 36 | 0 |

**Peptide Information**

| Calc. Mass | Obsrv. Mass | ± da | ± ppm | Start Seq. | End Seq. | Sequence | Ion Score | C. I. % | Modification | Rank | Result Type |
|---|---|---|---|---|---|---|---|---|---|---|---|
| 1365.6388 | 1365.6426 | 0.0038 | 3 | 1 | 13 | MGVLSVCEEAPSK | | | Oxidation (M)[1] | | Mascot |
| 1736.0105 | 1735.8551 | -0.1554 | -90 | 30 | 45 | QGLPLALTHLVGFLEK | | | | | Mascot |
| 2294.3052 | 2294.178 | -0.1272 | -55 | 25 | 45 | TGLMRQGLPLALTHLVGF LEK | | | | | Mascot |
| 2471.2783 | 2471.1096 | -0.1687 | -68 | 1 | 23 | MGVLSVCEEAPSKVFGV PLSHLR | | | Oxidation (M)[1] | | Mascot |

26 PREDICTED: hypothetical protein LOC565734 [Danio rerio] gi|68402113 30582.9 10.05 7 35 0

**Peptide Information**

| Calc. Mass | Obsrv. Mass | ± da | ± ppm | Start Seq. | End Seq. | Sequence | Ion Score | C. I. % | Modification | Rank | Result Type |
|---|---|---|---|---|---|---|---|---|---|---|---|
| 1165.6324 | 1165.7528 | 0.1204 | 103 | 118 | 128 | VSPNATHAQIK | | | | | Mascot |
| 1436.7943 | 1436.7474 | -0.0469 | -33 | 80 | 91 | HWCLLLRPVGSR | | | | | Mascot |
| 1475.7634 | 1475.7635 | 0.0001 | 0 | 1 | 14 | MAEVTGSVRNAVNK | | | | | Mascot |
| 1902.0093 | 1902.0004 | -0.0089 | -5 | 20 | 36 | TLLSLHHHPHGPVVDSR | | | | | Mascot |
| 2284.0571 | 2284.1877 | 0.1306 | 57 | 41 | 60 | KSNVCVHWCAADCVWA LHNK | | | | | Mascot |
| 2448.3259 | 2448.1565 | -0.1694 | -69 | 15 | 36 | LSAFKTLLSLHHHPHGPV VDSR | | | | | Mascot |
| 2479.5107 | 2479.3062 | -0.2045 | -82 | 246 | 269 | EQITGVSVILLLLLGGVIIN SLKS | | | | | Mascot |

27 NADH dehydrogenase (ubiquinone) 1, alpha/beta subcomplex 1-like [Danio rerio] gi|51010919 17698.1 6.16 2 34 0

**Peptide Information**

| Calc. Mass | Obsrv. Mass | ± da | ± ppm | Start Seq. | End Seq. | Sequence | Ion Score | C. I. % | Modification | Rank | Result Type |
|---|---|---|---|---|---|---|---|---|---|---|---|
| 901.54 | 901.5135 | -0.0265 | -29 | 1 | 8 | MAARILAR | 26 | 30.222 | | | Mascot |
| 901.54 | 901.5135 | -0.0265 | -29 | 1 | 8 | MAARILAR | | | | | Mascot |
| 1893.9817 | 1893.9919 | 0.0102 | 5 | 49 | 65 | SSLAQVSGSSLQIFQWR | | | | | Mascot |
| 1893.9817 | 1893.9919 | 0.0102 | 5 | 49 | 65 | SSLAQVSGSSLQIFQWR | | | | | Mascot |

28 PREDICTED: hypothetical protein LOC100535442 isoform 2 [Danio rerio] gi|326668097 11743 9.12 5 34 0

**Peptide Information**

| Calc. Mass | Obsrv. Mass | ± da | ± ppm | Start Seq. | End Seq. | Sequence | Ion Score | C. I. % | Modification | Rank | Result Type |
|---|---|---|---|---|---|---|---|---|---|---|---|
| 901.5101 | 901.5135 | 0.0034 | 4 | 31 | 38 | TNDLLAVR | 3 | 0 | | | Mascot |
| 901.5101 | 901.5135 | 0.0034 | 4 | 31 | 38 | TNDLLAVR | | | | | Mascot |

| | | | | | | | | | | | |
|---|---|---|---|---|---|---|---|---|---|---|---|
| 1128.6483 | 1128.5116 | -0.1367 | -121 | 29 | 38 | ARTNDLLAVR | | | | | Mascot |
| 1436.7493 | 1436.7474 | -0.0019 | -1 | 18 | 30 | LHTETPTDPAKAR | | | | | Mascot |
| 1488.7589 | 1488.793 | 0.0341 | 23 | 91 | 103 | LTNTCFVITFVCK | | | | | Mascot |
| 1488.7589 | 1488.793 | 0.0341 | 23 | 91 | 103 | LTNTCFVITFVCK | | | | | Mascot |
| 1893.9601 | 1893.9919 | 0.0318 | 17 | 91 | 105 | LTNTCFVITFVCKYK | | | Carbamidomethyl (C)[5,12] | | Mascot |
| 1893.9601 | 1893.9919 | 0.0318 | 17 | 91 | 105 | LTNTCFVITFVCKYK | | | Carbamidomethyl (C)[5,12] | | Mascot |

29 novel protein similar to human and mouse myeloid/lymphoid or mixed-lineage leukemia 3 (MLL3) [Danio | gi|157885936 | 82601.8 | 9.38 | 14 | 34 | 0

**Peptide Information**

| Calc. Mass | Obsrv. Mass | ± da | ± ppm | Start Seq. | End Seq. | Sequence | Ion Score | C. I. % | Modification | Rank | Result Type |
|---|---|---|---|---|---|---|---|---|---|---|---|
| 864.5189 | 864.4554 | -0.0635 | -73 | 636 | 642 | ELLYKAK | | | | | Mascot |
| 870.4679 | 870.5441 | 0.0762 | 88 | 140 | 147 | SISEGHLK | | | | | Mascot |
| 901.4308 | 901.5135 | 0.0827 | 92 | 286 | 294 | LGATSGCHR | | | | | Mascot |
| 901.4308 | 901.5135 | 0.0827 | 92 | 286 | 294 | LGATSGCHR | | | | | Mascot |
| 996.5948 | 996.6034 | 0.0086 | 9 | 462 | 470 | VLGPVAERR | | | | | Mascot |
| 1045.6152 | 1045.5679 | -0.0473 | -45 | 602 | 611 | IQGLGLFAAR | | | | | Mascot |
| 1060.4452 | 1060.4849 | 0.0397 | 37 | 17 | 24 | QQWCQHCK | | | | | Mascot |
| 1210.6538 | 1210.6071 | -0.0467 | -39 | 359 | 369 | QLAAAVQQPER | | | | | Mascot |
| 1707.8483 | 1707.7886 | -0.0597 | -35 | 499 | 513 | IAESLPGVEACSRYR | | | Carbamidomethyl (C)[11] | | Mascot |
| 1751.8724 | 1751.8267 | -0.0457 | -26 | 156 | 169 | LHSHLEDKQWHHGK | | | | | Mascot |
| 1880.9487 | 1880.9967 | 0.048 | 26 | 440 | 456 | VIEQGYEDLVLTDTSAK | | | | | Mascot |
| 1915.7554 | 1915.9689 | 0.2135 | 111 | 220 | 236 | CCFCQQIGDGMTDGPAR | | | Carbamidomethyl (C)[1,2] | | Mascot |
| 2225.0112 | 2225.1333 | 0.1221 | 55 | 668 | 686 | YINHSCSPNCVAEVVTFE R | | | Carbamidomethyl (C)[6] | | Mascot |
| 2283.1562 | 2283.1914 | 0.0352 | 15 | 328 | 348 | GAGAAAGLHVEHQLRCF SVFR | | | Carbamidomethyl (C)[16] | | Mascot |
| 2294.0554 | 2294.178 | 0.1226 | 53 | 297 | 315 | CLNIYHFTCALQAGCTFF K | | | Carbamidomethyl (C)[1,9] | | Mascot |

30 novel protein similar to vertebrate pim oncogene family [Danio rerio] | gi|55962880 | 28662.1 | 6.36 | 7 | 34 | 0

**Peptide Information**

| Calc. Mass | Obsrv. Mass | ± da | ± ppm | Start Seq. | End Seq. | Sequence | Ion Score | C. I. % | Modification | Rank | Result Type |
|---|---|---|---|---|---|---|---|---|---|---|---|
| 959.5342 | 959.5181 | -0.0161 | -17 | 106 | 113 | LIPSMNLR | | | Oxidation (M)[5] | | Mascot |
| 1342.7729 | 1342.6414 | -0.1315 | -98 | 83 | 93 | VKQIAELLDWK | | | | | Mascot |
| 1365.7446 | 1365.6426 | -0.102 | -75 | 168 | 179 | LIDFGCSVLLTK | | | Carbamidomethyl (C)[6] | | Mascot |
| 1475.8217 | 1475.7635 | -0.0582 | -39 | 94 | 105 | VEAERYIIVVER | | | | | Mascot |

| | | | | | | | | | | | |
|---|---|---|---|---|---|---|---|---|---|---|---|
| 1707.8127 | 1707.7886 | -0.0241 | -14 | 131 | 145 | IMFNVTIAAHTCCQR | | | | | Mascot |
| 1908.9241 | 1908.9456 | 0.0215 | 11 | 130 | 145 | KIMFNVTIAAHTCCQR | | | Carbamidomethyl (C)[13], Oxidation (M)[3] | | Mascot |
| 2432.3376 | 2432.1536 | -0.184 | -76 | 202 | 222 | YHGEPATVWSLGILQFLL LFK | | | | | Mascot |
| 2432.3376 | 2432.1536 | -0.184 | -76 | 202 | 222 | YHGEPATVWSLGILQFLL LFK | | | | | Mascot |

| | | | | | | | |
|---|---|---|---|---|---|---|---|
| 31 | cytoplasmic dynein 1 heavy chain 1 [Danio rerio] | gi\|112363126 | 532854.1 | 6.06 | 32 | 33 | 0 |

Peptide Information

| Calc. Mass | Obsrv. Mass | ± da | ± ppm | Start Seq. | End Seq. | Sequence | Ion Score | C. I. % | Modification | Rank | Result Type |
|---|---|---|---|---|---|---|---|---|---|---|---|
| 870.4389 | 870.5441 | 0.1052 | 121 | 656 | 662 | QLTAYMK | | | Oxidation (M)[6] | | Mascot |
| 959.5342 | 959.5181 | -0.0161 | -17 | 990 | 997 | MNILSLPR | | | Oxidation (M)[1] | | Mascot |
| 1028.5735 | 1028.5013 | -0.0722 | -70 | 2025 | 2033 | SNLPDNLKK | | | | | Mascot |
| 1045.5272 | 1045.5679 | 0.0407 | 39 | 2836 | 2843 | LVEDEERR | | | | | Mascot |
| 1060.5455 | 1060.4849 | -0.0606 | -57 | 4292 | 4300 | DIKMPDGIR | | | Oxidation (M)[4] | | Mascot |
| 1076.5153 | 1076.4808 | -0.0345 | -32 | 4563 | 4572 | LQGATCANNK | | | Carbamidomethyl (C)[6] | | Mascot |
| 1165.6212 | 1165.7528 | 0.1316 | 113 | 3438 | 3447 | DLEASIARYK | | | | | Mascot |
| 1179.6368 | 1179.6071 | -0.0297 | -25 | 2551 | 2560 | VPQIEVETHK | | | | | Mascot |
| 1342.7147 | 1342.6414 | -0.0733 | -55 | 1497 | 1508 | VKEHINSVSAMK | | | | | Mascot |
| 1365.7412 | 1365.6426 | -0.0986 | -72 | 1099 | 1110 | KEFGPVIIDYGK | | | | | Mascot |
| 1475.6835 | 1475.7635 | 0.08 | 54 | 3682 | 3694 | DPTVEFPPDLCSR | | | | | Mascot |
| 1542.9154 | 1542.8931 | -0.0223 | -14 | 754 | 766 | WLSFRVPLAIVNK | | | | | Mascot |
| 1707.7894 | 1707.7886 | -0.0008 | 0 | 1025 | 1040 | MPDGPSALEEAYNAVK | | | Oxidation (M)[1] | | Mascot |
| 1862.9656 | 1863.073 | 0.1074 | 58 | 4143 | 4158 | IFVFEPPPGMKANMLR | | | Oxidation (M)[10] | | Mascot |
| 1894.032 | 1893.9919 | -0.0401 | -21 | 2417 | 2434 | DAAAVLQPYFTSTGLVIK | | | | | Mascot |
| 1894.032 | 1893.9919 | -0.0401 | -21 | 2417 | 2434 | DAAAVLQPYFTSTGLVIK | | | | | Mascot |
| 1908.9813 | 1908.9456 | -0.0357 | -19 | 664 | 680 | VEDVLGKGWENHVEGLK | | | | | Mascot |
| 1915.9153 | 1915.9689 | 0.0536 | 28 | 2435 | 2450 | ALEHASKMEHIMDFTR | | | | | Mascot |
| 2113.9429 | 2114.1997 | 0.2568 | 121 | 1130 | 1147 | FGQMLGNNMQDFHSQIS K | | | Oxidation (M)[4,9] | | Mascot |
| 2113.9429 | 2114.1997 | 0.2568 | 121 | 1130 | 1147 | FGQMLGNNMQDFHSQIS K | | | Oxidation (M)[4,9] | | Mascot |
| 2157.145 | 2157.1462 | 0.0012 | 1 | 767 | 785 | AHQANQLYPFAISLIESVR | | | | | Mascot |
| 2175.1365 | 2175.1536 | 0.0171 | 8 | 3318 | 3336 | LALESICLLLGEETNDWK K | | | | | Mascot |
| 2175.1365 | 2175.1536 | 0.0171 | 8 | 3318 | 3336 | LALESICLLLGEETNDWK K | | | | | Mascot |
| 2197.1287 | 2197.137 | 0.0083 | 4 | 828 | 845 | LDPYVQRLAETVFNFQEK | | | | | Mascot |
| 2210.9944 | 2211.1179 | 0.1235 | 56 | 531 | 550 | EVDGLDVSKEGMEAWEA AMK | | | Oxidation (M)[12] | | Mascot |
| 2233.0952 | 2233.105 | 0.0098 | 4 | 4421 | 4439 | MLQDVRQDLTDVVQVCE | | | Carbamidomethyl (C)[16] | | Mascot |

| | | | | | | | | | | | |
|---|---|---|---|---|---|---|---|---|---|---|---|
| | | | | | | GK | | | | | |
| 2283.1624 | 2283.1914 | 0.029 | 13 | 2004 | 2024 | QVKVSAEMAIFITMNPGYAGR | | | | | Mascot |
| 2284.2561 | 2284.1877 | -0.0684 | -30 | 4504 | 4523 | NIHVNLGRLFVPEAYITATR | | | | | Mascot |
| 2294.1824 | 2294.178 | -0.0044 | -2 | 4092 | 4110 | WVMLENVHLAPGWLMQLEK | | | | | Mascot |
| 2357.0762 | 2357.1431 | 0.0669 | 28 | 1130 | 1149 | FGQMLGNNMQDFHSQISKSR | | | Oxidation (M)[4,9] | | Mascot |
| 2384.2642 | 2384.0769 | -0.1873 | -79 | 2046 | 2066 | QLIAQVMLYSQGFRTAETLAK | | | Oxidation (M)[7] | | Mascot |
| 2432.2917 | 2432.1536 | -0.1381 | -57 | 3448 | 3470 | EEYAVLISEAQAIKADLAAVEAK | | | | | Mascot |
| 2432.2917 | 2432.1536 | -0.1381 | -57 | 3448 | 3470 | EEYAVLISEAQAIKADLAAVEAK | | | | | Mascot |
| 2488.2349 | 2488.1079 | -0.127 | -51 | 3705 | 3726 | SSLQSQCLNEVLKAERPDVDEK | | | | | Mascot |
| 2515.1533 | 2515.2617 | 0.1084 | 43 | 1052 | 1070 | VWLQYQCLWDMQPENIYNR | | | Oxidation (M)[11] | | Mascot |
| 2531.2888 | 2531.2527 | -0.0361 | -14 | 261 | 282 | LDRDPASGTALQEISFWLNLER | | | | | Mascot |

32 Zgc:152851 protein [Danio rerio] gi|27503941 44653.3 8.79 1 33 0 33 87.79

**Peptide Information**

| Calc. Mass | Obsrv. Mass | ± da | ± ppm | Start Seq. | End Seq. | Sequence | Ion Score | C. I. % | Modification | Rank | Result Type |
|---|---|---|---|---|---|---|---|---|---|---|---|
| 901.4923 | 901.5135 | 0.0212 | 24 | 38 | 46 | ACVLPGSVR | 33 | 87.79 | | | Mascot |
| 901.4923 | 901.5135 | 0.0212 | 24 | 38 | 46 | ACVLPGSVR | | | | | Mascot |

33 complement component 3-like isoform 1 [Danio rerio] gi|153792045 188748 7.66 17 33 0

**Peptide Information**

| Calc. Mass | Obsrv. Mass | ± da | ± ppm | Start Seq. | End Seq. | Sequence | Ion Score | C. I. % | Modification | Rank | Result Type |
|---|---|---|---|---|---|---|---|---|---|---|---|
| 842.5206 | 842.5153 | -0.0053 | -6 | 516 | 523 | GKIIQAGR | | | | | Mascot |
| 1128.532 | 1128.5116 | -0.0204 | -18 | 1058 | 1066 | GYENQLNYK | | | | | Mascot |
| 1210.6578 | 1210.6071 | -0.0507 | -42 | 127 | 136 | LVAQFSNIYR | | | | | Mascot |
| 1365.6515 | 1365.6426 | -0.0089 | -7 | 1607 | 1617 | MFLSHASCRTR | | | Carbamidomethyl (C)[8] | | Mascot |
| 1475.7965 | 1475.7635 | -0.033 | -22 | 1457 | 1468 | EAEIISFRLNQR | | | | | Mascot |
| 1542.7546 | 1542.8931 | 0.1385 | 90 | 169 | 181 | AFVSDLEFRSSER | | | | | Mascot |
| 1719.9349 | 1719.8651 | -0.0698 | -41 | 363 | 377 | IVNSPYMLTIKDTPK | | | | | Mascot |
| 1719.9349 | 1719.8651 | -0.0698 | -41 | 363 | 377 | IVNSPYMLTIKDTPK | | | | | Mascot |
| 1735.9299 | 1735.8551 | -0.0748 | -43 | 363 | 377 | IVNSPYMLTIKDTPK | | | Oxidation (M)[7] | | Mascot |
| 1736.9581 | 1736.8948 | -0.0633 | -36 | 285 | 300 | GVAYVLFGIEINGEKK | | | | | Mascot |
| 1908.9714 | 1908.9456 | -0.0258 | -14 | 1310 | 1325 | SDVRWTFNPNLAYVAR | | | | | Mascot |
| 1951.7653 | 1951.9689 | 0.2036 | 104 | 1515 | 1532 | CSQGDCCVMKEDSASFGK | | | Carbamidomethyl (C)[1] | | Mascot |

| | | | | | | | | | | | |
|---|---|---|---|---|---|---|---|---|---|---|---|
| 2000.0851 | 1999.9546 | -0.1305 | -65 | 1471 | 1487 | VGLLQPSTVKVYQYYNK | | | | | Mascot |
| 2000.0851 | 1999.9546 | -0.1305 | -65 | 1471 | 1487 | VGLLQPSTVKVYQYYNK | | | | | Mascot |
| 2175.1738 | 2175.1536 | -0.0202 | -9 | 1 | 19 | MLLQLLLWATLLCQICDGK | | | | | Mascot |
| 2175.1738 | 2175.1536 | -0.0202 | -9 | 1 | 19 | MLLQLLLWATLLCQICDGK | | | | | Mascot |
| 2233.1533 | 2233.105 | -0.0483 | -22 | 911 | 932 | TGELPLQVTAVAASFMGQDAVK | | | | | Mascot |
| 2448.0442 | 2448.1565 | 0.1123 | 46 | 650 | 673 | DNMGVFSDAGLMFHSSTGGSTDSK | | | | | Mascot |
| 2489.2881 | 2489.1013 | -0.1868 | -75 | 980 | 1002 | SVPETFVNVRGDLLADSIDNSIK | | | | | Mascot |
| 2531.3542 | 2531.2527 | -0.1015 | -40 | 277 | 299 | YLYGAQVKGVAYVLFGIEINGEK | | | | | Mascot |
| 3103.6091 | 3103.6582 | 0.0491 | 16 | 1146 | 1173 | ASLTAFVLIALAEAQDKVTCQDQEVNIR | | | Carbamidomethyl (C)[20] | | Mascot |

34 UBX domain-containing protein 8 [Danio rerio] gi|66773122 31217 7.64 4 33 0

**Peptide Information**

| Calc. Mass | Obsrv. Mass | ± da | ± ppm | Start Seq. | End Seq. | Sequence | Ion Score | C. I. % | Modification | Rank | Result Type |
|---|---|---|---|---|---|---|---|---|---|---|---|
| 901.5101 | 901.5135 | 0.0034 | 4 | 76 | 82 | LKEEQVR | 22 | 0 | | | Mascot |
| 901.5101 | 901.5135 | 0.0034 | 4 | 76 | 82 | LKEEQVR | | | | | Mascot |
| 1767.8079 | 1767.8225 | 0.0146 | 8 | 139 | 154 | ICHAVDVDESPNQGAR | | | Carbamidomethyl (C)[2] | | Mascot |
| 1908.92 | 1908.9456 | 0.0256 | 13 | 225 | 240 | TGYPPALYTLYMSYPR | | | Oxidation (M)[12] | | Mascot |
| 1924.0386 | 1923.98 | -0.0586 | -30 | 177 | 194 | RVIVLPDEPSVDTEGAVK | | | | | Mascot |

35 vitellogenin 2 isoform 2 [Danio rerio] gi|160333705 93050.4 8.22 12 33 0

**Peptide Information**

| Calc. Mass | Obsrv. Mass | ± da | ± ppm | Start Seq. | End Seq. | Sequence | Ion Score | C. I. % | Modification | Rank | Result Type |
|---|---|---|---|---|---|---|---|---|---|---|---|
| 850.4702 | 850.5455 | 0.0753 | 89 | 750 | 756 | MKLESVK | | | Oxidation (M)[1] | | Mascot |
| 864.3945 | 864.4554 | 0.0609 | 70 | 612 | 618 | KDEESEK | | | | | Mascot |
| 1112.5769 | 1112.5123 | -0.0646 | -58 | 322 | 330 | TLTKMEAFR | | | Oxidation (M)[5] | | Mascot |
| 1365.6831 | 1365.6426 | -0.0405 | -30 | 428 | 439 | VTSKVTWGAECK | | | Carbamidomethyl (C)[11] | | Mascot |
| 1736.0356 | 1735.8551 | -0.1805 | -104 | 110 | 125 | WLLDTLPAVATPVVLK | | | | | Mascot |
| 1909.1117 | 1908.9456 | -0.1661 | -87 | 251 | 269 | TTASALPIGVQVDAILALR | | | | | Mascot |
| 1951.9187 | 1951.9689 | 0.0502 | 26 | 692 | 708 | IQVADWMKGQTCGLCGK | | | Carbamidomethyl (C)[12,15] | | Mascot |
| 2233.2446 | 2233.105 | -0.1396 | -63 | 171 | 191 | IATIPALREVVMLGYGSMIAK | | | | | Mascot |
| 2294.3706 | 2294.178 | -0.1926 | -84 | 278 | 297 | LVQPVALQLVLERALHPELR | | | | | Mascot |
| 2298.1655 | 2298.198 | 0.0325 | 14 | 298 | 318 | MVACILLFETKPSVAAMSSSR | | | Carbamidomethyl (C)[4] | | Mascot |
| 2457.3235 | 2457.3267 | 0.0032 | 1 | 619 | 640 | THLNVKLVDIDIDLYTLGT | | | | | Mascot |

| | | | | | | | | | | | |
|---|---|---|---|---|---|---|---|---|---|---|---|
| | | | | | | DAK | | | | | |
| 2457.3235 | 2457.3267 | 0.0032 | 1 | 619 | 640 | THLNVKLVDIDIDLYTLGTDAK | | | | | Mascot |
| 2479.2974 | 2479.3062 | 0.0088 | 4 | 1 | 22 | MMEATQSLVFVEFEKAPVVPVK | | | | | Mascot |

36 serine/threonine kinase 33 [Danio rerio] gi|220678423 8056.3 10.88 4 32 0

Peptide Information

| Calc. Mass | Obsrv. Mass | ± da | ± ppm | Start Seq. | End Seq. | Sequence | Ion Score | C. I. % | Modification | Rank | Result Type |
|---|---|---|---|---|---|---|---|---|---|---|---|
| 864.4421 | 864.4554 | 0.0133 | 15 | 16 | 23 | ETSTAVTR | | | | | Mascot |
| 901.4387 | 901.5135 | 0.0748 | 83 | 24 | 30 | TNHHHQK | | | | | Mascot |
| 901.4387 | 901.5135 | 0.0748 | 83 | 24 | 30 | TNHHHQK | | | | | Mascot |
| 1342.6863 | 1342.6414 | -0.0449 | -33 | 31 | 42 | HPLQHPTQAGEK | | | | | Mascot |
| 2225.1072 | 2225.1333 | 0.0261 | 12 | 24 | 42 | TNHHHQKHPLQHPTQAGEK | | | | | Mascot |

37 PREDICTED: b-cell lymphoma/leukemia 11B-like [Danio rerio] gi|326676947 84089.7 6.78 11 32 0

Peptide Information

| Calc. Mass | Obsrv. Mass | ± da | ± ppm | Start Seq. | End Seq. | Sequence | Ion Score | C. I. % | Modification | Rank | Result Type |
|---|---|---|---|---|---|---|---|---|---|---|---|
| 842.4227 | 842.5153 | 0.0926 | 110 | 463 | 469 | NRENGPR | | | | | Mascot |
| 1112.6245 | 1112.5123 | -0.1122 | -101 | 99 | 108 | LTPARGICPK | | | Carbamidomethyl (C)[8] | | Mascot |
| 1436.6184 | 1436.7474 | 0.129 | 90 | 666 | 677 | SDTCEYCGKVFK | | | Carbamidomethyl (C)[4] | | Mascot |
| 1488.8209 | 1488.793 | -0.0279 | -19 | 213 | 226 | LPSTPPFVSPPPPR | | | | | Mascot |
| 1488.8209 | 1488.793 | -0.0279 | -19 | 213 | 226 | LPSTPPFVSPPPPR | | | | | Mascot |
| 1707.8007 | 1707.7886 | -0.0121 | -7 | 623 | 639 | FSTPPGEILEGGGMSGR | | | Oxidation (M)[14] | | Mascot |
| 1719.9058 | 1719.8651 | -0.0407 | -24 | 541 | 557 | KPMPISSSSLSGSSAKR | | | | | Mascot |
| 1719.9058 | 1719.8651 | -0.0407 | -24 | 541 | 557 | KPMPISSSSLSGSSAKR | 3 | 0 | | | Mascot |
| 1735.9008 | 1735.8551 | -0.0457 | -26 | 541 | 557 | KPMPISSSSLSGSSAKR | | | Oxidation (M)[3] | | Mascot |
| 1862.8193 | 1863.073 | 0.2537 | 136 | 697 | 712 | CDLCSYACAQSSKLTR | | | Carbamidomethyl (C)[1,4] | | Mascot |
| 2294.0767 | 2294.178 | 0.1013 | 44 | 727 | 745 | CDICHMPFSVYSTLEKHVK | | | Carbamidomethyl (C)[1] | | Mascot |
| 2384.1848 | 2384.0769 | -0.1079 | -45 | 232 | 252 | LGPEEMGLLSQHPSAFERVMR | | | | | Mascot |
| 2515.1023 | 2515.2617 | 0.1594 | 63 | 359 | 380 | SHTGERPYKCHLCDHACSQASK | | | Carbamidomethyl (C)[10] | | Mascot |
| 3103.6067 | 3103.6582 | 0.0515 | 17 | 149 | 177 | IYLDTHPTSCSLTPRMALPPPLGADALPR | | | | | Mascot |

38 novel protein similar to vertebrate Rho guanine nucleotide exchange factor (GEF) 3 (ARHGEF3) [Danio gi|56207664 62009.9 8.98 10 32 0

Peptide Information

| Calc. Mass | Obsrv. Mass | ± da | ± ppm | Start Seq. | End Seq. | Sequence | Ion Score | C. I. % | Modification | Rank | Result Type |
|---|---|---|---|---|---|---|---|---|---|---|---|
| 1045.5095 | 1045.5679 | 0.0584 | 56 | 323 | 330 | DPRIEQCK | | | Carbamidomethyl (C)[7] | | Mascot |
| 1060.5356 | 1060.4849 | -0.0507 | -48 | 435 | 442 | QQWLNCLR | | | | | Mascot |
| 1165.6099 | 1165.7528 | 0.1429 | 123 | 314 | 322 | LEYLEDKQK | | | | | Mascot |
| 1488.7878 | 1488.793 | 0.0052 | 3 | 2 | 14 | VAYDEPCVVPIKR | | | | | Mascot |
| 1488.7878 | 1488.793 | 0.0052 | 3 | 2 | 14 | VAYDEPCVVPIKR | | | | | Mascot |
| 1736.8999 | 1736.8948 | -0.0051 | -3 | 210 | 225 | AYCSNQLAAKALLDQK | | | | | Mascot |
| 1893.9058 | 1893.9919 | 0.0861 | 45 | 435 | 449 | QQWLNCLRNAMSTQK | | | Carbamidomethyl (C)[6], Oxidation (M)[11] | | Mascot |
| 1893.9058 | 1893.9919 | 0.0861 | 45 | 435 | 449 | QQWLNCLRNAMSTQK | | | Carbamidomethyl (C)[6], Oxidation (M)[11] | | Mascot |
| 2175.0486 | 2175.1536 | 0.105 | 48 | 450 | 469 | DLTLSENEPLTTPATDVC AK | | | Carbamidomethyl (C)[18] | | Mascot |
| 2175.0486 | 2175.1536 | 0.105 | 48 | 450 | 469 | DLTLSENEPLTTPATDVC AK | | | Carbamidomethyl (C)[18] | | Mascot |
| 2224.9954 | 2225.1333 | 0.1379 | 62 | 303 | 320 | GESECQYYIDKLEYLEDK | | | | | Mascot |
| 2233.1345 | 2233.105 | -0.0295 | -13 | 121 | 139 | QEAIFELSRGEQDLIEDLK | | | | | Mascot |
| 2284.0002 | 2284.1877 | 0.1875 | 82 | 471 | 490 | RSSSVSTVIQMDEMDEN CPR | | | | | Mascot |
| 2357.0166 | 2357.1431 | 0.1265 | 54 | 471 | 490 | RSSSVSTVIQMDEMDEN CPR | | | Carbamidomethyl (C)[18], Oxidation (M)[11] | | Mascot |

39 DNA mismatch repair protein Msh3 [Danio rerio] gi|157841276 121710.5 6.91 10 32 0

**Peptide Information**

| Calc. Mass | Obsrv. Mass | ± da | ± ppm | Start Seq. | End Seq. | Sequence | Ion Score | C. I. % | Modification | Rank | Result Type |
|---|---|---|---|---|---|---|---|---|---|---|---|
| 842.4981 | 842.5153 | 0.0172 | 20 | 150 | 156 | EEPIKVK | | | | | Mascot |
| 870.577 | 870.5441 | -0.0329 | -38 | 687 | 693 | EVRLLLK | | | | | Mascot |
| 901.5101 | 901.5135 | 0.0034 | 4 | 545 | 552 | SVIDIQAR | 17 | 0 | | | Mascot |
| 901.5101 | 901.5135 | 0.0034 | 4 | 545 | 552 | SVIDIQAR | | | | | Mascot |
| 1076.5669 | 1076.4808 | -0.0861 | -80 | 583 | 591 | GLCSIYHKR | | | | | Mascot |
| 1179.5535 | 1179.6071 | 0.0536 | 45 | 895 | 905 | MGASDNISRGR | | | Oxidation (M)[1] | | Mascot |
| 1210.6613 | 1210.6071 | -0.0542 | -45 | 477 | 486 | ILLCSSSFKR | | | Carbamidomethyl (C)[4] | | Mascot |
| 1475.7562 | 1475.7635 | 0.0073 | 5 | 713 | 725 | NSMLSIVPADWVK | | | Oxidation (M)[3] | | Mascot |
| 1707.7828 | 1707.7886 | 0.0058 | 3 | 201 | 214 | QHVDTVLCVECGYK | | | Carbamidomethyl (C)[8,11] | | Mascot |
| 1901.9565 | 1902.0004 | 0.0439 | 23 | 186 | 200 | TIYTPLEEQYMEIKK | | | Oxidation (M)[11] | | Mascot |
| 2432.1431 | 2432.1536 | 0.0105 | 4 | 904 | 925 | GRSTFMEELLETSDVLAC ATSR | | | Oxidation (M)[6] | | Mascot |
| 2432.1431 | 2432.1536 | 0.0105 | 4 | 904 | 925 | GRSTFMEELLETSDVLAC ATSR | | | Oxidation (M)[6] | | Mascot |
| 2489.1646 | 2489.1013 | -0.0633 | -25 | 904 | 925 | GRSTFMEELLETSDVLAC ATSR | | | Carbamidomethyl (C)[18], Oxidation (M)[6] | | Mascot |

40 novel protein (zgc:101790) [Danio rerio] gi|148726473 30484.8 7.21 1 32 0 32 84.343

Peptide Information

| Calc. Mass | Obsrv. Mass | ± da | ± ppm | Start Seq. | End Seq. | Sequence | Ion Score | C. I. % | Modification | Rank | Result Type |
|---|---|---|---|---|---|---|---|---|---|---|---|
| 901.5464 | 901.5135 | -0.0329 | -36 | 176 | 183 | TGEIVKVR | 32 | 84.343 | | | Mascot |
| 901.5464 | 901.5135 | -0.0329 | -36 | 176 | 183 | TGEIVKVR | | | | | Mascot |

41 PREDICTED: NACHT, LRR and PYD domains-containing protein 12 [Danio rerio] gi|292621731 149261.8 5.29 15 32 0

Peptide Information

| Calc. Mass | Obsrv. Mass | ± da | ± ppm | Start Seq. | End Seq. | Sequence | Ion Score | C. I. % | Modification | Rank | Result Type |
|---|---|---|---|---|---|---|---|---|---|---|---|
| 842.4114 | 842.5153 | 0.1039 | 123 | 1214 | 1220 | SNSEHLR | | | | | Mascot |
| 864.5777 | 864.4554 | -0.1223 | -141 | 133 | 139 | KILVVHR | | | | | Mascot |
| 870.5593 | 870.5441 | -0.0152 | -17 | 1011 | 1017 | KLLPMIR | | | | | Mascot |
| 901.5829 | 901.5135 | -0.0694 | -77 | 507 | 514 | AIRTVLTK | | | | | Mascot |
| 901.5829 | 901.5135 | -0.0694 | -77 | 507 | 514 | AIRTVLTK | | | | | Mascot |
| 959.5156 | 959.5181 | 0.0025 | 3 | 872 | 880 | AVNEALQSK | | | | | Mascot |
| 1076.5007 | 1076.4808 | -0.0199 | -18 | 741 | 748 | HQKDYEEK | | | | | Mascot |
| 1436.7744 | 1436.7474 | -0.027 | -19 | 913 | 924 | SIQNNKTVEYIK | | | | | Mascot |
| 1488.7289 | 1488.793 | 0.0641 | 43 | 375 | 388 | SGDTKSDLSSVHQK | | | | | Mascot |
| 1488.7289 | 1488.793 | 0.0641 | 43 | 375 | 388 | SGDTKSDLSSVHQK | | | | | Mascot |
| 1880.884 | 1880.9967 | 0.1127 | 60 | 1081 | 1099 | LSNCGLTDEGCSALASAL R | | | | | Mascot |
| 1916.0157 | 1915.9689 | -0.0468 | -24 | 1115 | 1132 | LGDSVTLLSAVLEDPRCK | | | | | Mascot |
| 2175.0896 | 2175.1536 | 0.064 | 29 | 1250 | 1270 | LGLSNCGLTDEGCAALAL ALR | | | Carbamidomethyl (C)[6,13] | | Mascot |
| 2175.0896 | 2175.1536 | 0.064 | 29 | 1250 | 1270 | LGLSNCGLTDEGCAALAL ALR | | | Carbamidomethyl (C)[6,13] | | Mascot |
| 2225.2839 | 2225.1333 | -0.1506 | -68 | 890 | 908 | FLLGLSVESHQILLQRLM K | | | | | Mascot |
| 2284.0181 | 2284.1877 | 0.1696 | 74 | 380 | 400 | SDLSSVHQKSTESESSC VSMR | | | | | Mascot |
| 2357.0344 | 2357.1431 | 0.1087 | 46 | 380 | 400 | SDLSSVHQKSTESESSC VSMR | | | Carbamidomethyl (C)[17], Oxidation (M)[20] | | Mascot |
| 2448.2002 | 2448.1565 | -0.0437 | -18 | 414 | 435 | SGDTETDLSHEALNTFRS NLLK | | | | | Mascot |
| 2479.0752 | 2479.3062 | 0.231 | 93 | 277 | 297 | KDFCSADQEELLETLDNY SCR | | | | | Mascot |

42 novel protein [Danio rerio] gi|169146110 25865.2 5.07 7 32 0

Peptide Information

| Calc. Mass | Obsrv. Mass | ± da | ± ppm | Start Seq. | End Seq. | Sequence | Ion Score | C. I. % | Modification | Rank | Result Type |
|---|---|---|---|---|---|---|---|---|---|---|---|

| Calc. Mass | Obsrv. Mass | ± da | ± ppm | Start Seq. | End Seq. | Sequence | Ion Score | C. I. % | Modification | Rank | Result Type |
|---|---|---|---|---|---|---|---|---|---|---|---|
| 1045.5411 | 1045.5679 | 0.0268 | 26 | 99 | 107 | VIADLEEEK | | | | | Mascot |
| 1076.5331 | 1076.4808 | -0.0523 | -49 | 207 | 215 | GELESERTR | | | | | Mascot |
| 1112.6786 | 1112.5123 | -0.1663 | -149 | 85 | 94 | LLKQLAAAER | | | | | Mascot |
| 1179.5319 | 1179.6071 | 0.0752 | 64 | 73 | 82 | LVMAHCTNMK | | | Oxidation (M)[3,9] | | Mascot |
| 1436.6694 | 1436.7474 | 0.078 | 54 | 73 | 84 | LVMAHCTNMKEK | | | Oxidation (M)[3,9] | | Mascot |
| 1707.749 | 1707.7886 | 0.0396 | 23 | 111 | 126 | AQDAAAGDDITDMLER | | | Oxidation (M)[13] | | Mascot |
| 2432.2515 | 2432.1536 | -0.0979 | -40 | 150 | 172 | SVLSSQAVEDLAEQQQL SSTLAK | | | | | Mascot |
| 2432.2515 | 2432.1536 | -0.0979 | -40 | 150 | 172 | SVLSSQAVEDLAEQQQL SSTLAK | | | | | Mascot |

| | | | | | | | |
|---|---|---|---|---|---|---|---|
| 43 | si:ch1073-126c3.2 [Danio rerio] | gi\|221307488 | 24754.2 | 6.97 | 6 | 32 | 0 |

**Peptide Information**

| Calc. Mass | Obsrv. Mass | ± da | ± ppm | Start Seq. | End Seq. | Sequence | Ion Score | C. I. % | Modification | Rank | Result Type |
|---|---|---|---|---|---|---|---|---|---|---|---|
| 864.4243 | 864.4554 | 0.0311 | 36 | 1 | 8 | MESATIGR | | | | | Mascot |
| 1128.5242 | 1128.5116 | -0.0126 | -11 | 134 | 143 | LFEECSTATK | | | | | Mascot |
| 1951.9277 | 1951.9689 | 0.0412 | 21 | 204 | 221 | VETTESESTKLLCGNINN | | | | | Mascot |
| 2211.0295 | 2211.1179 | 0.0884 | 40 | 114 | 131 | IYCKPMCNAGYDFNFLRR | | | | | Mascot |
| 2284.0459 | 2284.1877 | 0.1418 | 62 | 114 | 131 | IYCKPMCNAGYDFNFLRR | | | Carbamidomethyl (C)[3], Oxidation (M)[6] | | Mascot |
| 2298.0503 | 2298.198 | 0.1477 | 64 | 112 | 130 | DKIYCKPMCNAGYDFNFL R | | | | | Mascot |
| 2448.2625 | 2448.1565 | -0.106 | -43 | 89 | 111 | LLPENCPAPAVPENGGLL CLSVK | | | Carbamidomethyl (C)[6,19] | | Mascot |

| | | | | | | | |
|---|---|---|---|---|---|---|---|
| 44 | PREDICTED: DNA mismatch repair protein Msh3-like [Danio rerio] | gi\|326668008 | 121301.1 | 6.7 | 9 | 32 | 0 |

**Peptide Information**

| Calc. Mass | Obsrv. Mass | ± da | ± ppm | Start Seq. | End Seq. | Sequence | Ion Score | C. I. % | Modification | Rank | Result Type |
|---|---|---|---|---|---|---|---|---|---|---|---|
| 870.577 | 870.5441 | -0.0329 | -38 | 687 | 693 | EVRLLLK | | | | | Mascot |
| 901.5101 | 901.5135 | 0.0034 | 4 | 545 | 552 | SVIDIQAR | 17 | 0 | | | Mascot |
| 901.5101 | 901.5135 | 0.0034 | 4 | 545 | 552 | SVIDIQAR | | | | | Mascot |
| 1179.5535 | 1179.6071 | 0.0536 | 45 | 895 | 905 | MGASDNISRGR | | | Oxidation (M)[1] | | Mascot |
| 1210.6613 | 1210.6071 | -0.0542 | -45 | 477 | 486 | ILLCSSSFKR | | | Carbamidomethyl (C)[4] | | Mascot |
| 1475.7529 | 1475.7635 | 0.0106 | 7 | 155 | 166 | IKQEFSFSQYAK | | | | | Mascot |
| 1707.7828 | 1707.7886 | 0.0058 | 3 | 201 | 214 | QHVDTVLCVECGYK | | | Carbamidomethyl (C)[8,11] | | Mascot |
| 1862.9099 | 1863.073 | 0.1631 | 88 | 486 | 502 | RLSSDSDHMLLSAATMK | | | | | Mascot |
| 1901.9565 | 1902.0004 | 0.0439 | 23 | 186 | 200 | TIYTPLEEQYMEIKK | | | Oxidation (M)[11] | | Mascot |
| 2432.1431 | 2432.1536 | 0.0105 | 4 | 904 | 925 | GRSTFMEELLETSDVLAC ATSR | | | Oxidation (M)[6] | | Mascot |

| | | | | | | | | | | | | |
|---|---|---|---|---|---|---|---|---|---|---|---|---|
| 2432.1431 | 2432.1536 | 0.0105 | 4 | 904 | 925 | GRSTFMEELLETSDVLACATSR | | | Oxidation (M)[6] | | Mascot |
| 2489.1646 | 2489.1013 | -0.0633 | -25 | 904 | 925 | GRSTFMEELLETSDVLACATSR | | | Carbamidomethyl (C)[18], Oxidation (M)[6] | | Mascot |

45 nitrogen fixation cluster-like [Danio rerio] gi|221307563 17420.2 8.83 6 32 0

Peptide Information

| Calc. Mass | Obsrv. Mass | ± da | ± ppm | Start Seq. | End Seq. | Sequence | Ion Score | C. I. % | Modification | Rank | Result Type |
|---|---|---|---|---|---|---|---|---|---|---|---|
| 882.5294 | 882.5723 | 0.0429 | 49 | 122 | 129 | ELSLPPVK | | | | | Mascot |
| 1028.5922 | 1028.5013 | -0.0909 | -88 | 8 | 16 | KCAAPQILK | | | Carbamidomethyl (C)[2] | | Mascot |
| 1045.5636 | 1045.5679 | 0.0043 | 4 | 42 | 51 | NVGSLDKNAK | | | | | Mascot |
| 1128.5432 | 1128.5116 | -0.0316 | -28 | 33 | 41 | VVDHYENPR | | | | | Mascot |
| 1901.9572 | 1902.0004 | 0.0432 | 23 | 49 | 68 | NAKNVGTGLVGAPACGDVMK | | | | | Mascot |
| 2157.0645 | 2157.1462 | 0.0817 | 38 | 86 | 106 | TFGCGSAIASSSLATEWVKGK | | | Carbamidomethyl (C)[4] | | Mascot |

46 chloride intracellular channel protein 3 [Danio rerio] gi|41054774 27155 6.95 7 32 0

Peptide Information

| Calc. Mass | Obsrv. Mass | ± da | ± ppm | Start Seq. | End Seq. | Sequence | Ion Score | C. I. % | Modification | Rank | Result Type |
|---|---|---|---|---|---|---|---|---|---|---|---|
| 842.4011 | 842.5153 | 0.1142 | 136 | 95 | 100 | LCCRYK | | | Carbamidomethyl (C)[2] | | Mascot |
| 882.5117 | 882.5723 | 0.0606 | 69 | 132 | 138 | FLKSLMK | | | Oxidation (M)[6] | | Mascot |
| 1365.7446 | 1365.6426 | -0.102 | -75 | 1 | 12 | MAEAAKIELFVK | | | Oxidation (M)[1] | | Mascot |
| 1436.7994 | 1436.7474 | -0.052 | -36 | 223 | 235 | DKEILLAYSSVAK | | | | | Mascot |
| 1488.7151 | 1488.793 | 0.0779 | 52 | 213 | 224 | EDVFHLTCPKDK | | | Carbamidomethyl (C)[8] | | Mascot |
| 1488.7151 | 1488.793 | 0.0779 | 52 | 213 | 224 | EDVFHLTCPKDK | | | Carbamidomethyl (C)[8] | | Mascot |
| 2471.1328 | 2471.1096 | -0.0232 | -9 | 7 | 28 | IELFVKASDDGESVGNCPFCQR | | | Carbamidomethyl (C)[17] | | Mascot |
| 2479.1995 | 2479.3062 | 0.1067 | 43 | 79 | 98 | IEEFLEDTLAPPQYPKLCCR | | | Carbamidomethyl (C)[18,19] | | Mascot |

47 novel protein with a Viral A-type inclusion protein repeat domain [Danio rerio] gi|169146304 27380.3 5.44 7 32 0

Peptide Information

| Calc. Mass | Obsrv. Mass | ± da | ± ppm | Start Seq. | End Seq. | Sequence | Ion Score | C. I. % | Modification | Rank | Result Type |
|---|---|---|---|---|---|---|---|---|---|---|---|
| 901.4988 | 901.5135 | 0.0147 | 16 | 182 | 189 | AVEEVVQK | | | | | Mascot |
| 901.4988 | 901.5135 | 0.0147 | 16 | 182 | 189 | AVEEVVQK | | | | | Mascot |
| 1060.527 | 1060.4849 | -0.0421 | -40 | 23 | 31 | LQDELAESR | | | | | Mascot |
| 1103.515 | 1103.5774 | 0.0624 | 57 | 224 | 233 | QCHSLKTATD | | | | | Mascot |

| Calc. Mass | Obsrv. Mass | ± da | ± ppm | Start Seq. | End Seq. | Sequence | Ion Score | C. I. % | Modification | Rank | Result Type |
|---|---|---|---|---|---|---|---|---|---|---|---|
| 1488.7329 | 1488.793 | 0.0601 | 40 | 2 | 13 | ESELLIGWQEER | | | | | Mascot |
| 1488.7805 | 1488.793 | 0.0125 | 8 | 198 | 210 | EVLTGYIGKEHSR | | | | | Mascot |
| 1915.929 | 1915.9689 | 0.0399 | 21 | 101 | 116 | CQALEQQAVGEERELR | | | Carbamidomethyl (C)[1] | | Mascot |
| 2157.1509 | 2157.1462 | -0.0047 | -2 | 137 | 155 | LEEEQQRSLGLSEVSALL R | | | | | Mascot |

| Rank | Protein Name | Accession No. | Protein MW | Protein PI | Pep. Count | Protein Score | Protein Score C. I. % | Total Ion Score | Total Ion C. I. % |
|---|---|---|---|---|---|---|---|---|---|
| 48 | novel protein similar to H.sapiens GTF2F2, general transcription factor IIF, polypeptide 2, 30kDa (GTF2F2, zgc:110187) [Danio rerio] | gi\|62955637 | 29399.3 | 9.31 | 1 | | | 28 | 56.577 |

Peptide Information

| Calc. Mass | Obsrv. Mass | ± da | ± ppm | Start Seq. | End Seq. | Sequence | Ion Score | C. I. % | Modification | Rank | Result Type |
|---|---|---|---|---|---|---|---|---|---|---|---|
| 901.5214 | 901.5135 | -0.0079 | -9 | 73 | 80 | ISSVRSPR | 28 | 56.577 | | 9 | Mascot |

| Rank | Protein Name | Accession No. | Protein MW | Protein PI | Pep. Count | Protein Score | Protein Score C. I. % | Total Ion Score | Total Ion C. I. % |
|---|---|---|---|---|---|---|---|---|---|
| 49 | novel protein similar to vertebrate 2-deoxyribose-5-phosphate aldolase homolog (C. elegans) (DERA, zgc:101610) [Danio rerio] | gi\|169158628 | 35033.1 | 9.23 | 1 | | | 15 | 0 |

Peptide Information

| Calc. Mass | Obsrv. Mass | ± da | ± ppm | Start Seq. | End Seq. | Sequence | Ion Score | C. I. % | Modification | Rank | Result Type |
|---|---|---|---|---|---|---|---|---|---|---|---|
| 1488.7073 | 1488.793 | 0.0857 | 58 | 205 | 218 | ASLVSMMAGSDFIK | 15 | 0 | Oxidation (M)[6,7] | 2 | Mascot |

| Rank | Protein Name | Accession No. | Protein MW | Protein PI | Pep. Count | Protein Score | Protein Score C. I. % | Total Ion Score | Total Ion C. I. % |
|---|---|---|---|---|---|---|---|---|---|
| 50 | frizzled-related protein [Danio rerio] | gi\|18858723 | 36007.6 | 8.44 | 1 | | | 14 | 0 |

Peptide Information

| Calc. Mass | Obsrv. Mass | ± da | ± ppm | Start Seq. | End Seq. | Sequence | Ion Score | C. I. % | Modification | Rank | Result Type |
|---|---|---|---|---|---|---|---|---|---|---|---|
| 1354.8053 | 1354.7772 | -0.0281 | -21 | 233 | 244 | EVLKSSLVNIPR | 14 | 0 | | 3 | Mascot |

| | | | | | |
|---|---|---|---|---|---|
| **Gel Idx/Pos** | 193/H20 | **Instr./Gel Origin** | BA2060/110707A | **Process Status** | Analysis Succeeded |
| **Plate [#] Name** | [1] 31496 | **Instrument Sample Name** | | **Spectra** | 11 |

| Rank | Protein Name | Accession No. | Protein MW | Protein PI | Pep. Count | Protein Score | Protein Score C. I. % | Total Ion Score | Total Ion C. I. % |
|---|---|---|---|---|---|---|---|---|---|
| 1 | vitellogenin 1 [Danio rerio] | gi\|94733730 | 149140.4 | 8.74 | 30 | 982 | 100 | 818 | 100 |

Peptide Information

| Calc. Mass | Obsrv. Mass | ± da | ± ppm | Start Seq. | End Seq. | Sequence | Ion Score | C. I. % | Modification | Rank | Result Type |
|---|---|---|---|---|---|---|---|---|---|---|---|
| 821.4628 | 821.4716 | 0.0088 | 11 | 545 | 551 | ALHPEVR | | | | | Mascot |
| 821.4628 | 821.4716 | 0.0088 | 11 | 545 | 551 | ALHPEVR | 58 | 99.958 | | | Mascot |
| 874.4669 | 874.5159 | 0.049 | 56 | 87 | 94 | DPFVPATK | | | | | Mascot |

| | | | | | | | | | | |
|---|---|---|---|---|---|---|---|---|---|---|
| 888.5301 | 888.538 | 0.0079 | 9 | 425 | 432 | FATIPALR | | | | Mascot |
| 888.5301 | 888.538 | 0.0079 | 9 | 425 | 432 | FATIPALR | 44 | 98.855 | | Mascot |
| 918.4965 | 918.5496 | 0.0531 | 58 | 1319 | 1326 | IPEITMSK | | | | Mascot |
| 926.5417 | 926.5511 | 0.0094 | 10 | 505 | 513 | TAANALPIR | | | | Mascot |
| 1023.5105 | 1023.5204 | 0.0099 | 10 | 195 | 203 | DVGLAYTER | 79 | 100 | | Mascot |
| 1023.5105 | 1023.5204 | 0.0099 | 10 | 195 | 203 | DVGLAYTER | | | | Mascot |
| 1097.6677 | 1097.6796 | 0.0119 | 11 | 514 | 523 | VQVDAILALR | | | | Mascot |
| 1097.6677 | 1097.6796 | 0.0119 | 11 | 514 | 523 | VQVDAILALR | 98 | 100 | | Mascot |
| 1115.6935 | 1115.7063 | 0.0128 | 11 | 333 | 341 | FVQLVQLLR | 71 | 99.998 | | Mascot |
| 1115.6935 | 1115.7063 | 0.0128 | 11 | 333 | 341 | FVQLVQLLR | | | | Mascot |
| 1121.599 | 1121.6111 | 0.0121 | 11 | 348 | 356 | IEAIWSQFK | | | | Mascot |
| 1177.6462 | 1177.6561 | 0.0099 | 8 | 260 | 269 | QTLAFVEIEK | | | | Mascot |
| 1215.6005 | 1215.616 | 0.0155 | 13 | 164 | 173 | THYVINEDPK | | | | Mascot |
| 1226.6991 | 1226.71 | 0.0109 | 9 | 472 | 482 | NDIPEITLALK | | | | Mascot |
| 1238.7831 | 1238.7933 | 0.0102 | 8 | 135 | 145 | GILNILQLNLK | | | | Mascot |
| 1259.5797 | 1259.5946 | 0.0149 | 12 | 182 | 191 | SKDLSHCQER | | | Carbamidomethyl (C)[7] | Mascot |
| 1379.7603 | 1379.7687 | 0.0084 | 6 | 433 | 445 | EVVMLGYGSLIAK | | | | Mascot |
| 1382.7889 | 1382.7999 | 0.011 | 8 | 303 | 315 | ISDAPAQIVEVLK | | | | Mascot |
| 1395.7551 | 1395.7643 | 0.0092 | 7 | 433 | 445 | EVVMLGYGSLIAK | | | Oxidation (M)[4] | Mascot |
| 1463.8944 | 1463.91 | 0.0156 | 11 | 532 | 544 | LVQPVALQLVLDR | 84 | 100 | | Mascot |
| 1463.8944 | 1463.91 | 0.0156 | 11 | 532 | 544 | LVQPVALQLVLDR | | | | Mascot |
| 1622.8536 | 1622.8712 | 0.0176 | 11 | 668 | 683 | AYLAGAAADVIEFGVR | 151 | 100 | | Mascot |
| 1622.8536 | 1622.8712 | 0.0176 | 11 | 668 | 683 | AYLAGAAADVIEFGVR | | | | Mascot |
| 1644.717 | 1644.8524 | 0.1354 | 82 | 1142 | 1157 | DTSSGSAAASFEQMQK | | | | Mascot |
| 1666.8767 | 1666.894 | 0.0173 | 10 | 483 | 498 | VMGNAGHPSSLKPIMK | | | | Mascot |
| 1682.8717 | 1682.8958 | 0.0241 | 14 | 483 | 498 | VMGNAGHPSSLKPIMK | | | Oxidation (M)[2] | Mascot |
| 1695.8999 | 1695.9147 | 0.0148 | 9 | 119 | 134 | VFAPAGVSPTVMNLHR | | | | Mascot |
| 1699.9377 | 1699.9528 | 0.0151 | 9 | 34 | 49 | YEALLLGGLPQEGLAR | | | | Mascot |
| 1699.9377 | 1699.9528 | 0.0151 | 9 | 34 | 49 | YEALLLGGLPQEGLAR | 89 | 100 | | Mascot |
| 1706.025 | 1706.0416 | 0.0166 | 10 | 364 | 379 | WLLDALPAVGTPVIIK | | | | Mascot |
| 1711.8948 | 1711.912 | 0.0172 | 10 | 119 | 134 | VFAPAGVSPTVMNLHR | 4 | 0 | Oxidation (M)[12] | Mascot |
| 1711.8948 | 1711.912 | 0.0172 | 10 | 119 | 134 | VFAPAGVSPTVMNLHR | | | Oxidation (M)[12] | Mascot |
| 1796.9078 | 1796.9176 | 0.0098 | 5 | 1279 | 1293 | HILTAAYDTGFRFER | | | | Mascot |
| 1812.8877 | 1812.9053 | 0.0176 | 10 | 72 | 86 | LMDPLLYEYAGTWPK | | | Oxidation (M)[2] | Mascot |
| 1843.9159 | 1843.9298 | 0.0139 | 8 | 577 | 592 | IEPNMHVASFAYSHIK | | | | Mascot |
| 1859.9109 | 1859.9304 | 0.0195 | 10 | 577 | 592 | IEPNMHVASFAYSHIK | | | Oxidation (M)[5] | Mascot |

| | | | | | | | | | | | |
|---|---|---|---|---|---|---|---|---|---|---|---|
| 1954.8745 | 1954.8923 | 0.0178 | 9 | 147 | 163 | TQNIYEMQEAGAQGVCR | | | Carbamidomethyl (C)[16] | | Mascot |
| 1954.8745 | 1954.8923 | 0.0178 | 9 | 147 | 163 | TQNIYEMQEAGAQGVCR | 146 | 100 | Carbamidomethyl (C)[16] | | Mascot |
| 1970.8695 | 1970.8909 | 0.0214 | 11 | 147 | 163 | TQNIYEMQEAGAQGVCR | | | Carbamidomethyl (C)[16], Oxidation (M)[7] | | Mascot |
| 2082.9695 | 2082.9944 | 0.0249 | 12 | 146 | 163 | KTQNIYEMQEAGAQGVCR | | | Carbamidomethyl (C)[17] | | Mascot |
| 2326.2 | 2326.2205 | 0.0205 | 9 | 283 | 302 | GSLQYEFATEILQTPIQLMK | | | Oxidation (M)[19] | | Mascot |
| 2938.5164 | 2938.5325 | 0.0161 | 5 | 446 | 471 | YCVAVPTCPAELLRPIHEIATEAISK | | | Carbamidomethyl (C)[2,8] | | Mascot |

| | | | | | | | | | |
|---|---|---|---|---|---|---|---|---|---|
| 2 | novel protein similar to vitellogenin 1 (vg1) [Danio rerio] | gi\|94733731 | 149169.2 | 8.84 | 25 | 882 | 100 | 774 | 100 |

**Peptide Information**

| Calc. Mass | Obsrv. Mass | ± da | ± ppm | Start Seq. | End Seq. | Sequence | Ion Score | C. I. % | Modification | Rank | Result Type |
|---|---|---|---|---|---|---|---|---|---|---|---|
| 821.4628 | 821.4716 | 0.0088 | 11 | 549 | 555 | ALHPEVR | | | | | Mascot |
| 821.4628 | 821.4716 | 0.0088 | 11 | 549 | 555 | ALHPEVR | 58 | 99.958 | | | Mascot |
| 874.5145 | 874.5159 | 0.0014 | 2 | 429 | 436 | FASIPALR | | | | | Mascot |
| 918.4965 | 918.5496 | 0.0531 | 58 | 1321 | 1328 | IPEITMSK | | | | | Mascot |
| 1023.5105 | 1023.5204 | 0.0099 | 10 | 199 | 207 | DVGLAYTER | 79 | 100 | | | Mascot |
| 1023.5105 | 1023.5204 | 0.0099 | 10 | 199 | 207 | DVGLAYTER | | | | | Mascot |
| 1097.6677 | 1097.6796 | 0.0119 | 11 | 518 | 527 | VQVDAILALR | | | | | Mascot |
| 1097.6677 | 1097.6796 | 0.0119 | 11 | 518 | 527 | VQVDAILALR | 98 | 100 | | | Mascot |
| 1115.6935 | 1115.7063 | 0.0128 | 11 | 337 | 345 | FVQLVQLLR | 71 | 99.998 | | | Mascot |
| 1115.6935 | 1115.7063 | 0.0128 | 11 | 337 | 345 | FVQLVQLLR | | | | | Mascot |
| 1121.599 | 1121.6111 | 0.0121 | 11 | 352 | 360 | IEAIWSQFK | | | | | Mascot |
| 1177.6462 | 1177.6561 | 0.0099 | 8 | 264 | 273 | QTLAFVEIEK | | | | | Mascot |
| 1215.6005 | 1215.616 | 0.0155 | 13 | 168 | 177 | THYVINEDPK | | | | | Mascot |
| 1226.6991 | 1226.71 | 0.0109 | 9 | 476 | 486 | NDIPEITLALK | | | | | Mascot |
| 1238.7831 | 1238.7933 | 0.0102 | 8 | 139 | 149 | GILNILQLNLK | | | | | Mascot |
| 1259.5797 | 1259.5946 | 0.0149 | 12 | 186 | 195 | SKDLSHCQER | | | Carbamidomethyl (C)[7] | | Mascot |
| 1379.7603 | 1379.7687 | 0.0084 | 6 | 437 | 449 | EVVMLGYGSLIAK | | | | | Mascot |
| 1395.7551 | 1395.7643 | 0.0092 | 7 | 437 | 449 | EVVMLGYGSLIAK | | | Oxidation (M)[4] | | Mascot |
| 1463.8944 | 1463.91 | 0.0156 | 11 | 536 | 548 | LVQPVALQLVLDR | 84 | 100 | | | Mascot |
| 1463.8944 | 1463.91 | 0.0156 | 11 | 536 | 548 | LVQPVALQLVLDR | | | | | Mascot |
| 1622.8536 | 1622.8712 | 0.0176 | 11 | 672 | 687 | AYLAGAAADVIEFGVR | 151 | 100 | | | Mascot |
| 1622.8536 | 1622.8712 | 0.0176 | 11 | 672 | 687 | AYLAGAAADVIEFGVR | | | | | Mascot |
| 1644.717 | 1644.8524 | 0.1354 | 82 | 1144 | 1159 | DTSSGSAAASFEQMQK | | | | | Mascot |
| 1648.821 | 1648.8413 | 0.0203 | 12 | 1201 | 1215 | VQLIASSIAENDNMK | | | Oxidation (M)[14] | | Mascot |
| 1666.8767 | 1666.894 | 0.0173 | 10 | 487 | 502 | VMGNAGHPSSLKPIMK | | | | | Mascot |

| | | | | | | | | | | | |
|---|---|---|---|---|---|---|---|---|---|---|---|
| 1682.8717 | 1682.8958 | 0.0241 | 14 | 487 | 502 | VMGNAGHPSSLKPIMK | | | Oxidation (M)[2] | | Mascot |
| 1699.9377 | 1699.9528 | 0.0151 | 9 | 38 | 53 | YEALLLGGLPQEGLAR | | | | | Mascot |
| 1699.9377 | 1699.9528 | 0.0151 | 9 | 38 | 53 | YEALLLGGLPQEGLAR | 89 | 100 | | | Mascot |
| 1706.025 | 1706.0416 | 0.0166 | 10 | 368 | 383 | WLLDALPAVGTPVIIK | | | | | Mascot |
| 1721.9043 | 1721.9209 | 0.0166 | 10 | 123 | 138 | VFAPAGVSPTVMNLYR | | | | | Mascot |
| 1737.8992 | 1737.9172 | 0.018 | 10 | 123 | 138 | VFAPAGVSPTVMNLYR | | | Oxidation (M)[12] | | Mascot |
| 1824.8989 | 1824.9142 | 0.0153 | 8 | 76 | 90 | LMDPLLYEYAGTWPR | | | | | Mascot |
| 1840.8938 | 1840.9137 | 0.0199 | 11 | 76 | 90 | LMDPLLYEYAGTWPR | | | Oxidation (M)[2] | | Mascot |
| 1954.8745 | 1954.8923 | 0.0178 | 9 | 151 | 167 | TQNIYEMQEAGAQGVCR | | | Carbamidomethyl (C)[16] | | Mascot |
| 1954.8745 | 1954.8923 | 0.0178 | 9 | 151 | 167 | TQNIYEMQEAGAQGVCR | 146 | 100 | Carbamidomethyl (C)[16] | | Mascot |
| 1970.8695 | 1970.8909 | 0.0214 | 11 | 151 | 167 | TQNIYEMQEAGAQGVCR | | | Carbamidomethyl (C)[16], Oxidation (M)[7] | | Mascot |
| 2082.9695 | 2082.9944 | 0.0249 | 12 | 150 | 167 | KTQNIYEMQEAGAQGVC R | | | Carbamidomethyl (C)[17] | | Mascot |
| 2914.4912 | 2914.5107 | 0.0195 | 7 | 450 | 475 | HCTAVPTCPAELLRPIHEI ATEAISK | | | Carbamidomethyl (C)[2,8] | | Mascot |

3 vitellogenin 5 [Danio rerio] gi|68448530 148868 8.77 25 881 100 774 100

**Peptide Information**

| Calc. Mass | Obsrv. Mass | ± da | ± ppm | Start Seq. | End Seq. | Sequence | Ion Score | C. I. % | Modification | Rank | Result Type |
|---|---|---|---|---|---|---|---|---|---|---|---|
| 821.4628 | 821.4716 | 0.0088 | 11 | 545 | 551 | ALHPEVR | | | | | Mascot |
| 821.4628 | 821.4716 | 0.0088 | 11 | 545 | 551 | ALHPEVR | 58 | 99.958 | | | Mascot |
| 874.5145 | 874.5159 | 0.0014 | 2 | 425 | 432 | FASIPALR | | | | | Mascot |
| 918.4965 | 918.5496 | 0.0531 | 58 | 1319 | 1326 | IPEITMSK | | | | | Mascot |
| 1023.5105 | 1023.5204 | 0.0099 | 10 | 195 | 203 | DVGLAYTER | 79 | 100 | | | Mascot |
| 1023.5105 | 1023.5204 | 0.0099 | 10 | 195 | 203 | DVGLAYTER | | | | | Mascot |
| 1097.6677 | 1097.6796 | 0.0119 | 11 | 514 | 523 | VQVDAILALR | | | | | Mascot |
| 1097.6677 | 1097.6796 | 0.0119 | 11 | 514 | 523 | VQVDAILALR | 98 | 100 | | | Mascot |
| 1115.6935 | 1115.7063 | 0.0128 | 11 | 333 | 341 | FVQLVQLLR | 71 | 99.998 | | | Mascot |
| 1115.6935 | 1115.7063 | 0.0128 | 11 | 333 | 341 | FVQLVQLLR | | | | | Mascot |
| 1121.599 | 1121.6111 | 0.0121 | 11 | 348 | 356 | IEAIWSQFK | | | | | Mascot |
| 1177.6462 | 1177.6561 | 0.0099 | 8 | 260 | 269 | QTLAFVEIEK | | | | | Mascot |
| 1215.6005 | 1215.616 | 0.0155 | 13 | 164 | 173 | THYVINEDPK | | | | | Mascot |
| 1226.6991 | 1226.71 | 0.0109 | 9 | 472 | 482 | NDIPEITLALK | | | | | Mascot |
| 1238.7831 | 1238.7933 | 0.0102 | 8 | 135 | 145 | GILNILQLNLK | | | | | Mascot |
| 1259.5797 | 1259.5946 | 0.0149 | 12 | 182 | 191 | SKDLSHCQER | | | Carbamidomethyl (C)[7] | | Mascot |
| 1379.7603 | 1379.7687 | 0.0084 | 6 | 433 | 445 | EVVMLGYGSLIAK | | | | | Mascot |
| 1395.7551 | 1395.7643 | 0.0092 | 7 | 433 | 445 | EVVMLGYGSLIAK | | | Oxidation (M)[4] | | Mascot |
| 1463.8944 | 1463.91 | 0.0156 | 11 | 532 | 544 | LVQPVALQLVLDR | 84 | 100 | | | Mascot |

| | | | | | | | | | | | |
|---|---|---|---|---|---|---|---|---|---|---|---|
| 1463.8944 | 1463.91 | 0.0156 | 11 | 532 | 544 | LVQPVALQLVLDR | | | | | Mascot |
| 1622.8536 | 1622.8712 | 0.0176 | 11 | 668 | 683 | AYLAGAAADVIEFGVR | 151 | 100 | | | Mascot |
| 1622.8536 | 1622.8712 | 0.0176 | 11 | 668 | 683 | AYLAGAAADVIEFGVR | | | | | Mascot |
| 1644.717 | 1644.8524 | 0.1354 | 82 | 1142 | 1157 | DTSSGSAAASFEQMQK | | | | | Mascot |
| 1648.821 | 1648.8413 | 0.0203 | 12 | 1199 | 1213 | VQLIASSIAENDNMK | | | Oxidation (M)[14] | | Mascot |
| 1666.8767 | 1666.894 | 0.0173 | 10 | 483 | 498 | VMGNAGHPSSLKPIMK | | | | | Mascot |
| 1682.8717 | 1682.8958 | 0.0241 | 14 | 483 | 498 | VMGNAGHPSSLKPIMK | | | Oxidation (M)[2] | | Mascot |
| 1699.9377 | 1699.9528 | 0.0151 | 9 | 34 | 49 | YEALLLGGLPQEGLAR | | | | | Mascot |
| 1699.9377 | 1699.9528 | 0.0151 | 9 | 34 | 49 | YEALLLGGLPQEGLAR | 89 | 100 | | | Mascot |
| 1706.025 | 1706.0416 | 0.0166 | 10 | 364 | 379 | WLLDALPAVGTPVIIK | | | | | Mascot |
| 1737.9568 | 1737.9172 | -0.0396 | -23 | 746 | 761 | SIIEEAIPMVTGPKPR | | | | | Mascot |
| 1824.8989 | 1824.9142 | 0.0153 | 8 | 72 | 86 | LMDPLLYEYAGTWPR | | | | | Mascot |
| 1840.8938 | 1840.9137 | 0.0199 | 11 | 72 | 86 | LMDPLLYEYAGTWPR | | | Oxidation (M)[2] | | Mascot |
| 1954.8745 | 1954.8923 | 0.0178 | 9 | 147 | 163 | TQNIYEMQEAGAQGVCR | | | Carbamidomethyl (C)[16] | | Mascot |
| 1954.8745 | 1954.8923 | 0.0178 | 9 | 147 | 163 | TQNIYEMQEAGAQGVCR | 146 | 100 | Carbamidomethyl (C)[16] | | Mascot |
| 1970.8695 | 1970.8909 | 0.0214 | 11 | 147 | 163 | TQNIYEMQEAGAQGVCR | | | Carbamidomethyl (C)[16], Oxidation (M)[7] | | Mascot |
| 2082.9695 | 2082.9944 | 0.0249 | 12 | 146 | 163 | KTQNIYEMQEAGAQGVC R | | | Carbamidomethyl (C)[17] | | Mascot |
| 2914.4912 | 2914.5107 | 0.0195 | 7 | 446 | 471 | HCTAVPTCPAELLRPIHEI ATEAISK | | | Carbamidomethyl (C)[2,8] | | Mascot |

| | | | | | | | | | | | |
|---|---|---|---|---|---|---|---|---|---|---|---|
| 4 | vitellogenin 1 [Danio rerio] | gi\|166795887 | 149452.5 | 8.68 | 30 | 747 | 100 | 590 | 100 | | |

**Peptide Information**

| Calc. Mass | Obsrv. Mass | ± da | ± ppm | Start Seq. | End Seq. | Sequence | Ion Score | C. I. % | Modification | Rank | Result Type |
|---|---|---|---|---|---|---|---|---|---|---|---|
| 821.4628 | 821.4716 | 0.0088 | 11 | 545 | 551 | ALHPEVR | | | | | Mascot |
| 821.4628 | 821.4716 | 0.0088 | 11 | 545 | 551 | ALHPEVR | 58 | 99.958 | | | Mascot |
| 874.4669 | 874.5159 | 0.049 | 56 | 87 | 94 | DPFVPATK | | | | | Mascot |
| 888.5301 | 888.538 | 0.0079 | 9 | 425 | 432 | FATIPALR | | | | | Mascot |
| 888.5301 | 888.538 | 0.0079 | 9 | 425 | 432 | FATIPALR | 44 | 98.855 | | | Mascot |
| 918.4965 | 918.5496 | 0.0531 | 58 | 1319 | 1326 | IPEITMSK | | | | | Mascot |
| 926.5417 | 926.5511 | 0.0094 | 10 | 505 | 513 | TAANALPIR | | | | | Mascot |
| 1097.6677 | 1097.6796 | 0.0119 | 11 | 514 | 523 | VQVDAILALR | | | | | Mascot |
| 1097.6677 | 1097.6796 | 0.0119 | 11 | 514 | 523 | VQVDAILALR | 98 | 100 | | | Mascot |
| 1115.6935 | 1115.7063 | 0.0128 | 11 | 333 | 341 | FVQLVQLLR | 71 | 99.998 | | | Mascot |
| 1115.6935 | 1115.7063 | 0.0128 | 11 | 333 | 341 | FVQLVQLLR | | | | | Mascot |
| 1121.599 | 1121.6111 | 0.0121 | 11 | 348 | 356 | IEAIWSQFK | | | | | Mascot |
| 1177.6462 | 1177.6561 | 0.0099 | 8 | 260 | 269 | QTLAFVEIEK | | | | | Mascot |

| | | | | | | | | | | | |
|---|---|---|---|---|---|---|---|---|---|---|---|
| 1215.6005 | 1215.616 | 0.0155 | 13 | 164 | 173 | THYVINEDPK | | | | | Mascot |
| 1226.6991 | 1226.71 | 0.0109 | 9 | 472 | 482 | NDIPEITLALK | | | | | Mascot |
| 1238.7831 | 1238.7933 | 0.0102 | 8 | 135 | 145 | GILNILQLNLK | | | | | Mascot |
| 1259.5797 | 1259.5946 | 0.0149 | 12 | 182 | 191 | SKDLSHCQER | | | Carbamidomethyl (C)[7] | | Mascot |
| 1379.7603 | 1379.7687 | 0.0084 | 6 | 433 | 445 | EVVMLGYGSLIAK | | | | | Mascot |
| 1382.7889 | 1382.7999 | 0.011 | 8 | 303 | 315 | ISDAPAQIVEVLK | | | | | Mascot |
| 1395.7551 | 1395.7643 | 0.0092 | 7 | 433 | 445 | EVVMLGYGSLIAK | | | Oxidation (M)[4] | | Mascot |
| 1463.8944 | 1463.91 | 0.0156 | 11 | 532 | 544 | LVQPVALQLVLDR | 84 | 100 | | | Mascot |
| 1463.8944 | 1463.91 | 0.0156 | 11 | 532 | 544 | LVQPVALQLVLDR | | | | | Mascot |
| 1638.8485 | 1638.8628 | 0.0143 | 9 | 668 | 683 | TYLAGAAADVVEFGVR | | | | | Mascot |
| 1644.717 | 1644.8524 | 0.1354 | 82 | 1142 | 1157 | DTSSGSAAASFEQMQK | | | | | Mascot |
| 1652.6665 | 1652.8809 | 0.2144 | 130 | 1098 | 1114 | SSSSSSQSSSSSMSSSR | | | | | Mascot |
| 1666.8767 | 1666.894 | 0.0173 | 10 | 483 | 498 | VMGNAGHPSSLKPIMK | | | | | Mascot |
| 1682.8717 | 1682.8958 | 0.0241 | 14 | 483 | 498 | VMGNAGHPSSLKPIMK | | | Oxidation (M)[2] | | Mascot |
| 1695.8999 | 1695.9147 | 0.0148 | 9 | 119 | 134 | VFAPAGVSPTVMNLHR | | | | | Mascot |
| 1699.9377 | 1699.9528 | 0.0151 | 9 | 34 | 49 | YEALLLGGLPQEGLAR | | | | | Mascot |
| 1699.9377 | 1699.9528 | 0.0151 | 9 | 34 | 49 | YEALLLGGLPQEGLAR | 89 | 100 | | | Mascot |
| 1706.025 | 1706.0416 | 0.0166 | 10 | 364 | 379 | WLLDALPAVGTPVIIK | | | | | Mascot |
| 1711.8948 | 1711.912 | 0.0172 | 10 | 119 | 134 | VFAPAGVSPTVMNLHR | 4 | 0 | Oxidation (M)[12] | | Mascot |
| 1711.8948 | 1711.912 | 0.0172 | 10 | 119 | 134 | VFAPAGVSPTVMNLHR | | | Oxidation (M)[12] | | Mascot |
| 1796.9078 | 1796.9176 | 0.0098 | 5 | 1279 | 1293 | HILTAAYDTGFRFER | | | | | Mascot |
| 1812.8877 | 1812.9053 | 0.0176 | 10 | 72 | 86 | LMDPLLYEYAGTWPK | | | Oxidation (M)[2] | | Mascot |
| 1954.8745 | 1954.8923 | 0.0178 | 9 | 147 | 163 | TQNIYEMQEAGAQGVCR | | | Carbamidomethyl (C)[16] | | Mascot |
| 1954.8745 | 1954.8923 | 0.0178 | 9 | 147 | 163 | TQNIYEMQEAGAQGVCR | 146 | 100 | Carbamidomethyl (C)[16] | | Mascot |
| 1970.8695 | 1970.8909 | 0.0214 | 11 | 147 | 163 | TQNIYEMQEAGAQGVCR | | | Carbamidomethyl (C)[16], Oxidation (M)[7] | | Mascot |
| 2082.9695 | 2082.9944 | 0.0249 | 12 | 146 | 163 | KTQNIYEMQEAGAQGVC R | | | Carbamidomethyl (C)[17] | | Mascot |
| 2233.2009 | 2233.1025 | -0.0984 | -44 | 593 | 614 | SLTRITAPDMAYVAGAAN VAIK | | | | | Mascot |
| 2326.2 | 2326.2205 | 0.0205 | 9 | 283 | 302 | GSLQYEFATEILQTPIQLM K | | | Oxidation (M)[19] | | Mascot |
| 2938.5164 | 2938.5325 | 0.0161 | 5 | 446 | 471 | YCVAVPTCPAELLRPIHEI ATEAISK | | | Carbamidomethyl (C)[2,8] | | Mascot |

5 vitellogenin 1 [Danio rerio] gi|21391472 127954.2 8.69 22 678 100 585 100

**Peptide Information**

| Calc. Mass | Obsrv. Mass | ± da | ± ppm | Start Seq. | End Seq. | Sequence | Ion Score | C. I. % | Modification | Rank | Result Type |
|---|---|---|---|---|---|---|---|---|---|---|---|
| 821.4628 | 821.4716 | 0.0088 | 11 | 354 | 360 | ALHPEVR | | | | | Mascot |
| 821.4628 | 821.4716 | 0.0088 | 11 | 354 | 360 | ALHPEVR | 58 | 99.958 | | | Mascot |

| | | | | | | | | | | | |
|---|---|---|---|---|---|---|---|---|---|---|---|
| 888.5301 | 888.538 | 0.0079 | 9 | 234 | 241 | FATIPALR | | | | | Mascot |
| 888.5301 | 888.538 | 0.0079 | 9 | 234 | 241 | FATIPALR | 44 | 98.855 | | | Mascot |
| 918.4965 | 918.5496 | 0.0531 | 58 | 1126 | 1133 | IPEITMSK | | | | | Mascot |
| 926.5417 | 926.5511 | 0.0094 | 10 | 314 | 322 | TAANALPIR | | | | | Mascot |
| 1023.5105 | 1023.5204 | 0.0099 | 10 | 4 | 12 | DVGLAYTER | 79 | 100 | | | Mascot |
| 1023.5105 | 1023.5204 | 0.0099 | 10 | 4 | 12 | DVGLAYTER | | | | | Mascot |
| 1097.6677 | 1097.6796 | 0.0119 | 11 | 323 | 332 | VQVDAILALR | | | | | Mascot |
| 1097.6677 | 1097.6796 | 0.0119 | 11 | 323 | 332 | VQVDAILALR | 98 | 100 | | | Mascot |
| 1115.6935 | 1115.7063 | 0.0128 | 11 | 142 | 150 | FVQLVQLLR | 71 | 99.998 | | | Mascot |
| 1115.6935 | 1115.7063 | 0.0128 | 11 | 142 | 150 | FVQLVQLLR | | | | | Mascot |
| 1121.599 | 1121.6111 | 0.0121 | 11 | 157 | 165 | IEAIWSQFK | | | | | Mascot |
| 1177.6462 | 1177.6561 | 0.0099 | 8 | 69 | 78 | QTLAFVEIEK | | | | | Mascot |
| 1226.6991 | 1226.71 | 0.0109 | 9 | 281 | 291 | NDIPEITLALK | | | | | Mascot |
| 1379.7603 | 1379.7687 | 0.0084 | 6 | 242 | 254 | EVVMLGYGSLIAK | | | | | Mascot |
| 1382.7889 | 1382.7999 | 0.011 | 8 | 112 | 124 | ISDAPAQIVEVLK | | | | | Mascot |
| 1395.7551 | 1395.7643 | 0.0092 | 7 | 242 | 254 | EVVMLGYGSLIAK | | | Oxidation (M)[4] | | Mascot |
| 1463.8944 | 1463.91 | 0.0156 | 11 | 341 | 353 | LVQPVALQLVLDR | 84 | 100 | | | Mascot |
| 1463.8944 | 1463.91 | 0.0156 | 11 | 341 | 353 | LVQPVALQLVLDR | | | | | Mascot |
| 1622.8536 | 1622.8712 | 0.0176 | 11 | 477 | 492 | AYLAGAAADVIEFGVR | 151 | 100 | | | Mascot |
| 1622.8536 | 1622.8712 | 0.0176 | 11 | 477 | 492 | AYLAGAAADVIEFGVR | | | | | Mascot |
| 1644.717 | 1644.8524 | 0.1354 | 82 | 949 | 964 | DTSSGSAAASFEQMQK | | | | | Mascot |
| 1666.8767 | 1666.894 | 0.0173 | 10 | 292 | 307 | VMGNAGHPSSLKPIMK | | | | | Mascot |
| 1682.8717 | 1682.8958 | 0.0241 | 14 | 292 | 307 | VMGNAGHPSSLKPIMK | | | Oxidation (M)[2] | | Mascot |
| 1699.9047 | 1699.9528 | 0.0481 | 28 | 406 | 423 | ITAPDMASVAGAANVAIK | | | | | Mascot |
| 1699.9047 | 1699.9528 | 0.0481 | 28 | 406 | 423 | ITAPDMASVAGAANVAIK | | | | | Mascot |
| 1706.025 | 1706.0416 | 0.0166 | 10 | 173 | 188 | WLLDALPAVGTPVIIK | | | | | Mascot |
| 1796.9078 | 1796.9176 | 0.0098 | 5 | 1086 | 1100 | HILTAAYDTGFRFER | | | | | Mascot |
| 1843.9159 | 1843.9298 | 0.0139 | 8 | 386 | 401 | IEPNMHVASFAYSHIK | | | | | Mascot |
| 1859.9109 | 1859.9304 | 0.0195 | 10 | 386 | 401 | IEPNMHVASFAYSHIK | | | Oxidation (M)[5] | | Mascot |
| 2326.2 | 2326.2205 | 0.0205 | 9 | 92 | 111 | GSLQYEFATEILQTPIQLM K | | | Oxidation (M)[19] | | Mascot |
| 2938.5164 | 2938.5325 | 0.0161 | 5 | 255 | 280 | YCVAVPTCPAELLRPIHEI ATEAISK | | | Carbamidomethyl (C)[2,8] | | Mascot |

| | | | | | | | | | | |
|---|---|---|---|---|---|---|---|---|---|---|
| 6 | Vtg1 protein [Danio rerio] | gi\|145337918 | 116565.5 | 9.07 | 19 | 581 | 100 | 506 | 100 | |

**Peptide Information**

| Calc. Mass | Obsrv. Mass | ± da | ± ppm | Start Seq. | End Seq. | Sequence | Ion Score | C. I. % | Modification | Rank | Result Type |
|---|---|---|---|---|---|---|---|---|---|---|---|
| 821.4628 | 821.4716 | 0.0088 | 11 | 251 | 257 | ALHPEVR | | | | | Mascot |

| Calc. Mass | Obsrv. Mass | ± da | ± ppm | Start Seq. | End Seq. | Sequence | Ion Score | C. I. % | Modification | Rank | Result Type |
|---|---|---|---|---|---|---|---|---|---|---|---|
| 821.4628 | 821.4716 | 0.0088 | 11 | 251 | 257 | ALHPEVR | 58 | 99.958 | | | Mascot |
| 888.5301 | 888.538 | 0.0079 | 9 | 131 | 138 | FATIPALR | | | | | Mascot |
| 888.5301 | 888.538 | 0.0079 | 9 | 131 | 138 | FATIPALR | 44 | 98.855 | | | Mascot |
| 918.4965 | 918.5496 | 0.0531 | 58 | 1025 | 1032 | IPEITMSK | | | | | Mascot |
| 926.5417 | 926.5511 | 0.0094 | 10 | 211 | 219 | TAANALPIR | | | | | Mascot |
| 1097.6677 | 1097.6796 | 0.0119 | 11 | 220 | 229 | VQVDAILALR | | | | | Mascot |
| 1097.6677 | 1097.6796 | 0.0119 | 11 | 220 | 229 | VQVDAILALR | 98 | 100 | | | Mascot |
| 1115.6935 | 1115.7063 | 0.0128 | 11 | 39 | 47 | FVQLVQLLR | 71 | 99.998 | | | Mascot |
| 1115.6935 | 1115.7063 | 0.0128 | 11 | 39 | 47 | FVQLVQLLR | | | | | Mascot |
| 1121.599 | 1121.6111 | 0.0121 | 11 | 54 | 62 | IEAIWSQFK | | | | | Mascot |
| 1226.6991 | 1226.71 | 0.0109 | 9 | 178 | 188 | NDIPEITLALK | | | | | Mascot |
| 1379.7603 | 1379.7687 | 0.0084 | 6 | 139 | 151 | EVVMLGYGSLIAK | | | | | Mascot |
| 1382.7889 | 1382.7999 | 0.011 | 8 | 9 | 21 | ISDAPAQIVEVLK | | | | | Mascot |
| 1395.7551 | 1395.7643 | 0.0092 | 7 | 139 | 151 | EVVMLGYGSLIAK | | | Oxidation (M)[4] | | Mascot |
| 1463.8944 | 1463.91 | 0.0156 | 11 | 238 | 250 | LVQPVALQLVLDR | 84 | 100 | | | Mascot |
| 1463.8944 | 1463.91 | 0.0156 | 11 | 238 | 250 | LVQPVALQLVLDR | | | | | Mascot |
| 1622.8536 | 1622.8712 | 0.0176 | 11 | 374 | 389 | AYLAGAAADVIEFGVR | 151 | 100 | | | Mascot |
| 1622.8536 | 1622.8712 | 0.0176 | 11 | 374 | 389 | AYLAGAAADVIEFGVR | | | | | Mascot |
| 1644.717 | 1644.8524 | 0.1354 | 82 | 848 | 863 | DTSSGSAAASFEQMQK | | | | | Mascot |
| 1666.8767 | 1666.894 | 0.0173 | 10 | 189 | 204 | VMGNAGHPSSLKPIMK | | | | | Mascot |
| 1682.8717 | 1682.8958 | 0.0241 | 14 | 189 | 204 | VMGNAGHPSSLKPIMK | | | Oxidation (M)[2] | | Mascot |
| 1699.9047 | 1699.9528 | 0.0481 | 28 | 303 | 320 | ITAPDMASVAGAANVAIK | | | | | Mascot |
| 1699.9047 | 1699.9528 | 0.0481 | 28 | 303 | 320 | ITAPDMASVAGAANVAIK | | | | | Mascot |
| 1706.025 | 1706.0416 | 0.0166 | 10 | 70 | 85 | WLLDALPAVGTPVIIK | | | | | Mascot |
| 1796.9078 | 1796.9176 | 0.0098 | 5 | 985 | 999 | HILTAAYDTGFRFER | | | | | Mascot |
| 1843.9159 | 1843.9298 | 0.0139 | 8 | 283 | 298 | IEPNMHVASFAYSHIK | | | | | Mascot |
| 1859.9109 | 1859.9304 | 0.0195 | 10 | 283 | 298 | IEPNMHVASFAYSHIK | | | Oxidation (M)[5] | | Mascot |
| 2938.5164 | 2938.5325 | 0.0161 | 5 | 152 | 177 | YCVAVPTCPAELLRPIHEI ATEAISK | | | Carbamidomethyl (C)[2,8] | | Mascot |

7 novel protein similar to vitellogenin 1 (vg1) [Danio rerio] gi|94733734 149808.5 8.83 23 571 100 478 100

**Peptide Information**

| Calc. Mass | Obsrv. Mass | ± da | ± ppm | Start Seq. | End Seq. | Sequence | Ion Score | C. I. % | Modification | Rank | Result Type |
|---|---|---|---|---|---|---|---|---|---|---|---|
| 821.4628 | 821.4716 | 0.0088 | 11 | 549 | 555 | ALHPEVR | | | | | Mascot |
| 821.4628 | 821.4716 | 0.0088 | 11 | 549 | 555 | ALHPEVR | 58 | 99.958 | | | Mascot |
| 918.4965 | 918.5496 | 0.0531 | 58 | 1326 | 1333 | IPEITMSK | | | | | Mascot |

| | | | | | | | | | | | |
|---|---|---|---|---|---|---|---|---|---|---|---|
| 1023.5105 | 1023.5204 | 0.0099 | 10 | 199 | 207 | DVGLAYTER | 79 | 100 | | | Mascot |
| 1023.5105 | 1023.5204 | 0.0099 | 10 | 199 | 207 | DVGLAYTER | | | | | Mascot |
| 1097.6677 | 1097.6796 | 0.0119 | 11 | 518 | 527 | VQVDAILALR | | | | | Mascot |
| 1097.6677 | 1097.6796 | 0.0119 | 11 | 518 | 527 | VQVDAILALR | 98 | 100 | | | Mascot |
| 1115.6935 | 1115.7063 | 0.0128 | 11 | 337 | 345 | FVQLVQLLR | 71 | 99.998 | | | Mascot |
| 1115.6935 | 1115.7063 | 0.0128 | 11 | 337 | 345 | FVQLVQLLR | | | | | Mascot |
| 1121.599 | 1121.6111 | 0.0121 | 11 | 352 | 360 | IEAIWSQFK | | | | | Mascot |
| 1177.6462 | 1177.6561 | 0.0099 | 8 | 264 | 273 | QTLAFVEIEK | | | | | Mascot |
| 1215.6005 | 1215.616 | 0.0155 | 13 | 168 | 177 | THYVINEDPK | | | | | Mascot |
| 1226.6991 | 1226.71 | 0.0109 | 9 | 476 | 486 | NDIPEITLALK | | | | | Mascot |
| 1238.7831 | 1238.7933 | 0.0102 | 8 | 139 | 149 | GILNILQLNLK | | | | | Mascot |
| 1259.5797 | 1259.5946 | 0.0149 | 12 | 186 | 195 | SKDLSHCQER | | | Carbamidomethyl (C)[7] | | Mascot |
| 1379.7603 | 1379.7687 | 0.0084 | 6 | 437 | 449 | EVVMLGYGSLIAK | | | | | Mascot |
| 1395.7551 | 1395.7643 | 0.0092 | 7 | 437 | 449 | EVVMLGYGSLIAK | | | Oxidation (M)[4] | | Mascot |
| 1463.8944 | 1463.91 | 0.0156 | 11 | 536 | 548 | LVQPVALQLVLDR | 84 | 100 | | | Mascot |
| 1463.8944 | 1463.91 | 0.0156 | 11 | 536 | 548 | LVQPVALQLVLDR | | | | | Mascot |
| 1638.8485 | 1638.8628 | 0.0143 | 9 | 672 | 687 | TYLAGAAADVVEFGVR | | | | | Mascot |
| 1644.717 | 1644.8524 | 0.1354 | 82 | 1149 | 1164 | DTSSGSAAASFEQMQK | | | | | Mascot |
| 1648.821 | 1648.8413 | 0.0203 | 12 | 1206 | 1220 | VQLIASSIAENDNMK | | | Oxidation (M)[14] | | Mascot |
| 1666.8767 | 1666.894 | 0.0173 | 10 | 487 | 502 | VMGNAGHPSSLKPIMK | | | | | Mascot |
| 1682.8717 | 1682.8958 | 0.0241 | 14 | 487 | 502 | VMGNAGHPSSLKPIMK | | | Oxidation (M)[2] | | Mascot |
| 1695.8999 | 1695.9147 | 0.0148 | 9 | 123 | 138 | VFAPAGVSPTVMNLHR | | | | | Mascot |
| 1699.9377 | 1699.9528 | 0.0151 | 9 | 38 | 53 | YEALLLGGLPQEGLAR | | | | | Mascot |
| 1699.9377 | 1699.9528 | 0.0151 | 9 | 38 | 53 | YEALLLGGLPQEGLAR | 89 | 100 | | | Mascot |
| 1706.025 | 1706.0416 | 0.0166 | 10 | 368 | 383 | WLLDALPAVGTPVIIK | | | | | Mascot |
| 1711.8948 | 1711.912 | 0.0172 | 10 | 123 | 138 | VFAPAGVSPTVMNLHR | 4 | 0 | Oxidation (M)[12] | | Mascot |
| 1711.8948 | 1711.912 | 0.0172 | 10 | 123 | 138 | VFAPAGVSPTVMNLHR | | | Oxidation (M)[12] | | Mascot |
| 1796.8928 | 1796.9176 | 0.0248 | 14 | 76 | 90 | LMDPLLYEYAGTWPK | | | | | Mascot |
| 1812.8877 | 1812.9053 | 0.0176 | 10 | 76 | 90 | LMDPLLYEYAGTWPK | | | Oxidation (M)[2] | | Mascot |
| 1936.9181 | 1936.937 | 0.0189 | 10 | 151 | 167 | TQNIYELQEAGAQGVCR | | | Carbamidomethyl (C)[16] | | Mascot |
| 2233.2009 | 2233.1025 | -0.0984 | -44 | 597 | 618 | SLTRITAPDMAYVAGAAN VAIK | | | | | Mascot |

8 vitellogenin 6 [Danio rerio] gi|303227889 150936.1 8.84 23 570 100 478 100

**Peptide Information**

| Calc. Mass | Obsrv. Mass | ± da | ± ppm | Start Seq. | End Seq. | Sequence | Ion Score | C. I. % | Modification | Rank | Result Type |
|---|---|---|---|---|---|---|---|---|---|---|---|
| 821.4628 | 821.4716 | 0.0088 | 11 | 556 | 562 | ALHPEVR | | | | | Mascot |

| Calc. Mass | Obsrv. Mass | ± da | ± ppm | Start | End | Sequence | Ion | C. I. % | Modification | Rank | Result Type |
|---|---|---|---|---|---|---|---|---|---|---|---|
| 821.4628 | 821.4716 | 0.0088 | 11 | 556 | 562 | ALHPEVR | 58 | 99.958 | | | Mascot |
| 918.4965 | 918.5496 | 0.0531 | 58 | 1337 | 1344 | IPEITMSK | | | | | Mascot |
| 1023.5105 | 1023.5204 | 0.0099 | 10 | 206 | 214 | DVGLAYTER | 79 | 100 | | | Mascot |
| 1023.5105 | 1023.5204 | 0.0099 | 10 | 206 | 214 | DVGLAYTER | | | | | Mascot |
| 1097.6677 | 1097.6796 | 0.0119 | 11 | 525 | 534 | VQVDAILALR | | | | | Mascot |
| 1097.6677 | 1097.6796 | 0.0119 | 11 | 525 | 534 | VQVDAILALR | 98 | 100 | | | Mascot |
| 1115.6935 | 1115.7063 | 0.0128 | 11 | 344 | 352 | FVQLVQLLR | 71 | 99.998 | | | Mascot |
| 1115.6935 | 1115.7063 | 0.0128 | 11 | 344 | 352 | FVQLVQLLR | | | | | Mascot |
| 1121.599 | 1121.6111 | 0.0121 | 11 | 359 | 367 | IEAIWSQFK | | | | | Mascot |
| 1177.6462 | 1177.6561 | 0.0099 | 8 | 271 | 280 | QTLAFVEIEK | | | | | Mascot |
| 1215.6005 | 1215.616 | 0.0155 | 13 | 175 | 184 | THYVINEDPK | | | | | Mascot |
| 1226.6991 | 1226.71 | 0.0109 | 9 | 483 | 493 | NDIPEITLALK | | | | | Mascot |
| 1238.7831 | 1238.7933 | 0.0102 | 8 | 146 | 156 | GILNILQLNLK | | | | | Mascot |
| 1259.5797 | 1259.5946 | 0.0149 | 12 | 193 | 202 | SKDLSHCQER | | | Carbamidomethyl (C)[7] | | Mascot |
| 1379.7603 | 1379.7687 | 0.0084 | 6 | 444 | 456 | EVVMLGYGSLIAK | | | | | Mascot |
| 1395.7551 | 1395.7643 | 0.0092 | 7 | 444 | 456 | EVVMLGYGSLIAK | | | Oxidation (M)[4] | | Mascot |
| 1463.8944 | 1463.91 | 0.0156 | 11 | 543 | 555 | LVQPVALQLVLDR | 84 | 100 | | | Mascot |
| 1463.8944 | 1463.91 | 0.0156 | 11 | 543 | 555 | LVQPVALQLVLDR | | | | | Mascot |
| 1638.8485 | 1638.8628 | 0.0143 | 9 | 679 | 694 | TYLAGAAADVVEFGVR | | | | | Mascot |
| 1644.717 | 1644.8524 | 0.1354 | 82 | 1160 | 1175 | DTSSGSAAASFEQMQK | | | | | Mascot |
| 1648.821 | 1648.8413 | 0.0203 | 12 | 1217 | 1231 | VQLIASSIAENDNMK | | | Oxidation (M)[14] | | Mascot |
| 1666.8767 | 1666.894 | 0.0173 | 10 | 494 | 509 | VMGNAGHPSSLKPIMK | | | | | Mascot |
| 1682.8717 | 1682.8958 | 0.0241 | 14 | 494 | 509 | VMGNAGHPSSLKPIMK | | | Oxidation (M)[2] | | Mascot |
| 1695.8999 | 1695.9147 | 0.0148 | 9 | 130 | 145 | VFAPAGVSPTVMNLHR | | | | | Mascot |
| 1699.9377 | 1699.9528 | 0.0151 | 9 | 45 | 60 | YEALLLGGLPQEGLAR | | | | | Mascot |
| 1699.9377 | 1699.9528 | 0.0151 | 9 | 45 | 60 | YEALLLGGLPQEGLAR | 89 | 100 | | | Mascot |
| 1706.025 | 1706.0416 | 0.0166 | 10 | 375 | 390 | WLLDALPAVGTPVIIK | | | | | Mascot |
| 1711.8948 | 1711.912 | 0.0172 | 10 | 130 | 145 | VFAPAGVSPTVMNLHR | 4 | 0 | Oxidation (M)[12] | | Mascot |
| 1711.8948 | 1711.912 | 0.0172 | 10 | 130 | 145 | VFAPAGVSPTVMNLHR | | | Oxidation (M)[12] | | Mascot |
| 1796.8928 | 1796.9176 | 0.0248 | 14 | 83 | 97 | LMDPLLYEYAGTWPK | | | | | Mascot |
| 1812.8877 | 1812.9053 | 0.0176 | 10 | 83 | 97 | LMDPLLYEYAGTWPK | | | Oxidation (M)[2] | | Mascot |
| 1936.9181 | 1936.937 | 0.0189 | 10 | 158 | 174 | TQNIYELQEAGAQGVCR | | | Carbamidomethyl (C)[16] | | Mascot |
| 2233.2009 | 2233.1025 | -0.0984 | -44 | 604 | 625 | SLTRITAPDMAYVAGAAN VAIK | | | | | Mascot |

| | | | | | | | | | | |
|---|---|---|---|---|---|---|---|---|---|---|
| 9 | hypothetical protein LOC678536 [Danio rerio] | gi\|160420306 | 148750.2 | 8.89 | 27 | 513 | 100 | 393 | 100 | |

**Peptide Information**

| Calc. Mass | Obsrv. Mass | ± da | ± ppm | Start | End | Sequence | Ion | C. I. % | Modification | Rank | Result Type |
|---|---|---|---|---|---|---|---|---|---|---|---|

| | | | | Seq. | Seq. | | Score | | | |
|---|---|---|---|---|---|---|---|---|---|---|
| 821.4628 | 821.4716 | 0.0088 | 11 | 545 | 551 | ALHPEVR | | | | Mascot |
| 821.4628 | 821.4716 | 0.0088 | 11 | 545 | 551 | ALHPEVR | 58 | 99.958 | | Mascot |
| 918.4778 | 918.5496 | 0.0718 | 78 | 1059 | 1066 | EILDTEAK | | | | Mascot |
| 1023.5105 | 1023.5204 | 0.0099 | 10 | 195 | 203 | DVGLAYTER | 79 | 100 | | Mascot |
| 1023.5105 | 1023.5204 | 0.0099 | 10 | 195 | 203 | DVGLAYTER | | | | Mascot |
| 1045.5465 | 1045.5737 | 0.0272 | 26 | 1346 | 1353 | DFLSWIHK | | | | Mascot |
| 1097.6677 | 1097.6796 | 0.0119 | 11 | 514 | 523 | VQVDAILALR | | | | Mascot |
| 1097.6677 | 1097.6796 | 0.0119 | 11 | 514 | 523 | VQVDAILALR | 98 | 100 | | Mascot |
| 1115.6935 | 1115.7063 | 0.0128 | 11 | 333 | 341 | FVQLVQLLR | 71 | 99.998 | | Mascot |
| 1115.6935 | 1115.7063 | 0.0128 | 11 | 333 | 341 | FVQLVQLLR | | | | Mascot |
| 1121.599 | 1121.6111 | 0.0121 | 11 | 348 | 356 | IEAIWSQFK | | | | Mascot |
| 1177.6462 | 1177.6561 | 0.0099 | 8 | 260 | 269 | QTLAFVEIEK | | | | Mascot |
| 1215.6005 | 1215.616 | 0.0155 | 13 | 164 | 173 | THYVINEDPK | | | | Mascot |
| 1226.6991 | 1226.71 | 0.0109 | 9 | 472 | 482 | NDIPEITLALK | | | | Mascot |
| 1238.7831 | 1238.7933 | 0.0102 | 8 | 135 | 145 | GILNILQLNLK | | | | Mascot |
| 1259.5797 | 1259.5946 | 0.0149 | 12 | 182 | 191 | SKDLSHCQER | | | Carbamidomethyl (C)[7] | Mascot |
| 1379.7351 | 1379.7687 | 0.0336 | 24 | 433 | 445 | EVAMLGYGSLIAR | | | | Mascot |
| 1382.7889 | 1382.7999 | 0.011 | 8 | 303 | 315 | ISDAPAQIVEVLK | | | | Mascot |
| 1395.7301 | 1395.7643 | 0.0342 | 25 | 192 | 203 | IMKDVGLAYTER | | | | Mascot |
| 1477.91 | 1477.9259 | 0.0159 | 11 | 532 | 544 | LVQPIALQLVLDR | | | | Mascot |
| 1644.717 | 1644.8524 | 0.1354 | 82 | 1140 | 1155 | DTSSGSAAASFEQMQK | | | | Mascot |
| 1648.821 | 1648.8413 | 0.0203 | 12 | 1197 | 1211 | VQLIASSIAENDNMK | | | Oxidation (M)[14] | Mascot |
| 1666.8767 | 1666.894 | 0.0173 | 10 | 483 | 498 | VMGNAGHPSSLKPIMK | | | | Mascot |
| 1682.8717 | 1682.8958 | 0.0241 | 14 | 483 | 498 | VMGNAGHPSSLKPIMK | | | Oxidation (M)[2] | Mascot |
| 1695.8999 | 1695.9147 | 0.0148 | 9 | 119 | 134 | VFAPAGVSPTVMNLHR | | | | Mascot |
| 1699.9377 | 1699.9528 | 0.0151 | 9 | 34 | 49 | YEALLLGGLPQEGLAR | 89 | 100 | | Mascot |
| 1699.9524 | 1699.9528 | 0.0004 | 0 | 1310 | 1324 | SLNVIARIPEITMSR | | | | Mascot |
| 1706.025 | 1706.0416 | 0.0166 | 10 | 364 | 379 | WLLDALPAVGTPVIIK | | | | Mascot |
| 1711.8948 | 1711.912 | 0.0172 | 10 | 119 | 134 | VFAPAGVSPTVMNLHR | 4 | 0 | Oxidation (M)[12] | Mascot |
| 1711.8948 | 1711.912 | 0.0172 | 10 | 119 | 134 | VFAPAGVSPTVMNLHR | | | Oxidation (M)[12] | Mascot |
| 1796.8928 | 1796.9176 | 0.0248 | 14 | 72 | 86 | LMDPLLYEYAGTWPK | | | | Mascot |
| 1812.8877 | 1812.9053 | 0.0176 | 10 | 72 | 86 | LMDPLLYEYAGTWPK | | | Oxidation (M)[2] | Mascot |
| 1936.9181 | 1936.937 | 0.0189 | 10 | 147 | 163 | TQNIYELQEAGAQGVCR | | | Carbamidomethyl (C)[16] | Mascot |
| 2233.2009 | 2233.1025 | -0.0984 | -44 | 593 | 614 | SLTRITAPDMAYVAGAANVAIK | | | | Mascot |
| 2326.2 | 2326.2205 | 0.0205 | 9 | 283 | 302 | GSLQYEFATEILQTPIQLM | | | Oxidation (M)[19] | Mascot |

K

| 10 | novel protein similar to vitellogenin 1 (vg1) [Danio rerio] | gi\|94732723 | 147084.2 | 8.73 | 24 | 410 | 100 | 314 | 100 |
|---|---|---|---|---|---|---|---|---|---|

Peptide Information

| Calc. Mass | Obsrv. Mass | ± da | ± ppm | Start Seq. | End Seq. | Sequence | Ion Score | C. I. % | Modification | Rank | Result Type |
|---|---|---|---|---|---|---|---|---|---|---|---|
| 821.4628 | 821.4716 | 0.0088 | 11 | 545 | 551 | ALHPEVR | | | | | Mascot |
| 821.4628 | 821.4716 | 0.0088 | 11 | 545 | 551 | ALHPEVR | 58 | 99.958 | | | Mascot |
| 874.4669 | 874.5159 | 0.049 | 56 | 87 | 94 | DPFVPATK | | | | | Mascot |
| 918.5407 | 918.5496 | 0.0089 | 10 | 425 | 432 | FTTIPALR | | | | | Mascot |
| 1097.6677 | 1097.6796 | 0.0119 | 11 | 514 | 523 | VQVDAILALR | | | | | Mascot |
| 1097.6677 | 1097.6796 | 0.0119 | 11 | 514 | 523 | VQVDAILALR | 98 | 100 | | | Mascot |
| 1115.6935 | 1115.7063 | 0.0128 | 11 | 333 | 341 | FVQLVQLLR | 71 | 99.998 | | | Mascot |
| 1115.6935 | 1115.7063 | 0.0128 | 11 | 333 | 341 | FVQLVQLLR | | | | | Mascot |
| 1177.6462 | 1177.6561 | 0.0099 | 8 | 260 | 269 | QTLAFVEIEK | | | | | Mascot |
| 1215.6005 | 1215.616 | 0.0155 | 13 | 164 | 173 | THYVINEDPK | | | | | Mascot |
| 1226.6991 | 1226.71 | 0.0109 | 9 | 472 | 482 | NDIPEITLALK | | | | | Mascot |
| 1238.7831 | 1238.7933 | 0.0102 | 8 | 135 | 145 | GILNILQLNLK | | | | | Mascot |
| 1259.5797 | 1259.5946 | 0.0149 | 12 | 182 | 191 | SKDLSHCQER | | | Carbamidomethyl (C)[7] | | Mascot |
| 1379.7603 | 1379.7687 | 0.0084 | 6 | 433 | 445 | EVVMLGYGSLIAK | | | | | Mascot |
| 1395.7551 | 1395.7643 | 0.0092 | 7 | 433 | 445 | EVVMLGYGSLIAK | | | Oxidation (M)[4] | | Mascot |
| 1477.91 | 1477.9259 | 0.0159 | 11 | 532 | 544 | LVQPIALQLVLDR | | | | | Mascot |
| 1622.7743 | 1622.8712 | 0.0969 | 60 | 981 | 994 | GCVEVHSYNAAFIR | | | Carbamidomethyl (C)[2] | | Mascot |
| 1622.7743 | 1622.8712 | 0.0969 | 60 | 981 | 994 | GCVEVHSYNAAFIR | | | Carbamidomethyl (C)[2] | | Mascot |
| 1644.717 | 1644.8524 | 0.1354 | 82 | 1123 | 1138 | DTSSGSAAASFEQMQK | | | | | Mascot |
| 1652.8643 | 1652.8809 | 0.0166 | 10 | 668 | 683 | TYLAGAAADVIEFGVR | | | | | Mascot |
| 1666.8767 | 1666.894 | 0.0173 | 10 | 483 | 498 | VMGNAGHPSSLKPIMK | | | | | Mascot |
| 1682.8717 | 1682.8958 | 0.0241 | 14 | 483 | 498 | VMGNAGHPSSLKPIMK | | | Oxidation (M)[2] | | Mascot |
| 1699.9377 | 1699.9528 | 0.0151 | 9 | 34 | 49 | YEALLLGGLPQEGLAR | | | | | Mascot |
| 1699.9377 | 1699.9528 | 0.0151 | 9 | 34 | 49 | YEALLLGGLPQEGLAR | 89 | 100 | | | Mascot |
| 1706.025 | 1706.0416 | 0.0166 | 10 | 364 | 379 | WLLDALPAVGTPVIIK | | | | | Mascot |
| 1721.9043 | 1721.9209 | 0.0166 | 10 | 119 | 134 | VFAPAGVSPTVMNLYR | | | | | Mascot |
| 1737.8992 | 1737.9172 | 0.018 | 10 | 119 | 134 | VFAPAGVSPTVMNLYR | | | Oxidation (M)[12] | | Mascot |
| 1796.8928 | 1796.9176 | 0.0248 | 14 | 72 | 86 | LMDPLLYEYAGTWPK | | | | | Mascot |
| 1812.8877 | 1812.9053 | 0.0176 | 10 | 72 | 86 | LMDPLLYEYAGTWPK | | | Oxidation (M)[2] | | Mascot |
| 1843.9159 | 1843.9298 | 0.0139 | 8 | 577 | 592 | IEPNMHVASFAYSHIK | | | | | Mascot |
| 1859.9109 | 1859.9304 | 0.0195 | 10 | 577 | 592 | IEPNMHVASFAYSHIK | | | Oxidation (M)[5] | | Mascot |

| | | | | | | | | | | | |
|---|---|---|---|---|---|---|---|---|---|---|---|
| 1886.7894 | 1886.811 | 0.0216 | 11 | 195 | 210 | DVGLAYTESCPECTER | | | Carbamidomethyl (C)[10,13] | | Mascot |
| 1936.9181 | 1936.937 | 0.0189 | 10 | 147 | 163 | TQNIYELQEAGAQGVCR | | | Carbamidomethyl (C)[16] | | Mascot |
| 2326.2 | 2326.2205 | 0.0205 | 9 | 283 | 302 | GSLQYEFATEILQTPIQLMK | | | Oxidation (M)[19] | | Mascot |

| | | | | | | | | | | | |
|---|---|---|---|---|---|---|---|---|---|---|---|
| 11 | vitellogenin 7 [Danio rerio] | gi\|156713467 | 148739.2 | 8.76 | 21 | 385 | 100 | 314 | 100 | | |

**Peptide Information**

| Calc. Mass | Obsrv. Mass | ± da | ± ppm | Start Seq. | End Seq. | Sequence | Ion Score | C. I. % | Modification | Rank | Result Type |
|---|---|---|---|---|---|---|---|---|---|---|---|
| 821.4628 | 821.4716 | 0.0088 | 11 | 545 | 551 | ALHPEVR | | | | | Mascot |
| 821.4628 | 821.4716 | 0.0088 | 11 | 545 | 551 | ALHPEVR | 58 | 99.958 | | | Mascot |
| 874.4669 | 874.5159 | 0.049 | 56 | 87 | 94 | DPFVPATK | | | | | Mascot |
| 918.5407 | 918.5496 | 0.0089 | 10 | 425 | 432 | FTTIPALR | | | | | Mascot |
| 1097.6677 | 1097.6796 | 0.0119 | 11 | 514 | 523 | VQVDAILALR | | | | | Mascot |
| 1097.6677 | 1097.6796 | 0.0119 | 11 | 514 | 523 | VQVDAILALR | 98 | 100 | | | Mascot |
| 1115.6935 | 1115.7063 | 0.0128 | 11 | 333 | 341 | FVQLVQLLR | 71 | 99.998 | | | Mascot |
| 1115.6935 | 1115.7063 | 0.0128 | 11 | 333 | 341 | FVQLVQLLR | | | | | Mascot |
| 1177.6462 | 1177.6561 | 0.0099 | 8 | 260 | 269 | QTLAFVEIEK | | | | | Mascot |
| 1215.6005 | 1215.616 | 0.0155 | 13 | 164 | 173 | THYVINEDPK | | | | | Mascot |
| 1226.6991 | 1226.71 | 0.0109 | 9 | 472 | 482 | NDIPEITLALK | | | | | Mascot |
| 1238.7831 | 1238.7933 | 0.0102 | 8 | 135 | 145 | GILNILQLNLK | | | | | Mascot |
| 1259.5797 | 1259.5946 | 0.0149 | 12 | 182 | 191 | SKDLSHCQER | | | Carbamidomethyl (C)[7] | | Mascot |
| 1379.7603 | 1379.7687 | 0.0084 | 6 | 433 | 445 | EVVMLGYGSLIAK | | | | | Mascot |
| 1395.7551 | 1395.7643 | 0.0092 | 7 | 433 | 445 | EVVMLGYGSLIAK | | | Oxidation (M)[4] | | Mascot |
| 1477.91 | 1477.9259 | 0.0159 | 11 | 532 | 544 | LVQPIALQLVLDR | | | | | Mascot |
| 1652.8643 | 1652.8809 | 0.0166 | 10 | 668 | 683 | TYLAGAAADVIEFGVR | | | | | Mascot |
| 1666.8767 | 1666.894 | 0.0173 | 10 | 483 | 498 | VMGNAGHPSSLKPIMK | | | | | Mascot |
| 1682.8717 | 1682.8958 | 0.0241 | 14 | 483 | 498 | VMGNAGHPSSLKPIMK | | | Oxidation (M)[2] | | Mascot |
| 1699.9377 | 1699.9528 | 0.0151 | 9 | 34 | 49 | YEALLLGGLPQEGLAR | | | | | Mascot |
| 1699.9377 | 1699.9528 | 0.0151 | 9 | 34 | 49 | YEALLLGGLPQEGLAR | 89 | 100 | | | Mascot |
| 1706.025 | 1706.0416 | 0.0166 | 10 | 364 | 379 | WLLDALPAVGTPVIIK | | | | | Mascot |
| 1721.9043 | 1721.9209 | 0.0166 | 10 | 119 | 134 | VFAPAGVSPTVMNLYR | | | | | Mascot |
| 1737.8992 | 1737.9172 | 0.018 | 10 | 119 | 134 | VFAPAGVSPTVMNLYR | | | Oxidation (M)[12] | | Mascot |
| 1796.8928 | 1796.9176 | 0.0248 | 14 | 72 | 86 | LMDPLLYEYAGTWPK | | | | | Mascot |
| 1812.8877 | 1812.9053 | 0.0176 | 10 | 72 | 86 | LMDPLLYEYAGTWPK | | | Oxidation (M)[2] | | Mascot |
| 1843.9159 | 1843.9298 | 0.0139 | 8 | 577 | 592 | IEPNMHVASFAYSHIK | | | | | Mascot |
| 1859.9109 | 1859.9304 | 0.0195 | 10 | 577 | 592 | IEPNMHVASFAYSHIK | | | Oxidation (M)[5] | | Mascot |
| 1886.7894 | 1886.811 | 0.0216 | 11 | 195 | 210 | DVGLAYTESCPECTER | | | Carbamidomethyl (C)[10,13] | | Mascot |

| Calc. Mass | Obsrv. Mass | ± da | ± ppm | Start Seq. | End Seq. | Sequence | Ion Score | C. I. % | Modification | Rank | Result Type |
|---|---|---|---|---|---|---|---|---|---|---|---|
| 2326.2 | 2326.2205 | 0.0205 | 9 | 283 | 302 | GSLQYEFATEILQTPIQLMK | | | Oxidation (M)[19] | | Mascot |

12 Zgc:136383 protein [Danio rerio] gi|159155252 123957.1 8.78 19 294 100 227 100

**Peptide Information**

| Calc. Mass | Obsrv. Mass | ± da | ± ppm | Start Seq. | End Seq. | Sequence | Ion Score | C. I. % | Modification | Rank | Result Type |
|---|---|---|---|---|---|---|---|---|---|---|---|
| 821.4628 | 821.4716 | 0.0088 | 11 | 322 | 328 | ALHPEVR | | | | | Mascot |
| 821.4628 | 821.4716 | 0.0088 | 11 | 322 | 328 | ALHPEVR | 58 | 99.958 | | | Mascot |
| 918.4778 | 918.5496 | 0.0718 | 78 | 836 | 843 | EILDTEAK | | | | | Mascot |
| 1045.5465 | 1045.5737 | 0.0272 | 26 | 1123 | 1130 | DFLSWIHK | | | | | Mascot |
| 1097.6677 | 1097.6796 | 0.0119 | 11 | 291 | 300 | VQVDAILALR | | | | | Mascot |
| 1097.6677 | 1097.6796 | 0.0119 | 11 | 291 | 300 | VQVDAILALR | 98 | 100 | | | Mascot |
| 1115.6935 | 1115.7063 | 0.0128 | 11 | 110 | 118 | FVQLVQLLR | 71 | 99.998 | | | Mascot |
| 1115.6935 | 1115.7063 | 0.0128 | 11 | 110 | 118 | FVQLVQLLR | | | | | Mascot |
| 1121.599 | 1121.6111 | 0.0121 | 11 | 125 | 133 | IEAIWSQFK | | | | | Mascot |
| 1177.6462 | 1177.6561 | 0.0099 | 8 | 37 | 46 | QTLAFVEIEK | | | | | Mascot |
| 1238.7355 | 1238.7933 | 0.0578 | 47 | 249 | 259 | NDIPEIILALK | | | | | Mascot |
| 1379.7351 | 1379.7687 | 0.0336 | 24 | 210 | 222 | EVAMLGYGSLIAR | | | | | Mascot |
| 1382.7889 | 1382.7999 | 0.011 | 8 | 80 | 92 | ISDAPAQIVEVLK | | | | | Mascot |
| 1395.73 | 1395.7643 | 0.0343 | 25 | 210 | 222 | EVAMLGYGSLIAR | | | Oxidation (M)[4] | | Mascot |
| 1477.91 | 1477.9259 | 0.0159 | 11 | 309 | 321 | LVQPIALQLVLDR | | | | | Mascot |
| 1644.717 | 1644.8524 | 0.1354 | 82 | 917 | 932 | DTSSGSAAASFEQMQK | | | | | Mascot |
| 1648.821 | 1648.8413 | 0.0203 | 12 | 974 | 988 | VQLIASSIAENDNMK | | | Oxidation (M)[14] | | Mascot |
| 1666.8767 | 1666.894 | 0.0173 | 10 | 260 | 275 | VMGNAGHPSSLKPIMK | | | | | Mascot |
| 1682.8717 | 1682.8958 | 0.0241 | 14 | 260 | 275 | VMGNAGHPSSLKPIMK | | | Oxidation (M)[2] | | Mascot |
| 1699.9524 | 1699.9528 | 0.0004 | 0 | 1087 | 1101 | SLNVIARIPEITMSR | | | | | Mascot |
| 1699.9524 | 1699.9528 | 0.0004 | 0 | 1087 | 1101 | SLNVIARIPEITMSR | | | | | Mascot |
| 1706.025 | 1706.0416 | 0.0166 | 10 | 141 | 156 | WLLDALPAVGTPVIIK | | | | | Mascot |
| 1721.9618 | 1721.9209 | -0.0409 | -24 | 523 | 538 | TIIEEAIPMVAGPKPR | | | | | Mascot |
| 1737.9568 | 1737.9172 | -0.0396 | -23 | 523 | 538 | TIIEEAIPMVAGPKPR | | | Oxidation (M)[9] | | Mascot |
| 2233.2009 | 2233.1025 | -0.0984 | -44 | 370 | 391 | SLTRITAPDMAYVAGAANVAIK | | | | | Mascot |
| 2326.2 | 2326.2205 | 0.0205 | 9 | 60 | 79 | GSLQYEFATEILQTPIQLMK | | | Oxidation (M)[19] | | Mascot |

13 Vtg1 protein [Danio rerio] gi|63100501 36409.1 9.23 5 162 100 146 100

**Peptide Information**

| Calc. Mass | Obsrv. Mass | ± da | ± ppm | Start Seq. | End Seq. | Sequence | Ion Score | C. I. % | Modification | Rank | Result Type |
|---|---|---|---|---|---|---|---|---|---|---|---|

| Calc. Mass | Obsrv. Mass | ± da | ± ppm | Start Seq. | End Seq. | Sequence | Ion Score | C. I. % | Modification | Rank | Result Type |
|---|---|---|---|---|---|---|---|---|---|---|---|
| 918.4965 | 918.5496 | 0.0531 | 58 | 279 | 286 | IPEITMSK | | | | | Mascot |
| 1215.6005 | 1215.616 | 0.0155 | 13 | 71 | 80 | THYVINEDPK | | | | | Mascot |
| 1644.717 | 1644.8524 | 0.1354 | 82 | 102 | 117 | DTSSGSAAASFEQMQK | | | | | Mascot |
| 1796.9078 | 1796.9176 | 0.0098 | 5 | 239 | 253 | HILTAAYDTGFRFER | | | | | Mascot |
| 1954.8745 | 1954.8923 | 0.0178 | 9 | 54 | 70 | TQNIYEMQEAGAQGVCR | | | Carbamidomethyl (C)[16] | | Mascot |
| 1954.8745 | 1954.8923 | 0.0178 | 9 | 54 | 70 | TQNIYEMQEAGAQGVCR | 146 | 100 | Carbamidomethyl (C)[16] | | Mascot |
| 1970.8695 | 1970.8909 | 0.0214 | 11 | 54 | 70 | TQNIYEMQEAGAQGVCR | | | Carbamidomethyl (C)[16], Oxidation (M)[7] | | Mascot |

14 vitellogenin 1 [Danio rerio] gi|21391474 21118.2 9 7 137 100 89 100

**Peptide Information**

| Calc. Mass | Obsrv. Mass | ± da | ± ppm | Start Seq. | End Seq. | Sequence | Ion Score | C. I. % | Modification | Rank | Result Type |
|---|---|---|---|---|---|---|---|---|---|---|---|
| 1215.6005 | 1215.616 | 0.0155 | 13 | 164 | 173 | THYVINEDPK | | | | | Mascot |
| 1238.7831 | 1238.7933 | 0.0102 | 8 | 135 | 145 | GILNILQLNLK | | | | | Mascot |
| 1259.5797 | 1259.5946 | 0.0149 | 12 | 182 | 191 | SKDLSHCQER | | | Carbamidomethyl (C)[7] | | Mascot |
| 1695.8999 | 1695.9147 | 0.0148 | 9 | 119 | 134 | VFAPAGVSPTVMNLHR | | | | | Mascot |
| 1699.9377 | 1699.9528 | 0.0151 | 9 | 34 | 49 | YEALLLGGLPQEGLAR | | | | | Mascot |
| 1699.9377 | 1699.9528 | 0.0151 | 9 | 34 | 49 | YEALLLGGLPQEGLAR | 89 | 100 | | | Mascot |
| 1711.8948 | 1711.912 | 0.0172 | 10 | 119 | 134 | VFAPAGVSPTVMNLHR | 4 | 0 | Oxidation (M)[12] | | Mascot |
| 1711.8948 | 1711.912 | 0.0172 | 10 | 119 | 134 | VFAPAGVSPTVMNLHR | | | Oxidation (M)[12] | | Mascot |
| 1796.8928 | 1796.9176 | 0.0248 | 14 | 72 | 86 | LMDPLLYEYAGTWPK | | | | | Mascot |
| 1812.8877 | 1812.9053 | 0.0176 | 10 | 72 | 86 | LMDPLLYEYAGTWPK | | | Oxidation (M)[2] | | Mascot |
| 1936.9181 | 1936.937 | 0.0189 | 10 | 147 | 163 | TQNIYELQEAGAQGVCR | | | Carbamidomethyl (C)[16] | | Mascot |

15 vitellogenin 2 isoform 1 [Danio rerio] gi|113678458 179782.8 8.7 15 129 100 98 100

**Peptide Information**

| Calc. Mass | Obsrv. Mass | ± da | ± ppm | Start Seq. | End Seq. | Sequence | Ion Score | C. I. % | Modification | Rank | Result Type |
|---|---|---|---|---|---|---|---|---|---|---|---|
| 1055.552 | 1055.5625 | 0.0105 | 10 | 20 | 28 | LVPEFAHDK | | | | | Mascot |
| 1097.6677 | 1097.6796 | 0.0119 | 11 | 514 | 523 | VQVDAILALR | | | | | Mascot |
| 1097.6677 | 1097.6796 | 0.0119 | 11 | 514 | 523 | VQVDAILALR | 98 | 100 | | | Mascot |
| 1115.6055 | 1115.7063 | 0.1008 | 90 | 1557 | 1566 | QVILNGQESK | 7 | 0 | | | Mascot |
| 1115.6055 | 1115.7063 | 0.1008 | 90 | 1557 | 1566 | QVILNGQESK | | | | | Mascot |
| 1149.6779 | 1149.6906 | 0.0127 | 11 | 333 | 341 | FVQLVQFLR | | | | | Mascot |
| 1177.606 | 1177.6561 | 0.0501 | 43 | 1146 | 1156 | STGSSLEQIQK | | | | | Mascot |
| 1226.6991 | 1226.71 | 0.0109 | 9 | 472 | 482 | NDIPEITLALK | | | | | Mascot |

| Calc. Mass | Obsrv. Mass | ± da | ± ppm | Start Seq. | End Seq. | Sequence | Ion Score | C. I. % | Modification | Rank | Result Type |
|---|---|---|---|---|---|---|---|---|---|---|---|
| 1477.91 | 1477.9259 | 0.0159 | 11 | 532 | 544 | LVQPVALQLVLER | | | | | Mascot |
| 1648.9203 | 1648.8413 | -0.079 | -48 | 483 | 498 | VLGNAGHPSSLKPIMK | | | | | Mascot |
| 1666.8799 | 1666.894 | 0.0141 | 8 | 668 | 683 | AYLAGAATDVIEFGLR | | | | | Mascot |
| 1682.8384 | 1682.8958 | 0.0574 | 34 | 1198 | 1212 | VQFIASSIAENDNFK | | | | | Mascot |
| 1689.9323 | 1689.9437 | 0.0114 | 7 | 119 | 134 | VFAPAGVSPSVLNLYR | | | | | Mascot |
| 1737.699 | 1737.9172 | 0.2182 | 126 | 1616 | 1631 | ETTDAHVACRCTEQCA | | | | | Mascot |
| 1955.0232 | 1954.8923 | -0.1309 | -67 | 1016 | 1033 | AEGPAVEKLEFEVQVGPR | | | | | Mascot |
| 1955.0232 | 1954.8923 | -0.1309 | -67 | 1016 | 1033 | AEGPAVEKLEFEVQVGPR | | | | | Mascot |
| 2233.2446 | 2233.1025 | -0.1421 | -64 | 425 | 445 | IATIPALREVVMLGYGSMIAK | | | | | Mascot |
| 2938.5164 | 2938.5325 | 0.0161 | 5 | 446 | 471 | YCVAVPTCPAELLRPIHEIATEAISK | | | Carbamidomethyl (C)[2,8] | | Mascot |

| | | | | | | | | | |
|---|---|---|---|---|---|---|---|---|---|
| 16 | PREDICTED: vitellogenin-like, partial [Danio rerio] | gi\|326681170 | 93358.3 | 9.2 | 10 | 122 | 100 | 98 | 100 |

**Peptide Information**

| Calc. Mass | Obsrv. Mass | ± da | ± ppm | Start Seq. | End Seq. | Sequence | Ion Score | C. I. % | Modification | Rank | Result Type |
|---|---|---|---|---|---|---|---|---|---|---|---|
| 1055.552 | 1055.5625 | 0.0105 | 10 | 20 | 28 | LVPEFAHDK | | | | | Mascot |
| 1097.6677 | 1097.6796 | 0.0119 | 11 | 514 | 523 | VQVDAILALR | | | | | Mascot |
| 1097.6677 | 1097.6796 | 0.0119 | 11 | 514 | 523 | VQVDAILALR | 98 | 100 | | | Mascot |
| 1149.6779 | 1149.6906 | 0.0127 | 11 | 333 | 341 | FVQLVQFLR | | | | | Mascot |
| 1226.6991 | 1226.71 | 0.0109 | 9 | 472 | 482 | NDIPEITLALK | | | | | Mascot |
| 1477.91 | 1477.9259 | 0.0159 | 11 | 532 | 544 | LVQPVALQLVLER | | | | | Mascot |
| 1648.9203 | 1648.8413 | -0.079 | -48 | 483 | 498 | VLGNAGHPSSLKPIMK | | | | | Mascot |
| 1666.8799 | 1666.894 | 0.0141 | 8 | 668 | 683 | AYLAGAATDVIEFGLR | | | | | Mascot |
| 1689.9323 | 1689.9437 | 0.0114 | 7 | 119 | 134 | VFAPAGVSPSVLNLYR | | | | | Mascot |
| 2233.2446 | 2233.1025 | -0.1421 | -64 | 425 | 445 | IATIPALREVVMLGYGSMIAK | | | | | Mascot |
| 2938.5164 | 2938.5325 | 0.0161 | 5 | 446 | 471 | YCVAVPTCPAELLRPIHEIATEAISK | | | Carbamidomethyl (C)[2,8] | | Mascot |

| | | | | | | | | | |
|---|---|---|---|---|---|---|---|---|---|
| 17 | Unknown (protein for MGC:174137) [Danio rerio] | gi\|152012733 | 17051.5 | 7.71 | 3 | 103 | 100 | 89 | 100 |

**Peptide Information**

| Calc. Mass | Obsrv. Mass | ± da | ± ppm | Start Seq. | End Seq. | Sequence | Ion Score | C. I. % | Modification | Rank | Result Type |
|---|---|---|---|---|---|---|---|---|---|---|---|
| 874.4669 | 874.5159 | 0.049 | 56 | 87 | 94 | DPFVPATK | | | | | Mascot |
| 1699.9377 | 1699.9528 | 0.0151 | 9 | 34 | 49 | YEALLLGGLPQEGLAR | | | | | Mascot |
| 1699.9377 | 1699.9528 | 0.0151 | 9 | 34 | 49 | YEALLLGGLPQEGLAR | 89 | 100 | | | Mascot |
| 1796.8928 | 1796.9176 | 0.0248 | 14 | 72 | 86 | LMDPLLYEYAGTWPK | | | | | Mascot |
| 1812.8877 | 1812.9053 | 0.0176 | 10 | 72 | 86 | LMDPLLYEYAGTWPK | | | Oxidation (M)[2] | | Mascot |

| 18 | novel protein similar to vitellogenin 1 (vg1) [Danio rerio] | gi\|94732724 | 24001.7 | 5.6 | 5 | 95 | 99.999 | 71 | 99.998 |
|---|---|---|---|---|---|---|---|---|---|

Peptide Information

| Calc. Mass | Obsrv. Mass | ± da | ± ppm | Start Seq. | End Seq. | Sequence | Ion Score | C. I. % | Modification | Rank | Result Type |
|---|---|---|---|---|---|---|---|---|---|---|---|
| 1115.6935 | 1115.7063 | 0.0128 | 11 | 128 | 136 | FVQLVQLLR | 71 | 99.998 | | | Mascot |
| 1115.6935 | 1115.7063 | 0.0128 | 11 | 128 | 136 | FVQLVQLLR | | | | | Mascot |
| 1177.6462 | 1177.6561 | 0.0099 | 8 | 55 | 64 | QTLAFVEIEK | | | | | Mascot |
| 1706.025 | 1706.0416 | 0.0166 | 10 | 159 | 174 | WLLDALPAVGTPVIIK | | | | | Mascot |
| 1796.9581 | 1796.9176 | -0.0405 | -23 | 137 | 151 | LSTLDKFEAIWAQFK | | | | | Mascot |
| 2326.2 | 2326.2205 | 0.0205 | 9 | 78 | 97 | GSLQYEFATEILQTPIQLM K | | | Oxidation (M)[19] | | Mascot |

| 19 | novel protein similar to vitellogenin 1 (vg1) [Danio rerio] | gi\|94732726 | 166271.1 | 8.99 | 14 | 61 | 96.299 | 19 | 0 |
|---|---|---|---|---|---|---|---|---|---|

Peptide Information

| Calc. Mass | Obsrv. Mass | ± da | ± ppm | Start Seq. | End Seq. | Sequence | Ion Score | C. I. % | Modification | Rank | Result Type |
|---|---|---|---|---|---|---|---|---|---|---|---|
| 821.3788 | 821.4716 | 0.0928 | 113 | 1476 | 1482 | SDWQASK | | | | | Mascot |
| 821.3788 | 821.4716 | 0.0928 | 113 | 1476 | 1482 | SDWQASK | 4 | 0 | | | Mascot |
| 918.5267 | 918.5496 | 0.0229 | 25 | 329 | 335 | TRPNFRK | | | | | Mascot |
| 926.473 | 926.5511 | 0.0781 | 84 | 1 | 7 | PQTYVYR | | | | | Mascot |
| 1023.5356 | 1023.5204 | -0.0152 | -15 | 168 | 176 | DVGLSYLEK | 19 | 0 | | | Mascot |
| 1023.5356 | 1023.5204 | -0.0152 | -15 | 168 | 176 | DVGLSYLEK | | | | | Mascot |
| 1115.6306 | 1115.7063 | 0.0757 | 68 | 656 | 665 | TDGIQEVLLK | 14 | 0 | | | Mascot |
| 1115.6306 | 1115.7063 | 0.0757 | 68 | 656 | 665 | TDGIQEVLLK | | | | | Mascot |
| 1137.6813 | 1137.6852 | 0.0039 | 3 | 841 | 850 | GKIHTLMPLK | | | | | Mascot |
| 1149.5754 | 1149.6906 | 0.1152 | 100 | 177 | 185 | CIECQQKIK | | | Carbamidomethyl (C)[1] | | Mascot |
| 1177.6688 | 1177.6561 | -0.0127 | -11 | 455 | 466 | VLGNAAHPASIK | | | | | Mascot |
| 1395.7552 | 1395.7643 | 0.0091 | 7 | 165 | 176 | IMKDVGLSYLEK | | | | | Mascot |
| 1638.7865 | 1638.8628 | 0.0763 | 47 | 418 | 431 | YCSGALECPVELLK | | | Carbamidomethyl (C)[2,8] | | Mascot |
| 1666.8325 | 1666.894 | 0.0615 | 37 | 1137 | 1151 | MCADAVLMSKQTIAK | | | Carbamidomethyl (C)[2] | | Mascot |
| 1682.9587 | 1682.8958 | -0.0629 | -37 | 455 | 470 | VLGNAAHPASIKYITK | | | | | Mascot |
| 1840.9679 | 1840.9137 | -0.0542 | -29 | 471 | 485 | FLPIFGTHIHQFPMR | | | | | Mascot |
| 2233.2161 | 2233.1025 | -0.1136 | -51 | 296 | 313 | EVHEDVPLKYIHLIQLMR | | | | | Mascot |

| 20 | PREDICTED: vitellogenin-1 [Danio rerio] | gi\|326678656 | 171273.8 | 8.99 | 12 | 53 | 78.7 | 19 | 0 |
|---|---|---|---|---|---|---|---|---|---|

Peptide Information

| Calc. Mass | Obsrv. Mass | ± da | ± ppm | Start | End | Sequence | Ion | C. I. % | Modification | Rank | Result Type |
|---|---|---|---|---|---|---|---|---|---|---|---|

| | | | | Seq. | Seq. | | Score | | | | |
|---|---|---|---|---|---|---|---|---|---|---|---|
| 821.3788 | 821.4716 | 0.0928 | 113 | 1522 | 1528 | SDWQASK | | | | | Mascot |
| 821.3788 | 821.4716 | 0.0928 | 113 | 1522 | 1528 | SDWQASK | 4 | 0 | | | Mascot |
| 918.5267 | 918.5496 | 0.0229 | 25 | 355 | 361 | TRPNFRK | | | | | Mascot |
| 1023.5356 | 1023.5204 | -0.0152 | -15 | 194 | 202 | DVGLSYLEK | 19 | 0 | | | Mascot |
| 1023.5356 | 1023.5204 | -0.0152 | -15 | 194 | 202 | DVGLSYLEK | | | | | Mascot |
| 1115.6306 | 1115.7063 | 0.0757 | 68 | 682 | 691 | TDGIQEVLLK | 14 | 0 | | | Mascot |
| 1115.6306 | 1115.7063 | 0.0757 | 68 | 682 | 691 | TDGIQEVLLK | | | | | Mascot |
| 1137.6813 | 1137.6852 | 0.0039 | 3 | 867 | 876 | GKIHTLMPLK | | | | | Mascot |
| 1149.5754 | 1149.6906 | 0.1152 | 100 | 203 | 211 | CIECQQKIK | | | Carbamidomethyl (C)[1] | | Mascot |
| 1177.6688 | 1177.6561 | -0.0127 | -11 | 481 | 492 | VLGNAAHPASIK | | | | | Mascot |
| 1395.7552 | 1395.7643 | 0.0091 | 7 | 191 | 202 | IMKDVGLSYLEK | | | | | Mascot |
| 1638.7865 | 1638.8628 | 0.0763 | 47 | 444 | 457 | YCSGALECPVELLK | | | Carbamidomethyl (C)[2,8] | | Mascot |
| 1682.9587 | 1682.8958 | -0.0629 | -37 | 481 | 496 | VLGNAAHPASIKYITK | | | | | Mascot |
| 1840.9679 | 1840.9137 | -0.0542 | -29 | 497 | 511 | FLPIFGTHIHQFPMR | | | | | Mascot |
| 2233.2161 | 2233.1025 | -0.1136 | -51 | 322 | 339 | EVHEDVPLKYIHLIQLMR | | | | | Mascot |

| | | | | | | | |
|---|---|---|---|---|---|---|---|
| 21 | signal-induced proliferation-associated 1-like protein 1 [Danio rerio] | gi\|113462023 | 187789.3 | 8.87 | 21 | 48 | 24.426 |

**Peptide Information**

| Calc. Mass | Obsrv. Mass | ± da | ± ppm | Start Seq. | End Seq. | Sequence | Ion Score | C. I. % | Modification | Rank | Result Type |
|---|---|---|---|---|---|---|---|---|---|---|---|
| 856.5363 | 856.5385 | 0.0022 | 3 | 1676 | 1682 | LQAQIKR | | | | | Mascot |
| 874.4893 | 874.5159 | 0.0266 | 30 | 1160 | 1166 | SGKWLQR | | | | | Mascot |
| 1055.5956 | 1055.5625 | -0.0331 | -31 | 1526 | 1535 | SPTTRGAPLR | | | | | Mascot |
| 1177.592 | 1177.6561 | 0.0641 | 54 | 1472 | 1483 | LSSSGPSSSGRR | | | | | Mascot |
| 1215.6997 | 1215.616 | -0.0837 | -69 | 793 | 802 | FPFISLAHKR | | | | | Mascot |
| 1238.6376 | 1238.7933 | 0.1557 | 126 | 781 | 792 | HVTSTPIDPSGK | | | | | Mascot |
| 1379.743 | 1379.7687 | 0.0257 | 19 | 1390 | 1401 | QGATSKYLIGWR | | | | | Mascot |
| 1477.7355 | 1477.9259 | 0.1904 | 129 | 864 | 877 | DVIGWTLGSPASMK | | | Oxidation (M)[13] | | Mascot |
| 1647.7981 | 1647.9065 | 0.1084 | 66 | 999 | 1012 | GCSELHRMPVVEYK | | | | | Mascot |
| 1652.781 | 1652.8809 | 0.0999 | 60 | 1346 | 1361 | HSVSPAVMSSSHSSPR | | | | | Mascot |
| 1666.8258 | 1666.894 | 0.0682 | 41 | 268 | 282 | SDDLGPPPKPWVCQK | | | | | Mascot |
| 1682.8245 | 1682.8958 | 0.0713 | 42 | 1167 | 1181 | EEAVASRPPAGQEWR | | | | | Mascot |
| 1721.8817 | 1721.9209 | 0.0392 | 23 | 1402 | 1417 | KPGSTINSVDFGDTRK | | | | | Mascot |
| 1737.9659 | 1737.9172 | -0.0487 | -28 | 688 | 701 | NIRSHFQHVFVVVR | | | | | Mascot |
| 1812.8915 | 1812.9053 | 0.0138 | 8 | 440 | 454 | SNILEHVDLGAYYYR | | | | | Mascot |

| | | | | | | | | | | | |
|---|---|---|---|---|---|---|---|---|---|---|---|
| 1844.0198 | 1843.9298 | -0.09 | -49 | 1652 | 1667 | VSQLEALVKMLQEDLK | | | | | Mascot |
| 1860.0146 | 1859.9304 | -0.0842 | -45 | 1652 | 1667 | VSQLEALVKMLQEDLK | | | Oxidation (M)[10] | | Mascot |
| 1886.9388 | 1886.811 | -0.1278 | -68 | 1143 | 1159 | SMPEGDLHTTGTLLSRR | | | Oxidation (M)[2] | | Mascot |
| 1970.9965 | 1970.8909 | -0.1056 | -54 | 1141 | 1158 | QKSMPEGDLHTTGTLLS R | | | | | Mascot |
| 2225.0364 | 2225.1382 | 0.1018 | 46 | 1545 | 1564 | MHGDLSASDTSLADLHD RQR | | | | | Mascot |
| 2233.1709 | 2233.1025 | -0.0684 | -31 | 1639 | 1660 | LSPSEGPSSYGGKVSQL EALVK | | | | | Mascot |
| 2938.459 | 2938.5325 | 0.0735 | 25 | 640 | 663 | DYEIMFHVSTMLPYTPNN KQQLLR | | | | | Mascot |

| | | | | | | | | | | |
|---|---|---|---|---|---|---|---|---|---|---|
| 22 | HORMA domain-containing protein 1 [Danio rerio] | gi\|50539782 | 41017.5 | 6.07 | 4 | 45 | 0 | 36 | 91.951 | |

**Peptide Information**

| Calc. Mass | Obsrv. Mass | ± da | ± ppm | Start Seq. | End Seq. | Sequence | Ion Score | C. I. % | Modification | Rank | Result Type |
|---|---|---|---|---|---|---|---|---|---|---|---|
| 821.4549 | 821.4716 | 0.0167 | 20 | 148 | 154 | SSMLLVR | | | Oxidation (M)[3] | | Mascot |
| 821.4549 | 821.4716 | 0.0167 | 20 | 148 | 154 | SSMLLVR | 36 | 91.951 | Oxidation (M)[3] | | Mascot |
| 1055.4608 | 1055.5625 | 0.1017 | 96 | 1 | 8 | MTCEQKSR | | | Carbamidomethyl (C)[3], Oxidation (M)[1] | | Mascot |
| 1638.7574 | 1638.8628 | 0.1054 | 64 | 133 | 147 | NGQSLTKMACDNTQK | | | | | Mascot |
| 1695.7789 | 1695.9147 | 0.1358 | 80 | 133 | 147 | NGQSLTKMACDNTQK | | | Carbamidomethyl (C)[10] | | Mascot |
| 1711.7738 | 1711.912 | 0.1382 | 81 | 133 | 147 | NGQSLTKMACDNTQK | | | Carbamidomethyl (C)[10], Oxidation (M)[8] | | Mascot |
| 1711.7738 | 1711.912 | 0.1382 | 81 | 133 | 147 | NGQSLTKMACDNTQK | | | Carbamidomethyl (C)[10], Oxidation (M)[8] | | Mascot |
| 2225.1094 | 2225.1382 | 0.0288 | 13 | 92 | 109 | YLRMVLLSIYCDPDNPQK | | | Carbamidomethyl (C)[11] | | Mascot |

| | | | | | | | | | | |
|---|---|---|---|---|---|---|---|---|---|---|
| 23 | titin a [Danio rerio] | gi\|110005909 | 3646149.2 | 6.18 | 70 | 44 | 0 | 8 | 0 | |

**Peptide Information**

| Calc. Mass | Obsrv. Mass | ± da | ± ppm | Start Seq. | End Seq. | Sequence | Ion Score | C. I. % | Modification | Rank | Result Type |
|---|---|---|---|---|---|---|---|---|---|---|---|
| 821.4185 | 821.4716 | 0.0531 | 65 | 11267 | 11273 | LTVEGMR | 3 | 0 | Oxidation (M)[6] | | Mascot |
| 821.4767 | 821.4716 | -0.0051 | -6 | 9353 | 9359 | EVFLSVK | | | | | Mascot |
| 842.5094 | 842.5182 | 0.0088 | 10 | 15232 | 15239 | LSIPGVTR | | | | | Mascot |
| 856.4886 | 856.5385 | 0.0499 | 58 | 7306 | 7312 | LTLQEPR | | | | | Mascot |
| 870.493 | 870.5502 | 0.0572 | 66 | 8546 | 8553 | TDIPLPSK | | | | | Mascot |
| 874.4992 | 874.5159 | 0.0167 | 19 | 22772 | 22779 | VSVVDVTR | | | | | Mascot |
| 882.4679 | 882.5753 | 0.1074 | 122 | 2876 | 2882 | YKVSTER | | | | | Mascot |
| 888.4785 | 888.538 | 0.0595 | 67 | 9303 | 9309 | EKDLDLR | | | | | Mascot |
| 888.5036 | 888.538 | 0.0344 | 39 | 31597 | 31603 | IIEEEKK | | | | | Mascot |
| 918.5077 | 918.5496 | 0.0419 | 46 | 22434 | 22441 | VTGLLEMR | | | | | Mascot |
| 926.5345 | 926.5511 | 0.0166 | 18 | 9484 | 9490 | YEIVFKK | | | | | Mascot |

| | | | | | | | | | | |
|---|---|---|---|---|---|---|---|---|---|---|
| 1023.4775 | 1023.5204 | 0.0429 | 42 | 29814 | 29822 | AMIATSEER | 13 | 0 | Oxidation (M)[2] | Mascot |
| 1023.5217 | 1023.5204 | -0.0013 | -1 | 7354 | 7362 | DIRTGGNYK | | | | Mascot |
| 1045.5347 | 1045.5737 | 0.039 | 37 | 7144 | 7153 | CQIGGTPEIK | | | | Mascot |
| 1055.552 | 1055.5625 | 0.0105 | 10 | 1809 | 1816 | EELLHWTK | | | | Mascot |
| 1097.6313 | 1097.6796 | 0.0483 | 44 | 11318 | 11327 | TVLTHVDGKK | | | | Mascot |
| 1097.679 | 1097.6796 | 0.0006 | 1 | 19211 | 19219 | RPVIDRTIK | | | | Mascot |
| 1115.6418 | 1115.7063 | 0.0645 | 58 | 2929 | 2937 | EITVLEKQR | | | | Mascot |
| 1115.6418 | 1115.7063 | 0.0645 | 58 | 2929 | 2937 | EITVLEKQR | | | | Mascot |
| 1121.6313 | 1121.6111 | -0.0202 | -18 | 16022 | 16030 | ITNYVVEKR | | | | Mascot |
| 1137.5834 | 1137.6852 | 0.1018 | 89 | 29617 | 29625 | NPVTMTRYR | | | | Mascot |
| 1149.6626 | 1149.6906 | 0.028 | 24 | 23110 | 23118 | ILIQDTRYK | | | | Mascot |
| 1177.6497 | 1177.6561 | 0.0064 | 5 | 7707 | 7716 | ETTVEMVKLK | | | | Mascot |
| 1214.6627 | 1214.6653 | 0.0026 | 2 | 3752 | 3762 | SPEVTEIQIAK | | | | Mascot |
| 1215.6514 | 1215.616 | -0.0354 | -29 | 19309 | 19319 | CNVPKNLQATK | | | | Mascot |
| 1226.635 | 1226.71 | 0.075 | 61 | 19949 | 19958 | LEHPGKTWMK | | | | Mascot |
| 1238.6449 | 1238.7933 | 0.1484 | 120 | 21716 | 21726 | ITGYIVEMQGK | | | | Mascot |
| 1259.6266 | 1259.5946 | -0.032 | -25 | 19551 | 19562 | ASEQPGPVAEFK | | | | Mascot |
| 1379.7417 | 1379.7687 | 0.027 | 20 | 26957 | 26968 | NTEYSTTLIIPK | | | | Mascot |
| 1382.6924 | 1382.7999 | 0.1075 | 78 | 14817 | 14827 | DWSRATERPHK | | | | Mascot |
| 1395.705 | 1395.7643 | 0.0593 | 42 | 24878 | 24889 | LAFTSITNNCVR | | | Carbamidomethyl (C)[10] | Mascot |
| 1463.8944 | 1463.91 | 0.0156 | 11 | 123 | 136 | LDVRVTGIPAPVVK | | | | Mascot |
| 1463.8944 | 1463.91 | 0.0156 | 11 | 123 | 136 | LDVRVTGIPAPVVK | | | | Mascot |
| 1477.8624 | 1477.9259 | 0.0635 | 43 | 32284 | 32297 | SPEPLVKSPVPSLK | | | | Mascot |
| 1555.775 | 1555.7659 | -0.0091 | -6 | 25283 | 25295 | VTGLEEGLQYQYR | | | | Mascot |
| 1622.6752 | 1622.8712 | 0.196 | 121 | 3468 | 3481 | KDYEGEYSCTASNR | | | | Mascot |
| 1622.8271 | 1622.8712 | 0.0441 | 27 | 30745 | 30759 | QTVELDVADVPDPPK | | | | Mascot |
| 1638.9061 | 1638.8628 | -0.0433 | -26 | 7730 | 7743 | DIDIVELLRNVDPK | | | | Mascot |
| 1644.8374 | 1644.8524 | 0.015 | 9 | 507 | 520 | HQEMLATKQQVTSK | | | Oxidation (M)[4] | Mascot |
| 1647.7869 | 1647.9065 | 0.1196 | 73 | 22715 | 22728 | VYSAVTTCNKMTWK | | | Oxidation (M)[11] | Mascot |
| 1648.8176 | 1648.8413 | 0.0237 | 14 | 3971 | 3985 | FSSEKPSPEKADAQK | | | | Mascot |
| 1652.8239 | 1652.8809 | 0.057 | 34 | 17318 | 17333 | ETWGGVSSGSTSTRLK | | | | Mascot |
| 1666.8945 | 1666.894 | -0.0005 | 0 | 8731 | 8744 | IPKQDISQTKPHMK | | | Oxidation (M)[13] | Mascot |
| 1682.8418 | 1682.8958 | 0.054 | 32 | 21716 | 21730 | ITGYIVEMQGKNSDK | | | | Mascot |
| 1689.9242 | 1689.9437 | 0.0195 | 12 | 207 | 221 | TIISASQISQTRQTR | | | | Mascot |
| 1695.8912 | 1695.9147 | 0.0235 | 14 | 22230 | 22244 | NTDFTTTLSVKEAIR | | | | Mascot |
| 1699.8538 | 1699.9528 | 0.099 | 58 | 1945 | 1959 | QVVSFTQTLEDAYAK | | | | Mascot |

| | | | | | | | | | | | |
|---|---|---|---|---|---|---|---|---|---|---|---|
| 1699.8538 | 1699.9528 | 0.099 | 58 | 1945 | 1959 | QVVSFTQTLEDAYAK | | | | | Mascot |
| 1705.9958 | 1706.0416 | 0.0458 | 27 | 8433 | 8448 | EPSKKPGAPLLLATQR | | | | | Mascot |
| 1711.7843 | 1711.912 | 0.1277 | 75 | 9785 | 9799 | EASLSADKDQEMFPK | | | Oxidation (M)[12] | | Mascot |
| 1711.8861 | 1711.912 | 0.0259 | 15 | 32021 | 32035 | RDEEAPTQIVPDITK | | | | | Mascot |
| 1721.9332 | 1721.9209 | -0.0123 | -7 | 10053 | 10067 | AVAFAKPEHLEERPK | | | | | Mascot |
| 1737.917 | 1737.9172 | 0.0002 | 0 | 19341 | 19356 | IGFGEPSEVPEKHLAK | | | | | Mascot |
| 1796.8094 | 1796.9176 | 0.1082 | 60 | 18756 | 18771 | HPFDPPSEPLNCCVNK | | | | | Mascot |
| 1812.9126 | 1812.9053 | -0.0073 | -4 | 21602 | 21618 | DSVSLIWDPPVIDGGSR | | | | | Mascot |
| 1824.9602 | 1824.9142 | -0.046 | -25 | 29529 | 29544 | LVEGTEYVFRVAATNR | | | | | Mascot |
| 1840.9301 | 1840.9137 | -0.0164 | -9 | 20615 | 20631 | DTATLAWTKPKHDGGSR | | | | | Mascot |
| 1843.9331 | 1843.9298 | -0.0033 | -2 | 2604 | 2619 | NQELKPSANVQIMSER | | | | | Mascot |
| 1859.928 | 1859.9304 | 0.0024 | 1 | 2604 | 2619 | NQELKPSANVQIMSER | | | Oxidation (M)[13] | | Mascot |
| 1886.9857 | 1886.811 | -0.1747 | -93 | 19335 | 19352 | VSAVNKIGFGEPSEVPEK | | | | | Mascot |
| 1906.9504 | 1906.8885 | -0.0619 | -32 | 20182 | 20200 | VSAVNQYGTGDVTQSGP VK | | | | | Mascot |
| 1936.9611 | 1936.937 | -0.0241 | -12 | 25798 | 25815 | IGPSEPSPETDPQVAKER | | | | | Mascot |
| 1954.9691 | 1954.8923 | -0.0768 | -39 | 11453 | 11469 | VVRPMYGVELFDGETAR | | | Oxidation (M)[5] | | Mascot |
| 1954.9691 | 1954.8923 | -0.0768 | -39 | 11453 | 11469 | VVRPMYGVELFDGETAR | | | Oxidation (M)[5] | | Mascot |
| 1970.877 | 1970.8909 | 0.0139 | 7 | 16626 | 16641 | DLWMPVTESCTRTSCK | | | Carbamidomethyl (C)[10,15] | | Mascot |
| 2083.0276 | 2082.9944 | -0.0332 | -16 | 13143 | 13161 | WTFGENALTAGDRVSMV TK | | | | | Mascot |
| 2141.9907 | 2142.2322 | 0.2415 | 113 | 15348 | 15366 | AENKYGISEACETEEVEI K | | | | | Mascot |
| 2211.1438 | 2211.125 | -0.0188 | -9 | 6982 | 7001 | SCEIALRGDTAILELHNID K | | | | | Mascot |
| 2225.1382 | 2225.1382 | 0 | 0 | 29539 | 29559 | VAATNRFGIGGFLQSEVV ECK | | | | | Mascot |
| 2232.9575 | 2233.1025 | 0.145 | 65 | 1525 | 1542 | MINEFGYCSLDYEVAYSR | | | Carbamidomethyl (C)[8], Oxidation (M)[1] | | Mascot |
| 2283.1899 | 2283.198 | 0.0081 | 4 | 3763 | 3783 | EHPSQVMSALTESVTPLT IVK | | | Oxidation (M)[7] | | Mascot |
| 2284.1819 | 2284.1936 | 0.0117 | 5 | 21598 | 21618 | EITKDSVSLIWDPPVIDGG SR | | | | | Mascot |
| 2326.1421 | 2326.2205 | 0.0784 | 34 | 16860 | 16881 | AVNAAGESEPAYVPEPV RAQDR | | | | | Mascot |
| 2914.4026 | 2914.5107 | 0.1081 | 37 | 3207 | 3233 | VDGETEVMDYSSKFPPQ ATSSPLTSIK | | | | | Mascot |
| 2938.4502 | 2938.5325 | 0.0823 | 28 | 4621 | 4647 | IQNLEVNVGSSAKFECEI EEAPGVTFK | | | | | Mascot |

| | | | | | | | | | |
|---|---|---|---|---|---|---|---|---|---|
| 24 | PREDICTED: titin [Danio rerio] | gi\|189523699 | 3645668 | 6.16 | 70 | 44 | 0 | 8 | 0 |

**Peptide Information**

| Calc. Mass | Obsrv. Mass | ± da | ± ppm | Start Seq. | End Seq. | Sequence | Ion Score | C. I. % | Modification | Rank | Result Type |
|---|---|---|---|---|---|---|---|---|---|---|---|
| 821.4185 | 821.4716 | 0.0531 | 65 | 11267 | 11273 | LTVEGMR | 3 | 0 | Oxidation (M)[6] | | Mascot |
| 821.4767 | 821.4716 | -0.0051 | -6 | 9353 | 9359 | EVFLSVK | | | | | Mascot |

| | | | | | | | | | | |
|---|---|---|---|---|---|---|---|---|---|---|
| 842.5094 | 842.5182 | 0.0088 | 10 | 15232 | 15239 | LSIPGVTR | | | | Mascot |
| 856.4886 | 856.5385 | 0.0499 | 58 | 7306 | 7312 | LTLQEPR | | | | Mascot |
| 870.4203 | 870.5502 | 0.1299 | 149 | 6908 | 6915 | LSDSGTYK | | | | Mascot |
| 874.4992 | 874.5159 | 0.0167 | 19 | 22772 | 22779 | VSVVDVTR | | | | Mascot |
| 882.4679 | 882.5753 | 0.1074 | 122 | 2876 | 2882 | YKVSTER | | | | Mascot |
| 888.4785 | 888.538 | 0.0595 | 67 | 9303 | 9309 | EKDLDLR | | | | Mascot |
| 888.5036 | 888.538 | 0.0344 | 39 | 31597 | 31603 | IIEEEKK | | | | Mascot |
| 918.5077 | 918.5496 | 0.0419 | 46 | 22434 | 22441 | VTGLLEMR | | | | Mascot |
| 926.5345 | 926.5511 | 0.0166 | 18 | 9484 | 9490 | YEIVFKK | | | | Mascot |
| 1023.4775 | 1023.5204 | 0.0429 | 42 | 29814 | 29822 | AMIATSEER | 13 | 0 | Oxidation (M)[2] | Mascot |
| 1023.5217 | 1023.5204 | -0.0013 | -1 | 7354 | 7362 | DIRTGGNYK | | | | Mascot |
| 1045.5347 | 1045.5737 | 0.039 | 37 | 7144 | 7153 | CQIGGTPEIK | | | | Mascot |
| 1055.552 | 1055.5625 | 0.0105 | 10 | 1809 | 1816 | EELLHWTK | | | | Mascot |
| 1097.6313 | 1097.6796 | 0.0483 | 44 | 11318 | 11327 | TVLTHVDGKK | | | | Mascot |
| 1097.679 | 1097.6796 | 0.0006 | 1 | 19211 | 19219 | RPVIDRTIK | | | | Mascot |
| 1115.6418 | 1115.7063 | 0.0645 | 58 | 2929 | 2937 | EITVLEKQR | | | | Mascot |
| 1115.6418 | 1115.7063 | 0.0645 | 58 | 2929 | 2937 | EITVLEKQR | | | | Mascot |
| 1121.6313 | 1121.6111 | -0.0202 | -18 | 16022 | 16030 | ITNYVVEKR | | | | Mascot |
| 1137.5834 | 1137.6852 | 0.1018 | 89 | 29617 | 29625 | NPVTMTRYR | | | | Mascot |
| 1149.6626 | 1149.6906 | 0.028 | 24 | 23110 | 23118 | ILIQDTRYK | | | | Mascot |
| 1177.6497 | 1177.6561 | 0.0064 | 5 | 7707 | 7716 | ETTVEMVKLK | | | | Mascot |
| 1214.6627 | 1214.6653 | 0.0026 | 2 | 3752 | 3762 | SPEVTEIQIAK | | | | Mascot |
| 1215.6514 | 1215.616 | -0.0354 | -29 | 19309 | 19319 | CNVPKNLQATK | | | | Mascot |
| 1226.635 | 1226.71 | 0.075 | 61 | 19949 | 19958 | LEHPGKTWMK | | | | Mascot |
| 1238.6449 | 1238.7933 | 0.1484 | 120 | 21716 | 21726 | ITGYIVEMQGK | | | | Mascot |
| 1259.6266 | 1259.5946 | -0.032 | -25 | 19551 | 19562 | ASEQPGPVAEFK | | | | Mascot |
| 1379.7417 | 1379.7687 | 0.027 | 20 | 26957 | 26968 | NTEYSTTLIIPK | | | | Mascot |
| 1382.6924 | 1382.7999 | 0.1075 | 78 | 14817 | 14827 | DWSRATERPHK | | | | Mascot |
| 1395.705 | 1395.7643 | 0.0593 | 42 | 24878 | 24889 | LAFTSITNNCVR | | | Carbamidomethyl (C)[10] | Mascot |
| 1463.8944 | 1463.91 | 0.0156 | 11 | 123 | 136 | LDVRVTGIPAPVVK | | | | Mascot |
| 1463.8944 | 1463.91 | 0.0156 | 11 | 123 | 136 | LDVRVTGIPAPVVK | | | | Mascot |
| 1477.8624 | 1477.9259 | 0.0635 | 43 | 32284 | 32297 | SPEPLVKSPVPSLK | | | | Mascot |
| 1555.775 | 1555.7659 | -0.0091 | -6 | 25283 | 25295 | VTGLEEGLQYQYR | | | | Mascot |
| 1622.6752 | 1622.8712 | 0.196 | 121 | 3468 | 3481 | KDYEGEYSCTASNR | | | | Mascot |
| 1622.8271 | 1622.8712 | 0.0441 | 27 | 30745 | 30759 | QTVELDVADVPDPPK | | | | Mascot |
| 1638.9061 | 1638.8628 | -0.0433 | -26 | 7730 | 7743 | DIDIVELLRNVDPK | | | | Mascot |

| | | | | | | | | |
|---|---|---|---|---|---|---|---|---|
| 1644.8374 | 1644.8524 | 0.015 | 9 | 507 | 520 | HQEMLATKQQVTSK | Oxidation (M)[4] | Mascot |
| 1647.7869 | 1647.9065 | 0.1196 | 73 | 22715 | 22728 | VYSAVTTCNKMTWK | Oxidation (M)[11] | Mascot |
| 1648.8806 | 1648.8413 | -0.0393 | -24 | 1364 | 1377 | NSDIISPHKYPHIK | | Mascot |
| 1652.849 | 1652.8809 | 0.0319 | 19 | 8755 | 8768 | TEEKETFSVQVVTR | | Mascot |
| 1666.8945 | 1666.894 | -0.0005 | 0 | 8731 | 8744 | IPKQDISQTKPHMK | Oxidation (M)[13] | Mascot |
| 1682.8418 | 1682.8958 | 0.054 | 32 | 21716 | 21730 | ITGYIVEMQGKNSDK | | Mascot |
| 1689.9242 | 1689.9437 | 0.0195 | 12 | 207 | 221 | TIISASQISQTRQTR | | Mascot |
| 1695.8912 | 1695.9147 | 0.0235 | 14 | 22230 | 22244 | NTDFTTTLSVKEAIR | | Mascot |
| 1699.8538 | 1699.9528 | 0.099 | 58 | 1945 | 1959 | QVVSFTQTLEDAYAK | | Mascot |
| 1699.8538 | 1699.9528 | 0.099 | 58 | 1945 | 1959 | QVVSFTQTLEDAYAK | | Mascot |
| 1705.9958 | 1706.0416 | 0.0458 | 27 | 8433 | 8448 | EPSKKPGAPLLLATQR | | Mascot |
| 1711.7843 | 1711.912 | 0.1277 | 75 | 9785 | 9799 | EASLSADKDQEMFPK | Oxidation (M)[12] | Mascot |
| 1711.8861 | 1711.912 | 0.0259 | 15 | 32021 | 32035 | RDEEAPTQIVPDITK | | Mascot |
| 1721.9332 | 1721.9209 | -0.0123 | -7 | 10053 | 10067 | AVAFAKPEHLEERPK | | Mascot |
| 1737.917 | 1737.9172 | 0.0002 | 0 | 19341 | 19356 | IGFGEPSEVPEKHLAK | | Mascot |
| 1796.8094 | 1796.9176 | 0.1082 | 60 | 18756 | 18771 | HPFDPPSEPLNCCVNK | | Mascot |
| 1812.9126 | 1812.9053 | -0.0073 | -4 | 21602 | 21618 | DSVSLIWDPPVIDGGSR | | Mascot |
| 1824.9602 | 1824.9142 | -0.046 | -25 | 29529 | 29544 | LVEGTEYVFRVAATNR | | Mascot |
| 1840.9301 | 1840.9137 | -0.0164 | -9 | 20615 | 20631 | DTATLAWTKPKHDGGSR | | Mascot |
| 1843.9331 | 1843.9298 | -0.0033 | -2 | 2604 | 2619 | NQELKPSANVQIMSER | | Mascot |
| 1859.928 | 1859.9304 | 0.0024 | 1 | 2604 | 2619 | NQELKPSANVQIMSER | Oxidation (M)[13] | Mascot |
| 1886.9857 | 1886.811 | -0.1747 | -93 | 19335 | 19352 | VSAVNKIGFGEPSEVPEK | | Mascot |
| 1906.9504 | 1906.8885 | -0.0619 | -32 | 20182 | 20200 | VSAVNQYGTGDVTQSGP VK | | Mascot |
| 1936.9611 | 1936.937 | -0.0241 | -12 | 25798 | 25815 | IGPSEPSPETDPQVAKER | | Mascot |
| 1954.9691 | 1954.8923 | -0.0768 | -39 | 11453 | 11469 | VVRPMYGVELFDGETAR | Oxidation (M)[5] | Mascot |
| 1954.9691 | 1954.8923 | -0.0768 | -39 | 11453 | 11469 | VVRPMYGVELFDGETAR | Oxidation (M)[5] | Mascot |
| 1970.877 | 1970.8909 | 0.0139 | 7 | 16626 | 16641 | DLWMPVTESCTRTSCK | Carbamidomethyl (C)[10,15] | Mascot |
| 2083.0276 | 2082.9944 | -0.0332 | -16 | 13143 | 13161 | WTFGENALTAGDRVSMV TK | | Mascot |
| 2141.9907 | 2142.2322 | 0.2415 | 113 | 15348 | 15366 | AENKYGISEACETEEVEI K | | Mascot |
| 2211.1438 | 2211.125 | -0.0188 | -9 | 6982 | 7001 | SCEIALRGDTAILELHNID K | | Mascot |
| 2225.1382 | 2225.1382 | 0 | 0 | 29539 | 29559 | VAATNRFGIGGFLQSEVV ECK | | Mascot |
| 2232.9575 | 2233.1025 | 0.145 | 65 | 1525 | 1542 | MINEFGYCSLDYEVAYSR | Carbamidomethyl (C)[8], Oxidation (M)[1] | Mascot |
| 2283.1899 | 2283.198 | 0.0081 | 4 | 3763 | 3783 | EHPSQVMSALTESVTPLT IVK | Oxidation (M)[7] | Mascot |
| 2284.1819 | 2284.1936 | 0.0117 | 5 | 21598 | 21618 | EITKDSVSLIWDPPVIDGG SR | | Mascot |
| 2326.1421 | 2326.2205 | 0.0784 | 34 | 16860 | 16881 | AVNAAGESEPAYVPEPV RAQDR | | Mascot |

| | | | | | | | |
|---|---|---|---|---|---|---|---|
| 2914.4026 | 2914.5107 | 0.1081 | 37 | 3207 | 3233 | VDGETEVMDYSSKFPPQ ATSSPLTSIK | Mascot |
| 2938.4502 | 2938.5325 | 0.0823 | 28 | 4621 | 4647 | IQNLEVNVGSSAKFECEI EEAPGVTFK | Mascot |

| | | | | | | | | | | |
|---|---|---|---|---|---|---|---|---|---|---|
| 25 | RecName: Full=HORMA domain-containing protein 1 | gi\|145558933 | 40949.4 | 5.97 | 3 | 44 | 0 | 36 | 91.951 |

Peptide Information

| Calc. Mass | Obsrv. Mass | ± da | ± ppm | Start Seq. | End Seq. | Sequence | Ion Score | C. I. % | Modification | Rank | Result Type |
|---|---|---|---|---|---|---|---|---|---|---|---|
| 821.4549 | 821.4716 | 0.0167 | 20 | 148 | 154 | SSMLLVR | | | Oxidation (M)[3] | | Mascot |
| 821.4549 | 821.4716 | 0.0167 | 20 | 148 | 154 | SSMLLVR | 36 | 91.951 | Oxidation (M)[3] | | Mascot |
| 1638.7574 | 1638.8628 | 0.1054 | 64 | 133 | 147 | NGQSLTKMACDNTQK | | | | | Mascot |
| 1695.7789 | 1695.9147 | 0.1358 | 80 | 133 | 147 | NGQSLTKMACDNTQK | | | Carbamidomethyl (C)[10] | | Mascot |
| 1711.7738 | 1711.912 | 0.1382 | 81 | 133 | 147 | NGQSLTKMACDNTQK | | | Carbamidomethyl (C)[10], Oxidation (M)[8] | | Mascot |
| 1711.7738 | 1711.912 | 0.1382 | 81 | 133 | 147 | NGQSLTKMACDNTQK | | | Carbamidomethyl (C)[10], Oxidation (M)[8] | | Mascot |
| 2225.1094 | 2225.1382 | 0.0288 | 13 | 92 | 109 | YLRMVLLSIYCDPDNPQK | | | Carbamidomethyl (C)[11] | | Mascot |

| | | | | | | | |
|---|---|---|---|---|---|---|---|
| 26 | PREDICTED: hypothetical protein LOC100004944 [Danio rerio] | gi\|125854141 | 69244.2 | 6.33 | 12 | 44 | 0 |

Peptide Information

| Calc. Mass | Obsrv. Mass | ± da | ± ppm | Start Seq. | End Seq. | Sequence | Ion Score | C. I. % | Modification | Rank | Result Type |
|---|---|---|---|---|---|---|---|---|---|---|---|
| 874.4265 | 874.5159 | 0.0894 | 102 | 526 | 532 | ENENQLK | | | | | Mascot |
| 1023.533 | 1023.5204 | -0.0126 | -12 | 133 | 140 | RDENKPHK | | | | | Mascot |
| 1023.533 | 1023.5204 | -0.0126 | -12 | 133 | 140 | RDENKPHK | | | | | Mascot |
| 1045.452 | 1045.5737 | 0.1217 | 116 | 472 | 479 | CHYSDHVK | | | Carbamidomethyl (C)[1] | | Mascot |
| 1259.6525 | 1259.5946 | -0.0579 | -46 | 299 | 309 | EAQIKCALNGR | | | Carbamidomethyl (C)[6] | | Mascot |
| 1622.8207 | 1622.8712 | 0.0505 | 31 | 220 | 233 | GIDFDIDIGMRLNK | | | Oxidation (M)[10] | | Mascot |
| 1622.8207 | 1622.8712 | 0.0505 | 31 | 220 | 233 | GIDFDIDIGMRLNK | | | Oxidation (M)[10] | | Mascot |
| 1666.8395 | 1666.894 | 0.0545 | 33 | 391 | 404 | HELKQAQDALQEEK | | | | | Mascot |
| 1689.9105 | 1689.9437 | 0.0332 | 20 | 343 | 357 | GMTGLTQFINNIKPR | | | | | Mascot |
| 1705.9054 | 1706.0416 | 0.1362 | 80 | 343 | 357 | GMTGLTQFINNIKPR | | | Oxidation (M)[2] | | Mascot |
| 1737.8258 | 1737.9172 | 0.0914 | 53 | 2 | 17 | ASKNSSLESLSCPVCR | | | Carbamidomethyl (C)[12] | | Mascot |
| 1796.9211 | 1796.9176 | -0.0035 | -2 | 234 | 249 | IDDLTHEINAVCLVNK | | | | | Mascot |
| 1859.7906 | 1859.9304 | 0.1398 | 75 | 457 | 471 | CTVMKDYHCTVCTNK | | | Carbamidomethyl (C)[1,9] | | Mascot |
| 2211.1147 | 2211.125 | 0.0103 | 5 | 153 | 171 | TTLVNVMVNYMLGVERE DK | | | | | Mascot |
| 2283.2595 | 2283.198 | -0.0615 | -27 | 544 | 563 | IALNTDSLNTLLHIDFLIEK | | | | | Mascot |

| | | | | | | | |
|---|---|---|---|---|---|---|---|
| 27 | novel protein similar to human ankyrin repeat and MYND domain containing 1 (ANKMY1) [Danio rerio] | gi\|148725364 | 49558.8 | 7.96 | 10 | 44 | 0 |

| Calc. Mass | Obsrv. Mass | ± da | ± ppm | Start Seq. | End Seq. | Sequence | Ion Score | C. I. % | Modification | Rank | Result Type |
|---|---|---|---|---|---|---|---|---|---|---|---|
| 918.454 | 918.5496 | 0.0956 | 104 | 380 | 386 | GRSWEQR | | | | | Mascot |
| 1055.5415 | 1055.5625 | 0.021 | 20 | 127 | 135 | TALHVACQR | | | Carbamidomethyl (C)[7] | | Mascot |
| 1137.615 | 1137.6852 | 0.0702 | 62 | 49 | 58 | TDIPLPPEQK | | | | | Mascot |
| 1382.7247 | 1382.7999 | 0.0752 | 54 | 401 | 412 | SNSGRIEPNRPR | | | | | Mascot |
| 1622.7335 | 1622.8712 | 0.1377 | 85 | 227 | 241 | AGADVLMPVMVCDGR | | | Carbamidomethyl (C)[12], Oxidation (M)[7,10] | | Mascot |
| 1622.7335 | 1622.8712 | 0.1377 | 85 | 227 | 241 | AGADVLMPVMVCDGR | | | Carbamidomethyl (C)[12], Oxidation (M)[7,10] | | Mascot |
| 1647.8547 | 1647.9065 | 0.0518 | 31 | 321 | 336 | SAVTSSLLNSPESISR | | | | | Mascot |
| 1648.6301 | 1648.8413 | 0.2112 | 128 | 368 | 379 | CHEVYYCSNTCR | | | Carbamidomethyl (C)[1,7,11] | | Mascot |
| 1689.8234 | 1689.9437 | 0.1203 | 71 | 227 | 242 | AGADVLMPVMVCDGRR | | | | | Mascot |
| 1705.8182 | 1706.0416 | 0.2234 | 131 | 227 | 242 | AGADVLMPVMVCDGRR | | | Oxidation (M)[7] | | Mascot |
| 1721.8131 | 1721.9209 | 0.1078 | 63 | 227 | 242 | AGADVLMPVMVCDGRR | | | Oxidation (M)[7,10] | | Mascot |
| 1937.016 | 1936.937 | -0.079 | -41 | 42 | 58 | LLECGARTDIPLPPEQK | | | Carbamidomethyl (C)[4] | | Mascot |
| 2211.0745 | 2211.125 | 0.0505 | 23 | 321 | 341 | SAVTSSLLNSPESISRMS CNK | | | | | Mascot |
| 2284.0908 | 2284.1936 | 0.1028 | 45 | 321 | 341 | SAVTSSLLNSPESISRMS CNK | | | Carbamidomethyl (C)[19], Oxidation (M)[17] | | Mascot |

| | | | | | | | | | | |
|---|---|---|---|---|---|---|---|---|---|---|
| 28 | novel protein [Danio rerio] | gi\|126540716 | 8191 | 6.01 | 2 | 43 | 0 | 30 | 72.092 | |

Peptide Information

| Calc. Mass | Obsrv. Mass | ± da | ± ppm | Start Seq. | End Seq. | Sequence | Ion Score | C. I. % | Modification | Rank | Result Type |
|---|---|---|---|---|---|---|---|---|---|---|---|
| 1097.679 | 1097.6796 | 0.0006 | 1 | 2 | 11 | PSRVSVAILR | | | | | Mascot |
| 1097.679 | 1097.6796 | 0.0006 | 1 | 2 | 11 | PSRVSVAILR | 30 | 72.092 | | | Mascot |
| 1906.9446 | 1906.8885 | -0.0561 | -29 | 55 | 71 | VNGEGKFISYALSHHFT | | | | | Mascot |

| | | | | | | | |
|---|---|---|---|---|---|---|---|
| 29 | Sipa1l1 protein [Danio rerio] | gi\|190340108 | 188873.8 | 8.64 | 20 | 42 | 0 |

Peptide Information

| Calc. Mass | Obsrv. Mass | ± da | ± ppm | Start Seq. | End Seq. | Sequence | Ion Score | C. I. % | Modification | Rank | Result Type |
|---|---|---|---|---|---|---|---|---|---|---|---|
| 856.5363 | 856.5385 | 0.0022 | 3 | 1703 | 1709 | LQAQIKR | | | | | Mascot |
| 1055.5956 | 1055.5625 | -0.0331 | -31 | 1553 | 1562 | SPTTRGAPLR | | | | | Mascot |
| 1177.592 | 1177.6561 | 0.0641 | 54 | 1499 | 1510 | LSSSGPSSSGRR | | | | | Mascot |
| 1215.6997 | 1215.616 | -0.0837 | -69 | 794 | 803 | FPFISLAHKR | | | | | Mascot |
| 1238.6376 | 1238.7933 | 0.1557 | 126 | 782 | 793 | HVTSTPIDPSGK | | | | | Mascot |
| 1379.743 | 1379.7687 | 0.0257 | 19 | 1417 | 1428 | QGATSKYLIGWR | | | | | Mascot |

| | | | | | | | | | | | |
|---|---|---|---|---|---|---|---|---|---|---|---|
| 1477.7355 | 1477.9259 | 0.1904 | 129 | 865 | 878 | DVIGWTLGSPASMK | | | Oxidation (M)[13] | | Mascot |
| 1647.7981 | 1647.9065 | 0.1084 | 66 | 1000 | 1013 | GCSELHRMPVVEYK | | | | | Mascot |
| 1652.781 | 1652.8809 | 0.0999 | 60 | 1373 | 1388 | HSVSPAVMSSSHSSPR | | | | | Mascot |
| 1666.8258 | 1666.894 | 0.0682 | 41 | 269 | 283 | SDDLGPPPKPWVCQK | | | | | Mascot |
| 1682.7803 | 1682.8958 | 0.1155 | 69 | 891 | 904 | SMNNNTEDFREVVK | | | | | Mascot |
| 1699.8134 | 1699.9528 | 0.1394 | 82 | 1179 | 1194 | VEDSSDRPPSDAPVTK | | | | | Mascot |
| 1699.8134 | 1699.9528 | 0.1394 | 82 | 1179 | 1194 | VEDSSDRPPSDAPVTK | | | | | Mascot |
| 1721.8817 | 1721.9209 | 0.0392 | 23 | 1429 | 1444 | KPGSTINSVDFGDTRK | | | | | Mascot |
| 1737.9659 | 1737.9172 | -0.0487 | -28 | 689 | 702 | NIRSHFQHVFVVVR | | | | | Mascot |
| 1812.8915 | 1812.9053 | 0.0138 | 8 | 441 | 455 | SNILEHVDLGAYYYR | | | | | Mascot |
| 1840.7654 | 1840.9137 | 0.1483 | 81 | 1356 | 1372 | DGSTYSLSDMASYSSNR | | | | | Mascot |
| 1844.0198 | 1843.9298 | -0.09 | -49 | 1679 | 1694 | VSQLEALVKMLQEDLK | | | | | Mascot |
| 1860.0146 | 1859.9304 | -0.0842 | -45 | 1679 | 1694 | VSQLEALVKMLQEDLK | | | Oxidation (M)[10] | | Mascot |
| 2225.0364 | 2225.1382 | 0.1018 | 46 | 1572 | 1591 | MHGDLSASDTSLADLHD RQR | | | | | Mascot |
| 2233.1709 | 2233.1025 | -0.0684 | -31 | 1666 | 1687 | LSPSEGPSSYGGKVSQL EALVK | | | | | Mascot |
| 2938.459 | 2938.5325 | 0.0735 | 25 | 641 | 664 | DYEIMFHVSTMLPYTPNN KQQLLR | | | | | Mascot |

30 PREDICTED: zinc finger protein 836-like [Danio rerio] gi|292610335 57699.7 8.44 13 42 0

**Peptide Information**

| Calc. Mass | Obsrv. Mass | ± da | ± ppm | Start Seq. | End Seq. | Sequence | Ion Score | C. I. % | Modification | Rank | Result Type |
|---|---|---|---|---|---|---|---|---|---|---|---|
| 856.4497 | 856.5385 | 0.0888 | 104 | 218 | 223 | VFFCKR | | | Carbamidomethyl (C)[4] | | Mascot |
| 1023.5179 | 1023.5204 | 0.0025 | 2 | 224 | 232 | CDISFPTLK | | | | | Mascot |
| 1023.5179 | 1023.5204 | 0.0025 | 2 | 224 | 232 | CDISFPTLK | | | | | Mascot |
| 1055.5012 | 1055.5625 | 0.0613 | 58 | 189 | 197 | TTFPCKQCK | | | | | Mascot |
| 1115.6418 | 1115.7063 | 0.0645 | 58 | 38 | 47 | EVASLEAKLR | 14 | 0 | | | Mascot |
| 1115.6418 | 1115.7063 | 0.0645 | 58 | 38 | 47 | EVASLEAKLR | | | | | Mascot |
| 1121.4536 | 1121.6111 | 0.1575 | 140 | 357 | 365 | MHQCLQCDK | | | Oxidation (M)[1] | | Mascot |
| 1177.6398 | 1177.6561 | 0.0163 | 14 | 185 | 194 | QKPKTTFPCK | | | | | Mascot |
| 1215.5687 | 1215.616 | 0.0473 | 39 | 314 | 323 | DGSHFKTHMR | | | | | Mascot |
| 1379.5718 | 1379.7687 | 0.1969 | 143 | 272 | 282 | EKPFHCSECDK | | | Carbamidomethyl (C)[6] | | Mascot |
| 1644.71 | 1644.8524 | 0.1424 | 87 | 1 | 14 | MLSMEVQVDVSCCK | | | Carbamidomethyl (C)[12], Oxidation (M)[1] | | Mascot |
| 1699.7714 | 1699.9528 | 0.1814 | 107 | 352 | 365 | IHSEKMHQCLQCDK | | | | | Mascot |
| 1699.7714 | 1699.9528 | 0.1814 | 107 | 352 | 365 | IHSEKMHQCLQCDK | | | | | Mascot |
| 1737.8137 | 1737.9172 | 0.1035 | 60 | 173 | 188 | DSSDEETASTSKQKPK | | | | | Mascot |
| 1824.8995 | 1824.9142 | 0.0147 | 8 | 296 | 310 | IHTGERPYKCPHCVK | | | Carbamidomethyl (C)[10] | | Mascot |

| | | | | | | | | | | | |
|---|---|---|---|---|---|---|---|---|---|---|---|
| 1936.7137 | 1936.937 | 0.2233 | 115 | 157 | 172 | DDHCDDDDFIPSDENK | | | Carbamidomethyl (C)[4] | | Mascot |

31 PREDICTED: galactose-3-O-sulfotransferase 2-like, partial [Danio rerio] gi|326674388 68895.3 9.51 5 40 0 30 73.348

**Peptide Information**

| Calc. Mass | Obsrv. Mass | ± da | ± ppm | Start Seq. | End Seq. | Sequence | Ion Score | C. I. % | Modification | Rank | Result Type |
|---|---|---|---|---|---|---|---|---|---|---|---|
| 821.4151 | 821.4716 | 0.0565 | 69 | 262 | 268 | FSSPEVR | | | | | Mascot |
| 821.4151 | 821.4716 | 0.0565 | 69 | 262 | 268 | FSSPEVR | 30 | 73.348 | | | Mascot |
| 870.5195 | 870.5502 | 0.0307 | 35 | 191 | 197 | NHIVFLK | | | | | Mascot |
| 1121.5408 | 1121.6111 | 0.0703 | 63 | 19 | 28 | TASPWTTCVR | | | | | Mascot |
| 1638.7089 | 1638.8628 | 0.1539 | 94 | 121 | 135 | EEEGTDQNNLSSSTK | | | | | Mascot |
| 1971.0116 | 1970.8909 | -0.1207 | -61 | 181 | 197 | ADNSVTCQPKNHIVFLK | | | Carbamidomethyl (C)[7] | | Mascot |

32 PREDICTED: myosin-Ie [Danio rerio] gi|326669395 126893.9 9.25 15 40 0

**Peptide Information**

| Calc. Mass | Obsrv. Mass | ± da | ± ppm | Start Seq. | End Seq. | Sequence | Ion Score | C. I. % | Modification | Rank | Result Type |
|---|---|---|---|---|---|---|---|---|---|---|---|
| 856.4423 | 856.5385 | 0.0962 | 112 | 621 | 627 | AGYAYRR | | | | | Mascot |
| 874.4741 | 874.5159 | 0.0418 | 48 | 556 | 564 | GRPTTAGSK | | | | | Mascot |
| 1023.4965 | 1023.5204 | 0.0239 | 23 | 164 | 172 | NNNSSRFGK | | | | | Mascot |
| 1023.4965 | 1023.5204 | 0.0239 | 23 | 164 | 172 | NNNSSRFGK | | | | | Mascot |
| 1097.6062 | 1097.6796 | 0.0734 | 67 | 353 | 362 | DALSKALHSR | | | | | Mascot |
| 1097.6062 | 1097.6796 | 0.0734 | 67 | 353 | 362 | DALSKALHSR | 6 | 0 | | | Mascot |
| 1115.6531 | 1115.7063 | 0.0532 | 48 | 556 | 566 | GRPTTAGSKIK | 2 | 0 | | | Mascot |
| 1115.6531 | 1115.7063 | 0.0532 | 48 | 556 | 566 | GRPTTAGSKIK | | | | | Mascot |
| 1177.5518 | 1177.6561 | 0.1043 | 89 | 474 | 484 | GEGADQTMLQK | | | | | Mascot |
| 1238.7256 | 1238.7933 | 0.0677 | 55 | 632 | 641 | FLNRYAILTK | | | | | Mascot |
| 1259.6842 | 1259.5946 | -0.0896 | -71 | 720 | 730 | EEASDLLLNKK | | | | | Mascot |
| 1689.8806 | 1689.9437 | 0.0631 | 37 | 680 | 693 | APESLFLLEETRER | | | | | Mascot |
| 1711.8201 | 1711.912 | 0.0919 | 54 | 5 | 17 | GHYRYHWQSHNVK | | | | | Mascot |
| 1711.8201 | 1711.912 | 0.0919 | 54 | 5 | 17 | GHYRYHWQSHNVK | | | | | Mascot |
| 1824.916 | 1824.9142 | -0.0018 | -1 | 715 | 729 | YVQMREEASDLLLNK | | | Oxidation (M)[4] | | Mascot |
| 1859.9255 | 1859.9304 | 0.0049 | 3 | 579 | 593 | CTPHYIRCIKPNETK | | | Carbamidomethyl (C)[1] | | Mascot |
| 2142.0393 | 2142.2322 | 0.1929 | 90 | 454 | 473 | INPPGIMSILDDVCATMHAK | | | Oxidation (M)[7] | | Mascot |
| 2284.0366 | 2284.1936 | 0.157 | 69 | 98 | 118 | NMMIDRENQCVIISGESGAGK | | | Oxidation (M)[2,3] | | Mascot |
| 2938.3589 | 2938.5325 | 0.1736 | 59 | 487 | 510 | MQINNHEHFNSWNQGFIIHHYAGK | | | Oxidation (M)[1] | | Mascot |

| | | | | | | | | | | |
|---|---|---|---|---|---|---|---|---|---|---|
| 33 | striated muscle preferentially expressed protein kinase [Danio rerio] | gi\|55741920 | 333474.3 | 8.12 | 15 | 40 | 0 | 30 | 72.11 |

Peptide Information

| Calc. Mass | Obsrv. Mass | ± da | ± ppm | Start Seq. | End Seq. | Sequence | Ion Score | C. I. % | Modification | Rank | Result Type |
|---|---|---|---|---|---|---|---|---|---|---|---|
| 842.4189 | 842.5182 | 0.0993 | 118 | 887 | 892 | KMTQYR | | | Oxidation (M)[2] | | Mascot |
| 874.5032 | 874.5159 | 0.0127 | 15 | 863 | 870 | VTGLPYPK | | | | | Mascot |
| 1055.5303 | 1055.5625 | 0.0322 | 31 | 1709 | 1717 | AVSMELPHR | | | Oxidation (M)[4] | | Mascot |
| 1097.6313 | 1097.6796 | 0.0483 | 44 | 2194 | 2203 | INDSPVLALR | | | | | Mascot |
| 1097.6313 | 1097.6796 | 0.0483 | 44 | 2194 | 2203 | INDSPVLALR | 33 | 84.908 | | | Mascot |
| 1226.6527 | 1226.71 | 0.0573 | 47 | 1260 | 1270 | EIGRGAFSYVK | | | | | Mascot |
| 1238.6449 | 1238.7933 | 0.1484 | 120 | 2445 | 2454 | VPCTYTIEKK | | | Carbamidomethyl (C)[3] | | Mascot |
| 1259.7544 | 1259.5946 | -0.1598 | -127 | 950 | 959 | MILLSWKKPK | | | Oxidation (M)[1] | | Mascot |
| 1382.6984 | 1382.7999 | 0.1015 | 73 | 1729 | 1739 | EDYALKLDLMR | | | Oxidation (M)[10] | | Mascot |
| 1644.9279 | 1644.8524 | -0.0755 | -46 | 1341 | 1354 | RTTILESEVQSIIR | | | | | Mascot |
| 1647.8676 | 1647.9065 | 0.0389 | 24 | 2914 | 2926 | ILNSYPWCRPTIK | | | Carbamidomethyl (C)[8] | | Mascot |
| 1695.9138 | 1695.9147 | 0.0009 | 1 | 2707 | 2720 | MFIAKIIPYDQQTK | | | | | Mascot |
| 1699.9298 | 1699.9528 | 0.023 | 14 | 698 | 712 | EELICTANLLVIPSK | | | Carbamidomethyl (C)[5] | | Mascot |
| 1699.9298 | 1699.9528 | 0.023 | 14 | 698 | 712 | EELICTANLLVIPSK | | | Carbamidomethyl (C)[5] | | Mascot |
| 1705.9343 | 1706.0416 | 0.1073 | 63 | 173 | 189 | ALGSRAPGLQEPLSPGR | | | | | Mascot |
| 1711.9087 | 1711.912 | 0.0033 | 2 | 2707 | 2720 | MFIAKIIPYDQQTK | | | Oxidation (M)[1] | | Mascot |
| 1711.9087 | 1711.912 | 0.0033 | 2 | 2707 | 2720 | MFIAKIIPYDQQTK | | | Oxidation (M)[1] | | Mascot |
| 1859.9255 | 1859.9304 | 0.0049 | 3 | 2735 | 2749 | CERIMALHEAYITPR | | | Carbamidomethyl (C)[1] | | Mascot |
| 1906.8521 | 1906.8885 | 0.0364 | 19 | 1907 | 1922 | VEEMKEQDSVPENMNK | | | | | Mascot |

| | | | | | | | | | | |
|---|---|---|---|---|---|---|---|---|---|---|
| 34 | PREDICTED: zinc finger protein 845-like isoform 1 [Danio rerio] | gi\|326664549 | 110747.2 | 8.71 | 14 | 40 | 0 | | |

Peptide Information

| Calc. Mass | Obsrv. Mass | ± da | ± ppm | Start Seq. | End Seq. | Sequence | Ion Score | C. I. % | Modification | Rank | Result Type |
|---|---|---|---|---|---|---|---|---|---|---|---|
| 1055.5554 | 1055.5625 | 0.0071 | 7 | 384 | 392 | GFSTKMQLK | | | Oxidation (M)[6] | | Mascot |
| 1115.6418 | 1115.7063 | 0.0645 | 58 | 39 | 48 | EVASLEAKLR | 14 | 0 | | | Mascot |
| 1115.6418 | 1115.7063 | 0.0645 | 58 | 39 | 48 | EVASLEAKLR | | | | | Mascot |
| 1121.552 | 1121.6111 | 0.0591 | 53 | 905 | 913 | CFSRPEAVR | | | Carbamidomethyl (C)[1] | | Mascot |
| 1149.6163 | 1149.6906 | 0.0743 | 65 | 585 | 592 | YYLLRHER | | | | | Mascot |
| 1214.635 | 1214.6653 | 0.0303 | 25 | 267 | 276 | CFGTKYNLLR | | | | | Mascot |
| 1215.7069 | 1215.616 | -0.0909 | -75 | 821 | 830 | HEIVHRGIVR | | | | | Mascot |

| | | | | | | | | | | | |
|---|---|---|---|---|---|---|---|---|---|---|---|
| 1644.71 | 1644.8524 | 0.1424 | 87 | 2 | 15 | MLSMEVQVDVSCCK | | | Carbamidomethyl (C)[12], Oxidation (M)[1] | | Mascot |
| 1682.9185 | 1682.8958 | -0.0227 | -13 | 214 | 229 | QQPTGKSSSGKPQKPK | | | | | Mascot |
| 1711.8328 | 1711.912 | 0.0792 | 46 | 773 | 787 | QALQCSYCPKLLCNK | 7 | 0 | | | Mascot |
| 1711.8328 | 1711.912 | 0.0792 | 46 | 773 | 787 | QALQCSYCPKLLCNK | | | | | Mascot |
| 1737.7683 | 1737.9172 | 0.1489 | 86 | 593 | 607 | THTGEKPYQCSQCQK | | | | | Mascot |
| 2225.0227 | 2225.1382 | 0.1155 | 52 | 729 | 747 | THEIIHTGEKPYQCSHCN K | | | | | Mascot |
| 2284.0386 | 2284.1936 | 0.155 | 68 | 919 | 937 | HTGEKPYHCSLCSERFS FR | | | | | Mascot |
| 2326.0273 | 2326.2205 | 0.1932 | 83 | 313 | 332 | THTGARPYQCSHCQKSF CSK | | | Carbamidomethyl (C)[10] | | Mascot |
| 2938.2627 | 2938.5325 | 0.2698 | 92 | 285 | 308 | TDDVERPYQCSHCQKCF VESYLCK | | | Carbamidomethyl (C)[10] | | Mascot |

| | | | | | | | | | | |
|---|---|---|---|---|---|---|---|---|---|---|
| 35 | novel protein similar to H.sapiens TTN, titin (TTN) [Danio rerio] | gi\|169154588 | 2451841.5 | 6.24 | 66 | 40 | 0 | 9 | 0 | |

**Peptide Information**

| Calc. Mass | Obsrv. Mass | ± da | ± ppm | Start Seq. | End Seq. | Sequence | Ion Score | C. I. % | Modification | Rank | Result Type |
|---|---|---|---|---|---|---|---|---|---|---|---|
| 821.4185 | 821.4716 | 0.0531 | 65 | 504 | 510 | LTVEGMR | 3 | 0 | Oxidation (M)[6] | | Mascot |
| 821.4515 | 821.4716 | 0.0201 | 24 | 17148 | 17153 | QYILER | | | | | Mascot |
| 842.5094 | 842.5182 | 0.0088 | 10 | 4439 | 4446 | LSIPGVTR | | | | | Mascot |
| 856.4634 | 856.5385 | 0.0751 | 88 | 1798 | 1804 | EIREGPR | | | | | Mascot |
| 874.4992 | 874.5159 | 0.0167 | 19 | 12103 | 12110 | VSVVDVTR | | | | | Mascot |
| 888.4461 | 888.538 | 0.0919 | 103 | 3978 | 3985 | FSPPSEPK | | | | | Mascot |
| 888.5036 | 888.538 | 0.0344 | 39 | 20928 | 20934 | IIEEEKK | | | | | Mascot |
| 918.5077 | 918.5496 | 0.0419 | 46 | 11765 | 11772 | VTGLLEMR | | | | | Mascot |
| 926.4842 | 926.5511 | 0.0669 | 72 | 8026 | 8032 | EYGFRVR | | | | | Mascot |
| 1023.4775 | 1023.5204 | 0.0429 | 42 | 19145 | 19153 | AMIATSEER | 13 | 0 | Oxidation (M)[2] | | Mascot |
| 1023.4775 | 1023.5204 | 0.0429 | 42 | 19145 | 19153 | AMIATSEER | | | Oxidation (M)[2] | | Mascot |
| 1045.6404 | 1045.5737 | -0.0667 | -64 | 5457 | 5466 | GTKFVVPGLK | | | | | Mascot |
| 1055.5078 | 1055.5625 | 0.0547 | 52 | 1356 | 1364 | YEMISEGVK | | | | | Mascot |
| 1097.6313 | 1097.6796 | 0.0483 | 44 | 555 | 564 | TVLTHVDGKK | | | | | Mascot |
| 1097.679 | 1097.6796 | 0.0006 | 1 | 8507 | 8515 | RPVIDRTIK | | | | | Mascot |
| 1115.5513 | 1115.7063 | 0.155 | 139 | 3967 | 3977 | VCAENIAGPGK | | | Carbamidomethyl (C)[2] | | Mascot |
| 1115.6418 | 1115.7063 | 0.0645 | 58 | 10041 | 10051 | TVVAKAGDNLK | | | | | Mascot |
| 1121.6313 | 1121.6111 | -0.0202 | -18 | 5229 | 5237 | ITNYVVEKR | | | | | Mascot |
| 1137.5834 | 1137.6852 | 0.1018 | 89 | 18948 | 18956 | NPVTMTRYR | | | | | Mascot |
| 1149.6626 | 1149.6906 | 0.028 | 24 | 12441 | 12449 | ILIQDTRYK | | | | | Mascot |
| 1177.6913 | 1177.6561 | -0.0352 | -30 | 17168 | 17176 | RPITELRHR | | | | | Mascot |

| | | | | | | | | |
|---|---|---|---|---|---|---|---|---|
| 1214.5293 | 1214.6653 | 0.136 | 112 | 1267 | 1276 | YDMVADGCKR | Carbamidomethyl (C)[8] | Mascot |
| 1215.6514 | 1215.616 | -0.0354 | -29 | 8605 | 8615 | CNVPKNLQATK | | Mascot |
| 1226.635 | 1226.71 | 0.075 | 61 | 9280 | 9289 | LEHPGKTWMK | | Mascot |
| 1238.6449 | 1238.7933 | 0.1484 | 120 | 11047 | 11057 | ITGYIVEMQGK | | Mascot |
| 1259.6266 | 1259.5946 | -0.032 | -25 | 8847 | 8858 | ASEQPGPVAEFK | | Mascot |
| 1379.7417 | 1379.7687 | 0.027 | 20 | 16288 | 16299 | NTEYSTTLIIPK | | Mascot |
| 1382.6924 | 1382.7999 | 0.1075 | 78 | 4024 | 4034 | DWSRATERPHK | | Mascot |
| 1395.705 | 1395.7643 | 0.0593 | 42 | 14209 | 14220 | LAFTSITNNCVR | Carbamidomethyl (C)[10] | Mascot |
| 1463.8767 | 1463.91 | 0.0333 | 23 | 6495 | 6508 | LPALMRGLPVPTAK | | Mascot |
| 1463.8767 | 1463.91 | 0.0333 | 23 | 6495 | 6508 | LPALMRGLPVPTAK | | Mascot |
| 1477.8624 | 1477.9259 | 0.0635 | 43 | 21615 | 21628 | SPEPLVKSPVPSLK | | Mascot |
| 1555.775 | 1555.7659 | -0.0091 | -6 | 14614 | 14626 | VTGLEEGLQYQYR | | Mascot |
| 1622.8271 | 1622.8712 | 0.0441 | 27 | 20076 | 20090 | QTVELDVADVPDPPK | | Mascot |
| 1622.97 | 1622.8712 | -0.0988 | -61 | 192 | 206 | QIAGKVQVNVIGLQR | | Mascot |
| 1644.8115 | 1644.8524 | 0.0409 | 25 | 7755 | 7769 | SYLATDPKSEPDPPK | | Mascot |
| 1647.7869 | 1647.9065 | 0.1196 | 73 | 12046 | 12059 | VYSAVTTCNKMTWK | Oxidation (M)[11] | Mascot |
| 1648.6432 | 1648.8413 | 0.1981 | 120 | 1372 | 1385 | SVFDDEAEYECDAR | | Mascot |
| 1652.8239 | 1652.8809 | 0.057 | 34 | 6614 | 6629 | ETWGGVSSGSTSTRLK | | Mascot |
| 1666.8317 | 1666.894 | 0.0623 | 37 | 6244 | 6258 | AEIETSGTTTKLEMR | | Mascot |
| 1682.8418 | 1682.8958 | 0.054 | 32 | 11047 | 11061 | ITGYIVEMQGKNSDK | | Mascot |
| 1689.8441 | 1689.9437 | 0.0996 | 59 | 5104 | 5119 | EALEGAEGFDVNIVAR | | Mascot |
| 1695.8912 | 1695.9147 | 0.0235 | 14 | 11561 | 11575 | NTDFTTTLSVKEAIR | | Mascot |
| 1699.7731 | 1699.9528 | 0.1797 | 106 | 4559 | 4573 | YGISEACETEEVEIK | | Mascot |
| 1699.8108 | 1699.9528 | 0.142 | 84 | 4604 | 4618 | DSGGSMITGYWLEKR | | Mascot |
| 1711.8134 | 1711.912 | 0.0986 | 58 | 2692 | 2707 | DDTATYIIEAVNSSGR | | Mascot |
| 1711.8861 | 1711.912 | 0.0259 | 15 | 21352 | 21366 | RDEEAPTQIVPDITK | | Mascot |
| 1721.8817 | 1721.9209 | 0.0392 | 23 | 14822 | 14837 | DSGQYVLTLQNVAGTR | | Mascot |
| 1737.917 | 1737.9172 | 0.0002 | 0 | 8637 | 8652 | IGFGEPSEVPEKHLAK | | Mascot |
| 1796.8094 | 1796.9176 | 0.1082 | 60 | 8052 | 8067 | HPFDPPSEPLNCCVNK | | Mascot |
| 1812.9126 | 1812.9053 | -0.0073 | -4 | 10933 | 10949 | DSVSLIWDPPVIDGGSR | | Mascot |
| 1824.9602 | 1824.9142 | -0.046 | -25 | 18860 | 18875 | LVEGTEYVFRVAATNR | | Mascot |
| 1840.9301 | 1840.9137 | -0.0164 | -9 | 9946 | 9962 | DTATLAWTKPKHDGGSR | | Mascot |
| 1843.9371 | 1843.9298 | -0.0073 | -4 | 910 | 925 | VVHMSEDGKIHTLSFK | Oxidation (M)[4] | Mascot |
| 1859.9021 | 1859.9304 | 0.0283 | 15 | 18050 | 18065 | ELDPGEKYSLTYTSTR | | Mascot |
| 1886.9857 | 1886.811 | -0.1747 | -93 | 8631 | 8648 | VSAVNKIGFGEPSEVPEK | | Mascot |
| 1906.9504 | 1906.8885 | -0.0619 | -32 | 9513 | 9531 | VSAVNQYGTGDVTQSGP VK | | Mascot |
| 1936.9611 | 1936.937 | -0.0241 | -12 | 15129 | 15146 | IGPSEPSPETDPQVAKER | | Mascot |

| | | | | | | | | | | | | |
|---|---|---|---|---|---|---|---|---|---|---|---|---|
| 1954.9691 | 1954.8923 | -0.0768 | -39 | 690 | 706 | VVRPMYGVELFDGETAR | | | Oxidation (M)[5] | | Mascot |
| 1954.9691 | 1954.8923 | -0.0768 | -39 | 690 | 706 | VVRPMYGVELFDGETAR | | | Oxidation (M)[5] | | Mascot |
| 1970.877 | 1970.8909 | 0.0139 | 7 | 5938 | 5953 | DLWMPVTESCTRTSCK | | | Carbamidomethyl (C)[10,15] | | Mascot |
| 2083.0276 | 2082.9944 | -0.0332 | -16 | 2364 | 2382 | WTFGENALTAGDRVSMVTK | | | | | Mascot |
| 2141.9907 | 2142.2322 | 0.2415 | 113 | 4555 | 4573 | AENKYGISEACETEEVEIK | | | | | Mascot |
| 2211.0713 | 2211.125 | 0.0537 | 24 | 19288 | 19307 | TPLCPPEPPSFPPEIMDVTK | | | Oxidation (M)[16] | | Mascot |
| 2225.1382 | 2225.1382 | 0 | 0 | 18870 | 18890 | VAATNRFGIGGFLQSEVVECK | | | | | Mascot |
| 2283.1511 | 2283.198 | 0.0469 | 21 | 4790 | 4809 | IFMDISADELLCVRAGTPFK | | | Carbamidomethyl (C)[12] | | Mascot |
| 2284.1819 | 2284.1936 | 0.0117 | 5 | 10929 | 10949 | EITKDSVSLIWDPPVIDGGSR | | | | | Mascot |
| 2326.1421 | 2326.2205 | 0.0784 | 34 | 6172 | 6193 | AVNAAGESEPAYVPEPVRAQDR | | | | | Mascot |
| 2938.4402 | 2938.5325 | 0.0923 | 31 | 5908 | 5933 | NYMVISWNPPLDDGGAAISNYIVEKR | | | Oxidation (M)[3] | | Mascot |

| | | | | | | | | | | |
|---|---|---|---|---|---|---|---|---|---|---|
| 36 | novel protein similar to vertebrate transmembrane protein 16H (TMEM16H) [Danio rerio] | gi\|169158193 | 112029.6 | 5.99 | 10 | 40 | 0 | 26 | 27.601 |

**Peptide Information**

| Calc. Mass | Obsrv. Mass | ± da | ± ppm | Start Seq. | End Seq. | Sequence | Ion Score | C. I. % | Modification | Rank | Result Type |
|---|---|---|---|---|---|---|---|---|---|---|---|
| 856.49 | 856.5385 | 0.0485 | 57 | 12 | 18 | GRLFHAR | | | | | Mascot |
| 1115.6782 | 1115.7063 | 0.0281 | 25 | 2 | 11 | SSAVLEKLLR | 26 | 27.601 | | | Mascot |
| 1115.6782 | 1115.7063 | 0.0281 | 25 | 2 | 11 | SSAVLEKLLR | | | | | Mascot |
| 1121.4891 | 1121.6111 | 0.122 | 109 | 614 | 624 | AAGGENNTMEK | | | | | Mascot |
| 1137.7354 | 1137.6852 | -0.0502 | -44 | 878 | 887 | LKQIIPLQGK | | | | | Mascot |
| 1149.6626 | 1149.6906 | 0.028 | 24 | 520 | 529 | SLISSYRVPK | | | | | Mascot |
| 1463.8654 | 1463.91 | 0.0446 | 30 | 437 | 448 | LKEMLATLLIFR | | | Oxidation (M)[4] | | Mascot |
| 1463.8654 | 1463.91 | 0.0446 | 30 | 437 | 448 | LKEMLATLLIFR | | | Oxidation (M)[4] | | Mascot |
| 1555.7903 | 1555.7659 | -0.0244 | -16 | 456 | 467 | EVLQPYLYEHHK | | | | | Mascot |
| 1666.921 | 1666.894 | -0.027 | -16 | 174 | 186 | RVILQMFPLHEQR | | | | | Mascot |
| 1682.9159 | 1682.8958 | -0.0201 | -12 | 174 | 186 | RVILQMFPLHEQR | | | Oxidation (M)[6] | | Mascot |
| 1906.9514 | 1906.8885 | -0.0629 | -33 | 187 | 202 | ILNQLMTSWVQAVCER | | | Oxidation (M)[6] | | Mascot |
| 2283.1477 | 2283.198 | 0.0503 | 22 | 769 | 788 | YVIHVAIPDVPNWVADEMAK | | | Oxidation (M)[18] | | Mascot |

| | | | | | | | | | | |
|---|---|---|---|---|---|---|---|---|---|---|
| 37 | PREDICTED: hypothetical protein LOC562947 [Danio rerio] | gi\|292610701 | 116105.4 | 5.91 | 10 | 39 | 0 | 26 | 27.601 |

**Peptide Information**

| Calc. Mass | Obsrv. Mass | ± da | ± ppm | Start Seq. | End Seq. | Sequence | Ion Score | C. I. % | Modification | Rank | Result Type |
|---|---|---|---|---|---|---|---|---|---|---|---|

| | | | | | | | | | | | |
|---|---|---|---|---|---|---|---|---|---|---|---|
| 856.49 | 856.5385 | 0.0485 | 57 | 12 | 18 | GRLFHAR | | | | | Mascot |
| 1115.6782 | 1115.7063 | 0.0281 | 25 | 2 | 11 | SSAVLEKLLR | 26 | 27.601 | | | Mascot |
| 1115.6782 | 1115.7063 | 0.0281 | 25 | 2 | 11 | SSAVLEKLLR | | | | | Mascot |
| 1121.4891 | 1121.6111 | 0.122 | 109 | 614 | 624 | AAGGENNTMEK | | | | | Mascot |
| 1137.7354 | 1137.6852 | -0.0502 | -44 | 878 | 887 | LKQIIPLQGK | | | | | Mascot |
| 1149.6626 | 1149.6906 | 0.028 | 24 | 520 | 529 | SLISSYRVPK | | | | | Mascot |
| 1463.8654 | 1463.91 | 0.0446 | 30 | 437 | 448 | LKEMLATLLIFR | | | Oxidation (M)[4] | | Mascot |
| 1463.8654 | 1463.91 | 0.0446 | 30 | 437 | 448 | LKEMLATLLIFR | | | Oxidation (M)[4] | | Mascot |
| 1555.7903 | 1555.7659 | -0.0244 | -16 | 456 | 467 | EVLQPYLYEHHK | | | | | Mascot |
| 1666.921 | 1666.894 | -0.027 | -16 | 174 | 186 | RVILQMFPLHEQR | | | | | Mascot |
| 1682.9159 | 1682.8958 | -0.0201 | -12 | 174 | 186 | RVILQMFPLHEQR | | | Oxidation (M)[6] | | Mascot |
| 1906.9514 | 1906.8885 | -0.0629 | -33 | 187 | 202 | ILNQLMTSWVQAVCER | | | Oxidation (M)[6] | | Mascot |
| 2283.1477 | 2283.198 | 0.0503 | 22 | 769 | 788 | YVIHVAIPDVPNWVADEM AK | | | Oxidation (M)[18] | | Mascot |

| | | | | | | | | |
|---|---|---|---|---|---|---|---|---|
| 38 | novel protein similar to vertebrate cytoplasmic linker associated protein 1 (CLASP1) [Danio rerio] | gi\|159569348 | 164674.4 | 8.99 | 18 | 38 | 0 | |

**Peptide Information**

| Calc. Mass | Obsrv. Mass | ± da | ± ppm | Start Seq. | End Seq. | Sequence | Ion Score | C. I. % | Modification | Rank | Result Type |
|---|---|---|---|---|---|---|---|---|---|---|---|
| 856.5502 | 856.5385 | -0.0117 | -14 | 1277 | 1284 | GALIELLK | | | | | Mascot |
| 870.577 | 870.5502 | -0.0268 | -31 | 1323 | 1329 | VLKEILR | | | | | Mascot |
| 874.5257 | 874.5159 | -0.0098 | -11 | 1057 | 1063 | LLHNHLK | | | | | Mascot |
| 1177.5696 | 1177.6561 | 0.0865 | 73 | 596 | 607 | SDIDVNAASSAK | | | | | Mascot |
| 1226.7831 | 1226.71 | -0.0731 | -60 | 1277 | 1287 | GALIELLKITR | | | | | Mascot |
| 1238.7467 | 1238.7933 | 0.0466 | 38 | 480 | 490 | HVAVLTETIKK | | | | | Mascot |
| 1382.7175 | 1382.7999 | 0.0824 | 60 | 333 | 343 | QDWEHRVTALK | | | | | Mascot |
| 1622.8649 | 1622.8712 | 0.0063 | 4 | 216 | 228 | LNVIFSRFDEVQR | | | | | Mascot |
| 1622.8649 | 1622.8712 | 0.0063 | 4 | 216 | 228 | LNVIFSRFDEVQR | | | | | Mascot |
| 1648.8007 | 1648.8413 | 0.0406 | 25 | 727 | 741 | SSRIPRPSMSQGCSR | | | | | Mascot |
| 1695.8081 | 1695.9147 | 0.1066 | 63 | 2 | 15 | EPNMEYCLTQVLQK | | | | | Mascot |
| 1699.7599 | 1699.9528 | 0.1929 | 113 | 266 | 282 | GGSMGSMRRPSSASGS R | | | Oxidation (M)[4,7] | | Mascot |
| 1699.7599 | 1699.9528 | 0.1929 | 113 | 266 | 282 | GGSMGSMRRPSSASGS R | | | Oxidation (M)[4,7] | | Mascot |
| 1705.8715 | 1706.0416 | 0.1701 | 100 | 552 | 567 | SSSSSQESLNRPLSVK | | | | | Mascot |
| 1711.803 | 1711.912 | 0.109 | 64 | 2 | 15 | EPNMEYCLTQVLQK | | | Oxidation (M)[4] | | Mascot |
| 1711.803 | 1711.912 | 0.109 | 64 | 2 | 15 | EPNMEYCLTQVLQK | | | Oxidation (M)[4] | | Mascot |
| 1721.817 | 1721.9209 | 0.1039 | 60 | 727 | 741 | SSRIPRPSMSQGCSR | | | Carbamidomethyl (C)[13], Oxidation (M)[9] | | Mascot |

| | | | | | | | | | | | |
|---|---|---|---|---|---|---|---|---|---|---|---|
| 1737.8265 | 1737.9172 | 0.0907 | 52 | 118 | 131 | IMEQSATPQYIWDR | | | | | Mascot |
| 1843.7915 | 1843.9298 | 0.1383 | 75 | 1224 | 1238 | EAVFDDDVEQFRDCR | | | | | Mascot |
| 1887.0017 | 1886.811 | -0.1907 | -101 | 763 | 779 | FGLIHQARISASVNAMR | | | Oxidation (M)[16] | | Mascot |
| 2083.0125 | 2082.9944 | -0.0181 | -9 | 1360 | 1379 | AAEEAASTLAGSIHPEQC IK | | | Carbamidomethyl (C)[18] | | Mascot |
| 2225.207 | 2225.1382 | -0.0688 | -31 | 379 | 399 | SQVVREACITLGHLSSLL GNK | | | | | Mascot |
| 2284.0769 | 2284.1936 | 0.1167 | 51 | 1 | 19 | MEPNMEYCLTQVLQKDV AR | | | Oxidation (M)[1] | | Mascot |

| | | | | | | | | | |
|---|---|---|---|---|---|---|---|---|---|
| 39 | PREDICTED: eukaryotic translation initiation factor 2-alpha kinase 4, partial [Danio rerio] | gi\|326681092 | 170018.8 | 6.07 | 12 | 38 | 0 | 3 | 0 |

Peptide Information

| Calc. Mass | Obsrv. Mass | ± da | ± ppm | Start Seq. | End Seq. | Sequence | Ion Score | C. I. % | Modification | Rank | Result Type |
|---|---|---|---|---|---|---|---|---|---|---|---|
| 856.4886 | 856.5385 | 0.0499 | 58 | 537 | 544 | IQVNPASK | | | | | Mascot |
| 874.4992 | 874.5159 | 0.0167 | 19 | 551 | 558 | GEVTLLSR | | | | | Mascot |
| 1115.6782 | 1115.7063 | 0.0281 | 25 | 549 | 558 | IKGEVTLLSR | 16 | 0 | | | Mascot |
| 1115.6782 | 1115.7063 | 0.0281 | 25 | 549 | 558 | IKGEVTLLSR | | | | | Mascot |
| 1214.674 | 1214.6653 | -0.0087 | -7 | 17 | 26 | QEKLNVELNK | | | | | Mascot |
| 1259.6014 | 1259.5946 | -0.0068 | -5 | 370 | 379 | EDIFEQSHVR | | | | | Mascot |
| 1648.767 | 1648.8413 | 0.0743 | 45 | 1133 | 1147 | LTHASNILCDAMSEK | | | Oxidation (M)[12] | | Mascot |
| 1689.7935 | 1689.9437 | 0.1502 | 89 | 1133 | 1147 | LTHASNILCDAMSEK | | | Carbamidomethyl (C)[9] | | Mascot |
| 1699.8901 | 1699.9528 | 0.0627 | 37 | 413 | 427 | EYPLTVPDTLPADLR | | | | | Mascot |
| 1699.8901 | 1699.9528 | 0.0627 | 37 | 413 | 427 | EYPLTVPDTLPADLR | 3 | 0 | | | Mascot |
| 1705.7883 | 1706.0416 | 0.2533 | 148 | 1133 | 1147 | LTHASNILCDAMSEK | | | Carbamidomethyl (C)[9], Oxidation (M)[12] | | Mascot |
| 1711.8907 | 1711.912 | 0.0213 | 12 | 339 | 354 | HLSASCVLLDARGDVR | 3 | 0 | | | Mascot |
| 1711.8907 | 1711.912 | 0.0213 | 12 | 339 | 354 | HLSASCVLLDARGDVR | | | | | Mascot |
| 1721.8552 | 1721.9209 | 0.0657 | 38 | 136 | 152 | QTSVSSGSPVDSSEVKK | | | | | Mascot |
| 1825.0065 | 1824.9142 | -0.0923 | -51 | 1275 | 1294 | GPTVSGVVPSAVGASLA LDK | | | | | Mascot |
| 1843.9219 | 1843.9298 | 0.0079 | 4 | 88 | 102 | LEEQMQNEILAEIQR | | | | | Mascot |
| 1859.9167 | 1859.9304 | 0.0137 | 7 | 88 | 102 | LEEQMQNEILAEIQR | | | Oxidation (M)[5] | | Mascot |
| 2326.2415 | 2326.2205 | -0.021 | -9 | 319 | 338 | HTQQLLSALHYLHTNSVV HK | | | | | Mascot |

| | | | | | | | |
|---|---|---|---|---|---|---|---|
| 40 | Golgb1 protein [Danio rerio] | gi\|141796874 | 53722.5 | 5.1 | 11 | 38 | 0 |

Peptide Information

| Calc. Mass | Obsrv. Mass | ± da | ± ppm | Start Seq. | End Seq. | Sequence | Ion Score | C. I. % | Modification | Rank | Result Type |
|---|---|---|---|---|---|---|---|---|---|---|---|
| 874.4265 | 874.5159 | 0.0894 | 102 | 191 | 198 | DQAVNEAK | | | | | Mascot |

| | | | | | | | | | | | |
|---|---|---|---|---|---|---|---|---|---|---|---|
| 918.4713 | 918.5496 | 0.0783 | 85 | 287 | 293 | QELEMLR | | | | | Mascot |
| 926.4689 | 926.5511 | 0.0822 | 89 | 118 | 125 | ETQAVHNK | | | | | Mascot |
| 1023.5152 | 1023.5204 | 0.0052 | 5 | 439 | 446 | VAFCSRQR | | | Carbamidomethyl (C)[4] | | Mascot |
| 1023.5152 | 1023.5204 | 0.0052 | 5 | 439 | 446 | VAFCSRQR | | | Carbamidomethyl (C)[4] | | Mascot |
| 1214.6639 | 1214.6653 | 0.0014 | 1 | 129 | 138 | ELRWEGGVLR | | | | | Mascot |
| 1259.6589 | 1259.5946 | -0.0643 | -51 | 374 | 383 | LDEESELRLR | | | | | Mascot |
| 1463.7094 | 1463.91 | 0.2006 | 137 | 239 | 249 | ERMLEQMQVQR | | | Oxidation (M)[3] | | Mascot |
| 1463.7094 | 1463.91 | 0.2006 | 137 | 239 | 249 | ERMLEQMQVQR | | | Oxidation (M)[3] | | Mascot |
| 1555.8439 | 1555.7659 | -0.078 | -50 | 382 | 394 | LRIEEQLIAAEDR | | | | | Mascot |
| 1622.8608 | 1622.8712 | 0.0104 | 6 | 26 | 39 | DSQHKQILAAQVER | | | | | Mascot |
| 1622.8608 | 1622.8712 | 0.0104 | 6 | 26 | 39 | DSQHKQILAAQVER | | | | | Mascot |
| 1647.8081 | 1647.9065 | 0.0984 | 60 | 154 | 166 | DLLMMEQQLLEER | | | | | Mascot |
| 2083.0566 | 2082.9944 | -0.0622 | -30 | 48 | 65 | EDLVNHIQAFEKDIAQGR | | | | | Mascot |

| | | | | | | | |
|---|---|---|---|---|---|---|---|
| 41 | Rbm34 protein [Danio rerio] | gi\|159155220 | 45856.6 | 10.19 | 10 | 38 | 0 |

Peptide Information

| Calc. Mass | Obsrv. Mass | ± da | ± ppm | Start Seq. | End Seq. | Sequence | Ion Score | C. I. % | Modification | Rank | Result Type |
|---|---|---|---|---|---|---|---|---|---|---|---|
| 821.4767 | 821.4716 | -0.0051 | -6 | 163 | 169 | DLLSVFK | | | | | Mascot |
| 821.4767 | 821.4716 | -0.0051 | -6 | 163 | 169 | DLLSVFK | | | | | Mascot |
| 842.473 | 842.5182 | 0.0452 | 54 | 74 | 80 | DPVQKQK | | | | | Mascot |
| 1055.5592 | 1055.5625 | 0.0033 | 3 | 238 | 246 | VDRVSQHSK | | | | | Mascot |
| 1121.5447 | 1121.6111 | 0.0664 | 59 | 355 | 363 | QEFRNNTGR | | | | | Mascot |
| 1149.6375 | 1149.6906 | 0.0531 | 46 | 171 | 180 | SGVIESVRFR | | | | | Mascot |
| 1379.7351 | 1379.7687 | 0.0336 | 24 | 149 | 161 | TVFVGNLPSSCKK | | | | | Mascot |
| 1647.7456 | 1647.9065 | 0.1609 | 98 | 97 | 112 | ESALQNADEEGQASAK | | | | | Mascot |
| 1666.9374 | 1666.894 | -0.0434 | -26 | 56 | 71 | IEPKVTPAVSDNAVVK | | | | | Mascot |
| 1711.7882 | 1711.912 | 0.1238 | 72 | 119 | 133 | ADGGEEEEERPTKHK | | | | | Mascot |
| 1711.7882 | 1711.912 | 0.1238 | 72 | 119 | 133 | ADGGEEEEERPTKHK | | | | | Mascot |
| 2283.1501 | 2283.198 | 0.0479 | 21 | 205 | 224 | QNINAYIVFKEEESATDALK | | | | | Mascot |

| | | | | | | | |
|---|---|---|---|---|---|---|---|
| 42 | PREDICTED: G protein-activated inward rectifier potassium channel 4-like [Danio rerio] | gi\|292623912 | 48753.5 | 5.51 | 9 | 37 | 0 |

Peptide Information

| Calc. Mass | Obsrv. Mass | ± da | ± ppm | Start Seq. | End Seq. | Sequence | Ion Score | C. I. % | Modification | Rank | Result Type |
|---|---|---|---|---|---|---|---|---|---|---|---|
| 821.4628 | 821.4716 | 0.0088 | 11 | 52 | 57 | QRYVQK | | | | | Mascot |

| Calc. Mass | Obsrv. Mass | ± da | ± ppm | Start Seq. | End Seq. | Sequence | Ion Score | C. I. % | Modification | Rank | Result Type |
|---|---|---|---|---|---|---|---|---|---|---|---|
| 821.4628 | 821.4716 | 0.0088 | 11 | 52 | 57 | QRYVQK | | | | | Mascot |
| 842.4842 | 842.5182 | 0.034 | 40 | 191 | 197 | ISQPKNR | | | | | Mascot |
| 1055.4615 | 1055.5625 | 0.101 | 96 | 285 | 292 | SPFWEMSR | | | Oxidation (M)[6] | | Mascot |
| 1214.5479 | 1214.6653 | 0.1174 | 97 | 213 | 221 | DNKMCLMFR | | | Carbamidomethyl (C)[5] | | Mascot |
| 1555.8367 | 1555.7659 | -0.0708 | -46 | 74 | 86 | YLSDLFTTLVDLR | | | | | Mascot |
| 1652.752 | 1652.8809 | 0.1289 | 78 | 7 | 20 | VHMDHDMEIGVTPR | | | Oxidation (M)[3] | | Mascot |
| 1689.7715 | 1689.9437 | 0.1722 | 102 | 390 | 404 | AHTTDPFQTTEDQAK | | | | | Mascot |
| 1695.7317 | 1695.9147 | 0.183 | 108 | 405 | 420 | HDADTPPETNNGSANR | | | | | Mascot |
| 1797.0269 | 1796.9176 | -0.1093 | -61 | 374 | 389 | ESPLLPHLPLLSPDLR | | | | | Mascot |

| | | | | | | | | | | |
|---|---|---|---|---|---|---|---|---|---|---|
| 43 | PREDICTED: protein argonaute-3 [Danio rerio] | gi\|189530413 | 101302 | 9.23 | 6 | 37 | 0 | 28 | 53.577 |

**Peptide Information**

| Calc. Mass | Obsrv. Mass | ± da | ± ppm | Start Seq. | End Seq. | Sequence | Ion Score | C. I. % | Modification | Rank | Result Type |
|---|---|---|---|---|---|---|---|---|---|---|---|
| 1115.5448 | 1115.7063 | 0.1615 | 145 | 1 | 9 | MNLLVCHGR | 28 | 53.577 | Carbamidomethyl (C)[6], Oxidation (M)[1] | | Mascot |
| 1115.5448 | 1115.7063 | 0.1615 | 145 | 1 | 9 | MNLLVCHGR | | | Carbamidomethyl (C)[6], Oxidation (M)[1] | | Mascot |
| 1379.72 | 1379.7687 | 0.0487 | 35 | 377 | 388 | KLTDNQTSTMIK | | | | | Mascot |
| 1395.7148 | 1395.7643 | 0.0495 | 35 | 377 | 388 | KLTDNQTSTMIK | | | Oxidation (M)[10] | | Mascot |
| 1622.8649 | 1622.8712 | 0.0063 | 4 | 97 | 110 | VTIFGDRRPVYDGK | | | | | Mascot |
| 1622.8649 | 1622.8712 | 0.0063 | 4 | 97 | 110 | VTIFGDRRPVYDGK | | | | | Mascot |
| 1666.8429 | 1666.894 | 0.0511 | 31 | 378 | 392 | LTDNQTSTMIKATAR | | | Oxidation (M)[9] | | Mascot |
| 1812.9425 | 1812.9053 | -0.0372 | -21 | 358 | 373 | HTYLPLEVCNIVAGQR | | | | | Mascot |
| 1936.9077 | 1936.937 | 0.0293 | 15 | 513 | 529 | ISKDAGMPIQGQPCFCK | | | Carbamidomethyl (C)[14,16] | | Mascot |

| | | | | | | | | |
|---|---|---|---|---|---|---|---|---|
| 44 | novel protein [Danio rerio] | gi\|55962229 | 275569.2 | 5.29 | 17 | 37 | 0 |

**Peptide Information**

| Calc. Mass | Obsrv. Mass | ± da | ± ppm | Start Seq. | End Seq. | Sequence | Ion Score | C. I. % | Modification | Rank | Result Type |
|---|---|---|---|---|---|---|---|---|---|---|---|
| 821.4515 | 821.4716 | 0.0201 | 24 | 287 | 293 | FDTVIAR | | | | | Mascot |
| 821.4515 | 821.4716 | 0.0201 | 24 | 287 | 293 | FDTVIAR | 5 | 0 | | | Mascot |
| 882.5195 | 882.5753 | 0.0558 | 63 | 1300 | 1306 | KAFFTLR | | | | | Mascot |
| 888.4785 | 888.538 | 0.0595 | 67 | 1699 | 1705 | ELQQKDK | | | | | Mascot |
| 888.4785 | 888.538 | 0.0595 | 67 | 1699 | 1705 | ELQQKDK | | | | | Mascot |
| 918.4938 | 918.5496 | 0.0558 | 61 | 2120 | 2126 | RMQAQIR | | | Oxidation (M)[2] | | Mascot |
| 1115.6055 | 1115.7063 | 0.1008 | 90 | 2384 | 2392 | LSEQEQLLR | 18 | 0 | | | Mascot |
| 1115.6055 | 1115.7063 | 0.1008 | 90 | 2384 | 2392 | LSEQEQLLR | | | | | Mascot |
| 1238.6892 | 1238.7933 | 0.1041 | 84 | 24 | 32 | LRIYFLEER | | | | | Mascot |

| Calc. Mass | Obsrv. Mass | ± da | ± ppm | Start Seq. | End Seq. | Sequence | Ion Score | C. I. % | Modification | Rank | Result Type |
|---|---|---|---|---|---|---|---|---|---|---|---|
| 1379.6848 | 1379.7687 | 0.0839 | 61 | 230 | 240 | ICARELYGNQR | | | Carbamidomethyl (C)[2] | | Mascot |
| 1477.738 | 1477.9259 | 0.1879 | 127 | 1389 | 1401 | ELLTETSVQQDSK | | | | | Mascot |
| 1638.7792 | 1638.8628 | 0.0836 | 51 | 404 | 417 | VTDSDYEAVCKVPR | | | Carbamidomethyl (C)[10] | | Mascot |
| 1647.7656 | 1647.9065 | 0.1409 | 86 | 522 | 535 | SCEYHPLQSQRGSR | | | | | Mascot |
| 1652.8602 | 1652.8809 | 0.0207 | 13 | 96 | 109 | QQETGHIQEILQTK | | | | | Mascot |
| 1666.8871 | 1666.894 | 0.0069 | 4 | 785 | 799 | HNQDLLTALESARAK | | | | | Mascot |
| 1711.8254 | 1711.912 | 0.0866 | 51 | 1600 | 1614 | GMCHILTQHLGDTTK | | | Carbamidomethyl (C)[3] | | Mascot |
| 1711.8254 | 1711.912 | 0.0866 | 51 | 1600 | 1614 | GMCHILTQHLGDTTK | | | Carbamidomethyl (C)[3] | | Mascot |
| 1936.9697 | 1936.937 | -0.0327 | -17 | 1 | 16 | MFNGYRTISQHLNDLK | | | | | Mascot |
| 1970.9712 | 1970.8909 | -0.0803 | -41 | 1234 | 1251 | TAQSLVSLQSCDQHQGLR | | | | | Mascot |
| 2326.1707 | 2326.2205 | 0.0498 | 21 | 1252 | 1273 | GCQDAVAEGPGLAEVKQLVEQK | | | Carbamidomethyl (C)[2] | | Mascot |
| 2938.3635 | 2938.5325 | 0.169 | 58 | 1622 | 1646 | SNDIDYYMGQSFRDQLSQSISLAQR | | | Oxidation (M)[8] | | Mascot |

45 PREDICTED: ninein [Danio rerio] gi|326673087 160970.7 4.92 15 37 0

**Peptide Information**

| Calc. Mass | Obsrv. Mass | ± da | ± ppm | Start Seq. | End Seq. | Sequence | Ion Score | C. I. % | Modification | Rank | Result Type |
|---|---|---|---|---|---|---|---|---|---|---|---|
| 821.3709 | 821.4716 | 0.1007 | 123 | 612 | 618 | AMVDEEK | | | | | Mascot |
| 821.3709 | 821.4716 | 0.1007 | 123 | 612 | 618 | AMVDEEK | 12 | 0 | | | Mascot |
| 888.5148 | 888.538 | 0.0232 | 26 | 1077 | 1083 | TEKELLR | | | | | Mascot |
| 888.5148 | 888.538 | 0.0232 | 26 | 1077 | 1083 | TEKELLR | | | | | Mascot |
| 1214.7103 | 1214.6653 | -0.045 | -37 | 1286 | 1295 | EVEITLRNLK | | | | | Mascot |
| 1226.6198 | 1226.71 | 0.0902 | 74 | 366 | 375 | HLLECVDLER | | | | | Mascot |
| 1259.6128 | 1259.5946 | -0.0182 | -14 | 840 | 848 | LRQQWDEER | | | | | Mascot |
| 1382.7208 | 1382.7999 | 0.0791 | 57 | 366 | 376 | HLLECVDLERR | | | | | Mascot |
| 1463.6907 | 1463.91 | 0.2193 | 150 | 964 | 975 | TDIAEQRSEVCR | | | Carbamidomethyl (C)[11] | | Mascot |
| 1463.6907 | 1463.91 | 0.2193 | 150 | 964 | 975 | TDIAEQRSEVCR | | | Carbamidomethyl (C)[11] | | Mascot |
| 1555.6805 | 1555.7659 | 0.0854 | 55 | 534 | 545 | SYEEQCRELQDR | | | | | Mascot |
| 1666.8065 | 1666.894 | 0.0875 | 52 | 1182 | 1195 | RQMNELSSELQTSK | | | Oxidation (M)[3] | | Mascot |
| 1682.8279 | 1682.8958 | 0.0679 | 40 | 895 | 908 | QHKEALQACLEQER | | | | | Mascot |
| 1695.8846 | 1695.9147 | 0.0301 | 18 | 362 | 375 | AEIRHLLECVDLER | | | | | Mascot |
| 1859.947 | 1859.9304 | -0.0166 | -9 | 1048 | 1064 | HALQEVIRQHDGSANGK | | | | | Mascot |
| 1936.9585 | 1936.937 | -0.0215 | -11 | 1158 | 1174 | VWAELSGLMEAEHHSLK | | | | | Mascot |
| 2142.0461 | 2142.2322 | 0.1861 | 87 | 289 | 308 | VFSDLDDGSGHAPVETLLNR | | | | | Mascot |
| 2284.1616 | 2284.1936 | 0.032 | 14 | 849 | 867 | ASMENKHHALLLQHLQEER | | | | | Mascot |

46 Synembryn-like [Danio rerio] gi|94574123 59482.8 5.37 8 36 0

Peptide Information

| Calc. Mass | Obsrv. Mass | ± da | ± ppm | Start Seq. | End Seq. | Sequence | Ion Score | C. I. % | Modification | Rank | Result Type |
|---|---|---|---|---|---|---|---|---|---|---|---|
| 842.5709 | 842.5182 | -0.0527 | -63 | 18 | 24 | VEKLLLK | | | | | Mascot |
| 1045.6769 | 1045.5737 | -0.1032 | -99 | 171 | 179 | LLFLLSALR | | | | | Mascot |
| 1115.6605 | 1115.7063 | 0.0458 | 41 | 46 | 55 | LCQGLLTVLR | 13 | 0 | | | Mascot |
| 1115.6605 | 1115.7063 | 0.0458 | 41 | 46 | 55 | LCQGLLTVLR | | | | | Mascot |
| 1214.7037 | 1214.6653 | -0.0384 | -32 | 503 | 512 | ELIRPMGVRK | | | Oxidation (M)[6] | | Mascot |
| 1382.7136 | 1382.7999 | 0.0863 | 62 | 399 | 410 | QGAAEFLFVLCK | | | Carbamidomethyl (C)[11] | | Mascot |
| 1695.8809 | 1695.9147 | 0.0338 | 20 | 315 | 328 | NMDTVQILLNFMIK | | | Oxidation (M)[2] | | Mascot |
| 1711.8757 | 1711.912 | 0.0363 | 21 | 315 | 328 | NMDTVQILLNFMIK | | | Oxidation (M)[2,12] | | Mascot |
| 1711.8757 | 1711.912 | 0.0363 | 21 | 315 | 328 | NMDTVQILLNFMIK | | | Oxidation (M)[2,12] | | Mascot |
| 1954.9539 | 1954.8923 | -0.0616 | -32 | 486 | 501 | EYEAQRLVSMIDELSR | | | Oxidation (M)[10] | | Mascot |
| 1954.9539 | 1954.8923 | -0.0616 | -32 | 486 | 501 | EYEAQRLVSMIDELSR | | | Oxidation (M)[10] | | Mascot |
| 2283.0193 | 2283.198 | 0.1787 | 78 | 98 | 118 | ICVAGHEGEPLEEAQSAE EER | | | | | Mascot |

| | | | | | | | | |
|---|---|---|---|---|---|---|---|---|
| 47 | Zgc:172007 protein [Danio rerio] | gi\|161611828 | 57368.6 | 8.44 | 12 | 36 | 0 | |

Peptide Information

| Calc. Mass | Obsrv. Mass | ± da | ± ppm | Start Seq. | End Seq. | Sequence | Ion Score | C. I. % | Modification | Rank | Result Type |
|---|---|---|---|---|---|---|---|---|---|---|---|
| 856.4497 | 856.5385 | 0.0888 | 104 | 215 | 220 | VFFCKR | | | Carbamidomethyl (C)[4] | | Mascot |
| 1023.5179 | 1023.5204 | 0.0025 | 2 | 221 | 229 | CDISFPTLK | | | | | Mascot |
| 1023.5179 | 1023.5204 | 0.0025 | 2 | 221 | 229 | CDISFPTLK | | | | | Mascot |
| 1055.5012 | 1055.5625 | 0.0613 | 58 | 186 | 194 | TTFPCKQCK | | | | | Mascot |
| 1115.6418 | 1115.7063 | 0.0645 | 58 | 35 | 44 | EVASLEAKLR | 14 | 0 | | | Mascot |
| 1115.6418 | 1115.7063 | 0.0645 | 58 | 35 | 44 | EVASLEAKLR | | | | | Mascot |
| 1121.4536 | 1121.6111 | 0.1575 | 140 | 354 | 362 | MHQCLQCDK | | | Oxidation (M)[1] | | Mascot |
| 1177.6398 | 1177.6561 | 0.0163 | 14 | 182 | 191 | QKPKTTFPCK | | | | | Mascot |
| 1215.5687 | 1215.616 | 0.0473 | 39 | 311 | 320 | DGSHFKTHMR | | | | | Mascot |
| 1379.5718 | 1379.7687 | 0.1969 | 143 | 269 | 279 | EKPFHCSECDK | | | Carbamidomethyl (C)[6] | | Mascot |
| 1699.7714 | 1699.9528 | 0.1814 | 107 | 349 | 362 | IHSEKMHQCLQCDK | | | | | Mascot |
| 1699.7714 | 1699.9528 | 0.1814 | 107 | 349 | 362 | IHSEKMHQCLQCDK | | | | | Mascot |
| 1737.8137 | 1737.9172 | 0.1035 | 60 | 170 | 185 | DSSDEETASTSKQKPK | | | | | Mascot |
| 1824.8995 | 1824.9142 | 0.0147 | 8 | 293 | 307 | IHTGERPYKCPHCVK | | | Carbamidomethyl (C)[10] | | Mascot |
| 1936.7137 | 1936.937 | 0.2233 | 115 | 154 | 169 | DDHCDDDDFIPSDENK | | | Carbamidomethyl (C)[4] | | Mascot |

| | | | | | | | | | |
|---|---|---|---|---|---|---|---|---|---|
| 48 | hypothetical protein LOC557841 [Danio rerio] | gi\|165972361 | 51603.4 | 6.11 | 7 | 36 | 0 | 28 | 54.634 |

Peptide Information

| Calc. Mass | Obsrv. Mass | ± da | ± ppm | Start Seq. | End Seq. | Sequence | Ion Score | C. I. % | Modification | Rank | Result Type |
|---|---|---|---|---|---|---|---|---|---|---|---|
| 870.4427 | 870.5502 | 0.1075 | 124 | 114 | 120 | DSEKHVR | | | | | Mascot |
| 1023.4927 | 1023.5204 | 0.0277 | 27 | 67 | 74 | NLCELFER | | | | | Mascot |
| 1023.4927 | 1023.5204 | 0.0277 | 27 | 67 | 74 | NLCELFER | | | | | Mascot |
| 1097.5837 | 1097.6796 | 0.0959 | 87 | 57 | 66 | DDPPSNLVLK | | | | | Mascot |
| 1097.5837 | 1097.6796 | 0.0959 | 87 | 57 | 66 | DDPPSNLVLK | 28 | 54.634 | | | Mascot |
| 1395.6838 | 1395.7643 | 0.0805 | 58 | 33 | 42 | ECIQQFWRTK | | | Carbamidomethyl (C)[2] | | Mascot |
| 1555.6953 | 1555.7659 | 0.0706 | 45 | 8 | 19 | EISCPICCEIFK | | | Carbamidomethyl (C)[4,7,8] | | Mascot |
| 1695.7742 | 1695.9147 | 0.1405 | 83 | 185 | 198 | DEEEIAMTSLREEK | | | Oxidation (M)[7] | | Mascot |
| 1737.8485 | 1737.9172 | 0.0687 | 40 | 99 | 113 | LFCLEDKQPACLVCR | | | | | Mascot |

| Rank | Protein Name | Accession No. | Protein MW | Protein PI | Pep. Count | Protein Score | Protein Score C. I. % | Total Ion Score | Total Ion C. I. % |
|---|---|---|---|---|---|---|---|---|---|
| 49 | SPEG complex locus [Danio rerio] | gi\|123295462 | 333650.8 | 8.15 | 1 | | | 30 | 72.11 |

Peptide Information

| Calc. Mass | Obsrv. Mass | ± da | ± ppm | Start Seq. | End Seq. | Sequence | Ion Score | C. I. % | Modification | Rank | Result Type |
|---|---|---|---|---|---|---|---|---|---|---|---|
| 1097.6313 | 1097.6796 | 0.0483 | 44 | 2193 | 2202 | INDSPVLALR | 33 | 84.908 | | 2 | Mascot |

| Rank | Protein Name | Accession No. | Protein MW | Protein PI | Pep. Count | Protein Score | Protein Score C. I. % | Total Ion Score | Total Ion C. I. % |
|---|---|---|---|---|---|---|---|---|---|
| 50 | PREDICTED: hypothetical protein LOC568908 [Danio rerio] | gi\|326666001 | 246789.2 | 9.26 | 1 | | | 30 | 70.844 |

Peptide Information

| Calc. Mass | Obsrv. Mass | ± da | ± ppm | Start Seq. | End Seq. | Sequence | Ion Score | C. I. % | Modification | Rank | Result Type |
|---|---|---|---|---|---|---|---|---|---|---|---|
| 1097.679 | 1097.6796 | 0.0006 | 1 | 720 | 729 | RLSTPAIALR | 30 | 70.844 | | 4 | Mascot |

| | | | | | |
|---|---|---|---|---|---|
| **Gel Idx/Pos** | 194/H21 | **Instr./Gel Origin** | BA2060/110707A | **Process Status** | Analysis Succeeded |
| **Plate [#] Name** | [1] 31496 | **Instrument Sample Name** | | **Spectra** | 11 |

| Rank | Protein Name | Accession No. | Protein MW | Protein PI | Pep. Count | Protein Score | Protein Score C. I. % | Total Ion Score | Total Ion C. I. % |
|---|---|---|---|---|---|---|---|---|---|
| 1 | Krt4 protein [Danio rerio] | gi\|161612220 | 53979.9 | 5.34 | 22 | 897 | 100 | 745 | 100 |

Peptide Information

| Calc. Mass | Obsrv. Mass | ± da | ± ppm | Start Seq. | End Seq. | Sequence | Ion Score | C. I. % | Modification | Rank | Result Type |
|---|---|---|---|---|---|---|---|---|---|---|---|
| 827.4297 | 827.4365 | 0.0068 | 8 | 125 | 131 | FASFIDK | | | | | Mascot |
| 915.4893 | 915.4971 | 0.0078 | 9 | 328 | 335 | AEIAELNR | | | | | Mascot |

| | | | | | | | | | | | |
|---|---|---|---|---|---|---|---|---|---|---|---|
| 950.4941 | 950.501 | 0.0069 | 7 | 249 | 256 | AVYEAELR | | | | | Mascot |
| 983.4468 | 983.4488 | 0.002 | 2 | 299 | 306 | AEAESWYK | | | | | Mascot |
| 987.5105 | 987.5178 | 0.0073 | 7 | 378 | 385 | ELEEALQR | | | | | Mascot |
| 1079.5116 | 1079.5217 | 0.0101 | 9 | 288 | 296 | AQYEDIANR | | | | | Mascot |
| 1226.5801 | 1226.5912 | 0.0111 | 9 | 297 | 306 | SRAEAESWYK | | | | | Mascot |
| 1248.6833 | 1248.6927 | 0.0094 | 8 | 210 | 220 | ASVENEFVLLK | | | | | Mascot |
| 1256.6957 | 1256.7084 | 0.0127 | 10 | 376 | 385 | IRELEEALQR | 47 | 99.402 | | | Mascot |
| 1256.6957 | 1256.7084 | 0.0127 | 10 | 376 | 385 | IRELEEALQR | | | | | Mascot |
| 1277.71 | 1277.7234 | 0.0134 | 10 | 405 | 415 | LALDIEIATYR | 73 | 99.999 | | | Mascot |
| 1277.71 | 1277.7234 | 0.0134 | 10 | 405 | 415 | LALDIEIATYR | | | | | Mascot |
| 1361.673 | 1361.6848 | 0.0118 | 9 | 276 | 287 | NLDMDSIVAEVR | | | | | Mascot |
| 1362.7013 | 1362.7101 | 0.0088 | 6 | 146 | 156 | WSLLQEQTTTR | | | | | Mascot |
| 1362.7013 | 1362.7101 | 0.0088 | 6 | 146 | 156 | WSLLQEQTTTR | 71 | 99.998 | | | Mascot |
| 1377.6678 | 1377.684 | 0.0162 | 12 | 276 | 287 | NLDMDSIVAEVR | | | Oxidation (M)[4] | | Mascot |
| 1432.743 | 1432.7574 | 0.0144 | 10 | 237 | 248 | VDALQDEINFLR | | | | | Mascot |
| 1432.743 | 1432.7574 | 0.0144 | 10 | 237 | 248 | VDALQDEINFLR | 118 | 100 | | | Mascot |
| 1443.7074 | 1443.7195 | 0.0121 | 8 | 352 | 364 | ANLEAQIAEAEER | | | | | Mascot |
| 1443.7074 | 1443.7195 | 0.0121 | 8 | 352 | 364 | ANLEAQIAEAEER | 93 | 100 | | | Mascot |
| 1695.8258 | 1695.8291 | 0.0033 | 2 | 222 | 236 | DVDAAYMNKVELEAK | | | | | Mascot |
| 1743.837 | 1743.854 | 0.017 | 10 | 157 | 171 | SNIDAMFEAYISNLR | 130 | 100 | | | Mascot |
| 1743.866 | 1743.854 | -0.012 | -7 | 16 | 32 | KGYTSQSAYAVPAGSTR | | | | | Mascot |
| 1759.8319 | 1759.8502 | 0.0183 | 10 | 157 | 171 | SNIDAMFEAYISNLR | | | Oxidation (M)[6] | | Mascot |
| 1759.8319 | 1759.8502 | 0.0183 | 10 | 157 | 171 | SNIDAMFEAYISNLR | 68 | 99.996 | Oxidation (M)[6] | | Mascot |
| 1846.7913 | 1846.8077 | 0.0164 | 9 | 309 | 324 | FEEMQSTAGQYGDDLR | | | | | Mascot |
| 1862.7861 | 1862.8049 | 0.0188 | 10 | 309 | 324 | FEEMQSTAGQYGDDLR | | | Oxidation (M)[4] | | Mascot |
| 1899.9381 | 1899.957 | 0.0189 | 10 | 157 | 172 | SNIDAMFEAYISNLRR | | | | | Mascot |
| 2041.0559 | 2041.0757 | 0.0198 | 10 | 352 | 370 | ANLEAQIAEAEERGELAVK | 131 | 100 | | | Mascot |
| 2041.0559 | 2041.0757 | 0.0198 | 10 | 352 | 370 | ANLEAQIAEAEERGELAVK | | | | | Mascot |
| 2102.1128 | 2101.9373 | -0.1755 | -83 | 231 | 248 | VELEAKVDALQDEINFLR | | | | | Mascot |
| 2102.9448 | 2102.957 | 0.0122 | 6 | 307 | 324 | QKFEEMQSTAGQYGDDLR | | | | | Mascot |
| 2118.9397 | 2118.9619 | 0.0222 | 10 | 307 | 324 | QKFEEMQSTAGQYGDDLR | | | Oxidation (M)[6] | | Mascot |
| 2118.9397 | 2118.9619 | 0.0222 | 10 | 307 | 324 | QKFEEMQSTAGQYGDDLR | 84 | 100 | Oxidation (M)[6] | | Mascot |

| | | | | | | | | | | |
|---|---|---|---|---|---|---|---|---|---|---|
| 2 | keratin 4 [Danio rerio] | gi\|18858947 | 54242 | 5.42 | 20 | 874 | 100 | 747 | 100 | |

**Peptide Information**

| Calc. Mass | Obsrv. Mass | ± da | ± ppm | Start | End | Sequence | Ion | C. I. % | Modification | Rank | Result Type |
|---|---|---|---|---|---|---|---|---|---|---|---|

| | | | | Seq. | Seq. | | Score | | | |
|---|---|---|---|---|---|---|---|---|---|---|
| 915.4893 | 915.4971 | 0.0078 | 9 | 329 | 336 | AEIAELNR | | | | Mascot |
| 950.4941 | 950.501 | 0.0069 | 7 | 250 | 257 | AVYEAELR | | | | Mascot |
| 983.4468 | 983.4488 | 0.002 | 2 | 300 | 307 | AEAESWYK | | | | Mascot |
| 987.5105 | 987.5178 | 0.0073 | 7 | 379 | 386 | ELEEALQR | | | | Mascot |
| 1079.5116 | 1079.5217 | 0.0101 | 9 | 289 | 297 | AQYEDIANR | | | | Mascot |
| 1226.5801 | 1226.5912 | 0.0111 | 9 | 298 | 307 | SRAEAESWYK | | | | Mascot |
| 1248.6833 | 1248.6927 | 0.0094 | 8 | 211 | 221 | ASVENEFVLLK | | | | Mascot |
| 1256.6957 | 1256.7084 | 0.0127 | 10 | 377 | 386 | IRELEEALQR | 47 | 99.402 | | Mascot |
| 1256.6957 | 1256.7084 | 0.0127 | 10 | 377 | 386 | IRELEEALQR | | | | Mascot |
| 1277.71 | 1277.7234 | 0.0134 | 10 | 406 | 416 | LALDIEIATYR | 73 | 99.999 | | Mascot |
| 1277.71 | 1277.7234 | 0.0134 | 10 | 406 | 416 | LALDIEIATYR | | | | Mascot |
| 1361.673 | 1361.6848 | 0.0118 | 9 | 277 | 288 | NLDMDSIVAEVR | | | | Mascot |
| 1362.7013 | 1362.7101 | 0.0088 | 6 | 147 | 157 | WSLLQEQTTTR | | | | Mascot |
| 1362.7013 | 1362.7101 | 0.0088 | 6 | 147 | 157 | WSLLQEQTTTR | 71 | 99.998 | | Mascot |
| 1377.6678 | 1377.684 | 0.0162 | 12 | 277 | 288 | NLDMDSIVAEVR | | | Oxidation (M)[4] | Mascot |
| 1432.743 | 1432.7574 | 0.0144 | 10 | 238 | 249 | VDALQDEINFLR | | | | Mascot |
| 1432.743 | 1432.7574 | 0.0144 | 10 | 238 | 249 | VDALQDEINFLR | 118 | 100 | | Mascot |
| 1443.7074 | 1443.7195 | 0.0121 | 8 | 353 | 365 | ANLEAQIAEAEER | | | | Mascot |
| 1443.7074 | 1443.7195 | 0.0121 | 8 | 353 | 365 | ANLEAQIAEAEER | 93 | 100 | | Mascot |
| 1695.8258 | 1695.8291 | 0.0033 | 2 | 223 | 237 | DVDAAYMNKVELEAK | | | | Mascot |
| 1743.837 | 1743.854 | 0.017 | 10 | 158 | 172 | SNIDAMFEAYISNLR | 130 | 100 | | Mascot |
| 1743.837 | 1743.854 | 0.017 | 10 | 158 | 172 | SNIDAMFEAYISNLR | | | | Mascot |
| 1759.8319 | 1759.8502 | 0.0183 | 10 | 158 | 172 | SNIDAMFEAYISNLR | | | Oxidation (M)[6] | Mascot |
| 1759.8319 | 1759.8502 | 0.0183 | 10 | 158 | 172 | SNIDAMFEAYISNLR | 68 | 99.996 | Oxidation (M)[6] | Mascot |
| 1846.7913 | 1846.8077 | 0.0164 | 9 | 310 | 325 | FEEMQSTAGQYGDDLR | | | | Mascot |
| 1862.7861 | 1862.8049 | 0.0188 | 10 | 310 | 325 | FEEMQSTAGQYGDDLR | | | Oxidation (M)[4] | Mascot |
| 1899.9381 | 1899.957 | 0.0189 | 10 | 158 | 173 | SNIDAMFEAYISNLRR | | | | Mascot |
| 2041.0559 | 2041.0757 | 0.0198 | 10 | 353 | 371 | ANLEAQIAEAEERGELAVK | 131 | 100 | | Mascot |
| 2041.0559 | 2041.0757 | 0.0198 | 10 | 353 | 371 | ANLEAQIAEAEERGELAVK | | | | Mascot |
| 2102.1128 | 2101.9373 | -0.1755 | -83 | 232 | 249 | VELEAKVDALQDEINFLR | | | | Mascot |
| 2102.9448 | 2102.957 | 0.0122 | 6 | 308 | 325 | QKFEEMQSTAGQYGDDLR | | | | Mascot |
| 2118.9397 | 2118.9619 | 0.0222 | 10 | 308 | 325 | QKFEEMQSTAGQYGDDLR | | | Oxidation (M)[6] | Mascot |
| 2118.9397 | 2118.9619 | 0.0222 | 10 | 308 | 325 | QKFEEMQSTAGQYGDDLR | 84 | 100 | Oxidation (M)[6] | Mascot |

| | | | | | | | | | | |
|---|---|---|---|---|---|---|---|---|---|---|
| 3 | Krt5 protein [Danio rerio] | gi\|39645432 | 57792.3 | 5.27 | 18 | 509 | 100 | 419 | 100 | |

Peptide Information

| Calc. Mass | Obsrv. Mass | ± da | ± ppm | Start Seq. | End Seq. | Sequence | Ion Score | C. I. % | Modification | Rank | Result Type |
|---|---|---|---|---|---|---|---|---|---|---|---|
| 827.4297 | 827.4365 | 0.0068 | 8 | 155 | 161 | FASFIDK | | | | | Mascot |
| 983.4468 | 983.4488 | 0.002 | 2 | 329 | 336 | AEAESWYK | | | | | Mascot |
| 1006.5203 | 1006.527 | 0.0067 | 7 | 279 | 286 | AIFEEELR | | | | | Mascot |
| 1079.5116 | 1079.5217 | 0.0101 | 9 | 318 | 326 | AQYEDIANR | | | | | Mascot |
| 1200.6583 | 1200.6678 | 0.0095 | 8 | 406 | 415 | IKDLEDALQR | | | | | Mascot |
| 1226.5801 | 1226.5912 | 0.0111 | 9 | 327 | 336 | SRAEAESWYK | | | | | Mascot |
| 1248.6252 | 1248.6927 | 0.0675 | 54 | 203 | 213 | QLDGLGNEKMK | | | Oxidation (M)[10] | | Mascot |
| 1277.71 | 1277.7234 | 0.0134 | 10 | 435 | 445 | LALDIEIATYR | 73 | 99.999 | | | Mascot |
| 1277.71 | 1277.7234 | 0.0134 | 10 | 435 | 445 | LALDIEIATYR | | | | | Mascot |
| 1345.678 | 1345.6891 | 0.0111 | 8 | 306 | 317 | NLDMDAIVAEVR | | | | | Mascot |
| 1361.673 | 1361.6848 | 0.0118 | 9 | 306 | 317 | NLDMDAIVAEVR | | | Oxidation (M)[4] | | Mascot |
| 1362.7013 | 1362.7101 | 0.0088 | 6 | 176 | 186 | WSLLQEQTTTR | | | | | Mascot |
| 1362.7013 | 1362.7101 | 0.0088 | 6 | 176 | 186 | WSLLQEQTTTR | 71 | 99.998 | | | Mascot |
| 1385.6365 | 1385.7264 | 0.0899 | 65 | 337 | 348 | QKFEEMQSSAGK | | | Oxidation (M)[6] | | Mascot |
| 1443.7074 | 1443.7195 | 0.0121 | 8 | 382 | 394 | ANLEAQIAEAEER | | | | | Mascot |
| 1443.7074 | 1443.7195 | 0.0121 | 8 | 382 | 394 | ANLEAQIAEAEER | 93 | 100 | | | Mascot |
| 1448.738 | 1448.7517 | 0.0137 | 9 | 267 | 278 | VDSLQDEINFLR | | | | | Mascot |
| 1476.8057 | 1476.7306 | -0.0751 | -51 | 164 | 175 | FLEQQNKVLETK | | | | | Mascot |
| 1695.8258 | 1695.8291 | 0.0033 | 2 | 252 | 266 | DVDAAYMNKVELEAK | | | | | Mascot |
| 1727.8422 | 1727.8553 | 0.0131 | 8 | 187 | 201 | SNIDAMFEAYIANLR | | | | | Mascot |
| 1743.837 | 1743.854 | 0.017 | 10 | 187 | 201 | SNIDAMFEAYIANLR | 51 | 99.766 | Oxidation (M)[6] | | Mascot |
| 1743.837 | 1743.854 | 0.017 | 10 | 187 | 201 | SNIDAMFEAYIANLR | | | Oxidation (M)[6] | | Mascot |
| 1899.9381 | 1899.957 | 0.0189 | 10 | 187 | 202 | SNIDAMFEAYIANLRR | | | Oxidation (M)[6] | | Mascot |
| 2041.0559 | 2041.0757 | 0.0198 | 10 | 382 | 400 | ANLEAQIAEAEERGELAV K | 131 | 100 | | | Mascot |
| 2041.0559 | 2041.0757 | 0.0198 | 10 | 382 | 400 | ANLEAQIAEAEERGELAV K | | | | | Mascot |

| | | | | | | | | | | |
|---|---|---|---|---|---|---|---|---|---|---|
| 4 | keratin 5 [Danio rerio] | gi\|18858425 | 58550.6 | 5.34 | 17 | 500 | 100 | 419 | 100 | |

Peptide Information

| Calc. Mass | Obsrv. Mass | ± da | ± ppm | Start Seq. | End Seq. | Sequence | Ion Score | C. I. % | Modification | Rank | Result Type |
|---|---|---|---|---|---|---|---|---|---|---|---|
| 827.4297 | 827.4365 | 0.0068 | 8 | 165 | 171 | FASFIDK | | | | | Mascot |
| 983.4468 | 983.4488 | 0.002 | 2 | 339 | 346 | AEAESWYK | | | | | Mascot |
| 1006.5203 | 1006.527 | 0.0067 | 7 | 289 | 296 | AIFEEELR | | | | | Mascot |

| Calc. Mass | Obsrv. Mass | ± da | ± ppm | Start Seq. | End Seq. | Sequence | Ion Score | C. I. % | Modification | Rank | Result Type |
|---|---|---|---|---|---|---|---|---|---|---|---|
| 1079.5116 | 1079.5217 | 0.0101 | 9 | 328 | 336 | AQYEDIANR | | | | | Mascot |
| 1226.5801 | 1226.5912 | 0.0111 | 9 | 337 | 346 | SRAEAESWYK | | | | | Mascot |
| 1248.6252 | 1248.6927 | 0.0675 | 54 | 213 | 223 | QLDGLGNEKMK | | | Oxidation (M)[10] | | Mascot |
| 1277.71 | 1277.7234 | 0.0134 | 10 | 445 | 455 | LALDIEIATYR | 73 | 99.999 | | | Mascot |
| 1277.71 | 1277.7234 | 0.0134 | 10 | 445 | 455 | LALDIEIATYR | | | | | Mascot |
| 1345.678 | 1345.6891 | 0.0111 | 8 | 316 | 327 | NLDMDAIVAEVR | | | | | Mascot |
| 1361.673 | 1361.6848 | 0.0118 | 9 | 316 | 327 | NLDMDAIVAEVR | | | Oxidation (M)[4] | | Mascot |
| 1362.7013 | 1362.7101 | 0.0088 | 6 | 186 | 196 | WSLLQEQTTTR | | | | | Mascot |
| 1362.7013 | 1362.7101 | 0.0088 | 6 | 186 | 196 | WSLLQEQTTTR | 71 | 99.998 | | | Mascot |
| 1385.6365 | 1385.7264 | 0.0899 | 65 | 347 | 358 | QKFEEMQSSAGK | | | Oxidation (M)[6] | | Mascot |
| 1443.7074 | 1443.7195 | 0.0121 | 8 | 392 | 404 | ANLEAQIAEAEER | | | | | Mascot |
| 1443.7074 | 1443.7195 | 0.0121 | 8 | 392 | 404 | ANLEAQIAEAEER | 93 | 100 | | | Mascot |
| 1448.738 | 1448.7517 | 0.0137 | 9 | 277 | 288 | VDSLQDEINFLR | | | | | Mascot |
| 1476.8057 | 1476.7306 | -0.0751 | -51 | 174 | 185 | FLEQQNKVLETK | | | | | Mascot |
| 1695.8258 | 1695.8291 | 0.0033 | 2 | 262 | 276 | DVDAAYMNKVELEAK | | | | | Mascot |
| 1727.8422 | 1727.8553 | 0.0131 | 8 | 197 | 211 | SNIDAMFEAYIANLR | | | | | Mascot |
| 1743.837 | 1743.854 | 0.017 | 10 | 197 | 211 | SNIDAMFEAYIANLR | 51 | 99.766 | Oxidation (M)[6] | | Mascot |
| 1743.837 | 1743.854 | 0.017 | 10 | 197 | 211 | SNIDAMFEAYIANLR | | | Oxidation (M)[6] | | Mascot |
| 1899.9381 | 1899.957 | 0.0189 | 10 | 197 | 212 | SNIDAMFEAYIANLRR | | | Oxidation (M)[6] | | Mascot |
| 2041.0559 | 2041.0757 | 0.0198 | 10 | 392 | 410 | ANLEAQIAEAEERGELAVK | 131 | 100 | | | Mascot |
| 2041.0559 | 2041.0757 | 0.0198 | 10 | 392 | 410 | ANLEAQIAEAEERGELAVK | | | | | Mascot |

| | | | | | | | | | |
|---|---|---|---|---|---|---|---|---|---|
| 5 | keratin 8 [Danio rerio] | gi\|29335502 | 55516.4 | 5.01 | 17 | 208 | 100 | 124 | 100 |

**Peptide Information**

| Calc. Mass | Obsrv. Mass | ± da | ± ppm | Start Seq. | End Seq. | Sequence | Ion Score | C. I. % | Modification | Rank | Result Type |
|---|---|---|---|---|---|---|---|---|---|---|---|
| 827.4297 | 827.4365 | 0.0068 | 8 | 105 | 111 | FASFIDK | | | | | Mascot |
| 1063.5419 | 1063.5519 | 0.01 | 9 | 229 | 236 | QIFEEEIR | | | | | Mascot |
| 1079.5116 | 1079.5217 | 0.0101 | 9 | 268 | 276 | AQYEDIANR | | | | | Mascot |
| 1200.6583 | 1200.6678 | 0.0095 | 8 | 356 | 365 | IKDLEDALQR | | | | | Mascot |
| 1248.6252 | 1248.6927 | 0.0675 | 54 | 153 | 163 | QLDSLGNDKMK | | | | | Mascot |
| 1270.5885 | 1270.7366 | 0.1481 | 117 | 277 | 286 | SRAEAEMWYK | | | | | Mascot |
| 1277.71 | 1277.7234 | 0.0134 | 10 | 385 | 395 | LALDIEIATYR | 73 | 99.999 | | | Mascot |
| 1277.71 | 1277.7234 | 0.0134 | 10 | 385 | 395 | LALDIEIATYR | | | | | Mascot |
| 1317.691 | 1317.7031 | 0.0121 | 9 | 126 | 136 | WSLLQNQTATR | | | | | Mascot |
| 1345.678 | 1345.6891 | 0.0111 | 8 | 256 | 267 | NLDMDAIVAEVR | | | | | Mascot |

| | | | | | | | | | | | |
|---|---|---|---|---|---|---|---|---|---|---|---|
| 1361.673 | 1361.6848 | 0.0118 | 9 | 256 | 267 | NLDMDAIVAEVR | | | Oxidation (M)[4] | | Mascot |
| 1381.6667 | 1381.6779 | 0.0112 | 8 | 190 | 200 | TECENEFVLIK | | | Carbamidomethyl (C)[3] | | Mascot |
| 1449.7584 | 1449.7637 | 0.0053 | 4 | 217 | 228 | LESLTDEINFLR | | | | | Mascot |
| 1486.7133 | 1486.728 | 0.0147 | 10 | 332 | 344 | ANLENQIAEAEER | | | | | Mascot |
| 1727.8422 | 1727.8553 | 0.0131 | 8 | 137 | 151 | SNIDAMFEAYIANLR | | | | | Mascot |
| 1743.837 | 1743.854 | 0.017 | 10 | 137 | 151 | SNIDAMFEAYIANLR | 51 | 99.766 | Oxidation (M)[6] | | Mascot |
| 1743.837 | 1743.854 | 0.017 | 10 | 137 | 151 | SNIDAMFEAYIANLR | | | Oxidation (M)[6] | | Mascot |
| 1899.9381 | 1899.957 | 0.0189 | 10 | 137 | 152 | SNIDAMFEAYIANLRR | | | Oxidation (M)[6] | | Mascot |
| 2018.1644 | 2018.1835 | 0.0191 | 9 | 74 | 91 | SLLAPLNLEIDPNIQIVR | | | | | Mascot |
| 2119.1282 | 2118.9619 | -0.1663 | -78 | 211 | 228 | VELEAKLESLTDEINFLR | | | | | Mascot |
| 2119.1282 | 2118.9619 | -0.1663 | -78 | 211 | 228 | VELEAKLESLTDEINFLR | 3 | 0 | | | Mascot |
| 2146.0193 | 2146.041 | 0.0217 | 10 | 332 | 350 | ANLENQIAEAEERGEMAVR | | | Oxidation (M)[16] | | Mascot |

6 keratin, type II cytoskeletal 8 [Danio rerio] gi|41056085 57723.4 5.15 17 204 100 124 100

**Peptide Information**

| Calc. Mass | Obsrv. Mass | ± da | ± ppm | Start Seq. | End Seq. | Sequence | Ion Score | C. I. % | Modification | Rank | Result Type |
|---|---|---|---|---|---|---|---|---|---|---|---|
| 827.4297 | 827.4365 | 0.0068 | 8 | 126 | 132 | FASFIDK | | | | | Mascot |
| 1063.5419 | 1063.5519 | 0.01 | 9 | 250 | 257 | QIFEEEIR | | | | | Mascot |
| 1079.5116 | 1079.5217 | 0.0101 | 9 | 289 | 297 | AQYEDIANR | | | | | Mascot |
| 1200.6583 | 1200.6678 | 0.0095 | 8 | 377 | 386 | IKDLEDALQR | | | | | Mascot |
| 1248.6252 | 1248.6927 | 0.0675 | 54 | 174 | 184 | QLDSLGNDKMK | | | | | Mascot |
| 1270.5885 | 1270.7366 | 0.1481 | 117 | 298 | 307 | SRAEAEMWYK | | | | | Mascot |
| 1277.71 | 1277.7234 | 0.0134 | 10 | 406 | 416 | LALDIEIATYR | 73 | 99.999 | | | Mascot |
| 1277.71 | 1277.7234 | 0.0134 | 10 | 406 | 416 | LALDIEIATYR | | | | | Mascot |
| 1317.691 | 1317.7031 | 0.0121 | 9 | 147 | 157 | WSLLQNQTATR | | | | | Mascot |
| 1345.678 | 1345.6891 | 0.0111 | 8 | 277 | 288 | NLDMDAIVAEVR | | | | | Mascot |
| 1361.673 | 1361.6848 | 0.0118 | 9 | 277 | 288 | NLDMDAIVAEVR | | | Oxidation (M)[4] | | Mascot |
| 1381.6667 | 1381.6779 | 0.0112 | 8 | 211 | 221 | TECENEFVLIK | | | Carbamidomethyl (C)[3] | | Mascot |
| 1449.7584 | 1449.7637 | 0.0053 | 4 | 238 | 249 | LESLTDEINFLR | | | | | Mascot |
| 1486.7133 | 1486.728 | 0.0147 | 10 | 353 | 365 | ANLENQIAEAEER | | | | | Mascot |
| 1727.8422 | 1727.8553 | 0.0131 | 8 | 158 | 172 | SNIDAMFEAYIANLR | | | | | Mascot |
| 1743.837 | 1743.854 | 0.017 | 10 | 158 | 172 | SNIDAMFEAYIANLR | 51 | 99.766 | Oxidation (M)[6] | | Mascot |
| 1743.837 | 1743.854 | 0.017 | 10 | 158 | 172 | SNIDAMFEAYIANLR | | | Oxidation (M)[6] | | Mascot |
| 1899.9381 | 1899.957 | 0.0189 | 10 | 158 | 173 | SNIDAMFEAYIANLRR | | | Oxidation (M)[6] | | Mascot |
| 2018.1644 | 2018.1835 | 0.0191 | 9 | 95 | 112 | SLLAPLNLEIDPNIQIVR | | | | | Mascot |
| 2119.1282 | 2118.9619 | -0.1663 | -78 | 232 | 249 | VELEAKLESLTDEINFLR | | | | | Mascot |

| | | | | | | | | | | | |
|---|---|---|---|---|---|---|---|---|---|---|---|
| 2119.1282 | 2118.9619 | -0.1663 | -78 | 232 | 249 | VELEAKLESLTDEINFLR | 3 | 0 | | | Mascot |
| 2146.0193 | 2146.041 | 0.0217 | 10 | 353 | 371 | ANLENQIAEAEERGEMA VR | | | Oxidation (M)[16] | | Mascot |

| | | | | | | | | | | |
|---|---|---|---|---|---|---|---|---|---|---|
| 7 | Keratin 8 [Danio rerio] | gi\|159155987 | 57624.4 | 5.1 | 16 | 197 | 100 | 124 | 100 | |

**Peptide Information**

| Calc. Mass | Obsrv. Mass | ± da | ± ppm | Start Seq. | End Seq. | Sequence | Ion Score | C. I. % | Modification | Rank | Result Type |
|---|---|---|---|---|---|---|---|---|---|---|---|
| 827.4297 | 827.4365 | 0.0068 | 8 | 126 | 132 | FASFIDK | | | | | Mascot |
| 1063.5419 | 1063.5519 | 0.01 | 9 | 250 | 257 | QIFEEEIR | | | | | Mascot |
| 1079.5116 | 1079.5217 | 0.0101 | 9 | 289 | 297 | AQYEDIANR | | | | | Mascot |
| 1200.6583 | 1200.6678 | 0.0095 | 8 | 377 | 386 | IKDLEDALQR | | | | | Mascot |
| 1248.6252 | 1248.6927 | 0.0675 | 54 | 174 | 184 | QLDSLGNDKMK | | | | | Mascot |
| 1270.5885 | 1270.7366 | 0.1481 | 117 | 298 | 307 | SRAEAEMWYK | | | | | Mascot |
| 1277.71 | 1277.7234 | 0.0134 | 10 | 406 | 416 | LALDIEIATYR | 73 | 99.999 | | | Mascot |
| 1277.71 | 1277.7234 | 0.0134 | 10 | 406 | 416 | LALDIEIATYR | | | | | Mascot |
| 1317.691 | 1317.7031 | 0.0121 | 9 | 147 | 157 | WSLLQNQTATR | | | | | Mascot |
| 1381.6667 | 1381.6779 | 0.0112 | 8 | 211 | 221 | TECENEFVLIK | | | Carbamidomethyl (C)[3] | | Mascot |
| 1449.7584 | 1449.7637 | 0.0053 | 4 | 238 | 249 | LESLTDEINFLR | | | | | Mascot |
| 1486.7133 | 1486.728 | 0.0147 | 10 | 353 | 365 | ANLENQIAEAEER | | | | | Mascot |
| 1727.8422 | 1727.8553 | 0.0131 | 8 | 158 | 172 | SNIDAMFEAYIANLR | | | | | Mascot |
| 1743.837 | 1743.854 | 0.017 | 10 | 158 | 172 | SNIDAMFEAYIANLR | 51 | 99.766 | Oxidation (M)[6] | | Mascot |
| 1743.837 | 1743.854 | 0.017 | 10 | 158 | 172 | SNIDAMFEAYIANLR | | | Oxidation (M)[6] | | Mascot |
| 1899.9381 | 1899.957 | 0.0189 | 10 | 158 | 173 | SNIDAMFEAYIANLRR | | | Oxidation (M)[6] | | Mascot |
| 2018.1644 | 2018.1835 | 0.0191 | 9 | 95 | 112 | SLLAPLNLEIDPNIQIVR | | | | | Mascot |
| 2119.1282 | 2118.9619 | -0.1663 | -78 | 232 | 249 | VELEAKLESLTDEINFLR | | | | | Mascot |
| 2119.1282 | 2118.9619 | -0.1663 | -78 | 232 | 249 | VELEAKLESLTDEINFLR | 3 | 0 | | | Mascot |
| 2146.0193 | 2146.041 | 0.0217 | 10 | 353 | 371 | ANLENQIAEAEERGEMA VR | | | Oxidation (M)[16] | | Mascot |

| | | | | | | | | | | |
|---|---|---|---|---|---|---|---|---|---|---|
| 8 | glial fibrillary acidic protein [Danio rerio] | gi\|20977259 | 42127.4 | 5.03 | 7 | 128 | 100 | 108 | 100 | |

**Peptide Information**

| Calc. Mass | Obsrv. Mass | ± da | ± ppm | Start Seq. | End Seq. | Sequence | Ion Score | C. I. % | Modification | Rank | Result Type |
|---|---|---|---|---|---|---|---|---|---|---|---|
| 850.4265 | 850.5497 | 0.1232 | 145 | 351 | 357 | ESTTERK | | | | | Mascot |
| 1277.71 | 1277.7234 | 0.0134 | 10 | 285 | 295 | LALDIEIATYR | 73 | 99.999 | | | Mascot |
| 1277.71 | 1277.7234 | 0.0134 | 10 | 285 | 295 | LALDIEIATYR | | | | | Mascot |
| 1308.6543 | 1308.6664 | 0.0121 | 9 | 187 | 198 | SKFADLTDAANR | | | | | Mascot |

| | | | | | | | | | | | |
|---|---|---|---|---|---|---|---|---|---|---|---|
| 1377.6857 | 1377.684 | -0.0017 | -1 | 345 | 356 | DGEIIKESTTER | | | | | Mascot |
| 1387.7726 | 1387.6953 | -0.0773 | -56 | 24 | 35 | MLVAELNQLRGK | | | Oxidation (M)[1] | | Mascot |
| 1432.7682 | 1432.7574 | -0.0108 | -8 | 118 | 129 | IEALQDEINFLK | | | | | Mascot |
| 1432.7682 | 1432.7574 | -0.0108 | -8 | 118 | 129 | IEALQDEINFLK | 34 | 89.818 | | | Mascot |
| 1688.816 | 1688.9028 | 0.0868 | 51 | 319 | 333 | DTSMDTKLTPEAHVK | | | Oxidation (M)[4] | | Mascot |

| | | | | | | | | | | |
|---|---|---|---|---|---|---|---|---|---|---|
| 9 | class III intermediate filament protein [Danio rerio] | gi\|38374177 | 51255 | 5.28 | 7 | 127 | 100 | 108 | 100 |

Peptide Information

| Calc. Mass | Obsrv. Mass | ± da | ± ppm | Start Seq. | End Seq. | Sequence | Ion Score | C. I. % | Modification | Rank | Result Type |
|---|---|---|---|---|---|---|---|---|---|---|---|
| 850.4265 | 850.5497 | 0.1232 | 145 | 435 | 441 | ESTTERK | | | | | Mascot |
| 1277.71 | 1277.7234 | 0.0134 | 10 | 369 | 379 | LALDIEIATYR | 73 | 99.999 | | | Mascot |
| 1277.71 | 1277.7234 | 0.0134 | 10 | 369 | 379 | LALDIEIATYR | | | | | Mascot |
| 1308.6543 | 1308.6664 | 0.0121 | 9 | 271 | 282 | SKFADLTDAANR | | | | | Mascot |
| 1377.6857 | 1377.684 | -0.0017 | -1 | 429 | 440 | DGEIIKESTTER | | | | | Mascot |
| 1387.7726 | 1387.6953 | -0.0773 | -56 | 108 | 119 | MLVAELNQLRGK | | | Oxidation (M)[1] | | Mascot |
| 1432.7682 | 1432.7574 | -0.0108 | -8 | 202 | 213 | IEALQDEINFLK | | | | | Mascot |
| 1432.7682 | 1432.7574 | -0.0108 | -8 | 202 | 213 | IEALQDEINFLK | 34 | 89.818 | | | Mascot |
| 1688.8749 | 1688.9028 | 0.0279 | 17 | 299 | 312 | RQIQGLTCDLESLR | | | Carbamidomethyl (C)[8] | | Mascot |

| | | | | | | | | | | |
|---|---|---|---|---|---|---|---|---|---|---|
| 10 | Gfap protein [Danio rerio] | gi\|124504410 | 51101 | 5.34 | 7 | 126 | 100 | 108 | 100 |

Peptide Information

| Calc. Mass | Obsrv. Mass | ± da | ± ppm | Start Seq. | End Seq. | Sequence | Ion Score | C. I. % | Modification | Rank | Result Type |
|---|---|---|---|---|---|---|---|---|---|---|---|
| 850.4265 | 850.5497 | 0.1232 | 145 | 434 | 440 | ESTTERK | | | | | Mascot |
| 1277.71 | 1277.7234 | 0.0134 | 10 | 368 | 378 | LALDIEIATYR | 73 | 99.999 | | | Mascot |
| 1277.71 | 1277.7234 | 0.0134 | 10 | 368 | 378 | LALDIEIATYR | | | | | Mascot |
| 1308.6543 | 1308.6664 | 0.0121 | 9 | 270 | 281 | SKFADLTDAANR | | | | | Mascot |
| 1377.6857 | 1377.684 | -0.0017 | -1 | 428 | 439 | DGEIIKESTTER | | | | | Mascot |
| 1387.7726 | 1387.6953 | -0.0773 | -56 | 107 | 118 | MLVAELNQLRGK | | | Oxidation (M)[1] | | Mascot |
| 1432.7682 | 1432.7574 | -0.0108 | -8 | 201 | 212 | IEALQDEINFLK | | | | | Mascot |
| 1432.7682 | 1432.7574 | -0.0108 | -8 | 201 | 212 | IEALQDEINFLK | 34 | 89.818 | | | Mascot |
| 1688.8749 | 1688.9028 | 0.0279 | 17 | 298 | 311 | RQIQGLTCDLESLR | | | Carbamidomethyl (C)[8] | | Mascot |

| | | | | | | | | | | |
|---|---|---|---|---|---|---|---|---|---|---|
| 11 | Zgc:158846 [Danio rerio] | gi\|158253572 | 55223 | 5.44 | 6 | 86 | 99.99 | 73 | 99.999 |

Peptide Information

| Calc. Mass | Obsrv. Mass | ± da | ± ppm | Start Seq. | End Seq. | Sequence | Ion Score | C. I. % | Modification | Rank | Result Type |
|---|---|---|---|---|---|---|---|---|---|---|---|

| Calc. Mass | Obsrv. Mass | ± da | ± ppm | Start Seq. | End Seq. | Sequence | Ion Score | C. I. % | Modification | Rank | Result Type |
|---|---|---|---|---|---|---|---|---|---|---|---|
| 827.4297 | 827.4365 | 0.0068 | 8 | 87 | 93 | FASFIDK | | | | | Mascot |
| 996.5472 | 996.6054 | 0.0582 | 58 | 421 | 429 | DIHNTGLVK | | | | | Mascot |
| 1124.5793 | 1124.6771 | 0.0978 | 87 | 437 | 448 | GGSSSSLTTVTK | | | | | Mascot |
| 1277.71 | 1277.7234 | 0.0134 | 10 | 367 | 377 | LALDIEIATYR | 73 | 99.999 | | | Mascot |
| 1277.71 | 1277.7234 | 0.0134 | 10 | 367 | 377 | LALDIEIATYR | | | | | Mascot |
| 1394.7162 | 1394.7837 | 0.0675 | 48 | 172 | 183 | SDSDNEFVLLKK | | | | | Mascot |
| 2434.123 | 2434.1011 | -0.0219 | -9 | 261 | 280 | AEAESWYQQKYEEMQLT ATK | | | | | Mascot |

| | | | | | | | | | | |
|---|---|---|---|---|---|---|---|---|---|---|
| 12 | glial fibrillary acidic protein [Danio rerio] | gi\|239596173 | 19203.6 | 4.89 | 3 | 85 | 99.986 | 73 | 99.999 | |

Peptide Information

| Calc. Mass | Obsrv. Mass | ± da | ± ppm | Start Seq. | End Seq. | Sequence | Ion Score | C. I. % | Modification | Rank | Result Type |
|---|---|---|---|---|---|---|---|---|---|---|---|
| 1277.71 | 1277.7234 | 0.0134 | 10 | 110 | 120 | LALDIEIATYR | 73 | 99.999 | | | Mascot |
| 1277.71 | 1277.7234 | 0.0134 | 10 | 110 | 120 | LALDIEIATYR | | | | | Mascot |
| 1308.6543 | 1308.6664 | 0.0121 | 9 | 12 | 23 | SKFADLTDAANR | | | | | Mascot |
| 1688.8749 | 1688.9028 | 0.0279 | 17 | 40 | 53 | RQIQGLTCDLESLR | | | Carbamidomethyl (C)[8] | | Mascot |

| | | | | | | | | | | |
|---|---|---|---|---|---|---|---|---|---|---|
| 13 | novel protein similar to vertebrate keratin family [Danio rerio] | gi\|157885973 | 46874.6 | 5.23 | 2 | 79 | 99.943 | 73 | 99.999 | |

Peptide Information

| Calc. Mass | Obsrv. Mass | ± da | ± ppm | Start Seq. | End Seq. | Sequence | Ion Score | C. I. % | Modification | Rank | Result Type |
|---|---|---|---|---|---|---|---|---|---|---|---|
| 1256.5793 | 1256.7084 | 0.1291 | 103 | 286 | 295 | EEAESWYKSK | | | | | Mascot |
| 1256.5793 | 1256.7084 | 0.1291 | 103 | 286 | 295 | EEAESWYKSK | | | | | Mascot |
| 1277.71 | 1277.7234 | 0.0134 | 10 | 392 | 402 | LALDIEIATYR | 73 | 99.999 | | | Mascot |
| 1277.71 | 1277.7234 | 0.0134 | 10 | 392 | 402 | LALDIEIATYR | | | | | Mascot |

| | | | | | | | | | | |
|---|---|---|---|---|---|---|---|---|---|---|
| 14 | keratin type II, tail domain [Danio rerio] | gi\|4107170 | 10583.9 | 8.29 | 1 | 78 | 99.931 | 73 | 99.999 | |

Peptide Information

| Calc. Mass | Obsrv. Mass | ± da | ± ppm | Start Seq. | End Seq. | Sequence | Ion Score | C. I. % | Modification | Rank | Result Type |
|---|---|---|---|---|---|---|---|---|---|---|---|
| 1277.71 | 1277.7234 | 0.0134 | 10 | 4 | 14 | LALDIEIATYR | 73 | 99.999 | | | Mascot |
| 1277.71 | 1277.7234 | 0.0134 | 10 | 4 | 14 | LALDIEIATYR | | | | | Mascot |

| | | | | | | | | | | |
|---|---|---|---|---|---|---|---|---|---|---|
| 15 | PREDICTED: keratin, type II cytoskeletal 8 [Danio rerio] | gi\|125849603 | 49959.2 | 5.46 | 2 | 73 | 99.797 | 73 | 99.999 | |

Peptide Information

| Calc. Mass | Obsrv. Mass | ± da | ± ppm | Start | End | Sequence | Ion | C. I. % | Modification | Rank | Result Type |
|---|---|---|---|---|---|---|---|---|---|---|---|

| | | | | Seq. | Seq. | | Score | | | | |
|---|---|---|---|---|---|---|---|---|---|---|---|
| 1256.5793 | 1256.7084 | 0.1291 | 103 | 264 | 273 | EEAESWYKSK | | | | | Mascot |
| 1256.5793 | 1256.7084 | 0.1291 | 103 | 264 | 273 | EEAESWYKSK | | | | | Mascot |
| 1277.71 | 1277.7234 | 0.0134 | 10 | 370 | 380 | LALDIEIATYR | 73 | 99.999 | | | Mascot |
| 1277.71 | 1277.7234 | 0.0134 | 10 | 370 | 380 | LALDIEIATYR | | | | | Mascot |

| 16 | tropomyosin alpha-3 chain isoform 2 [Danio rerio] | gi\|41393141 | 28826.9 | 4.76 | 9 | 46 | 0 |
|---|---|---|---|---|---|---|---|

**Peptide Information**

| Calc. Mass | Obsrv. Mass | ± da | ± ppm | Start Seq. | End Seq. | Sequence | Ion Score | C. I. % | Modification | Rank | Result Type |
|---|---|---|---|---|---|---|---|---|---|---|---|
| 950.4247 | 950.501 | 0.0763 | 80 | 156 | 162 | QMEEELR | | | Oxidation (M)[2] | | Mascot |
| 983.5156 | 983.4488 | -0.0668 | -68 | 147 | 155 | AELAESHVK | | | | | Mascot |
| 1248.6365 | 1248.6927 | 0.0562 | 45 | 1 | 12 | MAGSNSIDAVKR | | | | | Mascot |
| 1308.6178 | 1308.6664 | 0.0486 | 37 | 198 | 208 | EAETRAEFAER | | | | | Mascot |
| 1443.8053 | 1443.7195 | -0.0858 | -59 | 70 | 82 | LATALQKLEEAEK | | | | | Mascot |
| 1443.8053 | 1443.7195 | -0.0858 | -59 | 70 | 82 | LATALQKLEEAEK | | | | | Mascot |
| 1476.6813 | 1476.7306 | 0.0493 | 33 | 77 | 89 | LEEAEKAADESER | | | | | Mascot |
| 1688.849 | 1688.9028 | 0.0538 | 32 | 234 | 248 | IHATLDQTLSELNSF | | | | | Mascot |
| 1727.8922 | 1727.8553 | -0.0369 | -21 | 56 | 69 | IQLVEEELDRAQER | | | | | Mascot |
| 1914.9225 | 1914.9497 | 0.0272 | 14 | 147 | 162 | AELAESHVKQMEEELR | | | Oxidation (M)[11] | | Mascot |

| 17 | PREDICTED: hypothetical protein LOC565819 [Danio rerio] | gi\|189531659 | 67893.7 | 9.41 | 12 | 46 | 0 |
|---|---|---|---|---|---|---|---|

**Peptide Information**

| Calc. Mass | Obsrv. Mass | ± da | ± ppm | Start Seq. | End Seq. | Sequence | Ion Score | C. I. % | Modification | Rank | Result Type |
|---|---|---|---|---|---|---|---|---|---|---|---|
| 1006.4985 | 1006.527 | 0.0285 | 28 | 134 | 141 | ERMSQVEK | | | | | Mascot |
| 1079.5731 | 1079.5217 | -0.0514 | -48 | 38 | 46 | TLEAYQNLK | | | | | Mascot |
| 1226.5648 | 1226.5912 | 0.0264 | 22 | 487 | 496 | YNTITDSEQR | | | | | Mascot |
| 1256.6732 | 1256.7084 | 0.0352 | 28 | 231 | 242 | ADLSPVLDEVAK | | | | | Mascot |
| 1256.6732 | 1256.7084 | 0.0352 | 28 | 231 | 242 | ADLSPVLDEVAK | | | | | Mascot |
| 1277.6055 | 1277.7234 | 0.1179 | 92 | 6 | 16 | QEHRYGLSCGK | | | | | Mascot |
| 1277.6055 | 1277.7234 | 0.1179 | 92 | 6 | 16 | QEHRYGLSCGK | | | | | Mascot |
| 1345.6707 | 1345.6891 | 0.0184 | 14 | 108 | 119 | DRLEGQGSIQDK | | | | | Mascot |
| 1394.6733 | 1394.7837 | 0.1104 | 79 | 136 | 146 | MSQVEKDIWSR | | | Oxidation (M)[1] | | Mascot |
| 1448.7592 | 1448.7517 | -0.0075 | -5 | 283 | 296 | LQPLSSSSSSQLK | | | | | Mascot |
| 1688.9303 | 1688.9028 | -0.0275 | -16 | 187 | 201 | NGGPSLVTHRPLRER | | | | | Mascot |

| | | | | | | | | | | | |
|---|---|---|---|---|---|---|---|---|---|---|---|
| 1846.9215 | 1846.8077 | -0.1138 | -62 | 307 | 324 | TSGDSPSQLSPVKTPSMK | | | | | Mascot |
| 1862.9164 | 1862.8049 | -0.1115 | -60 | 307 | 324 | TSGDSPSQLSPVKTPSMK | | | Oxidation (M)[17] | | Mascot |
| 2041.0964 | 2041.0757 | -0.0207 | -10 | 576 | 593 | LSHIKGLILDYDQTQLPS | | | | | Mascot |
| 2041.0964 | 2041.0757 | -0.0207 | -10 | 576 | 593 | LSHIKGLILDYDQTQLPS | | | | | Mascot |
| 2119.0601 | 2118.9619 | -0.0982 | -46 | 320 | 338 | TPSMKRPVPSDHPESQTPK | | | | | Mascot |
| 2119.0601 | 2118.9619 | -0.0982 | -46 | 320 | 338 | TPSMKRPVPSDHPESQTPK | | | | | Mascot |

| | | | | | | | | | |
|---|---|---|---|---|---|---|---|---|---|
| 18 | WD repeat-containing protein 74 [Danio rerio] | gi\|115497604 | 41314.9 | 6.76 | 10 | 43 | 0 | | |

**Peptide Information**

| Calc. Mass | Obsrv. Mass | ± da | ± ppm | Start Seq. | End Seq. | Sequence | Ion Score | C. I. % | Modification | Rank | Result Type |
|---|---|---|---|---|---|---|---|---|---|---|---|
| 1063.5061 | 1063.5519 | 0.0458 | 43 | 131 | 138 | MRQNQSQR | | | Oxidation (M)[1] | | Mascot |
| 1270.5997 | 1270.7366 | 0.1369 | 108 | 167 | 176 | NVAHDWLEMR | | | | | Mascot |
| 1317.691 | 1317.7031 | 0.0121 | 9 | 202 | 212 | VRVYDPSTPQR | | | | | Mascot |
| 1387.744 | 1387.6953 | -0.0487 | -35 | 139 | 151 | HHVATGGKENPLK | | | | | Mascot |
| 1432.6914 | 1432.7574 | 0.066 | 46 | 353 | 367 | AAEKDEAAVTGADGK | | | | | Mascot |
| 1432.6914 | 1432.7574 | 0.066 | 46 | 353 | 367 | AAEKDEAAVTGADGK | | | | | Mascot |
| 1448.7605 | 1448.7517 | -0.0088 | -6 | 290 | 301 | VHSLEDRSLQHK | | | | | Mascot |
| 1695.8007 | 1695.8291 | 0.0284 | 17 | 74 | 89 | GIFTATKECGDPSQGK | | | Carbamidomethyl (C)[9] | | Mascot |
| 1846.9697 | 1846.8077 | -0.162 | -88 | 177 | 192 | VPVWVRDISFIADSDK | | | | | Mascot |
| 2005.0786 | 2005.0021 | -0.0765 | -38 | 2 | 20 | AAKSQICSIWLGSETGILK | | | | | Mascot |
| 2282.9968 | 2283.1985 | 0.2017 | 88 | 332 | 350 | AEEEDEVWDNMETVTEKTK | | | | | Mascot |

| | | | | | | | | | |
|---|---|---|---|---|---|---|---|---|---|
| 19 | Si:ch211-20b12.1 [Danio rerio] | gi\|67678177 | 55996.8 | 8.09 | 3 | 42 | 0 | 35 | 91.374 |

**Peptide Information**

| Calc. Mass | Obsrv. Mass | ± da | ± ppm | Start Seq. | End Seq. | Sequence | Ion Score | C. I. % | Modification | Rank | Result Type |
|---|---|---|---|---|---|---|---|---|---|---|---|
| 827.4522 | 827.4365 | -0.0157 | -19 | 393 | 399 | SPIAFHR | | | | | Mascot |
| 983.5533 | 983.4488 | -0.1045 | -106 | 392 | 399 | RSPIAFHR | | | | | Mascot |
| 1277.6307 | 1277.7234 | 0.0927 | 73 | 436 | 445 | CHDIIISTYR | 35 | 91.374 | Carbamidomethyl (C)[1] | | Mascot |
| 1277.6307 | 1277.7234 | 0.0927 | 73 | 436 | 445 | CHDIIISTYR | | | Carbamidomethyl (C)[1] | | Mascot |

| | | | | | | | | | |
|---|---|---|---|---|---|---|---|---|---|
| 20 | Ift81 protein [Danio rerio] | gi\|145337899 | 29908.3 | 5.76 | 9 | 39 | 0 | | |

**Peptide Information**

| Calc. Mass | Obsrv. Mass | ± da | ± ppm | Start Seq. | End Seq. | Sequence | Ion Score | C. I. % | Modification | Rank | Result Type |
|---|---|---|---|---|---|---|---|---|---|---|---|

| | | | | | | | | | | | |
|---|---|---|---|---|---|---|---|---|---|---|---|
| 1045.5636 | 1045.5718 | 0.0082 | 8 | 14 | 21 | TEEILRER | | | | | Mascot |
| 1063.5339 | 1063.5519 | 0.018 | 17 | 224 | 232 | DLETLLECK | | | | | Mascot |
| 1200.5123 | 1200.6678 | 0.1555 | 130 | 65 | 74 | TLDDMSEMVK | | | Oxidation (M)[5,8] | | Mascot |
| 1248.6504 | 1248.6927 | 0.0423 | 34 | 224 | 233 | DLETLLECKK | | | Carbamidomethyl (C)[8] | | Mascot |
| 1381.645 | 1381.6779 | 0.0329 | 24 | 63 | 74 | GRTLDDMSEMVK | | | | | Mascot |
| 1448.6976 | 1448.7517 | 0.0541 | 37 | 135 | 146 | TLREETAQEESR | | | | | Mascot |
| 1727.8018 | 1727.8553 | 0.0535 | 31 | 113 | 128 | AQYETCAAGLESNRSK | | | | | Mascot |
| 2225.0879 | 2225.1394 | 0.0515 | 23 | 147 | 163 | YHHINCEREIIESQIQR | | | Carbamidomethyl (C)[6] | | Mascot |
| 2283.1914 | 2283.1985 | 0.0071 | 3 | 234 | 254 | QCYLKAQSQAPIGHVIQD VGK | | | | | Mascot |

| | | | | | | | | |
|---|---|---|---|---|---|---|---|---|
| 21 | PREDICTED: probable ATP-dependent RNA helicase YTHDC2-like [Danio rerio] | gi\|326667516 | 148100.7 | 8.32 | 19 | 39 | 0 | |

Peptide Information

| Calc. Mass | Obsrv. Mass | ± da | ± ppm | Start Seq. | End Seq. | Sequence | Ion Score | C. I. % | Modification | Rank | Result Type |
|---|---|---|---|---|---|---|---|---|---|---|---|
| 842.4553 | 842.5168 | 0.0615 | 73 | 743 | 749 | HAVQMLK | | | Oxidation (M)[5] | | Mascot |
| 915.5258 | 915.4971 | -0.0287 | -31 | 259 | 266 | LESRVSPK | | | | | Mascot |
| 950.4359 | 950.501 | 0.0651 | 68 | 86 | 93 | KDGMEAQR | | | Oxidation (M)[4] | | Mascot |
| 983.4462 | 983.4488 | 0.0026 | 3 | 1256 | 1264 | MTSAVSSER | | | Oxidation (M)[1] | | Mascot |
| 987.5217 | 987.5178 | -0.0039 | -4 | 1208 | 1215 | NIELSQQR | | | | | Mascot |
| 1045.5823 | 1045.5718 | -0.0105 | -10 | 106 | 114 | ALVQSLMQR | | | | | Mascot |
| 1079.6095 | 1079.5217 | -0.0878 | -81 | 62 | 71 | LAQSLGYISK | | | | | Mascot |
| 1200.6848 | 1200.6678 | -0.017 | -14 | 150 | 160 | LSHGIPQVPPR | | | | | Mascot |
| 1248.6331 | 1248.6927 | 0.0596 | 48 | 161 | 171 | RSSAPELDAFR | | | | | Mascot |
| 1317.5232 | 1317.7031 | 0.1799 | 137 | 94 | 105 | SSMSCSLSPSCR | | | Carbamidomethyl (C)[5], Oxidation (M)[3] | | Mascot |
| 1381.7396 | 1381.6779 | -0.0617 | -45 | 303 | 314 | DGLTDFLLTKMK | | | | | Mascot |
| 1385.6915 | 1385.7264 | 0.0349 | 25 | 706 | 716 | MPLQELCLHTK | | | Carbamidomethyl (C)[7], Oxidation (M)[1] | | Mascot |
| 1743.9388 | 1743.854 | -0.0848 | -49 | 1045 | 1059 | LDPETAELVFGLRQR | | | | | Mascot |
| 1743.9388 | 1743.854 | -0.0848 | -49 | 1045 | 1059 | LDPETAELVFGLRQR | | | | | Mascot |
| 1900.0538 | 1899.957 | -0.0968 | -51 | 173 | 188 | ALPVYELQEQIIQSIR | | | | | Mascot |
| 2086.075 | 2085.9387 | -0.1363 | -65 | 496 | 514 | GFVSHTEQLLNMGANINI K | | | | | Mascot |
| 2102.0698 | 2101.9373 | -0.1325 | -63 | 496 | 514 | GFVSHTEQLLNMGANINI K | | | Oxidation (M)[12] | | Mascot |
| 2119.0566 | 2118.9619 | -0.0947 | -45 | 44 | 61 | ELDFPSSLTNSERAFIHR | | | | | Mascot |
| 2119.0566 | 2118.9619 | -0.0947 | -45 | 44 | 61 | ELDFPSSLTNSERAFIHR | | | | | Mascot |
| 2145.8779 | 2146.041 | 0.1631 | 76 | 87 | 105 | DGMEAQRSSMSCSLSPS CR | | | Carbamidomethyl (C)[12,18] | | Mascot |
| 2233.2195 | 2233.1072 | -0.1123 | -50 | 320 | 339 | MPNLRLVLSSAALDVSLF MR | | | | | Mascot |

| | | | | | | | | | | | |
|---|---|---|---|---|---|---|---|---|---|---|---|
| 2283.9604 | 2284.1965 | 0.2361 | 103 | 381 | 398 | EAQKEESQQCSLSEWCER | | | Carbamidomethyl (C)[10,16] | | Mascot |

| 22 | CDK5 regulatory subunit-associated protein 1 [Danio rerio] | gi\|160333602 | 65811.6 | 9.24 | 13 | 38 | 0 |
|---|---|---|---|---|---|---|---|

**Peptide Information**

| Calc. Mass | Obsrv. Mass | ± da | ± ppm | Start Seq. | End Seq. | Sequence | Ion Score | C. I. % | Modification | Rank | Result Type |
|---|---|---|---|---|---|---|---|---|---|---|---|
| 987.4022 | 987.5178 | 0.1156 | 117 | 303 | 310 | MQFSCSER | | | | | Mascot |
| 996.5295 | 996.6054 | 0.0759 | 76 | 1 | 8 | MNRISTFK | | | | | Mascot |
| 1051.6007 | 1051.7223 | 0.1216 | 116 | 204 | 213 | LLSLAHGGQR | | | | | Mascot |
| 1317.7021 | 1317.7031 | 0.001 | 1 | 374 | 385 | HLHLPVQSGSSR | | | | | Mascot |
| 1345.7507 | 1345.6891 | -0.0616 | -46 | 127 | 138 | ADVVLLVTCSIR | | | Carbamidomethyl (C)[9] | | Mascot |
| 1362.7627 | 1362.7101 | -0.0526 | -39 | 476 | 486 | LEELISVFREK | | | | | Mascot |
| 1362.7627 | 1362.7101 | -0.0526 | -39 | 476 | 486 | LEELISVFREK | | | | | Mascot |
| 1432.704 | 1432.7574 | 0.0534 | 37 | 563 | 575 | GHALNHSSPNDRK | | | | | Mascot |
| 1432.704 | 1432.7574 | 0.0534 | 37 | 563 | 575 | GHALNHSSPNDRK | | | | | Mascot |
| 1476.8057 | 1476.7306 | -0.0751 | -51 | 397 | 408 | EAYLELVENIRK | | | | | Mascot |
| 1695.8445 | 1695.8291 | -0.0154 | -9 | 538 | 552 | TQMACITPGDYVLVK | | | Carbamidomethyl (C)[5] | | Mascot |
| 1743.7902 | 1743.854 | 0.0638 | 37 | 303 | 316 | MQFSCSERNTQLSR | 2 | 0 | Carbamidomethyl (C)[5] | | Mascot |
| 1743.7902 | 1743.854 | 0.0638 | 37 | 303 | 316 | MQFSCSERNTQLSR | | | Carbamidomethyl (C)[5] | | Mascot |
| 1759.785 | 1759.8502 | 0.0652 | 37 | 303 | 316 | MQFSCSERNTQLSR | | | Carbamidomethyl (C)[5], Oxidation (M)[1] | | Mascot |
| 1759.785 | 1759.8502 | 0.0652 | 37 | 303 | 316 | MQFSCSERNTQLSR | | | Carbamidomethyl (C)[5], Oxidation (M)[1] | | Mascot |
| 1846.8468 | 1846.8077 | -0.0391 | -21 | 440 | 454 | EVGYNIGFFFTYSMR | | | Oxidation (M)[14] | | Mascot |
| 1862.9653 | 1862.8049 | -0.1604 | -86 | 369 | 385 | SNICKHLHLPVQSGSSR | | | | | Mascot |
| 2017.9535 | 2018.1835 | 0.23 | 114 | 50 | 67 | SKLTNGPSFQDFIMSSDK | | | Oxidation (M)[14] | | Mascot |

| 23 | PREDICTED: zinc finger protein 420 isoform 2 [Danio rerio] | gi\|326678086 | 70371 | 9.08 | 14 | 38 | 0 |
|---|---|---|---|---|---|---|---|

**Peptide Information**

| Calc. Mass | Obsrv. Mass | ± da | ± ppm | Start Seq. | End Seq. | Sequence | Ion Score | C. I. % | Modification | Rank | Result Type |
|---|---|---|---|---|---|---|---|---|---|---|---|
| 842.5206 | 842.5168 | -0.0038 | -5 | 25 | 31 | QRQGLLK | | | | | Mascot |
| 950.3706 | 950.501 | 0.1304 | 137 | 36 | 43 | MSDPEPCR | | | Oxidation (M)[1] | | Mascot |
| 987.5621 | 987.5178 | -0.0443 | -45 | 497 | 505 | TFITAGHLK | | | | | Mascot |
| 1045.536 | 1045.5718 | 0.0358 | 34 | 134 | 141 | FHCARNLK | | | Carbamidomethyl (C)[3] | | Mascot |
| 1200.5387 | 1200.6678 | 0.1291 | 108 | 178 | 188 | EKPYSCSVCGK | | | | | Mascot |
| 1248.5712 | 1248.6927 | 0.1215 | 97 | 36 | 45 | MSDPEPCRIK | | | Carbamidomethyl (C)[7], Oxidation (M)[1] | | Mascot |
| 1270.5667 | 1270.7366 | 0.1699 | 134 | 575 | 584 | CDQCGKSFLR | | | Carbamidomethyl (C)[1,4] | | Mascot |

| Calc. Mass | Obsrv. Mass | ± da | ± ppm | Start Seq. | End Seq. | Sequence | Ion Score | C. I. % | Modification | Rank | Result Type |
|---|---|---|---|---|---|---|---|---|---|---|---|
| 1727.8381 | 1727.8553 | 0.0172 | 10 | 556 | 571 | VSSSLNSHMLTHTGEK | | | | | Mascot |
| 1743.833 | 1743.854 | 0.021 | 12 | 556 | 571 | VSSSLNSHMLTHTGEK | | | Oxidation (M)[9] | | Mascot |
| 1743.9144 | 1743.854 | -0.0604 | -35 | 295 | 309 | CGHCGKTFLRPAVLK | | | Carbamidomethyl (C)[1,4] | | Mascot |
| 1759.9412 | 1759.8502 | -0.091 | -52 | 81 | 95 | IKIDHNFLMETTAVK | | | | | Mascot |
| 1759.9412 | 1759.8502 | -0.091 | -52 | 81 | 95 | IKIDHNFLMETTAVK | | | | | Mascot |
| 2004.9531 | 2005.0021 | 0.049 | 24 | 538 | 555 | VHTGEKPFACTHCGKSFR | | | | | Mascot |
| 2102.0085 | 2101.9373 | -0.0712 | -34 | 488 | 505 | EFVCFECEKTFITAGHLK | | | | | Mascot |
| 2118.9958 | 2118.9619 | -0.0339 | -16 | 538 | 555 | VHTGEKPFACTHCGKSFR | | | Carbamidomethyl (C)[10,13] | | Mascot |
| 2118.9958 | 2118.9619 | -0.0339 | -16 | 538 | 555 | VHTGEKPFACTHCGKSFR | | | Carbamidomethyl (C)[10,13] | | Mascot |
| 2211.0798 | 2211.1282 | 0.0484 | 22 | 450 | 468 | IHLRVHTNEKPYSCPECGK | | | | | Mascot |
| 2211.0798 | 2211.1282 | 0.0484 | 22 | 450 | 468 | IHLRVHTNEKPYSCPECGK | | | | | Mascot |
| 2283.0579 | 2283.1985 | 0.1406 | 62 | 227 | 245 | HQMIHTGEKPYKCSHCDLR | | | | | Mascot |

24 Anxa1c protein [Danio rerio] gi|79153964 31472.1 5.92 8 38 0

**Peptide Information**

| Calc. Mass | Obsrv. Mass | ± da | ± ppm | Start Seq. | End Seq. | Sequence | Ion Score | C. I. % | Modification | Rank | Result Type |
|---|---|---|---|---|---|---|---|---|---|---|---|
| 827.3967 | 827.4365 | 0.0398 | 48 | 249 | 254 | IMQEYK | | | Oxidation (M)[2] | | Mascot |
| 983.4978 | 983.4488 | -0.049 | -50 | 249 | 255 | IMQEYKR | | | Oxidation (M)[2] | | Mascot |
| 1006.4952 | 1006.527 | 0.0318 | 32 | 143 | 151 | ALFEAGENR | | | | | Mascot |
| 1361.6292 | 1361.6848 | 0.0556 | 41 | 127 | 138 | SEDRNIDDAQAK | | | | | Mascot |
| 1385.8362 | 1385.7264 | -0.1098 | -79 | 237 | 248 | IIVSRSEIDLLK | | | | | Mascot |
| 1759.9701 | 1759.8502 | -0.1199 | -68 | 77 | 93 | AAYQQSAGKPLADALKK | | | | | Mascot |
| 1759.9701 | 1759.8502 | -0.1199 | -68 | 77 | 93 | AAYQQSAGKPLADALKK | | | | | Mascot |
| 1900.0637 | 1899.957 | -0.1067 | -56 | 49 | 66 | AIETKGVDEATIIEVLAK | | | | | Mascot |
| 2102.0144 | 2101.9373 | -0.0771 | -37 | 189 | 207 | DLEGELSGHLEDCLMTLVK | | | | | Mascot |

25 zgc:101901 [Danio rerio] gi|157676757 62740 8.22 10 36 0

**Peptide Information**

| Calc. Mass | Obsrv. Mass | ± da | ± ppm | Start Seq. | End Seq. | Sequence | Ion Score | C. I. % | Modification | Rank | Result Type |
|---|---|---|---|---|---|---|---|---|---|---|---|
| 950.4611 | 950.501 | 0.0399 | 42 | 221 | 229 | GSQVTSMPK | | | Oxidation (M)[7] | | Mascot |
| 1308.5923 | 1308.6664 | 0.0741 | 57 | 132 | 142 | MNNNEMVSIPK | | | Oxidation (M)[1,6] | | Mascot |
| 1317.7261 | 1317.7031 | -0.023 | -17 | 392 | 404 | SLPTGTSVITVDK | | | | | Mascot |
| 1381.707 | 1381.6779 | -0.0291 | -21 | 292 | 304 | SDIPINGPPTNTR | | | | | Mascot |

| Calc. Mass | Obsrv. Mass | ± da | ± ppm | Start Seq. | End Seq. | Sequence | Ion Score | C. I. % | Modification | Rank | Result Type |
|---|---|---|---|---|---|---|---|---|---|---|---|
| 1387.61 | 1387.6953 | 0.0853 | 61 | 32 | 42 | YNHQFVDCAYK | | | | | Mascot |
| 1449.7189 | 1449.7637 | 0.0448 | 31 | 221 | 233 | GSQVTSMPKLNCK | | | Carbamidomethyl (C)[12] | | Mascot |
| 1727.7904 | 1727.8553 | 0.0649 | 38 | 321 | 336 | KEEGNYTCSAVNDIGK | | | | | Mascot |
| 2005.0422 | 2005.0021 | -0.0401 | -20 | 230 | 247 | LNCKAPSVSITYQPNIEK | | | | | Mascot |
| 2102.0959 | 2101.9373 | -0.1586 | -75 | 5 | 24 | YLMLIALGTALIGNAHCCP K | | | | | Mascot |
| 2211.1868 | 2211.1282 | -0.0586 | -27 | 43 | 64 | DLVEVPVGLPSNASTLSL SANK | 2 | 0 | | | Mascot |
| 2211.1868 | 2211.1282 | -0.0586 | -27 | 43 | 64 | DLVEVPVGLPSNASTLSL SANK | | | | | Mascot |

| | | | | | | | |
|---|---|---|---|---|---|---|---|
| 26 | novel protein similar to vertebrate kinesin family [Danio rerio] | gi\|123208287 | 41525.2 | 6.07 | 9 | 36 | 0 |

**Peptide Information**

| Calc. Mass | Obsrv. Mass | ± da | ± ppm | Start Seq. | End Seq. | Sequence | Ion Score | C. I. % | Modification | Rank | Result Type |
|---|---|---|---|---|---|---|---|---|---|---|---|
| 842.5709 | 842.5168 | -0.0541 | -64 | 359 | 365 | EVILKLK | | | | | Mascot |
| 850.4716 | 850.5497 | 0.0781 | 92 | 19 | 24 | IRVFCR | | | Carbamidomethyl (C)[5] | | Mascot |
| 1200.6106 | 1200.6678 | 0.0572 | 48 | 364 | 374 | LKSGQPVEEDV | | | | | Mascot |
| 1248.6946 | 1248.6927 | -0.0019 | -2 | 176 | 187 | KGLVFAQGAETK | | | | | Mascot |
| 1317.7572 | 1317.7031 | -0.0541 | -41 | 21 | 30 | VFCRIRPLTR | | | Carbamidomethyl (C)[3] | | Mascot |
| 1443.7373 | 1443.7195 | -0.0178 | -12 | 207 | 219 | HIAATKMNVESSR | | | | | Mascot |
| 1443.7373 | 1443.7195 | -0.0178 | -12 | 207 | 219 | HIAATKMNVESSR | | | | | Mascot |
| 1688.7623 | 1688.9028 | 0.1405 | 83 | 1 | 15 | DPEEEVLQHGGGHER | | | | | Mascot |
| 2040.9735 | 2041.0757 | 0.1022 | 50 | 138 | 153 | FEFKVSAYMLELYNDR | | | Oxidation (M)[9] | | Mascot |
| 2040.9735 | 2041.0757 | 0.1022 | 50 | 138 | 153 | FEFKVSAYMLELYNDR | | | Oxidation (M)[9] | | Mascot |
| 2102.0586 | 2101.9373 | -0.1213 | -58 | 37 | 55 | GHITVACLDDYSVILETPR | | | | | Mascot |

| | | | | | | | |
|---|---|---|---|---|---|---|---|
| 27 | Si:dkeyp-95d10.1 protein [Danio rerio] | gi\|81097645 | 20309.2 | 7.37 | 6 | 36 | 0 |

**Peptide Information**

| Calc. Mass | Obsrv. Mass | ± da | ± ppm | Start Seq. | End Seq. | Sequence | Ion Score | C. I. % | Modification | Rank | Result Type |
|---|---|---|---|---|---|---|---|---|---|---|---|
| 983.5156 | 983.4488 | -0.0668 | -68 | 140 | 148 | HLSGEEAIK | | | | | Mascot |
| 1226.6488 | 1226.5912 | -0.0576 | -47 | 138 | 148 | SRHLSGEEAIK | | | | | Mascot |
| 1256.6377 | 1256.7084 | 0.0707 | 56 | 127 | 137 | TGTMLACYLVK | | | Carbamidomethyl (C)[7] | | Mascot |
| 1256.6377 | 1256.7084 | 0.0707 | 56 | 127 | 137 | TGTMLACYLVK | | | Carbamidomethyl (C)[7] | | Mascot |
| 1381.7434 | 1381.6779 | -0.0655 | -47 | 140 | 151 | HLSGEEAIKEIR | | | | | Mascot |
| 1432.8522 | 1432.7574 | -0.0948 | -66 | 97 | 108 | SQILRFLSIVEK | | | | | Mascot |
| 1432.8522 | 1432.7574 | -0.0948 | -66 | 97 | 108 | SQILRFLSIVEK | | | | | Mascot |
| 1443.6658 | 1443.7195 | 0.0537 | 37 | 113 | 126 | GEGVAVHCAHGHGR | | | Carbamidomethyl (C)[8] | | Mascot |

| | | | | | | | | | | | | |
|---|---|---|---|---|---|---|---|---|---|---|---|---|
| 1443.6658 | 1443.7195 | 0.0537 | 37 | 113 | 126 | GEGVAVHCAHGHGR | | | Carbamidomethyl (C)[8] | | Mascot |

28 PREDICTED: eukaryotic translation initiation factor 5B [Danio rerio] gi|326664222 137612.9 5.61 15 35 0

**Peptide Information**

| Calc. Mass | Obsrv. Mass | ± da | ± ppm | Start Seq. | End Seq. | Sequence | Ion Score | C. I. % | Modification | Rank | Result Type |
|---|---|---|---|---|---|---|---|---|---|---|---|
| 842.4842 | 842.5168 | 0.0326 | 39 | 345 | 352 | GPNKATVR | | | | | Mascot |
| 915.5509 | 915.4971 | -0.0538 | -59 | 942 | 949 | ILGKDLEK | | | | | Mascot |
| 950.4247 | 950.501 | 0.0763 | 80 | 361 | 367 | MKEEEER | | | | | Mascot |
| 987.5105 | 987.5178 | 0.0073 | 7 | 379 | 386 | LEELEAQR | | | | | Mascot |
| 1079.5327 | 1079.5217 | -0.011 | -10 | 580 | 589 | ESSNKTTANK | | | | | Mascot |
| 1226.674 | 1226.5912 | -0.0828 | -67 | 435 | 446 | ALQAQGVEVPSK | | | | | Mascot |
| 1256.6416 | 1256.7084 | 0.0668 | 53 | 931 | 941 | HKEVCTAQGVK | | | Carbamidomethyl (C)[5] | | Mascot |
| 1256.6416 | 1256.7084 | 0.0668 | 53 | 931 | 941 | HKEVCTAQGVK | | | Carbamidomethyl (C)[5] | | Mascot |
| 1345.7507 | 1345.6891 | -0.0616 | -46 | 933 | 945 | EVCTAQGVKILGK | | | | | Mascot |
| 1377.7274 | 1377.684 | -0.0434 | -32 | 1198 | 1208 | QSIDALKNWFR | | | | | Mascot |
| 1394.746 | 1394.7837 | 0.0377 | 27 | 640 | 653 | APVVCVLGHVDTGK | | | | | Mascot |
| 1727.9326 | 1727.8553 | -0.0773 | -45 | 1140 | 1155 | GFVDIGIVTSIEVNHK | | | | | Mascot |
| 1915 | 1914.9497 | -0.0503 | -26 | 1065 | 1080 | IFSAEIIYHLFDAFTK | | | | | Mascot |
| 2102.0247 | 2101.9373 | -0.0874 | -42 | 47 | 67 | DELDALAAEIEGAGAGKEQSK | | | | | Mascot |
| 2118.9309 | 2118.9619 | 0.031 | 15 | 303 | 323 | AASLDLDGADVQGEEEAEGDK | | | | | Mascot |
| 2118.9309 | 2118.9619 | 0.031 | 15 | 303 | 323 | AASLDLDGADVQGEEEAEGDK | | | | | Mascot |
| 2268.9636 | 2269.0652 | 0.1016 | 45 | 505 | 524 | AEAALDDWEAMASDEEKEMK | | | | | Mascot |

29 coiled-coil domain-containing protein 123, mitochondrial precursor [Danio rerio] gi|41053710 79256.9 5.96 11 35 0

**Peptide Information**

| Calc. Mass | Obsrv. Mass | ± da | ± ppm | Start Seq. | End Seq. | Sequence | Ion Score | C. I. % | Modification | Rank | Result Type |
|---|---|---|---|---|---|---|---|---|---|---|---|
| 1124.6534 | 1124.6771 | 0.0237 | 21 | 69 | 78 | TVAIPSPRQR | | | | | Mascot |
| 1248.5889 | 1248.6927 | 0.1038 | 83 | 566 | 575 | TCEVEDLQVR | | | Carbamidomethyl (C)[2] | | Mascot |
| 1317.5488 | 1317.7031 | 0.1543 | 117 | 77 | 87 | QRSYSESDCSR | | | | | Mascot |
| 1443.7438 | 1443.7195 | -0.0243 | -17 | 430 | 441 | VRAEEVIQENEK | | | | | Mascot |
| 1443.7438 | 1443.7195 | -0.0243 | -17 | 430 | 441 | VRAEEVIQENEK | | | | | Mascot |
| 1448.7162 | 1448.7517 | 0.0355 | 25 | 420 | 431 | SCEEELQSLRVR | | | | | Mascot |
| 1728.0013 | 1727.8553 | -0.146 | -84 | 668 | 683 | QGVLTRIIESTVNLGK | | | | | Mascot |

| Calc. Mass | Obsrv. Mass | ± da | ± ppm | Start Seq. | End Seq. | Sequence | Ion Score | C. I. % | Modification | Rank | Result Type |
|---|---|---|---|---|---|---|---|---|---|---|---|
| 1743.8456 | 1743.854 | 0.0084 | 5 | 313 | 325 | HLQQELCHTHRER | | | Carbamidomethyl (C)[7] | | Mascot |
| 1743.8456 | 1743.854 | 0.0084 | 5 | 313 | 325 | HLQQELCHTHRER | | | Carbamidomethyl (C)[7] | | Mascot |
| 2103.1121 | 2102.957 | -0.1551 | -74 | 399 | 416 | YLSPLLLAYEDHLNAKDK | | | | | Mascot |
| 2146.0808 | 2146.041 | -0.0398 | -19 | 566 | 583 | TCEVEDLQVRLSALEVER | | | Carbamidomethyl (C)[2] | | Mascot |
| 2211.0823 | 2211.1282 | 0.0459 | 21 | 340 | 358 | QQAQELVDENDGLKMTVHR | | | | | Mascot |
| 2211.0823 | 2211.1282 | 0.0459 | 21 | 340 | 358 | QQAQELVDENDGLKMTVHR | | | | | Mascot |
| 2233.2188 | 2233.1072 | -0.1116 | -50 | 536 | 555 | LALEHSLSLAEHQTVTDKLK | | | | | Mascot |

| | | | | | | | | | |
|---|---|---|---|---|---|---|---|---|---|
| 30 | PREDICTED: transient receptor potential cation channel subfamily M member 4-like [Danio rerio] | gi\|326666046 | 137203 | 6.89 | 10 | 34 | 0 | 21 | 0 |

**Peptide Information**

| Calc. Mass | Obsrv. Mass | ± da | ± ppm | Start Seq. | End Seq. | Sequence | Ion Score | C. I. % | Modification | Rank | Result Type |
|---|---|---|---|---|---|---|---|---|---|---|---|
| 827.4556 | 827.4365 | -0.0191 | -23 | 591 | 597 | AMKHVNK | | | | | Mascot |
| 950.4677 | 950.501 | 0.0333 | 35 | 657 | 664 | LETETETK | | | | | Mascot |
| 1165.7667 | 1165.7545 | -0.0122 | -10 | 910 | 920 | QLGPKIIIVGK | | | | | Mascot |
| 1200.6372 | 1200.6678 | 0.0306 | 25 | 21 | 30 | AQKDQSWIPK | | | | | Mascot |
| 1317.6758 | 1317.7031 | 0.0273 | 21 | 541 | 553 | SQATNLTTPSAAR | | | | | Mascot |
| 1370.6304 | 1370.6625 | 0.0321 | 23 | 184 | 196 | HHCAMASSLSQAK | | | | | Mascot |
| 1432.7828 | 1432.7574 | -0.0254 | -18 | 1141 | 1152 | VDSVLKQMVEIR | | | Oxidation (M)[8] | | Mascot |
| 1432.7828 | 1432.7574 | -0.0254 | -18 | 1141 | 1152 | VDSVLKQMVEIR | 21 | 0 | Oxidation (M)[8] | | Mascot |
| 1443.6467 | 1443.7195 | 0.0728 | 50 | 184 | 196 | HHCAMASSLSQAK | | | Carbamidomethyl (C)[3], Oxidation (M)[5] | | Mascot |
| 1443.6467 | 1443.7195 | 0.0728 | 50 | 184 | 196 | HHCAMASSLSQAK | | | Carbamidomethyl (C)[3], Oxidation (M)[5] | | Mascot |
| 1899.9712 | 1899.957 | -0.0142 | -7 | 441 | 456 | VDIAKTELFNGDVHWR | | | | | Mascot |
| 2145.9724 | 2146.041 | 0.0686 | 32 | 78 | 97 | DTSDYPTDAFGQLEFVGAGR | | | | | Mascot |
| 2434.0366 | 2434.1011 | 0.0645 | 26 | 35 | 57 | RVCTTFVEDSCSNGALCQCGGAR | | | Carbamidomethyl (C)[3] | | Mascot |

| | | | | | | | |
|---|---|---|---|---|---|---|---|
| 31 | LOC561115 protein [Danio rerio] | gi\|124481604 | 94397 | 5.22 | 12 | 34 | 0 |

**Peptide Information**

| Calc. Mass | Obsrv. Mass | ± da | ± ppm | Start Seq. | End Seq. | Sequence | Ion Score | C. I. % | Modification | Rank | Result Type |
|---|---|---|---|---|---|---|---|---|---|---|---|
| 996.5108 | 996.6054 | 0.0946 | 95 | 149 | 157 | AVEENAHVK | | | | | Mascot |
| 1200.6218 | 1200.6678 | 0.046 | 38 | 396 | 406 | KVASEEDPGIR | | | | | Mascot |
| 1256.5793 | 1256.7084 | 0.1291 | 103 | 670 | 679 | QLEDYNFAEK | | | | | Mascot |
| 1256.5793 | 1256.7084 | 0.1291 | 103 | 670 | 679 | QLEDYNFAEK | | | | | Mascot |
| 1361.692 | 1361.6848 | -0.0072 | -5 | 513 | 523 | NTLLINHHDER | | | | | Mascot |

| Calc. Mass | Obsrv. Mass | ± da | ± ppm | Start Seq. | End Seq. | Sequence | Ion Score | C. I. % | Modification | Rank | Result Type |
|---|---|---|---|---|---|---|---|---|---|---|---|
| 1377.6216 | 1377.684 | 0.0624 | 45 | 548 | 558 | QSHEWTSLMSR | | | Oxidation (M)[9] | | Mascot |
| 1443.662 | 1443.7195 | 0.0575 | 40 | 354 | 364 | RQYCHYLSSMR | | | | | Mascot |
| 1443.662 | 1443.7195 | 0.0575 | 40 | 354 | 364 | RQYCHYLSSMR | | | | | Mascot |
| 1914.8949 | 1914.9497 | 0.0548 | 29 | 707 | 722 | ENAVYVICHNPMSPQR | | | Carbamidomethyl (C)[8] | | Mascot |
| 2040.9297 | 2041.0757 | 0.146 | 72 | 25 | 41 | SWETSLSAAVFEENEWR | | | | | Mascot |
| 2040.9297 | 2041.0757 | 0.146 | 72 | 25 | 41 | SWETSLSAAVFEENEWR | | | | | Mascot |
| 2211.0872 | 2211.1282 | 0.041 | 19 | 355 | 373 | QYCHYLSSMRLIQVPAACK | | | | | Mascot |
| 2211.0872 | 2211.1282 | 0.041 | 19 | 355 | 373 | QYCHYLSSMRLIQVPAACK | | | | | Mascot |
| 2233.031 | 2233.1072 | 0.0762 | 34 | 707 | 725 | ENAVYVICHNPMSPQRSCK | | | Carbamidomethyl (C)[8] | | Mascot |
| 2269.1531 | 2269.0652 | -0.0879 | -39 | 374 | 394 | SGSPIQQSRPQSSTEVVQTPR | | | | | Mascot |
| 2284.1714 | 2284.1965 | 0.0251 | 11 | 52 | 71 | AEDEQLIGSLVQAVQQPMRR | | | Oxidation (M)[18] | | Mascot |

32 39S ribosomal protein L30, mitochondrial [Danio rerio] gi|66472758 17600.6 10.15 6 34 0

**Peptide Information**

| Calc. Mass | Obsrv. Mass | ± da | ± ppm | Start Seq. | End Seq. | Sequence | Ion Score | C. I. % | Modification | Rank | Result Type |
|---|---|---|---|---|---|---|---|---|---|---|---|
| 996.6564 | 996.6054 | -0.051 | -51 | 142 | 149 | RLLKPLEK | | | | | Mascot |
| 1006.5468 | 1006.527 | -0.0198 | -20 | 71 | 77 | RPYWEKK | | | | | Mascot |
| 1270.6572 | 1270.7366 | 0.0794 | 62 | 81 | 91 | SLGLMKSHEPR | | | Oxidation (M)[5] | | Mascot |
| 1846.9558 | 1846.8077 | -0.1481 | -80 | 49 | 64 | YGGDPEQPHKLHIVTR | | | | | Mascot |
| 2102.0083 | 2101.9373 | -0.071 | -34 | 117 | 135 | LPHGLPAEEDMANTHLNSR | | | | | Mascot |
| 2146.1284 | 2146.041 | -0.0874 | -41 | 7 | 26 | ALLTSASNVSKNLTQVSPCR | | | Carbamidomethyl (C)[19] | | Mascot |

33 novel protein similar to elongation factor RNA polymerase II (ell) [Danio rerio] gi|122890999 34843 9.64 8 34 0

**Peptide Information**

| Calc. Mass | Obsrv. Mass | ± da | ± ppm | Start Seq. | End Seq. | Sequence | Ion Score | C. I. % | Modification | Rank | Result Type |
|---|---|---|---|---|---|---|---|---|---|---|---|
| 1006.4985 | 1006.527 | 0.0285 | 28 | 134 | 141 | ERMSQVEK | | | | | Mascot |
| 1079.5731 | 1079.5217 | -0.0514 | -48 | 38 | 46 | TLEAYQNLK | | | | | Mascot |
| 1256.6732 | 1256.7084 | 0.0352 | 28 | 231 | 242 | ADLSPVLDEVAK | | | | | Mascot |
| 1256.6732 | 1256.7084 | 0.0352 | 28 | 231 | 242 | ADLSPVLDEVAK | | | | | Mascot |
| 1277.6055 | 1277.7234 | 0.1179 | 92 | 6 | 16 | QEHRYGLSCGK | | | | | Mascot |
| 1277.6055 | 1277.7234 | 0.1179 | 92 | 6 | 16 | QEHRYGLSCGK | | | | | Mascot |
| 1345.6707 | 1345.6891 | 0.0184 | 14 | 108 | 119 | DRLEGQGSIQDK | | | | | Mascot |
| 1394.6733 | 1394.7837 | 0.1104 | 79 | 136 | 146 | MSQVEKDIWSR | | | Oxidation (M)[1] | | Mascot |

| | | | | | | | | | | | | |
|---|---|---|---|---|---|---|---|---|---|---|---|---|
| | 1448.7592 | 1448.7517 | -0.0075 | -5 | 283 | 296 | LQPLSSSSSSQLK | | | | | Mascot |
| | 1688.9303 | 1688.9028 | -0.0275 | -16 | 187 | 201 | NGGPSLVTHRPLRER | | | | | Mascot |

| | | | | | | | | |
|---|---|---|---|---|---|---|---|---|
| 34 | adenylate kinase 7 [Danio rerio] | gi\|289191303 | 79498.9 | 5.01 | 11 | 33 | 0 | |

Peptide Information

| Calc. Mass | Obsrv. Mass | ± da | ± ppm | Start Seq. | End Seq. | Sequence | Ion Score | C. I. % | Modification | Rank | Result Type |
|---|---|---|---|---|---|---|---|---|---|---|---|
| 850.5145 | 850.5497 | 0.0352 | 41 | 256 | 262 | IIDHKPK | | | | | Mascot |
| 950.4941 | 950.501 | 0.0069 | 7 | 487 | 494 | ATEPKYNK | | | | | Mascot |
| 1051.5908 | 1051.7223 | 0.1315 | 125 | 171 | 178 | RRPHPNFK | | | | | Mascot |
| 1124.6497 | 1124.6771 | 0.0274 | 24 | 359 | 369 | ICLLGPPAVGK | | | Carbamidomethyl (C)[2] | | Mascot |
| 1277.6493 | 1277.7234 | 0.0741 | 58 | 651 | 660 | SVPMRHYLMK | | | Oxidation (M)[4] | | Mascot |
| 1277.6493 | 1277.7234 | 0.0741 | 58 | 651 | 660 | SVPMRHYLMK | | | Oxidation (M)[4] | | Mascot |
| 1387.7362 | 1387.6953 | -0.0409 | -29 | 515 | 526 | VRSLPQNVAEMK | | | Oxidation (M)[11] | | Mascot |
| 1689.0494 | 1688.9028 | -0.1466 | -87 | 354 | 369 | LLPIKICLLGPPAVGK | | | Carbamidomethyl (C)[7] | | Mascot |
| 1727.8165 | 1727.8553 | 0.0388 | 22 | 661 | 675 | YVMPTVVQGLVDCCK | | | Carbamidomethyl (C)[13], Oxidation (M)[3] | | Mascot |
| 2017.9283 | 2018.1835 | 0.2552 | 126 | 57 | 75 | HDASSDEPCFQIVGTVAN K | | | | | Mascot |
| 2103.0354 | 2102.957 | -0.0784 | -37 | 495 | 512 | VMIPEFVFSLDATDDFLK | | | Oxidation (M)[2] | | Mascot |
| 2118.9404 | 2118.9619 | 0.0215 | 10 | 1 | 18 | MNDLHSNQTTNMANSER R | | | | | Mascot |
| 2118.9404 | 2118.9619 | 0.0215 | 10 | 1 | 18 | MNDLHSNQTTNMANSER R | | | | | Mascot |

| | | | | | | | | |
|---|---|---|---|---|---|---|---|---|
| 35 | annexin A1c [Danio rerio] | gi\|32308151 | 37637.3 | 5.39 | 8 | 33 | 0 | |

Peptide Information

| Calc. Mass | Obsrv. Mass | ± da | ± ppm | Start Seq. | End Seq. | Sequence | Ion Score | C. I. % | Modification | Rank | Result Type |
|---|---|---|---|---|---|---|---|---|---|---|---|
| 827.3967 | 827.4365 | 0.0398 | 48 | 306 | 311 | IMQEYK | | | Oxidation (M)[2] | | Mascot |
| 983.4978 | 983.4488 | -0.049 | -50 | 306 | 312 | IMQEYKR | | | Oxidation (M)[2] | | Mascot |
| 1006.4952 | 1006.527 | 0.0318 | 32 | 200 | 208 | ALFEAGENR | | | | | Mascot |
| 1361.6292 | 1361.6848 | 0.0556 | 41 | 184 | 195 | SEDRNIDDAQAK | | | | | Mascot |
| 1385.8362 | 1385.7264 | -0.1098 | -79 | 294 | 305 | IIVSRSEIDLLK | | | | | Mascot |
| 1759.9701 | 1759.8502 | -0.1199 | -68 | 77 | 93 | AAYQQSAGKPLADALKK | | | | | Mascot |
| 1759.9701 | 1759.8502 | -0.1199 | -68 | 77 | 93 | AAYQQSAGKPLADALKK | | | | | Mascot |
| 1900.0637 | 1899.957 | -0.1067 | -56 | 49 | 66 | AIETKGVDEATIIEVLAK | | | | | Mascot |
| 2102.0144 | 2101.9373 | -0.0771 | -37 | 246 | 264 | DLEGELSGHLEDCLMTLV K | | | | | Mascot |

| | | | | | | | | |
|---|---|---|---|---|---|---|---|---|
| 36 | novel protein similar to human and mouse ring finger protein 30 (RNF30) [Danio rerio] | gi\|56207578 | 13788 | 5.17 | 6 | 33 | 0 | |

Peptide Information

| Calc. Mass | Obsrv. Mass | ± da | ± ppm | Start Seq. | End Seq. | Sequence | Ion Score | C. I. % | Modification | Rank | Result Type |
|---|---|---|---|---|---|---|---|---|---|---|---|
| 1124.6171 | 1124.6771 | 0.06 | 53 | 56 | 64 | RQIQHISDK | | | | | Mascot |
| 1308.6477 | 1308.6664 | 0.0187 | 14 | 100 | 110 | RHGDHLEMSVK | | | | | Mascot |
| 1362.6458 | 1362.7101 | 0.0643 | 47 | 111 | 122 | LVETAIQSMEEP | | | Oxidation (M)[9] | | Mascot |
| 1362.6458 | 1362.7101 | 0.0643 | 47 | 111 | 122 | LVETAIQSMEEP | | | Oxidation (M)[9] | | Mascot |
| 1448.6686 | 1448.7517 | 0.0831 | 57 | 78 | 89 | ELIETVGCEQDR | | | Carbamidomethyl (C)[8] | | Mascot |
| 1914.9043 | 1914.9497 | 0.0454 | 24 | 2 | 19 | CTVDTICPSVICSVSMLK | | | Oxidation (M)[16] | | Mascot |
| 2085.9688 | 2085.9387 | -0.0301 | -14 | 2 | 19 | CTVDTICPSVICSVSMLK | | | Carbamidomethyl (C)[1,7,12], Oxidation (M)[16] | | Mascot |
| 2102.9663 | 2102.957 | -0.0093 | -4 | 1 | 19 | MCTVDTICPSVICSVSML K | | | Carbamidomethyl (C)[2], Oxidation (M)[1] | | Mascot |
| 2118.9612 | 2118.9619 | 0.0007 | 0 | 1 | 19 | MCTVDTICPSVICSVSML K | | | Carbamidomethyl (C)[2], Oxidation (M)[1,17] | | Mascot |
| 2118.9612 | 2118.9619 | 0.0007 | 0 | 1 | 19 | MCTVDTICPSVICSVSML K | | | Carbamidomethyl (C)[2], Oxidation (M)[1,17] | | Mascot |
| 2233.0042 | 2233.1072 | 0.103 | 46 | 1 | 19 | MCTVDTICPSVICSVSML K | | | Carbamidomethyl (C)[2,8,13], Oxidation (M)[1,17] | | Mascot |

37 Npepps protein [Danio rerio] gi|33585611 19640.3 8.56 6 33 0

Peptide Information

| Calc. Mass | Obsrv. Mass | ± da | ± ppm | Start Seq. | End Seq. | Sequence | Ion Score | C. I. % | Modification | Rank | Result Type |
|---|---|---|---|---|---|---|---|---|---|---|---|
| 1248.5637 | 1248.6927 | 0.129 | 103 | 26 | 35 | QADMQEEKNR | | | | | Mascot |
| 1362.8103 | 1362.7101 | -0.1002 | -74 | 39 | 51 | VLGAIPAPDLIQR | | | | | Mascot |
| 1362.8103 | 1362.7101 | -0.1002 | -74 | 39 | 51 | VLGAIPAPDLIQR | | | | | Mascot |
| 1394.8154 | 1394.7837 | -0.0317 | -23 | 99 | 110 | YQGGFLISRLIK | | | | | Mascot |
| 1432.8409 | 1432.7574 | -0.0835 | -58 | 108 | 120 | LIKLTVDGFAIDK | | | | | Mascot |
| 1432.8409 | 1432.7574 | -0.0835 | -58 | 108 | 120 | LIKLTVDGFAIDK | | | | | Mascot |
| 1486.7285 | 1486.728 | -0.0005 | 0 | 158 | 169 | DADDIHQYLLQR | | | | | Mascot |
| 2103.0837 | 2102.957 | -0.1267 | -60 | 140 | 157 | TVQQCCENILLNAAWLKR | | | | | Mascot |

38 cyclin-F [Danio rerio] gi|46309465 84852.2 5.63 12 33 0

Peptide Information

| Calc. Mass | Obsrv. Mass | ± da | ± ppm | Start Seq. | End Seq. | Sequence | Ion Score | C. I. % | Modification | Rank | Result Type |
|---|---|---|---|---|---|---|---|---|---|---|---|
| 915.4101 | 915.4971 | 0.087 | 95 | 576 | 583 | ADSMQAHR | | | | | Mascot |
| 950.4399 | 950.501 | 0.0611 | 64 | 495 | 502 | CFGQDVPK | | | Carbamidomethyl (C)[1] | | Mascot |
| 1006.5502 | 1006.527 | -0.0232 | -23 | 13 | 20 | CFSLPVRK | | | Carbamidomethyl (C)[1] | | Mascot |
| 1079.5084 | 1079.5217 | 0.0133 | 12 | 123 | 132 | ADLCGRMASR | | | | | Mascot |

| Calc. Mass | Obsrv. Mass | ± da | ± ppm | Start Seq. | End Seq. | Sequence | Ion Score | C. I. % | Modification | Rank | Result Type |
|---|---|---|---|---|---|---|---|---|---|---|---|
| 1124.6534 | 1124.6771 | 0.0237 | 21 | 506 | 515 | HVSLTGVKQR | | | | | Mascot |
| 1256.6238 | 1256.7084 | 0.0846 | 67 | 240 | 248 | YLQCMRTLR | | | Carbamidomethyl (C)[4], Oxidation (M)[5] | | Mascot |
| 1256.6238 | 1256.7084 | 0.0846 | 67 | 240 | 248 | YLQCMRTLR | | | Carbamidomethyl (C)[4], Oxidation (M)[5] | | Mascot |
| 1308.6405 | 1308.6664 | 0.0259 | 20 | 254 | 264 | GCWEAQLAFVK | | | Carbamidomethyl (C)[2] | | Mascot |
| 1317.658 | 1317.7031 | 0.0451 | 34 | 172 | 183 | LQAECQTSPGRK | | | | | Mascot |
| 1846.97 | 1846.8077 | -0.1623 | -88 | 349 | 364 | AQLQLLGIACMVICTR | | | Carbamidomethyl (C)[10,14] | | Mascot |
| 1862.9065 | 1862.8049 | -0.1016 | -55 | 324 | 339 | DFSSQVLHVTISCVDR | | | Carbamidomethyl (C)[13] | | Mascot |
| 2283.1689 | 2283.1985 | 0.0296 | 13 | 184 | 202 | GTLLYCLARVLQLFDDEE K | | | Carbamidomethyl (C)[6] | | Mascot |
| 2283.9778 | 2284.1965 | 0.2187 | 96 | 628 | 647 | DGEISTMEVQMELPLDC ADR | | | Oxidation (M)[7,11] | | Mascot |

39 protein BTG3 [Danio rerio] gi|55925203 18115.1 5.85 6 32 0

**Peptide Information**

| Calc. Mass | Obsrv. Mass | ± da | ± ppm | Start Seq. | End Seq. | Sequence | Ion Score | C. I. % | Modification | Rank | Result Type |
|---|---|---|---|---|---|---|---|---|---|---|---|
| 950.4988 | 950.501 | 0.0022 | 2 | 54 | 61 | GQAFRCIR | | | | | Mascot |
| 1200.5432 | 1200.6678 | 0.1246 | 104 | 44 | 53 | GHWYPDNPSK | | | | | Mascot |
| 1248.6583 | 1248.6927 | 0.0344 | 28 | 65 | 74 | FQKEDAELLR | | | | | Mascot |
| 1759.83 | 1759.8502 | 0.0202 | 11 | 44 | 58 | GHWYPDNPSKGQAFR | | | | | Mascot |
| 1759.83 | 1759.8502 | 0.0202 | 11 | 44 | 58 | GHWYPDNPSKGQAFR | 4 | 0 | | | Mascot |
| 2211.0208 | 2211.1282 | 0.1074 | 49 | 91 | 108 | ELTLWVDPGEVCCRYGE K | | | Carbamidomethyl (C)[12,13] | | Mascot |
| 2211.0208 | 2211.1282 | 0.1074 | 49 | 91 | 108 | ELTLWVDPGEVCCRYGE K | | | Carbamidomethyl (C)[12,13] | | Mascot |
| 2224.9412 | 2225.1394 | 0.1982 | 89 | 139 | 158 | VTSDYHSGSSSDEDSML VHR | | | Oxidation (M)[16] | | Mascot |

40 PREDICTED: high affinity cGMP-specific 3',5'-cyclic phosphodiesterase 9A-like [Danio rerio] gi|326668155 59790.4 5.68 11 32 0

**Peptide Information**

| Calc. Mass | Obsrv. Mass | ± da | ± ppm | Start Seq. | End Seq. | Sequence | Ion Score | C. I. % | Modification | Rank | Result Type |
|---|---|---|---|---|---|---|---|---|---|---|---|
| 987.4135 | 987.5178 | 0.1043 | 106 | 29 | 36 | HCSPCDIR | | | Carbamidomethyl (C)[2] | | Mascot |
| 1045.5312 | 1045.5718 | 0.0406 | 39 | 368 | 376 | HGEILDSFK | | | | | Mascot |
| 1270.5767 | 1270.7366 | 0.1599 | 126 | 148 | 158 | LKDEMTSGSMR | | | Oxidation (M)[5] | | Mascot |
| 1362.5955 | 1362.7101 | 0.1146 | 84 | 78 | 89 | VMSHSTNQGEEK | | | Oxidation (M)[2] | | Mascot |
| 1362.7198 | 1362.7101 | -0.0097 | -7 | 227 | 237 | EFNMNPITLKR | | | | | Mascot |
| 1377.6678 | 1377.684 | 0.0162 | 12 | 495 | 506 | QIEDAMSEAQKK | | | | | Mascot |
| 1381.7209 | 1381.6779 | -0.043 | -31 | 186 | 197 | YTLSQETIDALK | | | | | Mascot |
| 1486.7684 | 1486.728 | -0.0404 | -27 | 459 | 470 | FVLIPMFETVMK | | | Oxidation (M)[6,11] | | Mascot |

| | | | | | | | | | | | |
|---|---|---|---|---|---|---|---|---|---|---|---|
| 1862.8337 | 1862.8049 | -0.0288 | -15 | 377 | 391 | QKVDNFDCTNEEHVK | | | Carbamidomethyl (C)[8] | | Mascot |
| 1915.1045 | 1914.9497 | -0.1548 | -81 | 351 | 367 | QIRQAIITLILATDMAK | | | Oxidation (M)[15] | | Mascot |
| 2004.9761 | 2005.0021 | 0.026 | 13 | 2 | 20 | GSSSSSYTPKTIYLDVDGK | | | | | Mascot |

| | | | | | | | | |
|---|---|---|---|---|---|---|---|---|
| 41 | PREDICTED: MAGUK p55 subfamily member 6 isoform 1 [Danio rerio] | gi\|326674868 | 60958.3 | 6.56 | 10 | 32 | 0 | |

**Peptide Information**

| Calc. Mass | Obsrv. Mass | ± da | ± ppm | Start Seq. | End Seq. | Sequence | Ion Score | C. I. % | Modification | Rank | Result Type |
|---|---|---|---|---|---|---|---|---|---|---|---|
| 915.4642 | 915.4971 | 0.0329 | 36 | 380 | 386 | RPRDDEK | | | | | Mascot |
| 950.4611 | 950.501 | 0.0399 | 42 | 200 | 208 | DCNGSITLK | | | | | Mascot |
| 987.5217 | 987.5178 | -0.0039 | -4 | 181 | 189 | EVNGREVGK | | | | | Mascot |
| 1370.5927 | 1370.6625 | 0.0698 | 51 | 397 | 407 | EEMEMDIKASR | | | Oxidation (M)[3,5] | | Mascot |
| 1385.6954 | 1385.7264 | 0.031 | 22 | 423 | 434 | MDSIHEVVRAGR | | | Oxidation (M)[1] | | Mascot |
| 1449.7253 | 1449.7637 | 0.0384 | 26 | 293 | 307 | DLDGSGILCGTLTGK | | | | | Mascot |
| 2005.0382 | 2005.0021 | -0.0361 | -18 | 2 | 20 | TVSNAKSGTAMQQVLDNLK | | | | | Mascot |
| 2211.083 | 2211.1282 | 0.0452 | 20 | 502 | 519 | RAYNHYFDLTIVNDNLDK | | | | | Mascot |
| 2211.083 | 2211.1282 | 0.0452 | 20 | 502 | 519 | RAYNHYFDLTIVNDNLDK | | | | | Mascot |
| 2225.1746 | 2225.1394 | -0.0352 | -16 | 30 | 48 | DIDLIFLRGIMESPIAHER | | | | | Mascot |
| 2283.1729 | 2283.1985 | 0.0256 | 11 | 450 | 469 | TSEFMPFVVFIAAPELDTLR | | | | | Mascot |

| | | | | | | | | |
|---|---|---|---|---|---|---|---|---|
| 42 | hypothetical protein LOC450015 [Danio rerio] | gi\|54400574 | 36312.7 | 5.75 | 7 | 31 | 0 | |

**Peptide Information**

| Calc. Mass | Obsrv. Mass | ± da | ± ppm | Start Seq. | End Seq. | Sequence | Ion Score | C. I. % | Modification | Rank | Result Type |
|---|---|---|---|---|---|---|---|---|---|---|---|
| 827.4045 | 827.4365 | 0.032 | 39 | 258 | 263 | FNQQYK | | | | | Mascot |
| 842.4366 | 842.5168 | 0.0802 | 95 | 148 | 155 | TVTEAGHK | | | | | Mascot |
| 915.4907 | 915.4971 | 0.0064 | 7 | 281 | 287 | FVHSRNR | | | | | Mascot |
| 983.5018 | 983.4488 | -0.053 | -54 | 250 | 257 | MFGPFKEK | | | | | Mascot |
| 1277.6848 | 1277.7234 | 0.0386 | 30 | 288 | 299 | VGLSFSLGINDR | | | | | Mascot |
| 1277.6848 | 1277.7234 | 0.0386 | 30 | 288 | 299 | VGLSFSLGINDR | | | | | Mascot |
| 1345.6495 | 1345.6891 | 0.0396 | 29 | 70 | 80 | SRIDYYNGTTR | | | | | Mascot |
| 1743.8483 | 1743.854 | 0.0057 | 3 | 140 | 155 | GAQCEVWKTVTEAGHK | | | | | Mascot |
| 1743.8483 | 1743.854 | 0.0057 | 3 | 140 | 155 | GAQCEVWKTVTEAGHK | | | | | Mascot |

| | | | | | | | | |
|---|---|---|---|---|---|---|---|---|
| 43 | novel protein similar to vertebrate breast cancer anti-estrogen resistance 3 (BCAR3) [Danio rerio] | gi\|169158144 | 84068.2 | 8.52 | 12 | 31 | 0 | |

Peptide Information

| Calc. Mass | Obsrv. Mass | ± da | ± ppm | Start Seq. | End Seq. | Sequence | Ion Score | C. I. % | Modification | Rank | Result Type |
|---|---|---|---|---|---|---|---|---|---|---|---|
| 882.5083 | 882.5741 | 0.0658 | 75 | 607 | 613 | SLKPFYK | | | | | Mascot |
| 1045.5524 | 1045.5718 | 0.0194 | 19 | 45 | 52 | RELEEELK | | | | | Mascot |
| 1051.5895 | 1051.7223 | 0.1328 | 126 | 22 | 31 | SSVIGAKFSR | | | | | Mascot |
| 1079.6068 | 1079.5217 | -0.0851 | -79 | 665 | 674 | HLEGARAVAR | | | | | Mascot |
| 1256.5325 | 1256.7084 | 0.1759 | 140 | 675 | 685 | NADTYTCNAQR | | | | | Mascot |
| 1256.5325 | 1256.7084 | 0.1759 | 140 | 675 | 685 | NADTYTCNAQR | | | | | Mascot |
| 1294.6439 | 1294.7118 | 0.0679 | 52 | 60 | 70 | SHAWYHGAIPR | | | | | Mascot |
| 1317.7083 | 1317.7031 | -0.0052 | -4 | 452 | 462 | ENKPLEMTVLK | | | Oxidation (M)[7] | | Mascot |
| 1361.6592 | 1361.6848 | 0.0256 | 19 | 10 | 21 | TITAALCCFYQK | | | | | Mascot |
| 1381.6528 | 1381.6779 | 0.0251 | 18 | 374 | 385 | MNSLQANGYVER | | | | | Mascot |
| 1449.861 | 1449.7637 | -0.0973 | -67 | 317 | 329 | VPTVRVPCIPALK | | | Carbamidomethyl (C)[8] | | Mascot |
| 1476.7627 | 1476.7306 | -0.0321 | -22 | 288 | 300 | RMAFDTLPGEAIR | | | | | Mascot |
| 2018.002 | 2018.1835 | 0.1815 | 90 | 5 | 21 | CNILRTITAALCCFYQK | | | Carbamidomethyl (C)[1] | | Mascot |

| | | | | | | | | |
|---|---|---|---|---|---|---|---|---|
| 44 | PREDICTED: neurabin-1-like [Danio rerio] | gi\|326670455 | 143069.6 | 4.9 | 1 | 26 | 28.083 | |

Peptide Information

| Calc. Mass | Obsrv. Mass | ± da | ± ppm | Start Seq. | End Seq. | Sequence | Ion Score | C. I. % | Modification | Rank | Result Type |
|---|---|---|---|---|---|---|---|---|---|---|---|
| 1362.614 | 1362.7101 | 0.0961 | 71 | 371 | 383 | EQCLDPAGAGMVR | 26 | 28.083 | Oxidation (M)[11] | 2 | Mascot |

| | | | | | | | |
|---|---|---|---|---|---|---|---|
| 45 | PREDICTED: insulin receptor substrate 1-B-like [Danio rerio] | gi\|326674337 | 118937.4 | 8.49 | 1 | 22 | 0 |

Peptide Information

| Calc. Mass | Obsrv. Mass | ± da | ± ppm | Start Seq. | End Seq. | Sequence | Ion Score | C. I. % | Modification | Rank | Result Type |
|---|---|---|---|---|---|---|---|---|---|---|---|
| 1743.9421 | 1743.854 | -0.0881 | -51 | 179 | 194 | LNSDAAAVVLQLMNIR | 22 | 0 | Oxidation (M)[13] | 3 | Mascot |

| | | | | | | | |
|---|---|---|---|---|---|---|---|
| 46 | cell adhesion molecule 1a isoform 2 [Danio rerio] | gi\|158538876 | 48182.2 | 4.82 | 1 | 17 | 0 |

Peptide Information

| Calc. Mass | Obsrv. Mass | ± da | ± ppm | Start Seq. | End Seq. | Sequence | Ion Score | C. I. % | Modification | Rank | Result Type |
|---|---|---|---|---|---|---|---|---|---|---|---|
| 1362.6859 | 1362.7101 | 0.0242 | 18 | 107 | 119 | VSLSNVSLSDEGR | 17 | 0 | | 3 | Mascot |

| | | | | | | | |
|---|---|---|---|---|---|---|---|
| 47 | PREDICTED: PH and SEC7 domain-containing protein 4-like [Danio rerio] | gi\|326668322 | 90694.7 | 4.54 | 1 | 17 | 0 |

Peptide Information

| Calc. Mass | Obsrv. Mass | ± da | ± ppm | Start Seq. | End Seq. | Sequence | Ion Score | C. I. % | Modification | Rank | Result Type |
|---|---|---|---|---|---|---|---|---|---|---|---|
| 1743.833 | 1743.854 | 0.021 | 12 | 504 | 518 | SLMPETDREQDGPLR | 10 | 0 | | 8 | Mascot |
| 1759.828 | 1759.8502 | 0.0222 | 13 | 504 | 518 | SLMPETDREQDGPLR | 17 | 0 | Oxidation (M)[3] | 2 | Mascot |

| | | | | | | | | | |
|---|---|---|---|---|---|---|---|---|---|
| 48 | novel protein similar to vertebrate spectrin repeat containing, nuclear envelope 2 (SYNE2) [Danio rerio] | gi\|56207727 | 115485.2 | 5.17 | 1 | | | 16 | 0 |

Peptide Information

| Calc. Mass | Obsrv. Mass | ± da | ± ppm | Start Seq. | End Seq. | Sequence | Ion Score | C. I. % | Modification | Rank | Result Type |
|---|---|---|---|---|---|---|---|---|---|---|---|
| 1256.6705 | 1256.7084 | 0.0379 | 30 | 229 | 238 | RLAEQQDLQR | 16 | 0 | | 2 | Mascot |

| | | | | | | | | | |
|---|---|---|---|---|---|---|---|---|---|
| 49 | calcium-binding protein 1 [Danio rerio] | gi\|50539888 | 19114.3 | 4.69 | 1 | | | 16 | 0 |

Peptide Information

| Calc. Mass | Obsrv. Mass | ± da | ± ppm | Start Seq. | End Seq. | Sequence | Ion Score | C. I. % | Modification | Rank | Result Type |
|---|---|---|---|---|---|---|---|---|---|---|---|
| 1432.6737 | 1432.7574 | 0.0837 | 58 | 18 | 28 | ELRPEEMDELR | 16 | 0 | Oxidation (M)[7] | 4 | Mascot |

| | | | | | | | | | |
|---|---|---|---|---|---|---|---|---|---|
| 50 | hypothetical protein LOC556137 [Danio rerio] | gi\|113677313 | 19802 | 5.55 | 1 | | | 15 | 0 |

Peptide Information

| Calc. Mass | Obsrv. Mass | ± da | ± ppm | Start Seq. | End Seq. | Sequence | Ion Score | C. I. % | Modification | Rank | Result Type |
|---|---|---|---|---|---|---|---|---|---|---|---|
| 1759.811 | 1759.8502 | 0.0392 | 22 | 2 | 16 | LLDYCCRCCGTALIR | 15 | 0 | Carbamidomethyl (C)[5] | 3 | Mascot |

| | | | | | |
|---|---|---|---|---|---|
| **Gel Idx/Pos** | 195/H22 | **Instr./Gel Origin** | BA2060/110707A | **Process Status** | Analysis Succeeded |
| **Plate [#] Name** | [1] 31496 | **Instrument Sample Name** | | **Spectra** | 11 |

| Rank | Protein Name | Accession No. | Protein MW | Protein PI | Pep. Count | Protein Score | Protein Score C. I. % | Total Ion Score | Total Ion C. I. % |
|---|---|---|---|---|---|---|---|---|---|
| 1 | aldehyde dehydrogenase 2b [Danio rerio] | gi\|51571951 | 56513.4 | 6.06 | 24 | 706 | 100 | 520 | 100 |

Peptide Information

| Calc. Mass | Obsrv. Mass | ± da | ± ppm | Start Seq. | End Seq. | Sequence | Ion Score | C. I. % | Modification | Rank | Result Type |
|---|---|---|---|---|---|---|---|---|---|---|---|
| 876.4243 | 876.4282 | 0.0039 | 4 | 107 | 113 | LADCIER | | | Carbamidomethyl (C)[4] | | Mascot |
| 974.5152 | 974.5182 | 0.003 | 3 | 428 | 435 | SLEEVIER | | | | | Mascot |
| 1030.5428 | 1030.5532 | 0.0104 | 10 | 455 | 463 | ANYISHGLR | 55 | 99.909 | | | Mascot |
| 1030.5428 | 1030.5532 | 0.0104 | 10 | 455 | 463 | ANYISHGLR | | | | | Mascot |

| | | | | | | | | | | |
|---|---|---|---|---|---|---|---|---|---|---|
| 1051.6146 | 1051.7194 | 0.1048 | 100 | 368 | 377 | KVLGYISSGK | | | | Mascot |
| 1132.5238 | 1132.5328 | 0.009 | 8 | 383 | 393 | LMCGGAPAAER | | | Carbamidomethyl (C)[3] | Mascot |
| 1148.5187 | 1148.5253 | 0.0066 | 6 | 383 | 393 | LMCGGAPAAER | | | Carbamidomethyl (C)[3], Oxidation (M)[2] | Mascot |
| 1403.7603 | 1403.7678 | 0.0075 | 5 | 414 | 425 | EEIFGPVMQILK | | | | Mascot |
| 1403.7603 | 1403.7678 | 0.0075 | 5 | 414 | 425 | EEIFGPVMQILK | 67 | 99.994 | | Mascot |
| 1419.7552 | 1419.7617 | 0.0065 | 5 | 414 | 425 | EEIFGPVMQILK | | | Oxidation (M)[8] | Mascot |
| 1419.7552 | 1419.7617 | 0.0065 | 5 | 414 | 425 | EEIFGPVMQILK | 44 | 98.843 | Oxidation (M)[8] | Mascot |
| 1460.6984 | 1460.6359 | -0.0625 | -43 | 379 | 393 | EGAKLMCGGAPAAER | | | | Mascot |
| 1470.7627 | 1470.7699 | 0.0072 | 5 | 394 | 406 | GYFIQPTVFGDVK | | | | Mascot |
| 1473.6797 | 1473.6864 | 0.0067 | 5 | 147 | 158 | YYAGWADKWEGK | | | | Mascot |
| 1473.6797 | 1473.6864 | 0.0067 | 5 | 147 | 158 | YYAGWADKWEGK | 44 | 99.001 | | Mascot |
| 1497.7584 | 1497.7661 | 0.0077 | 5 | 441 | 454 | YGLAGAVFTQDIDK | | | | Mascot |
| 1515.8312 | 1515.7649 | -0.0663 | -44 | 101 | 113 | GLLLSRLADCIER | | | Carbamidomethyl (C)[10] | Mascot |
| 1572.7805 | 1572.7897 | 0.0092 | 6 | 38 | 50 | IFINNEWHDAVSK | | | | Mascot |
| 1600.8441 | 1600.8541 | 0.01 | 6 | 257 | 271 | VAFTGSTDVGHLIQR | 123 | 100 | | Mascot |
| 1600.8441 | 1600.8541 | 0.01 | 6 | 257 | 271 | VAFTGSTDVGHLIQR | | | | Mascot |
| 1619.7522 | 1619.7637 | 0.0115 | 7 | 159 | 171 | TIPIDGNYFCYTR | 101 | 100 | Carbamidomethyl (C)[10] | Mascot |
| 1619.7522 | 1619.7637 | 0.0115 | 7 | 159 | 171 | TIPIDGNYFCYTR | | | Carbamidomethyl (C)[10] | Mascot |
| 1661.7363 | 1661.7477 | 0.0114 | 7 | 492 | 505 | EMGEYGLENYTEVK | | | | Mascot |
| 1677.7312 | 1677.7407 | 0.0095 | 6 | 492 | 505 | EMGEYGLENYTEVK | | | Oxidation (M)[2] | Mascot |
| 1761.833 | 1761.8429 | 0.0099 | 6 | 324 | 337 | TFVQESIYDEFVER | 125 | 100 | | Mascot |
| 1761.833 | 1761.8429 | 0.0099 | 6 | 324 | 337 | TFVQESIYDEFVER | | | | Mascot |
| 1831.0575 | 1831.0667 | 0.0092 | 5 | 209 | 225 | VAEQTPLTALYIASLIK | | | | Mascot |
| 2233.0771 | 2233.0981 | 0.021 | 9 | 324 | 341 | TFVQESIYDEFVERSVER | | | | Mascot |
| 2273.127 | 2273.1455 | 0.0185 | 8 | 394 | 413 | GYFIQPTVFGDVKDDMTI AR | | | | Mascot |
| 2289.1221 | 2289.1477 | 0.0256 | 11 | 394 | 413 | GYFIQPTVFGDVKDDMTI AR | | | Oxidation (M)[16] | Mascot |
| 2289.1221 | 2289.1477 | 0.0256 | 11 | 394 | 413 | GYFIQPTVFGDVKDDMTI AR | 4 | 0 | Oxidation (M)[16] | Mascot |
| 2448.1804 | 2448.2031 | 0.0227 | 9 | 464 | 485 | AGTVWINCYNVFGVQAP FGGYK | | | Carbamidomethyl (C)[8] | Mascot |
| 2490.1782 | 2490.2053 | 0.0271 | 11 | 346 | 367 | IVGDPFDLNTEQGPQVN EDQFK | | | | Mascot |
| 2509.2834 | 2509.3057 | 0.0223 | 9 | 441 | 463 | YGLAGAVFTQDIDKANYI SHGLR | | | | Mascot |
| 2618.2732 | 2618.304 | 0.0308 | 12 | 346 | 368 | IVGDPFDLNTEQGPQVN EDQFKK | | | | Mascot |
| 2798.3665 | 2798.3958 | 0.0293 | 10 | 52 | 77 | TFPTINPATGEIICHVAEG DKADVDK | | | Carbamidomethyl (C)[14] | Mascot |

| | | | | | | | | | | |
|---|---|---|---|---|---|---|---|---|---|---|
| 2 | Aldh2b protein [Danio rerio] | gi\|81294186 | 52681.5 | 5.57 | 23 | 699 | 100 | 520 | 100 | |

**Peptide Information**

| Calc. Mass | Obsrv. Mass | ± da | ± ppm | Start Seq. | End Seq. | Sequence | Ion Score | C. I. % | Modification | Rank | Result Type |
|---|---|---|---|---|---|---|---|---|---|---|---|
| 876.4243 | 876.4282 | 0.0039 | 4 | 73 | 79 | LADCIER | | | Carbamidomethyl (C)[4] | | Mascot |
| 974.5152 | 974.5182 | 0.003 | 3 | 394 | 401 | SLEEVIER | | | | | Mascot |
| 1030.5428 | 1030.5532 | 0.0104 | 10 | 421 | 429 | ANYISHGLR | 55 | 99.909 | | | Mascot |
| 1030.5428 | 1030.5532 | 0.0104 | 10 | 421 | 429 | ANYISHGLR | | | | | Mascot |
| 1051.6146 | 1051.7194 | 0.1048 | 100 | 334 | 343 | KVLGYISSGK | | | | | Mascot |
| 1132.5238 | 1132.5328 | 0.009 | 8 | 349 | 359 | LMCGGAPAAER | | | Carbamidomethyl (C)[3] | | Mascot |
| 1148.5187 | 1148.5253 | 0.0066 | 6 | 349 | 359 | LMCGGAPAAER | | | Carbamidomethyl (C)[3], Oxidation (M)[2] | | Mascot |
| 1403.7603 | 1403.7678 | 0.0075 | 5 | 380 | 391 | EEIFGPVMQILK | | | | | Mascot |
| 1403.7603 | 1403.7678 | 0.0075 | 5 | 380 | 391 | EEIFGPVMQILK | 67 | 99.994 | | | Mascot |
| 1419.7552 | 1419.7617 | 0.0065 | 5 | 380 | 391 | EEIFGPVMQILK | | | Oxidation (M)[8] | | Mascot |
| 1419.7552 | 1419.7617 | 0.0065 | 5 | 380 | 391 | EEIFGPVMQILK | 44 | 98.843 | Oxidation (M)[8] | | Mascot |
| 1460.6984 | 1460.6359 | -0.0625 | -43 | 345 | 359 | EGAKLMCGGAPAAER | | | | | Mascot |
| 1470.7627 | 1470.7699 | 0.0072 | 5 | 360 | 372 | GYFIQPTVFGDVK | | | | | Mascot |
| 1473.6797 | 1473.6864 | 0.0067 | 5 | 113 | 124 | YYAGWADKWEGK | | | | | Mascot |
| 1473.6797 | 1473.6864 | 0.0067 | 5 | 113 | 124 | YYAGWADKWEGK | 44 | 99.001 | | | Mascot |
| 1497.7584 | 1497.7661 | 0.0077 | 5 | 407 | 420 | YGLAGAVFTQDIDK | | | | | Mascot |
| 1515.8312 | 1515.7649 | -0.0663 | -44 | 67 | 79 | GLLLSRLADCIER | | | Carbamidomethyl (C)[10] | | Mascot |
| 1600.8441 | 1600.8541 | 0.01 | 6 | 223 | 237 | VAFTGSTDVGHLIQR | 123 | 100 | | | Mascot |
| 1600.8441 | 1600.8541 | 0.01 | 6 | 223 | 237 | VAFTGSTDVGHLIQR | | | | | Mascot |
| 1619.7522 | 1619.7637 | 0.0115 | 7 | 125 | 137 | TIPIDGNYFCYTR | 101 | 100 | Carbamidomethyl (C)[10] | | Mascot |
| 1619.7522 | 1619.7637 | 0.0115 | 7 | 125 | 137 | TIPIDGNYFCYTR | | | Carbamidomethyl (C)[10] | | Mascot |
| 1661.7363 | 1661.7477 | 0.0114 | 7 | 458 | 471 | EMGEYGLENYTEVK | | | | | Mascot |
| 1677.7312 | 1677.7407 | 0.0095 | 6 | 458 | 471 | EMGEYGLENYTEVK | | | Oxidation (M)[2] | | Mascot |
| 1761.833 | 1761.8429 | 0.0099 | 6 | 290 | 303 | TFVQESIYDEFVER | 125 | 100 | | | Mascot |
| 1761.833 | 1761.8429 | 0.0099 | 6 | 290 | 303 | TFVQESIYDEFVER | | | | | Mascot |
| 1831.0575 | 1831.0667 | 0.0092 | 5 | 175 | 191 | VAEQTPLTALYIASLIK | | | | | Mascot |
| 2233.0771 | 2233.0981 | 0.021 | 9 | 290 | 307 | TFVQESIYDEFVERSVER | | | | | Mascot |
| 2273.127 | 2273.1455 | 0.0185 | 8 | 360 | 379 | GYFIQPTVFGDVKDDMTI AR | | | | | Mascot |
| 2289.1221 | 2289.1477 | 0.0256 | 11 | 360 | 379 | GYFIQPTVFGDVKDDMTI AR | | | Oxidation (M)[16] | | Mascot |
| 2289.1221 | 2289.1477 | 0.0256 | 11 | 360 | 379 | GYFIQPTVFGDVKDDMTI AR | 4 | 0 | Oxidation (M)[16] | | Mascot |
| 2448.1804 | 2448.2031 | 0.0227 | 9 | 430 | 451 | AGTVWINCYNVFGVQAP FGGYK | | | Carbamidomethyl (C)[8] | | Mascot |
| 2490.1782 | 2490.2053 | 0.0271 | 11 | 312 | 333 | IVGDPFDLNTEQGPQVN EDQFK | | | | | Mascot |
| 2509.2834 | 2509.3057 | 0.0223 | 9 | 407 | 429 | YGLAGAVFTQDIDKANYI SHGLR | | | | | Mascot |

| | | | | | | | | | | | |
|---|---|---|---|---|---|---|---|---|---|---|---|
| 2618.2732 | 2618.304 | 0.0308 | 12 | 312 | 334 | IVGDPFDLNTEQGPQVN EDQFKK | | | | | Mascot |
| 2798.3665 | 2798.3958 | 0.0293 | 10 | 18 | 43 | TFPTINPATGEIICHVAEG DKADVDK | | | Carbamidomethyl (C)[14] | | Mascot |

| | | | | | | | | | |
|---|---|---|---|---|---|---|---|---|---|
| 3 | Aldh2b protein [Danio rerio] | gi|94574478 | 56422.4 | 6.35 | 23 | 694 | 100 | 520 | 100 |

**Peptide Information**

| Calc. Mass | Obsrv. Mass | ± da | ± ppm | Start Seq. | End Seq. | Sequence | Ion Score | C. I. % | Modification | Rank | Result Type |
|---|---|---|---|---|---|---|---|---|---|---|---|
| 876.4243 | 876.4282 | 0.0039 | 4 | 107 | 113 | LADCIER | | | Carbamidomethyl (C)[4] | | Mascot |
| 974.5152 | 974.5182 | 0.003 | 3 | 428 | 435 | SLEEVIER | | | | | Mascot |
| 1030.5428 | 1030.5532 | 0.0104 | 10 | 455 | 463 | ANYISHGLR | 55 | 99.909 | | | Mascot |
| 1030.5428 | 1030.5532 | 0.0104 | 10 | 455 | 463 | ANYISHGLR | | | | | Mascot |
| 1051.6146 | 1051.7194 | 0.1048 | 100 | 368 | 377 | KVLGYISSGK | | | | | Mascot |
| 1132.5238 | 1132.5328 | 0.009 | 8 | 383 | 393 | LMCGGAPAAER | | | Carbamidomethyl (C)[3] | | Mascot |
| 1148.5187 | 1148.5253 | 0.0066 | 6 | 383 | 393 | LMCGGAPAAER | | | Carbamidomethyl (C)[3], Oxidation (M)[2] | | Mascot |
| 1403.7603 | 1403.7678 | 0.0075 | 5 | 414 | 425 | EEIFGPVMQILK | | | | | Mascot |
| 1403.7603 | 1403.7678 | 0.0075 | 5 | 414 | 425 | EEIFGPVMQILK | 67 | 99.994 | | | Mascot |
| 1419.7552 | 1419.7617 | 0.0065 | 5 | 414 | 425 | EEIFGPVMQILK | | | Oxidation (M)[8] | | Mascot |
| 1419.7552 | 1419.7617 | 0.0065 | 5 | 414 | 425 | EEIFGPVMQILK | 44 | 98.843 | Oxidation (M)[8] | | Mascot |
| 1460.6984 | 1460.6359 | -0.0625 | -43 | 379 | 393 | EGAKLMCGGAPAAER | | | | | Mascot |
| 1470.7627 | 1470.7699 | 0.0072 | 5 | 394 | 406 | GYFIQPTVFGDVK | | | | | Mascot |
| 1473.6797 | 1473.6864 | 0.0067 | 5 | 147 | 158 | YYAGWADKWEGK | | | | | Mascot |
| 1473.6797 | 1473.6864 | 0.0067 | 5 | 147 | 158 | YYAGWADKWEGK | 44 | 99.001 | | | Mascot |
| 1515.8312 | 1515.7649 | -0.0663 | -44 | 101 | 113 | GLLLSRLADCIER | | | Carbamidomethyl (C)[10] | | Mascot |
| 1572.7805 | 1572.7897 | 0.0092 | 6 | 38 | 50 | IFINNEWHDAVSK | | | | | Mascot |
| 1600.8441 | 1600.8541 | 0.01 | 6 | 257 | 271 | VAFTGSTDVGHLIQR | 123 | 100 | | | Mascot |
| 1600.8441 | 1600.8541 | 0.01 | 6 | 257 | 271 | VAFTGSTDVGHLIQR | | | | | Mascot |
| 1619.7522 | 1619.7637 | 0.0115 | 7 | 159 | 171 | TIPIDGNYFCYTR | 101 | 100 | Carbamidomethyl (C)[10] | | Mascot |
| 1619.7522 | 1619.7637 | 0.0115 | 7 | 159 | 171 | TIPIDGNYFCYTR | | | Carbamidomethyl (C)[10] | | Mascot |
| 1661.7363 | 1661.7477 | 0.0114 | 7 | 492 | 505 | EMGEYGLENYTEVK | | | | | Mascot |
| 1677.7312 | 1677.7407 | 0.0095 | 6 | 492 | 505 | EMGEYGLENYTEVK | | | Oxidation (M)[2] | | Mascot |
| 1761.833 | 1761.8429 | 0.0099 | 6 | 324 | 337 | TFVQESIYDEFVER | 125 | 100 | | | Mascot |
| 1761.833 | 1761.8429 | 0.0099 | 6 | 324 | 337 | TFVQESIYDEFVER | | | | | Mascot |
| 1831.0575 | 1831.0667 | 0.0092 | 5 | 209 | 225 | VAEQTPLTALYIASLIK | | | | | Mascot |
| 2012.0083 | 2011.9922 | -0.0161 | -8 | 436 | 454 | ANDSKYGLAGAVFTQNID K | | | | | Mascot |
| 2233.0771 | 2233.0981 | 0.021 | 9 | 324 | 341 | TFVQESIYDEFVERSVER | | | | | Mascot |
| 2273.127 | 2273.1455 | 0.0185 | 8 | 394 | 413 | GYFIQPTVFGDVKDDMTI AR | | | | | Mascot |

| Calc. Mass | Obsrv. Mass | ± da | ± ppm | Start Seq. | End Seq. | Sequence | Ion Score | C. I. % | Modification | Rank | Result Type |
|---|---|---|---|---|---|---|---|---|---|---|---|
| 2289.1221 | 2289.1477 | 0.0256 | 11 | 394 | 413 | GYFIQPTVFGDVKDDMTIAR | | | Oxidation (M)[16] | | Mascot |
| 2289.1221 | 2289.1477 | 0.0256 | 11 | 394 | 413 | GYFIQPTVFGDVKDDMTIAR | 4 | 0 | Oxidation (M)[16] | | Mascot |
| 2448.1804 | 2448.2031 | 0.0227 | 9 | 464 | 485 | AGTVWINCYNVFGVQAPFGGYK | | | Carbamidomethyl (C)[8] | | Mascot |
| 2490.1782 | 2490.2053 | 0.0271 | 11 | 346 | 367 | IVGDPFDLNTEQGPQVNEDQFK | | | | | Mascot |
| 2618.2732 | 2618.304 | 0.0308 | 12 | 346 | 368 | IVGDPFDLNTEQGPQVNEDQFKK | | | | | Mascot |
| 2798.3665 | 2798.3958 | 0.0293 | 10 | 52 | 77 | TFPTINPATGEIICHVAEGDKADVDK | | | Carbamidomethyl (C)[14] | | Mascot |

| | | | | | | | | | |
|---|---|---|---|---|---|---|---|---|---|
| 4 | PREDICTED: aldehyde dehydrogenase, mitochondrial [Danio rerio] | gi\|292614411 | 56442.4 | 5.82 | 24 | 583 | 100 | 397 | 100 |

Peptide Information

| Calc. Mass | Obsrv. Mass | ± da | ± ppm | Start Seq. | End Seq. | Sequence | Ion Score | C. I. % | Modification | Rank | Result Type |
|---|---|---|---|---|---|---|---|---|---|---|---|
| 876.4243 | 876.4282 | 0.0039 | 4 | 107 | 113 | LADCIER | | | Carbamidomethyl (C)[4] | | Mascot |
| 974.5152 | 974.5182 | 0.003 | 3 | 428 | 435 | SLEEVIER | | | | | Mascot |
| 1030.5428 | 1030.5532 | 0.0104 | 10 | 455 | 463 | ANYISHGLR | 55 | 99.909 | | | Mascot |
| 1030.5428 | 1030.5532 | 0.0104 | 10 | 455 | 463 | ANYISHGLR | | | | | Mascot |
| 1051.6146 | 1051.7194 | 0.1048 | 100 | 368 | 377 | KVLGYISSGK | | | | | Mascot |
| 1132.5238 | 1132.5328 | 0.009 | 8 | 383 | 393 | LMCGGAPAAER | | | Carbamidomethyl (C)[3] | | Mascot |
| 1148.5187 | 1148.5253 | 0.0066 | 6 | 383 | 393 | LMCGGAPAAER | | | Carbamidomethyl (C)[3], Oxidation (M)[2] | | Mascot |
| 1403.7603 | 1403.7678 | 0.0075 | 5 | 414 | 425 | EEIFGPVMQILK | | | | | Mascot |
| 1403.7603 | 1403.7678 | 0.0075 | 5 | 414 | 425 | EEIFGPVMQILK | 67 | 99.994 | | | Mascot |
| 1419.7552 | 1419.7617 | 0.0065 | 5 | 414 | 425 | EEIFGPVMQILK | | | Oxidation (M)[8] | | Mascot |
| 1419.7552 | 1419.7617 | 0.0065 | 5 | 414 | 425 | EEIFGPVMQILK | 44 | 98.843 | Oxidation (M)[8] | | Mascot |
| 1460.6984 | 1460.6359 | -0.0625 | -43 | 379 | 393 | EGAKLMCGGAPAAER | | | | | Mascot |
| 1470.7627 | 1470.7699 | 0.0072 | 5 | 394 | 406 | GYFIQPTVFGDVK | | | | | Mascot |
| 1473.6797 | 1473.6864 | 0.0067 | 5 | 147 | 158 | YYAGWADKWEGK | | | | | Mascot |
| 1473.6797 | 1473.6864 | 0.0067 | 5 | 147 | 158 | YYAGWADKWEGK | 44 | 99.001 | | | Mascot |
| 1497.7584 | 1497.7661 | 0.0077 | 5 | 441 | 454 | YGLAGAVFTQDIDK | | | | | Mascot |
| 1515.8312 | 1515.7649 | -0.0663 | -44 | 101 | 113 | GLLLSRLADCIER | | | Carbamidomethyl (C)[10] | | Mascot |
| 1572.7805 | 1572.7897 | 0.0092 | 6 | 38 | 50 | IFINNEWHDAVSK | | | | | Mascot |
| 1619.7522 | 1619.7637 | 0.0115 | 7 | 159 | 171 | TIPIDGNYFCYTR | 101 | 100 | Carbamidomethyl (C)[10] | | Mascot |
| 1619.7522 | 1619.7637 | 0.0115 | 7 | 159 | 171 | TIPIDGNYFCYTR | | | Carbamidomethyl (C)[10] | | Mascot |
| 1661.7363 | 1661.7477 | 0.0114 | 7 | 492 | 505 | EMGEYGLENYTEVK | | | | | Mascot |
| 1677.7312 | 1677.7407 | 0.0095 | 6 | 492 | 505 | EMGEYGLENYTEVK | | | Oxidation (M)[2] | | Mascot |
| 1761.833 | 1761.8429 | 0.0099 | 6 | 324 | 337 | TFVQESIYDEFVER | 125 | 100 | | | Mascot |
| 1761.833 | 1761.8429 | 0.0099 | 6 | 324 | 337 | TFVQESIYDEFVER | | | | | Mascot |

| Calc. Mass | Obsrv. Mass | ± da | ± ppm | Start Seq. | End Seq. | Sequence | Ion Score | C. I. % | Modification | Rank | Result Type |
|---|---|---|---|---|---|---|---|---|---|---|---|
| 1831.0575 | 1831.0667 | 0.0092 | 5 | 209 | 225 | VAEQTPLTALYIASLIK | | | | | Mascot |
| 2233.0771 | 2233.0981 | 0.021 | 9 | 324 | 341 | TFVQESIYDEFVERSVER | | | | | Mascot |
| 2273.127 | 2273.1455 | 0.0185 | 8 | 394 | 413 | GYFIQPTVFGDVKDDMTI AR | | | | | Mascot |
| 2289.1221 | 2289.1477 | 0.0256 | 11 | 394 | 413 | GYFIQPTVFGDVKDDMTI AR | | | Oxidation (M)[16] | | Mascot |
| 2289.1221 | 2289.1477 | 0.0256 | 11 | 394 | 413 | GYFIQPTVFGDVKDDMTI AR | 4 | 0 | Oxidation (M)[16] | | Mascot |
| 2331.1938 | 2331.2095 | 0.0157 | 7 | 257 | 279 | VAFTGSTDVGHLIQQASS ASNLK | | | | | Mascot |
| 2448.1804 | 2448.2031 | 0.0227 | 9 | 464 | 485 | AGTVWINCYNVFGVQAP FGGYK | | | Carbamidomethyl (C)[8] | | Mascot |
| 2490.1782 | 2490.2053 | 0.0271 | 11 | 346 | 367 | IVGDPFDLNTEQGPQVN EDQFK | | | | | Mascot |
| 2509.2834 | 2509.3057 | 0.0223 | 9 | 441 | 463 | YGLAGAVFTQDIDKANYI SHGLR | | | | | Mascot |
| 2618.2732 | 2618.304 | 0.0308 | 12 | 346 | 368 | IVGDPFDLNTEQGPQVN EDQFKK | | | | | Mascot |
| 2798.3665 | 2798.3958 | 0.0293 | 10 | 52 | 77 | TFPTINPATGEIICHVAEG DKADVDK | | | Carbamidomethyl (C)[14] | | Mascot |

| | | | | | | | | | | |
|---|---|---|---|---|---|---|---|---|---|---|
| 5 | novel protein similar to aldehyde dehydrogenase 2, like (aldh2l) [Danio rerio] | gi\|122891034 | 56503.5 | 5.82 | 22 | 557 | 100 | 397 | 100 | |

**Peptide Information**

| Calc. Mass | Obsrv. Mass | ± da | ± ppm | Start Seq. | End Seq. | Sequence | Ion Score | C. I. % | Modification | Rank | Result Type |
|---|---|---|---|---|---|---|---|---|---|---|---|
| 876.4243 | 876.4282 | 0.0039 | 4 | 107 | 113 | LADCIER | | | Carbamidomethyl (C)[4] | | Mascot |
| 974.5152 | 974.5182 | 0.003 | 3 | 428 | 435 | SLEEVIER | | | | | Mascot |
| 1030.5428 | 1030.5532 | 0.0104 | 10 | 455 | 463 | ANYISHGLR | 55 | 99.909 | | | Mascot |
| 1030.5428 | 1030.5532 | 0.0104 | 10 | 455 | 463 | ANYISHGLR | | | | | Mascot |
| 1051.6146 | 1051.7194 | 0.1048 | 100 | 368 | 377 | KVLGYISSGK | | | | | Mascot |
| 1132.5238 | 1132.5328 | 0.009 | 8 | 383 | 393 | LMCGGAPAAER | | | Carbamidomethyl (C)[3] | | Mascot |
| 1148.5187 | 1148.5253 | 0.0066 | 6 | 383 | 393 | LMCGGAPAAER | | | Carbamidomethyl (C)[3], Oxidation (M)[2] | | Mascot |
| 1403.7603 | 1403.7678 | 0.0075 | 5 | 414 | 425 | EEIFGPVMQILK | | | | | Mascot |
| 1403.7603 | 1403.7678 | 0.0075 | 5 | 414 | 425 | EEIFGPVMQILK | 67 | 99.994 | | | Mascot |
| 1419.7552 | 1419.7617 | 0.0065 | 5 | 414 | 425 | EEIFGPVMQILK | | | Oxidation (M)[8] | | Mascot |
| 1419.7552 | 1419.7617 | 0.0065 | 5 | 414 | 425 | EEIFGPVMQILK | 44 | 98.843 | Oxidation (M)[8] | | Mascot |
| 1460.6984 | 1460.6359 | -0.0625 | -43 | 379 | 393 | EGAKLMCGGAPAAER | | | | | Mascot |
| 1470.7627 | 1470.7699 | 0.0072 | 5 | 394 | 406 | GYFIQPTVFGDVK | | | | | Mascot |
| 1473.6797 | 1473.6864 | 0.0067 | 5 | 147 | 158 | YYAGWADKWEGK | | | | | Mascot |
| 1473.6797 | 1473.6864 | 0.0067 | 5 | 147 | 158 | YYAGWADKWEGK | 44 | 99.001 | | | Mascot |
| 1497.7584 | 1497.7661 | 0.0077 | 5 | 441 | 454 | YGLAGAVFTQDIDK | | | | | Mascot |
| 1515.8312 | 1515.7649 | -0.0663 | -44 | 101 | 113 | GLLLSRLADCIER | | | Carbamidomethyl (C)[10] | | Mascot |
| 1572.7805 | 1572.7897 | 0.0092 | 6 | 38 | 50 | IFINNEWHDAVSK | | | | | Mascot |

| Calc. Mass | Obsrv. Mass | ± da | ± ppm | Start Seq. | End Seq. | Sequence | Ion Score | C. I. % | Modification | Rank | Result Type |
|---|---|---|---|---|---|---|---|---|---|---|---|
| 1619.7522 | 1619.7637 | 0.0115 | 7 | 159 | 171 | TIPIDGNYFCYTR | 101 | 100 | Carbamidomethyl (C)[10] | | Mascot |
| 1619.7522 | 1619.7637 | 0.0115 | 7 | 159 | 171 | TIPIDGNYFCYTR | | | Carbamidomethyl (C)[10] | | Mascot |
| 1761.833 | 1761.8429 | 0.0099 | 6 | 324 | 337 | TFVQESIYDEFVER | 125 | 100 | | | Mascot |
| 1761.833 | 1761.8429 | 0.0099 | 6 | 324 | 337 | TFVQESIYDEFVER | | | | | Mascot |
| 1831.0575 | 1831.0667 | 0.0092 | 5 | 209 | 225 | VAEQTPLTALYIASLIK | | | | | Mascot |
| 2170.1025 | 2170.0898 | -0.0127 | -6 | 486 | 505 | ASGIGRELGEYGLDIYTEVK | | | | | Mascot |
| 2233.0771 | 2233.0981 | 0.021 | 9 | 324 | 341 | TFVQESIYDEFVERSVER | | | | | Mascot |
| 2273.127 | 2273.1455 | 0.0185 | 8 | 394 | 413 | GYFIQPTVFGDVKDDMTIAR | | | | | Mascot |
| 2289.1221 | 2289.1477 | 0.0256 | 11 | 394 | 413 | GYFIQPTVFGDVKDDMTIAR | | | Oxidation (M)[16] | | Mascot |
| 2289.1221 | 2289.1477 | 0.0256 | 11 | 394 | 413 | GYFIQPTVFGDVKDDMTIAR | 4 | 0 | Oxidation (M)[16] | | Mascot |
| 2331.1938 | 2331.2095 | 0.0157 | 7 | 257 | 279 | VAFTGSTDVGHLIQQASSASNLK | | | | | Mascot |
| 2448.1804 | 2448.2031 | 0.0227 | 9 | 464 | 485 | AGTVWINCYNVFGVQAPFGGYK | | | Carbamidomethyl (C)[8] | | Mascot |
| 2509.2834 | 2509.3057 | 0.0223 | 9 | 441 | 463 | YGLAGAVFTQDIDKANYISHGLR | | | | | Mascot |
| 2798.3665 | 2798.3958 | 0.0293 | 10 | 52 | 77 | TFPTINPATAEVICHVAEGDKADVDK | | | Carbamidomethyl (C)[14] | | Mascot |

6 aldehyde dehydrogenase 2 precursor [Danio rerio] gi|20339358 56526.6 6.05 20 527 100 392 100

**Peptide Information**

| Calc. Mass | Obsrv. Mass | ± da | ± ppm | Start Seq. | End Seq. | Sequence | Ion Score | C. I. % | Modification | Rank | Result Type |
|---|---|---|---|---|---|---|---|---|---|---|---|
| 864.4243 | 864.5198 | 0.0955 | 110 | 407 | 413 | DDMKIAR | | | Oxidation (M)[3] | | Mascot |
| 876.4243 | 876.4282 | 0.0039 | 4 | 107 | 113 | LADCIER | | | Carbamidomethyl (C)[4] | | Mascot |
| 974.5152 | 974.5182 | 0.003 | 3 | 428 | 435 | SLEEVIER | | | | | Mascot |
| 1030.5428 | 1030.5532 | 0.0104 | 10 | 455 | 463 | ANYISHGLR | 55 | 99.909 | | | Mascot |
| 1030.5428 | 1030.5532 | 0.0104 | 10 | 455 | 463 | ANYISHGLR | | | | | Mascot |
| 1051.6146 | 1051.7194 | 0.1048 | 100 | 368 | 377 | KVLGYISSGK | | | | | Mascot |
| 1132.5238 | 1132.5328 | 0.009 | 8 | 383 | 393 | LMCGGAPAAER | | | Carbamidomethyl (C)[3] | | Mascot |
| 1148.5187 | 1148.5253 | 0.0066 | 6 | 383 | 393 | LMCGGAPAAER | | | Carbamidomethyl (C)[3], Oxidation (M)[2] | | Mascot |
| 1403.7603 | 1403.7678 | 0.0075 | 5 | 414 | 425 | EEIFGPVMQILK | | | | | Mascot |
| 1403.7603 | 1403.7678 | 0.0075 | 5 | 414 | 425 | EEIFGPVMQILK | 67 | 99.994 | | | Mascot |
| 1419.7552 | 1419.7617 | 0.0065 | 5 | 414 | 425 | EEIFGPVMQILK | | | Oxidation (M)[8] | | Mascot |
| 1419.7552 | 1419.7617 | 0.0065 | 5 | 414 | 425 | EEIFGPVMQILK | 44 | 98.843 | Oxidation (M)[8] | | Mascot |
| 1454.7903 | 1454.741 | -0.0493 | -34 | 4 | 15 | AVFSRTFPQVFR | | | | | Mascot |
| 1460.6984 | 1460.6359 | -0.0625 | -43 | 379 | 393 | EGAKLMCGGAPAAER | | | | | Mascot |
| 1470.7627 | 1470.7699 | 0.0072 | 5 | 394 | 406 | GYFIQPTVFGDVK | | | | | Mascot |
| 1473.6797 | 1473.6864 | 0.0067 | 5 | 147 | 158 | YYAGWADKWEGK | | | | | Mascot |

| | | | | | | | | | | | |
|---|---|---|---|---|---|---|---|---|---|---|---|
| 1473.6797 | 1473.6864 | 0.0067 | 5 | 147 | 158 | YYAGWADKWEGK | 44 | 99.001 | | | Mascot |
| 1572.7805 | 1572.7897 | 0.0092 | 6 | 38 | 50 | IFINNEWHDAVSK | | | | | Mascot |
| 1619.7522 | 1619.7637 | 0.0115 | 7 | 159 | 171 | TIPIDGNYFCYTR | 101 | 100 | Carbamidomethyl (C)[10] | | Mascot |
| 1619.7522 | 1619.7637 | 0.0115 | 7 | 159 | 171 | TIPIDGNYFCYTR | | | Carbamidomethyl (C)[10] | | Mascot |
| 1761.833 | 1761.8429 | 0.0099 | 6 | 324 | 337 | TFVQESIYDEFVER | 125 | 100 | | | Mascot |
| 1761.833 | 1761.8429 | 0.0099 | 6 | 324 | 337 | TFVQESIYDEFVER | | | | | Mascot |
| 1831.0575 | 1831.0667 | 0.0092 | 5 | 209 | 225 | VAEQTPLTALYIASLIK | | | | | Mascot |
| 2170.1025 | 2170.0898 | -0.0127 | -6 | 486 | 505 | ASGIGRELGEYGLDIYTE VK | | | | | Mascot |
| 2233.0771 | 2233.0981 | 0.021 | 9 | 324 | 341 | TFVQESIYDEFVERSVER | | | | | Mascot |
| 2331.1938 | 2331.2095 | 0.0157 | 7 | 257 | 279 | VAFTGSTDVGHLIQQASS ASNLK | | | | | Mascot |
| 2448.1804 | 2448.2031 | 0.0227 | 9 | 464 | 485 | AGTVWINCYNVFGVQAP FGGYK | | | Carbamidomethyl (C)[8] | | Mascot |
| 2798.3665 | 2798.3958 | 0.0293 | 10 | 52 | 77 | TFPTINPATAEVICHVAEG DKADVDK | | | Carbamidomethyl (C)[14] | | Mascot |

| | | | | | | | | | | | |
|---|---|---|---|---|---|---|---|---|---|---|---|
| 7 | aldehyde dehydrogenase 2 precursor [Danio rerio] | gi\|41053732 | 56529.6 | 5.93 | 19 | 523 | 100 | 397 | 100 | | |

**Peptide Information**

| Calc. Mass | Obsrv. Mass | ± da | ± ppm | Start Seq. | End Seq. | Sequence | Ion Score | C. I. % | Modification | Rank | Result Type |
|---|---|---|---|---|---|---|---|---|---|---|---|
| 876.4243 | 876.4282 | 0.0039 | 4 | 107 | 113 | LADCIER | | | Carbamidomethyl (C)[4] | | Mascot |
| 974.5152 | 974.5182 | 0.003 | 3 | 428 | 435 | SLEEVIER | | | | | Mascot |
| 1030.5428 | 1030.5532 | 0.0104 | 10 | 455 | 463 | ANYISHGLR | 55 | 99.909 | | | Mascot |
| 1030.5428 | 1030.5532 | 0.0104 | 10 | 455 | 463 | ANYISHGLR | | | | | Mascot |
| 1051.6146 | 1051.7194 | 0.1048 | 100 | 368 | 377 | KVLGYISSGK | | | | | Mascot |
| 1132.5238 | 1132.5328 | 0.009 | 8 | 383 | 393 | LMCGGAPAAER | | | Carbamidomethyl (C)[3] | | Mascot |
| 1148.5187 | 1148.5253 | 0.0066 | 6 | 383 | 393 | LMCGGAPAAER | | | Carbamidomethyl (C)[3], Oxidation (M)[2] | | Mascot |
| 1403.7603 | 1403.7678 | 0.0075 | 5 | 414 | 425 | EEIFGPVMQILK | | | | | Mascot |
| 1403.7603 | 1403.7678 | 0.0075 | 5 | 414 | 425 | EEIFGPVMQILK | 67 | 99.994 | | | Mascot |
| 1419.7552 | 1419.7617 | 0.0065 | 5 | 414 | 425 | EEIFGPVMQILK | | | Oxidation (M)[8] | | Mascot |
| 1419.7552 | 1419.7617 | 0.0065 | 5 | 414 | 425 | EEIFGPVMQILK | 44 | 98.843 | Oxidation (M)[8] | | Mascot |
| 1460.6984 | 1460.6359 | -0.0625 | -43 | 379 | 393 | EGAKLMCGGAPAAER | | | | | Mascot |
| 1470.7627 | 1470.7699 | 0.0072 | 5 | 394 | 406 | GYFIQPTVFGDVK | | | | | Mascot |
| 1473.6797 | 1473.6864 | 0.0067 | 5 | 147 | 158 | YYAGWADKWEGK | | | | | Mascot |
| 1473.6797 | 1473.6864 | 0.0067 | 5 | 147 | 158 | YYAGWADKWEGK | 44 | 99.001 | | | Mascot |
| 1572.7805 | 1572.7897 | 0.0092 | 6 | 38 | 50 | IFINNEWHDAVSK | | | | | Mascot |
| 1619.7522 | 1619.7637 | 0.0115 | 7 | 159 | 171 | TIPIDGNYFCYTR | 101 | 100 | Carbamidomethyl (C)[10] | | Mascot |
| 1619.7522 | 1619.7637 | 0.0115 | 7 | 159 | 171 | TIPIDGNYFCYTR | | | Carbamidomethyl (C)[10] | | Mascot |
| 1761.833 | 1761.8429 | 0.0099 | 6 | 324 | 337 | TFVQESIYDEFVER | 125 | 100 | | | Mascot |

| Calc. Mass | Obsrv. Mass | ± da | ± ppm | Start Seq. | End Seq. | Sequence | Ion Score | C. I. % | Modification | Rank | Result Type |
|---|---|---|---|---|---|---|---|---|---|---|---|
| 1761.833 | 1761.8429 | 0.0099 | 6 | 324 | 337 | TFVQESIYDEFVER | | | | | Mascot |
| 1831.0575 | 1831.0667 | 0.0092 | 5 | 209 | 225 | VAEQTPLTALYIASLIK | | | | | Mascot |
| 2170.1025 | 2170.0898 | -0.0127 | -6 | 486 | 505 | ASGIGRELGEYGLDIYTE VK | | | | | Mascot |
| 2233.0771 | 2233.0981 | 0.021 | 9 | 324 | 341 | TFVQESIYDEFVERSVER | | | | | Mascot |
| 2273.127 | 2273.1455 | 0.0185 | 8 | 394 | 413 | GYFIQPTVFGDVKDDMTI AR | | | | | Mascot |
| 2289.1221 | 2289.1477 | 0.0256 | 11 | 394 | 413 | GYFIQPTVFGDVKDDMTI AR | | | Oxidation (M)[16] | | Mascot |
| 2289.1221 | 2289.1477 | 0.0256 | 11 | 394 | 413 | GYFIQPTVFGDVKDDMTI AR | 4 | 0 | Oxidation (M)[16] | | Mascot |
| 2331.1938 | 2331.2095 | 0.0157 | 7 | 257 | 279 | VAFTGSTDVGHLIQQASS ASNLK | | | | | Mascot |
| 2448.1804 | 2448.2031 | 0.0227 | 9 | 464 | 485 | AGTVWINCYNVFGVQAP FGGYK | | | Carbamidomethyl (C)[8] | | Mascot |
| 2798.3665 | 2798.3958 | 0.0293 | 10 | 52 | 77 | TFPTINPATAEVICHVAEG DKADVDK | | | Carbamidomethyl (C)[14] | | Mascot |

| | | | | | | | | | | |
|---|---|---|---|---|---|---|---|---|---|---|
| 8 | PREDICTED: aldehyde dehydrogenase, mitochondrial-like, partial [Danio rerio] | gi\|326681302 | 31349.7 | 5.28 | 17 | 517 | 100 | 374 | 100 | |

**Peptide Information**

| Calc. Mass | Obsrv. Mass | ± da | ± ppm | Start Seq. | End Seq. | Sequence | Ion Score | C. I. % | Modification | Rank | Result Type |
|---|---|---|---|---|---|---|---|---|---|---|---|
| 974.5152 | 974.5182 | 0.003 | 3 | 202 | 209 | SLEEVIER | | | | | Mascot |
| 1030.5428 | 1030.5532 | 0.0104 | 10 | 229 | 237 | ANYISHGLR | 55 | 99.909 | | | Mascot |
| 1030.5428 | 1030.5532 | 0.0104 | 10 | 229 | 237 | ANYISHGLR | | | | | Mascot |
| 1051.6146 | 1051.7194 | 0.1048 | 100 | 142 | 151 | KVLGYISSGK | | | | | Mascot |
| 1132.5238 | 1132.5328 | 0.009 | 8 | 157 | 167 | LMCGGAPAAER | | | Carbamidomethyl (C)[3] | | Mascot |
| 1148.5187 | 1148.5253 | 0.0066 | 6 | 157 | 167 | LMCGGAPAAER | | | Carbamidomethyl (C)[3], Oxidation (M)[2] | | Mascot |
| 1403.7603 | 1403.7678 | 0.0075 | 5 | 188 | 199 | EEIFGPVMQILK | | | | | Mascot |
| 1403.7603 | 1403.7678 | 0.0075 | 5 | 188 | 199 | EEIFGPVMQILK | 67 | 99.994 | | | Mascot |
| 1419.7552 | 1419.7617 | 0.0065 | 5 | 188 | 199 | EEIFGPVMQILK | | | Oxidation (M)[8] | | Mascot |
| 1419.7552 | 1419.7617 | 0.0065 | 5 | 188 | 199 | EEIFGPVMQILK | 44 | 98.843 | Oxidation (M)[8] | | Mascot |
| 1460.6984 | 1460.6359 | -0.0625 | -43 | 153 | 167 | EGAKLMCGGAPAAER | | | | | Mascot |
| 1470.7627 | 1470.7699 | 0.0072 | 5 | 168 | 180 | GYFIQPTVFGDVK | | | | | Mascot |
| 1497.7584 | 1497.7661 | 0.0077 | 5 | 215 | 228 | YGLAGAVFTQDIDK | | | | | Mascot |
| 1600.8441 | 1600.8541 | 0.01 | 6 | 31 | 45 | VAFTGSTDVGHLIQR | 123 | 100 | | | Mascot |
| 1600.8441 | 1600.8541 | 0.01 | 6 | 31 | 45 | VAFTGSTDVGHLIQR | | | | | Mascot |
| 1661.7363 | 1661.7477 | 0.0114 | 7 | 266 | 279 | EMGEYGLENYTEVK | | | | | Mascot |
| 1677.7312 | 1677.7407 | 0.0095 | 6 | 266 | 279 | EMGEYGLENYTEVK | | | Oxidation (M)[2] | | Mascot |
| 1761.833 | 1761.8429 | 0.0099 | 6 | 98 | 111 | TFVQESIYDEFVER | 125 | 100 | | | Mascot |
| 1761.833 | 1761.8429 | 0.0099 | 6 | 98 | 111 | TFVQESIYDEFVER | | | | | Mascot |
| 2233.0771 | 2233.0981 | 0.021 | 9 | 98 | 115 | TFVQESIYDEFVERSVER | | | | | Mascot |

| Calc. Mass | Obsrv. Mass | ± da | ± ppm | Start Seq. | End Seq. | Sequence | Ion Score | C. I. % | Modification | Rank | Result Type |
|---|---|---|---|---|---|---|---|---|---|---|---|
| 2273.127 | 2273.1455 | 0.0185 | 8 | 168 | 187 | GYFIQPTVFGDVKDDMTIAR | | | | | Mascot |
| 2289.1221 | 2289.1477 | 0.0256 | 11 | 168 | 187 | GYFIQPTVFGDVKDDMTIAR | | | Oxidation (M)[16] | | Mascot |
| 2289.1221 | 2289.1477 | 0.0256 | 11 | 168 | 187 | GYFIQPTVFGDVKDDMTIAR | 4 | 0 | Oxidation (M)[16] | | Mascot |
| 2448.1804 | 2448.2031 | 0.0227 | 9 | 238 | 259 | AGTVWINCYNVFGVQAPFGGYK | | | Carbamidomethyl (C)[8] | | Mascot |
| 2490.1782 | 2490.2053 | 0.0271 | 11 | 120 | 141 | IVGDPFDLNTEQGPQVNEDQFK | | | | | Mascot |
| 2509.2834 | 2509.3057 | 0.0223 | 9 | 215 | 237 | YGLAGAVFTQDIDKANYISHGLR | | | | | Mascot |
| 2618.2732 | 2618.304 | 0.0308 | 12 | 120 | 142 | IVGDPFDLNTEQGPQVNEDQFKK | | | | | Mascot |

| | | | | | | | | | | |
|---|---|---|---|---|---|---|---|---|---|---|
| 9 | short transient receptor potential channel 4-associated protein [Danio rerio] | gi\|18859441 | 88810.6 | 6.3 | 11 | 51 | 61.241 | 25 | 12.827 |

**Peptide Information**

| Calc. Mass | Obsrv. Mass | ± da | ± ppm | Start Seq. | End Seq. | Sequence | Ion Score | C. I. % | Modification | Rank | Result Type |
|---|---|---|---|---|---|---|---|---|---|---|---|
| 996.5407 | 996.6027 | 0.062 | 62 | 349 | 356 | HRLPQSMK | | | | | Mascot |
| 1454.7023 | 1454.741 | 0.0387 | 27 | 437 | 448 | LLQSFSDHHENK | | | | | Mascot |
| 1460.709 | 1460.6359 | -0.0731 | -50 | 521 | 533 | GATSYADQMFLLK | | | Oxidation (M)[9] | | Mascot |
| 1470.7985 | 1470.7699 | -0.0286 | -19 | 2 | 16 | ATLLGPESPCIGGKK | | | | | Mascot |
| 1473.744 | 1473.6864 | -0.0576 | -39 | 1 | 15 | MATLLGPESPCIGGK | | | | | Mascot |
| 1473.744 | 1473.6864 | -0.0576 | -39 | 1 | 15 | MATLLGPESPCIGGK | | | | | Mascot |
| 1515.7491 | 1515.7649 | 0.0158 | 10 | 712 | 722 | FWQHHYLNKDK | | | | | Mascot |
| 1600.8152 | 1600.8541 | 0.0389 | 24 | 521 | 534 | GATSYADQMFLLKR | 25 | 12.827 | | | Mascot |
| 1600.8152 | 1600.8541 | 0.0389 | 24 | 521 | 534 | GATSYADQMFLLKR | | | | | Mascot |
| 1761.9996 | 1761.8429 | -0.1567 | -89 | 188 | 203 | TFLQTATLIEDILGVK | | | | | Mascot |
| 1761.9996 | 1761.8429 | -0.1567 | -89 | 188 | 203 | TFLQTATLIEDILGVK | | | | | Mascot |
| 1830.8798 | 1831.0667 | 0.1869 | 102 | 623 | 638 | VVEVLSECCLLSYMAR | | | Oxidation (M)[14] | | Mascot |
| 1917.0084 | 1916.9781 | -0.0303 | -16 | 534 | 550 | RGLLEHILFCIIDSGCK | | | | | Mascot |
| 2273.0464 | 2273.1455 | 0.0991 | 44 | 751 | 769 | TSPCAIISYIDEPYMELDR | | | Carbamidomethyl (C)[4] | | Mascot |
| 2289.0413 | 2289.1477 | 0.1064 | 46 | 751 | 769 | TSPCAIISYIDEPYMELDR | | | Carbamidomethyl (C)[4], Oxidation (M)[15] | | Mascot |
| 2289.0413 | 2289.1477 | 0.1064 | 46 | 751 | 769 | TSPCAIISYIDEPYMELDR | | | Carbamidomethyl (C)[4], Oxidation (M)[15] | | Mascot |

| | | | | | | | |
|---|---|---|---|---|---|---|---|
| 10 | nitrogen fixation cluster-like [Danio rerio] | gi\|221307563 | 17420.2 | 8.83 | 8 | 46 | 0 |

**Peptide Information**

| Calc. Mass | Obsrv. Mass | ± da | ± ppm | Start Seq. | End Seq. | Sequence | Ion Score | C. I. % | Modification | Rank | Result Type |
|---|---|---|---|---|---|---|---|---|---|---|---|
| 835.4705 | 835.476 | 0.0055 | 7 | 1 | 8 | MAASLAKK | | | Oxidation (M)[1] | | Mascot |
| 882.5294 | 882.5712 | 0.0418 | 47 | 122 | 129 | ELSLPPVK | | | | | Mascot |

| | | | | | | | | | | | | |
|---|---|---|---|---|---|---|---|---|---|---|---|---|
| 974.5417 | 974.5182 | -0.0235 | -24 | 25 | 32 | TQIGYHKK | | | | | | Mascot |
| 1045.5636 | 1045.5658 | 0.0022 | 2 | 42 | 51 | NVGSLDKNAK | | | | | | Mascot |
| 1256.6382 | 1256.7491 | 0.1109 | 88 | 32 | 41 | KVVDHYENPR | | | | | | Mascot |
| 1403.6658 | 1403.7678 | 0.102 | 73 | 130 | 141 | LHCSMLAEDAIK | | | Carbamidomethyl (C)[3], Oxidation (M)[5] | | | Mascot |
| 1403.6658 | 1403.7678 | 0.102 | 73 | 130 | 141 | LHCSMLAEDAIK | | | Carbamidomethyl (C)[3], Oxidation (M)[5] | | | Mascot |
| 1528.7238 | 1528.7517 | 0.0279 | 18 | 151 | 164 | QEGEPVEETAVARN | | | | | | Mascot |
| 1661.7985 | 1661.7477 | -0.0508 | -31 | 52 | 68 | NVGTGLVGAPACGDVMK | | | Carbamidomethyl (C)[12], Oxidation (M)[16] | | | Mascot |

| | | | | | | | | |
|---|---|---|---|---|---|---|---|---|
| 11 | PREDICTED: complement C3 [Danio rerio] | gi\|326664394 | 182595 | 6.23 | 19 | 44 | 0 | |

Peptide Information

| Calc. Mass | Obsrv. Mass | ± da | ± ppm | Start Seq. | End Seq. | Sequence | Ion Score | C. I. % | Modification | Rank | Result Type |
|---|---|---|---|---|---|---|---|---|---|---|---|
| 835.4923 | 835.476 | -0.0163 | -20 | 810 | 816 | SLFIDLK | | | | | Mascot |
| 850.4668 | 850.5481 | 0.0813 | 96 | 1149 | 1156 | AVSFLEGK | | | | | Mascot |
| 882.4679 | 882.5712 | 0.1033 | 117 | 1021 | 1028 | DEAIGHIK | | | | | Mascot |
| 1148.6674 | 1148.5253 | -0.1421 | -124 | 50 | 59 | NAFEVKITVK | | | | | Mascot |
| 1256.613 | 1256.7491 | 0.1361 | 108 | 1029 | 1038 | TGYNRQQAYR | | | | | Mascot |
| 1403.7028 | 1403.7678 | 0.065 | 46 | 1362 | 1372 | LTMDFFYKNPK | | | | | Mascot |
| 1403.7028 | 1403.7678 | 0.065 | 46 | 1362 | 1372 | LTMDFFYKNPK | | | | | Mascot |
| 1419.6978 | 1419.7617 | 0.0639 | 45 | 1362 | 1372 | LTMDFFYKNPK | | | Oxidation (M)[3] | | Mascot |
| 1419.6978 | 1419.7617 | 0.0639 | 45 | 1362 | 1372 | LTMDFFYKNPK | | | Oxidation (M)[3] | | Mascot |
| 1448.8472 | 1448.9344 | 0.0872 | 60 | 1414 | 1426 | ALSDRGSLILYLK | | | | | Mascot |
| 1454.6224 | 1454.741 | 0.1186 | 82 | 711 | 721 | AFLHCCDEMKK | | | Carbamidomethyl (C)[5,6], Oxidation (M)[9] | | Mascot |
| 1470.889 | 1470.7699 | -0.1191 | -81 | 289 | 302 | IQILKGVGTAELTK | | | | | Mascot |
| 1473.7445 | 1473.6864 | -0.0581 | -39 | 1021 | 1033 | DEAIGHIKTGYNR | | | | | Mascot |
| 1473.8312 | 1473.6864 | -0.1448 | -98 | 596 | 607 | LTQTKIWDVIEK | | | | | Mascot |
| 1492.681 | 1492.7563 | 0.0753 | 50 | 1511 | 1523 | ACESGMDYVYKVK | | | | | Mascot |
| 1600.8363 | 1600.8541 | 0.0178 | 11 | 1133 | 1147 | ELCGAIVGSLPESIR | | | Carbamidomethyl (C)[3] | | Mascot |
| 1600.8363 | 1600.8541 | 0.0178 | 11 | 1133 | 1147 | ELCGAIVGSLPESIR | | | Carbamidomethyl (C)[3] | | Mascot |
| 1677.7611 | 1677.7407 | -0.0204 | -12 | 1508 | 1521 | NIKACESGMDYVYK | | | Carbamidomethyl (C)[5] | | Mascot |
| 1857.9381 | 1857.8822 | -0.0559 | -30 | 209 | 224 | FANTPQKTFTAEFEVK | | | | | Mascot |
| 1916.7605 | 1916.9781 | 0.2176 | 114 | 1484 | 1499 | GDMCQCAEENCSLQKK | | | Carbamidomethyl (C)[4,6], Oxidation (M)[3] | | Mascot |
| 2430.0596 | 2430.2925 | 0.2329 | 96 | 699 | 720 | ATYIGDGPECVKAFLHCC DEMK | | | | | Mascot |
| 2538.2466 | 2538.2896 | 0.043 | 17 | 1524 | 1545 | VKDMELAADADIYQMEV EQVLK | | | | | Mascot |
| 3969.0461 | 3968.8386 | -0.2075 | -52 | 774 | 809 | AMYLKDSITTWQVLAVS MSPTLGICVAEPEEIVVFK | | | | | Mascot |

| | | | | | | | | | | |
|---|---|---|---|---|---|---|---|---|---|---|
| 12 | Trpc4apa protein [Danio rerio] | gi\|141795453 | 37462.2 | 5.6 | 6 | 43 | 0 | 25 | 12.827 | |

Peptide Information

| Calc. Mass | Obsrv. Mass | ± da | ± ppm | Start Seq. | End Seq. | Sequence | Ion Score | C. I. % | Modification | Rank | Result Type |
|---|---|---|---|---|---|---|---|---|---|---|---|
| 1460.709 | 1460.6359 | -0.0731 | -50 | 71 | 83 | GATSYADQMFLLK | | | Oxidation (M)[9] | | Mascot |
| 1515.7491 | 1515.7649 | 0.0158 | 10 | 262 | 272 | FWQHHYLNKDK | | | | | Mascot |
| 1600.8152 | 1600.8541 | 0.0389 | 24 | 71 | 84 | GATSYADQMFLLKR | 25 | 12.827 | | | Mascot |
| 1600.8152 | 1600.8541 | 0.0389 | 24 | 71 | 84 | GATSYADQMFLLKR | | | | | Mascot |
| 1830.8798 | 1831.0667 | 0.1869 | 102 | 173 | 188 | VVEVLSECCLLSYMAR | | | Oxidation (M)[14] | | Mascot |
| 1917.0084 | 1916.9781 | -0.0303 | -16 | 84 | 100 | RGLLEHILFCIIDSGCK | | | | | Mascot |
| 2273.0464 | 2273.1455 | 0.0991 | 44 | 301 | 319 | TSPCAIISYIDEPYMELDR | | | Carbamidomethyl (C)[4] | | Mascot |
| 2289.0413 | 2289.1477 | 0.1064 | 46 | 301 | 319 | TSPCAIISYIDEPYMELDR | | | Carbamidomethyl (C)[4], Oxidation (M)[15] | | Mascot |
| 2289.0413 | 2289.1477 | 0.1064 | 46 | 301 | 319 | TSPCAIISYIDEPYMELDR | | | Carbamidomethyl (C)[4], Oxidation (M)[15] | | Mascot |

| | | | | | | | | | | |
|---|---|---|---|---|---|---|---|---|---|---|
| 13 | Trpc4apa protein [Danio rerio] | gi\|157278955 | 36793.8 | 5.75 | 6 | 43 | 0 | 25 | 12.827 |

Peptide Information

| Calc. Mass | Obsrv. Mass | ± da | ± ppm | Start Seq. | End Seq. | Sequence | Ion Score | C. I. % | Modification | Rank | Result Type |
|---|---|---|---|---|---|---|---|---|---|---|---|
| 1460.709 | 1460.6359 | -0.0731 | -50 | 65 | 77 | GATSYADQMFLLK | | | Oxidation (M)[9] | | Mascot |
| 1515.7491 | 1515.7649 | 0.0158 | 10 | 256 | 266 | FWQHHYLNKDK | | | | | Mascot |
| 1600.8152 | 1600.8541 | 0.0389 | 24 | 65 | 78 | GATSYADQMFLLKR | 25 | 12.827 | | | Mascot |
| 1600.8152 | 1600.8541 | 0.0389 | 24 | 65 | 78 | GATSYADQMFLLKR | | | | | Mascot |
| 1830.8798 | 1831.0667 | 0.1869 | 102 | 167 | 182 | VVEVLSECCLLSYMAR | | | Oxidation (M)[14] | | Mascot |
| 1917.0084 | 1916.9781 | -0.0303 | -16 | 78 | 94 | RGLLEHILFCIIDSGCK | | | | | Mascot |
| 2273.0464 | 2273.1455 | 0.0991 | 44 | 295 | 313 | TSPCAIISYIDEPYMELDR | | | Carbamidomethyl (C)[4] | | Mascot |
| 2289.0413 | 2289.1477 | 0.1064 | 46 | 295 | 313 | TSPCAIISYIDEPYMELDR | | | Carbamidomethyl (C)[4], Oxidation (M)[15] | | Mascot |
| 2289.0413 | 2289.1477 | 0.1064 | 46 | 295 | 313 | TSPCAIISYIDEPYMELDR | | | Carbamidomethyl (C)[4], Oxidation (M)[15] | | Mascot |

| | | | | | | | | |
|---|---|---|---|---|---|---|---|---|
| 14 | novel protein (sb:cb26) [Danio rerio] | gi\|220673387 | 183117.1 | 6.17 | 19 | 41 | 0 |

Peptide Information

| Calc. Mass | Obsrv. Mass | ± da | ± ppm | Start Seq. | End Seq. | Sequence | Ion Score | C. I. % | Modification | Rank | Result Type |
|---|---|---|---|---|---|---|---|---|---|---|---|
| 835.4923 | 835.476 | -0.0163 | -20 | 815 | 821 | SLFIDLK | | | | | Mascot |
| 850.4668 | 850.5481 | 0.0813 | 96 | 1154 | 1161 | AVSFLEGK | | | | | Mascot |
| 882.4679 | 882.5712 | 0.1033 | 117 | 1026 | 1033 | DEAIGHIK | | | | | Mascot |
| 1148.6674 | 1148.5253 | -0.1421 | -124 | 50 | 59 | NAFEVKITVK | | | | | Mascot |
| 1256.613 | 1256.7491 | 0.1361 | 108 | 1034 | 1043 | TGYNRQQAYR | | | | | Mascot |

| Calc. Mass | Obsrv. Mass | ± da | ± ppm | Start Seq. | End Seq. | Sequence | Ion Score | C. I. % | Modification | Rank | Result Type |
|---|---|---|---|---|---|---|---|---|---|---|---|
| 1403.7028 | 1403.7678 | 0.065 | 46 | 1367 | 1377 | LTMDFFYKNPK | | | | | Mascot |
| 1403.7028 | 1403.7678 | 0.065 | 46 | 1367 | 1377 | LTMDFFYKNPK | | | | | Mascot |
| 1419.6978 | 1419.7617 | 0.0639 | 45 | 1367 | 1377 | LTMDFFYKNPK | | | Oxidation (M)[3] | | Mascot |
| 1419.6978 | 1419.7617 | 0.0639 | 45 | 1367 | 1377 | LTMDFFYKNPK | | | Oxidation (M)[3] | | Mascot |
| 1448.8472 | 1448.9344 | 0.0872 | 60 | 1419 | 1431 | ALSDRGSLILYLK | | | | | Mascot |
| 1454.6224 | 1454.741 | 0.1186 | 82 | 711 | 721 | AFLHCCDEMKK | | | Carbamidomethyl (C)[5,6], Oxidation (M)[9] | | Mascot |
| 1470.889 | 1470.7699 | -0.1191 | -81 | 289 | 302 | IQILKGVGTAELTK | | | | | Mascot |
| 1473.7445 | 1473.6864 | -0.0581 | -39 | 1026 | 1038 | DEAIGHIKTGYNR | | | | | Mascot |
| 1473.8312 | 1473.6864 | -0.1448 | -98 | 596 | 607 | LTQTKIWDVIEK | | | | | Mascot |
| 1492.681 | 1492.7563 | 0.0753 | 50 | 1516 | 1528 | ACESGMDYVYKVK | | | | | Mascot |
| 1600.8363 | 1600.8541 | 0.0178 | 11 | 1138 | 1152 | ELCGAIVGSLPESIR | | | Carbamidomethyl (C)[3] | | Mascot |
| 1600.8363 | 1600.8541 | 0.0178 | 11 | 1138 | 1152 | ELCGAIVGSLPESIR | | | Carbamidomethyl (C)[3] | | Mascot |
| 1677.7611 | 1677.7407 | -0.0204 | -12 | 1513 | 1526 | NIKACESGMDYVYK | | | Carbamidomethyl (C)[5] | | Mascot |
| 1857.9381 | 1857.8822 | -0.0559 | -30 | 209 | 224 | FANTPQKTFTAEFEVK | | | | | Mascot |
| 1916.7605 | 1916.9781 | 0.2176 | 114 | 1489 | 1504 | GDMCQCAEENCSLQKK | | | Carbamidomethyl (C)[4,6], Oxidation (M)[3] | | Mascot |
| 2430.0596 | 2430.2925 | 0.2329 | 96 | 699 | 720 | ATYIGDGPECVKAFLHCC DEMK | | | | | Mascot |
| 2538.2466 | 2538.2896 | 0.043 | 17 | 1529 | 1550 | VKDMELAADADIYQMEV EQVLK | | | | | Mascot |
| 3969.0461 | 3968.8386 | -0.2075 | -52 | 779 | 814 | AMYLKDSITTWQVLAVS MSPTLGICVAEPEEIVVFK | | | | | Mascot |

15 glycerol-3-phosphate dehydrogenase 1 (soluble) [Danio rerio] gi|47550789 38154.7 6.71 9 41 0

**Peptide Information**

| Calc. Mass | Obsrv. Mass | ± da | ± ppm | Start Seq. | End Seq. | Sequence | Ion Score | C. I. % | Modification | Rank | Result Type |
|---|---|---|---|---|---|---|---|---|---|---|---|
| 1460.6184 | 1460.6359 | 0.0175 | 12 | 38 | 48 | MWVYEEMIDGR | | | Oxidation (M)[1,7] | | Mascot |
| 1528.6948 | 1528.7517 | 0.0569 | 37 | 161 | 175 | FCETTIGATNEASGK | | | | | Mascot |
| 1572.7185 | 1572.7897 | 0.0712 | 45 | 38 | 49 | MWVYEEMIDGRK | | | Oxidation (M)[1] | | Mascot |
| 1783.9436 | 1783.834 | -0.1096 | -61 | 121 | 137 | GIDEGPDGLTLISDIIR | | | | | Mascot |
| 1916.9421 | 1916.9781 | 0.036 | 19 | 161 | 178 | FCETTIGATNEASGKLFK | | | | | Mascot |
| 2011.9541 | 2011.9922 | 0.0381 | 19 | 205 | 224 | NIVAVGAGFCDGLGFGD NTK | | | Carbamidomethyl (C)[10] | | Mascot |
| 2158.0347 | 2158.064 | 0.0293 | 14 | 32 | 48 | FEPLVKMWVYEEMIDGR | | | Oxidation (M)[7] | | Mascot |
| 2233.1089 | 2233.0981 | -0.0108 | -5 | 140 | 160 | LEIEVCVLMGANIASEVA DEK | | | | | Mascot |
| 2448.2361 | 2448.2031 | -0.033 | -13 | 138 | 160 | AKLEIEVCVLMGANIASE VADEK | | | Oxidation (M)[11] | | Mascot |

16 PREDICTED: hypothetical protein LOC100537298 [Danio rerio] gi|326667816 6120 8.38 6 40 0

**Peptide Information**

| Calc. Mass | Obsrv. Mass | ± da | ± ppm | Start Seq. | End Seq. | Sequence | Ion Score | C. I. % | Modification | Rank | Result Type |
|---|---|---|---|---|---|---|---|---|---|---|---|
| 1165.5895 | 1165.7506 | 0.1611 | 138 | 10 | 18 | CLRVNYGQR | | | Carbamidomethyl (C)[1] | | Mascot |
| 1165.5895 | 1165.7506 | 0.1611 | 138 | 10 | 18 | CLRVNYGQR | | | Carbamidomethyl (C)[1] | | Mascot |
| 1470.7191 | 1470.7699 | 0.0508 | 35 | 1 | 12 | MAESSIFTRCLR | | | Carbamidomethyl (C)[10] | | Mascot |
| 1619.7776 | 1619.7637 | -0.0139 | -9 | 38 | 51 | VLSCMHLPTCAIMK | | | Carbamidomethyl (C)[4], Oxidation (M)[5] | | Mascot |
| 1619.7776 | 1619.7637 | -0.0139 | -9 | 38 | 51 | VLSCMHLPTCAIMK | | | Carbamidomethyl (C)[4], Oxidation (M)[5] | | Mascot |
| 1916.9829 | 1916.9781 | -0.0048 | -3 | 38 | 54 | VLSCMHLPTCAIMKVTL | | | Carbamidomethyl (C)[4] | | Mascot |
| 2011.8848 | 2011.9922 | 0.1074 | 53 | 19 | 36 | CANDCPDVLYNTAEQSLR | | | | | Mascot |
| 2225.0073 | 2225.1384 | 0.1311 | 59 | 19 | 37 | CANDCPDVLYNTAEQSLRR | | | Carbamidomethyl (C)[1] | | Mascot |
| 2225.0073 | 2225.1384 | 0.1311 | 59 | 19 | 37 | CANDCPDVLYNTAEQSLRR | | | Carbamidomethyl (C)[1] | | Mascot |

17 novel zinc finger protein [Danio rerio] | gi|130496620 | 52184.8 | 6.71 | 13 | 40 | 0

**Peptide Information**

| Calc. Mass | Obsrv. Mass | ± da | ± ppm | Start Seq. | End Seq. | Sequence | Ion Score | C. I. % | Modification | Rank | Result Type |
|---|---|---|---|---|---|---|---|---|---|---|---|
| 835.4341 | 835.476 | 0.0419 | 50 | 141 | 147 | SLQEKCK | | | | | Mascot |
| 974.491 | 974.5182 | 0.0272 | 28 | 104 | 111 | QPVCLVCR | | | Carbamidomethyl (C)[4] | | Mascot |
| 996.4818 | 996.6027 | 0.1209 | 121 | 67 | 74 | NVCESFLK | | | Carbamidomethyl (C)[3] | | Mascot |
| 1045.5887 | 1045.5658 | -0.0229 | -22 | 132 | 140 | KEELNTALK | | | | | Mascot |
| 1165.6285 | 1165.7506 | 0.1221 | 105 | 95 | 103 | LKLFCLEDK | | | Carbamidomethyl (C)[5] | | Mascot |
| 1165.6285 | 1165.7506 | 0.1221 | 105 | 95 | 103 | LKLFCLEDK | | | Carbamidomethyl (C)[5] | | Mascot |
| 1454.746 | 1454.741 | -0.005 | -3 | 438 | 450 | CFPSFRILASSVQ | | | | | Mascot |
| 1473.7961 | 1473.6864 | -0.1097 | -74 | 176 | 186 | QQFEKLHQFLR | | | | | Mascot |
| 1473.7961 | 1473.6864 | -0.1097 | -74 | 176 | 186 | QQFEKLHQFLR | | | | | Mascot |
| 1478.713 | 1478.7196 | 0.0066 | 4 | 20 | 32 | TPVLLSCSHSFCK | | | Carbamidomethyl (C)[7] | | Mascot |
| 1492.6584 | 1492.7563 | 0.0979 | 66 | 81 | 94 | SSGSEEICSLHSEK | | | | | Mascot |
| 1619.8248 | 1619.7637 | -0.0611 | -38 | 159 | 172 | TAQHIKAQAEHTER | | | | | Mascot |
| 1619.8248 | 1619.7637 | -0.0611 | -38 | 159 | 172 | TAQHIKAQAEHTER | | | | | Mascot |
| 1831.0337 | 1831.0667 | 0.033 | 18 | 116 | 131 | HLKHTFRPISEVAPAK | | | | | Mascot |
| 2283.1345 | 2283.2026 | 0.0681 | 30 | 420 | 437 | HLHTYTTTFTHTLYPFFR | | | | | Mascot |
| 2798.3462 | 2798.3958 | 0.0496 | 18 | 20 | 42 | TPVLLSCSHSFCKECLQQFWISK | | | Carbamidomethyl (C)[7,12] | | Mascot |

18 PREDICTED: hypothetical protein LOC797100 [Danio rerio] | gi|326681404 | 37240.7 | 5.09 | 7 | 39 | 0

**Peptide Information**

| Calc. Mass | Obsrv. Mass | ± da | ± ppm | Start | End | Sequence | Ion | C. I. % | Modification | Rank | Result Type |
|---|---|---|---|---|---|---|---|---|---|---|---|

| Calc. Mass | Obsrv. Mass | ± da | ± ppm | Start Seq. | End Seq. | Sequence | Ion Score | C. I. % | Modification | Rank | Result Type |
|---|---|---|---|---|---|---|---|---|---|---|---|
| 835.4164 | 835.476 | 0.0596 | 71 | 257 | 263 | LICEMAR | | | | | Mascot |
| 974.5087 | 974.5182 | 0.0095 | 10 | 227 | 234 | EGQVCLIR | | | Carbamidomethyl (C)[5] | | Mascot |
| 1030.6003 | 1030.5532 | -0.0471 | -46 | 99 | 107 | GSRTDLLLR | | | | | Mascot |
| 1030.6003 | 1030.5532 | -0.0471 | -46 | 99 | 107 | GSRTDLLLR | | | | | Mascot |
| 1136.5074 | 1136.5818 | 0.0744 | 65 | 89 | 98 | LCQECGLDAK | | | Carbamidomethyl (C)[2] | | Mascot |
| 1419.675 | 1419.7617 | 0.0867 | 61 | 125 | 137 | IWAASENPSSTEK | | | | | Mascot |
| 1419.675 | 1419.7617 | 0.0867 | 61 | 125 | 137 | IWAASENPSSTEK | 8 | 0 | | | Mascot |
| 1830.9708 | 1831.0667 | 0.0959 | 52 | 282 | 298 | ETPNNIVNGLPVHADLK | | | | | Mascot |
| 2169.9219 | 2170.0898 | 0.1679 | 77 | 245 | 263 | DMDSHIGNACMKLICEMA R | | | Oxidation (M)[2,11] | | Mascot |
| 2210.9482 | 2211.1226 | 0.1744 | 79 | 245 | 263 | DMDSHIGNACMKLICEMA R | | | Carbamidomethyl (C)[10], Oxidation (M)[2] | | Mascot |
| 2283.9646 | 2284.1956 | 0.231 | 101 | 245 | 263 | DMDSHIGNACMKLICEMA R | | | Carbamidomethyl (C)[10,15], Oxidation (M)[2,11] | | Mascot |

19 IgLON family member 5 [Xenopus (Silurana) tropicalis] gi|187608728 26279.4 8.49 7 38 0

Peptide Information

| Calc. Mass | Obsrv. Mass | ± da | ± ppm | Start Seq. | End Seq. | Sequence | Ion Score | C. I. % | Modification | Rank | Result Type |
|---|---|---|---|---|---|---|---|---|---|---|---|
| 1473.6328 | 1473.6864 | 0.0536 | 36 | 181 | 192 | HFGNYTCFASNR | | | Carbamidomethyl (C)[7] | | Mascot |
| 1473.6328 | 1473.6864 | 0.0536 | 36 | 181 | 192 | HFGNYTCFASNR | | | Carbamidomethyl (C)[7] | | Mascot |
| 1600.7061 | 1600.8541 | 0.148 | 92 | 2 | 15 | VADEGPYTCSFQAR | | | Carbamidomethyl (C)[9] | | Mascot |
| 1600.7061 | 1600.8541 | 0.148 | 92 | 2 | 15 | VADEGPYTCSFQAR | | | Carbamidomethyl (C)[9] | | Mascot |
| 1853.0063 | 1853.015 | 0.0087 | 5 | 219 | 235 | LSGVGLWFCLSISVLMK | | | | | Mascot |
| 2170.0168 | 2170.0898 | 0.073 | 34 | 1 | 19 | MVADEGPYTCSFQARNK PR | | | | | Mascot |
| 2284.2368 | 2284.1956 | -0.0412 | -18 | 109 | 129 | VTVNYPPIITDVKNMPAQ VGK | | | | | Mascot |
| 2331.126 | 2331.2095 | 0.0835 | 36 | 130 | 149 | TAILRCEAMAVPTASFEW YR | | | Oxidation (M)[9] | | Mascot |
| 2719.3296 | 2719.3252 | -0.0044 | -2 | 170 | 192 | SLLLFTNVTEKHFGNYTC FASNR | | | Carbamidomethyl (C)[18] | | Mascot |

20 matrin 3-like [Danio rerio] gi|328447229 129210.8 4.95 15 38 0

Peptide Information

| Calc. Mass | Obsrv. Mass | ± da | ± ppm | Start Seq. | End Seq. | Sequence | Ion Score | C. I. % | Modification | Rank | Result Type |
|---|---|---|---|---|---|---|---|---|---|---|---|
| 876.4971 | 876.4282 | -0.0689 | -79 | 419 | 425 | ELMAIRK | | | Oxidation (M)[3] | | Mascot |
| 1045.5095 | 1045.5658 | 0.0563 | 54 | 68 | 77 | AGSHISNTMK | | | | | Mascot |
| 1051.5935 | 1051.7194 | 0.1259 | 120 | 847 | 856 | IGPFFGSAKK | | | | | Mascot |
| 1148.6278 | 1148.5253 | -0.1025 | -89 | 507 | 515 | LIRMNLCTK | | | Carbamidomethyl (C)[7] | | Mascot |

| Calc. Mass | Obsrv. Mass | ± da | ± ppm | Start Seq. | End Seq. | Sequence | Ion Score | C. I. % | Modification | Rank | Result Type |
|---|---|---|---|---|---|---|---|---|---|---|---|
| 1393.6992 | 1393.7159 | 0.0167 | 12 | 1 | 12 | MSQKLTSDDLQK | | | | | Mascot |
| 1409.6941 | 1409.7487 | 0.0546 | 39 | 1 | 12 | MSQKLTSDDLQK | | | Oxidation (M)[1] | | Mascot |
| 1454.7961 | 1454.741 | -0.0551 | -38 | 302 | 316 | SDGLLGAAPIGSLQR | | | | | Mascot |
| 1473.6283 | 1473.6864 | 0.0581 | 39 | 898 | 908 | MRNECFIEMER | | | Oxidation (M)[1] | | Mascot |
| 1473.6903 | 1473.6864 | -0.0039 | -3 | 1125 | 1136 | KIHCSSQAHYDK | | | Carbamidomethyl (C)[4] | | Mascot |
| 1572.7917 | 1572.7897 | -0.002 | -1 | 834 | 846 | VYHSHTYATIQPR | | | | | Mascot |
| 1661.9485 | 1661.7477 | -0.2008 | -121 | 438 | 451 | RIVSQVVFFSNLPR | | | | | Mascot |
| 2211.1047 | 2211.1226 | 0.0179 | 8 | 814 | 833 | AMVNFYSGNVLPSVCGKPVR | | | Carbamidomethyl (C)[15], Oxidation (M)[2] | | Mascot |
| 2233.0894 | 2233.0981 | 0.0087 | 4 | 272 | 289 | RLLLQMYPDWDPQMPSSR | | | | | Mascot |
| 2509.0315 | 2509.3057 | 0.2742 | 109 | 900 | 920 | NECFIEMERGEDAEQMAEAYK | | | Oxidation (M)[7] | | Mascot |
| 3337.5754 | 3337.7844 | 0.209 | 63 | 1083 | 1111 | LEPSTATLESYDPNVPVGVEFVKMGYYCR | | | Carbamidomethyl (C)[28], Oxidation (M)[24] | | Mascot |
| 3968.8137 | 3968.8386 | 0.0249 | 6 | 19 | 58 | GLLAAAETLNFSMGETHSDHFGSSSQSRGGMSGLGGVDGK | | | Oxidation (M)[13] | | Mascot |

| | | | | | | | | | | |
|---|---|---|---|---|---|---|---|---|---|---|
| 21 | solute carrier family 25, member 32a [Danio rerio] | gi\|41053768 | 36048.6 | 9.25 | 7 | 37 | 0 | 10 | 0 | |

**Peptide Information**

| Calc. Mass | Obsrv. Mass | ± da | ± ppm | Start Seq. | End Seq. | Sequence | Ion Score | C. I. % | Modification | Rank | Result Type |
|---|---|---|---|---|---|---|---|---|---|---|---|
| 864.4318 | 864.5198 | 0.088 | 102 | 177 | 184 | GMMDALVK | | | | | Mascot |
| 1403.7642 | 1403.7678 | 0.0036 | 3 | 162 | 173 | LVLQYNADPSRK | | | | | Mascot |
| 1403.7642 | 1403.7678 | 0.0036 | 3 | 162 | 173 | LVLQYNADPSRK | | | | | Mascot |
| 1473.796 | 1473.6864 | -0.1096 | -74 | 185 | 196 | IYRHEGIPGLYR | | | | | Mascot |
| 1473.796 | 1473.6864 | -0.1096 | -74 | 185 | 196 | IYRHEGIPGLYR | | | | | Mascot |
| 1572.7441 | 1572.7897 | 0.0456 | 29 | 280 | 292 | RTWSNEGVEGFYK | | | | | Mascot |
| 1619.6685 | 1619.7637 | 0.0952 | 59 | 72 | 84 | MRPQYDGMLDCMK | 10 | 0 | Oxidation (M)[1,12] | | Mascot |
| 1619.6685 | 1619.7637 | 0.0952 | 59 | 72 | 84 | MRPQYDGMLDCMK | | | Oxidation (M)[1,8] | | Mascot |
| 1853.0432 | 1853.015 | -0.0282 | -15 | 248 | 263 | IFAVAVTYPYQVVRAR | | | | | Mascot |
| 2283.1226 | 2283.2026 | 0.08 | 35 | 281 | 300 | TWSNEGVEGFYKGMVPNLVR | | | | | Mascot |

| | | | | | | | | | | |
|---|---|---|---|---|---|---|---|---|---|---|
| 22 | catenin alpha-2 [Danio rerio] | gi\|226823293 | 95796.2 | 5.71 | 12 | 37 | 0 | 12 | 0 | |

**Peptide Information**

| Calc. Mass | Obsrv. Mass | ± da | ± ppm | Start Seq. | End Seq. | Sequence | Ion Score | C. I. % | Modification | Rank | Result Type |
|---|---|---|---|---|---|---|---|---|---|---|---|
| 835.4091 | 835.476 | 0.0669 | 80 | 98 | 104 | KQGDTMR | | | | | Mascot |
| 974.5087 | 974.5182 | 0.0095 | 10 | 291 | 299 | IVAECNAVR | | | | | Mascot |
| 1030.6143 | 1030.5532 | -0.0611 | -59 | 2 | 11 | TSATSPILLK | | | | | Mascot |

| Calc. Mass | Obsrv. Mass | ± da | ± ppm | Start Seq. | End Seq. | Sequence | Ion Score | C. I. % | Modification | Rank | Result Type |
|---|---|---|---|---|---|---|---|---|---|---|---|
| 1030.6143 | 1030.5532 | -0.0611 | -59 | 2 | 11 | TSATSPILLK | | | | | Mascot |
| 1045.571 | 1045.5658 | -0.0052 | -5 | 536 | 544 | LLSETVMPR | | | | | Mascot |
| 1132.5779 | 1132.5328 | -0.0451 | -40 | 164 | 174 | DEMAAARGALK | | | | | Mascot |
| 1148.5729 | 1148.5253 | -0.0476 | -41 | 164 | 174 | DEMAAARGALK | | | Oxidation (M)[3] | | Mascot |
| 1419.6104 | 1419.7617 | 0.1513 | 107 | 159 | 170 | DPQCRDEMAAAR | | | Carbamidomethyl (C)[4] | | Mascot |
| 1419.6104 | 1419.7617 | 0.1513 | 107 | 159 | 170 | DPQCRDEMAAAR | 12 | 0 | Carbamidomethyl (C)[4] | | Mascot |
| 1619.8032 | 1619.7637 | -0.0395 | -24 | 742 | 754 | IALYCHQLNICSK | | | Carbamidomethyl (C)[5,11] | | Mascot |
| 1619.8032 | 1619.7637 | -0.0395 | -24 | 742 | 754 | IALYCHQLNICSK | | | Carbamidomethyl (C)[5,11] | | Mascot |
| 1661.7733 | 1661.7477 | -0.0256 | -15 | 718 | 732 | LARAVADQCPDSACK | | | Carbamidomethyl (C)[9,14] | | Mascot |
| 1783.7015 | 1783.834 | 0.1325 | 74 | 677 | 690 | QMCMIMMEMTDFTR | | | Oxidation (M)[2] | | Mascot |
| 1852.8857 | 1853.015 | 0.1293 | 70 | 300 | 315 | QALQDLLSEYMNNTGR | | | | | Mascot |
| 2211.0381 | 2211.1226 | 0.0845 | 38 | 267 | 287 | LESIISGAALMADSSCTR DDR | | | | | Mascot |
| 2273.1553 | 2273.1455 | -0.0098 | -4 | 484 | 504 | CVIALQEGDVDTLDRTAG AIR | | | Carbamidomethyl (C)[1] | | Mascot |
| 2284.0544 | 2284.1956 | 0.1412 | 62 | 267 | 287 | LESIISGAALMADSSCTR DDR | | | Carbamidomethyl (C)[16], Oxidation (M)[11] | | Mascot |

23 novel protein similar to human matrin 3 (MATR3) [Danio rerio] gi|157888662 127451.9 4.93 14 36 0

**Peptide Information**

| Calc. Mass | Obsrv. Mass | ± da | ± ppm | Start Seq. | End Seq. | Sequence | Ion Score | C. I. % | Modification | Rank | Result Type |
|---|---|---|---|---|---|---|---|---|---|---|---|
| 876.4971 | 876.4282 | -0.0689 | -79 | 419 | 425 | ELMAIRK | | | Oxidation (M)[3] | | Mascot |
| 1045.5095 | 1045.5658 | 0.0563 | 54 | 68 | 77 | AGSHISNTMK | | | | | Mascot |
| 1148.6278 | 1148.5253 | -0.1025 | -89 | 507 | 515 | LIRMNLCTK | | | Carbamidomethyl (C)[7] | | Mascot |
| 1393.6992 | 1393.7159 | 0.0167 | 12 | 1 | 12 | MSQKLTSDDLQK | | | | | Mascot |
| 1409.6941 | 1409.7487 | 0.0546 | 39 | 1 | 12 | MSQKLTSDDLQK | | | Oxidation (M)[1] | | Mascot |
| 1454.7961 | 1454.741 | -0.0551 | -38 | 302 | 316 | SDGLLGAAPIGSLQR | | | | | Mascot |
| 1473.6283 | 1473.6864 | 0.0581 | 39 | 882 | 892 | MRNECFIEMER | | | Oxidation (M)[1] | | Mascot |
| 1473.6903 | 1473.6864 | -0.0039 | -3 | 1109 | 1120 | KIHCSSQAHYDK | | | Carbamidomethyl (C)[4] | | Mascot |
| 1619.7925 | 1619.7637 | -0.0288 | -18 | 834 | 847 | VYHSHTYATIQSGR | | | | | Mascot |
| 1619.7925 | 1619.7637 | -0.0288 | -18 | 834 | 847 | VYHSHTYATIQSGR | | | | | Mascot |
| 1661.9485 | 1661.7477 | -0.2008 | -121 | 438 | 451 | RIVSQVVFFSNLPR | | | | | Mascot |
| 2211.1047 | 2211.1226 | 0.0179 | 8 | 814 | 833 | AMVNFYSGNVLPSVCGK PVR | | | Carbamidomethyl (C)[15], Oxidation (M)[2] | | Mascot |
| 2233.0894 | 2233.0981 | 0.0087 | 4 | 272 | 289 | RLLLQMYPDWDPQMPSS R | | | | | Mascot |
| 2509.0315 | 2509.3057 | 0.2742 | 109 | 884 | 904 | NECFIEMERGEDAEQMA EAYK | | | Oxidation (M)[7] | | Mascot |
| 3337.5754 | 3337.7844 | 0.209 | 63 | 1067 | 1095 | LEPSTATLESYDPNVPVG VEFVKMGYYCR | | | Carbamidomethyl (C)[28], Oxidation (M)[24] | | Mascot |
| 3968.8137 | 3968.8386 | 0.0249 | 6 | 19 | 58 | GLLAAAETLNFSMGETHS | | | Oxidation (M)[13] | | Mascot |

DHFGSSSQSRGGMSGL GGVDGK

24 complement component C3A [Danio rerio] gi|2935365 6910.4 9.23 4 36 0

Peptide Information

| Calc. Mass | Obsrv. Mass | ± da | ± ppm | Start Seq. | End Seq. | Sequence | Ion Score | C. I. % | Modification | Rank | Result Type |
|---|---|---|---|---|---|---|---|---|---|---|---|
| 882.4679 | 882.5712 | 0.1033 | 117 | 27 | 34 | DEAIGHIK | | | | | Mascot |
| 1256.613 | 1256.7491 | 0.1361 | 108 | 35 | 44 | TGYNRQQAYR | | | | | Mascot |
| 1473.7445 | 1473.6864 | -0.0581 | -39 | 27 | 39 | DEAIGHIKTGYNR | | | | | Mascot |
| 1473.7445 | 1473.6864 | -0.0581 | -39 | 27 | 39 | DEAIGHIKTGYNR | | | | | Mascot |
| 2798.3528 | 2798.3958 | 0.043 | 15 | 1 | 24 | FMTLPVIATHYLDSTNQW EAIGMK | | | Oxidation (M)[2,23] | | Mascot |

25 PREDICTED: hypothetical protein LOC324021 [Danio rerio] gi|326681253 22209.7 5.13 2 36 0 27 44.616

Peptide Information

| Calc. Mass | Obsrv. Mass | ± da | ± ppm | Start Seq. | End Seq. | Sequence | Ion Score | C. I. % | Modification | Rank | Result Type |
|---|---|---|---|---|---|---|---|---|---|---|---|
| 1761.6448 | 1761.8429 | 0.1981 | 112 | 1 | 16 | MHCCGSLYEGCSGGER | 27 | 44.616 | Carbamidomethyl (C)[3], Oxidation (M)[1] | | Mascot |
| 1761.6448 | 1761.8429 | 0.1981 | 112 | 1 | 16 | MHCCGSLYEGCSGGER | | | Carbamidomethyl (C)[3], Oxidation (M)[1] | | Mascot |
| 3337.3911 | 3337.7844 | 0.3933 | 118 | 1 | 31 | MHCCGSLYEGCSGGER SVSISMASFQHSSSR | | | Carbamidomethyl (C)[3] | | Mascot |

26 PREDICTED: diacylglycerol kinase beta-like, partial [Danio rerio] gi|326674321 85995.9 6.78 12 35 0

Peptide Information

| Calc. Mass | Obsrv. Mass | ± da | ± ppm | Start Seq. | End Seq. | Sequence | Ion Score | C. I. % | Modification | Rank | Result Type |
|---|---|---|---|---|---|---|---|---|---|---|---|
| 835.4672 | 835.476 | 0.0088 | 11 | 448 | 454 | QVYNLAK | | | | | Mascot |
| 842.4229 | 842.5167 | 0.0938 | 111 | 153 | 158 | LEFMFR | | | | | Mascot |
| 864.4032 | 864.5198 | 0.1166 | 135 | 455 | 462 | SGPMPGYR | | | | | Mascot |
| 1045.4264 | 1045.5658 | 0.1394 | 133 | 270 | 278 | QGLCCSFCK | | | Carbamidomethyl (C)[4] | | Mascot |
| 1148.5404 | 1148.5253 | -0.0151 | -13 | 463 | 471 | SCRTVEYYK | | | | | Mascot |
| 1393.6265 | 1393.7159 | 0.0894 | 64 | 534 | 545 | DVQDSSEVMLDR | | | | | Mascot |
| 1409.6213 | 1409.7487 | 0.1274 | 90 | 534 | 545 | DVQDSSEVMLDR | | | Oxidation (M)[9] | | Mascot |
| 1661.7192 | 1661.7477 | 0.0285 | 17 | 349 | 363 | CASHVKPECDCGSLR | | | Carbamidomethyl (C)[1] | | Mascot |
| 1830.8083 | 1831.0667 | 0.2584 | 141 | 270 | 284 | QGLCCSFCKYTVHER | | | Carbamidomethyl (C)[4] | | Mascot |
| 2011.9463 | 2011.9922 | 0.0459 | 23 | 136 | 152 | DMICYLSLLEGGRPEDK | | | Carbamidomethyl (C)[4], Oxidation (M)[2] | | Mascot |
| 2283.2488 | 2283.2026 | -0.0462 | -20 | 115 | 135 | GSCPPLKVTSQSRPLSTV QLK | | | Carbamidomethyl (C)[3] | | Mascot |

| Calc. Mass | Obsrv. Mass | ± da | ± ppm | Start Seq. | End Seq. | Sequence | Ion Score | C. I. % | Modification | Rank | Result Type |
|---|---|---|---|---|---|---|---|---|---|---|---|
| 2284.0591 | 2284.1956 | 0.1365 | 60 | 153 | 171 | LEFMFRLYDTDGNGFLDSK | | | Oxidation (M)[4] | | Mascot |
| 2448.053 | 2448.2031 | 0.1501 | 61 | 303 | 322 | NTEIMHHFWVEGNCPSKCDK | | | Carbamidomethyl (C)[14], Oxidation (M)[5] | | Mascot |

| | | | | | | | | |
|---|---|---|---|---|---|---|---|---|
| 27 | threonine synthase-like 2 [Danio rerio] | gi\|83025070 | 53047 | 6.03 | 9 | 35 | 0 | |

**Peptide Information**

| Calc. Mass | Obsrv. Mass | ± da | ± ppm | Start Seq. | End Seq. | Sequence | Ion Score | C. I. % | Modification | Rank | Result Type |
|---|---|---|---|---|---|---|---|---|---|---|---|
| 842.3937 | 842.5167 | 0.123 | 146 | 2 | 7 | RYCSTR | | | Carbamidomethyl (C)[3] | | Mascot |
| 876.4686 | 876.4282 | -0.0404 | -46 | 423 | 429 | FQQAVQR | | | | | Mascot |
| 1030.4808 | 1030.5532 | 0.0724 | 70 | 413 | 422 | CCIATASPHK | | | | | Mascot |
| 1030.4808 | 1030.5532 | 0.0724 | 70 | 413 | 422 | CCIATASPHK | | | | | Mascot |
| 1393.678 | 1393.7159 | 0.0379 | 27 | 340 | 350 | SLMEEFQKTHK | | | Oxidation (M)[3] | | Mascot |
| 1409.6115 | 1409.7487 | 0.1372 | 97 | 455 | 465 | CSDDWEETLRR | | | | | Mascot |
| 1783.7626 | 1783.834 | 0.0714 | 40 | 450 | 464 | CADLKCSDDWEETLR | | | | | Mascot |
| 2225.2024 | 2225.1384 | -0.064 | -29 | 430 | 449 | AGLTLELPESLQVLDQLETR | | | | | Mascot |
| 2225.2024 | 2225.1384 | -0.064 | -29 | 430 | 449 | AGLTLELPESLQVLDQLETR | | | | | Mascot |
| 2273.1304 | 2273.1455 | 0.0151 | 7 | 53 | 70 | QLLCEVCELFIPEQEIPR | | | Carbamidomethyl (C)[4,7] | | Mascot |
| 2538.2087 | 2538.2896 | 0.0809 | 32 | 401 | 422 | HYQSPVRPGESRCCIATASPHK | | | Carbamidomethyl (C)[13,14] | | Mascot |

| | | | | | | | | |
|---|---|---|---|---|---|---|---|---|
| 28 | PREDICTED: tripartite motif-containing protein 35-like [Danio rerio] | gi\|326678855 | 52250.8 | 6.48 | 12 | 34 | 0 | |

**Peptide Information**

| Calc. Mass | Obsrv. Mass | ± da | ± ppm | Start Seq. | End Seq. | Sequence | Ion Score | C. I. % | Modification | Rank | Result Type |
|---|---|---|---|---|---|---|---|---|---|---|---|
| 835.4341 | 835.476 | 0.0419 | 50 | 141 | 147 | SLQEKCK | | | | | Mascot |
| 974.491 | 974.5182 | 0.0272 | 28 | 104 | 111 | QPVCLVCR | | | Carbamidomethyl (C)[4] | | Mascot |
| 996.4818 | 996.6027 | 0.1209 | 121 | 67 | 74 | NVCESFLK | | | Carbamidomethyl (C)[3] | | Mascot |
| 1045.5887 | 1045.5658 | -0.0229 | -22 | 132 | 140 | KEELNTALK | | | | | Mascot |
| 1165.6285 | 1165.7506 | 0.1221 | 105 | 95 | 103 | LKLFCLEDK | | | Carbamidomethyl (C)[5] | | Mascot |
| 1165.6285 | 1165.7506 | 0.1221 | 105 | 95 | 103 | LKLFCLEDK | | | Carbamidomethyl (C)[5] | | Mascot |
| 1470.6715 | 1470.7699 | 0.0984 | 67 | 330 | 343 | CLCVLGSEGFNSGK | | | Carbamidomethyl (C)[1] | | Mascot |
| 1473.7961 | 1473.6864 | -0.1097 | -74 | 176 | 186 | QQFEKLHQFLR | | | | | Mascot |
| 1473.7961 | 1473.6864 | -0.1097 | -74 | 176 | 186 | QQFEKLHQFLR | | | | | Mascot |
| 1478.713 | 1478.7196 | 0.0066 | 4 | 20 | 32 | TPVLLSCSHSFCK | | | Carbamidomethyl (C)[7] | | Mascot |
| 1492.6584 | 1492.7563 | 0.0979 | 66 | 81 | 94 | SSGSEEICSLHSEK | | | | | Mascot |
| 1619.8248 | 1619.7637 | -0.0611 | -38 | 159 | 172 | TAQHIKAQAEHTER | | | | | Mascot |

| Calc. Mass | Obsrv. Mass | ± da | ± ppm | Start Seq. | End Seq. | Sequence | Ion Score | C. I. % | Modification | Rank | Result Type |
|---|---|---|---|---|---|---|---|---|---|---|---|
| 1619.8248 | 1619.7637 | -0.0611 | -38 | 159 | 172 | TAQHIKAQAEHTER | | | | | Mascot |
| 2282.9534 | 2283.2026 | 0.2492 | 109 | 369 | 386 | GWGFFNSDVWCVCYGW SR | | | Carbamidomethyl (C)[11,13] | | Mascot |
| 2798.3462 | 2798.3958 | 0.0496 | 18 | 20 | 42 | TPVLLSCSHSFCKECLQQ FWISK | | | Carbamidomethyl (C)[7,12] | | Mascot |

| | | | | | | | |
|---|---|---|---|---|---|---|---|
| 29 | TPA: erythropoietin receptor [Danio rerio] | gi\|159885707 | 56496.1 | 5.22 | 9 | 34 | 0 |

**Peptide Information**

| Calc. Mass | Obsrv. Mass | ± da | ± ppm | Start Seq. | End Seq. | Sequence | Ion Score | C. I. % | Modification | Rank | Result Type |
|---|---|---|---|---|---|---|---|---|---|---|---|
| 850.4933 | 850.5481 | 0.0548 | 64 | 193 | 198 | YKVWVR | | | | | Mascot |
| 996.4567 | 996.6027 | 0.146 | 147 | 107 | 114 | DGQMLYNR | | | | | Mascot |
| 1030.5276 | 1030.5532 | 0.0256 | 25 | 325 | 332 | LVRDEDQR | | | | | Mascot |
| 1030.5276 | 1030.5532 | 0.0256 | 25 | 325 | 332 | LVRDEDQR | | | | | Mascot |
| 1132.4768 | 1132.5328 | 0.056 | 49 | 40 | 47 | DFTCFWEK | | | Carbamidomethyl (C)[4] | | Mascot |
| 1136.504 | 1136.5818 | 0.0778 | 68 | 163 | 172 | YAVEDSHMGK | | | | | Mascot |
| 1152.499 | 1152.5769 | 0.0779 | 68 | 163 | 172 | YAVEDSHMGK | | | Oxidation (M)[8] | | Mascot |
| 1460.7897 | 1460.6359 | -0.1538 | -105 | 259 | 270 | KLWPDIPTPEHK | | | | | Mascot |
| 1492.804 | 1492.7563 | -0.0477 | -32 | 173 | 185 | VEETKASTMLVLR | | | Oxidation (M)[9] | | Mascot |
| 2011.9542 | 2011.9922 | 0.038 | 19 | 333 | 349 | SDSGLTEPPHWLMEQLR | | | Oxidation (M)[13] | | Mascot |
| 2618.0842 | 2618.304 | 0.2198 | 84 | 440 | 464 | GPGYAYMAVADSGVSM DYSPMSSSR | | | Oxidation (M)[7,16] | | Mascot |

| | | | | | | | |
|---|---|---|---|---|---|---|---|
| 30 | Shadoo protein [Danio rerio] | gi\|37359664 | 13918 | 10.51 | 5 | 34 | 0 |

**Peptide Information**

| Calc. Mass | Obsrv. Mass | ± da | ± ppm | Start Seq. | End Seq. | Sequence | Ion Score | C. I. % | Modification | Rank | Result Type |
|---|---|---|---|---|---|---|---|---|---|---|---|
| 1030.5024 | 1030.5532 | 0.0508 | 49 | 79 | 86 | RPDDRSER | 3 | 0 | | | Mascot |
| 1030.5024 | 1030.5532 | 0.0508 | 49 | 79 | 86 | RPDDRSER | | | | | Mascot |
| 1165.6 | 1165.7506 | 0.1506 | 129 | 95 | 103 | TNWELYLAR | | | | | Mascot |
| 1165.6 | 1165.7506 | 0.1506 | 129 | 95 | 103 | TNWELYLAR | | | | | Mascot |
| 1830.9645 | 1831.0667 | 0.1022 | 56 | 117 | 132 | LSALLLPINYMMHFAP | | | | | Mascot |
| 2490.2263 | 2490.2053 | -0.021 | -8 | 4 | 25 | AVATCCIFLLLSAFLCDQV MSK | | | Carbamidomethyl (C)[5,6] | | Mascot |
| 2719.3438 | 2719.3252 | -0.0186 | -7 | 4 | 28 | AVATCCIFLLLSAFLCDQV MSKGGR | | | Carbamidomethyl (C)[5], Oxidation (M)[20] | | Mascot |

| | | | | | | | |
|---|---|---|---|---|---|---|---|
| 31 | PREDICTED: zinc finger protein 91-like [Danio rerio] | gi\|326670221 | 74732.2 | 9.59 | 13 | 34 | 0 |

**Peptide Information**

| Calc. Mass | Obsrv. Mass | ± da | ± ppm | Start Seq. | End Seq. | Sequence | Ion Score | C. I. % | Modification | Rank | Result Type |
|---|---|---|---|---|---|---|---|---|---|---|---|

| | | | | | | | | | | | |
|---|---|---|---|---|---|---|---|---|---|---|---|
| 1136.5154 | 1136.5818 | 0.0664 | 58 | 74 | 83 | SFNCSSHLNK | | | | | Mascot |
| 1409.6743 | 1409.7487 | 0.0744 | 53 | 270 | 281 | SFSCLSHLNHHK | | | | | Mascot |
| 1454.6879 | 1454.741 | 0.0531 | 37 | 567 | 578 | EKPFTCTQCGKR | | | Carbamidomethyl (C)[6] | | Mascot |
| 1478.7421 | 1478.7196 | -0.0225 | -15 | 242 | 254 | SFSLSSSLNIHMR | | | | | Mascot |
| 1515.7411 | 1515.7649 | 0.0238 | 16 | 382 | 394 | SFSHSSSLNRHTR | | | | | Mascot |
| 1600.7649 | 1600.8541 | 0.0892 | 56 | 102 | 114 | SFSQLSNLHQHMR | | | Oxidation (M)[12] | | Mascot |
| 1600.7649 | 1600.8541 | 0.0892 | 56 | 102 | 114 | SFSQLSNLHQHMR | | | Oxidation (M)[12] | | Mascot |
| 1619.7668 | 1619.7637 | -0.0031 | -2 | 255 | 269 | IHTGEKPFACTQCGK | | | | | Mascot |
| 1619.7668 | 1619.7637 | -0.0031 | -2 | 255 | 269 | IHTGEKPFACTQCGK | | | | | Mascot |
| 1661.8138 | 1661.7477 | -0.0661 | -40 | 59 | 73 | IHTGEKPFICTQCGK | | | | | Mascot |
| 1761.8159 | 1761.8429 | 0.027 | 15 | 367 | 381 | IHTGEKPFACTQCGR | | | Carbamidomethyl (C)[10,13] | | Mascot |
| 1761.8523 | 1761.8429 | -0.0094 | -5 | 619 | 633 | IHTGEKPFRCTQCGK | | | Carbamidomethyl (C)[10] | | Mascot |
| 1783.7811 | 1783.834 | 0.0529 | 30 | 2 | 17 | MSHTGEKPFLCTECGK | | | Oxidation (M)[1] | | Mascot |
| 2158.0103 | 2158.064 | 0.0537 | 25 | 364 | 381 | HMKIHTGEKPFACTQCG R | | | Carbamidomethyl (C)[13,16] | | Mascot |
| 3337.4717 | 3337.7844 | 0.3127 | 94 | 18 | 45 | SFSCSSHLNQHMRVHTG EKPFTCTQCGK | | | Carbamidomethyl (C)[4,23,26], Oxidation (M)[12] | | Mascot |

| | | | | | | | |
|---|---|---|---|---|---|---|---|
| 32 | PREDICTED: transformation/transcription domain-associated protein [Danio rerio] | gi\|326672350 | 433471.2 | 8.52 | 28 | 34 | 0 |

**Peptide Information**

| Calc. Mass | Obsrv. Mass | ± da | ± ppm | Start Seq. | End Seq. | Sequence | Ion Score | C. I. % | Modification | Rank | Result Type |
|---|---|---|---|---|---|---|---|---|---|---|---|
| 882.5155 | 882.5712 | 0.0557 | 63 | 2182 | 2189 | AVHSLLSR | | | | | Mascot |
| 1030.6044 | 1030.5532 | -0.0512 | -50 | 2608 | 2615 | LWKILSDR | | | | | Mascot |
| 1030.6044 | 1030.5532 | -0.0512 | -50 | 2608 | 2615 | LWKILSDR | | | | | Mascot |
| 1045.5095 | 1045.5658 | 0.0563 | 54 | 3604 | 3612 | EVQGNMVPR | | | Oxidation (M)[6] | | Mascot |
| 1132.553 | 1132.5328 | -0.0202 | -18 | 1696 | 1704 | FLCNMTFLK | | | Oxidation (M)[5] | | Mascot |
| 1136.5253 | 1136.5818 | 0.0565 | 50 | 2020 | 2030 | GMSVDSAQDVK | | | | | Mascot |
| 1152.5295 | 1152.5769 | 0.0474 | 41 | 3815 | 3823 | MDPAWHPWL | | | | | Mascot |
| 1165.5817 | 1165.7506 | 0.1689 | 145 | 606 | 615 | VANCQTVRMK | | | Oxidation (M)[9] | | Mascot |
| 1165.5817 | 1165.7506 | 0.1689 | 145 | 606 | 615 | VANCQTVRMK | | | Oxidation (M)[9] | | Mascot |
| 1256.6018 | 1256.7491 | 0.1473 | 117 | 941 | 950 | SANTEPYYRR | | | | | Mascot |
| 1393.6991 | 1393.7159 | 0.0168 | 12 | 728 | 740 | IVNSSMELAQSAK | | | Oxidation (M)[6] | | Mascot |
| 1409.7648 | 1409.7487 | -0.0161 | -11 | 2032 | 2045 | FRTAAGAVGTVFGR | | | | | Mascot |
| 1419.7527 | 1419.7617 | 0.009 | 6 | 1832 | 1843 | LMTFAWPCLLPK | | | | | Mascot |
| 1419.7527 | 1419.7617 | 0.009 | 6 | 1832 | 1843 | LMTFAWPCLLPK | | | | | Mascot |
| 1448.8293 | 1448.9344 | 0.1051 | 73 | 1147 | 1159 | LGGVVSIKFLMER | | | | | Mascot |
| 1470.6967 | 1470.7699 | 0.0732 | 50 | 958 | 970 | CFLVAMTSLEDNK | | | | | Mascot |

| | | | | | | | | | | | |
|---|---|---|---|---|---|---|---|---|---|---|---|
| 1473.8365 | 1473.6864 | -0.1501 | -102 | 2600 | 2610 | TWVQLFPRLWK | | | | | Mascot |
| 1473.8365 | 1473.6864 | -0.1501 | -102 | 2600 | 2610 | TWVQLFPRLWK | | | | | Mascot |
| 1492.769 | 1492.7563 | -0.0127 | -9 | 1832 | 1843 | LMTFAWPCLLPK | | | Carbamidomethyl (C)[8], Oxidation (M)[2] | | Mascot |
| 1515.7988 | 1515.7649 | -0.0339 | -22 | 3099 | 3112 | AFSAAVQMHDVLVK | | | | | Mascot |
| 1600.8689 | 1600.8541 | -0.0148 | -9 | 2365 | 2377 | SILLVKMMTYIEK | | | Oxidation (M)[7,8] | | Mascot |
| 1600.8689 | 1600.8541 | -0.0148 | -9 | 2365 | 2377 | SILLVKMMTYIEK | | | Oxidation (M)[7,8] | | Mascot |
| 1619.7893 | 1619.7637 | -0.0256 | -16 | 1950 | 1962 | HHLVQHMISAMQR | | | Oxidation (M)[7,11] | | Mascot |
| 1619.7893 | 1619.7637 | -0.0256 | -16 | 1950 | 1962 | HHLVQHMISAMQR | | | Oxidation (M)[7,11] | | Mascot |
| 1661.7839 | 1661.7477 | -0.0362 | -22 | 3311 | 3326 | CYSVAFEKSGAVSDAK | | | | | Mascot |
| 2158.0486 | 2158.064 | 0.0154 | 7 | 931 | 949 | AIETALDCLKSANTEPYYR | | | | | Mascot |
| 2170.1655 | 2170.0898 | -0.0757 | -35 | 1678 | 1695 | NYSEIELLFQLLRAFTGR | | | | | Mascot |
| 2211.0977 | 2211.1226 | 0.0249 | 11 | 619 | 637 | EVLEHFAGVFTMMNPLTFK | | | | | Mascot |
| 2273.1707 | 2273.1455 | -0.0252 | -11 | 3778 | 3798 | AVTAIMTRLHNLAQFEGGESK | | | | | Mascot |
| 2283.0981 | 2283.2026 | 0.1045 | 46 | 3617 | 3634 | EWALHTFPNATDYWTFRK | | | | | Mascot |
| 2284.1384 | 2284.1956 | 0.0572 | 25 | 1175 | 1196 | ALLFVMMDLTGEVSNGAVAMAK | | | Oxidation (M)[6] | | Mascot |
| 2289.1655 | 2289.1477 | -0.0178 | -8 | 3778 | 3798 | AVTAIMTRLHNLAQFEGGESK | | | Oxidation (M)[6] | | Mascot |
| 2289.1655 | 2289.1477 | -0.0178 | -8 | 3778 | 3798 | AVTAIMTRLHNLAQFEGGESK | | | Oxidation (M)[6] | | Mascot |
| 2331.2715 | 2331.2095 | -0.062 | -27 | 3635 | 3654 | MFTIQLALIGLAEFMLHLNR | | | | | Mascot |
| 2448.238 | 2448.2031 | -0.0349 | -14 | 2103 | 2122 | TALRPDMWPSSELKLQWFDK | | | | | Mascot |
| 2470.2798 | 2470.1809 | -0.0989 | -40 | 3273 | 3293 | ELHPTLLSSLEGIVDQMVWFR | | | | | Mascot |
| 2798.3481 | 2798.3958 | 0.0477 | 17 | 1075 | 1098 | GMDPLVLIDAIAICMAYEEKELCK | | | Carbamidomethyl (C)[14,23], Oxidation (M)[2] | | Mascot |

33 MAWD binding protein-like [Danio rerio] gi|57524661 31690.2 5.89 7 33 0

**Peptide Information**

| Calc. Mass | Obsrv. Mass | ± da | ± ppm | Start Seq. | End Seq. | Sequence | Ion Score | C. I. % | Modification | Rank | Result Type |
|---|---|---|---|---|---|---|---|---|---|---|---|
| 864.4308 | 864.5198 | 0.089 | 103 | 186 | 193 | SETTGEIK | | | | | Mascot |
| 1419.7729 | 1419.7617 | -0.0112 | -8 | 173 | 185 | FTSLSPVAEDLLK | | | | | Mascot |
| 1419.7729 | 1419.7617 | -0.0112 | -8 | 173 | 185 | FTSLSPVAEDLLK | 3 | 0 | | | Mascot |
| 1478.7242 | 1478.7196 | -0.0046 | -3 | 159 | 170 | WLMIRLADSCDR | | | | | Mascot |
| 1916.9786 | 1916.9781 | -0.0005 | 0 | 141 | 158 | AVVGDLPIEDVCYSPALR | | | | | Mascot |
| 2283.1768 | 2283.2026 | 0.0258 | 11 | 194 | 214 | SVIVTIKGAPNAQPGYDFYSR | | | | | Mascot |
| 2470.2646 | 2470.1809 | -0.0837 | -34 | 14 | 35 | VPFKGNPAAVCLLENELQDDIK | | | Carbamidomethyl (C)[11] | | Mascot |
| 2509.1777 | 2509.3057 | 0.128 | 51 | 113 | 133 | QHEDSIIMDFPLNTPQPQ | | | | | Mascot |

DQR

| | | | | | | | | | |
|---|---|---|---|---|---|---|---|---|---|
| 34 | PREDICTED: voltage-dependent calcium channel gamma-3 subunit-like [Danio rerio] | gi\|68360472 | 34310.1 | 9.42 | 4 | 33 | 0 | 23 | 0 |

Peptide Information

| Calc. Mass | Obsrv. Mass | ± da | ± ppm | Start Seq. | End Seq. | Sequence | Ion Score | C. I. % | Modification | Rank | Result Type |
|---|---|---|---|---|---|---|---|---|---|---|---|
| 1132.5779 | 1132.5328 | -0.0451 | -40 | 275 | 285 | AAMGALLNSER | | | | | Mascot |
| 1148.5729 | 1148.5253 | -0.0476 | -41 | 275 | 285 | AAMGALLNSER | | | Oxidation (M)[3] | | Mascot |
| 1419.6533 | 1419.7617 | 0.1084 | 76 | 239 | 252 | SSEPASRDASPMGK | | | | | Mascot |
| 1419.6533 | 1419.7617 | 0.1084 | 76 | 239 | 252 | SSEPASRDASPMGK | 23 | 0 | | | Mascot |
| 1473.6647 | 1473.6864 | 0.0217 | 15 | 66 | 78 | TCCLEGTFRGVCK | | | Carbamidomethyl (C)[2] | | Mascot |
| 1473.6647 | 1473.6864 | 0.0217 | 15 | 66 | 78 | TCCLEGTFRGVCK | | | Carbamidomethyl (C)[2] | | Mascot |
| 2538.1782 | 2538.2896 | 0.1114 | 44 | 79 | 99 | KIDHFPEDADYEQDAAEYLLR | | | | | Mascot |

| | | | | | | | |
|---|---|---|---|---|---|---|---|
| 35 | serpin peptidase inhibitor, clade B (ovalbumin), member 1, like 3 [Danio rerio] | gi\|41053961 | 42585.3 | 5.18 | 8 | 33 | 0 |

Peptide Information

| Calc. Mass | Obsrv. Mass | ± da | ± ppm | Start Seq. | End Seq. | Sequence | Ion Score | C. I. % | Modification | Rank | Result Type |
|---|---|---|---|---|---|---|---|---|---|---|---|
| 876.4608 | 876.4282 | -0.0326 | -37 | 74 | 80 | LLTEMNR | | | | | Mascot |
| 1165.6979 | 1165.7506 | 0.0527 | 45 | 160 | 169 | LVLVNAVYFK | | | | | Mascot |
| 1165.6979 | 1165.7506 | 0.0527 | 45 | 160 | 169 | LVLVNAVYFK | | | | | Mascot |
| 1419.7478 | 1419.7617 | 0.0139 | 10 | 250 | 261 | ALTLETLTDWTR | | | | | Mascot |
| 1419.7478 | 1419.7617 | 0.0139 | 10 | 250 | 261 | ALTLETLTDWTR | | | | | Mascot |
| 1478.7283 | 1478.7196 | -0.0087 | -6 | 353 | 364 | FCADHPFLMLIR | | | Oxidation (M)[9] | | Mascot |
| 1600.7502 | 1600.8541 | 0.1039 | 65 | 176 | 187 | FLEEHTHEQQFR | | | | | Mascot |
| 1600.7502 | 1600.8541 | 0.1039 | 65 | 176 | 187 | FLEEHTHEQQFR | | | | | Mascot |
| 2448.1248 | 2448.2031 | 0.0783 | 32 | 52 | 73 | DQMSQTLHFDGAESQIHAGFTK | | | | | Mascot |
| 2618.3799 | 2618.304 | -0.0759 | -29 | 203 | 225 | GRFPLAFIPDVNCQILELPYAGK | | | Carbamidomethyl (C)[13] | | Mascot |
| 3337.5576 | 3337.7844 | 0.2268 | 68 | 52 | 80 | DQMSQTLHFDGAESQIHAGFTKLLTEMNR | | | Oxidation (M)[3,27] | | Mascot |

| | | | | | | | |
|---|---|---|---|---|---|---|---|
| 36 | Mediator of cell motility 1 [Danio rerio] | gi\|160774203 | 33443.4 | 6.35 | 7 | 33 | 0 |

Peptide Information

| Calc. Mass | Obsrv. Mass | ± da | ± ppm | Start Seq. | End Seq. | Sequence | Ion Score | C. I. % | Modification | Rank | Result Type |
|---|---|---|---|---|---|---|---|---|---|---|---|
| 996.4536 | 996.6027 | 0.1491 | 150 | 1 | 8 | MSNRMVCR | | | | | Mascot |
| 1148.5629 | 1148.5253 | -0.0376 | -33 | 238 | 246 | KYHNTICGR | | | Carbamidomethyl (C)[7] | | Mascot |

| | | | | | | | | | | | | |
|---|---|---|---|---|---|---|---|---|---|---|---|---|
| 1473.8788 | 1473.6864 | -0.1924 | -131 | 247 | 260 | HPIGVLLNAVAELK | | | | | Mascot |
| 1473.8788 | 1473.6864 | -0.1924 | -131 | 247 | 260 | HPIGVLLNAVAELK | | | | | Mascot |
| 2210.9592 | 2211.1226 | 0.1634 | 74 | 262 | 280 | NGIDMNFSFLNYAQSSQCR | | | Oxidation (M)[5] | | Mascot |
| 2284.1716 | 2284.1956 | 0.024 | 11 | 219 | 238 | MGMGIIEQLDPISFSNYLKK | | | | | Mascot |
| 2509.1519 | 2509.3057 | 0.1538 | 61 | 199 | 218 | YTYYDESQGEIYRSIEHLDK | | | | | Mascot |
| 3968.7966 | 3968.8386 | 0.042 | 11 | 262 | 297 | NGIDMNFSFLNYAQSSQCRNWSDSSVSYAAGALIVH | | | Oxidation (M)[5] | | Mascot |

37 PREDICTED: retrotransposable element Tf2 155 kDa protein type 1-like [Danio rerio] gi|326673646 147767 9.63 15 33 0

Peptide Information

| Calc. Mass | Obsrv. Mass | ± da | ± ppm | Start Seq. | End Seq. | Sequence | Ion Score | C. I. % | Modification | Rank | Result Type |
|---|---|---|---|---|---|---|---|---|---|---|---|
| 864.421 | 864.5198 | 0.0988 | 114 | 74 | 81 | TAGFSEPR | | | | | Mascot |
| 996.5043 | 996.6027 | 0.0984 | 99 | 903 | 911 | LACHPGVSR | | | Carbamidomethyl (C)[3] | | Mascot |
| 1030.5714 | 1030.5532 | -0.0182 | -18 | 1189 | 1197 | TVCNKLAPK | | | Carbamidomethyl (C)[3] | | Mascot |
| 1030.5714 | 1030.5532 | -0.0182 | -18 | 1189 | 1197 | TVCNKLAPK | | | Carbamidomethyl (C)[3] | | Mascot |
| 1256.7684 | 1256.7491 | -0.0193 | -15 | 1149 | 1159 | QTLLQVGLRTK | | | | | Mascot |
| 1403.6953 | 1403.7678 | 0.0725 | 52 | 814 | 825 | FDFSISYRPGSK | | | | | Mascot |
| 1403.6953 | 1403.7678 | 0.0725 | 52 | 814 | 825 | FDFSISYRPGSK | | | | | Mascot |
| 1454.713 | 1454.741 | 0.028 | 19 | 931 | 943 | NFVLACSVCAVSK | | | Carbamidomethyl (C)[6,9] | | Mascot |
| 1492.7518 | 1492.7563 | 0.0045 | 3 | 920 | 930 | FWWPVMARDIR | | | Oxidation (M)[6] | | Mascot |
| 1572.8744 | 1572.7897 | -0.0847 | -54 | 884 | 896 | LFVPEELRSDVIR | | | | | Mascot |
| 1677.7499 | 1677.7407 | -0.0092 | -5 | 143 | 157 | LPCCSSFDTFSTELK | | | | | Mascot |
| 1831.084 | 1831.0667 | -0.0173 | -9 | 1198 | 1214 | FIGPFSVTKIISPVAVR | | | | | Mascot |
| 1853.0028 | 1853.015 | 0.0122 | 7 | 1002 | 1018 | LPSARETAAAVIDHVFR | | | | | Mascot |
| 2157.9988 | 2158.064 | 0.0652 | 30 | 1272 | 1289 | GRGFQYLVDWEGYGPEER | | | | | Mascot |
| 2448.2253 | 2448.2031 | -0.0222 | -9 | 317 | 340 | SQSPTQAIIDSGAEGNFIDSALVK | | | | | Mascot |
| 2618.3684 | 2618.304 | -0.0644 | -25 | 1007 | 1030 | ETAAAVIDHVFRIHGLPTDVVSDR | | | | | Mascot |
| 2798.3486 | 2798.3958 | 0.0472 | 17 | 259 | 285 | SPFPAVTVVPGQEATPLADPEGMQMGR | | | Oxidation (M)[23] | | Mascot |

38 Erythropoietin receptor [Danio rerio] gi|190337418 57852.8 5.53 9 32 0

Peptide Information

| Calc. Mass | Obsrv. Mass | ± da | ± ppm | Start Seq. | End Seq. | Sequence | Ion Score | C. I. % | Modification | Rank | Result Type |
|---|---|---|---|---|---|---|---|---|---|---|---|
| 850.4933 | 850.5481 | 0.0548 | 64 | 204 | 209 | YKVWVR | | | | | Mascot |

| | | | | | | | | | | | | |
|---|---|---|---|---|---|---|---|---|---|---|---|---|
| 996.4567 | 996.6027 | 0.146 | 147 | 118 | 125 | DGQMLYNR | | | | | | Mascot |
| 1030.5276 | 1030.5532 | 0.0256 | 25 | 336 | 343 | LVRDEDQR | | | | | | Mascot |
| 1030.5276 | 1030.5532 | 0.0256 | 25 | 336 | 343 | LVRDEDQR | | | | | | Mascot |
| 1132.4768 | 1132.5328 | 0.056 | 49 | 51 | 58 | DFTCFWEK | | | Carbamidomethyl (C)[4] | | | Mascot |
| 1136.504 | 1136.5818 | 0.0778 | 68 | 174 | 183 | YAVEDSHMGK | | | | | | Mascot |
| 1152.499 | 1152.5769 | 0.0779 | 68 | 174 | 183 | YAVEDSHMGK | | | Oxidation (M)[8] | | | Mascot |
| 1460.7897 | 1460.6359 | -0.1538 | -105 | 270 | 281 | KLWPDIPTPEHK | | | | | | Mascot |
| 1492.804 | 1492.7563 | -0.0477 | -32 | 184 | 196 | VEETKASTMLVLR | | | Oxidation (M)[9] | | | Mascot |
| 2011.9542 | 2011.9922 | 0.038 | 19 | 344 | 360 | SDSGLTEPPHWLMEQLR | | | Oxidation (M)[13] | | | Mascot |
| 2618.0842 | 2618.304 | 0.2198 | 84 | 451 | 475 | GPGYAYMAVADSGVSM DYSPMSSSR | | | Oxidation (M)[7,16] | | | Mascot |

| | | | | | | | | |
|---|---|---|---|---|---|---|---|---|
| 39 | PREDICTED: transmembrane protein 136-like [Danio rerio] | gi\|125837712 | 27021.8 | 8.05 | 6 | 32 | 0 | |

**Peptide Information**

| Calc. Mass | Obsrv. Mass | ± da | ± ppm | Start Seq. | End Seq. | Sequence | Ion Score | C. I. % | Modification | Rank | Result Type |
|---|---|---|---|---|---|---|---|---|---|---|---|
| 996.4719 | 996.6027 | 0.1308 | 131 | 218 | 224 | YQVWMNR | | | | | Mascot |
| 1030.5581 | 1030.5532 | -0.0049 | -5 | 143 | 150 | WFLKHSGR | | | | | Mascot |
| 1030.5581 | 1030.5532 | -0.0049 | -5 | 143 | 150 | WFLKHSGR | | | | | Mascot |
| 1393.6926 | 1393.7159 | 0.0233 | 17 | 227 | 238 | MMAEVNGLNVKR | | | Oxidation (M)[1,2] | | Mascot |
| 1470.7556 | 1470.7699 | 0.0143 | 10 | 225 | 237 | HKMMAEVNGLNVK | | | | | Mascot |
| 2448.2883 | 2448.2031 | -0.0852 | -35 | 151 | 171 | YDSILGDVVDILFVALFVF MR | | | Oxidation (M)[20] | | Mascot |
| 3968.8665 | 3968.8386 | -0.0279 | -7 | 1 | 36 | MSPLVLGVGVCLAGWIAL YALLCYTNGSCGYEWNC R | | | Carbamidomethyl (C)[11], Oxidation (M)[1] | | Mascot |

| | | | | | | | | |
|---|---|---|---|---|---|---|---|---|
| 40 | Retinal pigment epithelium-specific protein b [Danio rerio] | gi\|62132948 | 34488.1 | 6.37 | 7 | 32 | 0 | |

**Peptide Information**

| Calc. Mass | Obsrv. Mass | ± da | ± ppm | Start Seq. | End Seq. | Sequence | Ion Score | C. I. % | Modification | Rank | Result Type |
|---|---|---|---|---|---|---|---|---|---|---|---|
| 835.3944 | 835.476 | 0.0816 | 98 | 299 | 305 | YINNPSQ | | | | | Mascot |
| 850.4417 | 850.5481 | 0.1064 | 125 | 70 | 76 | FDLKDGR | | | | | Mascot |
| 864.4131 | 864.5198 | 0.1067 | 123 | 217 | 224 | SDPLAMSK | | | Oxidation (M)[6] | | Mascot |
| 1152.6082 | 1152.5769 | -0.0313 | -27 | 199 | 208 | NMSLAYNIVK | | | | | Mascot |
| 1460.7314 | 1460.6359 | -0.0955 | -65 | 1 | 13 | MVSRLEHPAGGYK | | | Oxidation (M)[1] | | Mascot |
| 2170.0847 | 2170.0898 | 0.0051 | 2 | 14 | 33 | KVFESCEELAEPIPAHVS GK | | | | | Mascot |
| 2798.3606 | 2798.3958 | 0.0352 | 13 | 45 | 69 | MGPGLFEIGDEPFNHLFD GQALIHK | | | Oxidation (M)[1] | | Mascot |

41 novel protein similar to tyrosine hydroxylase (th) [Danio rerio] gi|56207960 54280 5.7 9 32 0

Peptide Information

| Calc. Mass | Obsrv. Mass | ± da | ± ppm | Start Seq. | End Seq. | Sequence | Ion Score | C. I. % | Modification | Rank | Result Type |
|---|---|---|---|---|---|---|---|---|---|---|---|
| 842.5206 | 842.5167 | -0.0039 | -5 | 468 | 475 | ALGRLGQK | | | | | Mascot |
| 1030.5463 | 1030.5532 | 0.0069 | 7 | 451 | 459 | IQNALGQMR | | | | | Mascot |
| 1030.5463 | 1030.5532 | 0.0069 | 7 | 451 | 459 | IQNALGQMR | | | | | Mascot |
| 1256.6521 | 1256.7491 | 0.097 | 77 | 74 | 84 | AGKVFETFETK | | | | | Mascot |
| 1403.7212 | 1403.7678 | 0.0466 | 33 | 214 | 225 | TLRSLYPSHACR | | | | | Mascot |
| 1403.7212 | 1403.7678 | 0.0466 | 33 | 214 | 225 | TLRSLYPSHACR | | | | | Mascot |
| 1460.7427 | 1460.6359 | -0.1068 | -73 | 214 | 225 | TLRSLYPSHACR | | | Carbamidomethyl (C)[11] | | Mascot |
| 1473.6791 | 1473.6864 | 0.0073 | 5 | 98 | 109 | RSSGEDLEFFMR | | | | | Mascot |
| 1473.6791 | 1473.6864 | 0.0073 | 5 | 98 | 109 | RSSGEDLEFFMR | | | | | Mascot |
| 1478.7096 | 1478.7196 | 0.01 | 7 | 179 | 190 | RGFISEMAFNYK | | | Oxidation (M)[7] | | Mascot |
| 1600.8363 | 1600.8541 | 0.0178 | 11 | 145 | 158 | KISDLDTCNHLITK | | | | | Mascot |
| 1600.8363 | 1600.8541 | 0.0178 | 11 | 145 | 158 | KISDLDTCNHLITK | | | | | Mascot |
| 1852.9221 | 1853.015 | 0.0929 | 50 | 1 | 17 | FSMKSDSIAQNVPLSGR | | | Oxidation (M)[3] | | Mascot |
| 2283.979 | 2284.1956 | 0.2166 | 95 | 159 | 177 | YDPDLDQDHPGFSDSEYRK | | | | | Mascot |

42 PREDICTED: zinc finger protein 729 [Danio rerio] gi|326668180 104096.8 9.15 18 32 0

Peptide Information

| Calc. Mass | Obsrv. Mass | ± da | ± ppm | Start Seq. | End Seq. | Sequence | Ion Score | C. I. % | Modification | Rank | Result Type |
|---|---|---|---|---|---|---|---|---|---|---|---|
| 1030.5568 | 1030.5532 | -0.0036 | -3 | 91 | 99 | SFAYKSSLK | 6 | 0 | | | Mascot |
| 1030.5568 | 1030.5532 | -0.0036 | -3 | 91 | 99 | SFAYKSSLK | | | | | Mascot |
| 1045.5387 | 1045.5658 | 0.0271 | 26 | 511 | 519 | CYSTALFLK | | | | | Mascot |
| 1136.5339 | 1136.5818 | 0.0479 | 42 | 44 | 52 | SVACFHCKK | | | Carbamidomethyl (C)[4,7] | | Mascot |
| 1148.6721 | 1148.5253 | -0.1468 | -128 | 212 | 220 | IHMLIHTRK | | | | | Mascot |
| 1403.6082 | 1403.7678 | 0.1596 | 114 | 253 | 263 | CEECGKFFAQK | | | Carbamidomethyl (C)[1,4] | | Mascot |
| 1403.6082 | 1403.7678 | 0.1596 | 114 | 253 | 263 | CEECGKFFAQK | | | Carbamidomethyl (C)[1,4] | | Mascot |
| 1470.7457 | 1470.7699 | 0.0242 | 16 | 54 | 65 | FTCKAHLQVHMR | | | | | Mascot |
| 1497.6614 | 1497.7661 | 0.1047 | 70 | 111 | 123 | HTCDQCGKSFPFK | | | | | Mascot |
| 1619.7246 | 1619.7637 | 0.0391 | 24 | 631 | 642 | CFTFTHCWKSFR | | | Carbamidomethyl (C)[1] | | Mascot |
| 1619.7246 | 1619.7637 | 0.0391 | 24 | 631 | 642 | CFTFTHCWKSFR | | | Carbamidomethyl (C)[1] | | Mascot |
| 1761.7795 | 1761.8429 | 0.0634 | 36 | 799 | 813 | CSHCDTSFSQIQHLR | 1 | 0 | | | Mascot |

| | | | | | | | | | | | |
|---|---|---|---|---|---|---|---|---|---|---|---|
| 1761.7795 | 1761.8429 | 0.0634 | 36 | 799 | 813 | CSHCDTSFSQIQHLR | | | | | Mascot |
| 1852.807 | 1853.015 | 0.208 | 112 | 613 | 627 | IQNHHHDEGEHDQTR | | | | | Mascot |
| 1916.9244 | 1916.9781 | 0.0537 | 28 | 387 | 403 | EVKPYTCDQCGKGFTLK | | | | | Mascot |
| 2170.0281 | 2170.0898 | 0.0617 | 28 | 674 | 692 | EHTRIHTGEKPFACAQCGK | | | Carbamidomethyl (C)[14] | | Mascot |
| 2233.031 | 2233.0981 | 0.0671 | 30 | 184 | 202 | IHMLIHTGEKPHECDQCGK | | | Carbamidomethyl (C)[14] | | Mascot |
| 2284.0459 | 2284.1956 | 0.1497 | 66 | 799 | 817 | CSHCDTSFSQIQHLRTHQR | | | | | Mascot |
| 2289.105 | 2289.1477 | 0.0427 | 19 | 156 | 174 | IHMLIHTGEKPHKCDQCGK | | | Carbamidomethyl (C)[14,17] | | Mascot |
| 2289.105 | 2289.1477 | 0.0427 | 19 | 156 | 174 | IHMLIHTGEKPHKCDQCGK | | | Carbamidomethyl (C)[14,17] | | Mascot |
| 2430.1111 | 2430.2925 | 0.1814 | 75 | 694 | 714 | FGCSSNLKQHMMTHTGEKPHK | | | Oxidation (M)[11,12] | | Mascot |
| 2719.2749 | 2719.3252 | 0.0503 | 18 | 180 | 202 | GEVKIHMLIHTGEKPHECDQCGK | | | Carbamidomethyl (C)[18,21], Oxidation (M)[7] | | Mascot |
| 2798.2695 | 2798.3958 | 0.1263 | 45 | 124 | 146 | VYLEQHMLIHTGETLHECDQCGK | | | Carbamidomethyl (C)[18,21] | | Mascot |

| | | | | | | | |
|---|---|---|---|---|---|---|---|
| 43 | calcium-binding mitochondrial carrier protein SCaMC-2-A [Danio rerio] | gi\|47086085 | 52424 | 8.79 | 9 | 32 | 0 |

**Peptide Information**

| Calc. Mass | Obsrv. Mass | ± da | ± ppm | Start Seq. | End Seq. | Sequence | Ion Score | C. I. % | Modification | Rank | Result Type |
|---|---|---|---|---|---|---|---|---|---|---|---|
| 842.4981 | 842.5167 | 0.0186 | 22 | 253 | 260 | IAPETALK | | | | | Mascot |
| 876.4244 | 876.4282 | 0.0038 | 4 | 202 | 209 | TCTAPLDR | | | | | Mascot |
| 1045.5023 | 1045.5658 | 0.0635 | 61 | 261 | 268 | FMAYEQIK | | | Oxidation (M)[2] | | Mascot |
| 1256.6238 | 1256.7491 | 0.1253 | 100 | 212 | 222 | VLMQVHGCQGK | | | Carbamidomethyl (C)[8] | | Mascot |
| 1497.8029 | 1497.7661 | -0.0368 | -25 | 210 | 222 | LKVLMQVHGCQGK | | | Carbamidomethyl (C)[10] | | Mascot |
| 1515.7039 | 1515.7649 | 0.061 | 40 | 223 | 235 | SMCLMSGLTQMIK | | | Carbamidomethyl (C)[3], Oxidation (M)[2] | | Mascot |
| 1852.9877 | 1853.015 | 0.0273 | 15 | 253 | 268 | IAPETALKFMAYEQIK | | | | | Mascot |
| 2430.3755 | 2430.2925 | -0.083 | -34 | 446 | 467 | VIPAVSISYVVYEHIKSTLGVR | | | | | Mascot |
| 2618.2622 | 2618.304 | 0.0418 | 16 | 405 | 428 | MQAQASVEGSSQVSMTGLFKQIMK | | | Oxidation (M)[1,15] | | Mascot |

| | | | | | | | |
|---|---|---|---|---|---|---|---|
| 44 | Zgc:103710 protein [Danio rerio] | gi\|115291999 | 35746.4 | 5.67 | 7 | 32 | 0 |

**Peptide Information**

| Calc. Mass | Obsrv. Mass | ± da | ± ppm | Start Seq. | End Seq. | Sequence | Ion Score | C. I. % | Modification | Rank | Result Type |
|---|---|---|---|---|---|---|---|---|---|---|---|
| 835.4308 | 835.476 | 0.0452 | 54 | 210 | 216 | FTNTPQK | | | | | Mascot |
| 850.4338 | 850.5481 | 0.1143 | 134 | 66 | 72 | NLEIMSK | | | Oxidation (M)[5] | | Mascot |
| 1473.7559 | 1473.6864 | -0.0695 | -47 | 193 | 205 | YMVPQPASPGIWK | | | | | Mascot |
| 1473.7559 | 1473.6864 | -0.0695 | -47 | 193 | 205 | YMVPQPASPGIWK | | | | | Mascot |

| Calc. Mass | Obsrv. Mass | ± da | ± ppm | Start Seq. | End Seq. | Sequence | Ion Score | C. I. % | Modification | Rank | Result Type |
|---|---|---|---|---|---|---|---|---|---|---|---|
| 1761.8993 | 1761.8429 | -0.0564 | -32 | 190 | 205 | SGKYMVPQPASPGIWK | | | Oxidation (M)[5] | | Mascot |
| 1761.8993 | 1761.8429 | -0.0564 | -32 | 190 | 205 | SGKYMVPQPASPGIWK | | | Oxidation (M)[5] | | Mascot |
| 1916.96 | 1916.9781 | 0.0181 | 9 | 304 | 320 | QTILQTFPDISQPSVEN | | | | | Mascot |
| 2490.1619 | 2490.2053 | 0.0434 | 17 | 258 | 279 | YLHGNNVNGHAFVVFGV MEDDR | | | | | Mascot |
| 2618.2568 | 2618.304 | 0.0472 | 18 | 258 | 280 | YLHGNNVNGHAFVVFGV MEDDRK | | | | | Mascot |

45 PREDICTED: hypothetical protein LOC100535704 [Danio rerio] gi|326676179 16043 4.68 1 32 0 27 47.714

Peptide Information

| Calc. Mass | Obsrv. Mass | ± da | ± ppm | Start Seq. | End Seq. | Sequence | Ion Score | C. I. % | Modification | Rank | Result Type |
|---|---|---|---|---|---|---|---|---|---|---|---|
| 1403.8257 | 1403.7678 | -0.0579 | -41 | 113 | 123 | EIIYLDRQILK | | | | | Mascot |
| 1403.8257 | 1403.7678 | -0.0579 | -41 | 113 | 123 | EIIYLDRQILK | 27 | 47.714 | | | Mascot |

46 complement component 3-like isoform 1 [Danio rerio] gi|153792045 188748 7.66 17 32 0

Peptide Information

| Calc. Mass | Obsrv. Mass | ± da | ± ppm | Start Seq. | End Seq. | Sequence | Ion Score | C. I. % | Modification | Rank | Result Type |
|---|---|---|---|---|---|---|---|---|---|---|---|
| 842.5206 | 842.5167 | -0.0039 | -5 | 516 | 523 | GKIIQAGR | | | | | Mascot |
| 1132.568 | 1132.5328 | -0.0352 | -31 | 27 | 35 | RTVWGPNMR | | | Oxidation (M)[8] | | Mascot |
| 1256.627 | 1256.7491 | 0.1221 | 97 | 1058 | 1067 | GYENQLNYKK | | | | | Mascot |
| 1478.5991 | 1478.7196 | 0.1205 | 81 | 694 | 704 | DQLEYDYMEEK | | | Oxidation (M)[8] | | Mascot |
| 1515.8451 | 1515.7649 | -0.0802 | -53 | 862 | 875 | STPSVNVVMVILEK | | | | | Mascot |
| 1600.8845 | 1600.8541 | -0.0304 | -19 | 124 | 136 | YVKLVAQFSNIYR | | | | | Mascot |
| 1600.8845 | 1600.8541 | -0.0304 | -19 | 124 | 136 | YVKLVAQFSNIYR | | | | | Mascot |
| 1661.9696 | 1661.7477 | -0.2219 | -134 | 61 | 75 | QNILVEGHNLAKVVK | | | | | Mascot |
| 1853.0167 | 1853.015 | -0.0017 | -1 | 269 | 284 | LEVEITARYLYGAQVK | | | | | Mascot |
| 2012.0658 | 2011.9922 | -0.0736 | -37 | 259 | 276 | TSHLSLDAEKLEVEITAR | | | | | Mascot |
| 2158.1763 | 2158.064 | -0.1123 | -52 | 2 | 19 | LLQLLLWATLLCQICDGK | | | Carbamidomethyl (C)[12,15] | | Mascot |
| 2233.1533 | 2233.0981 | -0.0552 | -25 | 911 | 932 | TGELPLQVTAVAASFMG QDAVK | | | | | Mascot |
| 2289.2168 | 2289.1477 | -0.0691 | -30 | 1 | 19 | MLLQLLLWATLLCQICDG K | | | Carbamidomethyl (C)[13,16] | | Mascot |
| 2289.2168 | 2289.1477 | -0.0691 | -30 | 1 | 19 | MLLQLLLWATLLCQICDG K | | | Carbamidomethyl (C)[13,16] | | Mascot |
| 2331.2588 | 2331.2095 | -0.0493 | -21 | 855 | 875 | NSTLEVRSTPSVNVVMVI LEK | | | Oxidation (M)[16] | | Mascot |
| 2448.0442 | 2448.2031 | 0.1589 | 65 | 650 | 673 | DNMGVFSDAGLMFHSST GGSTDSK | | | | | Mascot |
| 2470.2581 | 2470.1809 | -0.0772 | -31 | 1190 | 1212 | LTRPYTAAIACYALAVSN NACVK | | | Carbamidomethyl (C)[11] | | Mascot |
| 2618.46 | 2618.304 | -0.156 | -60 | 406 | 428 | VTFLKRPISVHSGTINIHIN | | | Oxidation (M)[21] | | Mascot |

| | | | | | | | | | | | | |
|---|---|---|---|---|---|---|---|---|---|---|---|---|
| 2798.4797 | 2798.3958 | -0.0839 | -30 | 1571 | 1593 | MPK VKLISISQSHYYQYEMEIV QVIK | | | | | Mascot |

47 PREDICTED: transcriptional repressor NF-X1-like [Danio rerio] gi\|326669534 93150.5 8.23 17 31 0

**Peptide Information**

| Calc. Mass | Obsrv. Mass | ± da | ± ppm | Start Seq. | End Seq. | Sequence | Ion Score | C. I. % | Modification | Rank | Result Type |
|---|---|---|---|---|---|---|---|---|---|---|---|
| 842.4301 | 842.5167 | 0.0866 | 103 | 442 | 448 | LSCGLHR | | | Carbamidomethyl (C)[3] | | Mascot |
| 882.4614 | 882.5712 | 0.1098 | 124 | 552 | 559 | LLPCGSHR | | | | | Mascot |
| 1148.5074 | 1148.5253 | 0.0179 | 16 | 336 | 346 | ACTDPIPSCGK | | | Carbamidomethyl (C)[2] | | Mascot |
| 1393.5544 | 1393.7159 | 0.1615 | 116 | 156 | 168 | TEIPHSCGDMCGK | | | Oxidation (M)[10] | | Mascot |
| 1403.7311 | 1403.7678 | 0.0367 | 26 | 785 | 798 | SACPNTSLAALISR | | | | | Mascot |
| 1403.7311 | 1403.7678 | 0.0367 | 26 | 785 | 798 | SACPNTSLAALISR | | | | | Mascot |
| 1409.6035 | 1409.7487 | 0.1452 | 103 | 667 | 678 | LECDQECATLER | | | | | Mascot |
| 1460.7526 | 1460.6359 | -0.1167 | -80 | 785 | 798 | SACPNTSLAALISR | | | Carbamidomethyl (C)[3] | | Mascot |
| 1478.7454 | 1478.7196 | -0.0258 | -17 | 312 | 325 | LVRSCSCGQTPLSK | | | | | Mascot |
| 1497.6638 | 1497.7661 | 0.1023 | 68 | 620 | 633 | ETVPCADAASSYQR | | | | | Mascot |
| 1572.6884 | 1572.7897 | 0.1013 | 64 | 8 | 21 | GAEEEGERPEEANR | | | | | Mascot |
| 1619.6975 | 1619.7637 | 0.0662 | 41 | 156 | 169 | TEIPHSCGDMCGKK | | | Carbamidomethyl (C)[7,11] | | Mascot |
| 1619.6975 | 1619.7637 | 0.0662 | 41 | 156 | 169 | TEIPHSCGDMCGKK | | | Carbamidomethyl (C)[7,11] | | Mascot |
| 1761.7175 | 1761.8429 | 0.1254 | 71 | 420 | 433 | HKCGELCCVDMEHK | | | Carbamidomethyl (C)[3,7], Oxidation (M)[11] | | Mascot |
| 1761.7175 | 1761.8429 | 0.1254 | 71 | 420 | 433 | HKCGELCCVDMEHK | | | Carbamidomethyl (C)[3,7], Oxidation (M)[11] | | Mascot |
| 1783.76 | 1783.834 | 0.074 | 41 | 270 | 285 | KFDGSGYFSCCKPCGK | | | Carbamidomethyl (C)[10] | | Mascot |
| 1857.8226 | 1857.8822 | 0.0596 | 32 | 434 | 448 | CSMICGYKLSCGLHR | | | Carbamidomethyl (C)[1,5,11], Oxidation (M)[3] | | Mascot |
| 2169.8933 | 2170.0898 | 0.1965 | 91 | 294 | 311 | CQQSCHPGECQPCPLSP K | | | Carbamidomethyl (C)[1,5,10,13] | | Mascot |
| 2272.9771 | 2273.1455 | 0.1684 | 74 | 391 | 409 | EVPCAAIQTEEDMVFTCE K | | | Carbamidomethyl (C)[4,17], Oxidation (M)[13] | | Mascot |
| 2283.8704 | 2284.1956 | 0.3252 | 142 | 422 | 441 | CGELCCVDMEHKCSMIC GYK | | | Oxidation (M)[9,15] | | Mascot |
| 2538.1467 | 2538.2896 | 0.1429 | 56 | 294 | 314 | CQQSCHPGECQPCPLSP KLVR | | | Carbamidomethyl (C)[1,5,10,13] | | Mascot |

48 novel protein similar to vertebrate complement component 3 (C3) [Danio rerio] gi\|169153736 187400.5 8.04 17 31 0

**Peptide Information**

| Calc. Mass | Obsrv. Mass | ± da | ± ppm | Start Seq. | End Seq. | Sequence | Ion Score | C. I. % | Modification | Rank | Result Type |
|---|---|---|---|---|---|---|---|---|---|---|---|
| 835.4494 | 835.476 | 0.0266 | 32 | 140 | 146 | VMPVSFR | | | | | Mascot |
| 842.5206 | 842.5167 | -0.0039 | -5 | 516 | 523 | GKIIQAGR | | | | | Mascot |

| | | | | | | | | | | | |
|---|---|---|---|---|---|---|---|---|---|---|---|
| 1132.6361 | 1132.5328 | -0.1033 | -91 | 431 | 440 | IPYGNSQILK | | | | | Mascot |
| 1256.627 | 1256.7491 | 0.1221 | 97 | 1068 | 1077 | GYENQLNYKK | | | | | Mascot |
| 1515.8451 | 1515.7649 | -0.0802 | -53 | 862 | 875 | STPSVNVVMVILEK | | | | | Mascot |
| 1528.739 | 1528.7517 | 0.0127 | 8 | 169 | 181 | GFVSDLEFRSSER | | | | | Mascot |
| 1600.8845 | 1600.8541 | -0.0304 | -19 | 124 | 136 | YVKLVAQFSNIYR | | | | | Mascot |
| 1600.8845 | 1600.8541 | -0.0304 | -19 | 124 | 136 | YVKLVAQFSNIYR | | | | | Mascot |
| 1661.9696 | 1661.7477 | -0.2219 | -134 | 61 | 75 | QNILVEGHNLAKVVK | | | | | Mascot |
| 1853.0167 | 1853.015 | -0.0017 | -1 | 269 | 284 | LEVEITARYLYGAQVK | | | | | Mascot |
| 2012.0658 | 2011.9922 | -0.0736 | -37 | 259 | 276 | TSHLSLDAEKLEVEITAR | | | | | Mascot |
| 2158.1914 | 2158.064 | -0.1274 | -59 | 411 | 429 | RPISVHSGTINIHINMPKK | | | Oxidation (M)[16] | | Mascot |
| 2233.1533 | 2233.0981 | -0.0552 | -25 | 911 | 932 | TGELPLQVTAVAASFMG QDAVK | | | | | Mascot |
| 2331.2588 | 2331.2095 | -0.0493 | -21 | 855 | 875 | NSTLEVRSTPSVNVVMVI LEK | | | Oxidation (M)[16] | | Mascot |
| 2448.0442 | 2448.2031 | 0.1589 | 65 | 650 | 673 | DNMGVFSDAGLMFHSST GGSTDSK | | | | | Mascot |
| 2470.2581 | 2470.1809 | -0.0772 | -31 | 1200 | 1222 | LTRPYTAAIACYALAVSN NACVK | | | Carbamidomethyl (C)[11] | | Mascot |
| 2618.46 | 2618.304 | -0.156 | -60 | 406 | 428 | VTFLKRPISVHSGTINIHIN MPK | | | Oxidation (M)[21] | | Mascot |
| 2798.4797 | 2798.3958 | -0.0839 | -30 | 1559 | 1581 | VKLISISQSHYYQYEMEIV QVIK | | | | | Mascot |

| | | | | | | | | | | |
|---|---|---|---|---|---|---|---|---|---|---|
| 49 | PREDICTED: phospholipase C-like 2, partial [Danio rerio] | gi\|292630098 | 111384 | 5.95 | 1 | | | 21 | 0 | |

Peptide Information

| Calc. Mass | Obsrv. Mass | ± da | ± ppm | Start Seq. | End Seq. | Sequence | Ion Score | C. I. % | Modification | Rank | Result Type |
|---|---|---|---|---|---|---|---|---|---|---|---|
| 1403.829 | 1403.7678 | -0.0612 | -44 | 248 | 260 | KSISLCTAVQLIK | 23 | 0 | | 3 | Mascot |

| | | | | | | | | | | |
|---|---|---|---|---|---|---|---|---|---|---|
| 50 | PREDICTED: serine/threonine-protein kinase pim-3-like [Danio rerio] | gi\|292621981 | 33318.9 | 6.25 | 1 | | | 18 | 0 | |

Peptide Information

| Calc. Mass | Obsrv. Mass | ± da | ± ppm | Start Seq. | End Seq. | Sequence | Ion Score | C. I. % | Modification | Rank | Result Type |
|---|---|---|---|---|---|---|---|---|---|---|---|
| 1419.7703 | 1419.7617 | -0.0086 | -6 | 160 | 173 | GGSLGETVARGLFR | 18 | 0 | | 3 | Mascot |

| | | | | | |
|---|---|---|---|---|---|
| Gel Idx/Pos | 196/H23 | Instr./Gel Origin | BA2060/110707A | Process Status | Analysis Succeeded |
| Plate [#] Name | [1] 31496 | Instrument Sample Name | | Spectra | 11 |

| Rank | Protein Name | Accession No. | Protein MW | Protein PI | Pep. Count | Protein Score | Protein Score C. I. % | Total Ion Score | Total Ion C. I. % |
|---|---|---|---|---|---|---|---|---|---|
| 1 | alpha-aminoadipic semialdehyde dehydrogenase [Danio rerio] | gi\|47086597 | 55568.2 | 5.94 | 18 | 443 | 100 | 328 | 100 |

Peptide Information

| Calc. Mass | Obsrv. Mass | ± da | ± ppm | Start Seq. | End Seq. | Sequence | Ion Score | C. I. % | Modification | Rank | Result Type |
|---|---|---|---|---|---|---|---|---|---|---|---|
| 942.5479 | 942.5549 | 0.007 | 7 | 88 | 95 | QIGEALRR | | | | | Mascot |
| 982.5356 | 982.5555 | 0.0199 | 20 | 73 | 81 | VWADVPAPK | | | | | Mascot |
| 1107.5286 | 1107.5391 | 0.0105 | 9 | 253 | 261 | QVAMMVQER | | | Oxidation (M)[4] | | Mascot |
| 1123.5234 | 1123.5309 | 0.0075 | 7 | 253 | 261 | QVAMMVQER | | | Oxidation (M)[4,5] | | Mascot |
| 1230.6729 | 1230.6799 | 0.007 | 6 | 501 | 511 | DLPLAQGIKFE | | | | | Mascot |
| 1402.7689 | 1402.7797 | 0.0108 | 8 | 239 | 252 | VGLLSFTGSTHVGK | | | | | Mascot |
| 1460.6512 | 1460.64 | -0.0112 | -8 | 473 | 486 | HTGGGRESGSDSWK | | | | | Mascot |
| 1509.8015 | 1509.7767 | -0.0248 | -16 | 97 | 110 | IKALGSLMSLEMGK | | | Oxidation (M)[8,12] | | Mascot |
| 1523.7588 | 1523.7728 | 0.014 | 9 | 54 | 66 | QATIAEYEETIQK | | | | | Mascot |
| 1560.838 | 1560.8516 | 0.0136 | 9 | 348 | 361 | QAVQQYLAAIEQAK | 82 | 100 | | | Mascot |
| 1560.838 | 1560.8516 | 0.0136 | 9 | 348 | 361 | QAVQQYLAAIEQAK | | | | | Mascot |
| 1582.7019 | 1582.8284 | 0.1265 | 80 | 412 | 424 | TEEEAFSWNNEVK | | | | | Mascot |
| 1706.853 | 1706.8643 | 0.0113 | 7 | 308 | 321 | LMLHESIHDEVVER | | | | | Mascot |
| 1718.9113 | 1718.9227 | 0.0114 | 7 | 2 | 15 | STLLINQPEYNWLK | 69 | 99.997 | | | Mascot |
| 1718.9113 | 1718.9227 | 0.0114 | 7 | 2 | 15 | STLLINQPEYNWLK | | | | | Mascot |
| 1722.8479 | 1722.8663 | 0.0184 | 11 | 308 | 321 | LMLHESIHDEVVER | | | Oxidation (M)[2] | | Mascot |
| 1857.8654 | 1857.8779 | 0.0125 | 7 | 410 | 424 | FKTEEEAFSWNNEVK | 91 | 100 | | | Mascot |
| 1857.8654 | 1857.8779 | 0.0125 | 7 | 410 | 424 | FKTEEEAFSWNNEVK | | | | | Mascot |
| 1862.9541 | 1862.9628 | 0.0087 | 5 | 307 | 321 | RLMLHESIHDEVVER | | | | | Mascot |
| 1878.949 | 1878.9661 | 0.0171 | 9 | 307 | 321 | RLMLHESIHDEVVER | | | Oxidation (M)[3] | | Mascot |
| 1923.96 | 1923.9751 | 0.0151 | 8 | 331 | 347 | IGDPWDPNTLYGPLHTK | 86 | 100 | | | Mascot |
| 1923.96 | 1923.9751 | 0.0151 | 8 | 331 | 347 | IGDPWDPNTLYGPLHTK | | | | | Mascot |
| 2506.1877 | 2506.2161 | 0.0284 | 11 | 447 | 472 | GSDCGIVNVNIPTSGAEI GGAFGGEK | | | Carbamidomethyl (C)[4] | | Mascot |
| 3355.4556 | 3355.4919 | 0.0363 | 11 | 21 | 51 | EDNDGVYNGSWGGQGE VVTSYCPANNEPIAR | | | Carbamidomethyl (C)[22] | | Mascot |
| 3895.7827 | 3895.8374 | 0.0547 | 14 | 16 | 51 | ELGLKEDNDGVYNGSW GGQGEVVTSYCPANNEP IAR | | | Carbamidomethyl (C)[27] | | Mascot |

| | | | | | | | | |
|---|---|---|---|---|---|---|---|---|
| 2 | PREDICTED: alpha-(1,3)-fucosyltransferase-like [Danio rerio] | gi\|292612044 | 43290 | 7.62 | 10 | 50 | 54.462 | |

Peptide Information

| Calc. Mass | Obsrv. Mass | ± da | ± ppm | Start Seq. | End Seq. | Sequence | Ion Score | C. I. % | Modification | Rank | Result Type |
|---|---|---|---|---|---|---|---|---|---|---|---|
| 850.4451 | 850.5497 | 0.1046 | 123 | 231 | 237 | ETLTRCK | | | | | Mascot |

| | | | | | | | | | | | |
|---|---|---|---|---|---|---|---|---|---|---|---|
| 982.5138 | 982.5555 | 0.0417 | 42 | 336 | 343 | ACQYIRTK | | | | | Mascot |
| 1169.5619 | 1169.6438 | 0.0819 | 70 | 70 | 79 | KFDLNSCSAK | | | Carbamidomethyl (C)[7] | | Mascot |
| 1185.6738 | 1185.6406 | -0.0332 | -28 | 209 | 218 | KHIDIYALGR | | | | | Mascot |
| 1303.61 | 1303.772 | 0.162 | 124 | 325 | 335 | LVSYPEEHACR | | | | | Mascot |
| 1582.8071 | 1582.8284 | 0.0213 | 13 | 223 | 235 | QLSDTQYKETLTR | | | | | Mascot |
| 1761.874 | 1761.8502 | -0.0238 | -14 | 184 | 197 | DKLVCWIVSNWNER | | | | | Mascot |
| 2284.2739 | 2284.1958 | -0.0781 | -34 | 51 | 69 | EKPLENSTILLIWIWPFGK | | | | | Mascot |
| 2284.2739 | 2284.1958 | -0.0781 | -34 | 51 | 69 | EKPLENSTILLIWIWPFGK | | | | | Mascot |
| 2297.0762 | 2297.2095 | 0.1333 | 58 | 238 | 255 | FYLSFENTNLHYDYITEK | | | | | Mascot |
| 2528.1802 | 2528.1924 | 0.0122 | 5 | 236 | 255 | CKFYLSFENTNLHYDYITEK | | | | | Mascot |

3 Sb:cb283 protein [Danio rerio] gi|81097694 57058.3 8.43 12 44 0

**Peptide Information**

| Calc. Mass | Obsrv. Mass | ± da | ± ppm | Start Seq. | End Seq. | Sequence | Ion Score | C. I. % | Modification | Rank | Result Type |
|---|---|---|---|---|---|---|---|---|---|---|---|
| 1062.5691 | 1062.6975 | 0.1284 | 121 | 101 | 110 | FRDAAANGIK | | | | | Mascot |
| 1107.5728 | 1107.5391 | -0.0337 | -30 | 1 | 9 | GLLDFCRAR | | | Carbamidomethyl (C)[6] | | Mascot |
| 1123.5167 | 1123.5309 | 0.0142 | 13 | 438 | 445 | REDYEFHK | | | | | Mascot |
| 1169.6201 | 1169.6438 | 0.0237 | 20 | 207 | 216 | VAEYVQSVFK | | | | | Mascot |
| 1279.6437 | 1279.7867 | 0.143 | 112 | 244 | 254 | CANAVPRHQAR | | | Carbamidomethyl (C)[1] | | Mascot |
| 1582.724 | 1582.8284 | 0.1044 | 66 | 54 | 67 | APLQDYSAVDCCIK | | | Carbamidomethyl (C)[11] | | Mascot |
| 1619.9227 | 1619.7642 | -0.1585 | -98 | 68 | 82 | DEVVVLRVPGLPGNR | | | | | Mascot |
| 1761.9493 | 1761.8502 | -0.0991 | -56 | 423 | 438 | AGEVLGDLFEVSTIRR | | | | | Mascot |
| 1903.9265 | 1903.9333 | 0.0068 | 4 | 234 | 250 | EYPCLAAVNRCANAVPR | | | Carbamidomethyl (C)[4] | | Mascot |
| 2025.9116 | 2025.9406 | 0.029 | 14 | 83 | 99 | LVCSCTGPVNRDYDDVR | | | Carbamidomethyl (C)[3,5] | | Mascot |
| 2211.0276 | 2211.1243 | 0.0967 | 44 | 354 | 373 | VGNTDAEGRMVMVDLLCEMK | | | | | Mascot |
| 2211.0276 | 2211.1243 | 0.0967 | 44 | 354 | 373 | VGNTDAEGRMVMVDLLCEMK | | | | | Mascot |
| 2283.2642 | 2283.2009 | -0.0633 | -28 | 112 | 132 | ALKAGLQRPLLVCPPNSSYAK | | | Carbamidomethyl (C)[13] | | Mascot |
| 2284.0439 | 2284.1958 | 0.1519 | 67 | 354 | 373 | VGNTDAEGRMVMVDLLCEMK | | | Carbamidomethyl (C)[17], Oxidation (M)[10] | | Mascot |
| 2284.0439 | 2284.1958 | 0.1519 | 67 | 354 | 373 | VGNTDAEGRMVMVDLLCEMK | | | Carbamidomethyl (C)[17], Oxidation (M)[10] | | Mascot |
| 2300.0388 | 2300.1975 | 0.1587 | 69 | 354 | 373 | VGNTDAEGRMVMVDLLCEMK | | | Carbamidomethyl (C)[17], Oxidation (M)[10,12] | | Mascot |

4 hypothetical protein LOC767682 [Danio rerio] gi|115495185 55486.4 7.89 11 41 0

**Peptide Information**

| Calc. Mass | Obsrv. Mass | ± da | ± ppm | Start Seq. | End Seq. | Sequence | Ion Score | C. I. % | Modification | Rank | Result Type |
|---|---|---|---|---|---|---|---|---|---|---|---|

| Calc. Mass | Obsrv. Mass | ± da | ± ppm | Start Seq. | End Seq. | Sequence | Ion Score | C. I. % | Modification | Rank | Result Type |
|---|---|---|---|---|---|---|---|---|---|---|---|
| 1062.5691 | 1062.6975 | 0.1284 | 121 | 88 | 97 | FRDAAANGIK | | | | | Mascot |
| 1123.5167 | 1123.5309 | 0.0142 | 13 | 425 | 432 | REDYEFHK | | | | | Mascot |
| 1169.6201 | 1169.6438 | 0.0237 | 20 | 194 | 203 | VAEYVQSVFK | | | | | Mascot |
| 1279.6437 | 1279.7867 | 0.143 | 112 | 231 | 241 | CANAVPRHQAR | | | Carbamidomethyl (C)[1] | | Mascot |
| 1582.724 | 1582.8284 | 0.1044 | 66 | 41 | 54 | APLQDYSAVDCCIK | | | Carbamidomethyl (C)[11] | | Mascot |
| 1619.9227 | 1619.7642 | -0.1585 | -98 | 55 | 69 | DEVVVLRVPGLPGNR | | | | | Mascot |
| 1761.9493 | 1761.8502 | -0.0991 | -56 | 410 | 425 | AGEVLGDLFEVSTIRR | | | | | Mascot |
| 1903.9265 | 1903.9333 | 0.0068 | 4 | 221 | 237 | EYPCLAAVNRCANAVPR | | | Carbamidomethyl (C)[4] | | Mascot |
| 2025.9116 | 2025.9406 | 0.029 | 14 | 70 | 86 | LVCSCTGPVNRDYDDVR | | | Carbamidomethyl (C)[3,5] | | Mascot |
| 2211.0276 | 2211.1243 | 0.0967 | 44 | 341 | 360 | VGNTDAEGRMVMVDLLCEMK | | | | | Mascot |
| 2211.0276 | 2211.1243 | 0.0967 | 44 | 341 | 360 | VGNTDAEGRMVMVDLLCEMK | | | | | Mascot |
| 2283.2642 | 2283.2009 | -0.0633 | -28 | 99 | 119 | ALKAGLQRPLLVCPPNSSYAK | | | Carbamidomethyl (C)[13] | | Mascot |
| 2284.0439 | 2284.1958 | 0.1519 | 67 | 341 | 360 | VGNTDAEGRMVMVDLLCEMK | | | Carbamidomethyl (C)[17], Oxidation (M)[10] | | Mascot |
| 2284.0439 | 2284.1958 | 0.1519 | 67 | 341 | 360 | VGNTDAEGRMVMVDLLCEMK | | | Carbamidomethyl (C)[17], Oxidation (M)[10] | | Mascot |
| 2300.0388 | 2300.1975 | 0.1587 | 69 | 341 | 360 | VGNTDAEGRMVMVDLLCEMK | | | Carbamidomethyl (C)[17], Oxidation (M)[10,12] | | Mascot |

5 PREDICTED: zinc finger protein 271-like [Danio rerio] gi|326666948 40261.5 9.08 11 39 0

**Peptide Information**

| Calc. Mass | Obsrv. Mass | ± da | ± ppm | Start Seq. | End Seq. | Sequence | Ion Score | C. I. % | Modification | Rank | Result Type |
|---|---|---|---|---|---|---|---|---|---|---|---|
| 996.4641 | 996.6023 | 0.1382 | 139 | 233 | 241 | LFTCPQCGK | | | | | Mascot |
| 996.4641 | 996.6023 | 0.1382 | 139 | 233 | 241 | LFTCPQCGK | | | | | Mascot |
| 1185.5358 | 1185.6406 | 0.1048 | 88 | 298 | 307 | NFYCSSHLSK | | | | | Mascot |
| 1230.5063 | 1230.6799 | 0.1736 | 141 | 64 | 73 | NCFICTQCGK | | | Carbamidomethyl (C)[2,5] | | Mascot |
| 1574.7493 | 1574.7871 | 0.0378 | 24 | 270 | 282 | SFSQSSHLNKHMR | | | Oxidation (M)[12] | | Mascot |
| 1706.7988 | 1706.8643 | 0.0655 | 38 | 314 | 328 | IHTGEKPFTCTQCGK | | | Carbamidomethyl (C)[10] | | Mascot |
| 1718.8352 | 1718.9227 | 0.0875 | 51 | 227 | 241 | IHTGEKLFTCPQCGK | | | Carbamidomethyl (C)[10] | | Mascot |
| 1718.8352 | 1718.9227 | 0.0875 | 51 | 227 | 241 | IHTGEKLFTCPQCGK | | | Carbamidomethyl (C)[10] | | Mascot |
| 1722.7938 | 1722.8663 | 0.0725 | 42 | 115 | 129 | IHTGEKPYTCTQCGK | | | Carbamidomethyl (C)[10] | | Mascot |
| 1862.8015 | 1862.9628 | 0.1613 | 87 | 64 | 78 | NCFICTQCGKSLSCK | | | Carbamidomethyl (C)[2,5,8,14] | | Mascot |
| 2193.9912 | 2194.1267 | 0.1355 | 62 | 83 | 101 | IHMMIHTGEKPFTCTQCGK | | | Oxidation (M)[3,4] | | Mascot |
| 2211.0723 | 2211.1243 | 0.052 | 24 | 214 | 232 | SFSLSSNLNHHMRIHTGEK | | | Oxidation (M)[12] | | Mascot |
| 2211.0723 | 2211.1243 | 0.052 | 24 | 214 | 232 | SFSLSSNLNHHMRIHTGEK | | | Oxidation (M)[12] | | Mascot |
| 2247.0776 | 2247.1389 | 0.0613 | 27 | 21 | 38 | QEDLQEQTELIEENKWSK | | | | | Mascot |

| 6 | aldehyde dehydrogenase 2 precursor [Danio rerio] | gi\|20339358 | 56526.6 | 6.05 | 10 | 38 | 0 |
|---|---|---|---|---|---|---|---|

Peptide Information

| Calc. Mass | Obsrv. Mass | ± da | ± ppm | Start Seq. | End Seq. | Sequence | Ion Score | C. I. % | Modification | Rank | Result Type |
|---|---|---|---|---|---|---|---|---|---|---|---|
| 848.51 | 848.5325 | 0.0225 | 27 | 2 | 8 | LRAVFSR | | | | | Mascot |
| 864.4243 | 864.5179 | 0.0936 | 108 | 407 | 413 | DDMKIAR | | | Oxidation (M)[3] | | Mascot |
| 1051.6146 | 1051.7208 | 0.1062 | 101 | 368 | 377 | KVLGYISSGK | | | | | Mascot |
| 1402.6572 | 1402.7797 | 0.1225 | 87 | 144 | 154 | CLRYYAGWADK | | | Carbamidomethyl (C)[1] | | Mascot |
| 1460.6984 | 1460.64 | -0.0584 | -40 | 379 | 393 | EGAKLMCGGAPAAER | | | | | Mascot |
| 1619.7522 | 1619.7642 | 0.012 | 7 | 159 | 171 | TIPIDGNYFCYTR | | | Carbamidomethyl (C)[10] | | Mascot |
| 1761.833 | 1761.8502 | 0.0172 | 10 | 324 | 337 | TFVQESIYDEFVER | | | | | Mascot |
| 2026.0239 | 2025.9406 | -0.0833 | -41 | 436 | 454 | ANDSKYGLAAAVFTQNID K | | | | | Mascot |
| 2233.0771 | 2233.1045 | 0.0274 | 12 | 324 | 341 | TFVQESIYDEFVERSVER | | | | | Mascot |
| 2741.345 | 2741.3174 | -0.0276 | -10 | 52 | 77 | TFPTINPATAEVICHVAEG DKADVDK | | | | | Mascot |

| 7 | LOC561115 protein [Danio rerio] | gi\|124481604 | 94397 | 5.22 | 13 | 36 | 0 |
|---|---|---|---|---|---|---|---|

Peptide Information

| Calc. Mass | Obsrv. Mass | ± da | ± ppm | Start Seq. | End Seq. | Sequence | Ion Score | C. I. % | Modification | Rank | Result Type |
|---|---|---|---|---|---|---|---|---|---|---|---|
| 942.5441 | 942.5549 | 0.0108 | 11 | 365 | 373 | LIQVPAACK | | | | | Mascot |
| 996.5108 | 996.6023 | 0.0915 | 92 | 149 | 157 | AVEENAHVK | | | | | Mascot |
| 996.5108 | 996.6023 | 0.0915 | 92 | 149 | 157 | AVEENAHVK | | | | | Mascot |
| 1106.5299 | 1106.5522 | 0.0223 | 20 | 760 | 767 | WQMEQEKK | | | | | Mascot |
| 1523.7371 | 1523.7728 | 0.0357 | 23 | 497 | 510 | DLGMKDSSISTNQK | | | | | Mascot |
| 1560.9108 | 1560.8516 | -0.0592 | -38 | 130 | 142 | FQLLIIKNTDVTR | | | | | Mascot |
| 1560.9108 | 1560.8516 | -0.0592 | -38 | 130 | 142 | FQLLIIKNTDVTR | | | | | Mascot |
| 1718.7902 | 1718.9227 | 0.1325 | 77 | 407 | 421 | QEPDSPVLSTEVDMR | | | Oxidation (M)[14] | | Mascot |
| 1718.7902 | 1718.9227 | 0.1325 | 77 | 407 | 421 | QEPDSPVLSTEVDMR | | | Oxidation (M)[14] | | Mascot |
| 1722.9061 | 1722.8663 | -0.0398 | -23 | 71 | 84 | RLFSVLSWEDTLEK | | | | | Mascot |
| 1740.8877 | 1740.9028 | 0.0151 | 9 | 96 | 110 | TKDVPMFFEVTEVAK | | | | | Mascot |
| 1857.8734 | 1857.8779 | 0.0045 | 2 | 707 | 722 | ENAVYVICHNPMSPQR | | | | | Mascot |
| 1857.8734 | 1857.8779 | 0.0045 | 2 | 707 | 722 | ENAVYVICHNPMSPQR | | | | | Mascot |
| 2040.9297 | 2040.9497 | 0.02 | 10 | 25 | 41 | SWETSLSAAVFEENEWR | | | | | Mascot |
| 2211.0872 | 2211.1243 | 0.0371 | 17 | 355 | 373 | QYCHYLSSMRLIQVPAAC K | | | | | Mascot |
| 2211.0872 | 2211.1243 | 0.0371 | 17 | 355 | 373 | QYCHYLSSMRLIQVPAAC K | | | | | Mascot |

| | | | | | | | | | | | |
|---|---|---|---|---|---|---|---|---|---|---|---|
| 2233.031 | 2233.1045 | 0.0735 | 33 | 707 | 725 | ENAVYVICHNPMSPQRSCK | | | Carbamidomethyl (C)[8] | | Mascot |
| 2284.1714 | 2284.1958 | 0.0244 | 11 | 52 | 71 | AEDEQLIGSLVQAVQQPMRR | | | Oxidation (M)[18] | | Mascot |
| 2284.1714 | 2284.1958 | 0.0244 | 11 | 52 | 71 | AEDEQLIGSLVQAVQQPMRR | | | Oxidation (M)[18] | | Mascot |

| | | | | | | | |
|---|---|---|---|---|---|---|---|
| 8 | PREDICTED: zinc finger and BTB domain-containing protein 17-like [Danio rerio] | gi\|326679165 | 95241.3 | 5.8 | 13 | 36 | 0 |

Peptide Information

| Calc. Mass | Obsrv. Mass | ± da | ± ppm | Start Seq. | End Seq. | Sequence | Ion Score | C. I. % | Modification | Rank | Result Type |
|---|---|---|---|---|---|---|---|---|---|---|---|
| 864.4825 | 864.5179 | 0.0354 | 41 | 51 | 57 | TLFLDQK | | | | | Mascot |
| 942.4275 | 942.5549 | 0.1274 | 135 | 849 | 856 | VHSESQQQ | | | | | Mascot |
| 1045.5425 | 1045.5698 | 0.0273 | 26 | 531 | 540 | AFADPGALQR | | | | | Mascot |
| 1107.5277 | 1107.5391 | 0.0114 | 10 | 201 | 210 | SESEAERATK | | | | | Mascot |
| 1123.5565 | 1123.5309 | -0.0256 | -23 | 447 | 456 | AFSDPTAKMR | | | | | Mascot |
| 1169.4939 | 1169.6438 | 0.1499 | 128 | 466 | 474 | GHKCQHCDK | | | Carbamidomethyl (C)[4,7] | | Mascot |
| 1460.6863 | 1460.64 | -0.0463 | -32 | 666 | 678 | RLVVAEDDDGEDK | | | | | Mascot |
| 1523.7787 | 1523.7728 | -0.0059 | -4 | 37 | 50 | AHKAVLAACSSYFR | | | | | Mascot |
| 1543.7234 | 1543.8207 | 0.0973 | 63 | 163 | 176 | TEETHEAVSNVAEK | | | | | Mascot |
| 1862.8635 | 1862.9628 | 0.0993 | 53 | 348 | 362 | IHTGEKPFTCRDCNK | | | Carbamidomethyl (C)[10,13] | | Mascot |
| 2194.0579 | 2194.1267 | 0.0688 | 31 | 513 | 530 | HLRVHSGEKPFICMHCQR | | | Oxidation (M)[14] | | Mascot |
| 2247.0103 | 2247.1389 | 0.1286 | 57 | 372 | 390 | AHEKTHSPVKPYCCSTCGK | | | Carbamidomethyl (C)[13,14,17] | | Mascot |
| 2298.0012 | 2298.2048 | 0.2036 | 89 | 748 | 767 | VQEADPNTQILYACDSCGEK | | | Carbamidomethyl (C)[14,17] | | Mascot |

| | | | | | | | |
|---|---|---|---|---|---|---|---|
| 9 | mitogen-activated protein kinase 1 [Danio rerio] | gi\|40254655 | 42048.5 | 6.32 | 8 | 35 | 0 |

Peptide Information

| Calc. Mass | Obsrv. Mass | ± da | ± ppm | Start Seq. | End Seq. | Sequence | Ion Score | C. I. % | Modification | Rank | Result Type |
|---|---|---|---|---|---|---|---|---|---|---|---|
| 864.4362 | 864.5179 | 0.0817 | 95 | 363 | 369 | FQPGYRP | | | | | Mascot |
| 1107.5681 | 1107.5391 | -0.029 | -26 | 354 | 362 | ELIFEETAR | | | | | Mascot |
| 1303.7732 | 1303.772 | -0.0012 | -1 | 271 | 281 | NYLLSLPLRSK | | | | | Mascot |
| 1523.7675 | 1523.7728 | 0.0053 | 3 | 201 | 212 | WYRAPEIMLNSK | | | Oxidation (M)[8] | | Mascot |
| 1878.928 | 1878.9661 | 0.0381 | 20 | 65 | 79 | ISPFEHQTYCQRTLR | | | | | Mascot |
| 2040.9767 | 2040.9497 | -0.027 | -13 | 2 | 24 | ATAAVSAPAGGGPNPGSGAEMVR | | | Oxidation (M)[21] | | Mascot |
| 2246.9844 | 2247.1389 | 0.1545 | 69 | 34 | 54 | YSNLSYIGGGAYGMVCSAYDR | | | | | Mascot |
| 2719.3945 | 2719.3137 | -0.0808 | -30 | 217 | 240 | SIDIWSVGCILAEMLSNRPIFPGK | | | Carbamidomethyl (C)[9], Oxidation (M)[14] | | Mascot |
| 2719.3945 | 2719.3137 | -0.0808 | -30 | 217 | 240 | SIDIWSVGCILAEMLSNR | | | Carbamidomethyl (C)[9], Oxidation (M)[14] | | Mascot |

PIFPGK

| 10 | PREDICTED: layilin [Danio rerio] | gi\|326668116 | 39321.9 | 5.6 | 8 | 34 | 0 |
|---|---|---|---|---|---|---|---|

Peptide Information

| Calc. Mass | Obsrv. Mass | ± da | ± ppm | Start Seq. | End Seq. | Sequence | Ion Score | C. I. % | Modification | Rank | Result Type |
|---|---|---|---|---|---|---|---|---|---|---|---|
| 982.4662 | 982.5555 | 0.0893 | 91 | 10 | 17 | GTEKPCYK | | | Carbamidomethyl (C)[6] | | Mascot |
| 1156.5602 | 1156.6102 | 0.05 | 43 | 1 | 9 | MFSQQICKR | | | Oxidation (M)[1] | | Mascot |
| 1509.6467 | 1509.7767 | 0.13 | 86 | 92 | 103 | YYWMDGSPATFR | | | Oxidation (M)[4] | | Mascot |
| 1619.7958 | 1619.7642 | -0.0316 | -20 | 252 | 265 | CNSPSPDVYNVIRR | | | | | Mascot |
| 1706.8442 | 1706.8643 | 0.0201 | 12 | 39 | 54 | SDGGDLLSIESKTEQK | | | | | Mascot |
| 1722.8882 | 1722.8663 | -0.0219 | -13 | 265 | 279 | RQHESDLAGTRPDIK | | | | | Mascot |
| 2719.1187 | 2719.3137 | 0.195 | 72 | 81 | 103 | GYEENGNCSSKYYWMD GSPATFR | | | Carbamidomethyl (C)[8] | | Mascot |
| 2719.1187 | 2719.3137 | 0.195 | 72 | 81 | 103 | GYEENGNCSSKYYWMD GSPATFR | | | Carbamidomethyl (C)[8] | | Mascot |
| 3897.2576 | 3896.8367 | -0.4209 | -108 | 190 | 226 | TVVSGLPDNALNIAYIVLP TIPLLLLLVVATGVFCFK | | | | | Mascot |

| 11 | novel protein [Danio rerio] | gi\|148725296 | 79374.4 | 6.72 | 11 | 33 | 0 |
|---|---|---|---|---|---|---|---|

Peptide Information

| Calc. Mass | Obsrv. Mass | ± da | ± ppm | Start Seq. | End Seq. | Sequence | Ion Score | C. I. % | Modification | Rank | Result Type |
|---|---|---|---|---|---|---|---|---|---|---|---|
| 848.477 | 848.5325 | 0.0555 | 65 | 506 | 512 | MRLIGSR | | | Oxidation (M)[1] | | Mascot |
| 864.4937 | 864.5179 | 0.0242 | 28 | 433 | 439 | EVFRVSK | | | | | Mascot |
| 982.4992 | 982.5555 | 0.0563 | 57 | 534 | 541 | GYYIPNQK | | | | | Mascot |
| 1156.6685 | 1156.6102 | -0.0583 | -50 | 349 | 359 | EILTAARPGTK | | | | | Mascot |
| 1165.6034 | 1165.756 | 0.1526 | 131 | 225 | 234 | FCEQAVTIVR | | | | | Mascot |
| 1165.6034 | 1165.756 | 0.1526 | 131 | 225 | 234 | FCEQAVTIVR | | | | | Mascot |
| 1460.7566 | 1460.64 | -0.1166 | -80 | 331 | 343 | EMQAAIVLQAGWK | | | Oxidation (M)[2] | | Mascot |
| 1722.8778 | 1722.8663 | -0.0115 | -7 | 221 | 234 | ACLRFCEQAVTIVR | | | Carbamidomethyl (C)[2,6] | | Mascot |
| 1862.8523 | 1862.9628 | 0.1105 | 59 | 389 | 404 | HMVTNGEGIAELYPCR | | | Carbamidomethyl (C)[15], Oxidation (M)[2] | | Mascot |
| 1878.9708 | 1878.9661 | -0.0047 | -3 | 140 | 156 | ESSTRPGSLTAKPFSWK | | | | | Mascot |
| 2283.1875 | 2283.2009 | 0.0134 | 6 | 447 | 466 | MFSPIPVCILHVINNDTLE K | | | | | Mascot |
| 2528.2812 | 2528.1924 | -0.0888 | -35 | 367 | 388 | TLQEMWASVESDVEKHA VSLLR | | | | | Mascot |

| 12 | Sb:cb283 protein [Danio rerio] | gi\|45709356 | 55978.7 | 8.16 | 10 | 33 | 0 |
|---|---|---|---|---|---|---|---|

Peptide Information

| Calc. Mass | Obsrv. Mass | ± da | ± ppm | Start Seq. | End Seq. | Sequence | Ion Score | C. I. % | Modification | Rank | Result Type |
|---|---|---|---|---|---|---|---|---|---|---|---|

| Calc. Mass | Obsrv. Mass | ± da | ± ppm | Start Seq. | End Seq. | Sequence | Ion Score | C. I. % | Modification | Rank | Result Type |
|---|---|---|---|---|---|---|---|---|---|---|---|
| 1062.5691 | 1062.6975 | 0.1284 | 121 | 92 | 101 | FRDAAANGIK | | | | | Mascot |
| 1123.5167 | 1123.5309 | 0.0142 | 13 | 429 | 436 | REDYEFHK | | | | | Mascot |
| 1169.6201 | 1169.6438 | 0.0237 | 20 | 198 | 207 | VAEYVQSVFK | | | | | Mascot |
| 1279.6437 | 1279.7867 | 0.143 | 112 | 235 | 245 | CANAVPRHQAR | | | Carbamidomethyl (C)[1] | | Mascot |
| 1582.724 | 1582.8284 | 0.1044 | 66 | 45 | 58 | APLQDYSAVDCCIK | | | Carbamidomethyl (C)[11] | | Mascot |
| 1619.9227 | 1619.7642 | -0.1585 | -98 | 59 | 73 | DEVVVLRVPGLPGNR | | | | | Mascot |
| 1761.9493 | 1761.8502 | -0.0991 | -56 | 414 | 429 | AGEVLGDLFEVSTIRR | | | | | Mascot |
| 1903.9265 | 1903.9333 | 0.0068 | 4 | 225 | 241 | EYPCLAAVNRCANAVPR | | | Carbamidomethyl (C)[4] | | Mascot |
| 2025.9116 | 2025.9406 | 0.029 | 14 | 74 | 90 | LVCSCTGPVNRDYDDVR | | | Carbamidomethyl (C)[3,5] | | Mascot |
| 2211.0276 | 2211.1243 | 0.0967 | 44 | 345 | 364 | VGNTDAEGRMVMVDLLCEMK | | | | | Mascot |
| 2211.0276 | 2211.1243 | 0.0967 | 44 | 345 | 364 | VGNTDAEGRMVMVDLLCEMK | | | | | Mascot |
| 2284.0439 | 2284.1958 | 0.1519 | 67 | 345 | 364 | VGNTDAEGRMVMVDLLCEMK | | | Carbamidomethyl (C)[17], Oxidation (M)[10] | | Mascot |
| 2284.0439 | 2284.1958 | 0.1519 | 67 | 345 | 364 | VGNTDAEGRMVMVDLLCEMK | | | Carbamidomethyl (C)[17], Oxidation (M)[10] | | Mascot |
| 2300.0388 | 2300.1975 | 0.1587 | 69 | 345 | 364 | VGNTDAEGRMVMVDLLCEMK | | | Carbamidomethyl (C)[17], Oxidation (M)[10,12] | | Mascot |

13 shadow of prion protein precursor [Danio rerio] gi|99028940 13848.9 10.26 5 33 0

**Peptide Information**

| Calc. Mass | Obsrv. Mass | ± da | ± ppm | Start Seq. | End Seq. | Sequence | Ion Score | C. I. % | Modification | Rank | Result Type |
|---|---|---|---|---|---|---|---|---|---|---|---|
| 1165.6 | 1165.756 | 0.156 | 134 | 95 | 103 | TNWELYLAR | | | | | Mascot |
| 1165.6 | 1165.756 | 0.156 | 134 | 95 | 103 | TNWELYLAR | | | | | Mascot |
| 1862.9543 | 1862.9628 | 0.0085 | 5 | 117 | 132 | LSALLLPINYMMHFAP | | | Oxidation (M)[11,12] | | Mascot |
| 2506.2212 | 2506.2161 | -0.0051 | -2 | 4 | 25 | AVATCCIFLLLSAFLCDQVMSK | | | Carbamidomethyl (C)[5,6], Oxidation (M)[20] | | Mascot |
| 2719.3438 | 2719.3137 | -0.0301 | -11 | 4 | 28 | AVATCCIFLLLSAFLCDQVMSKGGR | | | Carbamidomethyl (C)[5], Oxidation (M)[20] | | Mascot |
| 2719.3438 | 2719.3137 | -0.0301 | -11 | 2 | 25 | NRAVATCCIFLLLSAFLCDQVMSK | | | Carbamidomethyl (C)[7], Oxidation (M)[22] | | Mascot |

14 PREDICTED: glycerophosphodiester phosphodiesterase domain-containing protein 1-like [Danio rerio] gi|189525714 37212.2 7.67 9 33 0

**Peptide Information**

| Calc. Mass | Obsrv. Mass | ± da | ± ppm | Start Seq. | End Seq. | Sequence | Ion Score | C. I. % | Modification | Rank | Result Type |
|---|---|---|---|---|---|---|---|---|---|---|---|
| 1230.7568 | 1230.6799 | -0.0769 | -62 | 133 | 142 | RFVLLENVLK | | | | | Mascot |
| 1279.7521 | 1279.7867 | 0.0346 | 27 | 249 | 258 | NRFVVYLLQK | | | | | Mascot |
| 1460.7216 | 1460.64 | -0.0816 | -56 | 37 | 47 | LEFYCRHISHR | | | | | Mascot |
| 1706.7448 | 1706.8643 | 0.1195 | 70 | 193 | 205 | QNPSMPYMFTMRR | | | Oxidation (M)[5,8,11] | | Mascot |

| Calc. Mass | Obsrv. Mass | ± da | ± ppm | Start Seq. | End Seq. | Sequence | Ion Score | C. I. % | Modification | Rank | Result Type |
|---|---|---|---|---|---|---|---|---|---|---|---|
| 1740.8989 | 1740.9028 | 0.0039 | 2 | 235 | 248 | IINRTYIPEEAYMK | | | | | Mascot |
| 1862.842 | 1862.9628 | 0.1208 | 65 | 190 | 204 | CLKQNPSMPYMFTMR | | | Oxidation (M)[8] | | Mascot |
| 1878.8369 | 1878.9661 | 0.1292 | 69 | 190 | 204 | CLKQNPSMPYMFTMR | | | Oxidation (M)[8,11] | | Mascot |
| 1903.8685 | 1903.9333 | 0.0648 | 34 | 190 | 204 | CLKQNPSMPYMFTMR | | | Carbamidomethyl (C)[1] | | Mascot |
| 2297.1052 | 2297.2095 | 0.1043 | 45 | 174 | 192 | EHITVWASEEHEVMAKC LK | | | Carbamidomethyl (C)[17] | | Mascot |
| 2506.3447 | 2506.2161 | -0.1286 | -51 | 134 | 154 | FVLLENVLKNFPNMPMTL EIK | | | Oxidation (M)[14] | | Mascot |
| 3897.1646 | 3896.8367 | -0.3279 | -84 | 1 | 33 | MDTVSWVLLLCLAVLFYV VCSLFLLRYPLILHK | | | Oxidation (M)[1] | | Mascot |

15 Shadoo protein [Danio rerio] gi|37359664 13918 10.51 5 33 0

**Peptide Information**

| Calc. Mass | Obsrv. Mass | ± da | ± ppm | Start Seq. | End Seq. | Sequence | Ion Score | C. I. % | Modification | Rank | Result Type |
|---|---|---|---|---|---|---|---|---|---|---|---|
| 1165.6 | 1165.756 | 0.156 | 134 | 95 | 103 | TNWELYLAR | | | | | Mascot |
| 1165.6 | 1165.756 | 0.156 | 134 | 95 | 103 | TNWELYLAR | | | | | Mascot |
| 1862.9543 | 1862.9628 | 0.0085 | 5 | 117 | 132 | LSALLLPINYMMHFAP | | | Oxidation (M)[11,12] | | Mascot |
| 2506.2212 | 2506.2161 | -0.0051 | -2 | 4 | 25 | AVATCCIFLLLSAFLCDQV MSK | | | Carbamidomethyl (C)[5,6], Oxidation (M)[20] | | Mascot |
| 2719.3438 | 2719.3137 | -0.0301 | -11 | 4 | 28 | AVATCCIFLLLSAFLCDQV MSKGGR | | | Carbamidomethyl (C)[5], Oxidation (M)[20] | | Mascot |
| 2719.3438 | 2719.3137 | -0.0301 | -11 | 2 | 25 | NRAVATCCIFLLLSAFLCD QVMSK | | | Carbamidomethyl (C)[7], Oxidation (M)[22] | | Mascot |

16 PREDICTED: transformation/transcription domain-associated protein [Danio rerio] gi|326672350 433471.2 8.52 28 32 0

**Peptide Information**

| Calc. Mass | Obsrv. Mass | ± da | ± ppm | Start Seq. | End Seq. | Sequence | Ion Score | C. I. % | Modification | Rank | Result Type |
|---|---|---|---|---|---|---|---|---|---|---|---|
| 848.4083 | 848.5325 | 0.1242 | 146 | 3257 | 3263 | ATAPMWR | | | Oxidation (M)[5] | | Mascot |
| 882.5155 | 882.5728 | 0.0573 | 65 | 2182 | 2189 | AVHSLLSR | | | | | Mascot |
| 1045.5095 | 1045.5698 | 0.0603 | 58 | 3604 | 3612 | EVQGNMVPR | | | Oxidation (M)[6] | | Mascot |
| 1106.5952 | 1106.5522 | -0.043 | -39 | 2034 | 2045 | TAAGAVGTVFGR | | | | | Mascot |
| 1165.5817 | 1165.756 | 0.1743 | 150 | 606 | 615 | VANCQTVRMK | | | Oxidation (M)[9] | | Mascot |
| 1165.5817 | 1165.756 | 0.1743 | 150 | 606 | 615 | VANCQTVRMK | | | Oxidation (M)[9] | | Mascot |
| 1169.6677 | 1169.6438 | -0.0239 | -20 | 3327 | 3336 | ITPHTLNFVK | | | | | Mascot |
| 1185.5061 | 1185.6406 | 0.1345 | 113 | 2167 | 2178 | GIAACMTCGNTK | | | Oxidation (M)[6] | | Mascot |
| 1303.6641 | 1303.772 | 0.1079 | 83 | 3368 | 3379 | AQATAQDPVFQK | | | | | Mascot |
| 1402.7623 | 1402.7797 | 0.0174 | 12 | 108 | 119 | SHAKNILSVMFR | | | | | Mascot |
| 1448.8293 | 1448.9343 | 0.105 | 72 | 1147 | 1159 | LGGVVSIKFLMER | | | | | Mascot |
| 1509.7076 | 1509.7767 | 0.0691 | 46 | 1311 | 1322 | LFTMDLNVMEHK | | | Oxidation (M)[4,9] | | Mascot |

| | | | | | | | | | | | |
|---|---|---|---|---|---|---|---|---|---|---|---|
| 1543.8124 | 1543.8207 | 0.0083 | 5 | 1666 | 1677 | LLAFCLLSYCKR | | | Carbamidomethyl (C)[5,10] | | Mascot |
| 1560.8455 | 1560.8516 | 0.0061 | 4 | 793 | 805 | DLFVELCLTVPVR | | | Carbamidomethyl (C)[7] | | Mascot |
| 1560.8455 | 1560.8516 | 0.0061 | 4 | 793 | 805 | DLFVELCLTVPVR | | | Carbamidomethyl (C)[7] | | Mascot |
| 1619.7893 | 1619.7642 | -0.0251 | -15 | 1950 | 1962 | HHLVQHMISAMQR | | | Oxidation (M)[7,11] | | Mascot |
| 1707.0164 | 1706.8643 | -0.1521 | -89 | 330 | 344 | ELLIAAKHILTTDLR | | | | | Mascot |
| 1718.8054 | 1718.9227 | 0.1173 | 68 | 3311 | 3326 | CYSVAFEKSGAVSDAK | | | Carbamidomethyl (C)[1] | | Mascot |
| 1718.8054 | 1718.9227 | 0.1173 | 68 | 3311 | 3326 | CYSVAFEKSGAVSDAK | | | Carbamidomethyl (C)[1] | | Mascot |
| 1722.903 | 1722.8663 | -0.0367 | -21 | 3039 | 3053 | QQVKCYLQLAGVMGK | | | Carbamidomethyl (C)[5] | | Mascot |
| 1903.8928 | 1903.9333 | 0.0405 | 21 | 3489 | 3504 | IYPYLVMNDACLTESR | | | Oxidation (M)[7] | | Mascot |
| 1924.0031 | 1923.9751 | -0.028 | -15 | 541 | 557 | TLVCGVKTITWGITSCK | | | Carbamidomethyl (C)[4,16] | | Mascot |
| 1924.0031 | 1923.9751 | -0.028 | -15 | 541 | 557 | TLVCGVKTITWGITSCK | | | Carbamidomethyl (C)[4,16] | | Mascot |
| 2009.8903 | 2010.0215 | 0.1312 | 65 | 1382 | 1400 | ALNSTNSELQEAGEACM GK | | | Carbamidomethyl (C)[16] | | Mascot |
| 2025.8851 | 2025.9406 | 0.0555 | 27 | 1382 | 1400 | ALNSTNSELQEAGEACM GK | | | Carbamidomethyl (C)[16], Oxidation (M)[17] | | Mascot |
| 2211.0977 | 2211.1243 | 0.0266 | 12 | 619 | 637 | EVLEHFAGVFTMMNPLTF K | | | | | Mascot |
| 2211.0977 | 2211.1243 | 0.0266 | 12 | 619 | 637 | EVLEHFAGVFTMMNPLTF K | | | | | Mascot |
| 2230.0769 | 2230.2168 | 0.1399 | 63 | 1323 | 1341 | VFYTELLNLCEAEDAALM K | | | Carbamidomethyl (C)[10] | | Mascot |
| 2283.0981 | 2283.2009 | 0.1028 | 45 | 3617 | 3634 | EWALHTFPNATDYWTFR K | | | | | Mascot |
| 2284.0908 | 2284.1958 | 0.105 | 46 | 1075 | 1094 | GMDPLVLIDAIAICMAYEE K | | | Carbamidomethyl (C)[14], Oxidation (M)[2,15] | | Mascot |
| 2284.1384 | 2284.1958 | 0.0574 | 25 | 1175 | 1196 | ALLFVMMDLTGEVSNGA VAMAK | | | Oxidation (M)[6] | | Mascot |
| 2297.0537 | 2297.2095 | 0.1558 | 68 | 1696 | 1713 | FLCNMTFLKEYMEEEIPK | | | Oxidation (M)[5,12] | | Mascot |
| 2300.1335 | 2300.1975 | 0.064 | 28 | 1175 | 1196 | ALLFVMMDLTGEVSNGA VAMAK | | | Oxidation (M)[6,7] | | Mascot |
| 2741.3269 | 2741.3174 | -0.0095 | -3 | 1075 | 1098 | GMDPLVLIDAIAICMAYEE KELCK | | | Carbamidomethyl (C)[14], Oxidation (M)[2] | | Mascot |
| 3355.6641 | 3355.4919 | -0.1722 | -51 | 3099 | 3127 | AFSAAVQMHDVLVKAWA MWGDYLENIFVK | | | Oxidation (M)[8] | | Mascot |

| | | | | | | | |
|---|---|---|---|---|---|---|---|
| 17 | MOV10-like 1 [Danio rerio] | gi\|116268043 | 122527.1 | 5.89 | 14 | 32 | 0 |

Peptide Information

| Calc. Mass | Obsrv. Mass | ± da | ± ppm | Start Seq. | End Seq. | Sequence | Ion Score | C. I. % | Modification | Rank | Result Type |
|---|---|---|---|---|---|---|---|---|---|---|---|
| 1045.5021 | 1045.5698 | 0.0677 | 65 | 19 | 26 | RAQEEEQR | | | | | Mascot |
| 1106.6204 | 1106.5522 | -0.0682 | -62 | 493 | 502 | EFSLTGAILR | | | | | Mascot |
| 1230.5419 | 1230.6799 | 0.138 | 112 | 327 | 338 | CEDGGTEAPPVR | | | | | Mascot |
| 1460.847 | 1460.64 | -0.207 | -142 | 855 | 868 | LASVFGLGVSLLER | | | | | Mascot |
| 1560.6824 | 1560.8516 | 0.1692 | 108 | 352 | 365 | MCVCVCCTAKTLGR | | | Carbamidomethyl (C)[2], Oxidation (M)[1] | | Mascot |
| 1560.6824 | 1560.8516 | 0.1692 | 108 | 352 | 365 | MCVCVCCTAKTLGR | | | Carbamidomethyl (C)[2], Oxidation (M)[1] | | Mascot |

| Calc. Mass | Obsrv. Mass | ± da | ± ppm | Start Seq. | End Seq. | Sequence | Ion Score | C. I. % | Modification | Rank | Result Type |
|---|---|---|---|---|---|---|---|---|---|---|---|
| 1582.8158 | 1582.8284 | 0.0126 | 8 | 907 | 919 | LFYAGELCVRAQR | | | Carbamidomethyl (C)[8] | | Mascot |
| 1718.8795 | 1718.9227 | 0.0432 | 25 | 935 | 949 | GFPLIFHGVRGSEMR | | | Oxidation (M)[14] | | Mascot |
| 1718.8795 | 1718.9227 | 0.0432 | 25 | 935 | 949 | GFPLIFHGVRGSEMR | | | Oxidation (M)[14] | | Mascot |
| 1862.8378 | 1862.9628 | 0.125 | 67 | 1078 | 1092 | EPCFSALLQYSYDNR | | | Carbamidomethyl (C)[3] | | Mascot |
| 1923.8535 | 1923.9751 | 0.1216 | 63 | 755 | 770 | VNATCRPEESMSEELR | | | Carbamidomethyl (C)[5], Oxidation (M)[11] | | Mascot |
| 1923.8535 | 1923.9751 | 0.1216 | 63 | 755 | 770 | VNATCRPEESMSEELR | | | Carbamidomethyl (C)[5], Oxidation (M)[11] | | Mascot |
| 2233.1807 | 2233.1045 | -0.0762 | -34 | 1 | 18 | MVSVVQCLMSRLLWPLWR | | | Oxidation (M)[1] | | Mascot |
| 2247.239 | 2247.1389 | -0.1001 | -45 | 705 | 723 | TITLIEAILQVHHRISCSR | | | Carbamidomethyl (C)[17] | | Mascot |
| 2283.1624 | 2283.2009 | 0.0385 | 17 | 869 | 889 | LMGNSLYACGERGFNPLLVTK | | | | | Mascot |
| 2719.2202 | 2719.3137 | 0.0935 | 34 | 339 | 361 | MLEEPLDIHPGQKMCVCVCCTAK | | | Carbamidomethyl (C)[15,17,19] | | Mascot |
| 2719.2202 | 2719.3137 | 0.0935 | 34 | 339 | 361 | MLEEPLDIHPGQKMCVCVCCTAK | | | Carbamidomethyl (C)[15,17,19] | | Mascot |
| 3338.7551 | 3338.7876 | 0.0325 | 10 | 528 | 557 | VVLKKPCSGGVVIEYIAYVTEISDEDVSLR | | | Carbamidomethyl (C)[7] | | Mascot |

| | | | | | | | |
|---|---|---|---|---|---|---|---|
| 18 | muskelin [Danio rerio] | gi\|47086651 | 84322.2 | 5.91 | 12 | 32 | 0 |

Peptide Information

| Calc. Mass | Obsrv. Mass | ± da | ± ppm | Start Seq. | End Seq. | Sequence | Ion Score | C. I. % | Modification | Rank | Result Type |
|---|---|---|---|---|---|---|---|---|---|---|---|
| 850.4781 | 850.5497 | 0.0716 | 84 | 333 | 339 | QIYTLGR | | | | | Mascot |
| 942.5189 | 942.5549 | 0.036 | 38 | 73 | 80 | THVCNLKK | | | | | Mascot |
| 1106.5048 | 1106.5522 | 0.0474 | 43 | 301 | 309 | ENQWACISR | | | | | Mascot |
| 1230.587 | 1230.6799 | 0.0929 | 75 | 451 | 460 | IGHCMLFHTR | | | Oxidation (M)[5] | | Mascot |
| 1460.6774 | 1460.64 | -0.0374 | -26 | 113 | 123 | HKIDEQMFPCR | | | Carbamidomethyl (C)[10] | | Mascot |
| 1582.6699 | 1582.8284 | 0.1585 | 100 | 380 | 392 | LVFDHQMCMDSEK | | | | | Mascot |
| 1722.8446 | 1722.8663 | 0.0217 | 13 | 180 | 193 | QHNYTEAFESLQKK | | | | | Mascot |
| 1740.8221 | 1740.9028 | 0.0807 | 46 | 564 | 578 | ENPTKALQEEEPCPR | | | | | Mascot |
| 2194.2278 | 2194.1267 | -0.1011 | -46 | 196 | 213 | IALEHPMLTHLHERLVLR | | | Oxidation (M)[7] | | Mascot |
| 2225.0947 | 2225.1379 | 0.0432 | 19 | 702 | 720 | TQLFDTLVNFFPDNMTPPK | | | | | Mascot |
| 2225.0947 | 2225.1379 | 0.0432 | 19 | 702 | 720 | TQLFDTLVNFFPDNMTPPK | | | | | Mascot |
| 2233.0408 | 2233.1045 | 0.0637 | 29 | 682 | 701 | SSSDFIPLGFSDVDQTYAQR | | | | | Mascot |
| 2298.1626 | 2298.2048 | 0.0422 | 18 | 226 | 244 | AVRDGLFNQYISQQDYKPR | | | | | Mascot |

| | | | | | | | |
|---|---|---|---|---|---|---|---|
| 19 | Si:rp71-61h23.4 protein [Danio rerio] | gi\|33991821 | 45666.6 | 5.53 | 8 | 32 | 0 |

Peptide Information

| Calc. Mass | Obsrv. Mass | ± da | ± ppm | Start Seq. | End Seq. | Sequence | Ion Score | C. I. % | Modification | Rank | Result Type |
|---|---|---|---|---|---|---|---|---|---|---|---|

| Calc. Mass | Obsrv. Mass | ± da | ± ppm | Start Seq. | End Seq. | Sequence | Ion Score | C. I. % | Modification | Rank | Result Type |
|---|---|---|---|---|---|---|---|---|---|---|---|
| 1106.551 | 1106.5522 | 0.0012 | 1 | 9 | 18 | MADNVKTAEK | | | | | Mascot |
| 1123.4935 | 1123.5309 | 0.0374 | 33 | 314 | 322 | DKEQEMESK | | | | | Mascot |
| 1156.6797 | 1156.6102 | -0.0695 | -60 | 234 | 243 | ALEQSLLRAR | | | | | Mascot |
| 1185.611 | 1185.6406 | 0.0296 | 25 | 293 | 302 | EGQPKELQEK | | | | | Mascot |
| 1303.6562 | 1303.772 | 0.1158 | 89 | 138 | 150 | DAVVGAVMEGVEK | | | | | Mascot |
| 1560.8049 | 1560.8516 | 0.0467 | 30 | 138 | 152 | DAVVGAVMEGVEKTR | | | | | Mascot |
| 1560.8049 | 1560.8516 | 0.0467 | 30 | 138 | 152 | DAVVGAVMEGVEKTR | | | | | Mascot |
| 2271.1614 | 2271.1245 | -0.0369 | -16 | 196 | 216 | ELAALAEPRPGEEEGFIV STR | | | | | Mascot |
| 3355.7573 | 3355.4919 | -0.2654 | -79 | 393 | 422 | QSLDGVVEYLLNNTPLN WLVGPFAPQITEK | | | | | Mascot |

| | | | | | | | |
|---|---|---|---|---|---|---|---|
| 20 | PREDICTED: hypothetical protein LOC100329705 [Danio rerio] | gi\|326676237 | 8666.2 | 4.61 | 4 | 31 | 0 |

**Peptide Information**

| Calc. Mass | Obsrv. Mass | ± da | ± ppm | Start Seq. | End Seq. | Sequence | Ion Score | C. I. % | Modification | Rank | Result Type |
|---|---|---|---|---|---|---|---|---|---|---|---|
| 1279.647 | 1279.7867 | 0.1397 | 109 | 20 | 29 | YFVYFAKEGR | | | | | Mascot |
| 1402.696 | 1402.7797 | 0.0837 | 60 | 47 | 58 | NVSFVKSYSSER | | | | | Mascot |
| 2009.8374 | 2010.0215 | 0.1841 | 92 | 59 | 74 | CELWEENGFYSYAWVN | | | | | Mascot |
| 2719.1406 | 2719.3137 | 0.1731 | 64 | 53 | 74 | SYSSERCELWEENGFYS YAWVN | | | | | Mascot |
| 2719.1406 | 2719.3137 | 0.1731 | 64 | 53 | 74 | SYSSERCELWEENGFYS YAWVN | | | | | Mascot |

| | | | | | | | |
|---|---|---|---|---|---|---|---|
| 21 | PREDICTED: solute carrier family 12 member 6-like, partial [Danio rerio] | gi\|326668968 | 26763.8 | 8.83 | 6 | 31 | 0 |

**Peptide Information**

| Calc. Mass | Obsrv. Mass | ± da | ± ppm | Start Seq. | End Seq. | Sequence | Ion Score | C. I. % | Modification | Rank | Result Type |
|---|---|---|---|---|---|---|---|---|---|---|---|
| 850.4781 | 850.5497 | 0.0716 | 84 | 133 | 139 | TFINTVR | | | | | Mascot |
| 942.4825 | 942.5549 | 0.0724 | 77 | 74 | 80 | HLMEKER | | | | | Mascot |
| 1402.7623 | 1402.7797 | 0.0174 | 12 | 83 | 94 | GFVQCIVAQKPR | | | Carbamidomethyl (C)[5] | | Mascot |
| 1718.8458 | 1718.9227 | 0.0769 | 45 | 29 | 42 | YIEYHGYVTMTTLK | | | | | Mascot |
| 1718.8458 | 1718.9227 | 0.0769 | 45 | 29 | 42 | YIEYHGYVTMTTLK | | | | | Mascot |
| 2211.3223 | 2211.1243 | -0.198 | -90 | 133 | 152 | TFINTVRVTTTAHLALLVL K | | | | | Mascot |
| 2211.3223 | 2211.1243 | -0.198 | -90 | 133 | 152 | TFINTVRVTTTAHLALLVL K | | | | | Mascot |
| 2230.0664 | 2230.2168 | 0.1504 | 67 | 197 | 215 | CALRIFTVAQMEDNSIQM K | | | Oxidation (M)[11,18] | | Mascot |
| 2271.093 | 2271.1245 | 0.0315 | 14 | 197 | 215 | CALRIFTVAQMEDNSIQM K | | | Carbamidomethyl (C)[1], Oxidation (M)[11] | | Mascot |

| | | | | | | | |
|---|---|---|---|---|---|---|---|
| 22 | RecName: Full=Contactin-1a; AltName: | gi\|55976512 | 114263.1 | 6.25 | 12 | 30 | 0 |

Full=F3/F11/contactin; AltName: Full=Neural cell recognition m

Peptide Information

| Calc. Mass | Obsrv. Mass | ± da | ± ppm | Start Seq. | End Seq. | Sequence | Ion Score | C. I. % | Modification | Rank | Result Type |
|---|---|---|---|---|---|---|---|---|---|---|---|
| 1045.5173 | 1045.5698 | 0.0525 | 50 | 562 | 569 | DAQHYQRK | | | | | Mascot |
| 1062.5513 | 1062.6975 | 0.1462 | 138 | 442 | 449 | VVFECRPR | | | Carbamidomethyl (C)[5] | | Mascot |
| 1106.5323 | 1106.5522 | 0.0199 | 18 | 625 | 634 | VDEVTSDSVR | | | | | Mascot |
| 1107.6885 | 1107.5391 | -0.1494 | -135 | 285 | 294 | KLDGVLPPLR | | | | | Mascot |
| 1303.6827 | 1303.772 | 0.0893 | 68 | 2 | 12 | IPEAFQPRAMK | | | Oxidation (M)[10] | | Mascot |
| 2224.9749 | 2225.1379 | 0.163 | 73 | 388 | 406 | FSRLGFEDSGMYQCVAE NR | | | Oxidation (M)[11] | | Mascot |
| 2225.1772 | 2225.1379 | -0.0393 | -18 | 695 | 716 | VIATNTLGTGPPSEPSPK TTTR | | | | | Mascot |
| 2230.0305 | 2230.2168 | 0.1863 | 84 | 114 | 134 | SKHAGNYTCVASNQYGS VTSR | | | | | Mascot |
| 2283.092 | 2283.2009 | 0.1089 | 48 | 594 | 614 | YSCTAQTPVDNTTASAEL VVR | | | Carbamidomethyl (C)[3] | | Mascot |
| 2284.2131 | 2284.1958 | -0.0173 | -8 | 442 | 460 | VVFECRPRAAPRPNITW SK | | | Carbamidomethyl (C)[5] | | Mascot |
| 2284.2131 | 2284.1958 | -0.0173 | -8 | 442 | 460 | VVFECRPRAAPRPNITW SK | | | Carbamidomethyl (C)[5] | | Mascot |
| 3338.7102 | 3338.7876 | 0.0774 | 23 | 256 | 284 | SPDSWALLGQNVTLECF ALGNPIPQIRWR | | | Carbamidomethyl (C)[16] | | Mascot |
| 3897.1306 | 3896.8367 | -0.2939 | -75 | 994 | 1032 | AHSEGGDGAVAQVRITG SAPAPALASALLLLPLLW TLML | | | Oxidation (M)[38] | | Mascot |

23 PREDICTED: leiomodin-3 [Danio rerio] gi|326679036 76410.3 4.83 11 30 0

Peptide Information

| Calc. Mass | Obsrv. Mass | ± da | ± ppm | Start Seq. | End Seq. | Sequence | Ion Score | C. I. % | Modification | Rank | Result Type |
|---|---|---|---|---|---|---|---|---|---|---|---|
| 942.5302 | 942.5549 | 0.0247 | 26 | 101 | 108 | VPVGMRQR | | | | | Mascot |
| 982.5316 | 982.5555 | 0.0239 | 24 | 639 | 646 | DHLLSEIR | | | | | Mascot |
| 1045.4731 | 1045.5698 | 0.0967 | 92 | 52 | 60 | DASHEMTVR | | | | | Mascot |
| 1062.5977 | 1062.6975 | 0.0998 | 94 | 453 | 461 | MVVTNLLTR | | | Oxidation (M)[1] | | Mascot |
| 1303.6252 | 1303.772 | 0.1468 | 113 | 442 | 452 | MGYHFELPGPR | | | | | Mascot |
| 1523.7522 | 1523.7728 | 0.0206 | 14 | 402 | 413 | CLQFNETLTELR | | | Carbamidomethyl (C)[1] | | Mascot |
| 1560.8527 | 1560.8516 | -0.0011 | -1 | 453 | 465 | MVVTNLLTRNLDR | | | Oxidation (M)[1] | | Mascot |
| 1560.8527 | 1560.8516 | -0.0011 | -1 | 453 | 465 | MVVTNLLTRNLDR | | | Oxidation (M)[1] | | Mascot |
| 1574.6904 | 1574.7871 | 0.0967 | 61 | 573 | 584 | DPLLEWNQCDER | | | Carbamidomethyl (C)[9] | | Mascot |
| 1619.6854 | 1619.7642 | 0.0788 | 49 | 1 | 13 | MSERTEQESYTDK | | | Oxidation (M)[1] | | Mascot |
| 1903.9629 | 1903.9333 | -0.0296 | -16 | 419 | 434 | HMLGHHAEMEVSRLLK | | | Oxidation (M)[2] | | Mascot |

| | | | | | | | | | | | | |
|---|---|---|---|---|---|---|---|---|---|---|---|---|
| 2284.1238 | 2284.1958 | 0.072 | 32 | 30 | 49 | QLQSEMDDIAPDERVPV GLR | | | Oxidation (M)[6] | | Mascot |
| 2284.1238 | 2284.1958 | 0.072 | 32 | 30 | 49 | QLQSEMDDIAPDERVPV GLR | | | Oxidation (M)[6] | | Mascot |

| | | | | | | | | |
|---|---|---|---|---|---|---|---|---|
| 24 | novel protein similar to vertebrate inositol polyphosphate-5-phosphatase, 72 kDa (INPP5E, zgc:16561 | gi\|220673191 | 66013.6 | 8.76 | 10 | 30 | 0 | |

**Peptide Information**

| Calc. Mass | Obsrv. Mass | ± da | ± ppm | Start Seq. | End Seq. | Sequence | Ion Score | C. I. % | Modification | Rank | Result Type |
|---|---|---|---|---|---|---|---|---|---|---|---|
| 1106.6317 | 1106.5522 | -0.0795 | -72 | 550 | 558 | SLYLEGIRR | | | | | Mascot |
| 1107.5792 | 1107.5391 | -0.0401 | -36 | 31 | 39 | NLSFDEKVR | | | | | Mascot |
| 1123.5491 | 1123.5309 | -0.0182 | -16 | 487 | 495 | QRTPSYTDR | | | | | Mascot |
| 1402.7802 | 1402.7797 | -0.0005 | 0 | 193 | 204 | STYSVLNPIRPR | | | | | Mascot |
| 1740.7891 | 1740.9028 | 0.1137 | 65 | 568 | 583 | EAVMKNQNNSTVCSIS | | | Oxidation (M)[4] | | Mascot |
| 1761.8113 | 1761.8502 | 0.0389 | 22 | 474 | 488 | FDIGCDVYDTTTKQR | | | | | Mascot |
| 2194.2026 | 2194.1267 | -0.0759 | -35 | 519 | 537 | TSDHRPVIGMFQVKLRP GR | | | | | Mascot |
| 2283.1443 | 2283.2009 | 0.0566 | 25 | 454 | 473 | EGSIFKGFQEASIHFPPT YK | | | | | Mascot |
| 2284.0874 | 2284.1958 | 0.1084 | 47 | 92 | 112 | IRTGSLSETDVSPHDLCD PNK | | | | | Mascot |
| 2284.0874 | 2284.1958 | 0.1084 | 47 | 92 | 112 | IRTGSLSETDVSPHDLCD PNK | | | | | Mascot |
| 2300.0459 | 2300.1975 | 0.1516 | 66 | 94 | 114 | TGSLSETDVSPHDLCDP NKER | | | | | Mascot |

| | | | | | | | |
|---|---|---|---|---|---|---|---|
| 25 | Zgc:165612 protein [Danio rerio] | gi\|152012517 | 46099.5 | 8.16 | 8 | 30 | 0 |

**Peptide Information**

| Calc. Mass | Obsrv. Mass | ± da | ± ppm | Start Seq. | End Seq. | Sequence | Ion Score | C. I. % | Modification | Rank | Result Type |
|---|---|---|---|---|---|---|---|---|---|---|---|
| 1106.6317 | 1106.5522 | -0.0795 | -72 | 370 | 378 | SLYLEGIRR | | | | | Mascot |
| 1123.5491 | 1123.5309 | -0.0182 | -16 | 307 | 315 | QRTPSYTDR | | | | | Mascot |
| 1402.7802 | 1402.7797 | -0.0005 | 0 | 13 | 24 | STYSVLNPIRPR | | | | | Mascot |
| 1740.7891 | 1740.9028 | 0.1137 | 65 | 388 | 403 | EAVMKNQNNSTVCSIS | | | Oxidation (M)[4] | | Mascot |
| 1761.8113 | 1761.8502 | 0.0389 | 22 | 294 | 308 | FDIGCDVYDTTTKQR | | | | | Mascot |
| 2194.2026 | 2194.1267 | -0.0759 | -35 | 339 | 357 | TSDHRPVIGMFQVKLRP GR | | | | | Mascot |
| 2283.1443 | 2283.2009 | 0.0566 | 25 | 274 | 293 | EGSIFKGFQEASIHFPPT YK | | | | | Mascot |
| 2719.3655 | 2719.3137 | -0.0518 | -19 | 1 | 24 | MVSDNTSVHSMKSTYSV LNPIRPR | | | | | Mascot |
| 2719.3655 | 2719.3137 | -0.0518 | -19 | 1 | 24 | MVSDNTSVHSMKSTYSV LNPIRPR | | | | | Mascot |

| | | | | | | | |
|---|---|---|---|---|---|---|---|
| 26 | PREDICTED: hypothetical protein LOC100332625 [Danio rerio] | gi\|292619215 | 9203.5 | 8.69 | 4 | 30 | 0 |

Peptide Information

| Calc. Mass | Obsrv. Mass | ± da | ± ppm | Start Seq. | End Seq. | Sequence | Ion Score | C. I. % | Modification | Rank | Result Type |
|---|---|---|---|---|---|---|---|---|---|---|---|
| 1862.7811 | 1862.9628 | 0.1817 | 98 | 2 | 15 | TGWNCWFEYCMLPR | | | Carbamidomethyl (C)[5] | | Mascot |
| 1878.776 | 1878.9661 | 0.1901 | 101 | 2 | 15 | TGWNCWFEYCMLPR | | | Carbamidomethyl (C)[5], Oxidation (M)[11] | | Mascot |
| 2009.8165 | 2010.0215 | 0.205 | 102 | 1 | 15 | MTGWNCWFEYCMLPR | | | Carbamidomethyl (C)[6], Oxidation (M)[1] | | Mascot |
| 2025.8114 | 2025.9406 | 0.1292 | 64 | 1 | 15 | MTGWNCWFEYCMLPR | | | Carbamidomethyl (C)[6], Oxidation (M)[1,12] | | Mascot |
| 2193.9741 | 2194.1267 | 0.1526 | 70 | 1 | 17 | MTGWNCWFEYCMLPRLK | | | Oxidation (M)[1] | | Mascot |
| 2233.1208 | 2233.1045 | -0.0163 | -7 | 18 | 37 | FKPSMSDLSLTFPYAVNTSK | | | | | Mascot |

27 PREDICTED: zinc finger protein 850-like [Danio rerio] gi|326667171 77347.8 9.3 13 30 0

Peptide Information

| Calc. Mass | Obsrv. Mass | ± da | ± ppm | Start Seq. | End Seq. | Sequence | Ion Score | C. I. % | Modification | Rank | Result Type |
|---|---|---|---|---|---|---|---|---|---|---|---|
| 1230.5063 | 1230.6799 | 0.1736 | 141 | 64 | 73 | NCFICTQCGK | | | Carbamidomethyl (C)[2,5] | | Mascot |
| 1509.7114 | 1509.7767 | 0.0653 | 43 | 550 | 562 | SFSQSSSLNLHMR | | | Oxidation (M)[12] | | Mascot |
| 1560.6971 | 1560.8516 | 0.1545 | 99 | 354 | 366 | SFSQSSNLNHHMR | | | Oxidation (M)[12] | | Mascot |
| 1560.6971 | 1560.8516 | 0.1545 | 99 | 354 | 366 | SFSQSSNLNHHMR | | | Oxidation (M)[12] | | Mascot |
| 1574.7493 | 1574.7871 | 0.0378 | 24 | 242 | 254 | SFSQSSHLNKHMR | | | Oxidation (M)[12] | | Mascot |
| 1619.7383 | 1619.7642 | 0.0259 | 16 | 522 | 534 | SFSQSPYLNHHMR | | | Oxidation (M)[12] | | Mascot |
| 1706.7988 | 1706.8643 | 0.0655 | 38 | 563 | 577 | IHTGEKPFTCTQCGK | | | Carbamidomethyl (C)[10] | | Mascot |
| 1718.7988 | 1718.9227 | 0.1239 | 72 | 255 | 269 | IHTGEKPYTCPQCGK | | | Carbamidomethyl (C)[10] | | Mascot |
| 1718.7988 | 1718.9227 | 0.1239 | 72 | 255 | 269 | IHTGEKPYTCPQCGK | | | Carbamidomethyl (C)[10] | | Mascot |
| 1722.8302 | 1722.8663 | 0.0361 | 21 | 451 | 465 | IHTGEKLFTCTQCGK | | | Carbamidomethyl (C)[10] | | Mascot |
| 1862.8015 | 1862.9628 | 0.1613 | 87 | 64 | 78 | NCFICTQCGKSLSCK | | | Carbamidomethyl (C)[2,5,8,14] | | Mascot |
| 1923.8584 | 1923.9751 | 0.1167 | 61 | 616 | 630 | HMMIHTGEKEFMCLK | | | Carbamidomethyl (C)[13], Oxidation (M)[2,3] | | Mascot |
| 1923.8584 | 1923.9751 | 0.1167 | 61 | 616 | 630 | HMMIHTGEKEFMCLK | | | Carbamidomethyl (C)[13], Oxidation (M)[2,3] | | Mascot |
| 2193.9912 | 2194.1267 | 0.1355 | 62 | 83 | 101 | IHMMIHTGEKPFTCTQCGK | | | Oxidation (M)[3,4] | | Mascot |
| 2247.0776 | 2247.1389 | 0.0613 | 27 | 21 | 38 | QEDLQEQTELIEENKWSK | | | | | Mascot |
| 3355.5154 | 3355.4919 | -0.0235 | -7 | 283 | 310 | IHTGEKPFTSNQCGKNFYCSSNLNQHMR | | | Carbamidomethyl (C)[13,19] | | Mascot |

28 novel protein [Danio rerio] gi|94734127 8833.8 12.02 4 30 0

Peptide Information

| Calc. Mass | Obsrv. Mass | ± da | ± ppm | Start Seq. | End Seq. | Sequence | Ion Score | C. I. % | Modification | Rank | Result Type |
|---|---|---|---|---|---|---|---|---|---|---|---|

| Calc. Mass | Obsrv. Mass | ± da | ± ppm | Start Seq. | End Seq. | Sequence | Ion Score | C. I. % | Modification | Rank | Result Type |
|---|---|---|---|---|---|---|---|---|---|---|---|
| 942.5254 | 942.5549 | 0.0295 | 31 | 9 | 18 | SPAAPVSVSK | | | | | Mascot |
| 1045.5272 | 1045.5698 | 0.0426 | 41 | 52 | 62 | SPGASQNGSLK | | | | | Mascot |
| 1740.8987 | 1740.9028 | 0.0041 | 2 | 52 | 69 | SPGASQNGSLKSPGQGLR | | | | | Mascot |
| 1923.9956 | 1923.9751 | -0.0205 | -11 | 20 | 39 | APSTPPISKPTLCSSPAGGR | | | | | Mascot |
| 1923.9956 | 1923.9751 | -0.0205 | -11 | 20 | 39 | APSTPPISKPTLCSSPAGGR | | | | | Mascot |

| | | | | | | | |
|---|---|---|---|---|---|---|---|
| 29 | PREDICTED: protein-glutamine gamma-glutamyltransferase 2-like [Danio rerio] | gi\|326668941 | 74951.8 | 5.69 | 10 | 30 | 0 |

**Peptide Information**

| Calc. Mass | Obsrv. Mass | ± da | ± ppm | Start Seq. | End Seq. | Sequence | Ion Score | C. I. % | Modification | Rank | Result Type |
|---|---|---|---|---|---|---|---|---|---|---|---|
| 1045.5425 | 1045.5698 | 0.0273 | 26 | 437 | 445 | AVYTEAVHR | | | | | Mascot |
| 1156.6758 | 1156.6102 | -0.0656 | -57 | 177 | 186 | LLDLNPKCLK | | | | | Mascot |
| 1230.6188 | 1230.6799 | 0.0611 | 50 | 647 | 657 | LMATFNSATFK | | | | | Mascot |
| 1574.8132 | 1574.7871 | -0.0261 | -17 | 616 | 630 | STEKSSTVQLGPGQR | | | | | Mascot |
| 1582.8013 | 1582.8284 | 0.0271 | 17 | 524 | 537 | ALSFTLPYSAYGHR | | | | | Mascot |
| 1862.875 | 1862.9628 | 0.0878 | 47 | 255 | 270 | YGQCWVFAAVMCTVLR | | | Oxidation (M)[11] | | Mascot |
| 1878.9095 | 1878.9661 | 0.0566 | 30 | 483 | 499 | GSRAQCVCVNISAQVMR | | | Carbamidomethyl (C)[6] | | Mascot |
| 1903.9015 | 1903.9333 | 0.0318 | 17 | 255 | 270 | YGQCWVFAAVMCTVLR | | | Carbamidomethyl (C)[4] | | Mascot |
| 2211.0017 | 2211.1243 | 0.1226 | 55 | 2 | 22 | CSSSSSNTPDQGVRVDLECVK | | | | | Mascot |
| 2211.0017 | 2211.1243 | 0.1226 | 55 | 2 | 22 | CSSSSSNTPDQGVRVDLECVK | | | | | Mascot |
| 2528.2424 | 2528.1924 | -0.05 | -20 | 524 | 545 | ALSFTLPYSAYGHRMLENNCIK | | | | | Mascot |
| 2719.2798 | 2719.3137 | 0.0339 | 12 | 255 | 277 | YGQCWVFAAVMCTVLRCLGIPCR | | | Carbamidomethyl (C)[4,12], Oxidation (M)[11] | | Mascot |
| 2719.2798 | 2719.3137 | 0.0339 | 12 | 255 | 277 | YGQCWVFAAVMCTVLRCLGIPCR | | | Carbamidomethyl (C)[4,12], Oxidation (M)[11] | | Mascot |

| | | | | | | | |
|---|---|---|---|---|---|---|---|
| 30 | Wu:fd12d03 protein [Danio rerio] | gi\|67678171 | 54699.2 | 5.26 | 8 | 30 | 0 |

**Peptide Information**

| Calc. Mass | Obsrv. Mass | ± da | ± ppm | Start Seq. | End Seq. | Sequence | Ion Score | C. I. % | Modification | Rank | Result Type |
|---|---|---|---|---|---|---|---|---|---|---|---|
| 1156.6547 | 1156.6102 | -0.0445 | -38 | 33 | 41 | VMIYRFVTK | | | | | Mascot |
| 1165.6211 | 1165.756 | 0.1349 | 116 | 38 | 47 | FVTKASVEER | | | | | Mascot |
| 1165.6211 | 1165.756 | 0.1349 | 116 | 38 | 47 | FVTKASVEER | | | | | Mascot |
| 1169.6273 | 1169.6438 | 0.0165 | 14 | 395 | 406 | RAAAIAAGEDPK | | | | | Mascot |
| 1230.6298 | 1230.6799 | 0.0501 | 41 | 245 | 255 | SEAANSRRPSR | | | | | Mascot |
| 1862.8137 | 1862.9628 | 0.1491 | 80 | 150 | 164 | DEDEEEEDVDREIIK | | | | | Mascot |
| 1924.0062 | 1923.9751 | -0.0311 | -16 | 77 | 92 | QELDDILKFGTEQLFK | | | | | Mascot |

| | | | | | | | | | | | | |
|---|---|---|---|---|---|---|---|---|---|---|---|---|
| 1924.0062 | 1923.9751 | -0.0311 | -16 | 77 | 92 | QELDDILKFGTEQLFK | | | | | Mascot |
| 2225.0569 | 2225.1379 | 0.081 | 36 | 407 | 428 | TPSTGTPADTQPNTPAP EDLSK | | | | | Mascot |
| 2225.0569 | 2225.1379 | 0.081 | 36 | 407 | 428 | TPSTGTPADTQPNTPAP EDLSK | | | | | Mascot |
| 3896.5146 | 3896.8367 | 0.3221 | 83 | 218 | 251 | DWQDDQSDNQSDYSVA SEEGDEDFDERSEAANS R | | | | | Mascot |

| | | | | | | | | |
|---|---|---|---|---|---|---|---|---|
| 31 | methyltransferase-like protein 13 [Danio rerio] | gi\|217416336 | 75976.5 | 5.99 | 10 | 30 | 0 | |

Peptide Information

| Calc. Mass | Obsrv. Mass | ± da | ± ppm | Start Seq. | End Seq. | Sequence | Ion Score | C. I. % | Modification | Rank | Result Type |
|---|---|---|---|---|---|---|---|---|---|---|---|
| 864.4032 | 864.5179 | 0.1147 | 133 | 504 | 510 | DFVPCAR | | | Carbamidomethyl (C)[5] | | Mascot |
| 942.5406 | 942.5549 | 0.0143 | 15 | 166 | 173 | LAVEHFVK | | | | | Mascot |
| 1062.6306 | 1062.6975 | 0.0669 | 63 | 589 | 597 | KVYSLLSPR | | | | | Mascot |
| 1165.6221 | 1165.756 | 0.1339 | 115 | 598 | 607 | GLFMLNLVCR | | | | | Mascot |
| 1165.6221 | 1165.756 | 0.1339 | 115 | 598 | 607 | GLFMLNLVCR | | | | | Mascot |
| 1402.726 | 1402.7797 | 0.0537 | 38 | 240 | 250 | ERQAYNLMLHK | | | | | Mascot |
| 1543.8689 | 1543.8207 | -0.0482 | -31 | 228 | 241 | LASVEELLSAVKER | | | | | Mascot |
| 2040.9086 | 2040.9497 | 0.0411 | 20 | 7 | 22 | TAEEFSSADYWERFFR | | | | | Mascot |
| 2283.1826 | 2283.2009 | 0.0183 | 8 | 536 | 556 | LKVTLGDGLDHITTLESE GER | | | | | Mascot |
| 2300.1226 | 2300.1975 | 0.0749 | 33 | 52 | 72 | VLVVGCGNSELSEQLYD VGYR | | | | | Mascot |
| 3337.5254 | 3337.8066 | 0.2812 | 84 | 453 | 482 | SVDRGFLCCTHHEVMVA GLAMLGMDAINNK | | | Carbamidomethyl (C)[8], Oxidation (M)[15,21,24] | | Mascot |

| | | | | | | | | |
|---|---|---|---|---|---|---|---|---|
| 32 | PREDICTED: nucleoporin NUP188 homolog [Danio rerio] | gi\|189533646 | 194083 | 6.04 | 17 | 30 | 0 | |

Peptide Information

| Calc. Mass | Obsrv. Mass | ± da | ± ppm | Start Seq. | End Seq. | Sequence | Ion Score | C. I. % | Modification | Rank | Result Type |
|---|---|---|---|---|---|---|---|---|---|---|---|
| 1156.5931 | 1156.6102 | 0.0171 | 15 | 1493 | 1501 | MLHHYLQAK | | | Oxidation (M)[1] | | Mascot |
| 1230.6875 | 1230.6799 | -0.0076 | -6 | 1535 | 1545 | ALQAVQCSLLK | | | Carbamidomethyl (C)[7] | | Mascot |
| 1460.5927 | 1460.64 | 0.0473 | 32 | 1 | 12 | MADSEMCVRSSR | | | Carbamidomethyl (C)[7], Oxidation (M)[1,6] | | Mascot |
| 1509.7961 | 1509.7767 | -0.0194 | -13 | 860 | 871 | YIYHKHDPALPR | | | | | Mascot |
| 1523.7522 | 1523.7728 | 0.0206 | 14 | 1098 | 1110 | SLSESQMLISAWR | | | Oxidation (M)[7] | | Mascot |
| 1560.9467 | 1560.8516 | -0.0951 | -61 | 1157 | 1170 | LGSMMVTLLLILLK | | | Oxidation (M)[4] | | Mascot |
| 1560.9467 | 1560.8516 | -0.0951 | -61 | 1157 | 1170 | LGSMMVTLLLILLK | | | Oxidation (M)[4] | | Mascot |
| 1582.9414 | 1582.8284 | -0.113 | -71 | 442 | 456 | TGPLLQLLTALLSDK | | | | | Mascot |
| 1619.9401 | 1619.7642 | -0.1759 | -109 | 1395 | 1408 | LSLSLMESLLKTLR | | | Oxidation (M)[6] | | Mascot |

| | | | | | | | | | | | |
|---|---|---|---|---|---|---|---|---|---|---|---|
| 1707.0415 | 1706.8643 | -0.1772 | -104 | 1308 | 1323 | LPVLPSVLSALELSLR | | | | | Mascot |
| 1740.8875 | 1740.9028 | 0.0153 | 9 | 22 | 36 | SALREPAQIEDELNR | | | | | Mascot |
| 1862.9105 | 1862.9628 | 0.0523 | 28 | 123 | 136 | IADYYYEERLCVLR | | | Carbamidomethyl (C)[11] | | Mascot |
| 1903.9794 | 1903.9333 | -0.0461 | -24 | 1529 | 1545 | EEAESKALQAVQCSLLK | | | Carbamidomethyl (C)[13] | | Mascot |
| 2010.1633 | 2010.0215 | -0.1418 | -71 | 680 | 697 | GEYSVTIAFLRLVTTLVK | | | | | Mascot |
| 2230.071 | 2230.2168 | 0.1458 | 65 | 469 | 486 | MSFYTEAYKHRPNDVISR | | | Oxidation (M)[1] | | Mascot |
| 2247.1128 | 2247.1389 | 0.0261 | 12 | 137 | 153 | CVLHLLTYFQDERHPYR | | | Carbamidomethyl (C)[1] | | Mascot |
| 2271.1221 | 2271.1245 | 0.0024 | 1 | 1275 | 1293 | DQQDSVCVLALHLSKEL CR | | | Carbamidomethyl (C)[7,18] | | Mascot |
| 3896.0486 | 3895.8374 | -0.2112 | -54 | 1344 | 1382 | TPQGAAAVAGAGVIQSIC LPLLSVYEGPANGANQA FVRK | | | Carbamidomethyl (C)[18] | | Mascot |

33 novel protein similar to H.sapiens LAYN, layilin (LAYN) [Danio rerio] gi|148725485 38956.8 5.61 7 30 0

Peptide Information

| Calc. Mass | Obsrv. Mass | ± da | ± ppm | Start Seq. | End Seq. | Sequence | Ion Score | C. I. % | Modification | Rank | Result Type |
|---|---|---|---|---|---|---|---|---|---|---|---|
| 982.4662 | 982.5555 | 0.0893 | 91 | 7 | 14 | GTEKPCYK | | | Carbamidomethyl (C)[6] | | Mascot |
| 1509.6467 | 1509.7767 | 0.13 | 86 | 89 | 100 | YYWMDGSPATFR | | | Oxidation (M)[4] | | Mascot |
| 1619.7958 | 1619.7642 | -0.0316 | -20 | 249 | 262 | CNSPSPDVYNVIRR | | | | | Mascot |
| 1706.8442 | 1706.8643 | 0.0201 | 12 | 36 | 51 | SDGGDLLSIESKTEQK | | | | | Mascot |
| 1722.8882 | 1722.8663 | -0.0219 | -13 | 262 | 276 | RQHESDLAGTRPDIK | | | | | Mascot |
| 2719.1187 | 2719.3137 | 0.195 | 72 | 78 | 100 | GYEENGNCSSKYYWMD GSPATFR | | | Carbamidomethyl (C)[8] | | Mascot |
| 2719.1187 | 2719.3137 | 0.195 | 72 | 78 | 100 | GYEENGNCSSKYYWMD GSPATFR | | | Carbamidomethyl (C)[8] | | Mascot |
| 3897.2576 | 3896.8367 | -0.4209 | -108 | 187 | 223 | TVVSGLPDNALNIAYIVLP TIPLLLLLVVATGVFCFK | | | | | Mascot |

34 PREDICTED: heat-stable enterotoxin receptor-like [Danio rerio] gi|326665604 115779.3 7.15 12 29 0

Peptide Information

| Calc. Mass | Obsrv. Mass | ± da | ± ppm | Start Seq. | End Seq. | Sequence | Ion Score | C. I. % | Modification | Rank | Result Type |
|---|---|---|---|---|---|---|---|---|---|---|---|
| 850.4781 | 850.5497 | 0.0716 | 84 | 921 | 927 | RGETFLK | | | | | Mascot |
| 1051.5935 | 1051.7208 | 0.1273 | 121 | 629 | 636 | LYLVQYPR | | | | | Mascot |
| 1165.6212 | 1165.756 | 0.1348 | 116 | 504 | 513 | GSLRYVLDDK | | | | | Mascot |
| 1165.6212 | 1165.756 | 0.1348 | 116 | 504 | 513 | GSLRYVLDDK | | | | | Mascot |
| 1460.6863 | 1460.64 | -0.0463 | -32 | 49 | 61 | DVVETAVEDQNNK | | | | | Mascot |
| 1523.8904 | 1523.7728 | -0.1176 | -77 | 899 | 911 | IHVSESTIKILQR | | | | | Mascot |
| 1706.7731 | 1706.8643 | 0.0912 | 53 | 514 | 527 | ISYPDASFMDLEFR | | | Oxidation (M)[9] | | Mascot |

| | | | | | | | | | | | |
|---|---|---|---|---|---|---|---|---|---|---|---|
| 1857.9388 | 1857.8779 | -0.0609 | -33 | 537 | 553 | GMSYLHSSNVGVHGRLK | | | Oxidation (M)[2] | | Mascot |
| 1857.9388 | 1857.8779 | -0.0609 | -33 | 537 | 553 | GMSYLHSSNVGVHGRLK | | | Oxidation (M)[2] | | Mascot |
| 1878.9596 | 1878.9661 | 0.0065 | 3 | 316 | 331 | DLNLSVVYTTGSYKYR | | | | | Mascot |
| 1903.8789 | 1903.9333 | 0.0544 | 29 | 874 | 889 | MPRYCLFGDTVNTASR | | | Carbamidomethyl (C)[5], Oxidation (M)[1] | | Mascot |
| 1923.9904 | 1923.9751 | -0.0153 | -8 | 162 | 176 | KIAEFFFHFWHTSVK | | | | | Mascot |
| 1923.9904 | 1923.9751 | -0.0153 | -8 | 162 | 176 | KIAEFFFHFWHTSVK | | | | | Mascot |
| 2719.4976 | 2719.3137 | -0.1839 | -68 | 848 | 873 | HLPGIPLWIRIGIHSGPCA AGVVGNK | | | Carbamidomethyl (C)[18] | | Mascot |
| 2719.4976 | 2719.3137 | -0.1839 | -68 | 848 | 873 | HLPGIPLWIRIGIHSGPCA AGVVGNK | | | Carbamidomethyl (C)[18] | | Mascot |
| 3896.0632 | 3895.8374 | -0.2258 | -58 | 629 | 662 | LYLVQYPRGPNVFRPDL SFEGVGETEAELFVLIK | | | | | Mascot |

| | | | | | | | |
|---|---|---|---|---|---|---|---|
| 35 | PREDICTED: probable serine/threonine-protein kinase kinX-like [Danio rerio] | gi\|326667489 | 12182.4 | 4.79 | 5 | 29 | 0 |

**Peptide Information**

| Calc. Mass | Obsrv. Mass | ± da | ± ppm | Start Seq. | End Seq. | Sequence | Ion Score | C. I. % | Modification | Rank | Result Type |
|---|---|---|---|---|---|---|---|---|---|---|---|
| 1106.496 | 1106.5522 | 0.0562 | 51 | 85 | 95 | AAQSDAVDSDK | | | | | Mascot |
| 1303.729 | 1303.772 | 0.043 | 33 | 67 | 78 | VDAVVKMDSVLK | | | | | Mascot |
| 1402.7365 | 1402.7797 | 0.0432 | 31 | 96 | 108 | FAGVSTLAFTFNK | | | | | Mascot |
| 1862.7825 | 1862.9628 | 0.1803 | 97 | 1 | 18 | MDEGAMMGVGAMMGAV LK | | | Oxidation (M)[1,6,7,12] | | Mascot |
| 1878.7773 | 1878.9661 | 0.1888 | 100 | 1 | 18 | MDEGAMMGVGAMMGAV LK | | | Oxidation (M)[1,6,7,12,13] | | Mascot |
| 2506.1189 | 2506.2161 | 0.0972 | 39 | 1 | 24 | MDEGAMMGVGAMMGAV LKMDAVVK | | | Oxidation (M)[1,6,7,12] | | Mascot |

| | | | | | | | |
|---|---|---|---|---|---|---|---|
| 36 | PREDICTED: zinc finger and SCAN domain-containing protein 2-like [Danio rerio] | gi\|326680717 | 88496.4 | 9.79 | 13 | 29 | 0 |

**Peptide Information**

| Calc. Mass | Obsrv. Mass | ± da | ± ppm | Start Seq. | End Seq. | Sequence | Ion Score | C. I. % | Modification | Rank | Result Type |
|---|---|---|---|---|---|---|---|---|---|---|---|
| 842.5094 | 842.5154 | 0.006 | 7 | 335 | 341 | ESRLIPK | | | | | Mascot |
| 996.5724 | 996.6023 | 0.0299 | 30 | 575 | 583 | SFIKSSSLK | | | | | Mascot |
| 996.6564 | 996.6023 | -0.0541 | -54 | 338 | 346 | LIPKASLVR | | | | | Mascot |
| 1169.6499 | 1169.6438 | -0.0061 | -5 | 233 | 242 | IKAFLGYMAR | | | | | Mascot |
| 1185.6449 | 1185.6406 | -0.0043 | -4 | 233 | 242 | IKAFLGYMAR | | | Oxidation (M)[8] | | Mascot |
| 1460.6951 | 1460.64 | -0.0551 | -38 | 15 | 26 | VQQEYVMPSAHR | | | Oxidation (M)[7] | | Mascot |
| 1706.7988 | 1706.8643 | 0.0655 | 38 | 672 | 686 | IHTGEKPFTCTQCGK | | | Carbamidomethyl (C)[10] | | Mascot |
| 1718.8352 | 1718.9227 | 0.0875 | 51 | 504 | 518 | IHTGEKPFTCIQCGK | | | Carbamidomethyl (C)[10] | | Mascot |
| 1718.8352 | 1718.9227 | 0.0875 | 51 | 504 | 518 | IHTGEKPFTCIQCGK | | | Carbamidomethyl (C)[10] | | Mascot |
| 1740.949 | 1740.9028 | -0.0462 | -27 | 354 | 369 | KIPALLDSLESNPSTR | | | | | Mascot |

| | | | | | | | | | | | |
|---|---|---|---|---|---|---|---|---|---|---|---|
| 1761.8411 | 1761.8502 | 0.0091 | 5 | 756 | 770 | IHTGEKPFVCTQCGK | | | Carbamidomethyl (C)[10,13] | | Mascot |
| 1862.9442 | 1862.9628 | 0.0186 | 10 | 155 | 172 | ASGGFGTHCLPVPGPRP R | | | Carbamidomethyl (C)[9] | | Mascot |
| 2211.1516 | 2211.1243 | -0.0273 | -12 | 355 | 373 | IPALLDSLESNPSTRQQW R | | | | | Mascot |
| 2211.1516 | 2211.1243 | -0.0273 | -12 | 355 | 373 | IPALLDSLESNPSTRQQW R | | | | | Mascot |
| 2230.0679 | 2230.2168 | 0.1489 | 67 | 584 | 602 | LHMRIHTGDKPFTCTQC GK | | | Carbamidomethyl (C)[14] | | Mascot |
| 3337.541 | 3337.8066 | 0.2656 | 80 | 687 | 714 | SFSCSSHLNHHIRIHTGE KPFTCPQCEK | | | Carbamidomethyl (C)[4,23] | | Mascot |

| | | | | | | | | | | |
|---|---|---|---|---|---|---|---|---|---|---|
| 37 | PREDICTED: ATP-binding cassette sub-family B member 7, mitochondrial [Danio rerio] | gi\|125834513 | 82250.8 | 9.26 | 10 | 29 | 0 | 6 | 0 | |

**Peptide Information**

| Calc. Mass | Obsrv. Mass | ± da | ± ppm | Start Seq. | End Seq. | Sequence | Ion Score | C. I. % | Modification | Rank | Result Type |
|---|---|---|---|---|---|---|---|---|---|---|---|
| 842.5458 | 842.5154 | -0.0304 | -36 | 19 | 25 | LSLLLQR | | | | | Mascot |
| 850.576 | 850.5497 | -0.0263 | -31 | 2 | 9 | APLLVPLK | | | | | Mascot |
| 1107.5792 | 1107.5391 | -0.0401 | -36 | 192 | 201 | TGSALFNELR | | | | | Mascot |
| 1165.5848 | 1165.756 | 0.1712 | 147 | 47 | 56 | NTYLLSDPSR | | | | | Mascot |
| 1165.5848 | 1165.756 | 0.1712 | 147 | 47 | 56 | NTYLLSDPSR | | | | | Mascot |
| 1460.6951 | 1460.64 | -0.0551 | -38 | 585 | 596 | MPHKYDTQVGER | | | | | Mascot |
| 1543.8777 | 1543.8207 | -0.057 | -37 | 575 | 588 | LAGIHDAILKMPHK | | | | | Mascot |
| 2194.1477 | 2194.1267 | -0.021 | -10 | 111 | 128 | ILGAMFTYVWPKDRPDL R | | | Oxidation (M)[5] | | Mascot |
| 2211.0425 | 2211.1243 | 0.0818 | 37 | 47 | 65 | NTYLLSDPSRESSTWAN NR | | | | | Mascot |
| 2211.0425 | 2211.1243 | 0.0818 | 37 | 47 | 65 | NTYLLSDPSRESSTWAN NR | 6 | 0 | | | Mascot |
| 2283.2554 | 2283.2009 | -0.0545 | -24 | 658 | 678 | LSTIVDADEIIVLNQGKVA ER | | | | | Mascot |
| 3338.7817 | 3338.7876 | 0.0059 | 2 | 250 | 279 | GISFVLSALVFNLGPTLFE MMLVSGILYYK | | | Oxidation (M)[20] | | Mascot |

| | | | | | | | | | | |
|---|---|---|---|---|---|---|---|---|---|---|
| 38 | 72 kDa inositol polyphosphate 5-phosphatase [Danio rerio] | gi\|320091327 | 71875.5 | 8.03 | 10 | 29 | 0 | | | |

**Peptide Information**

| Calc. Mass | Obsrv. Mass | ± da | ± ppm | Start Seq. | End Seq. | Sequence | Ion Score | C. I. % | Modification | Rank | Result Type |
|---|---|---|---|---|---|---|---|---|---|---|---|
| 1106.6317 | 1106.5522 | -0.0795 | -72 | 602 | 610 | SLYLEGIRR | | | | | Mascot |
| 1107.5792 | 1107.5391 | -0.0401 | -36 | 83 | 91 | NLSFDEKVR | | | | | Mascot |
| 1123.5491 | 1123.5309 | -0.0182 | -16 | 539 | 547 | QRTPSYTDR | | | | | Mascot |
| 1402.7802 | 1402.7797 | -0.0005 | 0 | 245 | 256 | STYSVLNPIRPR | | | | | Mascot |
| 1740.7891 | 1740.9028 | 0.1137 | 65 | 620 | 635 | EAVMKNQNNSTVCSIS | | | Oxidation (M)[4] | | Mascot |

| Calc. Mass | Obsrv. Mass | ± da | ± ppm | Start Seq. | End Seq. | Sequence | Ion Score | C. I. % | Modification | Rank | Result Type |
|---|---|---|---|---|---|---|---|---|---|---|---|
| 1761.8113 | 1761.8502 | 0.0389 | 22 | 526 | 540 | FDIGCDVYDTTTKQR | | | | | Mascot |
| 2194.2026 | 2194.1267 | -0.0759 | -35 | 571 | 589 | TSDHRPVIGMFQVKLRP GR | | | | | Mascot |
| 2283.1443 | 2283.2009 | 0.0566 | 25 | 506 | 525 | EGSIFKGFQEASIHFPPT YK | | | | | Mascot |
| 2284.0874 | 2284.1958 | 0.1084 | 47 | 144 | 164 | IRTGSLSETDVSPHDLCD PNK | | | | | Mascot |
| 2284.0874 | 2284.1958 | 0.1084 | 47 | 144 | 164 | IRTGSLSETDVSPHDLCD PNK | | | | | Mascot |
| 2300.0459 | 2300.1975 | 0.1516 | 66 | 146 | 166 | TGSLSETDVSPHDLCDP NKER | | | | | Mascot |

39 PREDICTED: gastrula zinc finger protein XlCGF57.1-like [Danio rerio] gi|292613293 40724.7 9.24 10 29 0

**Peptide Information**

| Calc. Mass | Obsrv. Mass | ± da | ± ppm | Start Seq. | End Seq. | Sequence | Ion Score | C. I. % | Modification | Rank | Result Type |
|---|---|---|---|---|---|---|---|---|---|---|---|
| 942.4938 | 942.5549 | 0.0611 | 65 | 194 | 201 | GNLKSHMR | | | | | Mascot |
| 982.4523 | 982.5555 | 0.1032 | 105 | 342 | 349 | SHMNVHNK | | | Oxidation (M)[3] | | Mascot |
| 1543.7144 | 1543.8207 | 0.1063 | 69 | 124 | 137 | TFNCPVCGNAYARK | | | | | Mascot |
| 1718.7737 | 1718.9227 | 0.149 | 87 | 234 | 248 | IHSGEKPFACQECGR | | | Carbamidomethyl (C)[10] | | Mascot |
| 1718.7737 | 1718.9227 | 0.149 | 87 | 234 | 248 | IHSGEKPFACQECGR | | | Carbamidomethyl (C)[10] | | Mascot |
| 2040.9452 | 2040.9497 | 0.0045 | 2 | 290 | 307 | VHSGEKPYACVQCGKCF K | | | Carbamidomethyl (C)[10] | | Mascot |
| 2230.0015 | 2230.2168 | 0.2153 | 97 | 174 | 193 | IHSGEKTFNCQVCGNNY STK | | | | | Mascot |
| 2297.1165 | 2297.2095 | 0.093 | 40 | 262 | 281 | IHSGEKPFSCVLCGSRFS TK | | | Carbamidomethyl (C)[10,13] | | Mascot |
| 2297.9883 | 2298.2048 | 0.2165 | 94 | 234 | 253 | IHSGEKPFACQECGRCC STK | | | Carbamidomethyl (C)[10,13] | | Mascot |
| 2300.1208 | 2300.1975 | 0.0767 | 33 | 258 | 277 | VHMRIHSGEKPFSCVLC GSR | | | Carbamidomethyl (C)[14] | | Mascot |
| 2528.1956 | 2528.1924 | -0.0032 | -1 | 282 | 304 | GNLDVHMRVHSGEKPYA CVQCGK | | | | | Mascot |

40 protein-tyrosine phosphatase mu [Danio rerio] gi|257215050 15158.5 6.11 5 29 0

**Peptide Information**

| Calc. Mass | Obsrv. Mass | ± da | ± ppm | Start Seq. | End Seq. | Sequence | Ion Score | C. I. % | Modification | Rank | Result Type |
|---|---|---|---|---|---|---|---|---|---|---|---|
| 1045.4806 | 1045.5698 | 0.0892 | 85 | 66 | 74 | CMDVLPPDR | | | | | Mascot |
| 1230.6324 | 1230.6799 | 0.0475 | 39 | 24 | 34 | LDPQTNSSQIK | | | | | Mascot |
| 1303.7772 | 1303.772 | -0.0052 | -4 | 121 | 131 | LVPGLPLYIYR | | | | | Mascot |
| 1706.8894 | 1706.8643 | -0.0251 | -15 | 35 | 48 | EEFRTLNMVTPTLR | | | | | Mascot |
| 1722.8843 | 1722.8663 | -0.018 | -10 | 35 | 48 | EEFRTLNMVTPTLR | | | Oxidation (M)[8] | | Mascot |
| 2233.019 | 2233.1045 | 0.0855 | 38 | 16 | 34 | SGYYDMNRLDPQTNSSQ IK | | | Oxidation (M)[6] | | Mascot |

41 novel protein similar to human titin (TTN) [Danio rerio] gi|94732358 2437518.2 5.95 60 29 0

Peptide Information

| Calc. Mass | Obsrv. Mass | ± da | ± ppm | Start Seq. | End Seq. | Sequence | Ion Score | C. I. % | Modification | Rank | Result Type |
|---|---|---|---|---|---|---|---|---|---|---|---|
| 842.4478 | 842.5154 | 0.0676 | 80 | 14643 | 14649 | RDLPEGR | | | | | Mascot |
| 848.4876 | 848.5325 | 0.0449 | 53 | 14548 | 14554 | TSILWTK | | | | | Mascot |
| 850.4668 | 850.5497 | 0.0829 | 97 | 11818 | 11824 | FTVENLK | | | | | Mascot |
| 864.5189 | 864.5179 | -0.001 | -1 | 1322 | 1328 | KYEIIAK | | | | | Mascot |
| 882.4791 | 882.5728 | 0.0937 | 106 | 7328 | 7334 | KEEHAIR | | | | | Mascot |
| 942.573 | 942.5549 | -0.0181 | -19 | 8640 | 8647 | DRQGIIIK | | | | | Mascot |
| 982.5468 | 982.5555 | 0.0087 | 9 | 7963 | 7969 | EYIFRVR | | | | | Mascot |
| 996.5359 | 996.6023 | 0.0664 | 67 | 3662 | 3669 | LSYEQTKK | | | | | Mascot |
| 996.5359 | 996.6023 | 0.0664 | 67 | 3662 | 3669 | LSYEQTKK | | | | | Mascot |
| 1051.6146 | 1051.7208 | 0.1062 | 101 | 10946 | 10955 | VSEKPLPPGK | | | | | Mascot |
| 1062.6055 | 1062.6975 | 0.092 | 87 | 12317 | 12325 | IIAQNRYGK | | | | | Mascot |
| 1106.6317 | 1106.5522 | -0.0795 | -72 | 14635 | 14643 | VTGYIIERR | | | | | Mascot |
| 1107.5292 | 1107.5391 | 0.0099 | 9 | 20800 | 20808 | HFVPFSDMK | | | | | Mascot |
| 1123.524 | 1123.5309 | 0.0069 | 6 | 20800 | 20808 | HFVPFSDMK | | | Oxidation (M)[8] | | Mascot |
| 1156.5858 | 1156.6102 | 0.0244 | 21 | 1768 | 1776 | EGPRYHLER | | | | | Mascot |
| 1165.6212 | 1165.756 | 0.1348 | 116 | 422 | 431 | QAKFESTINK | | | | | Mascot |
| 1165.6324 | 1165.756 | 0.1236 | 106 | 5141 | 5149 | ITNYTIERR | | | | | Mascot |
| 1169.6525 | 1169.6438 | -0.0087 | -7 | 2630 | 2639 | ADLVLKPEER | | | | | Mascot |
| 1185.6626 | 1185.6406 | -0.022 | -19 | 17301 | 17311 | GKPIPTASWTK | | | | | Mascot |
| 1230.6841 | 1230.6799 | -0.0042 | -3 | 18967 | 18977 | GKPTPTSKWTK | | | | | Mascot |
| 1279.7844 | 1279.7867 | 0.0023 | 2 | 4556 | 4567 | VNRAPVTKPAVK | | | | | Mascot |
| 1303.7844 | 1303.772 | -0.0124 | -10 | 14040 | 14051 | AGGSLRLFITIR | | | | | Mascot |
| 1402.5977 | 1402.7797 | 0.182 | 130 | 648 | 660 | DKGVYVCDCGTDK | | | | | Mascot |
| 1448.7267 | 1448.9343 | 0.2076 | 143 | 17957 | 17969 | AEYTPPKAELDSK | | | | | Mascot |
| 1460.6396 | 1460.64 | 0.0004 | 0 | 16170 | 16182 | GDPMEMEEPVQAK | | | | | Mascot |
| 1509.8536 | 1509.7767 | -0.0769 | -51 | 14847 | 14859 | LAWTLVHGEVKTR | | | | | Mascot |
| 1523.8502 | 1523.7728 | -0.0774 | -51 | 8687 | 8700 | VLDTPGPPLNLMIK | | | Oxidation (M)[12] | | Mascot |
| 1543.834 | 1543.8207 | -0.0133 | -9 | 14505 | 14519 | QPGPPSTPRVAHATK | | | | | Mascot |
| 1560.8843 | 1560.8516 | -0.0327 | -21 | 21524 | 21538 | DISASADTIVKLTVK | | | | | Mascot |
| 1560.8843 | 1560.8516 | -0.0327 | -21 | 21524 | 21538 | DISASADTIVKLTVK | | | | | Mascot |
| 1574.8424 | 1574.7871 | -0.0553 | -35 | 14114 | 14127 | VLDTPSSPQKFEVK | | | | | Mascot |
| 1582.8145 | 1582.8284 | 0.0139 | 9 | 18471 | 18485 | ITGYIIEACKAGTDK | | | | | Mascot |
| 1706.8265 | 1706.8643 | 0.0378 | 22 | 555 | 569 | EVSLDDTCQIKAEAK | | | Carbamidomethyl (C)[8] | | Mascot |

| | | | | | | | | |
|---|---|---|---|---|---|---|---|---|
| 1718.8894 | 1718.9227 | 0.0333 | 19 | 1060 | 1073 | EEGTKHMLILYNVR | Oxidation (M)[7] | Mascot |
| 1718.8894 | 1718.9227 | 0.0333 | 19 | 1060 | 1073 | EEGTKHMLILYNVR | Oxidation (M)[7] | Mascot |
| 1722.7712 | 1722.8663 | 0.0951 | 55 | 18717 | 18731 | YGTGEYLQSEVVMCK | Oxidation (M)[13] | Mascot |
| 1740.9102 | 1740.9028 | -0.0074 | -4 | 7820 | 7835 | VEALVSGKPAPACQWK | Carbamidomethyl (C)[13] | Mascot |
| 1761.9104 | 1761.8502 | -0.0602 | -34 | 11999 | 12013 | TFAAVVTNCHKLSWK | Carbamidomethyl (C)[9] | Mascot |
| 1857.9415 | 1857.8779 | -0.0636 | -34 | 10380 | 10396 | LEADVAGQPLPSMVWTK | Oxidation (M)[13] | Mascot |
| 1857.9415 | 1857.8779 | -0.0636 | -34 | 10380 | 10396 | LEADVAGQPLPSMVWTK | Oxidation (M)[13] | Mascot |
| 1862.9283 | 1862.9628 | 0.0345 | 19 | 6540 | 6556 | KPWEPWSVVSSGSTSTK | | Mascot |
| 1878.9443 | 1878.9661 | 0.0218 | 12 | 4049 | 4065 | DGAEPDKEHVVIEAEIK | | Mascot |
| 1903.9396 | 1903.9333 | -0.0063 | -3 | 21786 | 21802 | NVYTLEIHNATIEDSGK | | Mascot |
| 1924.0386 | 1923.9751 | -0.0635 | -33 | 5606 | 5622 | INVPDRPEELEVTAITK | | Mascot |
| 1924.0386 | 1923.9751 | -0.0635 | -33 | 5606 | 5622 | INVPDRPEELEVTAITK | | Mascot |
| 2010.0138 | 2010.0215 | 0.0077 | 4 | 5634 | 5652 | NDGGSEVTSYILEARTIG K | | Mascot |
| 2026.0928 | 2025.9406 | -0.1522 | -75 | 2640 | 2658 | ITIDTKPGHSTLSIANTTR | | Mascot |
| 2040.9985 | 2040.9497 | -0.0488 | -24 | 3146 | 3162 | YRVTDVDEGTEVQFQVR | | Mascot |
| 2194.1389 | 2194.1267 | -0.0122 | -6 | 2918 | 2935 | IESYIIELQKTGTEEWVR | | Mascot |
| 2211.1147 | 2211.1243 | 0.0096 | 4 | 15930 | 15948 | VVWSVISEAMESRMVTV QK | Oxidation (M)[10,14] | Mascot |
| 2211.1147 | 2211.1243 | 0.0096 | 4 | 15930 | 15948 | VVWSVISEAMESRMVTV QK | Oxidation (M)[10,14] | Mascot |
| 2230.0808 | 2230.2168 | 0.136 | 61 | 9022 | 9041 | DSITNMWVTVSSSVVTNS FR | | Mascot |
| 2233.0076 | 2233.1045 | 0.0969 | 43 | 13895 | 13914 | VSAENETGVGECSDLSL FYR | Carbamidomethyl (C)[12] | Mascot |
| 2271.2019 | 2271.1245 | -0.0774 | -34 | 17420 | 17439 | STQTSLRVADLLEGVPYF FK | | Mascot |
| 2283.0269 | 2283.2009 | 0.174 | 76 | 1430 | 1450 | MDDAGEYHCKLPASETT GTLK | Oxidation (M)[1] | Mascot |
| 2284.0583 | 2284.1958 | 0.1375 | 60 | 9228 | 9248 | ANTVNVIDCAYTVADLQE GCK | Carbamidomethyl (C)[9] | Mascot |
| 2284.0583 | 2284.1958 | 0.1375 | 60 | 9228 | 9248 | ANTVNVIDCAYTVADLQE GCK | Carbamidomethyl (C)[9] | Mascot |
| 2297.2095 | 2297.2095 | 0 | 0 | 17558 | 17578 | EPRIEPTADLSGITNQLVT SR | | Mascot |
| 2298.2703 | 2298.2048 | -0.0655 | -28 | 9621 | 9641 | DTSVPLRITEFVVPNLEA GLK | | Mascot |
| 2300.1616 | 2300.1975 | 0.0359 | 16 | 11905 | 11925 | ESAQIETSSSYTSLVIDKV SR | | Mascot |
| 2506.3774 | 2506.2161 | -0.1613 | -64 | 9978 | 10000 | AGENIKVEVPVSGRPKPT VSWQK | | Mascot |
| 2528.2258 | 2528.1924 | -0.0334 | -13 | 140 | 161 | NLEEETSVLKGQPMYLT CELSK | Oxidation (M)[14] | Mascot |
| 2719.3474 | 2719.3137 | -0.0337 | -12 | 16737 | 16760 | INGLIEGLAYEFRVYAQN DAGYSR | | Mascot |
| 2719.3474 | 2719.3137 | -0.0337 | -12 | 16737 | 16760 | INGLIEGLAYEFRVYAQN DAGYSR | | Mascot |
| 2741.3337 | 2741.3174 | -0.0163 | -6 | 2209 | 2233 | DSVQLSWETPISNGGSMI TGYIIEK | Oxidation (M)[17] | Mascot |
| 3337.5527 | 3337.8066 | 0.2539 | 76 | 11839 | 11869 | LGVGEHADVAGPICPED KTEEPDLDIDAEFR | | Mascot |

| Calc. Mass | Obsrv. Mass | ± da | ± ppm | Start Seq. | End Seq. | Sequence | Ion Score | C. I. % | Modification | Rank | Result Type |
|---|---|---|---|---|---|---|---|---|---|---|---|
| 3338.5608 | 3338.7876 | 0.2268 | 68 | 15242 | 15269 | AWVCVSSNCHSLTYKVE DLQEGAIYYFR | | | Carbamidomethyl (C)[4] | | Mascot |
| 3355.5786 | 3355.4919 | -0.0867 | -26 | 17909 | 17937 | CNYTNVTDTNFTITALGE GEEYEFRVIAK | | | Carbamidomethyl (C)[1] | | Mascot |
| 3896.0361 | 3895.8374 | -0.1987 | -51 | 13297 | 13333 | DDLGKYAVTATNSVCAIT EDINIVILDKPGPPTGPIK | | | Carbamidomethyl (C)[15] | | Mascot |

| | | | | | | | |
|---|---|---|---|---|---|---|---|
| 42 | retinoid x receptor, beta [Danio rerio] | gi\|94733756 | 53341.7 | 8.14 | 9 | 29 | 0 |

Peptide Information

| Calc. Mass | Obsrv. Mass | ± da | ± ppm | Start Seq. | End Seq. | Sequence | Ion Score | C. I. % | Modification | Rank | Result Type |
|---|---|---|---|---|---|---|---|---|---|---|---|
| 848.4294 | 848.5325 | 0.1031 | 122 | 182 | 188 | DCLVDKR | | | | | Mascot |
| 982.463 | 982.5555 | 0.0925 | 94 | 201 | 208 | CLAMGMKR | | | Carbamidomethyl (C)[1], Oxidation (M)[4] | | Mascot |
| 1106.5146 | 1106.5522 | 0.0376 | 34 | 179 | 187 | DNKDCLVDK | | | Carbamidomethyl (C)[5] | | Mascot |
| 1156.564 | 1156.6102 | 0.0462 | 40 | 12 | 21 | ARLHNMGDSR | | | | | Mascot |
| 1303.5808 | 1303.772 | 0.1912 | 147 | 14 | 24 | LHNMGDSRDSR | | | Oxidation (M)[4] | | Mascot |
| 1460.709 | 1460.64 | -0.069 | -47 | 419 | 430 | EKVYASLEAYCK | | | Carbamidomethyl (C)[11] | | Mascot |
| 1543.8325 | 1543.8207 | -0.0118 | -8 | 407 | 420 | GLSSPSEVELLREK | | | | | Mascot |
| 1718.7261 | 1718.9227 | 0.1966 | 114 | 148 | 162 | SSGKHYGVYSCEGCK | | | Carbamidomethyl (C)[11,14] | | Mascot |
| 1718.7261 | 1718.9227 | 0.1966 | 114 | 148 | 162 | SSGKHYGVYSCEGCK | | | Carbamidomethyl (C)[11,14] | | Mascot |
| 1923.8124 | 1923.9751 | 0.1627 | 85 | 230 | 247 | DGEVESSSAANEEMPVE K | | | Oxidation (M)[14] | | Mascot |
| 1923.8124 | 1923.9751 | 0.1627 | 85 | 230 | 247 | DGEVESSSAANEEMPVE K | | | Oxidation (M)[14] | | Mascot |

| | | | | | | | |
|---|---|---|---|---|---|---|---|
| 43 | PREDICTED: chromodomain-helicase-DNA-binding protein 4 isoform 1 [Danio rerio] | gi\|292622418 | 221223.8 | 5.72 | 17 | 29 | 0 |

Peptide Information

| Calc. Mass | Obsrv. Mass | ± da | ± ppm | Start Seq. | End Seq. | Sequence | Ion Score | C. I. % | Modification | Rank | Result Type |
|---|---|---|---|---|---|---|---|---|---|---|---|
| 942.5441 | 942.5549 | 0.0108 | 11 | 501 | 509 | CLSPPLKGK | | | | | Mascot |
| 1045.516 | 1045.5698 | 0.0538 | 51 | 1701 | 1710 | DGVKPEDSAK | | | | | Mascot |
| 1062.5942 | 1062.6975 | 0.1033 | 97 | 1815 | 1823 | GNFLEIKNK | | | | | Mascot |
| 1107.6594 | 1107.5391 | -0.1203 | -109 | 1058 | 1067 | ASGKLMLLFK | | | | | Mascot |
| 1123.6544 | 1123.5309 | -0.1235 | -110 | 1058 | 1067 | ASGKLMLLFK | | | Oxidation (M)[6] | | Mascot |
| 1156.6547 | 1156.6102 | -0.0445 | -38 | 1179 | 1187 | VMIYRFVTK | | | | | Mascot |
| 1165.6211 | 1165.756 | 0.1349 | 116 | 1184 | 1193 | FVTKASVEER | | | | | Mascot |
| 1165.6211 | 1165.756 | 0.1349 | 116 | 1184 | 1193 | FVTKASVEER | | | | | Mascot |
| 1169.6273 | 1169.6438 | 0.0165 | 14 | 1541 | 1552 | RAAAIAAGEDPK | | | | | Mascot |
| 1230.6298 | 1230.6799 | 0.0501 | 41 | 1391 | 1401 | SEAANSRRPSR | | | | | Mascot |
| 1582.8005 | 1582.8284 | 0.0279 | 18 | 1881 | 1895 | ESMSGNKPANAVLHK | | | | | Mascot |

| | | | | | | | | | | | |
|---|---|---|---|---|---|---|---|---|---|---|---|
| 1761.9857 | 1761.8502 | -0.1355 | -77 | 706 | 721 | KTERPPANPVVDPTIK | | | | | Mascot |
| 1862.8564 | 1862.9628 | 0.1064 | 57 | 1028 | 1044 | CCNHPYLFPTAATEAPK | | | | | Mascot |
| 1923.9558 | 1923.9751 | 0.0193 | 10 | 824 | 839 | AVIRENEFTFEDNAIR | | | | | Mascot |
| 1923.9558 | 1923.9751 | 0.0193 | 10 | 824 | 839 | AVIRENEFTFEDNAIR | | | | | Mascot |
| 2225.0569 | 2225.1379 | 0.081 | 36 | 1553 | 1574 | TPSTGTPADTQPNTPAP EDLSK | | | | | Mascot |
| 2225.0569 | 2225.1379 | 0.081 | 36 | 1553 | 1574 | TPSTGTPADTQPNTPAP EDLSK | | | | | Mascot |
| 2229.9822 | 2230.2168 | 0.2346 | 105 | 155 | 172 | DIDYTFTQEDYNSLTNYK | | | | | Mascot |
| 2528.1082 | 2528.1924 | 0.0842 | 33 | 409 | 429 | APEGTWSCPHCEKEGIQ WEAR | | | Carbamidomethyl (C)[8,11] | | Mascot |
| 3895.5496 | 3895.8374 | 0.2878 | 74 | 362 | 395 | KMDEDADGYETDHQDYC EVCQQGGEIILCDTCPR | | | Oxidation (M)[2] | | Mascot |
| 3896.5146 | 3896.8367 | 0.3221 | 83 | 1364 | 1397 | DWQDDQSDNQSDYSVA SEEGDEDFDERSEAANS R | | | | | Mascot |
[truncated: 500,022 more chars]
